# Cell-Based Systems Biology Analysis of Human AS03-Adjuvanted H5N1 Avian Influenza Vaccine Responses: A Phase I Randomized Controlled Trial

**DOI:** 10.1371/journal.pone.0167488

**Published:** 2017-01-18

**Authors:** Leigh M. Howard, Kristen L. Hoek, Johannes B. Goll, Parimal Samir, Allison Galassie, Tara M. Allos, Xinnan Niu, Laura E. Gordy, C. Buddy Creech, Nripesh Prasad, Travis L. Jensen, Heather Hill, Shawn E. Levy, Sebastian Joyce, Andrew J. Link, Kathryn M. Edwards

**Affiliations:** 1 Vanderbilt Vaccine Research Program, Department of Pediatrics, Vanderbilt University School of Medicine, Nashville, TN, United States of America; 2 Department of Pathology, Microbiology and Immunology, Vanderbilt University School of Medicine, Nashville, TN, United States of America; 3 The Emmes Corporation, Rockville, MD, United States of America; 4 Department of Biochemistry, Vanderbilt University School of Medicine, Nashville, TN, United States of America; 5 Department of Chemistry, Vanderbilt University, Nashville, TN, United States of America; 6 HudsonAlpha Institute for Biotechnology; Huntsville, AL, United States of America; 7 Veterans Administration Tennessee Valley Healthcare System, Nashville, TN, United States of America; Public Health England, UNITED KINGDOM

## Abstract

**Background:**

Vaccine development for influenza A/H5N1 is an important public health priority, but H5N1 vaccines are less immunogenic than seasonal influenza vaccines. Adjuvant System 03 (AS03) markedly enhances immune responses to H5N1 vaccine antigens, but the underlying molecular mechanisms are incompletely understood.

**Objective and Methods:**

We compared the safety (primary endpoint), immunogenicity (secondary), gene expression (tertiary) and cytokine responses (exploratory) between AS03-adjuvanted and unadjuvanted inactivated split-virus H5N1 influenza vaccines. In a double-blinded clinical trial, we randomized twenty adults aged 18–49 to receive two doses of either AS03-adjuvanted (n = 10) or unadjuvanted (n = 10) H5N1 vaccine 28 days apart. We used a systems biology approach to characterize and correlate changes in serum cytokines, antibody titers, and gene expression levels in six immune cell types at 1, 3, 7, and 28 days after the first vaccination.

**Results:**

Both vaccines were well-tolerated. Nine of 10 subjects in the adjuvanted group and 0/10 in the unadjuvanted group exhibited seroprotection (hemagglutination inhibition antibody titer > 1:40) at day 56. Within 24 hours of AS03-adjuvanted vaccination, increased serum levels of IL-6 and IP-10 were noted. Interferon signaling and antigen processing and presentation-related gene responses were induced in dendritic cells, monocytes, and neutrophils. Upregulation of MHC class II antigen presentation-related genes was seen in neutrophils. Three days after AS03-adjuvanted vaccine, upregulation of genes involved in cell cycle and division was detected in NK cells and correlated with serum levels of IP-10. Early upregulation of interferon signaling-related genes was also found to predict seroprotection 56 days after first vaccination.

**Conclusions:**

Using this cell-based systems approach, novel mechanisms of action for AS03-adjuvanted pandemic influenza vaccination were observed.

**Trial Registration:**

ClinicalTrials.gov NCT01573312

## Introduction

Since avian A/H5N1 influenza viruses have been associated with a high morbidity and mortality in humans, vaccine development has been a public health priority [1, 2]. Relative to seasonal influenza vaccines, however, A/H5N1 influenza vaccines have been less immunogenic, inducing weaker vaccine strain hemagglutinin inhibition (HAI) and neutralizing antibody responses after a single vaccination [3]. Adjuvants, such as aluminum salts, monophosphoryl lipid A (MPL) with aluminum salt (AS04) [4, 5], and oil-in-water emulsion-based formulations (MF59, AF03, AS03) [6–8], have been shown to increase the immunogenicity of both seasonal and avian influenza vaccines and decrease the amount of antigen required (antigen-sparing) [9–11]. One such emulsion-based, TLR-independent adjuvant, Adjuvant System 03 (AS03) [6], has been shown to markedly increase the immune response to avian influenza vaccines and to stimulate significantly higher antibody responses compared to MF59 when administered with H7N9 vaccine [12–18].

Initial studies of the mechanisms of action of AS03 in animal models suggest that the adjuvant enhances antigen delivery, immune cell recruitment, stimulation of local immune cells, and induction of complement and other stress signals [19]. AS03 has also been shown to increase innate immune responses and promote recruitment of antigen-presenting cells (APC) to draining lymph nodes [19]. Transcriptomic analysis of mouse muscle cells at vaccine injection sites has demonstrated that other oil-in-water adjuvants also stimulate local expression of genes involved in innate immune responses including cytokine activity, leukocyte migration, and antigen presentation [20, 21]. Finally, a recent AS03-adjuvanted H1N1 human vaccine study identified early cytokine responses and gene responses related to interferon signaling and antigen presentation in peripheral blood mononuclear cells (PBMC) at day 1, as well as increased incidence of plasma cells and plasma cell-associated genes at day 7 [22]. However, our understanding of the molecular interactions by which AS03 enhances human immune responses to H5N1 vaccine is still incomplete.

Systems vaccinology offers a comprehensive approach to dissect the human immune response to vaccination by correlating traditional antibody and cell-mediated immune responses with changes in global molecular signatures of gene, protein, metabolite, and lipid expression [23–28]. We recently reported the use of a systems biology approach to study the immune responses in six individual immune cell types following vaccination with seasonal trivalent influenza vaccine [29]. That study revealed that monocytes, neutrophils, myeloid dendritic cells (mDC), natural killer (NK) cells, T cells, and B cells showed unique transcriptomic profiles and changing biological networks at early time points after vaccination. Such cell type-specific systems information provides a unique approach to monitor transcriptional responses after vaccination.

Using this approach, we conducted a small, single center study with the objectives to assess the safety, reactogenicity, immunogenicity, and molecular immune responses to intramuscular (IM) administration of an inactivated monovalent influenza A/H5N1 (Hemagglutinin [HA] of A/Indonesia/05/2005) split-virus vaccine given with or without the AS03 adjuvant. Our comprehensive cell-based systems analysis of human transcriptomic responses to H5N1 vaccine indicates that AS03 modulates early innate immune gene expression and increased serum cytokine responses, with the strongest effect for genes expressed in neutrophils, monocytes, and dendritic cells. Potentially novel mechanisms of action for the AS03 adjuvant when administered with avian influenza vaccine were identified, including up-regulation of antigen processing and presentation genes in neutrophils and induction of cellular proliferation responses that correlated with serum IP-10 levels in NK cells.

## Materials and Methods

### Study Products

Inactivated monovalent influenza A/Indonesia/05/2005 H5N1 split-virus vaccine at a dosage of 3.75 mcg and phosphate buffered saline (PBS) diluent were both manufactured by Sanofi Pasteur. The AS03 adjuvant was manufactured by GlaxoSmithKline (GSK) and contains 4.86 mg polysorbate 80, 11.86 mg α-tocopherol, and 10.69 mg squalene per 0.5 mL vaccine dose in an oil-in-water emulsion. The vaccine and adjuvant were provided by the US Department of Health and Human Services Biomedical Advanced Research and Development Authority from the National Pre-pandemic Influenza Vaccine Stockpile.

### Study Design

**Fig 1:**
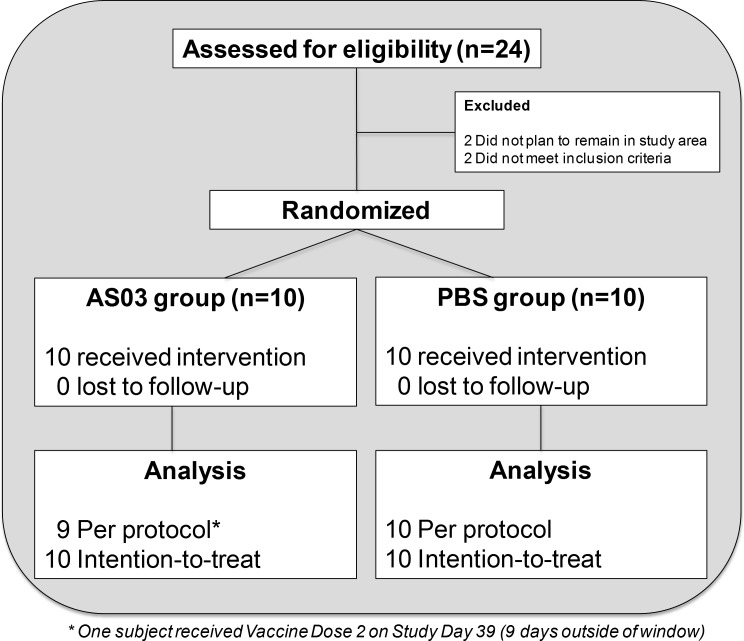
CONSORT diagram outlining study group enrollment and randomization.

A single-center, randomized, double-blinded, controlled, Phase I study in healthy male and non-pregnant female subjects 18 to 49 years old was designed to assess the safety, reactogenicity, immunogenicity, and molecular immune responses of an intramuscular (IM) inactivated monovalent influenza A/H5N1 (hemagglutinin [HA] of A/Indonesia/05/2005) split-virus (SV) vaccine administered at 3.75 mcg given with the AS03 adjuvant (SV-AS03) or PBS diluent (SV-PBS) (**S1 Text**). After informed consent, eligible subjects were screened for inclusion and exclusion criteria (enrollment criteria described in **Section 2.1 in S2 Text**). Twenty subjects were randomized 1:1 in a double-blind fashion to receive either 3.75 mcg of H5 hemagglutinin + AS03 (n = 10) or 3.75 mcg of H5 hemagglutinin + PBS (n = 10). Permuted block randomization with random block sizes of 2 and 4 were applied to improve balance between vaccine groups. The treatment allocation sequence was generated by the study statistician using a random number generator. A secure web portal was used to assign treatment codes to subjects after verification of eligibility. An unblinded pharmacist prepared the treatment per the assigned code, which was then administered by an unblinded vaccine administrator who had no further contact with the subject. All subjects received two 0.5 mL intramuscular injections 28 days apart containing equal volumes of the inactivated monovalent influenza A/H5N1 split-virus vaccine admixed by the study pharmacist prior to administration with either AS03 or PBS. As the adjuvant had a milky white appearance, the subject was asked to look away during injection. The subjects and the study team, with the exception of the unblinded vaccinator, as well as the study investigators, research coordinators, and laboratory personnel were blinded to vaccine assignment (**Fig 1**). No subject was lost to follow-up (**Fig 1, Tables A1 and A4 in S2 Text**).

The sample size in this Phase I study was not based on a formal hypothesis but practical considerations to gather initial information on the safety, reactogenicity, immunogenicity, and molecular immune responses. Study recruitment commenced May 22, 2012, and the final study follow-up visit occurred January 2, 2014.

### Safety and Reactogenicity Assessments

The occurrence of solicited local and systemic reactogenicity events were captured for 8 days following each vaccine dose. Unsolicited adverse events and new-onset medical conditions were also recorded until one year after the second vaccination, and events were graded for severity and for relationship to study product. All reactogenicity assessments were collected according to standardized methodology (**Section 2.3 in S2 Text**).

### Laboratory Procedures

For cytokine/chemokine levels, cell sorting, cell activation, and RNA-Seq assessments, blood samples were collected on study visit days -28, -14, 0 (pre-vaccination), and 1, 3, 7, 28 (post-first vaccination). Sera for hemagglutination inhibition (HAI) and neutralizing antibody (Nt) assays were collected at study visit days 0, 1, 3, 7, 28, and 56.

#### Hemagglutination inhibition (HAI) and neutralizing antibody (Nt) assays

Sera for hemagglutinin inhibition (HAI) and neutralizing antibody (Nt) responses against the homologous A/Indonesia/05/2005 virus were measured as previously described (Southern Research; Birmingham, AL) [30]. For analysis, values below the limit of detection (1:10 for each assay) were imputed as half the limit of detection.

#### Cytokine and chemokine assays

Cytometric bead arrays (CBAs) for IL-1α, IL-1β, IL-6, IL-8, IL-10, IL-12p70, IP-10, TNF, LT-α, RANTES, eotaxin-1, MCP-1, MIP-1α, and IFN-γ were performed in duplicate according to the manufacturer's instructions **(**BD Biosciences). Data were acquired on a BD LSRII flow cytometer **(**BD Biosciences). CBA results below the lower limit of detection (LOD) were imputed using ½ of the LOD estimate provided by BD Biosciences (see **S2 Text**).

#### Cell sorting

Fractionation of collected blood samples for neutrophils, monocytes, DC, NK cells, T cells and B cells was performed as previously described [29]. Briefly, whole blood samples (90 mL) underwent Ficoll separation to yield PBMC and polymorphonuclear cell (PMN) populations. Single cell suspensions of PBMC or PMN were subjected to magnetic bead separation. T cells, monocytes, and neutrophils were enriched by positive selection using directly conjugated anti-CD3, anti-CD14, and anti-CD15 microbeads (Miltenyi Biotec), respectively. B cells were enriched by positive selection using anti-PE beads after staining with anti-CD19-PE antibody (Miltenyi Biotech). NK/mDC were enriched by negative selection using Streptavadin microbeads (Miltenyi Biotec) after staining with biotinylated anti-CD19, anti-CD15, anti-CD14, and anti-CD3 antibodies (eBioscience). MACS enriched cells were stained with 7-aminoactinomycin D (7-AAD), CD11c-FITC, CD15-APC and CD56-PE-Cy7 (BD Biosciences), as well as CD19-PE, CD3-VioBlue, and CD14-VioGreen (Miltenyi Biotec), and were subjected to fluorescence-activated cell sorting (FACS) on a BD FACSAriaIII flow cytometer (BD Biosciences). Cell purity of ≥98% was confirmed after the sorting procedures. Purified immune cells (≥0.5x10^6^) were frozen in homogenization buffer (Maxwell 16 LEV simply RNA kit, Promega) and stored at -80°C.

#### Cell phenotype and activation assessments

Multi-parameter flow cytometric analysis (FCM) was performed on a daily basis using the same antibody panel from the cell sort, without 7AAD and including CD69-APC-Cy7, CD86-PerCP-Cy5.5 and CD134-PE-Cy5 (BD Biosciences), to assess the phenotype and cellular activation status of each cell type in whole blood, the PBMC and PMN fractions, and post-sorted cell samples as previously described (see **S2 Text** for additional details) [29].

#### RNA-Sequencing and data processing

RNA-Seq experiments were performed as previously described [29]. Briefly, RNA was extracted from sorted cells using the automated Maxwell 16 magnetic particle processor and a Maxwell 16 LEV simply RNA kit (Promega). Polyadenylated RNAs were isolated using NEBNext magnetic oligo d(T)_25_ beads (New England BioLabs), individually bar-coded, and next generation sequencing expression libraries were prepared using the NEBNext mRNA Library Prep Reagent Set for Illumina (New England BioLabs). Each library was diluted to a final concentration of 12.5nM and pooled equimolar prior to clustering. Paired-End (PE) sequencing (25 million, 50-bp, paired-end reads) was performed using a 200 cycle TruSeq SBS HS v3 kit (Illumina) on an Illumina HiSeq2000 sequencer. Image analysis and base calling were performed using the standard Illumina Pipeline consisting of *Real time Analysis* (RTA) v1.13. Raw reads were de-multiplexed using a *bcl2fastq* conversion software v1.8.3 (Illumina) with default settings. Paired-end reads were mapped against the human reference genome (GRCh37) using *TopHat* v2.0.0. Human gene models and annotations were obtained from ENSEMBL v63 (June 2011). Gene expression quantification was conducted on the gene level using *Subread* v1.4.6 counting mapped paired-reads to obtain fragment counts per gene (see **S2 Text** for additional details).

### Statistical Analysis

#### Serum antibody, cytokine/chemokine, and cell activation analyses

Serum HAI and Nt GMTs were compared at each post-vaccination time point using a *t*-test under the assumption that titers are log normally distributed adjusting for unequal variances between vaccine groups, if necessary (Satterthwaite approximation if the equality of variances based on a Folded form F-test was rejected). The proportion of subjects with a pre-vaccination titer <1:10 and a minimum four-fold rise to a post-baseline titer ≥ 1:40 (seroconversion) and those achieving post-vaccination antibody titers ≥1:40 (seroprotection) [31, 32] were summarized per post-vaccination time point using exact 95% CIs and compared using a Fisher’s exact test. The 95% CIs for median serum cytokine/chemokine baseline fold changes for each post-vaccination time point were calculated using the bootstrap method [33] with 1,000 replications. Fold changes between vaccine groups were compared using a non-parametric exact Wilcoxon rank-sum test. The same non-parametric methods were used to visualize and compare percent cell activation. All hypothesis tests described in this section were carried out in a two-sided manner using an individual alpha level of 0.05. Bootstrap-based median CIs were calculated using the R statistical software (Version 3.01). All other analyses described in this section were executed using the SAS statistical software (Version 9.3).

#### RNA-Seq analyses

*edgeR* [34] (Version 3.2.4) was used to correct systematic sample differences in fragment counts using TMM normalization [35] to calculate moderated log_2_ fragment counts per million (LCPM), and to model fragment counts using a discrete probability distribution. LCPMs were used as input for multivariate analyses to detect outliers and to filter out lowly expressed genes (see **S2 Text**). Log_2_ fold changes from baseline (LFCPM) were calculated for each subject and immune cell type by subtracting the mean of the log_2_ baseline values (days -28, -14, 0) from each of the subject’s post-baseline days. Differentially expressed (DE) genes (SV-AS03 vs. SV-PBS) were identified for each post-vaccination day and immune cell type by fitting negative binomial generalized linear models as implemented in *edgeR* under the assumption that discrete fragment counts are negative binomial distributed. Models included fixed effects for subject, study visit day (baseline or post-vaccination day), and a study visit day x vaccine group interaction term (see **S2 Text**). The subject effect for estimating subject-specific mean baseline levels was added to account for correlations between samples from the same subject. The interaction term represents the log_2_ fold change difference (LFCD) between SV-AS03 and SV-PBS vaccine group responses. The statistical significance of the interaction term was evaluated for each gene using a likelihood ratio test. To control for testing multiple genes, the false-discovery rate (FDR) based on the Benjamini-Hochberg procedure [36] as implemented in the p.adjust R function was applied. Based on results from our previous study [29], genes with an FDR-adjusted p-value ≤ 0.05 and a fold change of ≥ 1.5-fold (up or down regulation based on LFCD) between the two vaccine groups were deemed to be significantly differentially expressed. A global GC-content bias was observed when visualizing vaccine group effect by gene GC-content (**Fig A41-A46 in S2 Text)** [37]. This confounding effect was successfully corrected for by adding mean sample GC-content as a covariate to the negative binomial models when determining DE genes (see **S2 Text**). To identify robust clusters of co-expressed genes based on LFCPM, multiscale bootstrapping was carried out using the *pvclust R* package [38]. Gene set enrichment analysis to identify significantly enriched KEGG [39] (v70.0), and *MSigDB* [40] (v4.0) functional modules was carried out using the *goseq* R package [41] (v1.12.0) correcting for gene length bias and multiple testing (FDR-adjusted p-value ≤ 0.01). The *mixOmics* (v5.0–3), and *glmnet* (v2.0–2) R packages were used for regularized canonical and logistic regression analysis to identify gene responses (based on LFCPM) that correlated with cytokine/antibody responses as well as genes that predicted seroprotection status (HAI titer ≥1:40). In both cases, leave-one-out cross-validation was used to select optimal models (see **S2 Text**). Human-human and human-influenza protein-protein interaction networks were downloaded from the IntAct database (01/14/2015). RNA-Seq analysis was carried out using the R statistical software (Version 3.01).

### Ethics Statement

This study was approved by the Vanderbilt University Institutional Review Board. The study was conducted in accordance with Good Clinical Practice, the Declaration of Helsinki, the US Code of Federal Regulations for the Protection of Human Subjects, and the Department of Health and Human Services Belmont Report. The study is registered on ClinicalTrials.gov (No. NCT01573312). All subjects provided written informed consent prior to initiation of study procedures. Subjects were assigned de-identified code numbers and their privacy strictly held in trust by study personnel, sponsors, and their agents. This confidentiality extends to cover testing of biological samples, in addition to the clinical information relating to participants.

### Data Availability

In order to avoid compromising study participants' privacy in accordance with the Informed Consent Document and the Vanderbilt University Medical Center Institutional Review Board, raw sequencing data and reference alignments containing variant information generated as part of this study will not be made publicly available. However, gene expression quantifications, and all other non-genomics measurements, including serum antibodies, cytokines, and cell activation used for the analysis are contained within the supplemental **S1, S2 and S3 Tables.**

## Results

### Safety and Tolerability Assessments

All 20 subjects received both doses of vaccine and are included in the primary analysis. The demographic characteristics of study participants are summarized in **Table A1 in S2 Text**. Both vaccines were well-tolerated, with no severe reactions or adverse events following either AS03-adjuvanted or unadjuvanted H5N1 vaccination. Most reactions were rated as mild and were self-limited. The occurrence of local and systemic reactions and adverse events are summarized in **Tables A2-A3 and Fig A2 in S2 Text**.

### Immunogenicity Assessments

**Fig 2:**
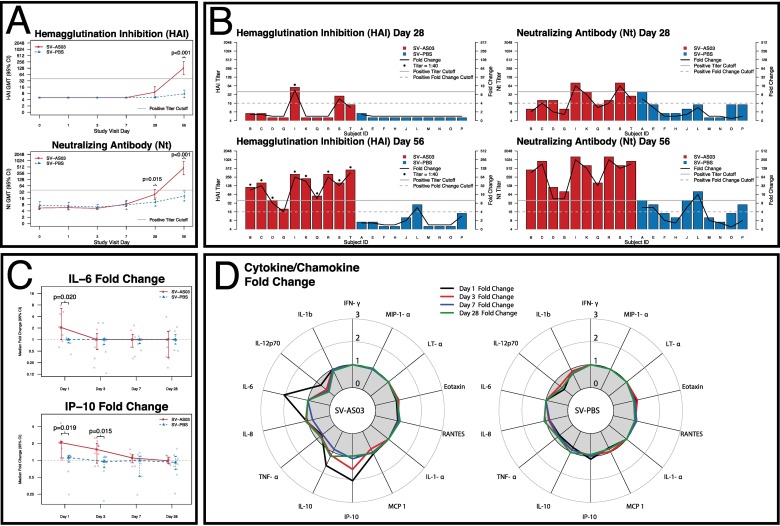
AS03-adjuvanted H5N1 vaccination induced early IP-10 and IL-6 serum cytokine and later protective immune responses relative to non-adjuvanted vaccine. Serum Antibody and cytokine responses by vaccine group (red: SV-AS03 (n = 10), blue: SV-PBS (n = 10)). **(A**) HAI and Nt antibody GMT and 95% CI at each time point by vaccine group; p-values are based on two-sided t-test on the log scale adjusting for unequal variances if necessary. No multiple-testing adjustment was carried out. **(B)** Hemagglutination inhibition (HAI) and neutralizing antibody (Nt) titers in individual subjects at days 28 and 56 by vaccine group; titer is represented by bar height (left y-axis) while fold change from baseline is shown as a connected black line (right y-axis), cut offs are indicated by grey lines (solid: 1:40 titer, dashed: 4-fold change). **(C)** Median fold change and 95% bootstrap CI for serum cytokines/chemokines with significantly different responses at each post-vaccination time point by vaccine group; individual subject fold changes are shown in lighter colors; p-values are based on two-sided non-parametric exact Wilcoxon rank-sum test. No multiple-testing adjustment was carried out. **(D)** Radar chart of cytokines/chemokine median fold changes at each time point by vaccine group.

HAI and Nt titers did not increase from baseline during the first 7 days after vaccination in either SV-AS03 or SV-PBS vaccinated subjects (**Fig 2A; Table A4 in S2 Text**). Similarly, at day 28, there were no significant differences in HAI GMT between the SV-AS03 and SV-PBS groups, (**Fig 2A**) with no subjects exhibiting an HAI response in the PBS group, while one subject in the SV-AS03 group exhibited an HAI titer ≥1:40 and > 4-fold rise in HAI titer (**Fig 2B)**. By day 56 (28 days after second vaccination), HAI GMT increased to 130.0 for the SV-AS03 subjects and 7.6 for the SV-PBS control group. Nine subjects in the SV-AS03 group exhibited seroconversion and seroprotection at day 56, while there were none who seroconverted (or reached seroprotection titers) in the SV-PBS group (p<0.001) (**Table A4 in S2 Text**). At day 28, Nt GMT was significantly higher in the SV-AS03 group than the SV-PBS group and by day 56, Nt GMT increased to 452.5 in the SV-AS03 group and 20.7 in the SV-PBS group (p<0.001) (**Fig 2A)**. All 10 subjects in the SV-AS03 group exhibited seroconversion and seroprotection at day 56, while only 3 subjects in the SV-PBS group had positive Nt responses (p = 0.003). The HAI and Nt log titers were significantly positively correlated with each other at both day 28 (r = 0.8, 0.73) and day 56 (r = 0.96, 0.85) for both the adjuvanted and unadjuvanted vaccine recipients, respectively.

At baseline, serum concentrations of IL-1α, IL-1β, LT-α, MIP-1α, IFN-γ, and IL-6 in blood samples were below or close to the limit of detection (LOD). However, subjects in the SV-AS03 group demonstrated a significant IL-6 response on the day after vaccination, with a median fold change of 2.06 in the SV-AS03 group compared to no change in the SV-PBS group (p = 0.02; **Fig 2C** and **2D**). A differential response was also observed for IP-10, with median fold changes of 2.06 and 1.56 at days 1 and 3 in the SV-AS03 group, and median fold changes of 1.12 and 0.95 at days 1 and 3 in the SV-PBS group (day 1 p = 0.019; day 3 p = 0.015; **Fig 2C** and **2D**). The IL-10 fold change was also moderately increased at day 1 in the SV-AS03 group (median fold change of 1.66) compared to the SV-PBS group (median fold change of 0.79), although this difference did not reach statistical significance (**Fig 2D, Fig A9 in S2 Text**). For all other cytokines/chemokines, no discernable differences in the two vaccine groups were observed (**Fig 2D; Fig A3-A16 in S2 Text**).

In subjects from the SV-AS03 group, transient increases were observed in circulating neutrophils at day 1 (median fold change of 1.41 in number and 1.22 in proportion), monocytes at day 1 (median fold change of 1.26 in number; no change in proportion) and day 3 (median fold change of 1.11 in number and 1.321 in proportion), and dendritic cells at day 3 (median fold change of 1.26 in number and 1.43 in proportion) (**Fig A212-213** and **Tables A161-A162 in S2 Text**), consistent with other recent reports [22, 42]. These increases returned to baseline by day 7.

Cellular activation analysis at each step of the purification process (whole blood, PBMC/PMN fractions, and post-sorted cells) revealed that sorted cells were not activated by the sorting process [29]. No statistically significant changes in activation marker expression in whole blood were noted in SV-AS03-adjuvanted relative to SV-PBS immune cell samples at early time points (day 1 and day 3) after vaccination. At day 7, monocytes showed an increase in the percentage of cells expressing CD134 (median fold change of 1.2 vs. 0.99; *p* = 0.035), NK cells showed an increase in the percentage of cells expressing CD134 (median fold change of 1.11 vs. 0.99; *p* = 0.028), and B cells showed an increase in the percentage of cells expressing CD134 (median fold change of 1.15 vs. 0.68; *p* = 0.043). At day 28, neutrophils showed an increase in the percentage of cells expressing CD69 (median fold change of 1.55 vs. 0.89; *p* = 0.028). For the remaining cell types and time points, no significant difference in percent of cells expressing activation markers between the two vaccine groups was observed (**Fig A194-A211 in S2 Text)**.

### Summary of RNA-Seq Analysis of Immune Cells

For the six immune cell types and seven time points analyzed by RNA-Seq (839 RNA-Seq samples), an average of 56.2 million reads were mapped, of which 83.4% uniquely mapped to a location on the human reference genome (**Table A11 in S2 Text**). The vast majority (81.6%) of the sequence tags mapped to known exon regions followed by intronic regions (16.6%), and intergenic regions (1.8%) (**Table A12 in S2 Text**). On average, 16.7 million fragments (reconstituted paired-end reads that mapped to the same gene) were counted for each sample in the expression quantification step to determine TMM-normalized fragment counts for differential gene analysis as well as LCPM and LFCPM for multivariate analyses (**Table A11 in S2 Text**). **Table A10 in S2 Text** summarizes the number of genes that passed the low expression cut off at each post-vaccination time point. Principal component analysis (PCA) of all samples, based on LCPM, revealed that most of the total variation in gene expression was attributable to immune cell type with three distinct cell clusters: (1) neutrophils, (2) lymphocytes (T cells, B cells, NK cells), and (3) dendritic cells/monocytes (**Fig A28 in S2 Text**). Non-metric multidimensional scaling biplots and hierarchical clustering analysis confirmed cell-specific global gene expression patterns (**Fig A29-A30 in S2 Text**).

### Multiple Time Points to Measure Baseline Gene Expression

To evaluate sources of baseline variability and impact on fold change estimates, we investigated expression levels at days -28, -14, and 0 prior to vaccination. Similar to global analysis of gene expression, PCA of the three baseline time points (days -28, -14, 0; 360 RNA-Seq samples) revealed that most of the total variation was attributable to the immune cell type (**Fig A39 in S2 Text**). In addition, PCA results summarizing baseline variability showed that between-subject variability was significantly larger than within-subject variability for all six immune cell types (ANOVA subject factor p < 0.001, **Tables A35-46 and Fig A40 in S2 Text**). Comparison of genes with an absolute mean fold change (≥ 1.5) using the mean gene expression of the three baseline measures (days -28, -14, 0) compared to either two (days -14 and 0) or one (day 0) showed that inclusion of additional baseline measures resulted in increasingly conservative and consistent fold change estimates reducing the impact of transient baseline signals (see **S2 Text** and **Table A53 in S2 Text**). In the following, we present DE gene results adjusted for per-subject mean baseline expression levels estimated using all three baseline measures as part of the negative binomial model (results were also adjusted for sample GC content; see Methods and **S2 Text**).

### Transcriptomic Analysis Shows that AS03-Adjuvanted Vaccination Primarily Modulates Early Innate Immune Cell Activity

To determine how AS03 boosts the immune response to vaccination, we first investigated genes induced in response to AS03-adjuvanted (SV-AS03) relative to unadjuvanted (SV-PBS) vaccine in each cell type. Neutrophils had the highest total number of DE genes (610 genes) between adjuvanted and unadjuvanted vaccines at all post-vaccination days (1, 3, 7, 28) followed by monocytes (406 genes), and dendritic cells (229 genes). Lymphocytes had fewer DE genes, with NK cells obtaining the highest number of differential genes (157 genes), followed by T cells (49 genes) and B cells (14 genes) (**S4 Table, Fig A54 in S2 Text**). The majority of DE genes (~87%; ranging from 84–100% depending on cell type and time point) were classified as protein-coding genes, followed by processed transcripts (~6%; 0–7%), pseudogenes (~4%; 0–8%) and lncRNAs (~2.5%; 0–4%). Several other transcript classes were represented by 1–2 genes on a per-cell type and/or time point basis (**Table A54 in S2 Text**). Except for NK cells, the majority of these DE genes were reported for day 1. The strongest responses at day 1 in terms of number of genes occurred in neutrophils, monocytes, and dendritic cells, with 572, 406, and 225 DE genes reported, respectively. Among these three immune cell types, genes expressed in neutrophils showed a noticeably persistent differential response at day 3 (140 genes, 103 of which were also identified at day 1) (**Fig A54 in S2 Text**). Genes expressed in T cells, NK cells, and B cells showed much less pronounced responses at day 1, with 14–49 DE genes. NK cells showed distinct responses at day 3 and day 28, with 70 and 63 DE genes, respectively.

### Response Time Trends of AS03-Modulated Genes and Gene Clusters

**Fig 3:**
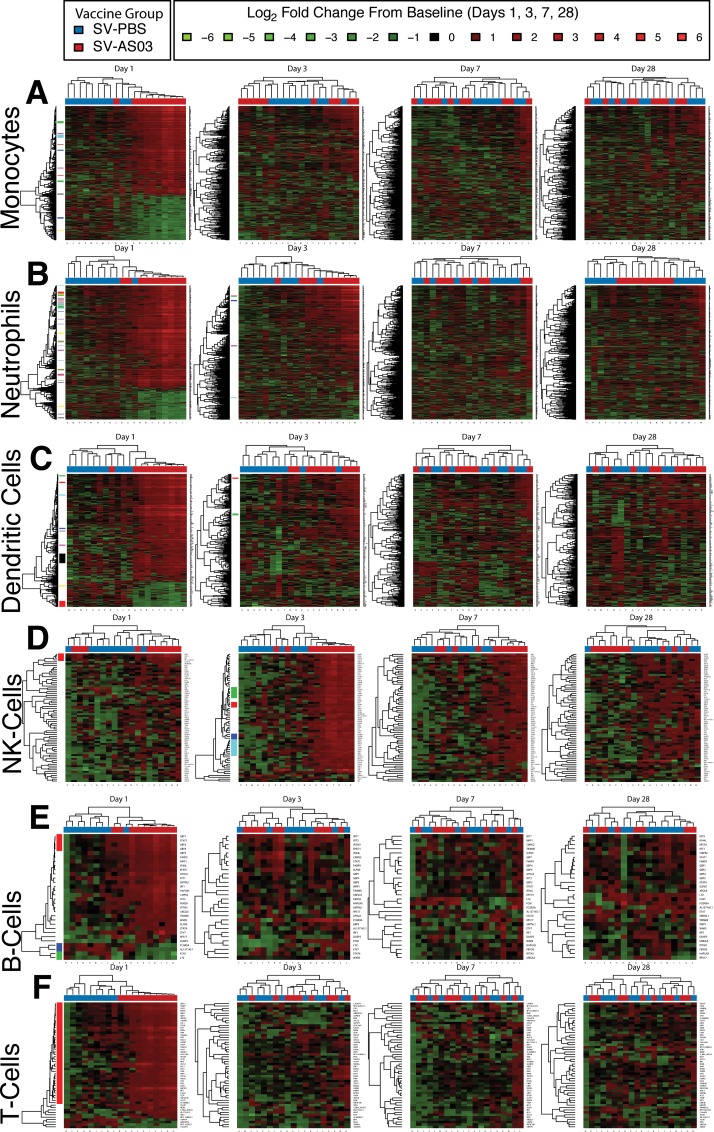
AS03-adjuvanted H5N1 vaccination primarily modulated early innate immune cell gene expression responses. Heatmaps of baseline log_2_ fold changes of DE genes for each cell type at each post-vaccination time point (A: monocytes, B: neutrophils, C: DC, D: NK cells, E: B cells, F: T cells). Dendograms were obtained using complete linkage clustering of uncentered pairwise Pearson correlation distances between log_2_ fold changes. Co-expressed gene cluster membership based on multiscale bootstrapping is highlighted along the gene dendogram on the left side. Cells are color-coded by log_2_ fold change (in red: upregulated from baseline; in green: downregulated from baseline.

To determine the response dynamics of individual and co-expressed DE genes over time, we performed heatmap and gene clustering analyses. With the exception of NK cells, heatmaps summarizing responses (LFCPM) for all subjects by cell type and time point showed that most DE genes were increased from baseline for the SV-AS03 group at day 1 with a decreasing AS03 effect (subject clustering by vaccine group) over time (**Fig 3A–3F**). At day 3, neutrophils and NK cells showed a sustained AS03 effect that was driven by increased responses over baseline for a subset of AS03-modulated genes (**Fig 3B and 3D)**. In contrast to other immune cells and earlier time points, the differential effect observed for NK cells at day 28 was driven by a set of genes with increased responses for the unadjuvanted group and reduced responses in the SV-AS03 group relative to baseline (**Fig 3D**).

**Fig 4:**
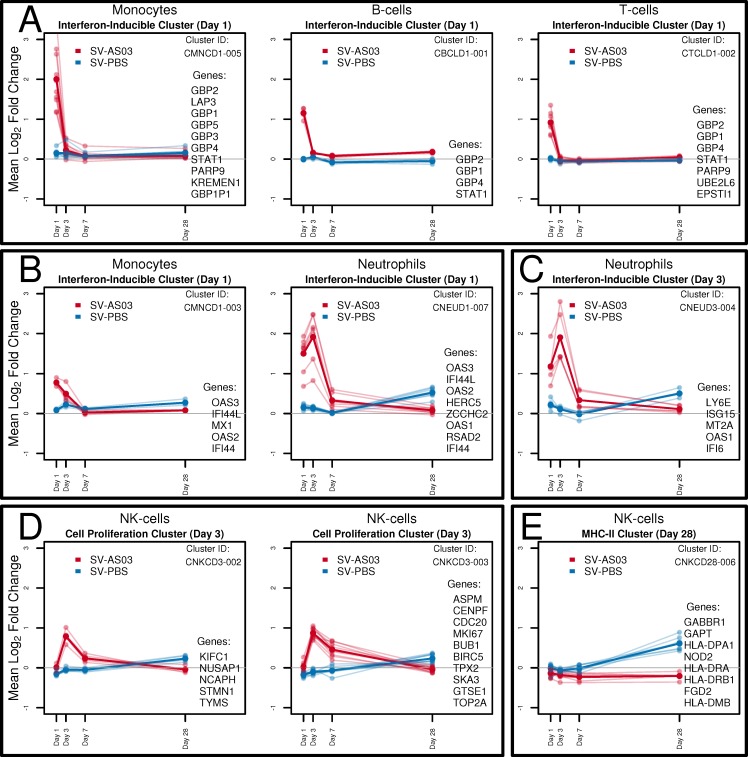
Response time trends of clusters of co-expressed differential genes by vaccine group. Co-expressed gene cluster time trends of baseline log_2_ fold change in LCPM of DE genes by vaccine group. Header indicates cell type, primary functional classification, and time point. Mean log_2_ fold change across cluster genes is drawn in bold. Individual mean gene log_2_ fold changes are plotted in lighter colors. Multiscale bootstrapping was used to determine co-expressed gene clusters. **(A)** Interferon-inducible clusters with increased responses for the AS03-SV group identified in monocytes, B cells and T cells at day 1. **(B)** Interferon-inducible clusters with similar gene composition but different responses for the AS03-SV group in monocytes and neutrophils at day 1. **(C)** Additional interferon-inducible cluster modulated by AS03-SV identified in neutrophils at day 3. **(D)** Cell proliferation-related clusters with increased responses for the AS03-SV group identified in NK cells at day 3. **(E)** MHC class-II-related cluster with increased responses for the PBS-SV group identified in NK cells at day 28.

Gene cluster analysis based on correlations between LFCPM following vaccination at days 1, 3, 7, and/or 28 provided a dynamic view of co-regulated gene responses for each group over time. For the six immune cell types, we identified 100 cell-type specific gene clusters for which we visualized time trends by vaccine group (AS03-adjuvanted or unadjuvated vaccine; **Fig A101-A105 and Tables A58-A72 in S2 Text)**. A selection of clusters that showed marked differences in time trends for the two groups is shown in **Fig 4**. At day 1, several gene clusters containing the transcriptional regulator *STAT1* and interferon inducible genes encoding for guanine binding family proteins (*GBP1-GBP4*) were jointly upregulated in multiple cell types for the SV-AS03 group, but not for the SV-PBS group (**Fig 4A**). For most of these genes, expression returned to baseline levels by day 3. For monocytes and neutrophils, two gene clusters were identified that shared 4 interferon-inducible anti-viral genes (*IF44*, *IFI44L*, *OAS2*, *OAS3*) whose expression is regulated by *STAT1* (**Fig 4B**). Time trends for these clusters in both cell types indicated a similar enhanced response for the SV-AS03 group at day 1. While expression of all cluster members decreased to near-baseline levels after day 1 in monocytes, responses for this cluster peaked at day 3 in neutrophils. A second neutrophil cluster containing antiviral and interferon-inducible genes also peaked at day 3 (**Fig 4C**). AS03-adjuvanted vaccination-induced responses in both cell types were markedly reduced by day 7 and returned to baseline at day 28.

For NK cells, robust gene clusters were identified at day 3 (**Fig 4D**). These clusters showed similar time trends with near baseline levels at day 1 for both SV-AS03 and SV-PBS groups but significantly increased levels for the SV-AS03 group at day 3. The largest of these clusters contained 10 genes, all of which share functions related to cell division/mitosis/DNA replication (*ASPM*, *BIRC5*, *BUB1*, *CDC20*, *CENPF*, *GTSE1*, *MKI67*, *SKA3*, *TOP2A*, *TPX2*). The other cluster contained 5 genes (*KIFC1*, *NUSAP1*, *NCAPH*, *STMN1*, *TYMS*) also known to play a role in cell division, indicating that AS03 increased NK cell division activity at day 3. Expression of genes in these AS03-modulated clusters declined by day 7 and returned to baseline levels at day 28.

At day 28, NK cell gene responses for 8 genes were significantly correlated (*GABBR1*, *NOD2*, *HLA-DRB1*, *HLA-DRA*, *HLA-DPA1*, *HLA-DMB*, *GAPT*, *FGD2*). Time trends showed similar near-baseline average responses for both groups at day 1, 3, and 7 (**Fig 4E**). However, by day 28, the SV-PBS group exhibited higher responses compared to the SV-AS03 group, which exhibited a slight decrease. Four genes in this cluster encode for major histocompatibility complex (MHC) class II proteins indicating that, at day 28, NK cells suggest increased antigen presenting activity in the unadjuvanted vaccine group. Similar trends with increased day 28 responses for the unadjuvanted group were observed for several other gene clusters in NK cells (**Fig A105 in S2 Text**).

### AS03-Adjuvanted Vaccination Triggers Interferon Signaling, Cell Proliferation and Antigen Processing and Presentation

**Fig 5:**
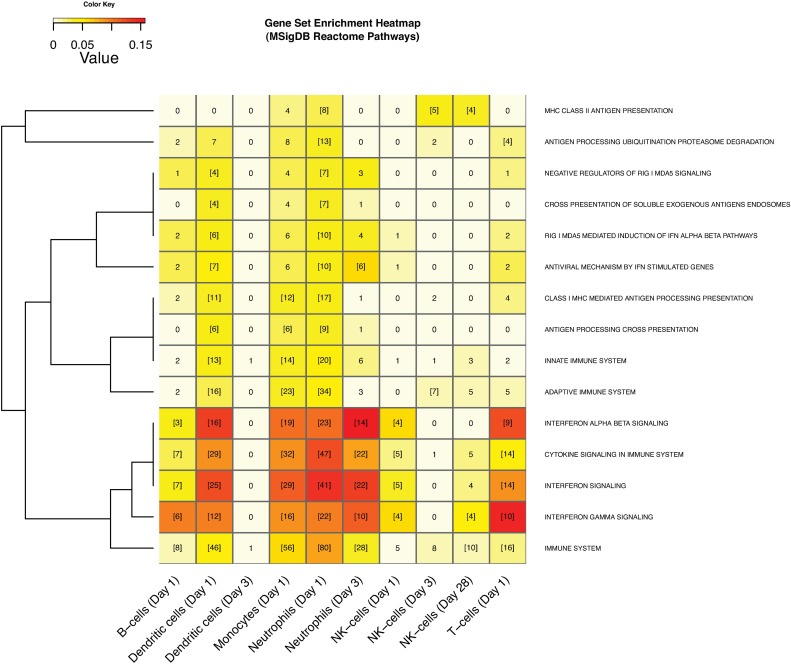
AS03-adjuvanted H5N1 vaccination triggered immune system pathways including interferon signaling, cell proliferation and antigen processing and presentation. Heatmap of MSigDB Reactome pathways enriched in DE genes. Gene sets significantly enriched in at least two conditions (time point or cell type) are shown. Heatmap cell counts represent the number of DE genes in a pathway. Cells with numbers in brackets indicate significantly enriched sets. Cells are color-coded by Jaccard similarity index (in dark-red: high level of similarity, in light-yellow: low level of similarity). The index was calculated by comparing DE genes that had any Reactome annotation with genes in a certain Reactome pathway. Pathways were clustered based on the Jaccard distance between binary enrichment patterns across conditions.

To determine the functional and/or biological categorization of genes modulated by AS03, we performed gene set enrichment analysis based on the Reactome database [43] (**S5 Table**). This analysis showed that DE genes with pathway annotations were primarily involved in known immune system processes (41–100% of annotated differential genes). At day 1, *Immune system* genes were significantly enriched in AS03-modulated genes expressed in all immune cells except for NK cells (**Fig 5**, source: Reactome). *Immune system* genes were also enriched in AS03-modulated genes after adjuvanted vaccine in neutrophils at day 3 and genes primarily upregulated in NK cells in the SV-PBS group at day 28. *Cytokine signaling*, in particular *Interferon signaling*, was significantly enhanced by SV-AS03 in all immune cells at day 1, as well as in neutrophils at day 3. Despite the presence of α-tocopherol in the AS03 preparation, we did not detect any significant associations between AS03-modulated genes and Vitamin E uptake/metabolism-related pathways.

Several human infectious disease pathways were overrepresented in AS03-modulated genes (**Fig A112 in S2 Text,** source: KEGG pathways). Among these, the highest gene overlap was observed for the *Influenza A* pathway. This pathway was significantly enriched in AS03-modulated genes expressed in monocytes (day 1: 14 genes), neutrophils (day 1: 23 genes and day 3: 8 genes), and T-cells (day 1: 5 genes), as well as in unadjuvanted vaccine-modulated genes in NK cells (day 28: 7 genes). *Influenza A* pathway maps colored by treatment effect (LFCD) show a relative upregulation of the *CXCL10* gene encoding for the IP-10 cytokine in the adjuvanted group relative to the unadjuvanted group in monocytes (LFCD = 1.7, 3.3 fold) and in dendritic cells (LFCD = 1.9, 3.7 fold) (**S4 Table, Fig A118-A119 in S2 Text).** Relative upregulation was also observed for Jak-STAT sub-pathway genes in neutrophils from the SV-AS03 group at day 1 and day 3 (**Fig A120-A121 in S2 Text**), and to a lesser extent in monocytes (**Fig A119 in S2 Text**) and dendritic cells (**Fig A118 in S2 Text**). For neutrophils, related innate immune signaling pathways including *RIG-I-like receptor signaling* and *NOD-like receptor signaling* were significantly enriched (**Fig A136-A137 in S2 Text)**. The *complement and coagulation cascade pathway* was significantly enriched in dendritic cells from the adjuvanted group (**Fig A135 in S2 Text**). Genes expressed in NK-cells in the SV-PBS group showed a delayed upregulated response for several genes in the *Influenza A* pathway at day 28 (**Fig A122 in S2 Text**).

**Fig 6:**
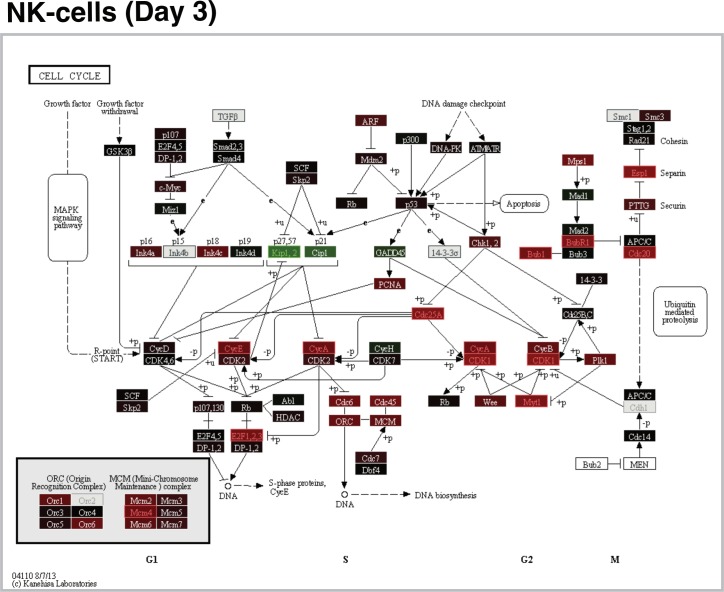
AS03-adjuvanted H5N1 vaccination resulted in increased cell cycle responses in NK-cells relative to non-adjuvanted vaccine 3 days post-vaccination. The pathway map is based on the KEGG Cell cycle pathway. Pathway node color gradient encodes log_2_ fold change difference (LFCD) between vaccine groups (for multi-gene nodes the median LFCD is used). In red: up-regulated for the SV-AS03 group compared to the SV-PBS group, in green: down-regulated for the SV-AS03 group, in black: fold change close to 1, in dark grey: genes filtered out due to low overall expression, light grey: gene missing database mapping, white: non-human gene. DE genes are highlighted using red and green label colors.

*Cell proliferation* was significantly enriched for AS03-modulated genes expressed in monocytes and neutrophils at day 1 and in NK cells at day 3 (**Fig A113 in S2 Text,** source: GO Biological Processes). The majority [28/33 (85%)] of NK cell genes that were differentially expressed at day 3 with any Reactome pathway annotations were involved in *Cell cycle*, in particular, *Cell cycle mitotic* [27/33 (82%)] (**S5 Table**). Inspection of the *Cell cycle* KEGG pathway map showed a consistent differential vaccine group effect (LFCD) pattern for most of these genes with higher fold changes for the SV-AS03 group (**Fig 6**).

**Fig 7:**
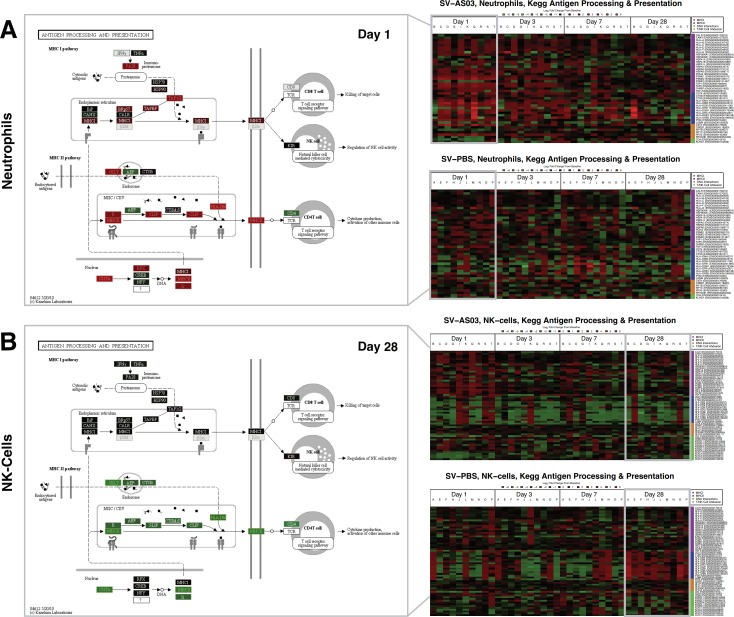
Antigen processing and presentation responses for Neutrophils and NK-cells showed increased MHC-II sub-pathway activity that differed between vaccine groups. **(A)** Neutrophil responses. **(B)** NK-cell responses. To the right: Heatmaps of subject-specific baseline log_2_ fold changes for KEGG Antigen processing and presentation genes across post-vaccination days by vaccine group. Colored in red: up regulated from baseline; colored in green: down regulated from baseline. To the left: KEGG pathway maps for post-vaccination days at which this pathway was significantly perturbed (day 1 for neutrophils, day 28 for NK-cells). Pathway node color gradient encodes log_2_ fold change difference (LFCD) between vaccine groups (for multi-gene nodes the median LFCD was used). In red: increased log_2_ fold change response for the SV-AS03 group relative to the SV-PBS group, in green: decreased log_2_ fold change response for the SV-AS03 group relative to the SV-AS03 group, and vice versa, in black: fold change close to 1, in dark grey: genes filtered out due to low overall expression, light grey: gene missing database mapping, white: non-human gene. DE genes are highlighted using red and green label colors.

At day 1, *MHC class I mediated antigen processing presentation* was enriched in AS03-modulated genes expressed in dendritic cells, monocytes, and neutrophils (**Fig 5**, source: Reactome). Expression of *NLRC5* (NOD-, LRR-and CARD-containing 5), a transactivator of MHC class-I genes, and genes associated with ER translocation machinery (*TAP1* and *TAP2)*, were found to be upregulated in neutrophils, monocytes, and DC at day 1 (**S4 Table**) [44]. Additionally, *ERAP2* and *TPP2*, which are involved in peptide cleavage, were identified as AS03-responsive in neutrophils at day 1 (**S4 Table**). At day 1, genes involved in the *Proteasome* pathway (*PSMB8/9/10*, and *PSME1/2*), in particular, genes related to immunoproteasome subunit formation, were also overrepresented in neutrophils from the SV-AS03 group (**S4 Table**, **Fig A128 in S2 Text,** source: KEGG). *MHC class II antigen presentation* was significantly enriched in SV-AS03-modulated genes expressed in neutrophils at day 1, NK cells at day 3, and in genes primarily upregulated in NK cells from the unadjuvanted group at day 28 (**Fig 5**, source: Reactome). The *Phagosome* pathway was significantly upregulated in monocytes (day 1) and neutrophils (day 1) from the SV-AS03 group and in NK cells from the SV-PBS group (day 28) (**Fig A130-A132 in S2 Text).** Color-coded *Antigen processing and presentation* KEGG pathways (**Fig 7)** show a significant relative upregulation of many MHC II sub-pathway genes in neutrophils from the SV-AS03 group at day 1 and NK cells from the SV-PBS group at day 28. In both cases, this included *CIITA*, the MHC class II transactivator gene and genes encoding for MHC class II HLA molecules (**Fig 7, S4 Table)**. Additionally, Regulatory Factor X subunit, *RFX5*, which acts in conjunction with *CIITA* to induce transcription of MHC class II HLA genes, and *CD74*, which encodes the MHC class-II-associated Ii (invariant chain) essential for targeting MHC II molecules to the endosomal-lysosomal antigen-processing compartment [45, 46] were found to be differentially expressed in response to SV-AS03 in neutrophils at day 1 (**Fig 7A, S4 Table**). For monocytes, although genes encoding for MHC II proteins were significantly differentially expressed at day 1, the AS03 effect for the MHC II sub-pathway was much less pronounced (**Fig A125 in S2 Text**). All of these findings were confirmed when inspecting subject-level response heatmaps (based on LFCPM) for this pathway (**Fig 7A and 7B and Fig A106-109 in S2 Text**). In contrast, NK cells from the SV-AS03 group generally demonstrated a trend for downregulation of MHC-class II genes at all time points at the subject-level. Upregulation of MHC-related genes was not consistently observed in unadjuvanted vaccine recipients in any of these cell types at early time points. However, induction of MHC class II genes was observed in NK cells at day 28 in the unadjuvanted group (**Fig 7B)**. Overall, the observed MHC II effects for neutrophils and NK cells were stronger than the MHC I effects in terms of LFCD and LCPM (**Fig 7A and 7B)**.

### AS03-Responsive Genes are Shared among Immune Cells

**Fig 8:**
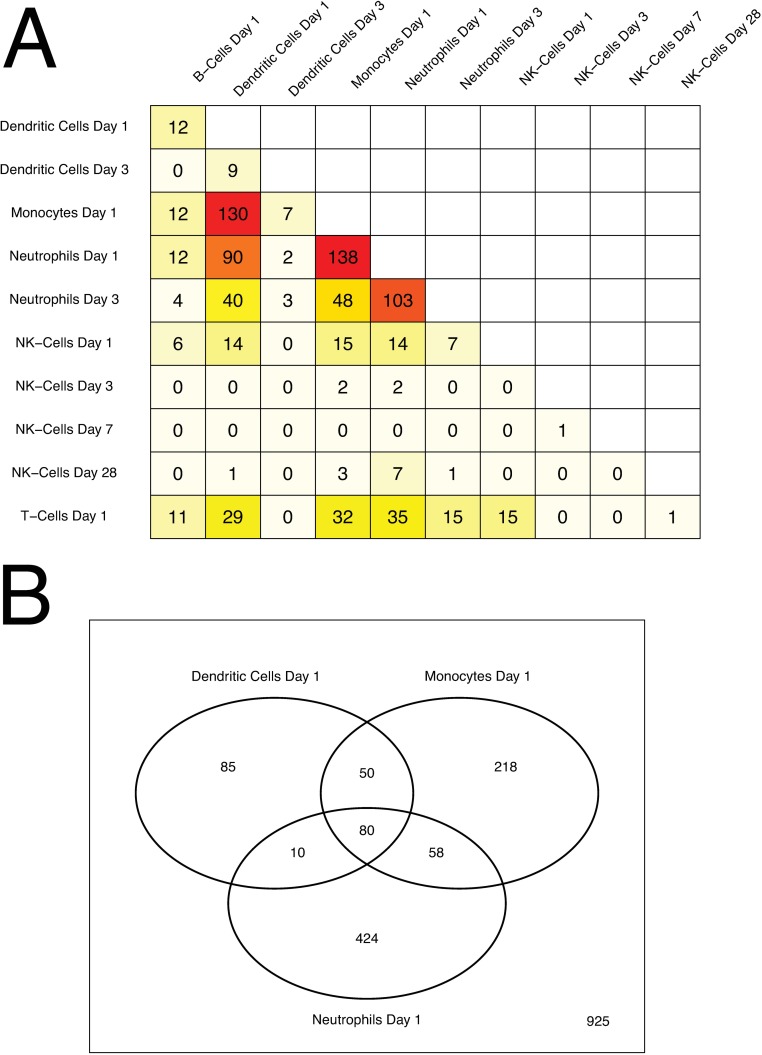
Differential gene overlap between immune cell types. **(A)** Numbers of overlapping DE genes for each cell type and post-vaccination time point combination. Cells are color-coded by number of overlapping genes (dark-red: high overlap, light-yellow: low overlap). **(B)** Venn diagram summarizing DE gene overlap between neutrophils, monocytes, and dendritic cells at day 1.

To determine the amount of overlap in gene expression between cell types, we compared DE lists from each cell type and time point. The highest overlap of AS03-responsive genes among immune cell types was observed for day 1 (**S6 Table, Fig 8**). Expression of 5 genes (*GBP1*, *IRF1*, *STAT1*, *EPSTI1*, *PARP9* [47]) was significantly enhanced by adjuvanted vaccine in all six immune cell types (**S6 Table**). Of these, *GBP1*, *IRF1*, and *STAT1* are known to play a role in innate antiviral immune responses and interferon (IFN) signaling. *IRF1* and *STAT1* are transcriptional regulators that cooperate in the regulation of antiviral genes that constitute antiviral cell states [48, 49] including *GBP1*, which is known to inhibit viral replication [50, 51]. The highest overlap between AS03-modulated genes was observed for monocytes, dendritic cells, and neutrophils. Monocytes and dendritic cells shared 130 genes (32% and 58% of their respective gene sets) while neutrophils and monocytes had 138 genes in common (24% and 34% of their respective gene sets). Neutrophils and dendritic cells shared 90 genes covering 22% and 40% of their significant genes, respectively. Twenty five out of 49 differential T cell genes (51%) and 14 out of 24 differential NK cell genes (58%) overlapped with genes reported for monocytes, neutrophils, and dendritic cells. Genes that were differentially expressed in NK cells at day 3 (70 genes) and day 28 (63 genes) shared only a maximum of 3–7 genes with sets from any of the other cell types, indicating that most of these later responses were unique to NK cells.

**Fig 9:**
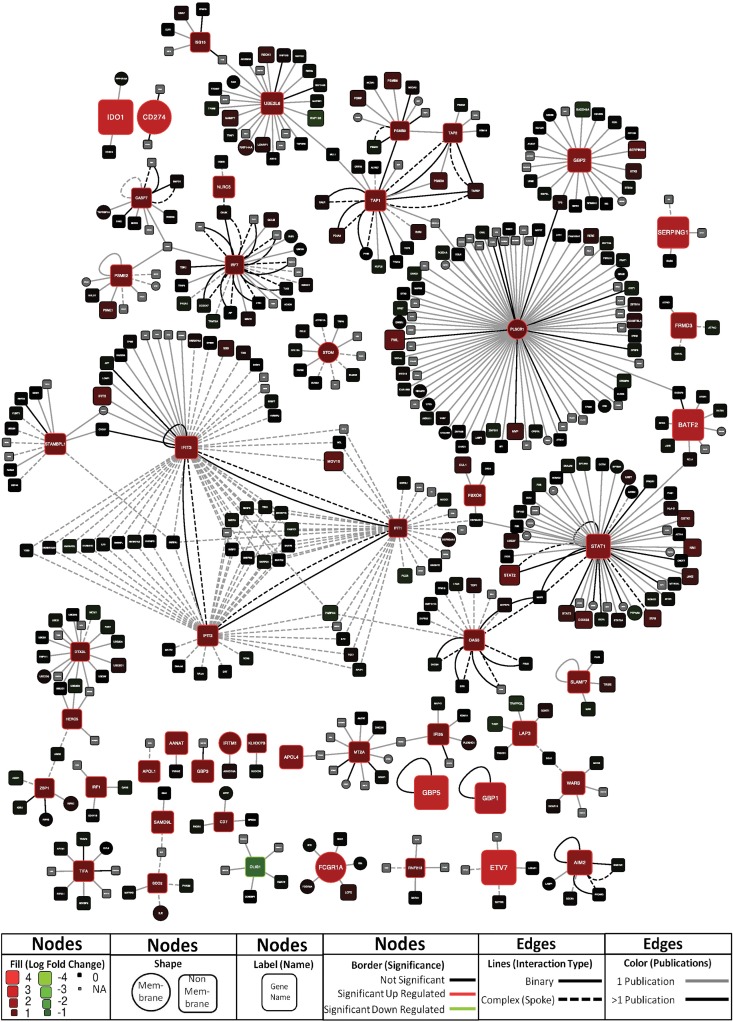
Network analysis of proteins encoded by the 80 core AS03-responsive genes shared between neutrophils, monocytes, and dendritic cells 1 day post-vaccination. Nodes are labeled by corresponding gene name. Edges represent experimentally determined (reported in at least two publications) binary protein-protein interactions or protein complex membership. Color and size of individual nodes indicates the degree of mean log_2_ fold change difference (LFCD) in corresponding gene expression between vaccine groups. Further details are provided as part of the legend within the figure.

Eighty DE genes that we have defined “core AS03-responsive genes” were shared between neutrophils, monocytes and dendritic cells at day 1 (**S7 Table, Fig 8**). Of these genes, at least 30 are known interferon-response genes. Gene set enrichment analysis showed that *Interferon signaling*, *RIG I MDA5 signaling*, *Antigen processing and cross presentation*, as well as *Antiviral mechanisms by IFN stimulated genes* represented the top enriched gene sets for the 80 gene cohort (**S5 Table).** To further characterize these “core AS03-responsive genes”, we analyzed them in the context of their known interacting partners. Network analysis revealed a number of interesting sub-networks (**Fig 9)**. One predominant cluster of nodes is centralized around *PLSCR1*, an IFN-inducible gene that potentially could have a role in antiviral responses through its interaction with TLR, specifically *TLR9*, in dendritic cells [52]. Another large cluster of nodes centers around *IFIT1*, *IFIT2*, and *IFIT3*, IFN-induced genes that have recently emerged as having a wide range of antiviral functions, including binding and sequestering cytoplasmic viral RNA, inhibiting viral protein translation, and possibly playing a role in downstream immune signaling [53]. Another node centers around *TAP1* and *TAP2*, ATP-binding cassette (ABC) transporter genes that are necessary for peptide translocation across the endoplasmic reticulum for association with MHC class I molecules [54, 55], and *PSMB9* [56], a proteasome complex involved in the processing of class I MHC peptides. Smaller networks of interest are present surrounding *OAS3*, an IFN-induced gene product that inhibits cellular protein synthesis and is involved in antiviral functions [57], and *IRF7*, a key transcriptional regulator of type I IFN responses [58]. Networks for AS03-responsive genes identified for each individual immune cell type (monocytes, neutrophils, and dendritic cells) are shown in **Figs A139-A141 in S2 Text.**

### Comparisons to Published Immune Response Transcriptome Studies

To determine which AS03-modulated genes have previously been identified in other global transcriptomic studies of immune responses, we compared our cell-specific data sets with manually curated sets of human and mouse gene immune responses (MSigDB Immunologic Signatures, 1,910 gene sets). These gene sets were primarily microarray-based immunological response studies. Gene sets with the most similarity to our data included the yellow fever viral vaccine (genes upregulated in human PBMCs at Day 7 following vaccination vs. control (GSE13485)) [59], live Newcastle virus infection (genes upregulated at 8h following infection of human dendritic cells (GSE18791)) [60], and *in vitro* stimulation with either IFN-γ (genes upregulated at 24h following stimulation of human fetal microglia macrophages (GSE1432)) [61] or LPS (genes upregulated at 4h following stimulation of human dendritic cells (GSE14000)) [62] (**S8 Table**). For these four immune response gene sets, the highest overlap determined by Jaccard similarity index was reported with genes that were AS03-modulated at day 1 in neutrophils, monocytes, and dendritic cells. Approximately half of the core AS03-responsive genes overlapped with each of these four gene sets (**S8 Table**). Fifteen of the 80 transcripts were not present in any of the significantly enriched Immunologic Signatures gene sets (**S8 Table**). These potentially novel immune system-related genes are comprised of both protein coding and non-coding genes, including pseudogene, lncRNA, and antisense RNA transcripts.

Non-human transcriptomic studies have utilized animal models to investigate the response to adjuvants by sampling injection site muscle tissue and draining lymph nodes for gene modulation following adjuvant treatment [19–21]. The highest overlap with cytokine genes identified by Morel *et al*. 24h after injection of AS03 was observed at day 1 in monocytes, dendritic cells and neutrophils from our study, and included upregulation of CCL2, CCL3, CXCL2, CXCL9, CXCL10 (IP-10), IL-1β, PTGS2, and TNF-α (**Fig A214 in S2 Text)**. Of the 168 genes reported by Mosca *et al*. as differentially expressed in response to MF59 adjuvant treatment, 105 genes mapped to MGI mouse annotations with human orthologs, and 87 genes were uniquely mapped in Ensembl. Approximately 50% of these 87 genes were upregulated and 10% were downregulated in response to AS03-adjuvanted compared to unadjuvanted H5N1 vaccine in our data set (**Fig A215 in S2 Text)**. The highest overlap of these 87 genes with our DE set was detected at day 1 in monocytes (20 genes), neutrophils (18 genes) and dendritic cells (15 genes). Nine primarily interferon-responsive genes (*FCGR1A*, *GBP4*, *IFI44L*, *IFIT2*, *IFIT3*, *IRF7*, *ISG15*, *PARP14*, *RNF213*) overlapped with the 80 core AS03-responsive genes in our study.

### AS03-Adjuvanted Vaccine Gene Responses Correlated with Cytokine and Antibody Responses

**Fig 10:**
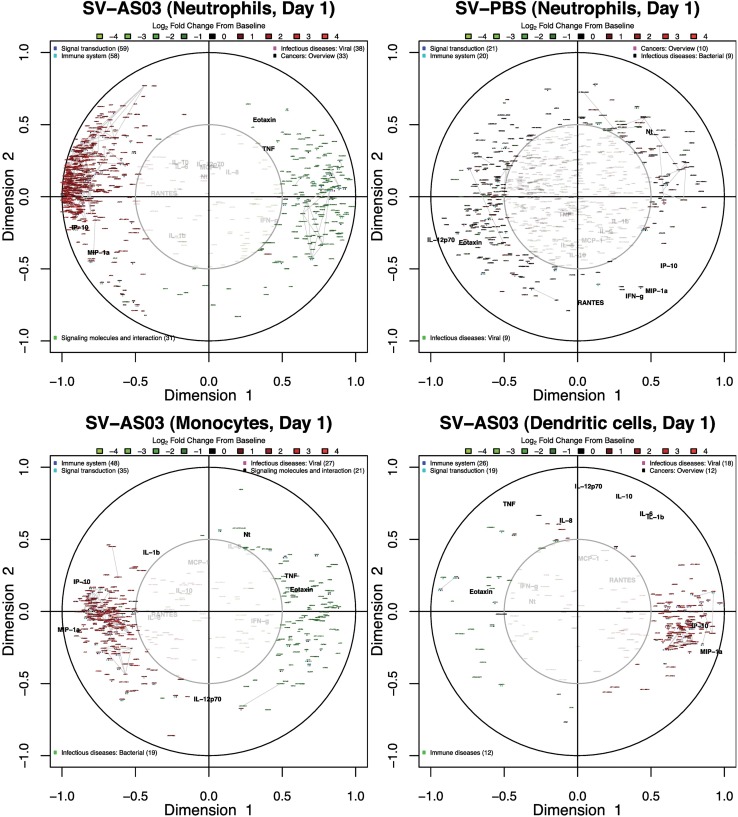
AS03-adjuvanted vaccine gene responses in neutrophils, monocytes and dendritic cells correlated with serum IP-10 and MIP−1α serum cytokine responses 1 day post-vaccination. Canonical correlation plots summarizing key correlation patterns between changes in serum cytokine/antibody responses and gene responses at day 1 in **(A,B)** neutrophils (SV-AS03 and SV-PBS, respectively), **(C)** monocytes (SV-AS03), and **(D)** dendritic cells (SV-AS03). Red: upregulated genes; Green: downregulated genes; +/- gene name prefix: DE gene (yes/no); colored-dots: KEGG pathway class (BRITE level 2); grey lines: co-expressed gene clusters. The serum-based variable set (in black) included baseline log fold changes for 12 serum cytokines as well as baseline log fold changes of antibody titers (HAI: hemagglutination inhibition, Nt: microneutralization inhibition). The strength of the correlation is encoded by the distance from the center of the circle as well as by the angle between variables when viewed as vectors originating from the center. An acute angle (<90°) between variables represents a positive correlation, an obtuse angle (>90°) indicates a negative correlation, while a right angle is observed for zero correlation. Thus, maximum correlation is achieved when variables are closely placed together in the outer circle (or directly opposed on the outer circle.

**Fig 11:**
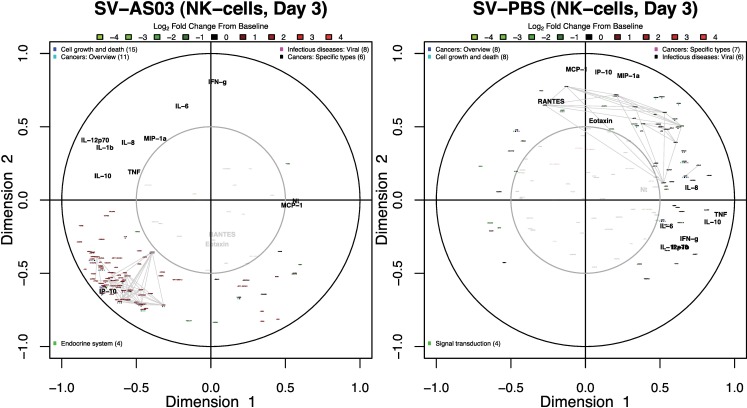
AS03-adjuvanted vaccine NK-cell gene responses correlated with serum IP-10 cytokine responses 3 days post-vaccination. Canonical correlation plots summarizing key correlation patterns between changes in serum cytokine/antibody responses and gene responses at day 3 in NK cells; **(A)** SV-AS03, **(B)** SV-PBS. See **Fig 10**legend for additional information.

To determine if and when cell type-specific gene responses correlated with serological responses, we performed canonical correlation analysis separately for each post-vaccination day. Our results identified strong, positive correlations between upregulated gene responses and changes in IP-10 cytokine concentration in the SV-AS03 group at day 1 in neutrophils, monocytes, and DC (**Fig 10, and Figs A152, A155, and A158 in S2 Text)**. To a lesser extent, these positive correlation patterns were observed also for changes in MIP-1-α concentration. However, unlike IP-10, the serum response for MIP-1-α was not strong enough to induce a noticeable shift in median fold change (**Fig 2D**). Most strikingly, at day 3, changes in IP-10 concentration were strongly correlated with changes in NK cell gene responses in the SV-AS03 but not the SV-PBS group (**Fig 11)**. Identified genes were upregulated from baseline, formed three robust gene clusters, and were primarily involved in cell proliferation (**Fig 4C, Table A68 and Fig A105 in S2 Text**). High positive correlation with increased IP-10 levels were observed for positive changes in *CDC6* and *CDC45* (DNA replication initiators) as well as *E2F* transcription factor expression in NK cells. To assess correlations with antibody response, these models included both changes in Nt (for all post-vaccination days) and HAI (for day 28 as no variation was observed for earlier time points). The analysis did not identify noticeable antibody correlations for the early post-vaccination time points. However, at day 28, several genes were identified that were moderately positively correlated with HAI and Nt responses in the SV-AS03 group including *GPR133* (significantly downregulated for SV-AS03 relative to SV-PBS) and *TMEM176A* (upregulated) in NK cells (**Fig A164 in S2 Text**) as well as miRNA AC027319.1, lncRNA RP11-66D17.3, and processed pseudogene RP11-229P13.2, which were all downregulated in B cells (**Fig A151 in S2 Text).**

### Early Gene Responses Differentiate between Seroprotection Status

To determine which early (day 1) cell type-specific gene responses best predict seroprotection status at day 56 (HAI titer ≥ 1:40), we performed logistic regression analysis. Results are summarized in **S9 Table**. Many of the selected predictive genes are known to play a role in the immune system, in particular, cytokine signaling (source: Reactome) [43]. *STAT1* was identified to predict seroprotection for all six immune cell types. In all cases, increased expression resulted in increased odds for achieving seroprotection. The same was true for *IRF1* in all cell types except monocytes. Increased expression of *PARP*9, which induces the expression of IFN-γ-responsive genes, predicted seroprotection in all cell types except monocytes and B cells. *IRF9*, another transcription factor that mediates signaling by type I IFNs, was a predictor of seroprotection in neutrophil, NK and T cell models and had the highest impact on the odds for achieving seroprotection in the latter two cell types. *FCGR1A* and *FCGR1B*, molecules that are known to bind the Fc region of IgG, were identified as positive predictors of seroprotection in neutrophil and monocyte models. For B cells, increase in expression of a non-functional IG D gene (*IGHD4-23*) had the highest positive impact on the odds for seroconversion, while expression of *CAMSAP1L1*, a gene associated with epilepsy [63, 64], had the highest negative impact. In summary, while predictive models differed between immune cell types, early up-regulation of genes involved in interferon signaling positively impacted the likelihood of achieving later seroprotection. However, given the small sample size, the predictive ability of these immune cell-specific models needs to be validated in the context of future studies.

## Discussion

**Fig 12:**
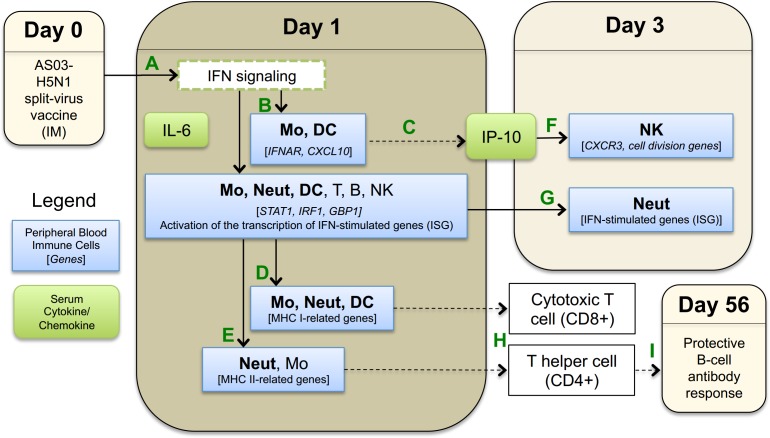
Key molecular immune events observed in peripheral blood cells after AS03-adjuvanted H5N1 split-virus vaccination. **(A)** Intramuscular (IM) administration of AS03-adjuvanted H5N1 vaccine results in interferon (IFN) signaling. **(B)** Activation of IFN-inducible genes, in particular for monocytes (Mo), dendritic cells (DC), and neutrophils (Neut). **(C)** Significant up-regulation of CXCL10, the gene encoding IP-10, in peripheral blood Mo and DC implies that these cells contribute to increased serum IP-10 levels (in addition to local immune cells). **(D)** MHC class I- related antigen presentation genes are upregulated in monocytes Mo, Neut, and DC. **(E)** MHC class II- related antigen presentation genes are upregulated in Neut and, to a lesser extent, in Mo. At day 3, **(F)** IP-10 stimulates expansion of NK cells. **(G)** IFN-stimulated genes remain upregulated in Neut. **(H)** Upregulation of MHC class I and class II molecules stimulate cytotoxic T cells (CD8+) and T helper cells (CD4+), respectively, to initiate transition to the adaptive immune response. **(I)** Generation of protective H5-specific antibodies after a 2-dose vaccine regimen. Solid lines indicate that direct evidence for these events was seen in our serologic or transcriptomic data, while dashed lines indicate no direct evidence was observed.

Adjuvants enhance the immunogenicity of avian influenza vaccines, but the mechanisms by which they drive this function are poorly understood in humans. We conducted a randomized, controlled, Phase I clinical trial to comprehensively analyze transcriptomic responses to an H5N1 vaccine administered either with or without the AS03 adjuvant in six immune cell types isolated from human peripheral blood to begin to dissect cell-specific mechanisms of adjuvant action. Nearly all subjects who received the AS03-adjuvanted vaccine achieved seroconversion and seroprotection at day 56. No subject in the unadjuvanted group achieved seroconversion or seroprotection. In the following, based on our peripheral blood immune cell-specific gene expression and cytokine results, we summarize key molecular immune events that differentiated AS03-adjuvanted H5N1 vaccination from unadjuvanted vaccination (a visual summary is provided in **Fig 12**).

At early time points after the first vaccination, SV-AS03 induced upregulation of early-response genes involved in interferon signaling, some of which were predictive of seroprotection. These differential innate transcriptomic responses were similar to early responses observed after influenza infection [65]. Three of the five genes detected in all six immune cell types at day 1 in the adjuvanted vaccine group were also identified in the study by Loo *et al*. [65], which assessed the role of RIG-I signaling during influenza A viral infection of mouse fibroblasts [65]. The RIG-I signaling pathway is known to alert cells to the presence of viral products, especially viral RNA, and to trigger expression of alpha/beta interferons, which in turn activate interferon inducible genes [65]. In our studies, both RIG-I related pathways and alpha/beta interferon signaling were significantly enriched in core AS03-responsive genes at day 1 (neutrophils, monocytes, and dendritic cells). Additionally, substantial overlap of our data with previously published immunological response gene sets, including vaccination with live attenuated virus vaccines, infection (viral or bacterial), cytokine signaling, and LPS stimulation, suggests that the majority of genes differentially expressed in response to AS03-adjuvanted vaccine are also induced by other immunological events.

One explanation for the early transcriptional signatures that we observed could be that addition of AS03 to vaccine preparations results in a “pseudo-infection” in which induction of cytokine production at the injection site recruits and activates local immune cells. In support of this idea, comparative analysis of the gene sets published by Ramilo *et al*. [66]. and Ioannidis *et al*. [67]. in their studies of infections with influenza and other infectious agents, as well as other studies of live-virus vaccines [59] or stimulation of immune cells with live virus [60], revealed a strong overlap with genes identified in our study at day 1 in neutrophils, monocytes, dendritic cells, and our 80 core AS03-responsive genes, and to a lesser extent with NK cells and T cells (**S8 Table**). Additionally, compared to the gene sets published by Nakaya *et al*. in their systems study of the response to seasonal influenza vaccines [68], our data set shows a stronger overlap with LAIV-induced genes compared to TIV-induced genes, most notably in neutrophils and monocytes at day 1 (**S8 Table**). While we observed that AS03 modulated multiple interferon-inducible genes in all cell types, we did not observe a strong differential signal for the genes encoding α/β interferons themselves in any cell type, as has also been reported recently in a study with AS03-adjuvanted H1N1 vaccine [22]. Nevertheless, genes encoding for the interferon receptor for interferon alpha/beta (IFNAR) were slightly upregulated in monocytes, dendritic cells, and neutrophils from the AS03-adjuvanted vaccine group at day 1 (**Figs A118-A120 in S2 Text**). Additionally, differential results showed that many genes in the JAK/STAT pathway were upregulated in the adjuvanted group, resulting in an upregulation of clusters of interferon inducible genes, particularly in monocytes, dendritic cells, and neutrophils (**Fig 4**, **S4 and S5 Tables,** and **Figs A118-A120 in S2 Text**). Neutrophils showed a prolonged response up to day 3, with a cluster of interferon induced genes reaching their peak at this time (**Fig 4**). For monocytes and dendritic cells, but not neutrophils, SOCS3 [69, 70], a negative regulator that inhibits pro-inflammatory signaling mediated by STAT genes, was significantly upregulated at day 1, potentially explaining the short-lived response in these cell types (**S4 Table** and **Figs A118 and A119 in S2 Text**). Although our data do not indicate whether this activation is direct, indirect, or a combination of both, taken together, the data suggest that AS03 in combination with the split-virus avian vaccine acts to promote an early, innate response that may mimic infection, thus impacting activation and gene expression within neutrophils, monocytes and dendritic cells.

Our data indicate that AS03 induces class I antigen processing and presentation in multiple cell types after vaccination, as expected given that all cell types can potentially present antigen in the context of MHC class I. Additionally, we found that genes involved in controlling MHC class II-restricted antigen processing and presentation pathways were differentially expressed in monocytes and neutrophils following AS03-adjuvanted vaccination relative to unadjuvanted vaccination. Monocytes are known to function as APC under inflammatory conditions. However, our results show that neutrophils also increased expression of MHC class II (HLAs) genes and other molecules associated with antigen processing and presentation following vaccination with AS03-adjuvanted vaccine (**Fig 7**). Evidence is emerging that suggests neutrophils may participate in MHC class-II-restricted antigen processing and presentation; however, their ability to serve as APCs is currently debated [71–76]. Several previous studies administering adjuvanted vaccines in animal models have shown that although neutrophils are recruited to the site of vaccination, take up antigen, and subsequently traffic to the draining lymph nodes, they may be dispensable for generation of protective antibody responses [77, 78]. In fact, the study by Yang *et al*. suggests that neutrophils may act as a “rheostat” for antigen presentation and signaling after the delivery of adjuvanted vaccines by sequestering antigen away from professional APCs and thus dampening the level of antigen presentation. It is important to note that neither of these earlier studies utilized AS03 as an adjuvant, although Calabro *et al*. utilized MF59 [77], another oil-in-water adjuvant. Thus, increased MHC activity in neutrophils in response to AS03-adjuvanted H5N1 vaccination could function to either increase or decrease antigen presentation to antigen-specific T cells, depending on whether neutrophils play an active or regulatory role in MHC class II antigen processing and presentation. Additional studies are needed to determine the potential role that neutrophils may play in antigen presentation following AS03-adjuvanted H5N1 vaccination[71].

NK cells demonstrated increased expression of cell activation genes in the SV-AS03 vaccine group at day 3, which likely implies a more rapid expansion of these lymphocytes in response to the adjuvanted vaccine (**Fig 6**). These gene expression changes in NK cells were highly correlated with changes in serum IP-10 concentration, with the strongest correlations observed for *CDC6* and *CDC45*. *CDC6* expression is limited to proliferating cells and is regulated by *E2F* transcription factors, which were also upregulated and correlated with IP-10 [79]. These results imply a causal relationship in which AS03 in combination with the split-virus triggers IP-10-mediated NK cell proliferation via upregulation of co-expressed gene modules. IP-10 is a known chemoattractant and activator of NK cells [80–84]. Accordingly, expression of the receptor for IP-10 (*CXCR3*) was increased in NK cells from the AS03-adjuvanted vaccine group (**Fig A216 in S2 Text**). Additionally, while initially identified as MHC class-II related genes, the kinesins (*Reactome* pathway; *KIF2C*, *KIF15*, *KIF18A*, *KIF20A*, *KIF23*) modulated by AS03 at day 3 in NK more likely play a role in cell division, as multiple modules of proliferation- and cell division-related genes were identified in this cell type at this time point. These data suggest that NK cells were activated in response to SV-AS03 vaccination. However, NK cells typically function in the context of infection, where they are recruited to the site of infection and actively kill infected cells. The role that these cells might play in response to adjuvanted avian influenza vaccine remains to be determined.

NK cells from the SV-PBS group exhibited a significant increase in expression of MHC class-II related genes at day 28, the only significant increase in MHC class-II related genes in any cell type in the unadjuvanted vaccine group at any time point (**Fig 7**). While not considered classical APC, NK cells have been previously reported to upregulate MHC class-II and present antigen to T cells [85–87]. Subjects receiving unadjuvanted vaccine did not achieve seroprotection; however, H5-specific antibody titers were increased relative to baseline, suggesting that low-level immune induction occurred. In support of this theory, Gillard *et al*. have previously shown that a 2-dose prime regimen with 3.75mcg of split-virus H5N1 vaccine (A/Vietnam/1194/2004) without adjuvants led to cross-reactive seroprotection following a second 2-dose boost regimen with a heterologous 3.75mcg split-virus unadjuvanted H5N1 vaccine (A/Indonesia/5/2005) administered 6 months later [88]. Accordingly, the monocyte and neutrophil time trend responses that we observed to accompany early upregulation of MHC class-II responses in those cell types in the SV-AS03 group were also increased slightly at day 28 in the unadjuvanted vaccine group (**Fig 4**). These data suggest that following unadjuvanted low-dose H5N1 vaccine, the cell types and mechanisms utilized for class-II antigen presentation may be different, and the magnitude and kinetics of the immune response delayed, compared to that obtained following AS03-adjuvanted vaccine. These observations will need to be confirmed.

The cytokine findings, similar to transcriptomic data, indicate that AS03 modulated early innate responses (day 1 and day 3). IP-10 and IL-6 showed a significant differential response between vaccine groups, with both cytokines displaying higher responses for the SV-AS03 group at day 1, consistent with previous studies [19, 22]. IP-10 also sustained an increased, albeit a weaker, response at day 3 post-vaccination. Consistent with serum cytokine levels, monocytes and dendritic cells in the SV-AS03 group showed significant upregulation of *CXCL10*, the gene encoding IP-10, at day 1 (**S4 Table**). The lack of a differential gene response in any cell type and the low concentration of IFN-γ in serum cytokine measurements at days 1, 3, and 7 after vaccination implies that either this cytokine did not play a role in modulating early innate responses, that the IFN response was driven by type I IFNs, or, more likely, that the IFN response occurred transiently and/or locally and was not captured by our study time points/blood immune cell selection [19]. Our immune cell-specific predictive models of seroprotection did show that an early upregulation of genes related to IFN signaling increased the likelihood of later seroprotection. In this regard, an increase in *STAT1* expression was predictive of seroprotection for all six immune cell types.

Additional studies will be required to confirm the observed immune cell responses, implicated pathways, and predictive ability of the presented models. Additionally, as both vaccine groups received the H5N1 split-virus, a distinction between immune responses against AS03 and split-virus antigens was not possible. The small sample size of 10 subjects in each group also limits the generalizability of our results. However, our study highlights the strengths of assessing transcriptomic responses of multiple individual immune cell types in parallel. By including additional baseline measurements, we increased the robustness of fold change estimates. The use of RNA-Seq enabled more sensitive determination of responses at the transcriptional level than is possible using microarrays. Furthermore, the granular information that we obtained might have been lost by an assessment of mixed populations of immune cells such as whole blood or PBMC. For example, our cell-based analysis clearly shows that SV-AS03 vaccination induced stronger MHC-II representation-related gene responses in neutrophils compared to DC and monocytes (**Figs A106-108 in S2 Text**), and NK cell-specific proliferation (**Fig 6)**. Other than the absence of plasma cell-related gene signatures in our data, likely reflecting the lack of pre-exposure levels in our subjects to H5N1 influenza, which has not been detected in humans in the US [2], our findings generally overlap with the recently published study by Sobolev et al., which also noted an increase in blood monocytes, as well as interferon- and antigen presentation-related gene expression signals 24h after immunization with the AS03-adjuvanted H1N1 pandemic vaccine [22]. However, by assaying individual cell types, our study identified more precisely the cellular sources of these and other gene signatures. In summary, our findings demonstrate that application of a cell-based systems approach reveals potentially novel mechanisms of action for the AS03 adjuvant, including up-regulation of antigen processing and presentation genes in neutrophils and induction of cellular proliferation processes that correlated with serum IP-10 levels in NK cells at early time points after vaccination, and provides a greater understanding of the biological functions underlying immune responses to vaccination.

## Supporting Information

S1 Study Protocol (PDF)

S1 Table Antibody, cytokine, and immune cell measures by subject and time point. (XLSX)

S2 Table RNA-Seq fragment counts for human genes (Ensembl, Version 63) by subject, immune cell type, and time point. Exclusion of known ribosomal, transfer, and mitochondrial RNA genes and genes located on the X or Y chromosomes is indicated in the ‘Excluded’ field.
(BZ2)

S3 Table RNA-Seq moderated log_2_ fragment counts per million (LCPM) for human genes (Ensembl, Version 63). Known ribosomal, transfer, and mitochondrial RNA genes, genes located on the X or Y chromosomes (indicated in **S8 **Table), and genes that did not pass the low-expression cut-off by subject, immune cell, and time point, were excluded. LCPM for outlying samples are not included.
(ZIP)

S4 Table Significantly differentially expressed genes (RNA-Seq, SV-AS03 vs. SV-PBS). Genes are sorted by descending likelihood ratio statistic. Gene model summaries and annotations are based on Ensembl Version 63 (June 2011).
(XLSX)

S5 Table Significantly enriched MSigDB Reactome pathways. Gene sets are sorted by FDR and Jaccard similarity index.
(XLSX)

S6 Table Intersection of significantly differentially expressed gene sets between cell types and time points. (XLSX)

S7 Table Core AS03-responsive gene set (dendritic cells, monocytes, and neutrophils day 1). (XLSX)

S8 Table Significantly enriched MSigDB Immunological Signature gene sets. Gene sets are sorted by FDR and Jaccard similarity index.
(XLSX)

S9 Table Combination of genes differentiating between seroprotection status (HAI ≥ 1:40 vs. HAI < 1:40). Sorted by descending logistic regression coefficient. Gene model summaries and annotations are based on Ensembl Version 63 (June 2011).
(XLSX)

S1 Text CONSORT checklist. (DOC)

S2 Text Supplemental Information: Appendix. (PDF)
