# Supplementary material for: Cell-Based Systems Biology Analysis of Human AS03-Adjuvanted H5N1 Avian Influenza Vaccine Responses: A Phase I Randomized Controlled Trial
Source: PLoS One. 2017 Jan 18;12(1):e0167488. doi: 10.1371/journal.pone.0167488 (PMC5242433; doi:10.1371/journal.pone.0167488)
Supplement: S1 Study Protocol — (PDF) [file pone.0167488.s001.pdf]

A Randomized, Double-Blinded, Controlled, Phase I Study in Healthy Adults to Assess the Safety, Reactogenicity, and Immunogenicity of Intramuscular Subvirion Inactivated Monovalent Influenza A/H5N1 Virus Vaccine Administered With and Without AS03 Adjuvant: Standard & Systems Biology Analyses

[redacted]

[redacted]

[redacted]

[redacted]

[redacted]

[redacted]

[redacted]

[redacted]

[redacted]

[redacted]

[redacted]

[redacted] ____________________ [redacted] ____________________

[redacted]

Table of Contents

Statement of Compliance i
Signature Page ii
List of Abbreviations vi
Protocol Summary 1

1 Key Roles 6
2 Background Information and Scientific Rationale 8
2.1 Background Information 8
2.1.1 Public Readiness and Emergency Preparedness Act 12
2.2 Rationale 13
2.3 Potential Risks and Benefits 14
2.3.1 Potential Risks 14
2.3.2 Known Potential Benefits 17
3 Objectives 19
4 Study Design 22
5 Study Population 25
5.1.1 Subject Inclusion Criteria 25
5.1.2 Subject Exclusion Criteria 26
6 Enrollment/Randomization/Masking Procedures 29
7 Study Procedures/Evaluations 30
7.1 Clinical Evaluations 30
7.2 Concomitant Medications/Treatments 31
7.3 Laboratory Evaluations 31
7.3.1 Clinical Laboratory Evaluations 31
7.3.2 Immunogenicity Evaluations 31
7.3.3 Special Assays or Procedures 32
7.3.4 Specimen Preparation, Handling, and Shipping 34
8 Study Schedule 35
8.1 Screening and Enrollment 35
8.1.1 Day -28, Visit 00A 35
8.1.2 Day -14, Visit 00B 36
8.1.3 Day 0, Visit 01, Dose 1 36
8.2 Follow-Up 37
8.2.1 Day 1, Visit 02 37
8.2.2 Day 3, Visit 03 37
8.2.3 Day 7, Visit 04 38
8.2.4 Day 28, Visit 05, Dose 2 38
8.2.5 Day 30, Visit 05a, Telephone Call 39
8.2.6 Day 35 Visit 06 39

8.2.7 Day 56, Visit 07 40
8.2.8 Day 208, Visit 08, Follow-up Phone Call 40
8.2.9 Day 393, Visit 09, Follow-up Phone Call 40
8.3 Early Termination Visit 41
9 Study Investigational Product 42
9.1 Study Product Acquisition 42
9.1.1 Formulation, Packaging and Labeling 42
9.2 Product Storage and Stability 42
9.4 Accountability Procedures for the Study Intervention/Investigational Product(s) ...43
10 Assessment of Scientific Objectives 44
10.1 Specification of the Appropriate Outcome Measures 44
10.1.1 Primary Outcome Measures 44
10.1.2 Secondary Outcome Measures 44
10.1.3 Tertiary Outcome Measures 44
10.1.4 Exploratory Outcome Measures 45
10.2 Methods and Timing for Assessing, Recording, and Analyzing Appropriate Outcome Measures 45
10.3 Modification and Discontinuation of Study Intervention/Investigational Product for a Subject 46
10.3.1 Dose/Schedule Modifications for a Subject 46
10.3.2 Criteria for Discontinuation of Study Intervention/Product for Withdrawal of a Subject 46
10.3.3 Deferral of Second Dose of Vaccine 47
11 Assessment of Safety 48
11.1 Specification of Safety Parameters 48
11.2 Methods and Timing for Assessing, Recording, and Analyzing Safety Parameters 48
11.2.1 Adverse Events 48
11.2.2 Reactogenicity 50
11.2.3 Serious Adverse Events 52
11.2.4 Procedures to be Followed in the Event of Abnormal Laboratory Test Values or Abnormal Clinical Findings 53
11.3 Reporting Procedures 53
11.3.1 Serious Adverse Events 53
11.3.2 Regulatory Reporting For Studies Conducted Under DMID- Sponsored Investigational New Drug (IND) Application 54
11.3.3 Reporting of Pregnancy 54
11.4 Type and Duration of Follow-up of Subjects after Adverse Events 55
11.5 Halting Rules 55
11.6 Safety Oversight (ISM plus SMC) 56
12 Clinical Monitoring Structure 58
12.1 Site Monitoring Plan 58

13 Statistical Considerations 59
13.1 Introduction 59
13.2 Study Objectives 59
13.3 Study Population 61
13.4 Study Design 62
13.5 Study Outcome Measures 63
13.6 Study Hypotheses 64
13.7 Sample Size Considerations 64
13.8 Subject Enrollment and Follow-up 64
13.9 Interim Analysis 65
13.9.1 Safety Review 65
13.9.2 Immunogenicity Review 65
13.10 Final Analysis Plan 66
13.10.1 Safety 66
13.10.2 Immunogenicity 66
13.10.3 Systems Biology 66
14 Data Collection Forms and Access to Source Data/Data Collection Forms .. 68
15 Quality Control and Quality Assurance 69
16 Ethics/Protection of Human Subjects 70
16.1 Ethical Standards 70
16.2 Institutional Review Board 70
16.3 Informed Consent Process 70
16.4 Exclusion of Women, Minorities, and Children (Special Populations) 71
16.5 Subject Confidentiality 71
16.6 Study Discontinuation 72
16.7 Costs, Subject Compensation, Research Related Injuries 72
16.8 Future Use of Stored Specimens 73
17 Data Handling and Record Keeping 74
17.1 Data Management Responsibilities 74
17.2 Data Capture Methods 74
17.3 Types of Data 75
17.4 Timing/Reports 75
17.5 Study Records Retention 75
17.6 Protocol Deviations 75
18 Publication Policy 77
19 Literature References 78

APPENDICES
Appendix A: Schedule of Events

List of Abbreviations

A/H5N1	Influenza A Virus of the H5N1 Subtype
AE	Adverse Event
BP	Blood Pressure
BPM	Beats Per Minute
CBA	Cytometric Bead Array
CD	Cluster of Differentiation
CFR	Code of Federal Regulations
CMI	Cell-Mediated Immunity
CRF	Case Report Form
DHHS	Department of Health and Human Services
DMID	Division of Microbiology and Infectious Diseases, NIAID, NIH
eCRF	Electronic CRF
FDA	Food and Drug Administration
FDR	False Discovery Rate
GBS	Guillain-Barré Syndrome
GSK	GlaxoSmithKline Biologicals
GCP	Good Clinical Practice
GMT	Geometric Mean Titer
H1N1	Influenza A Virus of the H1N1 Subtype
HA	Hemagglutinin
HAI	Hemagglutination Inhibition Assay
HEENT	Head, Eyes, Ears, Nose, and Throat
HIPAA	Health Insurance Portability and Accountability Act
ICH	International Conference on Harmonisation
IDES	Internet Data Entry System
IL	Interleukin
IM	Intramuscular
IND	Investigational New Drug application
INF	Interferon
IRB	Institutional Review Board
ISM	Independent Safety Monitor
iTRAQ	Isobaric Tags for Relative and Absolute Quantitation
mcg	Micrograms
MedDRA®	Medical Dictionary for Regulatory Activities
MOP	Manual of Procedures
mRNA	messenger RNA
ncRNA	Noncoding RNA
NIAID	National Institute of Allergy and Infectious Diseases, NIH
NIH	National Institutes of Health
NK	Natural Killer
Nt	Neutralizing Antibody Titer
PBMC	Peripheral Blood Mononuclear Cell
PBS	Phosphate Buffered Saline
PMN	Polymorphonuclear Leukocyte

RNA-Seq	next generation RNA Sequencing
RPKM	Reads Per Kilobase of exon model per Million mapped reads
SAE	Serious Adverse Event
SMC	Safety Monitoring Committee
US	United States
VTEU	Vaccine and Treatment Evaluation Unit Site(s)

Protocol Summary

Title: A Randomized, Double-Blinded, Controlled, Phase I Study in Healthy Adults to Assess the Safety, Reactogenicity, and Immunogenicity of Intramuscular Subvirion Inactivated Monovalent Influenza A/H5N1 Virus Vaccine Adminstered With and Without AS03 Adjuvant: Standard & Systems Biology Analyses

Phase: I

Population: Approximately 20 healthy male and non-pregnant female subjects aged 18 to 49 years old, inclusive, in the United States (US)

Number of Sites: One: Vanderbilt University

Study Participant Duration: Approximately 14 months

Study Duration: Approximately 18 - 24 months

Description of Agent: Two doses of a subvirion inactivated monovalent influenza A/H5N1 (Hemagglutinin (HA) of A/Indonesia/05/2005) virus vaccine manufactured by Sanofi Pasteur, delivered intramuscularly as 3.75 micrograms (mcg) per dose, with the AS03 adjuvant manufactured by GlaxoSmithKline Biologicals (GSK) or PBS diluent manufactured by Sanofi Pasteur

Objectives:

Primary Objectives:

- To examine the safety and tolerability of subvirion inactivated A/H5N1 virus vaccine mixed with the AS03 adjuvant in healthy adults.
- To determine the potential for AS03 adjuvant to enhance the early development of vaccine-specific hemagglutination inhibition (HAI) antibody titers and neutralization (Nt) antibody titers approximately 1, 3, 7, and 28 days following receipt of the first dose of subvirion inactivated A/H5N1 virus vaccine administered at 3.75 mcg per dose in healthy adults.

Secondary Objective:

- To determine the potential for AS03 adjuvant to enhance the development of vaccine-specific hemagglutination inhibition (HAI) antibody titers and neutralization (Nt) antibody titers approximately 28 days following receipt of the second dose of subvirion inactivated A/H5N1 virus vaccine administered at 3.75 mcg per dose in healthy adults.

Tertiary Objectives:

- To identify differentially expressed messenger RNAs (mRNA), noncoding RNAs (ncRNA), RNA splice junctions, and nonsynonymous base substitutions in human immune blood cells as determined by RNA-Seq analysis at Days 1, 3, 7, and 28 after vaccination with the first dose of A/H5N1 virus vaccine with and without AS03 adjuvant.
- To identify differentially expressed cellular proteins in human immune blood cells as determined by shotgun proteomics at Days 1, 3, 7, and 28 after vaccination with the first dose of A/H5N1 virus vaccine with and without AS03 adjuvant.

Endpoints:

Primary Endpoints:

- Occurrence of vaccine-associated serious adverse events (SAEs) from the time of first vaccination through 13 months after the first vaccination.
- Occurrence of solicited local and systemic reactogenicity in the 8 days (Days 0-7) after each vaccination.
- GMT of HAI antibody, proportion of subjects achieving a serum HAI antibody titer of 1:40 or greater, and frequency of 4-fold or greater increases of HAI antibodies in each group against the subvirion inactivated A/H5N1 virus vaccine approximately 1, 3, 7 and 28 days after receipt of the first dose of vaccine.
- GMT of neutralizing antibody, proportion of subjects achieving a serum neutralizing antibody titer of 1:40 or greater, and frequency of 4-fold or greater increases of neutralizing antibodies in each group against the subvirion inactivated A/H5N1 virus vaccine approximately 1, 3, 7 and 28 days after receipt of the first dose of vaccine.

Secondary Endpoints:

- GMT of HAI antibody, proportion of subjects achieving a serum HAI antibody titer of 1:40 or greater, and frequency of 4-fold or greater increases of HAI antibodies in each group against the subvirion inactivated A/H5N1 virus vaccine 28 days after receipt of the second dose of vaccine (approximately Day 56).
- GMT of neutralizing antibody, proportion of subjects achieving a serum neutralizing antibody titer of 1:40 or greater, and frequency of 4-fold or greater

increases of neutralizing antibodies in each group against the subvirion inactivated A/H5N1 virus vaccine 28 days after receipt of the second dose of vaccine (approximately Day 56).

Tertiary Endpoints:

- RNA, RNA splice junctions, nonsynonymous base substitutions, or protein expression in blood immune cells at Days 1, 3, 7, and 28 after the first vaccination that differentiates one dose of an AS03-adjuvanted H5N1 vaccine compared to one dose of the unadjuvanted H5N1 vaccine.
- RNA, RNA splice junctions, nonsynonymous base substitutions, or protein expression in blood immune cells at Days 1, 3, 7, and 28 after the first vaccination that correlates with the occurrence of solicited local and systemic reactogenicity 8 days (Days 0-7) after the first dose of an AS03-adjuvanted H5N1 vaccine or the first dose of the unadjuvanted H5N1 vaccine.

Exploratory Endpoints:

- Exploration of unsolicited adverse events (AEs) reported in the 28 days after each vaccination.
- Occurrence of new-onset chronic medical conditions through 13 months after the first vaccination.
- Development of serum antibody responses against antigenically drifted variants of the H5N1 virus.
- Development of a systems biology model of early human immune response of AS03-adjuvanted and unadjuvanted A/H5N1 virus vaccine that integrates quantitative changes in RNA and protein expression with the innate and humoral immune responses as determined by cytokine/chemokine levels, hemagglutination inhibition (HAI), neutralizing antibody titers (Nt), and immune cell activation status, respectively.
- Identification of early RNA and protein expression signatures in human immune cells that indicate successful immunological response and protection against the influenza pathogen.

Description of Study Design:

This is a single center, randomized, double-blinded, controlled, Phase I, small targeted prospective study in healthy male and non-pregnant female subjects, 18 to 49 years old, inclusive, designed to determine the safety, reactogenicity, and immunogenicity of an intramuscular subvirion inactivated monovalent influenza A/H5N1 (HA of A/Indonesia/05/2005) virus vaccine manufactured by Sanofi Pasteur administered at 3.75 mcg per dose given with or without AS03 adjuvant manufactured by GSK. In the study, each subject will receive two doses administered 28 days apart. This study will use a systems biology approach to assess the human early gene and protein signatures expressed at Days 1, 3, 7, and 28 after the first

vaccination. The systems data will be integrated with immunogenicity and reactogenicity data to develop a systems model of the human immune response to A/H5N1 vaccine with or without AS03 adjuvant.

The proposal will use venous blood samples and subject data collected from a total of twenty H5N1 vaccinated subjects divided into two equal groups. The first group (n=10) will be vaccinated with 3.75µg of H5N1 hemagglutinin plus AS03. The second group (n=10) will be vaccinated with 3.75µg of H5N1 hemagglutinin alone. Venous blood samples (approximately 100 mL) will be collected from all subjects at Days -28, -14, and 0 (prior to vaccination) and at Days 1, 3, 7, and 28 (prior to the second dose of vaccine) after the first vaccination for systems biology studies (cytokine/chemokine profiles, immune activation status, and transcriptome and proteome profiles). Serologic assessment (HAI and Nt) will also be conducted at each of these visits except on Days -28 and -14. Using a systems biology approach, we will identify and quantify the early changes in the subject's serum cytokine/chemokine levels, and immune cell activation status, and the whole transcriptome and proteome of the major immune cells after the first vaccination. The venous blood sample collected at Days -28, -14, and 0 will be used to measure baseline serum chemokine and cytokine levels, immune activation status, and the whole transcriptome and proteome levels of the major blood immune cells prior to the first vaccination. In addition, at the Day 28 follow-up visit, subjects will be given a second dose of the same vaccine that they received initially, since two doses of vaccine are required to generate a protective immune response in the majority of vaccines. After the second dose of vaccine is given, a final blood sample (approximately 10 mL) will be collected for serologic assessment (HAI and Nt) only at 28 days after the second vaccination (Day 56). The systems biology studies will be conducted only after the first vaccine dose since we are seeking to determine the initial responses associated with vaccine and adjuvant, and not the booster responses. A urine sample will be collected from each subject at Days -28, -14, 0, 1, 3, 7, and 28 for future research.

At screening (Day -28) or on the day of, but prior to, first vaccination (Day 0), vital signs (oral temperature, pulse and blood pressure; oral temperature only at Day 0 if all vital signs collected at screening), height and weight will be collected and a complete physical exam (without genital and rectal exam) will be performed. Targeted physical exams may be performed if indicated based on review of interim health status prior to each vaccination and at the Days 1, 3, 7, 35 and 56 clinic visits. Vaccine reactions will be assessed for at least 20 minutes following each vaccination on Days 0 and 28, and continue through 8 days following each vaccination. The vaccination site will be examined at the end of the 20-minute observation period following each vaccination and in the clinic on Days 1, 3, 7 and 35. Subjects will be asked to record oral temperature, solicited vaccine reactions and any unsolicited AE/SAEs on a memory aid for 8 days following each vaccination (Days 0-7 and Days 28-35, respectively). Subjects will be encouraged to take their temperature around the same time each day. Each subject will be seen in the clinic on Days 1, 3, 7, 28, 35 and 56 to review AE/SAEs, concomitant medications, health status and the events recorded on the memory aid. Each subject will be contacted by telephone 1-3 days after the second vaccination (Day 30) to review the memory aid, AE/SAEs

and concomitant medications. Subjects will also be contacted by telephone at 6 months and 1 year after the second vaccination (Days 208 and 393, respectively) to review serious adverse events and new-onset chronic medical conditions. Based on this information, subjects may be asked to return to clinic to be evaluated.

The duration of the study for each subject will be approximately 14 months.

See Appendix A for Schedule of Procedures/Evaluations.

A Safety Monitoring Committee (SMC) will be convened by DMID to review participant safety data which may include solicited and unsolicited AE/SAEs, concomitant medications, and any physical examinations. Safety data will be reviewed by the SMC per the SMC charter for this study. As an outcome of each review/meeting, the SMC will make a recommendation at that time as to the advisability of proceeding with vaccinations, and to continue, modify or terminate the study.

[redacted]

[redacted]

[redacted]

[redacted]

[redacted]

[redacted]

[redacted]

[redacted]

[redacted]

[redacted]

2 BACKGROUND INFORMATION AND SCIENTIFIC RATIONALE

2.1 Background Information

Influenza remains a formidable global health threat, as evidenced by the emergence of novel influenza A virus strains in human populations in recent years (including subtypes A/H5N1, H7N7, and H9N2 viruses). Three pandemics occurred during the 20th century, most notably the 1918 influenza A/H1N1 pandemic, which is estimated to have caused 40 million deaths (1). Excess mortality, high morbidity, and social disruption were also noted during the 1957 influenza A/H2N2 and the 1968 influenza A/H3N2 pandemics (2). Most recently, in April 2009, a novel influenza A/H1N1 virus of swine-origin emerged in humans causing the first influenza pandemic of the 21st century. This has underscored needed efforts to prepare and refine plans prior to the next influenza pandemic (3-7). This pandemic was controlled by substantial public health efforts and the availability of an effective 2009 H1N1 vaccine. The foundations for influenza disease prevention include enhanced surveillance for novel influenza strains, antiviral medications, improved ability to generate large quantities of safe and effective vaccines, and an efficient public health infrastructure to distribute these vaccines.

The threat of pandemic influenza in 1976 (swine influenza) and again in 1977 (Russian influenza) resulted in an inactivated influenza virus vaccine development program that provided important insights into variables influencing the immune responses to vaccination (8,9). Vaccine factors affecting the immunogenicity of inactivated influenza virus vaccines included; dosage of hemagglutinin, the number of doses administered, the use of adjuvants, and the preparation of the product (whole virus, split virus, or purified surface antigen). Host factors included; age, prior priming, presence of underlying disease, and other medical treatments.

Serum IgG antibody to the influenza virus hemagglutinin (HA) has a major role in protective immunity to influenza virus infection (10). Resistance to infection with seasonal influenza virus strains correlates directly with both serum hemagglutination inhibition (HAI) and neutralizing antibody titers (Nt), and measurements of serum HAI and Nt antibody are used to assess the immunogenicity of both seasonal and pandemic influenza vaccines and to predict their effectiveness.

In contrast to the 2009 H1N1 pandemic influenza strain, vaccine development for the H5 avian strains has been hindered by poor immunogenicity. One approach to improve the immunogenicity of inactivated influenza vaccines is to increase the dosage of hemagglutinin antigen contained in the vaccine. A number of studies evaluating the effect of HA dosage on immune responses to seasonal inactivated influenza vaccines have been performed over the past 35 years and have shown dose-related increases in serum and mucosal antibody

responses (11-19). Higher vaccine dosage levels have also been associated with higher titers of serum antibodies against antigenically distinct drift variants (20).

Studies of H5N1 vaccines have shown that the H5 HAs are relatively less immunogenic than HAs of viruses included in seasonal vaccines or the limited H9N2 vaccines that have been studied. For example, 2 doses of 90 micrograms (mcg) of a recombinant baculovirus expressed recombinant H5 HA vaccine elicited antibody responses in only approximately 50% of young healthy adult subjects (21). More recently, the safety and immunogenicity of a subvirion inactivated influenza A/Vietnam (H5N1) vaccine prepared by Sanofi Pasteur was evaluated among healthy young adults (22). Dosage levels that were studied ranged from 7.5 mcg to 90 mcg. The vaccine was well tolerated at all dosage levels; however, analysis of the responses indicated that two 90-mcg doses of vaccine given 28 days apart were required to stimulate an antibody response in approximately 57% of young healthy adults. A similar study by Nicholson et al found that only 4 of 11 subjects had antibody titers ≥ 1:40 after two 30 µg doses of non-adjuvanted H5N3 vaccine (23). Finally, Belshe et al reported that administering either the Vietnam or Indonesia H5N1 strains, the highest antibody responses occurred when the dosing interval between two 90 mcg doses was extended to 180 days (24).

The use of adjuvants is another promising approach to improve the immunogenicity of influenza vaccines. Adjuvants have the potential to decrease the amount of antigen needed in the vaccine (dose-sparing), and to improve the immune responses among groups that generally respond poorly to inactivated antigens (e.g., immunocompromised, elderly) (25). In most studies to date, the use of adjuvants has also been associated with an increase in injection site reactogenicity. Aluminum salts are licensed as adjuvants in the United States, but they have shown either none, or very modest, enhancement of immune responses compared to non-adjuvanted subvirion H5N1 influenza vaccines (25-27).

Oil-in-water emulsion adjuvants have shown promise in stimulating increased antibody responses to influenza vaccines (28), and the MF59-adjuvanted influenza vaccine (FLUAD®) by Novartis Vaccines and Diagnostics, has been licensed for commercial sale in a seasonal influenza vaccine in several European and non-European countries since 1997. MF59 is also the adjuvant in the EU licensed monovalent A/H1N1 swine flu subunit egg-derived (Focetria™) and cell-derived (Celtura™) pandemic vaccines.

During clinical development, trials with investigational vaccines containing MF59 adjuvant have been or are being performed in different age groups (from newborns to elderly) in Europe and the US and have shown an increased immunogenicity of co-administered antigens, associated with a good safety and tolerability profile (23, 29). In a 2005 study sponsored by the Division of Microbiology and Infectious Diseases (DMID), inactivated influenza A/H9N2 vaccines formulated with MF59 adjuvant were well tolerated at all dosage levels (3.75 to 30 mcg), and subjects receiving the adjuvanted vaccine demonstrated antibody responses that were higher than in those without adjuvant (30). In a separate multicenter study conducted at Vanderbilt, an inactivated influenza A/H5N1 vaccine adjuvanted with either aluminum hydroxide or MF59 was

evaluated in a clinical trial (31). As in other studies, MF59 stimulated antibody responses were higher than those generated following receipt of non-adjuvanted vaccine. Although there were a number of local adverse events noted in the adjuvanted vaccine recipients, no serious adverse events were associated with the vaccine.

In collaboration with scientists at the FDA (32), genome-fragment phage display libraries (GFPDL) and surface Plasmon resonance (SPR) analyses were conducted on the sera of the Vanderbilt vaccines to characterize the antibody responses to nonadjuvanted, alum-adjuvanted, and MF59-adjuvanted H5N1 vaccine. The study demonstrated that H5N1/MF59 vaccine induced epitope spreading from HA2 to HA1, targeted large conformational epitopes in HA1 that correlated with cross-clade neutralization, and induced antibodies against epitopes in the C-terminus of neuraminidase (NA), a region that juxtaposes to the sialic acid-binding site of NA. Others have demonstrated that expansion of influenza-specific $CD4^+$ T cells predicts the rise of neutralizing antibodies after MF59-adjuvanted H5N1 influenza vaccination (33).

GlaxoSmithKline (GSK) has developed a tocopherol based oil-in-water emulsion (squalene) adjuvant system (AS03) for use with inactivated influenza virus vaccines. In an initial study, dosage levels evaluated ranged from 3.8 to 30 mcg of H5N1 antigen both with and without extemporaneously mixed AS03 adjuvant (34). The geometric mean titers (GMTs) showed that a clear adjuvant-effect was observed after each of the 2 vaccine doses, which was much more apparent after the second dose. After the first vaccine dose, the Committee for Medicinal Products for Human Use (CHMP) criterion for seroconversion rate (>40%) was met by adjuvanted formulations containing antigen doses that were 7.5 mcg or more, but by none of the non-adjuvanted formulations. After the second vaccine dose, the adjuvanted formulations at all dose levels complied with both CHMP and United States (US) Food and Drug Administration (FDA) criteria for seroconversion and seroprotection rates, whereas from the non-adjuvanted groups only the 30 mcg formulation met the CHMP criterion for seroconversion rate. Adjuvantation substantially improved homologous neutralizing antibody responses *in vitro* in the adjuvanted groups compared with the non-adjuvanted groups, with increases of 5-8 times in GMTs recorded after the second dose for the 3.8 mcg, 7.5 mcg, and 15 mcg antigen formulations. A convincing adjuvant effect on heterologous neutralizing responses was further shown by the increased GMTs after the second dose, with increases of 5-6 times more than in the corresponding non-adjuvanted groups for the antigen doses of 3.8 mcg and 7.5 mcg. No antigen dose-response relation was evident for the nonadjuvanted formulations after the second dose.

The cellular and molecular mechanisms of action by which novel oil-in-water emulsion adjuvants enhance the immune response to vaccination are not completely understood (35, 36). Recently published data from mouse models suggest that AS03 works locally to stimulate immune effectors (37). AS03 also induces the local production of monocyte-, dendritic cell (DC)-, and neutrophil-recruiting chemokines in mice, as well as the pro-inflammatory cytokines IL-6, IL-1ß, IL-1α, TNFα, and IFN-gamma, and promotes recruitment of antigen-loaded monocytes to draining lymph nodes. AS03 is unique among adjuvants in that it includes α-tocopherol, a

highly bio-available form of Vitamin E. The presence of α-tocopherol appears to be necessary to induce local cytokine responses and stimulate the antigen-specific adaptive responses. The mechanism of action in humans is not well-defined. Elucidating the mechanisms by which AS03 augments the immune response to influenza vaccine in humans is important not only in developing more efficient vaccine regimens, but also in determining the safety and the potential for adverse events after adjuvanted vaccine.

Recently, an increased incidence of narcolepsy was noted in patients aged 4-19 after receiving Pandemrix^{TM}, a monovalent H1N1 vaccine adjuvanted with AS03 licensed for use in Europe during the 2009-2010 influenza season (38). In Finland, the risk of developing narcolepsy was nine times higher in patients aged 4-19 years who had received the pandemic vaccine than in those who had not. All of these patients had the (HLA) DQB1*0602 genotype, a genotype which has been strongly linked to narcolepsy (39). The pathophysiology of narcolepsy is thought to be related to low levels of the hypothalamic peptide hypocretin (also known as orexin) in the central nervous system, which may occur as a result of selective cell death of hypocretin-containing neurons. Many have hypothesized an immune-mediated phenomenon is responsible for hypocretin cell death since the disease is tightly associated with HLA haplotypes. This report from Finland underscores the need for an improved understanding of the mechanisms of action of AS03 and other novel adjuvants, both to ensure the safety of adjuvanted vaccines, as well as to develop dose-sparing, more immunogenic approaches to vaccination. Our innovative systems biology approach (40-43) will allow us to understand more completely the cellular and molecular genomic and proteomic signals generated by AS03-adjuvanted vaccine and the mechanisms by which the innate immune response leads to an antigen-specific adaptive response.

The development of effective vaccines for H5N1 influenza has been challenging, because the dosage of antigen required to elicit a potentially protective response is higher than that used in the seasonal influenza vaccines. It may also be impractical to use very high levels of antigen for mass vaccination, especially if multiple administrations are required. Additionally, using vaccines with high antigen content would likely severely limit the global influenza vaccine supply. The study of novel adjuvants is critical to the development of a dose-sparing approach to pandemic influenza vaccination, which has the potential to result in a greater global distribution of vaccine.

In 2009, the Department of Health and Human Services (DHHS) procured inactivated 2009 H1N1 vaccine from one US-licensed manufacturer and a supply of oil-in-water emulsion adjuvant from a different manufacturer. DMID sponsored a Phase II clinical trial to assess the safety and dosage-related immunogenicity of 2 doses of the 2009 H1N1 vaccine given with or without the adjuvant, AS03. As of June 2010, no safety signals have been identified in that study that have not already been identified in the investigator brochures. While the immunogenicity data from this study are not yet publicly available, similar studies using oil-in-water emulsion adjuvants with a 2009 H1N1 vaccine confirmed that the adjuvant did enhance antibody responses (44). Adjuvanted H5N1 vaccines are likely to be a critical part of the public

health response to an H5N1 pandemic if it occurs in the near term. To address this, the DHHS has similarly procured a stockpile of H5N1 vaccines and a stockpile of the oil-in-water based adjuvant, AS03.

This study will use a systems biology approach to assess the human early gene and protein signatures expressed after a 3.75 mcg intramuscular dose of subvirion inactivated monovalent influenza A/H5N1 (HA of A/Indonesia/05/2005) virus vaccine manufactured by sanofi pasteur is administered to healthy adults with or without AS03 adjuvant manufactured by GSK. Safety, reactogenicity, and immunogenicity will also be assessed.

2.1.1 Public Readiness and Emergency Preparedness Act

This protocol and the vaccine tested are covered under the Public Readiness and Emergency Preparedness Act (PREP Act). Under the PREP Act, covered persons are immune from liability actions brought from the administration or use of a covered countermeasure that is the subject of a declaration.

On June 15, 2009, HHS secretary Kathleen Sebelius had issued an amendment to the Declaration for use of the PREP act to include the H1N1 vaccines and any associated adjuvants (Federal Register, Volume 74, Number 121, Pages: 30294-30297). The PREP act provides immunity for covered persons (such as Manufacturers, Distributers, Program planners and other Qualified persons who prescribe, administer or dispense the vaccine) from tort liability, unless the injury was caused by willful misconduct.

The PREP Act also authorized a “Covered Countermeasures Process Fund” to provide compensation to eligible individuals who suffer specified injuries from administration or use of a countermeasure pursuant to the declaration. Any requests for compensation must be filed within one year of administration or use of the countermeasure. Requests would go to the HRSA Preparedness Countermeasures Injury Compensation Program (http://www.hrsa.gov/countermeasurescomp/default.htm). Compensation may then be available for medical benefits, lost wages and death benefits to eligible individuals for specified injuries in accordance with regulations published by the Secretary. Eligibility for compensation and the injuries for which compensation may be available are further defined by regulation.

An individual who suffers a serious physical injury or death from administration and use of the vaccine must first seek compensation from the Covered Countermeasures Process Fund. A serious physical injury means an injury that is life threatening, results in, or requires medical or surgical intervention to prevent, permanent impairment of a body function or permanent damage to body structure. Any compensation will be reduced by public or private insurance or worker’s compensation available to the injured individual.

If no funds have been appropriated to the compensation program, the Secretary does not make a final determination on the individual's request within 240 days, or if the individual decides not to accept the compensation, the injured individual or his representative may pursue a tort claim in the United States District Court for the District of Columbia, but only if the claim involves willful misconduct, is pled with particularity required under the PREP Act, verified, and accompanied by an affidavit by a physician who did not treat the individual and certified medical records. Any award is reduced by any public or private insurance or worker's compensation available to the injured individual. Awards for non-economic damages, such as pain, suffering, physical impairment, mental anguish, and loss of consortium are also limited. If the individual accepts compensation, or if there is no willful misconduct, the individual does not have a tort claim that can be filed in a United States Federal or a State court.

2.2 Rationale

There is great public concern over the safety of vaccines. Currently, the use of adjuvants with influenza vaccines to boost the immunological response is debated. A critical consideration for adjuvanted vaccines is whether the product is safe and effective. Experimental evidence of an effective and safe Sanofi H5N1-adjuvanted vaccine is necessary for licensing and widespread use of this product combination. Using a systems biology approach, this proposal measures multiple signals induced by the GSK AS03 adjuvanted and unadjuvanted H5N1 vaccine during the early human immune response. The systems approach allows us to carefully and meticulously evaluate and integrate the immune parameters induced by the adjuvanted vaccine response with the gene expression signatures of the immune system.

This study will determine the safety, reactogenicity, and immunogenicity of an intramuscular subvirion inactivated monovalent influenza A/H5N1 virus vaccine manufactured by Sanofi Pasteur administered to healthy adults at 3.75 given with or without the AS03 adjuvant manufactured by GSK. A primary goal of this study is to assess the safety and tolerability of the vaccine formulation and the potential of the adjuvant to enhance the immune response to A/H5N1 virus vaccine in healthy adults. The vaccine was made with HA antigen derived from the influenza A/Indonesia/05/2005 virus. Two doses of the A/H5N1 virus vaccine with or without AS03 adjuvant will be administered 28 days apart. Serum HAI and neutralizing antibody titers (Nt) will be evaluated prior to each vaccination, approximately 1, 3, 7 and 28 days after the first vaccination and approximately 28 days after the second vaccination. After each vaccination, vaccine reactions will be assessed and the patients will either visit the clinic or will be contacted to review the memory aid, AE/SAEs and concomitant medications.

Although much has been learned about the response of the human immune cells in isolation, very little information is known on the global changes that occur within immune cells found in blood during the course of immunization with adjuvants. An additional goal of this proposal uses in-depth experimental and bioinformatics approaches to analyze and integrate the human early immune cell changes in global RNA and protein expression during H5N1 influenza

vaccination with and without AS03 adjuvant. Using this approach, we also aim to develop a comprehensive model of the mechanism of action of AS03.

Currently, the time to evaluate the safety and efficacy of a new vaccine takes weeks or months. In the event of a pandemic or national emergency, a rapid, simple test is needed to quickly evaluate the safety of new vaccines and to predict a successful immune response. This proposal seeks to identify human early gene and/or protein signatures in blood cells that correlate with seroprotection, as defined by a hemagglutinin inhibition titer≥ 1:40, which may provide the basis for the ability to predict whether or not a patient has been successfully immunized. It is hoped that these findings will serve as the basis for larger, more targeted studies in the future.

2.3 Potential Risks and Benefits

2.3.1 Potential Risks

The risks of this study include intramuscular (IM) injection of the vaccine and adjuvant combinations, possible reactions to the vaccine or adjuvant, having blood drawn and breach of confidentiality.

Occasionally, adult recipients of influenza vaccines may develop influenza-like reactions such as fever, feverishness, chills, shivering, headache, malaise/fatigue, myalgia/body aches, and/or nausea. These may occur more frequently in people who are given higher dosage levels of vaccine or vaccine with the adjuvant. These reactions are usually greatest within the first 24 hours after vaccination and last 1 to 2 days. Some subjects may develop reactions at the site of vaccination (bruising, redness, swelling, pain, or tenderness), and individuals in this study who receive the adjuvant have a greater likelihood of developing these reactions. Analgesics (e.g. acetaminophen) and rest will generally relieve or moderate these symptoms. These reactions should go away in 1 to 4 days and should not require additional treatment. Bruising is not expected to be related to the vaccine or adjuvant. Rather, it can sometimes be due to the vaccination procedure.

Acute and potentially life-threatening allergic reactions are also possible. Very rarely, occurring in about 1 in 4 million people given a vaccination, there can be a serious allergic reaction to a vaccine. These reactions can cause skin rash (hives), swelling around the mouth, throat, or eyes, difficulty breathing, a fast pulse, sweating, or loss of blood pressure (fainting). If these reactions occur they can usually be stopped by the study personnel giving emergency medications. As with any vaccine or medication, there is a very small chance of a fatal reaction, although researchers do not expect this to occur.

During the swine influenza (H1N1) vaccine campaign of 1976, about 1 per 100,000 vaccine recipients developed a paralytic illness called Guillain-Barré Syndrome (GBS). Guillain-Barré

Syndrome is an acute inflammatory neuropathy characterized by weakness, hyporeflexia or areflexia, and elevated protein concentrations in cerebrospinal fluid. This syndrome has not been seen consistently with other influenza vaccines. Most persons who develop GBS recover completely, although the recovery period may be as little as a few weeks or as long as a few years. About 30% of those with GBS still have residual weakness after 3 years and about 3% may suffer a relapse of muscle weakness and tingling sensations many years after the initial attack. Intensive surveillance of GBS after administration of inactivated influenza vaccines since 1976 has shown a slight increase in risk over background cases (more than 1 additional case of GBS per million persons) following vaccination, typically with onset within 6 weeks after vaccination (45). Interestingly, although vaccination rates have increased in the last 10 years the numbers of reported cases of vaccine-associated GBS have declined (46).

GlaxoSmithKline conducted a dose-ranging study with 3.8 to 30 mcg doses of a novel H5N1 vaccine administered with or without AS03 adjuvant (34). All vaccine formulations were well tolerated, and no immediate allergic reactions or other serious adverse events were reported during the trial. Pain at the injection site was the most common local symptom in all groups and it was reported significantly more frequently by participants who received the adjuvanted vaccine than by those who received the unadjuvanted vaccine. Pain at the injection site of Grade 3 intensity was rare (reported by a total of 4 participants from the groups that received the 15 mcg and 30 mcg adjuvanted formulations). Other injection site symptoms were less common and most were mild to moderate in intensity. Most of these events resolved or decreased in intensity within 48 hours. The general symptoms most commonly reported were fatigue and headache, which tended to be more frequent in the adjuvanted-vaccine groups than in the unadjuvanted-vaccine groups. The same pattern was recorded for arthralgia, fever, muscle aches, shivering, and sweating. Most reported general solicited symptoms were mild or moderate in intensity. No participants had a temperature above 39°C, and only 1 subject had a temperature above 38°C for longer than 24 hours. Swollen or enlarged axillary or supraclavicular lymph nodes were reported by 7 participants in the adjuvanted groups, and in all but one the swelling was thought to be related to vaccination. All but 2 cases were recorded as easily tolerated (duration 4–13 days). The others were reported as interfering with normal activity (duration 2–4 days).

To date, more than 11,000 subjects in clinical trials have received H5N1 influenza vaccine adjuvanted with AS03. Local site reactions for pain, redness, swelling and induration were all statistically significantly more common in the adjuvant group than in the controls. But these local site reactions were most commonly mild, transient and did not increase with the second dose. Systemic reactions of arthralgia, myalgia, shivering, feverishness and low grade fever were also statistically significantly more common in the adjuvant group than in the control group. But these systemic reactions were mostly mild as well. The people who received adjuvanted vaccine in placebo controlled trials were also more likely to self-report injection site pruritus, injection site warmth, nausea, headache, insomnia and malaise. In study subjects receiving AS03-adjuvanted pandemic influenza vaccine, the following potentially immune-mediated

diseases (pIMDs) were observed in more than one subject: 7th cranial nerve palsy, polymyalgia rheumatica or temporal arteritis, psoriasis, autoimmune thyroiditis, celiac disease, multiple sclerosis, thrombocytopenia, radiculitis, uveitis, ulcerative colitis, and rheumatoid arthritis. Each occurrence was investigated by GSK, and, while temporally related in some circumstances, available data did not support a causal relationship between receipt of adjuvanted pandemic influenza vaccine and any of these conditions. Although a slightly increased risk for GBS may exist in subjects who receive AS03-adjuvanted H1N1 vaccine, no cases of GBS have been reported among H5N1 vaccine recipients to date.

There is also substantial clinical experience with AS03 adjuvant outside the United States. As of May 2010, approximately 148 million doses of Arepanrix™ (Quebec-manufactured AS03-adjuvanted H1N1 influenza vaccine “Q-PAN H1N1”) and 143 million doses of Pandemrix™ and Prepandrix™ (Dresden-manufactured AS03-adjuvanted H1N1 influenza vaccines “D-PAN H1N1”) have been distributed. Arepanrix™ has been licensed for use in Canada, Mexico, Korea, Japan, Chile, and the European Union (27 countries). Pandemrix™ and Prepandrix™ have been licensed in the European Union, Switzerland, Australia, as well as in 5 Asian countries (Hong-Kong, Singapore, Malaysia, South Korea and Turkey) and are currently under review in 14 additional countries. The overall rate of adverse event reports is either somewhat higher than usual, as see in Canada or approximately as expected, as reported in the UK.

Beginning in July 2010, GSK received reports of narcolepsy in patients following immunization with AS03-adjuvanted monovalent pandemic influenza vaccines, Arepanrix™ and Pandemrix™ (38). The majority of cases have occurred in Sweden and Finland, involved subjects less than 18 years of age, and followed receipt of Pandemrix™. GSK has concluded that currently available data are insufficient to assess the likelihood of a causal relationship. The Committee for Medicinal Products for Human Use (CHMP) considered that an association with narcolepsy and Pandemrix™ vaccination may exist in children and adolescents in those countries. The results indicate a six to 13-fold increased risk of narcolepsy in vaccinated vs. unvaccinated children and adolescents. The increased risk was not seen in adults (over age 20 years). A similar risk has not been confirmed but cannot be ruled out in other countries. The mechanism of this potential association is not well understood and may be more substantial in patients with HLA haplotype DQB1*0602, the haplotype strongly linked with narcolepsy.

There is no evidence of a vaccine-associated risk in pregnancy, or of increased fetal loss. The rate of anaphylaxis after doses does not exceed reported rates (0.1-1 per 100,000) from other vaccination programs. A variety of other, non-serious, allergic reactions are noted. The increase in risk of Guillain-Barré syndrome, if any, is small, and not significantly greater than recently published US estimates which reflect the use of unadjuvanted H1N1 vaccines. The balance of risk and benefit associated with the vaccines remains positive. It is unknown if this vaccine or adjuvant pose any risks to an unborn child. As such, women of childbearing potential, i.e. women who have not reached menopause ≥ 1 year, or who have not been surgically sterilized, i.e. tubal ligation, bilateral oophorectomy, or hysterectomy, must agree to

use an effective method of birth control while they are on study at least for 30 days following the second dose. A highly effective method of birth control is defined as one which results in a low failure rate (i.e. less than 1% per year) when used consistently and correctly such as implants, injectables combined oral contraceptives, some IUDs, sexual abstinence or vasectomized partner (47). Other effective birth control includes barrier method, such as condom or diaphragm, with spermicide or other licensed products. In addition to contraceptive use, women of child-bearing potential will be required to have a negative serum or urine pregnancy test within 24 hours prior to receiving the first and second dose of vaccine. If a female subject becomes pregnant while participating in this study, we will ask her permission to follow-up with her about her health and the health of her baby through pregnancy outcome.

The potential risks and discomforts related to drawing blood include bruising at the blood draw or vaccination site and feeling light-headed or dizzy. Drawing blood and intramuscular injection causes transient discomfort and may cause fainting. This discomfort may occur more frequently in people who are given the vaccine with the adjuvant. Fainting will pass by having the participant lie down. Bruising at the blood draw site may occur but can be prevented or lessened by applying pressure to the draw site for several minutes. Drawing blood and intramuscular injection also may cause infection. This risk is small, and the use of proper technique and sterile equipment will make infection extremely unlikely.

The potential risks associated with human gene signature response identification are those associated with the loss of confidentiality. Subjects will be asked to provide personal health information. All attempts will be made to keep this personal health information confidential within the limits of the law. However, there is a chance that unauthorized persons will see the subjects' personal health information. Every effort will be made to protect subject confidentiality by using bar-coded, de-identified samples, stripped of subject ID numbers. All records will be kept in a locked file or maintained in a locked room at the participating clinical study site. Electronic files will be password protected. Only people who are involved in the conduct, oversight, or auditing of this study will be allowed access to the personal health information that is collected. Any publications from this study will not use information that will identify subjects by name. Organizations that may inspect and/or copy research records for quality assurance and data analysis include groups such as: National Institute of Allergy and Infectious Diseases and Food and Drug Administration.

There may be other unknown risks.

2.3.2 Known Potential Benefits

There are no known benefits attributable to the receipt of this subvirion inactivated monovalent influenza A/H5N1 virus vaccine with or without AS03 adjuvant. It is possible that vaccination with the A/H5N1 virus vaccine with or without AS03 adjuvant will result in some protection against infection caused by H5N1 viruses, and may provide protection against infection with

H5N1 influenza, should the virus be contracted. The duration of any such protection is currently unknown. However, this strain of influenza, H5N1, is not currently a risk to the general public and the H5N1 virus vaccine with or without AS03 adjuvant will offer no protection against circulating seasonal influenza. There may be pandemic preparedness benefits to society in the future if the vaccines being evaluated here prove to be sufficiently safe and immunogenic and can be employed if a need for widespread H5N1 vaccination occurs.

3 OBJECTIVES

This study will determine the safety, reactogenicity, and immunogenicity of an intramuscular subvirion inactivated monovalent influenza A/H5N1 (HA of A/Indonesia/05/2005) virus vaccine manufactured by Sanofi Pasteur administered at 3.75 mcg per dose given with or without AS03 adjuvant manufactured by GSK. In addition, the study will use a systems biology approach to assess the early cytokine and chemokine responses and the blood's immune cells' gene and protein signatures expressed after the first dose of the vaccine.

Objectives:

Primary Objectives:

- To examine the safety and tolerability of subvirion inactivated A/H5N1 virus vaccine mixed with the AS03 adjuvant in healthy adults.
- To determine the potential for AS03 adjuvant to enhance the early development of vaccine-specific hemagglutination inhibition (HAI) antibody titers and neutralization (Nt) antibody titers approximately 1, 3, 7, and 28 days following receipt of the first dose of subvirion inactivated A/H5N1 virus vaccine administered at 3.75 mcg per dose in healthy adults.

Secondary Objective:

- To determine the potential for AS03 adjuvant to enhance the development of vaccine-specific hemagglutination inhibition (HAI) antibody titers and neutralization (Nt) antibody titers approximately 28 days following receipt of the second dose of subvirion inactivated A/H5N1 virus vaccine administered at 3.75 mcg per dose in healthy adults.

Tertiary Objectives:

- To identify differentially expressed messenger RNAs (mRNA), noncoding RNAs (ncRNA), RNA splice junctions, and nonsynonymous base substitutions in human immune blood cells as determined by RNA-Seq analysis at Days 1, 3, 7, and 28 after vaccination with the first dose of A/H5N1 virus vaccine with and without AS03 adjuvant.
- To identify differentially expressed cellular proteins in human immune blood cells as determined by shotgun proteomics at Days 1, 3, 7, and 28 after vaccination with the first dose of A/H5N1 virus vaccine with and without AS03 adjuvant.

Endpoints:

Primary Endpoints:

- Occurrence of vaccine-associated serious adverse events (SAEs) from the time of first vaccination through 13 months after the first vaccination.
- Occurrence of solicited local and systemic reactogenicity in the 8 days (Days 0-7) after each vaccination.
- GMT of HAI antibody, proportion of subjects achieving a serum HAI antibody titer of 1:40 or greater, and frequency of 4-fold or greater increases of HAI antibodies in each group against the subvirion inactivated A/H5N1 virus vaccine approximately 1, 3, 7 and 28 days after receipt of the first dose of vaccine.
- GMT of neutralizing antibody, proportion of subjects achieving a serum neutralizing antibody titer of 1:40 or greater, and frequency of 4-fold or greater increases of neutralizing antibodies in each group against the subvirion inactivated A/H5N1 virus vaccine approximately 1, 3, 7 and 28 days after receipt of the first dose of vaccine.

Secondary Endpoints:

- GMT of HAI antibody, proportion of subjects achieving a serum HAI antibody titer of 1:40 or greater, and frequency of 4-fold or greater increases of HAI antibodies in each group against the subvirion inactivated A/H5N1 virus vaccine 28 days after receipt of the second dose of vaccine (approximately Day 56).
- GMT of neutralizing antibody, proportion of subjects achieving a serum neutralizing antibody titer of 1:40 or greater, and frequency of 4-fold or greater increases of neutralizing antibodies in each group against the subvirion inactivated A/H5N1 virus vaccine 28 days after receipt of the second dose of vaccine (approximately Day 56).

Tertiary Endpoints:

- RNA, RNA splice junctions, nonsynonymous base substitutions, or protein expression in blood immune cells at Days 1, 3, 7, and 28 after the first vaccination that differentiates one dose of an AS03-adjuvanted H5N1 vaccine compared to one dose of the unadjuvanted H5N1 vaccine.
- RNA, RNA splice junctions, nonsynonymous base substitutions, or protein expression in blood immune cells at Days 1, 3, 7, and 28 after the first vaccination that correlates with the occurrence of solicited local and systemic reactogenicity 8 days (Days 0-7) after the first dose of an AS03-adjuvanted H5N1 vaccine or the first dose of the unadjuvanted H5N1 vaccine.

Exploratory Endpoints:

- Exploration of unsolicited adverse events (AEs) reported in the 28 days after each vaccination.
- Occurrence of new-onset chronic medical conditions through 13 months after the first vaccination.
- Development of serum antibody responses against antigenically drifted variants of the H5N1 virus.
- Development of a systems biology model of early human immune response of AS03-adjuvanted and unadjuvanted A/H5N1 virus vaccine that integrates quantitative changes in RNA and protein expression with the innate and humoral immune responses as determined by cytokine/chemokine levels, hemagglutination inhibition (HAI), neutralizing antibody titers (Nt), and immune cell activation status, respectively.
- Identification of early RNA and protein expression signatures in human immune cells that indicate successful immunological response and protection against the influenza pathogen.

4 STUDY DESIGN

This is a single center, randomized, double-blinded, controlled, Phase I, small targeted prospective study in healthy male and non-pregnant female subjects, 18 to 49 years old, inclusive, designed to determine the safety, reactogenicity, and immunogenicity of an intramuscular subvirion inactivated monovalent influenza A/H5N1 (HA of A/Indonesia/05/2005) virus vaccine manufactured by Sanofi Pasteur administered at 3.75 mcg per dose given with or without AS03 adjuvant manufactured by GSK. In the study, each subject will receive two doses administered 28 days apart. Using venous blood samples collected from all subjects, vaccine-specific hemagglutination inhibition (HAI) antibody titers and neutralization (Nt) antibody titers will be used to measure the baseline immune response at Day 0 (prior to vaccination) and the immunogenicity of the vaccines at Days 1, 3, 7, and 28 (prior to the second dose of vaccine) after the first vaccination and 28 days after the second vaccination (Day 56). Reactogenicity will be collected during the 8 days following each vaccination (Days 0-7 and Days 28-35, respectively). All subjects will be monitored for AEs through Day 56 and SAEs and new-onset chronic medical conditions through Day 393. In addition, the study will use a systems biology approach to assess the human early gene and protein signatures expressed at Days 1, 3, 7, and 28 after the first vaccination. The systems data will be integrated with immunogenicity and reactogenicity data to develop a systems model of the human immune response to A/H5N1 vaccine with or without AS03 adjuvant.

Approximately twenty subjects who provide informed consent will be considered for eligibility. Subjects will be recruited from the target population reflecting the community at large at a participating Vaccine and Treatment Evaluation Unit (VTEU) site, Vanderbilt University, and enrolled over a 6-month period.

Subjects will be screened for health status by history, concomitant medications, vital signs (oral temperature, pulse and blood pressure) and complete physical examination (without genital and rectal exam) to include supraclavicular and axillary lymph node assessment prior to vaccination. All female subjects of childbearing potential must have a negative urine or serum pregnancy test within 24 hours prior to receipt of each vaccination.

Subjects who meet the entry criteria for the study and provide informed consent will be randomized 1:1 between adjuvanted and unadjuvanted vaccine and placed into one of 2 groups to receive two doses approximately 28 days apart of an intramuscular subvirion inactivated monovalent influenza A/H5N1 (HA of A/Indonesia/05/2005) virus vaccine administered at a dosage of 3.75 mcg given with or without AS03 adjuvant.

Vaccine preparation will be performed by the participating clinical study site pharmacist and administration will be performed by an unblinded vaccine administrator. The unblinded vaccine administrator is a study clinician licensed to administer medications/vaccines and may also

participate in dose preparation, but will not be involved in study-related assessments or have subject contact for data collection following vaccine administration. All study-related assessments will be performed by blinded study personnel. Subjects and laboratory personnel will also be blinded to group assignment.

The first vaccination will occur on Day 0 and the second vaccination will occur on Day 28. Targeted physical exams may be performed if indicated based on review of interim health status prior to each vaccination on Days 0 and 28 as well as at the Days 1, 3, 7, 35 and 56 clinic visits. Special attention will be given to the lymph node examination at baseline prior to the first vaccination (Day -28 or Day 0), Days 1, 3, and 7, Day 28 prior to the second vaccination, and Days 35 and 56.

Vaccine reactions will be assessed for at least 20 minutes following each vaccination on Days 0 and 28, and self-assessments will continue through 8 days following each vaccination. The vaccination site will be examined at the end of the 20-minute observation period following each vaccination and in the clinic on Days 1, 3, 7 and 35. Subjects will be asked to record oral temperature, solicited vaccine reactions and any unsolicited AE/SAEs on a memory aid for 8 days following the each vaccination (Days 0-7 and Days 28-35, respectively). Subjects will be encouraged to take their temperature around the same time each day. Each subject will be seen in the clinic on Days 1, 3, 7, 28, 35 and 56 to review AE/SAEs, concomitant medications, health status and the events recorded on the memory aid. Each subject will be contacted by telephone 1-3 days following the second vaccination (Day 30) to review the memory aid, AE/SAEs and concomitant medications. Subjects will also be contacted by telephone at 6 months and 1 year following the second vaccination (Days 208 and 393, respectively) to review serious adverse events and new-onset chronic medical conditions. Based on this information, subjects may be asked to return to clinic to be evaluated.

Venous blood samples (approximately 100 mL) will be collected from all subjects at Days -28, -14, and 0 (prior to vaccination) and at Days 1, 3, 7, and 28 (prior to the second dose of vaccine) after the first vaccination for systems biology studies (cytokine/chemokine profiles, immune cell activation status, and transcriptome and proteome profiles). Serologic assessment (HAI and Nt) will also be conducted at each of these visits except on Days -28 and -14. Using a systems biology approach, we will identify and quantify the early changes in the subject's serum cytokine/chemokine levels, immune cell activation responses, and the whole transcriptome and proteome of the major immune cells after the first vaccination. The venous blood sample collected at Days -28, -14, and 0 will be used to measure baseline serum chemokine and cytokine levels, and immune cell activation status, and the whole transcriptome and proteome levels of the major blood immune cells prior to the first vaccination. In addition, at the Day 28 follow-up visit, subjects will be given a second dose of the same vaccine that they received initially, since two doses of vaccine are required to generate a protective immune response in the majority of vaccines. After the second dose of vaccine is given, a final blood sample (approximately 10 mL) will be collected for serologic assessment (HAI and Nt) only at 28 days after the second vaccination (Day 56). The systems biology studies will be conducted only after

the first vaccine dose since we are seeking to determine the initial responses associated with vaccine and adjuvant, and not the booster responses. A urine sample will be collected from each subject at Days -28, -14, 0, 1, 3, 7, and 28 for future research.

The duration of the study for each subject will be approximately 14 months.

Further details of all study procedures and evaluations to be performed at each subject visit by study day are described in Sections 7 and 8.

See Appendix A for Schedule of Procedures/Evaluations.

A Safety Monitoring Committee (SMC) will be convened by DMID to review participant safety data which may include solicited and unsolicited AE/SAEs, concomitant medications, and any physical examinations. Safety data will be reviewed by the SMC per the SMC charter for this study. As an outcome of each review/meeting, the SMC will make a recommendation at that time as to the advisability of proceeding with vaccinations, and to continue, modify or terminate the study.

Outcome measures will be the occurrence of SAEs and solicited local and systemic reactogenicity in each group, and GMT of HAI and neutralizing antibody, proportion of subjects achieving a serum HAI and neutralizing antibody titer of 1:40 or greater, and frequency of 4-fold or greater increases of HAI and neutralizing antibodies against the subvirion inactivated influenza A/H5N1 virus vaccine in each group on Days 1, 3, 7, 28, and 56. Additionally, unsolicited adverse events in the 28 days after each vaccination, SAEs and new-onset chronic medical conditions through 13 months after the first vaccination and development of serum antibody responses against antigenically drifted variants of the H5N1virus will be evaluated.

For systems biology analysis of the subject's immune and blood cells response to A/H5N1 with or without AS03 adjuvant, cytokine/chemokine levels, and immune cell activation status will be determined in venous blood samples at Days -28, -14, 0, 1, 3, 7, 28 after the first vaccination. RNA transcript changes in blood immune cells at Days -28, -14, 0, 1, 3, 7, and 28 after the first vaccination will be determined by RNA-Seq analysis and measured in Reads Per Kilobase of Exon model per Million reads (RPKM). Relative cellular protein changes in subject's blood immune cells at Days -28, -14, 0, 1, 3, 7, and 28 after the first vaccination will be identified by shotgun proteomics and quantified using isobaric tags for relative and absolute quantitation (iTRAQ) (48). Refer to Section 7.3.3 for further details on the systems biology studies assays and procedures.

5 STUDY POPULATION

Approximately twenty healthy male and non-pregnant female subjects, 18 to 49 years old, inclusive, will be enrolled in this study from one participating VTEU site. Enrollment will occur over a 6-month period, and the target population should reflect the community at large at the participating clinical study site. Information regarding the study may be mailed or emailed to potential subjects who have previously participated in vaccine trials conducted at the participating clinical study site. Other advertisements may also be used. The local Institutional Review Boards (IRBs) will approve all materials prior to their use.

5.1 Eligibility Criteria

It is the intent of this study to enroll subjects who are considered "normal, healthy volunteers." Subjects with pre-existing clinically significant conditions, even if medically stable, are not considered "normal, healthy volunteers."

Subjects who meet the study entry eligibility criteria will receive a single dose of vaccine per randomized assignment (Dose 1). Subjects who continue to meet eligibility criteria will receive the second single dose of vaccine per randomized assignment (Dose 2).

Inclusion and Exclusion Criteria must be assessed by a study clinician licensed to make medical diagnoses and listed on the Form FDA 1572 as the site principal investigator or sub-investigator.

5.1.1 Subject Inclusion Criteria

Subjects must meet all of the following inclusion criteria to participate in this protocol:

1. Are males or non-pregnant females between the ages of 18 and 49 years, inclusive.

2. Women of childbearing potential (not surgically sterile via tubal ligation, bilateral oophorectomy or hysterectomy or who are not postmenopausal for ≥ 1 year) must agree to practice adequate contraception (that may include, but is not limited to, abstinence, monogamous relationship with vasectomized partner, barrier methods such as condoms or diaphragms with spermicide or foam, intrauterine devices, and licensed hormonal methods) during the study for at least 30 days following the last vaccination. Method of contraception will be captured on the appropriate case report form (CRF).

3. Are in good health, as determined by vital signs (oral temperature, pulse and blood pressure), medical history and <u>complete</u> physical examination (without genital and rectal exam) to ensure no existing chronic medical diagnoses or conditions are present.

4. For women of childbearing potential, negative urine or serum pregnancy test within 24 hours prior to vaccination.
5. Are able to understand and comply with planned study procedures.
6. Provide written informed consent prior to initiation of any study procedures.

5.1.2 Subject Exclusion Criteria

Subjects who meet any of the following exclusion criteria will be excluded from study participation:

1. Have a known allergy to eggs or other components of the vaccine (including gelatin, formaldehyde, octoxinol-9, thimerosal and chicken protein), or allergy to squalene-based adjuvants.
2. Women who are breastfeeding or plan to breastfeed at any given time from the first vaccination until 30 days after the last vaccination.
3. Have long term use (defined as taken for 2 weeks or more in total at any time during the past 2 months) of high dose oral or parenteral glucocorticoids (high dose defined as prednisone ≥ 20 mg total daily dose, or equivalent dose of other glucocorticoids); or high-dose inhaled steroids (high dose defined as >800 mcg/day of beclomethasone dipropionate or equivalent); or systemic corticosteroids of any dose within the past 4 weeks.
4. Have immunosuppression as a result of an underlying illness or treatment, or use of anticancer chemotherapy or radiation therapy (cytotoxic) within the preceding 36 months.
5. Have an active neoplastic disease or a history of any hematologic malignancy.
6. Have a diagnosis of schizophrenia, bipolar disease, or other major psychiatric diagnosis.
7. Hospitalized for psychiatric illness, history of suicide attempt, or confinement for danger to self or others, within the past 10 years.
8. Receiving systemic, prescription medications for the treatment of chronic medical conditions, unless such use is on a PRN (as needed) basis only. Non-PRN use of systemic, over-the-counter medications and PRN systemic, prescription medication may be allowed if, in the opinion of the investigator, they pose no additional risk to subject safety or assessment of immunogenicity/reactogenicity. *Note: Topical, nasal, and inhaled medications; vitamins; and contraceptives are also permitted.*

9. Received **pre-medication** with analgesic or antipyretic agents in the 6 hours prior to first vaccination, or **planned** medication with analgesic or antipyretic in the week following first vaccination. This criterion should not preclude subjects receiving such medication if the need arises. However, pre-medication is to be discouraged.

10. Received immunoglobulin or other blood products (with exception of Rho D immune globulin) within the 3 months prior to the first vaccination.

11. Received any live licensed vaccines within 4 weeks or inactivated licensed vaccines within 2 weeks prior to the first vaccination or plan receipt of such vaccines within 56 days following the first vaccination. This is inclusive of licensed seasonal influenza vaccines.

12. Have an acute or chronic medical condition that, in the opinion of the site principal investigator or appropriate sub-investigator, would render vaccination unsafe, would interfere with the evaluation of responses or is not generally seen in “normal, healthy subjects”.

13. Have a history of severe reactions following previous immunization with contemporary influenza virus vaccines.

14. Have an acute illness, including an oral temperature greater than or equal to 100.4°F, within 3 days prior to the first vaccination.

15. Pulse is less than 55 bpm or greater than 100 bpm.

16. Systolic blood pressure is less than 90 mm Hg or greater than 140 mm Hg.

17. Diastolic blood pressure is less than 60 mm Hg or greater than 90 mmHg.

18. Received an experimental agent (vaccine, drug, biologic, device, blood product, or medication) within 1 month prior to the first vaccination or expects to receive an experimental agent, other than from participation in this study, during the 14-month study period.

19. Are participating or plan to participate in another clinical trial with a licensed product during the 14-month study period.

20. Have any condition that, in the opinion of the site principal investigator or appropriate sub-investigator, would place the subject at an unacceptable risk of injury, or render them unable to meet the requirements of the protocol, or confound the interpretation of results.

21. Participated in an influenza A/H5 vaccine study in the past in a group receiving vaccine (does not apply to documented placebo recipients) or have a history of A/H5 infection prior to enrollment.

22. Have known active HIV, Hepatitis B, or Hepatitis C infection.

23. Have a history of alcohol or drug abuse in the last 5 years.

24. Plan to travel outside the U.S. in the time between the first vaccination and 56 days following the first vaccination.

25. Have a history of Guillain-Barré Syndrome.

6 ENROLLMENT/RANDOMIZATION/MASKING PROCEDURES

Approximately twenty subjects who have signed informed consent, have been screened, and are eligible for participation in this study will be enrolled, randomly assigned to one of 2 groups and receive at least one dose of vaccine. All subjects will receive 2 doses of the same vaccine. Subjects will receive 2 doses (Day 0, Day 28) of an intramuscular subvirion inactivated monovalent influenza A/H5N1 virus vaccine administered at 3.75 mcg per dose given with or without the AS03 adjuvant in a 1:1 ratio (n=10 per adjuvanted and unadjuvanted group). Subjects who withdraw from the study before receiving any vaccine may be replaced. Subjects who withdraw from the study after receiving at least one dose of vaccine will not be replaced. Enrollment will be done online using the enrollment module of the Data Coordinating Center's (DCC's) Internet Data Entry System (IDES). The randomization code will be prepared by statisticians at the DCC and included in the enrollment module for the trial. The randomization code will link to the group assignment. IDES will assign each volunteer a randomization code after the demographic and eligibility data have been entered into the system. A designated individual at the participating clinical study site will be provided with a code list for emergency unblinding purposes, which will be kept in a secure place.

Instructions for use of the enrollment module are included in the IDES User's Guide. Manual back-up procedures and instructions are provided for use in the event that the participating clinical study site temporarily loses access to the Internet or the online enrollment system is unavailable.

Per International Conference on Harmonisation (ICH) guideline E6: Good Clinical Practice (GCP), screening records will be kept at the participating clinical study site to document the reason why an individual was screened but failed trial entry criteria. The reasons why individuals failed screening will be recorded in IDES.

Further details of all study procedures and evaluations to be performed at each subject visit by study day are described in Sections 4, 7 and 8.

See Appendix A for Schedule of Procedures/Evaluations.

Vaccine preparation will be performed by the participating clinical study site pharmacist and administered by an unblinded vaccine administrator. The unblinded vaccine administrator is a study clinician licensed to administer medications/vaccines and may also participate in dose preparation, but will not be involved in study-related assessments or have subject contact for data collection following vaccine administration. All study-related assessments will be performed by blinded study personnel. Subjects and laboratory personnel performing assays will also be blinded to group assignment.

7 STUDY PROCEDURES/EVALUATIONS

7.1 Clinical Evaluations

Medical History: Will be obtained by interview of the subjects. Subjects will be queried regarding a history of significant medical disorders of the head, eyes, ears, nose, throat (HEENT), mouth, cardiovascular system, lungs, gastrointestinal tract, liver, pancreas, kidney, urologic, nervous system, blood, lymph glands, endocrine system, musculoskeletal system, skin, and genital/reproductive tract. A history of any allergies, cancer, immunodeficiency, psychiatric illness, substance abuse, and autoimmune disease will be solicited.

Medications History: All current medications and medications taken in the 28 days prior to enrollment (prescription and over-the-counter drugs will be included as well as vitamins and supplements) through 28 days after the second vaccination (Day 56) or early termination (if prior to Day 56), whichever occurs first. Assessment of eligibility also will include a review of permitted and prohibited medications (per the exclusion criteria).

Physical Examination: At screening (Day -28) or on the day of, but prior to, first vaccination (Day 0), vital signs (oral temperature, pulse and blood pressure; oral temperature only at Day 0 if all vital signs collected at screening), height and weight will be collected and a complete physical exam (without genital and rectal exam) will be performed by appropriate study personnel to assess general appearance and will include the following areas/systems: skin, supraclavicular and axillary lymph nodes, HEENT, neck, cardiovascular, pulmonary, abdomen, extremities, musculoskeletal, and neurological. The physical examination should specifically address issues identified by the medical history of the subject. At visits following the first vaccination a targeted physical examination may be conducted based on interim medical history. Oral temperature will also be collected prior to each vaccination on Days 0 and 28.

Reactogenicity Assessments: Will include brief history for assessment of AE/SAEs just prior to and following vaccination, which includes an assessment of erythema/redness, induration/swelling, pain and tenderness at the injection site; fever, feverishness, chills, shivering, arthralgia/joint pain, asthenia/weakness, malaise/fatigue, myalgia/body aches, headache and nausea. The vaccination site will be examined at the end of the 20 minute observation period following each vaccination on Days 0 and 28 as well as in the clinic on Days 1, 3, and 7 following the first vaccination, and 8 days following the second vaccination (Day 35). Special attention will be given to the lymph node examination at baseline prior to the first vaccination (Day -28 or Day 0), Days 1, 3, and 7, Day 28 prior to the second vaccination and Days 35 and 56. Supraclavicular and axillary lymph nodes will be assessed by appropriate study personnel to determine size, presence of pain, tenderness, fluctuance, mobility and overlying skin changes (erythema, heat, ulceration or drainage).

Memory Aids: All subjects will complete a subject memory aid for 8 days (Days 0-7) following each vaccination. Subject memory aids will be reviewed with the subject for AE/SAEs at the Days 1, 3, and 7 clinic visits following the first vaccination. Subject memory aids will be reviewed with the subject by telephone call for AE/SAEs 1-3 days (Day 30) following the second vaccination and at the clinic visit 8 days after the second vaccination (Day 35). Following review and appropriate electronic case report form (eCRF) completion, subject memory aids will be discarded.

7.2 Concomitant Medications/Treatments

Administration of any medications, therapies or vaccines will be documented in the subject's data collection form and reported on the subject's data acquisition screen. Concomitant medications will include all current medications and medications taken from within the 28 days prior to enrollment (prescription and over-the-counter drugs will be included as well as vitamins and supplements) through 28 days after the second vaccination (Day 56) or early termination (if prior to Day 56), whichever occurs first.

Use of new medication should prompt evaluation for the presence of a new diagnosis of chronic medical disease or chronic medical condition.

Medications that might interfere with the evaluation of the investigational product should not be used unless absolutely necessary. Medications in this category include, but are not limited to, glucocorticoids, i.e., oral, parenteral and high-dose inhaled steroids, and immunosuppressive or cytotoxic drugs. Other than from participation in this study, subjects should not receive experimental agents including vaccines during the 14-month study period. The administration of licensed vaccines should be delayed until 28 days after the last study vaccination. Subjects should also not receive licensed products from participation in another clinical trial.

7.3 Laboratory Evaluations

7.3.1 Clinical Laboratory Evaluations

Urine or serum pregnancy tests will be performed within 24 hours prior to each vaccination on all female subjects of childbearing potential. Results must be negative and known prior to vaccination.

7.3.2 Immunogenicity Evaluations

Venous blood samples (approximately 10 mL) will be collected from each subject prior to each vaccination on Days 0 and 28, on Days 1, 3, and 7 following the first vaccination, and 28 days (Day 56) after the second vaccination to evaluate for serum HAI and neutralizing antibody titers

(Nt). Subjects who withdraw early (prior to Day 56) will have assays run on available sera. Assays to determine serum HAI and neutralizing antibody titers (Nt) against the subvirion inactivated influenza A/H5N1 virus vaccine will be performed by a central (immunology) laboratory. Samples will be provided to the central (immunology) laboratory for testing in a blinded manner as they become available to the DMID Clinical Agent Repository.

7.3.3 Special Assays or Procedures

Venous blood samples collected from each subject at time points and amounts described below will be used to measure changes in cytokine and chemokine levels and changes in the whole transcriptome and proteome of major immune cells to identify and quantify changes in gene expression. We will use mathematical modeling of the accumulated data to identify RNA or protein expression signatures that correlate significantly with the observed serum HAI and Nt response, cytokine/chemokine response, and immune cell activation response. A urine sample will be collected from each subject at Days -28, -14, 0, 1, 3, 7, and 28 for future research. By comparing individuals vaccinated with H5N1 vaccine with or without AS03, our study may identify biomarkers that indicate that the adjuvant has successfully enhanced the immunological response.

(a) A peripheral venous blood sample will be collected prior to vaccine administration at Days -28, -14, and 0. The prevaccination time points permit setting the baseline immune activity for each individual enrolled in the study. This is a significant advantage because it will correct for baseline variations between individuals that are caused by uncontrollable parameters such as genomic and/or metagenomic variations, asymptomatic infections, personal habits (e.g., tobacco use), or even over-the-counter medications.

(b) After intramuscular injection of the H5N1 vaccine, with or without AS03 adjuvant, additional peripheral venous blood samples at Days 1, 3, 7, 28 and 56 will be collected to monitor the immune response.

For the Days -28, -14, 0, 1, 3, 7, and 28 time points, approximately 100 mL total will be collected. For the final serological assessment at Day 56, approximately 10 mL total will be collected. The samples will be immediately processed to avoid possible sample variation caused by freezing and thawing cells.

Day -28: Baseline prevaccination time point #1 – approximately 10 ml for cytokine and chemokine assessment + 90 mL for special assays

Day -14: Baseline prevaccination time point #2 – approximately 10 ml for cytokine and chemokine assessment + 90 mL for special assays

Day 0: Baseline prevaccination time point #3 – approximately 10 ml for immunological assessment (cytokine, chemokine, and antibody responses) + 90mL for special

assays

Day 1: To monitor early innate immune response – approximately 10mL for immunological assessment + 90mL for special assays

Day 3: To monitor intermediate innate immune response – approximately 10mL for immunological assessment + 90mL for special assays

Day 7: To monitor late innate response & early adaptive immune response – approximately 10mL for immunological assessment + 90mL for special assays

Day 28: To monitor the mature adaptive immune response. Prior to 2nd vaccination – approximately 10mL for immunological assessment + 90mL for special assays

Day 56: Final serological assessment (HAI and Nt) – approximately 10mL for immunological assessment

(c) A fraction of the collected venous blood samples will be analyzed as follows to measure the immunological response signature for each volunteer in response to vaccination. The emerging immunologic signatures will be used in systems biology modeling.

(d) ***HAI- and Nt-specific Antibody Response*****:** Using the contract laboratory at [redacted] HAI and Nt-specific and antibody response will be measured at specified time points to confirm the effective influenza immunity and to correlate with the systems biology data.

(e) ***Cytokine and Chemokine Response*****:** Using the Vanderbilt Immunology Core, cytokine and chemokine levels in each venous blood sample will be quantitatively measured using CBA analysis at Days -28, 14, 0, 1, 3, 7, and 28 as listed in (b).

The majority of the collected venous blood samples will be used to isolate immune cell subsets to profile the transcriptome and proteome response in response to vaccination. The gene expression signatures along with the immunologic signatures will be used in the systems approach to identify biomarkers or gene expression signatures that predict an immunological response. Approximately 700 mL of blood will be collected over a two month period from these healthy adults, within the limits of blood that can be obtained per our Institutional Review Board.

(f) **Fractionation of Peripheral Blood into Immune Cell Subsets:** Magnetic cell fractionation using antibody-coated magnetic beads (MACS) is a fast and efficient way to isolate specific immune cell populations directly from blood samples. Using immune subset marker-specific magnetic beads with the fresh blood samples and flow cytometry, we will isolate 6 major immune cell subsets:

1. Neutrophils (PMN)
2. Monocytes
3. NK cells

4. B-cells
5. T-cells
6. Dendritic cells

All isolated immune cell subsets and blood samples will be stored at -80°C. The subjects' samples will be used for in depth systems biology analysis to optimize the discovery of gene expression signatures that predicts an early immunological response and protection.

(g) **Immune Cell Subsets Purity and Activation Status:** Each immune cell subset at Days -28, -14, 0, 1, 3, 7, and 28 as listed above in (b) and (f) will be stained with fluorescently labeled cell-specific lineage marker antibodies and fluorescently-labeled activation antibody markers and analyzed by flow cytometry to ascertain the purity and activation status of each immune cell subset.

(h) **RNA-Seq Transcriptome Sequencing:** Transcript profiling using RNA-Seq will be performed on immune cell samples at Days -28, -14, 0, 1, 3, 7, and 28 as listed above in (b) and (f) to identify and quantify the RNA transcript response after vaccination. Total RNA will be isolated using standard methods and subjected to ultra-high-throughput parallel next generation sequencing.

(i) **Proteomics Profiling:** To identify and quantify changes in the immune cell's proteome after vaccination, iTRAQ and shotgun proteomics using multidimensional liquid chromatography coupled with tandem mass spectrometry will be performed on immune cell samples from volunteers vaccinated against H5N1 influenza with or without AS03 adjuvant at Days -28, -14, 0, 1, 3, 7, and 28 as listed above in (b) and (f).

(j) **Systems Biology Analysis:** Our goal is to identify the unique RNA and protein biomarkers that correlate with the immunological responses from H5N1 vaccines given with or without AS03 adjuvant.

7.3.4 Specimen Preparation, Handling, and Shipping

7.3.4.1 Instructions for Specimen Preparation, Handling, and Storage

Instructions for specimen preparation, handling, and storage are included in the protocol-specific Manual of Procedures (MOP).

7.3.4.2 Specimen Shipment

Instructions for specimen shipment are included in the protocol-specific MOP.

8 STUDY SCHEDULE

8.1 Screening and Enrollment

8.1.1 Day -28, Visit 00A (window: -7, +14 days)

- Potential subjects may be screened for eligibility up to 35 days prior to vaccination. Potential subjects will be provided with a description of the study (purpose and study procedures). Subjects will be asked to read and sign the consent form. The consent form will be signed prior to performing any screening procedures.
- Eligibility criteria will be reviewed with the subject – see Section 5. Documentation will include whether the subject received licensed 2010-2011 and/or 2011-2012 seasonal influenza vaccine, what type (LAIV, TIV, and/or MIV) and the approximate date of vaccination if known. NOTE: Prior receipt of the licensed 2010-2011 and/or 2011-2012 seasonal influenza vaccine is not an exclusion criterion, as long as it has been administered outside the exclusionary window.
- Review current health status.
- Vital signs will be obtained, including oral temperature, pulse, and blood pressure.
- Height and weight will be obtained.
- Medical history will be reviewed.
- All concomitant medications will be recorded and assessed for eligibility.
- A complete physical examination (without genital and rectal exam) will be performed by a study clinician licensed to make medical diagnoses and listed on the Form FDA 1572 as the site principal investigator or sub-investigator.
- Supraclavicular and axillary lymph nodes will be assessed bilaterally by a study clinician licensed to make medical diagnoses and listed on the Form FDA 1572 as the site principal investigator or sub-investigator.
- Additional physical assessments may be performed as indicated.
- A urine or serum pregnancy test will be performed for all female subjects of childbearing potential.
- Urine sample collection for future research.
- Approximately 100 mL of venous blood will be collected for cytokine/chemokine levels and systems biology studies.

8.1.2 Day -14, Visit 00B (window: ± 14 days)

- Urine sample collection for future research.
- Approximately 100 mL of venous blood will be collected for cytokine/chemokine levels and systems biology studies.

8.1.3 Day 0, Visit 01, Dose 1

- Eligibility criteria will be reviewed with the subject – see Section 5.
  - Vaccination of subjects should be deferred until acute illness, including an oral temperature greater than or equal to 100.4°F, has resolved. If resolution then the subject may receive the first vaccination.
- Review current health status and note any changes since the last visit.
- Vital signs will be obtained, including oral temperature, pulse, and blood pressure. **NOTE:** Oral temperature only will be obtained if all vital signs noted above were collected at screening.
- Height and weight will be obtained if not collected at screening.
- Medical history will be reviewed to assure continued eligibility.
- All concomitant medications will be reviewed for accuracy and completeness. Any new medications will be recorded and assessed for continuing eligibility.
- A targeted physical examination may be performed, as indicated, based on subject's recent clinical history since the screening visit.
- Lymph node assessment will be reviewed for any changes based on subject's recent clinical history since the screening visit.
- A urine or serum pregnancy test must be performed within 24 hours prior to vaccination for all female subjects of childbearing potential. Results must be negative and known prior to vaccination. If the pregnancy test is performed > 24 hours prior to vaccination, it must be repeated to determine eligibility.
- Urine sample collection for future research.
- Approximately 100 mL of venous blood will be collected for HAI and Nt antibody titers, cytokine/chemokine levels, and systems biology studies.
- Subjects will be enrolled in IDES and randomly assigned to a group.
- Subjects will receive a single dose of vaccine per randomized assignment via IM injection in the deltoid muscle of the preferred arm. Subjects will be observed in the

clinic for at least 20 minutes following vaccination. The vaccination site will be examined, and any AE/SAEs will be assessed prior to discharge from the clinic.

- Subjects will be provided with a memory aid and other study-related materials to record daily oral temperature and systemic and local AE/SAEs. Subjects will be encouraged to take their temperature around the same time each day. Subjects will be instructed on how to use the memory aid and how to measure and record AE/SAEs prior to discharge from the clinic. Subjects will be instructed to notify the study center if they develop any severe reactions following vaccination. If the site principal investigator or appropriate sub-investigator deems the reaction severe enough, s/he will give further instructions on the proper course of action, including a return to the clinic for immediate evaluation if appropriate.

8.2 Follow-Up

8.2.1 Day 1, Visit 02 (window: +1 day)

- Review current health status and note any changes since the last visit.
- Examine the vaccination site.
- A targeted physical examination may be performed, if indicated based on review of current health status.
- Supraclavicular and axillary lymph nodes will be assessed bilaterally by a study clinician licensed to make medical diagnoses and listed on the Form FDA 1572 as the site principal investigator or sub-investigator.
- Study personnel will review the memory aid information with the subject and record all AE/SAEs and concomitant medications on the appropriate case report form (CRF).
- Urine sample collection for future research.
- Approximately 100 mL of venous blood will be collected for HAI and Nt antibody titers, cytokine/chemokine levels, and systems biology studies.

8.2.2 Day 3, Visit 03 (window: +1 day)

- Review current health status and note any changes since the last visit.
- Examine the vaccination site.
- A targeted physical examination may be performed, if indicated based on review of current health status.

- Supraclavicular and axillary lymph nodes will be assessed bilaterally by a study clinician licensed to make medical diagnoses and listed on the Form FDA 1572 as the site principal investigator or sub-investigator.
- Study personnel will review the memory aid information with the subject and record all AE/SAEs and concomitant medications on the appropriate case report form (CRF).
- Urine sample collection for future research.
- Approximately 100 mL of venous blood will be collected for HAI and Nt antibody titers, cytokine/chemokine levels, and systems biology studies.

8.2.3 Day 7, Visit 04 (window: +2 days)

- Review current health status and note any changes since the last visit.
- Examine the vaccination site.
- A targeted physical examination may be performed, if indicated based on review of current health status.
- Supraclavicular and axillary lymph nodes will be assessed bilaterally by a study clinician licensed to make medical diagnoses and listed on the Form FDA 1572 as the site principal investigator or sub-investigator.
- Study personnel will review the memory aid information with the subject and record all AE/SAEs and concomitant medications on the appropriate case report form (CRF).
- Urine sample collection for future research.
- Approximately 100 mL of venous blood will be collected for HAI and Nt antibody titers, cytokine/chemokine levels, and systems biology studies.

8.2.4 Day 28, Visit 05, Dose 2 (window: ±2 days)

- Eligibility criteria will be reviewed with the subject – see Section 5.
    - Vaccination of subjects should be deferred until acute illness, including an oral temperature greater than or equal to 100.4°F, has resolved. If resolution occurs, then vaccination should be rescheduled within the acceptable window for the second vaccination – see Section 10.3.3.
- Review current health status and note any changes since the last visit.
- Oral temperature will be obtained.

- A targeted physical examination may be performed, if indicated based on review of current health status.
- Supraclavicular and axillary lymph nodes will be assessed bilaterally by a study clinician licensed to make medical diagnoses and listed on the Form FDA 1572 as the site principal investigator or sub-investigator.
- All concomitant medications will be recorded.
- All AE/SAEs will be recorded.
- A urine or serum pregnancy test must be performed within 24 hours prior to vaccination for all female subjects of childbearing potential. Results must be negative and known prior to vaccination. If the pregnancy test is performed > 24 hours prior to vaccination, it must be repeated to determine eligibility.
- Urine sample collection for future research.
- Approximately 100 mL of venous blood will be collected for HAI and Nt antibody titers, cytokine/chemokine levels, and systems biology studies.
- Subjects will receive a single dose of vaccine per randomized assignment via IM injection in the deltoid muscle of the preferred arm. Subjects will be observed in the clinic for at least 20 minutes following vaccination. The vaccination site will be examined, and any AE/SAEs will be assessed prior to discharge from the clinic.
- Subjects will be provided with a memory aid and other study related materials to record daily oral temperature and systemic and local AE/SAEs. Subjects will be encouraged to take their temperature around the same time each day. Subjects will be instructed on how to use the memory aid and how to measure and record any AE/SAEs prior to discharge from the clinic. Subjects will be instructed to notify the study center if they develop any severe reactions following vaccination. If the site principal investigator or appropriate sub-investigator deems the reaction severe enough, s/he will give further instructions on the proper course of action, including a return to the clinic for immediate evaluation if appropriate.

8.2.5 Day 30, Visit 05a, Telephone Call (window: ±1 day)

Study personnel will contact the subject by telephone to collect any AE/SAE and concomitant medication information and review information on the memory aid and remind the subject to complete the information on the memory aid.

8.2.6 Day 35 Visit 06 (window: +2 days)

- Review current health status and note any changes since the last visit.
- Examine the vaccination site.
- A targeted physical examination may be performed, if indicated based on review of current health status.
- Supraclavicular and axillary lymph nodes will be assessed bilaterally by a study clinician licensed to make medical diagnoses and listed on the Form FDA 1572 as the site principal investigator or sub-investigator.
- Study personnel will review the memory aid information with the subject and record all concomitant medications and AE/SAEs on the appropriate CRF.

8.2.7 Day 56, Visit 07 (window: ±2 days)

- Review current health status and note any changes since the last visit.
- A targeted physical examination may be performed, if indicated based on review of current health status.
- Supraclavicular and axillary lymph nodes will be assessed bilaterally by a study clinician licensed to make medical diagnoses and listed on the Form FDA 1572 as the site principal investigator or sub-investigator.
- All concomitant medications will be recorded.
- All AE/SAEs will be recorded.
- Approximately 10 mL of venous blood will be collected for HAI and Nt antibody titers.

8.2.8 Day 208, Visit 08, Follow-up Phone Call (window: ±14 days)

Subjects will be contacted by phone to query for safety events. Adverse events limited to new-onset chronic medical conditions and SAEs that have occurred since the last visit will be collected. Based on the information, subjects may be asked to return to the clinic for evaluation.

Subjects will also be questioned as to whether or not they have received non-study influenza vaccines since the last visit.

8.2.9 Day 393, Visit 09, Follow-up Phone Call (window: ±14 days)

Subjects will be contacted by phone to query for safety events. Adverse events limited to new-onset chronic medical conditions and SAEs that have occurred since the last contact will be collected. Based on the information, subjects may be asked to return to the clinic for evaluation.

Subjects will also be questioned as to whether or not they have received non-study influenza vaccines since the last contact.

8.3 Early Termination Visit

- Review current health status and note any changes since the last visit.
- All concomitant medications will be recorded. Concomitant medications will be restricted to whether or not they have received non-study influenza vaccines since the last contact, if after Day 56 visit.
- Approximately 100 mL of venous blood will be collected for HAI and Nt antibody titers, cytokine/chemokine levels, and systems biology studies (if prior to Day 28 visit) or only approximately 10 mL of venous blood will be collected for HAI and Nt antibody titers (if after Day 28 visit but prior to Day 56 visit).
- A targeted physical examination may be performed, if indicated based on review of current health status.
- Supraclavicular and axillary lymph nodes will be assessed bilaterally by a study clinician licensed to make medical diagnoses and listed on the Form FDA 1572 as the site principal investigator or sub-investigator.
- Information regarding AE/SAEs will be collected. AEs will be restricted to new-onset chronic medical conditions and SAEs that have occurred since the last contact, if after Day 56 visit.

Subjects may voluntarily withdraw their consent for participation in the study at any time and for any reason, without penalty. Subjects may also withdraw voluntarily from receiving the study product for any reason. The site principal investigator may also withdraw a subject from receiving study product. Follow-up safety assessments, immunogenicity evaluations, and systems biology studies will be completed, if possible, whether the subject withdraws from the study or is withdrawn from receiving further study product. Subjects will be encouraged to permit continued follow-up of AE/SAEs and to donate scheduled venous blood samples for immunogenicity and systems biology studies, if possible – see the protocol-specific MOP for alternate follow-up requirements.

9 STUDY INVESTIGATIONAL PRODUCT

9.1 Study Product Acquisition

The vaccine used in this study is a subvirion inactivated monovalent influenza A/H5N1 (HA of A/Indonesia/05/2005) virus vaccine provided in multidose vials containing 20 mcg/mL A/H5N1 HA as determined by single radial immunodiffusion, and manufactured by Sanofi Pasteur. The phosphate buffered saline (PBS) diluent will be provided by sanofi pasteur. The adjuvant for this study is AS03, manufactured by GlaxoSmithKline Biologicals (GSK).

9.1.1 Formulation, Packaging and Labeling

The subvirion inactivated monovalent influenza A/H5N1 virus vaccine and PBS diluent will be provided to the DMID Clinical Agent Repository by Sanofi Pasteur and AS03 adjuvant by GSK, as directed by DHHS.

The subvirion inactivated monovalent influenza A/H5N1 virus vaccine provided by sanofi pasteur will be packaged in multidose vials in concentrations of 20 mcg/mL.

The PBS diluent will be provided by Sanofi Pasteur in single-use vials.

The AS03 adjuvant will be provided by GSK in single-use vials.

Sterile empty vials (10 mL) will be provided by the DMID Clinical Agent Repository.

Further details regarding formulation, packaging and labeling are included in the protocol-specific MOP.

9.2 Product Storage and Stability

The subvirion inactivated monovalent influenza A/H5N1 virus vaccine, AS03 adjuvant and PBS diluent will be stored in a secure temperature-monitored refrigerator at 35.6°F to 46.4°F [2°C to 8°C] until needed. The temperature of the storage unit must be monitored during the duration of the trial, and documentation of proper dedicated storage will be maintained. In the event of accidental deep-freezing or disruption of the cold chain, study products must not be administered and the site principal investigator or the responsible person should contact the DMID Clinical Agent Repository for further instructions. The DMID Clinical Agent Repository information is contained in the protocol-specific MOP.

9.3 Preparation and Administration of Study Intervention/Investigational Products

See the protocol-specific MOP for detailed information on the preparation of vaccine and mixing instructions for each group prior to administration.

All doses of the vaccine with or without adjuvant will be administered as a single 0.5 mL IM injection in the lateral portion of the deltoid muscle of the preferred arm. Aseptic technique will be used for the withdrawal of each dose using a needle appropriate in length for each individual. Once mixed, the admixture should be stored at room temperature (20-25°C) in an upright position and must be used within 8 hours.

Vaccine preparation will be performed by the participating clinical study site pharmacist and administration will be performed by an unblinded vaccine administrator. The unblinded vaccine administrator is a study clinician licensed to administer medications/vaccines who may also participate in dose preparation, but will not be involved in study-related assessments or have subject contact for data collection following vaccine administration. Vaccine must be administered within 15 minutes of drawing into a syringe.

9.4 Accountability Procedures for the Study Intervention/Investigational Product(s)

Vaccine, adjuvant and diluent will be sent to DMID Clinical Agent Repository and then will be supplied to the participating clinical study sites prior to the start of the study. The randomization scheme will be generated by the DCC and provided to unblinded study personnel at the participating clinical study sites.

After receipt of the vaccine, adjuvant and diluent, the site principal investigator is responsible for distribution and disposition of these study products, and has ultimate responsibility for drug accountability. Logs of receipt, temperature, maintenance, and disposal must be maintained in the study file.

Vaccine, adjuvant and diluent (used and unused vials) will be retained until monitored and released for disposition. Final disposition of vaccine, adjuvant and diluent will be determined by DMID and communicated to the participating clinical study sites.

10 ASSESSMENT OF SCIENTIFIC OBJECTIVES

10.1 Specification of the Appropriate Outcome Measures

10.1.1 Primary Outcome Measures

The primary endpoints are:

- Occurrence of vaccine-associated serious adverse events (SAEs) from the time of first vaccination through 13 months after the first vaccination.
- Occurrence of solicited local and systemic reactogenicity in the 8 days (Days 0-7) after each vaccination.
- GMT of HAI antibody, proportion of subjects achieving a serum HAI antibody titer of 1:40 or greater, and frequency of 4-fold or greater increases of HAI antibodies in each group against the subvirion inactivated A/H5N1 virus vaccine approximately 1, 3, 7 and 28 days after receipt of the first dose of vaccine.
- GMT of neutralizing antibody, proportion of subjects achieving a serum neutralizing antibody titer of 1:40 or greater, and frequency of 4-fold or greater increases of neutralizing antibodies in each group against the subvirion inactivated A/H5N1 virus vaccine approximately 1, 3, 7 and 28 days after receipt of the first dose of vaccine.

10.1.2 Secondary Outcome Measures

The secondary endpoints are:

- GMT of HAI antibody, proportion of subjects achieving a serum HAI antibody titer of 1:40 or greater, and frequency of 4-fold or greater increases of HAI antibodies in each group against the subvirion inactivated A/H5N1 virus vaccine 28 days after receipt of the second dose of vaccine (approximately Day 56).
- GMT of neutralizing antibody, proportion of subjects achieving a serum neutralizing antibody titer of 1:40 or greater, and frequency of 4-fold or greater increases of neutralizing antibodies in each group against the subvirion inactivated A/H5N1 virus vaccine 28 days after receipt of the second dose of vaccine (approximately Day 56).

10.1.3 Tertiary Outcome Measures

The tertiary endpoints are:

- RNA, RNA splice junctions, nonsynonymous base substitutions, or protein expression in blood immune cells at Days 1, 3, 7, and 28 after the first

vaccination that differentiates one dose of an AS03-adjuvanted H5N1 vaccine compared to one dose of the unadjuvanted H5N1 vaccine.

- RNA, RNA splice junctions, nonsynonymous base substitutions, or protein expression in blood immune cells at Days 1, 3, 7, and 28 after the first vaccination that correlates with the occurrence of solicited local and systemic reactogenicity 8 days (Days 0-7) after the first dose of an AS03-adjuvanted H5N1 vaccine or the first dose of the unadjuvanted H5N1 vaccine.

10.1.4 Exploratory Outcome Measures

The exploratory endpoints are:

- Exploration of unsolicited adverse events (AEs) reported in the 28 days after each vaccination.
- Occurrence of new-onset chronic medical conditions through 13 months after the first vaccination.
- Development of serum antibody responses against antigenically drifted variants of the H5N1 virus.
- Development of a systems biology model of early human immune response of AS03-adjuvanted and unadjuvanted A/H5N1 virus vaccine that integrates quantitative changes in RNA and protein expression with the innate and humoral immune responses as determined by cytokine/chemokine levels, hemagglutination inhibition (HAI), neutralizing antibody titers (Nt), and immune cell activation status, respectively.
- Identification of early RNA and protein expression signatures in human immune cells that indicate successful immunological response and protection against the influenza pathogen.

10.2 Methods and Timing for Assessing, Recording, and Analyzing Appropriate Outcome Measures

Safety outcome measures will be assessed, recorded and analyzed per Section 11.

Immunogenicity evaluations (Section 7.3.2) will be determined by measuring serum HAI and neutralizing antibody titers (Nt) against the subvirion inactivated influenza A/H5N1 virus vaccine. The pre-immunization and post-immunization antibody responses after the first and second dose of vaccine will be evaluated among the 2 groups. The central (immunology) laboratory information is contained in the protocol-specific MOP.

For systems biology analysis of the subject’s immune and blood cells response to A/H5N1 with or without AS03 adjuvant, cytokine/chemokine levels will be determined in venous blood samples using cytometric bead arrays (CBA) analysis and measured in pg/ml at Days -28, -14, 0, 1, 3, 7, 28 after the first vaccination. Immune cell activation status of immune cell subsets will

be measured using fluorescently labeled cell-specific lineage marker antibodies and a cell-specific activation marker using flow cytometric detection at Days -28, -14, 0, 1, 3, 7, and 28. RNA transcript changes in blood immune cells at Days -28, -14, 0, 1, 3, 7, and 28 after the first vaccination will be determined by RNA-Seq analysis and measured in Reads Per Kilobase of Exon model per Million reads (RPKM). Relative cellular protein changes in subject's blood immune cells at Days -28, -14, 0, 1, 3, 7, and 28 after the first vaccination will be determined by iTRAQ and shotgun proteomics. Refer to Section 7.3.3 for further details on the systems biology studies assays and procedures.

Cross-reacting antibodies will be evaluated by HAI and neutralizing antibodies using one or more H5N1 viruses not included in the vaccine and representing different clades or subclades of H5N1.

10.3 Modification and Discontinuation of Study Intervention/Investigational Product for a Subject

10.3.1 Dose/Schedule Modifications for a Subject

There will be no dose modifications. Subjects who do not receive the second vaccination will be asked to return for safety assessments and for scheduled venous blood sample collections for immunogenicity and systems biology evaluations and will be followed for the duration of the study – see the protocol-specific MOP for further details.

10.3.2 Criteria for Discontinuation of Study Intervention/Product for Withdrawal of a Subject

Subjects may voluntarily withdraw their consent for participation in the study at any time and for any reason, without penalty. Subjects may also withdraw voluntarily from receiving the study product for any reason. The site principal investigator may also withdraw a subject from receiving study product.

Vaccinations will not be administered to a subject if any of the following criteria are met:

- Any unresolved or continuing solicited or unsolicited Grade 2 or greater severity adverse events, including reactogenicity events, that have occurred without a clear, alternative etiology. Unresolved or continuing Grade 1 adverse events are permissible unless, in the opinion of the site principal investigator or appropriate sub-investigator, they would render vaccination unsafe, would interfere with the evaluation of responses or are not generally seen in "normal, healthy subjects".
- Severe or sustained reaction/disability related to receipt of a previous vaccine injection.

- Medical disease or condition, or any new clinical findings for which continued participation, in the opinion of the site principal investigator or appropriate sub-investigator, would compromise the safety of the subject, or would interfere with the subject's successful completion of the study, or would interfere with the evaluation of responses.
- New onset of illness, or medical disease or condition that meets exclusion criteria.
- Presence of Grade 2 or greater severity signs or symptoms that could confound or confuse assessment of vaccine reactogenicity.
- Subject no longer meets inclusion/exclusion criteria (Section 5).
- As deemed necessary by the site principal investigator for noncompliance or other reasons.
- Subject withdrawal of consent.
- Subject refuses further vaccination. Subjects who do not receive the second vaccination will be asked to return for safety assessments and for scheduled venous blood sample collections for immunogenicity and systems biology evaluations and will be followed for the duration of the study – see the protocol-specific MOP for further details.
- Loss to follow-up.
- Termination of the study. Reasons for termination include but are not limited to study closure due to SMC review and at the discretion of DMID.

10.3.3 Deferral of Second Dose of Vaccine

If a subject's second vaccination is deferred, it should be rescheduled to occur within the acceptable window for this visit. If this period elapses, the site must obtain prior approval from the DMID medical monitor and study team to administer the second vaccination. Subjects who do not receive the second vaccination will be asked to return for safety assessments and for scheduled venous blood sample collections for immunogenicity and systems biology evaluations and will be followed for the duration of the study – see the protocol-specific MOP for further details.

11 ASSESSMENT OF SAFETY

11.1 Specification of Safety Parameters

Safety will be based on the frequency and severity of:

1. Vaccine-associated serious adverse events occurring throughout the course of the study.
2. Solicited Adverse Events – reactogenicity following both vaccinations:
   a) Local reactions including pain, tenderness, erythema/redness, and induration/swelling at the injection site.
   b) Systemic reactions including fever, feverishness, chills, shivering, arthralgia/joint pain (exclusive of the injection site), asthenia/weakness, malaise/fatigue, myalgia/body aches (exclusive of the injection site), nausea, headache, and supraclavicular and axillary lymphadenopathy.
3. Unsolicited Adverse Events
   a) Non-serious events occurring 28 days following each dose of vaccine (through approximately Day 56).
4. New-onset chronic medical conditions occurring throughout the course of the study.

11.2 Methods and Timing for Assessing, Recording, and Analyzing Safety Parameters

11.2.1 Adverse Events

Adverse Event (AE): ICH E6 defines an AE as any untoward medical occurrence in a patient or clinical investigation subject administered a pharmaceutical product regardless of its causal relationship to the study treatment. An AE can therefore be any unfavorable and unintended sign (including an abnormal laboratory finding), symptom, or disease temporally associated with the use of medicinal (investigational) product. The occurrence of an AE may come to the attention of study personnel during study visits and interviews of a study recipient presenting for medical care, or upon review by a study monitor.

All AEs including local and systemic reactions not meeting the protocol-defined criteria for serious adverse events (SAEs) will be captured on the appropriate data collection form and eCRF. Information to be collected includes event description, date of onset, study clinician's assessment of severity and relationship to study product and alternate etiology (if not

associated to study product) (assessed only by those with the training and authority to make a diagnosis, which would include MD, PA, Nurse Practitioner, DO, or DDS – i.e., a study clinician licensed to make medical diagnoses and listed on the Form FDA 1572 as the site principal investigator or sub-investigator), date of resolution/stabilization of the event, seriousness and outcome. All AEs occurring while on study will be documented appropriately regardless of relationship. All AEs will be followed to adequate resolution.

Any medical condition that is present at the time that the subject is screened will be considered as baseline and not reported as an AE. However, if the severity of any pre-existing medical condition increases at any time during the study, it will be recorded as an AE.

All AEs must be graded for severity and relationship to study product. AEs characterized as intermittent require documentation of onset and duration of each episode. The start and stop date of each reported AE will be recorded on the appropriate data collection form and eCRF.

FDA defines an AE as any untoward medical occurrence associated with the use of a drug in humans, whether or not considered drug related.

Severity of Event: All AEs will be assessed by the site principal investigator or appropriate sub-investigator using a protocol-defined grading system (Section 11.2.2). For events not included in the protocol-defined grading system, the following guidelines will be used to quantify severity:

- Mild (Grade 1): events require minimal or no treatment and do not interfere with the patient's daily activities.
- Moderate (Grade 2): events result in a low level of inconvenience or concern with the therapeutic measures. Moderate events may cause some interference with functioning and daily activities.
- Severe (Grade3): events interrupt a patient's usual daily activity and may require systemic drug therapy or other treatment. Severe events are usually incapacitating.
- Life-threatening (Grade 4): any adverse drug experience that places the patient or subject, in the view of the site principal investigator, at immediate risk of death from the reaction as it occurred, i.e., it does not include a reaction that had it occurred in a more severe form, might have caused death.

Relationship to Study Products: The study clinician's assessment of an AE's relationship to study product (vaccine or study drug) is part of the documentation process, but it is not a factor in determining what is or is not reported in the study. If there is any doubt as to whether a clinical observation is an AE, the event should be reported. All AEs must have their relationship to study product assessed using the terms: related or not related. In a clinical trial, the study product must always be suspect. To help assess, the following guidelines are used.

- Related – There is a reasonable possibility that the study product caused the adverse event. Reasonable possibility means that there is evidence to suggest a causal relationship between the study product and the adverse event.

- Not Related – There is not a reasonable possibility that the administration of the study product caused the event.

11.2.2 Reactogenicity

Reactogenicity events are AEs that are known to occur with this type of vaccine. Reactogenicity will be analyzed using the following grading systems:

**Local Reaction**	**Mild (Grade 1)**	**Moderate (Grade 2)**	**Severe (Grade 3)**
Pain – experienced without touching the injection site	Subject is aware of pain but it does not interfere with daily activity **and** no pain medication is taken	Subject is aware of pain; there is interference with daily activity **or** it requires use of pain medication	Subject is aware of pain **and** it prevents daily activity
Tenderness – hurts only when injection site is touched	The area immediately surrounding the injection site hurts only when touched **and** it does **not** interfere with daily activity	The area immediately surrounding the injection site hurts when touched **and** it interferes with daily activity	The area immediately surrounding the injection site hurts when touched **and** it prevents daily activity
Erythema/Redness*	Does not interfere with activity	Interferes with activity	Prevents daily activity
Induration/ Swelling*	Does not interfere with activity	Interferes with activity	Prevents daily activity

* will be also measured in mm but size will not be used as halting criteria

Erythema/redness and induration/swelling as analyzed by measurement will be graded as follows:

	**Small**	**Medium**	**Large**
Erythema/Redness*	<20 mm	20-50 mm	>50 mm
Induration/Swelling*	<20 mm	20-50 mm	>50 mm

* measurement will not be used as halting criteria.

Temperature will be graded according to the following grading scales:

**Systemic (Quantitative)**	**Mild (Grade 1)**	**Moderate (Grade 2)**	**Severe (Grade 3)**
Fever (°C)*	38.0-38.4° C	38.5-38.9° C	≥39°C

	100.4-101.1° F	101.2-102° F	≥102.1°F

*Oral temperature, no recent hot or cold beverages or smoking. Note: A fever can be considered not related to the study product if an alternative etiology can be documented and it is confirmed to be not related to the study product by the Independent Safety Monitor at the site.

**Systemic (Subjective)**	**Mild (Grade 1)**	**Moderate (Grade 2)**	**Severe (Grade 3)**
Feverishness	No interference with activity	Some interference with activity	Significant interference, prevents daily activity
Chills	No interference with activity	Some interference with activity	Significant interference, prevents daily activity
Shivering	No interference with activity	Some interference with activity	Significant interference, prevents daily activity
Arthralgia/Joint Pain*	No interference with activity	Some interference with activity	Significant interference, prevents daily activity
Asthenia/Weakness	No interference with activity	Some interference with activity	Significant interference, prevents daily activity
Malaise/Fatigue	No interference with activity	Some interference with activity	Significant interference, prevents daily activity
Myalgia/Body Aches*	No interference with activity	Some interference with activity	Significant interference, prevents daily activity
Headache	No interference with activity	Some interference with activity	Significant interference, prevents daily activity
Nausea	No interference with activity	Some interference with activity	Significant interference, prevents daily activity

*not at injection site

Severity Grading for Lymphadenopathy

The site principal investigator or appropriate sub-investigator will assess the severity of lymphadenopathy in the supraclavicular fossae and axillae bilaterally by applying the scale in the following table separately to each anatomic group of nodes. Data will be collected separately in the eCRF for the anatomic groups on the left and right sides of the body.

**Grade**	**Definition**
[1]Severe (Grade 3)	**At least one of the following criteria:**<br>• At least 1 node of any size that is tender to light touch or spontaneously reported as painful **AND** causing significant limitation of normal everyday activities.<br>**OR**<br>• Evidence of any one of the following: palpable fluctuance or heat, fixation to underlying tissues, or visible erythema, ulceration or drainage.

A positive finding of lymphadenopathy may be at 1 or more sites and be on either one or both sides of the body.

[1]Grade 3 (Severe) lymphadenopathy must be reported on the adverse event form. If ulceration or drainage is present, it must be reported as a SAE.

11.2.3 Serious Adverse Events

Serious Adverse Event (SAE): An adverse event or suspected adverse reaction is considered "serious" if, in the view of either the site principal investigator or sponsor, it results in any of the following outcomes:

- Death,
- A life-threatening adverse event*,
- Inpatient hospitalization or prolongation of existing hospitalization,
- A persistent or significant incapacity or substantial disruption of the ability to conduct normal life functions, or
- A congenital anomaly/birth defect.
- Important medical events that may not result in death, be life-threatening, or require hospitalizations may be considered serious adverse events when, based upon appropriate medical judgment they may jeopardize the patient or subject and may require medical or surgical intervention to prevent one of the outcomes listed in this definition. Examples of such medical events include allergic bronchospasm requiring intensive treatment in an emergency room or at home, blood dyscrasias or convulsions that do not result in inpatient hospitalization, or the development of drug dependency or drug abuse.

*Life-threatening adverse event. An adverse event is considered "life-threatening" if, in the view of either the investigator or sponsor, its occurrence places the patient or subject at immediate risk of death. It does not include an adverse event, had it occurred in a more severe form, might have caused death.

All SAEs will be:

- Assessed by a study clinician licensed to make medical diagnoses and listed on the Form FDA 1572 as the site principal investigator or sub-investigator.
- Recorded on the appropriate SAE form and eCRF.
- Followed through resolution by a study clinician licensed to make medical diagnoses and listed on the Form FDA 1572 as the site principal investigator or sub-investigator.
- Reviewed and evaluated by an Independent Safety Monitor (ISM), the SMC (periodic review unless associated), DMID, and the IRB.

11.2.4 Procedures to be Followed in the Event of Abnormal Laboratory Test Values or Abnormal Clinical Findings

The site principal investigator is responsible for reporting all AE/SAEs that are observed or reported during the study, regardless of their relationship to study product. AE/SAEs will be documented, reported and followed appropriately.

11.3 Reporting Procedures

AEs will be documented from the first study intervention, Visit 01 (Day 0), through Visit 07 (Day 56).

SAEs will be documented from the first study intervention, Visit 01 (Day 0), through Visit 09 (Day 393).

11.3.1 Serious Adverse Events

Any AE that meets a protocol-defined serious criterion must be submitted immediately (within 24 hours of site awareness) on an SAE form to the DMID Pharmacovigilance Group, at the following address:

[redacted]

In addition to the SAE form, selected SAE data fields must also be entered into IDES. (Please see the protocol-specific MOP for details regarding this procedure.)

Other supporting documentation of the event may be requested by the DMID Pharmacovigilance Group and should be provided as soon as possible.

The DMID medical monitor and clinical protocol manager will be notified of the SAE by the DMID Pharmacovigilance Group. The DMID medical monitor will review and assess the SAE for regulatory reporting and potential impact on study subject safety and protocol conduct.

At any time after completion of the study, if the investigator becomes aware of an SAE that is suspected to be related to study product, the investigator will report the event to the DMID Pharmacovigilance Group.

11.3.2 Regulatory Reporting For Studies Conducted Under DMID-Sponsored Investigational New Drug (IND) Application

Following notification from the investigator, DMID, the IND sponsor, will report any suspected adverse reaction that is both serious and unexpected. DMID will report an adverse event as a suspected adverse reaction only if there is evidence to suggest a causal relationship between the study drug and the adverse event. DMID will notify the FDA and all participating site investigators (i.e., all investigators to whom DMID is providing study drug under its INDs or under any investigator's IND) in an IND safety report of potential serious risks from clinical trials or any other source, as soon as possible, but in no case later than 15 calendar days after the sponsor determines that the information qualifies for reporting as specified in 21 CFR Part 312.32. DMID will also notify the FDA of any unexpected fatal or life-threatening suspected adverse reaction as soon as possible, but in no case later than 7 calendar days after the sponsor's initial receipt of the information. Relevant follow-up information to an IND safety report will be submitted as soon as the information is available. Upon request from FDA, DMID will submit to the FDA any additional data or information that the agency deems necessary, as soon as possible, but in no case later than 15 calendar days after receiving the request.

All serious events designated as "not related" to study drug, will be reported to the FDA at least annually in a summary format.

11.3.3 Reporting of Pregnancy

Pregnancies occurring in study subjects will be reported in IDES. No further vaccinations will be administered to pregnant subjects, but all study-mandated venous blood samples will be collected and the subject will continue in follow-up for safety events. Efforts will be made to follow all pregnancies reported during the course of the study to pregnancy outcome pending the subject's permission.

11.4 Type and Duration of Follow-up of Subjects after Adverse Events

AE/SAEs will be followed until resolution even if this extends beyond the study-reporting period. Resolution of an AE/SAE is defined as the return to pretreatment status or stabilization of the condition with the expectation that it will remain chronic.

If the site principal investigator becomes aware of an acute febrile illness and the investigator decides to bring the subject in for an evaluation to determine etiology, then the investigator, at their own discretion, can determine if specific viral testing should be done by either culture or PCR to determine if the infectious agent was influenza and what strain.

11.5 Halting Rules

Further enrollment and vaccinations will be halted for SMC review/recommendation if;

- Any death occurring within the 56 days following administration of the first vaccination that was not the result of trauma or accident.
- Any subject experiences ulceration, abscess, or necrosis at the injection site associated with study product administration.
- Any subject experiences laryngospasm, bronchospasm, or anaphylaxis within 24 hours following administration of study product and associated with study product administration.
- 2 or more subjects experience generalized urticaria within 72 hours of administration of study product and associated with study product administration.
- Any subject experiences a vaccine-related SAE from the time of the first vaccination through the last study visit, unless the subject terminated prior to that time.

The study will also be halted for SMC review/recommendation if, during the 8 days (Days 0-7) after each vaccination, any of the following occurs **in a single group**:

- 2 or more of subjects who received at least one dose of vaccine to date experience the same severe (Grade 3 or higher) vaccine-related local reaction.
- 2 or more of subjects who received at least one dose of vaccine to date experience the same severe (Grade 3 or higher) vaccine-related quantitative systemic reaction.
- 2 or more of subjects who received at least one dose of vaccine to date experience the same severe (Grade 3 or higher) vaccine-related subjective systemic reaction, of which the severity (grade) is corroborated by study personnel.

If any of the halting rules are met following the first or second vaccination, the study will not proceed with the remaining enrollments/vaccinations without a review by and recommendation from the SMC to proceed. Suspension of vaccine administration based on frequencies and severities of AE/SAEs is shown in the following halting rules table:

**Halting Rules** within 8 days (Days 0-7) following vaccination unless otherwise stated	**Local Reaction***	**Systemic Reaction (Quantitative)**	**Systemic Reaction (Subjective)**	**SAE****
Any death occurring within the 56 days following administration of the first vaccination that was not the result of trauma or accident.	Vaccine-related grade 3 in 2 or more of subjects enrolled in a single group for the same local reaction.	Vaccine-related grade 3 in 2 or more of subjects enrolled in a single group for the same quantitative systemic reaction.	Vaccine-related grade 3 in 2 or more subjects enrolled in a single group for the same subjective reaction. The severity (grade) is corroborated by study personnel	Any vaccine-related SAE occurring through the last study visit or subject termination.

*Associated generalized urticaria: occurring within 72 hours of administration of study product and related to study product administration ≥ 2 events across groups will trigger review, or any ulceration, abscess, or necrosis at the injection site associated with study product administration will trigger review.
**Any associated laryngospasm, bronchospasm, or anaphylaxis occurring within 24 hours following vaccine administration will be considered a SAE.

Grading scales for unsolicited adverse events are included in Section 11.2.1. Grading scales for solicited local and systemic reactions are included in Section 11.2.2.

11.6 Safety Oversight (ISM plus SMC)

Safety oversight will be conducted by a Safety Monitoring Committee (SMC) which is an independent group of experts that monitor subject safety and advises DMID. Safety Monitoring Committee members will be separate and independent of study personnel participating in this study and should not have scientific, financial or other conflict of interest related to the study. The SMC will consist of members with appropriate expertise to contribute to the interpretation of the data from this study, and may include Independent Safety Monitors (ISMs). Independent Safety Monitors are physicians with relevant expertise and whose primary responsibility is to provide timely independent safety monitoring. An ISM is assigned to the participating clinical

study site, is in close proximity to the study site and has the authority to readily access study participant records. The ISM reviews any SAE that occurs at the study site in real time and provides an assessment to DMID.

The SMC will review safety data at the following time points:

- At specified times during the course of study as defined in the SMC Charter.
- Ad hoc when a halting rule is met or as needed.

The SMC will operate under the rules of a DMID-approved charter that will be written at the organizational meeting of the SMC. Procedures for SMC reviews/meetings will be defined in the charter. At this time, each data element that the SMC needs to assess will be clearly defined. The SMC will review applicable data to include but not limited to enrollment, demographic, dosing, laboratory and safety data which may include solicited and unsolicited AE/SAEs, concomitant medications, and any physical examinations. Safety data will be reviewed by the SMC per the SMC charter for this study. Interim statistical reports may be generated as deemed necessary and appropriate by DMID. The SMC may receive data in aggregate and presented by group, but without the group assignment identified. The SMC may be unblinded to study group assignment, as needed, to assess safety issues. As an outcome of each review/meeting, the SMC will make a recommendation as to the advisability of proceeding with vaccinations (as appropriate), and to continue, modify or terminate the study.

DMID or the SMC chair may convene the SMC on an ad hoc basis according to protocol criteria or if there are immediate concerns regarding observations during the course of the study. The DMID Medical Monitor is empowered to stop study enrollment and vaccinations if adverse events that meet the halting criteria are reported. The DMID Medical monitor and the ISM will be responsible for reviewing SAEs in real time. The SMC will review SAEs on a regular basis and ad hoc during the study.

12 CLINICAL MONITORING STRUCTURE

12.1 Site Monitoring Plan

Site monitoring will be conducted to ensure that human subject protections, study procedures, laboratory procedures, study intervention administration, and data collection processes are of high quality and meet sponsor, ICH E6, and regulatory guidelines, and that the study is conducted in accordance with the protocol and sponsor standard operating procedures. DMID, the sponsoring agency, or its designee will conduct site-monitoring visits as detailed in the clinical monitoring plan.

Site visits will be made at standard intervals as defined by DMID and may be made more frequently as directed by DMID. Monitoring visits will include, but are not limited to, review of regulatory files, accountability records, eCRFs, informed consent forms, medical and laboratory reports, and protocol compliance. Site monitors will have reasonable access to the study site, study personnel, and all study documentation. Study monitors will meet with the site principal investigator to discuss any problems and actions to be taken and document visit findings and discussions.

13 STATISTICAL CONSIDERATIONS

13.1 Introduction

This is a single center, randomized, double-blinded, controlled, Phase I, small targeted prospective study in healthy male and non-pregnant female subjects, 18 to 49 years old, inclusive, designed to determine the safety, reactogenicity, and immunogenicity of an intramuscular subvirion inactivated monovalent influenza A/H5N1 (HA of A/Indonesia/05/2005) virus vaccine manufactured by Sanofi Pasteur administered at 3.75 mcg per dose given with or without AS03 adjuvant manufactured by GSK. In the study, each subject will receive two doses administered 28 days apart. Using venous blood samples collected from all subjects, vaccine-specific hemagglutination inhibition (HAI) antibody titers and neutralization (Nt) antibody titers will be used to measure the baseline immune response at Day 0 (prior to vaccination) and the immunogenicity of the vaccines at Days 1, 3, 7, and 28 (prior to the second dose of vaccine) after the first vaccination and 28 days after the second vaccination (Day 56). Reactogenicity will be collected during the 8 days following each vaccination (Days 0-7 and Days 28-35, respectively). All subjects will be monitored for AEs through Day 56 and SAEs and new-onset chronic medical conditions through Day 393. In addition, the study will use a systems biology approach to assess the human early gene and protein signatures expressed at Days 1, 3, 7, and 28 after the first vaccination. The systems data will be integrated with immunogenicity and reactogenicity data to develop a systems model of the human immune response to A/H5N1 vaccine with or without AS03 adjuvant.

13.2 Study Objectives

Objectives:

Primary Objectives:

- To examine the safety and tolerability of subvirion inactivated A/H5N1 virus vaccine mixed with the AS03 adjuvant in healthy adults.
- To determine the potential for AS03 adjuvant to enhance the early development of vaccine-specific hemagglutination inhibition (HAI) antibody titers and neutralization (Nt) antibody titers approximately 1, 3, 7, and 28 days following receipt of the first dose of subvirion inactivated A/H5N1 virus vaccine administered at 3.75 mcg per dose in healthy adults.

Secondary Objective:

- To determine the potential for AS03 adjuvant to enhance the development of vaccine-specific hemagglutination inhibition (HAI) antibody titers and neutralization

(Nt) antibody titers approximately 28 days following receipt of the second dose of subvirion inactivated A/H5N1 virus vaccine administered at 3.75 mcg per dose in healthy adults.

Tertiary Objectives:

- To identify differentially expressed messenger RNAs (mRNA), noncoding RNAs (ncRNA), RNA splice junctions, and nonsynonymous base substitutions in human immune blood cells as determined by RNA-Seq analysis at Days 1, 3, 7, and 28 after vaccination with the first dose of A/H5N1 virus vaccine with and without AS03 adjuvant.
- To identify differentially expressed cellular proteins in human immune blood cells as determined by shotgun proteomics at Days 1, 3, 7, and 28 after vaccination with the first dose of A/H5N1 virus vaccine with and without AS03 adjuvant.

Endpoints:

Primary Endpoints:

- Occurrence of vaccine-associated serious adverse events (SAEs) from the time of first vaccination through 13 months after the first vaccination.
- Occurrence of solicited local and systemic reactogenicity in the 8 days (Days 0-7) after each vaccination.
- GMT of HAI antibody, proportion of subjects achieving a serum HAI antibody titer of 1:40 or greater, and frequency of 4-fold or greater increases of HAI antibodies in each group against the subvirion inactivated A/H5N1 virus vaccine approximately 1, 3, 7 and 28 days after receipt of the first dose of vaccine.
- GMT of neutralizing antibody, proportion of subjects achieving a serum neutralizing antibody titer of 1:40 or greater, and frequency of 4-fold or greater increases of neutralizing antibodies in each group against the subvirion inactivated A/H5N1 virus vaccine approximately 1, 3, 7 and 28 days after receipt of the first dose of vaccine.

Secondary Endpoints:

- GMT of HAI antibody, proportion of subjects achieving a serum HAI antibody titer of 1:40 or greater, and frequency of 4-fold or greater increases of HAI antibodies in each group against the subvirion inactivated A/H5N1 virus vaccine 28 days after receipt of the second dose of vaccine (approximately Day 56).
- GMT of neutralizing antibody, proportion of subjects achieving a serum neutralizing antibody titer of 1:40 or greater, and frequency of 4-fold or greater increases of neutralizing antibodies in each group against the subvirion

inactivated A/H5N1 virus vaccine 28 days after receipt of the second dose of vaccine (approximately Day 56).

Tertiary Endpoints:

- RNA, RNA splice junctions, nonsynonymous base substitutions, or protein expression in blood immune cells at Days 1, 3, 7, and 28 after the first vaccination that differentiates one dose of an AS03-adjuvanted H5N1 vaccine compared to one dose of the unadjuvanted H5N1 vaccine.
- RNA, RNA splice junctions, nonsynonymous base substitutions, or protein expression in blood immune cells at Days 1, 3, 7, and 28 after the first vaccination that correlates with the occurrence of solicited local and systemic reactogenicity 8 days (Days 0-7) after the first dose of an AS03-adjuvanted H5N1 vaccine or the first dose of the unadjuvanted H5N1 vaccine.

Exploratory Endpoints:

- Exploration of unsolicited adverse events (AEs) reported in the 28 days after each vaccination.
- Occurrence of new-onset chronic medical conditions through 13 months after the first vaccination.
- Development of serum antibody responses against antigenically drifted variants of the H5N1 virus.
- Development of a systems biology model of early human immune response of AS03-adjuvanted and unadjuvanted A/H5N1 virus vaccine that integrates quantitative changes in RNA and protein expression with the innate and humoral immune responses as determined by cytokine/chemokine levels, hemagglutination inhibition (HAI), neutralizing antibody titers (Nt), and immune cell activation status, respectively.
- Identification of early RNA and protein expression signatures in human immune cells that indicate successful immunological response and protection against the influenza pathogen.

13.3 Study Population

The study population for this protocol is healthy male and non-pregnant female subjects, 18 to 49 years old, inclusive. The subjects must not have a known allergy to eggs or other components of the vaccine or squalene-based adjuvants, must consent to all protocol requirements, and meet additional inclusion and exclusion criteria specified in Section 5 that are typical for such studies. The subjects will be recruited from the general population at the participating clinical study site.

13.4 Study Design

This is a single center, randomized, double-blinded, controlled, Phase I, small targeted prospective study in healthy male and non-pregnant female subjects, 18 to 49 years old, inclusive, designed to determine the safety, reactogenicity, and immunogenicity of an intramuscular subvirion inactivated monovalent influenza A/H5N1 (HA of A/Indonesia/05/2005) virus vaccine manufactured by Sanofi Pasteur administered at 3.75 mcg per dose given with or without AS03 adjuvant manufactured by GSK. In the study, each subject will receive two doses administered 28 days apart. This study will use a systems biology approach to assess the human early gene and protein signatures expressed at Days 1, 3, 7, and 28 after the first vaccination. The systems data will be integrated with immunogenicity and reactogenicity data to develop a systems model of the human immune response to A/H5N1 vaccine with or without AS03 adjuvant.

The proposal will use venous blood samples and subject data collected from a total of twenty H5N1 vaccinated subjects divided into two equal groups. The first group (n=10) will be vaccinated with 3.75µg of H5N1 hemagglutinin plus AS03. The second group (n=10) will be vaccinated with 3.75µg of H5N1 hemagglutinin alone. Venous blood samples (approximately 100 mL) will be collected from all subjects at Days -28, -14, and 0 (prior to vaccination) and at Days 1, 3, 7, and 28 (prior to the second dose of vaccine) after the first vaccination for systems biology studies (cytokine/chemokine profiles, immune activation status, and transcriptome and proteome profiles). Serologic assessment (HAI and Nt) will also be conducted at each of these visits except on Days -28 and -14. Using a systems biology approach, we will identify and quantify the early changes in the subject’s serum cytokine/chemokine levels, and immune cell activation status, and the whole transcriptome and proteome of the major immune cells after the first vaccination. The venous blood sample collected at Days -28, -14, and 0 will be used to measure baseline serum chemokine and cytokine levels, immune activation status, and the whole transcriptome and proteome levels of the major blood immune cells prior to the first vaccination. In addition, at the Day 28 follow-up visit, subjects will be given a second dose of the same vaccine that they received initially, since two doses of vaccine are required to generate a protective immune response in the majority of vaccines. After the second dose of vaccine is given, a final blood sample (approximately 10 mL) will be collected for serologic assessment (HAI and Nt) only at 28 days after the second vaccination (Day 56). The systems biology studies will be conducted only after the first vaccine dose since we are seeking to determine the initial responses associated with vaccine and adjuvant, and not the booster responses. A urine sample will be collected from each subject at Days -28, -14, 0, 1, 3, 7, and 28 for future research.

Vaccine reactions will be assessed for at least 20 minutes following each vaccination on Days 0 and 28, and continue through 8 days following each vaccination. The vaccination site will be examined at the end of the 20-minute observation period following each vaccination and in the clinic on Days 1, 3, 7 and 35. Subjects will be asked to record oral temperature, solicited

vaccine reactions and any unsolicited AE/SAEs on a memory aid for 8 days following the each vaccination (Days 0-7 and Days 28-35, respectively). Subjects will be encouraged to take their temperature around the same time each day. Each subject will be seen in the clinic on Days 1, 3, 7, 28, 35 and 56 to review AE/SAEs, concomitant medications, health status and the events recorded on the memory aid. Each subject will be contacted by telephone 1-3 days following the second vaccination (Day 30) to review the memory aid, AE/SAEs and concomitant medications. Subjects will also be contacted by telephone at 6 months and 1 year following the second vaccination (Days 208 and 393, respectively) to review serious adverse events and new-onset chronic medical conditions. Based on this information, subjects may be asked to return to clinic to be evaluated.

The duration of the study for each subject will be approximately 14 months.

Further details of all study procedures and evaluations to be performed at each subject visit by study day are described in Sections 4, 7 and 8.

See Appendix A for Schedule of Procedures/Evaluations.

13.5 Study Outcome Measures

Safety will be based on the frequency and severity of:

1. Vaccine-associated serious adverse events occurring throughout the course of the study.

2. Solicited Adverse Events – reactogenicity following both vaccinations:
   a) Local reactions including pain, tenderness, erythema/redness, and induration/swelling at the injection site.
   b) Systemic reactions including fever, feverishness, chills, shivering, arthralgia/joint pain (exclusive of the injection site), asthenia/weakness, malaise/fatigue, myalgia/body aches (exclusive of the injection site), nausea, headache, and supraclavicular and axillary lymphadenopathy.

3. Unsolicited Adverse Events
   a) Non-serious events occurring 28 days following each dose of vaccine (through approximately Day 56).

4. New-onset chronic medical conditions occurring throughout the course of the study.

Immunogenicity will be based on H5N1 vaccine strain-specific serum HAI and Nt antibody titers measured prior to the first vaccination on Day 0 and on Days 1, 3, 7 and 28 (prior to the second dose of vaccine) after first vaccination, and at Day 56 after the second vaccination.

For systems biology analysis of the subject's immune and blood cells response to A/H5N1 with or without AS03 adjuvant, cytokine/chemokine levels will be determined in venous blood samples using cytometric bead arrays (CBA) analysis and measured in pg/ml at Days -28, -14, 0, 1, 3, 7, and 28 after the first vaccination. Immune cell activation status of immune cell subsets will be measured using fluorescently labeled cell-specific lineage marker antibodies and a cell-specific activation marker using flow cytometric detection at Days -28, -14, 0, 1, 3, 7, and 28. RNA transcript changes in blood immune cells at Days -28, -14, 0, 1, 3, 7, and 28 after the first vaccination will be determined by RNA-Seq analysis and measured in Reads Per Kilobase of Exon model per Million reads (RPKM). Relative cellular protein changes in subject's blood immune cells at Days -28, -14, 0, 1, 3, 7, and 28 after the first vaccination will be determined by iTRAQ and shotgun proteomics.

Cross-reacting antibodies will be evaluated by HAI and neutralizing antibodies using one or more H5N1 viruses not included in the vaccine and representing different clades or subclades of H5N1.

13.6 Study Hypotheses

This is a Phase I study and is not designed to test a specific hypothesis. Rather, it is intended to examine the safety, reactogenicity, and immunogenicity of an adjuvant mixed with the influenza A/H5N1 virus vaccine, and gather data for a systems biology analysis.

13.7 Sample Size Considerations

Approximately twenty subjects will be enrolled and randomized; 10 to receive sanofi H5N1 antigen 3.75 mcg plus GSK AS03 adjuvant, and 10 to receive sanofi H5N1 antigen 3.75 mcg plus PBS diluent. If a subject withdrawals after randomization but before the first vaccination, the subject may be replaced. If a subject withdraws after vaccination the subject will not be replaced. The sample size for each dose group in this Phase I study is based on practical considerations with the goal of gathering information on the safety, tolerability, and immunogenicity of the vaccine. The sample size is not based on a formal hypothesis. No formal hypothesis tests will be performed.

13.8 Subject Enrollment and Follow-up

Subjects for this study will be recruited from one participating VTEU site that has substantial experience conducting influenza vaccine studies. Based on the accrual rate for this study, it seems reasonable to expect that the VTEU will be able to enroll the study in a timely fashion. Subjects who are enrolled and randomized but do not receive vaccine may be replaced. Subjects who are enrolled, randomized, and vaccinated and subsequently withdraw from study or are lost to follow-up will not be replaced.

Follow-up will consist of 2 segments. The first encompasses the core data for this study and will consist of results for all visits and telephone calls through Day 56. The second segment consists of a safety follow-up assessment at approximately 7 months and 13 months after the completion of the first vaccination. Unblinding and analysis of the results for the core data set will not be delayed for the 7- or 13-month follow-up. Appropriate measures will be taken to minimize the risk of biased data collection at 7 and 13 months due to any results available at that time.

13.9 Interim Analysis

An analysis of antibody responses comparing venous blood samples collected on Day 0, 1, 3, 7, 28 and 56 after the first vaccination is likely to be performed before the final 13-month follow up is complete. Public health needs may also dictate interim safety and/or immunogenicity analysis be performed on all available information to date. Note that none of these analyses represent a formal interim analysis, such as those associated for example with group sequential methods. In particular, there is no plan to halt the trial prior to full enrollment and follow-up based on immunogenicity results. It is not anticipated that early examination of immunogenicity results will have an impact thus no adjustments considering Type I error are currently specified. Additionally, the study will be monitored to determine if any of the safety halting rules described in Section 11.5 are met.

13.9.1 Safety Review

A Safety Monitoring Committee (SMC) will be convened by DMID to review participant safety data which may include solicited and unsolicited AE/SAEs, concomitant medications, and any physical examinations. Safety data will be reviewed by the SMC per the SMC charter for this study. Interim statistical reports may be generated as deemed necessary and appropriate by DMID. The SMC may receive data in aggregate and presented by group, but without the group assignment identified. The SMC may be unblinded to group assignment, as needed, to assess safety issues. As an outcome of each review/meeting, the SMC will make a recommendation at that time as to the advisability of proceeding with vaccinations, and to continue, modify or terminate the study.

13.9.2 Immunogenicity Review

The immunogenicity data may be evaluated prior to the completion of the study. This analysis is not an interim analysis, but the final analysis for these data. Therefore no adjustment will be made to Type I error for this or any other comparison.

13.10 Final Analysis Plan

13.10.1 Safety

Solicited AEs will be analyzed by taking the most severe response over the follow-up period, dichotomizing into a binary variable (none versus mild, moderate or severe) and using standard techniques, such as exact confidence intervals, to summarize the reactogenicity rates. The possibility of an adjuvant response relationship will be explored using logistic regression. Analyses will be conducted separately for each vaccination. Both vaccinations will also be compared, for example, to determine if the response to the first dose is predictive of the response to the second dose.

Unsolicited AEs will be coded by the Medical Dictionary for Regulatory Activities (MedDRA®) for preferred term and system organ class. The rate and exact 95% confidence intervals of AEs in aggregate, and by MedDRA® categories, will be computed. The number of SAEs is likely to be small in this study and will be reported by a detailed listing showing the type, MedDRA® coding, relevant dates (vaccinations and adverse events), severity, relatedness, and outcome for each event.

13.10.2 Immunogenicity

Immunogenicity assessments at Days 0 (prior to first vaccination), 1, 3, 7, and 28 (prior to the second dose of vaccine) after the first vaccination and 28 days (Day 56) after the second vaccination will be measured by H5-specific HAI and Nt titers. Geometric mean titers (GMT) of antibodies and their confidence intervals will be computed at all time points for each treatment group by transforming results to a logarithmic scale, assuming asymptotic normality conditions were satisfied on this scale and converting back to the original scale. The proportion of subjects with a four-fold rise at Days 1, 3, 7, 28 and 56 and the proportion of subjects with antibody titers of 1:40 or greater at each times point will be computed for each treatment group. Exact confidence intervals will be reported for all proportional endpoints. The correlation between HAI and Nt will be explored.

13.10.3 Systems Biology

For final systems biology analysis of the subjects' blood cells response to A/H5N1 vaccination with or without ASO3 adjuvant, cytokine/chemokine levels will be determined in venous blood samples using cytometric bead arrays (CBA) analysis and measured in pg/ml at Days -28, -14, 0, 1, 3, 7, and 28 after the first vaccination. Immune cell activation status of immune cell subsets will be measured using fluorescently labeled cell-specific lineage marker antibodies and a cell-specific activation marker using flow cytometric detection at Days -28, -14, 0, 1, 3, 7, and 28.

RNA transcript changes in blood immune cells at Days -28, -14, 0, 1, 3, 7, and 28 after the first vaccination will be determined by RNA-Seq analysis and measured in Reads Per Kilobase of Exon model per Million reads (RPKM). Relative cellular protein changes in subject's blood immune cells at Days -28, -14, 0, 1, 3, 7, and 28 after the first vaccination will be determined by iTRAQ and shotgun proteomics. From the RNA-Seq and shotgun proteomics data, we will identify differentially expressed messenger RNAs (mRNA), noncoding RNAs (ncRNA), RNA splice junctions, nonsynonymous base substitutions, and cellular protein in human immune blood cells with an absolute value of the $\log_2$ change >1 and a false discovery rate (FDR) < 0.05 at Days 1, 3, 7, and 28 after vaccination with the first dose of A/H5N1 virus vaccine with and without ASO3 adjuvant.

Parametric and nonparametric statistical methods, such as hierarchical clustering, Student *t*-test, ANOVA, and other approaches will be used to identify RNA and protein expression differences that are $\log_2$ difference >1 with a FDR < 0.05 between subjects vaccinated with A/H5N1 virus vaccine with and without AS03 adjuvant to identify and select the most significant changes in RNA and protein expression. Ingenuity Pathway Analysis will be used to annotate the RNAs and proteins that are differentially expressed significantly with pathway, chromosomal and gene ontology information.

14 DATA COLLECTION FORMS AND ACCESS TO SOURCE DATA/DATA COLLECTION FORMS

Each participating clinical study site will maintain appropriate medical and research records for this trial, in compliance with ICH E6, Section 4.9 and regulatory and institutional requirements for the protection of confidentiality of subjects. Each participating clinical study site will permit authorized representatives of the DMID, its designees, and appropriate regulatory agencies to examine (and when required by applicable law, to copy) clinical records for the purposes of quality assurance reviews, audits, and evaluation of the study safety and progress. These representatives will be permitted access to all source data, which include, but are not limited to, hospital records, clinical and office charts, laboratory notes, memoranda, evaluation checklists, pharmacy dispensing records, recorded data from automated instruments, copies or transcriptions certified after verification as being accurate and complete, microfiches, photographic negatives, microfilm or magnetic media, x-rays, and subject files and records kept at the pharmacy, at the laboratories, and medico-technical departments involved in the clinical trial. Source data are all information, original records of clinical findings, observations, or other activities in a clinical trial necessary for the reconstruction and evaluation of the trial. Forms for data collection will be derived from the eCRFs and be provided by the (DCC).

15 QUALITY CONTROL AND QUALITY ASSURANCE

Following a written DMID-accepted site quality management plan, the participating clinical study site is responsible for conducting routine quality assurance (QA) and quality control (QC) activities to internally monitor study progress and protocol compliance. The site principal investigator will provide direct access to all trial-related sites, source data/data collection forms, and reports for the purpose of monitoring and auditing by the sponsor, and inspection by local and regulatory authorities. The site principal investigator will ensure all study personnel are appropriately trained and applicable documentations are maintained on site.

DMID-designated clinical monitors will verify that the clinical trial is conducted and data are generated, documented (recorded), and reported in compliance with the protocol, GCP, MOP and the applicable regulatory requirements. Clinical monitoring reports will be submitted to DMID.

The DCC will implement quality control procedures beginning with the data entry system and generate data quality control checks that will be run on the database. Any missing data or data anomalies will be communicated to the site(s) for clarification and resolution.

16 ETHICS/PROTECTION OF HUMAN SUBJECTS

16.1 Ethical Standards

The site principal investigator will ensure that this study is conducted in full conformity with the principles of the Belmont Report: Ethical Principles and Guidelines for the Protection of Human Subjects of Research of the National Commission for the Protection of Human Subjects of Biomedical and Behavioral Research (April 18, 1979) and codified in 45 CFR 46, 21 CFR 50 and 56, and ICH E6; 62 Federal Regulations 25691 (1997), if applicable. The site principal investigator's Institution will hold a current Federal Wide Assurance (FWA) issued by OHRP for federally funded research.

16.2 Institutional Review Board

Prior to enrollment of subjects into this trial, the approved protocol and the informed consent form will be reviewed and approved by the appropriate IRB listed on their FWA.

The responsible official for the IRB will sign the IRB letter of approval of the protocol prior to the start of this trial and a copy will be provided to DMID. The IRB Federal Wide Assurance number will be provided to DMID.

Should amendments to the protocol be required, the amendments will be written by the sponsor and provided to the site principal investigator for submission to the IRB.

16.3 Informed Consent Process

The site principal investigator will choose subjects in accordance with the eligibility criteria detailed in Section 5. All subjects must sign an informed consent form that complies with the requirements of 21 CFR Part 50 and 45 CFR 46.

Informed consent is a process that is initiated prior to the individual's agreeing to participate in the study and continuing throughout the individual's study participation. Prior to participation in the trial, subjects will receive a comprehensive explanation of the proposed procedures and vaccine, including the nature and risks of the trial, alternate therapies, any known AEs, the investigational status of the components, and the other elements that are part of obtaining proper informed consent. Subjects will also receive a detailed explanation of the proposed use and disclosure of their protected health information, including specifically their serum samples. Subjects will be allowed sufficient time to consider participation in the trial, after having the nature and risks of the trial explained to them, and having the opportunity to discuss the study with their family, friends or legally authorized representative or think about it prior to agreeing to participate.

Consent forms describing in detail the study interventions/products, study procedures, risks and possible benefits are given to the subject. Consent forms must not include any exculpatory statements. Consent forms will be IRB-approved and the subject will be asked to read and review the document. Upon reviewing the document, the site principal investigator will explain the research study to the subject and answer any questions that may arise. The subjects must sign the informed consent form and written documentation of informed consent is required prior to starting any procedures/interventions being done specifically for the study including administering study product.

DMID will provide the site principal investigator, in writing, any new information that significantly bears on the subjects' risk to receive the investigational product. This new information will be communicated by the site principal investigator to subjects who consent to participate in the trial in accordance with IRB requirements. The informed consent document will be updated and subjects will be re-consented, if necessary.

Study personnel may employ recruitment efforts prior to the subject consenting; however, before any protocol-specific procedures are performed to determine protocol eligibility an informed consent form must be signed. Subjects will be given a copy of all consent forms that they sign.

By signing the informed consent form, the subject agrees to complete all evaluations required by the trial, unless the subject withdraws voluntarily or is terminated from the trial for any reason. The rights and welfare of the subjects will be protected by emphasizing to them that the quality of their medical care will not be adversely affected if they decline to participate in this study.

16.4 Exclusion of Women, Minorities, and Children (Special Populations)

This study will be inclusive of all healthy adults who meet the eligibility criteria detailed in Section 5, regardless of religion, sex, or ethnic background. Should the outcome of this study be deemed acceptable, additional trials may be initiated in other populations.

16.5 Subject Confidentiality

Subjects will have code numbers and will not be identified by name. Subject confidentiality is strictly held in trust by the site principal investigator, their study personnel, the sponsor(s), and their agents. Access to study documents will be limited to the site principal investigator and Vanderbilt VTEU staff, the FDA, the Vanderbilt IRB, the National Institutes of Health, and its authorized associates. This confidentiality is extended to cover testing of biological samples, in addition to the clinical information relating to participating subjects

The study protocol, documentation, data, and all other information generated will be held in strict confidence. No information concerning the study or the data will be released to any unauthorized third party without prior written approval of the sponsor.

All information provided by the Sponsor and all data and information generated by the participating clinical study site as part of the study (other than a subject's medical records) will be kept confidential by the site principal investigator and other study personnel. This information and data will not be used by the site principal investigator or other study personnel for any purpose other than conducting the study. These restrictions do not apply to: (1) information which becomes publicly available through no fault of the site principal investigator or other study personnel; (2) information which it is necessary to disclose in confidence to an IRB solely for the evaluation of the study; (3) information which it is necessary to disclose in order to provide appropriate medical care to a study subject; or (4) study results which may be published as described in Section 18. If a written contract for the conduct of the study which includes confidentiality provisions inconsistent with this statement is executed, that contract's confidentiality provisions shall apply rather than this statement.

The study monitor or other authorized representatives of the sponsor may inspect all documents and records required to be maintained by the site principal investigator. This includes, but is not limited to, medical records (office, clinic, or hospital) and pharmacy records for the subjects in this study. The participating clinical study site will permit access to such records.

16.6 Study Discontinuation

If the study is discontinued, enrolled subjects will continue to be followed for safety assessments. No further doses of vaccine will be administered.

16.7 Costs, Subject Compensation, Research Related Injuries

There is no cost to the subject for taking part in this study.

Subjects may be compensated for their participation in this study. Compensation will be in accordance with the local IRB's policies and procedures, and subject to IRB approval.

If it is determined by the participating clinical study site and the site principal investigator that an injury occurred as a direct result of the tests or treatments that are done for research, then the subject and/or the subject's insurance will not have to pay for the cost of immediate medical care provided at the participating clinical study site to treat the injury. There are no plans for the participating clinical study site to pay for the costs of any additional care. There are no plans for the participating clinical study site to give the subject money for the injury.

16.8 Future Use of Stored Specimens

Subjects will be asked for permission to keep any remaining specimen for possible use in future research studies, such as testing for antibodies against other viruses or bacteria. A urine sample will be collected from each subject at Days -28, -14, 0, 1, 3, 7, and 28 for future research. Some samples may be stored at the local site and some at a central clinical storage facility. Samples may be shared with other investigators at other institutions. The samples will not be sold or used directly for production of any commercial product. No human genetic tests will be performed on samples. Each sample will be encoded (labeled) *only* with a barcode and a unique tracking number to protect subject's confidentiality.

There are no benefits to subjects in the collection, storage and subsequent research use of specimens. Reports about future research done with subject's samples will NOT be kept in their health records. Subjects may be given the option to decide if they want their samples to be used for future research or have their samples destroyed at the end of the study. A subject's decision can be changed at any time prior to the end of the study by notifying the study doctors or nurses in writing. However, if a subject consents to future use and some of their venous blood or serum or urine has already been used for research purposes, the information from that research may still be used.

17 DATA HANDLING AND RECORD KEEPING

The site principal investigator is responsible to ensure the accuracy, completeness, legibility, and timeliness of the data reported.

Data collection forms will be derived from the electronic case report form (eCRF) and provided by the DCC to record and maintain data for each subject enrolled in the study. All data collection forms should be completed in a neat, legible manner to ensure accurate interpretation of data. Black or blue ink is required to ensure clarity of reproduced copies. When making a change or correction, cross out the original entry with a single line and initial and date the change. Do not erase, overwrite, or use correction fluid or tape on the original.

Copies of the electronic CRF (eCRF) will be provided for use as data collection forms and maintained for recording data for each subject enrolled in the study. Data reported in the eCRF derived from the data collection forms should be consistent with the data collection forms or the discrepancies should be explained.

The sponsor and/or its designee will provide guidance to site principal investigators and other study personnel on making corrections to the data collection forms and eCRF.

17.1 Data Management Responsibilities

All data collection forms must be reviewed by the clinical team and data entry personnel, who will ensure that they are accurate and complete. Adverse events must be assessed for severity and relationship, and reviewed by the site principal investigator or appropriate sub-investigator.

Data collection is the responsibility of the study personnel at the participating clinical study site under the supervision of the site principal investigator. During the study, the site principal investigator must maintain complete and accurate documentation for the study.

The Data Coordinating Center (DCC) for this study will be responsible for data management, quality review, analysis, and reporting of the study data.

17.2 Data Capture Methods

Clinical data (including AE/SAEs, concomitant medications, physical assessments, and reactogenicity data) and immunogenicity data will be entered into a 21 CFR Part 11-compliant IDES provided by the DCC. The data system includes password protection and internal quality checks, such as automatic range checks to identify data that appear inconsistent, incomplete, or inaccurate. Clinical data will be entered directly from the data collection forms completed by the study personnel. Immunogenicity data will be transferred directly into IDES from the respective

central labs by secure data transmission that includes similar automatic range checks as well as other logic validations at the time of transfer.

17.3 Types of Data

Data for this study will include safety, laboratory and outcome measures (e.g., reactogenicity, immunogenicity, and processed systems biology data and analyses).

17.4 Timing/Reports

Safety data will be reviewed by the SMC per the SMC charter for this study. Interim statistical reports may be generated as deemed necessary and appropriate by DMID. The SMC may receive data in aggregate and presented by group, but without the group assignment identified. The SMC may be unblinded to group assignment, as needed, to assess safety issues. As an outcome of each review/meeting, the SMC will make a recommendation at that time as to the advisability of proceeding with vaccinations (as appropriate), and to continue, modify or terminate the study.

17.5 Study Records Retention

Records and documents pertaining to the conduct of this study, including eCRFs, data collection forms, consent forms, laboratory test results, and study product accountability records, must be retained by the site principal investigator for at least 2 years following approval of a Biologics License Application in an ICH region (the date a marketing application is approved for the vaccine); and until there are no pending or contemplated marketing applications; in an ICH region or, if no application is to be filed or if the application is not approved for such indication, until at least 2 years have elapsed since the formal discontinuation of clinical development of the investigational product and the FDA is notified or until DMID authorizes transfer or destruction of study records and documents. These documents should be retained for a longer period, however, if required by local regulations. No study records will be destroyed without prior authorization and written consent of the sponsor, if applicable. It is the responsibility of the sponsor to inform the site principal investigator when these documents no longer need to be retained.

17.6 Protocol Deviations

A protocol deviation is any noncompliance with the protocol, GCP or MOP requirements. The noncompliance may be either on the part of the subject, the site principal investigator, or the study personnel. As a result of deviations, corrective actions are to be developed by the site and implemented promptly.

These practices are consistent with ICH E6:

4.5 Compliance with Protocol, sections 4.5.1, 4.5.2, and 4.5.3
5.1 Quality Assurance and Quality Control, section 5.1.1
5.20 Noncompliance, sections 5.20.1, and 5.20.2.

It is the responsibility of the site to use continuous vigilance to identify and report deviations within 5 working days of identification of the protocol deviation, or within 5 working days of the scheduled protocol-required activity. All deviations must be promptly reported to DMID, via the DCC's IDES.

All deviations from the protocol must be addressed in study subject data collection forms. A completed copy of the DMID Protocol Deviation (PD) Form must be maintained in the regulatory file, as well as in the subject's chart. Protocol deviations must be sent to the local IRB per their guidelines. The site principal investigator and/or study personnel are responsible for knowing and adhering to their IRB requirements.

18 PUBLICATION POLICY

All investigators funded by the NIH must submit or have submitted for them to the National Library of Medicine's PubMed Central an electronic version of their final, peer-reviewed manuscripts upon acceptance for publication, to be made publicly available no later than 12 months after the official date of publication. The NIH Public Access Policy ensures the public has access to the published results of NIH funded research. It requires investigators to submit final peer-reviewed journal manuscripts that arise from NIH funds to the digital archive PubMed Central upon acceptance for publication. Further, the policy stipulates that these papers must be accessible to the public on PubMed Central no later than 12 months after publication.

Refer to:

- http://publicaccess.nih.gov/
- http://grants.nih.gov/grants/guide/notice-files/NOT-OD-08-033.html

Following completion of the study, the principal investigator is expected to publish the results of this research in a scientific journal. The International Committee of Medical Journal Editors (ICMJE) member journals have adopted a trials-registration policy as a condition for publication. This policy requires that all clinical trials be registered in a public trials registry such as ClinicalTrials.gov*, which is sponsored by the National Library of Medicine. Other biomedical journals are considering adopting similar policies.

It is the responsibility of DMID to register this trial in an acceptable registry. Any clinical trial starting enrollment after 01 July 2005 must be registered on or before patient enrollment. For trials that began enrollment prior to this date, the ICMJE member journals will require registration by 13 September 2005, before considering the results of the trial for publication.

The ICMJE defines a clinical trial as any research project that prospectively assigns human subjects to intervention or comparison groups to study the cause-and-effect relationship between a medical intervention and a health outcome. Studies designed for other purposes, such as to study pharmacokinetics or major toxicity (e.g., Phase I trials), would be exempt from this policy.

For trials in which DMID is not the IND/IDE sponsor, or there is no IND/IDE, and DMID does not provide data management services, it is the responsibility of the investigator to register the trial and post results in compliance with Public Law 110-85, the Food and Drug Administration Amendments Act of 2007 (FDAAA).

Refer to:

- Public Law 110-85, Section 801, Clinical Trial Databases

*Journal Citation: De Angelis C, Drazen JM, Frizelle FA, Haug C, Hoey J, Horton R, et al. Clinical trial registration: a statement from the International Committee of Medical Journal Editors. N Engl J Med. 2004; 351:1250-1.

19 LITERATURE REFERENCES

1. Taubenberger JK, Morens DM. 1918 Influenza: the mother of all pandemics. Emerg Infect Dis 2006; 12(1):15-22.

2. Oxford JS. Influenza A pandemics of the 20th century with special reference to 1918: virology, pathology and epidemiology. Rev Med Virol. 2000; 10:119-33.

3. Subbarao K, Klimov A, Katz J, Regnery H, Lim W, Hall H, et al. Characterization of an avian influenza A (H5N1) virus isolated from a child with a fatal respiratory illness. Science.1998; 279:393-6.

4. Peiris M, Yuen KY, Leung CW, Chan KH, Ip PL, Lai RW, et al. Human infection with influenza H9N2. Lancet. 1999; 354:916-7.

5. Novel Swine-Origin Influenza A (H1N1) Virus Investigation Team. Emergence of a Novel Swine-Origin Influenza A (H1N1) Virus in Humans; N Engl J Med 360; 25 p 2605-15.

6. New Ref: Centers for Disease Control. CDC estimates of 2009 H1N1 cases and related hospitalizations and deaths from April–October 17, 2009, by age group. Atlanta, GA: Emerging Infections Program: Centers for Disease Control and Prevention; 2009.

7. Reed C, Angulo FJ, Swerdlow DL, Lipsitch M, Meltzer MI, Jernigan D, Finelli L. Estimates of the prevalence of pandemic (H1N1) 2009, United States, April–July 2009. Emerg Infect Dis 2009; 15:2004–2007.

8. La Montagne JR, Noble GR, Quinnan GV, et al. Summary of clinical trials of inactivated influenza vaccine – 1978. Rev Infect Dis 1983; 5:723-36.

9. Galasso GJ, Tyeryar FJ Jr, La Montagne JR. Overview of clinical trials of influenza vaccines, 1976. J Infect Dis. 1977; 136 (Suppl):S425-8.

10. Couch RB, Kasel JA. Immunity to influenza in man. Annu Rev Microbiol 1983;37:529-49.

11. Ruben FL, Jackson GG. A new subunit influenza vaccine: Acceptability compared with standard vaccines and effect of dose on antigenicity. J Infect Dis 1972; 125:656-64.

12. Mostow SR, Dowdle WR, Schoenbaum SC, Coleman MT, Kaye HS. Inactivated vaccines. 1. Volunteer studies with very high doses of influenza vaccine purified by zonal ultracentrifugation. Postgrad Med J 1973; 49:152-58.

13. Ruben FL, Potter CW, Stuart-Harris CH. Humoral and secretory antibody responses to immunization with low and high dosage split influenza virus vaccine. Arch Virol 1975; 47:157-166.

14. Ennis FA, Mayner RE, Barry DW, et al. Correlation of laboratory studies with clinical responses to A/New Jersey influenza vaccines. J Infect Dis 1977; 236 (Dec. Suppl.): S397-406.

15. Matzkin H, Nili E. Accidental tenfold overdose of influenza vaccine: a clinical and serological study. Israel J Med Soc 1984; 20:411-15.

16. Palache AM, Beyer WE, Sprenger MJ, et al. Antibody response after influenza immunization with various vaccine doses: A double-blind, placebo-controlled, multi-centre, dose-response study in elderly nursing home residents and young volunteers. Vaccine 1993; 11:3-9.

17. Gross PA, Quinnan GV, Weksler ME, et al. Immunization of elderly people with high doses of influenza vaccine. J Am Geriatr Soc 1988; 36:209-12.

18. Remarque EJ, van Beek WC, Ligthart GJ, et al. Improvement of the immunoglobulin subclass response to influenza vaccine in elderly nursing home residents by the use of high-dose vaccines. Vaccine 1993; 11:649-54.

19. Keitel WA, Couch RB, Cate TR, et al. High doses of purified influenza A virus hemagglutinin significantly augment serum and nasal secretion antibody responses in healthy young adults. J Clin Microbiol 1994; 32:2468-73.

20. Keitel WA, Cate TR, Atmar RL, et al. Increasing doses of purified influenza virus hemagglutinin and subvirion vaccines enhance antibody responses in the elderly. Clin Diag Lab Immunol 1996; 3:507-10.

21. Treanor JJ, Wilkinson BE, Masseoud F, Hu-Primmer J, Battaglia R, O’Brien D, et al. Safety and immunogenicity of a recombinant hemagglutinin vaccine for H5N1influenza in humans. Vaccine. 2001; 19:1732-7.

22. Treanor JJ, Campbell JD, Zangwill KM, Rowe T, Wolff M. Safety and immunogenicity of an inactavted subvirion influenza A (H5N1) vaccine. N Engl J Med 2006; 354:1343-1351.

23. Nicholson KG, Colegate AE, Podda A, Stephenson I, Wood J, Ypma E, et al. Safety and antigenicity of non-adjuvanted and MF59-adjuvanted influenza A/Duck/Singapore/97 (H5N3) vaccine: a randomised trial of two potential vaccines against H5N1 influenza. Lancet. 2001; 357:1937-43.

24. Belshe RB, et al. Safety and Immunogenicity of Influenza A H5 Subunit Vaccines: Effect of Vaccine Schedule and Antigenic Variant. J Infec Dis. 2011; 203(5):666-673).

25. Atmar RL, Keitel WA. Adjuvants for pandemic influenza vaccines. In Compans RW, et al. (eds): Vaccines for Pandemic Influenza. Current Topics in Microbiology, vol. 333. Springer Verlag: Berlin, 2009, in press.

26. Bresson JL, Perronne C, Launay O, et al. Safety and immunogenicity of an inactivated split-virion influenza A/Vietnam/1194/2004 (H5N1) vaccine: phase I randomised trial. Lancet 2006; 367:1657-64.

27. Keitel WA, Campbell JD, Treanor JJ, et al. Safety and immunogenicity of an inactivated influenza A/H5N1 vaccine given with or without aluminum hydroxide to healthy adults: results of a phase I-II randomized clinical trial. J Infect Dis 2008; 198:1309-16.

28. Vogel FR, Caillet C, Kusters IC, and Haensler J. Emulsion-based adjuvants for influenza vaccines. Expert Rev. Vaccines 8 (4), 483-492 (2009).

29. Pellegrini M, Nicolay U, Lindert K, et al. MF59-adjuvanted versus non-adjuvanted influenza vaccines: integrated analysis from a large safety database. Vaccine 2009; 27(49):6959-6965.

30. Atmar,RL, Keitel WA, Patel SM, Katz JM, She D, El Sahly H, Pompey J, Cate TR, Couch RB. Safety and immunogenicity of nonadjuvanted and MF-59 adjuvanted influenza A/H9N2 vaccine preparations. Clin Infect Dis. 2006; Nov 1; 43(9): 1135-42.

31. Bernstein DI, Edwards KM, Dekker CL, Belshe R, Talbot HK, Graham IL, Noah DL, He F, Hill H. Effects of adjuvants on the safety and immunogenicity of an avian influenza H5N1 vaccine in adults. J Infect Dis. 2008 Mar 1; 197(5):667-75.

32. Khurana S, Chearwae W, Castellino F, Manishchewitz, King LR, Honorkiewicz A, Rock MT, Edwards KM et al. Vaccines with MF59 adjuvant expand the antibody repertoire to target protective sites of pandemic avian H5N1 influenza virus. Sci Transl Med. 2010 Jan 20;2(15):15ra5.

33. Galli G, Medini D, Borgogni E, Zedda L et al. Adjuvanted H5N1 vaccine induces early CD4+T-cell response that predicts the long-term persistence of protective antibody levels. Proc Natl Acad Sci USA. 2009 Mar 10; 106(10):3877-82.

34. Leroux-Roels I, Borkowski A, Vanwolleghem T, Drame M, Clement F, Hons E, et al. Antigen sparing and cross-reactive immunity with an adjuvanted rH5N1 prototype pandemic influenza vaccine: a randomised controlled trial. Lancet 2007; 370:580-9.

35. Seubert A, Monaci E, Pizza M, O'Hagan DT, Wack A. The adjuvants aluminum hydroxide and MF59 induce monocyte and granulocyte chemoattractants and enhance monocyte differentiation toward dendritic cells. J Immunol. 2008 Apr 15; 180(8):5402-12.

36. Didierlaurent AM, Morel S, Lockman L, Giannini AL et al. AS04, an aluminum salt- and TLR4 agonist-based adjuvant system, induced a transient localized innate immune response leading to enhanced adaptive immunity. J Immunol 2009 Nov 15; 183(10):6186-97.

37. Morel S et al. Adjuvant system AS03 containing alpha-tocopherol modulates innate immune response and leads to improved adaptive immunity. Vaccine 2011 Mar 16; 29(13):2461-73.

38. World Health Organization. Statement on narcolepsy and vaccination. (Accessed 14 April 2011 at http://www.who.int/vaccine_safety/topics/influenza/pandemic/h1n1_safety_assessing/narcolepsy_february2011/en/).

39. Nishino S et al. Hypocretin/orexin and narcolepsy: new basic and clinical insights. Acta Physiol 2010 Mar; 198(3):209-22.

40. Pulendran B, Li S, Nakaya H. Systems vaccinology. Immunity 2010 Oct 29;33(4):516-529.

41. Querec TD, Akondy RS et al. Systems biology approach predicts immunogenicity of the yellow fever vaccine in humans. Nat Immunol 2009 Jan;10(1):116-125.

42. Lominadze G, Powell DW, Luerman GC, Link AJ et al. Proteomic Analysis of Human Neutrophil Granules. Mol Cell Proteomics. 2005 Oct; 4(10):1503-21.

43. McAfee KJ, Duncan DT, Assink M, Link AJ. Analyzing proteomes and protein function using graphical comparative analysis of tandem mass spectrometry results. Mol Cell Proteomics. 2006 Aug;5(8):1497-513.

44. Clark, TW, Pareek, M. et. al. Trial of 2009 Influenza A (H1N1) Monovalent MF59-Adjuvanted Vaccine. NEJM 2009; Dec 17; 361: 2424-35.

45. Lasky T, Tarracciano GJ, Magder L, Koski CL, Ballesteros M, Nash D, Clark S, Haber P, Stolley PD, Schonberger LB and Chen RT. The Guillain-Barre syndrome and the 1992-1993 and 1993-1994 influenza vaccines. N. Engl. J. Med. 1998; 339:1797-1802.

46. Haber P, DeStefano F, Angulo FJ, Iskander J, Shadomy SV, Weintraub E and Chen RT. Guillain-Barre syndrome following influenza vaccination. JAMA 2004; 292:2478-2481.

47. ICH M3(R2) Guideline: Guidance on Nonclinical Safety Studies for the conduct of Human Clinical Trials and Marketing Authorization for Pharmaceuticals, 15 July 2008.

48. Ross, PL, Huang,YN, et. al. Multiplexed protein quantitation in *Saccharomyces cerevisiaie* using amine-reactive isobaric tagging reagents. Mol. Cell. Proteomics 2004; 3:1154-1169.

Appendix A: Table 1. Schedule of Procedures/Evaluations

**Study Visit Number**	**V00A**	**V00B**	**V01**	**V02**	**V03**	**V04**	**V05**	**V05a***	**V06**	**V07**	**V08***	**V09***	**Early Termination**
**Study Day**	**D-28 -7, +14d**[@]	**D-14 ± 14d**[@]	**D0**	**D1+1d**	**D3+1d**	**D7+2d**	**D28±2d**	**D30±1d**	**D35+2d**	**D56±2d**	**D208±14d**	**D393±14d**	
**Procedure**			**Dose 1**				**Dose 2**						
Obtain Informed Consent	X												
Review Eligibility Criteria	X		X[†¬]				X[†]						
Review Health Status	X		X[†¬]	X	X	X	X[†]		X	X			X
Vital Signs (Oral Temp, Pulse, BP)	X		X[%†]										
Oral Temp			X[†]				X[†]						
Height and Weight	X		X[$]										
Medical History	X		X[†¬]										
Concomitant Medications	X		X[†¬]	X	X	X	X[†]	X	X	X	X[&]	X[&]	X[&]
Complete Physical Examination (without genital and rectal exam)	X												
Targeted Physical Examination			{X}[†]	{X}	{X}	{X}	{X}[†]		{X}	{X}			{X}
Lymph Node Assessment	X		X[†¬]	X	X	X	X[†]		X	X			X
Serum or Urine Pregnancy Test	X		X[^]				X[^]						
Urine collection	X	X	X[†]	X	X	X	X[†]						
Venous Blood Sample Collection for HAI and Nt Antibody Titers			X[†]	X	X	X	X[†]			X			X (if after Day 28 but prior to Day 56)
Venous Blood Sample Collection for Systems Biology Studies	X	X	X[†]	X	X	X	X[†]						X (if prior to Day 28)

Study Visit Number	V00A	V00B	V01	V02	V03	V04	V05	V05a*	V06	V07	V08*	V09*	Early Termination
Study Day	D-28 -7, +14d[@]	D-14 ± 14d[@]	D0	D1+1d	D3+1d	D7+2d	D28±2d	D30±1d	D35+2d	D56±2d	D208±14d	D393±14d	
**Procedure**			**Dose 1**				**Dose 2**						
Randomization			X[†]										
Vaccination			X				X						
Examine Vaccination Site			X	X	X	X	X		X				
20-minute Evaluation Following Vaccination			X				X						
Distribute Memory Aid and Study Related Materials			X				X						
Review Memory Aid				X	X	X	X	X	X				
AE/SAE Assessment			X	X	X	X	X	X	X	X	X[#]	X[#]	X[#]

* Telephone call assessment.

[@] Study Visits V00A, V00B, and V01 must be separated by at least six days.

{} Targeted physical examination if indicated based on review of interim health status.

[†] Prior to vaccination.

% Oral temperature only will be obtained at Day 0 if all vital signs collected at screening.

$ Height and weight will be obtained at Day 0 if not collected at screening.

^ Must be performed within 24 hours prior to vaccination and results must be negative and known prior to vaccination.

¬ Review/confirm information or activity in subjects previously consented and screened.

& Solicitation for receipt of non-study influenza vaccines only after Day 56.

New-onset chronic medical conditions and SAEs only after Day 56.
