# Supplementary material for: Cell-Based Systems Biology Analysis of Human AS03-Adjuvanted H5N1 Avian Influenza Vaccine Responses: A Phase I Randomized Controlled Trial
Source: PLoS One. 2017 Jan 18;12(1):e0167488. doi: 10.1371/journal.pone.0167488 (PMC5242433; doi:10.1371/journal.pone.0167488)
Supplement: S2 Text — (PDF) [file pone.0167488.s012.pdf]

S2 Text Manuscript Appendix "Cell-based Systems Biology Analysis of Human AS03-adjuvanted H5N1 Avian Influenza Vaccine Responses: A Phase I Randomized Controlled Trial"
Version 1.0

November 1, 2016

Table of Contents

1 Introduction 15

2 Supplemental methods 16

2.1 Selection of study population . . . 16
2.1.1 Inclusion criteria . . . 16
2.1.2 Exclusion criteria . . . 17
2.2 Study visit windows . . . 18
2.3 Reactogenicity definitions . . . 19
2.4 Analysis population . . . 19
2.5 Cytokine/chemokine cytometric bead array methods . . . 19
2.6 RNA-Seq methods . . . 20
2.6.1 RNA-Seq experiment . . . 20
2.6.2 RNA-Seq data preprocessing . . . 20
2.6.3 RNA-Seq statistical analysis . . . 21
2.7 Flow cytometric immune cell phenotyping and activation methods . . . 25
2.7.1 Flow cytometric immune cell phenotyping and activation data processing . . . 25
2.7.2 Flow cytometric immune cell activation outlier detection and normalization . . . 25
2.8 Software . . . 26

3 Supplemental results 26

3.1 Safety and immunogenicity results . . . 27
3.1.1 Local and systemic reactogenicity . . . 27
3.1.2 Adverse events . . . 27

3.2 Cytokine/chemokine cytometric bead array results 27
3.3 RNA-Seq results 27
3.3.1 Reference alignment statistics 27
3.3.2 Impact of TMM normalization 28
3.3.3 Global gene expression patterns, confounding effects, and outlying samples . . . 29
3.3.4 Multiple time points to measure baseline expression 30
3.3.5 Adjustment of global GC content bias 31
3.3.6 Response time trends of differential genes and gene clusters 31
3.3.7 Gene set enrichment results 32
3.3.8 Protein-protein interaction networks 32
3.3.9 Gene responses correlated with changes in serum cytokine/chemokine, or antibody concentration 32
3.3.10 Gene responses that differentiate between seroprotection status 33
3.4 Flow cytometric immune cell phenotyping and activation results 33

List of Figures

Figure A1 Consort diagram. 35
Figure A2 Local and systemic reactogenicity by severity and study arm. 36
Figure A3 Eotaxin-1 (CCL11) Time Trend Plots 37
Figure A4 Interferon Gamma (IFN-γ) Time Trend Plots 38
Figure A5 Interleukin-1 Alpha (IL-1α) Time Trend Plots 39
Figure A6 Interleukin-1 Beta (IL-1β) Time Trend Plots 40
Figure A7 Interleukin-6 (IL-6) Time Trend Plots 41
Figure A8 Interleukin-8 (IL-8) Time Trend Plots 42
Figure A9 Interleukin-10 (IL-10) Time Trend Plots 43
Figure A10 Interleukin-12 p70 (IL-12p70) Time Trend Plots 44
Figure A11 Lymphotoxin-alpha (LT-α) Time Trend Plots 45
Figure A12 Interferon Gamma-Induced Protein 10 (IP-10) Time Trend Plots 46
Figure A13 Monocyte Chemoattractant Protein-1 (MCP-1) Time Trend Plots 47
Figure A14 Macrophage Inflammatory Protein (MIP-1α) Time Trend Plots 48
Figure A15 Chemokine (C-C motif) Ligand 5 (RANTES) Time Trend Plots 49
Figure A16 Tumor Necrosis Factors (TNF) Time Trend Plots 50
Figure A17 Boxplots of technical variables by cell type (RNA-Seq) 51
Figure A18 Boxplots of gene model variables by cell type (RNA-Seq) 52
Figure A19 Starplots of human genome reference alignment statistics (RNA-Seq, B-cells) 53
Figure A20 Starplots of human genome reference alignment statistics (RNA-Seq, Dendritic cells) . . 54
Figure A21 Starplots of human genome reference alignment statistics (RNA-Seq, Monocytes) 55
Figure A22 Starplots of human genome reference alignment statistics (RNA-Seq, Neutrophils) 56
Figure A23 Starplots of human genome reference alignment statistics (RNA-Seq, NK-cells) 57
Figure A24 Starplots of human genome reference alignment statistics (RNA-Seq, T-cells) 58
Figure A25 Boxplots of log_2 counts per million (RNA-Seq, before TMM normalization). 59
Figure A26 Boxplots of log_2 counts per million (RNA-Seq after TMM normalization). 60
Figure A27 ECDF plots of log_2 counts per million (RNA-Seq, before and after TMM normalization). . 61
Figure A28 PCA biplots (RNA-Seq, n=839) 62
Figure A29 Non-metric multidimensional scaling biplots (RNA-Seq, n=839) 63
Figure A30 Hierarchical clustering plots (RNA-Seq, n=839). 64
Figure A31 Heatmap plot of log_2 cpm (RNA-Seq, top-ranking 136 genes, n=839). 65
Figure A32 PCA and non-metric multidimensional scaling biplots (RNA-Seq, B-cells and Dendritic cells, n=139) 66
Figure A33 PCA and non-metric multidimensional scaling biplots (RNA-Seq, Monocytes and Neutrophils, n=139) 67
Figure A34 PCA and non-metric multidimensional scaling biplots (RNA-Seq, NK-cells and T-cells, n=140) 68
Figure A35 Boxplots of baseline correlation differences by cell type and subject (RNA-Seq, n=20). . . 69
Figure A36 Variance component plots (RNA-Seq) 70
Figure A37 Correlation with mean %GC content (RNA-Seq). 71
Figure A38 Example genes with mean %GC content association (RNA-Seq). 72

Figure A39 Baseline PCA biplots (RNA-Seq, n=360) . . . 73
Figure A40 Baseline variance component plots (RNA-Seq) . . . 74
Figure A41 Impact of GC on fold change (RNA-Seq, B-cells). . . . 75
Figure A42 Impact of GC on fold change (RNA-Seq, Dendritic cells). . . . 76
Figure A43 Impact of GC on fold change (RNA-Seq, Monocytes). . . . 77
Figure A44 Impact of GC on fold change (RNA-Seq, Neutrophils). . . . 78
Figure A45 Impact of GC on fold change (RNA-Seq, NK-cells). . . . 79
Figure A46 Impact of GC on fold change (RNA-Seq, T-cells). . . . 80
Figure A47 Model QC summary plots (RNA-Seq, B-cells). . . . 82
Figure A48 Model QC summary plots (RNA-Seq, Dendritic cells). . . . 83
Figure A49 Model QC summary plots (RNA-Seq, Monocytes). . . . 84
Figure A50 Model QC summary plots (RNA-Seq, Neutrophils). . . . 85
Figure A51 Model QC summary plots (RNA-Seq, NK-cells). . . . 86
Figure A52 Model QC summary plots (RNA-Seq, T-cells). . . . 87
Figure A53 Venn diagrams of significant gene counts by day and cell type (RNA-Seq, before GC adjustment). . . . 88
Figure A54 Venn diagrams of significant gene counts by day and cell type (RNA-Seq, after GC adjustment). . . . 89
Figure A55 Heatmap of baseline log fold change (RNA-Seq, B-cells, Day 1) . . . 90
Figure A56 Heatmap of baseline log fold change (RNA-Seq, B-cells, Day 3) . . . 91
Figure A57 Heatmap of baseline log fold change (RNA-Seq, B-cells, Day 7) . . . 92
Figure A58 Heatmap of baseline log fold change (RNA-Seq, B-cells, Day 28) . . . 93
Figure A59 Heatmap of baseline log fold change (RNA-Seq, Dendritic cells, Day 1) . . . 94
Figure A60 Heatmap of baseline log fold change (RNA-Seq, Dendritic cells, Day 3) . . . 95
Figure A61 Heatmap of baseline log fold change (RNA-Seq, Dendritic cells, Day 7) . . . 96
Figure A62 Heatmap of baseline log fold change (RNA-Seq, Dendritic cells, Day 28) . . . 97
Figure A63 Heatmap of baseline log fold change (RNA-Seq, Monocytes, Day 1) . . . 98
Figure A64 Heatmap of baseline log fold change (RNA-Seq, Monocytes, Day 3) . . . 99
Figure A65 Heatmap of baseline log fold change (RNA-Seq, Monocytes, Day 7) . . . 100
Figure A66 Heatmap of baseline log fold change (RNA-Seq, Monocytes, Day 28) . . . 101
Figure A67 Heatmap of baseline log fold change (RNA-Seq, Neutrophils, Day 1) . . . 102
Figure A68 Heatmap of baseline log fold change (RNA-Seq, Neutrophils, Day 3) . . . 103
Figure A69 Heatmap of baseline log fold change (RNA-Seq, Neutrophils, Day 7) . . . 104
Figure A70 Heatmap of baseline log fold change (RNA-Seq, Neutrophils, Day 28) . . . 105
Figure A71 Heatmap of baseline log fold change (RNA-Seq, NK-cells, Day 1) . . . 106
Figure A72 Heatmap of baseline log fold change (RNA-Seq, NK-cells, Day 3) . . . 107
Figure A73 Heatmap of baseline log fold change (RNA-Seq, NK-cells, Day 7) . . . 108
Figure A74 Heatmap of baseline log fold change (RNA-Seq, NK-cells, Day 28) . . . 109
Figure A75 Heatmap of baseline log fold change (RNA-Seq, T-cells, Day 1) . . . 110
Figure A76 Heatmap of baseline log fold change (RNA-Seq, T-cells, Day 3) . . . 111
Figure A77 Heatmap of baseline log fold change (RNA-Seq, T-cells, Day 7) . . . 112
Figure A78 Heatmap of baseline log fold change (RNA-Seq, T-cells, Day 28) . . . 113

Figure A79 Heatmaps of GC adjusted log fold change (RNA-Seq, B-cells) 114
Figure A80 Heatmaps of GC adjusted log fold change (RNA-Seq, Dendritic cells) 115
Figure A81 Heatmaps of GC adjusted log fold change (RNA-Seq, Monocytes) 116
Figure A82 Heatmaps of GC adjusted log fold change (RNA-Seq, Neutrophils) 117
Figure A83 Heatmaps of GC adjusted log fold change (RNA-Seq, NK-cells) 118
Figure A84 Heatmaps of GC adjusted log fold change (RNA-Seq, T-cells) 119
Figure A85 Gene cluster dendrogram with bootstrap probabilities (RNA-Seq, B-cells, Day 1) 120
Figure A86 Gene cluster dendrogram with bootstrap probabilities (RNA-Seq, B-cells, Day 1-28) . . . 120
Figure A87 Gene cluster dendrogram with bootstrap probabilities (RNA-Seq, Dendritic cells, Day 1) . 121
Figure A88 Gene cluster dendrogram with bootstrap probabilities (RNA-Seq, Dendritic cells, Day 3) . 121
Figure A89 Gene cluster dendrogram with bootstrap probabilities (RNA-Seq, Dendritic cells, Day 1-28) 121
Figure A90 Gene cluster dendrogram with bootstrap probabilities (RNA-Seq, Monocytes, Day 1) . . . 122
Figure A91 Gene cluster dendrogram with bootstrap probabilities (RNA-Seq, Monocytes, Day 1-28) . 122
Figure A92 Gene cluster dendrogram with bootstrap probabilities (RNA-Seq, Neutrophils, Day 1) . . 122
Figure A93 Gene cluster dendrogram with bootstrap probabilities (RNA-Seq, Neutrophils, Day 3) . . 123
Figure A94 Gene cluster dendrogram with bootstrap probabilities (RNA-Seq, Neutrophils, Day 1-28) . 123
Figure A95 Gene cluster dendrogram with bootstrap probabilities (RNA-Seq, NK-cells, Day 1) 123
Figure A96 Gene cluster dendrogram with bootstrap probabilities (RNA-Seq, NK-cells, Day 3) 124
Figure A97 Gene cluster dendrogram with bootstrap probabilities (RNA-Seq, NK-cells, Day 28) . . . 124
Figure A98 Gene cluster dendrogram with bootstrap probabilities (RNA-Seq, NK-cells, Day 1-28) . . 124
Figure A99 Gene cluster dendrogram with bootstrap probabilities (RNA-Seq, T-cells, Day 1) 125
Figure A100 Gene cluster dendrogram with bootstrap probabilities (RNA-Seq, T-cells, Day 1-28) . . . 125
Figure A101 Gene cluster time trends of baseline log fold change by vaccine group (RNA-Seq, B-cells, Dendritic cells, and Monocytes). 126
Figure A102 Gene cluster time trends of baseline log fold change by vaccine group (RNA-Seq, Monocytes and Neutrophils). 127
Figure A103 Gene cluster time trends of baseline log fold change by vaccine group (RNA-Seq, Neutrophils continued). 128
Figure A104 Gene cluster time trends of baseline log fold change by vaccine group (RNA-Seq, Neutrophils continued). 129
Figure A105 Gene cluster time trends of baseline log fold change by vaccine group (RNA-Seq, NK-cells and T-cells). 130
Figure A106 Heatmap of baseline log fold changes for known antigen presentation genes by vaccine group (RNA-Seq, Dendritic cells). 131
Figure A107 Heatmap of baseline log fold changes for known antigen presentation genes by vaccine group (RNA-Seq, Monocytes). 132
Figure A108 Heatmap of baseline log fold changes for known antigen presentation genes by vaccine group (RNA-Seq, Neutrophils). 133
Figure A109 Heatmap of baseline log fold changes for known antigen presentation genes by vaccine group (RNA-Seq, NK-cells). 134
Figure A110 Time trends of baseline log fold change for MHC I genes by cell type and vaccine group (RNA-Seq). 135

Figure A111 Time trends of baseline log fold change for MHC II genes by cell type and vaccine group (RNA-Seq). 136
Figure A112 Heatmap of enriched KEGG Pathways (RNA-Seq) 137
Figure A113 Heatmap of enriched MSigDB GO Biological Processes (RNA-Seq) 138
Figure A114 Heatmap of enriched MSigDB GO Cellular Components (RNA-Seq) 139
Figure A115 Heatmap of enriched MSigDB Reactome Pathways (RNA-Seq) 140
Figure A116 Heatmap of enriched MSigDB Chemical/Genetic Perturbation gene sets (RNA-Seq) . . . 141
Figure A117 Heatmap of enriched MSigDB Immunologic Signature gene sets (RNA-Seq) 142
Figure A118 Pathway map - Influenza A (RNA-Seq, Dendritic cells, Day 1) 143
Figure A119 Pathway map - Influenza A (RNA-Seq, Monocytes, Day 1) 144
Figure A120 Pathway map - Influenza A (RNA-Seq, Neutrophils, Day 1) 145
Figure A121 Pathway map - Influenza A (RNA-Seq, Neutrophils, Day 3) 146
Figure A122 Pathway map - Influenza A (RNA-Seq, NK-cells, Day 28) 147
Figure A123 Pathway map - Influenza A (RNA-Seq, T-cells, Day 1) 148
Figure A124 Pathway map - Antigen processing and presentation (RNA-Seq, Dendritic cells, Day 1) . 149
Figure A125 Pathway map - Antigen processing and presentation (RNA-Seq, Monocytes, Day 1) . . . 150
Figure A126 Pathway map - Antigen processing and presentation (RNA-Seq, Neutrophils, Day 1) . . . 151
Figure A127 Pathway map - Antigen processing and presentation (RNA-Seq, NK-cells, Day 28) 152
Figure A128 Pathway map - Proteasome (RNA-Seq, Neutrophils, Day 1) 153
Figure A129 Pathway map - Cell cycle (RNA-Seq, NK-cells, Day 3) 154
Figure A130 Pathway map - Phagosome (RNA-Seq, Monocytes, Day 1) 155
Figure A131 Pathway map - Phagosome (RNA-Seq, Neutrophils, Day 1) 156
Figure A132 Pathway map - Phagosome (RNA-Seq, NK-cells, Day 28) 157
Figure A133 Pathway map - Cell adhesion molecules (CAMs) (RNA-Seq, Neutrophils, Day 1) 158
Figure A134 Pathway map - Cell adhesion molecules (CAMs) (RNA-Seq, NK-cells, Day 28) 159
Figure A135 Pathway map - Complement and coagulation cascades (RNA-Seq, Dendritic cells, Day 1) 160
Figure A136 Pathway map - NOD-like receptor signaling pathway (RNA-Seq, Neutrophils, Day 1) . . . 161
Figure A137 Pathway map - RIG-I-like receptor signaling pathway (RNA-Seq, Neutrophils, Day 1) . . . 162
Figure A138 Protein-protein interaction network based on significant genes (RNA-Seq, B-cells, Day 1) 163
Figure A139 Protein-protein interaction network based on significant genes (RNA-Seq, Dendritic cells, Day 1) 164
Figure A140 Protein-protein interaction network based on significant genes (RNA-Seq, Monocytes, Day 1) 165
Figure A141 Protein-protein interaction network based on significant genes (RNA-Seq, Neutrophils, Day 1) 166
Figure A142 Protein-protein interaction network based on significant genes (RNA-Seq, Neutrophils, Day 3) 167
Figure A143 Protein-protein interaction network based on significant genes (RNA-Seq, NK-cells, Day 1) 168
Figure A144 Protein-protein interaction network based on significant genes (RNA-Seq, NK-cells, Day 3) 169
Figure A145 Protein-protein interaction network based on significant genes (RNA-Seq, NK-cells, Day 28) 170
Figure A146 Protein-protein interaction network based on significant genes (RNA-Seq, T-cells, Day 1) 171

Figure A147 Protein-protein interaction network based on significant genes (RNA-Seq, Dendritic cells, Monocytes, and Neutrophils, Day 1) . . . 172
Figure A148 Canonical correlation plots (RNA-Seq, B-cells, Day 1) . . . 173
Figure A149 Canonical correlation plots (RNA-Seq, B-cells, Day 3) . . . 173
Figure A150 Canonical correlation plots (RNA-Seq, B-cells, Day 7) . . . 174
Figure A151 Canonical correlation plots (RNA-Seq, B-cells, Day 28) . . . 174
Figure A152 Canonical correlation plots (RNA-Seq, Dendritic cells, Day 1) . . . 175
Figure A153 Canonical correlation plots (RNA-Seq, Dendritic cells, Day 3) . . . 176
Figure A154 Canonical correlation plots (RNA-Seq, Dendritic cells, Day 7) . . . 177
Figure A155 Canonical correlation plots (RNA-Seq, Monocytes, Day 1) . . . 177
Figure A156 Canonical correlation plots (RNA-Seq, Monocytes, Day 3) . . . 178
Figure A157 Canonical correlation plots (RNA-Seq, Monocytes, Day 28) . . . 178
Figure A158 Canonical correlation plots (RNA-Seq, Neutrophils, Day 1) . . . 179
Figure A159 Canonical correlation plots (RNA-Seq, Neutrophils, Day 7) . . . 179
Figure A160 Canonical correlation plots (RNA-Seq, Neutrophils, Day 28) . . . 180
Figure A161 Canonical correlation plots (RNA-Seq, NK-cells, Day 1) . . . 180
Figure A162 Canonical correlation plots (RNA-Seq, NK-cells, Day 3) . . . 181
Figure A163 Canonical correlation plots (RNA-Seq, NK-cells, Day 7) . . . 181
Figure A164 Canonical correlation plots (RNA-Seq, NK-cells, Day 28) . . . 182
Figure A165 Canonical correlation plots (RNA-Seq, T-cells, Day 1) . . . 182
Figure A166 Canonical correlation plots (RNA-Seq, T-cells, Day 3) . . . 183
Figure A167 Canonical correlation plots (RNA-Seq, T-cells, Day 7) . . . 183
Figure A168 Canonical correlation plots (RNA-Seq, T-cells, Day 28) . . . 184
Figure A169 Canonical correlation plots (RNA-Seq, B-cells, Day 1, SV-AS03 vs. SV-PBS) . . . 184
Figure A170 Canonical correlation plots (RNA-Seq, B-cells, Day 3, SV-AS03 vs. SV-PBS) . . . 185
Figure A171 Canonical correlation plots (RNA-Seq, Dendritic cells, Day 3, SV-AS03 vs. SV-PBS) . . . 186
Figure A172 Canonical correlation plots (RNA-Seq, Monocytes, Day 3, SV-AS03 vs. SV-PBS) . . . 187
Figure A173 Canonical correlation plots (RNA-Seq, Monocytes, Day 28, SV-AS03 vs. SV-PBS) . . . 188
Figure A174 Canonical correlation plots (RNA-Seq, Neutrophils, Day 1, SV-AS03 vs. SV-PBS) . . . 189
Figure A175 Canonical correlation plots (RNA-Seq, Neutrophils, Day 28, SV-AS03 vs. SV-PBS) . . . 190
Figure A176 Canonical correlation plots (RNA-Seq, NK-cells, Day 3, SV-AS03 vs. SV-PBS) . . . 191
Figure A177 Canonical correlation plots (RNA-Seq, T-cells, Day 3, SV-AS03 vs. SV-PBS) . . . 192
Figure A178 Boxplots of percent cell activation by cell type (FACS, CD69) . . . 193
Figure A179 Boxplots of percent cell activation by cell type (FACS, CD86) . . . 194
Figure A180 Boxplots of percent cell activation by cell type (FACS, CD134) . . . 195
Figure A181 Non-metric multidimensional scaling biplots for each cell activation marker (FACS). . . . 196
Figure A182 Scatterplots of percent cell activation by detector setting (FACS, CD69, T-cells, neutrophils, monocytes, colored by vaccine group) . . . 198
Figure A183 Scatterplots of percent cell activation by detector setting (FACS, CD69, B-cells, dendritic cells, NK-cells, colored by vaccine group) . . . 199
Figure A184 Scatterplots of percent cell activation by detector setting (FACS, CD69, T-cells, neutrophils, monocytes, colored by study visit day) . . . 200

Figure A185 Scatterplots of percent cell activation by detector setting (FACS, CD69, B-cells, dendritic cells, NK-cells, colored by study visit day) . . . 201
Figure A186 Scatterplots of percent cell activation by detector setting (FACS, CD86, T-cells, neutrophils, monocytes, colored by vaccine group) . . . 202
Figure A187 Scatterplots of percent cell activation by detector setting (FACS, CD86, B-cells, dendritic cells, NK-cells, colored by vaccine group) . . . 203
Figure A188 Scatterplots of percent cell activation by detector setting (FACS, CD86, T-cells, neutrophils, monocytes, colored by study visit day) . . . 204
Figure A189 Scatterplots of percent cell activation by detector setting (FACS, CD86, B-cells, dendritic cells, NK-cells, colored by study visit day) . . . 205
Figure A190 Scatterplots of percent cell activation by detector setting (FACS, CD134, T-cells, neutrophils, monocytes, colored by vaccine group) . . . 206
Figure A191 Scatterplots of percent cell activation by detector setting (FACS, CD134, B-cells, dendritic cells, NK-cells, colored by vaccine group) . . . 207
Figure A192 Scatterplots of percent cell activation by detector setting (FACS, CD134, T-cells, neutrophils, monocytes, colored by study visit day) . . . 208
Figure A193 Scatterplots of percent cell activation by detector setting (FACS, CD134, B-cells, dendritic cells, NK-cells, colored by study visit day) . . . 209
Figure A194 Median normalized cell activation plots for CD69 (FACS, B-cells) . . . 210
Figure A195 Median normalized cell activation plots for CD86 (FACS, B-cells) . . . 211
Figure A196 Median normalized cell activation plots for CD134 (FACS, B-cells) . . . 212
Figure A197 Median normalized cell activation plots for CD69 (FACS, Dendritic Cells) . . . 213
Figure A198 Median normalized cell activation plots for CD86 (FACS, Dendritic Cells) . . . 214
Figure A199 Median normalized cell activation plots for CD134 (FACS, Dendritic Cells) . . . 215
Figure A200 Median normalized cell activation plots for CD69 (FACS, Monocytes) . . . 216
Figure A201 Median normalized cell activation plots for CD86 (FACS, Monocytes) . . . 217
Figure A202 Median normalized cell activation plots for CD134 (FACS, Monocytes) . . . 218
Figure A203 Median normalized cell activation plots for CD69 (FACS, Neutrophils) . . . 219
Figure A204 Median normalized cell activation plots for CD86 (FACS, Neutrophils) . . . 220
Figure A205 Median normalized cell activation plots for CD134 (FACS, Neutrophils) . . . 221
Figure A206 Median normalized cell activation plots for CD69 (FACS, NK-Cells) . . . 222
Figure A207 Median normalized cell activation plots for CD86 (FACS, NK-Cells) . . . 223
Figure A208 Median normalized cell activation plots for CD134 (FACS, NK-Cells) . . . 224
Figure A209 Median normalized cell activation plots for CD69 (FACS, T-Cells) . . . 225
Figure A210 Median normalized cell activation plots for CD86 (FACS, T-Cells) . . . 226
Figure A211 Median normalized cell activation plots for CD134 (FACS, T-Cells) . . . 227
Figure A212 Spider plots of median fold change in the percentage of live cells for each immune cell type. 228
Figure A213 Spider plots of median fold change in the number of live cells per mL for each immune cell type. . . . 228
Figure A214 Morel et al. (2011) SV-AS03-responsive genes in mouse (RNA-Seq). . . . 229
Figure A215 Mosca et al. (2008) MF59, CpG, MF59+CpG, and Alum-responsive genes in mouse (RNA-Seq). . . . 230

Figure A216 Time trends of baseline log fold change for CXCR3 by cell type and vaccine group (RNA-Seq). 231

List of Tables

Table A1 Demographic data by study group . . . 231
Table A2 Maximum severity of solicited adverse events by vaccination (SV-AS03) . . . 232
Table A3 Maximum severity of solicited adverse events by vaccination (SV-PBS) . . . 233
Table A4 Intend-to-treat population. Summary of hemagglutination inhibition titer/responses against A/Indonesia/05/2005 by study arm and visit (Immunogenicity). . . . 234
Table A5 Per-protocol population. Summary of hemagglutination inhibition titer/responses against A/Indonesia/05/2005 by study arm and visit (Immunogenicity). . . . 235
Table A6 Intend-to-treat population. Summary of neutralizing antibody titer/response against A/Indonesia/05/2005 by study arm and visit (Immunogenicity). . . . 236
Table A7 Per-protocol population. Summary of neutralizing antibody titer/response against A/Indonesia/05/2005 by study arm and visit (Immunogenicity). . . . 237
Table A8 Cytokine and chemokine limits of detection. . . . 238
Table A9 Number of excluded genes by category/chromosome (RNA-Seq). . . . 238
Table A10 Number of genes that passed the low expression cut off (RNA-Seq). . . . 238
Table A11 Summary statistics (RNA-Seq, technical variables) . . . 239
Table A12 Summary statistics (RNA-Seq, gene model variables) . . . 239
Table A13 Spearman correlation with technical variables (RNA-Seq, original variables). . . . 240
Table A14 Spearman correlation with technical variables (RNA-Seq, standardized variables). . . . 240
Table A15 Spearman correlation with gene model variables (RNA-Seq, original variables). . . . 241
Table A16 Spearman correlation with gene model variables (RNA-Seq, standardized variables). . . . 241
Table A17 Sources of variation (n=839, standardized variables, PC1) . . . 241
Table A18 Sources of variation (n=839, standardized variables, PC2) . . . 241
Table A19 Outlying observations (RNA-Seq). . . . 242
Table A20 Correlation between TMM normalization factors (RNA-Seq, all versus per-cell). . . . 242
Table A21 Spearman correlation with technical variables (RNA-Seq, B-cells). . . . 242
Table A22 Spearman correlation with technical variables (RNA-Seq, Dendritic cells). . . . 243
Table A23 Spearman correlation with technical variables (RNA-Seq, Monocytes). . . . 243
Table A24 Spearman correlation with technical variables (RNA-Seq, Neutrophils). . . . 243
Table A25 Spearman correlation with technical variables (RNA-Seq, NK-cells). . . . 244
Table A26 Spearman correlation with technical variables (RNA-Seq, T-cells). . . . 244
Table A27 Spearman correlation with gene model variables (RNA-Seq, B-cells). . . . 245
Table A28 Spearman correlation with gene model variables (RNA-Seq, Dendritic cells). . . . 245
Table A29 Spearman correlation with gene model variables (RNA-Seq, Monocytes). . . . 246
Table A30 Spearman correlation with gene model variables (RNA-Seq, Neutrophils). . . . 246
Table A31 Spearman correlation with gene model variables (RNA-Seq, NK-cells). . . . 247
Table A32 Spearman correlation with gene model variables (RNA-Seq, T-cells). . . . 247
Table A33 Baseline sources of variation (RNA-Seq, All Cells, PC1) . . . 248
Table A34 Baseline sources of variation (RNA-Seq, All Cells, PC2) . . . 248
Table A35 Baseline sources of variation (RNA-Seq, B-cells, PC1) . . . 248
Table A36 Baseline sources of variation (RNA-Seq, B-cells, PC2) . . . 248
Table A37 Baseline sources of variation (RNA-Seq, Dendritic cells, PC1) . . . 248

Table A38 Baseline sources of variation (RNA-Seq, Dendritic cells, PC2) 248
Table A39 Baseline sources of variation (RNA-Seq, Monocytes, PC1) 248
Table A40 Baseline sources of variation (RNA-Seq, Monocytes, PC2) 248
Table A41 Baseline sources of variation (RNA-Seq, Neutrophils, PC1) 248
Table A42 Baseline sources of variation (RNA-Seq, Neutrophils, PC2) 249
Table A43 Baseline sources of variation (RNA-Seq, NK-cells, PC1) 249
Table A44 Baseline sources of variation (RNA-Seq, NK-cells, PC2) 249
Table A45 Baseline sources of variation (RNA-Seq, T-cells, PC1) 249
Table A46 Baseline sources of variation (RNA-Seq, T-cells, PC2) 249
Table A47 Genes with high baseline variability (RNA-Seq, B-cells) 250
Table A48 Genes with high baseline variability (RNA-Seq, Dendritic cells) 252
Table A49 Genes with high baseline variability (RNA-Seq, Monocytes) 253
Table A50 Genes with high baseline variability (RNA-Seq, Neutrophils) 253
Table A51 Genes with high baseline variability (RNA-Seq, NK-cells) 254
Table A52 Genes with high baseline variability (RNA-Seq, T-cells) 255
Table A53 Impact of using multiple baseline measures on gene response (RNA-Seq). 255
Table A54 Statistically significant genes by gene type, cell type, and day (RNA-Seq). 256
Table A55 Statistically significant genes by gene type and cell type, (RNA-Seq). 256
Table A56 Overlap of significantly differentially expressed genes between cell types (RNA-Seq, Day 1) 257
Table A57 Genes significantly differentially expressed in all six cell types (RNA-Seq, Day 1) 257
Table A58 Significant gene clusters (RNA-Seq, B-cells, Day 1) 257
Table A59 Significant gene clusters (RNA-Seq, B-cells, Day 1-28) 258
Table A60 Significant gene clusters (RNA-Seq, Dendritic cells, Day 1) 259
Table A61 Significant gene clusters (RNA-Seq, Dendritic cells, Day 1-28) 259
Table A62 Significant gene clusters (RNA-Seq, Monocytes, Day 1) 261
Table A63 Significant gene clusters (RNA-Seq, Monocytes, Day 1-28) 263
Table A64 Significant gene clusters (RNA-Seq, Neutrophils, Day 1) 266
Table A65 Significant gene clusters (RNA-Seq, Neutrophils, Day 3) 268
Table A66 Significant gene clusters (RNA-Seq, Neutrophils, Day 1-28) 269
Table A67 Significant gene clusters (RNA-Seq, NK-cells, Day 1) 270
Table A68 Significant gene clusters (RNA-Seq, NK-cells, Day 3) 271
Table A69 Significant gene clusters (RNA-Seq, NK-cells, Day 28) 272
Table A70 Significant gene clusters (RNA-Seq, NK-cells, Day 1-28) 272
Table A71 Significant gene clusters (RNA-Seq, T-cells, Day 1) 273
Table A72 Significant gene clusters (RNA-Seq, T-cells, Day 1-28) 274
Table A73 Overview of gene sets used for the enrichment analysis (RNA-Seq). 275
Table A74 Overview of differential genes that mapped to any gene set by category (RNA-Seq). 275
Table A75 Significantly enriched MSigDB Chemical/Genetic Perturbation Sets (RNA-Seq, B-cells, Day 1) 277
Table A76 Significantly enriched KEGG Pathways (RNA-Seq, Dendritic cells, Day 1) 277
Table A77 Significantly enriched MSigDB GO Biological Processes (RNA-Seq, Dendritic cells, Day 1) 277
Table A78 Significantly enriched MSigDB GO Cellular Components (RNA-Seq, Dendritic cells, Day 1) 278

Table A79 Significantly enriched MSigDB BioCarta Pathways (RNA-Seq, Dendritic cells, Day 1) . . . 278
Table A80 Significantly enriched MSigDB Chemical/Genetic Perturbation Sets (RNA-Seq, Dendritic cells, Day 1) . . . 285
Table A81 Significantly enriched MSigDB Chemical/Genetic Perturbation Sets (RNA-Seq, Dendritic cells, Day 3) . . . 285
Table A82 Significantly enriched KEGG Pathways (RNA-Seq, Monocytes, Day 1) . . . 286
Table A83 Significantly enriched MSigDB GO Biological Processes (RNA-Seq, Monocytes, Day 1) . . 287
Table A84 Significantly enriched MSigDB GO Cellular Components (RNA-Seq, Monocytes, Day 1) . . 287
Table A85 Significantly enriched MSigDB GO Molecular Functions (RNA-Seq, Monocytes, Day 1) . . 287
Table A86 Significantly enriched MSigDB BioCarta Pathways (RNA-Seq, Monocytes, Day 1) 287
Table A87 Significantly enriched MSigDB Chemical/Genetic Perturbation Sets (RNA-Seq, Monocytes, Day 1) . . . 296
Table A88 Significantly enriched KEGG Pathways (RNA-Seq, Neutrophils, Day 1) . . . 297
Table A89 Significantly enriched KEGG Modules (RNA-Seq, Neutrophils, Day 1) . . . 297
Table A90 Significantly enriched MSigDB GO Biological Processes (RNA-Seq, Neutrophils, Day 1) . . 298
Table A91 Significantly enriched MSigDB GO Cellular Components (RNA-Seq, Neutrophils, Day 1) . . 298
Table A92 Significantly enriched MSigDB GO Molecular Functions (RNA-Seq, Neutrophils, Day 1) . . 298
Table A93 Significantly enriched MSigDB Chemical/Genetic Perturbation Sets (RNA-Seq, Neutrophils, Day 1) . . . 309
Table A94 Significantly enriched KEGG Pathways (RNA-Seq, Neutrophils, Day 3) . . . 309
Table A95 Significantly enriched MSigDB GO Biological Processes (RNA-Seq, Neutrophils, Day 3) . . 310
Table A96 Significantly enriched MSigDB Chemical/Genetic Perturbation Sets (RNA-Seq, Neutrophils, Day 3) . . . 313
Table A97 Significantly enriched MSigDB Chemical/Genetic Perturbation Sets (RNA-Seq, NK-cells, Day 1) . . . 315
Table A98 Significantly enriched KEGG Pathways (RNA-Seq, NK-cells, Day 3) . . . 315
Table A99 Significantly enriched MSigDB GO Biological Processes (RNA-Seq, NK-cells, Day 3) . . . 316
Table A100 Significantly enriched MSigDB GO Cellular Components (RNA-Seq, NK-cells, Day 3) . . . 317
Table A101 Significantly enriched MSigDB GO Molecular Functions (RNA-Seq, NK-cells, Day 3) 317
Table A102 Significantly enriched MSigDB BioCarta Pathways (RNA-Seq, NK-cells, Day 3) 317
Table A103 Significantly enriched MSigDB Chemical/Genetic Perturbation Sets (RNA-Seq, NK-cells, Day 3) . . . 326
Table A104 Significantly enriched KEGG Pathways (RNA-Seq, NK-cells, Day 28) . . . 327
Table A105 Significantly enriched MSigDB GO Biological Processes (RNA-Seq, NK-cells, Day 28) . . . 327
Table A106 Significantly enriched MSigDB GO Cellular Components (RNA-Seq, NK-cells, Day 28) . . . 327
Table A107 Significantly enriched MSigDB GO Molecular Functions (RNA-Seq, NK-cells, Day 28) . . . 327
Table A108 Significantly enriched MSigDB BioCarta Pathways (RNA-Seq, NK-cells, Day 28) 328
Table A109 Significantly enriched MSigDB Chemical/Genetic Perturbation Sets (RNA-Seq, NK-cells, Day 28) . . . 330
Table A110 Significantly enriched KEGG Pathways (RNA-Seq, T-cells, Day 1) . . . 330
Table A111 Significantly enriched MSigDB Chemical/Genetic Perturbation Sets (RNA-Seq, T-cells, Day 1) . . . 334

Table A112 Canonical correlation analysis summary statistics (RNA-Seq, B-cells) 335
Table A113 Canonical correlation analysis summary statistics (RNA-Seq, Dendritic cells) 335
Table A114 Canonical correlation analysis summary statistics (RNA-Seq, Monocytes) 335
Table A115 Canonical correlation analysis summary statistics (RNA-Seq, Neutrophils) 336
Table A116 Canonical correlation analysis summary statistics (RNA-Seq, NK-cells) 336
Table A117 Canonical correlation analysis summary statistics (RNA-Seq, T-cells) 336
Table A118 Canonical loadings and explained variance (RNA-Seq, B-cells, Day 1, Gene variables) . . 337
Table A119 Canonical loadings and explained variance (RNA-Seq, B-cells, Day 1, IMO variables) . . . 338
Table A120 Canonical loadings and explained variance (RNA-Seq, B-cells, Day 3, Gene variables) . . 339
Table A121 Canonical loadings and explained variance (RNA-Seq, B-cells, Day 3, IMO variables) . . . 340
Table A122 Canonical loadings and explained variance (RNA-Seq, B-cells, Day 7, Gene variables) . . 341
Table A123 Canonical loadings and explained variance (RNA-Seq, B-cells, Day 7, IMO variables) . . . 341
Table A124 Canonical loadings and explained variance (RNA-Seq, B-cells, Day 28, Gene variables) . . 342
Table A125 Canonical loadings and explained variance (RNA-Seq, B-cells, Day 28, IMO variables) . . 342
Table A126 Canonical loadings and explained variance (RNA-Seq, Dendritic cells, Day 1, Gene variables) 347
Table A127 Canonical loadings and explained variance (RNA-Seq, Dendritic cells, Day 1, IMO variables) 348
Table A128 Canonical loadings and explained variance (RNA-Seq, Dendritic cells, Day 3, Gene variables) 349
Table A129 Canonical loadings and explained variance (RNA-Seq, Dendritic cells, Day 3, IMO variables) 350
Table A130 Canonical loadings and explained variance (RNA-Seq, Dendritic cells, Day 7, Gene variables) 350
Table A131 Canonical loadings and explained variance (RNA-Seq, Dendritic cells, Day 7, IMO variables) 351
Table A132 Canonical loadings and explained variance (RNA-Seq, Monocytes, Day 1, Gene variables) 359
Table A133 Canonical loadings and explained variance (RNA-Seq, Monocytes, Day 1, IMO variables) . 360
Table A134 Canonical loadings and explained variance (RNA-Seq, Monocytes, Day 3, Gene variables) 361
Table A135 Canonical loadings and explained variance (RNA-Seq, Monocytes, Day 3, IMO variables) . 361
Table A136 Canonical loadings and explained variance (RNA-Seq, Monocytes, Day 28, Gene variables) 362
Table A137 Canonical loadings and explained variance (RNA-Seq, Monocytes, Day 28, IMO variables) 362
Table A138 Canonical loadings and explained variance (RNA-Seq, Neutrophils, Day 1, Gene variables) 377
Table A139 Canonical loadings and explained variance (RNA-Seq, Neutrophils, Day 1, IMO variables) . 378
Table A140 Canonical loadings and explained variance (RNA-Seq, Neutrophils, Day 7, Gene variables) 379
Table A141 Canonical loadings and explained variance (RNA-Seq, Neutrophils, Day 7, IMO variables) . 379
Table A142 Canonical loadings and explained variance (RNA-Seq, Neutrophils, Day 28, Gene variables) 381
Table A143 Canonical loadings and explained variance (RNA-Seq, Neutrophils, Day 28, IMO variables) 381
Table A144 Canonical loadings and explained variance (RNA-Seq, NK-cells, Day 1, Gene variables) . . 384
Table A145 Canonical loadings and explained variance (RNA-Seq, NK-cells, Day 1, IMO variables) . . 384
Table A146 Canonical loadings and explained variance (RNA-Seq, NK-cells, Day 3, Gene variables) . . 387
Table A147 Canonical loadings and explained variance (RNA-Seq, NK-cells, Day 3, IMO variables) . . 387
Table A148 Canonical loadings and explained variance (RNA-Seq, NK-cells, Day 7, Gene variables) . . 388
Table A149 Canonical loadings and explained variance (RNA-Seq, NK-cells, Day 7, IMO variables) . . 388
Table A150 Canonical loadings and explained variance (RNA-Seq, NK-cells, Day 28, Gene variables) . 394
Table A151 Canonical loadings and explained variance (RNA-Seq, NK-cells, Day 28, IMO variables) . . 395
Table A152 Canonical loadings and explained variance (RNA-Seq, T-cells, Day 1, Gene variables) . . . 396
Table A153 Canonical loadings and explained variance (RNA-Seq, T-cells, Day 1, IMO variables) . . . 397

Table A154 Canonical loadings and explained variance (RNA-Seq, T-cells, Day 3, Gene variables) . . . 397
Table A155 Canonical loadings and explained variance (RNA-Seq, T-cells, Day 3, IMO variables) . . . 398
Table A156 Canonical loadings and explained variance (RNA-Seq, T-cells, Day 7, Gene variables) . . . 398
Table A157 Canonical loadings and explained variance (RNA-Seq, T-cells, Day 7, IMO variables) . . . 399
Table A158 Canonical loadings and explained variance (RNA-Seq, T-cells, Day 28, Gene variables) . . 399
Table A159 Canonical loadings and explained variance (RNA-Seq, T-cells, Day 28, IMO variables) . . . 400
Table A160 Regularized seroprotection logistic regression model summary statistics (RNA-Seq, Day 1) 401
Table A161 Median fold change in the percentage of live cells by immune cell type and treatment group. 402
Table A162 Median fold change in the number of live cells per mL by immune cell type and treatment group. 403
Table A163 Samples with incomplete cell activation measures (FACS). 404
Table A164 Outlying cell activation samples (FACS). 405
Table A165 List of R packages and versions used for the analyses presented in this report. 406

1 Introduction

This appendix provides supporting information for the manuscript entitled "'Cell-based Systems Biology Analysis of Human AS03-adjuvanted H5N1 Avian Influenza Vaccine Responses: A Phase I Randomized Controlled Trial"'. It provides additional information about reactogenicity (primary), serum antibody (secondary) as well as gene expression (tertiary), cytokine (exploratory), and cell activation (exploratory) outcomes and methods. The study for which this data was collected, was a single center randomized, double-blinded, controlled, Phase I small targeted prospective study in healthy male and non-pregnant female subjects, 19 to 39 years old, inclusive, designed to assess the safety, reactogenicity, immunogenicity, and molecular immune responses of an intramuscular (IM) inactivated monovalent influenza A/H5N1 (Hemagglutinin [HA] of A/Indonesia/05/2005) split-virus (SV) vaccine manufactured by Sanofi Pasteur administered at 3.75 mcg given with the AS03 adjuvant (SV-AS03) manufactured by GlaxoSmithKline (GSK) or Phosphate Buffered Saline (PBS) diluent (SV-PBS). Subjects who met the entry criteria for the study and provided informed consent were randomized 1:1 to SV-AS03 and SV-PBS vaccine and each subject received two doses of the respective vaccine approximately 28 days apart (**Figure A1**).The primary and secondary endpoints of the study were defined as:

Primary Safety Endpoints:

- Occurrence of vaccine - associated serious adverse events (SAEs) from the time of first vaccination through 13 months after the first vaccination.
- Occurrence of solicited local and systemic reactogenicity in the 8 days (Days 0-7) after each vaccination.

Primary Immunogenicity Endpoints:

- GMT of HAI antibody, proportion of subjects achieving a serum HAI antibody titer of 1:40 or greater, and frequency of 4-fold or greater increases of HAI antibodies in each group against the subvirion inactivated A/H5N1 virus vaccine approximately 1, 3, 7 and 28 days after receipt of the first dose of vaccine.
- GMT of HAI antibody, proportion of subjects achieving a serum HAI antibody titer of 1:40 or greater, and frequency of 4-fold or greater increases of HAI antibodies in each group against the subvirion inactivated A/H5N1 virus vaccine approximately 1, 3, 7 and 28 days after receipt of the first dose of vaccine.
- GMT of neutralizing antibody, proportion of subjects achieving a serum neutralizing antibody titer of 1:40 or greater, and frequency of 4-fold or greater increases of neutralizing antibodies in each group against the subvirion inactivated A/H5N1 virus vaccine approximately 1, 3, 7 and 28 days after receipt of the first dose of vaccine.

Secondary Immunogenicity Endpoints:

- GMT of HAI antibody, proportion of subjects achieving a serum HAI antibody titer of 1:40 or greater, and frequency of 4-fold or greater increases of HAI antibodies in each group against the subvirion inactivated A/H5N1 virus vaccine 28 days after receipt of the second dose of vaccine (approximately Day 56).
- GMT of neutralizing antibody, proportion of subjects achieving a serum neutralizing antibody titer of 1:40 or greater, and frequency of 4-fold or greater increases of neutralizing antibodies in each group against the subvirion inactivated A/H5N1 virus vaccine 28 days after receipt of the second dose of vaccine (approximately Day 56).

2 Supplemental methods

2.1 Selection of study population

Twenty healthy male and non-pregnant female subjects, 19 to 39 years old (actual age), inclusive, were enrolled in this study. Subjects were recruited from the community at large at a single Vaccine and Treatment Evaluation Unit (VTEU), Vanderbilt University, and enrolled over a 6-month period.

2.1.1 Inclusion criteria

Subjects had to meet all of the following protocol-defined inclusion criteria to participate in this protocol:

- Males or non-pregnant females between the ages of 18 and 49 years
- Women of childbearing potential (not surgically sterile via tubal ligation, bilateral oophorectomy or hysterectomy or who were not postmenopausal for $\geq$ 1 year) must agree to practice adequate contraception (that included, but was not limited to, abstinence, monogamous relationship with vasectomized partner, barrier methods such as condoms or diaphragms with spermicide or foam, intrauterine devices, and licensed hormonal methods) during the study for at least 30 days following the last vaccination. Method of contraception was captured on the appropriate case report form (CRF).
- Were in good health, as determined by vital signs (oral temperature, pulse and blood pressure), medical history, and complete physical examination (without genital and rectal exam) to ensure no existing chronic medical diagnoses or conditions were present.
- For women of childbearing potential, negative urine or serum pregnancy test within 24 hours prior to vaccination.
- Were able to understand and comply with planned study procedures.
- Provide written informed consent prior to initiation of any study procedures.

2.1.2 Exclusion criteria

Subjects who met any of the following exclusion criteria were excluded from study participation:

- Had a known allergy to eggs or other components of the vaccine (including gelatin, formaldehyde, octoxinol-9, thimerosal and chicken protein), or allergy to squalene-based adjuvants.
- Women who were breastfeeding or planned to breastfeed at any given time from the first vaccination until 30 days after the last vaccination.
- Long term use (defined as taken for 2 weeks or more in total at any time during the past 2 months) of high dose oral or parenteral glucocorticoids (high dose defined as prednisone $\geq$20 mg total daily dose, or equivalent dose of other glucocorticoids); or high-dose inhaled steroids (high dose defined as 800 mcg/day of beclomethasone dipropionate or equivalent); or systemic corticosteroids of any dose within the past 4 weeks.
- Had immunosuppression as a result of an underlying illness or treatment, or use of anticancer chemotherapy or radiation therapy (cytotoxic) within the preceding 36 months.
- Had an active neoplastic disease or a history of any hematologic malignancy.
- Had a diagnosis of schizophrenia, bipolar disease, or other major psychiatric diagnosis.
- Hospitalized for psychiatric illness, history of suicide attempt, or confinement for danger to self or others, within the past 10 years.
- Received systemic, prescription medications for the treatment of chronic medical conditions, unless such use is on a PRN (as needed) basis only. Non-PRN use of systemic, over-the-counter medications and PRN systemic, prescription medication may have been allowed if, in the opinion of the investigator, they posed no additional risk to subject safety or assessment of immunogenicity/reactogenicity. Note: Topical, nasal, and inhaled medications, vitamins, and contraceptives were also permitted.
- Received pre-medication with analgesic or antipyretic agents in the 6 hours prior to the first vaccination, or planned medication with analgesic or antipyretic in the week following the first vaccination. This criterion did not preclude subjects receiving such medication if the need arose. However, pre-medication was to be discouraged.
- Received immunoglobulin or other blood products (with exception of Rho D immune globulin) within the 3 months prior to the first vaccination.
- Received any live licensed vaccines within 4 weeks or inactivated licensed vaccines within 2 weeks prior to the first vaccination or planned receipt of such vaccines within 56 days following the first vaccination. This was inclusive of licensed seasonal influenza vaccines.
- Had an acute or chronic medical condition that, in the opinion of the site PI or appropriate sub-investigator, would render vaccination unsafe, would interfere with the evaluation of responses, or is not generally seen in "normal, healthy subjects".
- Had a history of severe reactions following previous immunization with contemporary influenza virus vaccines.

- Had an acute illness, including an oral temperature greater than or equal to 100.4°F, within 3 days prior to the first vaccination.
- Pulse was less than 55 bpm or greater than 100 bpm.
- Systolic blood pressure was less than 90 mmHg or greater than 140 mmHg.
- Diastolic blood pressure was less than 60 mmHg or greater than 90 mmHg.
- Received an experimental agent (vaccine, drug, biologic, device, blood product, or medication) within 1 month prior to the first vaccination or expects to receive an experimental agent, other than from participation in this study, during the 14-month study period.
- Were participating or planned to participate in another clinical trial with a licensed product during the 14-month study period.
- Had any condition that, in the opinion of the site PI or appropriate sub-investigator, would place the subject at an unacceptable risk of injury, or render them unable to meet the requirements of the protocol, or confound the interpretation of results.
- Participated in an influenza A/H5 vaccine study in the past in a group receiving vaccine (did not apply to documented placebo recipients) or have a history of A/H5 infection prior to enrollment.
- Had known active HIV, Hepatitis B, or Hepatitis C infection.
- Had a history of alcohol or drug abuse in the last 5 years.
- Planned to travel outside the U.S. in the time between the first vaccination and 56 days following the first vaccination.
- Had a history of Guillain-Barre Syndrome.

2.2 Study visit windows

Study visits of interest and their windows are described in the following ways:

- Day -28 (visit 00A) window: -7, +14 days.
- Day -14 (visit 00B) window: ±14 days.
- Day 0 (Visit 1, Dose 1).
- Day 1 (visit 2) window: +1 day.
- Day 3 (visit 3) window: +1 day.
- Day 7 (visit 4) window: +2 days.
- Day 28 (visit 5) window: ±2 days.
- Day 56 (visit 7) window: ±2 days.

2.3 Reactogenicity definitions

Local reactions included pain, tenderness, erythema, and induration. Pain (experienced without touching the injection site) was defined as mild if the subject was aware of pain but it did not interfere with daily activity and no pain medication was taken, as moderate if the subject was aware of pain and it interfered with daily activity or it required use of pain medication, and as severe if the subject was aware of pain and it prevented daily activity. Tenderness (hurts only when injection site is touched) was defined as mild if the area immediately surrounding the injection site hurt only when touched and it did not interfere with daily activity, as moderate if the area immediately surrounding the injection site hurt when touched and it interfered with daily activity, and severe if the area immediately surrounding the injection site hurt when touched and it prevented daily activity. Both erythema (redness) and induration (swelling) were defined as mild if it did not interfere with daily activity, as moderate if it interfered with daily activity, and as severe if it prevented daily activity. Fever was defined as mild if the oral temperature was 38.0-38.4°C (100.4-101.1°F), as moderate if the oral temperature was 38.5-38.9°C (101.2-102°F), and severe if the oral temperature was $\geq$39° ($\geq$102.1°F). Systemic reactions included feverishness, chills, shivering, arthralgia (joint pain), asthenia (weakness), malaise (fatigue), myalgia (body aches - not at injection site), headache and nausea. All of the systemic reactions were described as mild if there was no interference with activity, as moderate if there was some interference with activity, and as severe if there was significant interference that prevented daily activity.

Please refer to the study protocol for information about definition and collection of additional safety events including unsolicited adverse events and serious adverse events.

2.4 Analysis population

The systems biology analysis population (RNA-Seq, cytokines, cell activation) included all subjects who met all inclusion and exclusion criteria, who contributed at least one pre-vaccination blood sample (at Day -28, -14, or 0) and at least one post-vaccination blood sample (at Day 1, 3, 7, or 28) for which valid results were reported. The analyses included subjects by study product actually received. This population included 20 subjects (10 for each vaccine group randomly labeled A-T) including subject B in the SV-AS03 group, who received the second vaccination (Day 28) out-of-window. Time points with outlying measurements were identified separately for each assay (see following sections for details).

2.5 Cytokine/chemokine cytometric bead array methods

Cytometric bead array results below the lower limit of detection (LOD) were imputed using one half of the LOD estimate provided by BD Biosciences (**Table A8**). Concentrations from technical replicates were combined using the mean concentration. Baseline concentration was determined using the mean concentrations among pre-vaccination measurements. A 95% CI for the median cytokine concentration

as well as median fold change from baseline for each time point and vaccine group was obtained using the bootstrap method with 1,000 bootstrap replicates each. In both cases, significance of a shift in distribution for each molecule and study visit day was evaluated using a two-sided exact Wilcoxon Rank Sum test. An individual alpha level of 0.05 was applied in each case.

2.6 RNA-Seq methods

2.6.1 RNA-Seq experiment

For each study visit day, total RNA was extracted from PBMCs and sorted immune cells ($< 0.5x10^6$ cells) using the automated Maxwell 16 magnetic particle processor and the Maxwell 16 LEV simply RNA kit (Promega Corp.). RNA was quantified by Quant-iT RiboGreen RNA Assay, and samples were evaluated on the Caliper LabChip GX to assess RNA integrity. 100ng total RNA and RNA integrity numbers greater than 7 were required for proceeding to downstream RNA-Seq applications. Poly(A) mRNAs were isolated using NEBNext magnetic oligo d(T)25 beads. NEBNext mRNA Library Prep Reagent Set for Illumina (New England BioLabs Inc.) was used to prepare individually bar-coded next generation sequencing expression libraries. Libraries were pooled (10mM each sample). Overall, 839 RNA sequence libraries were constructed, one for each subject, cell type and study visit combination with one library (Subject I, T-cell, Day 3) failing due to insufficient RNA. Approximately 25 million, 50-bp, paired-end read sequencing was performed for each library utilizing a 200 cycle TruSeq SBS HS v3 kit on an Illumina HiSeq2000 sequencer (Illumina, Inc.). Sequencing libraries are henceforth also referred to as samples.

2.6.2 RNA-Seq data preprocessing

Reference Consortium Human Build 37 (GRCh37) genomic assembly was used as reference sequence. Human gene models and annotations were obtained from the Ensembl database (Version 63, June 2011). Paired-end reads were mapped against the human reference genome using the *TopHat* splice-aware read aligner (Version 2.0.0). Ensembl gene models were used to guide the alignment process. Alignment QC statistics were obtained using the *RSeQC* software (Version 2.3.7). Gene expression quantification was carried out using the *featureCounts* function as implemented in the *Subread* software (Version 1.4.6). *featureCounts* options were set to count fragments, i.e. paired-end reads that reconstitute a fragment for which both ends were successfully mapped to a gene. Reads/fragments that overlapped with multiple genes or had multiple mapping locations on the reference genome were excluded. Systematic sample differences in sequencing depth (number of fragments) were corrected for by calculating RNA composition-robust scaling factors for each sample using the trimmed mean of M-values (TMM) method (Robinson et al. 2010) as implemented in the *edgeR* software (Version 3.2.4). TMM normalization was executed across all 839 libraries for evaluating global biases and outliers, and separately for each cell type excluding outliers for differential analysis. In either case, post normaliza-

tion, 4,185 genes that were known ribosomal, transfer, and mitochondrial RNA genes or genes located on the X or Y chromosomes were excluded to avoid gender-specific effects (**Table A9**) leaving a total of 46,870 genes to be analyzed. For data visualization and multivariate analyses, TMM normalized moderated log_2 fragment counts per million (LCPM) were computed using *edgeR*. To avoid taking the log of zero values, a TMM-scaled fragment count of 0.5 was added to each gene (the imputed value was different for each sample based on its TMM scaling factor). Genes with less than 1 count per million in 60% of the libraries at any post-vaccination study visit (Days 1, 3, 7, 28) were deemed lowly expressed and were excluded (at a minimum, 8 libraries were required to pass the cut-off). Filtering was carried out for all libraries and separately within cell type, keeping genes that met the cut-off at any post-vaccination day for time-trend analyses. For differential analysis, filtering was carried out within cell type for each post-vaccination study visit sample. **Table A10** lists the number of genes that were retained for each cell type and day. Subject specific log_2 fold changes from baseline (LFCPM) were calculated for each subject and post-vaccination day (Day 1, 3, 7, 28) by subtracting the mean of the log_2 baseline values from each of the subject's post-vaccination days.

2.6.3 RNA-Seq statistical analysis

2.6.3.1 Statistical model for identifying differentially expressed genes Differential expressed (DE) genes (SV-AS03 vs. SV-PBS) were identified for each post-vaccination day and immune cell type by fitting negative binomial generalized linear models as implemented in edgeR (Robinson et al, 2010) (v3.2.4) under the assumption that discrete fragment counts are negative binomial distributed. Trended dispersion estimates based on the mean-variance trend across all genes for a particular cell type and study visit day combination were obtained, and gene-wise negative binomial dispersion parameters were shrunk towards the trended dispersion estimates using the empirical Bayes method as implemented in edgeR. For each model, the design matrix included fixed effects for subject, study visit day (baseline or post-vaccination day), and a study visit day x vaccine group interaction term. The study visit day and vaccine group effects had two fixed levels (0: for baseline days -28, -14 or 0, and 1: for the respective post-vaccination day), and (0:SV-PBS and 1:SV-AS03), respectively. The subject effect for estimating subject-specific mean baseline levels was added to account for correlations between samples from the same subject. Maximum likelihood estimates of the coefficients including baseline levels were estimated using the parallelized line search algorithm described by McCarthy et al. (2012). The interaction term represents the log_2 fold change difference (LFCD) between SV-AS03 and SV-PBS vaccine group responses. The statistical significance of the interaction term was evaluated for each gene using a likelihood ratio test to identify genes that significantly differed in their response from baseline between vaccine groups. To control for testing multiple genes, the false-discovery rate (FDR) based on the Benjamini-Hochberg procedure as implemented in the p.adjust() R function was applied. Genes with a FDR-adjusted p-value $\leq$ 0.05 and a fold change of $\geq$ 1.5-fold (up or down regulation based on LFCD) were deemed to be significantly differentially expressed between vaccine groups.

2.6.3.2 Determination of significant gene clusters Pairwise distances between genes based on their baseline log_2 changes (LCPM) were calculated separately for each cell type and post-vaccination study day (Day 1, 3, 7, 28) and for all study days (Day 1-28). Uncentered Pearson correlation distance was used as distance measure as log fold changes are, by definition, centered around zero. For all these comparisons, the list of genes included genes that were differentially expressed for a certain cell type at any of the post-vaccination days. Clusters were obtained using the hierarchical complete linkage clustering algorithm. To evaluate robustness of gene clusters, multiscale bootstrapping (Suzuki and Shimodaira, 2006) was carried out separately for each cell type and post-vaccination day using varying dataset sizes (0.4*N, 0.5*N, 0.6*N, 0.7*N, 0.8*N, 0.9*N, 1*N, 1.1*N, 1.2*N, 1.3*N; where N stands for the respective dataset size). For each dataset size bin, 1,000 bootstrap samples were obtained, and bootstrap probabilities and p-values were calculated. A p-value cut-off of 0.05 was applied for lymphocytes (B-cells, NK-cells, and T-cells), and 0.01 for neutrophils, dendritic cells, and monocytes when determining significant clusters to reduce the impact of false-positives for the latter group with many more differentially expressed genes. In either case, the maximum distance to form a significant cluster was set to 0.5 (equivalent to minimum uncentered Pearson correlation of 0.5). Clusters that were formed at larger distance were excluded.

2.6.3.3 Gene set enrichment analysis Gene set enrichment analysis was carried out separately for each time point and cell type. Genes were grouped and analyzed based on 8,101 known gene sets obtained from the KEGG database (Version 70.0, 06/09/2014) and MSigDB (Version 4.0, 05/31/2013). The KEGG subset included KEGG pathways and KEGG modules. The MSigDB gene set collection comprised GO Biological Processes, GO Cellular Components, GO Molecular Functions, Reactome Pathway, BioCarta Pathway, Chemical/Genetic Perturbations, and Immunologic Signature gene sets. **Table A73** summarizes the gene set collection used in this report. For each of these gene sets, enrichment was evaluated using the *goseq* R package (Version 1.12.0). The GOseq algorithm adjusts for gene length bias when modeling the null distribution (Young et al., 2010). Gene length was specified as the length of the longest cDNA of a gene. Gene length information was obtained from the *tweeDEseq-CountData* R package (Version 1.0.9). GOseq probability weighting functions were estimated for each cell type and post-vaccination study day based on differentially expressed genes. Null distributions were generated using GOseq's random sampling option (100,000 randomizations were applied). Lists of enriched gene sets were obtained separately for each of the nine category types (KEGG pathways, KEGG modules, GO Biological Processes, etc.). To adjust for testing multiple gene sets per category type, the Benjamini-Hochberg procedure as implemented in the p.adjust() R function was applied to each list. Gene sets with a FDR-adjusted p-value $\leq$ 0.01 were considered to be significantly enriched. Enrichment trends across study visit days and cell subsets were visualized using heatmaps and binary Jaccard-distance based complete-linkage hierarchical clustering. For significantly enriched KEGG pathways, pathways were color-coded by log_2 fold change between vaccine groups. If nodes in the pathway referred to multiple genes, the median log_2 fold change was used to set the background color

of a node (red: up-regulated in the SV-AS03 group compared to the SV-PBS group, green: down-regulated in the SV-AS03 group compared to the SV-PBS group). If one of the genes of a multi-gene node was significantly enriched, the node label and border was color-coded (red: up-regulated, green: down-regulated, blue: conflict if one gene was up but another was down regulated).

2.6.3.4 Protein-protein interaction network integration Ensembl gene IDs were used to obtain UniProt IDs and gene symbols using the UniProt ID mapping tool (http://www.UniProt.org/uploadlists). Human-human and human-*Influenza A* protein-protein interactions were downloaded from the IntAct database (http://www.ebi.ac.uk/intact/) using the following IntAct search term: (taxidA: 9606 AND taxidB: 9606) OR ((taxidA: 9606 AND taxidB: 197911) OR (taxidB: 9606 AND taxidA: 197911)). The resulting dataset contained 109,291 interactions in MITab27 format and was downloaded 01/14/2015. For a certain cell type and study visit day, this network was filtered for nodes with a network distance of 1 to a protein encoded by a significant gene were retained (i.e., at least one of the interacting proteins had to be encoded by a significant gene). Protein-protein interaction network visualizations were generated using the *Cytoscape* software package (Version 3.1.1). An additional filter requiring two distinct publications to report a human-human interacting protein pair was used to reduce network complexity if needed (in this case, human-*Influenza A* protein interactions with a single publication were still included).

2.6.3.5 Regularized canonical correlation analysis To identify combinations of gene responses that best correlate with changes in cytokine, chemokine, and antibody responses, regularized Canonical Correlation Analysis (RCCA) was be carried out for each cell type, post-vaccination time point, and vaccine group combination using the *mixOmics* R package (Version 5.0-3). RCCA identifies linear combinations within two variable sets that maximize the inter-set correlation, here, the correlation between genes and immunogenicity outcomes. The gene variable set was based on log fold changes (LFCPM) and included genes with an average absolute baseline fold change of $\geq$ 1.5 in at least one of the two vaccine groups (group 1: SV-AS03, group 2: SV-PBS). The immunogenicity variable set (referred to as IMO variable set) included baseline log fold changes of cytokine concentrations (up-to 12 cytokines: Interferon gamma (IFN-γ), Interleukin-1 beta (IL-1β), Interleukin-12 p70 (IL-12p70), Interleukin-6 (IL-6), Interleukin-8 (IL-8), Tumor necrosis factors (TNF), Interleukin-10 (IL-10), Interferon gamma-induced protein 10 (IP-10), Monocyte Chemoattractant protein-1 (MCP-1), Chemokine (C-C motif) Ligand 5 (RANTES), Eotaxin-1 (Eotaxin), Macrophage inflammatory protein (MIP-1α), as well as baseline log fold changes of antibody titers (hemagglutination inhibition (HAI), and microneutralization inhibition (Nt) assays). Interleukin-1 alpha (IL-1α) and, Lymphotoxin-alpha (LT-α) were excluded for all cell types and post-vaccination days as no changes from baseline were observed.

A regularization step was included to avoid overfitting and handle collinearity within variable sets. Regularization parameters (λ_1 and λ_2) were estimated using leave-one-out cross validation (grid-search of

40,000 parameter combinations ranging from 0.00001 to 1 in 200 increments). The top two canonical variate pairs that maximize inter-set canonical correlation were identified. Correlations between original variables (Pearson correlation) and corresponding canonical variates were calculated (canonical loadings). Canonical loadings were squared to obtain the percent-explained variance for each variable. Except for high level summaries, RCCA results with a cross-validation score of < 70 were excluded from tabular and graphical summaries.

Canonical loadings for each variable set were visualized for the first two canonical variates using 2-dimensional canonical correlation circle plots. Gene and IMO variables for which the first two canonical variates explained less than 25% of their total variance (placed within the inner circle of the correlation circle plots) were grayed out. Variables or groups of variables that are closely positioned in these plots are positively correlated while those diametrically opposed are negatively correlated. The strength of the correlation is encoded by the distance from the center of the circle as well as by the angle between variables when viewed as vectors originating from the center. A sharp angle between variables represents a positive correlation, an obtuse angle indicates a negative correlation while a right angle is observed for zero correlation. Thus, maximum correlation is achieved when variables are closely placed together to the outer circle (or directly opposed on the outer circle). KEGG BRITE pathway hierarchy (second level) information for the top five pathway categories (in terms of number of genes that were correlated) was overlaid on top of the correlation circle plots to highlight known functional organization. Identified robust gene clusters (see **Section 2.6.3.2**) were integrated by connecting correlated genes that formed a robust gene cluster. Correlation results between vaccine groups were contrasted by plotting canonical loadings for the SV-AS03 group against those obtained for the SV-PBS group.

2.6.3.6 Regularized logistic regression For each cell type, a regularized logistic regression model was fit to identify Day 1 gene responses that are correlated with seroprotection as defined by a HAI antibody titer of $\geq$ 1:40 at Day 56 using the *glmnet* R package (Version 2.0-2). The predictor variable set was based on log baseline fold changes (LFCPM) and included genes with an average absolute fold change from baseline $\geq$ 1.5 in at least one of the two seroprotection response groups (group 1: HAI $\geq$ 1:40, group 2: HAI $<$ 1:40). To avoid overfitting (n $<<$ p and collinearity among gene responses) and facilitate variable selection, an elastic net regularization step (combination of L1 Lasso and L2 ridge penalization, α = 0.5) was included as part of the fitting procedure. Leave-one-out cross validation was used to determine the optimum regularization parameter λ that minimizes the misclassification error between seroprotection response groups.

2.7 Flow cytometric immune cell phenotyping and activation methods

2.7.1 Flow cytometric immune cell phenotyping and activation data processing

After gating live cells, individual cell types were gated sequentially: 1) CD15+ neutrophils and CD3+ T cells; 2) from the CD15-CD3-, CD19+ B-cells and CD14+ monocytes; 3) from the CD15-CD3-CD19-CD14- fraction, CD11c dendritic cells and CD56+ NK-cells were gated. The percentage of each cell type in whole blood was calculated using the following formula: # of gated cell events/# of total live events. The number of each cell type/mL of whole blood was calculated by multiplying the percentage of each cell type by the concentration of blood cells in the whole blood sample. The FMO gates for activation marker expression were set to contain the same percentage of events across each cell type and activation marker on a per-subject basis, excluding obvious outliers. This value was based on the number of events contained in each cell type gate. For the whole blood samples, the values were as follows:

- 99.9% for samples with >20,000 events (most neutrophil samples, a few T-cell samples)
- 99.8% for samples with 10,000-20,000 events (most T-cell samples, a few neutrophil samples)
- 99.5% was used for medium cell populations, containing 500-5,000 events (B-cell, monocyte, and some NK-cell samples)
- 99.0% was used for small cell populations containing less than 500 events (dendritic cells and some NK-cells)

These gates were then copied from the FMO sample to the corresponding cocktail sample, and cells outside of the FMO gate were considered “expressing cells”. The percentage of expressing cells was calculated for each cell type using the following formula: # of activated cells/# of cell type population. Percentages of expressing cell obtained for whole blood samples for each of the 3 markers (CD134, CD69, CD86) and 6 cell types (T-cells, neutrophils, monocytes, B-cells, dendritic cells, and NK-cells) were used for downstream analyses.

2.7.2 Flow cytometric immune cell activation outlier detection and normalization

The percentages of expressing cells for the whole blood samples were inspected for potential outliers using univariate (boxplots) and multivariate methods (non-metric multidimensional scaling biplots). Boxplots were generated for each cell type and marker combination summarizing the percentage of expressing cells across all samples (i.e. all subject and time point combinations) with non-missing values. Non-metric multidimensional scaling biplots were generated for each marker. Subject and time point combinations (samples) were represented by a mutivariate vector that included all 6 cell type measurements (only samples for which percentages for all six cell types were recorded were included in this analysis). Pairwise distances between samples were then calculated using the Euclidean distance.

Following outlier removal, correlation between detector settings and percent cell activation was evaluated for each marker and cell type using scatter plots as well as Pearson and Spearman correlation metrics. Normalization for a subset of marker and cell type combinations was carried out as follows:

(1) a linear regression model was fit to obtain slope and intercept coefficients that together describe the mean trend

(2) the center of the detector range was obtained by calculating the mean between the minimum and maximum reported detector setting

(3) the estimated mean percentage of expressing cells at the center of the detector range (2) was calculated based on the regression fit obtained in (1)

(4) the estimated mean percentage of expressing cells for each observation was calculated based on the respective detector setting and the regression fit obtained in (1)

(5) a scaling factor for each observation was then calculated as the ratio between the estimated mean percentage of expressing cells at the center of detector range (3) and the estimated mean percentage of expressing cells based on the detector setting for each observation (4)

(6) the percentage of expressing cells for each observation was then normalized by multiplying it with its corresponding scaling factor

The impact of normalization and potential association with vaccine group and study visit day was visually inspected using scatter plots. In addition, scaling factor distributions for the two vaccine groups were compared for each marker and cell type combination using a two-sided non-parametric Wilcoxon Rank-Sum test. Scaling factor distributions between the seven study visit days were compared using a non-parametric two-sided Kruskal-Wallis Rank-Sum test.

2.8 Software

Data was analyzed using the *R statistical programming language* and *R Bioconductor* packages. A complete listing of R and package versions are given in **Table A165**. This report was generated using the *knitr* R package (Version 1.5) and *LaTeX* typesetting software (Version TeX Live 2012/Debian). The operating system used was *Ubuntu* (Version 13.04).

3 Supplemental results

Demographic and baseline characteristics for the analysis population are summarized in **Table A1**. Overall, the majority of subjects were female (55%), and all subjects were non-Hispanic and White. A greater percentage of subjects in the SV-PBS study group were female (70%) than in the SV-AS03 group (40% female). Ethnicity and race distributions were similar across groups. The mean age for all subjects was 28.3 years (range: 19.3 to 39.3 years), with a median age of 27.6. The median age for the SV-AS03 group was 26.1 years, and the median age for the SV-PBS was 29.1 years of age.

3.1 Safety and immunogenicity results

A summary of local and systemic reactions by severity and study arm is given in **Figure A2** and **Tables A2 and A3**. HAI and Nt titer statistics for the intent-to-treat and per-protocol populations are listed in **Table A7**.

3.1.1 Local and systemic reactogenicity

No subjects reported severe solicited local or systemic reactions during the 8-day post-vaccination period for either dose. Following the first vaccination, 2 subjects (20%) reported moderate and 7 subjects (70%) reported mild solicited local reactions in the SV-AS03 group, while 4 subjects (40%) reported mild local reactions in the SV-PBS group, and no subjects reported moderate local reactions in the SV-PBS group (**Tables A2 and A3**). The most frequently reported local reactions were injection site pain and tenderness. One subject (10%) reported moderate and 5 subjects (50%) reported mild solicited systemic symptoms during the 8 days post vaccination 1 in the SV-AS03 group. Two subjects (20%) reported moderate and 2 subjects (20%) reported mild systemic symptoms in the SV-PBS group. The most frequently reported systemic symptoms following both vaccinations were headache and malaise (**Tables A2 and A3**).

3.1.2 Adverse events

Two unsolicited AE occurred in the SV-AS03 group; both AE were classified as 'mild' and unrelated to vaccination. Seven unsolicited AE occurred in the PBS group; one (neck pain classified as 'mild' was considered related to vaccination and resolved within 24 hours. Six of the unsolicited AE in the PBS group were classified as 'mild', while one event was classified as 'moderate' (upper respiratory infection). No severe AE or new-onset chronic medical conditions occurred in either study group.

3.2 Cytokine/chemokine cytometric bead array results

Time trends for median cytokine/chemokine concentrations as well as baseline fold changes with corresponding 95% CIs are shown in **Figures A3 to A16**.

3.3 RNA-Seq results

3.3.1 Reference alignment statistics

Tables A11 and A12 summarize key technical variables and alignment statistics. On average, 56.2 million reads were mapped for each sample against the reference, out of which, 83.4% uniquely mapped to a location on the human reference assembly. Paired-end reads that mapped to different chromosomes were hardly existing (only 4 cases were reported overall). When mapped against known gene

models, on average, 16.7 million fragments (combined paired reads that mapped to the same gene) were uniquely counted in the expression quantification step. Sample mean %GC content of mapped sequences ranged from 47% to 54%. The number of Illumina flow cells per library/sample ranged from 1 to 7 with 1-15 lanes per sample (on average 3 flow cells and 10 lanes).

The vast majority (on average 81.7%) of tags (spliced reads) mapped to known[1] exon regions followed by intronic (16.6%), and intergenic regions (1.8%). Coverage for coding DNA sequence regions was, on average, 485 tags per Kb of sequence, compared to 6.6 tags per Kb for introns, and a maximum of 3.34 tags per Kb for intragenic regions (10Kb up/downstream). For most of the detected splice junctions (90.9% on average), both splice sites were part of the gene models with a small fraction (2.5%) for which neither of the two splice sites were reported, indicating that the applied gene model captured nearly all of the splicing junction information in the data. For a newer version of Ensembl[2], the average percent of known splice junctions was 91.4%. The small increase (0.5%) in known junctions implied that recent gene model additions did not substantially improve coverage of identified junctions.

Boxplots of subsets of these statistics by cell type are given in **Figures A17 and A18**. Overall, neutrophils showed the most distinct patterns with higher FACS cell counts, a higher proportion of uniquely mapped reads, a higher number of tags for the 3' untranslated region, an overall lower number of reads spanning splicing junctions, and among those, the highest fraction of novel junctions compared to the other cell types. Dendritic cells had comparatively low FACS cell counts compared to the rest. B-cells, as for neutrophils, had a higher fraction of novel splicing junctions. B-cells also had a comparatively high percentage of intergenic tags. Dendritic cell and monocyte samples contained a higher percentage of tags that mapped against known exon regions.

Starplots that visualize alignment statistics across cell types, time points, and vaccine groups are shown in **Figures A19 to A24**. Most variation was observed between cell types with more homogeneous across-sample profiles for B-cells, dendritic cells, and NK-cells. Although subject-specific patterns were observed (e.g. subject D for dendritic cells or Q and C for neutrophils), no strong effects related to vaccine group or study visit day were evident.

3.3.2 Impact of TMM normalization

TMM normalization reduced sample-to-sample variation with improved alignment of log_2 counts per million within cell types (**Figures A25 to A27**). Neutrophils showed distinct distributional properties compared to the other cell types, with higher variation and a lower median. This observation was due to a higher fraction of lowly expressed genes when compared to the other five cell types (**Figure A27**).

[1] Ensembl gene models (Version 63, June 2011)
[2] Ensembl gene models (Version 74, December 2013)

These neutrophil-specific distributional proprieties were consistently observed before and after normalization and across time points, pointing towards a different RNA composition for neutrophils. At Day 28, two samples showed outlying profiles: (1) sample [subject B, B-cells, day 28] with a distributional profile more similar to that of neutrophils, and (2) sample [subject J, dendritic cells, day 28] with very low log_2 counts per million.

3.3.3 Global gene expression patterns, confounding effects, and outlying samples

Principal component analysis of the original and standardized gene variables [log_2 counts per million] revealed that most of the total variation in gene expression was attributable to cell type (**Figure A28**) with three distinct cell clusters: (1) neutrophils, (2) lymphocytes (T-cells, B-cells, NK-cells), and (3) dendritic cells/monocytes. This finding was further confirmed by patterns observed when visualizing the data using non-metric multidimensional scaling, hierarchical clustering, and heatmap plots - all of which showed a strong separation of samples by cell type (**Figures A29 to A31**). Analysis of variance of the first two Principal Components identified cell type as a major source of total variation, followed, to a much smaller extent, by variability introduced by subjects (**Tables A17 and A18**). Study visit day did not play a significant role in describing total variation. This observation was also evident when plotting the first Principal Component against each of these factors (**Figure A36**).

Overall explained variance in gene expression ranged from 45.1% to 52.2% for the first Principal Component (PC1) and 17.2% to 21.6% for the second Principal Component (PC2). Among cell types, the major drivers (PC1, x-axis) were neutrophils to the right and lymphocytes (T-cells, B-cells, NK-cells) to the left. The second major trend (PC2, y-axis) was observed for the dendritic/monocyte cluster at the top versus the rest at the middle/bottom of the spectrum.

99.6% bivariate confidence ellipses indicate the boundaries for each cell type cluster outside of which one would expect 0.5 outliers (given the number of samples per cell type assuming a bivariate t-distribution). Among the 839 samples, two extreme outliers far beyond their respective boundaries were observed: (1) sample [subject J, dendritic cells, day 28] and (2) sample [subject B, B-cells, day 28] (**Figure A28**). These samples were already identified based on their outlying univariate log_2 distributions. Sample [subject B, B-cells, day 28] appeared to have been wrongly labeled as a B-cell sample as its expression profile was most similar to that of neutrophils. For both of these samples, experimental issues in the corresponding FACS experiment were recorded. PCA and non-metric multidimensional scaling biplots that focus on the plot region for each cell type are given in **Figures A32 to A34**. Eight additional samples including three baseline samples were flagged as strong outliers based on their placement compared to other cell type samples (blue circles). Samples belonging to subject H (Day -28, highlighted in red) exhibited moderately outlying patterns for dendritic cells, monocytes, and NK-cells. This observation was further inspected by plotting the difference between maximum and

minimum Spearman correlation between log_2 counts per million of baseline samples for each subject (**Figure A35**). The baseline correlation analysis confirmed the 3 flagged baseline outliers identified earlier. In addition, the boxplots showed subject H (Day -28) as an outlier for 4 cell types (dendritic cells, monocytes, NK-cells, and neutrophils). Inspection of the laboratory data showed that a very low PBMC count ($106x10^6$ total cells) after initial fractionation of the whole blood was obtained for this subject and time point. Based on these findings, it was decided to flag these four baseline samples as additional outliers. A listing of all outliers and issue records are provided in **Table A19**. Outlying samples were removed from downstream analysis.

Spearman correlations between technical and gene model variables with the first two Principal Components before and after normalization are summarized in **Tables A13 and A16**. Results for the first Principal Component (PC1) are described here. Correlations were very similar for the original and normalized data with strong associations with percent uniquely mapped reads and the number of splicing junctions. As shown earlier, both of these variables, when inspected across cell types, showed distinct patterns for neutrophils (**Figures A17 to A18**). As PC1 mainly explained the difference between neutrophils and the rest of the cell types, it is expected to see higher correlations for these neutrophil-associated variables. Correlation results tabulated separately by cell type are given in **Tables A21 to A32**. While the association with percent uniquely mapped reads and number of slicing junctions was greatly reduced, cell specific expression components showed much stronger correlations with sample mean %GC content (**Figure A37**). Example genes that were highly correlated were identified and the relationship was visualized in **Figure A38**. Wilcoxon Rank Sum tests showed that %GC content did not significantly differ between vaccine groups.

3.3.4 Multiple time points to measure baseline expression

As for the complete sample set (**Figure A28**), at baseline, most variation was driven by cell type with distinct clusters for lymphocytes, dendritic cells/monocytes, and neutrophils (**Figure A39**). To evaluate factors that drive baseline variability (Day -28, -14, and 0), two ANOVA models were fit separately for the first two Principal Components. Models were fit across all cell types and separately by cell type. For both Principal Components, for the combined set, most of the total variance was explained by cell type, which was significant (**Tables A33 and A34**). The subject factor was significant as well but explained much less of the total variation. The study day factor was non-significant. Per-cell type ANOVA results showed that between-subject variability was significantly larger than within-subject variability for all six immune cell types and for both Principal Components (**Tables A35 and A46**). These trends were also evident when plotting factor distribution against the 1st Principal Component for each cell type (**Figure A40**). Genes with high baseline variability based on Pearson correlation with the 1st Principal Component are listed in **Tables A47 to A52**.

To evaluate the impact on fold change estimates, we compared genes with mean baseline fold changes $\geq$ 1.5 (up or down) when using all three baseline measures compared to one or two baseline measures in the group that received the SV-AS03 vaccine at day 1 and 3 post-vaccination (**Table A53**). The average sensitivity was 0.85 and 0.86 when using one or two baseline measures, respectively. That is 85-86% of genes identified when using three baseline measures were also identified when using one or two baseline measures. The average false discovery (proportion of non-matching genes) was 0.4 for the set based on a single baseline measure but was greatly reduced (to 0.18) when including a second baseline measure (**Table A53**). These results show that inclusion of additional baseline measures resulted in increasingly conservative and consistent fold change estimates reducing the impact of transient baseline signals.

3.3.5 Adjustment of global GC content bias

The observed strong association between expression pattern components and sample %GC content indicated that %GC plays a substantial role in modulating log_2 fragment counts per million. To evaluate the potential impact on the vaccine group effect (log fold change difference between SV-AS03 vs. SV-PBS group), negative binomial models were fit with and without sample-specific %GC added as a covariate and the LFCD was estimated. Under the assumption that most genes are not DE and GC content does not have a biasing influence on the vaccine group effect, the global center of the estimated fold change differences is expected to be zero. Any deviation from this pattern would indicate a systematic bias. **Figures A41 to A46** show the estimated log_2 fold change differences (SV-AS03 vs. SV-PBS group) plotted against gene-specific %GC content before and after correcting for % sample GC content (first two columns). The two columns to the right show MA plots with log_2 fold change differences (SV-AS03 vs. SV-PBS group) plotted against log_2 average counts with genes colored according to their GC content (in red: >%60) and (in blue: <%40). Generally, none or slight effects were observed (e.g. B-cells Day 1, monocytes Day 28). However, strong bias of log_2 fold change was observed for B-cells at Day 28 (**Figure A41**), Neutrophils at Day 1 (**Figure A44**), and NK-cells at Day 1 (**Figure A45**). In each case, inclusion of sample % GC content as a covariate corrected the bias reestablishing the center of fold change difference at zero and random pattern for high/low GC genes. Venn diagrams that summarize the overlap in the number of significant genes across post-vaccination days before and after GC correction are shown in **Figures A53 and A54**. Negative binomial dispersion trends of GC content-adjusted models by cell type and study visit combination and MA plots with significant genes highlighted are shown in **Figures A47 to A52**.

3.3.6 Response time trends of differential genes and gene clusters

Genes significantly differentially expressed for at least on post-vaccination day per cell type were evaluated for time, subject, and correlated gene patterns. **Figures A55 to A78** present heatmaps with

genes and subjects clustered by their fold change profile (based on uncentered Pearson correlation and complete linkage clustering). GC-adjusted log_2 fold change between vaccine groups for Days 1-28 are shown in **Figures A79 to A84**.

Tables A58 to A71 list gene clusters that were found to form significant clusters determined by multi-scale bootstrap resampling. Dendrograms with bootstrap probabilities and gene cluster highlights are summarized in **Figures A85 to A97**. Gene cluster results were integrated with baseline log_2 fold change heatmaps (**Figures A55 to A78**). To further investigate the relationship between genes over time, per-subject log_2 fold changes from baseline were plotted for each significant cluster (**Figures A101 to A105**).

3.3.7 Gene set enrichment results

Gene set enrichment analysis was carried out for each cell type study visit day combination using published gene sets outlined in **Table A73**. Except for MSigDB Immunologic Signature and Chemical/-Genetic Perturbation gene sets, typically less than half of significant genes were mappable to known gene sets (**Table A74**). Coverage for Chemical/Genetic Perturbations and Immunologic Signatures gene sets ranged between 82% and 100%. Taken together, this indicates that most of the differential genes in this study have biological functions (by responding to certain external stimuli) but a major proportion are not well characterized or annotated.

Significantly enriched gene sets for MSigDB Reactome and Immunologic Signatures sets are listed in supplemental tables S3 and S6 (not in this report). Significantly enriched gene sets for the other seven category types are given in **Tables A75 to A111**. Trends across cell types and time points for gene sets that were significantly enriched in at least two conditions are shown in **Figures A112 to A117**. KEGG pathway maps color-coded by expression fold change and fold change significance are shown in **Figures A118 to A137**.

3.3.8 Protein-protein interaction networks

Networks of experimental protein-protein interactions centered around significantly differentially expressed genes for each cell type and post-vaccination day are shown in **Figures A138 to A146** (nodes are colored by fold change between vaccine groups).

3.3.9 Gene responses correlated with changes in serum cytokine/chemokine, or antibody concentration

Regularized canonical correlation analysis was carried out to identify subsets of genes (Gene variables) that, based on their log fold change from baseline, maximize correlations with subsets of cytokine and

antibody responses (IMO variables). Summary statistics, including number of variables for each set, cross validation score, canonical correlations, and average explained percent variance for the first two canonical variates by cell type are listed in **Tables A112 to A117**. Canonical loadings and percent explained variance for each variable are given in **Tables A118 to A159**. Correlation circles summarizing associations between variable sets based on the first two canonical variates are shown in **Figures A148 to A168**. Graphical contrasts between vaccine groups are given in **Figures A169 to A177**. **Section 2.6.3.5** provides information on how to interpret correlations between variable based on their 2-dimensional placement in these graphs.

3.3.10 Gene responses that differentiate between seroprotection status

A regularized logistic regression model was fit for each cell type to identify combinations of Day 1 gene responses that best differentiate between seroprotection status. **Table A160** summarizes regularized logistic regression model statistics including the number of subjects by seroprotection status, as well as minimum cross-validated mean misclassification error (MME) for each model.

3.4 Flow cytometric immune cell phenotyping and activation results

Spider plots that summarize median fold changes in the percentage of live cells as well as cell concentration (#cells/mL) for each immune cell type are shown in **Figures A212 to A213**, respectively. Corresponding median values are listed in **Tables A161 to A162**.

Detector settings for activation marker detection changed daily during the study. Although data were corrected for these changes, results presented below should be treated with caution. The data is provided for completeness so that they can be confirmed in the context of future cell activation studies.

Of the 140 samples assayed (20 subjects and 7 time points), complete measurements, i.e. measurements for all 3 markers (CD134, CD69, CD86) and 6 cell types, were obtained for 129 samples: 7 samples were missing all measurements and 4 samples were missing some of the measurements. For 52 of the samples, experimental issues including detector changes were recorded.

Boxplots of percentage of cell activation for each marker and cell type are shown in **Figures A178 to A180**. Non-metric multidimensional scaling biplots are given in **Figure A181**. Based on these plots and in combination with reported experimental issues, 35 measurements obtained for 14 samples were flagged as outliers and were removed from downstream analyses.

Correlation between detector settings and percent cell activation before and after normalization was evaluated for each marker and cell type combination using scatter plots as well as Pearson and Spear-

man correlation metrics. **Figures A182 to A193** show results for CD69, CD86, and CD134, colored by vaccine group or study visit day. CD69 showed the strongest linear (Pearson) correlation between detector settings and percentage of cell activation (T-cells and NK-cells: 0.47, B-cells: 0.41, Dendritic cells: 0.38, monocytes: 0.19, and neutrophils: 0.1; **Figures A182 to A183**). Except for neutrophils, all CD69 correlations were statistically significant. Observed linear correlations for CD134 were weaker and, if a trend was observed, were negative, i.e. the percentage of cells expressing CD134 decreased with increasing detector settings (**Figures A190 to A191**). Significant CD134 correlations were observed for T-cells (-0.26), monocytes (-0.23), and dendritic cells (-0.27). Non-significant correlations were reported for neutrophils (0.04), B-cells (-0.05), and NK-cells (-0.14). CD86 did not show any significant linear associations between detector settings and percent expressing cells (**Figures A186 to A187**). Visual inspection of CD69 and CD134 scatterplots before and after normalization did not show strong detector setting-specific groupings by vaccine group or study visit day. In all these cases, Wilcoxon Rank-Sum test results to evaluate distribution shifts between normalization scaling factors for the two vaccine groups were not statistically significant (**Figures A182 to A185** and **Figures A190 to A193**). The same was true when comparing scaling factor distribution between study visits using a Kruskal-Wallis Rank-Sum test. For the following cell type combinations, normalized values were used for downstream analyses: T-cells (CD69 and CD134), monocytes (CD69 and CD134), dendritic cells (CD69 and CD134), NK-cells (CD69 and CD134), B-cells (CD69).

Figures

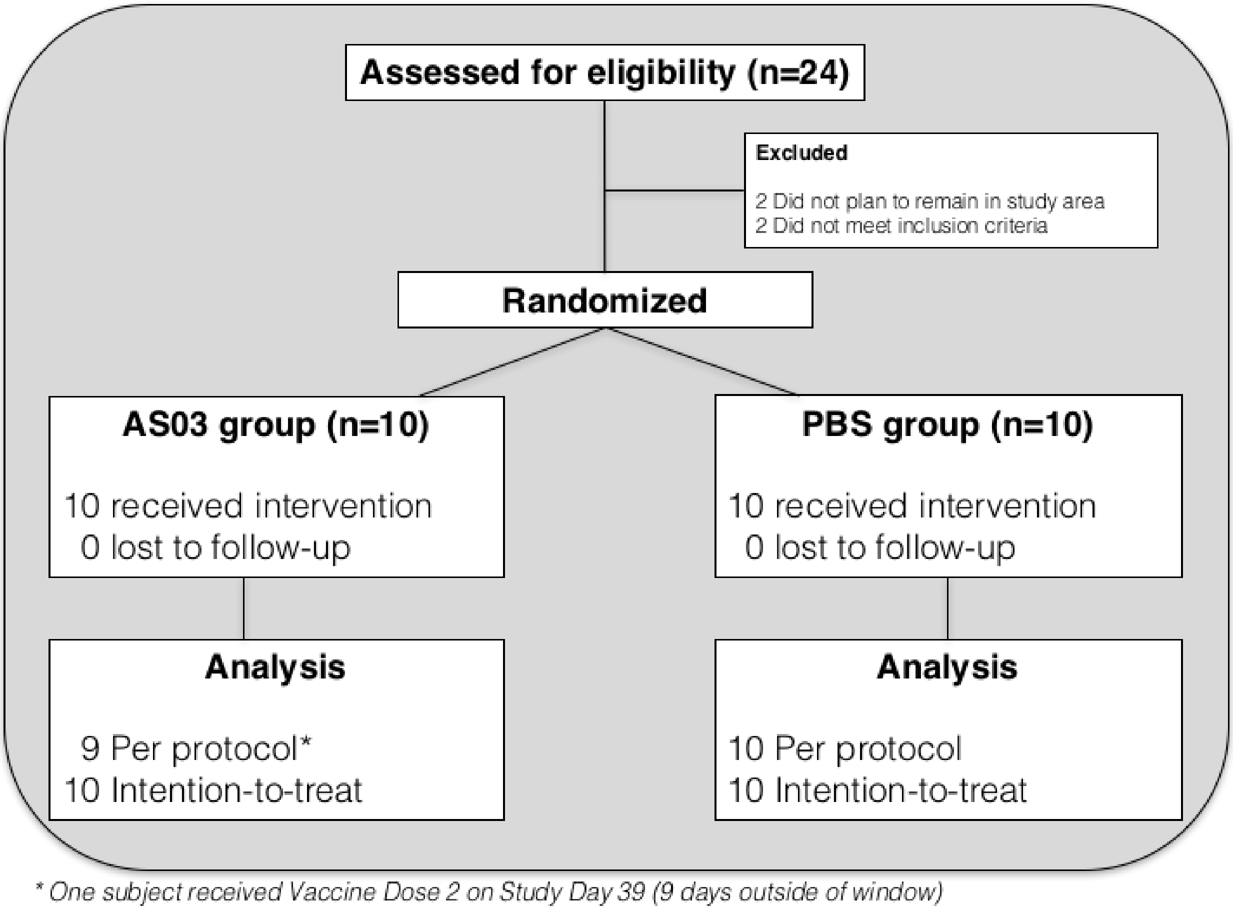

Figure A1: Consort diagram outlining study group enrollment and randomization.

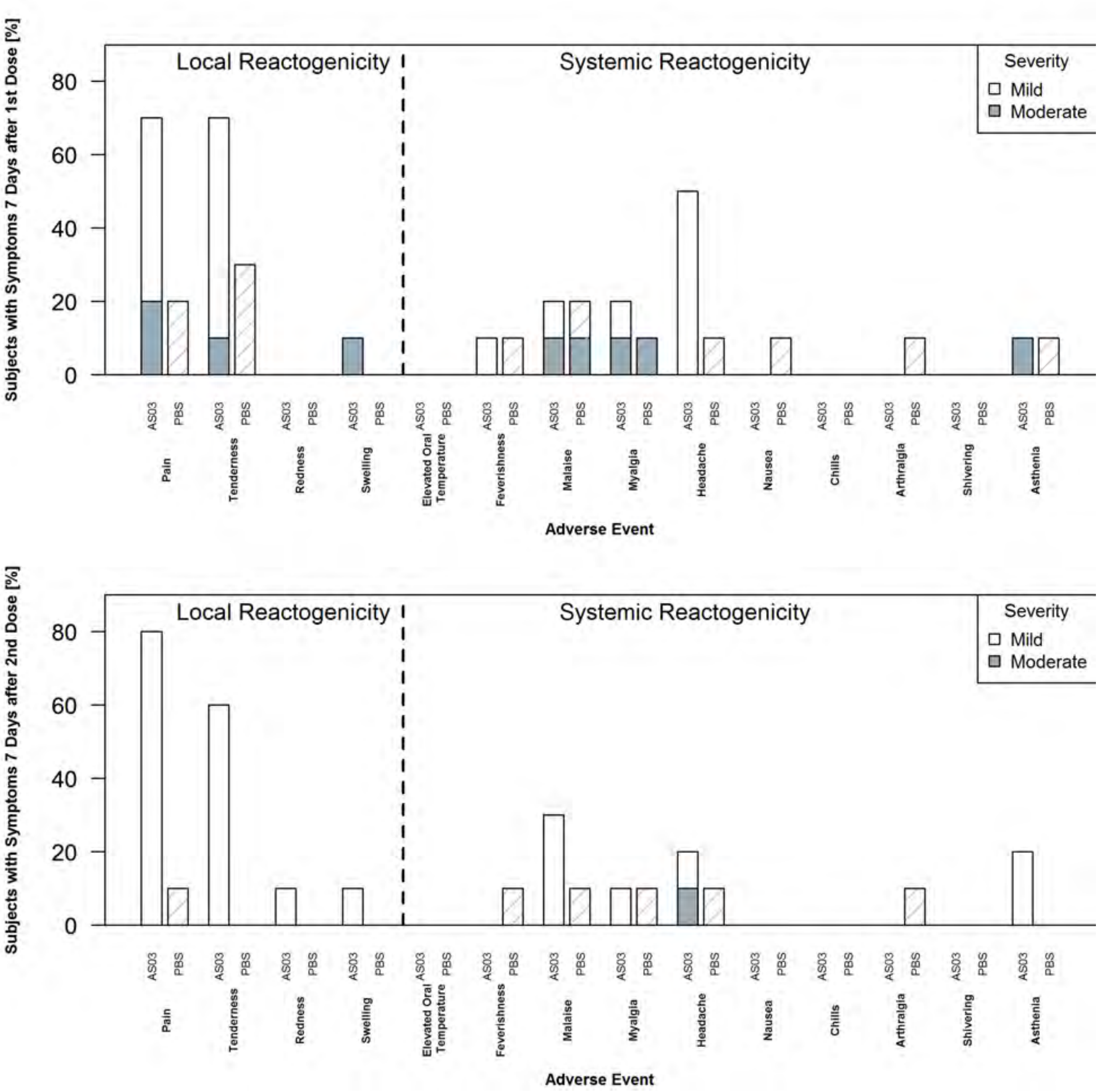

Figure A2: Local and systemic reactogenicity by severity and study arm.

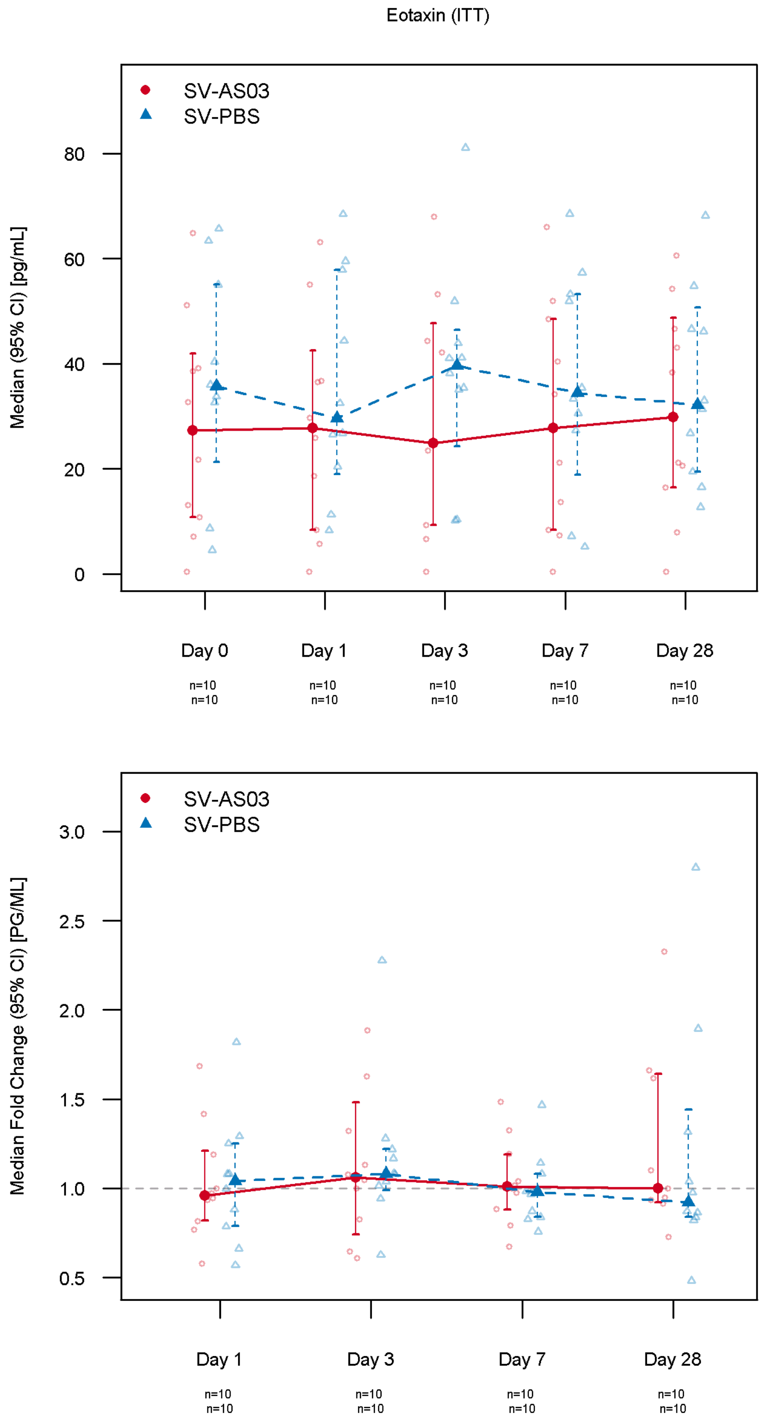

Figure A3: Eotaxin-1 (CCL11) Median Concentration and Baseline Fold Change.

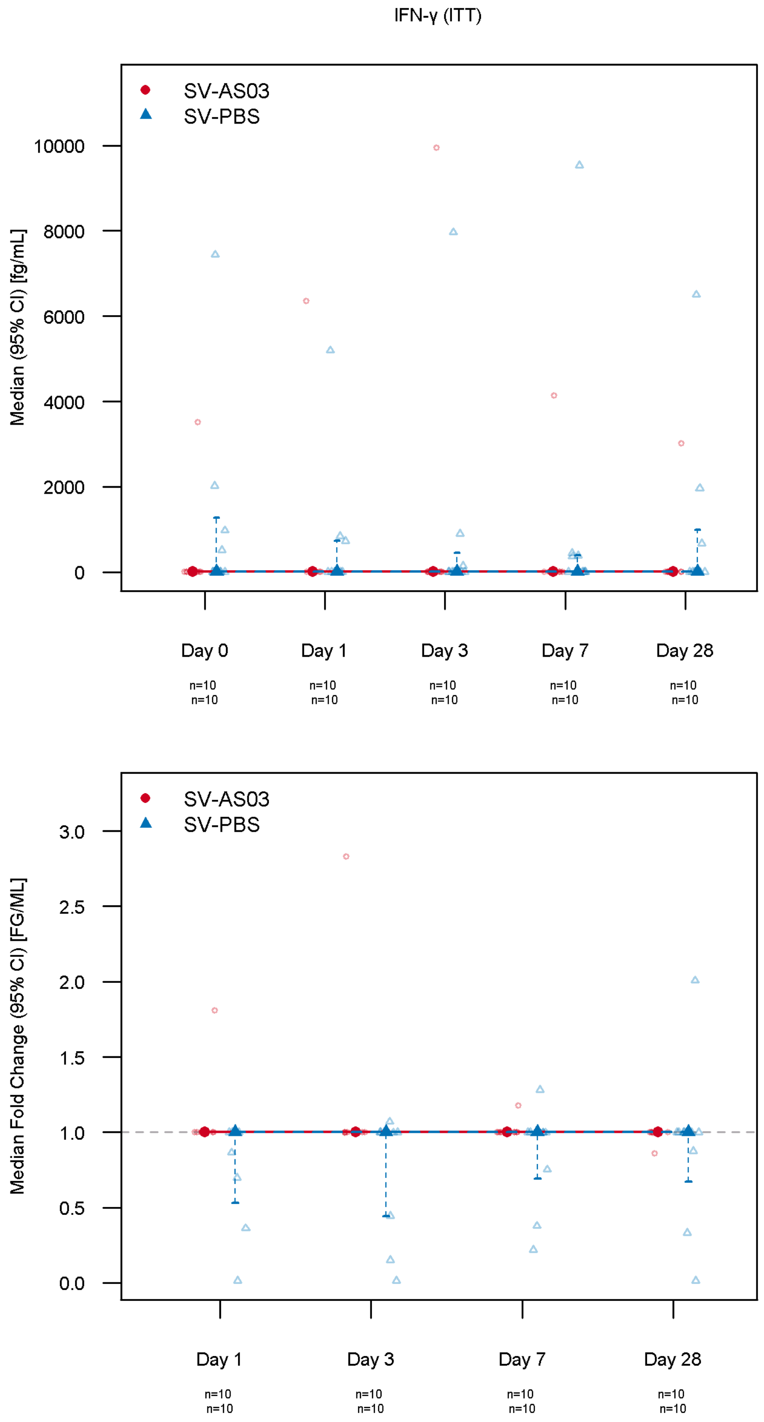

Figure A4: Interferon Gamma (IFN-γ) Median Concentration and Baseline Fold Change.

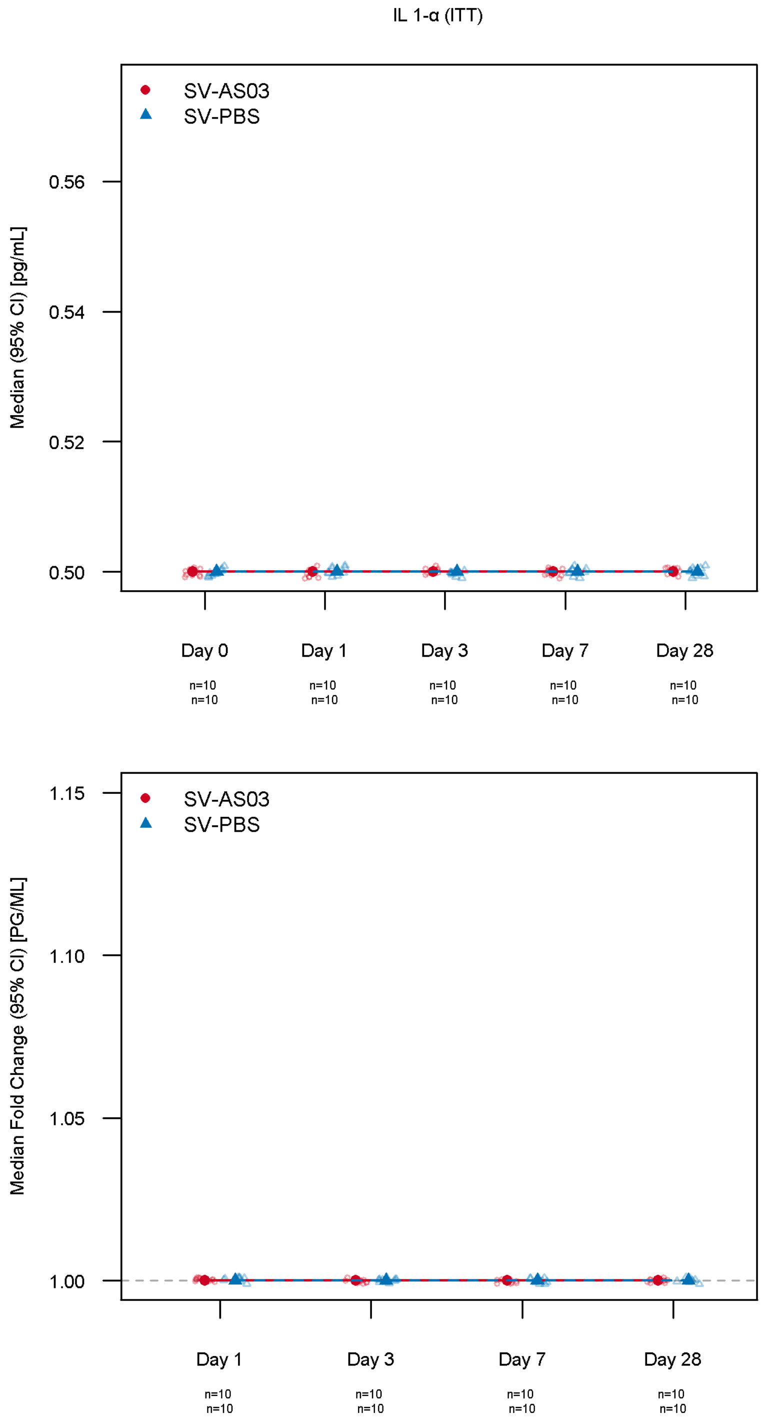

Figure A5: Interleukin-1 Alpha (IL-1α) Median Concentration and Baseline Fold Change.

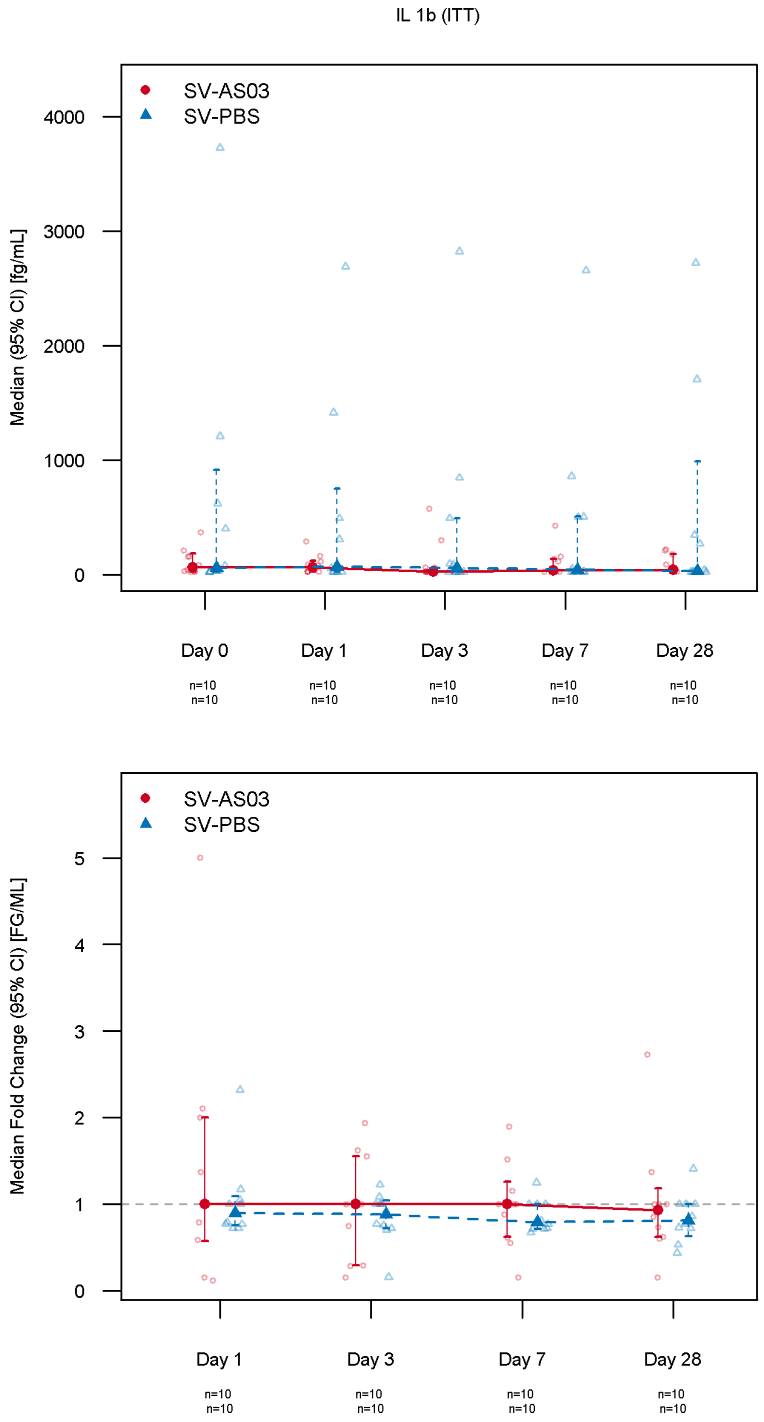

Figure A6: Interleukin-1 Beta (IL-1β) Median Concentration and Baseline Fold Change.

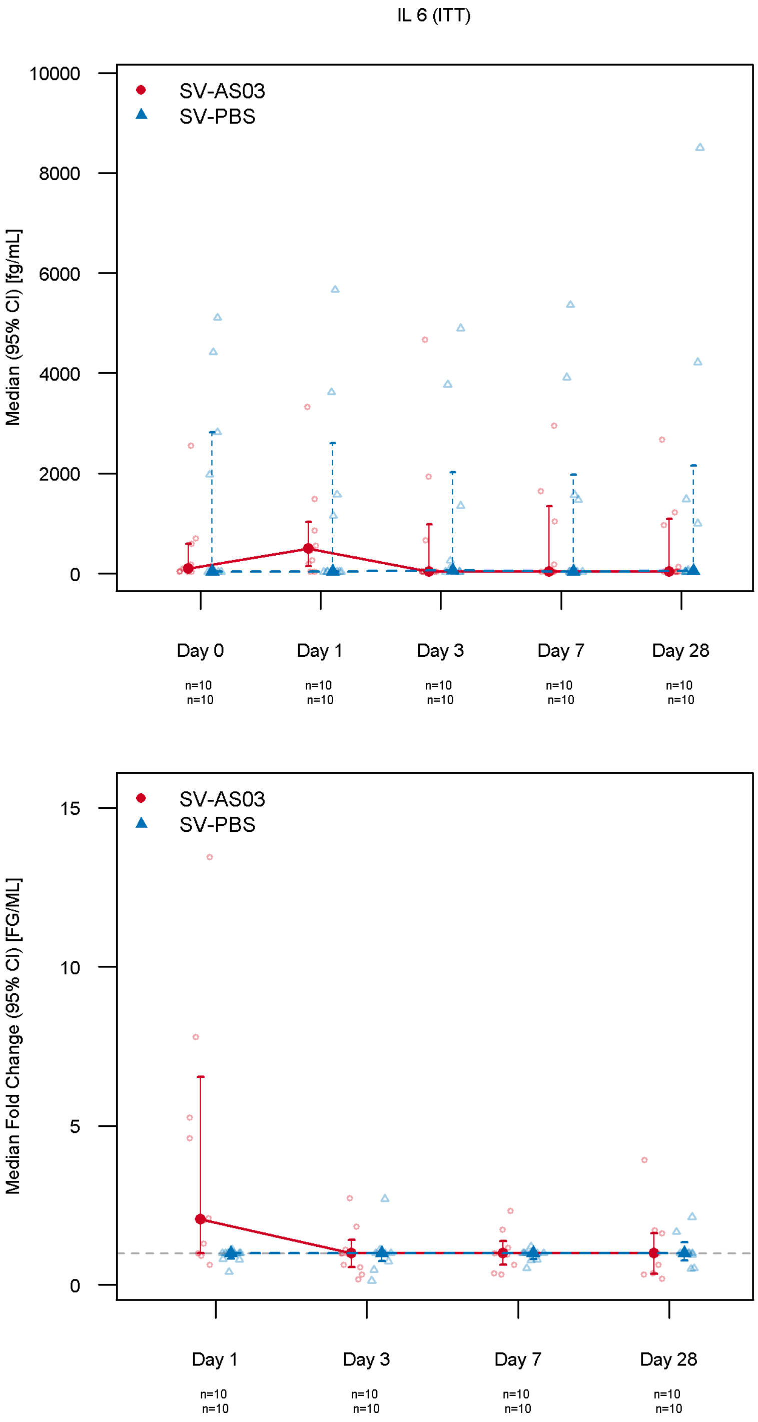

Figure A7: Interleukin-6 (IL-6) Median Concentration and Baseline Fold Change.

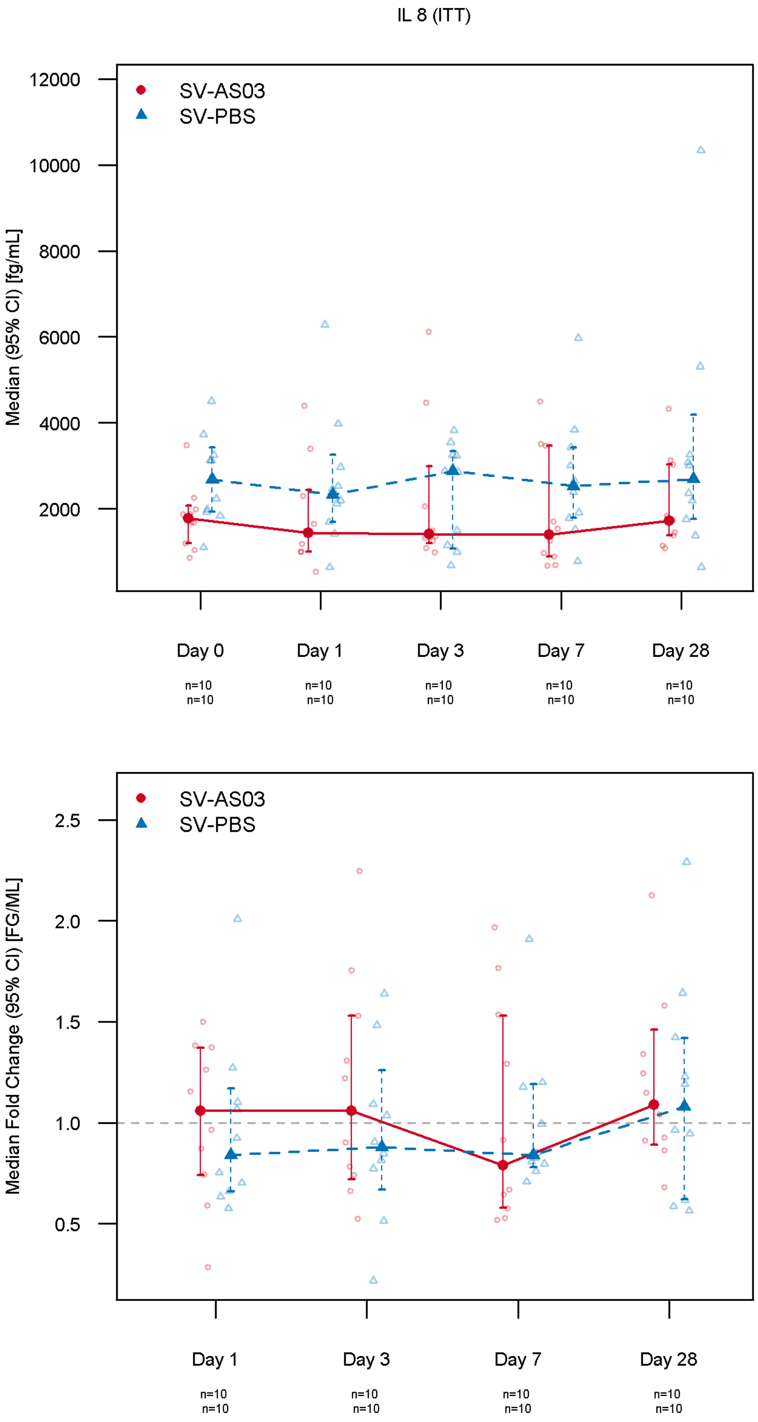

Figure A8: Interleukin-8 (IL-8) Median Concentration and Baseline Fold Change.

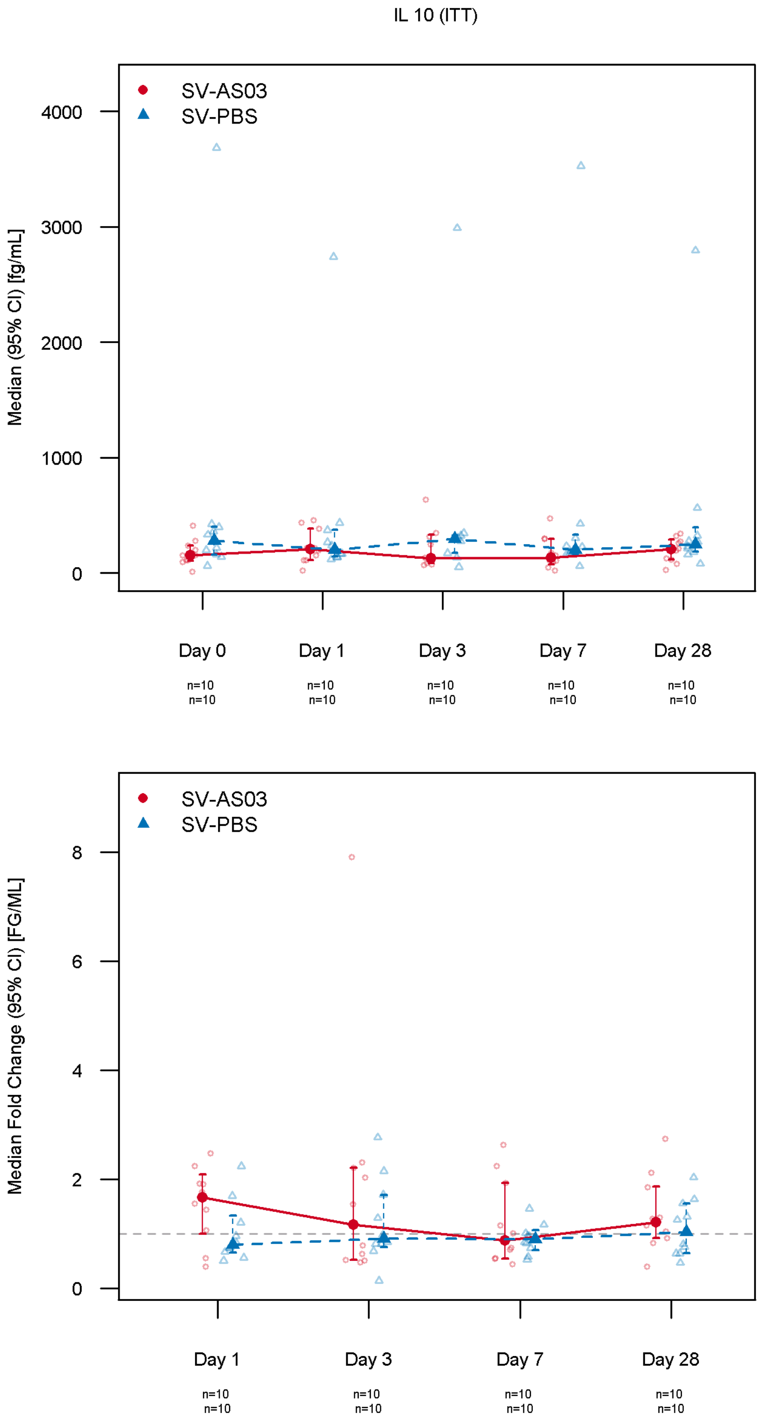

Figure A9: Interleukin-10 (IL-10) Median Concentration and Baseline Fold Change.

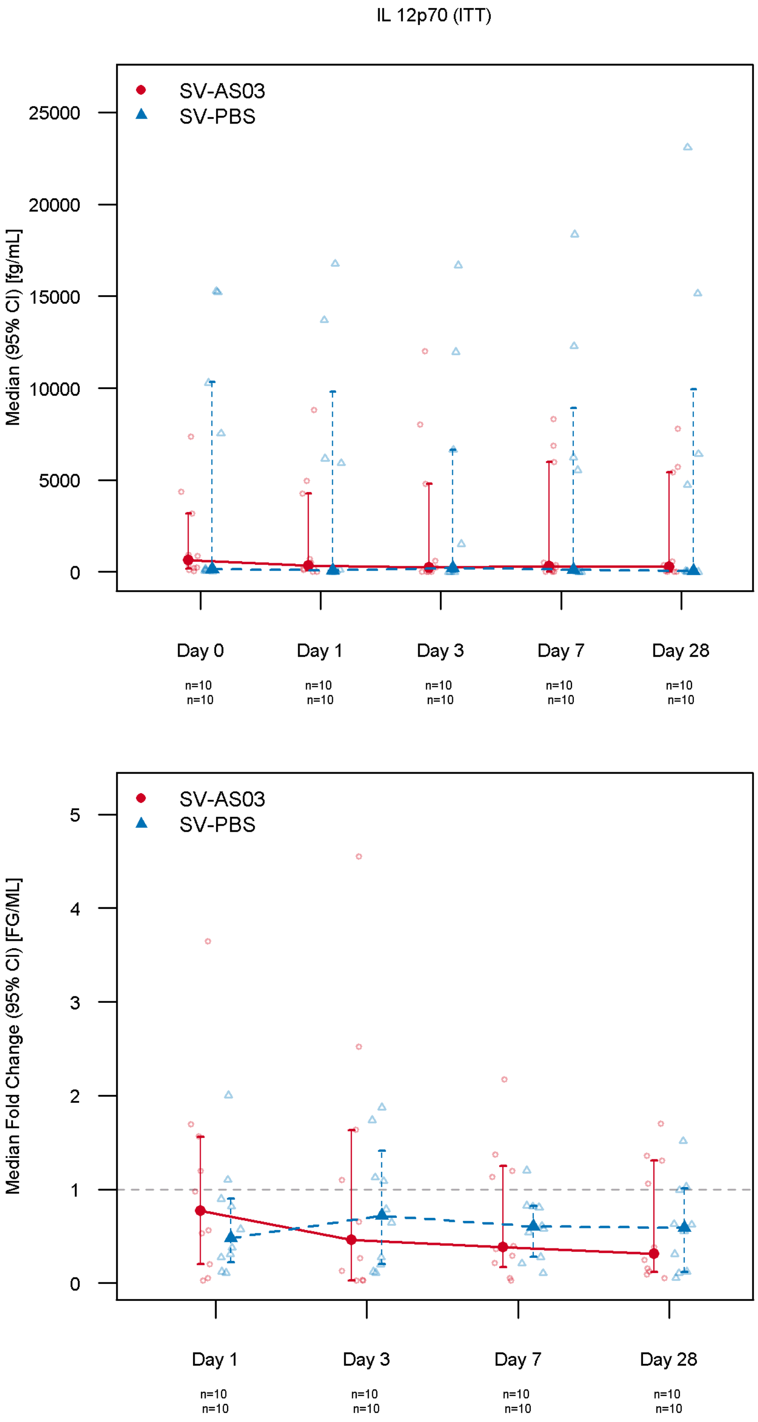

Figure A10: Interleukin-12 p70 (IL-12p70) Median Concentration and Baseline Fold Change.

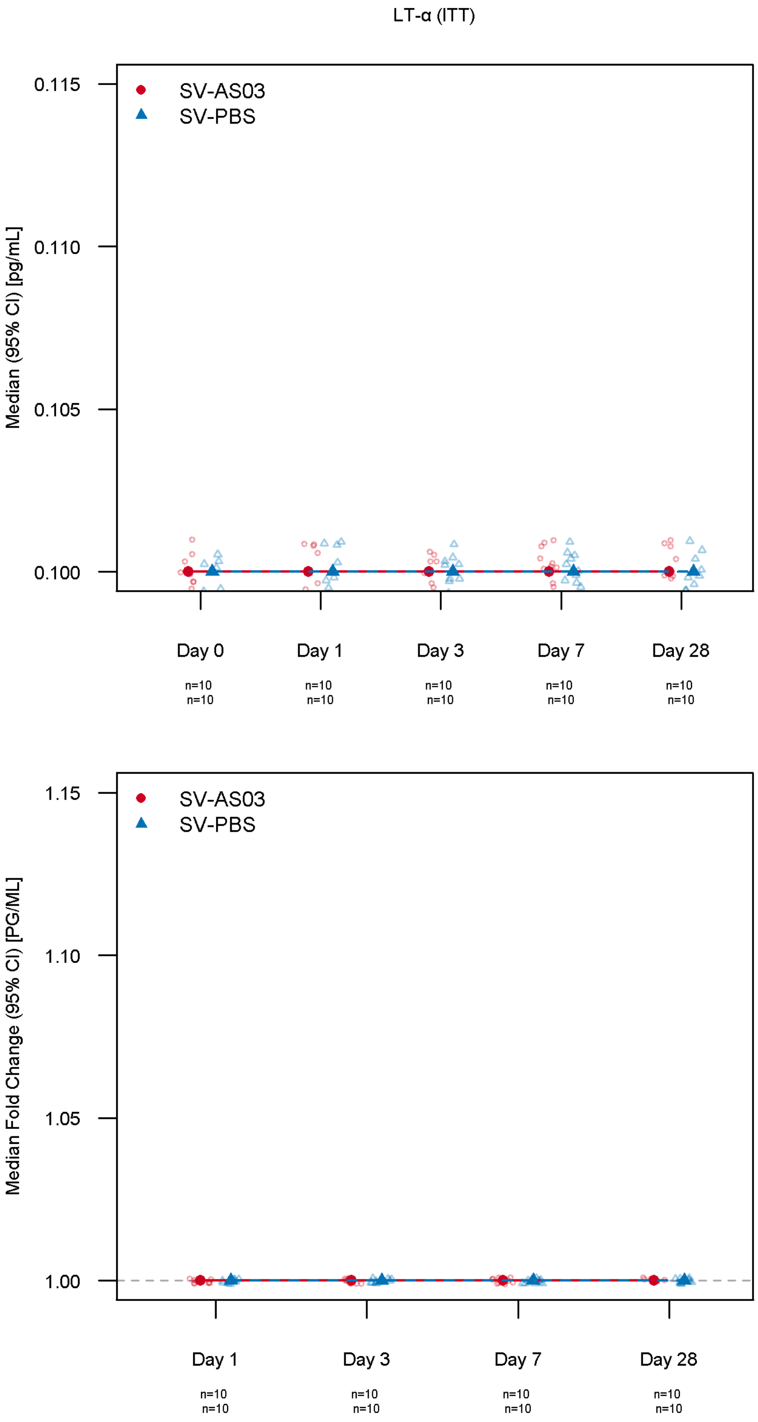

Figure A11: Lymphotoxin-alpha (LT-α) Median Concentration and Baseline Fold Change.

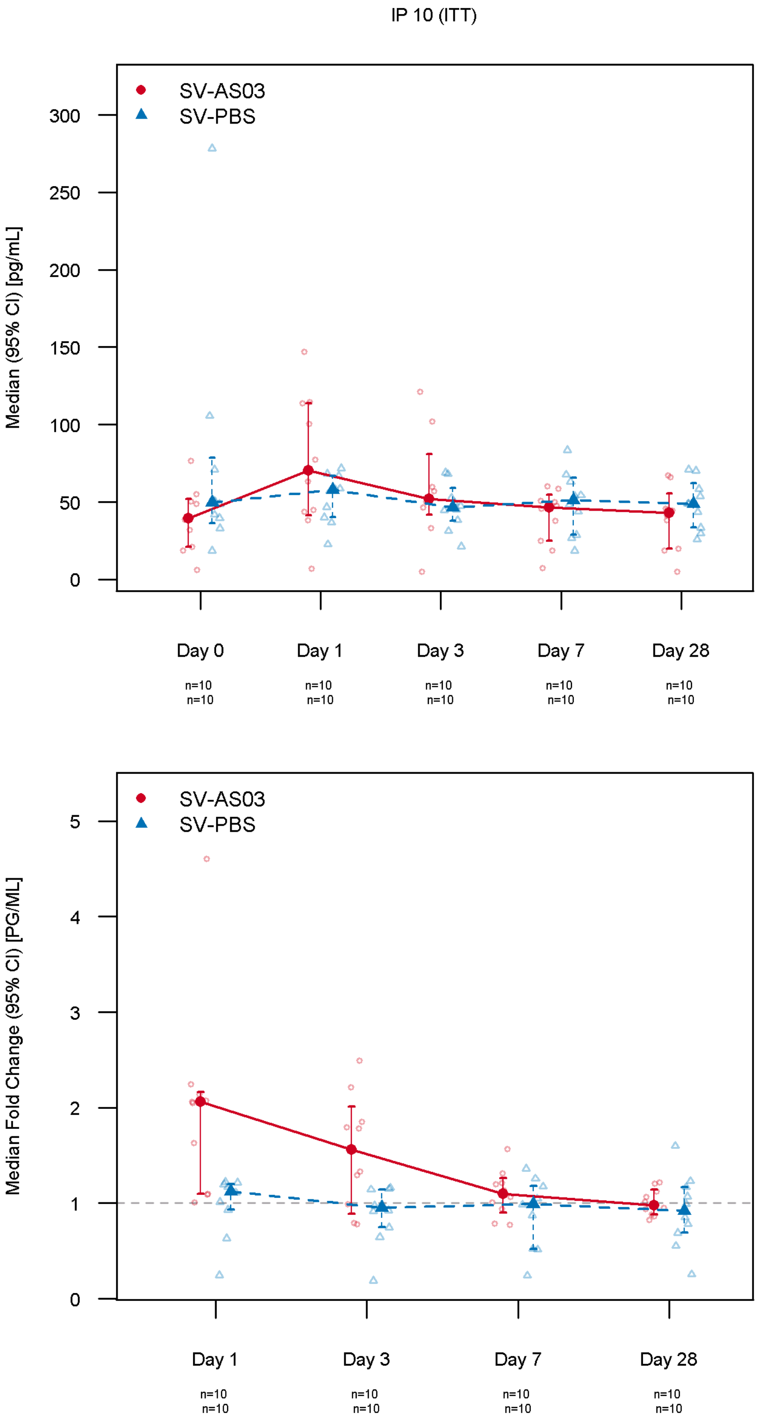

Figure A12: Interferon Gamma-Induced Protein 10 (IP-10) Median Concentration and Baseline Fold Change.

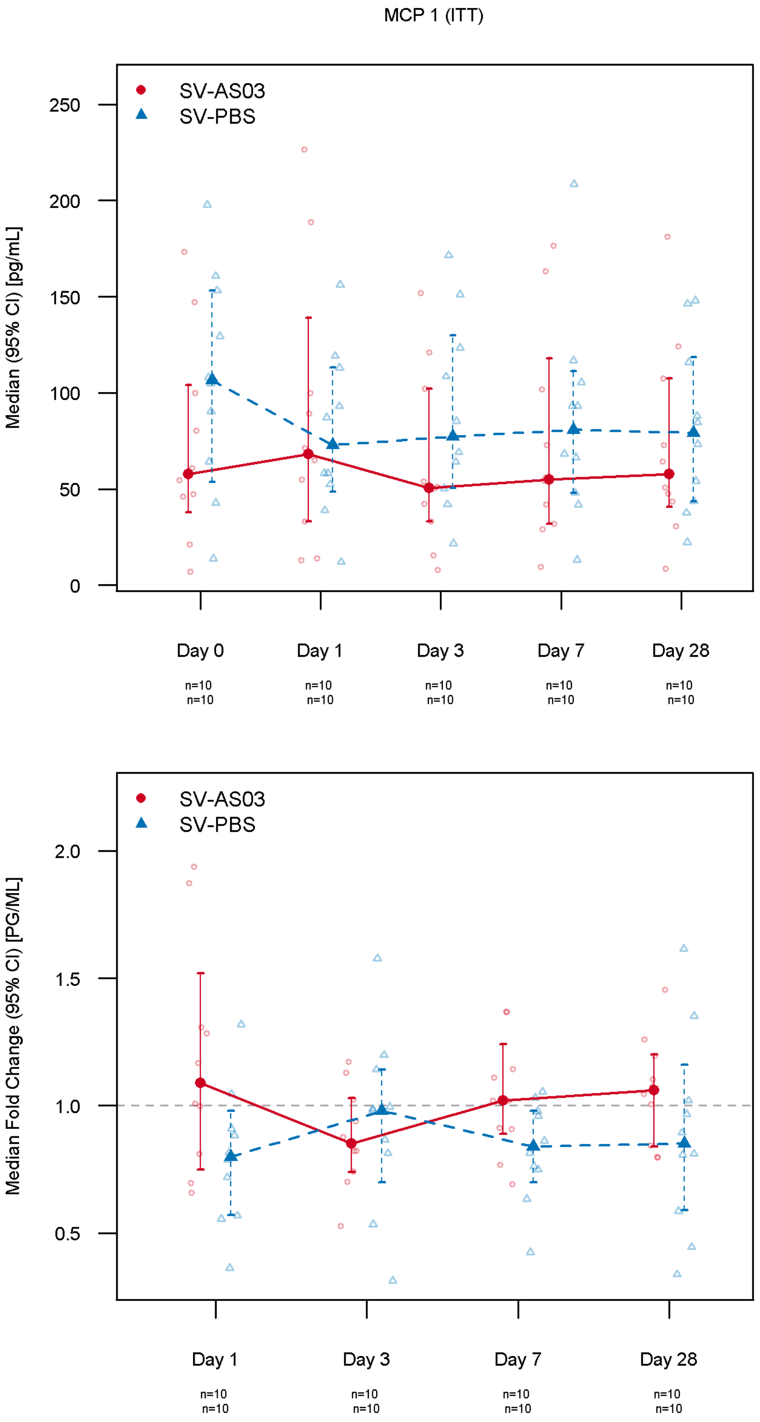

Figure A13: Monocyte Chemoattractant Protein-1 (MCP-1) Median Concentration and Baseline Fold Change.

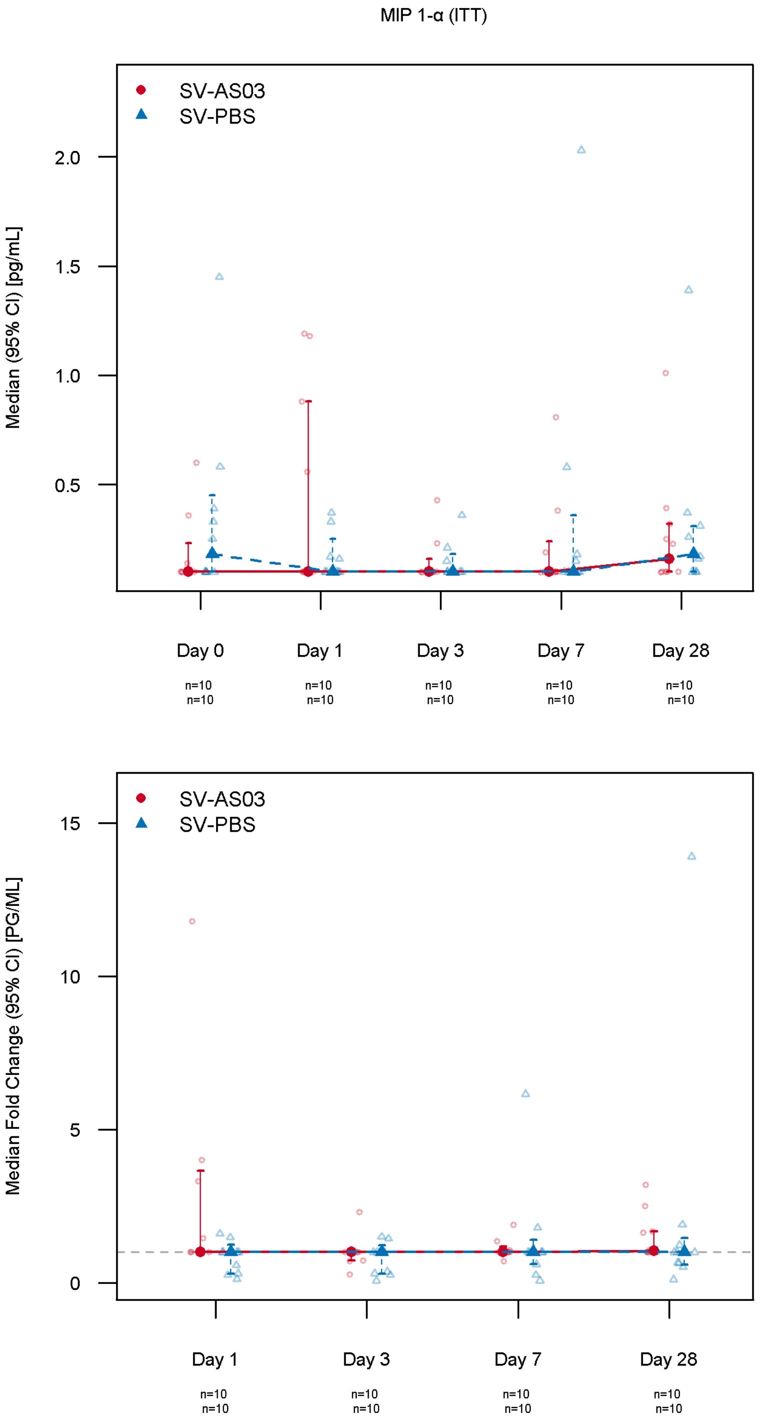

Figure A14: Macrophage Inflammatory Protein (MIP-1α) Median Concentration and Baseline Fold Change.

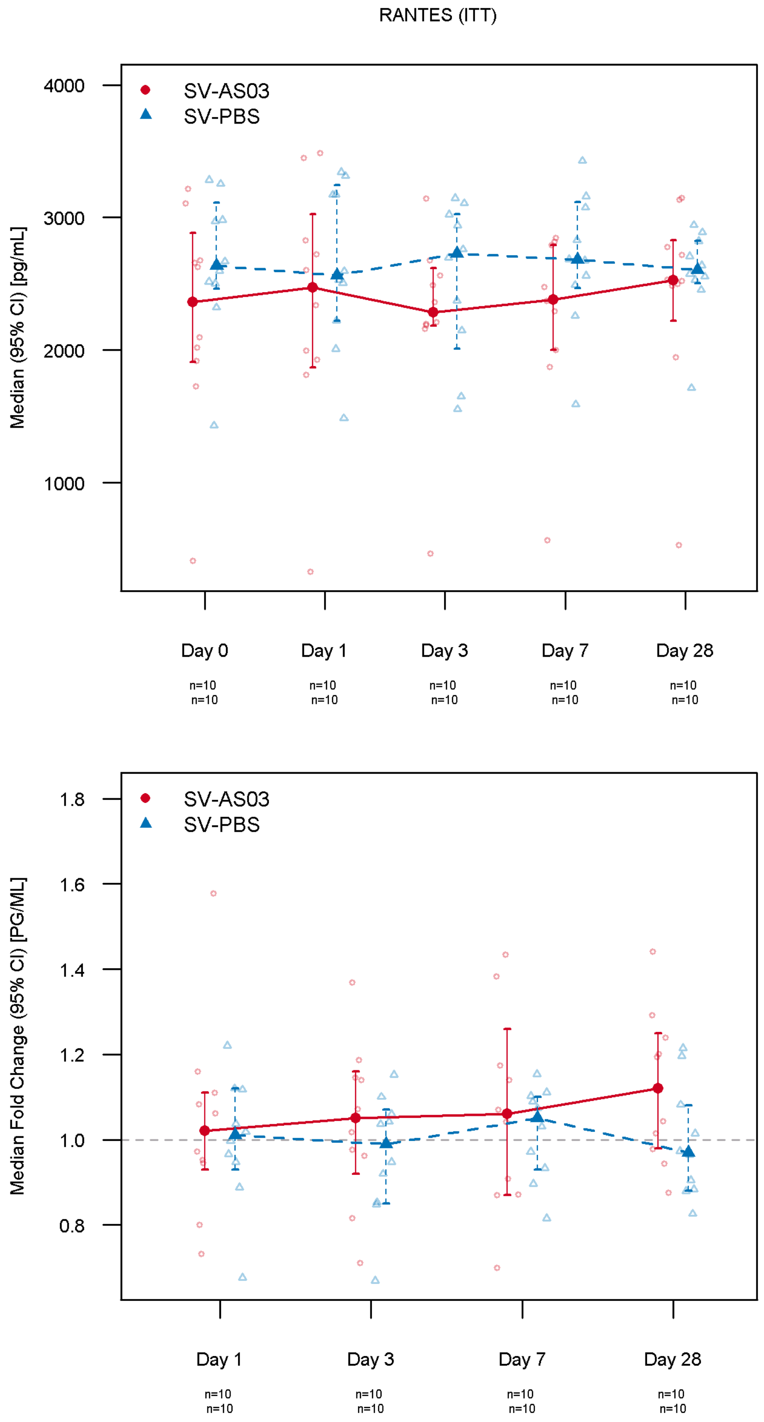

Figure A15: Chemokine (C-C motif) Ligand 5 (RANTES) Median Concentration and Baseline Fold Change.

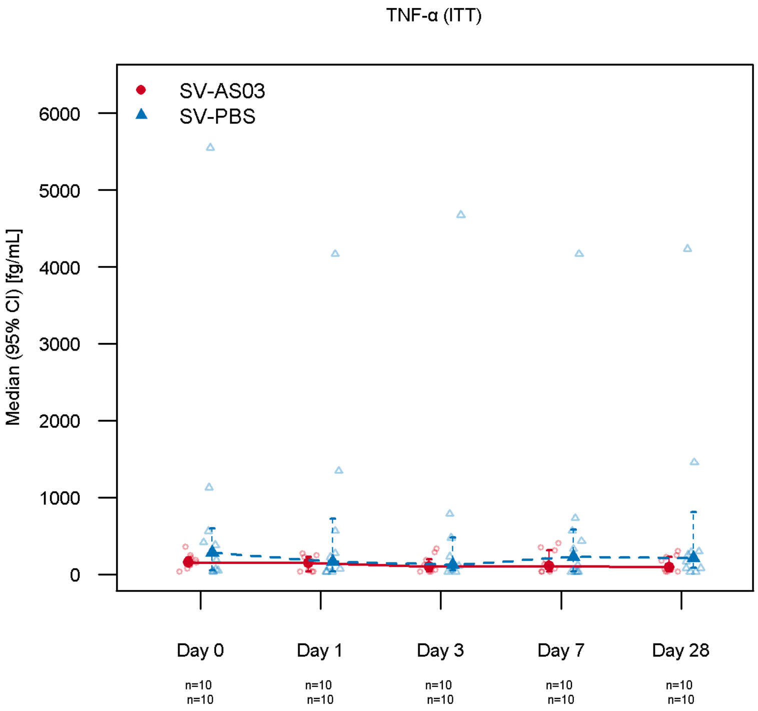

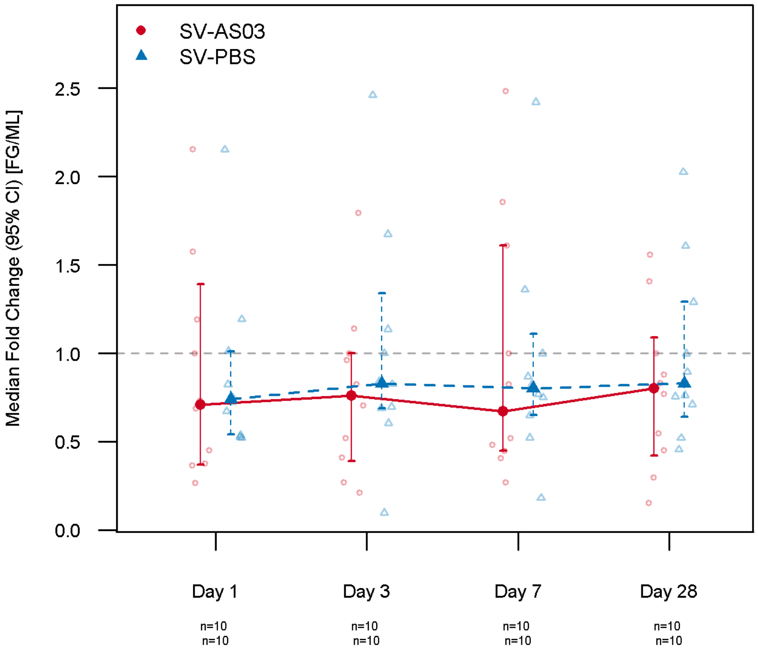

Figure A16: Tumor Necrosis Factors (TNF) Median Concentration and Baseline Fold Change.

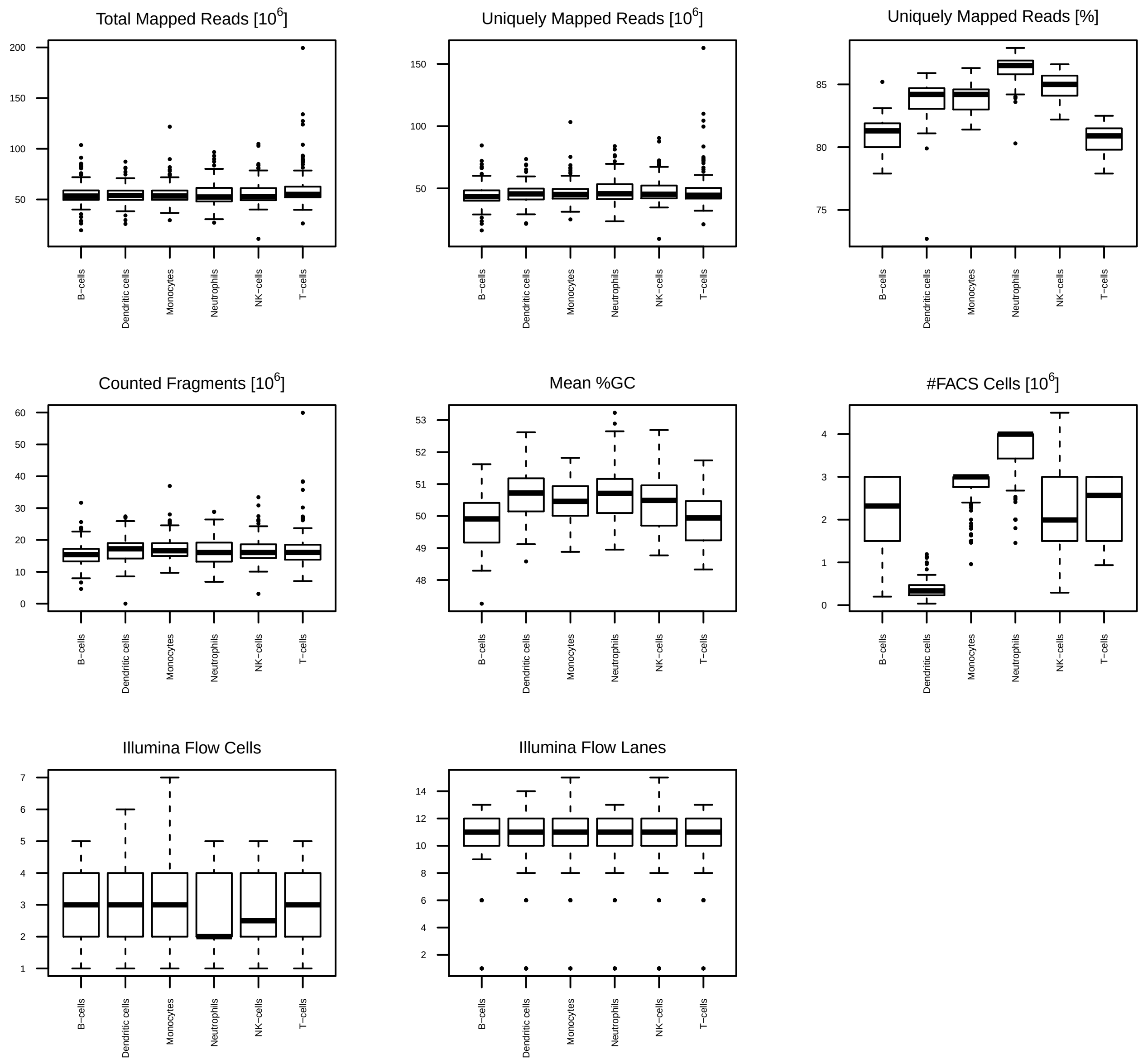

Figure A17: Boxplots of technical variables by cell type (RNA-Seq).

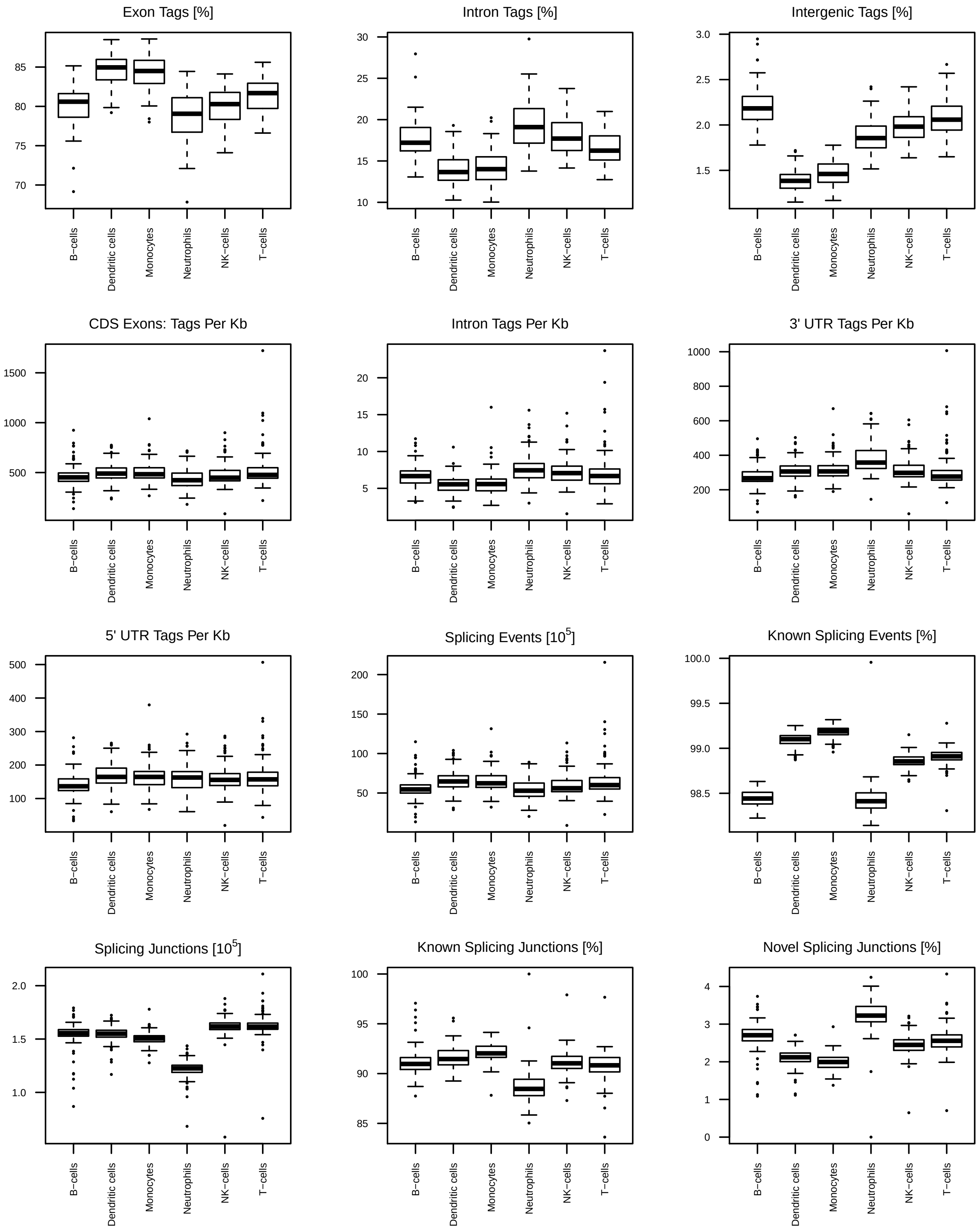

Figure A18: Boxplots of gene model variables by cell type (RNA-Seq).

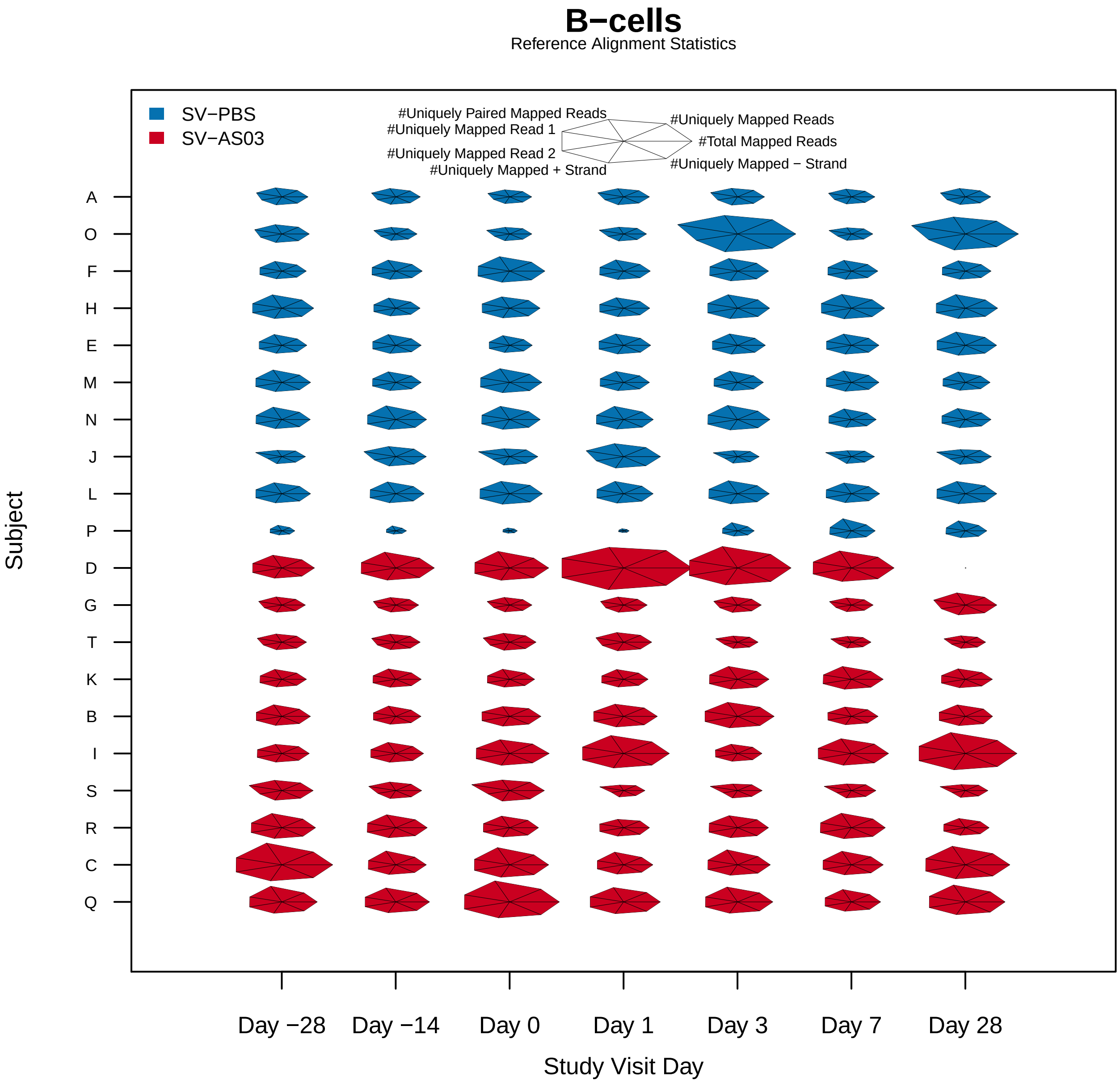

Figure A19: Starplots of human genome reference alignment statistics (RNA-Seq, B-cells).

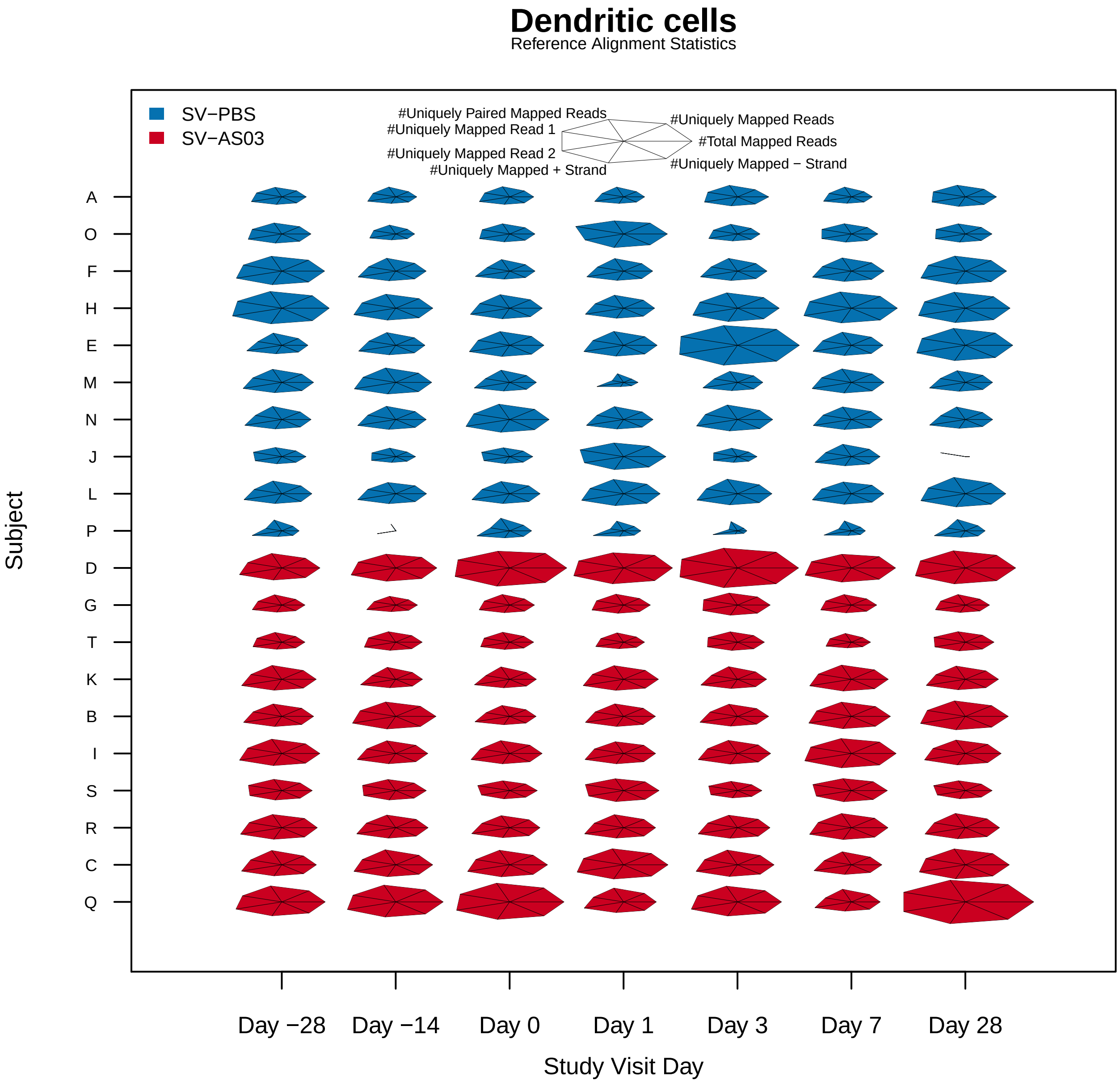

Figure A20: Starplots of human genome reference alignment statistics (RNA-Seq, Dendritic cells).

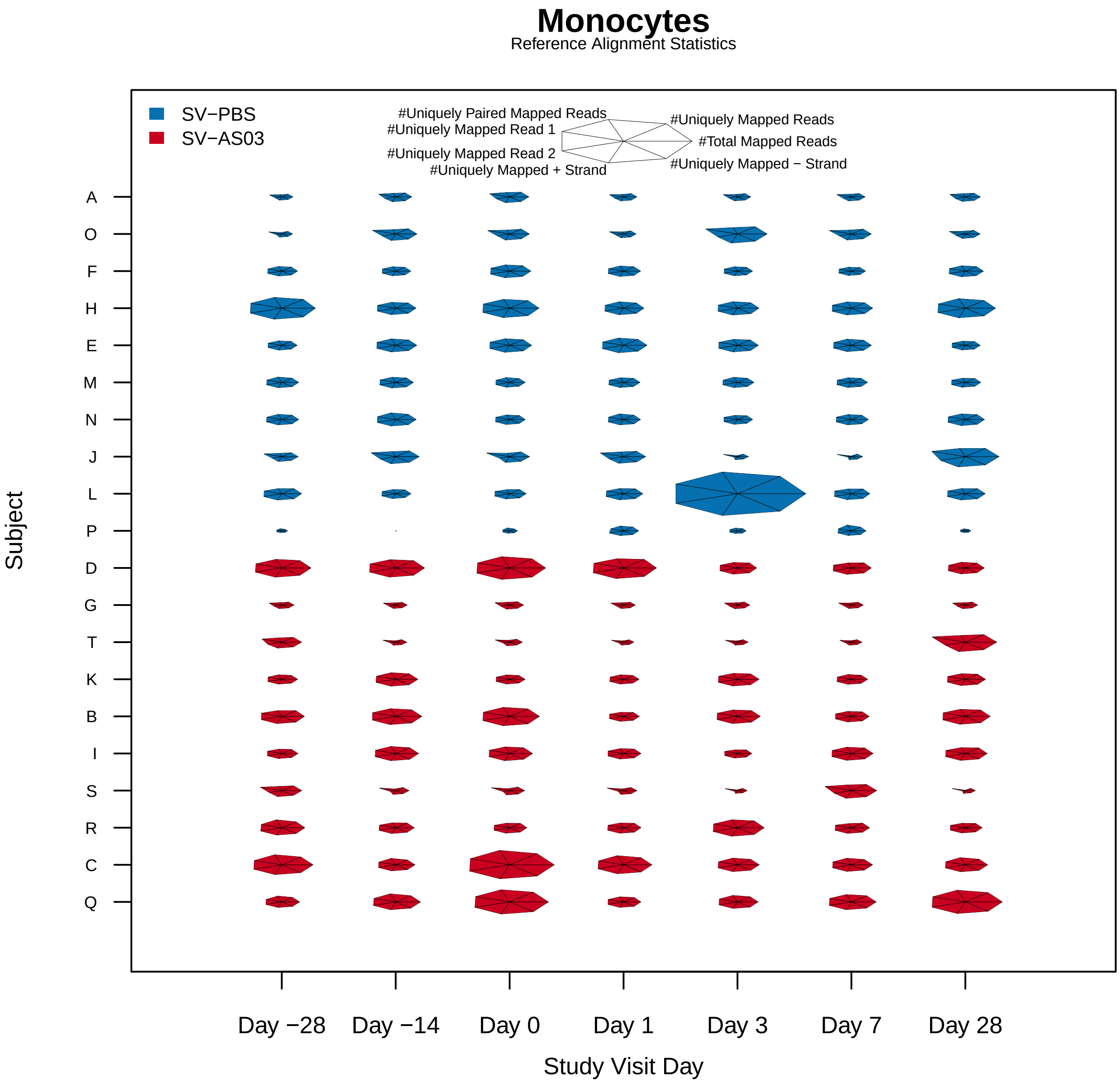

Figure A21: Starplots of human genome reference alignment statistics (RNA-Seq, Monocytes).

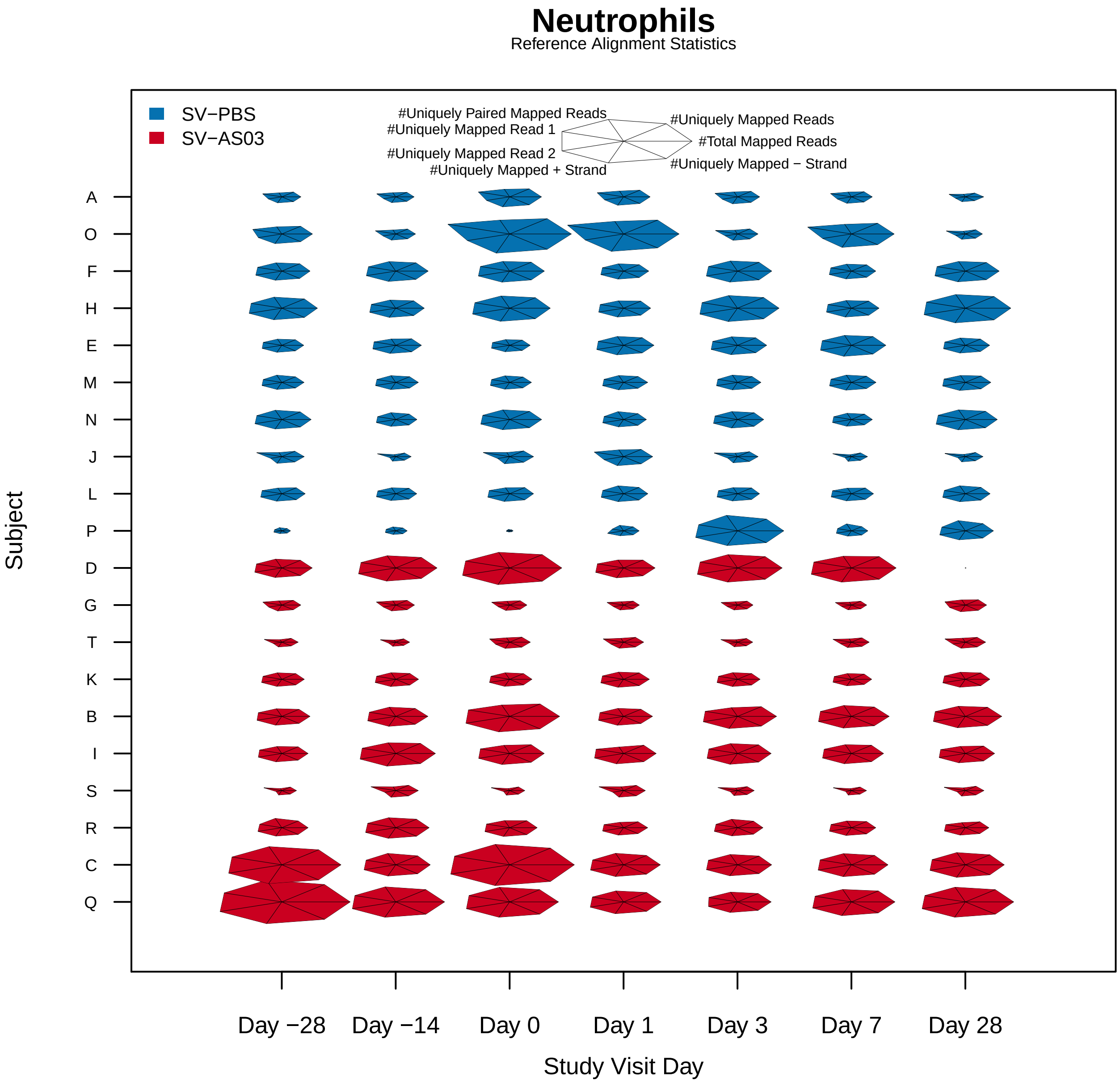

Figure A22: Starplots of human genome reference alignment statistics (RNA-Seq, Neutrophils).

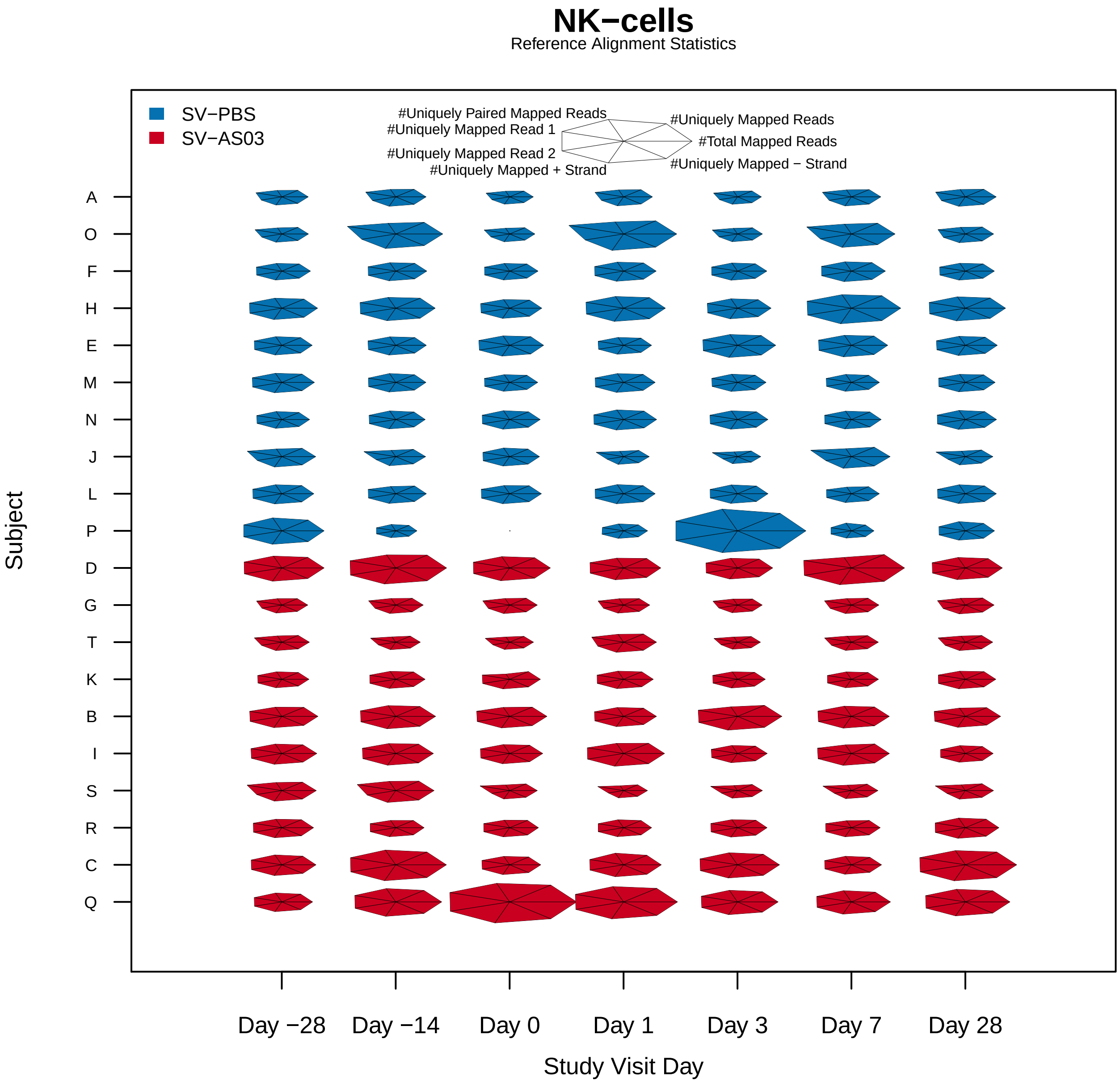

Figure A23: Starplots of human genome reference alignment statistics (RNA-Seq, NK-cells).

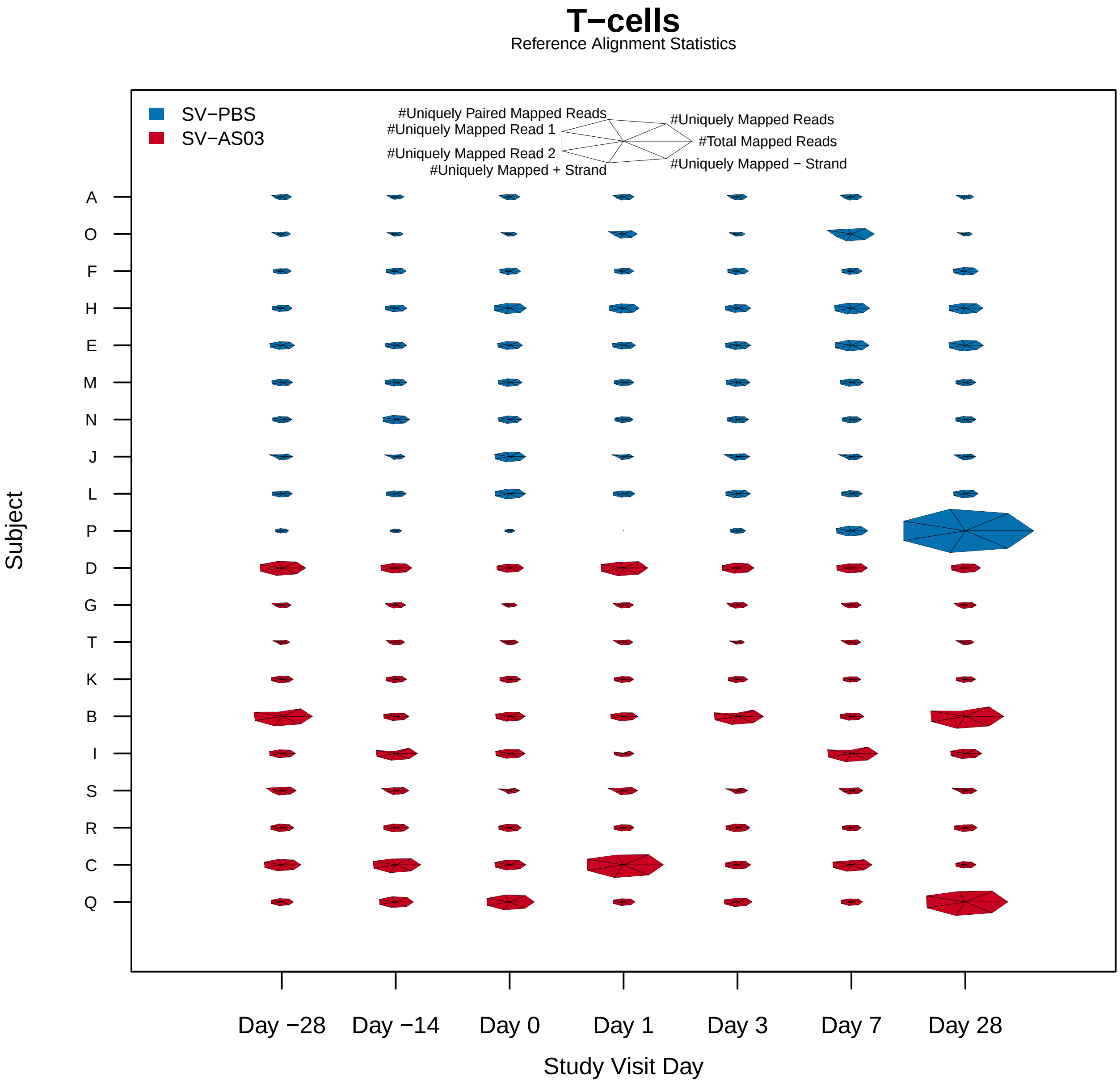

Figure A24: Starplots of human genome reference alignment statistics (RNA-Seq, T-cells).

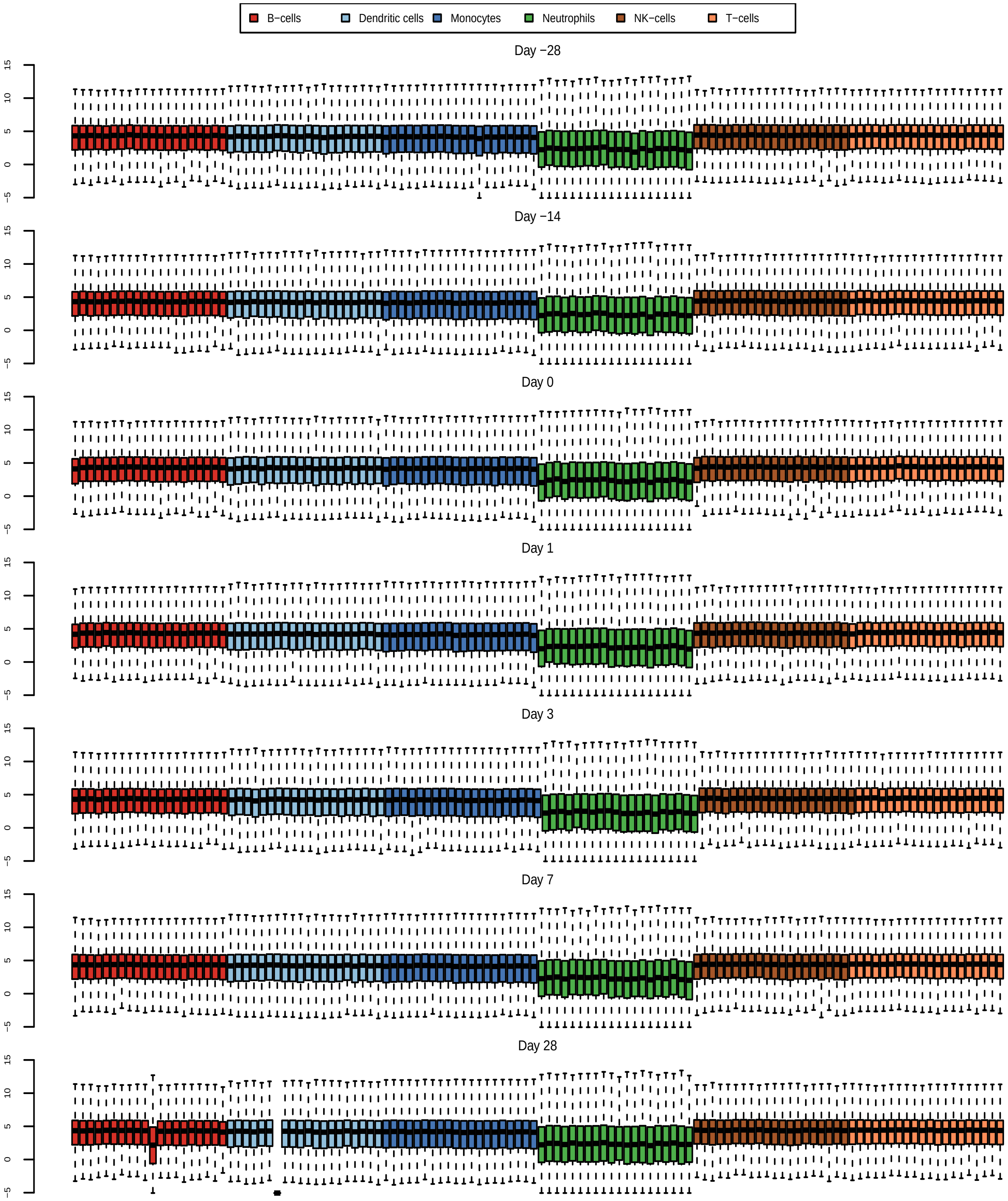

Figure A25: Boxplots of log_2 counts per million before TMM normalization. Boxplot outliers are omitted for clarity. Ordering by day was chosen to optimize visual representation.

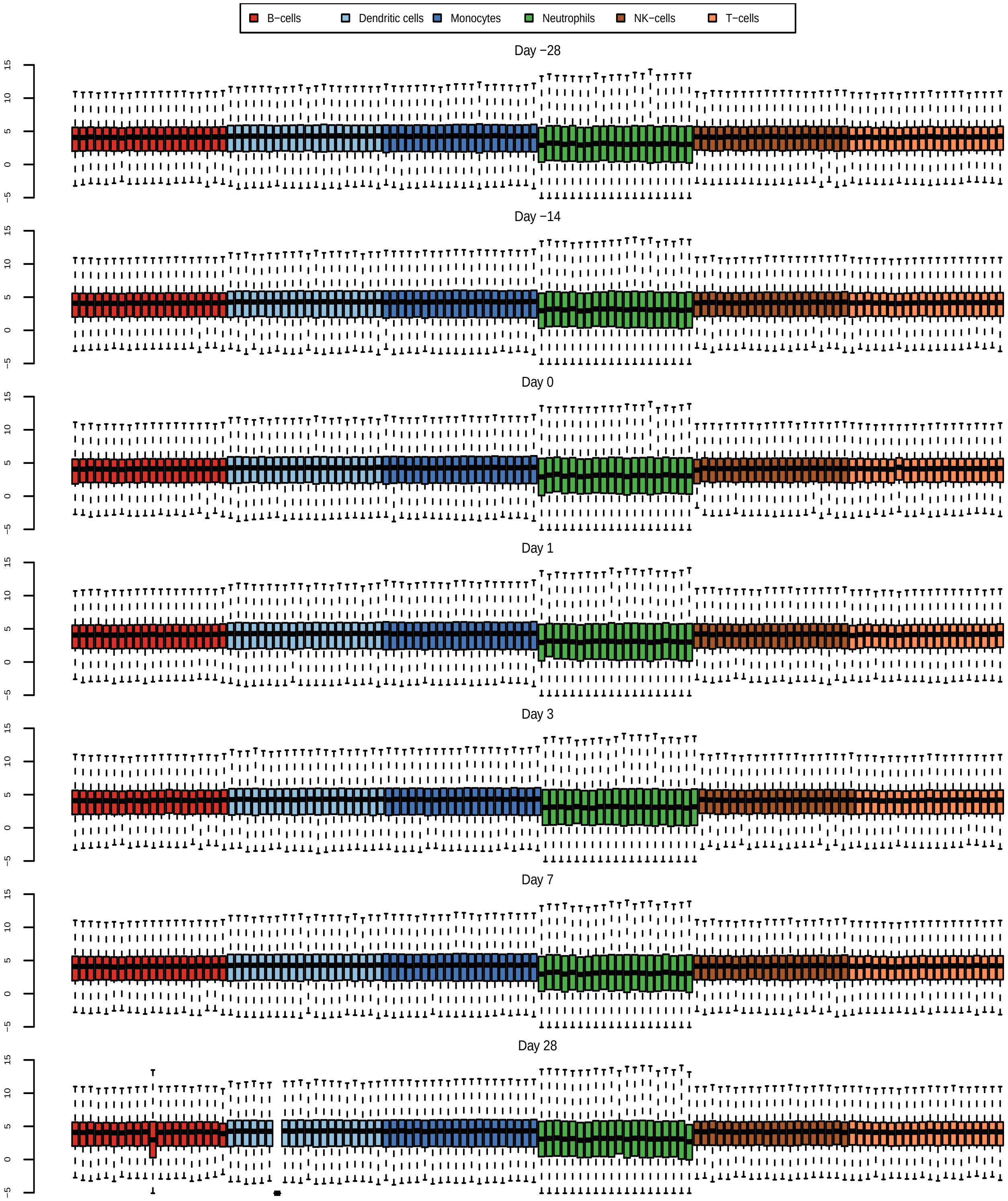

Figure A26: Boxplots of log_2 counts per million after TMM normalization. Boxplo outliers are omitted for clarity. Ordering by day was chosen to optimize visual representation (RNA-Seq).

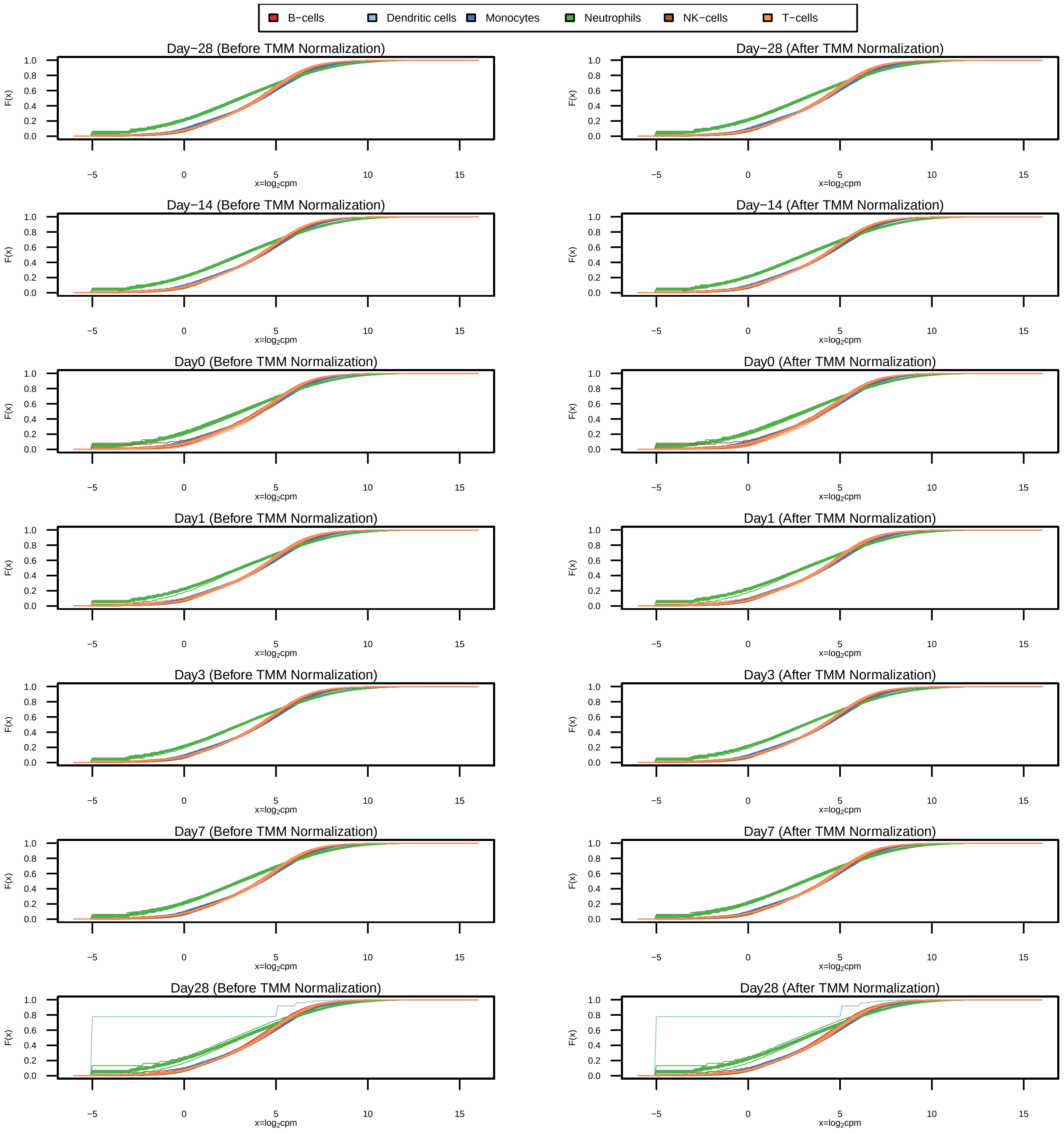

Figure A27: Empirical cumulative distribution function plots of log_2 counts per million before and after TMM normalization (RNA-Seq).

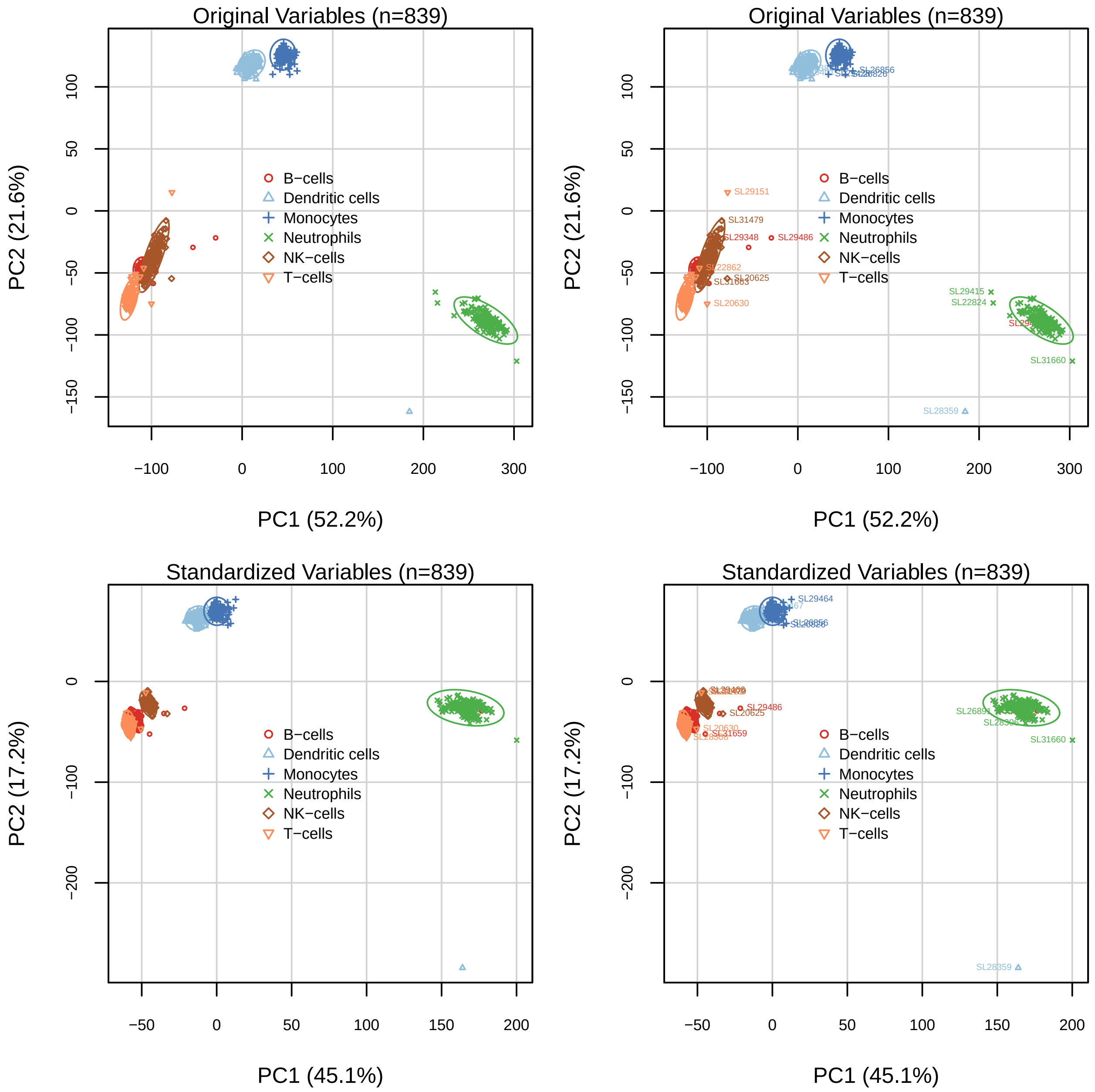

Figure A28: PCA biplots (RNA-Seq, n=839). 99.6% confidence ellipses are drawn for each cell type. The right panels are identical to the left panels except that they show dataset labels for the three most outlying samples per cell type based on maximum Mahalanobis distance.

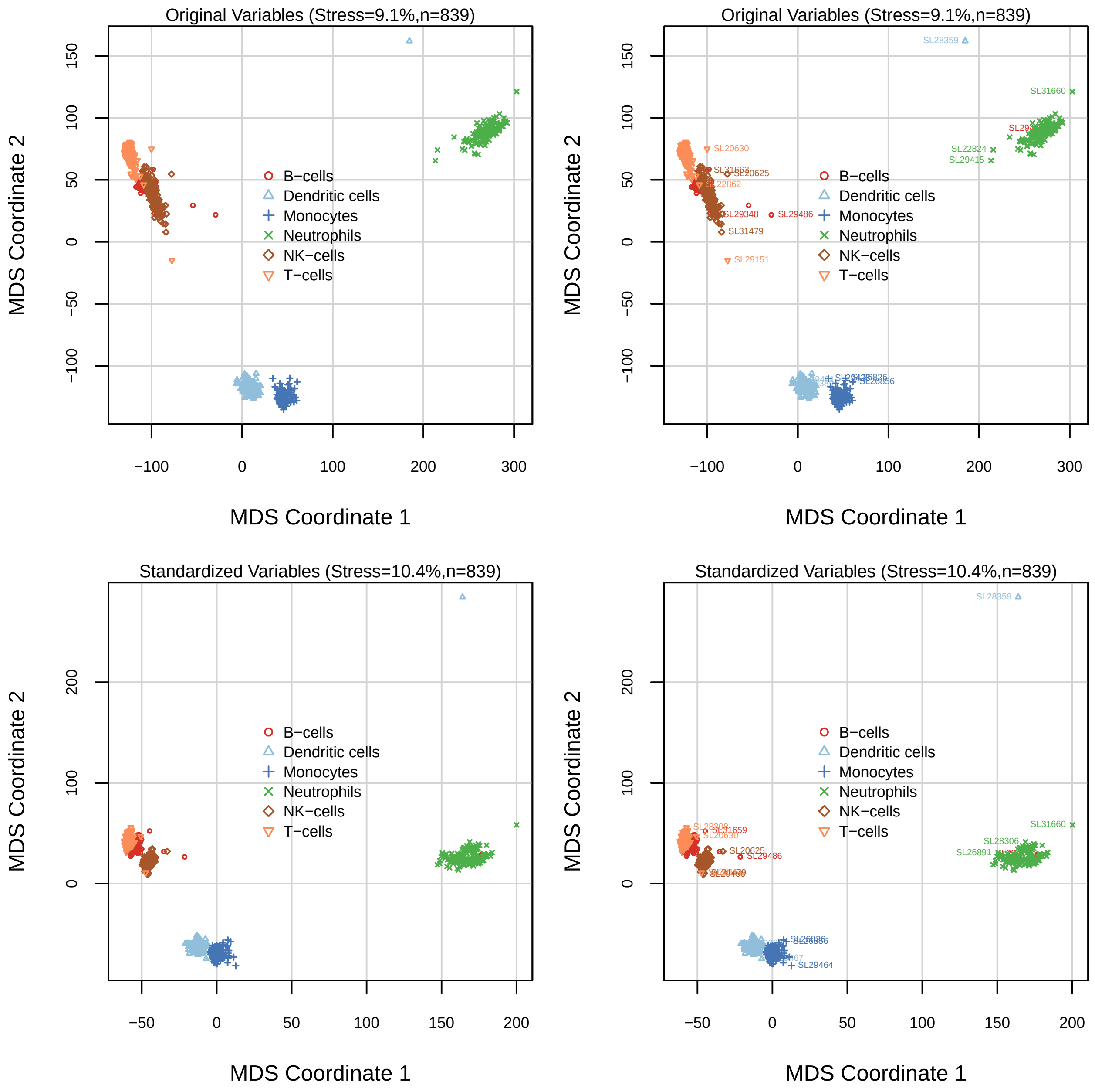

Figure A29: Non-metric multidimensional scaling biplots (RNA-Seq, n=839). Euclidean distance was used to determine pairwise differences. The right panels are identical to the left panels except that they show dataset labels for the three most outlying samples per cell type based on maximum Mahalanobis distance.

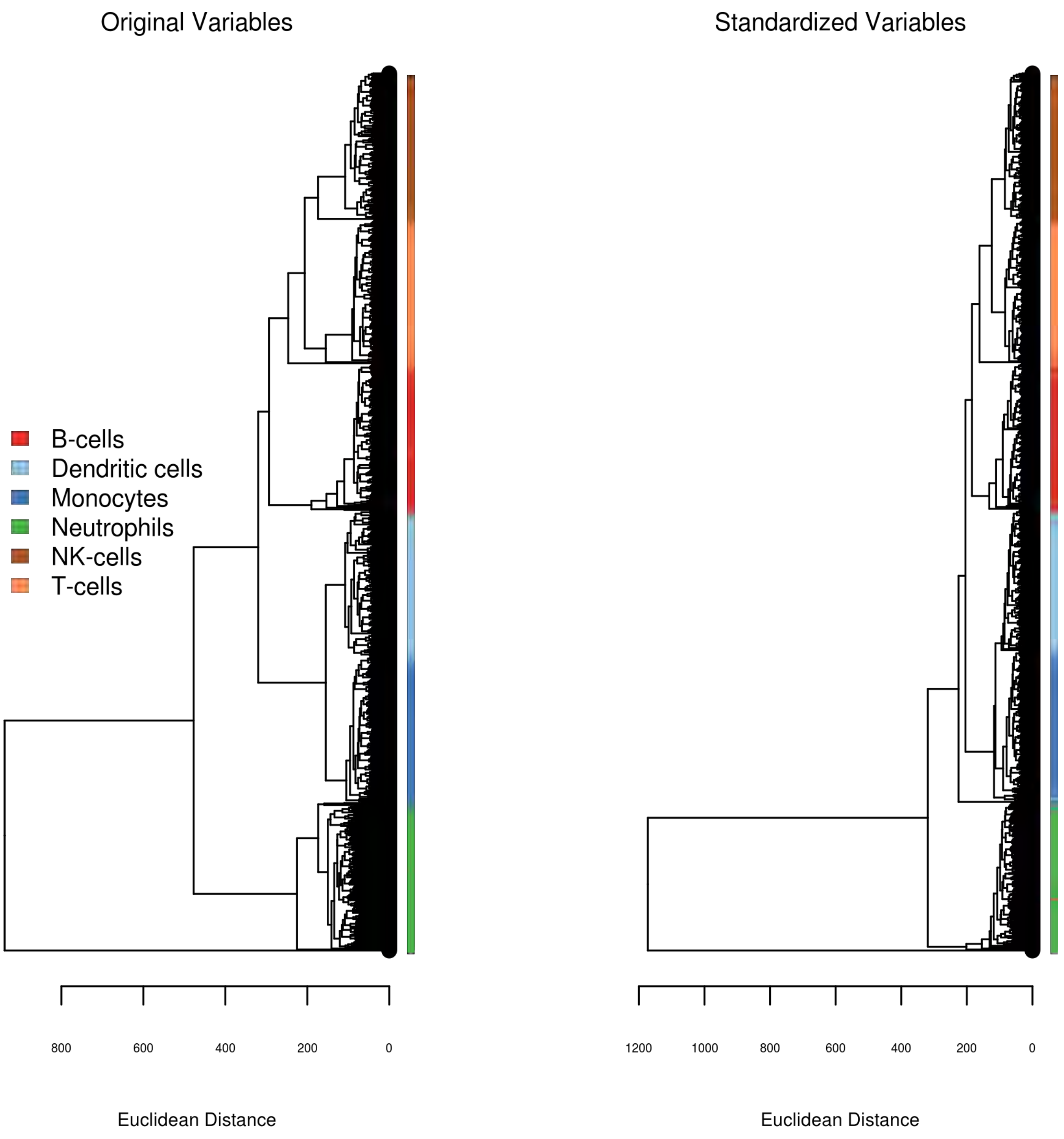

Figure A30: Hierarchical Clustering Plots (RNA-Seq, n=839). Euclidean distances hierarchically clustered using the complete linkage clustering algorithm. Samples are color coded by cell type.

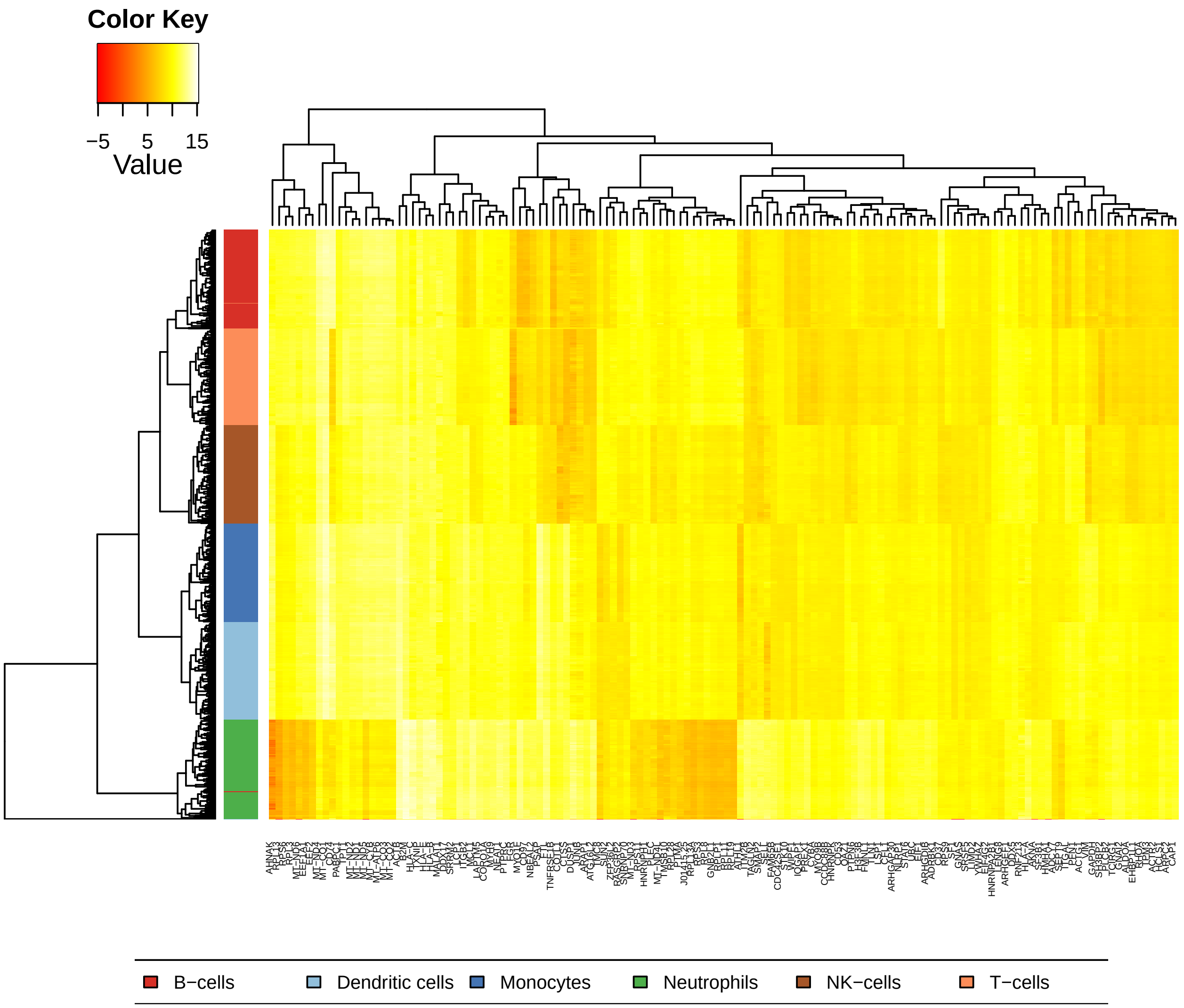

Figure A31: Heatmap of log_2 cpm (RNA-Seq, top-ranking 136 genes, n=839). Euclidean distances hierarchically clustered using the complete linkage clustering algorithm. Top-ranking genes were selected by selecting the upper 10th percentile of genes ranked by descending sum of lcpm across all 839 samples.

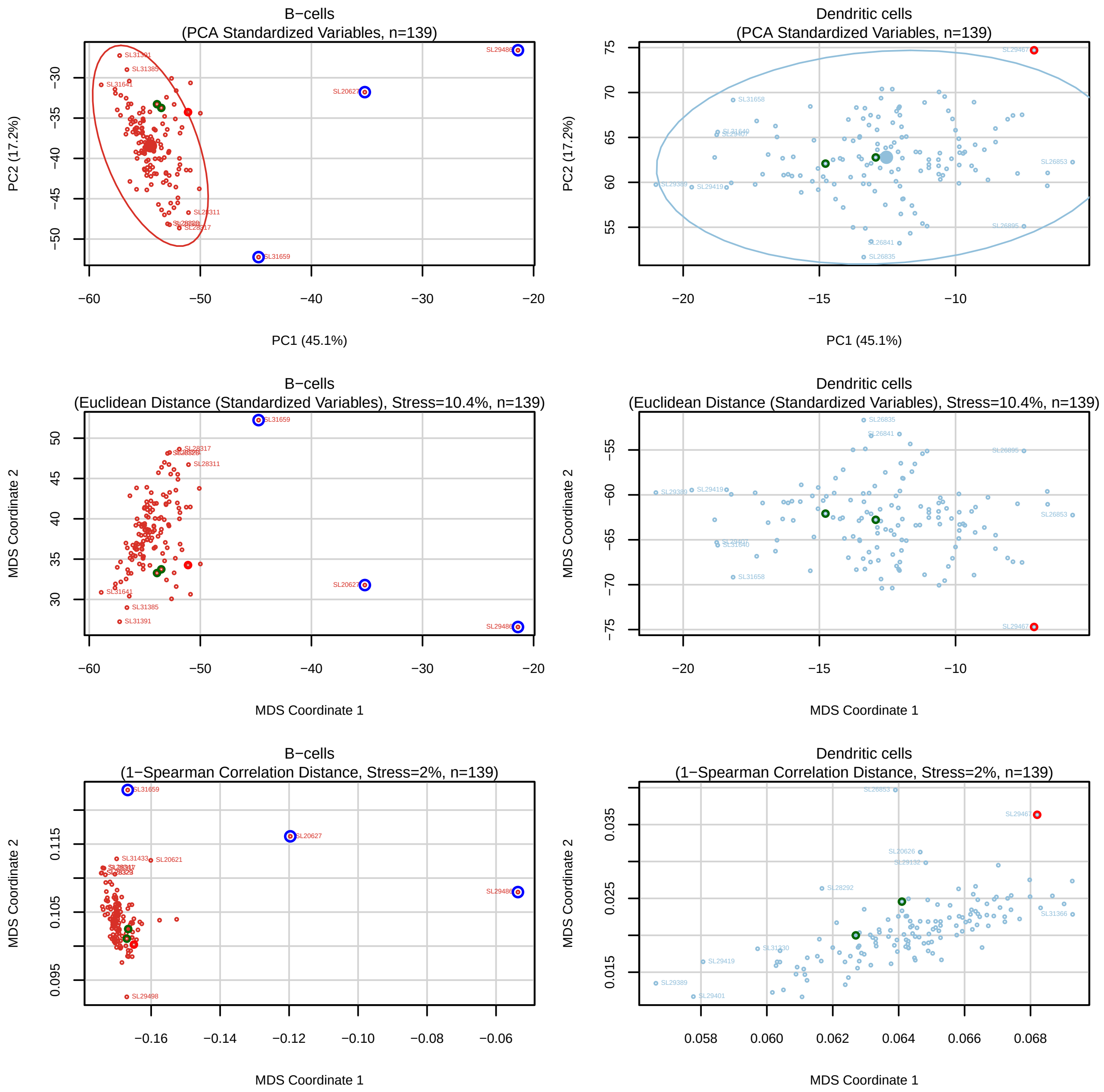

Figure A32: PCA and non-metric multidimensional scaling biplots (RNA-Seq, B-cells and Dendritic cells, n=140). PCA biplots with bivariate 99.6% confidence ellipses for standardized variables are shown at the top. Non-metric MDS results for standardized variables and pairwise differences based on Euclidean distance are shown in the middle. Non-metric MDS results for original variables based on 1-Spearman correlation distance are shown at the bottom. Strong outliers are highlighted in blue. Samples belonging to subject H (Day -28) are highlighted in red. Samples belonging to subject H (Day -14 and Day 0) are highlighted in green. Labels for the 10 most outlying samples per cell type based on maximum Mahalanobis distance are shown.

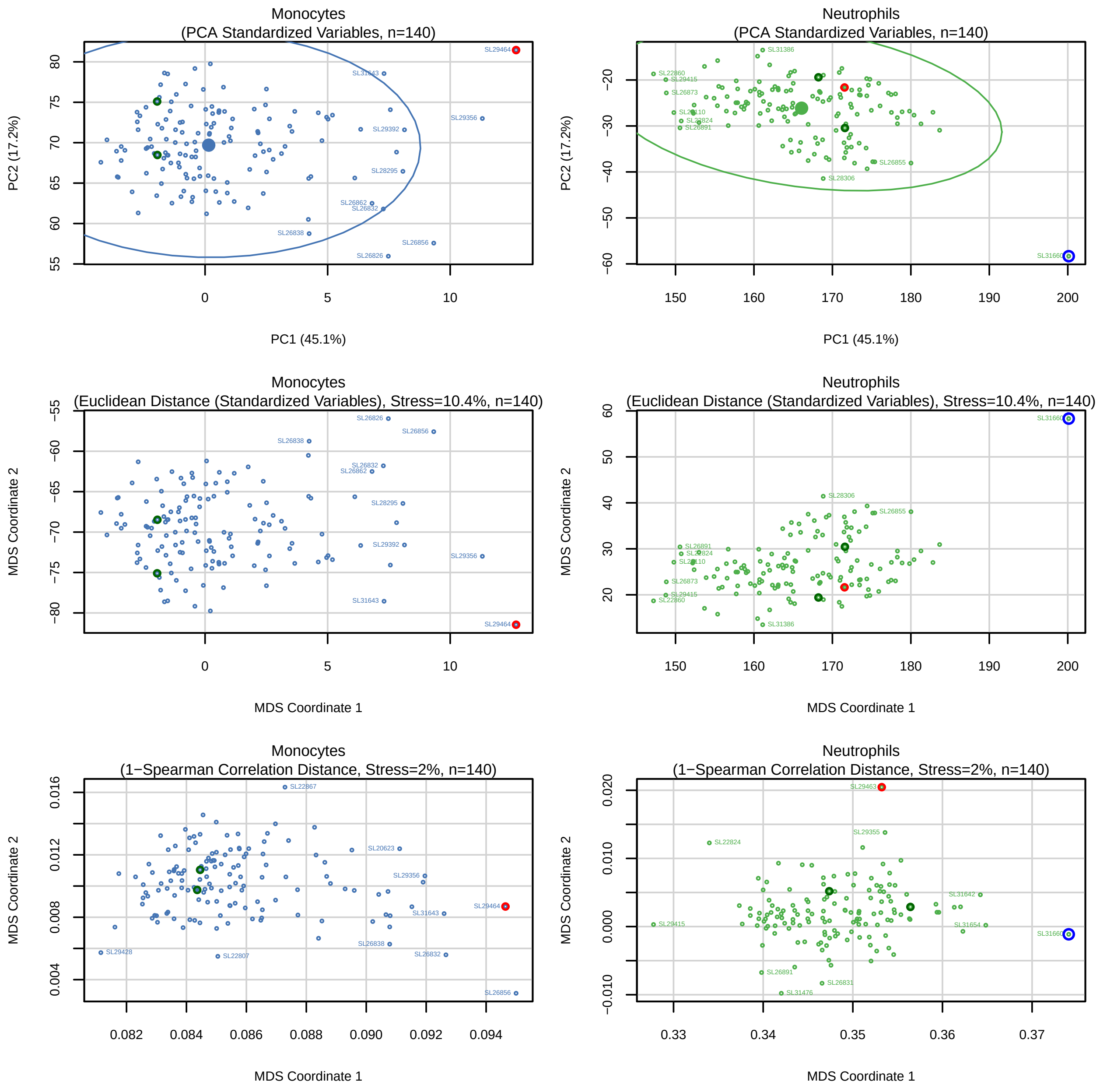

Figure A33: PCA and non-metric multidimensional scaling biplots (RNA-Seq, Monocytes and Neutrophils, n=140). PCA biplots with bivariate 99.6% confidence ellipses for standardized variables are shown at the top. Non-metric MDS results for standardized variables and pairwise differences based on Euclidean distance are shown in the middle. Non-metric MDS results for original variables based on 1-Spearman correlation distance are shown at the bottom. Strong outliers are highlighted in blue. Samples belonging to subject H (Day -28) are highlighted in red. Samples belonging to subject H (Day -14 and Day 0) are highlighted in green. Labels for the 10 most outlying samples per cell type based on maximum Mahalanobis distance are shown.

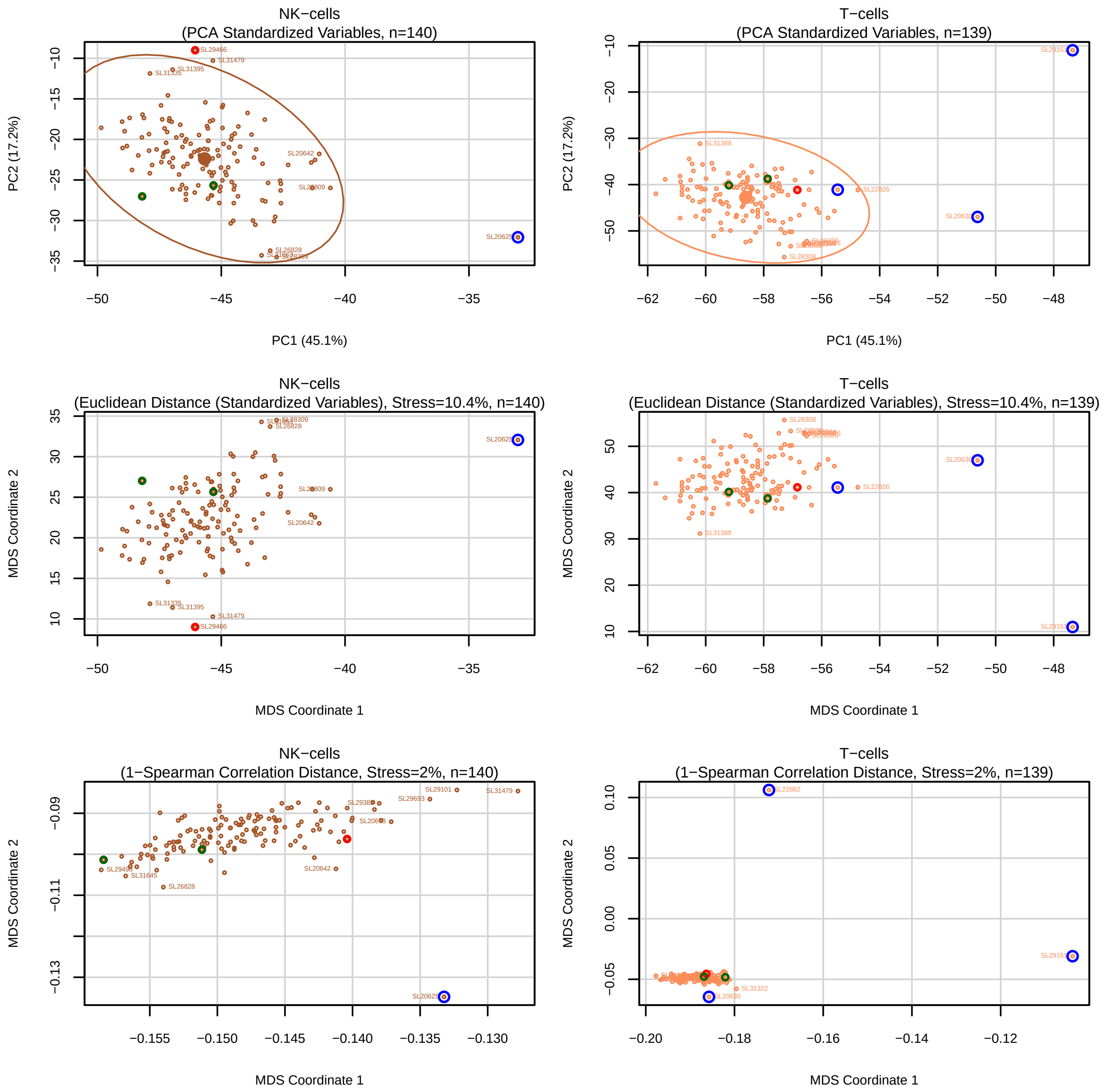

Figure A34: PCA and non-metric multidimensional scaling biplots (RNA-Seq, NK-cells and T-cells, n=140). PCA biplots with bivariate 99.6% confidence ellipses for standardized variables are shown at the top. Non-metric MDS results for standardized variables and pairwise differences based on Euclidean distance are shown in the middle. Non-metric MDS results for original variables based on 1-Spearman correlation distance are shown at the bottom. Strong outliers are highlighted in blue. Samples belonging to subject H (Day -28) are highlighted in red. Samples belonging to subject H (Day -14 and Day 0) are highlighted in green. Labels for the 10 most outlying samples per cell type based on maximum Mahalanobis distance are shown.

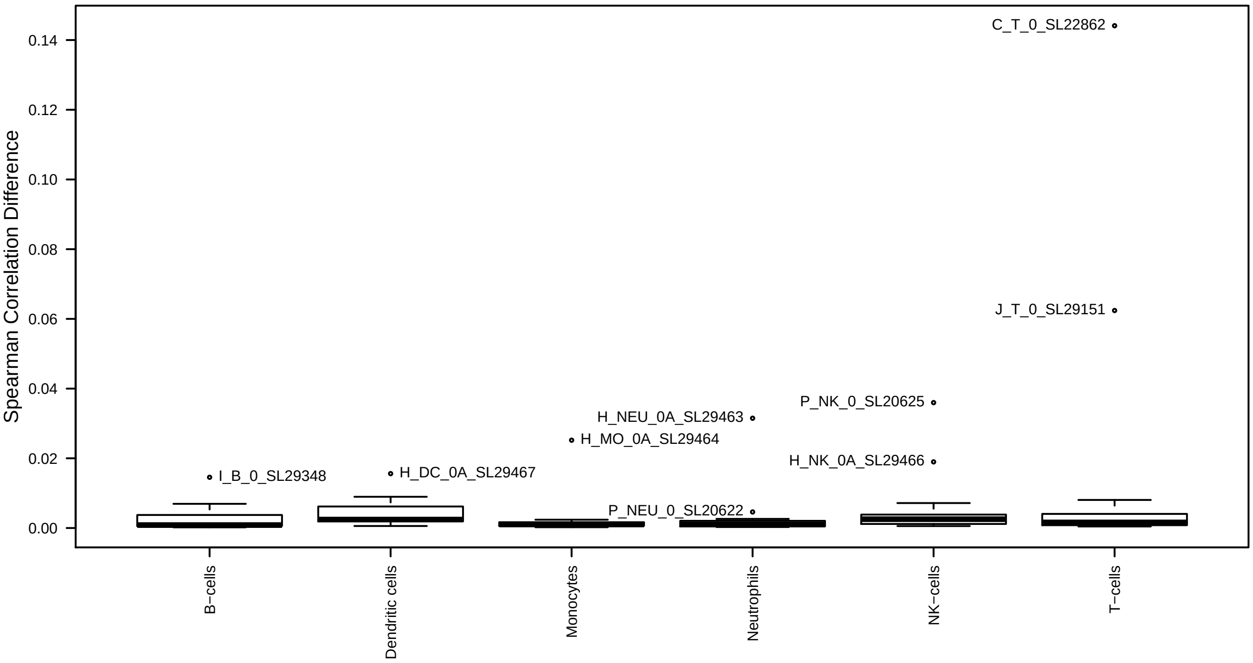

Figure A35: Boxplots summarizing the difference between maximum and minimum Spearman correlation among baseline samples (Day -28, Day -14, Day 0) for each cell type and subject (RNA-Seq, n=20).

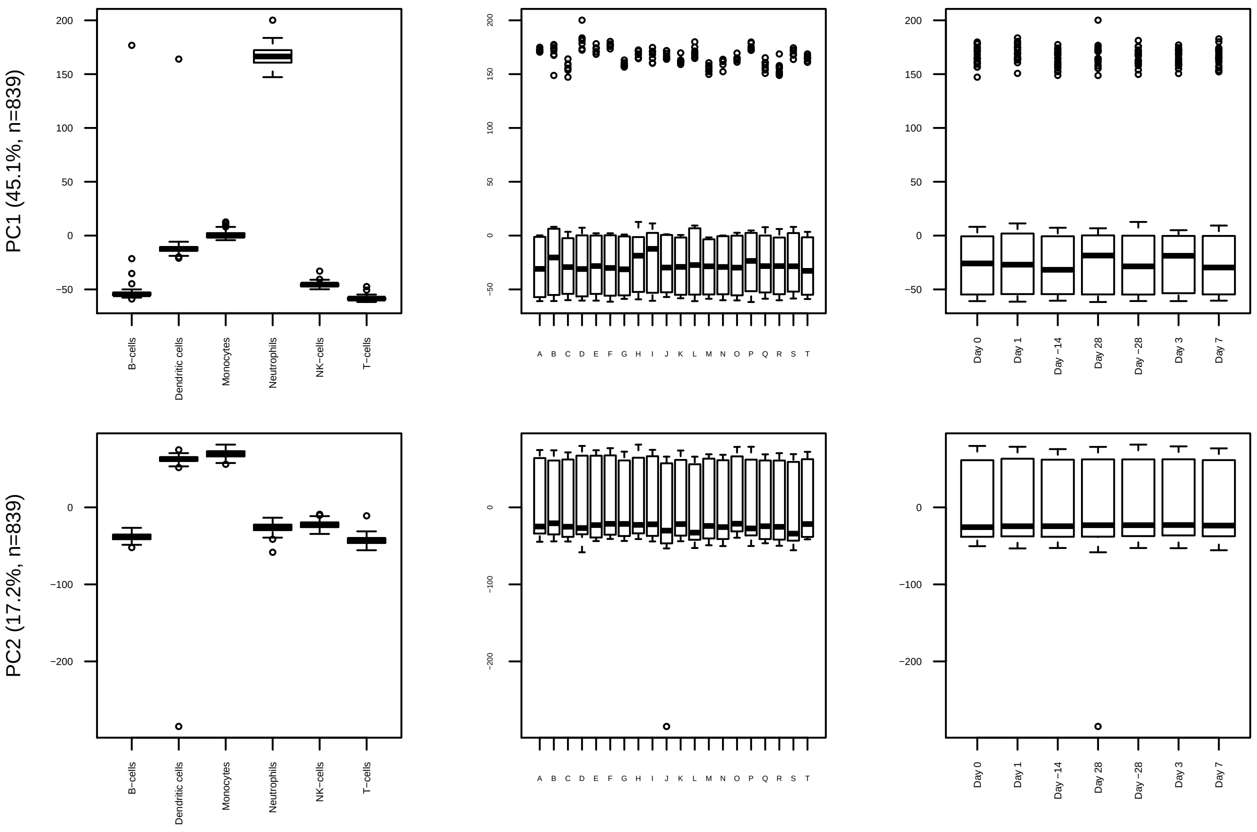

Figure A36: Variance component plots (RNA-Seq, n=839, PC1 and PC2).

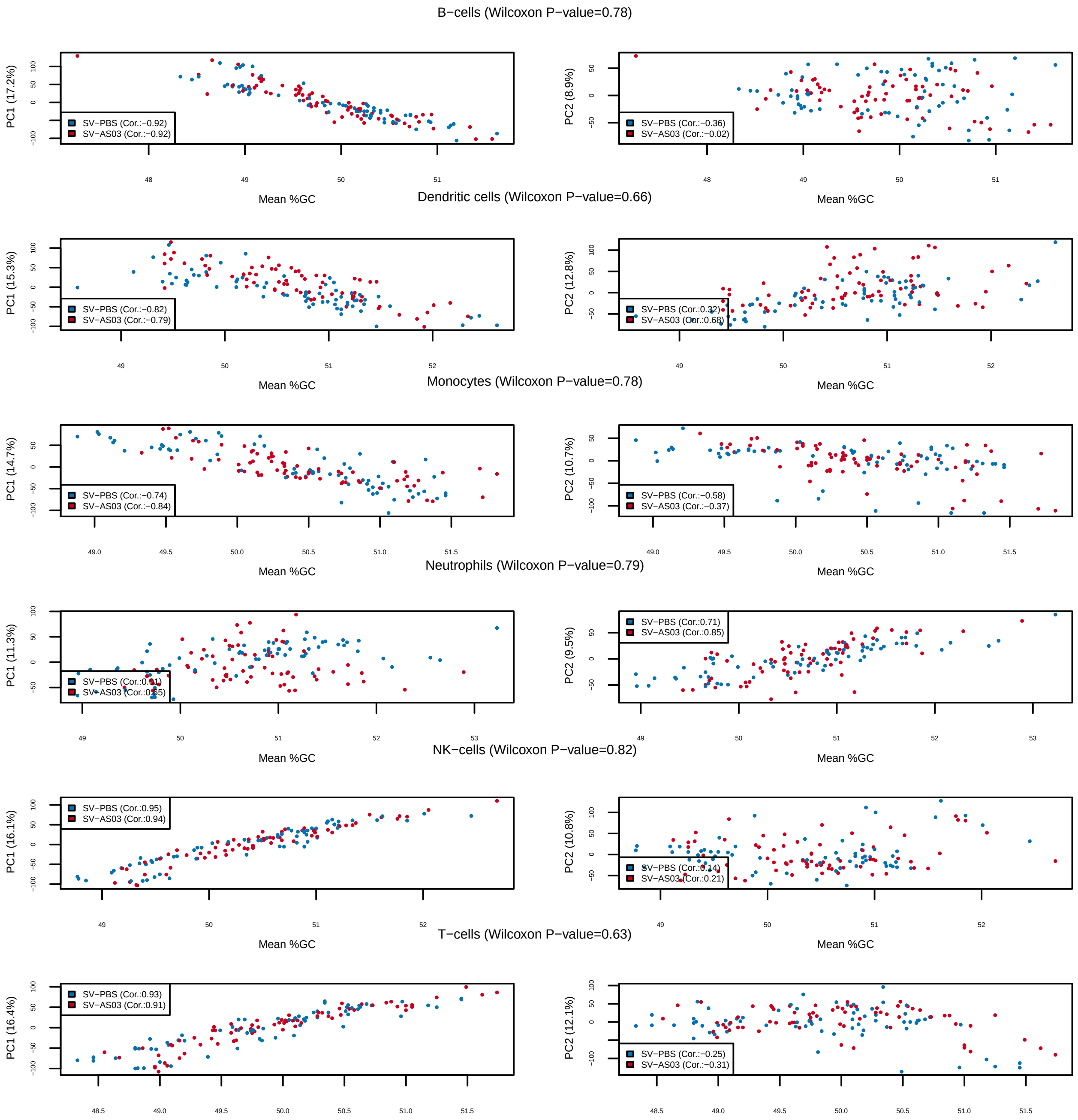

Figure A37: Correlation with mean %GC content. Wilcoxon Rank-Sum test P-value for comparing mean %GC content between treatments is shown in the header for each cell subset (RNA-Seq).

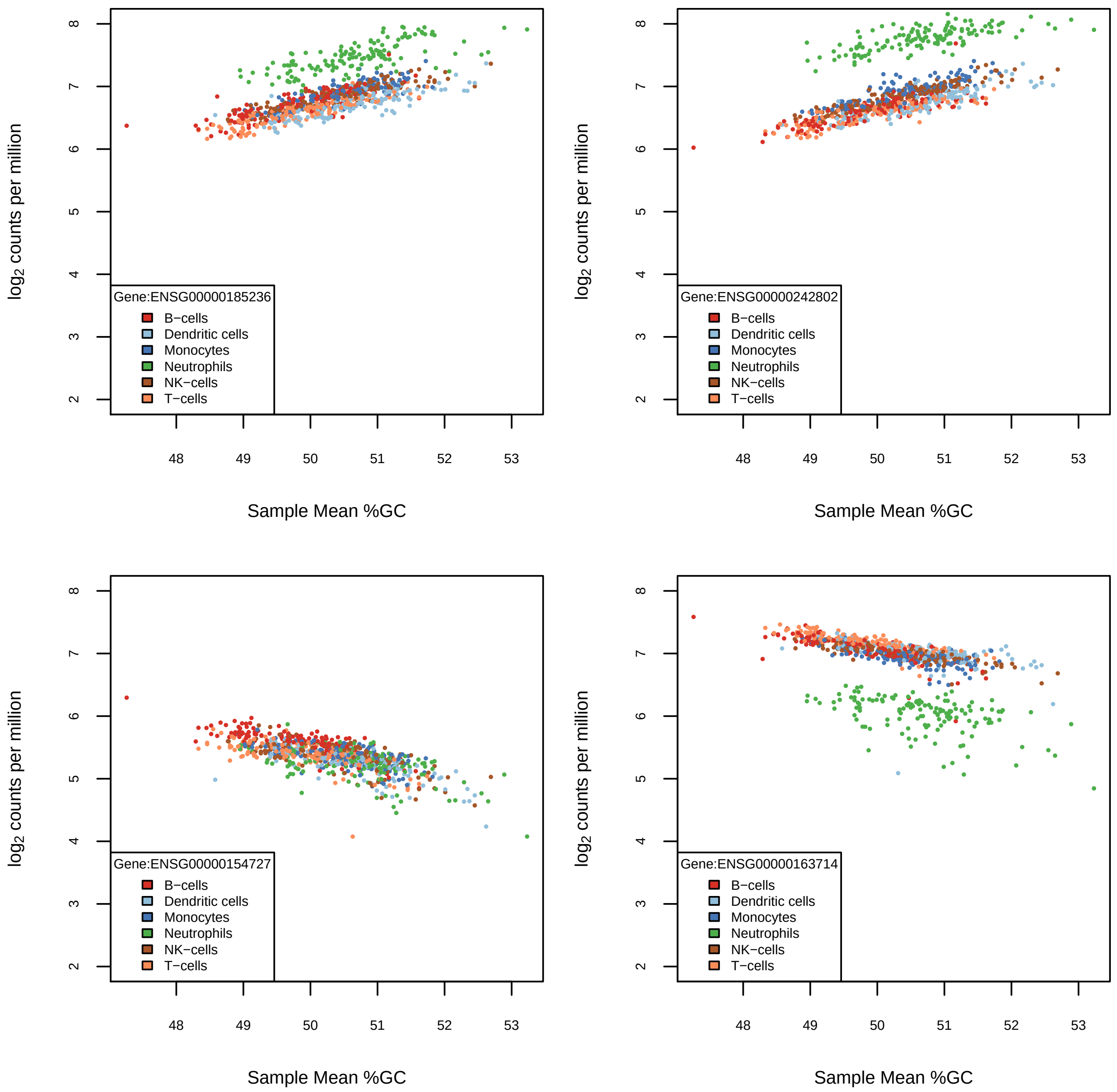

Figure A38: Example genes with strong mean %GC content association (RNA-Seq).

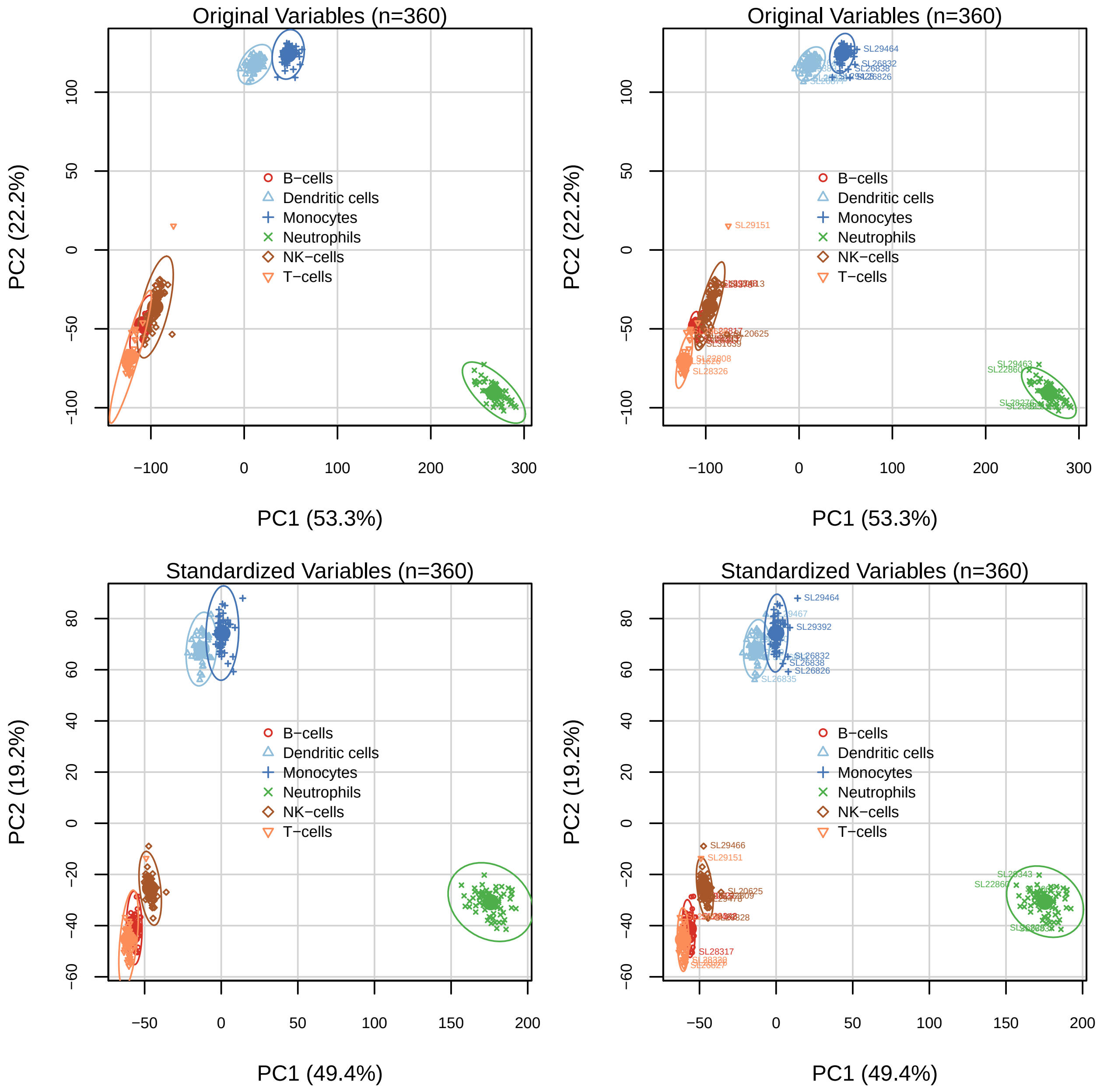

Figure A39: Baseline PCA biplots (RNA-Seq, n=360). 99.2% confidence ellipses are drawn for each cell type. The right panels are identical to the left panels except that they show dataset labels for the three most outlying samples per cell type based on maximum Mahalanobis distance.

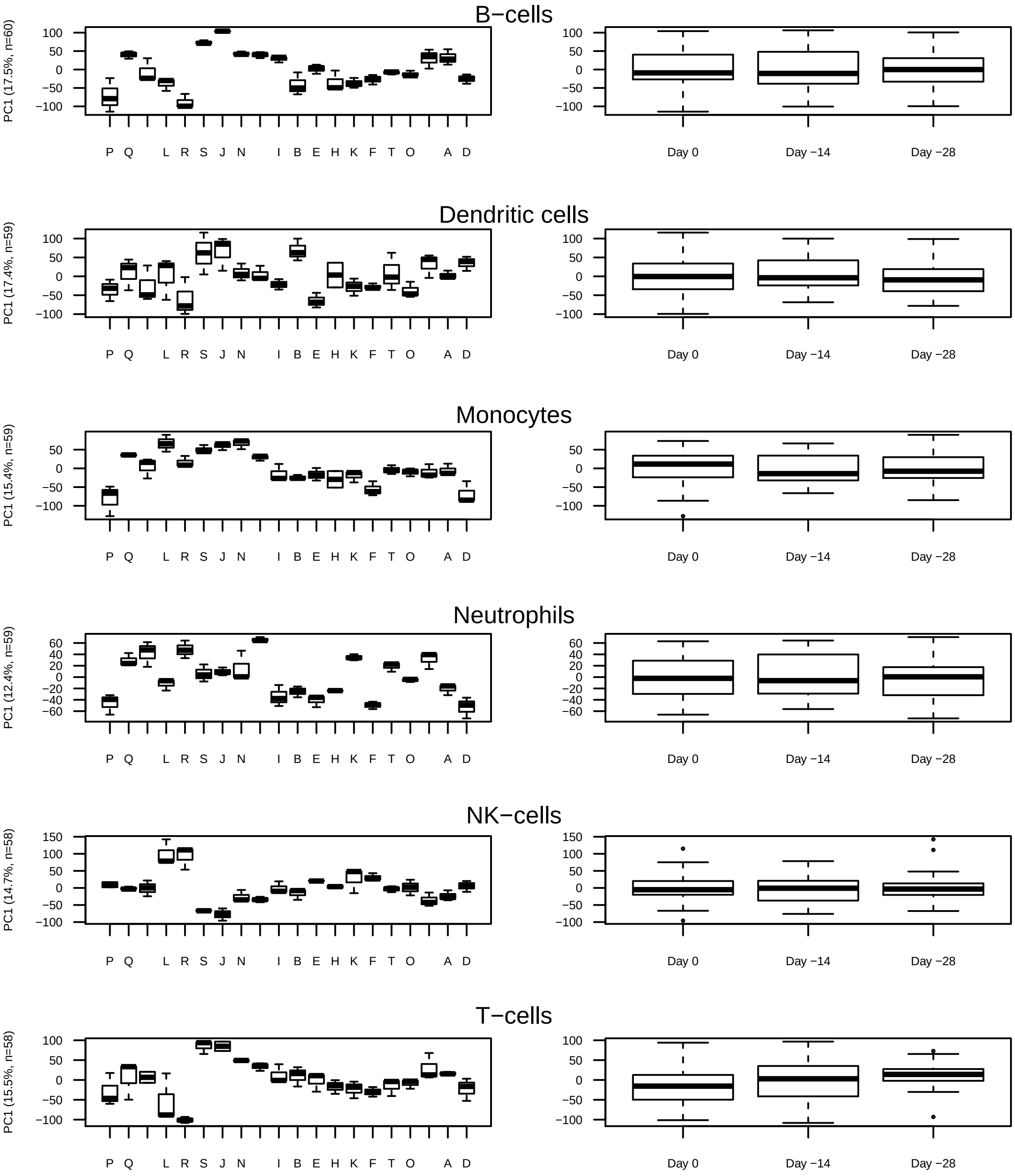

Figure A40: Baseline variance component plots (RNA-Seq, PC1).s

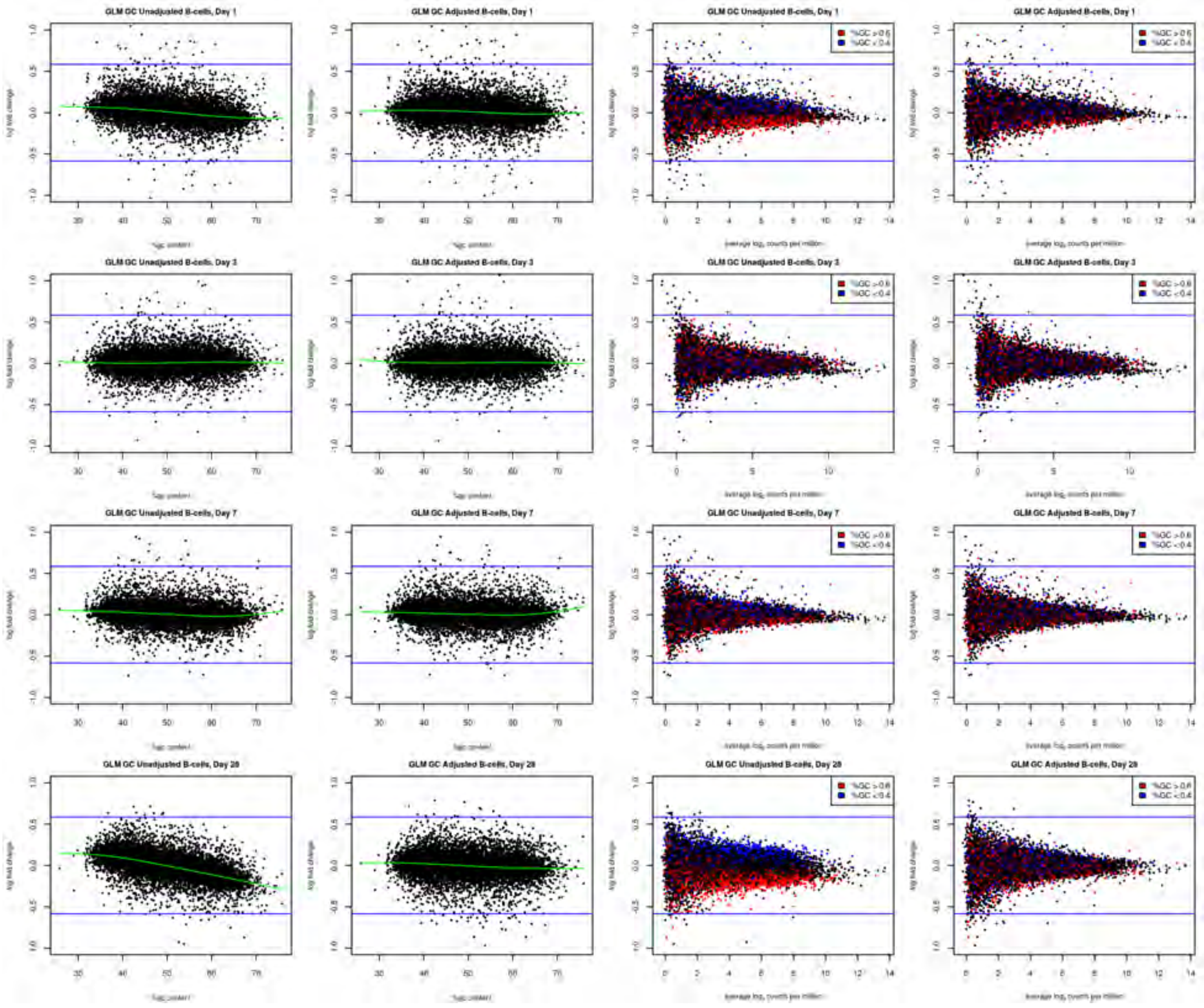

Figure A41: Impact of GC on fold change (RNA-Seq, B-cells).

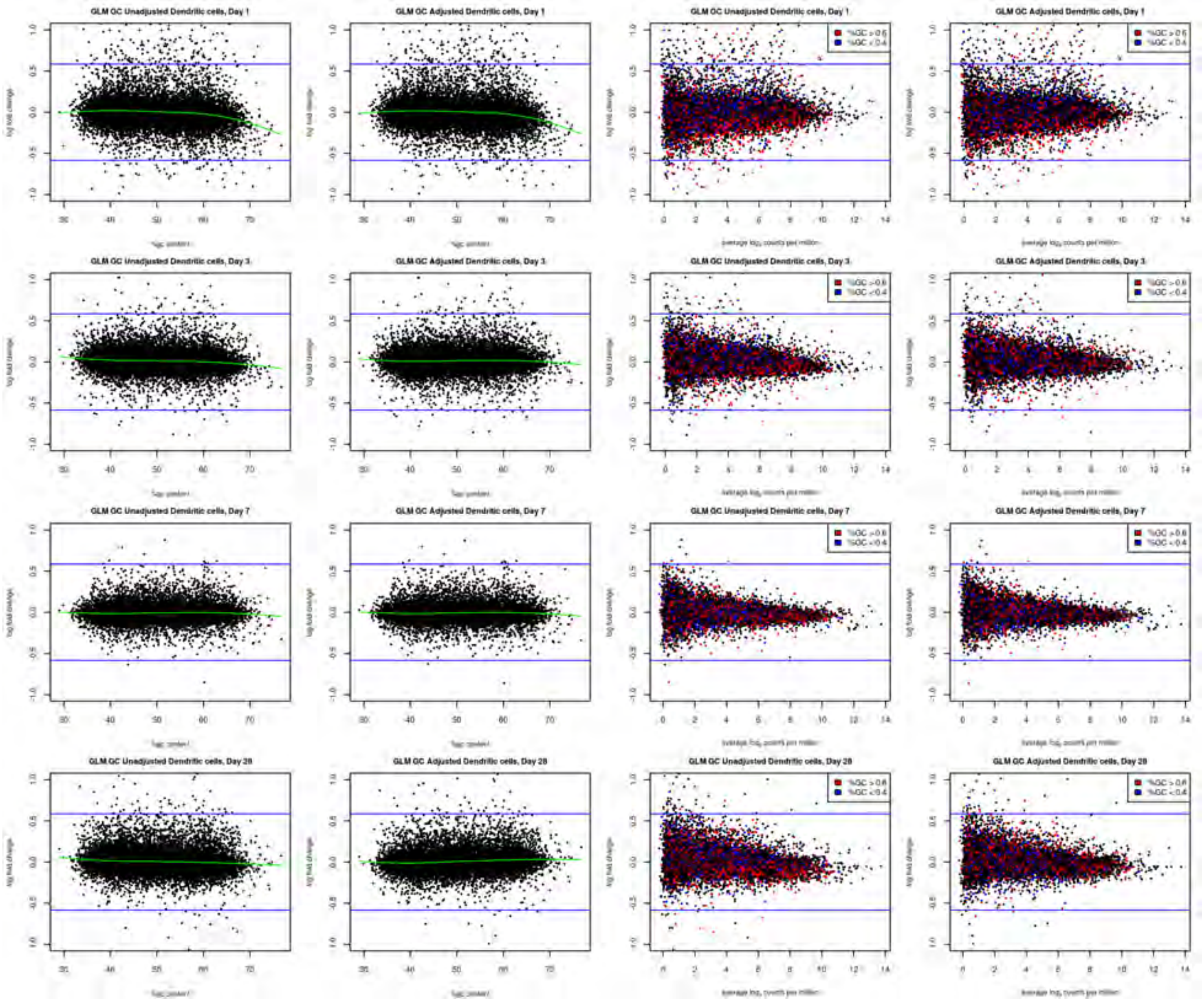

Figure A42: Impact of GC on fold change (RNA-Seq, Dendritic cells).

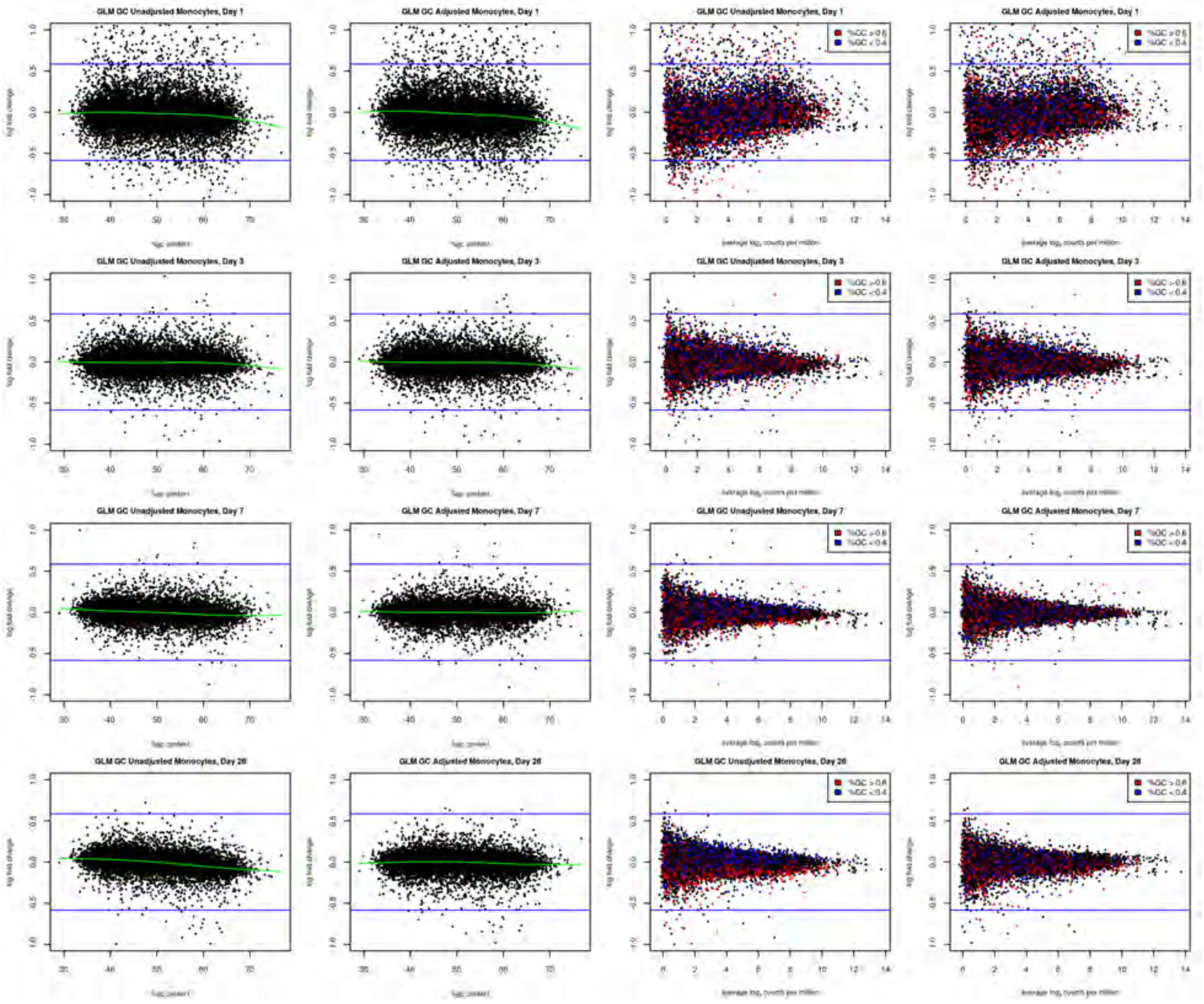

Figure A43: Impact of GC on fold change (RNA-Seq, Monocytes).

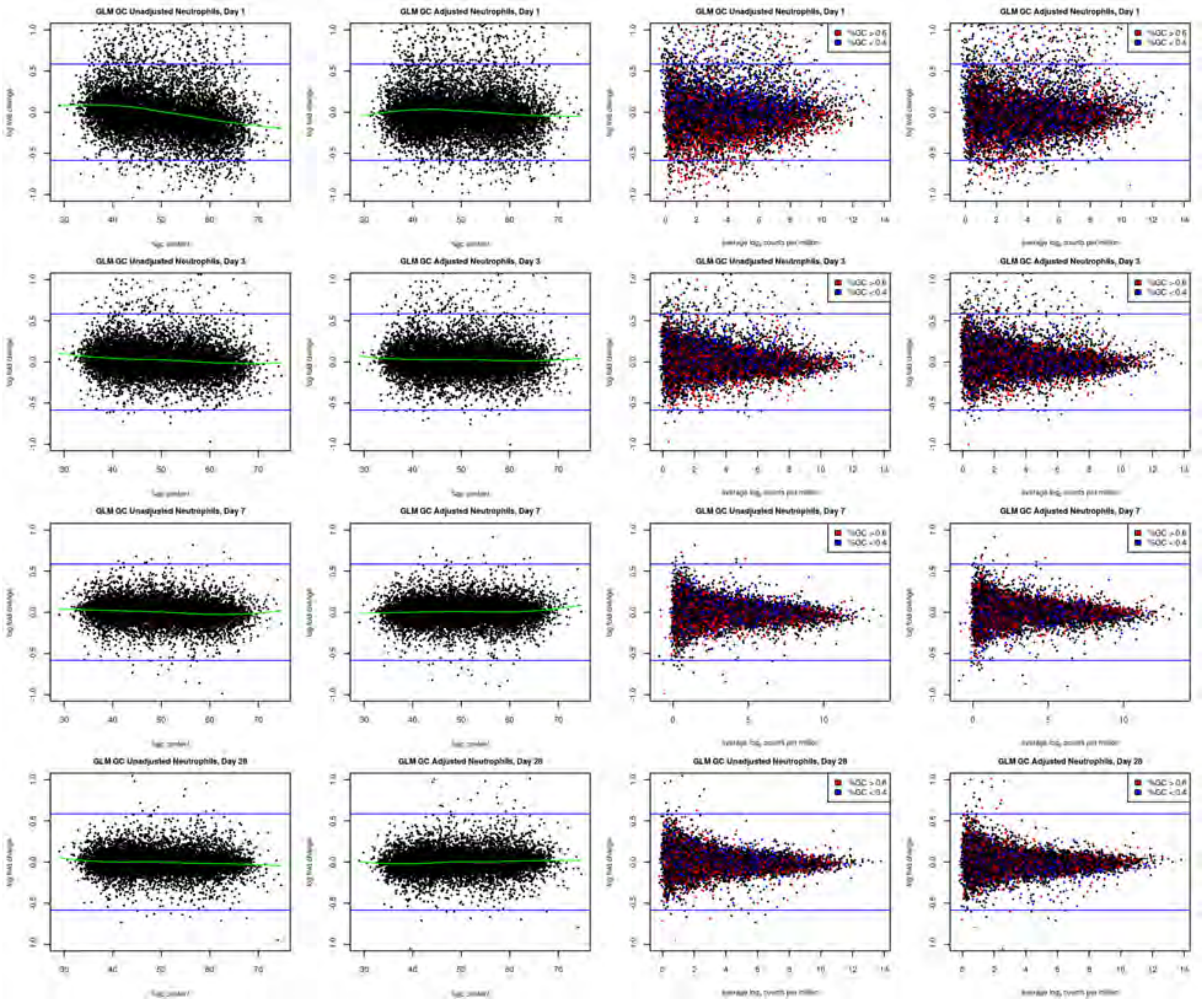

Figure A44: Impact of GC on fold change (RNA-Seq, Neutrophils).

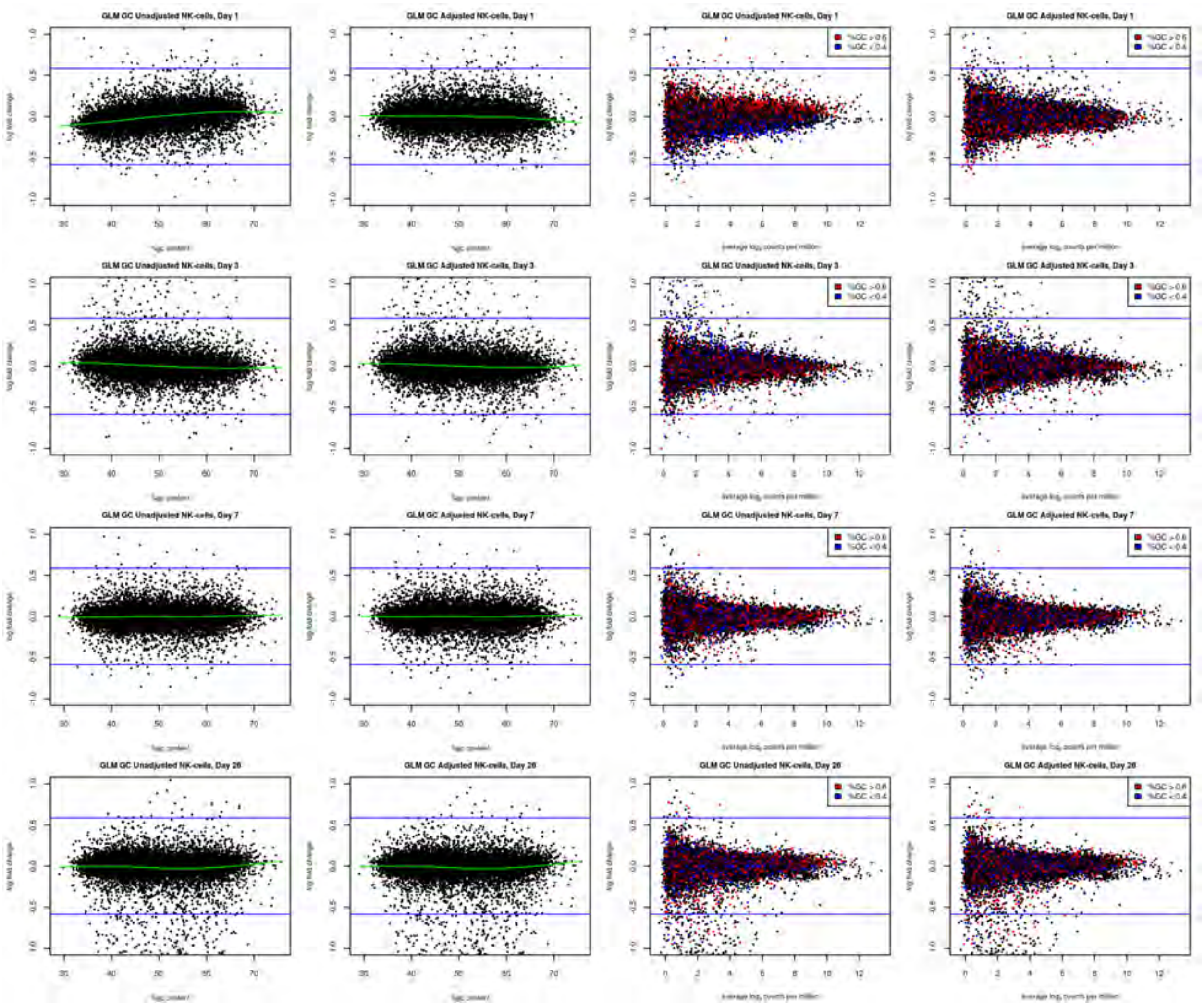

Figure A45: Impact of GC on fold change (RNA-Seq, NK-cells).

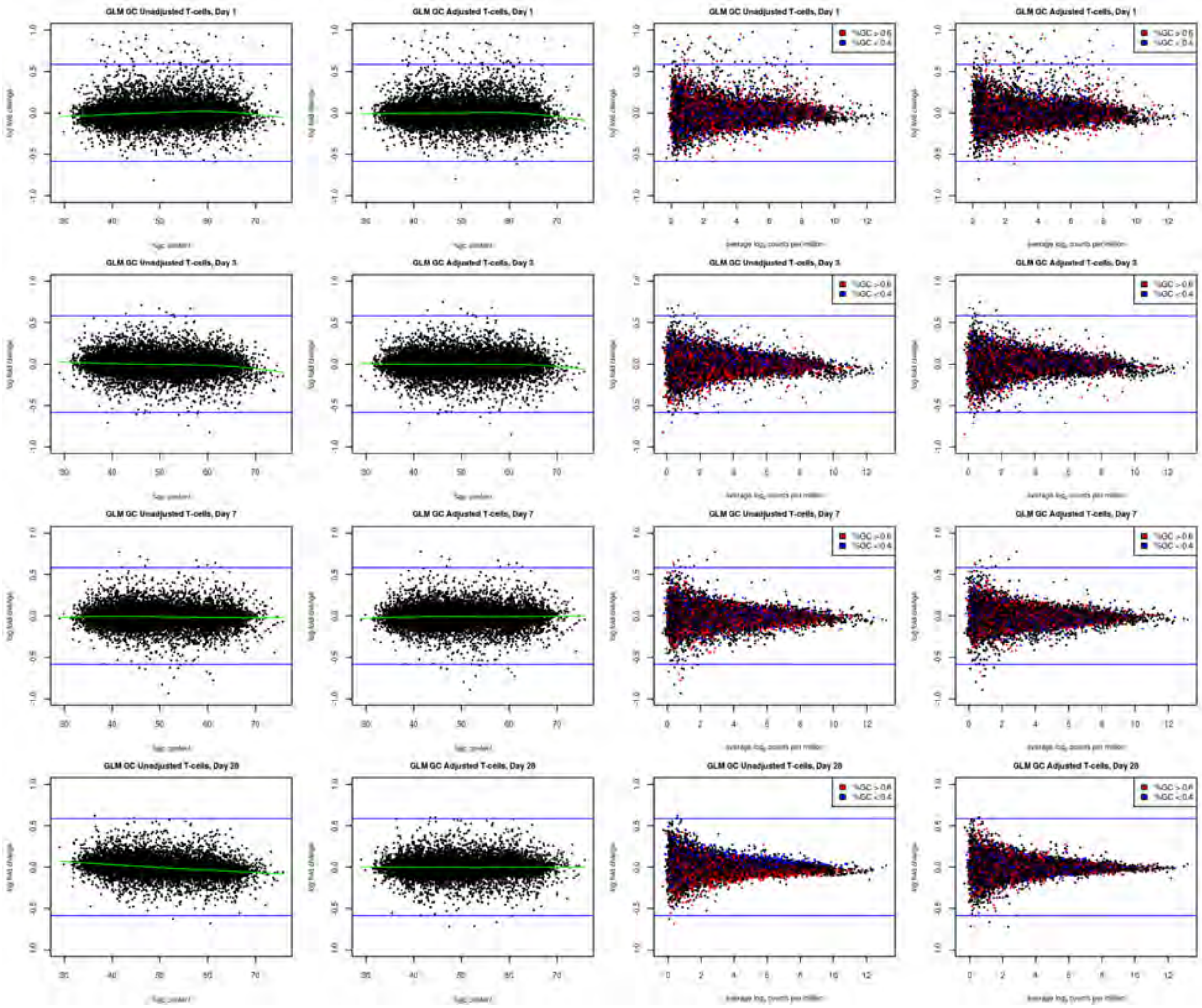

Figure A46: Impact of GC on fold change (RNA-Seq, T-cells).

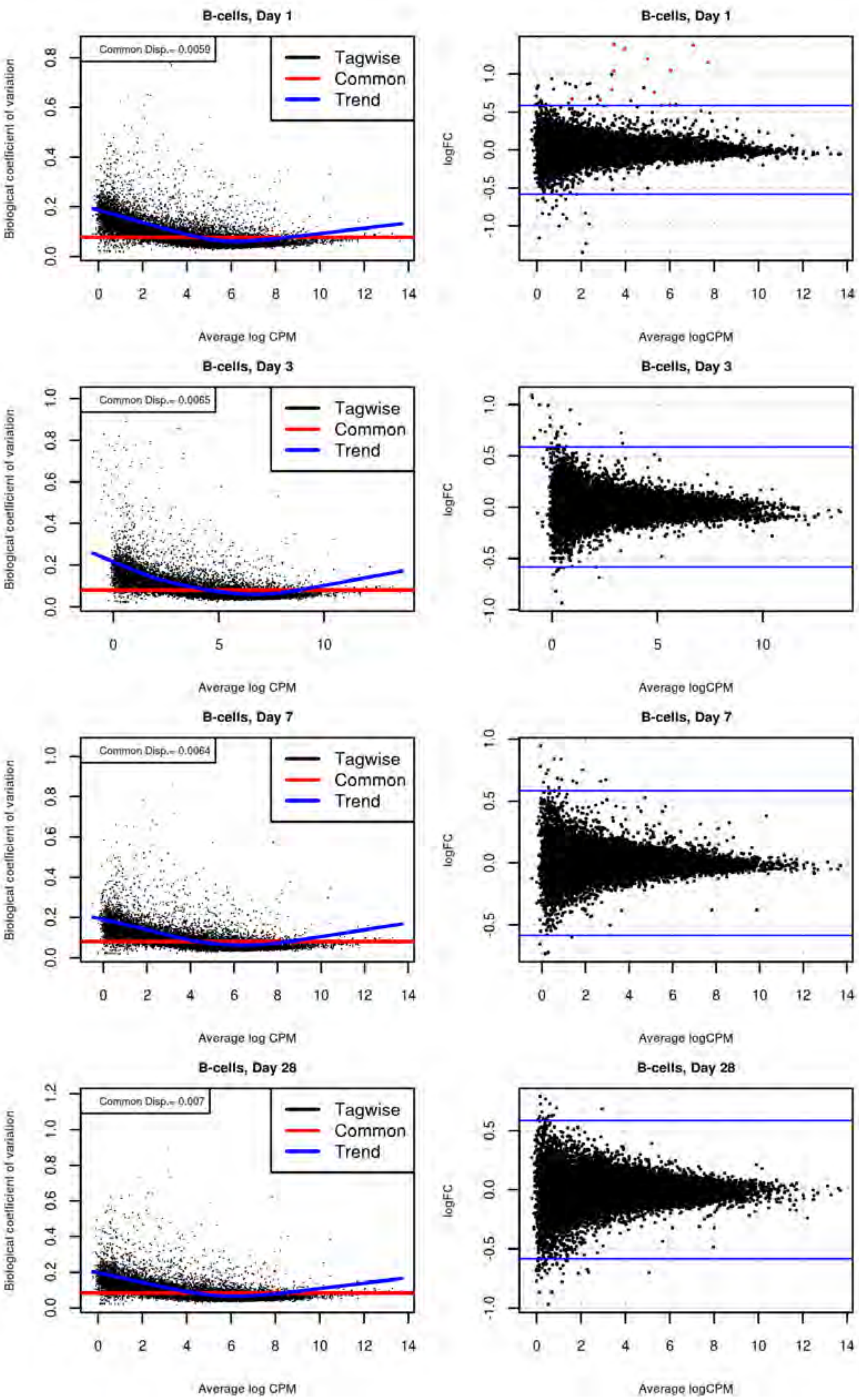

Figure A47: Model QC summary plots (RNA-Seq, B-cells).

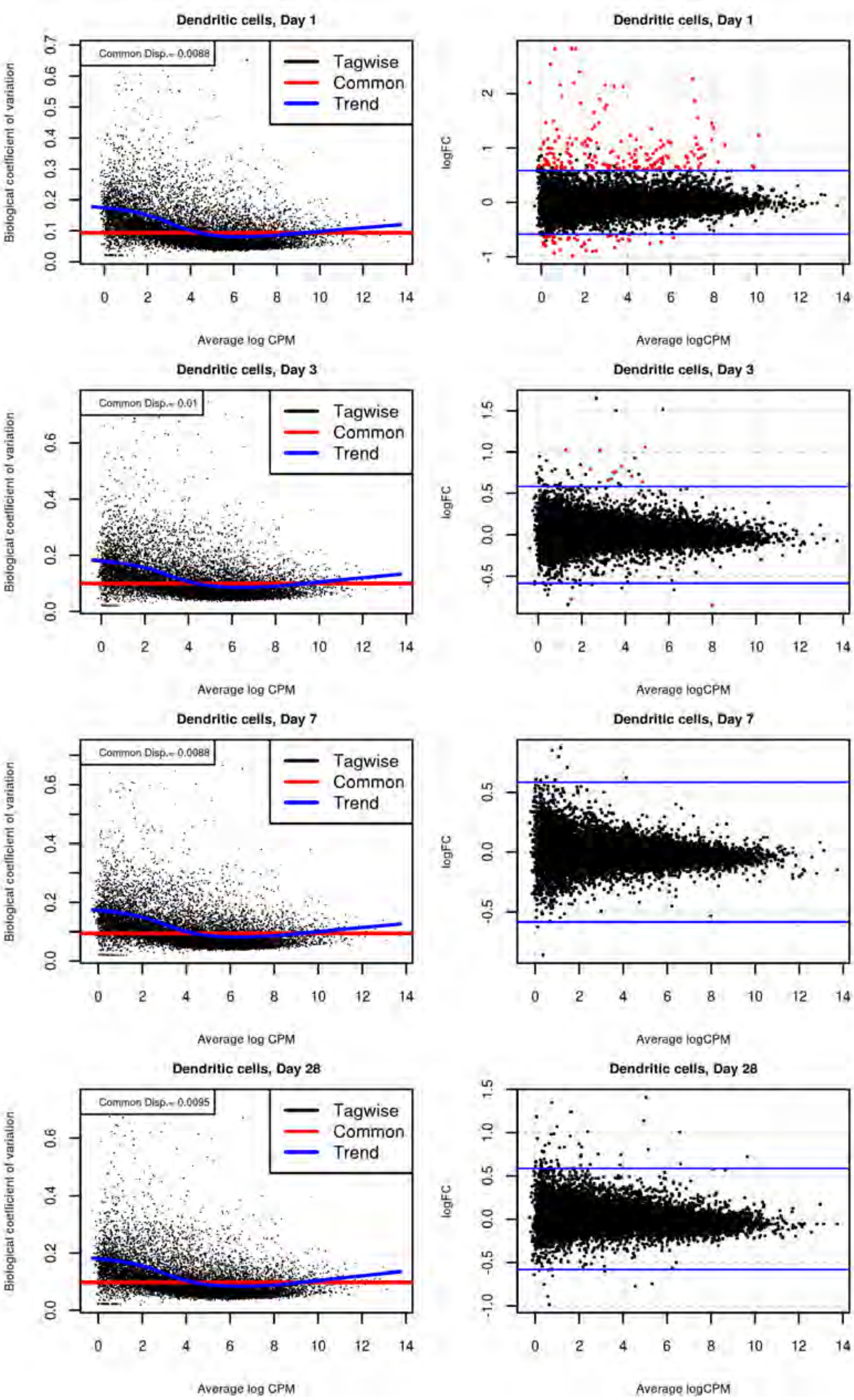

Figure A48: Model QC summary plots (RNA-Seq, Dendritic cells).

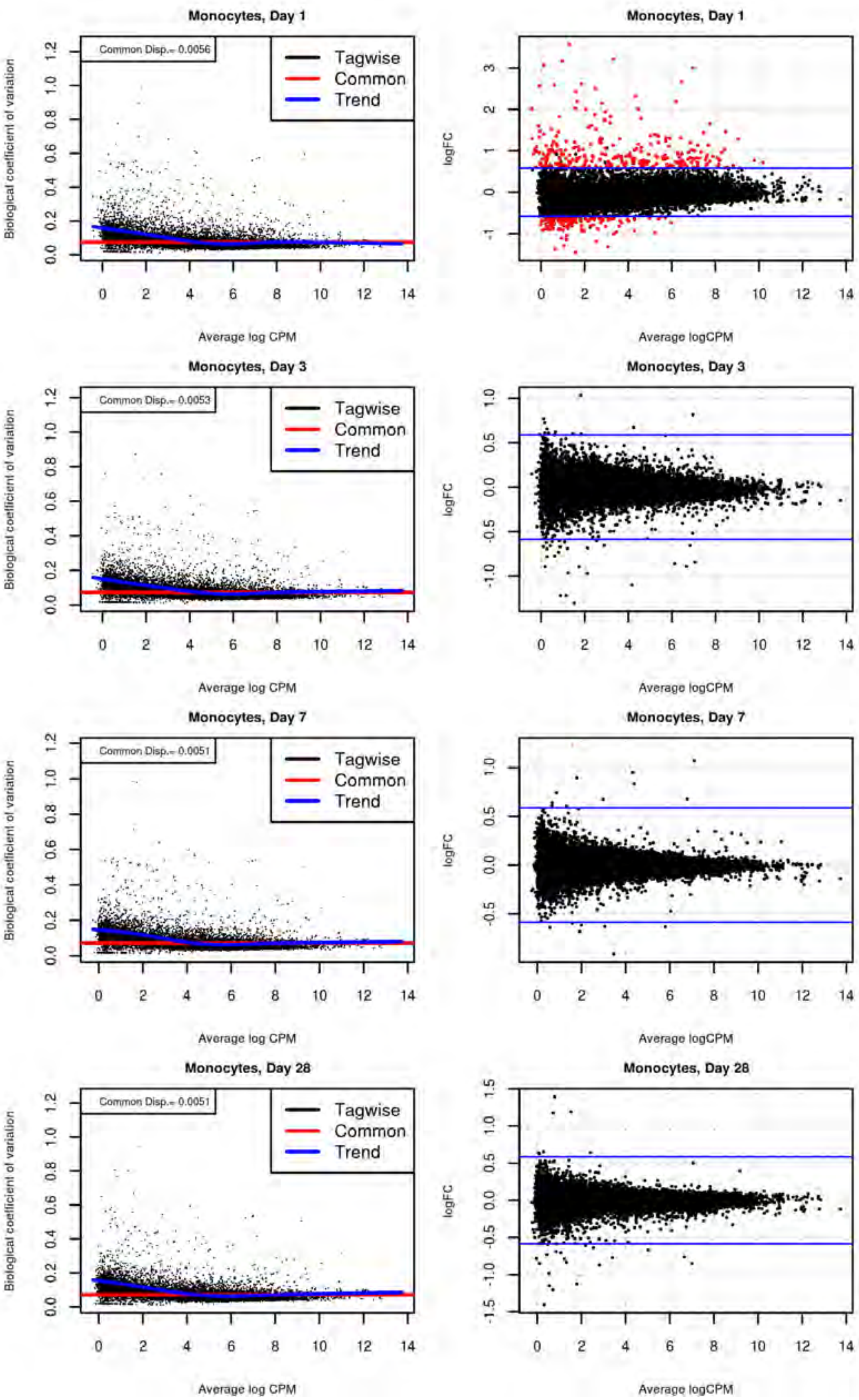

Figure A49: Model QC summary plots (RNA-Seq, Monocytes).

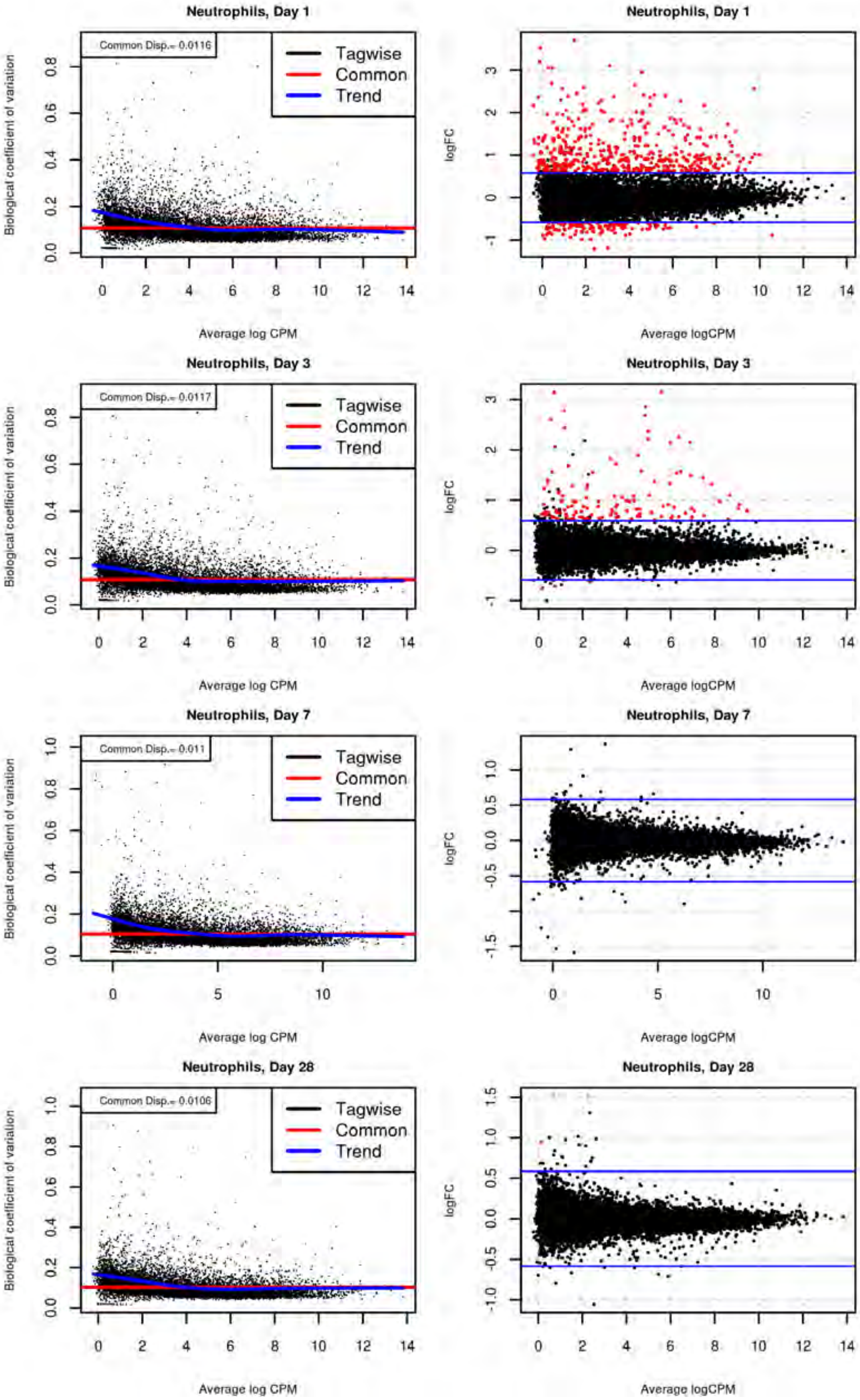

Figure A50: Model QC summary plots (RNA-Seq, Neutrophils).

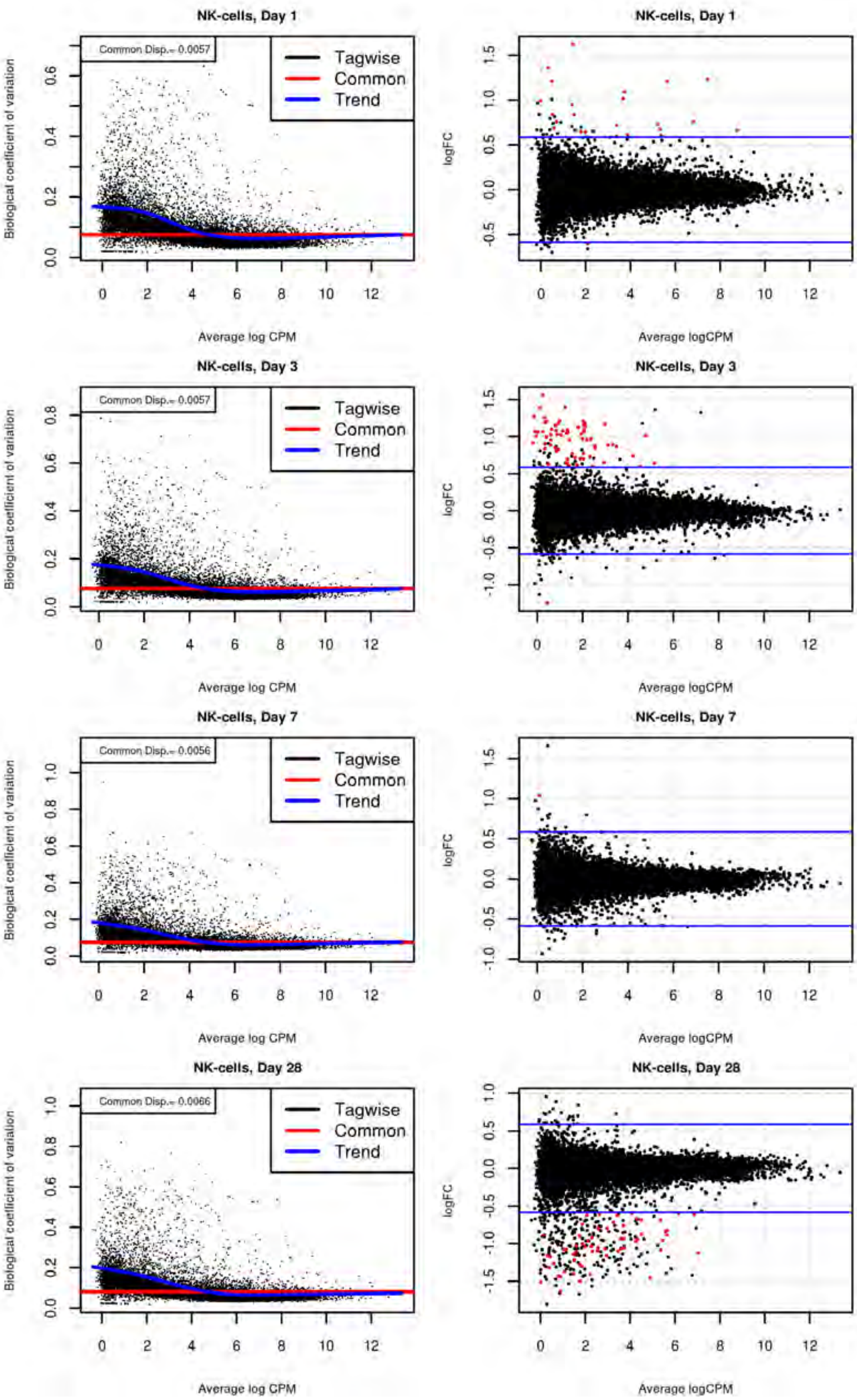

Figure A51: Model QC summary plots (RNA-Seq, NK-cells).

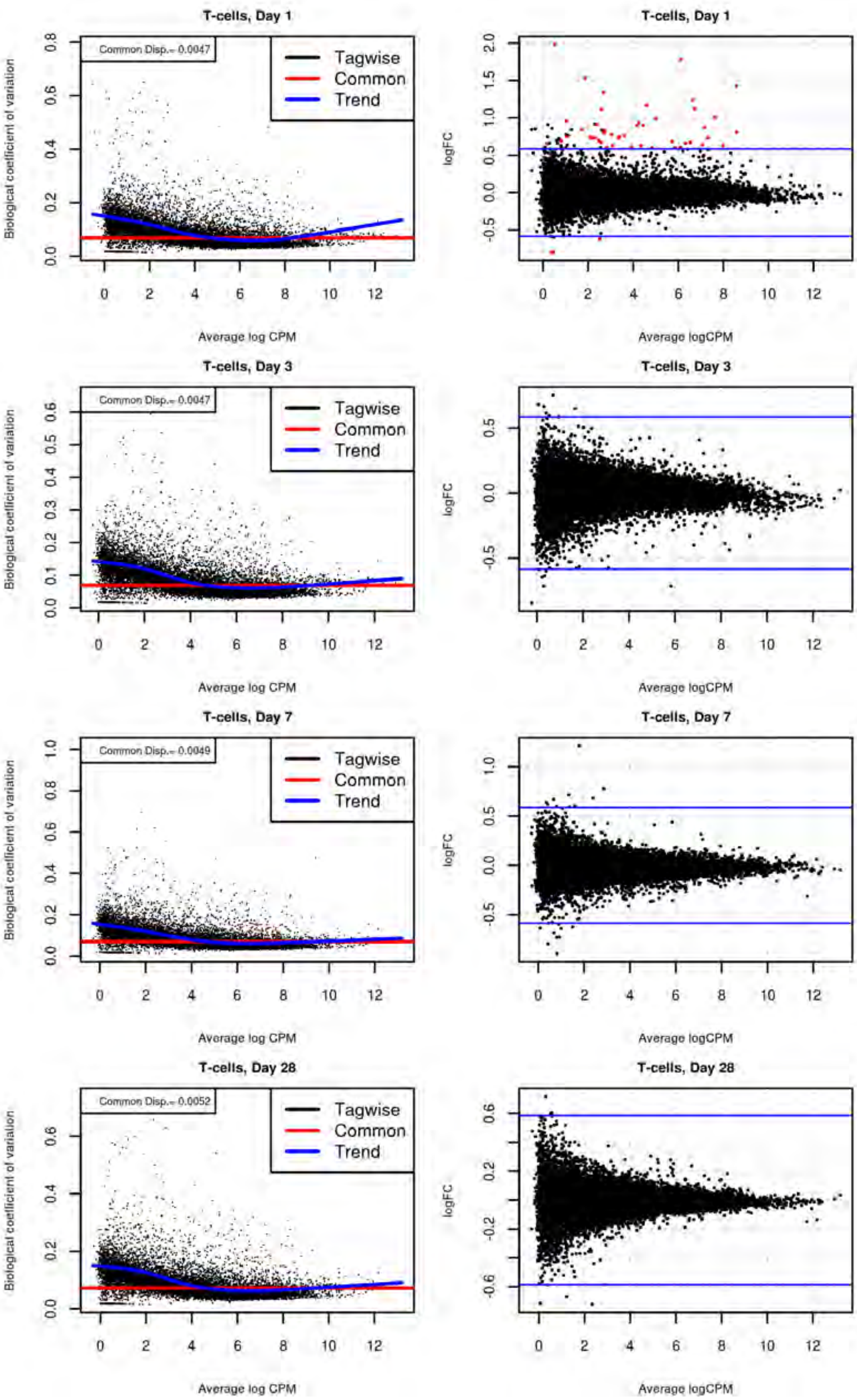

Figure A52: Model QC summary plots (RNA-Seq, T-cells).

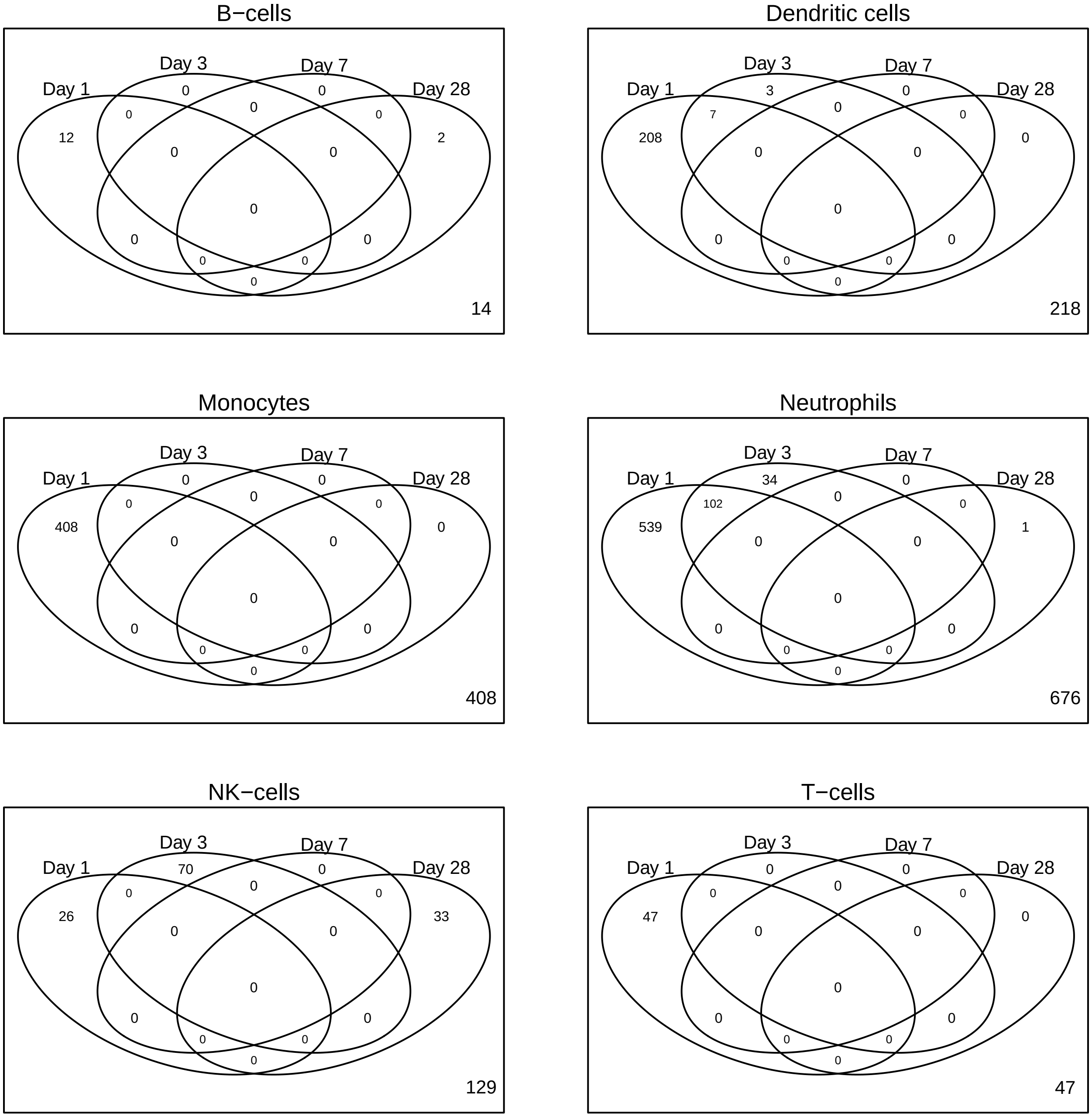

Figure A53: Venn diagrams of significant gene counts by day and cell type (RNA-Seq, before GC adjustment).

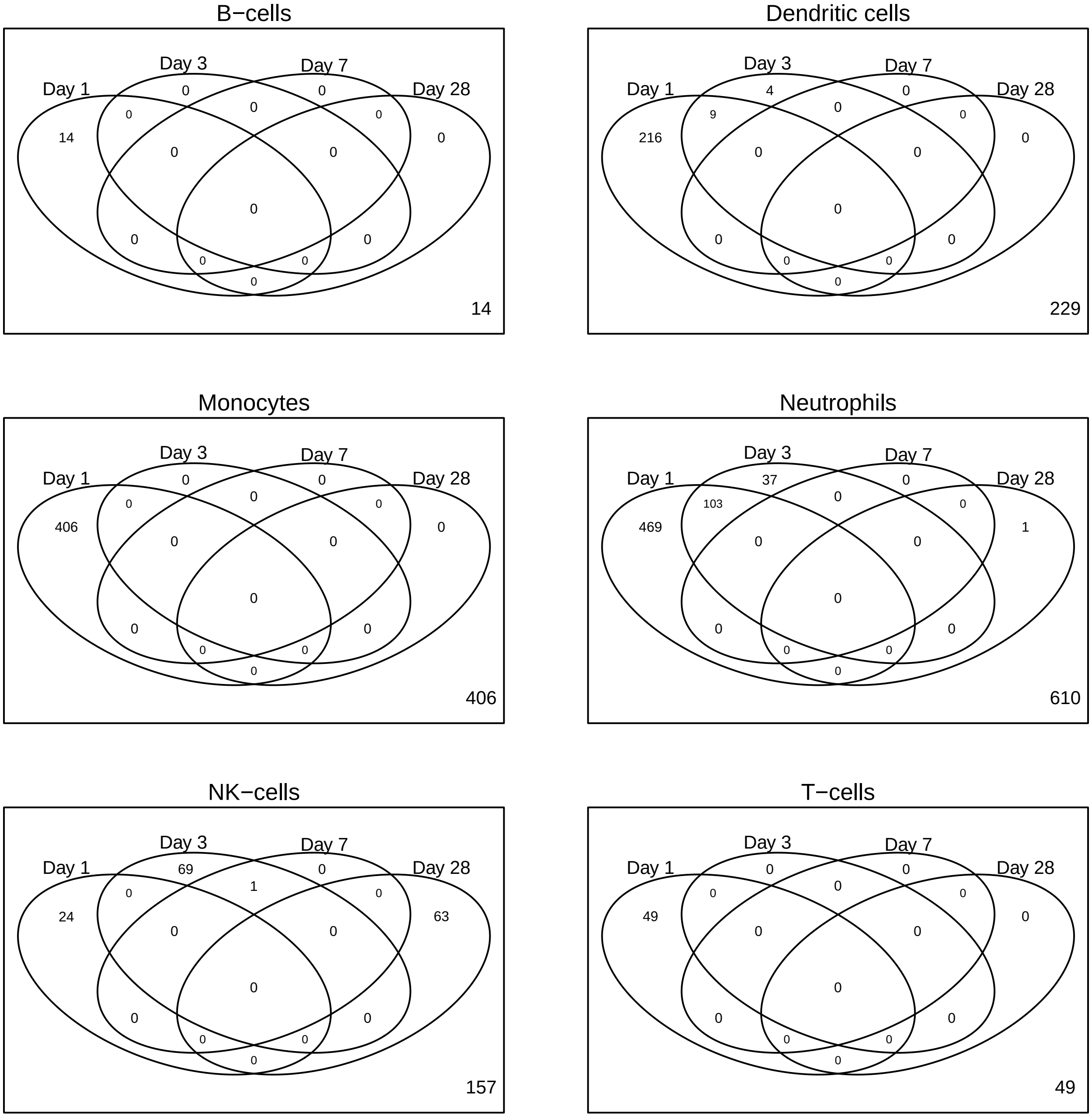

Figure A54: Venn diagrams of significant gene counts by day and cell type (RNA-Seq, after GC adjustment).

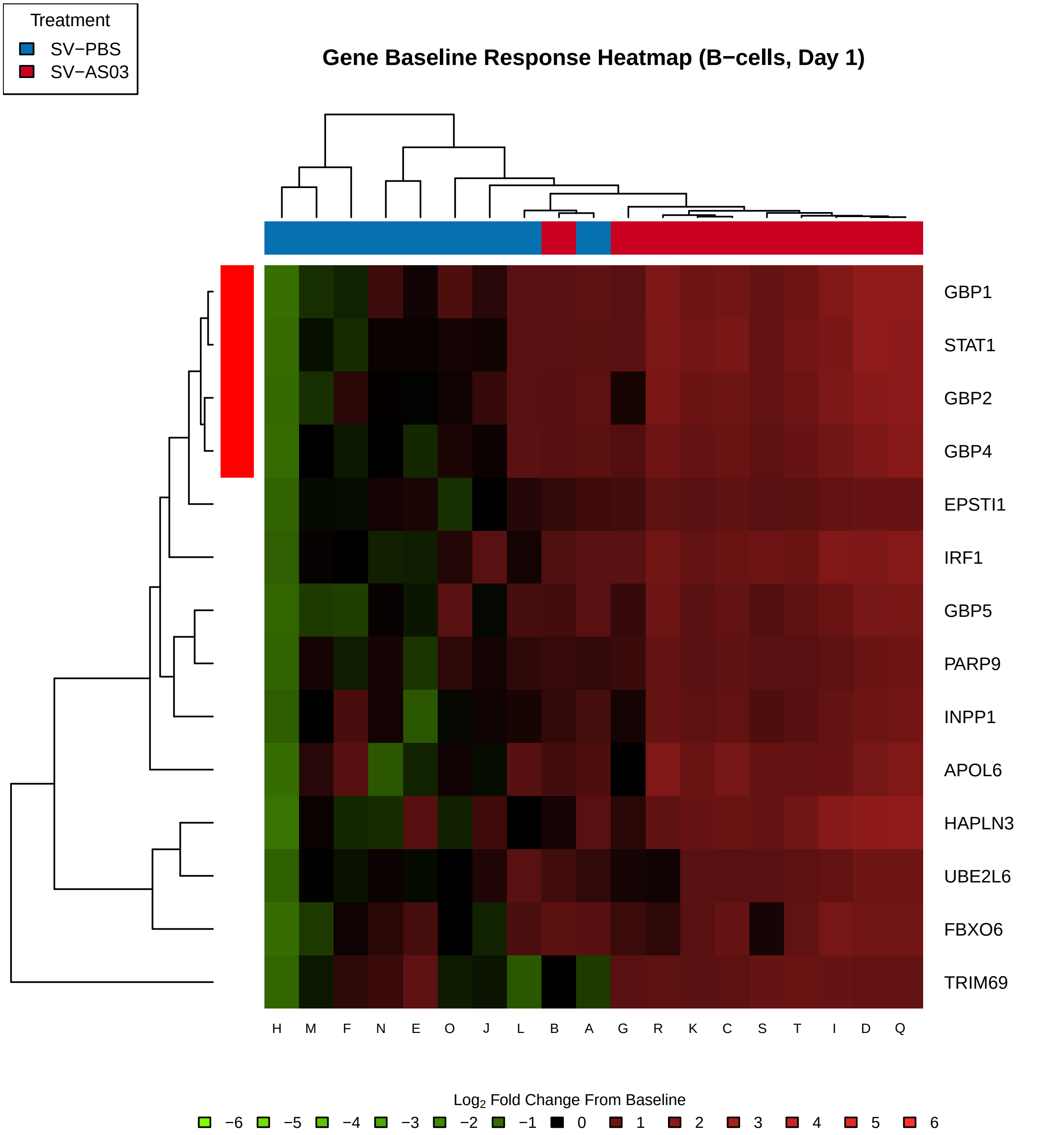

Figure A55: Heatmap of baseline log fold change (RNA-Seq, B-cells, Day 1). Colored in red: up regulated from baseline; colored in green: down regulated from baseline. Dendrograms were obtained using complete linkage clustering of uncentered pairwise Pearson correlation distances between log fold changes. Significant gene cluster membership is highlighted along the gene dendrogram on the left side; vaccine group membership is highlighted below the subject dendrogram.

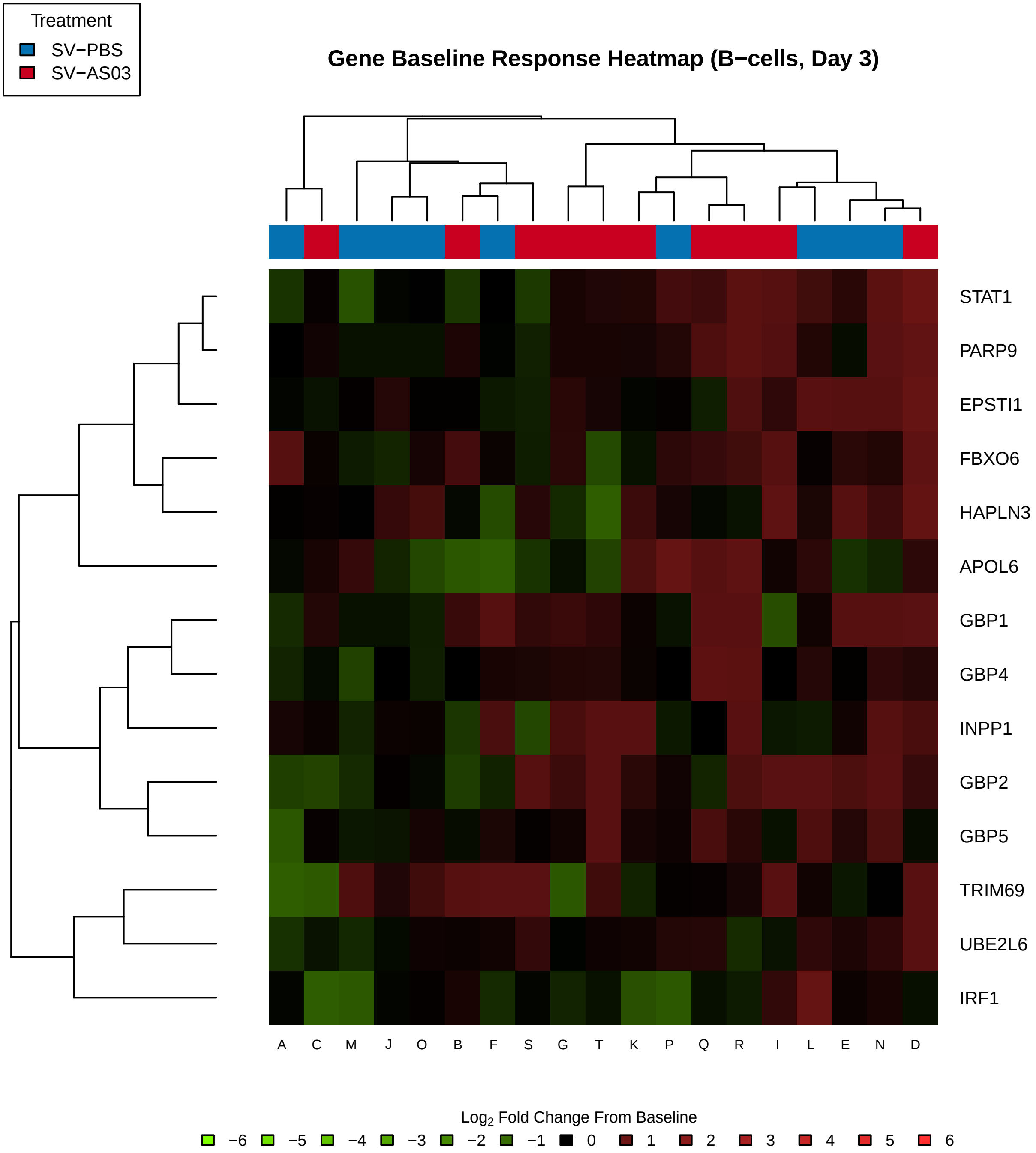

Figure A56: Heatmap of baseline log fold change (RNA-Seq, B-cells, Day 3). Colored in red: up regulated from baseline; colored in green: down regulated from baseline. Dendrograms were obtained using complete linkage clustering of uncentered pairwise Pearson correlation distances between log fold changes. Significant gene cluster membership is highlighted along the gene dendrogram on the left side; vaccine group membership is highlighted below the subject dendrogram.

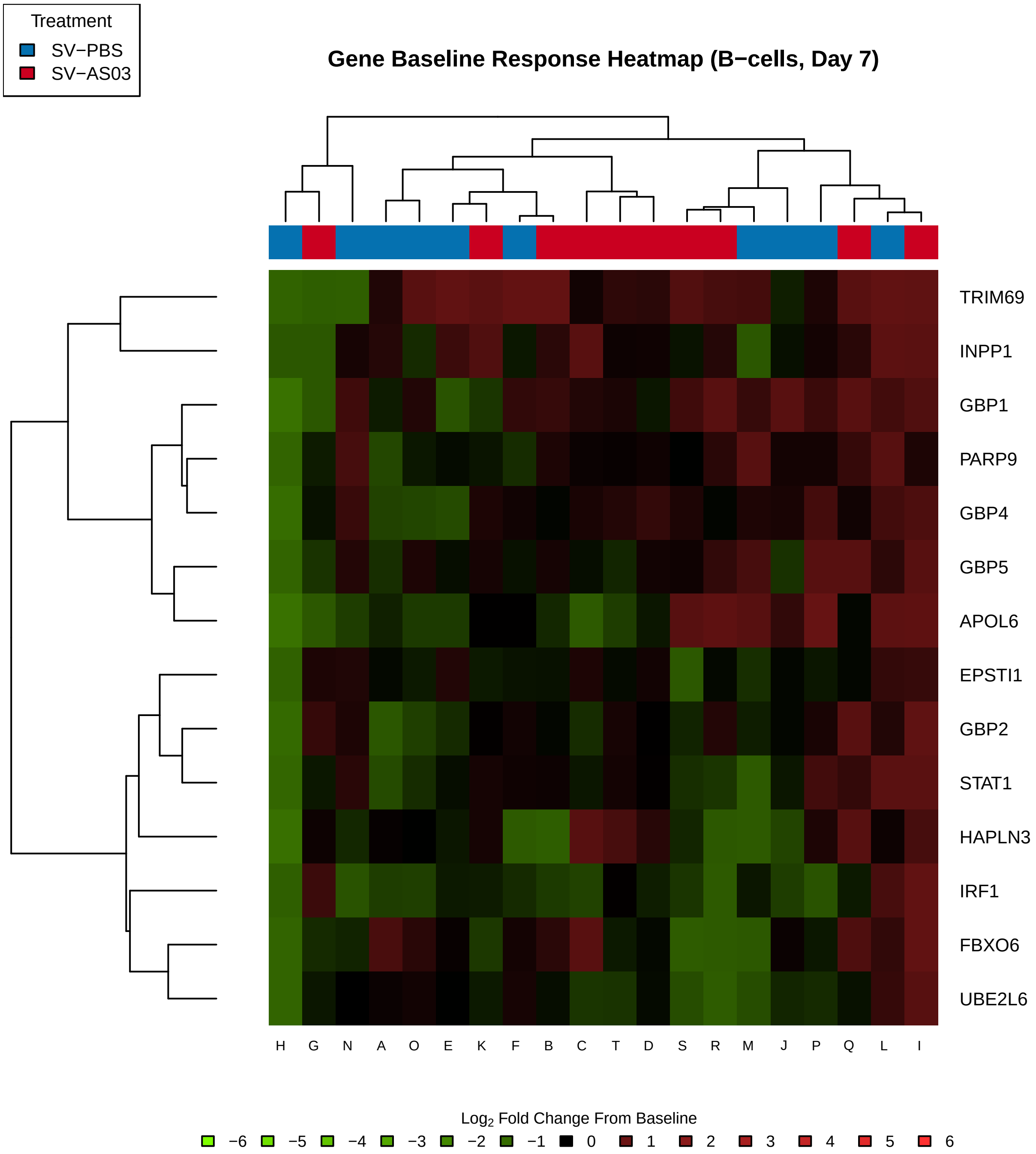

Figure A57: Heatmap of baseline log fold change (RNA-Seq, B-cells, Day 7). Colored in red: up regulated from baseline; colored in green: down regulated from baseline. Dendrograms were obtained using complete linkage clustering of uncentered pairwise Pearson correlation distances between log fold changes. Significant gene cluster membership is highlighted along the gene dendrogram on the left side; vaccine group membership is highlighted below the subject dendrogram.

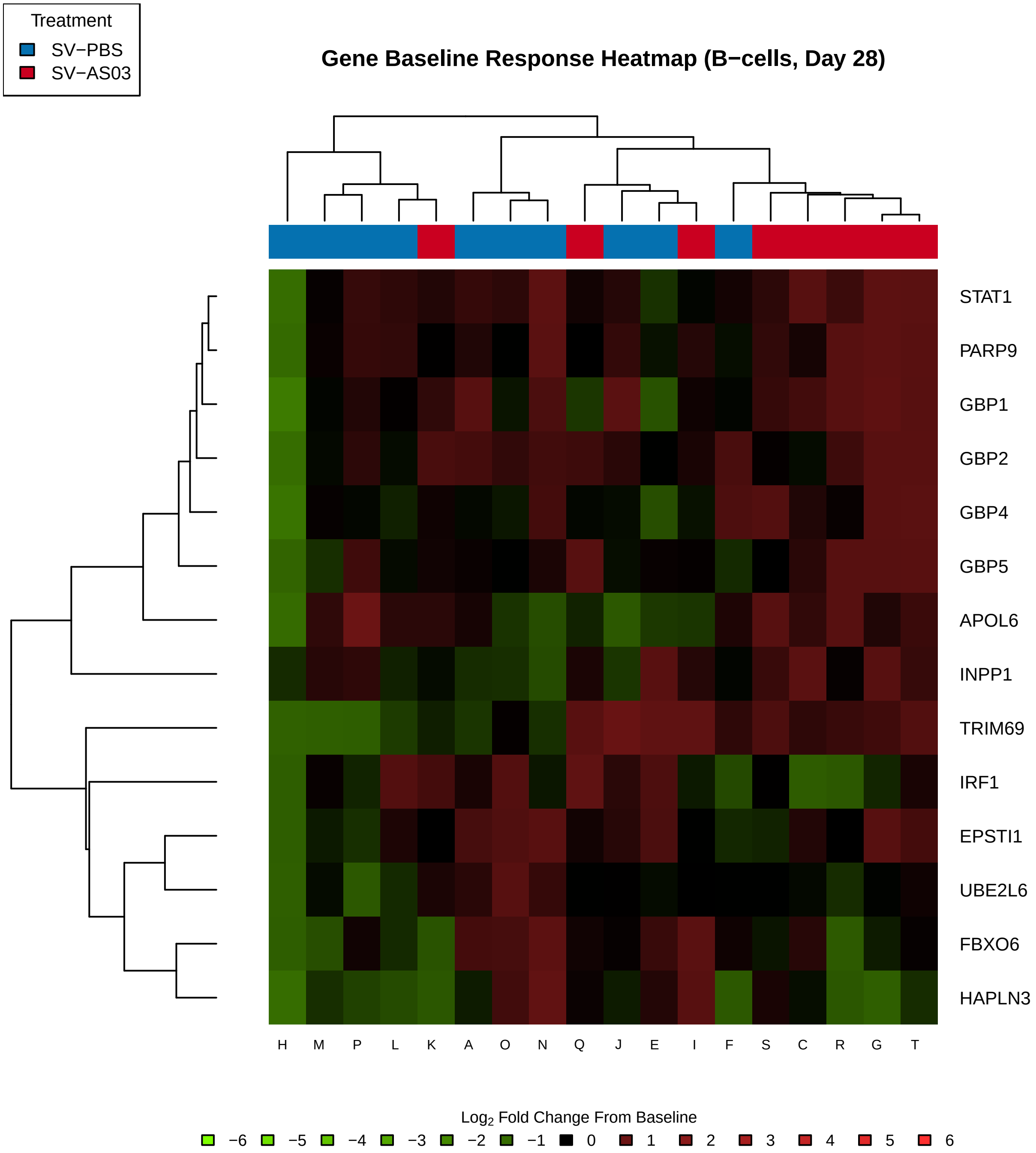

Figure A58: Heatmap of baseline log fold change (RNA-Seq, B-cells, Day 28). Colored in red: up regulated from baseline; colored in green: down regulated from baseline. Dendrograms were obtained using complete linkage clustering of uncentered pairwise Pearson correlation distances between log fold changes. Significant gene cluster membership is highlighted along the gene dendrogram on the left side; vaccine group membership is highlighted below the subject dendrogram.

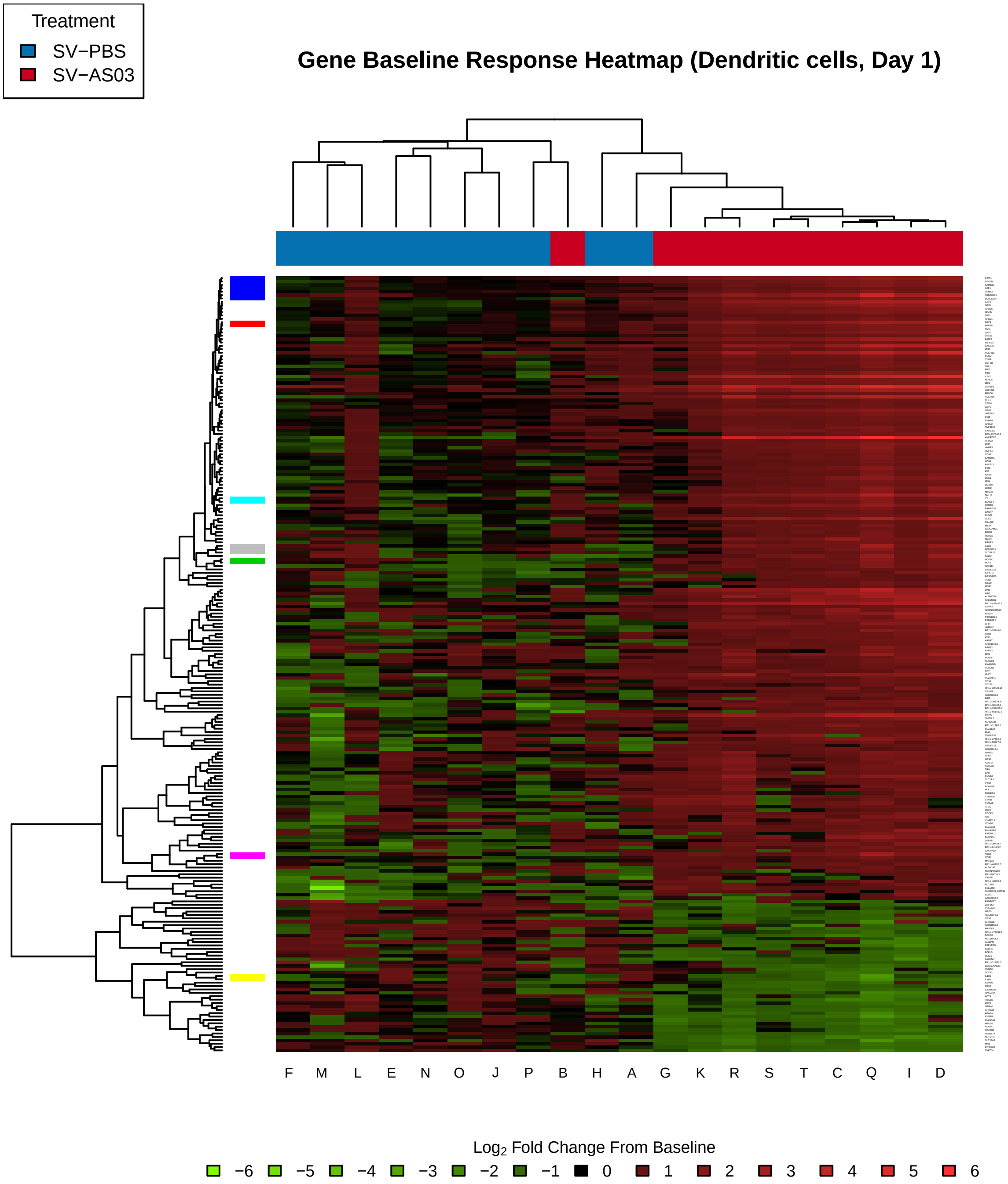

Figure A59: Heatmap of baseline log fold change (RNA-Seq, Dendritic cells, Day 1). Colored in red: up regulated from baseline; colored in green: down regulated from baseline. Dendrograms were obtained using complete linkage clustering of uncentered pairwise Pearson correlation distances between log fold changes. Significant gene cluster membership is highlighted along the gene dendrogram on the left side; vaccine group membership is highlighted below the subject dendrogram.

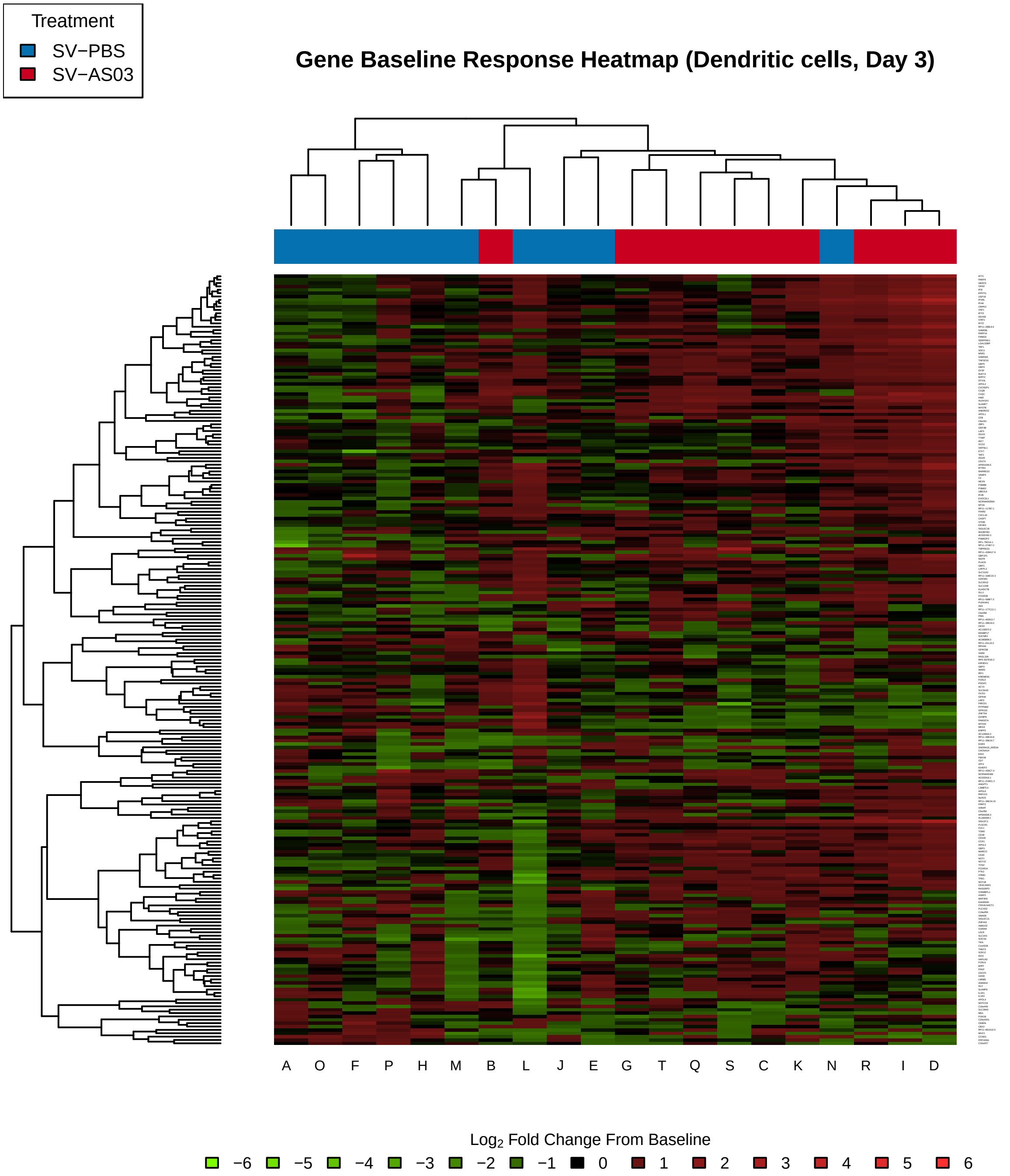

Figure A60: Heatmap of baseline log fold change (RNA-Seq, Dendritic cells, Day 3). Colored in red: up regulated from baseline; colored in green: down regulated from baseline. Dendrograms were obtained using complete linkage clustering of uncentered pairwise Pearson correlation distances between log fold changes. Significant gene cluster membership is highlighted along the gene dendrogram on the left side; vaccine group membership is highlighted below the subject dendrogram.

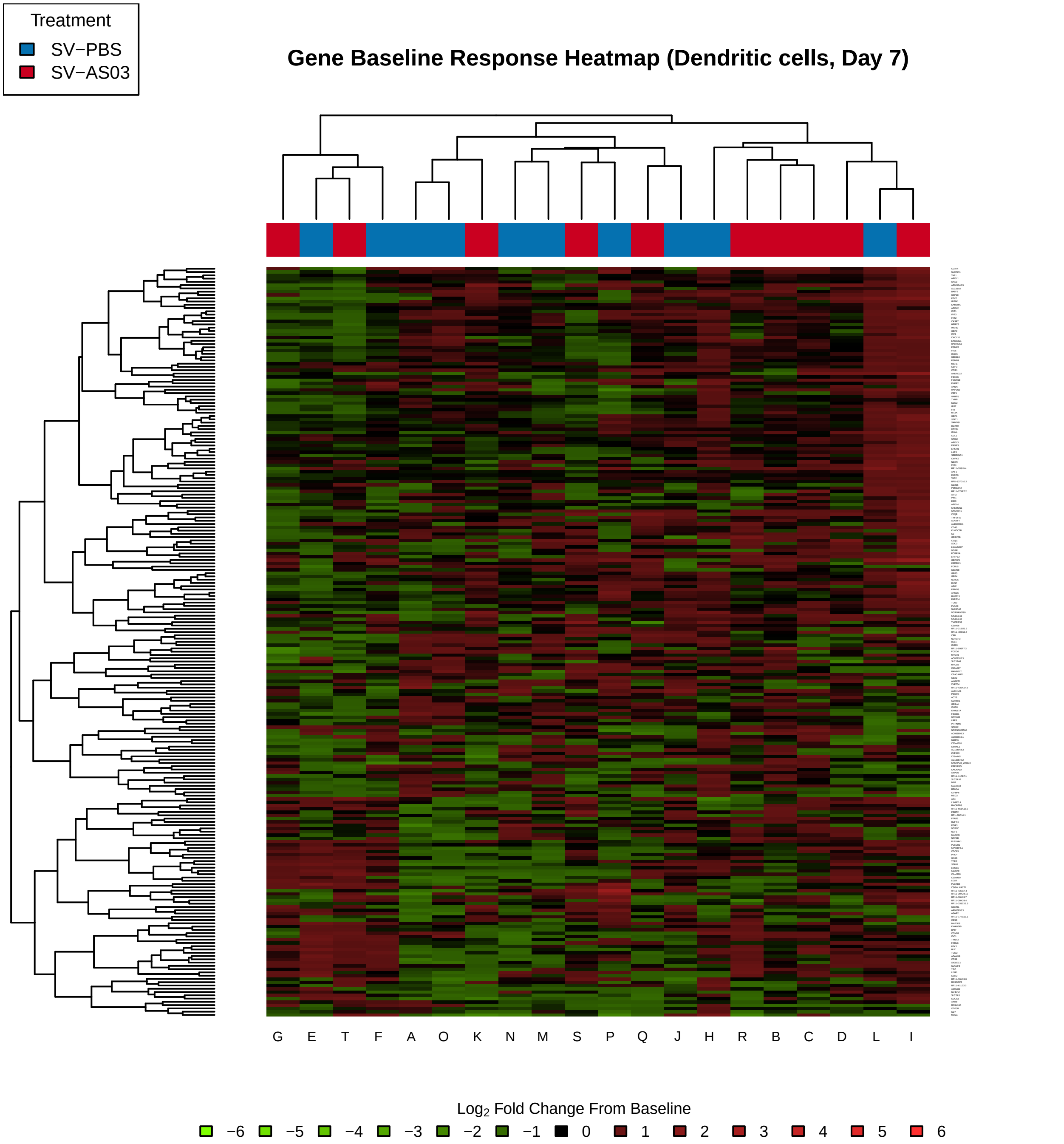

Figure A61: Heatmap of baseline log fold change (RNA-Seq, Dendritic cells, Day 7). Colored in red: up regulated from baseline; colored in green: down regulated from baseline. Dendrograms were obtained using complete linkage clustering of uncentered pairwise Pearson correlation distances between log fold changes. Significant gene cluster membership is highlighted along the gene dendrogram on the left side; vaccine group membership is highlighted below the subject dendrogram.

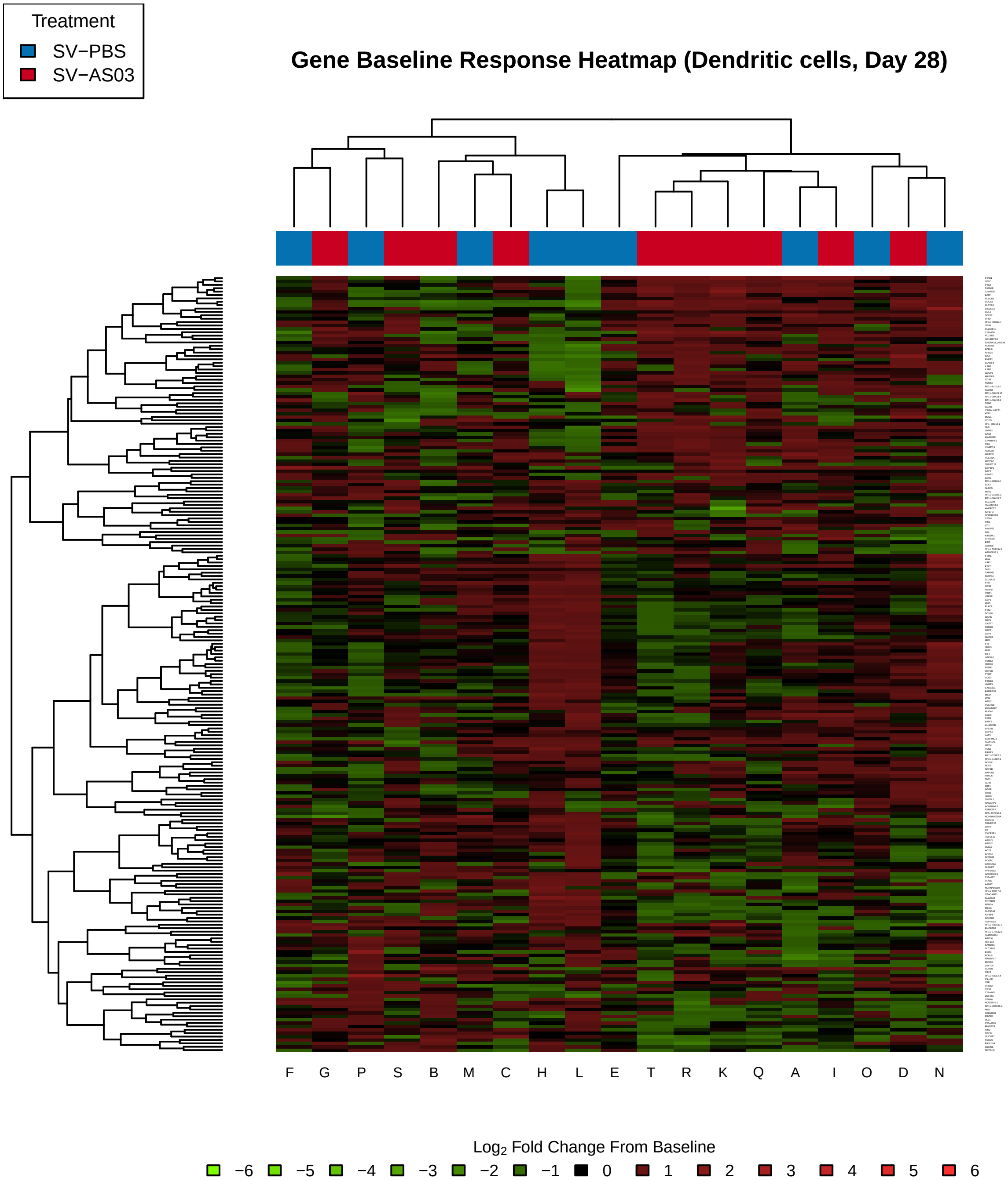

Figure A62: Heatmap of baseline log fold change (RNA-Seq, Dendritic cells, Day 28). Colored in red: up regulated from baseline; colored in green: down regulated from baseline. Dendrograms were obtained using complete linkage clustering of uncentered pairwise Pearson correlation distances between log fold changes. Significant gene cluster membership is highlighted along the gene dendrogram on the left side; vaccine group membership is highlighted below the subject dendrogram.

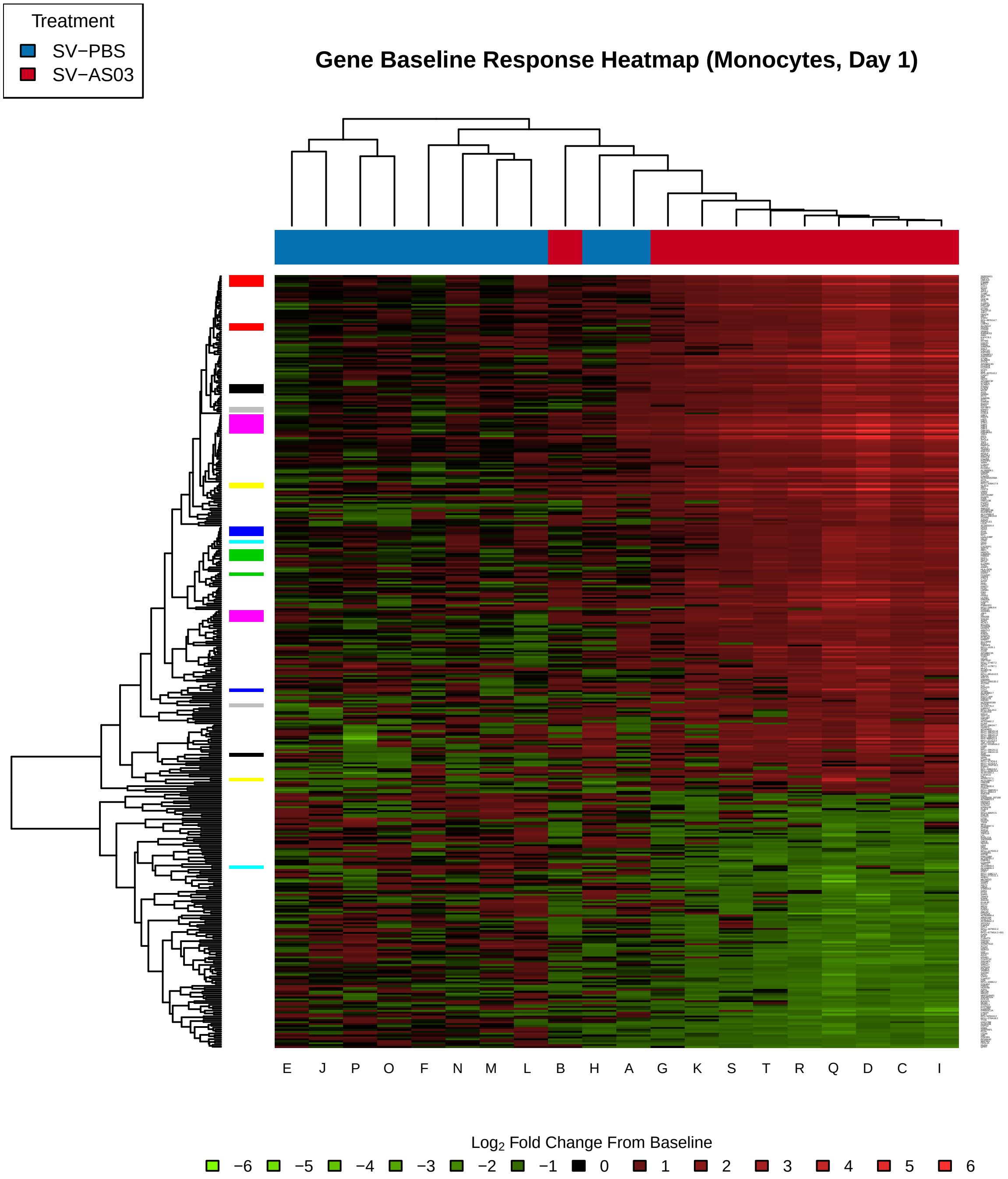

Figure A63: Heatmap of baseline log fold change (RNA-Seq, Monocytes, Day 1). Colored in red: up regulated from baseline; colored in green: down regulated from baseline. Dendrograms were obtained using complete linkage clustering of uncentered pairwise Pearson correlation distances between log fold changes. Significant gene cluster membership is highlighted along the gene dendrogram on the left side; vaccine group membership is highlighted below the subject dendrogram.

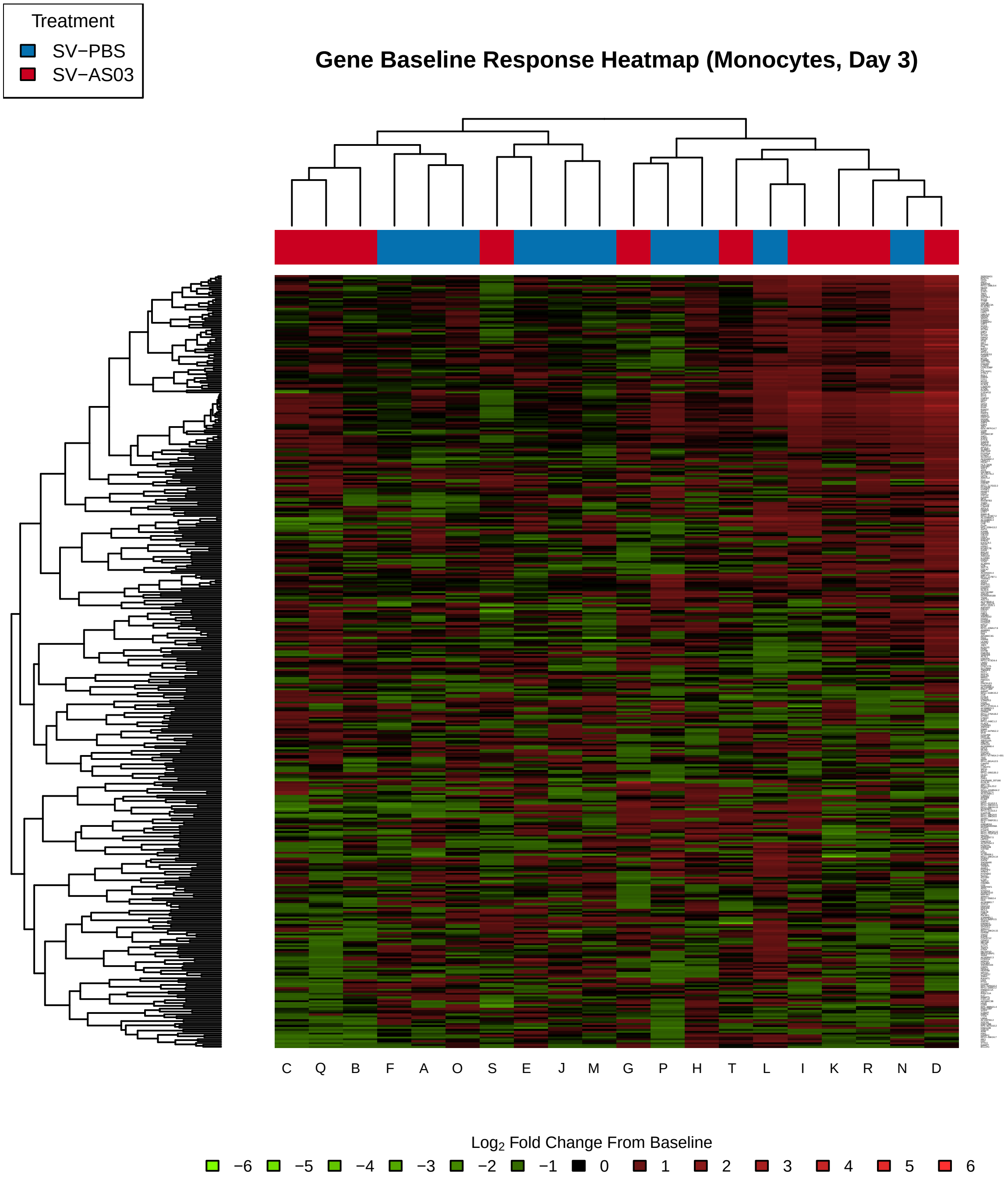

Figure A64: Heatmap of baseline log fold change (RNA-Seq, Monocytes, Day 3). Colored in red: up regulated from baseline; colored in green: down regulated from baseline. Dendrograms were obtained using complete linkage clustering of uncentered pairwise Pearson correlation distances between log fold changes. Significant gene cluster membership is highlighted along the gene dendrogram on the left side; vaccine group membership is highlighted below the subject dendrogram.

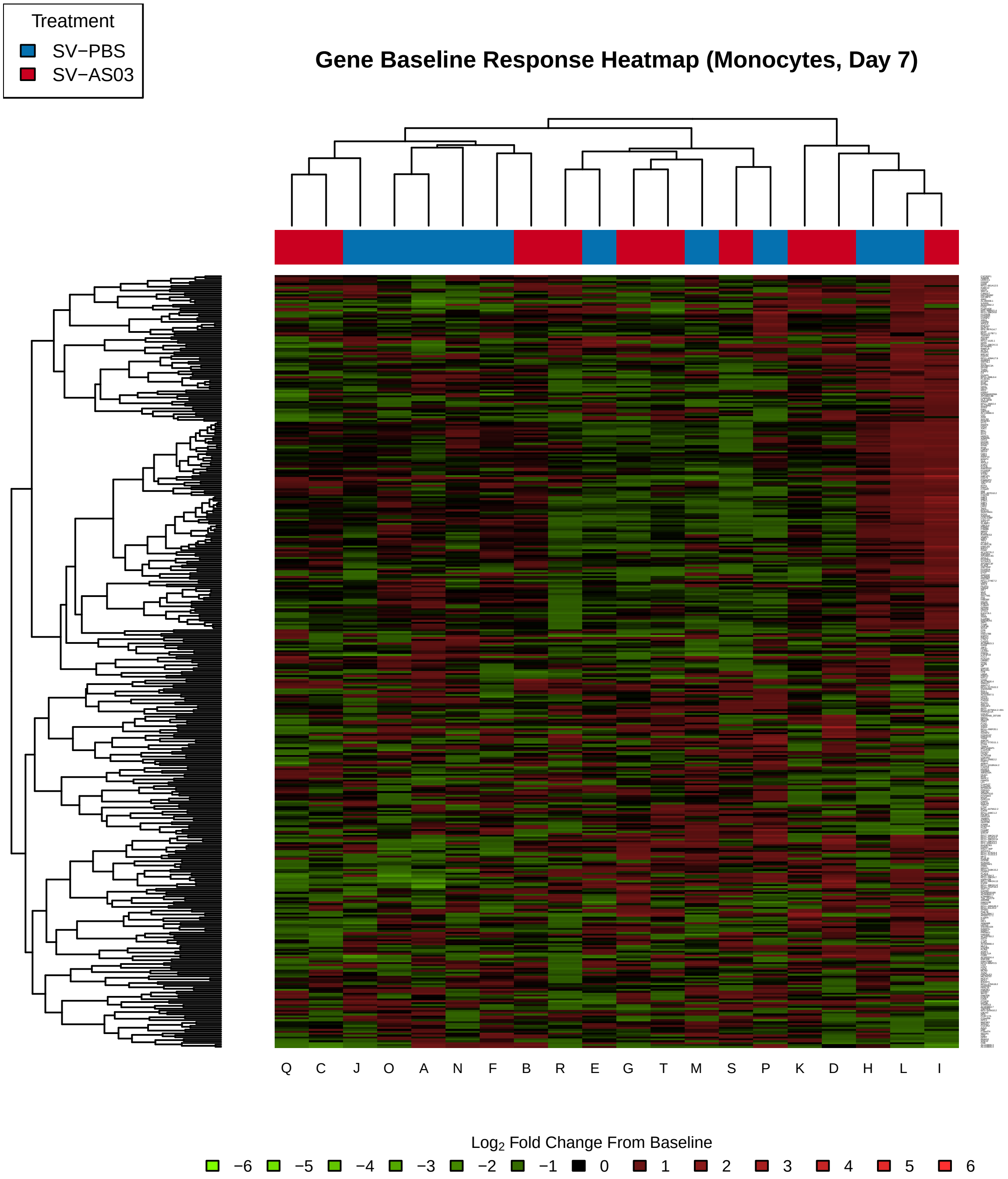

Figure A65: Heatmap of baseline log fold change (RNA-Seq, Monocytes, Day 7). Colored in red: up regulated from baseline; colored in green: down regulated from baseline. Dendrograms were obtained using complete linkage clustering of uncentered pairwise Pearson correlation distances between log fold changes. Significant gene cluster membership is highlighted along the gene dendrogram on the left side; vaccine group membership is highlighted below the subject dendrogram.

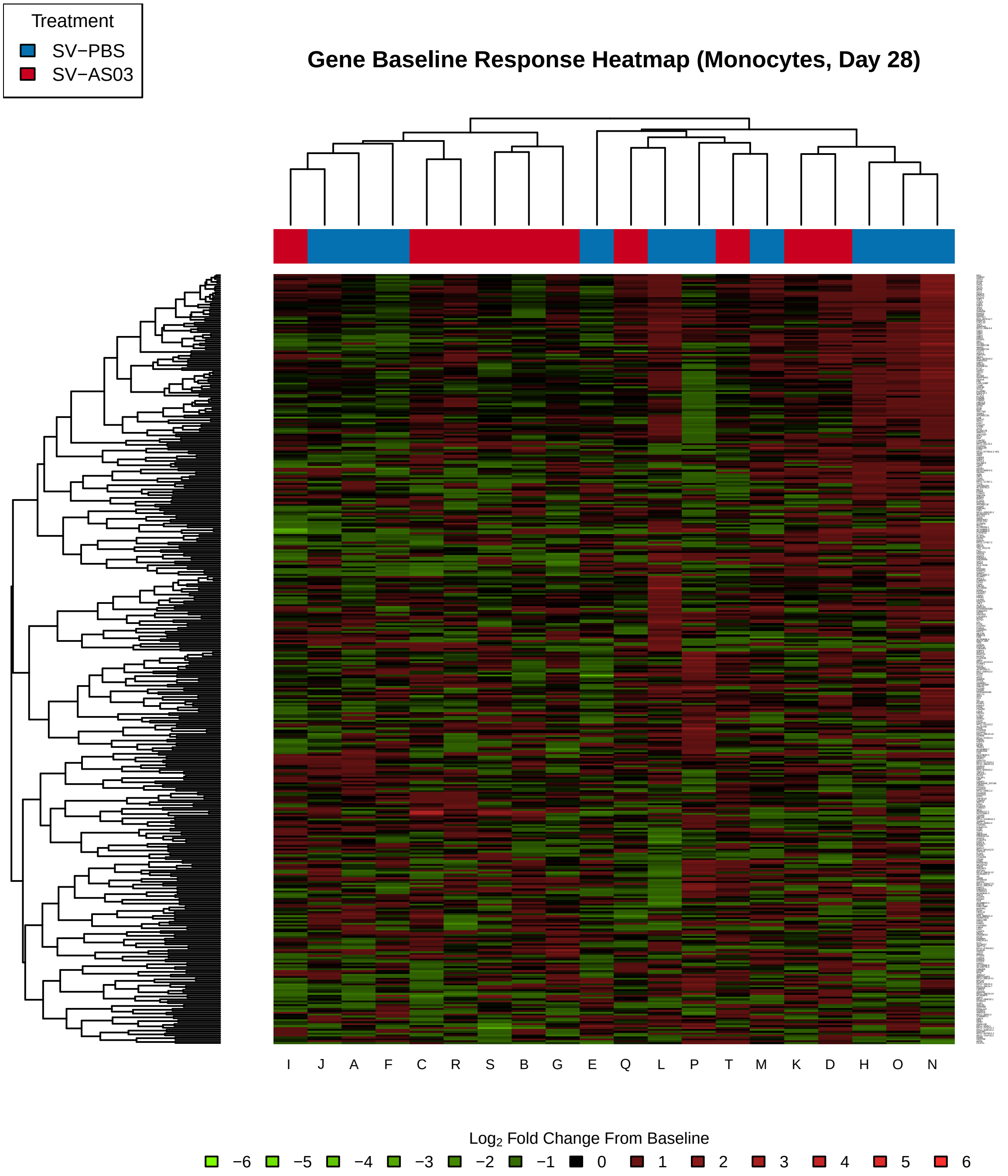

Figure A66: Heatmap of baseline log fold change (RNA-Seq, Monocytes, Day 28). Colored in red: up regulated from baseline; colored in green: down regulated from baseline. Dendrograms were obtained using complete linkage clustering of uncentered pairwise Pearson correlation distances between log fold changes. Significant gene cluster membership is highlighted along the gene dendrogram on the left side; vaccine group membership is highlighted below the subject dendrogram.

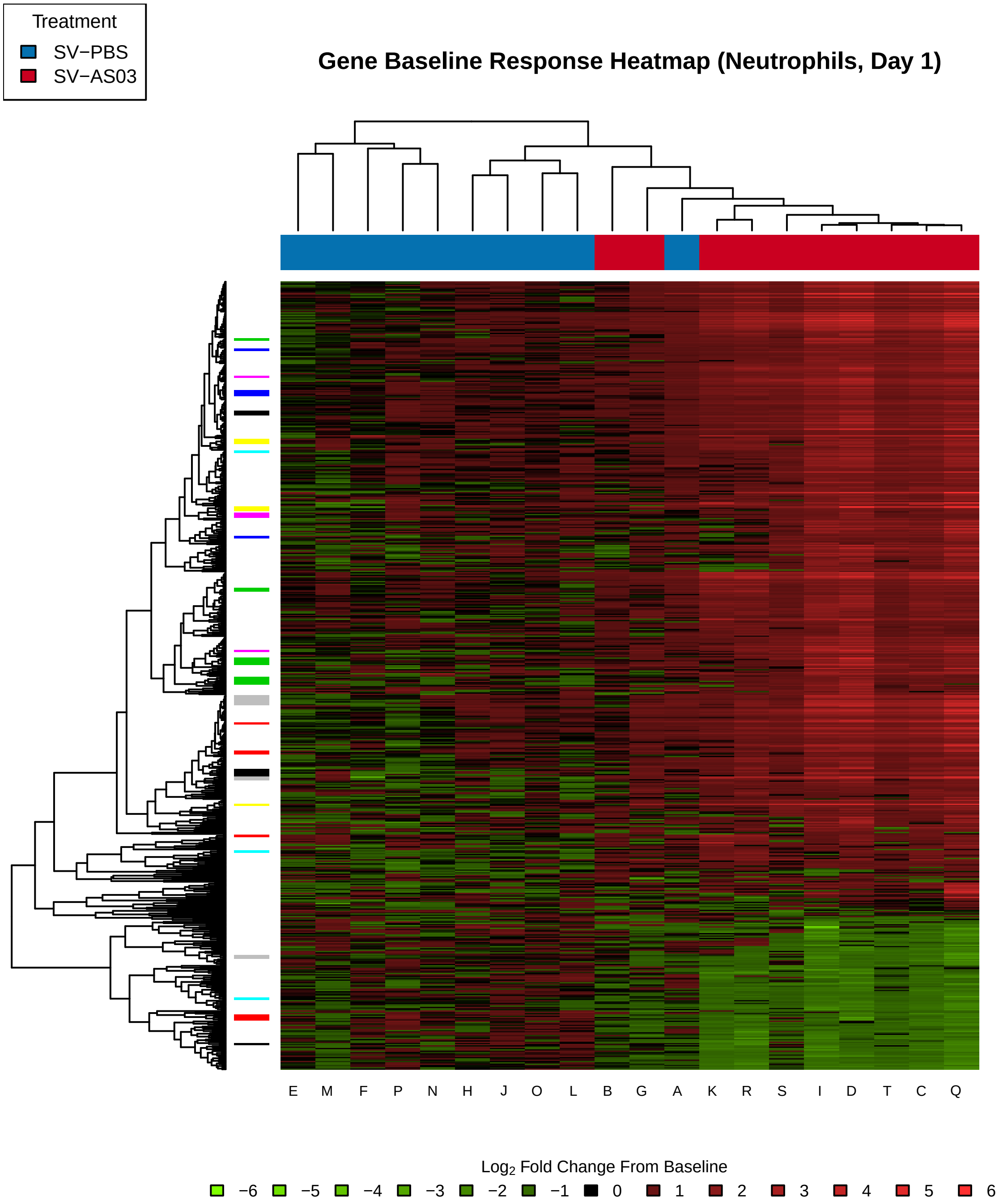

Figure A67: Heatmap of baseline log fold change (RNA-Seq, Neutrophils, Day 1). Colored in red: up regulated from baseline; colored in green: down regulated from baseline. Dendrograms were obtained using complete linkage clustering of uncentered pairwise Pearson correlation distances between log fold changes. Significant gene cluster membership is highlighted along the gene dendrogram on the left side; vaccine group membership is highlighted below the subject dendrogram.

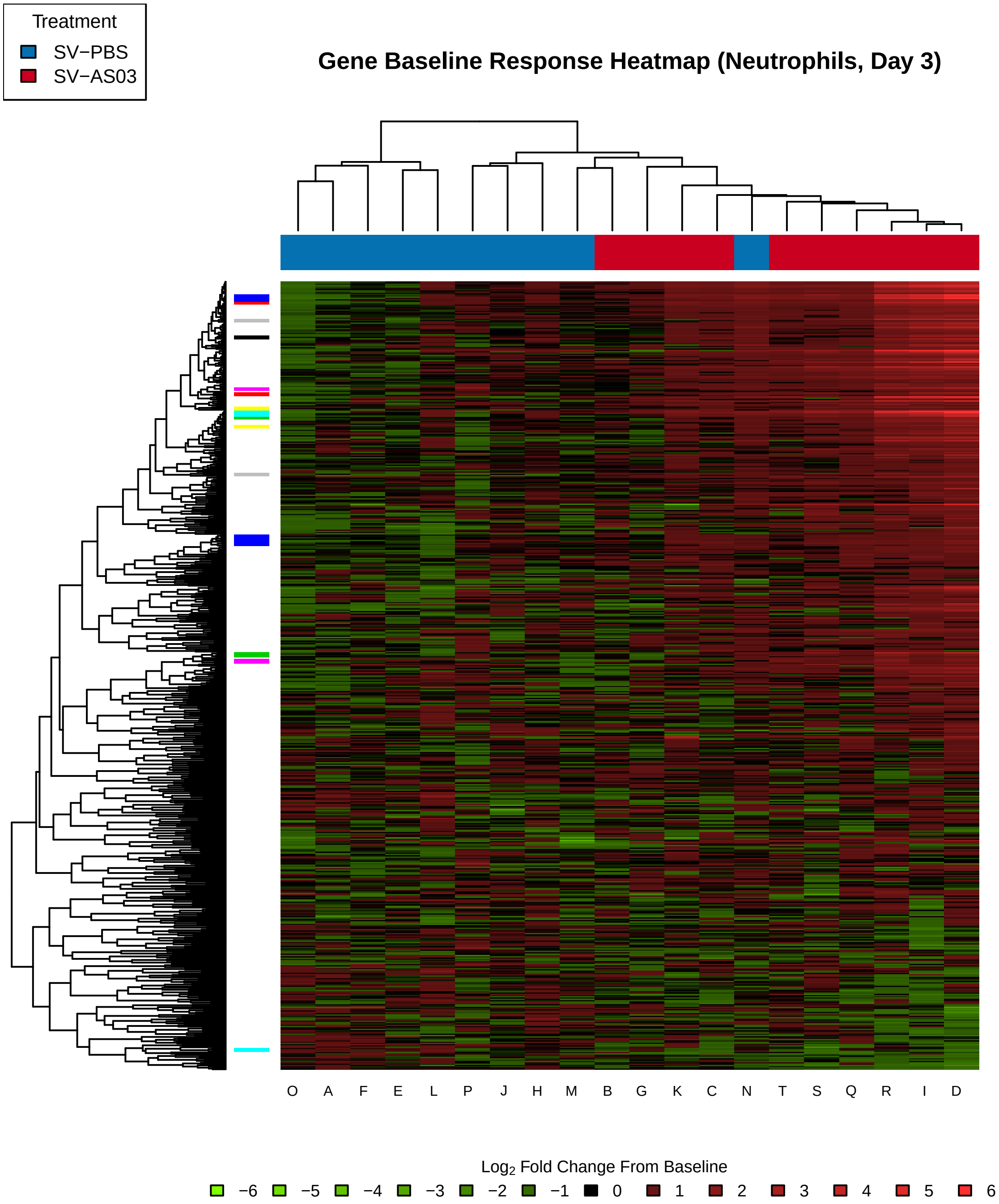

Figure A68: Heatmap of baseline log fold change (RNA-Seq, Neutrophils, Day 3). Colored in red: up regulated from baseline; colored in green: down regulated from baseline. Dendrograms were obtained using complete linkage clustering of uncentered pairwise Pearson correlation distances between log fold changes. Significant gene cluster membership is highlighted along the gene dendrogram on the left side; vaccine group membership is highlighted below the subject dendrogram.

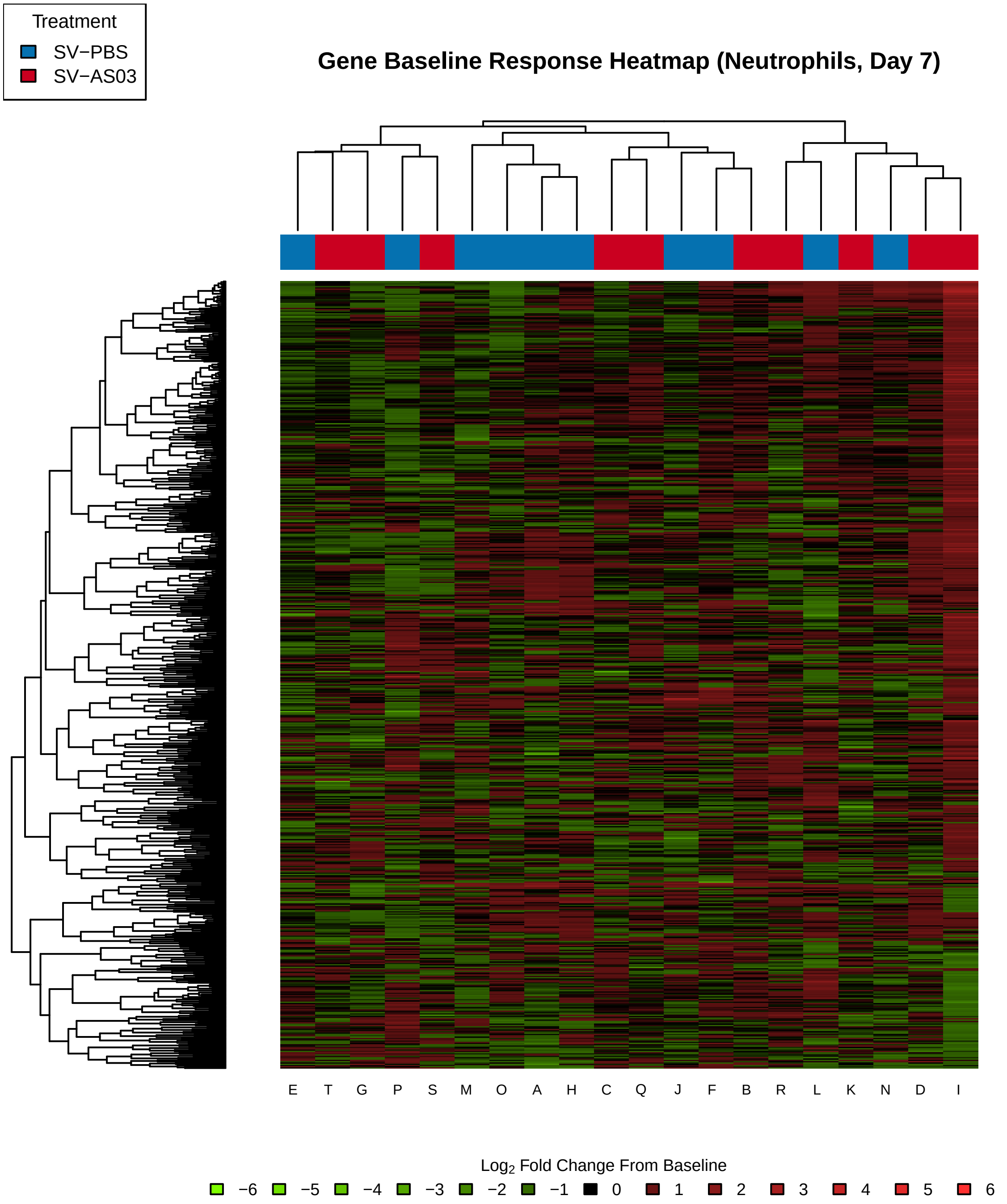

Figure A69: Heatmap of baseline log fold change (RNA-Seq, Neutrophils, Day 7). Colored in red: up regulated from baseline; colored in green: down regulated from baseline. Dendrograms were obtained using complete linkage clustering of uncentered pairwise Pearson correlation distances between log fold changes. Significant gene cluster membership is highlighted along the gene dendrogram on the left side; vaccine group membership is highlighted below the subject dendrogram.

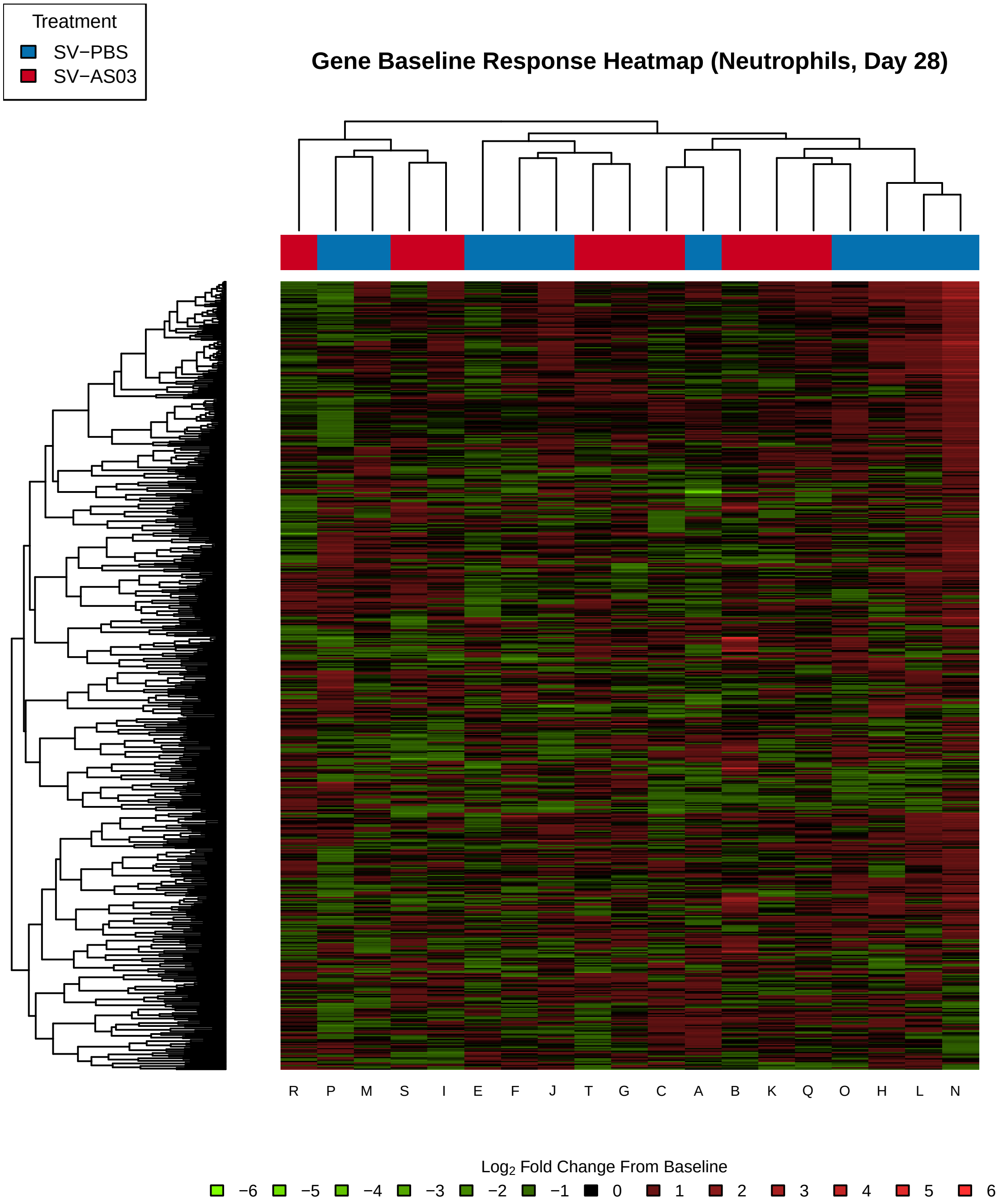

Figure A70: Heatmap of baseline log fold change (RNA-Seq, Neutrophils, Day 28). Colored in red: up regulated from baseline; colored in green: down regulated from baseline. Dendrograms were obtained using complete linkage clustering of uncentered pairwise Pearson correlation distances between log fold changes. Significant gene cluster membership is highlighted along the gene dendrogram on the left side; vaccine group membership is highlighted below the subject dendrogram.

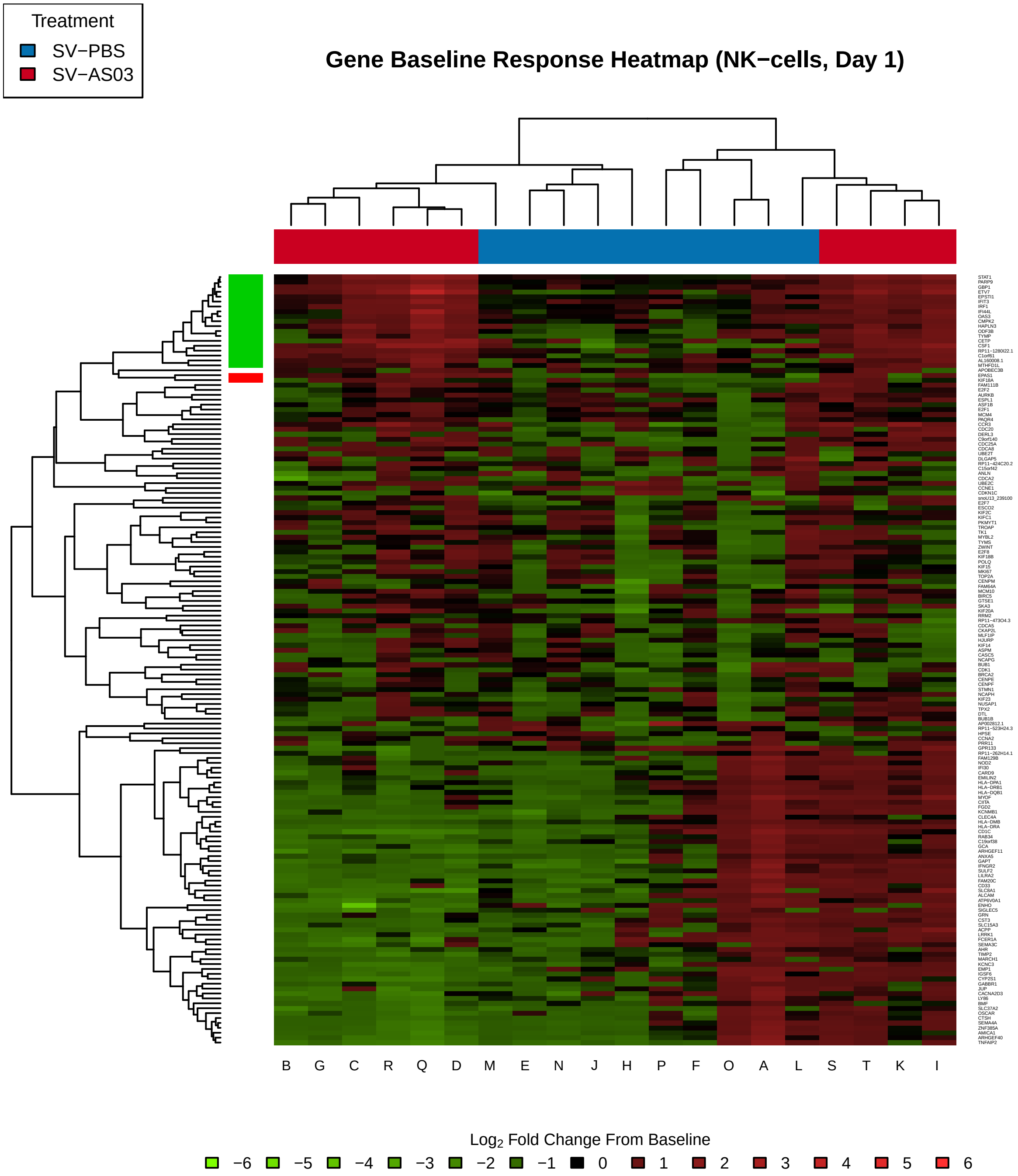

Figure A71: Heatmap of baseline log fold change (RNA-Seq, NK-cells, Day 1). Colored in red: up regulated from baseline; colored in green: down regulated from baseline. Dendrograms were obtained using complete linkage clustering of uncentered pairwise Pearson correlation distances between log fold changes. Significant gene cluster membership is highlighted along the gene dendrogram on the left side; vaccine group membership is highlighted below the subject dendrogram.

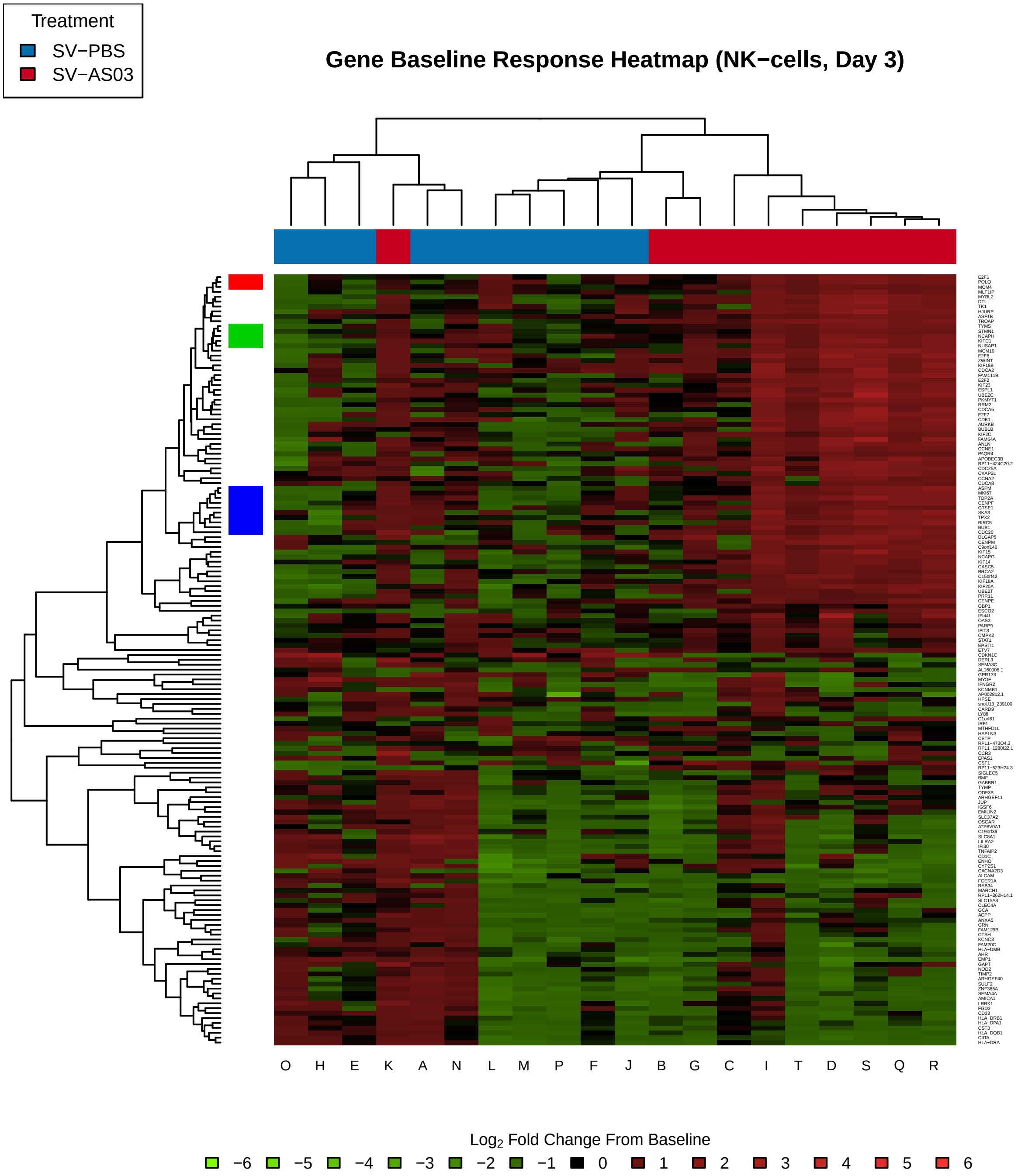

Figure A72: Heatmap of baseline log fold change (RNA-Seq, NK-cells, Day 3). Colored in red: up regulated from baseline; colored in green: down regulated from baseline. Dendrograms were obtained using complete linkage clustering of uncentered pairwise Pearson correlation distances between log fold changes. Significant gene cluster membership is highlighted along the gene dendrogram on the left side; vaccine group membership is highlighted below the subject dendrogram.

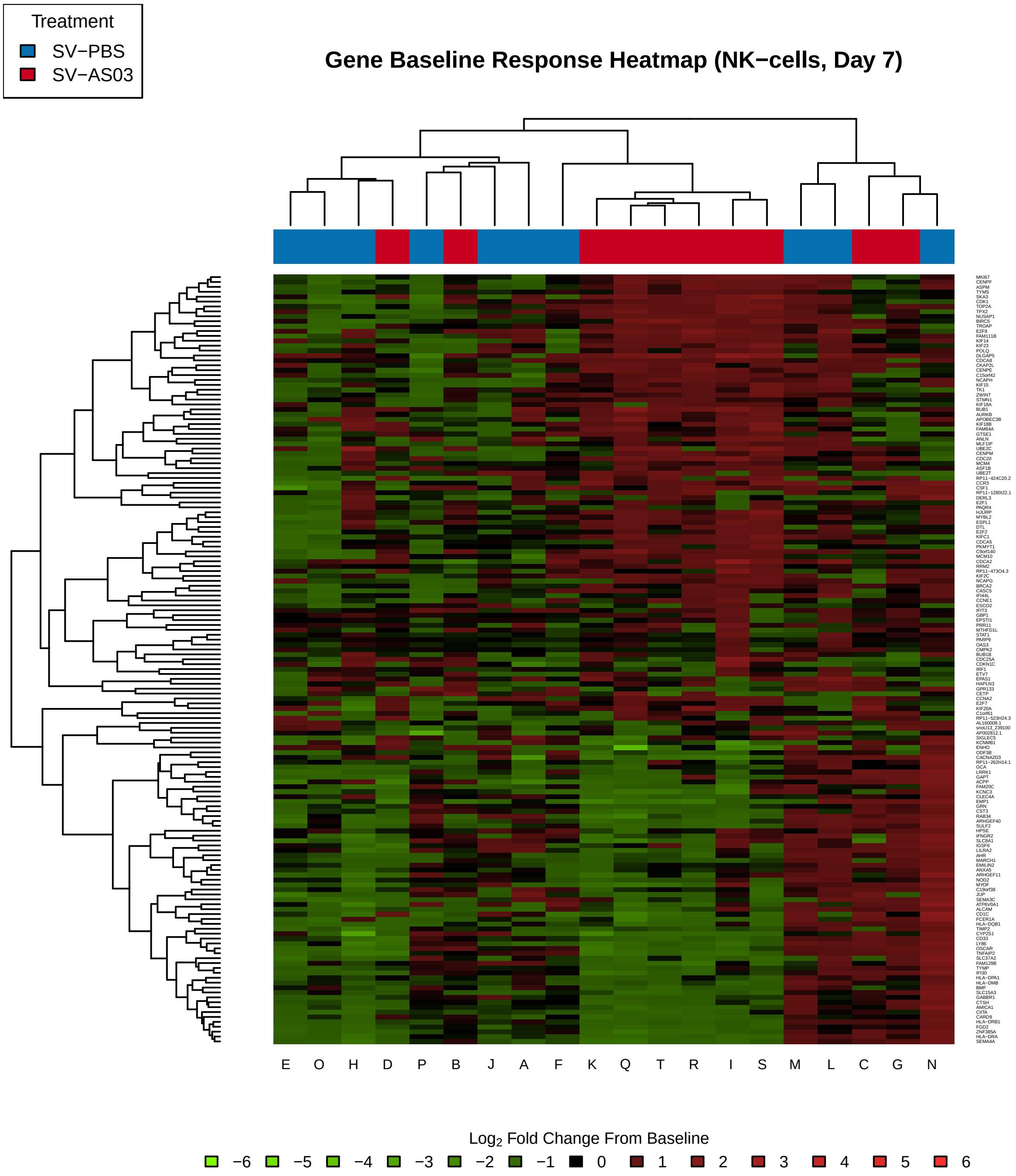

Figure A73: Heatmap of baseline log fold change (RNA-Seq, NK-cells, Day 7). Colored in red: up regulated from baseline; colored in green: down regulated from baseline. Dendrograms were obtained using complete linkage clustering of uncentered pairwise Pearson correlation distances between log fold changes. Significant gene cluster membership is highlighted along the gene dendrogram on the left side; vaccine group membership is highlighted below the subject dendrogram.

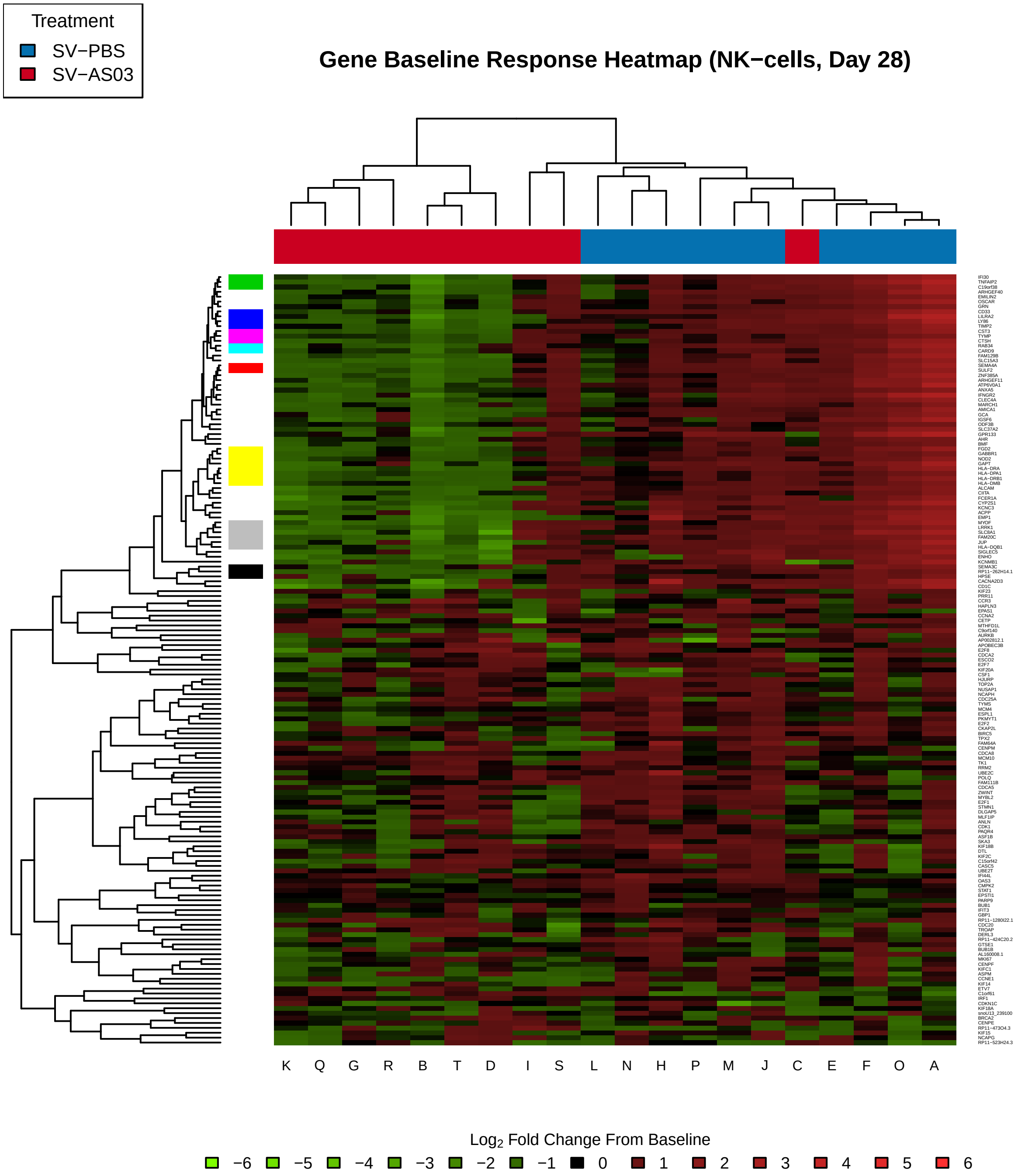

Figure A74: Heatmap of baseline log fold change (RNA-Seq, NK-cells, Day 28). Colored in red: up regulated from baseline; colored in green: down regulated from baseline. Dendrograms were obtained using complete linkage clustering of uncentered pairwise Pearson correlation distances between log fold changes. Significant gene cluster membership is highlighted along the gene dendrogram on the left side; vaccine group membership is highlighted below the subject dendrogram.

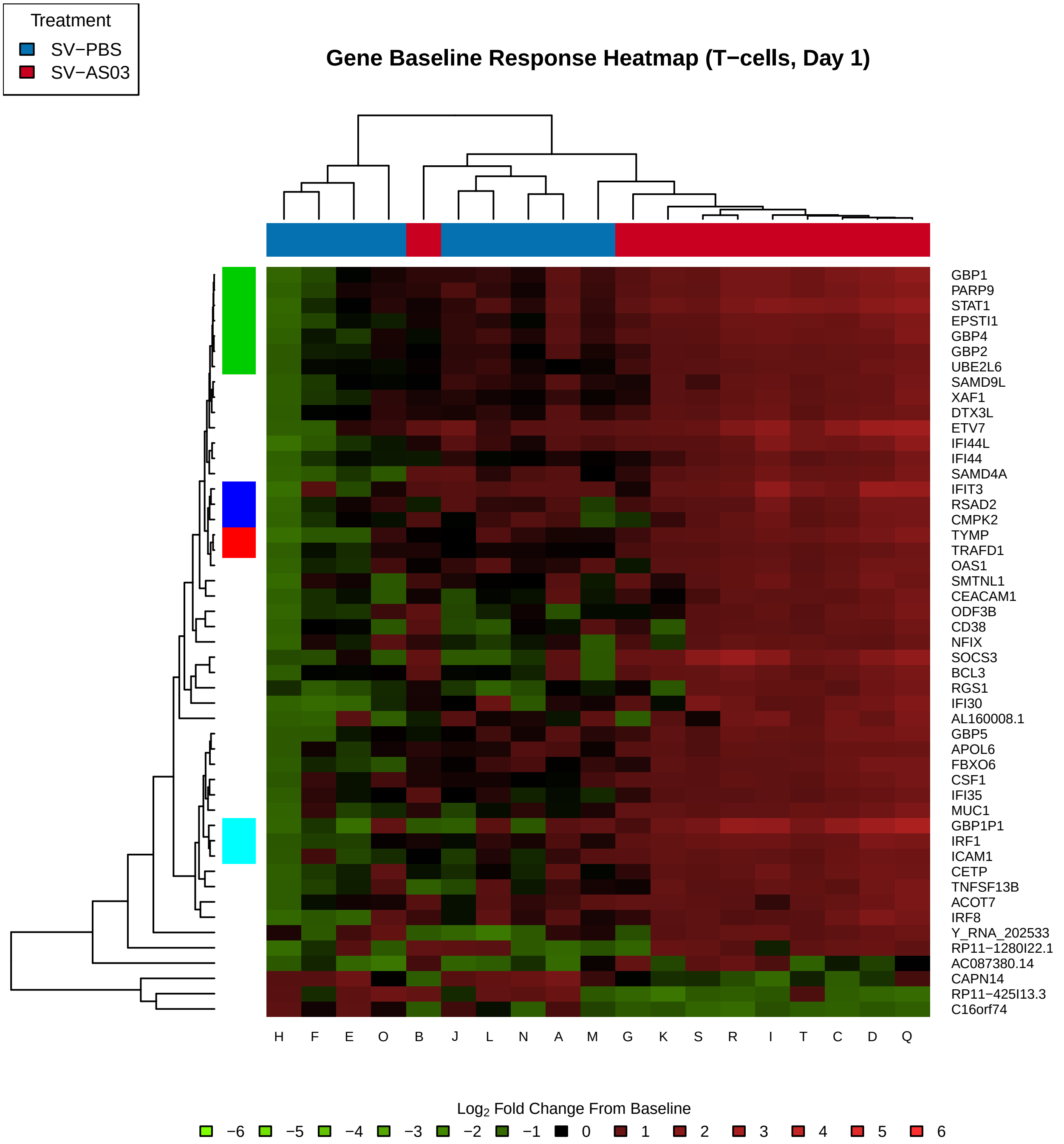

Figure A75: Heatmap of baseline log fold change (RNA-Seq, T-cells, Day 1). Colored in red: up regulated from baseline; colored in green: down regulated from baseline. Dendrograms were obtained using complete linkage clustering of uncentered pairwise Pearson correlation distances between log fold changes. Significant gene cluster membership is highlighted along the gene dendrogram on the left side; vaccine group membership is highlighted below the subject dendrogram.

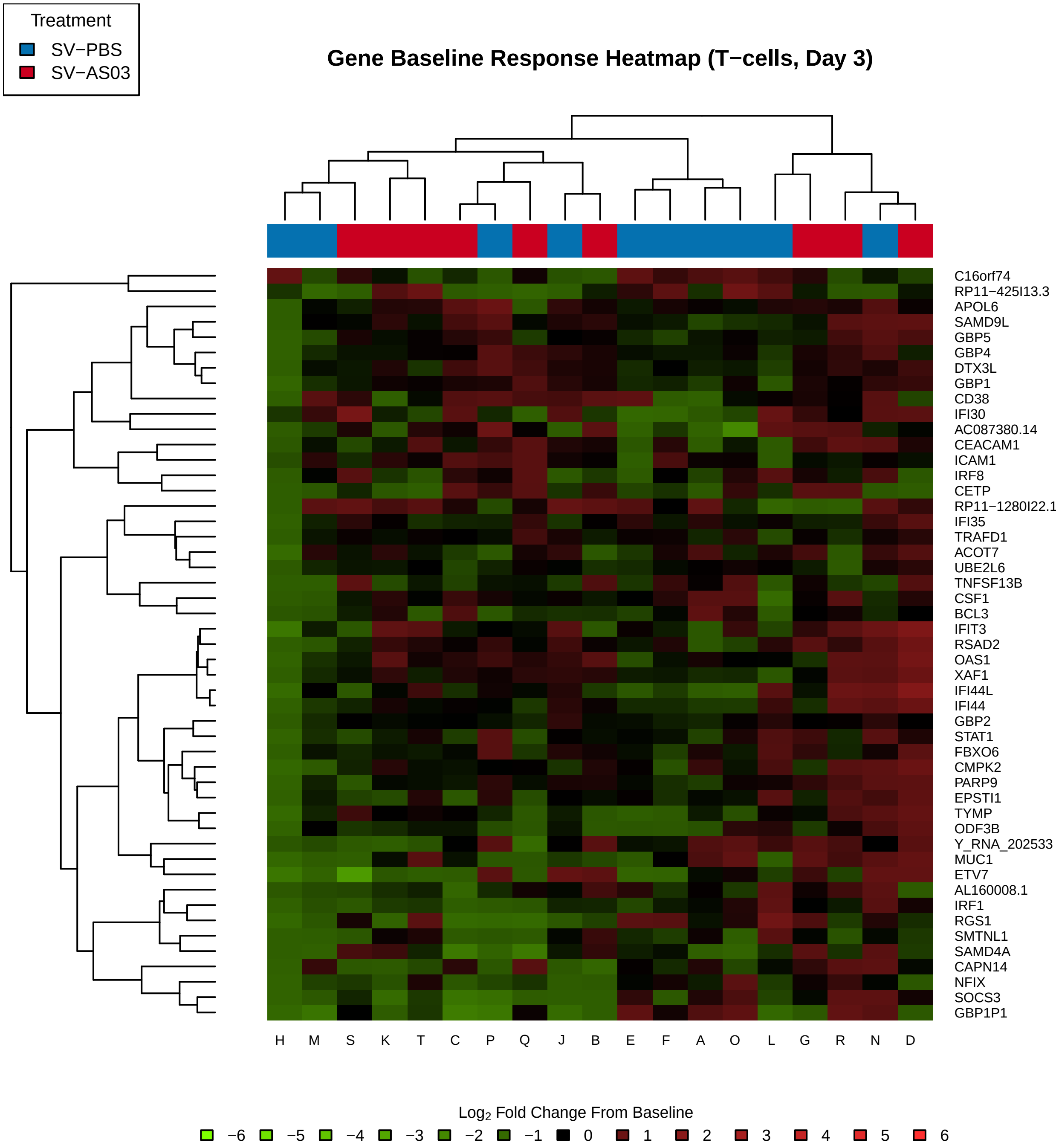

Figure A76: Heatmap of baseline log fold change (RNA-Seq, T-cells, Day 3). Colored in red: up regulated from baseline; colored in green: down regulated from baseline. Dendrograms were obtained using complete linkage clustering of uncentered pairwise Pearson correlation distances between log fold changes. Significant gene cluster membership is highlighted along the gene dendrogram on the left side; vaccine group membership is highlighted below the subject dendrogram.

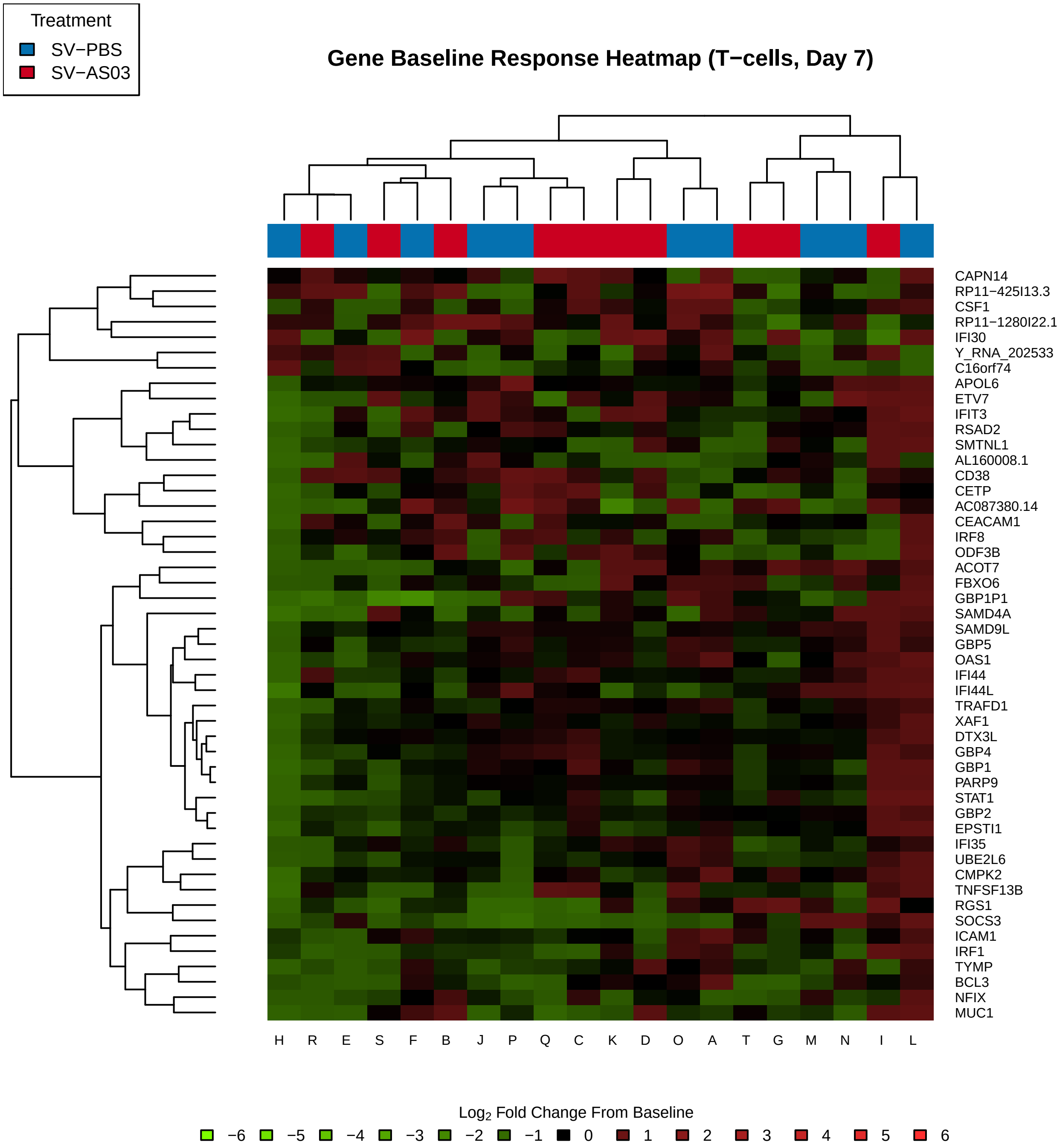

Figure A77: Heatmap of baseline log fold change (RNA-Seq, T-cells, Day 7). Colored in red: up regulated from baseline; colored in green: down regulated from baseline. Dendrograms were obtained using complete linkage clustering of uncentered pairwise Pearson correlation distances between log fold changes. Significant gene cluster membership is highlighted along the gene dendrogram on the left side; vaccine group membership is highlighted below the subject dendrogram.

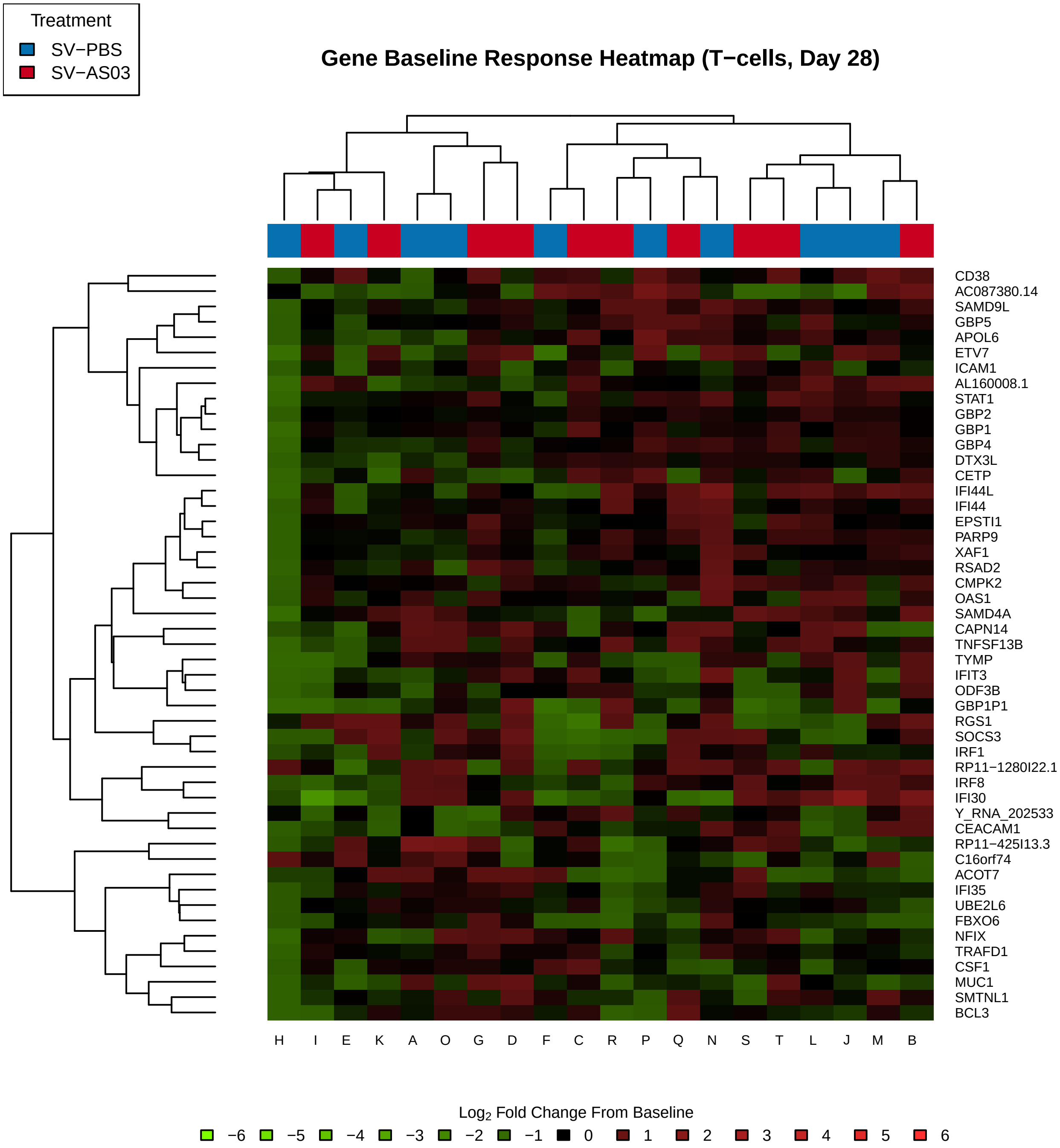

Figure A78: Heatmap of baseline log fold change (RNA-Seq, T-cells, Day 28). Colored in red: up regulated from baseline; colored in green: down regulated from baseline. Dendrograms were obtained using complete linkage clustering of uncentered pairwise Pearson correlation distances between log fold changes. Significant gene cluster membership is highlighted along the gene dendrogram on the left side; vaccine group membership is highlighted below the subject dendrogram.

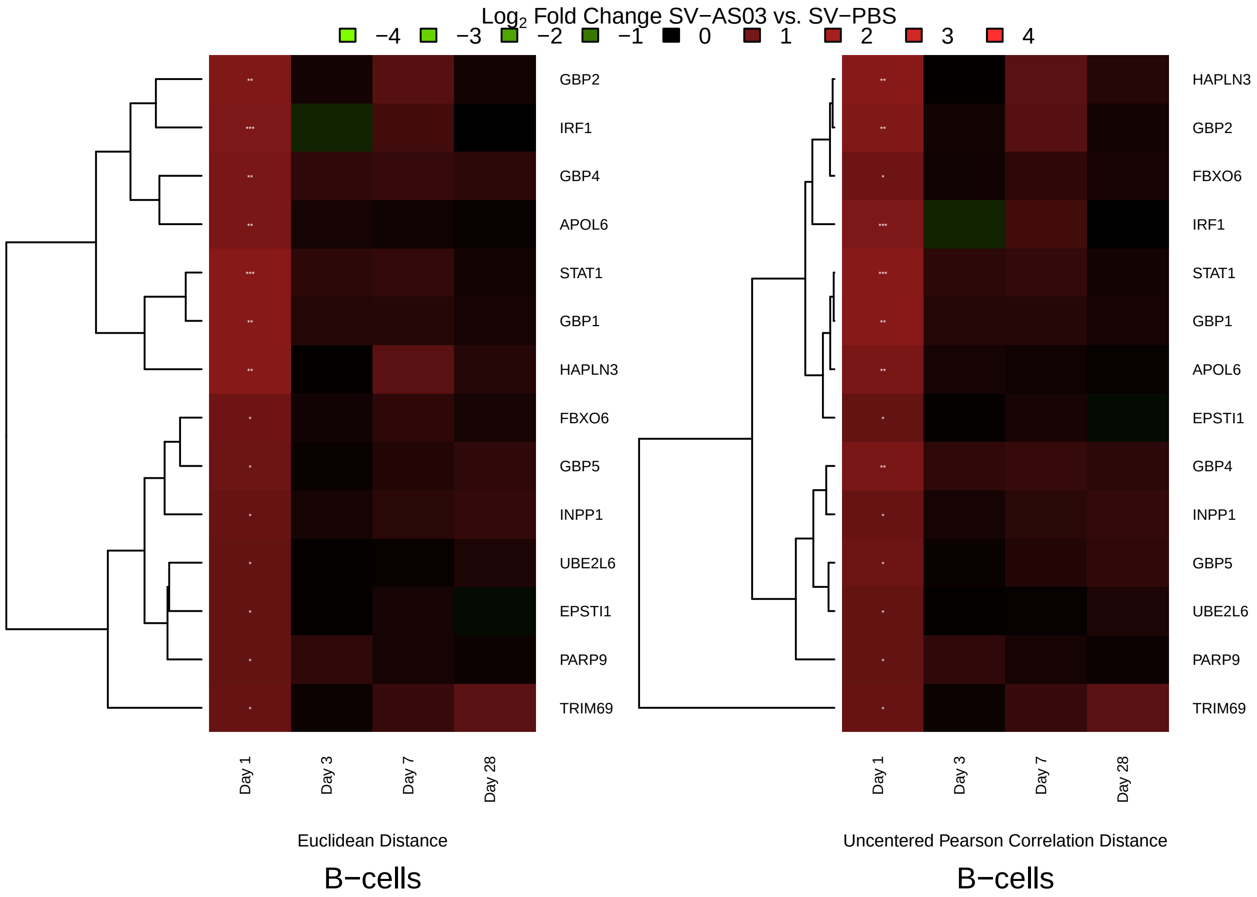

Figure A79: Heatmaps of GC adjusted log fold change (SV-AS03 vs. SV-PBS) over time (RNA-Seq, B-cells). Colored in red: up-regulated for SV-AS03; colored in green: down-regulated for SV-AS03. Asterisks mark genes that are significantly expressed at a certain day; FDR level (*:0.05, **:0.01, ***:0.001). Dendrograms were obtained using complete linkage clustering. Distances between fold changes were either based on the Euclidean (right panel) or uncentered pairwise Pearson correlation distance (right panel).

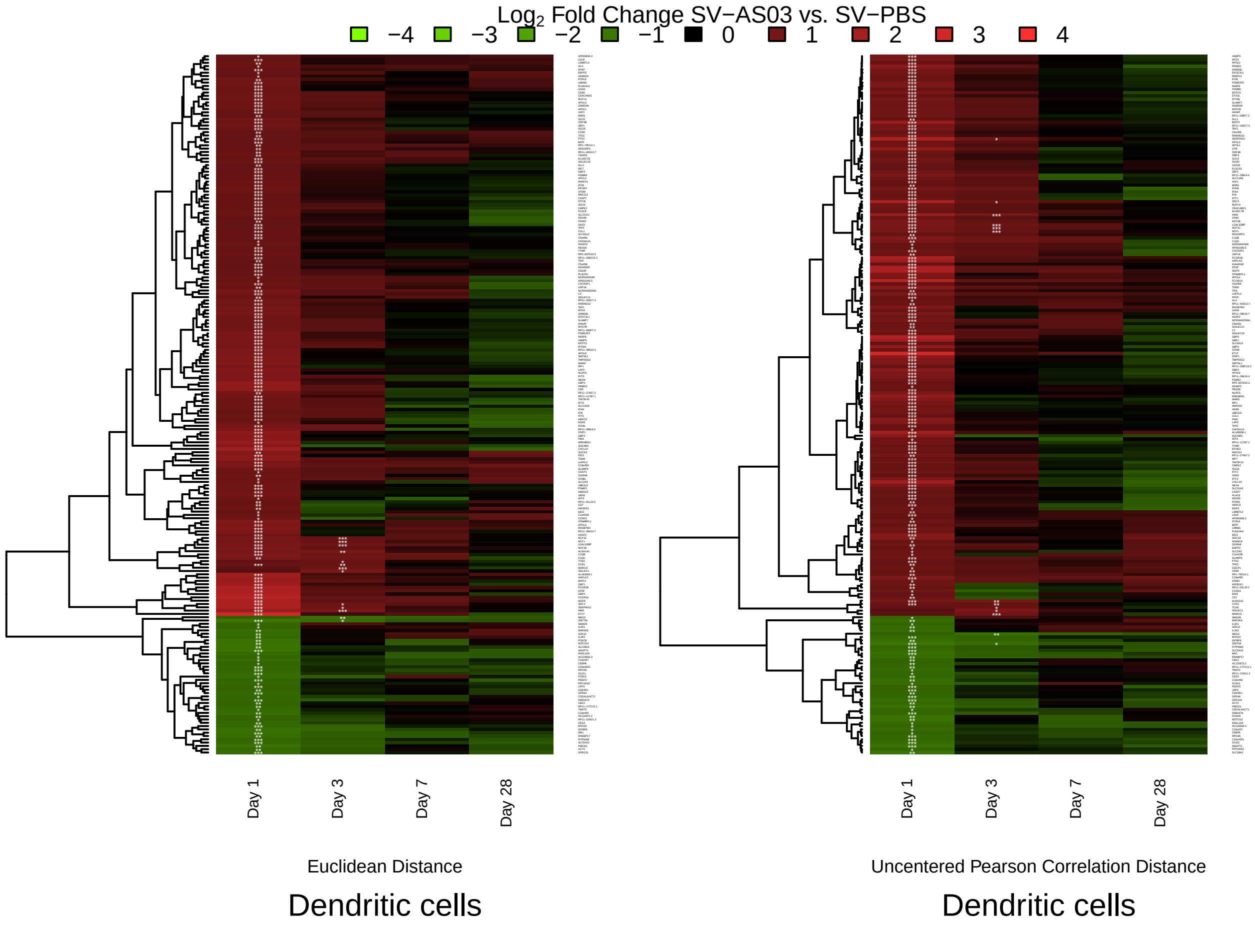

Figure A80: Heatmaps of GC adjusted log fold change (SV-AS03 vs. SV-PBS) over time (RNA-Seq, Dendritic cells). Colored in red: up-regulated for SV-AS03; colored in green: down-regulated for SV-AS03. Asterisks mark genes that are significantly expressed at a certain day; FDR level (*:0.05, **:0.01, ***:0.001). Dendrograms were obtained using complete linkage clustering. Distances between fold changes were either based on the Euclidean (right panel) or uncentered pairwise Pearson correlation distance (right panel).

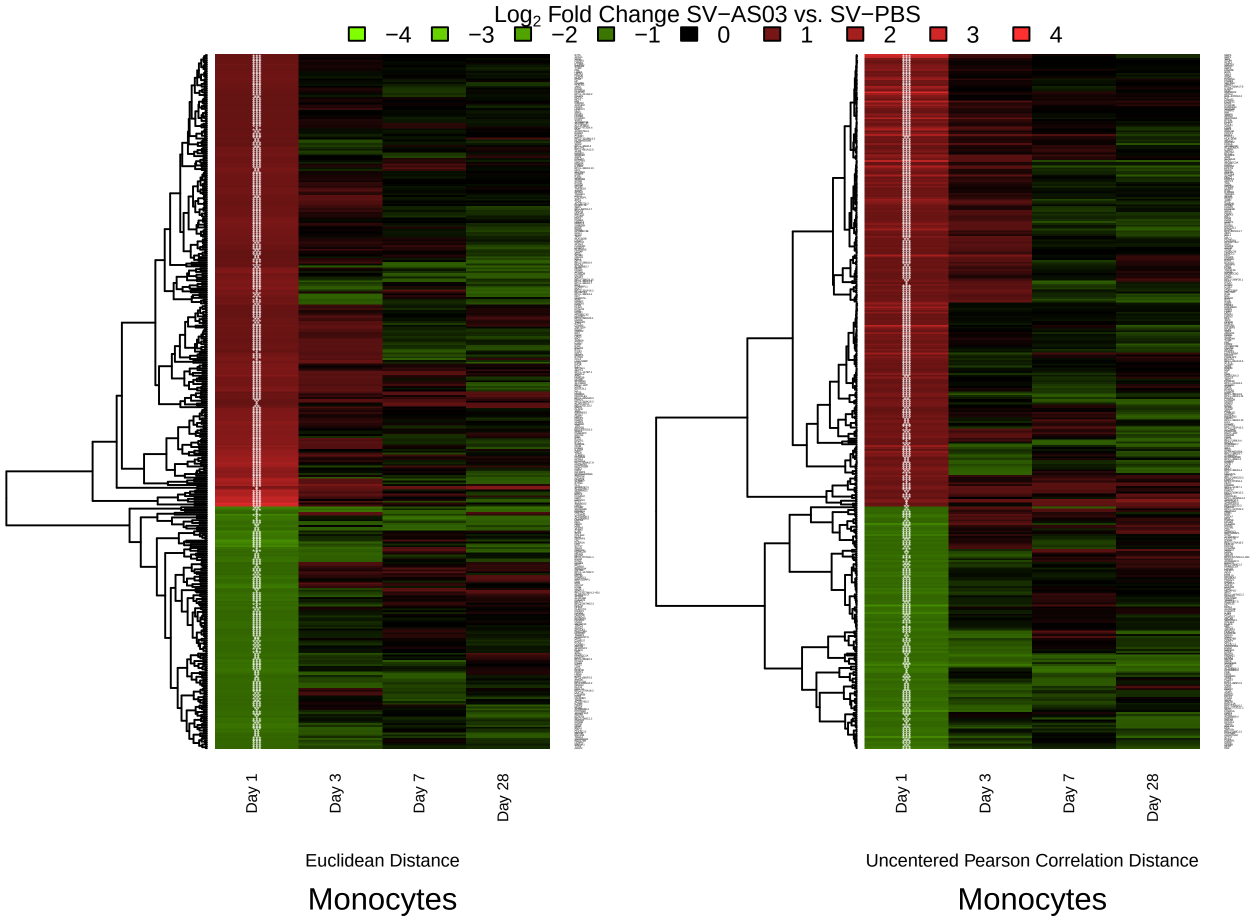

Figure A81: Heatmaps of GC adjusted log fold change (SV-AS03 vs. SV-PBS) over time (RNA-Seq, Monocytes). Colored in red: up-regulated for SV-AS03; colored in green: down-regulated for SV-AS03. Asterisks mark genes that are significantly expressed at a certain day; FDR level (*:0.05, **:0.01, ***:0.001). Dendrograms were obtained using complete linkage clustering. Distances between fold changes were either based on the Euclidean (right panel) or uncentered pairwise Pearson correlation distance (right panel).

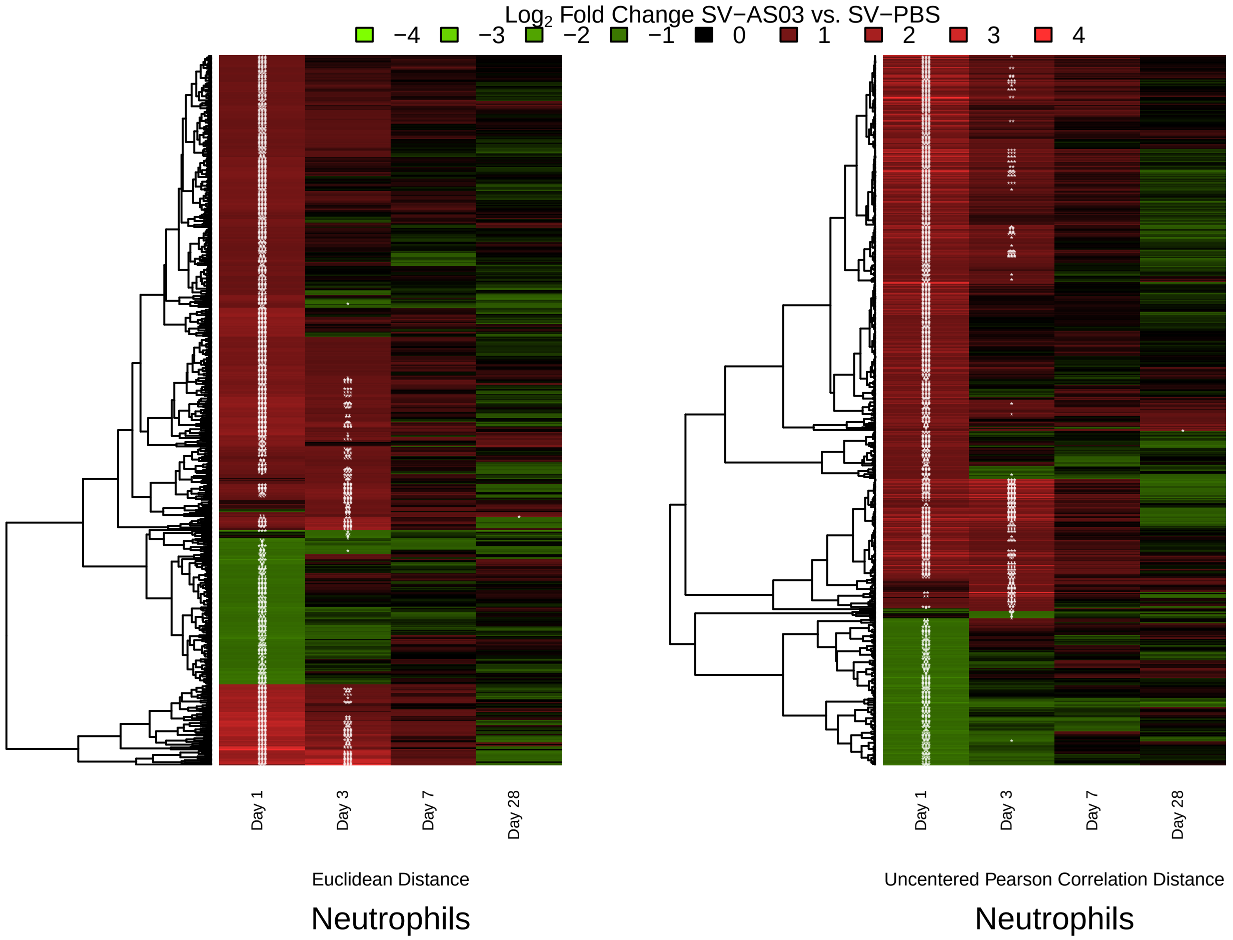

Figure A82: Heatmaps of GC adjusted log fold change (SV-AS03 vs. SV-PBS) over time (RNA-Seq, Neutrophils). Colored in red: up-regulated for SV-AS03; colored in green: down-regulated for SV-AS03. Asterisks mark genes that are significantly expressed at a certain day; FDR level (*:0.05, **:0.01, ***:0.001). Dendrograms were obtained using complete linkage clustering. Distances between fold changes were either based on the Euclidean (right panel) or uncentered pairwise Pearson correlation distance (right panel).

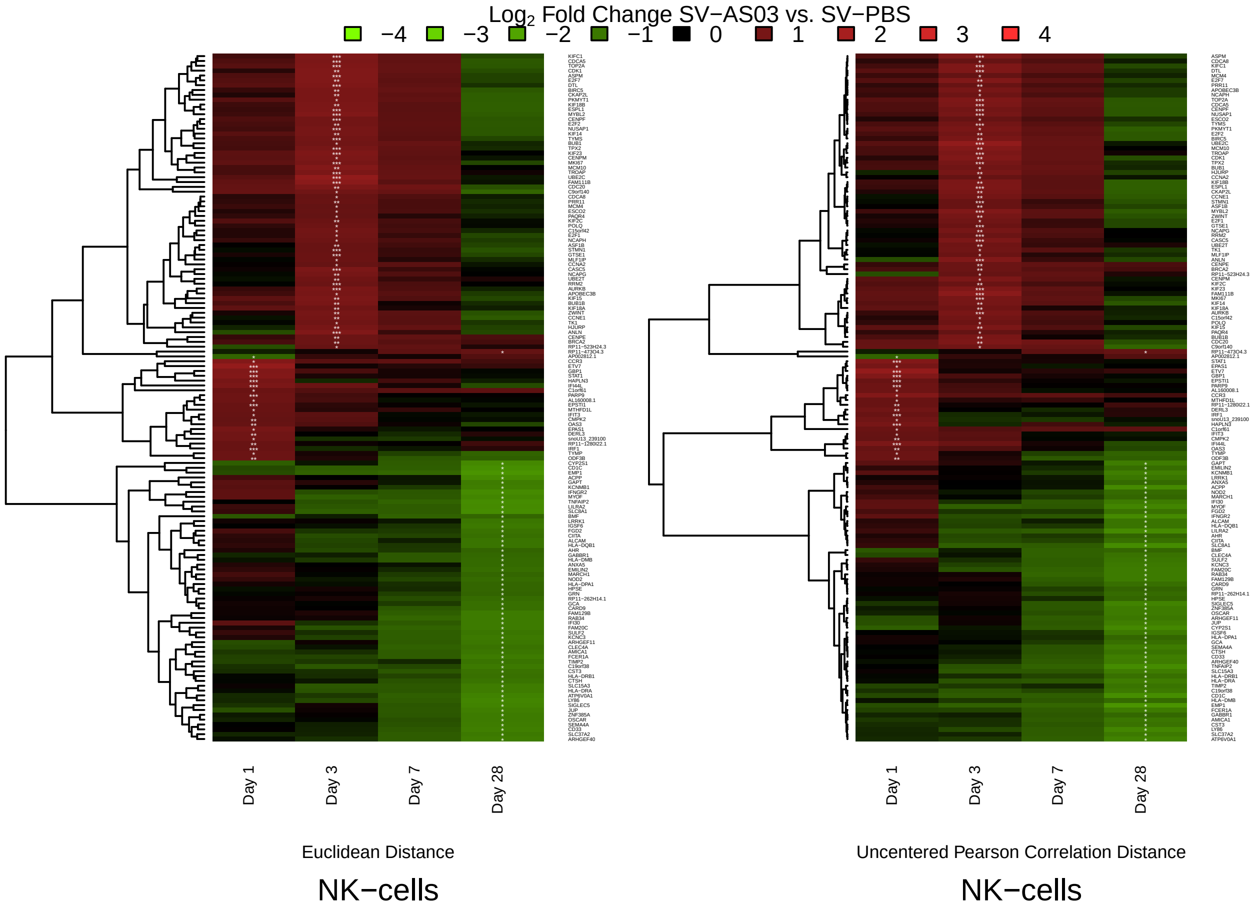

Figure A83: Heatmaps of GC adjusted log fold change (SV-AS03 vs. SV-PBS) over time (RNA-Seq, NK-cells). Colored in red: up-regulated for SV-AS03; colored in green: down-regulated for SV-AS03. Asterisks mark genes that are significantly expressed at a certain day; FDR level (*:0.05, **:0.01, ***:0.001). Dendrograms were obtained using complete linkage clustering. Distances between fold changes were either based on the Euclidean (right panel) or uncentered pairwise Pearson correlation distance (right panel).

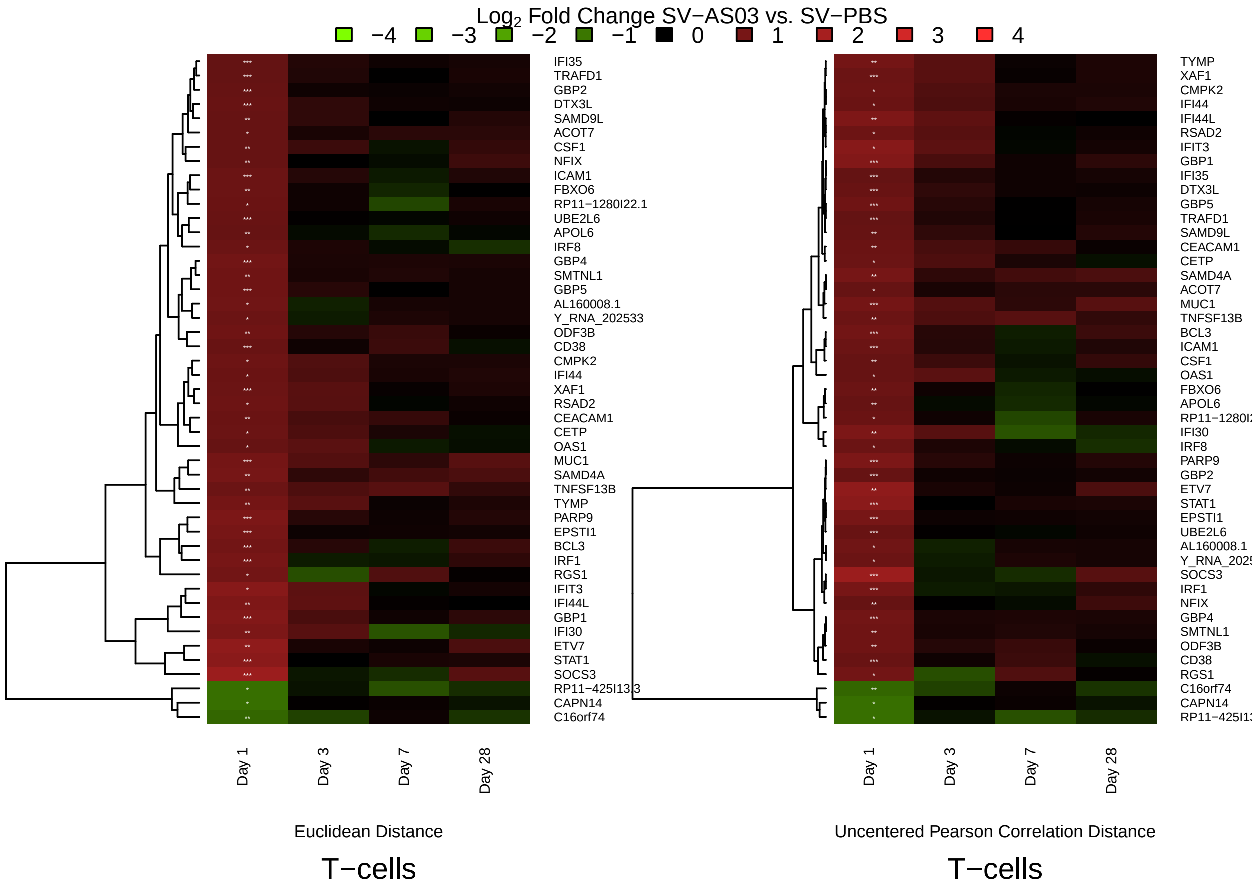

Figure A84: Heatmaps of GC adjusted log fold change (SV-AS03 vs. SV-PBS) over time (RNA-Seq, T-cells). Colored in red: up-regulated for SV-AS03; colored in green: down-regulated for SV-AS03. Asterisks mark genes that are significantly expressed at a certain day; FDR level (*:0.05, **:0.01, ***:0.001). Dendrograms were obtained using complete linkage clustering. Distances between fold changes were either based on the Euclidean (right panel) or uncentered pairwise Pearson correlation distance (right panel).

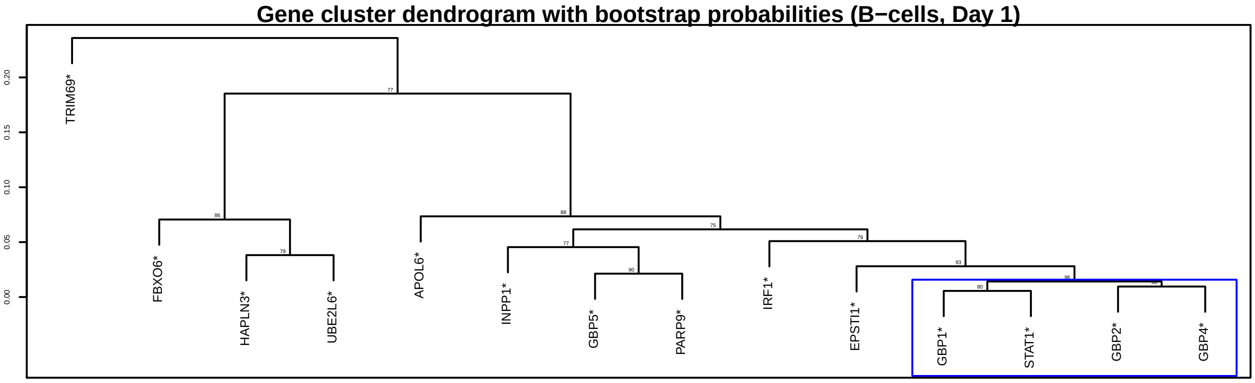

Figure A85: Gene cluster dendrogram with bootstrap probabilities (RNA-Seq, B-cells, Day 1). The y axis shows the distance at which clusters were formed by the complete linkage clustering algorithm based on uncentered Pearson correlation distance between log fold changes. Multiscale bootstrap probabilities are shown at each branch intersection. Significant clusters are highlighted in blue. Asterisks indicate genes that were significantly expressed at a certain day.

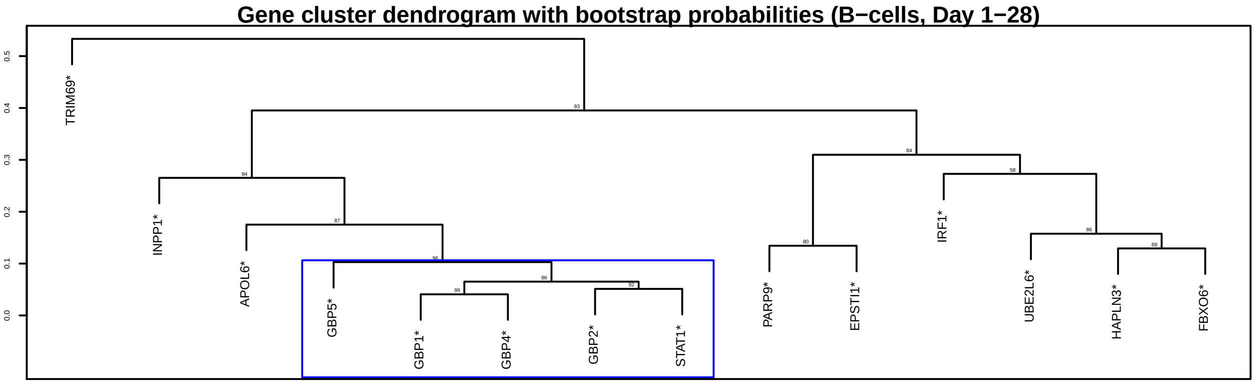

Figure A86: Gene cluster dendrogram with bootstrap probabilities (RNA-Seq, B-cells, Day 1-28). The y axis shows the distance at which clusters were formed by the complete linkage clustering algorithm based on uncentered Pearson correlation distance between log fold changes. Multiscale bootstrap probabilities are shown at each branch intersection. Significant clusters are highlighted in blue. Asterisks indicate genes that were significantly expressed at a certain day.

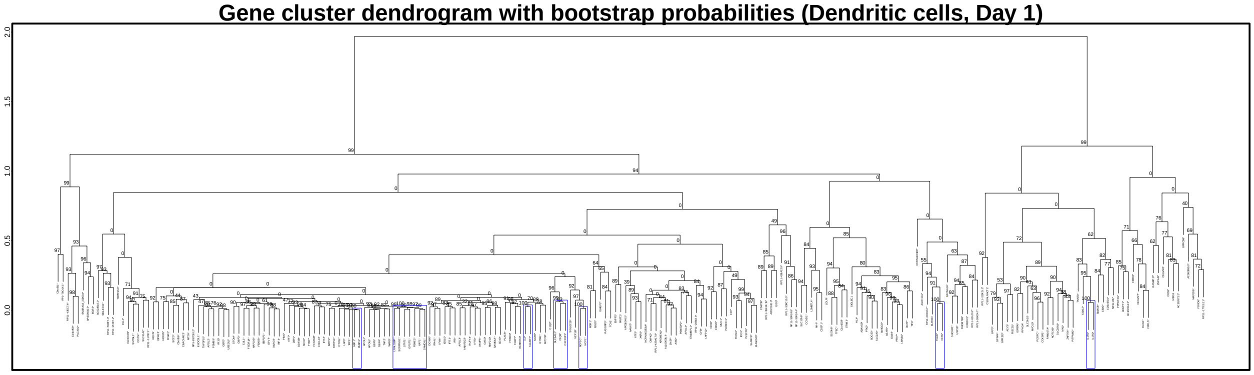

Figure A87: Gene cluster dendrogram with bootstrap probabilities (RNA-Seq, Dendritic cells, Day 1). The y axis shows the distance at which clusters were formed by the complete linkage clustering algorithm based on uncentered Pearson correlation distance between log fold changes. Multiscale bootstrap probabilities are shown at each branch intersection. Significant clusters are highlighted in blue. Asterisks indicate genes that were significantly expressed at a certain day.

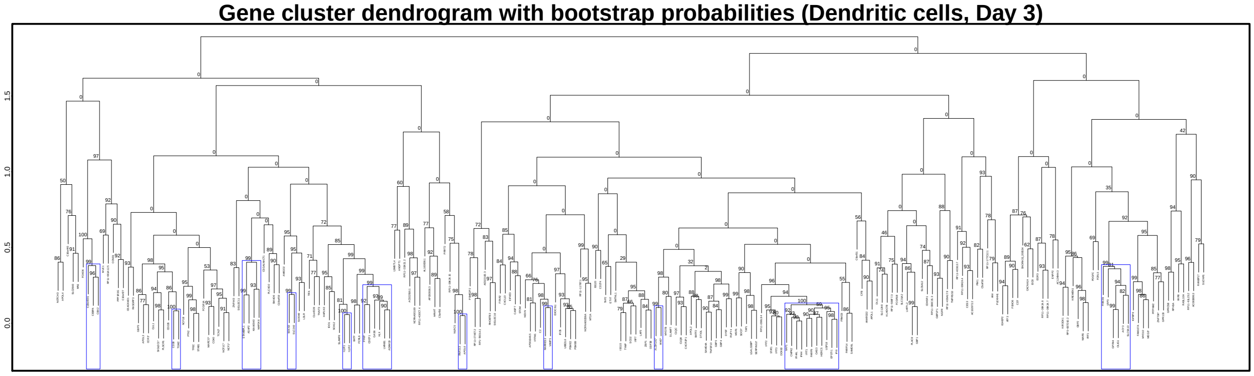

Figure A88: Gene cluster dendrogram with bootstrap probabilities (RNA-Seq, Dendritic cells, Day 3). The y axis shows the distance at which clusters were formed by the complete linkage clustering algorithm based on uncentered Pearson correlation distance between log fold changes. Multiscale bootstrap probabilities are shown at each branch intersection. Significant clusters are highlighted in blue. Asterisks indicate genes that were significantly expressed at a certain day.

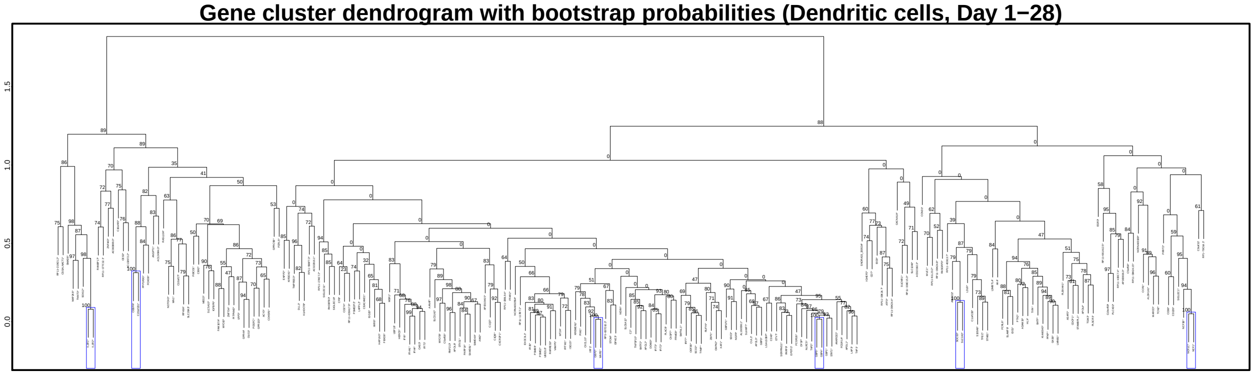

Figure A89: Gene cluster dendrogram with bootstrap probabilities (RNA-Seq, Dendritic cells, Day 1-28). The y axis shows the distance at which clusters were formed by the complete linkage clustering algorithm based on uncentered Pearson correlation distance between log fold changes. Multiscale bootstrap probabilities are shown at each branch intersection. Significant clusters are highlighted in blue. Asterisks indicate genes that were significantly expressed at a certain day.

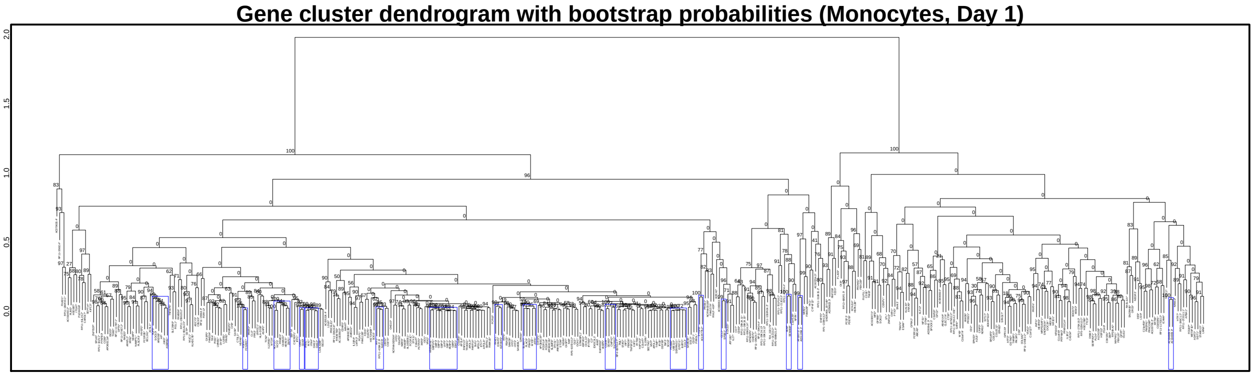

Figure A90: Gene cluster dendrogram with bootstrap probabilities (RNA-Seq, Monocytes, Day 1). The y axis shows the distance at which clusters were formed by the complete linkage clustering algorithm based on uncentered Pearson correlation distance between log fold changes. Multiscale bootstrap probabilities are shown at each branch intersection. Significant clusters are highlighted in blue. Asterisks indicate genes that were significantly expressed at a certain day.

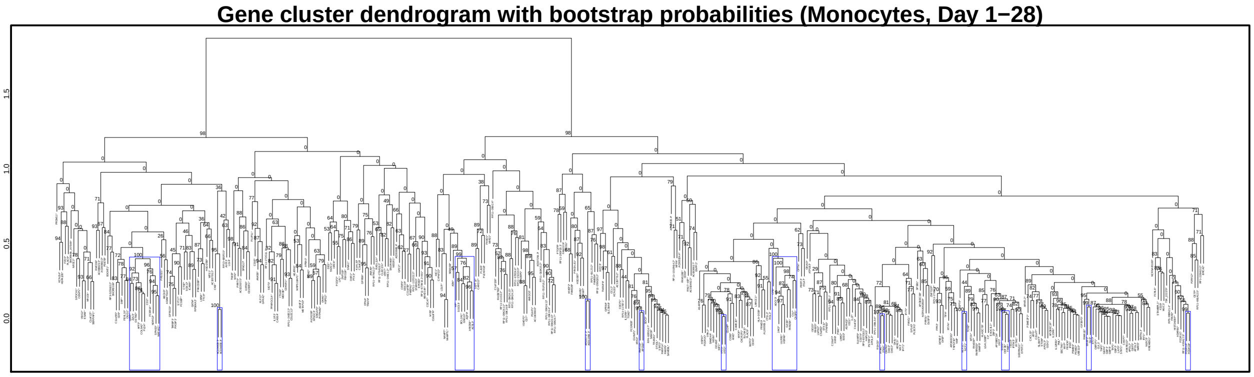

Figure A91: Gene cluster dendrogram with bootstrap probabilities (RNA-Seq, Monocytes, Day 1-28). The y axis shows the distance at which clusters were formed by the complete linkage clustering algorithm based on uncentered Pearson correlation distance between log fold changes. Multiscale bootstrap probabilities are shown at each branch intersection. Significant clusters are highlighted in blue. Asterisks indicate genes that were significantly expressed at a certain day.

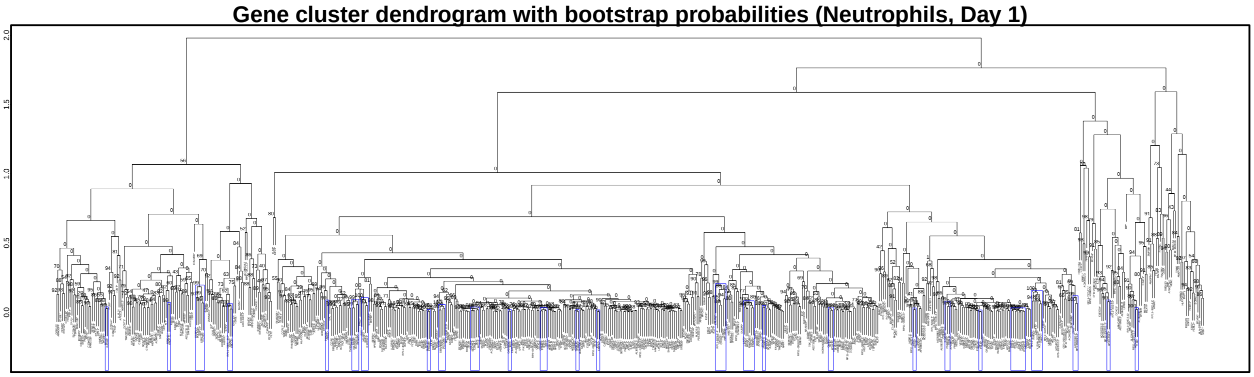

Figure A92: Gene cluster dendrogram with bootstrap probabilities (RNA-Seq, Neutrophils, Day 1). The y axis shows the distance at which clusters were formed by the complete linkage clustering algorithm based on uncentered Pearson correlation distance between log fold changes. Multiscale bootstrap probabilities are shown at each branch intersection. Significant clusters are highlighted in blue. Asterisks indicate genes that were significantly expressed at a certain day.

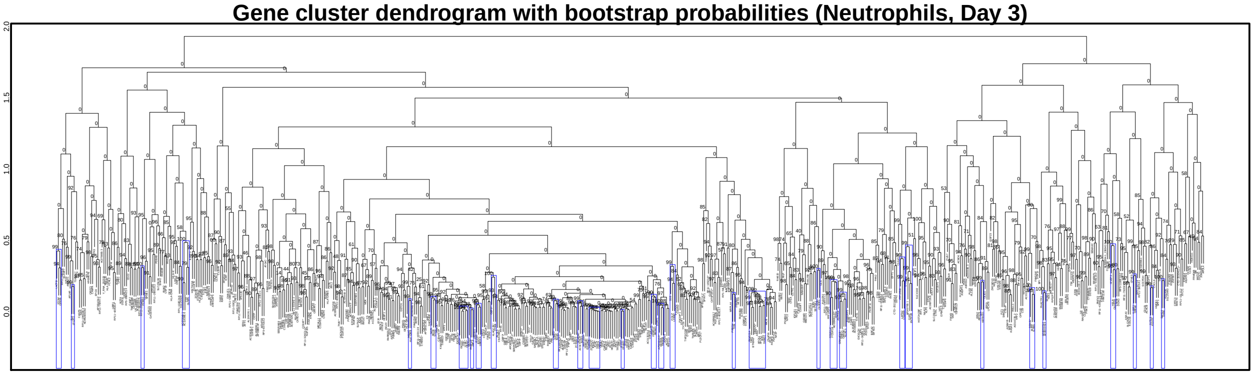

Figure A93: Gene cluster dendrogram with bootstrap probabilities (RNA-Seq, Neutrophils, Day 3). The y axis shows the distance at which clusters were formed by the complete linkage clustering algorithm based on uncentered Pearson correlation distance between log fold changes. Multiscale bootstrap probabilities are shown at each branch intersection. Significant clusters are highlighted in blue. Asterisks indicate genes that were significantly expressed at a certain day.

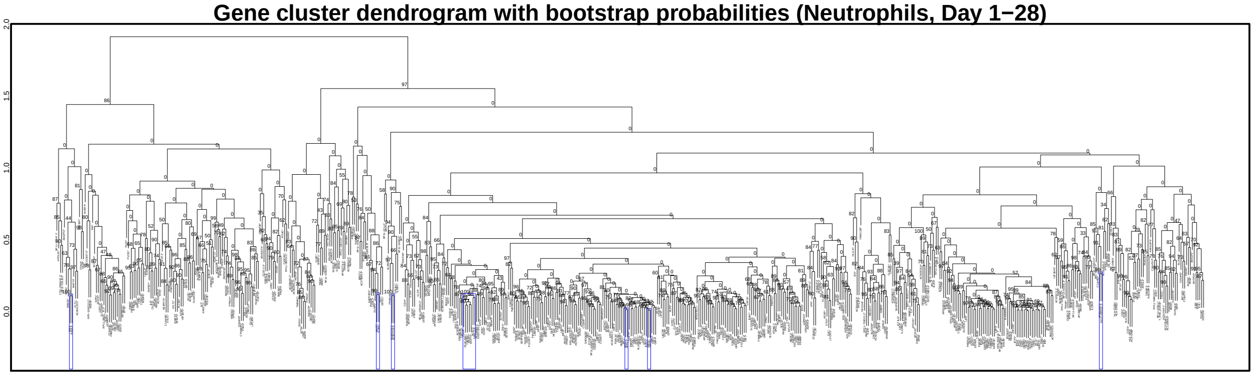

Figure A94: Gene cluster dendrogram with bootstrap probabilities (RNA-Seq, Neutrophils, Day 1-28). The y axis shows the distance at which clusters were formed by the complete linkage clustering algorithm based on uncentered Pearson correlation distance between log fold changes. Multiscale bootstrap probabilities are shown at each branch intersection. Significant clusters are highlighted in blue. Asterisks indicate genes that were significantly expressed at a certain day.

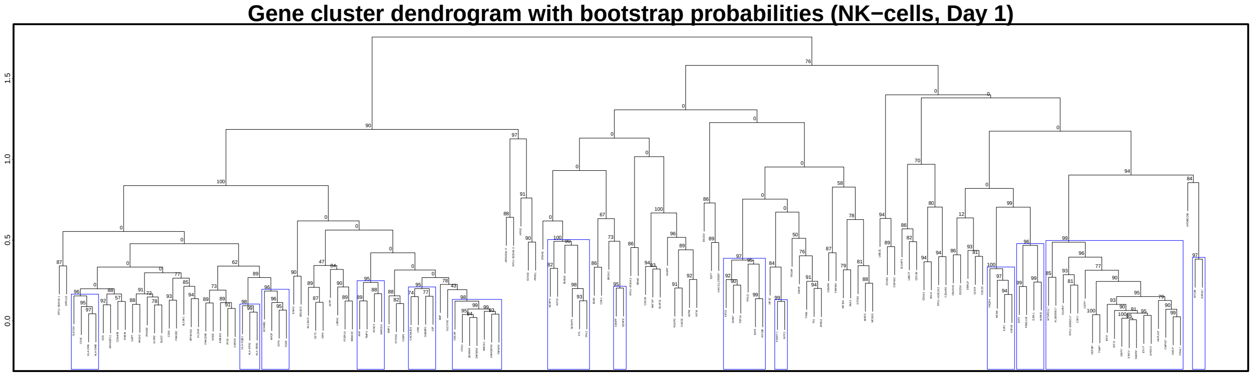

Figure A95: Gene cluster dendrogram with bootstrap probabilities (RNA-Seq, NK-cells, Day 1). The y axis shows the distance at which clusters were formed by the complete linkage clustering algorithm based on uncentered Pearson correlation distance between log fold changes. Multiscale bootstrap probabilities are shown at each branch intersection. Significant clusters are highlighted in blue. Asterisks indicate genes that were significantly expressed at a certain day.

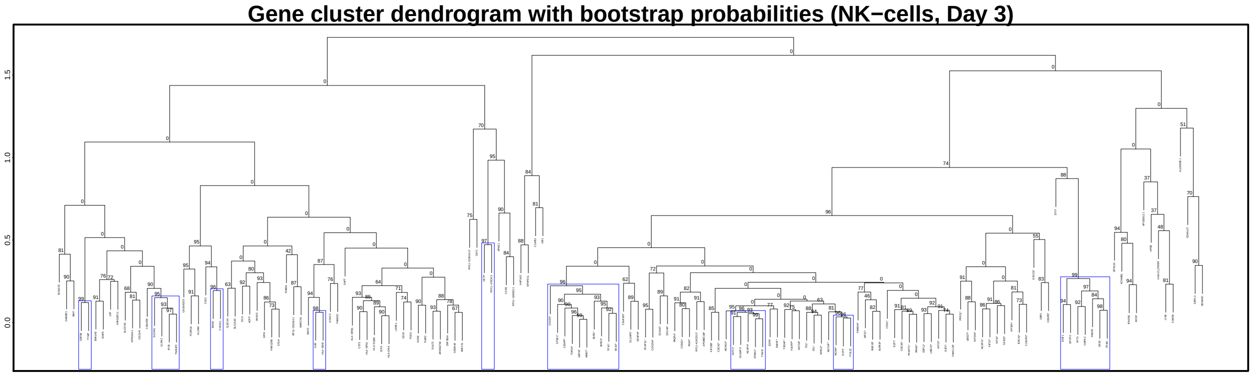

Figure A96: Gene cluster dendrogram with bootstrap probabilities (RNA-Seq, NK-cells, Day 3). The y axis shows the distance at which clusters were formed by the complete linkage clustering algorithm based on uncentered Pearson correlation distance between log fold changes. Multiscale bootstrap probabilities are shown at each branch intersection. Significant clusters are highlighted in blue. Asterisks indicate genes that were significantly expressed at a certain day.

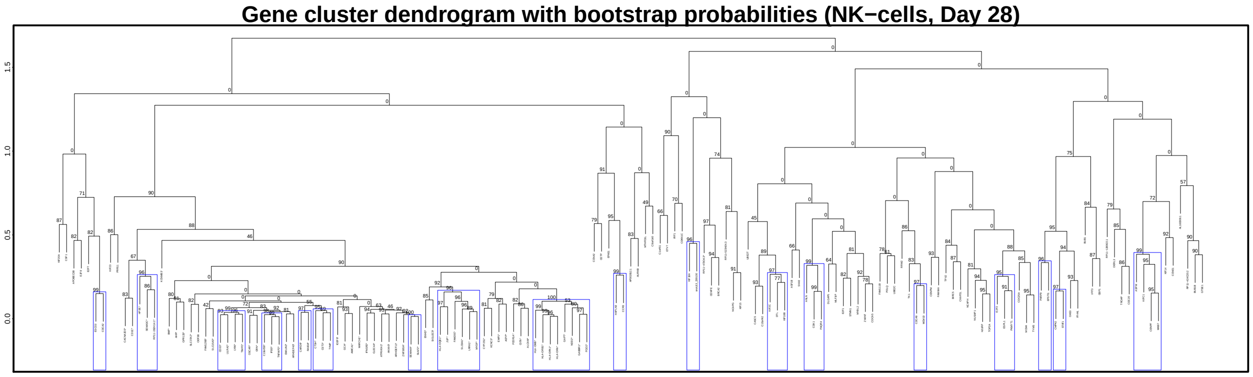

Figure A97: Gene cluster dendrogram with bootstrap probabilities (RNA-Seq, NK-cells, Day 28). The y axis shows the distance at which clusters were formed by the complete linkage clustering algorithm based on uncentered Pearson correlation distance between log fold changes. Multiscale bootstrap probabilities are shown at each branch intersection. Significant clusters are highlighted in blue. Asterisks indicate genes that were significantly expressed at a certain day.

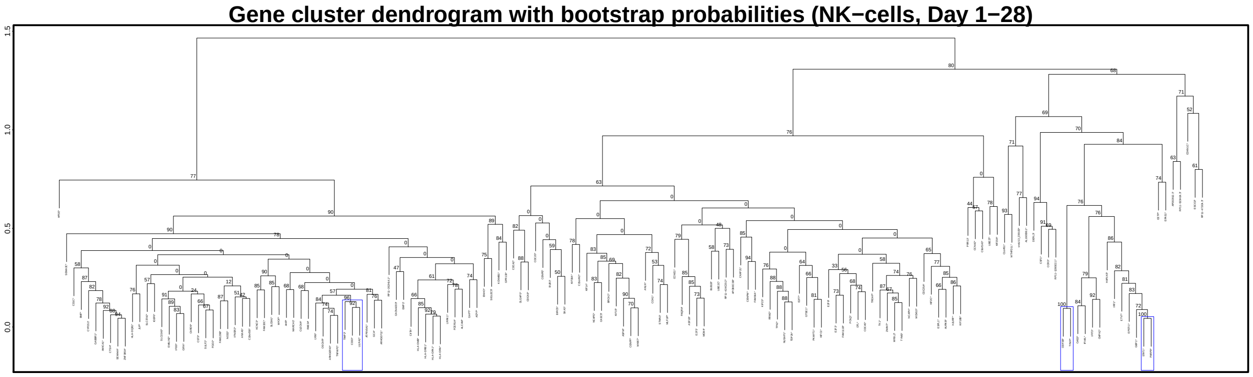

Figure A98: Gene cluster dendrogram with bootstrap probabilities (RNA-Seq, NK-cells, Day 1-28). The y axis shows the distance at which clusters were formed by the complete linkage clustering algorithm based on uncentered Pearson correlation distance between log fold changes. Multiscale bootstrap probabilities are shown at each branch intersection. Significant clusters are highlighted in blue. Asterisks indicate genes that were significantly expressed at a certain day.

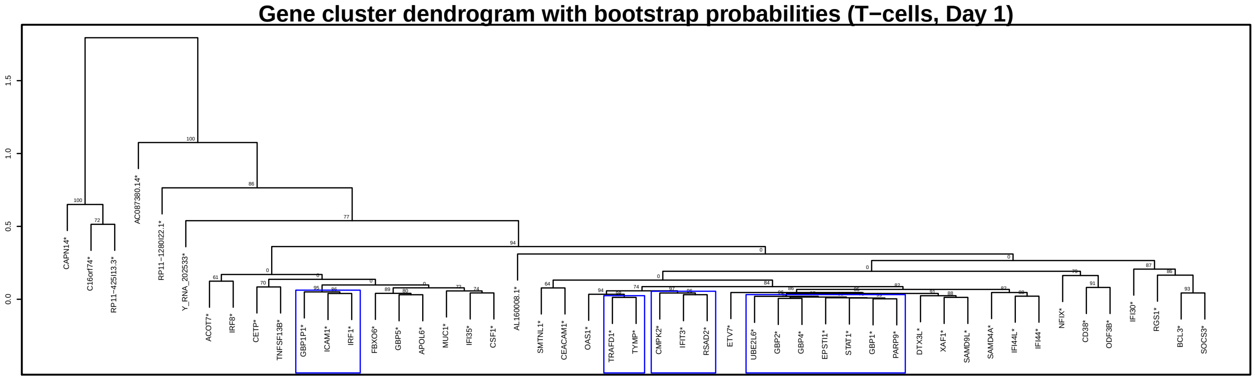

Figure A99: Gene cluster dendrogram with bootstrap probabilities (RNA-Seq, T-cells, Day 1). The y axis shows the distance at which clusters were formed by the complete linkage clustering algorithm based on uncentered Pearson correlation distance between log fold changes. Multiscale bootstrap probabilities are shown at each branch intersection. Significant clusters are highlighted in blue. Asterisks indicate genes that were significantly expressed at a certain day.

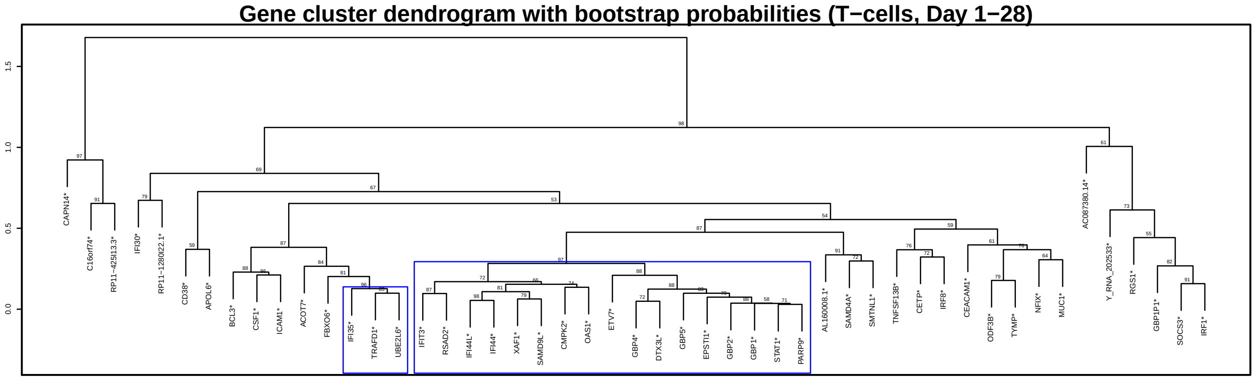

Figure A100: Gene cluster dendrogram with bootstrap probabilities (RNA-Seq, T-cells, Day 1-28). The y axis shows the distance at which clusters were formed by the complete linkage clustering algorithm based on uncentered Pearson correlation distance between log fold changes. Multiscale bootstrap probabilities are shown at each branch intersection. Significant clusters are highlighted in blue. Asterisks indicate genes that were significantly expressed at a certain day.

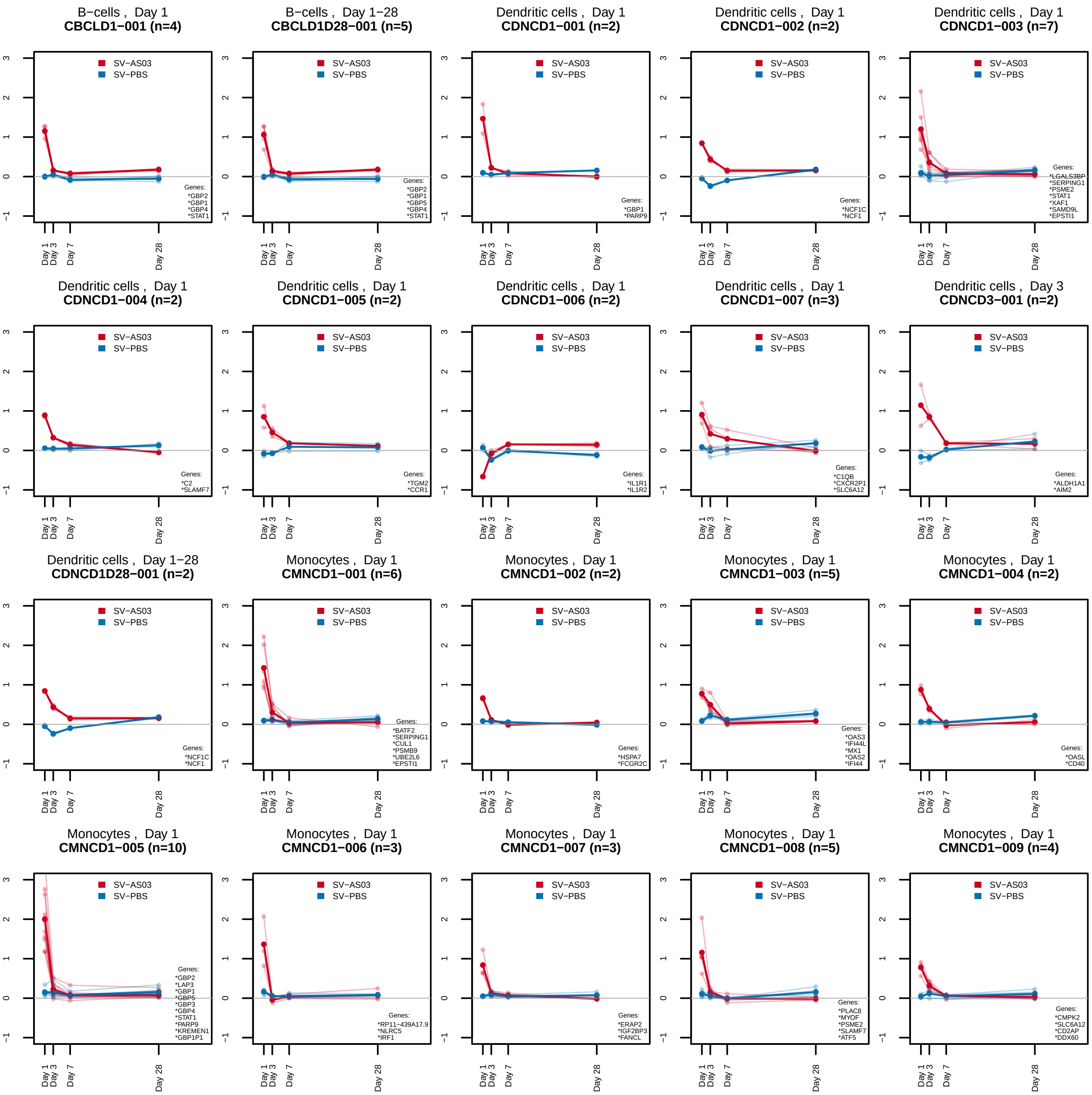

Figure A101: Gene cluster time trends of baseline log fold change by vaccine group (RNA-Seq, B-cells, Dendritic cells, and Monocytes). Header indicates cluster ID. Mean log fold change across cluster genes is drawn in bold. Individual mean gene log fold changes are plotted in lighter colors. Asterisks indicate significantly differentially expressed genes for a given day.

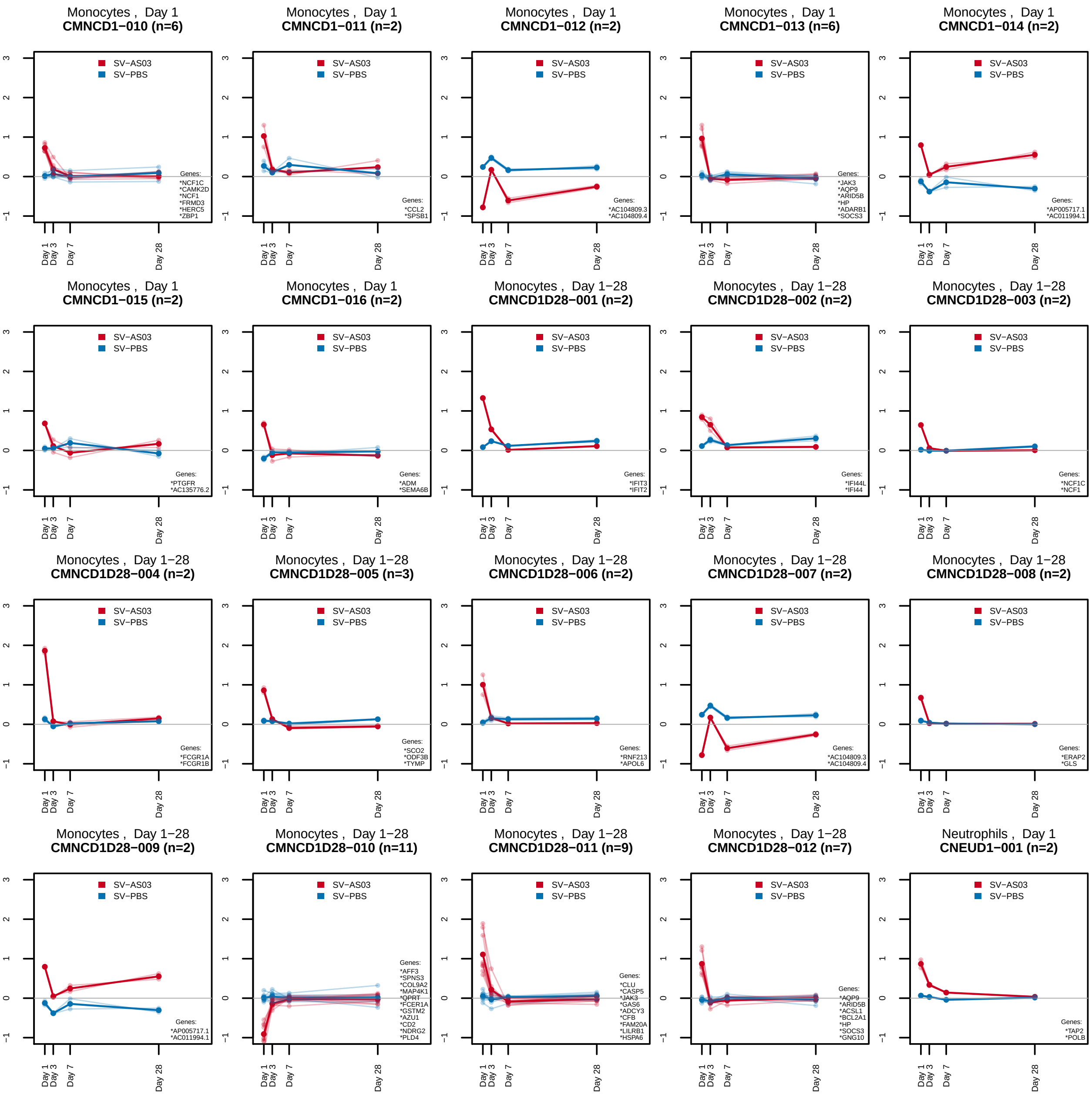

Figure A102: Gene cluster time trends of baseline log fold change by vaccine group (RNA-Seq, Monocytes and Neutrophils). Header indicates cluster ID. Mean log fold change across cluster genes is drawn in bold. Individual gene mean log fold changes are plotted in lighter colors. Asterisks indicate significantly differentially expressed genes for a given day.

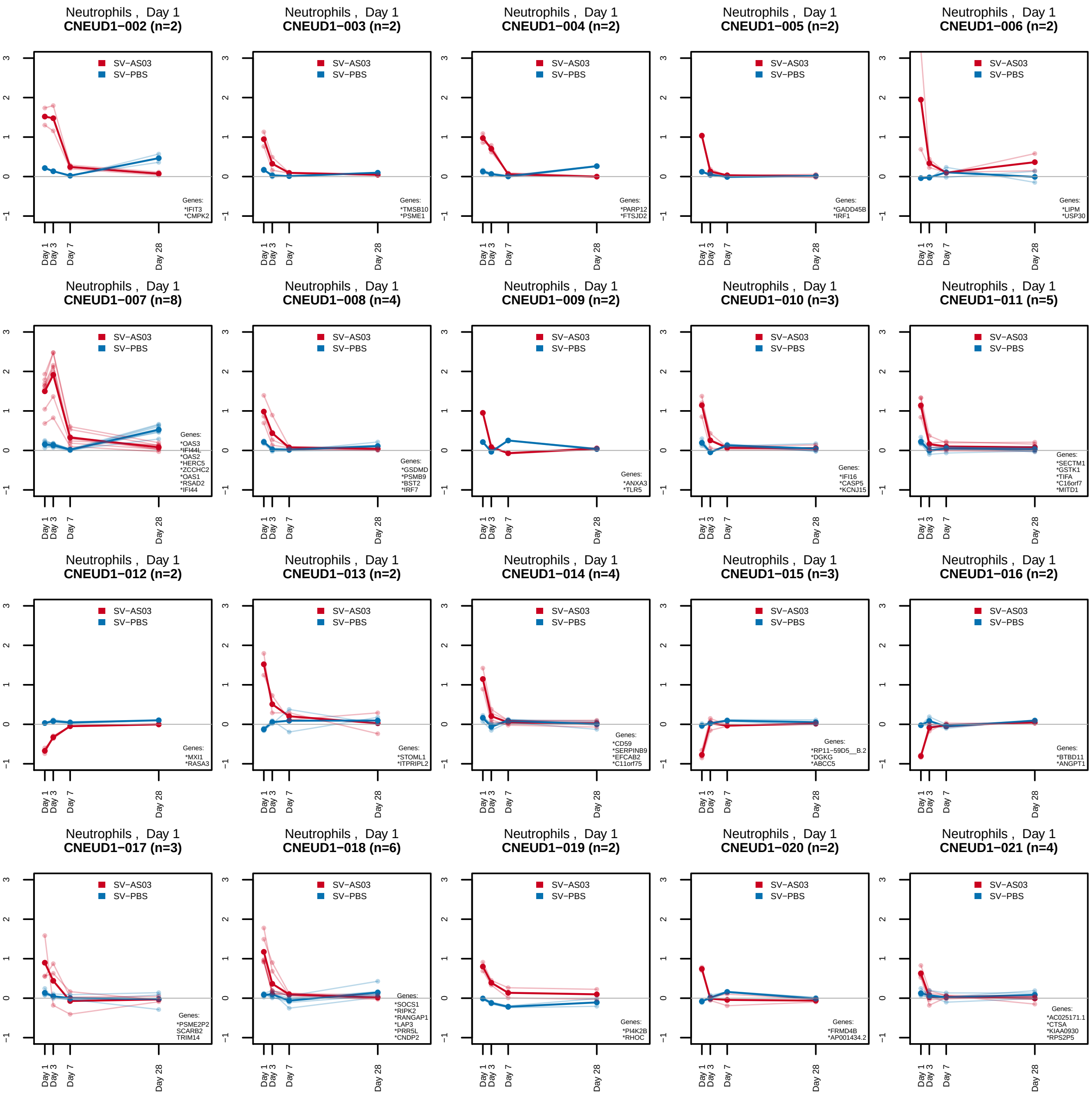

Figure A103: Gene cluster time trends of baseline log fold change by vaccine group (RNA-Seq, Neutrophils continued). Header indicates cluster ID. Mean log fold change across cluster genes is drawn in bold. Individual mean gene log fold changes are plotted in lighter colors. Asterisks indicate significantly differentially expressed genes for a given day.

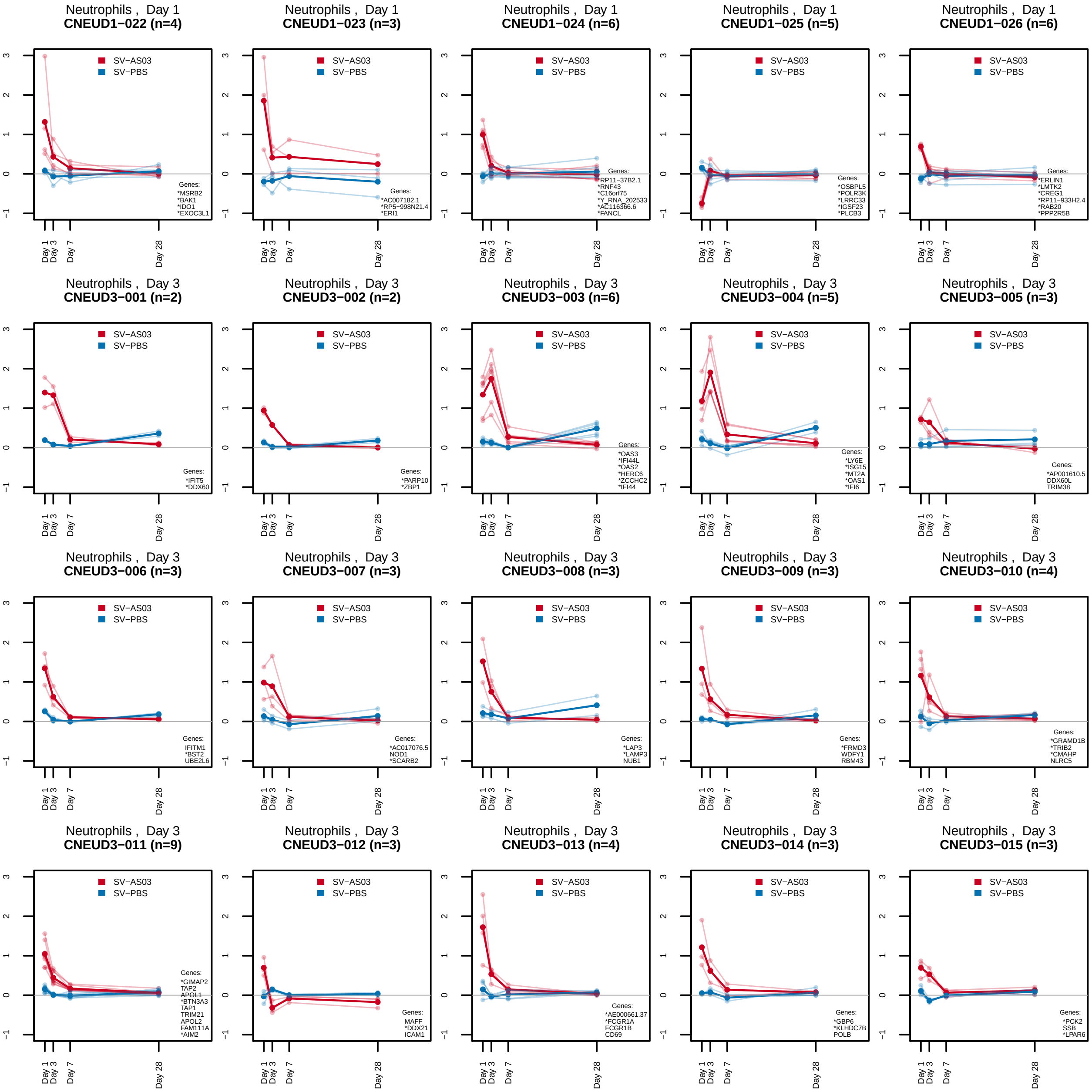

Figure A104: Gene cluster time trends of baseline log fold change by vaccine group (RNA-Seq, Neutrophils continued). Header indicates cluster ID. Mean log fold change across cluster genes is drawn in bold. Individual mean gene log fold changes are plotted in lighter colors. Asterisks indicate significantly differentially expressed genes for a given day.

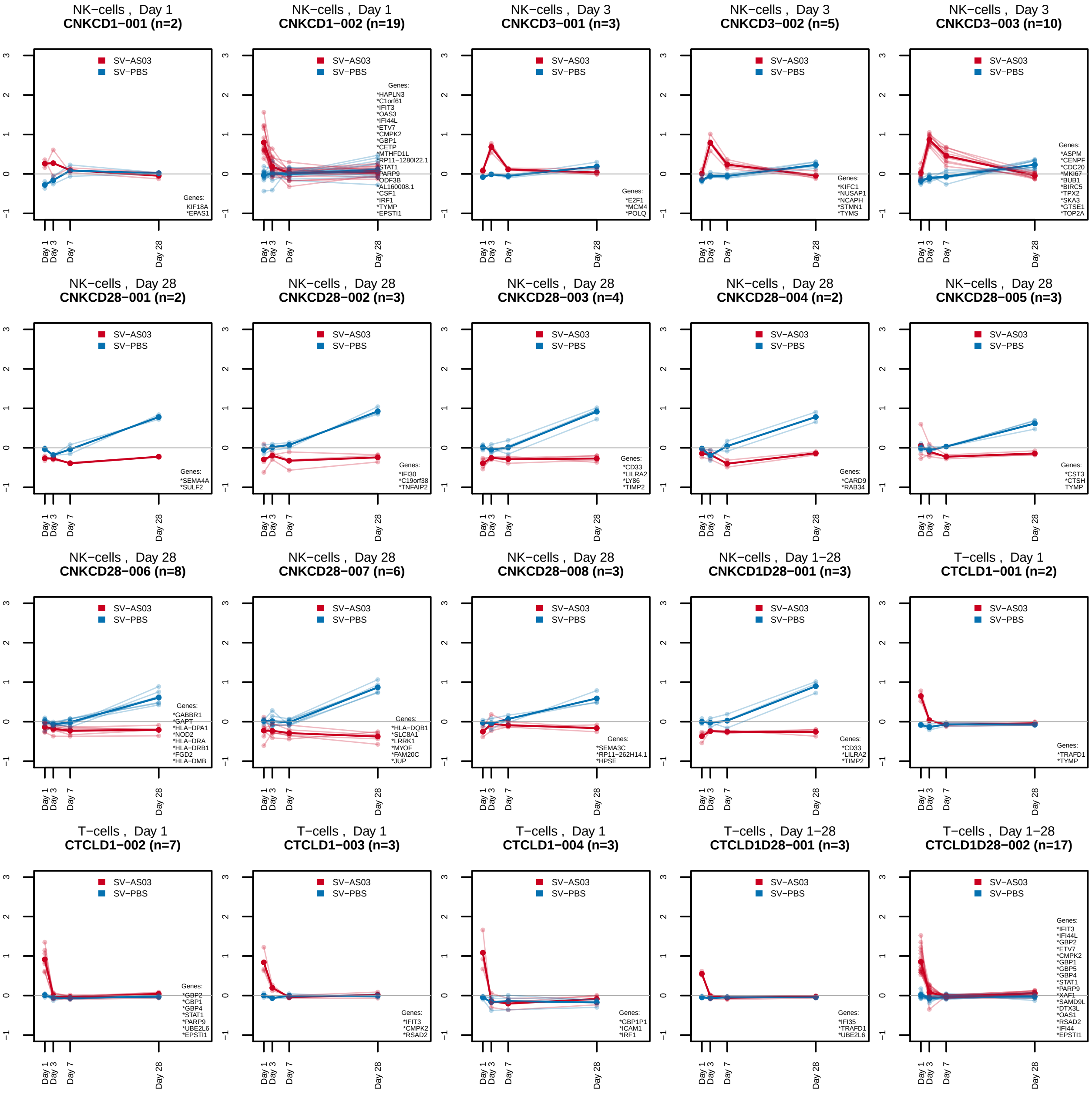

Figure A105: Gene cluster time trends of baseline log fold change by vaccine group (RNA-Seq, NK-cells and T-cells). Header indicates cluster ID. Mean log fold change across cluster genes is drawn in bold. Individual mean gene log fold changes are plotted in lighter colors. Asterisks indicate significantly differentially expressed genes for a given day.

SV−AS03, Dendritic cells, Kegg Antigen Processing & Presentation

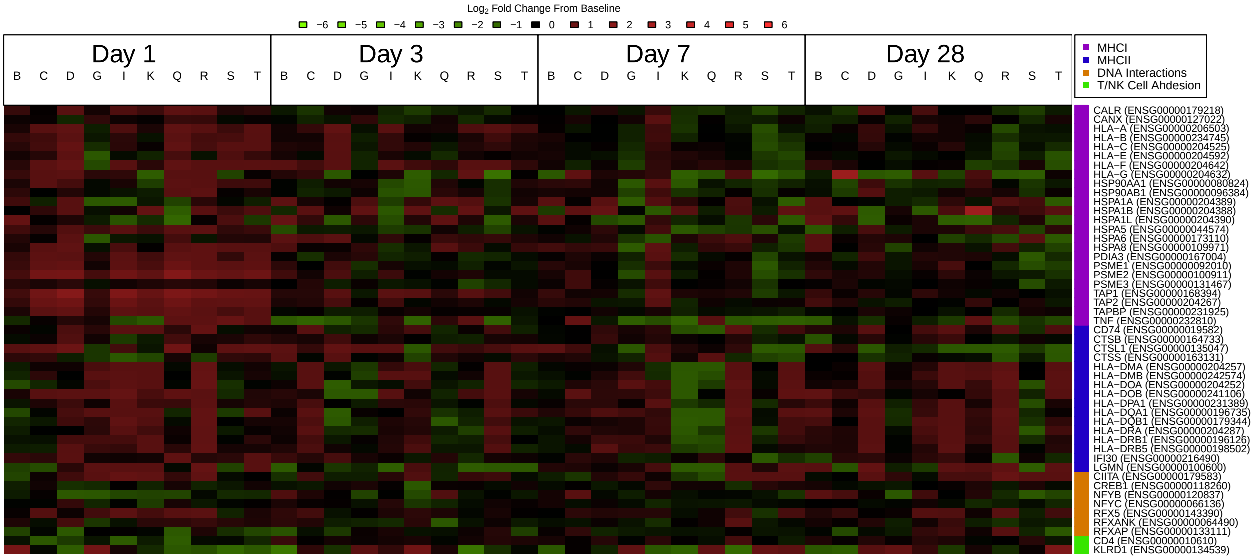

SV−PBS, Dendritic cells, Kegg Antigen Processing & Presentation

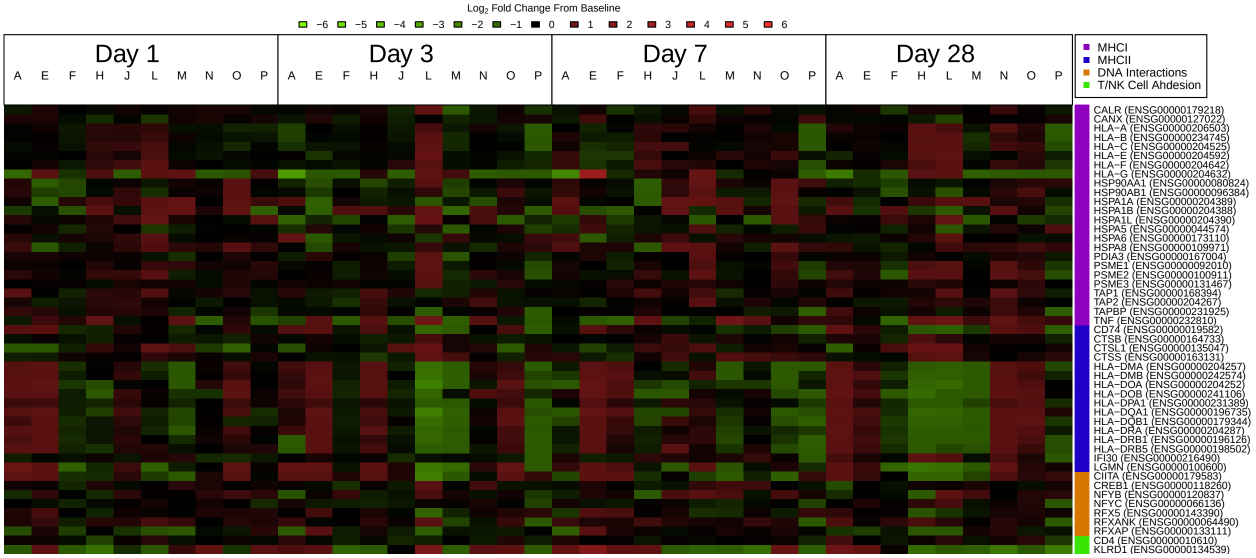

Figure A106: Heatmap of baseline log fold changes for known antigen presentation genes by vaccine group (RNA-Seq, Dendritic cells). Colored in red: up regulated from baseline; colored in green: down regulated from baseline. Genes were selected based on the KEGG antigen processing and presentation pathway.

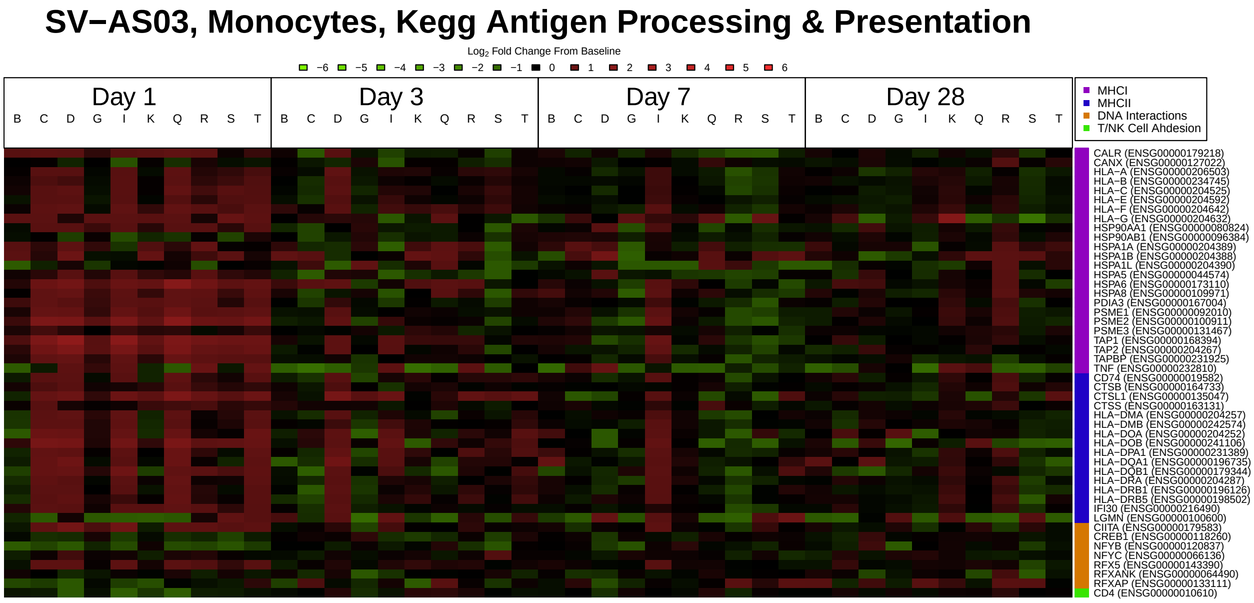

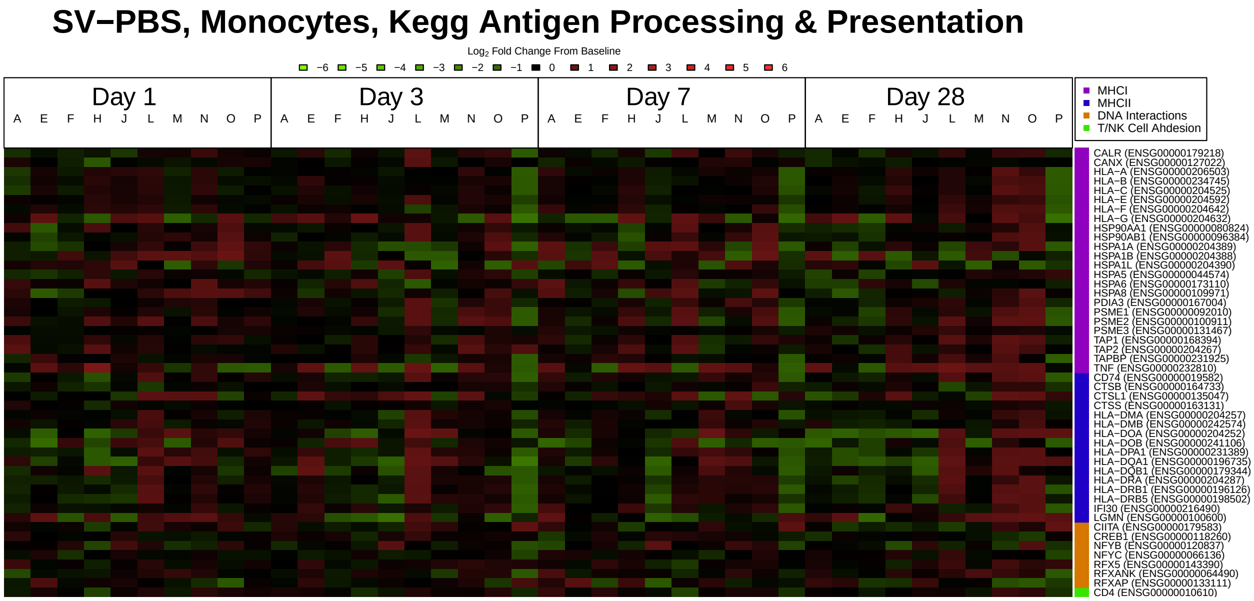

Figure A107: Heatmap of baseline log fold changes for known antigen presentation genes by vaccine group (RNA-Seq, Monocytes). Colored in red: up regulated from baseline; colored in green: down regulated from baseline. Genes were selected based on the KEGG antigen processing and presentation pathway.

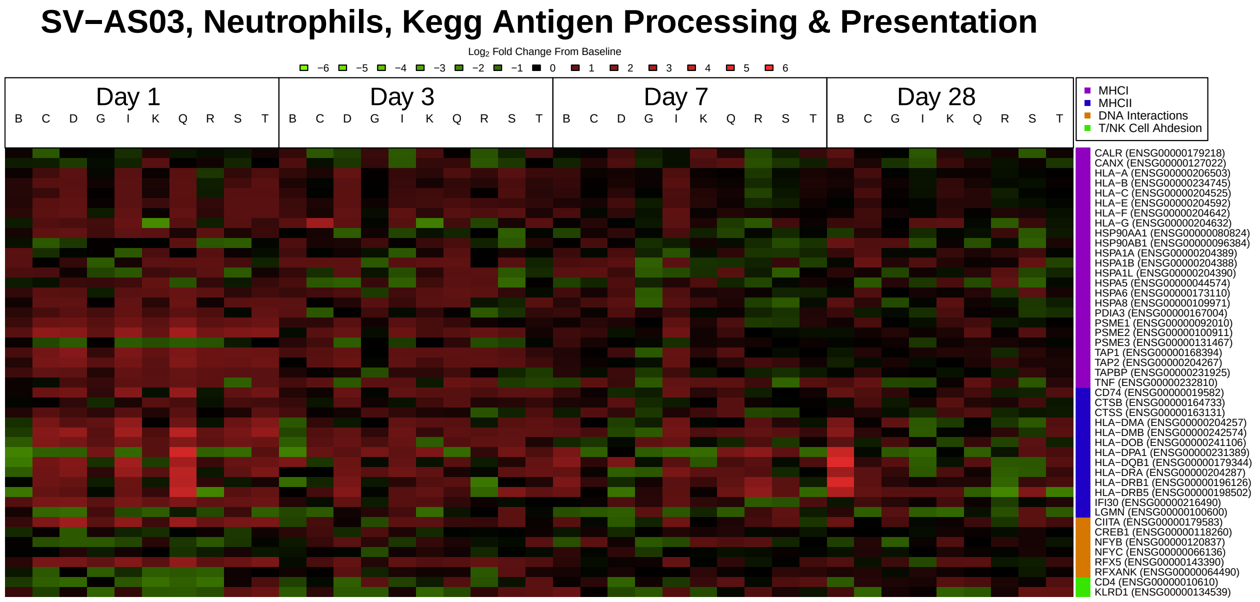

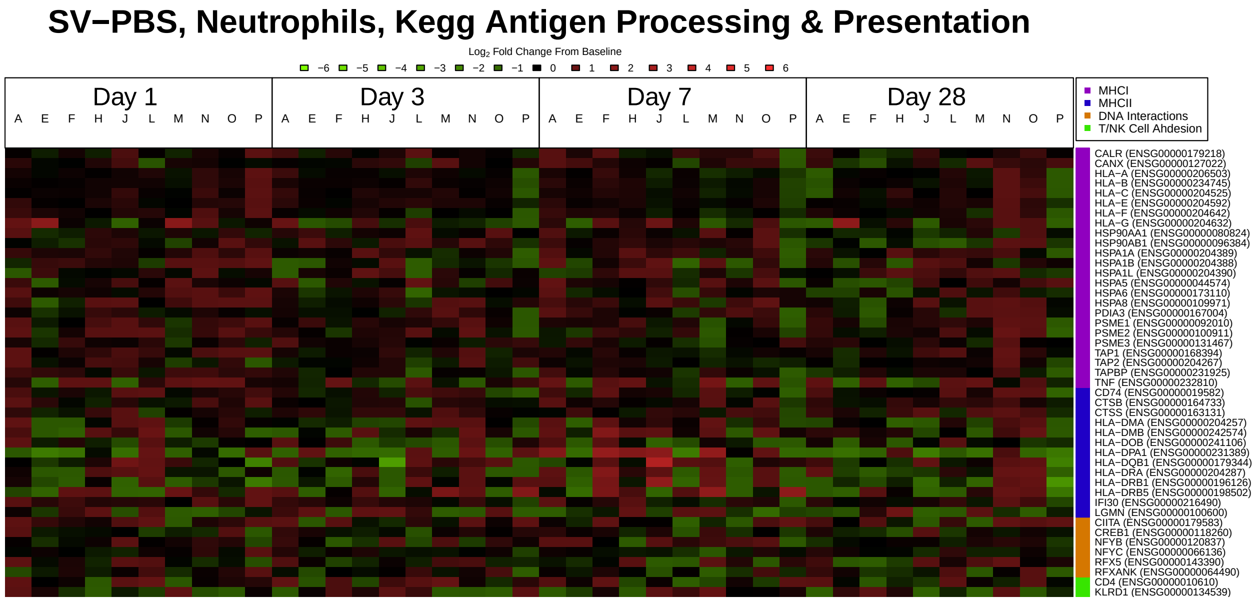

Figure A108: Heatmap of baseline log fold changes for known antigen presentation genes by vaccine group (RNA-Seq, Neutrophils). Colored in red: up regulated from baseline; colored in green: down regulated from baseline. Genes were selected based on the KEGG antigen processing and presentation pathway.

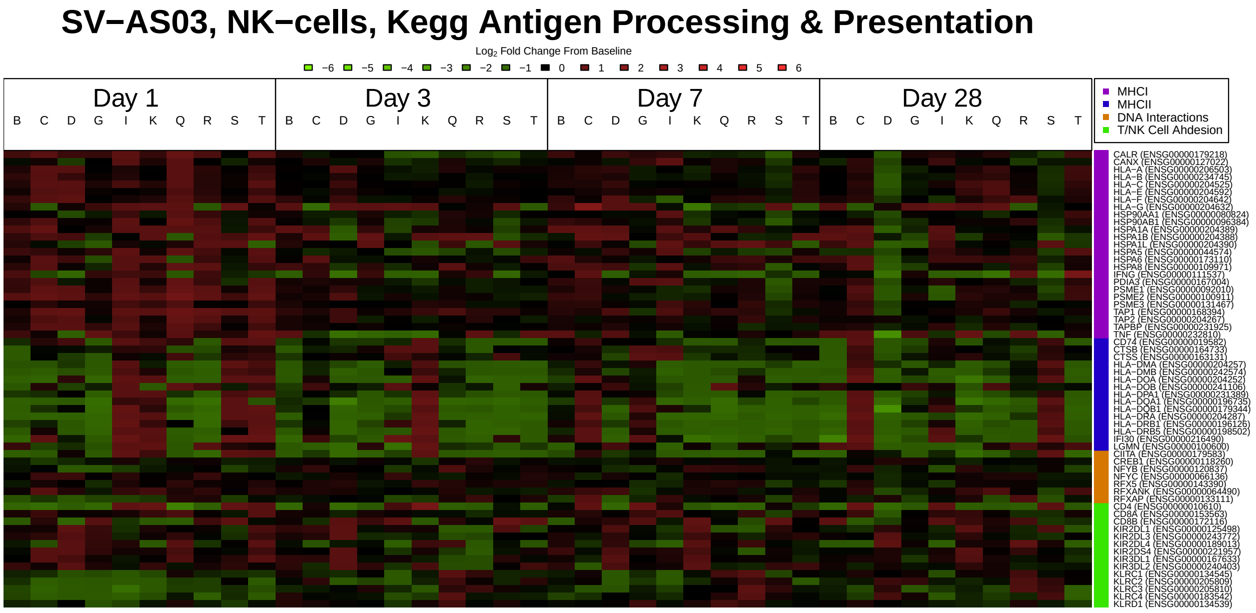

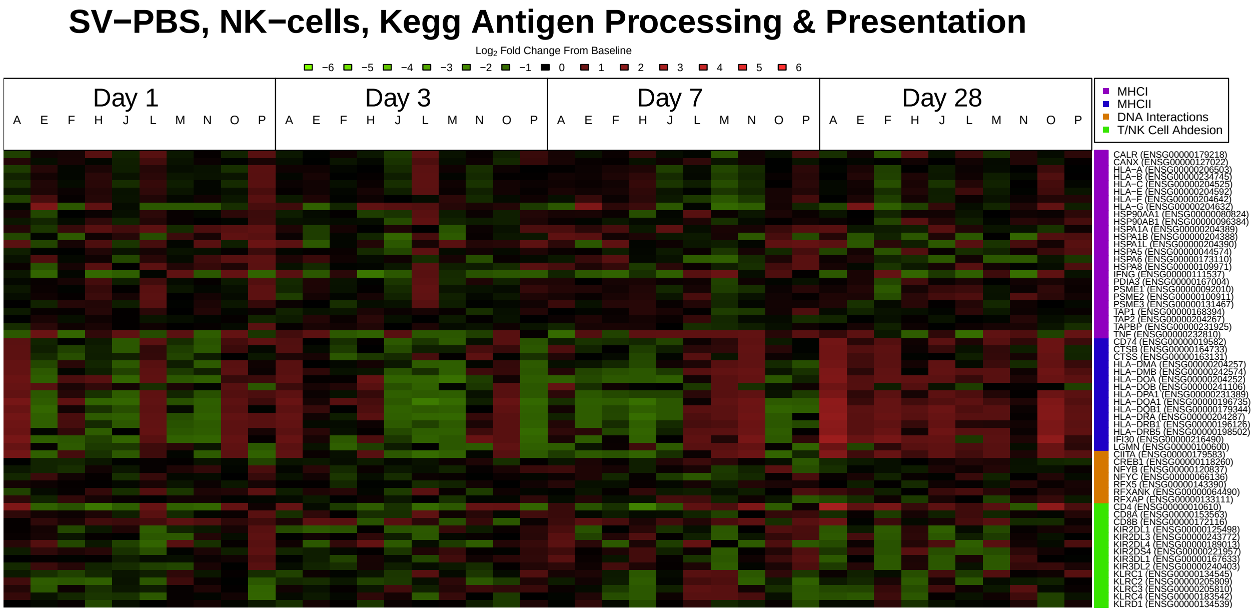

Figure A109: Heatmap of baseline log fold changes for known antigen presentation genes by vaccine group (RNA-Seq, NK-cells). Colored in red: up regulated from baseline; colored in green: down regulated from baseline. Genes were selected based on the KEGG antigen processing and presentation pathway.

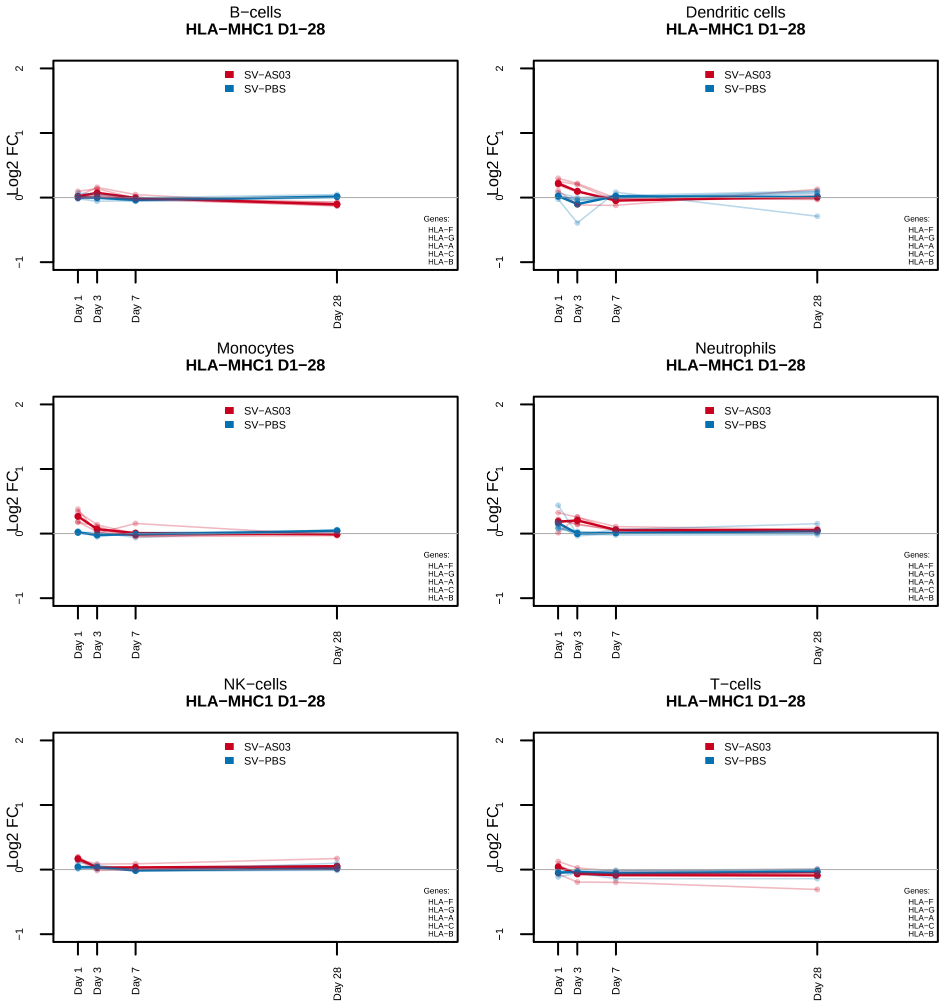

Figure A110: Time trends of baseline log fold change for MHC I genes by cell type and vaccine group (RNA-Seq). Mean log fold change across MHC I genes is drawn in bold. Individual gene mean log fold changes are plotted in lighter colors. Asterisks indicate that the gene was significantly differentially expressed for at least one of the post-vaccination days.

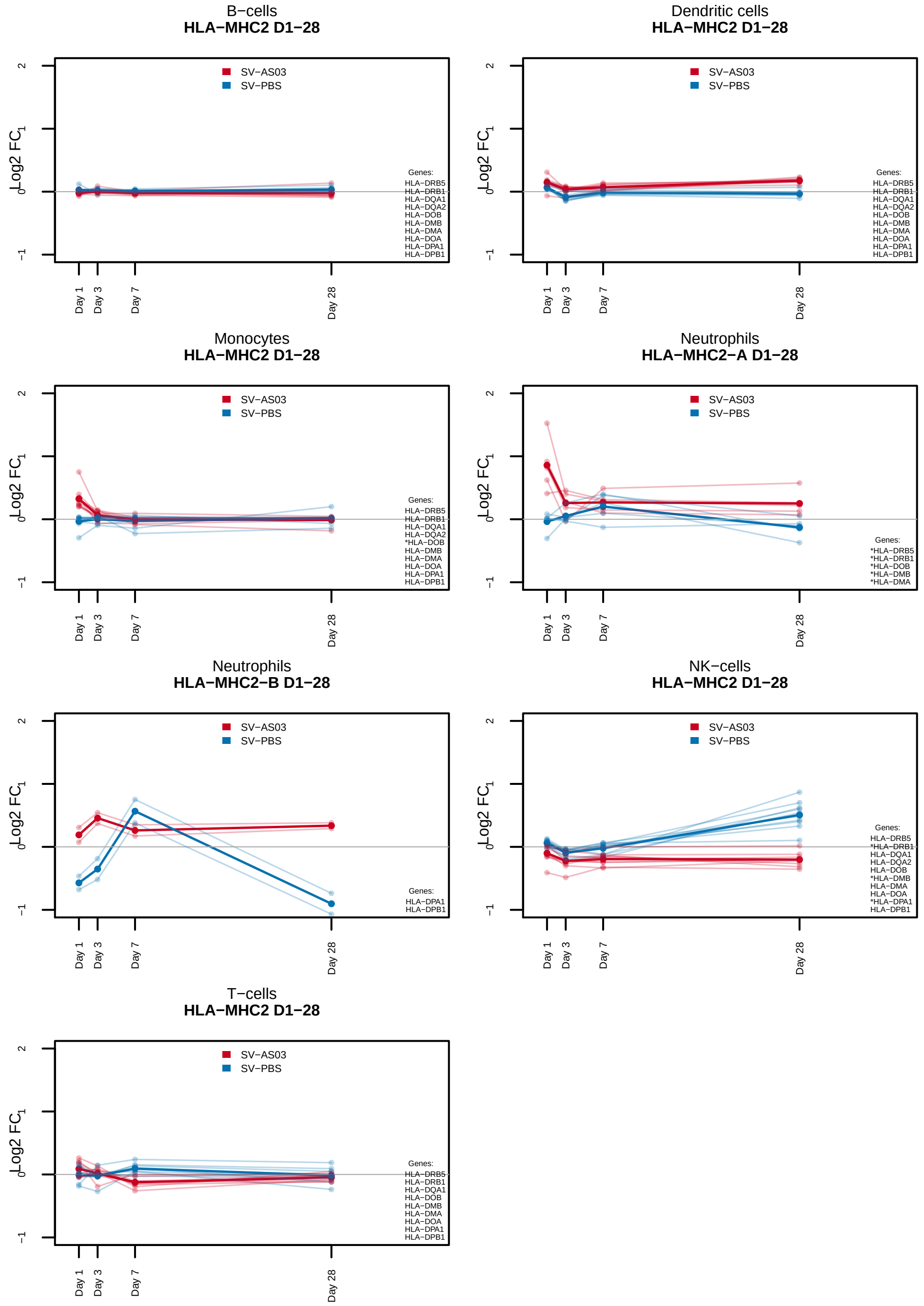

Figure A111: Time trends of baseline log fold change for MHC II genes by cell type and vaccine group (RNA-Seq). Mean log fold change across MHC II genes is drawn in bold. Individual gene mean log fold changes are plotted in lighter colors. Asterisks indicate that the gene was significantly differentially expressed for at least one of the post-vaccination days.

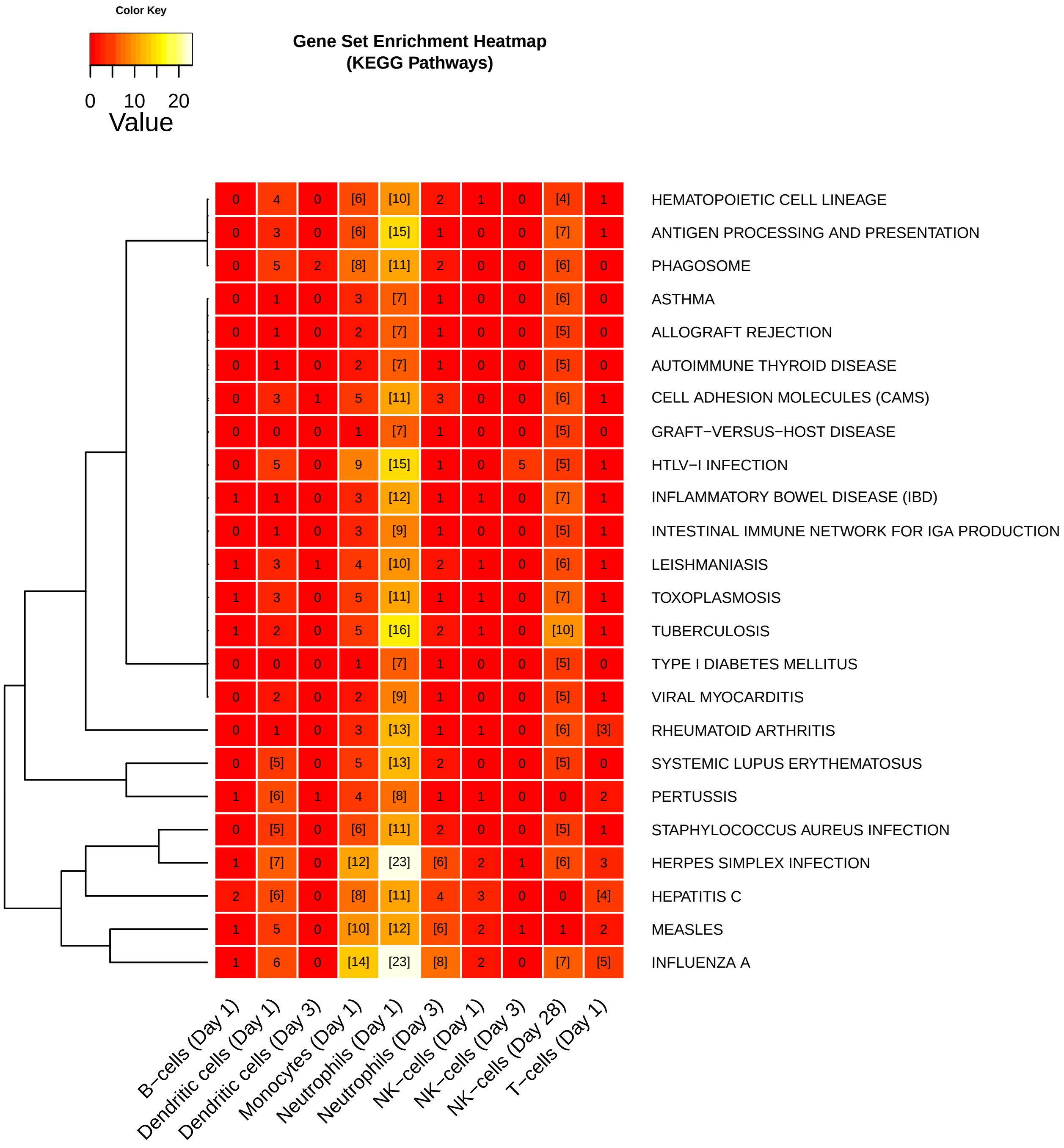

Figure A112: Heatmap of enriched KEGG Pathways (RNA-Seq). Gene sets significantly enriched in at least two conditions are shown. Cells are color-coded by the number of significant genes in a set. Cells with gene numbers in brackets indicate significantly enriched sets. Sets were clustered based on the Jaccard distance between their binary enrichment pattern.

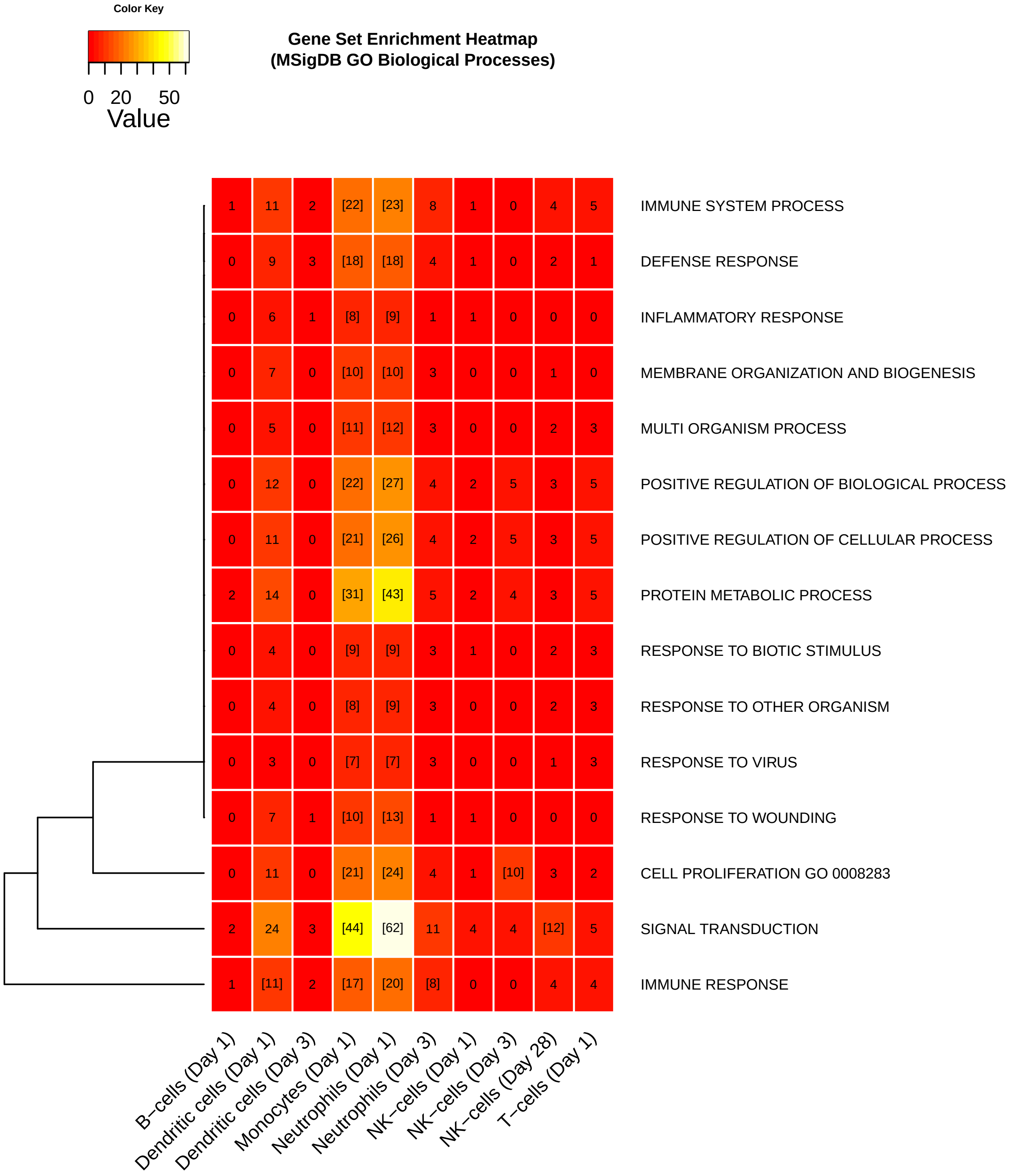

	B−cells (Day 1)	Dendritic cells (Day 1)	Dendritic cells (Day 3)	Monocytes (Day 1)	Neutrophils (Day 1)	Neutrophils (Day 3)	NK−cells (Day 1)	NK−cells (Day 3)	NK−cells (Day 28)	T−cells (Day 1)
IMMUNE SYSTEM PROCESS	1	11	2	[22]	[23]	8	1	0	4	5
DEFENSE RESPONSE	0	9	3	[18]	[18]	4	1	0	2	1
INFLAMMATORY RESPONSE	0	6	1	[8]	[9]	1	1	0	0	0
MEMBRANE ORGANIZATION AND BIOGENESIS	0	7	0	[10]	[10]	3	0	0	1	0
MULTI ORGANISM PROCESS	0	5	0	[11]	[12]	3	0	0	2	3
POSITIVE REGULATION OF BIOLOGICAL PROCESS	0	12	0	[22]	[27]	4	2	5	3	5
POSITIVE REGULATION OF CELLULAR PROCESS	0	11	0	[21]	[26]	4	2	5	3	5
PROTEIN METABOLIC PROCESS	2	14	0	[31]	[43]	5	2	4	3	5
RESPONSE TO BIOTIC STIMULUS	0	4	0	[9]	[9]	3	1	0	2	3
RESPONSE TO OTHER ORGANISM	0	4	0	[8]	[9]	3	0	0	2	3
RESPONSE TO VIRUS	0	3	0	[7]	[7]	3	0	0	1	3
RESPONSE TO WOUNDING	0	7	1	[10]	[13]	1	1	0	0	0
CELL PROLIFERATION GO 0008283	0	11	0	[21]	[24]	4	1	[10]	3	2
SIGNAL TRANSDUCTION	2	24	3	[44]	[62]	11	4	4	[12]	5
IMMUNE RESPONSE	1	[11]	2	[17]	[20]	[8]	0	0	4	4

Figure A113: Heatmap of enriched MSigDB GO Biological Processes (RNA-Seq). Gene sets significantly enriched in at least two conditions are shown. Cells are color-coded by the number of significant genes in a set. Cells with gene numbers in brackets indicate significantly enriched sets. Sets were clustered based on the Jaccard distance between their binary enrichment pattern.

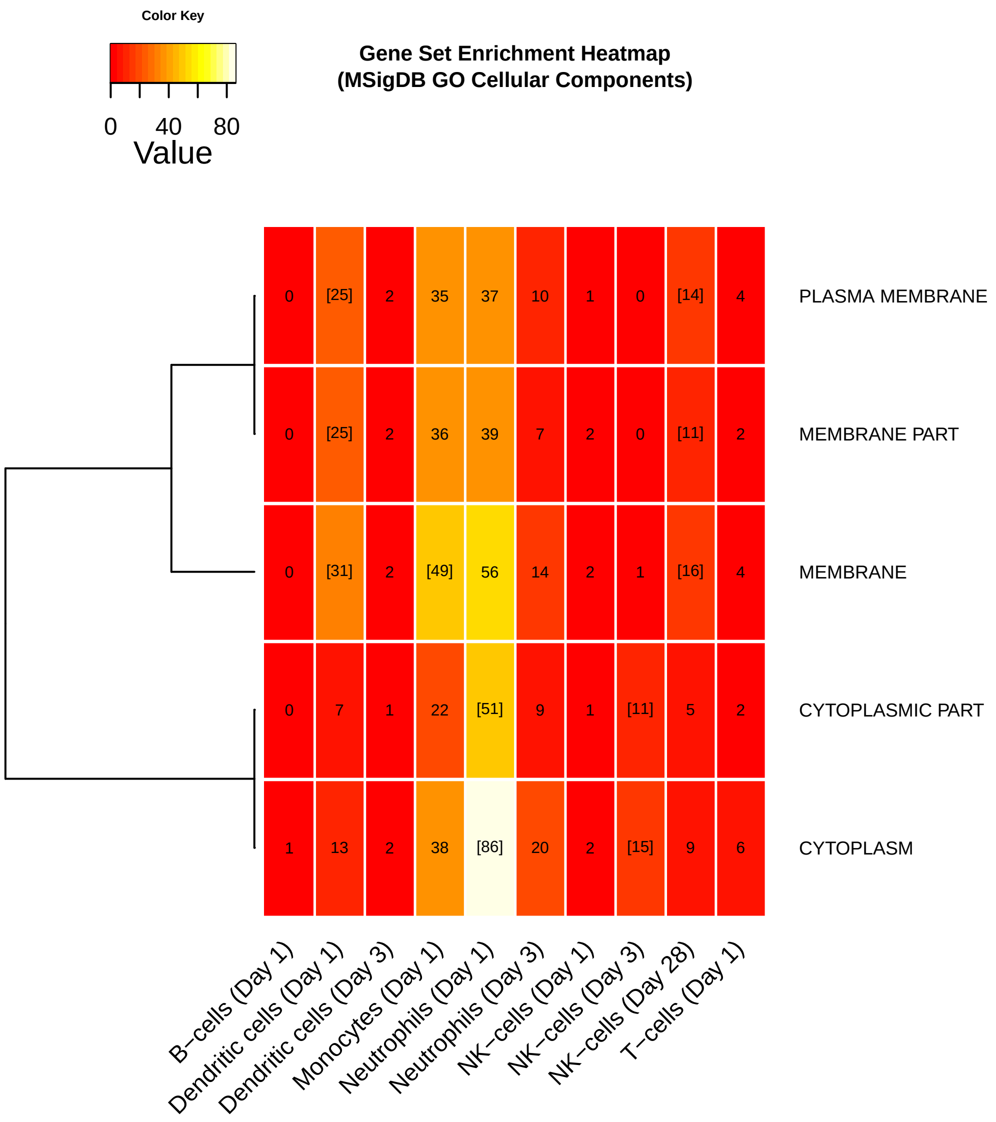

Figure A114: Heatmap of enriched MSigDB GO Cellular Components (RNA-Seq). Gene sets significantly enriched in at least two conditions are shown. Cells are color-coded by the number of significant genes in a set. Cells with gene numbers in brackets indicate significantly enriched sets. Sets were clustered based on the Jaccard distance between their binary enrichment pattern.

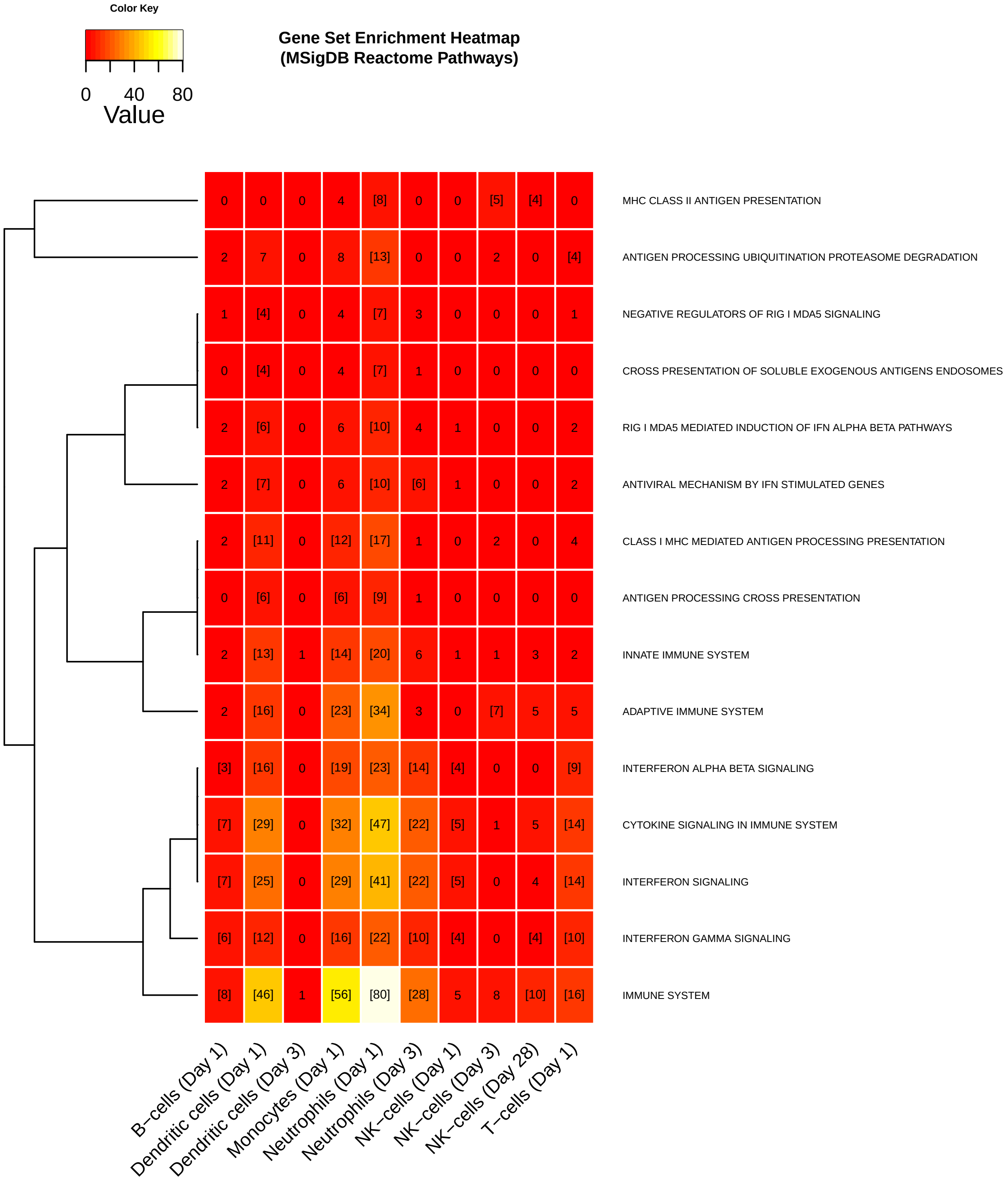

Figure A115: Heatmap of enriched MSigDB Reactome Pathways (RNA-Seq). Gene sets significantly enriched in at least two conditions are shown. Cells are color-coded by the number of significant genes in a set. Cells with gene numbers in brackets indicate significantly enriched sets. Sets were clustered based on the Jaccard distance between their binary enrichment pattern.

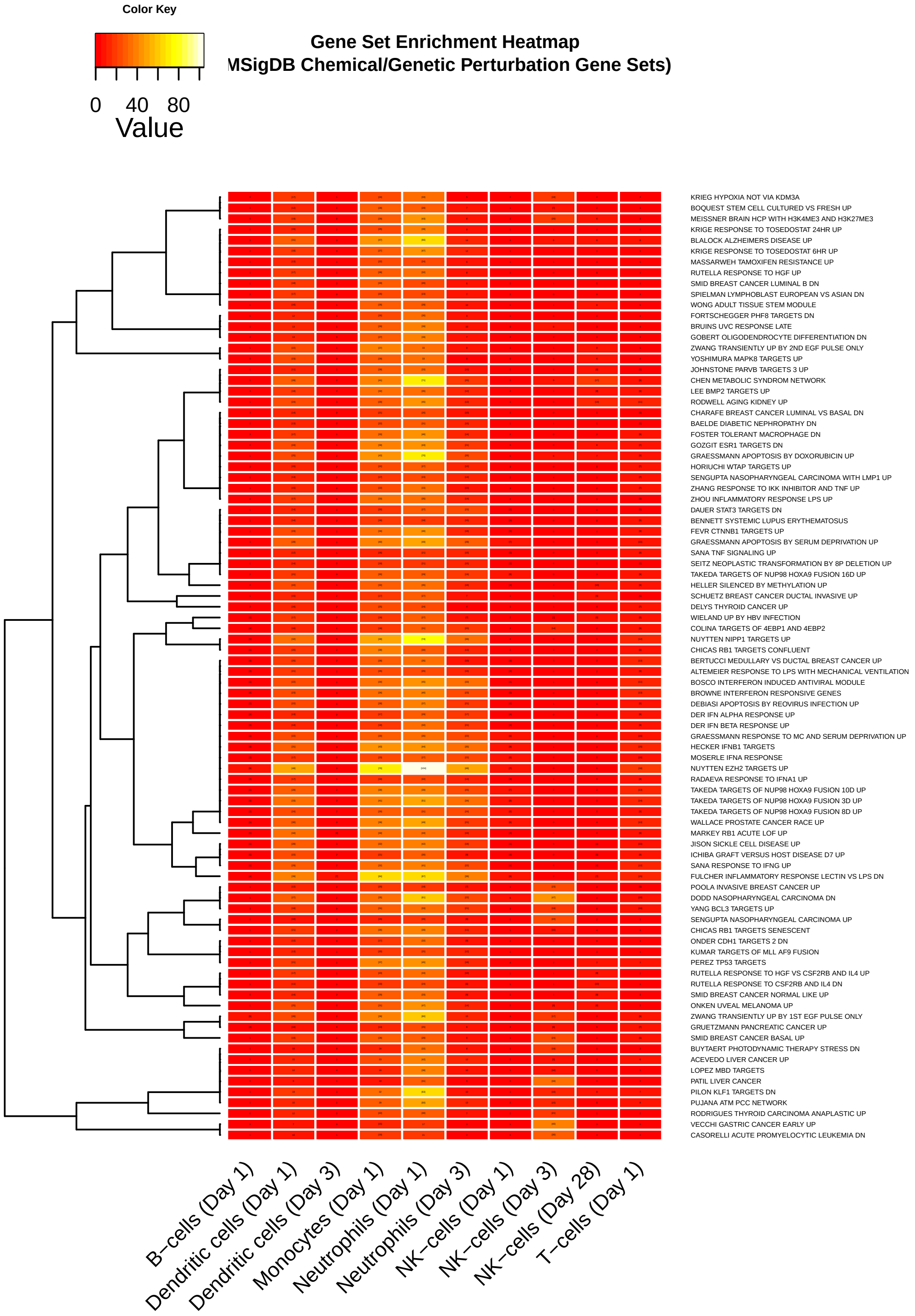

Figure A116: Heatmap of enriched MSigDB Chemical/Genetic Perturbation gene sets (RNA-Seq). Gene sets significantly enriched in at least two conditions are shown. Cells are color-coded by the number of significant genes in a set. Cells with gene numbers in brackets indicate significantly enriched sets. Sets were clustered based on the Jaccard distance between their binary enrichment pattern. Gene sets were further filtered to only include sets whose sum of gene counts across conditions exceeded the 75% quantile of all sets.

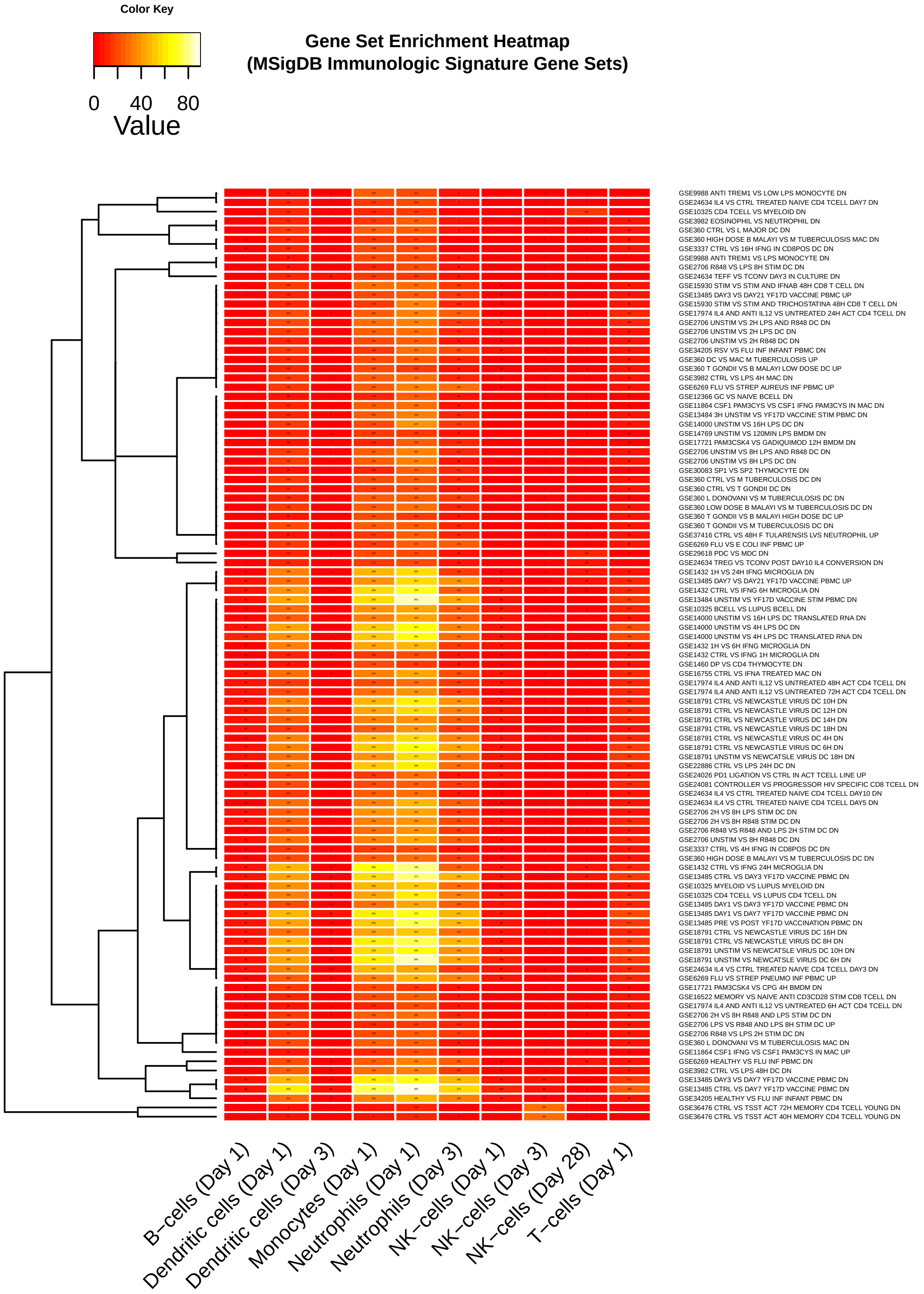

Figure A117: Heatmap of enriched MSigDB Immunologic Signature gene sets (RNA-Seq). Gene sets significantly enriched in at least two conditions are shown. Cells are color-coded by the number of significant genes in a set. Cells with gene numbers in brackets indicate significantly enriched sets. Sets were clustered based on the Jaccard distance between their binary enrichment pattern. Gene sets were further filtered to only include sets whose sum of gene counts across conditions exceeded the 75% quantile of all sets.

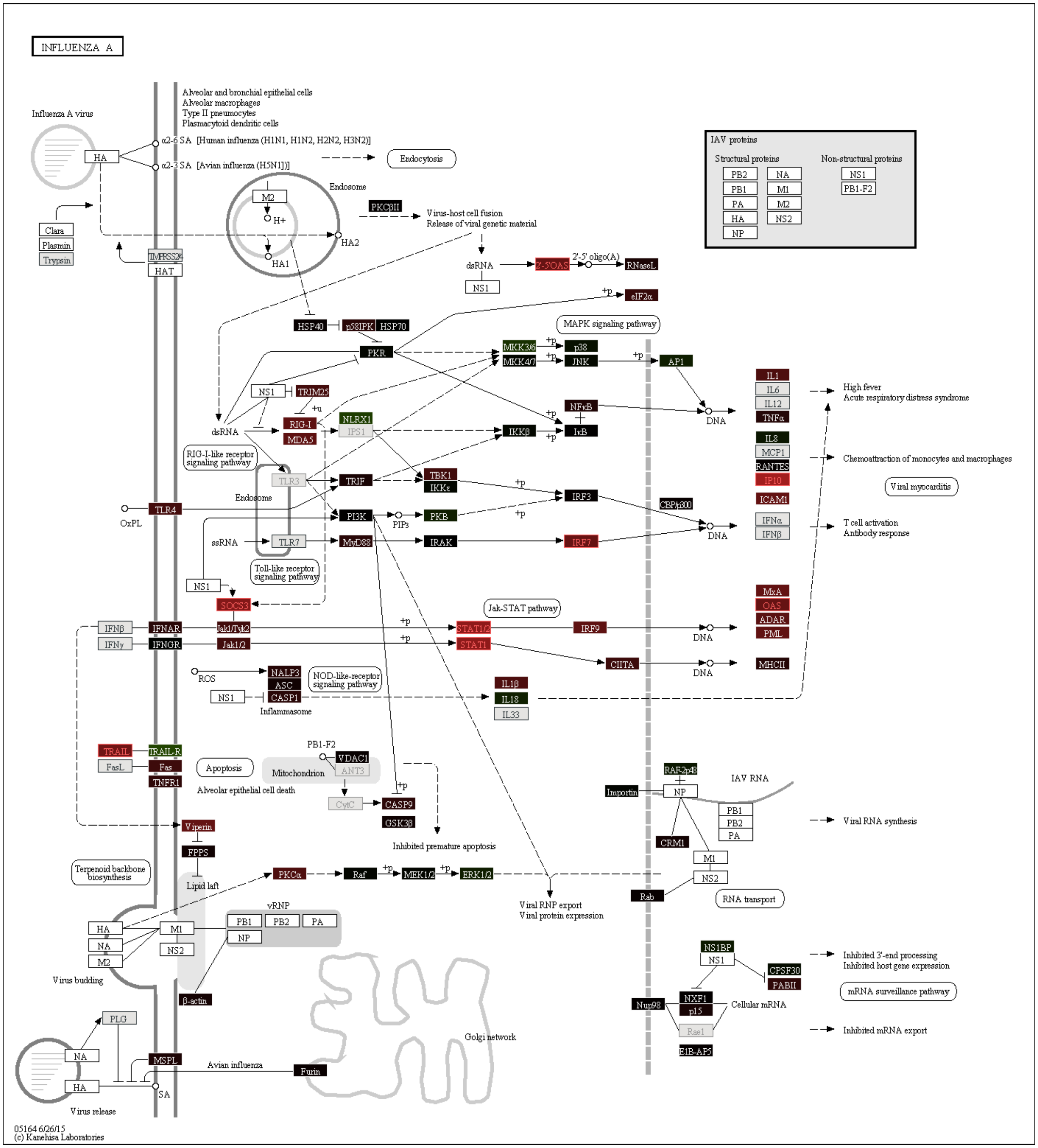

Figure A118: KEGG Pathway Map - Influenza A (RNA-Seq, Dendritic cells, Day 1). Node color gradient encodes fold change between vaccine groups (for multi-gene nodes the median fold change is used). In red: increased log fold change response for the SV-AS03 group relative to the SV-PBS group, in green: decreased log fold change response for the SV-AS03 group relative to the SV-AS03 group, and vice versa, in black: fold change close to 1, in dark grey: genes filtered out due to low overall expression, light grey: gene missing database mapping, white: non-human gene. Statistically significant fold changes are highlighted using red and green label colors.

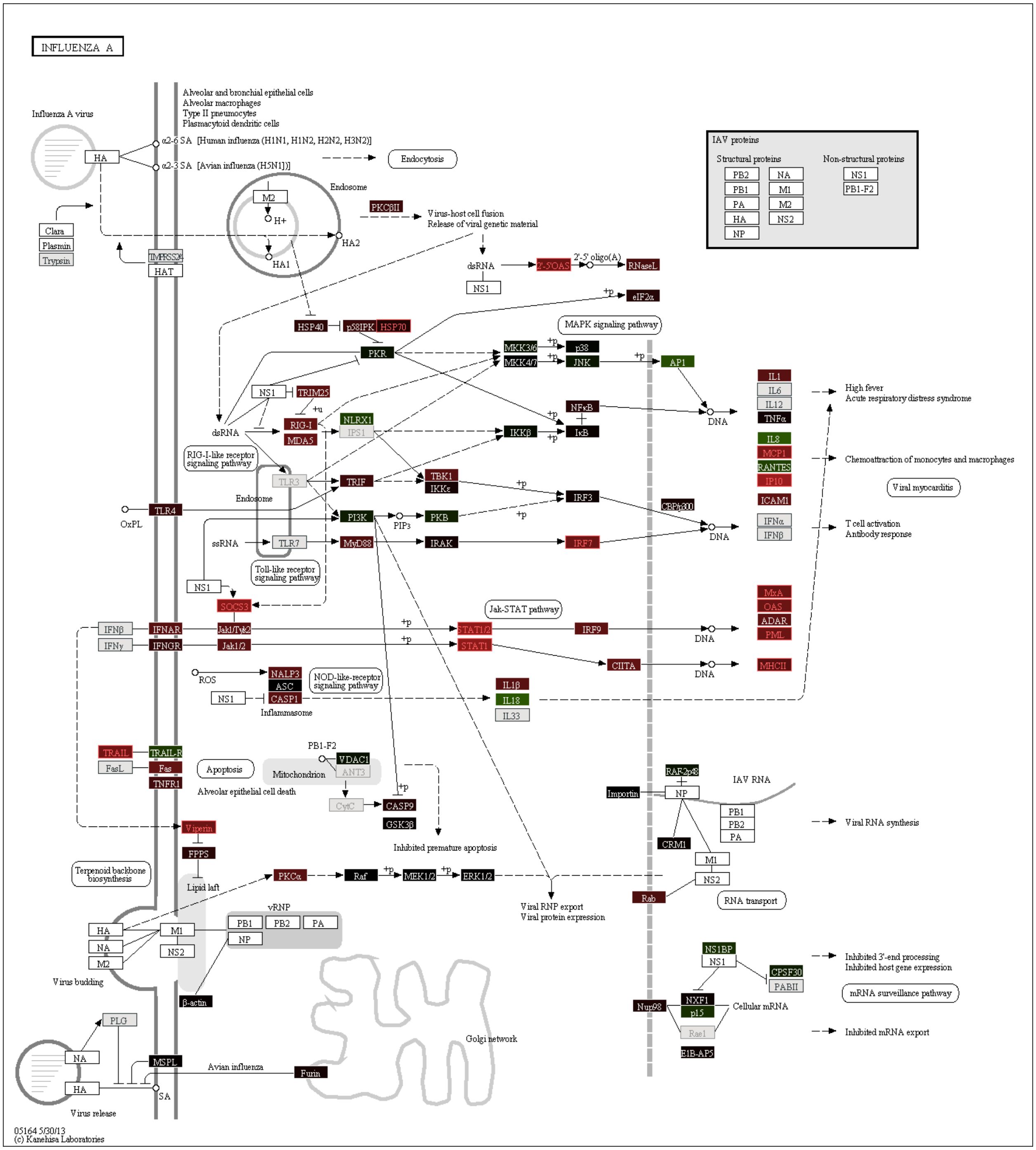

Figure A119: KEGG Pathway Map - Influenza A (RNA-Seq, Monocytes, Day 1). Node color gradient encodes fold change between vaccine groups (for multi-gene nodes the median fold change is used). In red: increased log fold change response for the SV-AS03 group relative to the SV-PBS group, in green: decreased log fold change response for the SV-AS03 group relative to the SV-AS03 group, and vice versa, in black: fold change close to 1, in dark grey: genes filtered out due to low overall expression, light grey: gene missing database mapping, white: non-human gene. Statistically significant fold changes are highlighted using red and green label colors.

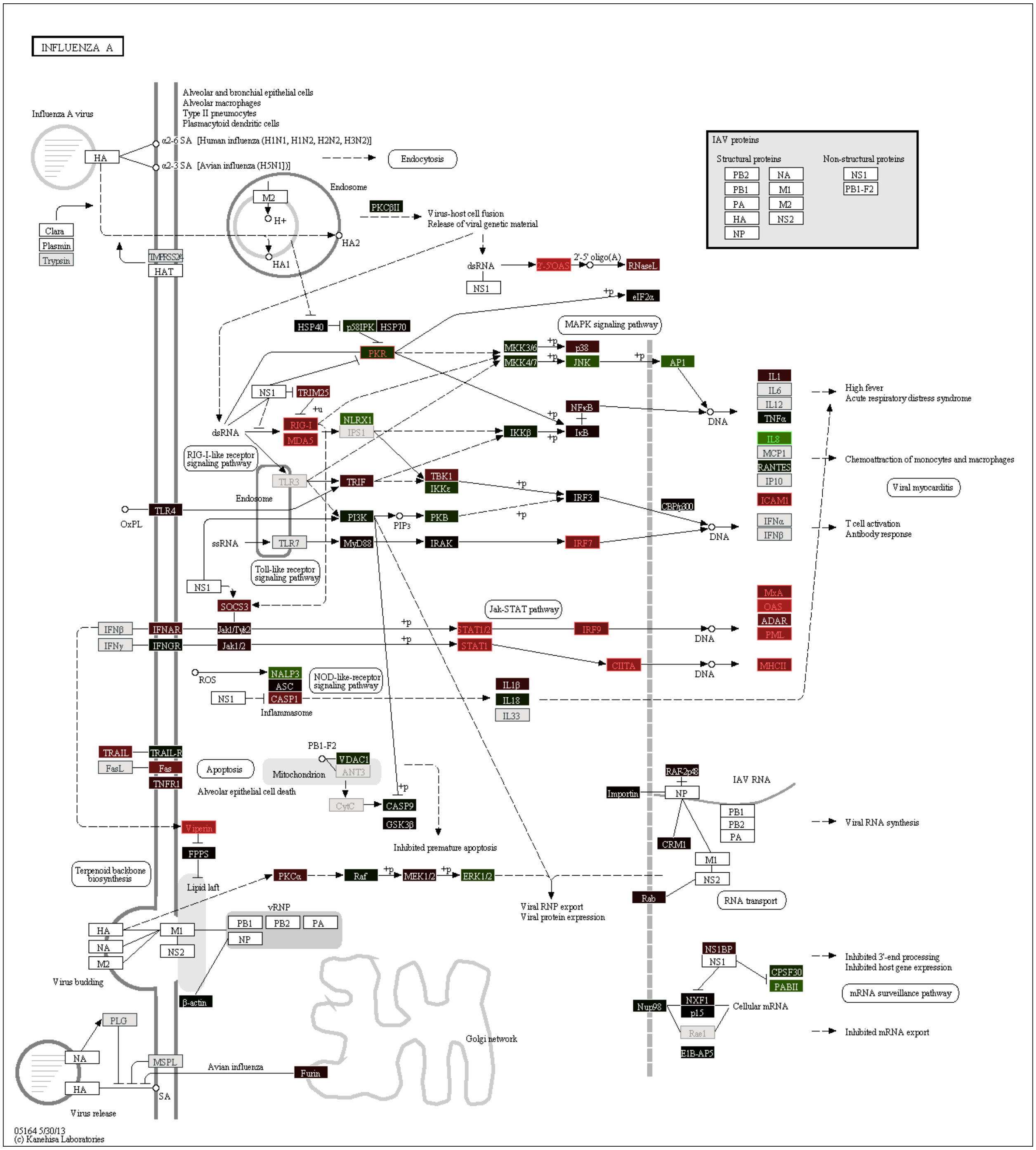

Figure A120: KEGG Pathway Map - Influenza A (RNA-Seq, Neutrophils, Day 1). Node color gradient encodes fold change between vaccine groups (for multi-gene nodes the median fold change is used). In red: increased log fold change response for the SV-AS03 group relative to the SV-PBS group, in green: decreased log fold change response for the SV-AS03 group relative to the SV-AS03 group, and vice versa, in black: fold change close to 1, in dark grey: genes filtered out due to low overall expression, light grey: gene missing database mapping, white: non-human gene. Statistically significant fold changes are highlighted using red and green label colors.

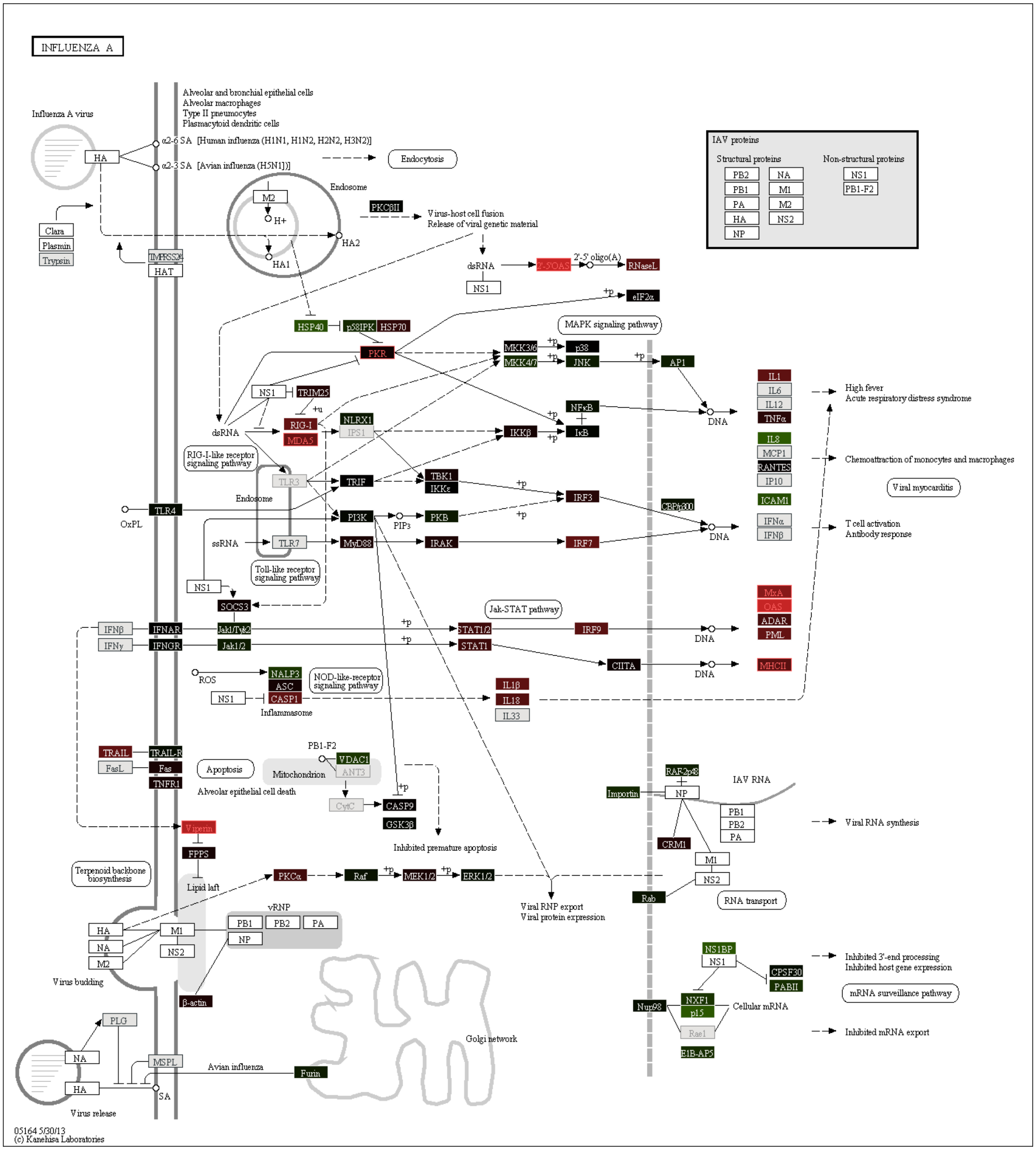

Figure A121: KEGG Pathway Map - Influenza A (RNA-Seq, Neutrophils, Day 3). Node color gradient encodes fold change between vaccine groups (for multi-gene nodes the median fold change is used). In red: increased log fold change response for the SV-AS03 group relative to the SV-PBS group, in green: decreased log fold change response for the SV-AS03 group relative to the SV-AS03 group, and vice versa, in black: fold change close to 1, in dark grey: genes filtered out due to low overall expression, light grey: gene missing database mapping, white: non-human gene. Statistically significant fold changes are highlighted using red and green label colors.

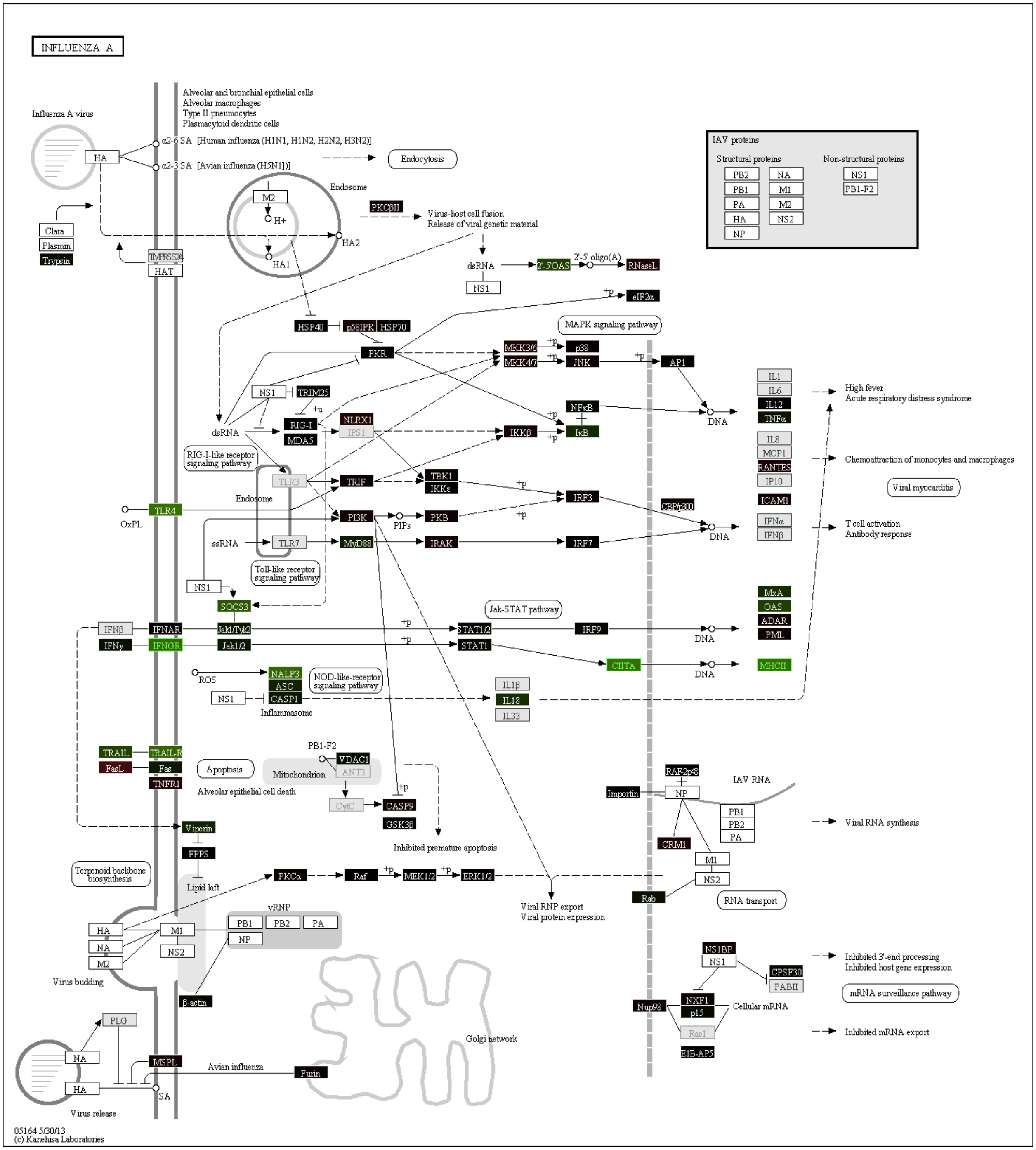

Figure A122: KEGG Pathway Map - Influenza A (RNA-Seq, NK-cells, Day 28). Node color gradient encodes fold change between vaccine groups (for multi-gene nodes the median fold change is used). In red: increased log fold change response for the SV-AS03 group relative to the SV-PBS group, in green: decreased log fold change response for the SV-AS03 group relative to the SV-AS03 group, and vice versa, in black: fold change close to 1, in dark grey: genes filtered out due to low overall expression, light grey: gene missing database mapping, white: non-human gene. Statistically significant fold changes are highlighted using red and green label colors.

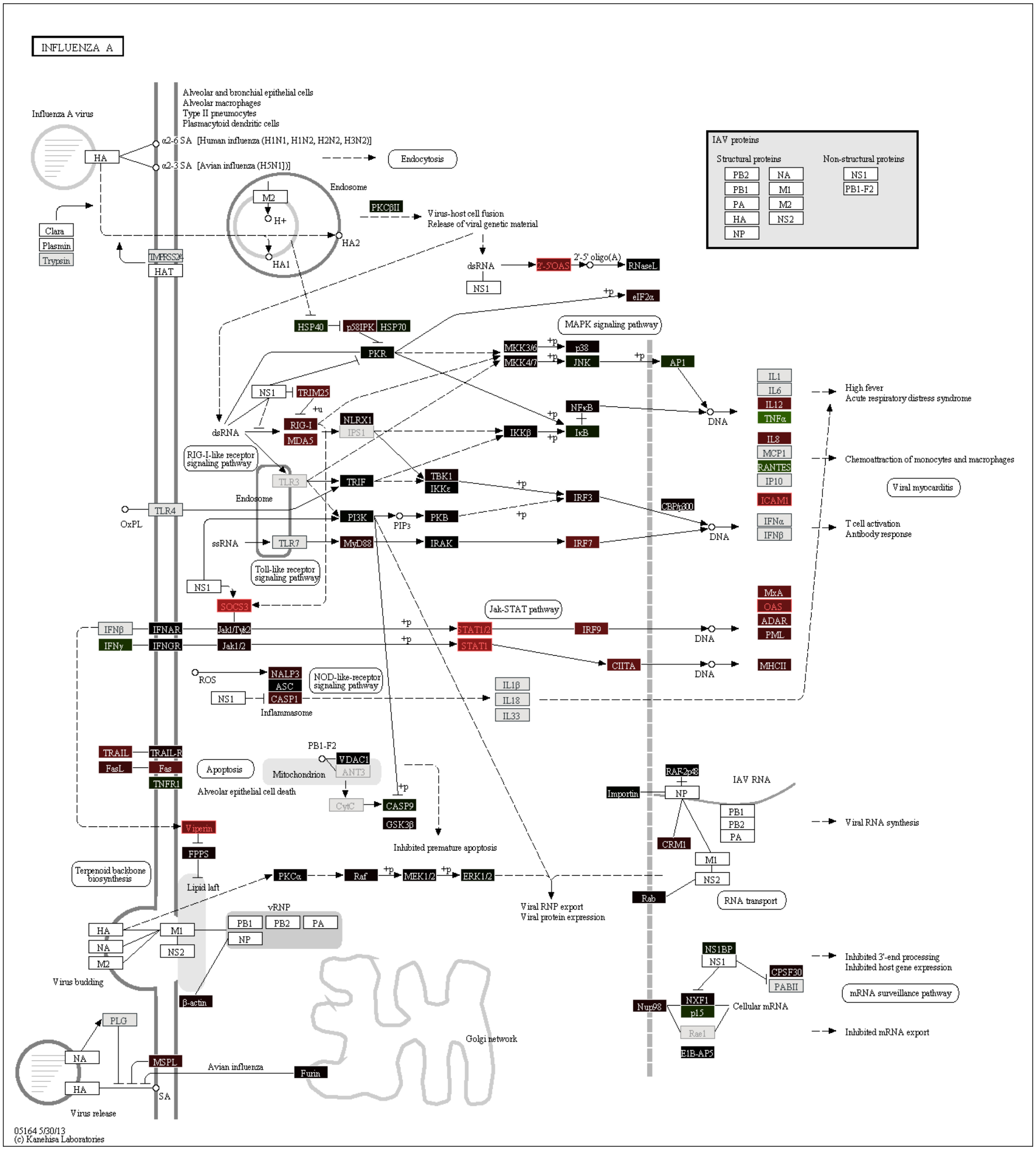

Figure A123: KEGG Pathway Map - Influenza A (RNA-Seq, T-cells, Day 1). Node color gradient encodes fold change between vaccine groups (for multi-gene nodes the median fold change is used). In red: increased log fold change response for the SV-AS03 group relative to the SV-PBS group, in green: decreased log fold change response for the SV-AS03 group relative to the SV-AS03 group, and vice versa, in black: fold change close to 1, in dark grey: genes filtered out due to low overall expression, light grey: gene missing database mapping, white: non-human gene. Statistically significant fold changes are highlighted using red and green label colors.

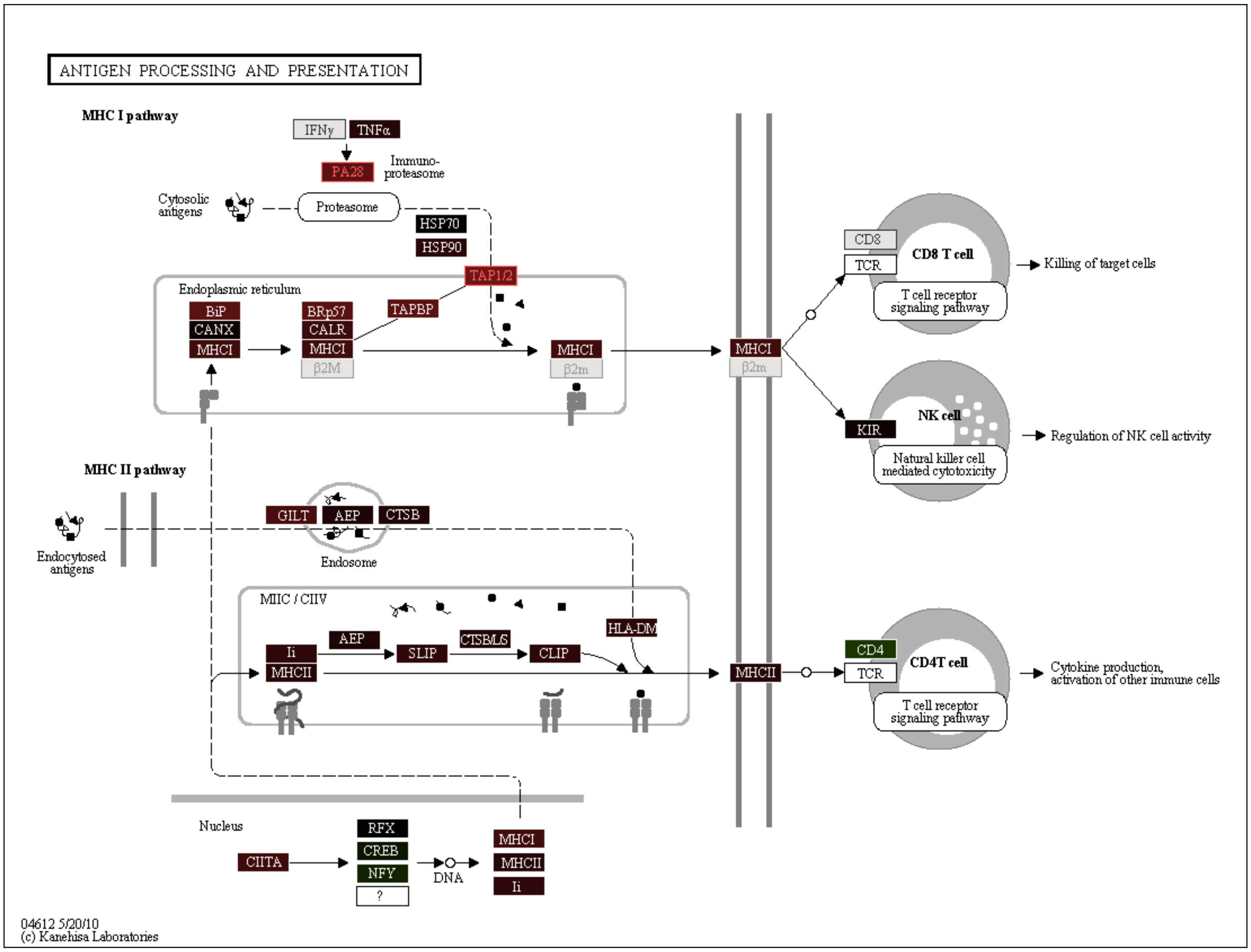

Figure A124: KEGG Pathway Map - Antigen processing and presentation (RNA-Seq, Dendritic cells, Day 1). Node color gradient encodes fold change between vaccine groups (for multi-gene nodes the median fold change is used). In red: increased log fold change response for the SV-AS03 group relative to the SV-PBS group, in green: decreased log fold change response for the SV-AS03 group relative to the SV-AS03 group, and vice versa, in black: fold change close to 1, in dark grey: genes filtered out due to low overall expression, light grey: gene missing database mapping, white: non-human gene. Statistically significant fold changes are highlighted using red and green label colors.

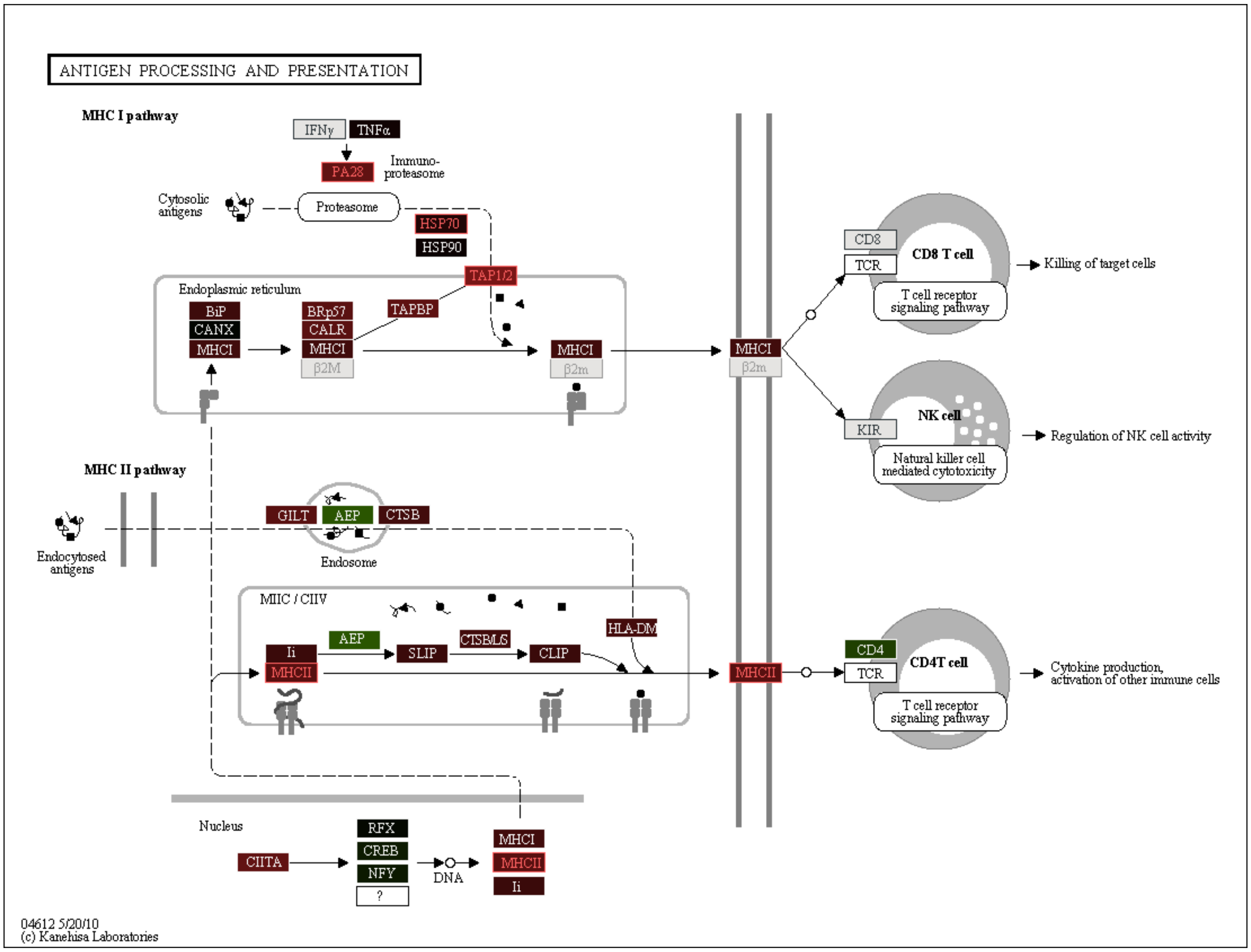

Figure A125: KEGG Pathway Map - Antigen processing and presentation (RNA-Seq, Monocytes, Day 1). Node color gradient encodes fold change between vaccine groups (for multi-gene nodes the median fold change is used). In red: increased log fold change response for the SV-AS03 group relative to the SV-PBS group, in green: decreased log fold change response for the SV-AS03 group relative to the SV-AS03 group, and vice versa, in black: fold change close to 1, in dark grey: genes filtered out due to low overall expression, light grey: gene missing database mapping, white: non-human gene. Statistically significant fold changes are highlighted using red and green label colors.

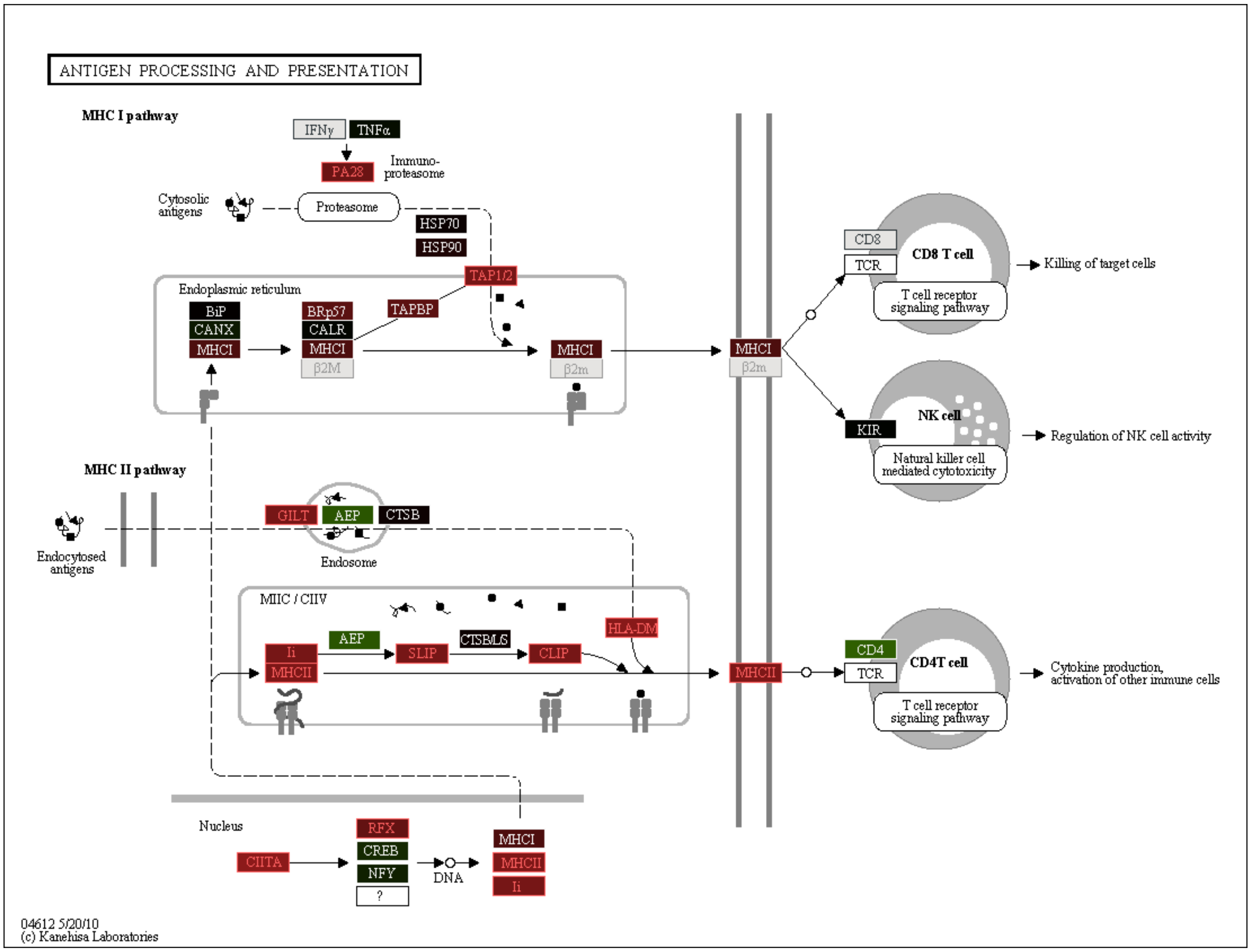

Figure A126: KEGG Pathway Map - Antigen processing and presentation (RNA-Seq, Neutrophils, Day 1). Node color gradient encodes fold change between vaccine groups (for multi-gene nodes the median fold change is used). In red: increased log fold change response for the SV-AS03 group relative to the SV-PBS group, in green: decreased log fold change response for the SV-AS03 group relative to the SV-AS03 group, and vice versa, in black: fold change close to 1, in dark grey: genes filtered out due to low overall expression, light grey: gene missing database mapping, white: non-human gene. Statistically significant fold changes are highlighted using red and green label colors.

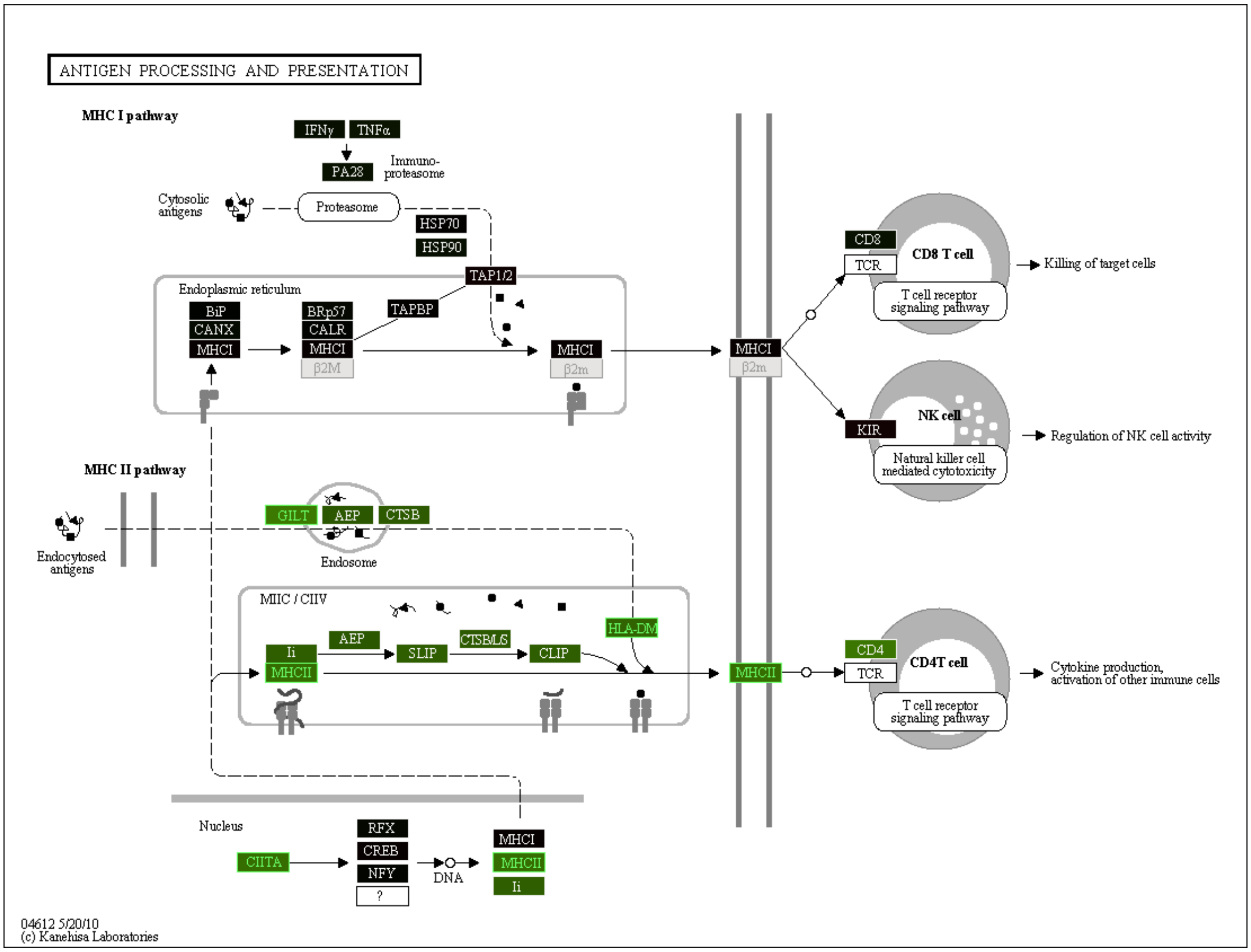

Figure A127: KEGG Pathway Map - Antigen processing and presentation (RNA-Seq, NK-cells, Day 28). Node color gradient encodes fold change between vaccine groups (for multi-gene nodes the median fold change is used). In red: increased log fold change response for the SV-AS03 group relative to the SV-PBS group, in green: decreased log fold change response for the SV-AS03 group relative to the SV-AS03 group, and vice versa, in black: fold change close to 1, in dark grey: genes filtered out due to low overall expression, light grey: gene missing database mapping, white: non-human gene. Statistically significant fold changes are highlighted using red and green label colors.

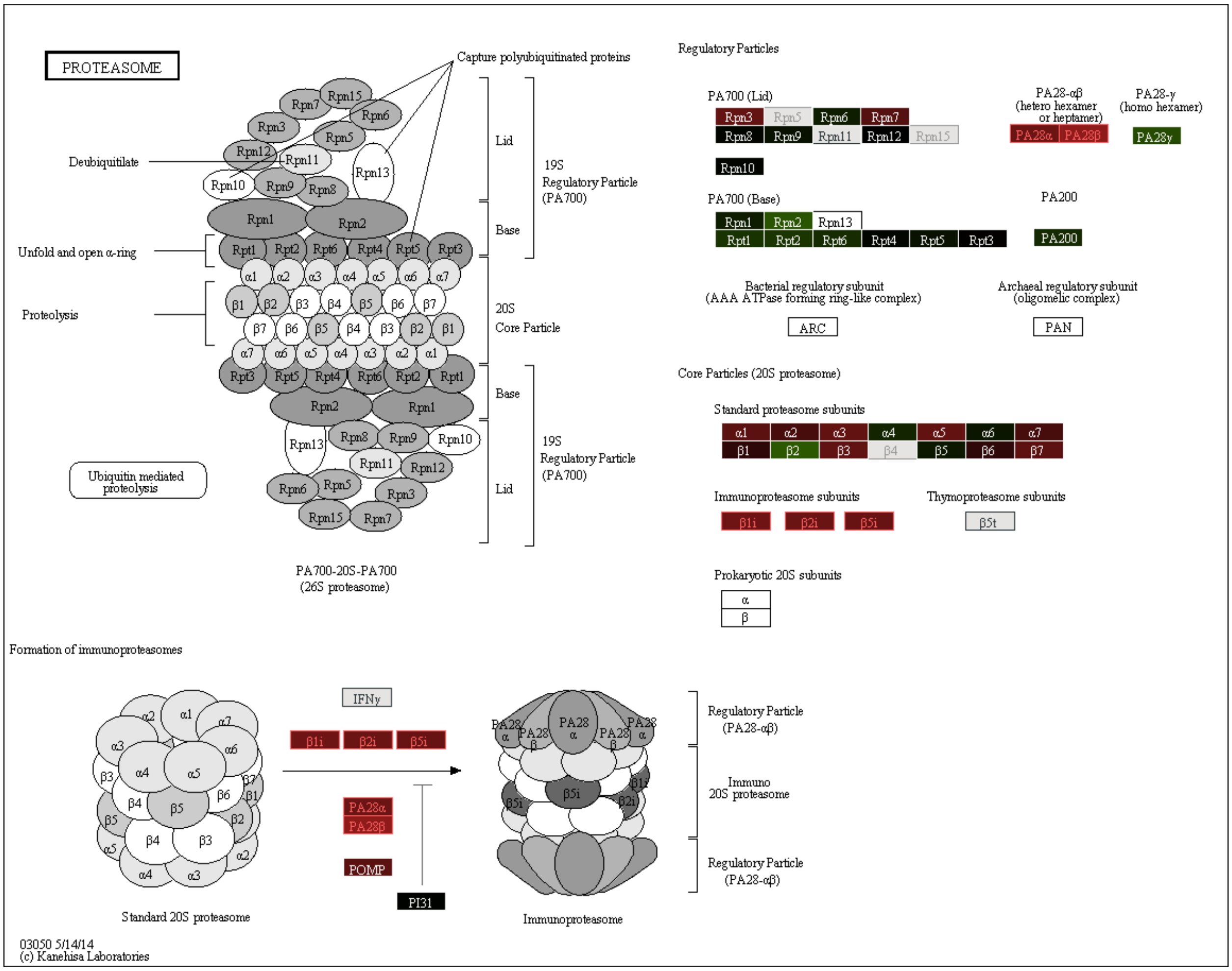

Figure A128: KEGG Pathway Map - Proteasome (RNA-Seq, Neutrophils, Day 1). Node color gradient encodes fold change between vaccine groups (for multi-gene nodes the median fold change is used). In red: increased log fold change response for the SV-AS03 group relative to the SV-PBS group, in green: decreased log fold change response for the SV-AS03 group relative to the SV-AS03 group, and vice versa, in black: fold change close to 1, in dark grey: genes filtered out due to low overall expression, light grey: gene missing database mapping, white: non-human gene. Statistically significant fold changes are highlighted using red and green label colors.

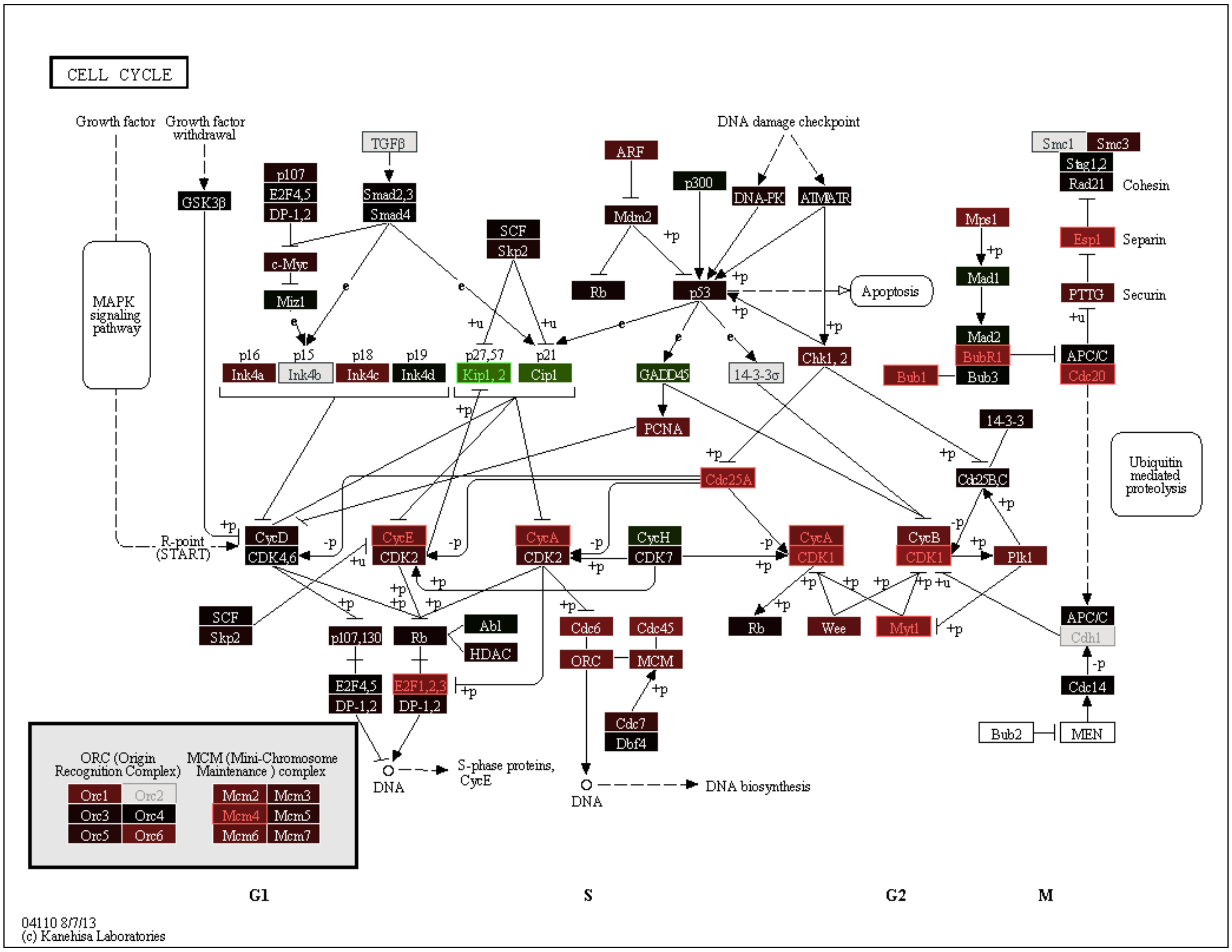

Figure A129: KEGG Pathway Map - Cell cycle (RNA-Seq, NK-cells, Day 3). Node color gradient encodes fold change between vaccine groups (for multi-gene nodes the median fold change is used). In red: increased log fold change response for the SV-AS03 group relative to the SV-PBS group, in green: decreased log fold change response for the SV-AS03 group relative to the SV-AS03 group, and vice versa, in black: fold change close to 1, in dark grey: genes filtered out due to low overall expression, light grey: gene missing database mapping, white: non-human gene. Statistically significant fold changes are highlighted using red and green label colors.

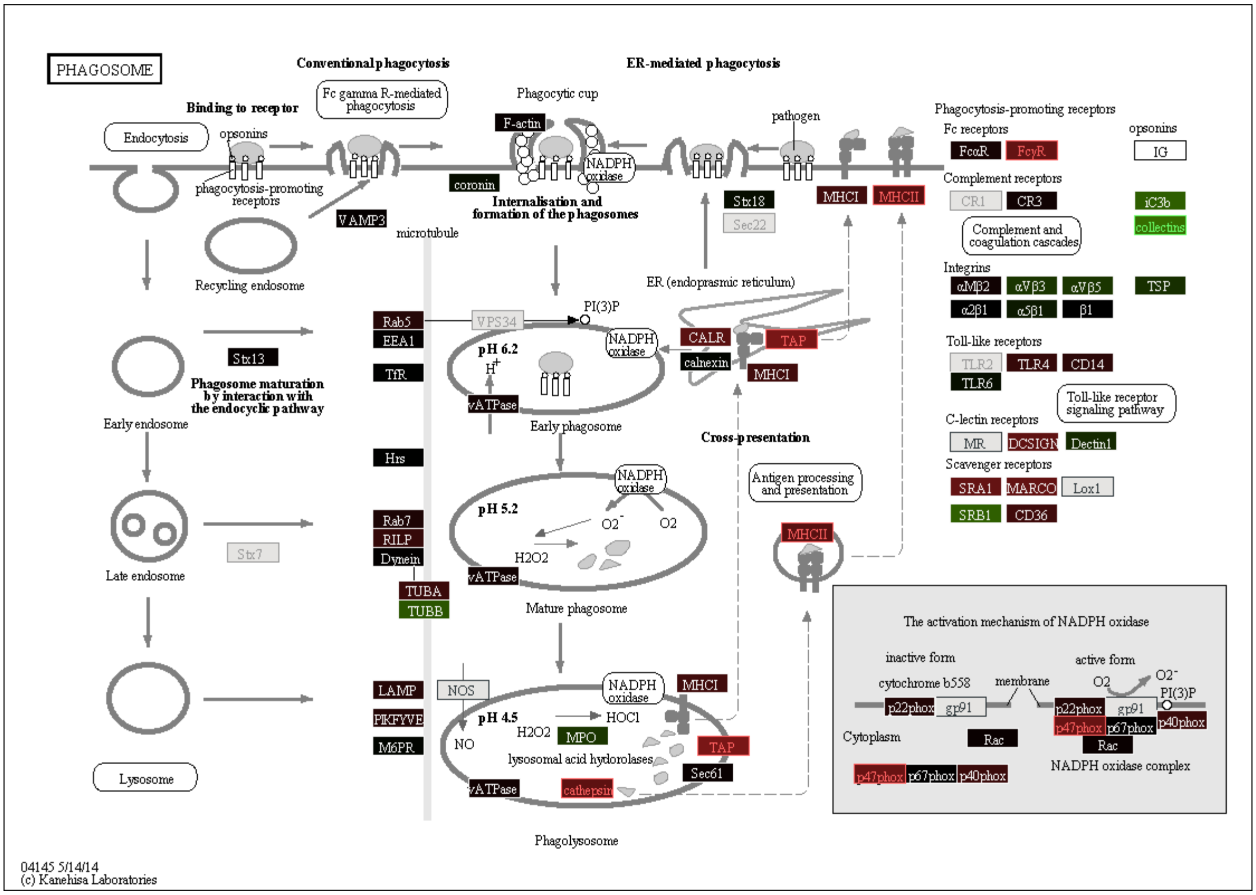

Figure A130: KEGG Pathway Map - Phagosome (RNA-Seq, Monocytes, Day 1). Node color gradient encodes fold change between vaccine groups (for multi-gene nodes the median fold change is used). In red: increased log fold change response for the SV-AS03 group relative to the SV-PBS group, in green: decreased log fold change response for the SV-AS03 group relative to the SV-AS03 group, and vice versa, in black: fold change close to 1, in dark grey: genes filtered out due to low overall expression, light grey: gene missing database mapping, white: non-human gene. Statistically significant fold changes are highlighted using red and green label colors.

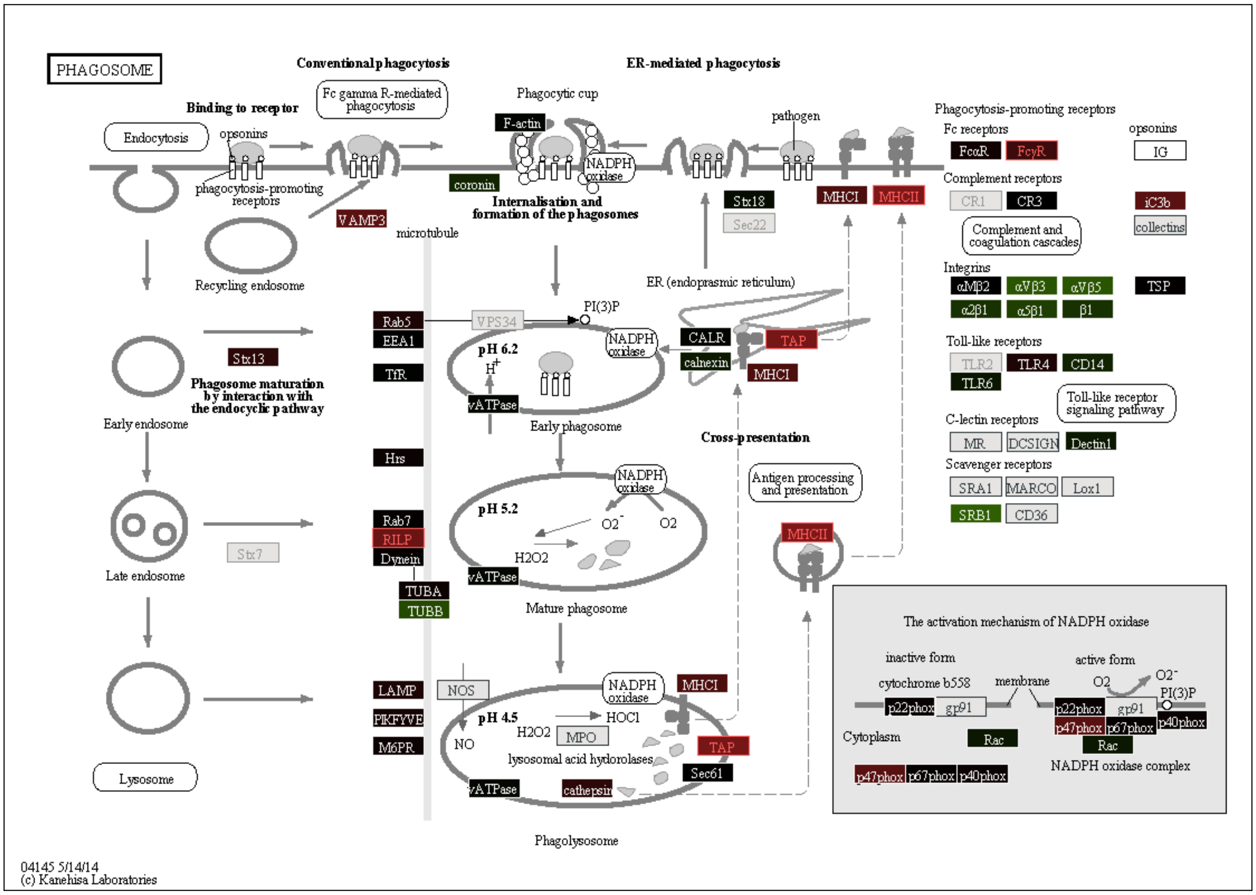

Figure A131: KEGG Pathway Map - Phagosome (RNA-Seq, Neutrophils, Day 1). Node color gradient encodes fold change between vaccine groups (for multi-gene nodes the median fold change is used). In red: increased log fold change response for the SV-AS03 group relative to the SV-PBS group, in green: decreased log fold change response for the SV-AS03 group relative to the SV-AS03 group, and vice versa, in black: fold change close to 1, in dark grey: genes filtered out due to low overall expression, light grey: gene missing database mapping, white: non-human gene. Statistically significant fold changes are highlighted using red and green label colors.

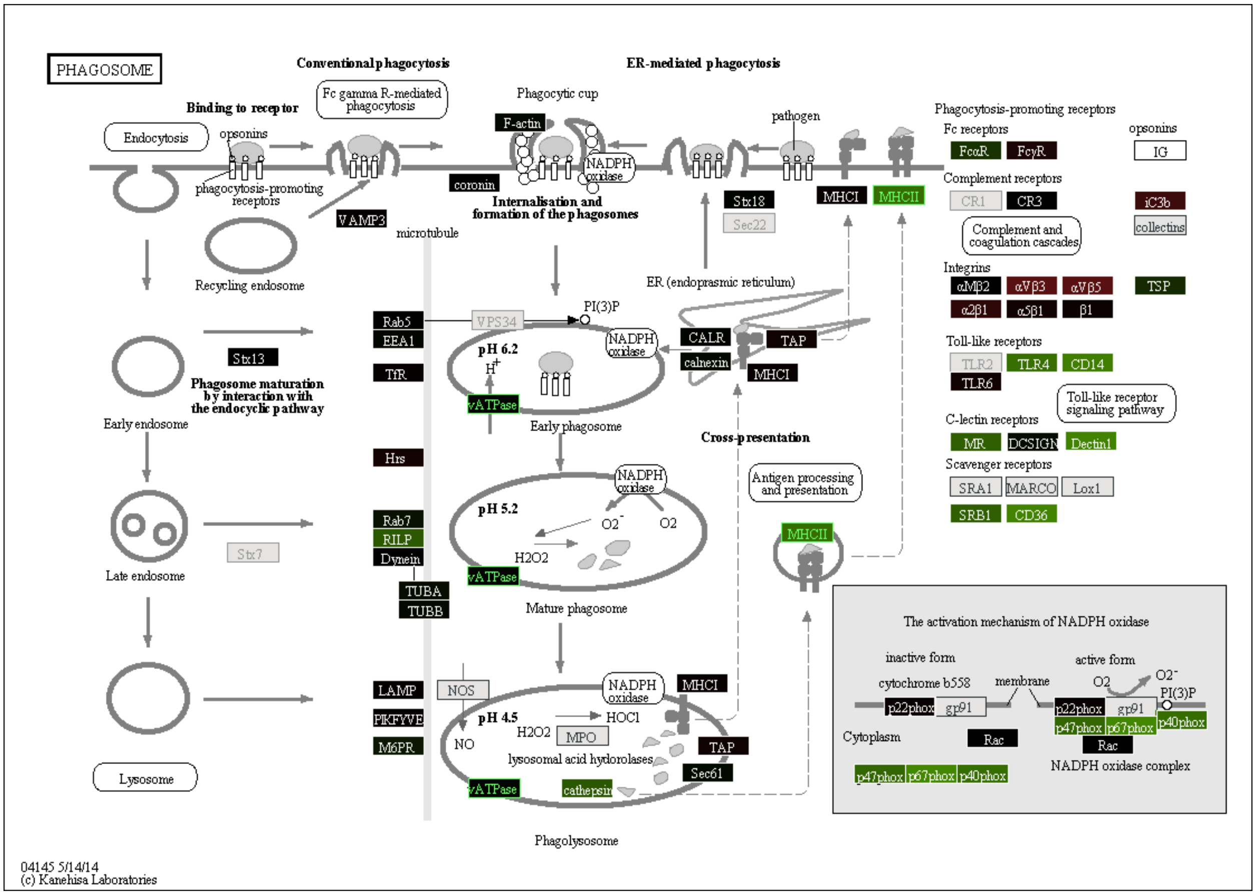

Figure A132: KEGG Pathway Map - Phagosome (RNA-Seq, NK-cells, Day 28). Node color gradient encodes fold change between vaccine groups (for multi-gene nodes the median fold change is used). In red: increased log fold change response for the SV-AS03 group relative to the SV-PBS group, in green: decreased log fold change response for the SV-AS03 group relative to the SV-AS03 group, and vice versa, in black: fold change close to 1, in dark grey: genes filtered out due to low overall expression, light grey: gene missing database mapping, white: non-human gene. Statistically significant fold changes are highlighted using red and green label colors.

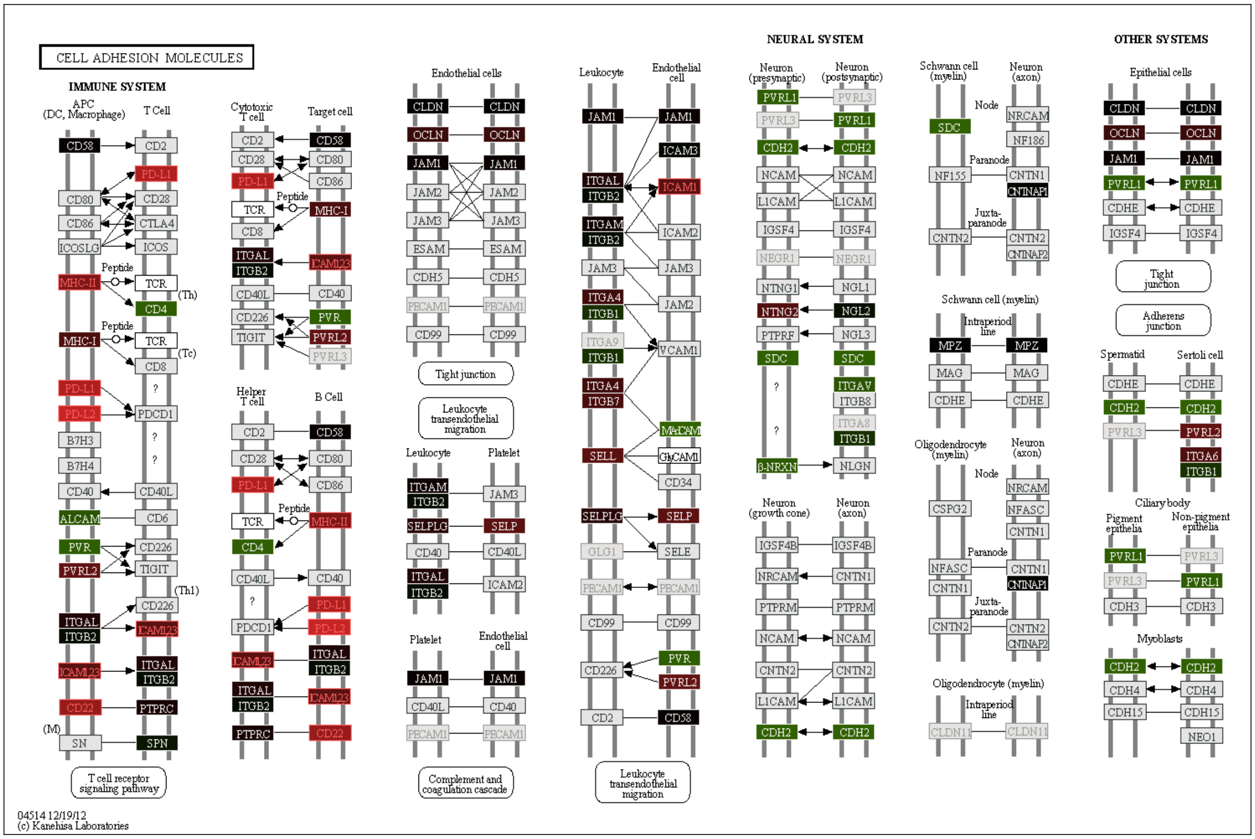

Figure A133: KEGG Pathway Map - Cell adhesion molecules (CAMs) (RNA-Seq, Neutrophils, Day 1). Node color gradient encodes fold change between vaccine groups (for multi-gene nodes the median fold change is used). In red: increased log fold change response for the SV-AS03 group relative to the SV-PBS group, in green: decreased log fold change response for the SV-AS03 group relative to the SV-AS03 group, and vice versa, in black: fold change close to 1, in dark grey: genes filtered out due to low overall expression, light grey: gene missing database mapping, white: non-human gene. Statistically significant fold changes are highlighted using red and green label colors.

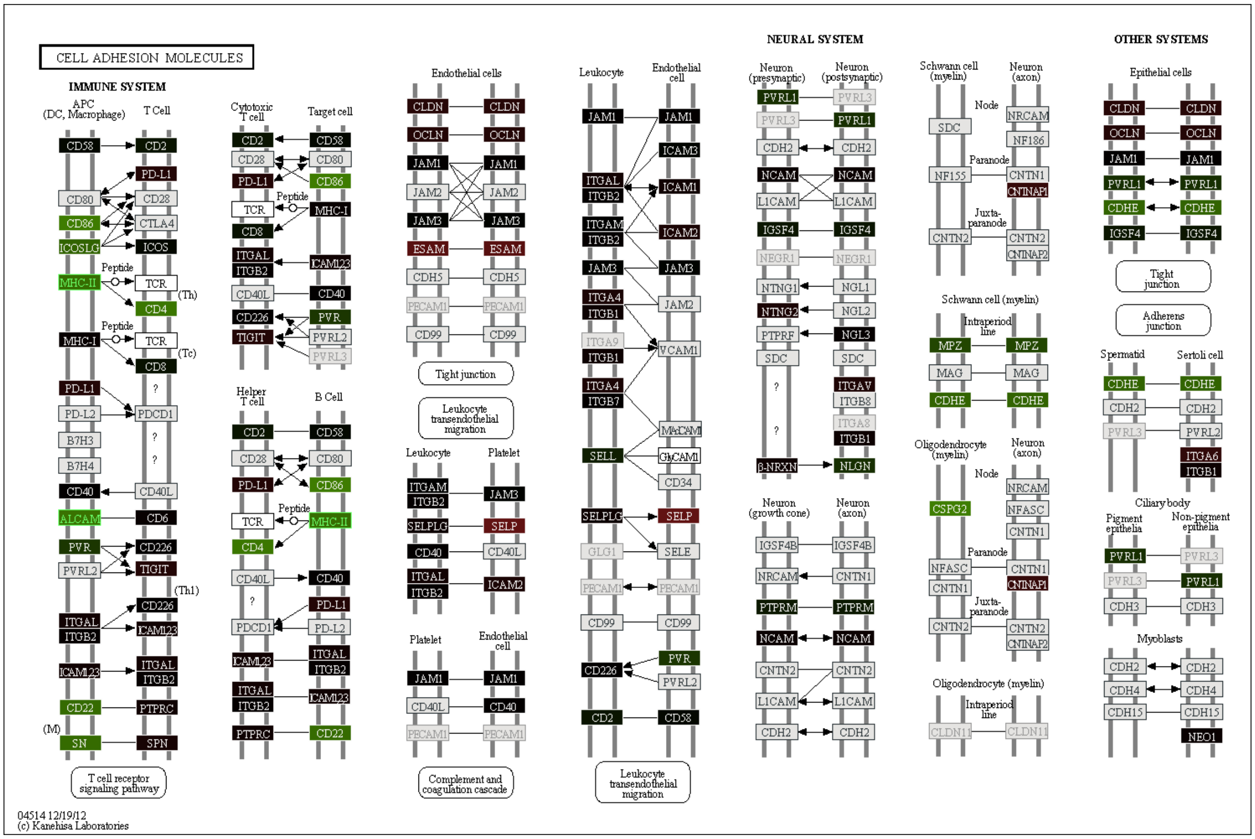

Figure A134: KEGG Pathway Map - Cell adhesion molecules (CAMs) (RNA-Seq, NK-cells, Day 28). Node color gradient encodes fold change between vaccine groups (for multi-gene nodes the median fold change is used). In red: increased log fold change response for the SV-AS03 group relative to the SV-PBS group, in green: decreased log fold change response for the SV-AS03 group relative to the SV-AS03 group, and vice versa, in black: fold change close to 1, in dark grey: genes filtered out due to low overall expression, light grey: gene missing database mapping, white: non-human gene. Statistically significant fold changes are highlighted using red and green label colors.

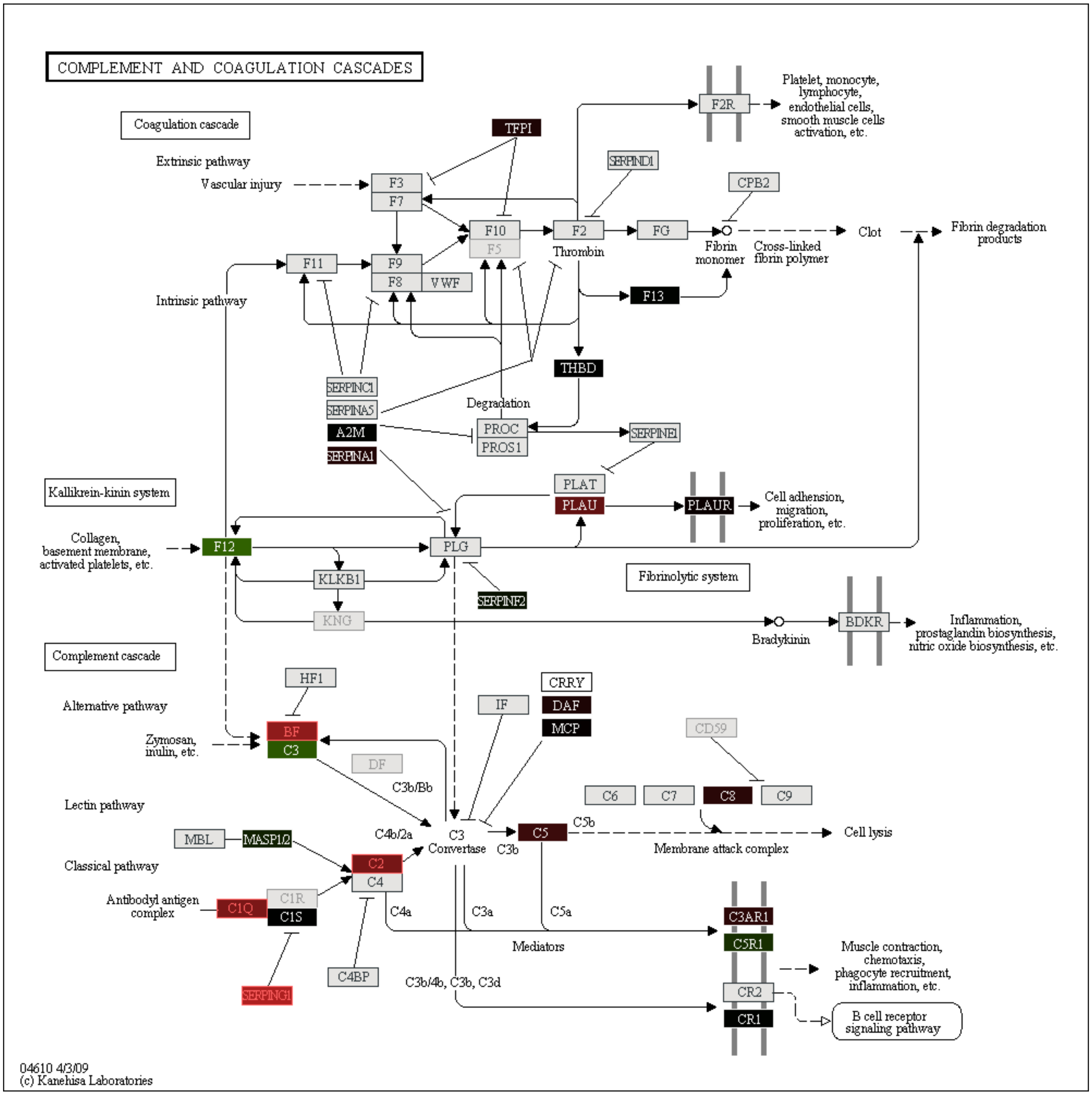

Figure A135: KEGG Pathway Map - Complement and coagulation cascades (RNA-Seq, Dendritic cells, Day 1). Node color gradient encodes fold change between vaccine groups (for multi-gene nodes the median fold change is used). In red: increased log fold change response for the SV-AS03 group relative to the SV-PBS group, in green: decreased log fold change response for the SV-AS03 group relative to the SV-AS03 group, and vice versa, in black: fold change close to 1, in dark grey: genes filtered out due to low overall expression, light grey: gene missing database mapping, white: non-human gene. Statistically significant fold changes are highlighted using red and green label colors.

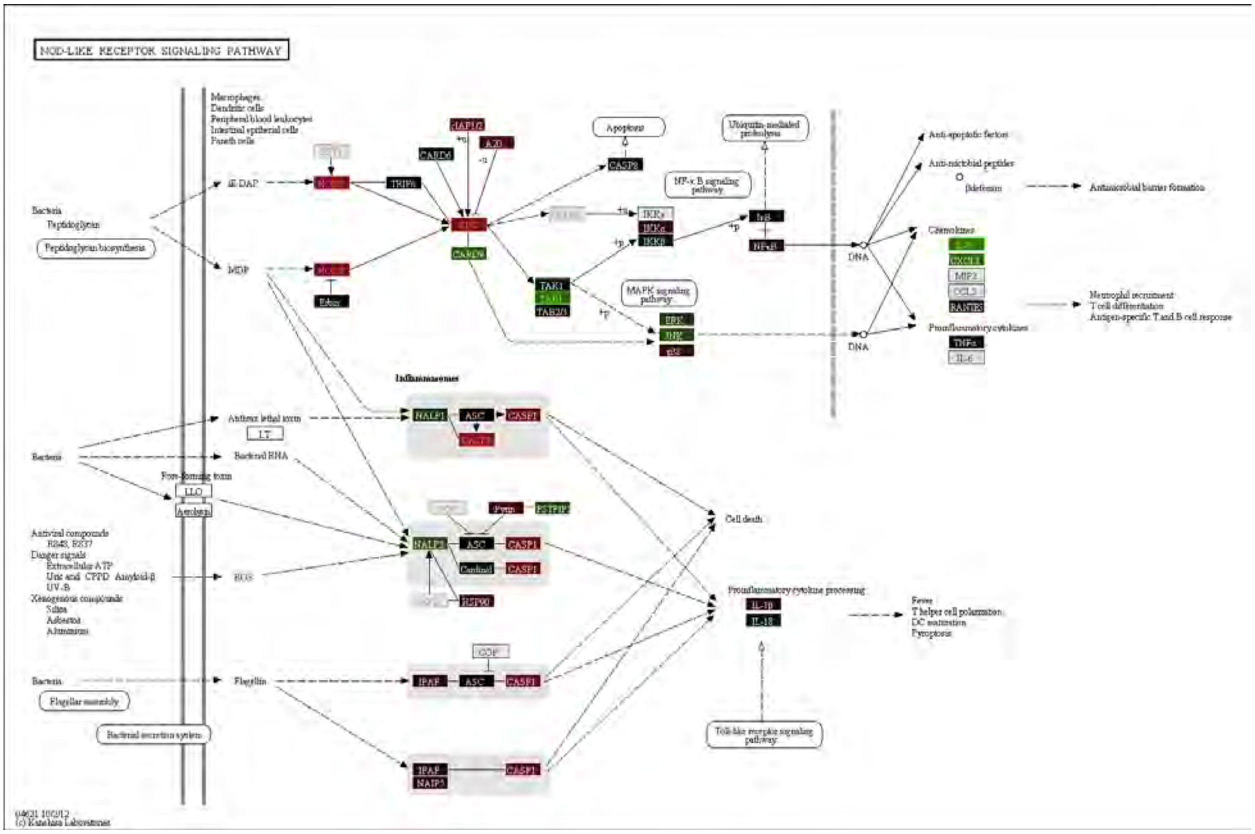

Figure A136: KEGG Pathway Map - NOD-like receptor signaling pathway (RNA-Seq, Neutrophils, Day 1). Node color gradient encodes fold change between vaccine groups (for multi-gene nodes the median fold change is used). In red: increased log fold change response for the SV-AS03 group relative to the SV-PBS group, in green: decreased log fold change response for the SV-AS03 group relative to the SV-AS03 group, and vice versa, in black: fold change close to 1, in dark grey: genes filtered out due to low overall expression, light grey: gene missing database mapping, white: non-human gene. Statistically significant fold changes are highlighted using red and green label colors.

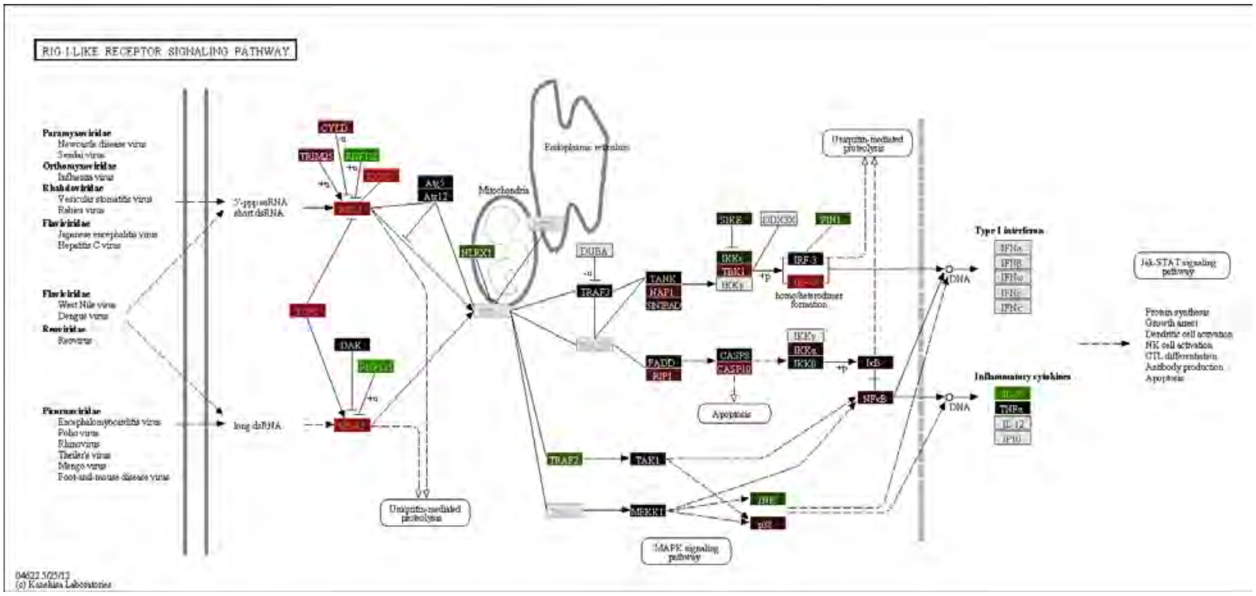

Figure A137: KEGG Pathway Map - RIG-I-like receptor signaling pathway (RNA-Seq, Neutrophils, Day 1). Node color gradient encodes fold change between vaccine groups (for multi-gene nodes the median fold change is used). In red: increased log fold change response for the SV-AS03 group relative to the SV-PBS group, in green: decreased log fold change response for the SV-AS03 group relative to the SV-AS03 group, and vice versa, in black: fold change close to 1, in dark grey: genes filtered out due to low overall expression, light grey: gene missing database mapping, white: non-human gene. Statistically significant fold changes are highlighted using red and green label colors.

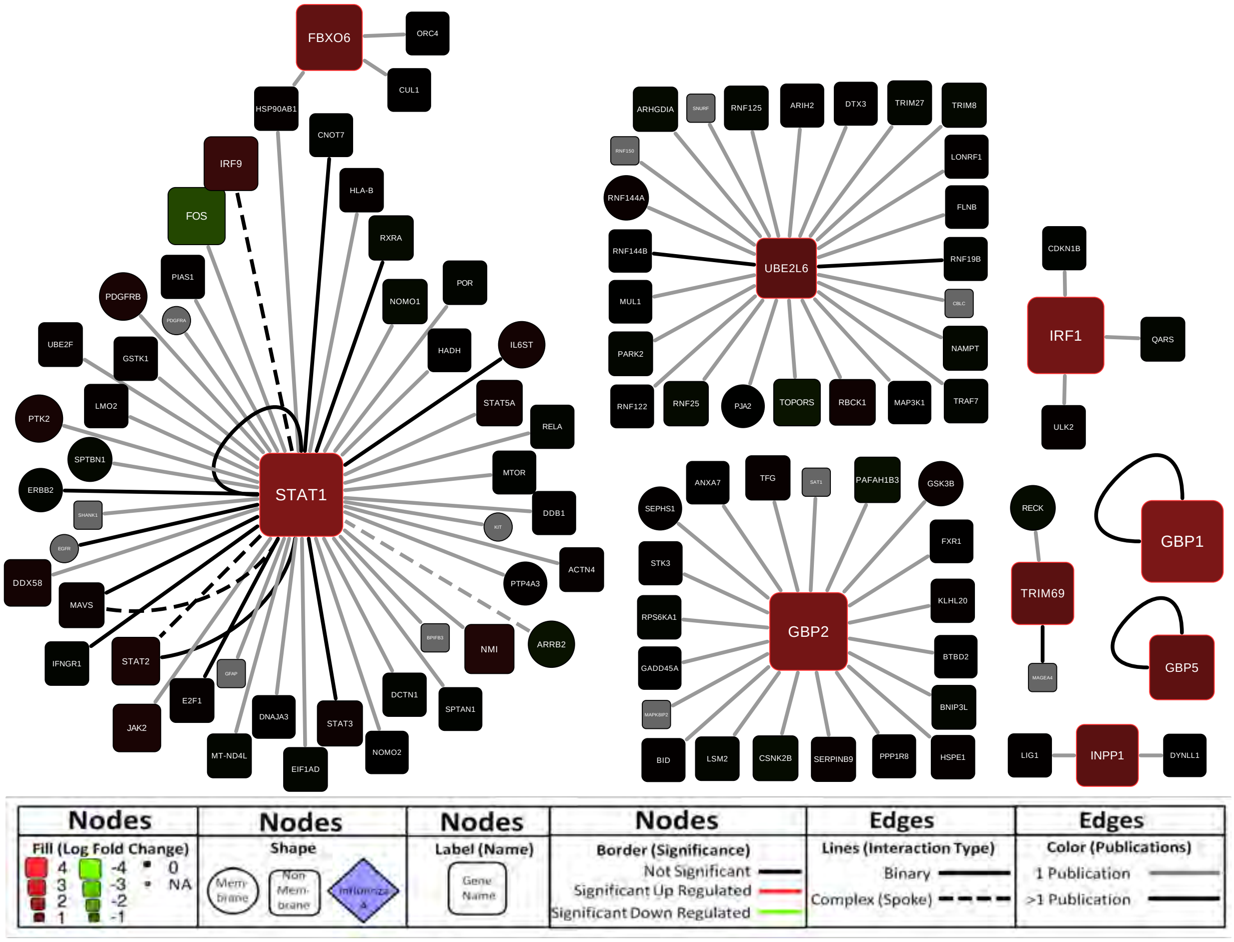

Figure A138: Integrated protein-protein interaction network based on significant genes (B-cells, Day 1). Human-human and human-*Influenza A* experimental interactions for which one interacting partner is encoded by a significant gene.

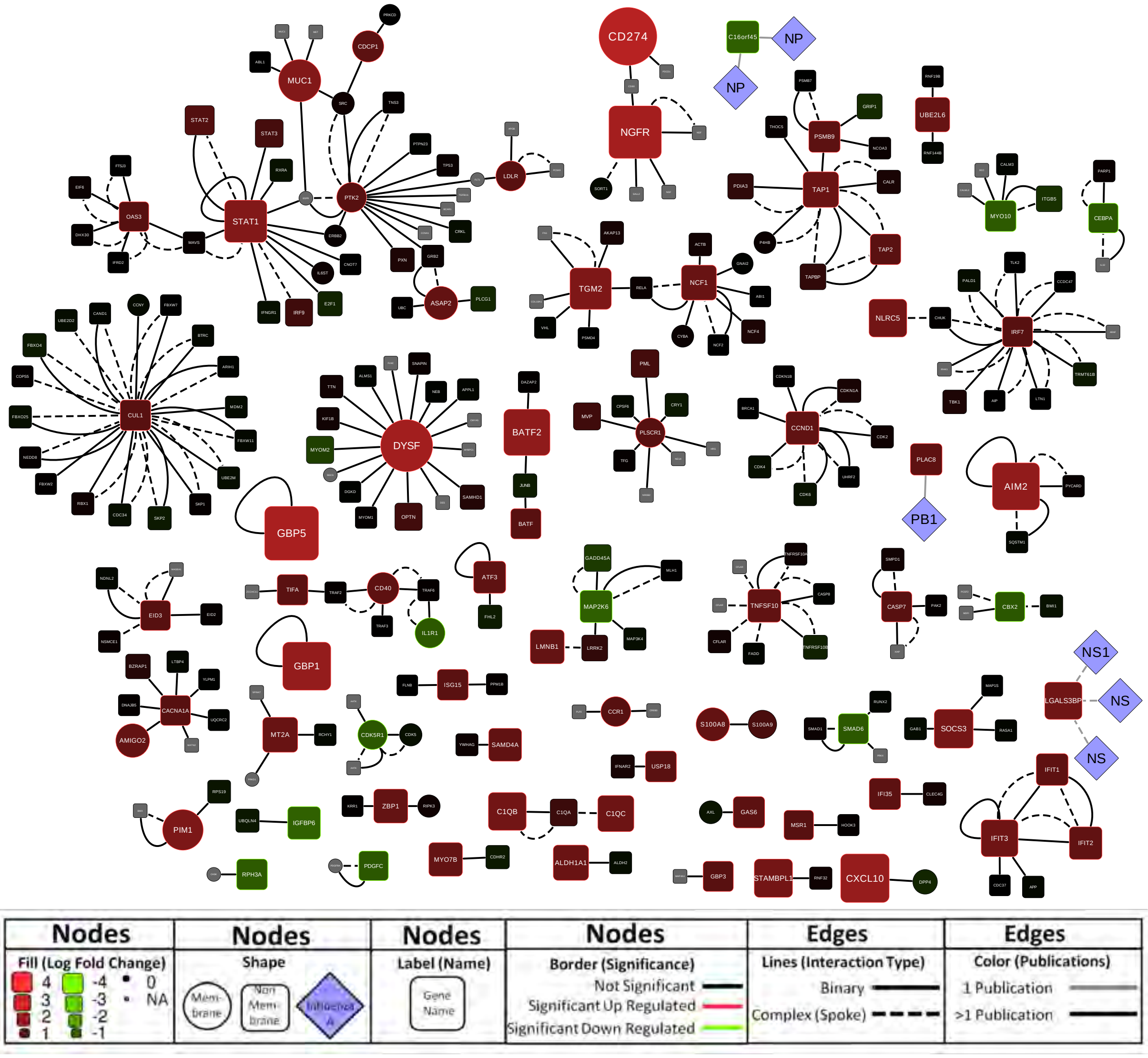

Figure A139: Integrated protein-protein interaction network based on significant genes (Dendritic cells, Day 1). Human-human and human-*Influenza A* experimental interactions for which one interacting partner is encoded by a significant gene.

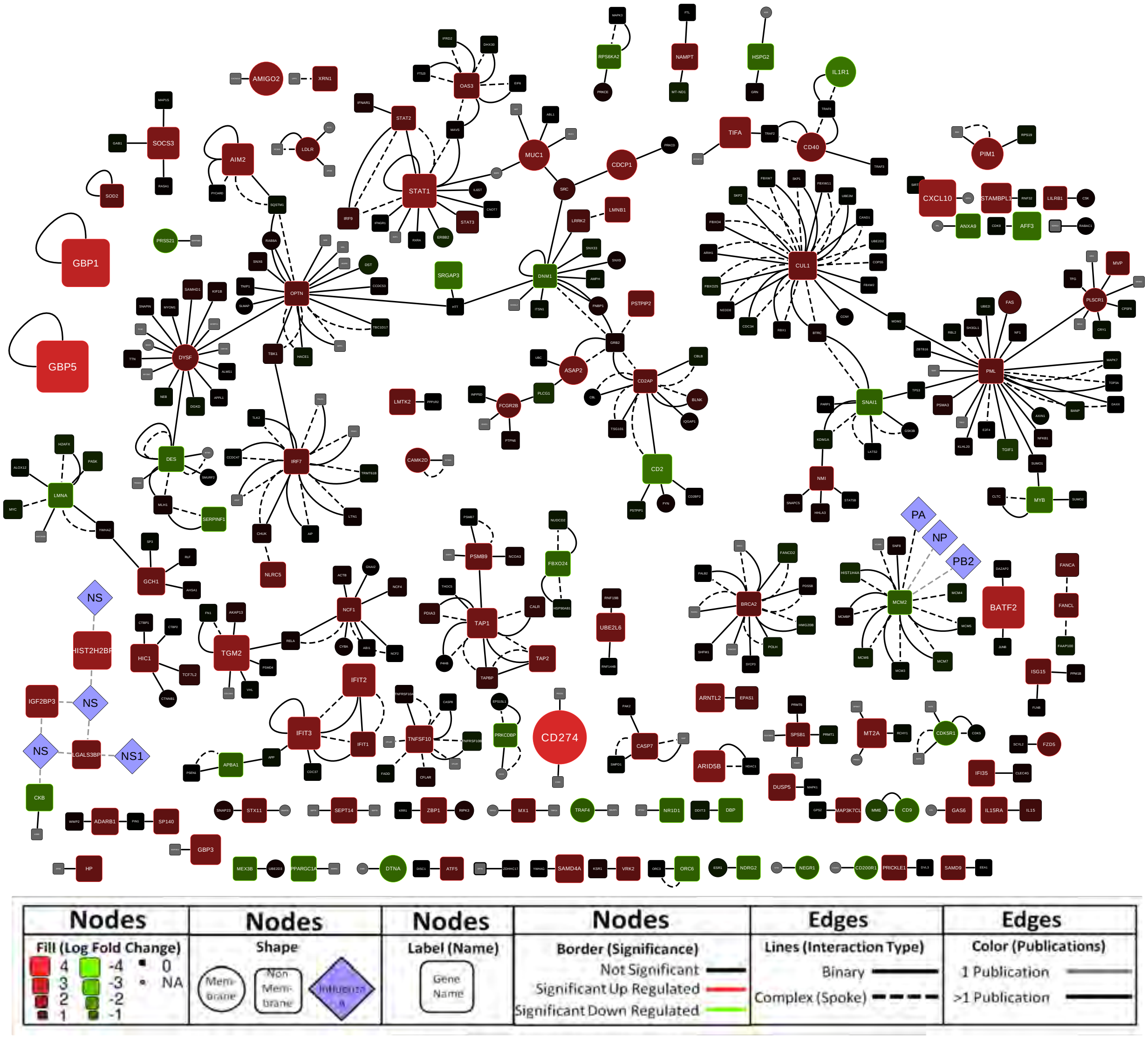

Figure A140: Integrated protein-protein interaction network based on significant genes (Monocytes Day 1). Human-human and human-*Influenza A* experimental interactions for which one interacting partner is encoded by a significant gene (human-human protein interactions are only shown if supported by at least two publications).

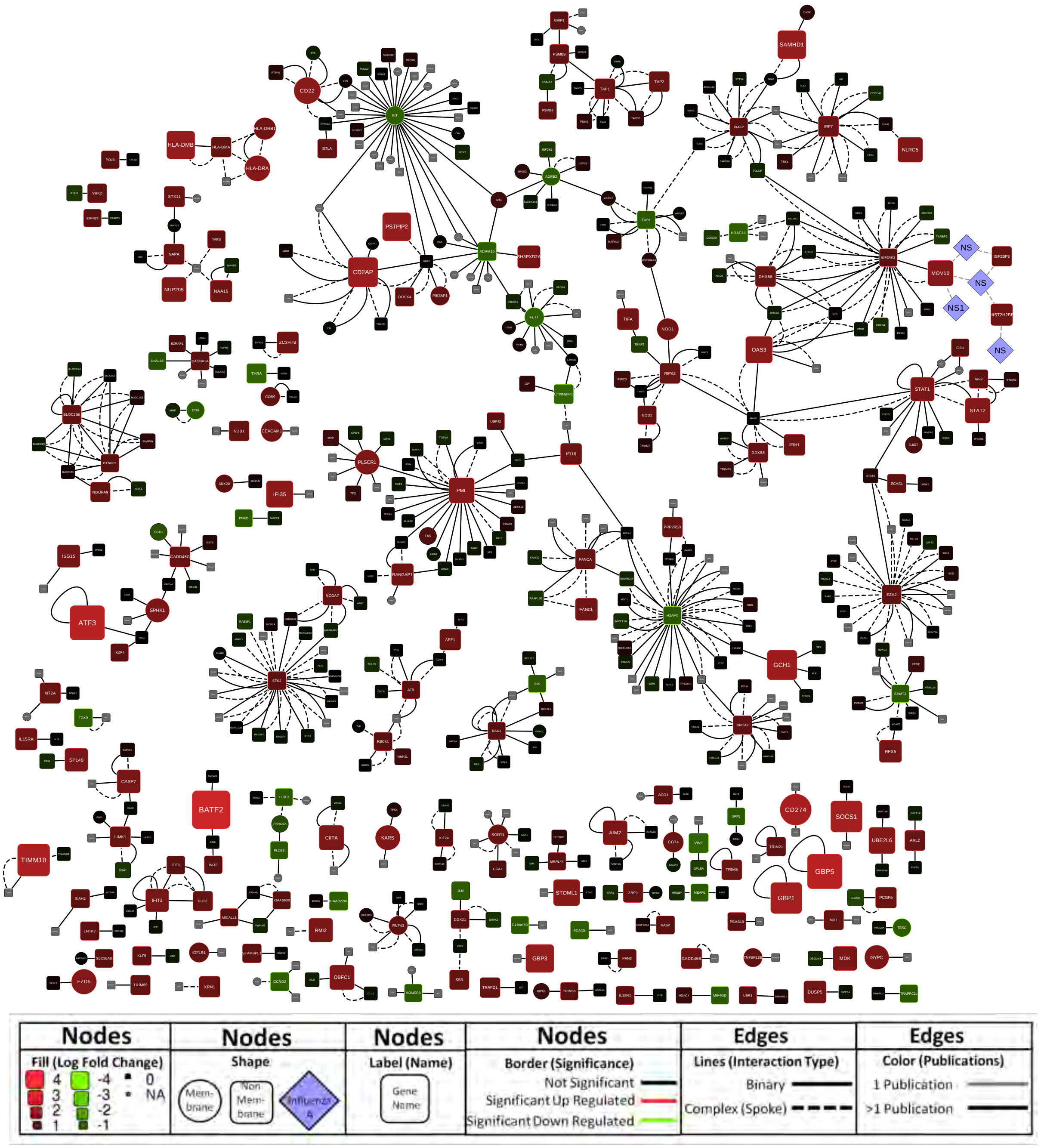

Figure A141: Integrated protein-protein interaction network based on significant genes (Neutrophils Day 1). Human-human and human-*Influenza A* experimental interactions for which one interacting partner is encoded by a significant gene (human-human protein interactions are only shown if supported by at least two publications).

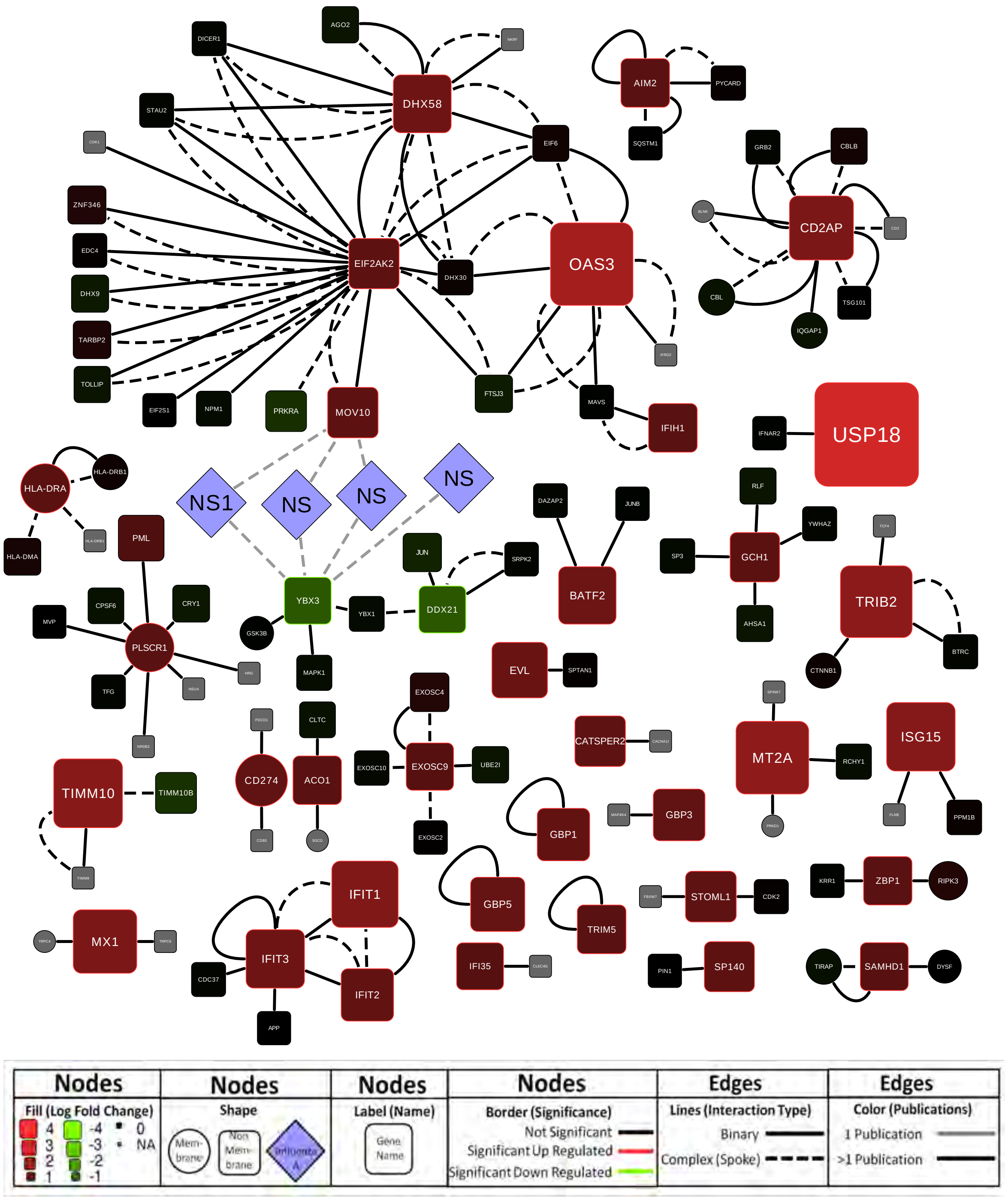

Figure A142: Integrated protein-protein interaction network based on significant genes (Neutrophils Day 1). Human-human and human-*Influenza A* experimental interactions for which one interacting partner is encoded by a significant gene (human-human protein interactions are only shown if supported by at least two publications).

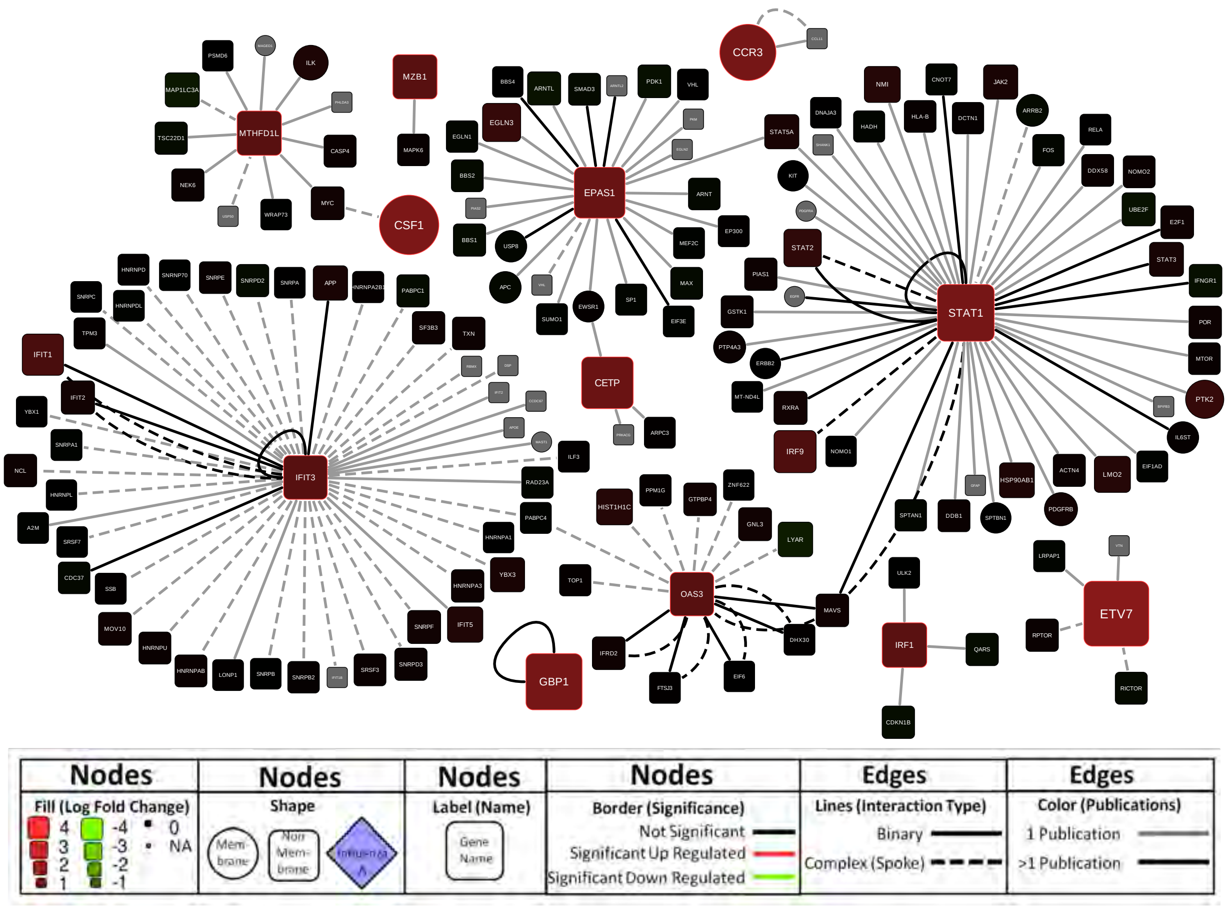

Figure A143: Integrated protein-protein interaction network based on significant genes (NK-cells Day 1). Human-human and human-*Influenza A* experimental interactions for which one interacting partner is encoded by a significant gene.

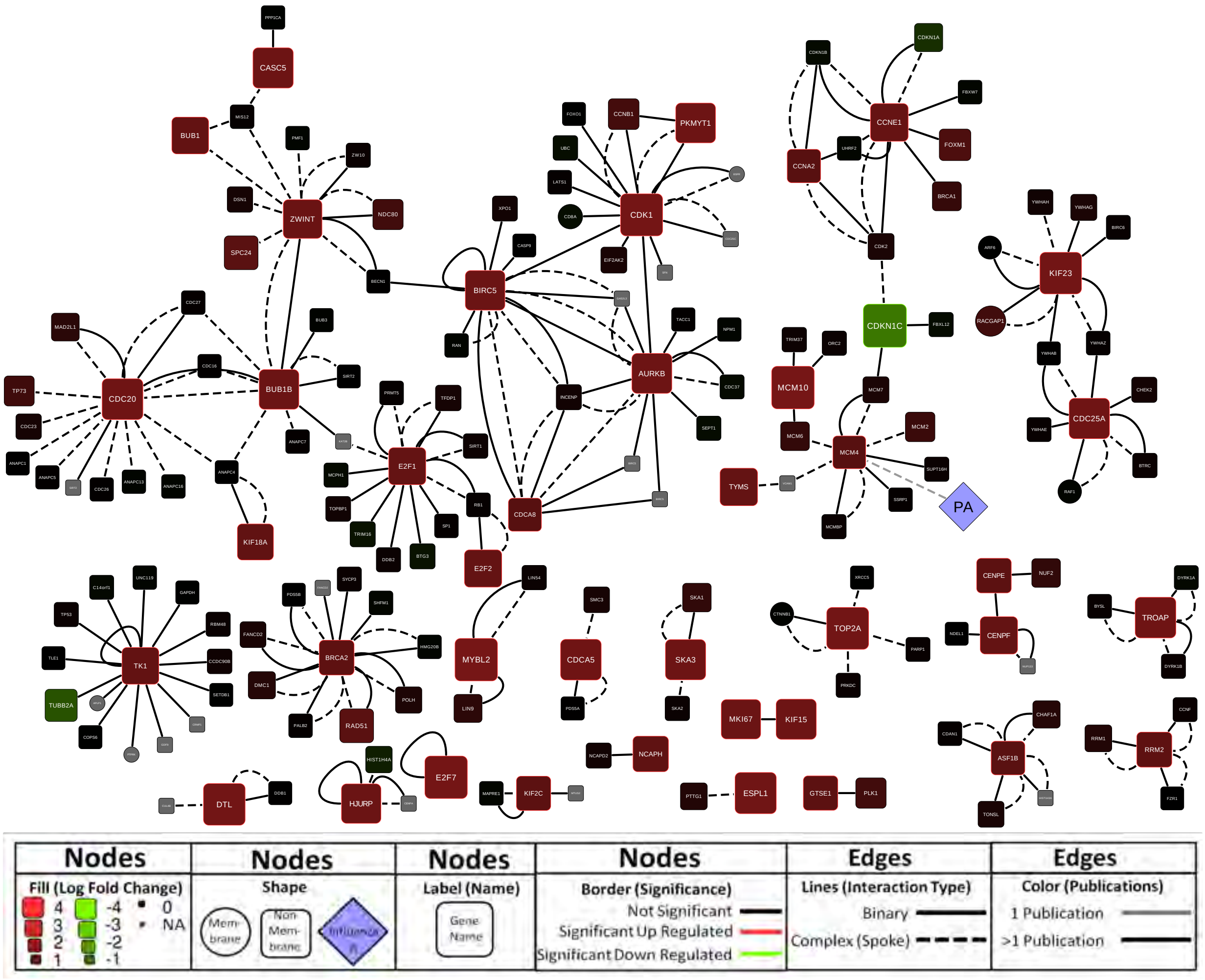

Figure A144: Integrated protein-protein interaction network based on significant genes (NK-cells Day 3). Human-human and human-*Influenza A* experimental interactions for which one interacting partner is encoded by a significant gene (human-human protein interactions are only shown if supported by at least two publications).

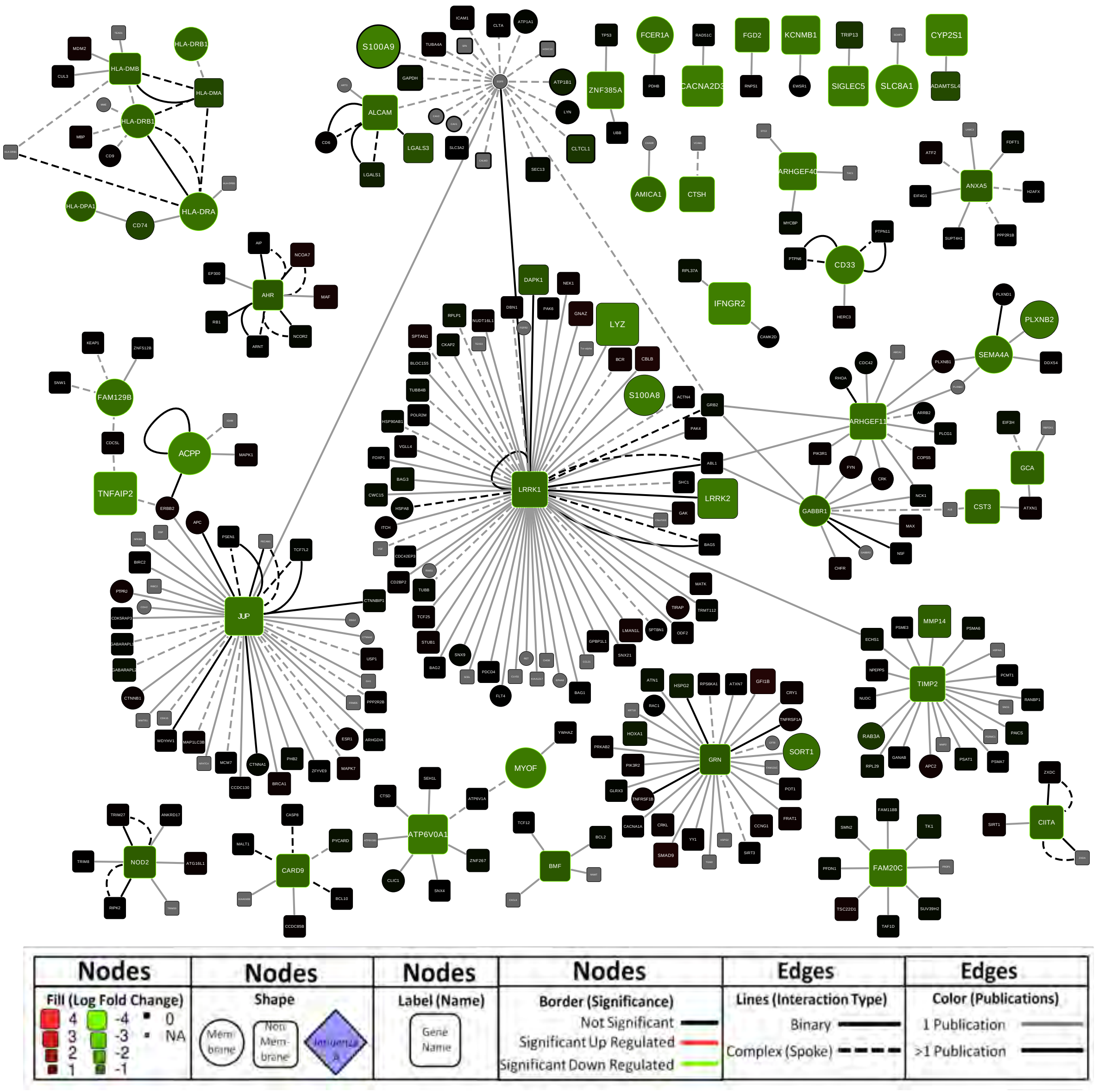

Figure A145: Integrated protein-protein interaction network based on significant genes (NK-cells Day 28). Human-human and human-*Influenza A* experimental interactions for which one interacting partner is encoded by a significant gene.

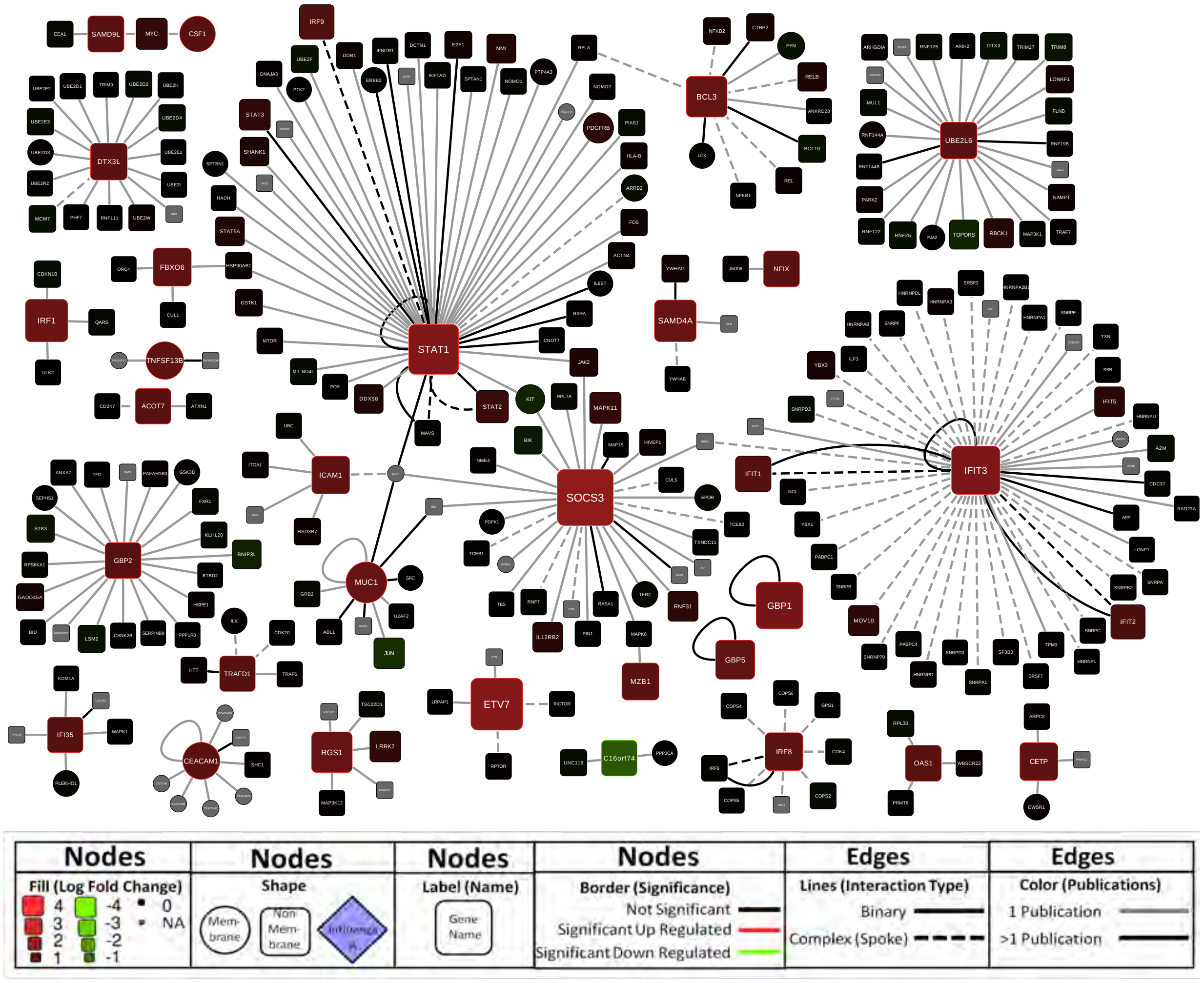

Figure A146: Integrated protein-protein interaction network based on significant genes (T-cells Day 1). Human-human and human-*Influenza A* experimental interactions for which one interacting partner is encoded by a significant gene.

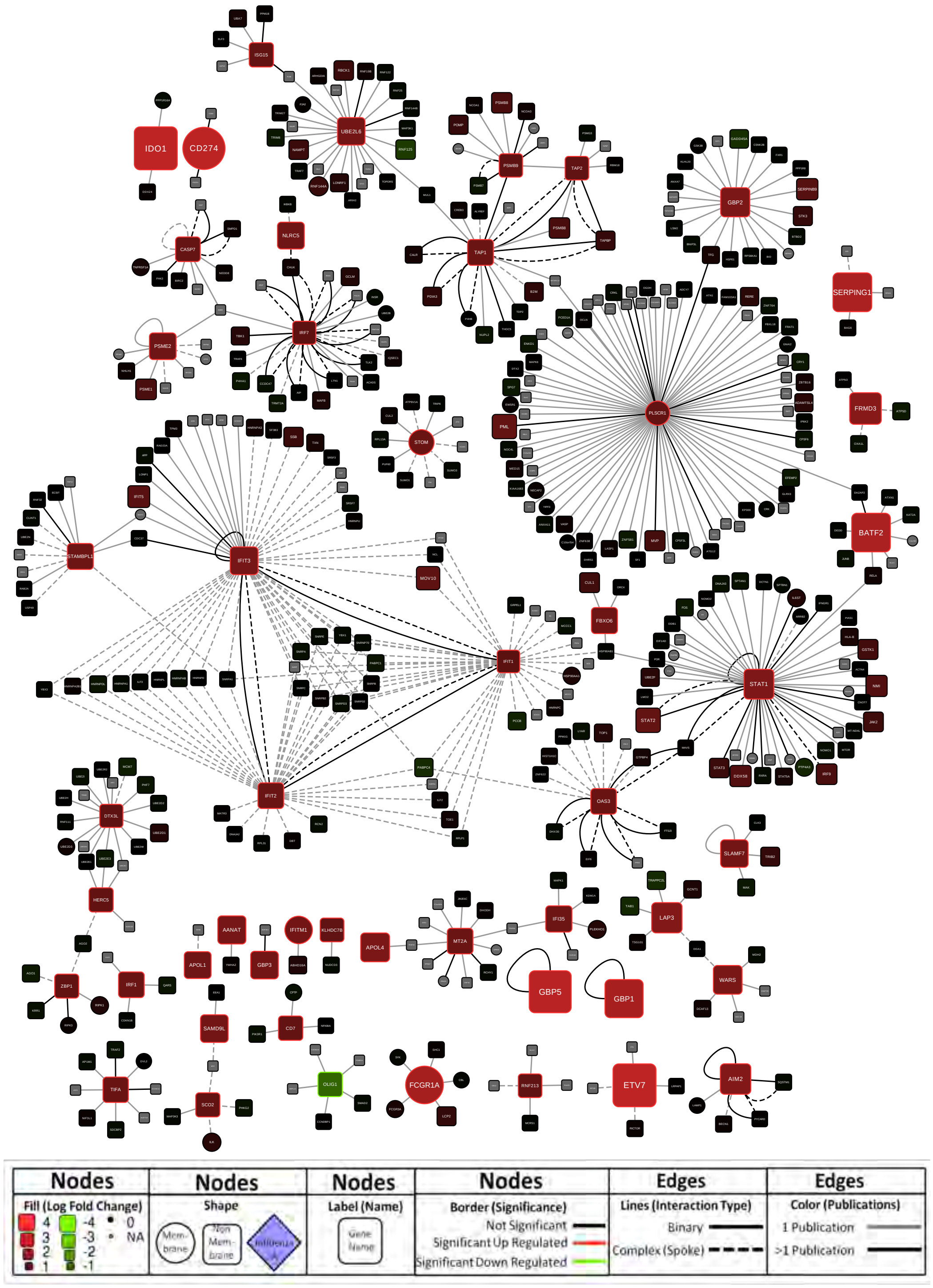

Figure A147: Integrated protein-protein interaction network based on overlapping significant genes at Day 1 (Dendritic cells, Monocytes, and Neutrophils). Human-human and human-*Influenza A* experimental interactions for which one interacting partner is encoded by a significant gene (human-human protein interactions are only shown if supported by at least two publications).

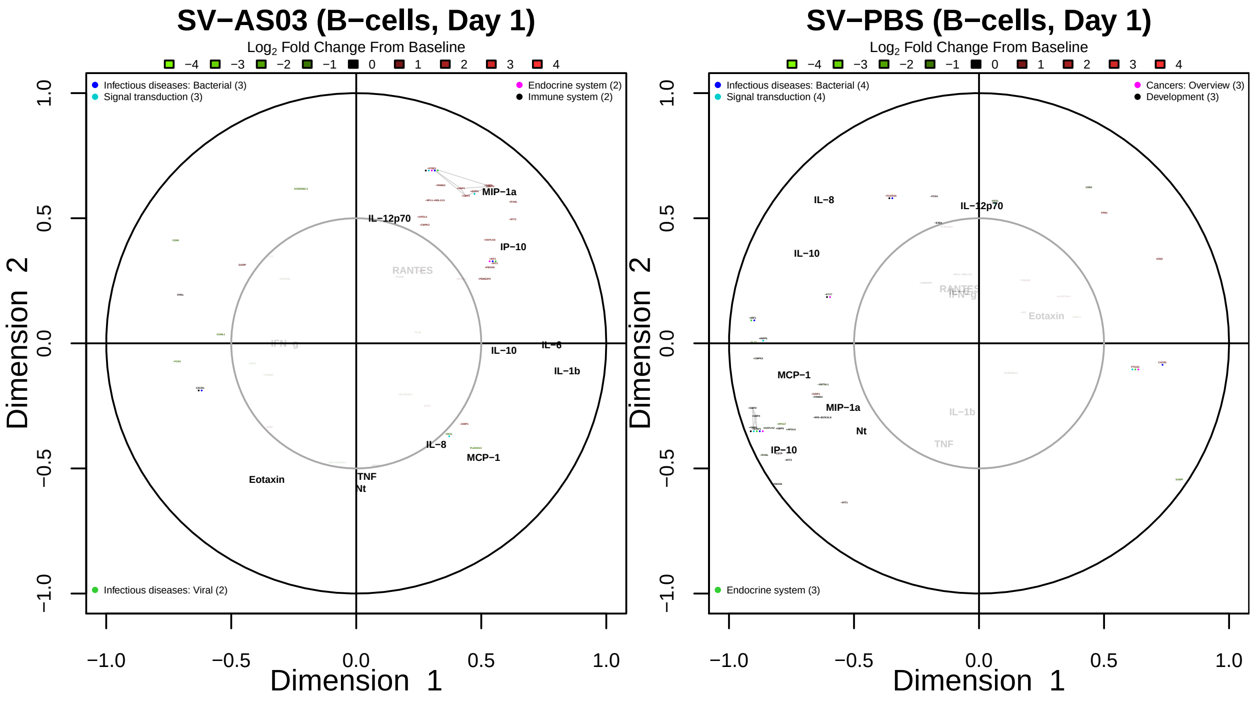

Figure A148: Canonical correlation plots (RNA-Seq, B-cells, Day 1). In black: IMO variables, green-red: gene variables color-coded by baseline fold change, +/- prefix: SDEG yes/no, colored-dots: pathway class, grey lines: robust clusters.

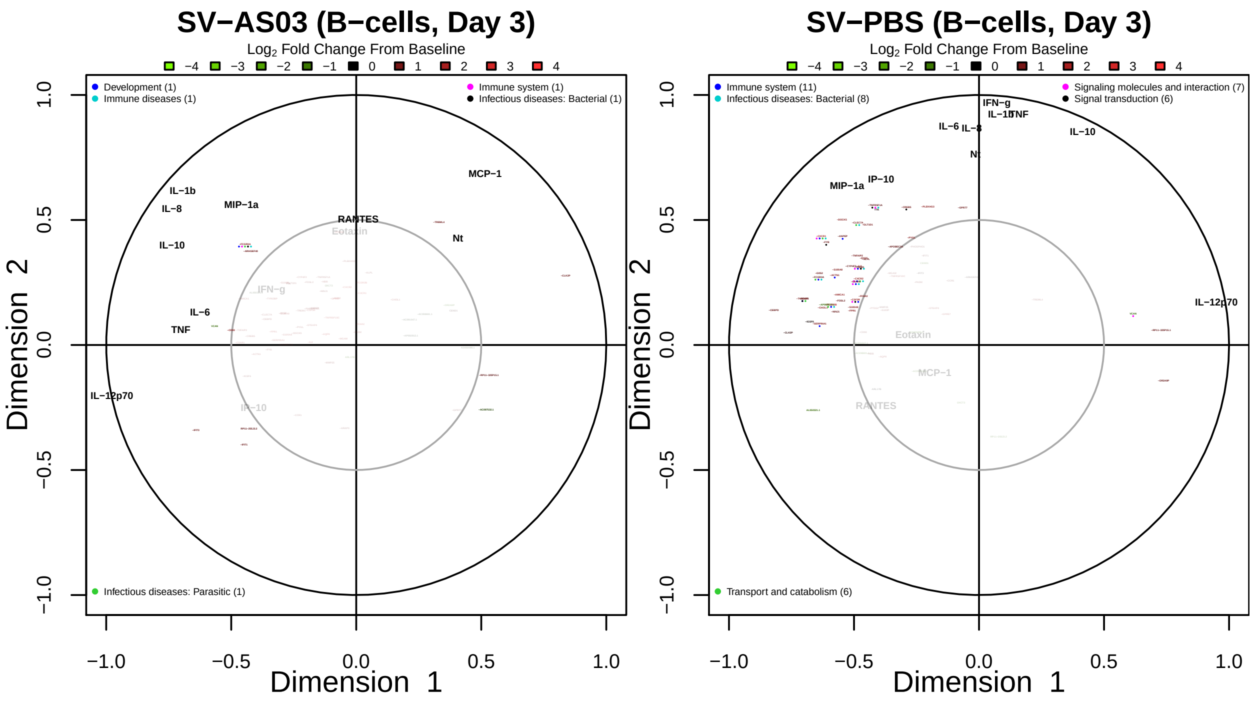

Figure A149: Canonical correlation plots (RNA-Seq, B-cells, Day 3). In black: IMO variables, green-red: gene variables color-coded by baseline fold change, +/- prefix: SDEG yes/no, colored-dots: pathway class, grey lines: robust clusters.

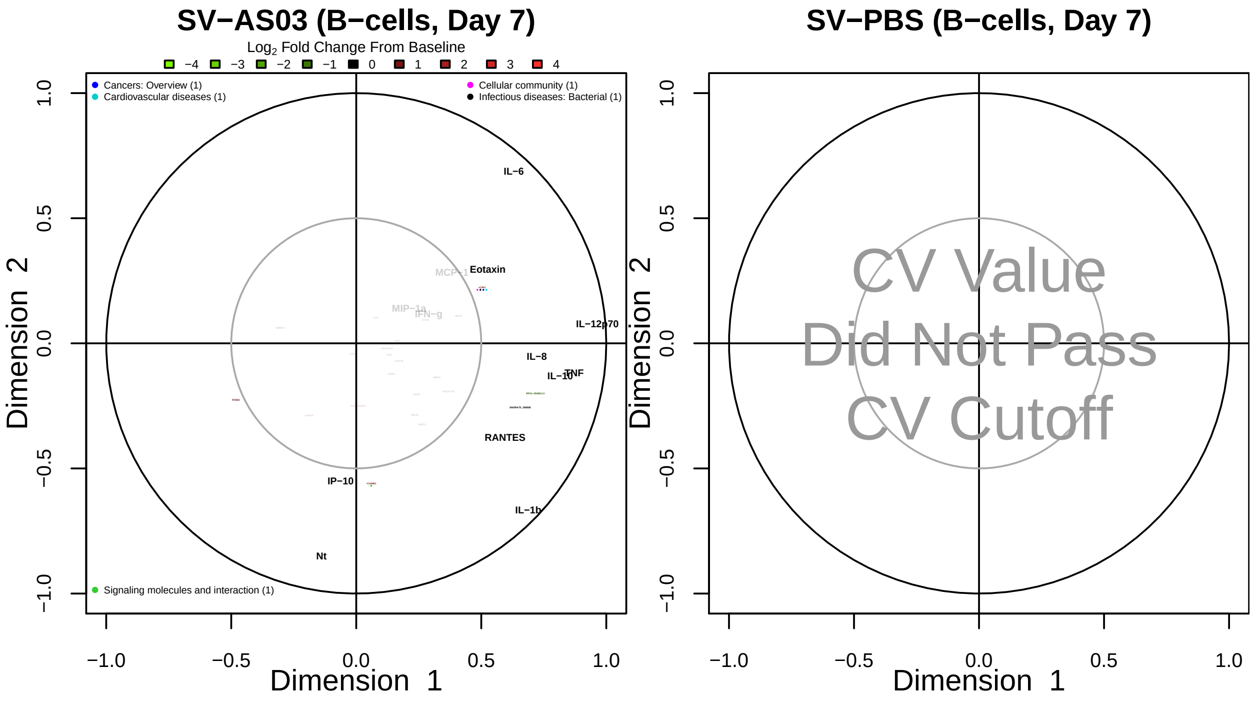

Figure A150: Canonical correlation plots (RNA-Seq, B-cells, Day 7). In black: IMO variables, green-red: gene variables color-coded by baseline fold change, +/- prefix: SDEG yes/no, colored-dots: pathway class, grey lines: robust clusters.

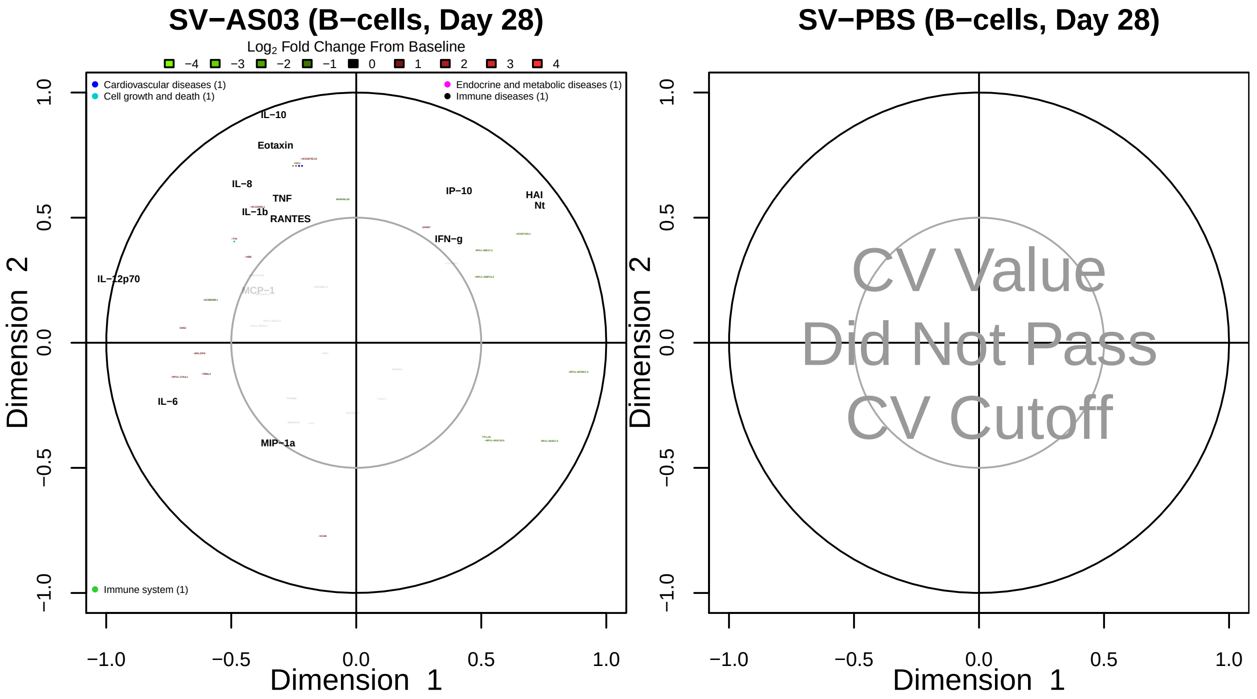

Figure A151: Canonical correlation plots (RNA-Seq, B-cells, Day 28). In black: IMO variables, green-red: gene variables color-coded by baseline fold change, +/- prefix: SDEG yes/no, colored-dots: pathway class, grey lines: robust clusters.

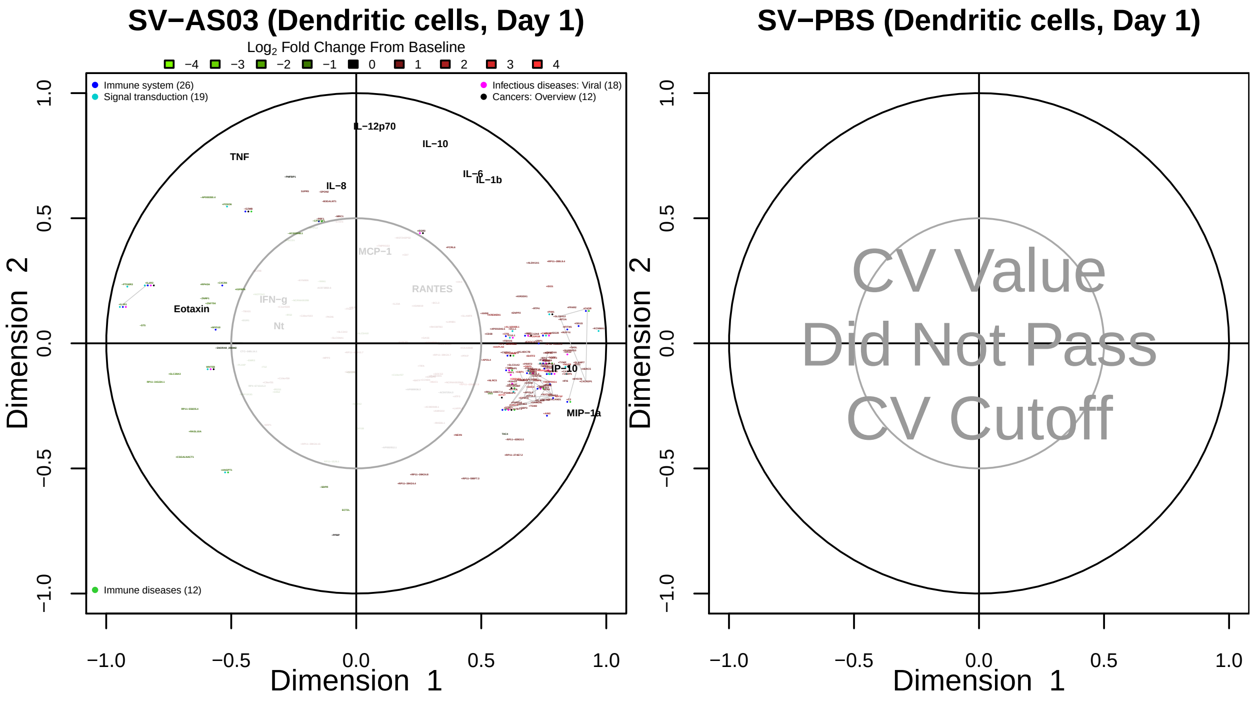

Figure A152: Canonical correlation plots (RNA-Seq, Dendritic cells, Day 1). In black: IMO variables, green-red: gene variables color-coded by baseline fold change, +/- prefix: SDEG yes/no, colored-dots: pathway class, grey lines: robust clusters.

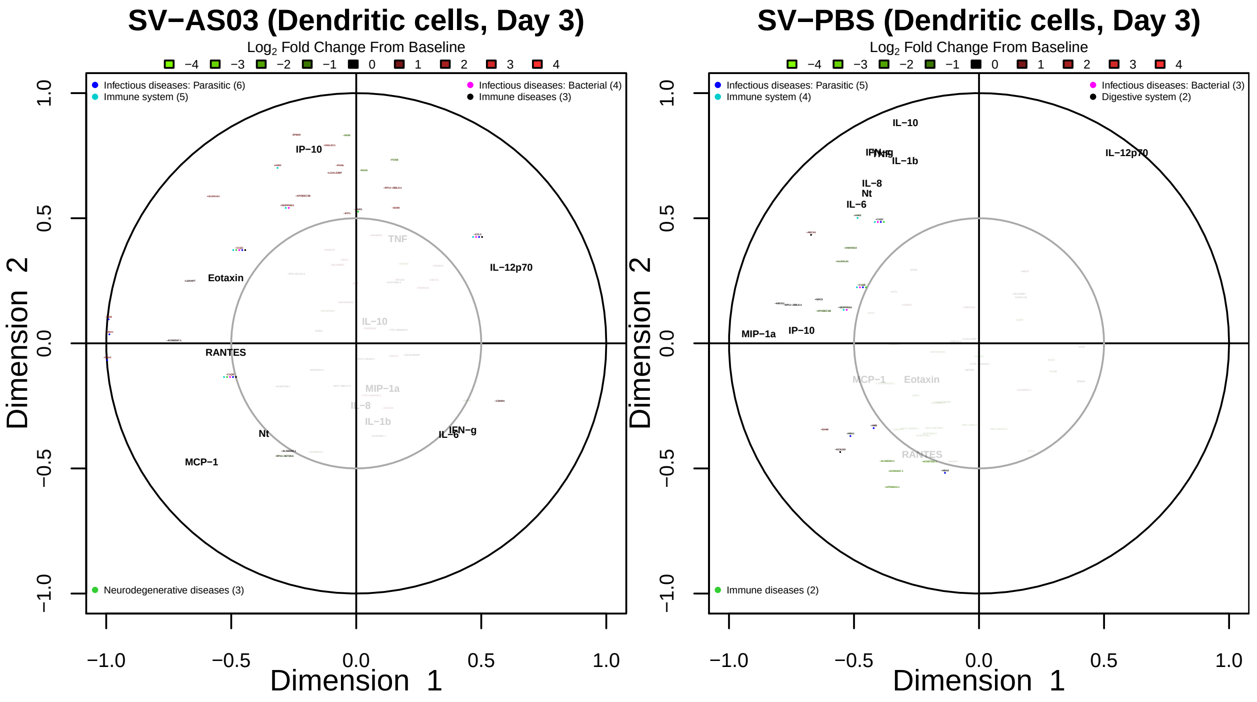

Figure A153: Canonical correlation plots (RNA-Seq, Dendritic cells, Day 3). In black: IMO variables, green-red: gene variables color-coded by baseline fold change, +/- prefix: SDEG yes/no, colored-dots: pathway class, grey lines: robust clusters.

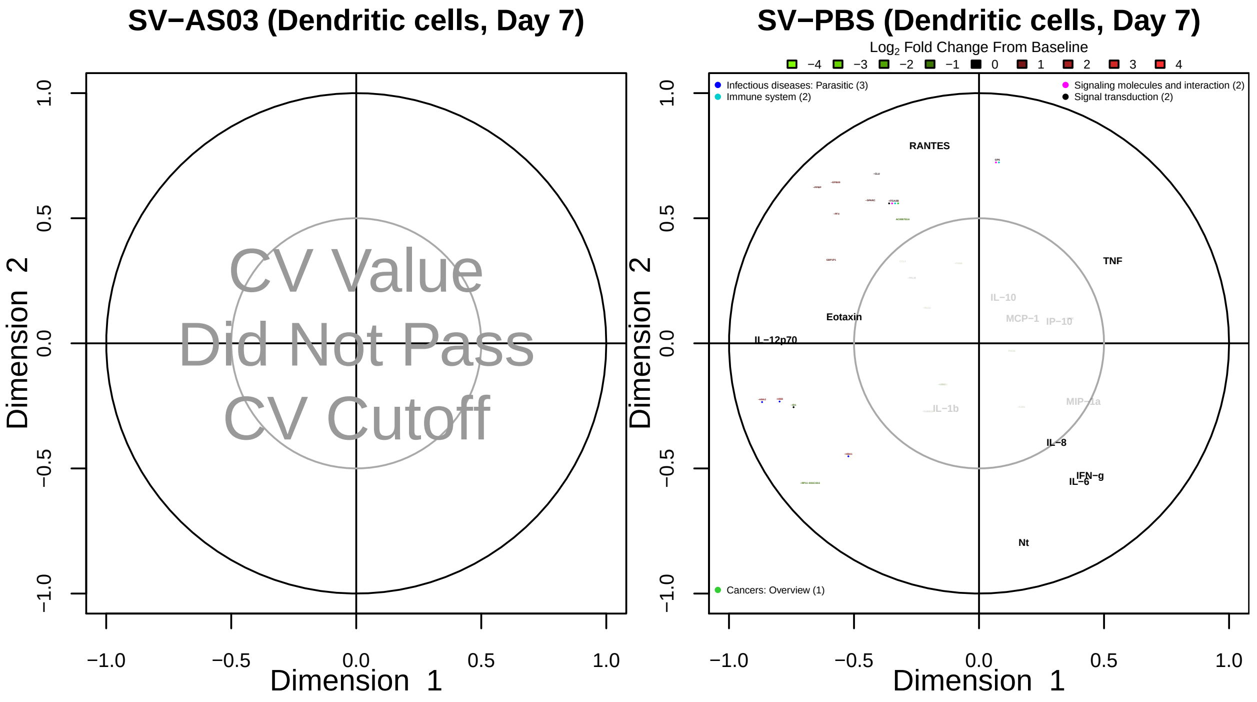

Figure A154: Canonical correlation plots (RNA-Seq, Dendritic cells, Day 7). In black: IMO variables, green-red: gene variables color-coded by baseline fold change, +/- prefix: SDEG yes/no, colored-dots: pathway class, grey lines: robust clusters.

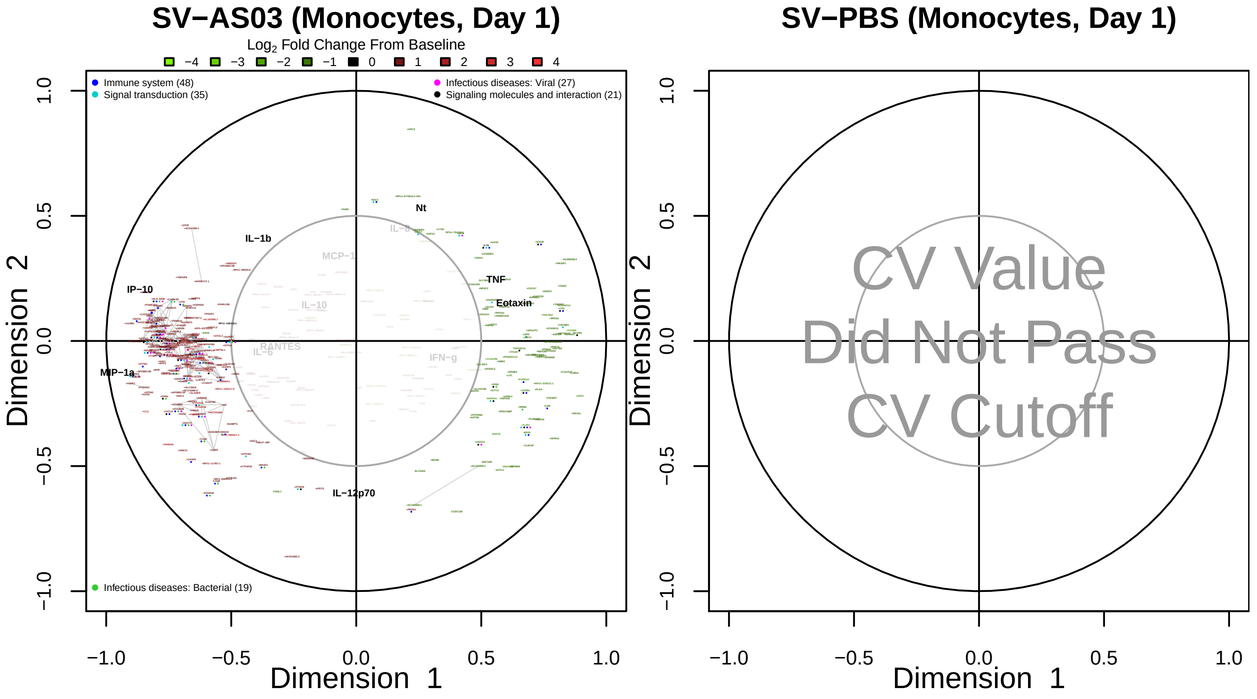

Figure A155: Canonical correlation plots (RNA-Seq, Monocytes, Day 1). In black: IMO variables, green-red: gene variables color-coded by baseline fold change, +/- prefix: SDEG yes/no, colored-dots: pathway class, grey lines: robust clusters.

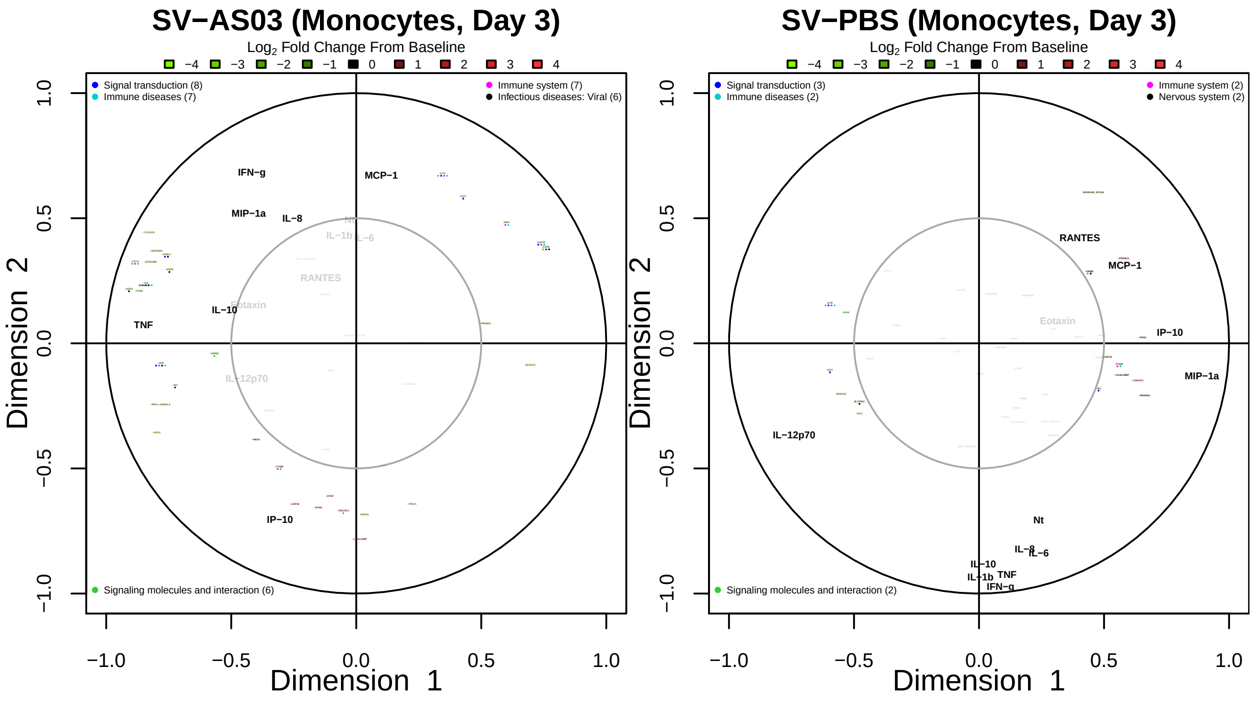

Figure A156: Canonical correlation plots (RNA-Seq, Monocytes, Day 3). In black: IMO variables, green-red: gene variables color-coded by baseline fold change, +/- prefix: SDEG yes/no, colored-dots: pathway class, grey lines: robust clusters.

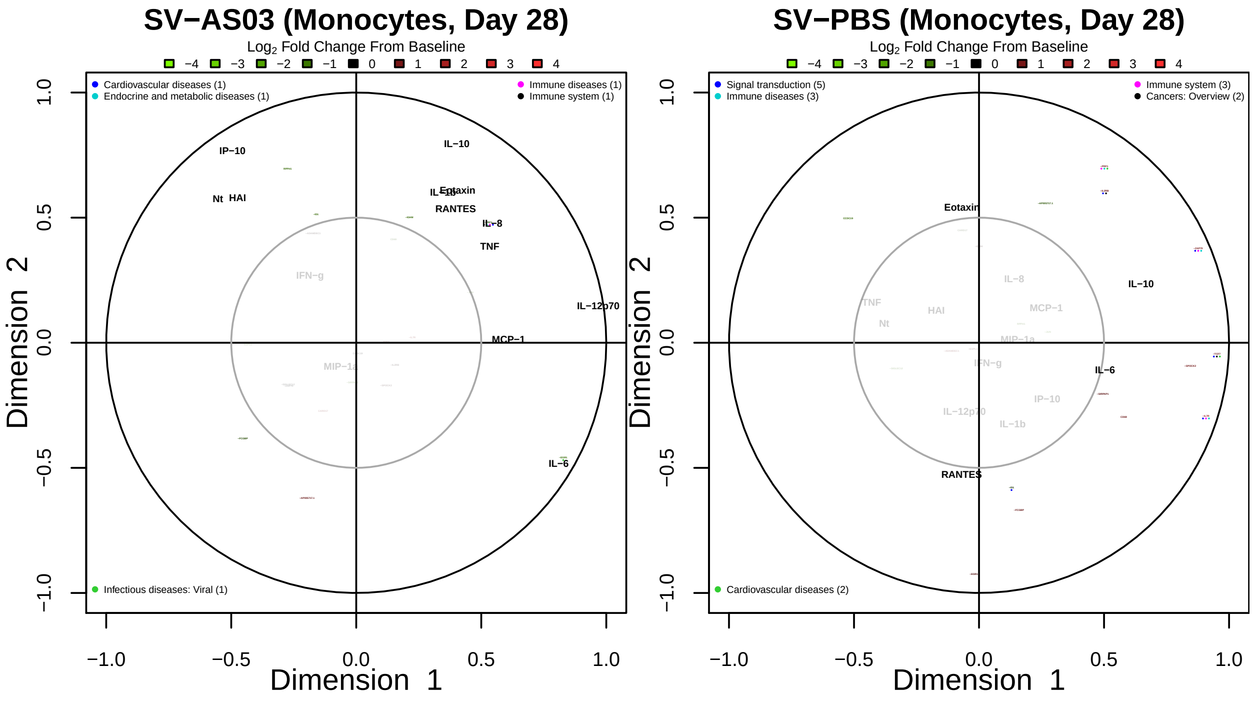

Figure A157: Canonical correlation plots (RNA-Seq, Monocytes, Day 28). In black: IMO variables, green-red: gene variables color-coded by baseline fold change, +/- prefix: SDEG yes/no, colored-dots: pathway class, grey lines: robust clusters.

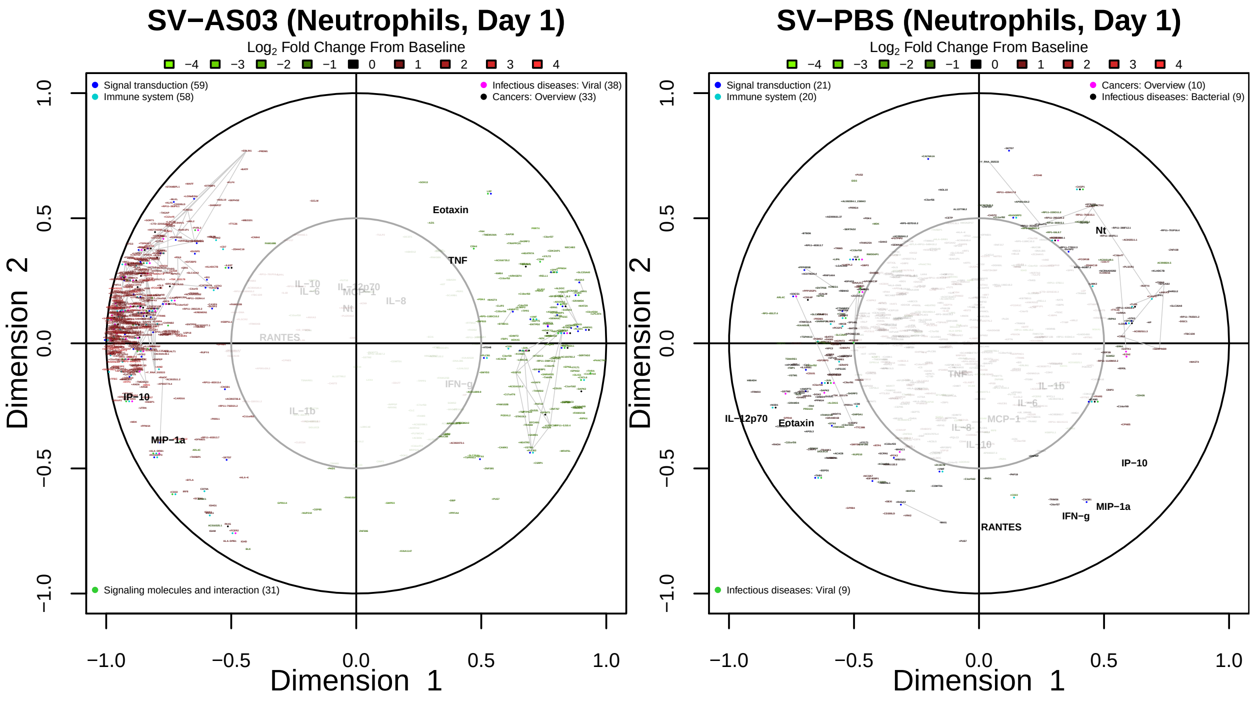

Figure A158: Canonical correlation plots (RNA-Seq, Neutrophils, Day 1). In black: IMO variables, green-red: gene variables color-coded by baseline fold change, +/- prefix: SDEG yes/no, colored-dots: pathway class, grey lines: robust clusters.

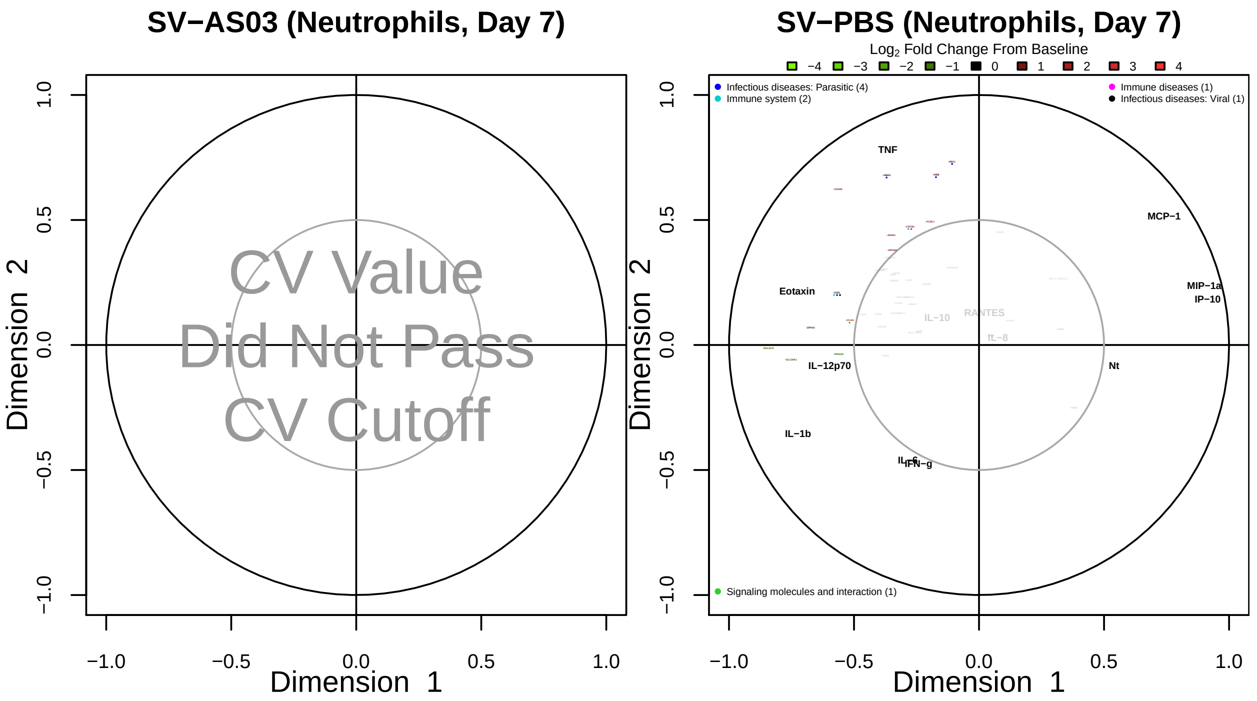

Figure A159: Canonical correlation plots (RNA-Seq, Neutrophils, Day 7). In black: IMO variables, green-red: gene variables color-coded by baseline fold change, +/- prefix: SDEG yes/no, colored-dots: pathway class, grey lines: robust clusters.

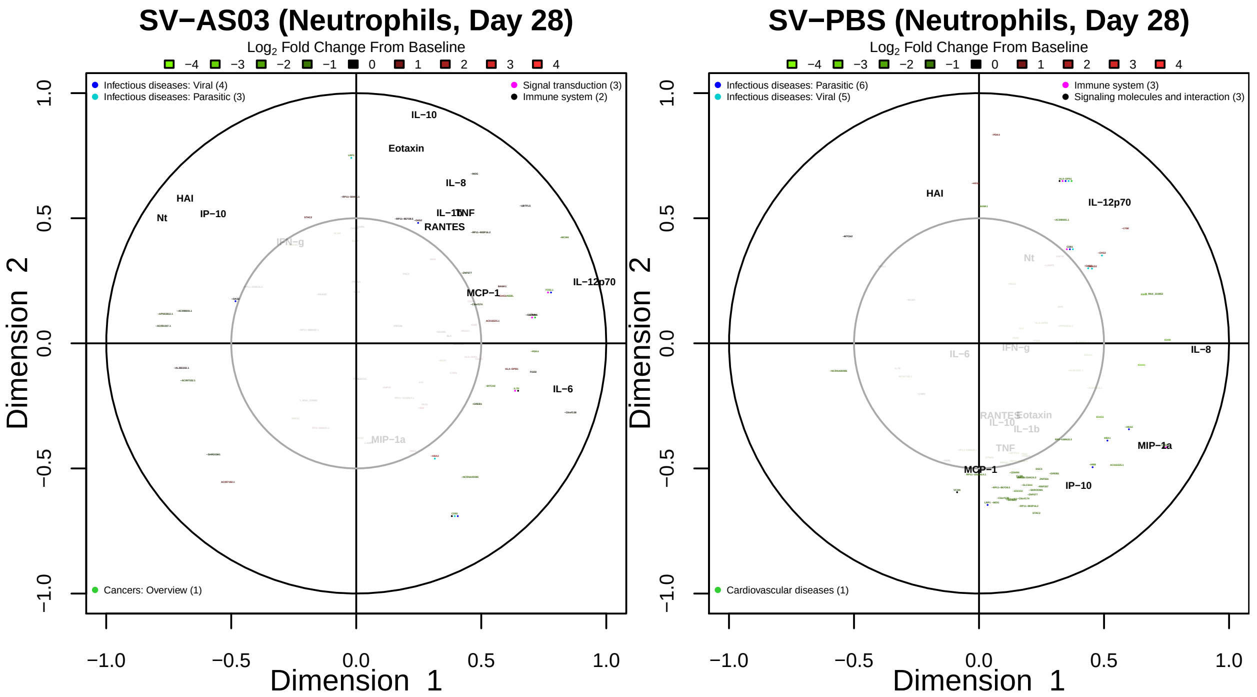

Figure A160: Canonical correlation plots (RNA-Seq, Neutrophils, Day 28). In black: IMO variables, green-red: gene variables color-coded by baseline fold change, +/- prefix: SDEG yes/no, colored-dots: pathway class, grey lines: robust clusters.

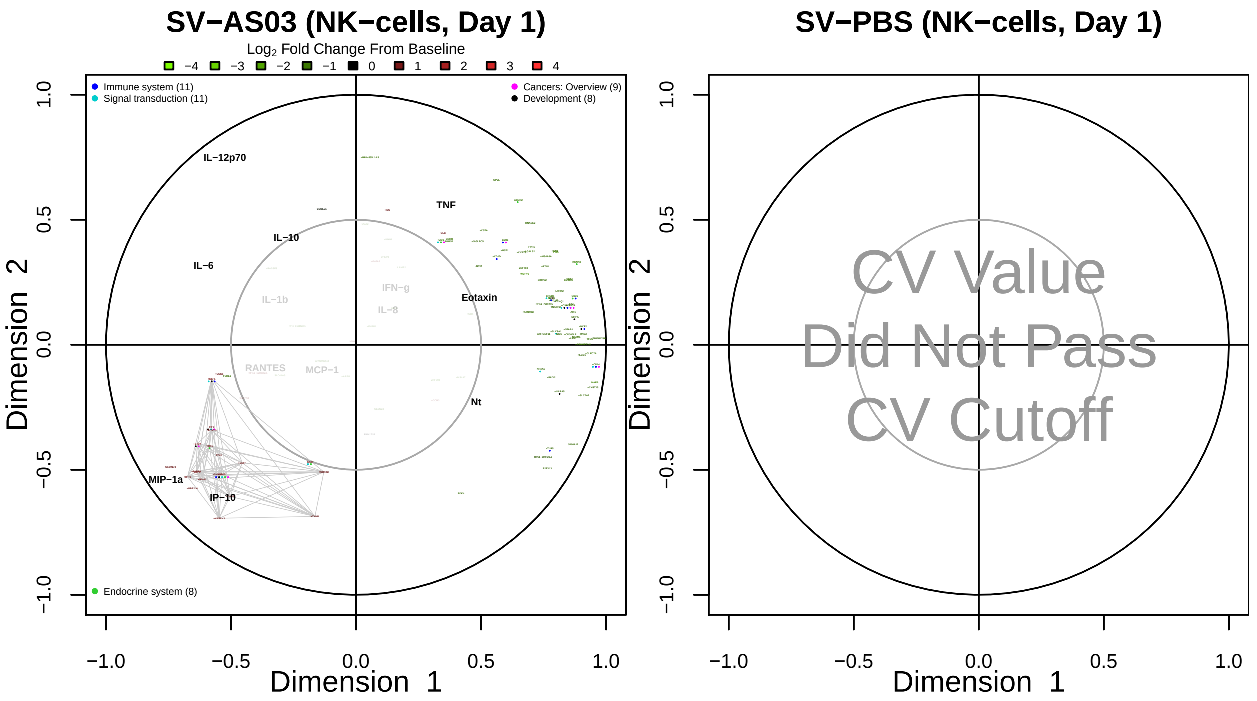

Figure A161: Canonical correlation plots (RNA-Seq, NK-cells, Day 1). In black: IMO variables, green-red: gene variables color-coded by baseline fold change, +/- prefix: SDEG yes/no, colored-dots: pathway class, grey lines: robust clusters.

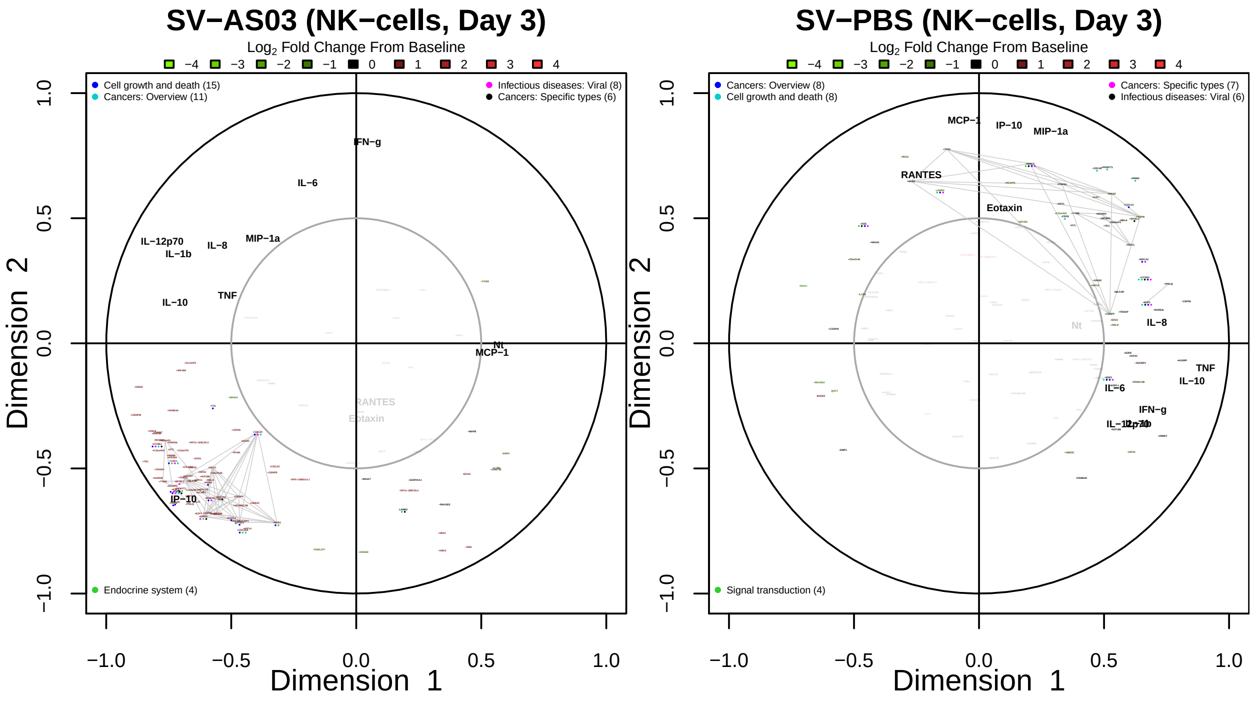

Figure A162: Canonical correlation plots (RNA-Seq, NK-cells, Day 3). In black: IMO variables, green-red: gene variables color-coded by baseline fold change, +/- prefix: SDEG yes/no, colored-dots: pathway class, grey lines: robust clusters.

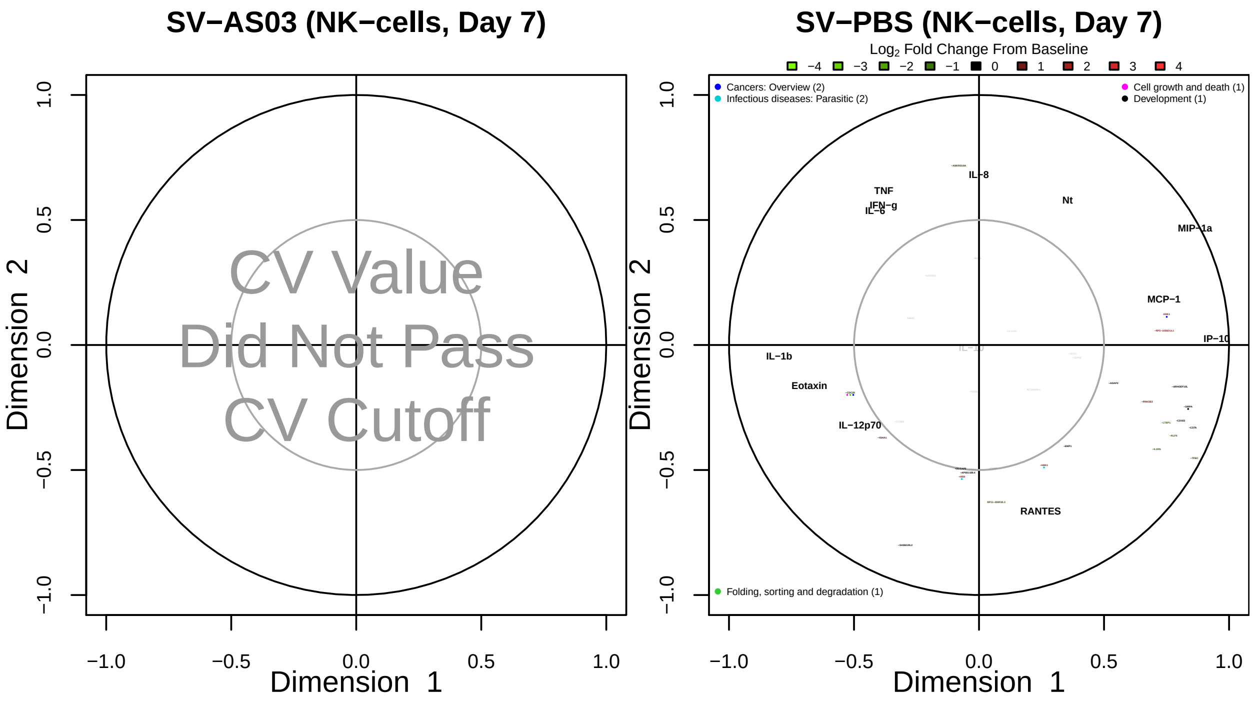

Figure A163: Canonical correlation plots (RNA-Seq, NK-cells, Day 7). In black: IMO variables, green-red: gene variables color-coded by baseline fold change, +/- prefix: SDEG yes/no, colored-dots: pathway class, grey lines: robust clusters.

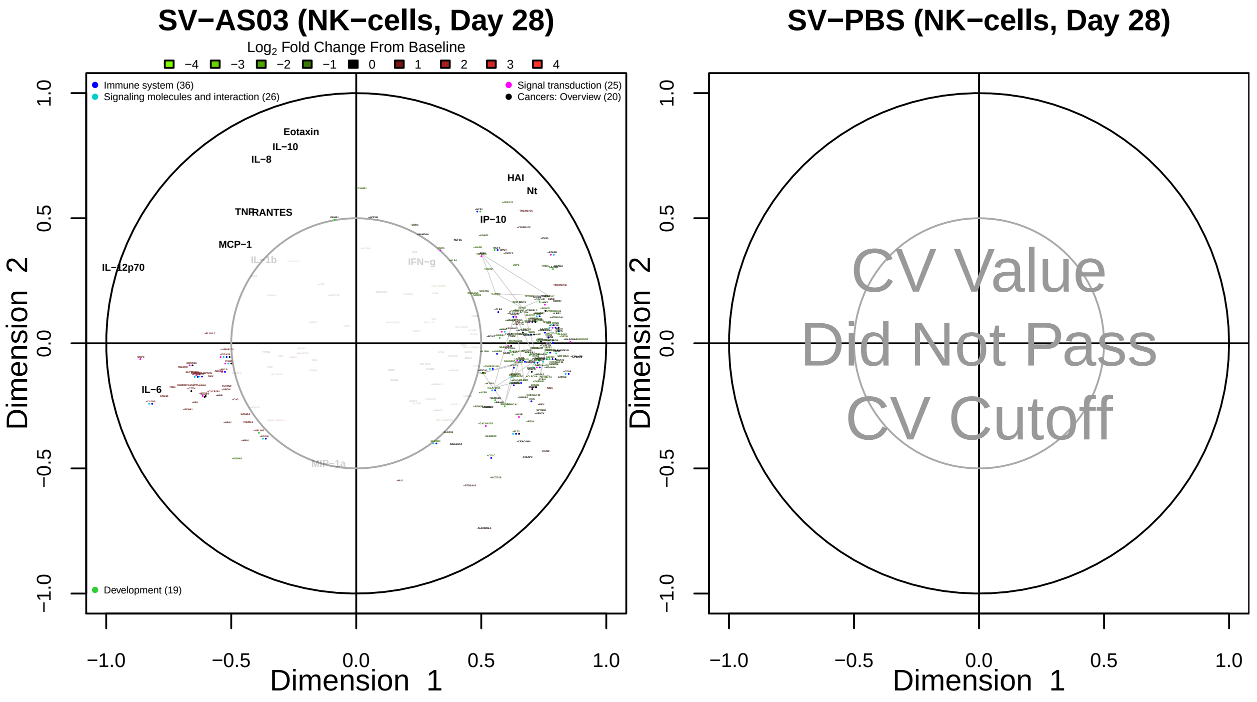

Figure A164: Canonical correlation plots (RNA-Seq, NK-cells, Day 28). In black: IMO variables, green-red: gene variables color-coded by baseline fold change, +/- prefix: SDEG yes/no, colored-dots: pathway class, grey lines: robust clusters.

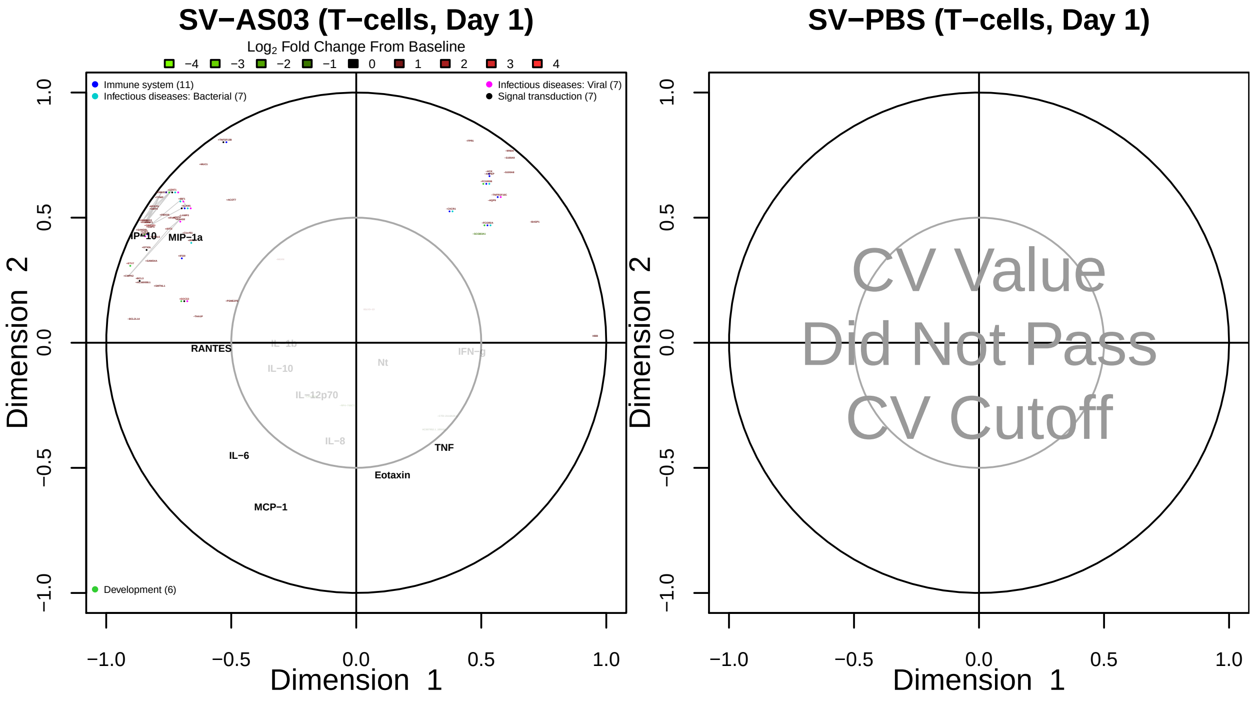

Figure A165: Canonical correlation plots (RNA-Seq, T-cells, Day 1). In black: IMO variables, green-red: gene variables color-coded by baseline fold change, +/- prefix: SDEG yes/no, colored-dots: pathway class, grey lines: robust clusters.

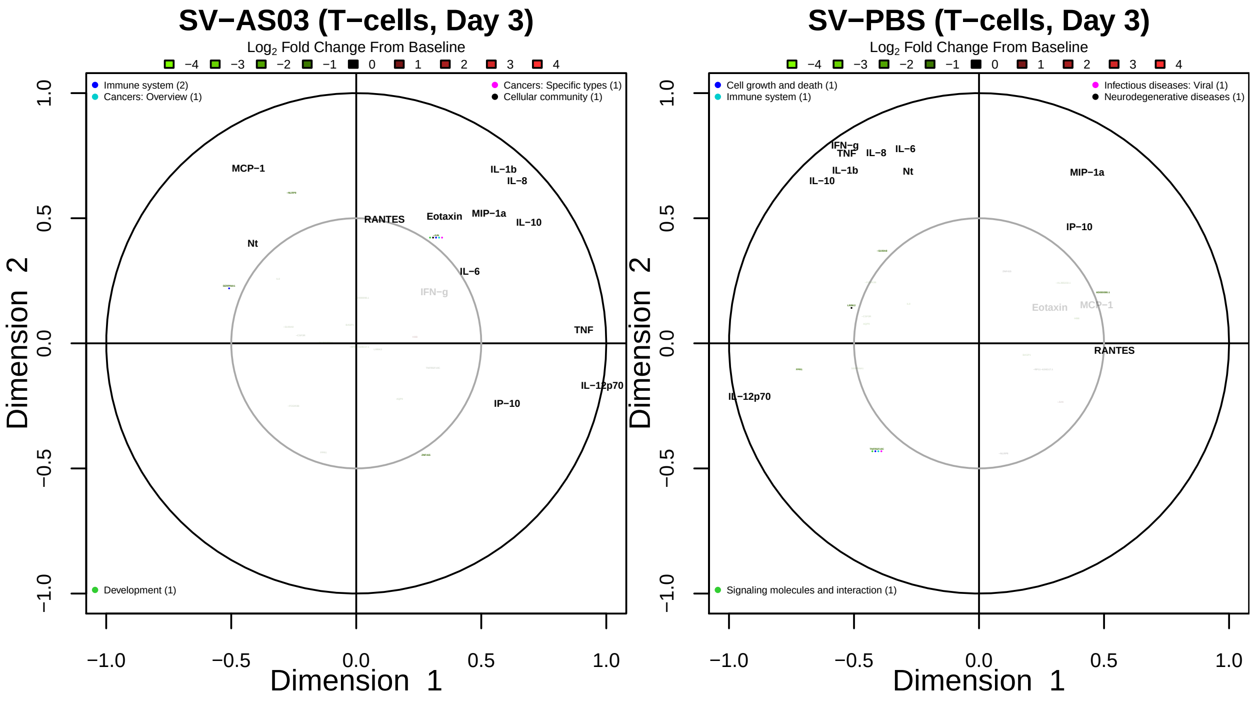

Figure A166: Canonical correlation plots (RNA-Seq, T-cells, Day 3). In black: IMO variables, green-red: gene variables color-coded by baseline fold change, +/- prefix: SDEG yes/no, colored-dots: pathway class, grey lines: robust clusters.

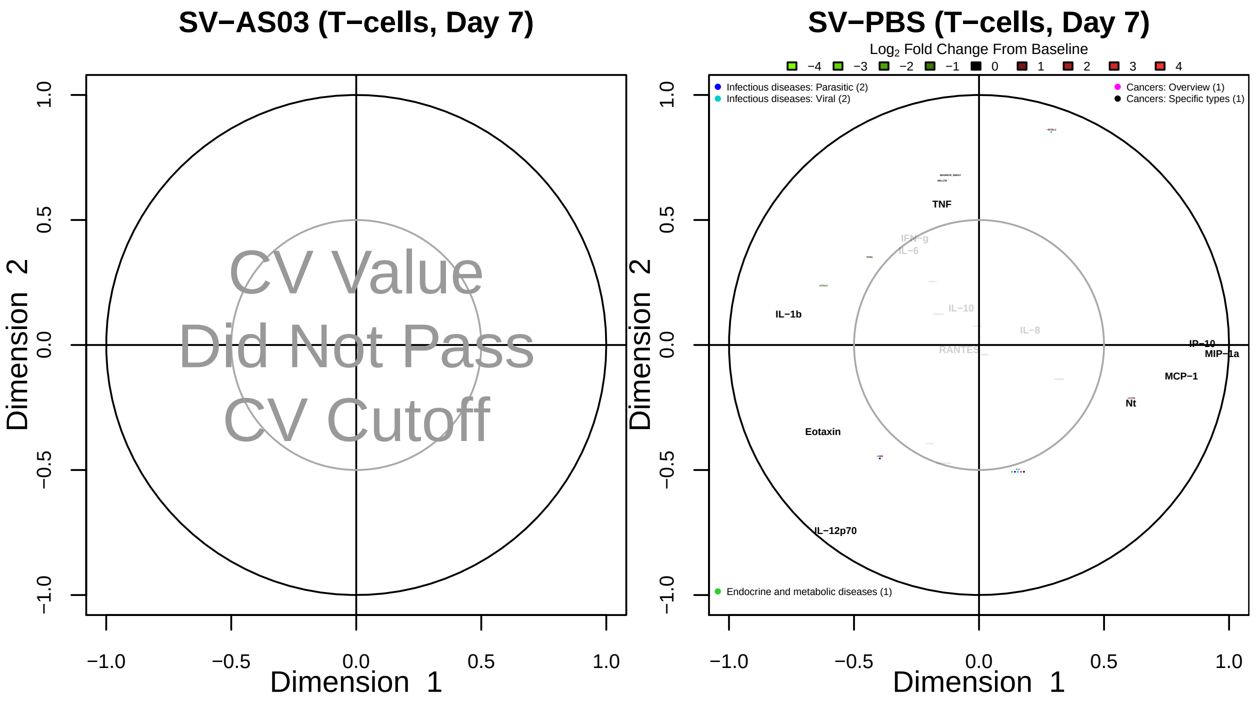

Figure A167: Canonical correlation plots (RNA-Seq, T-cells, Day 7). In black: IMO variables, green-red: gene variables color-coded by baseline fold change, +/- prefix: SDEG yes/no, colored-dots: pathway class, grey lines: robust clusters.

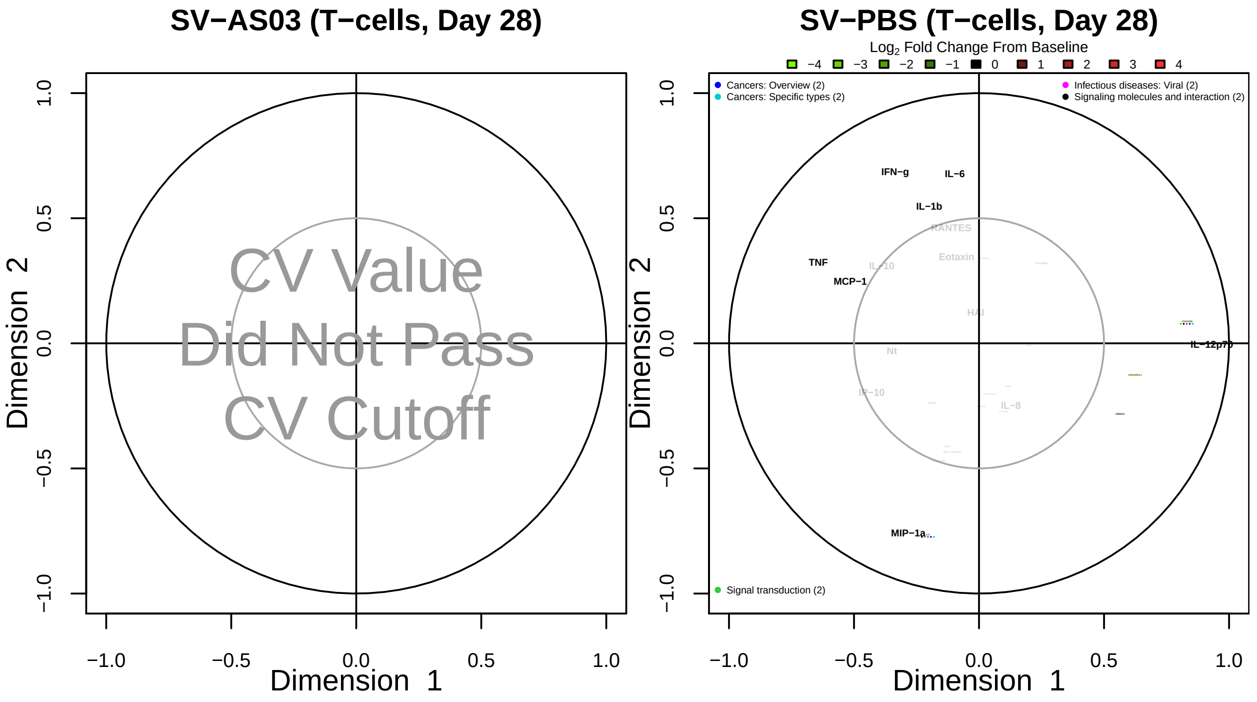

Figure A168: Canonical correlation plots (RNA-Seq, T-cells, Day 28). In black: IMO variables, green-red: gene variables color-coded by baseline fold change, +/- prefix: SDEG yes/no, colored-dots: pathway class, grey lines: robust clusters.

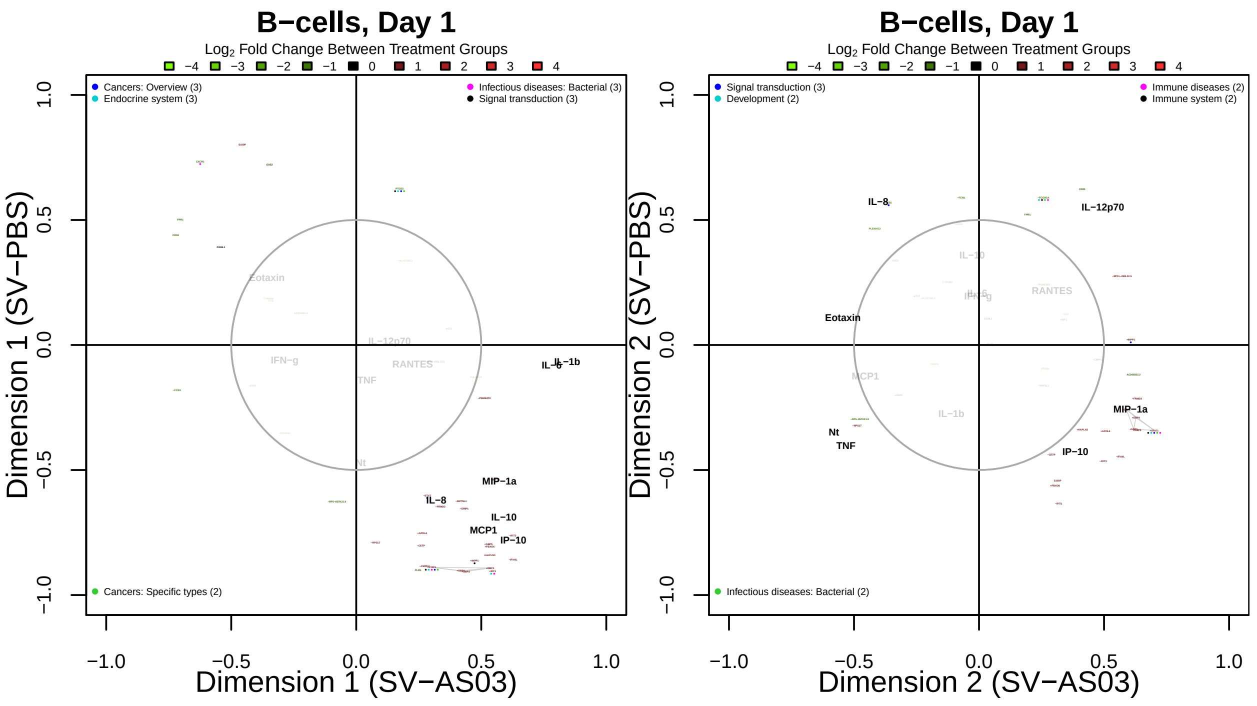

Figure A169: Canonical correlation plots (RNA-Seq, B-cells, Day 1, SV-AS03 vs. SV-PBS). In black: IMO variables, green-red: gene variables color-coded by vaccine group fold change difference (LFCD), +/- prefix: SDEG yes/no, colored-dots: pathway class, grey lines: robust clusters.

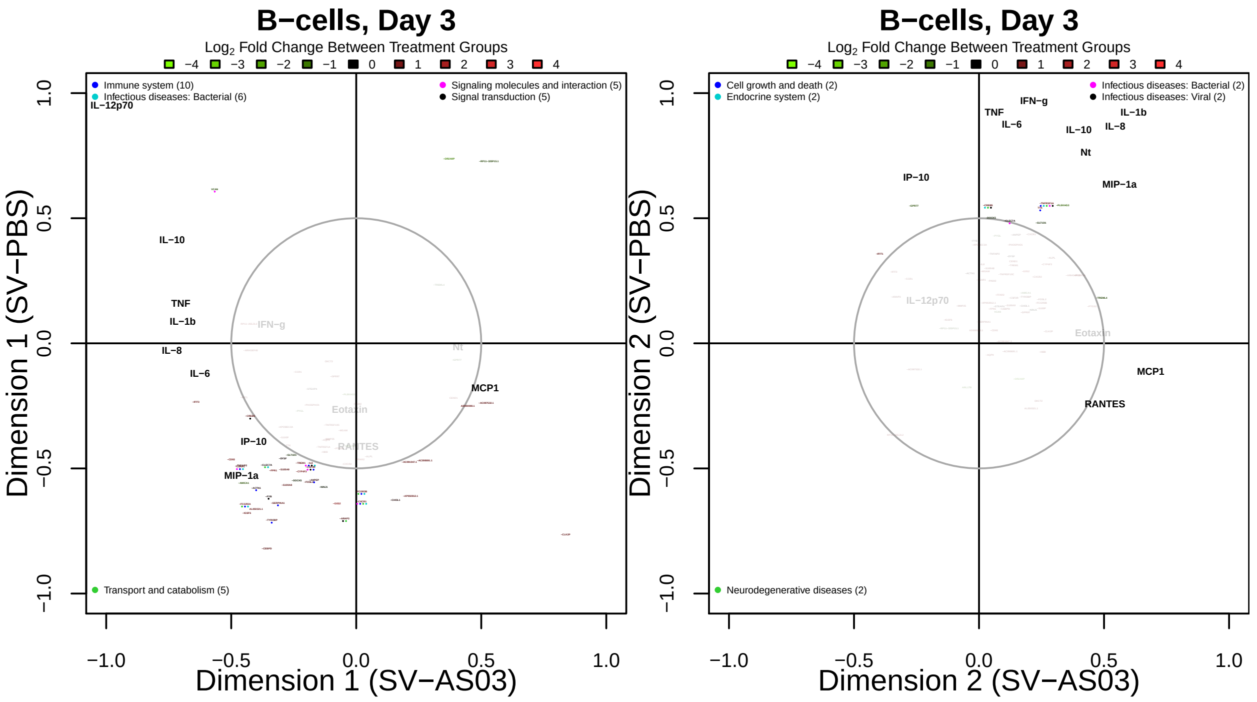

Figure A170: Canonical correlation plots (RNA-Seq, B-cells, Day 3, SV-AS03 vs. SV-PBS). In black: IMO variables, green-red: gene variables color-coded by vaccine group fold change difference (LFCD), +/- prefix: SDEG yes/no, colored-dots: pathway class, grey lines: robust clusters.

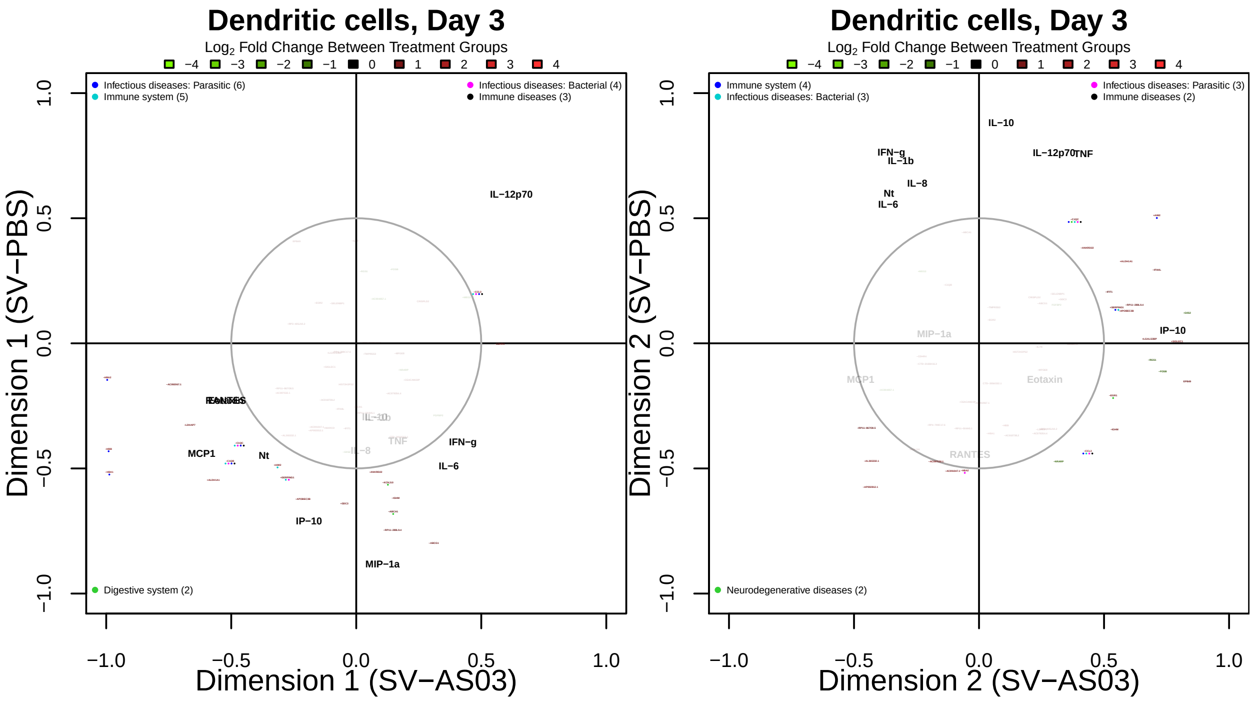

Figure A171: Canonical correlation plots (RNA-Seq, Dendritic cells, Day 3, SV-AS03 vs. SV-PBS). In black: IMO variables, green-red: gene variables color-coded by vaccine group fold change difference (LFCD), +/- prefix: SDEG yes/no, colored-dots: pathway class, grey lines: robust clusters.

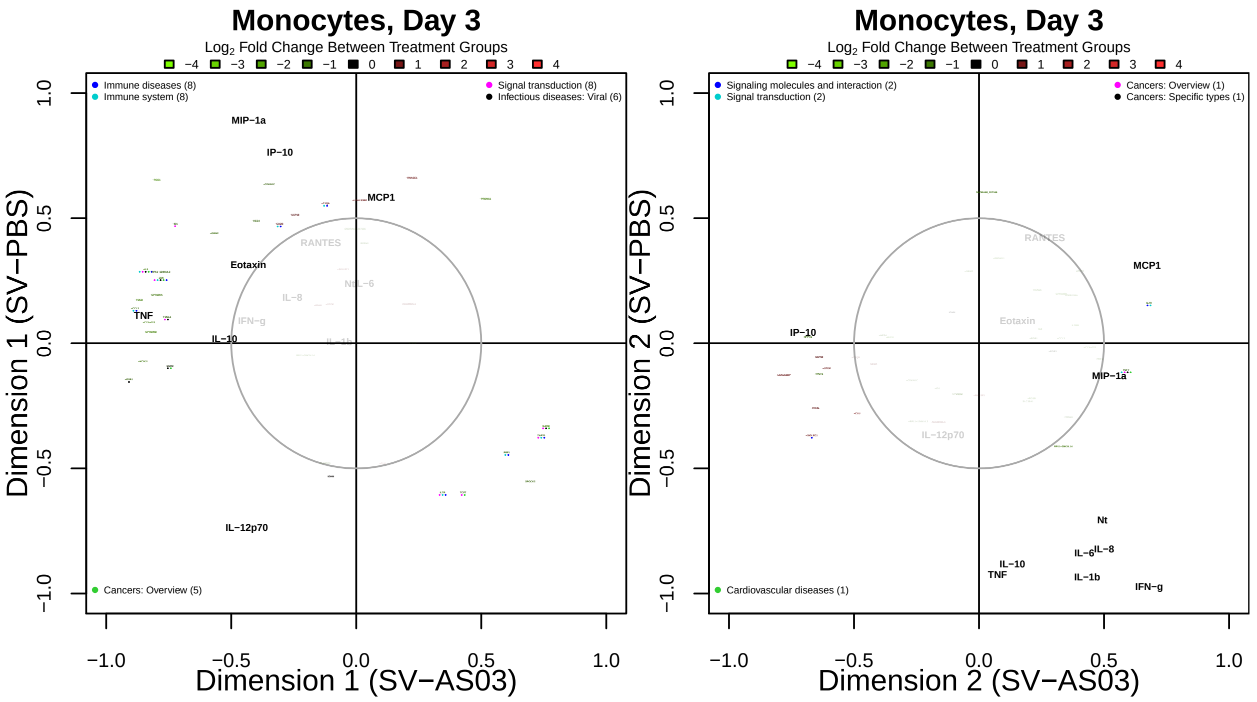

Figure A172: Canonical correlation plots (RNA-Seq, Monocytes, Day 3, SV-AS03 vs. SV-PBS). In black: IMO variables, green-red: gene variables color-coded by vaccine group fold change difference (LFCD), +/- prefix: SDEG yes/no, colored-dots: pathway class, grey lines: robust clusters.

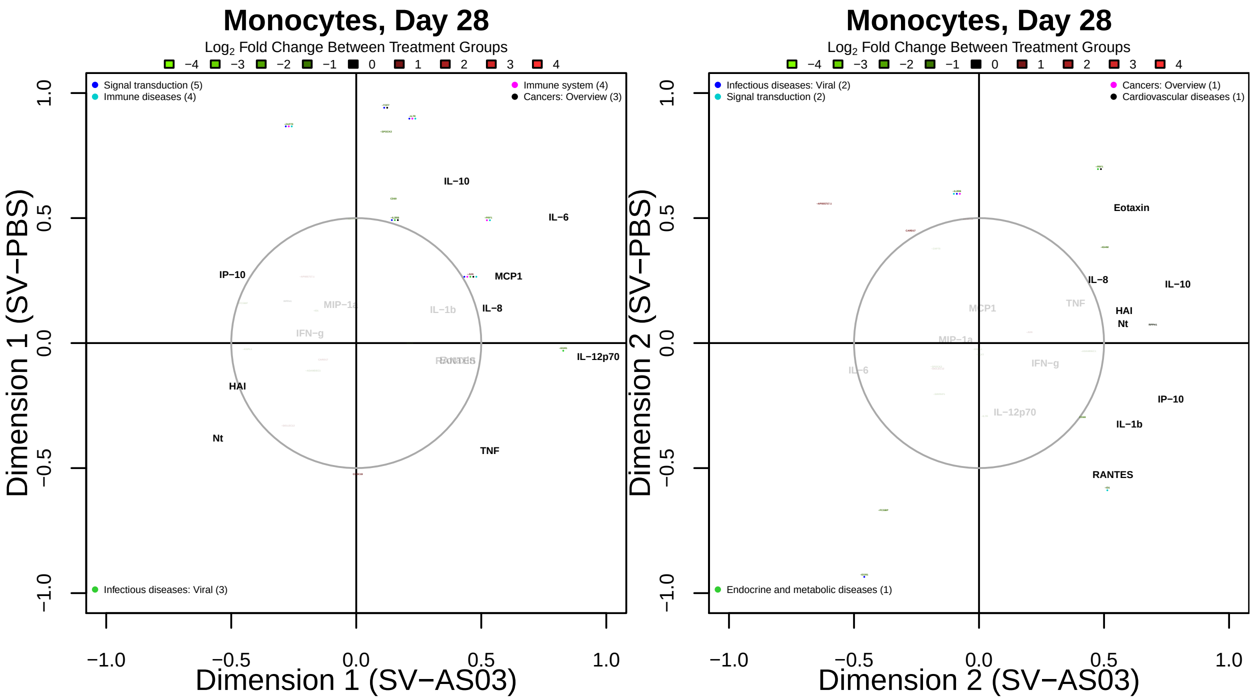

Figure A173: Canonical correlation plots (RNA-Seq, Monocytes, Day 28, SV-AS03 vs. SV-PBS). In black: IMO variables, green-red: gene variables color-coded by vaccine group fold change difference (LFCD), +/- prefix: SDEG yes/no, colored-dots: pathway class, grey lines: robust clusters.

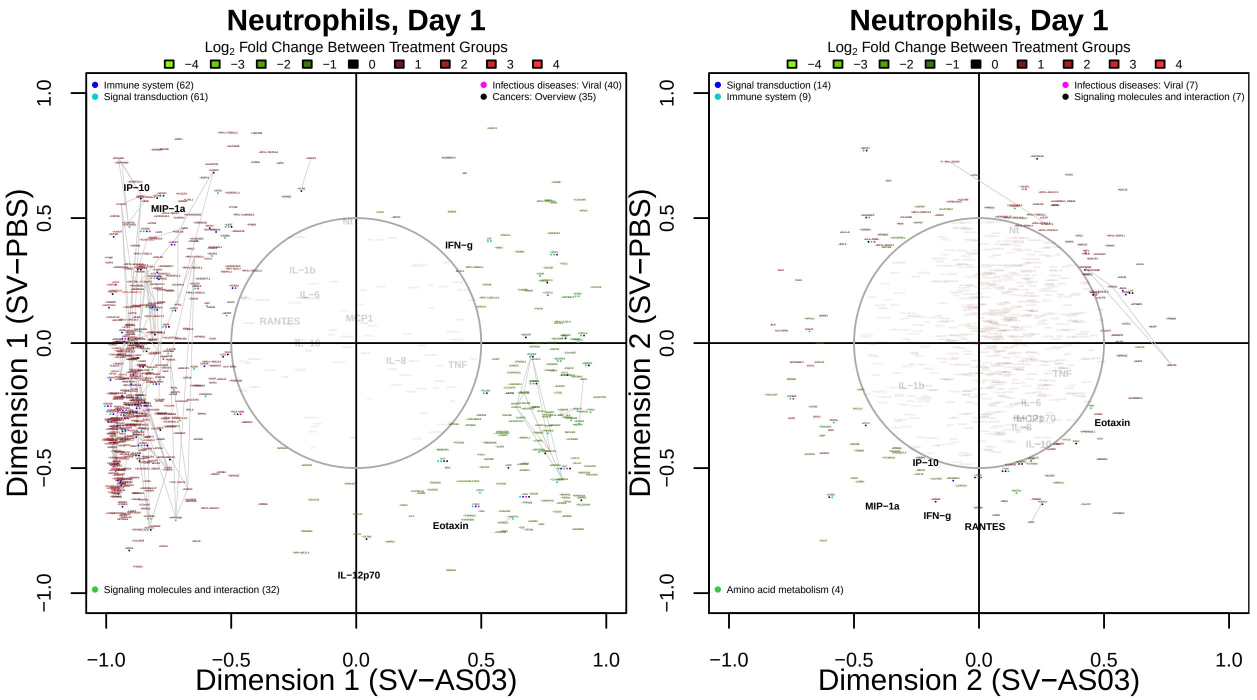

Figure A174: Canonical correlation plots (RNA-Seq, Neutrophils, Day 1, SV-AS03 vs. SV-PBS). In black: IMO variables, green-red: gene variables color-coded by vaccine group fold change difference (LFCD), +/- prefix: SDEG yes/no, colored-dots: pathway class, grey lines: robust clusters.

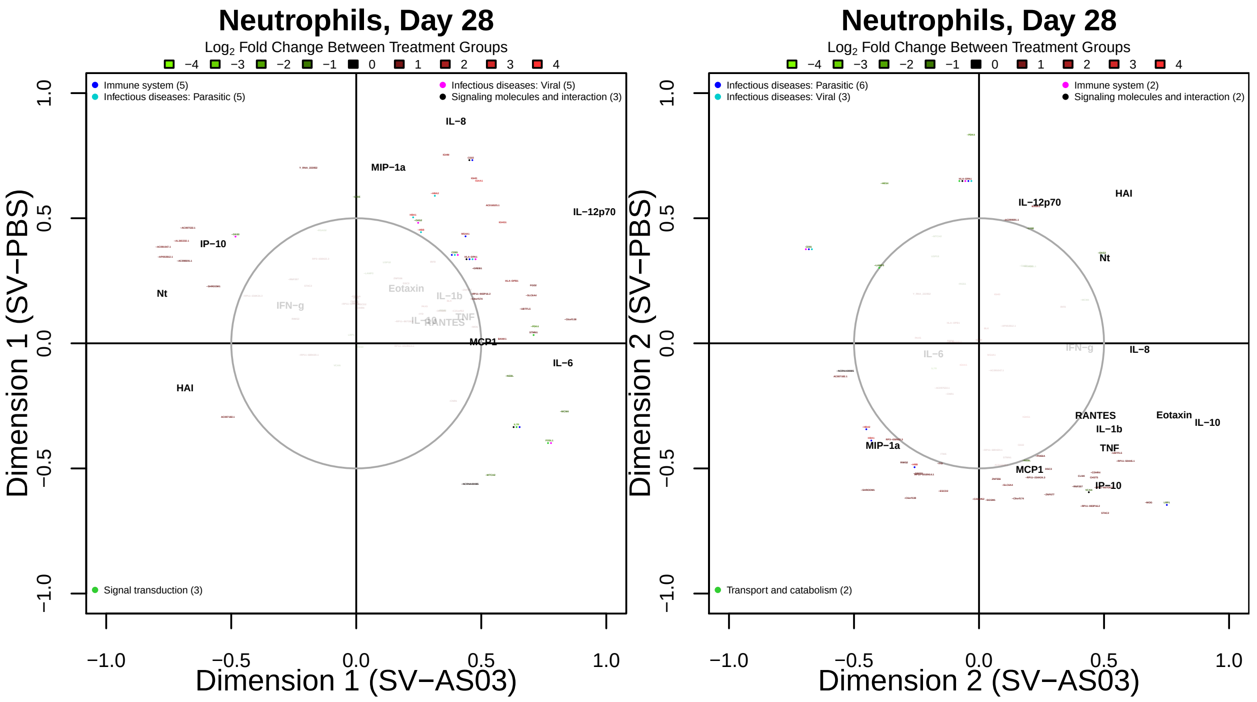

Figure A175: Canonical correlation plots (RNA-Seq, Neutrophils, Day 28, SV-AS03 vs. SV-PBS). In black: IMO variables, green-red: gene variables color-coded by vaccine group fold change difference (LFCD), +/- prefix: SDEG yes/no, colored-dots: pathway class, grey lines: robust clusters.

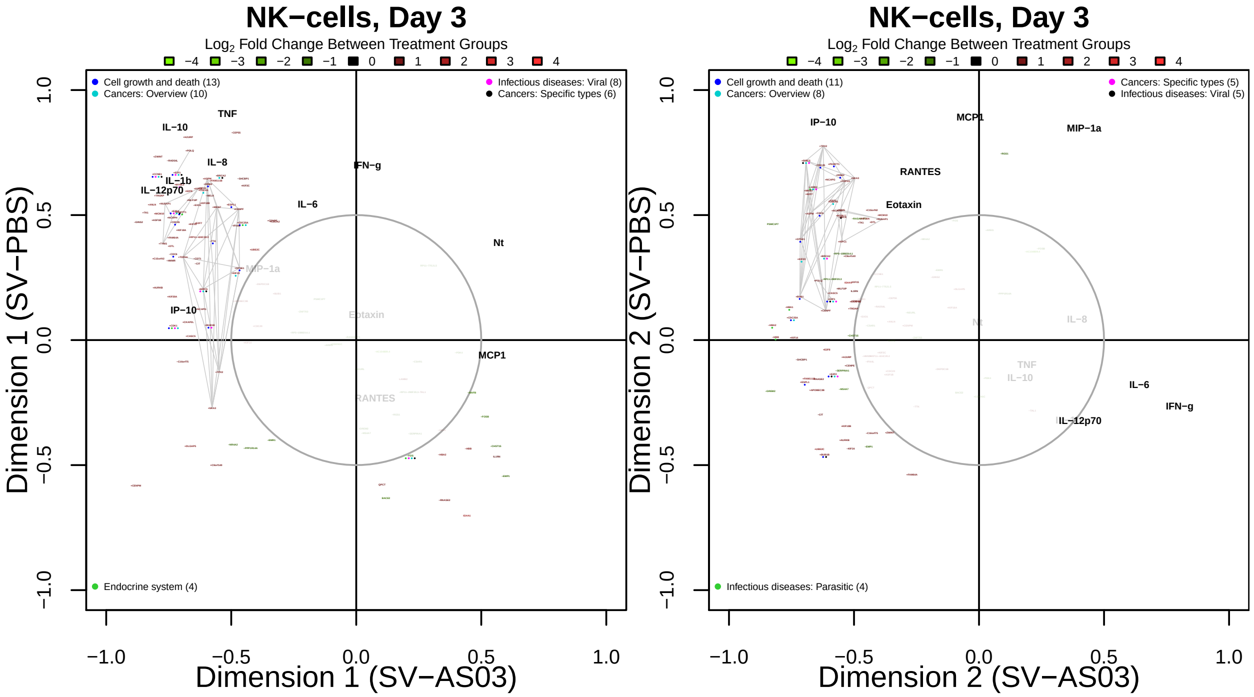

Figure A176: Canonical correlation plots (RNA-Seq, NK-cells, Day 3, SV-AS03 vs. SV-PBS). In black: IMO variables, green-red: gene variables color-coded by vaccine group fold change difference (LFCD), +/- prefix: SDEG yes/no, colored-dots: pathway class, grey lines: robust clusters.

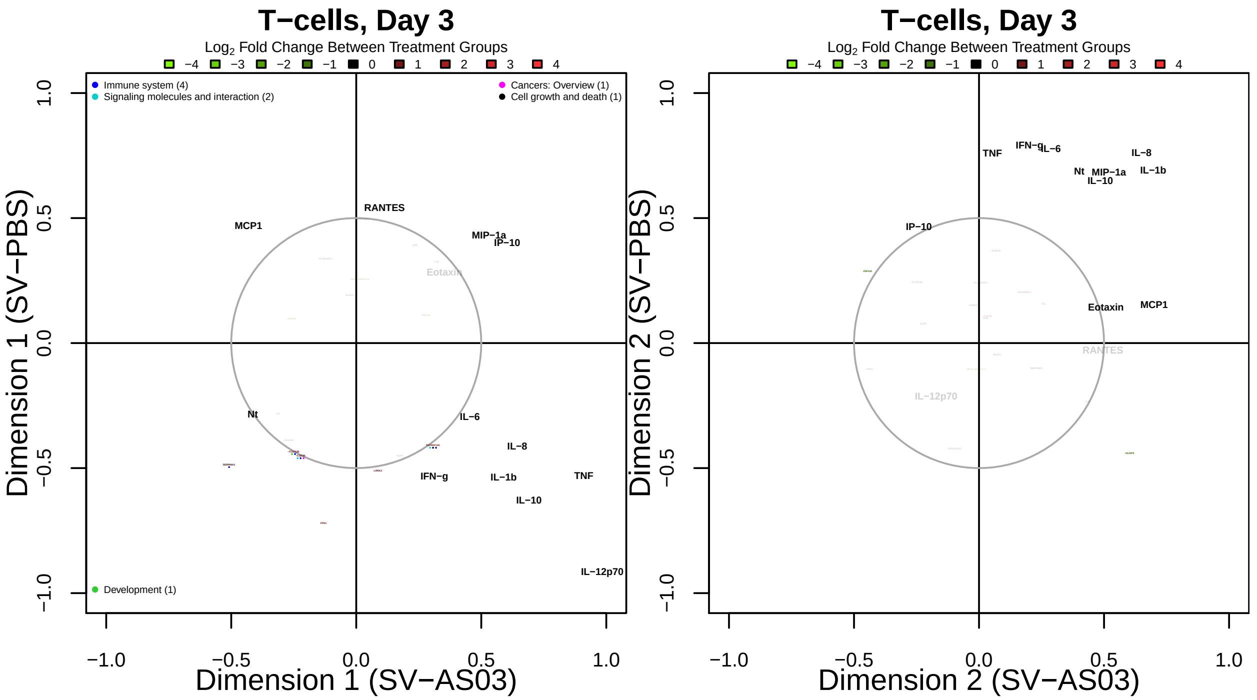

Figure A177: Canonical correlation plots (RNA-Seq, T-cells, Day 3, SV-AS03 vs. SV-PBS). In black: IMO variables, green-red: gene variables color-coded by vaccine group fold change difference (LFCD), +/- prefix: SDEG yes/no, colored-dots: pathway class, grey lines: robust clusters.

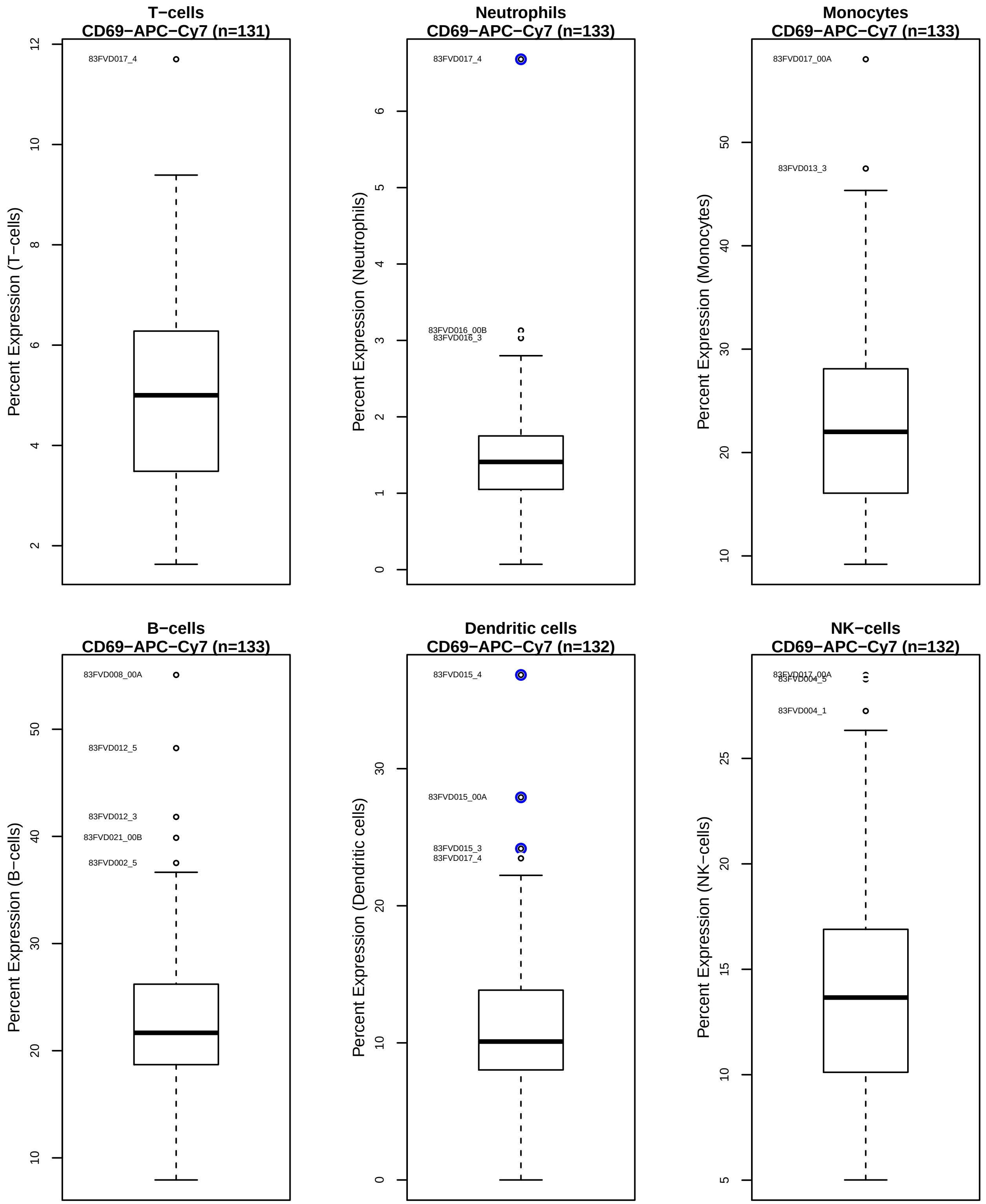

Figure A178: Boxplots of percent cell activation by cell type (FACS, CD69). Identified outliers are colored in blue.

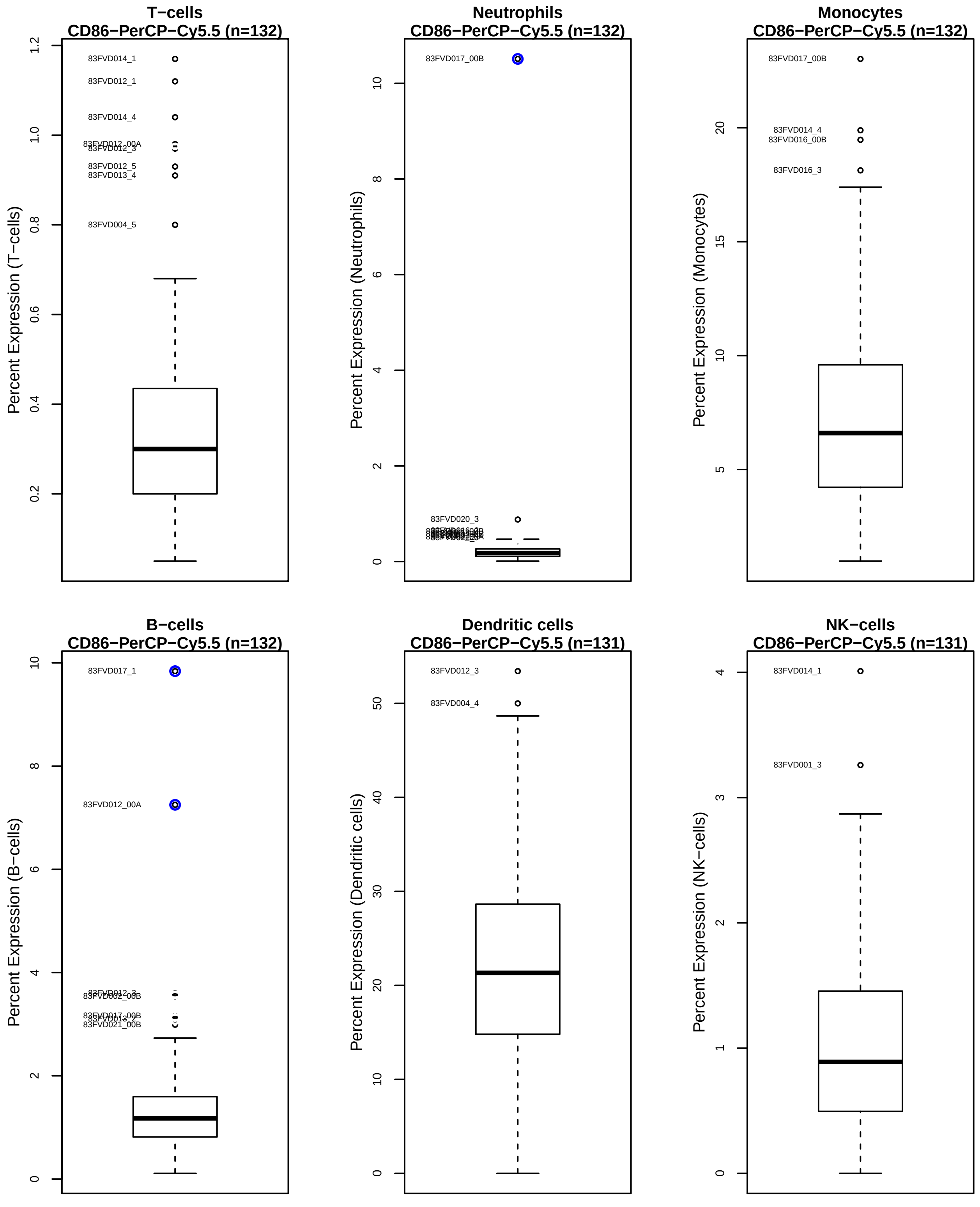

Figure A179: Boxplots of percent cell activation by cell type (FACS, CD86). Identified outliers are colored in blue.

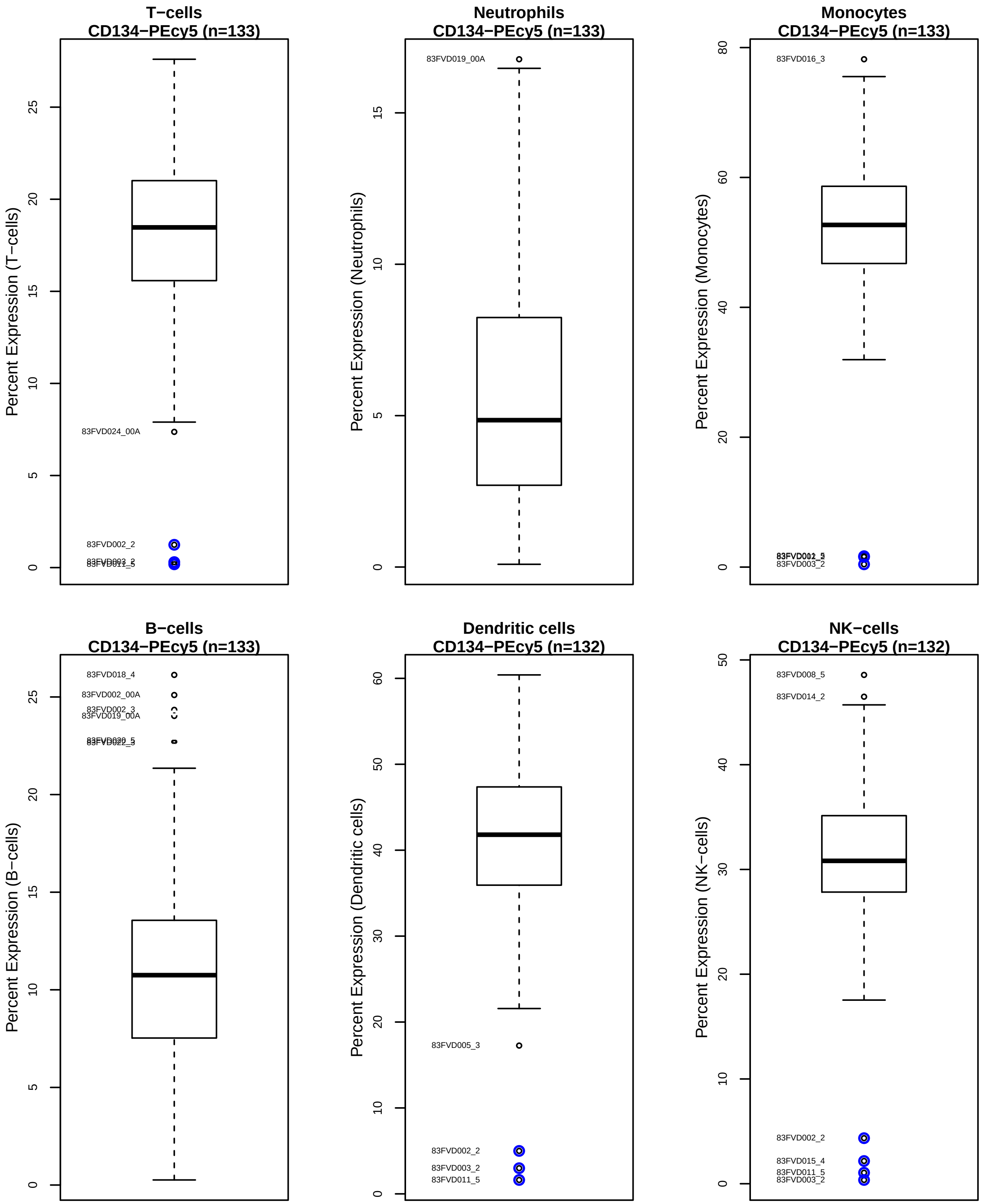

Figure A180: Boxplots of percent cell activation by cell type (FACS, CD134). Identified outliers are colored in blue.

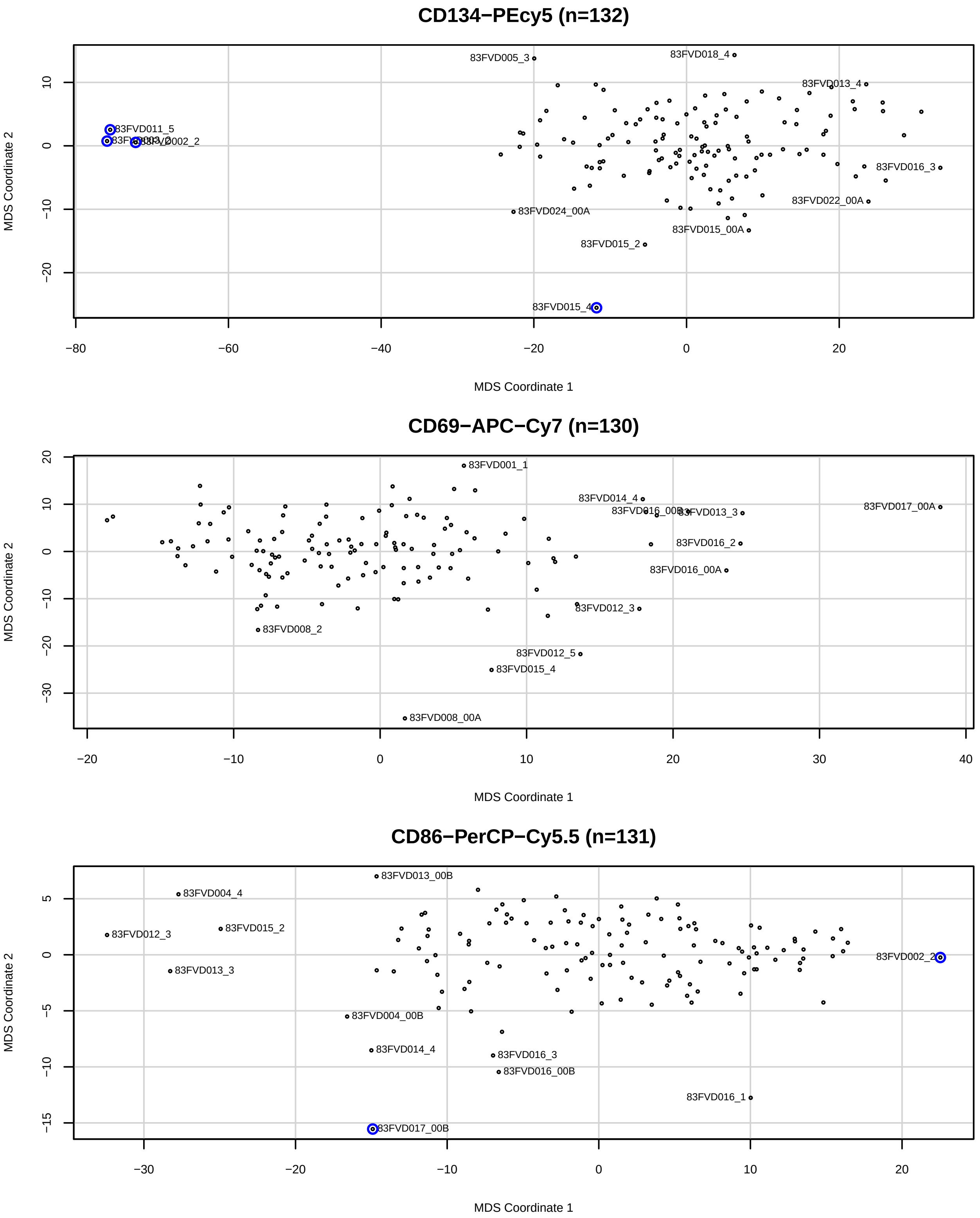

Figure A181: Non-metric multidimensional scaling biplots for each cell activation marker (FACS). For each marker, each subject and time point combination (sample) was represented by a mutivariate vector that included all 6 celltype measurements. Paiwise distances between samples were calculated using the Eucldiean distance. Identified outlying samples are highlighted in blue.

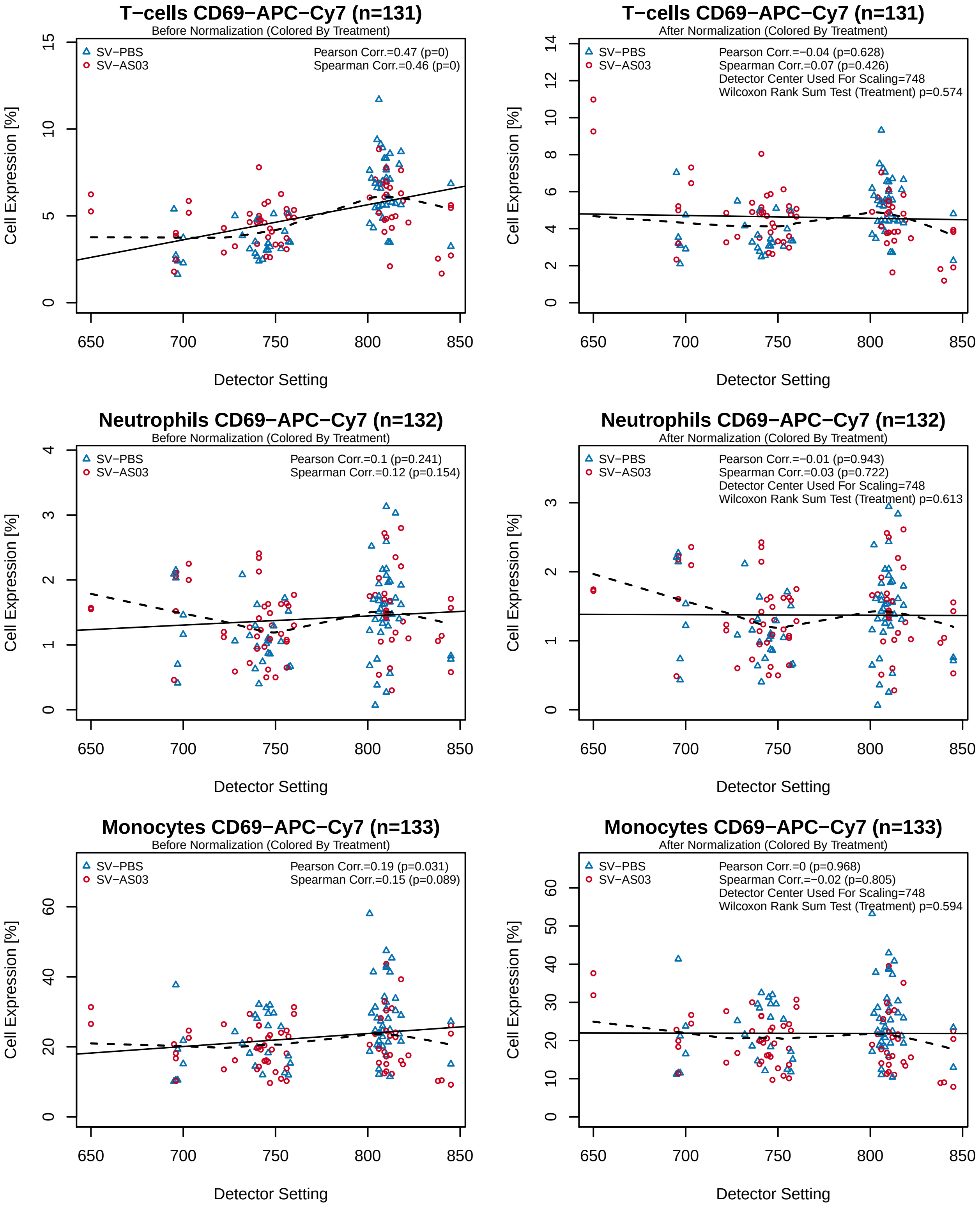

Figure A182: Scatterplots of percent cell activation by detector setting (FACS, CD69, T-cells, neutrophils, monocytes, colored by vaccine group). The left column shows scatter plots before normalization, the right column shows the data after normalization.

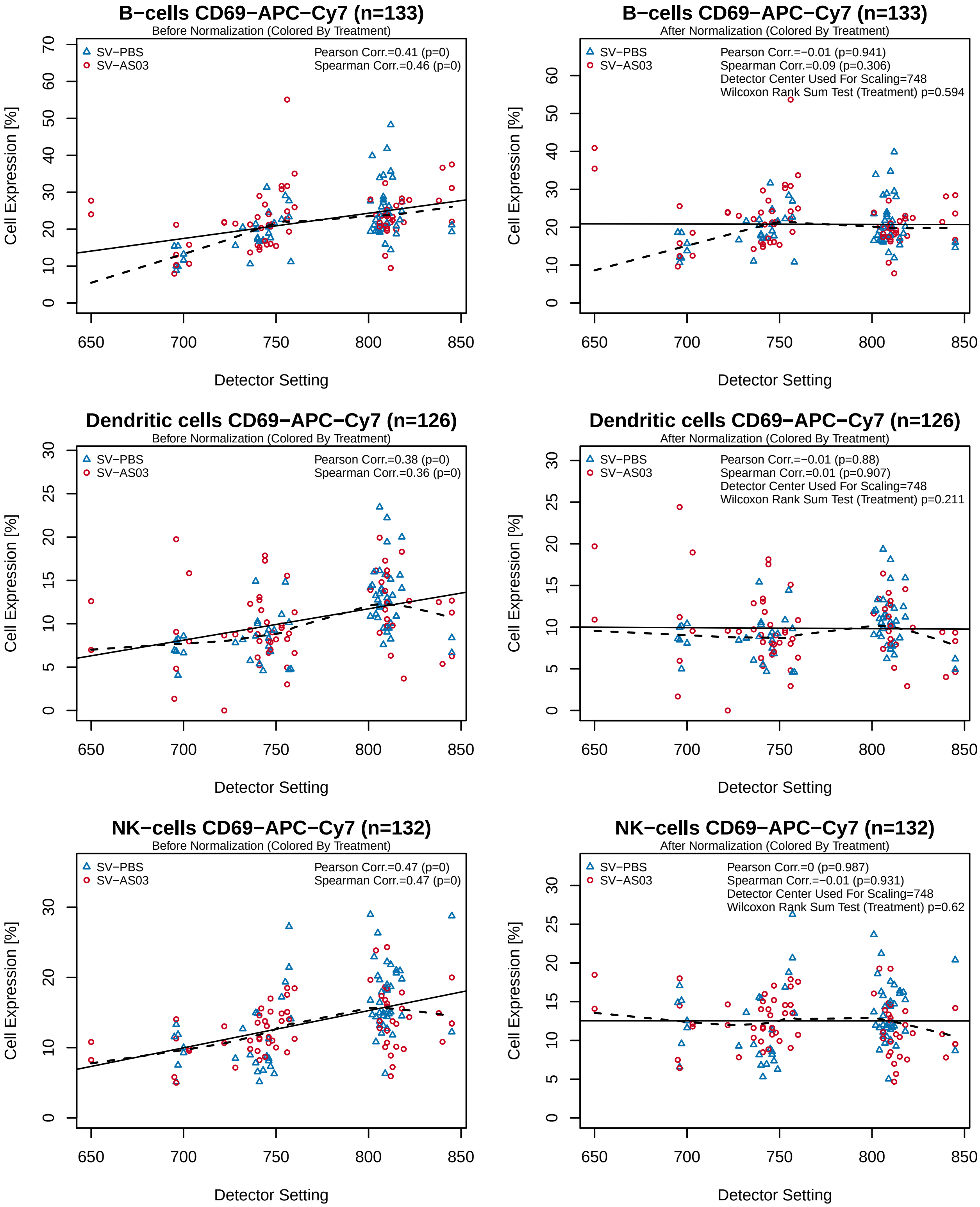

Figure A183: Scatterplots of percent cell activation by detector setting (FACS, CD69, B-cells, dendritic cells, NK-cells, colored by vaccine group). The left column shows scatter plots before normalization, the right column shows the data after normalization.

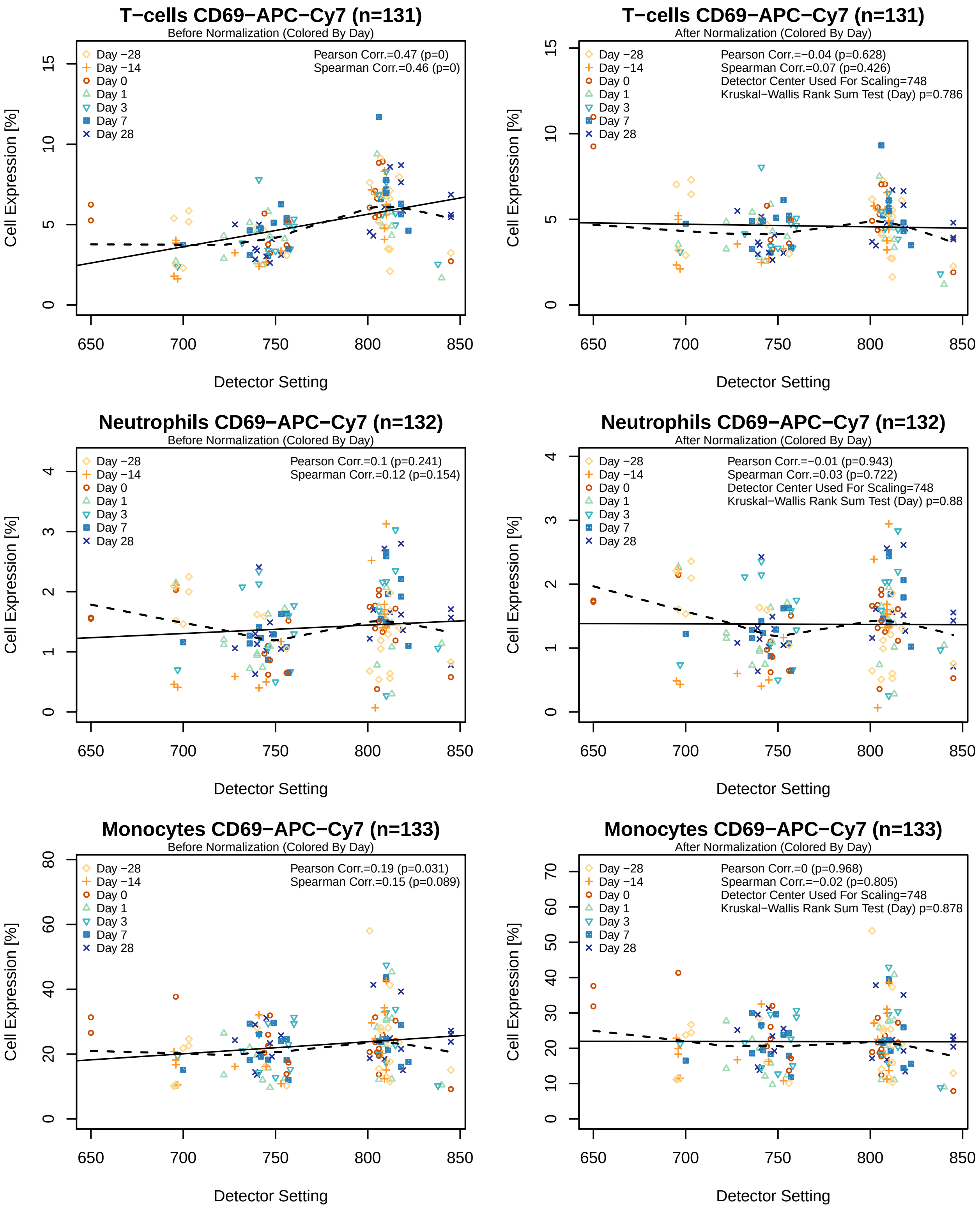

Figure A184: Scatterplots of percent cell activation by detector setting (FACS, CD69, T-cells, neutrophils, monocytes, colored by study visit day). The left column shows scatter plots before normalization, the right column shows the data after normalization.

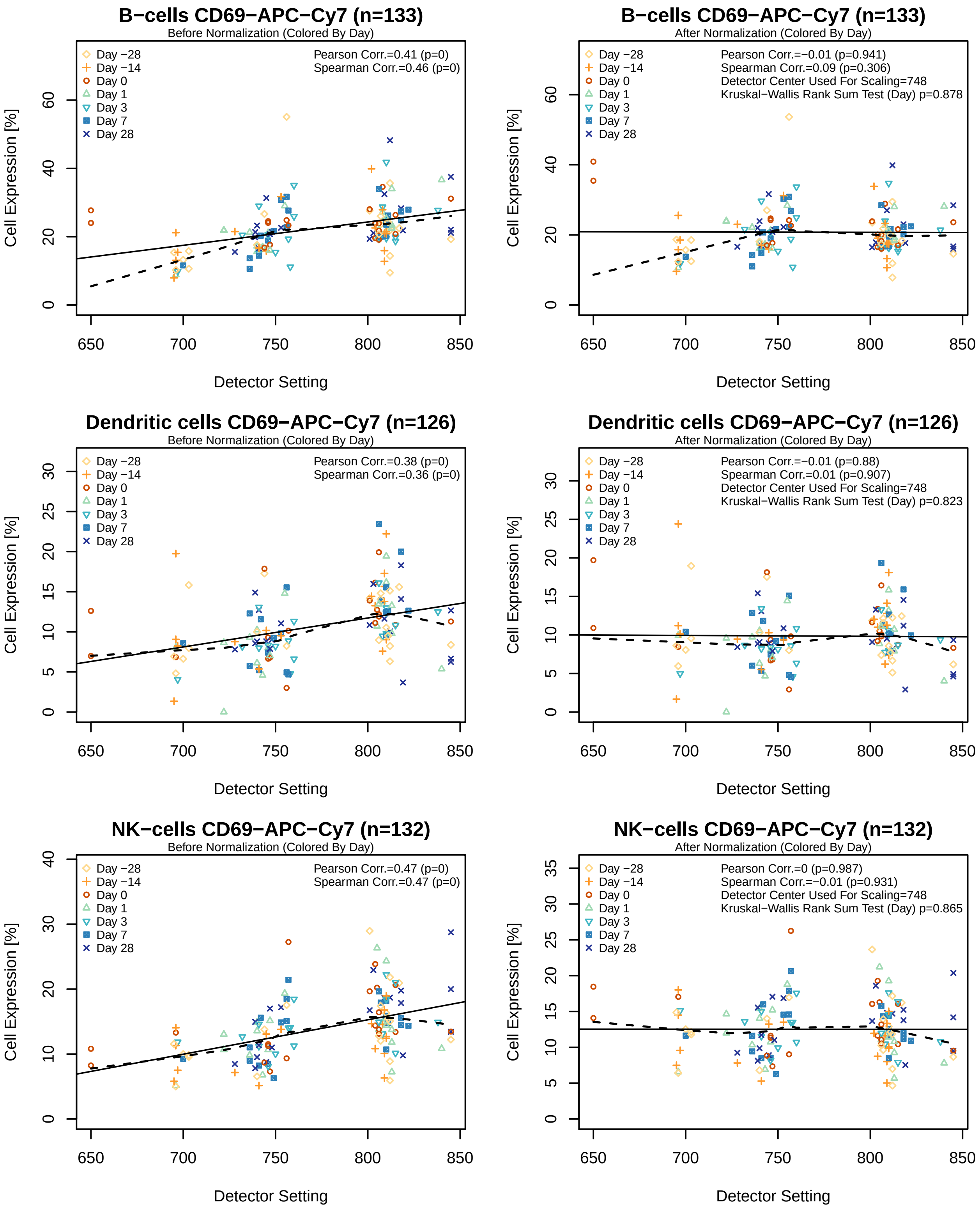

Figure A185: Scatterplots of percent cell activation by detector setting (FACS, CD69, B-cells, dendritic cells, NK-cells, colored by study visit day). The left column shows scatter plots before normalization, the right column shows the data after normalization.

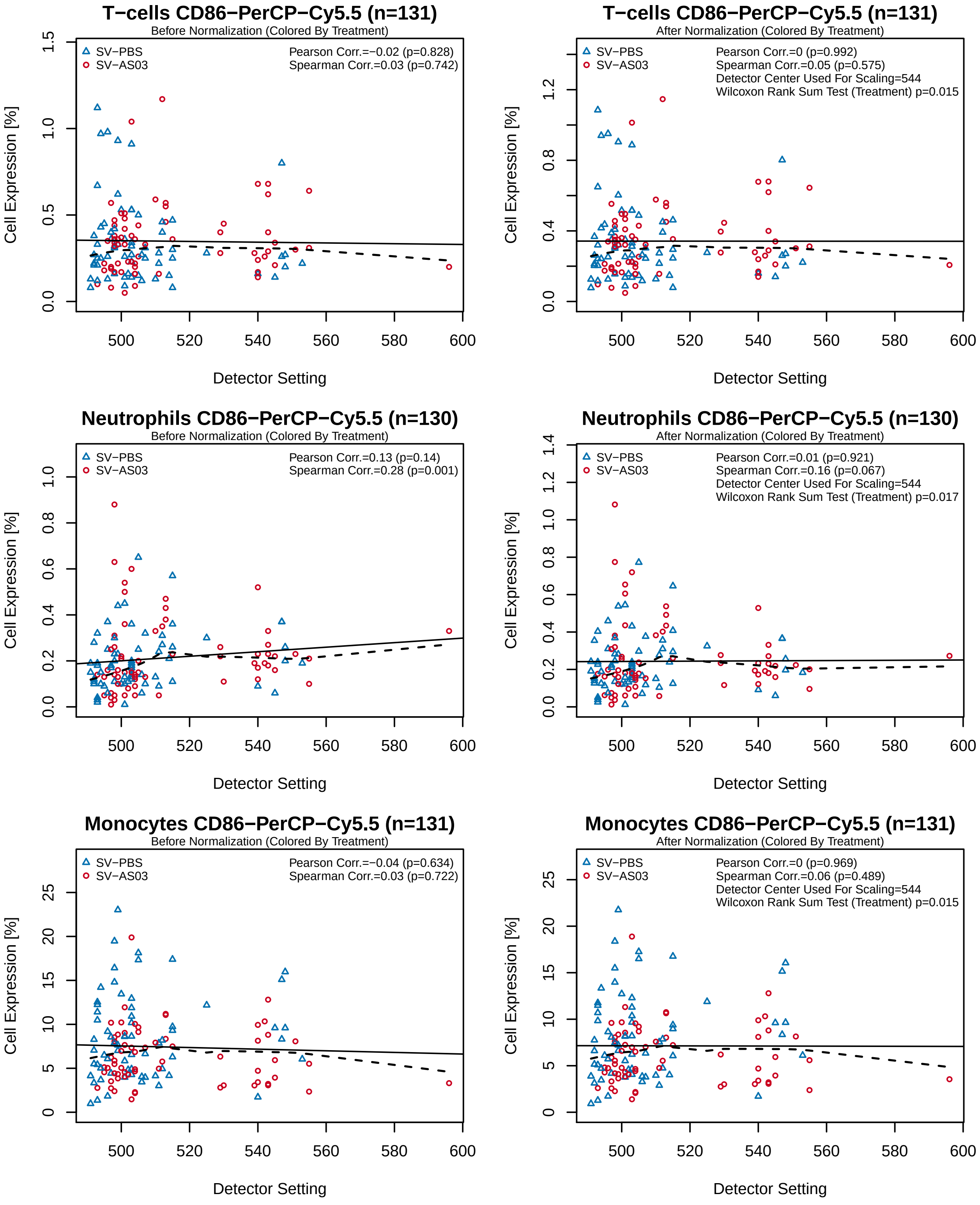

Figure A186: Scatterplots of percent cell activation by detector setting (FACS, CD86, T-cells, neutrophils, monocytes, colored by vaccine group). The left column shows scatter plots before normalization, the right column shows the data after normalization.

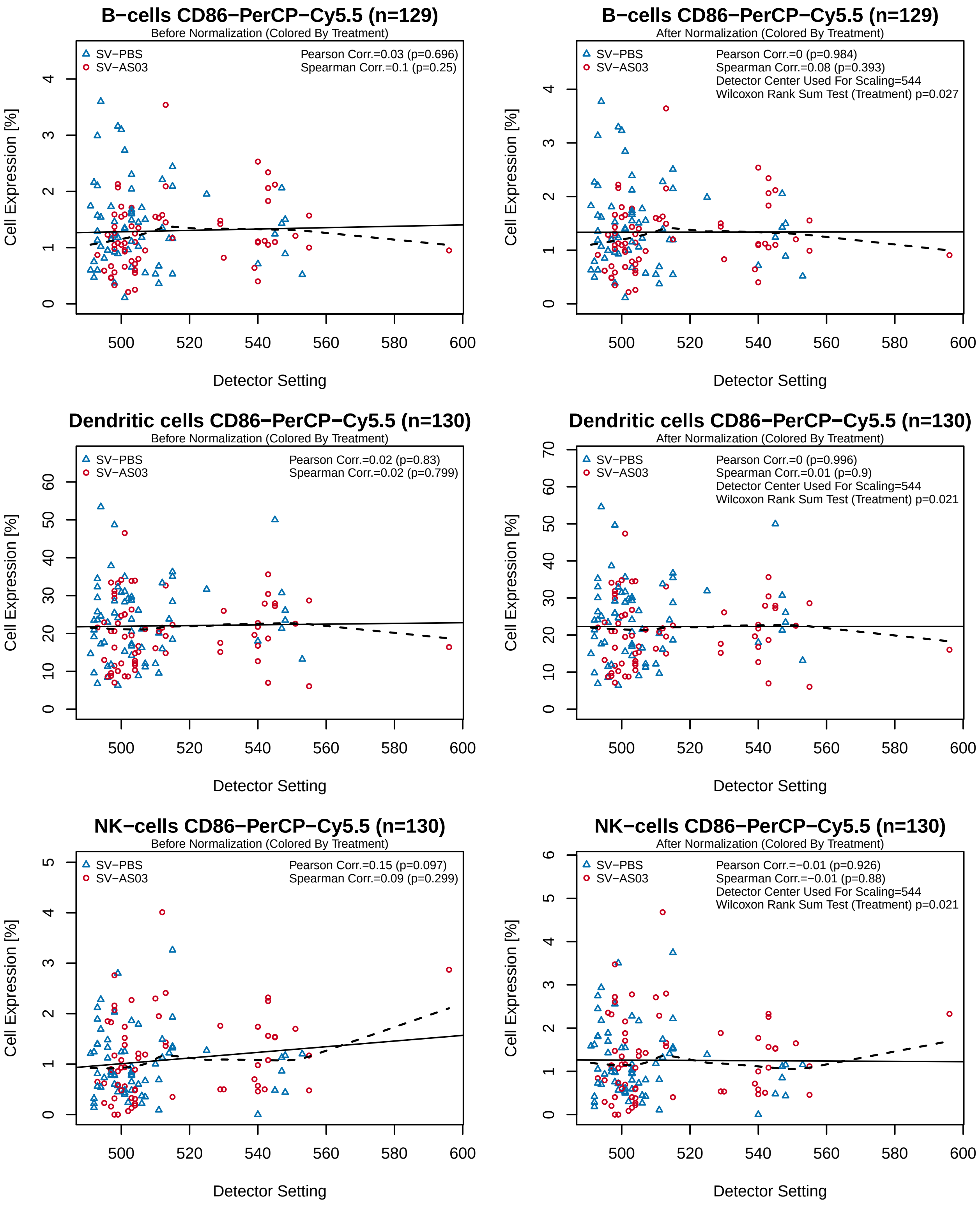

Figure A187: Scatterplots of percent cell activation by detector setting (FACS, CD86, B-cells, dendritic cells, NK-cells, colored by vaccine group). The left column shows scatter plots before normalization, the right column shows the data after normalization.

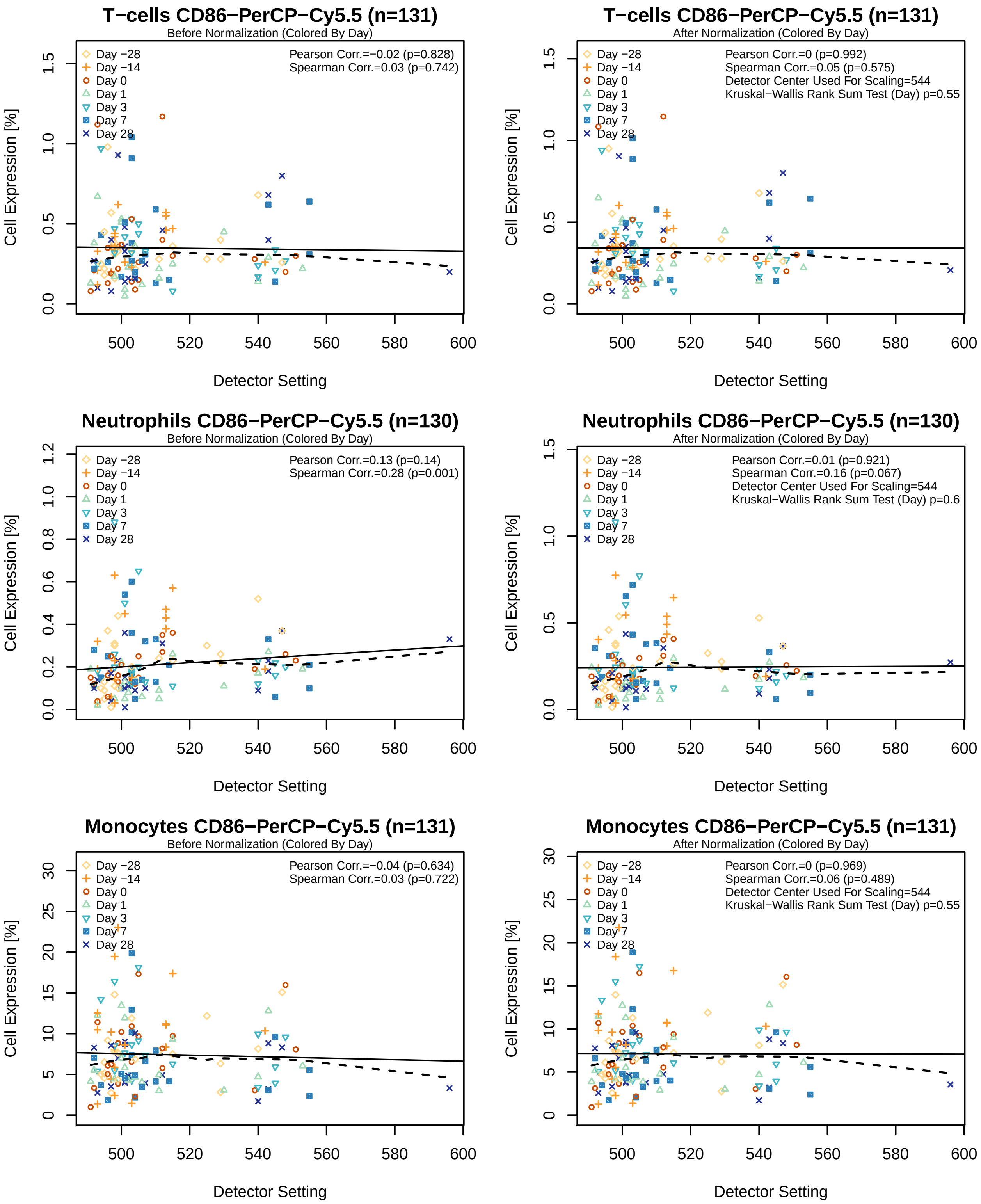

Figure A188: Scatterplots of percent cell activation by detector setting (FACS, CD86, T-cells, neutrophils, monocytes, colored by study visit day). The left column shows scatter plots before normalization, the right column shows the data after normalization.

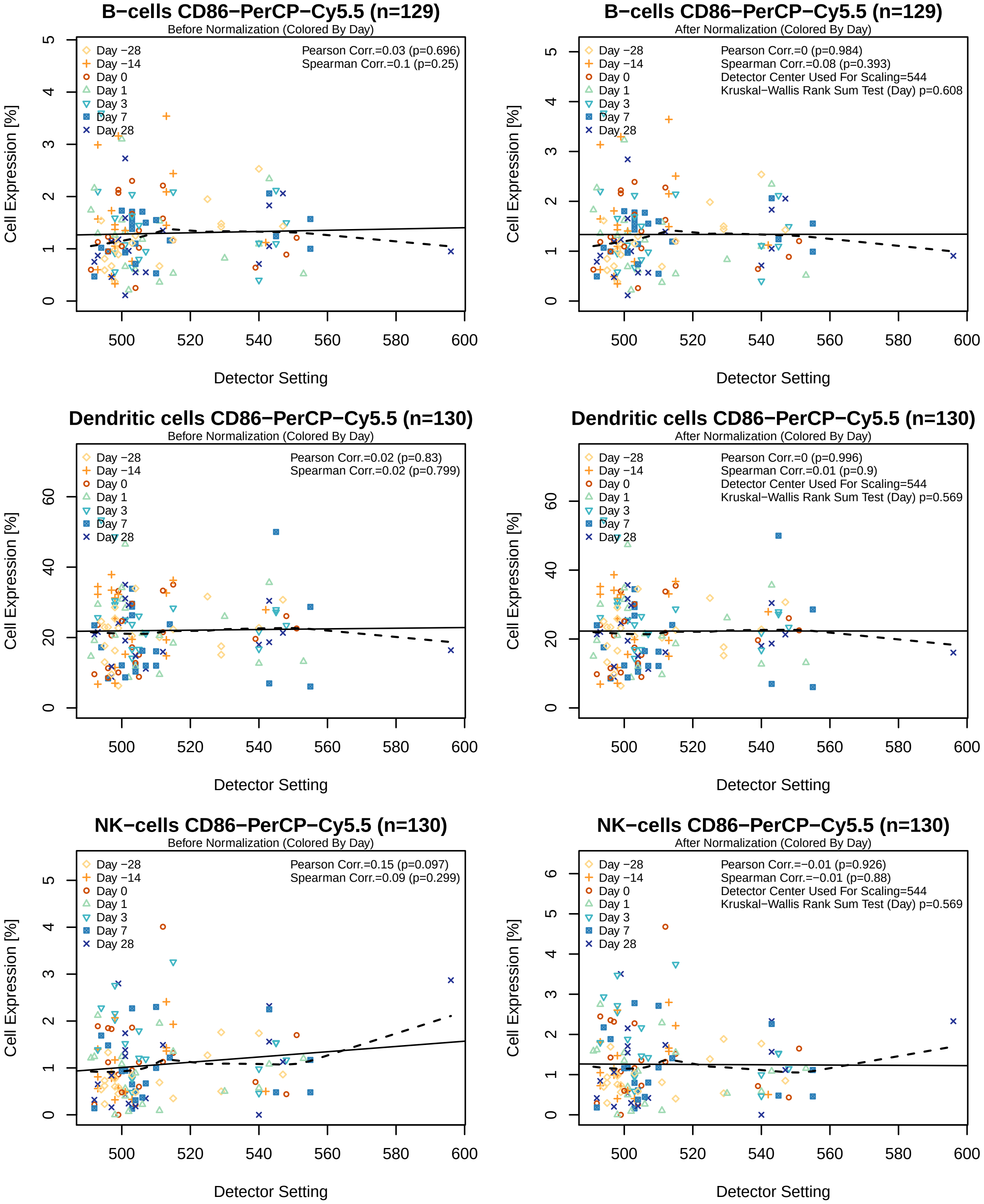

Figure A189: Scatterplots of percent cell activation by detector setting (FACS, CD86, B-cells, dendritic cells, NK-cells, colored by study visit day). The left column shows scatter plots before normalization, the right column shows the data after normalization.

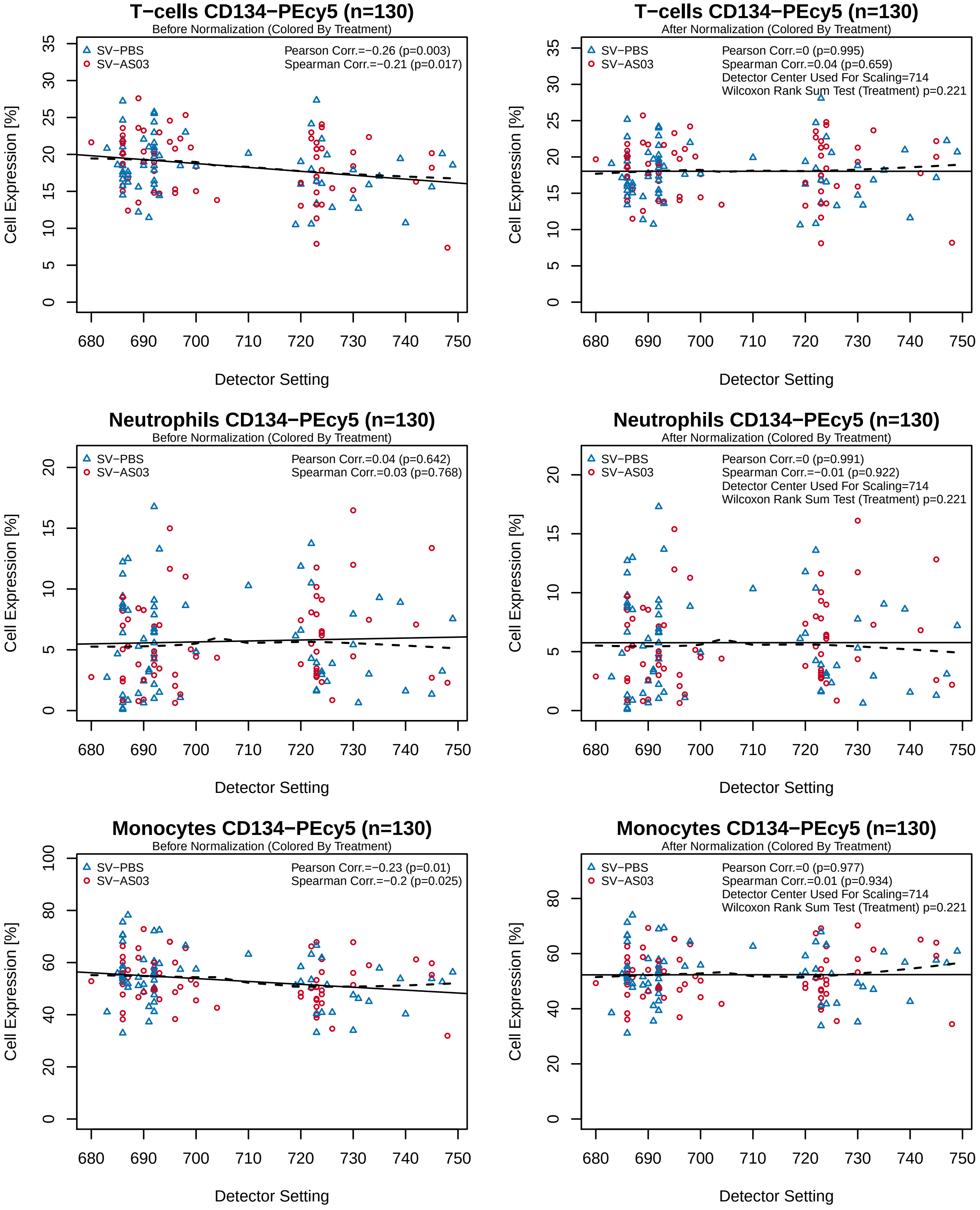

Figure A190: Scatterplots of percent cell activation by detector setting (FACS, CD134, T-cells, neutrophils, monocytes, colored by vaccine group). The left column shows scatter plots before normalization, the right column shows the data after normalization.

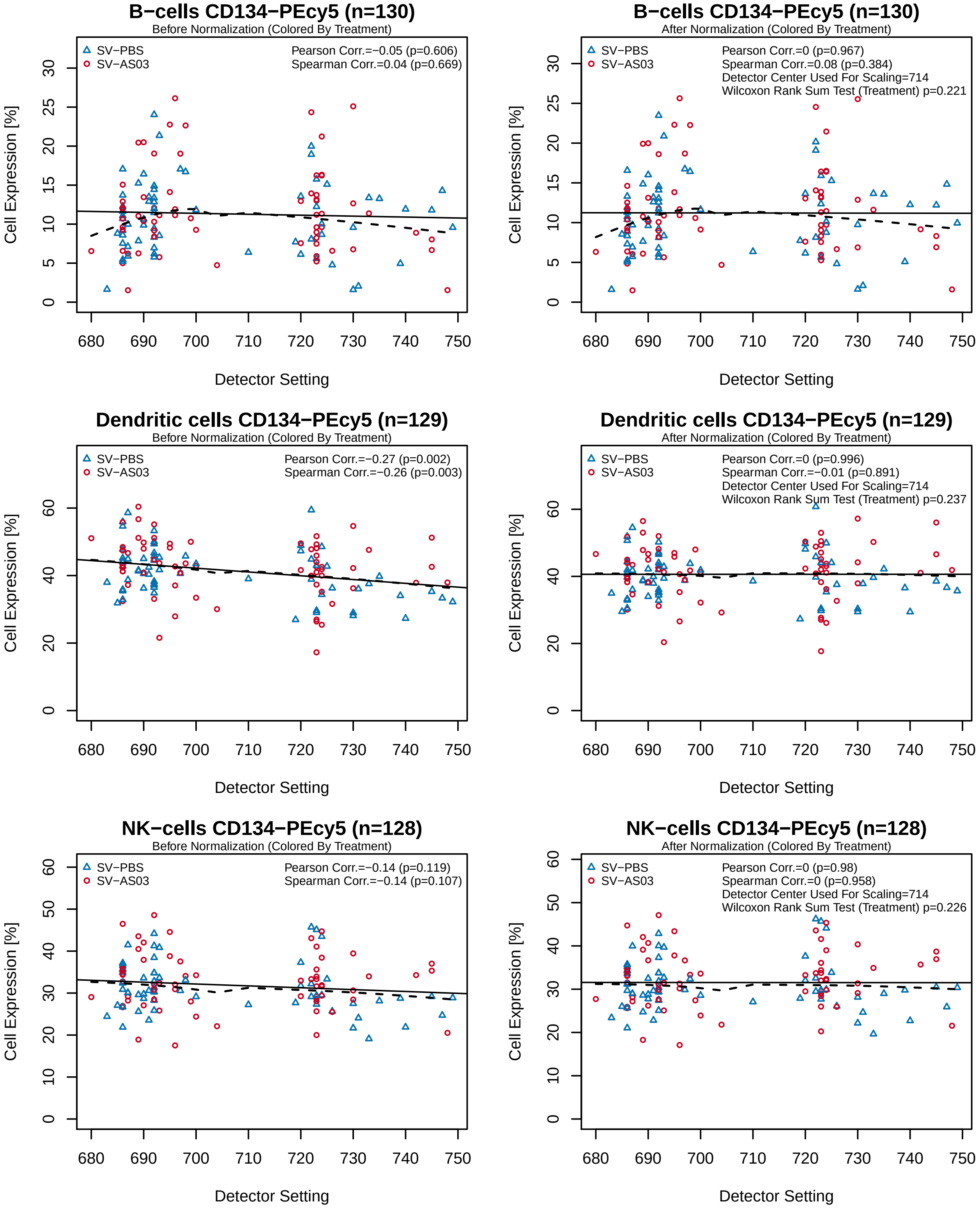

Figure A191: Scatterplots of percent cell activation by detector setting (FACS, CD134, B-cells, dendritic cells, NK-cells, colored by vaccine group). The left column shows scatter plots before normalization, the right column shows the data after normalization.

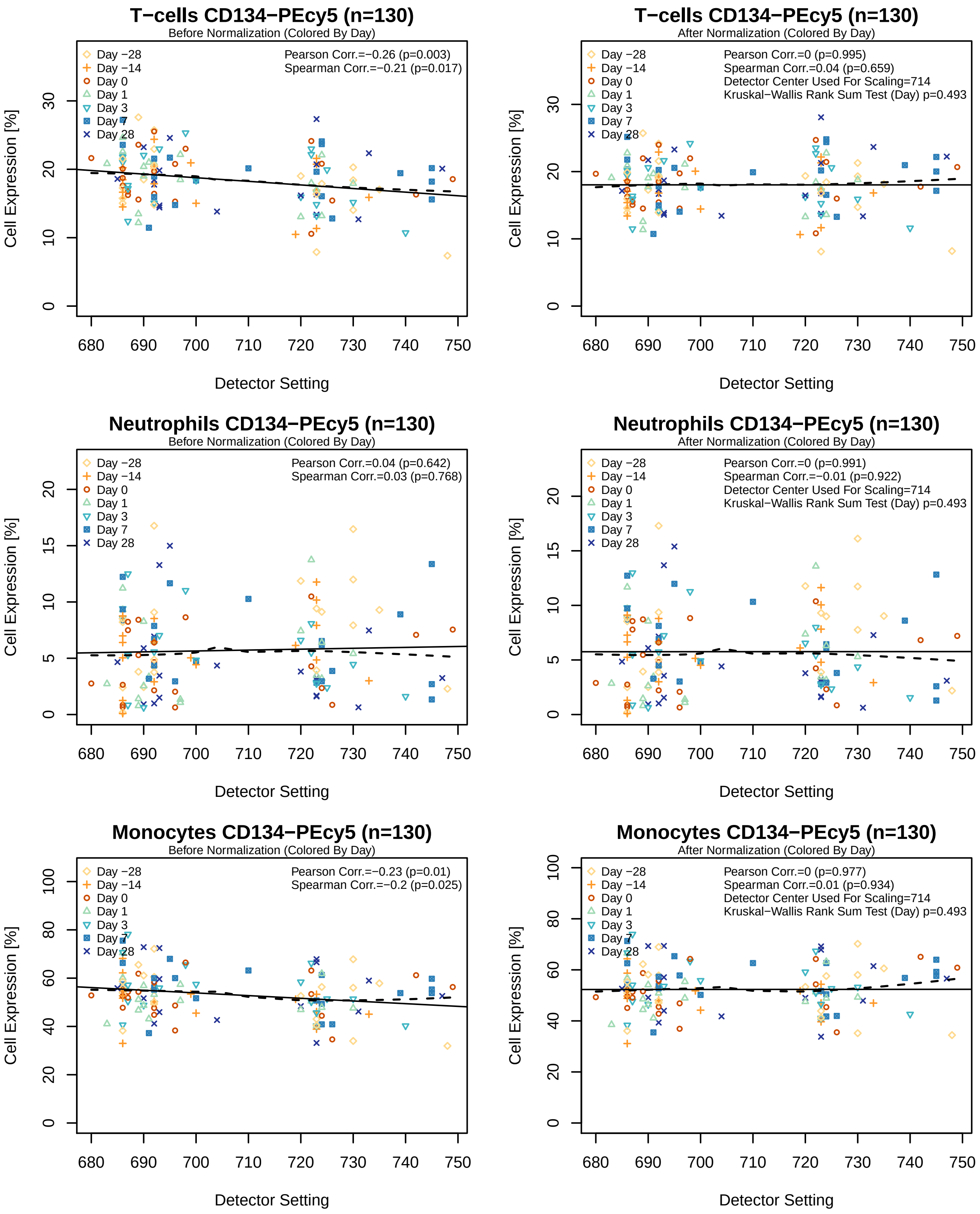

Figure A192: Scatterplots of percent cell activation by detector setting (FACS, CD134, T-cells, neutrophils, monocytes, colored by study visit day). The left column shows scatter plots before normalization, the right column shows the data after normalization.

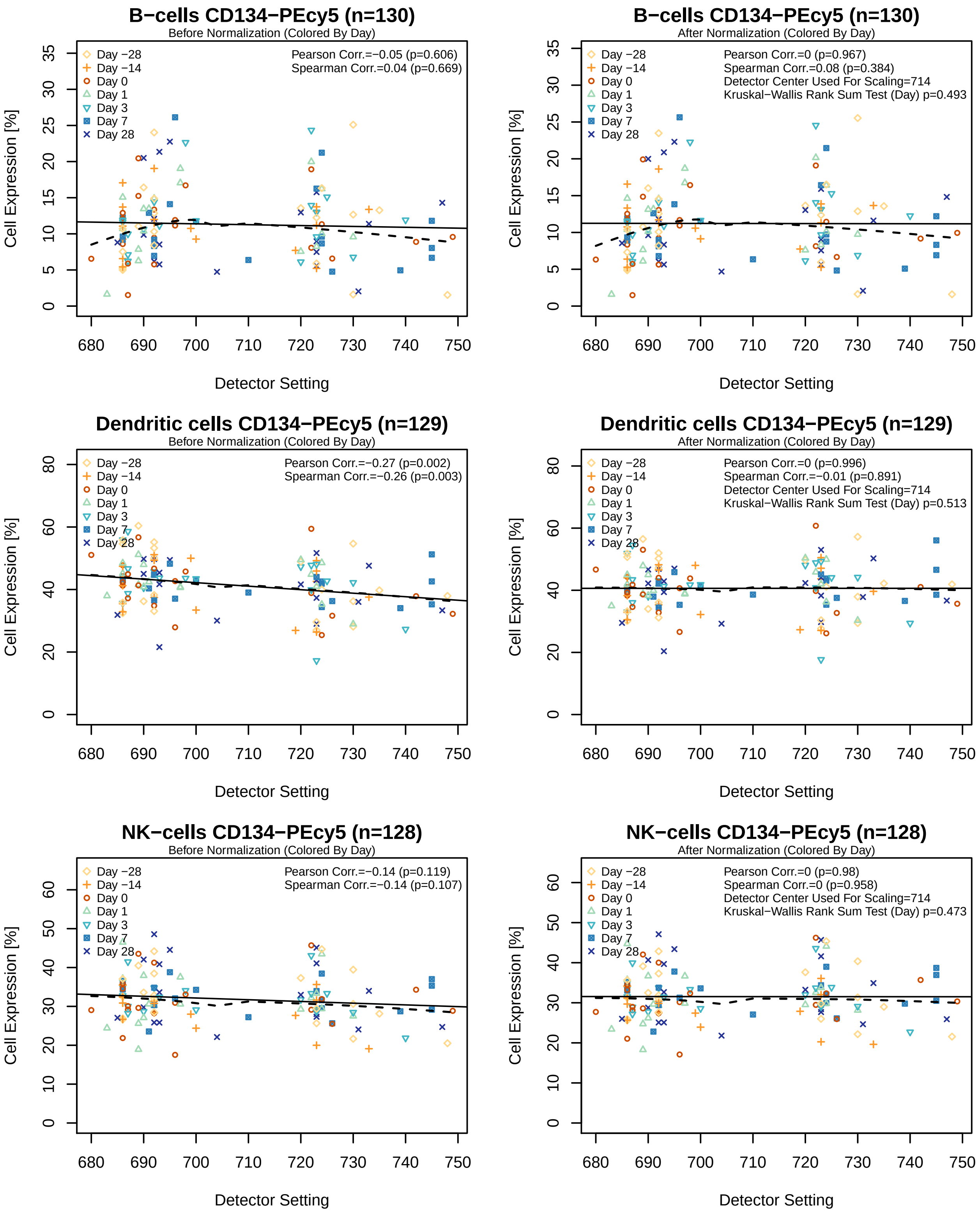

Figure A193: Scatterplots of percent cell activation by detector setting (FACS, CD134, B-cells, dendritic cells, NK-cells, colored by study visit day). The left column shows scatter plots before normalization, the right column shows the data after normalization.

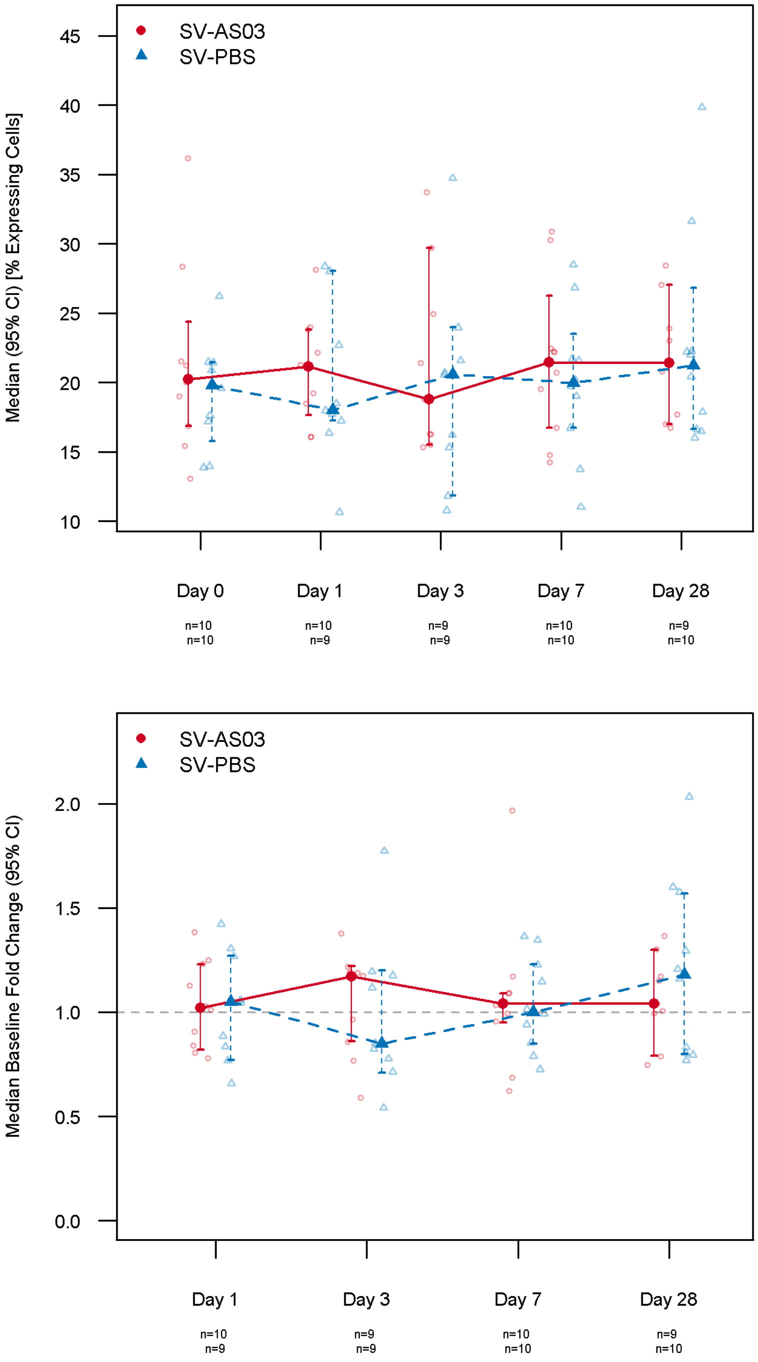

Figure A194: Median normalized cell activation plots for CD69 (FACS, B-cells)

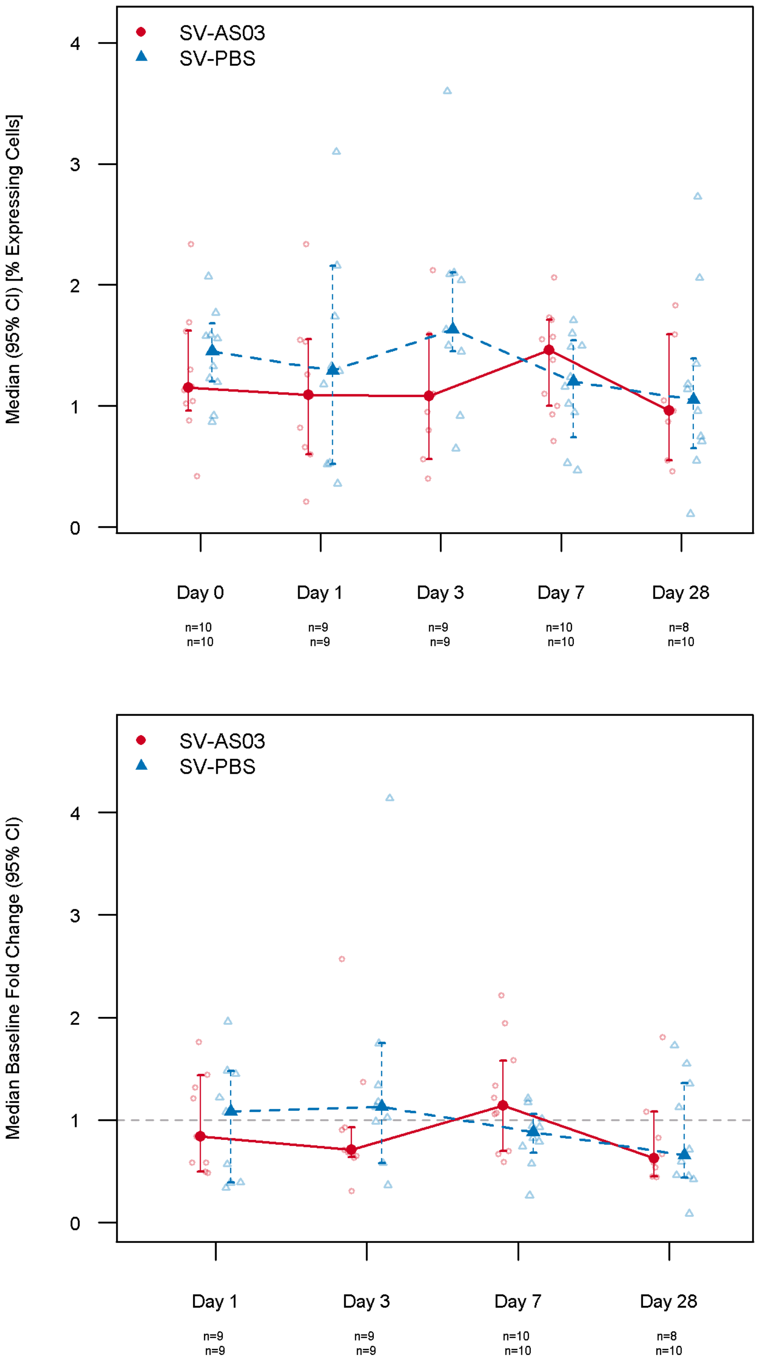

Figure A195: Median normalized cell activation plots for CD86 (FACS, B-cells)

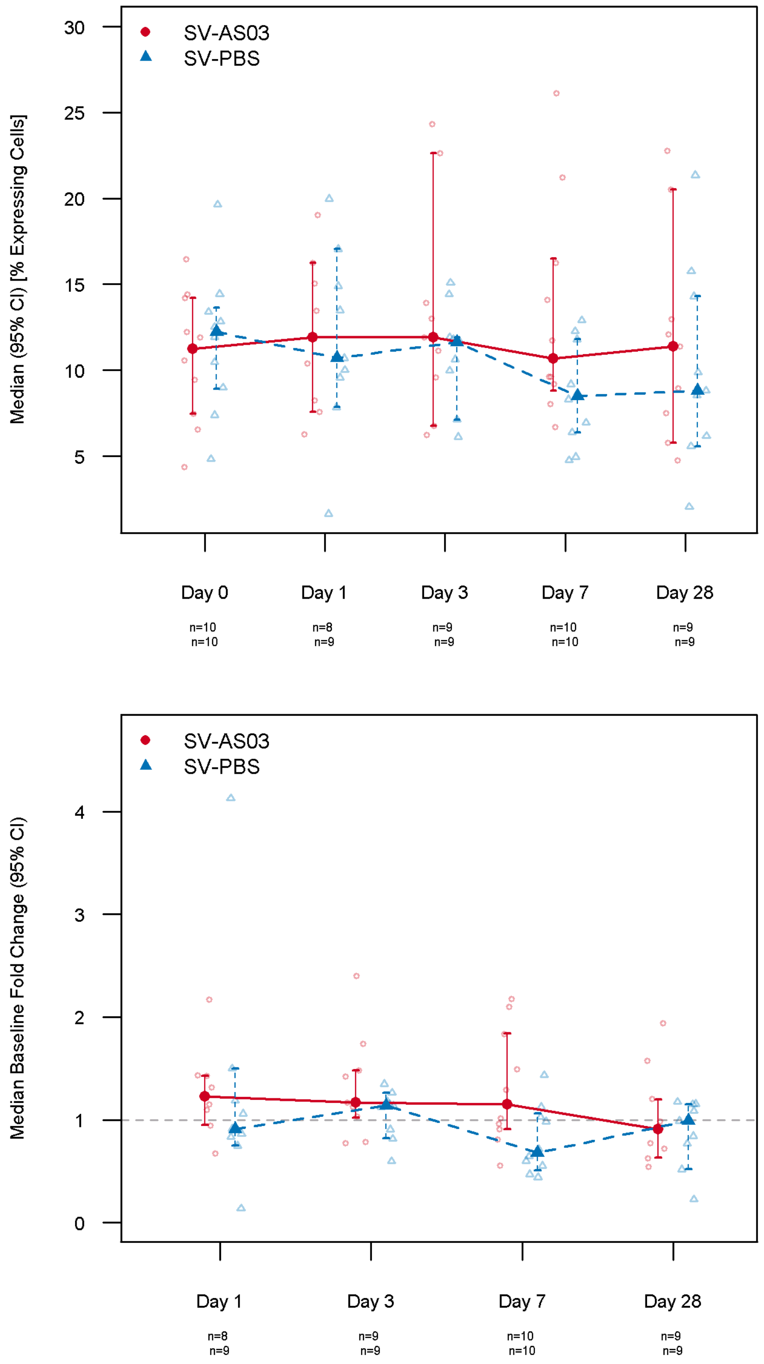

Figure A196: Median normalized cell activation plots for CD134 (FACS, B-cells)

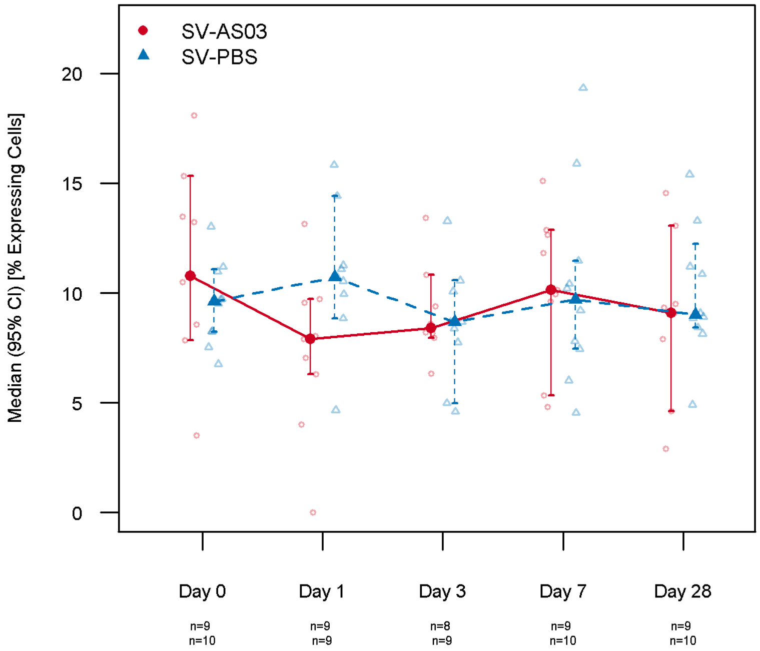

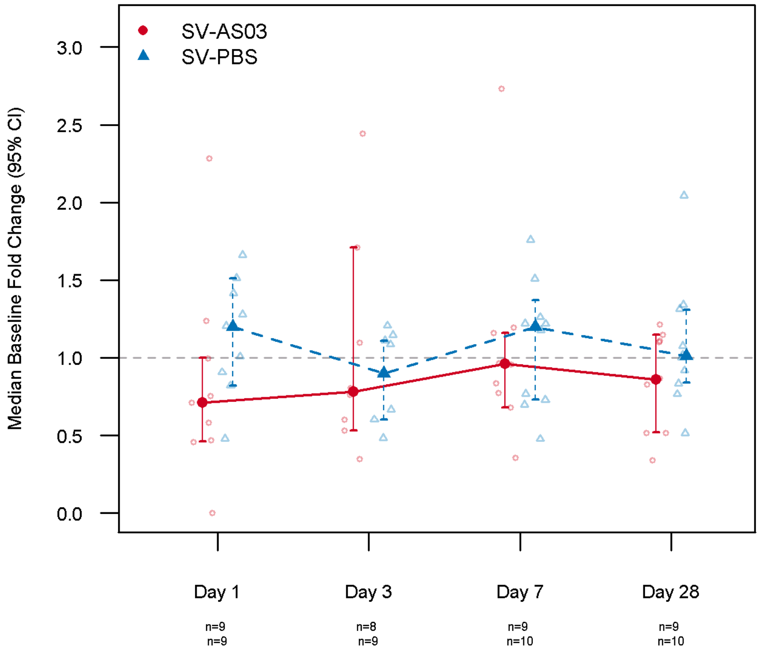

Figure A197: Median normalized cell activation plots for CD69 (FACS, Dendritic Cells)

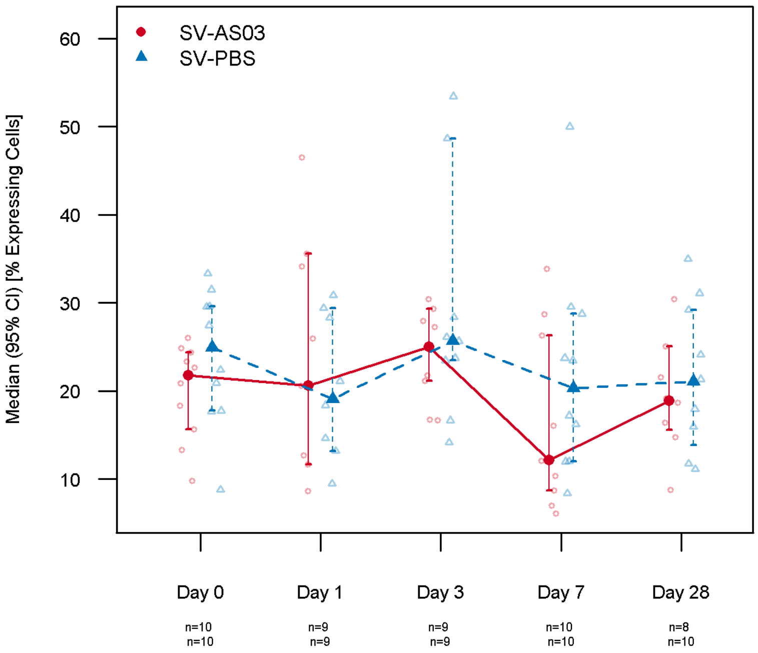

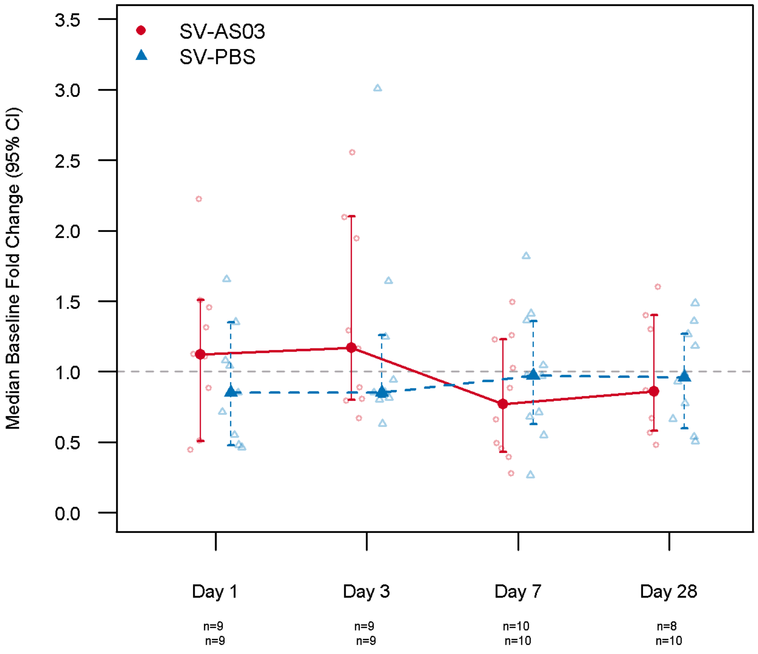

Figure A198: Median normalized cell activation plots for CD86 (FACS, Dendritic Cells)

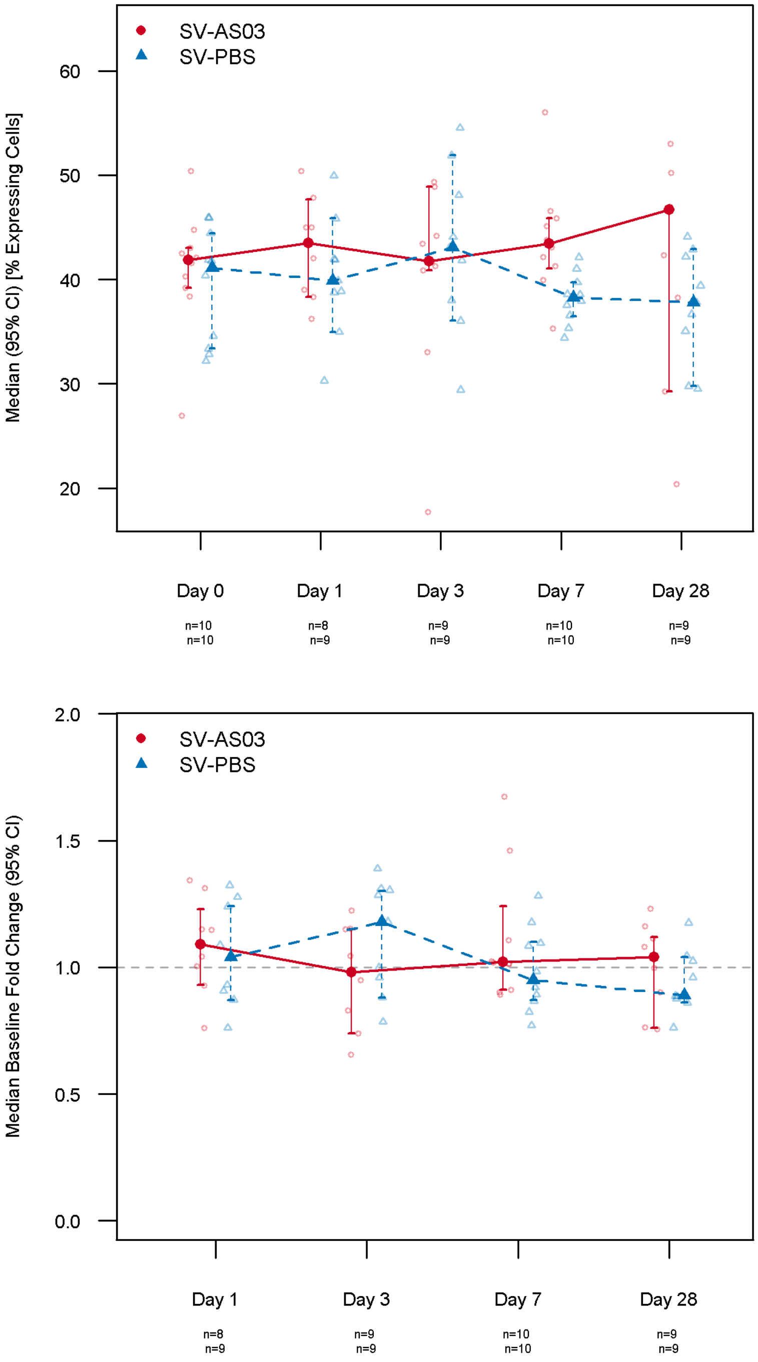

Figure A199: Median normalized cell activation plots for CD134 (FACS, Dendritic Cells)

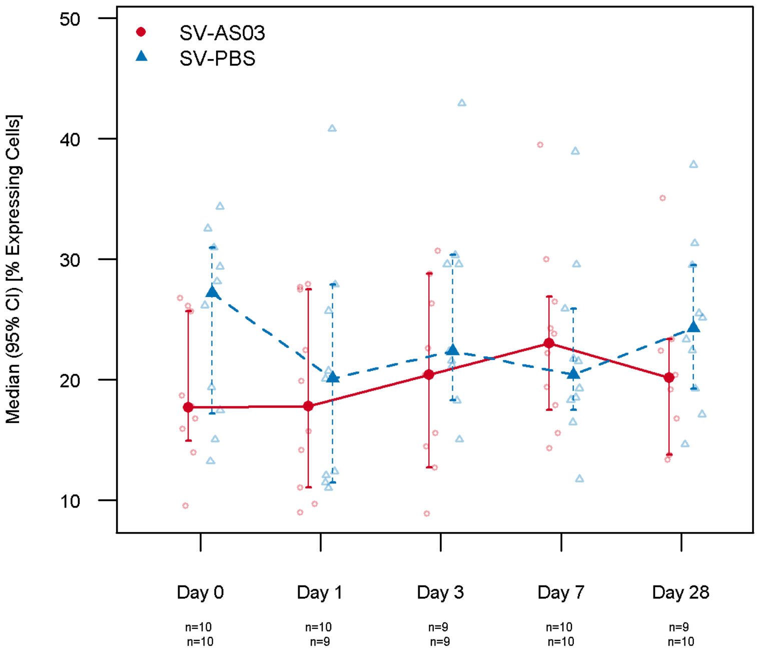

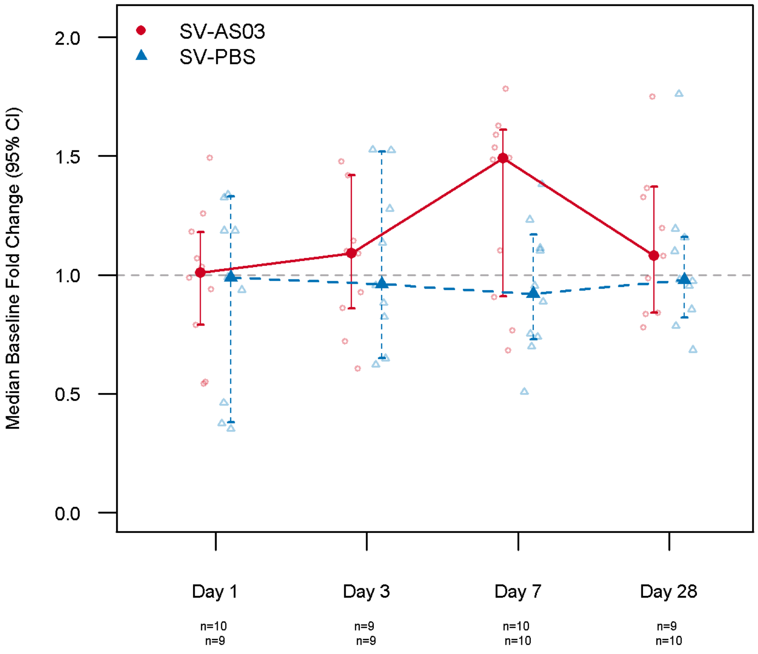

Figure A200: Median normalized cell activation plots for CD69 (FACS, Monocytes)

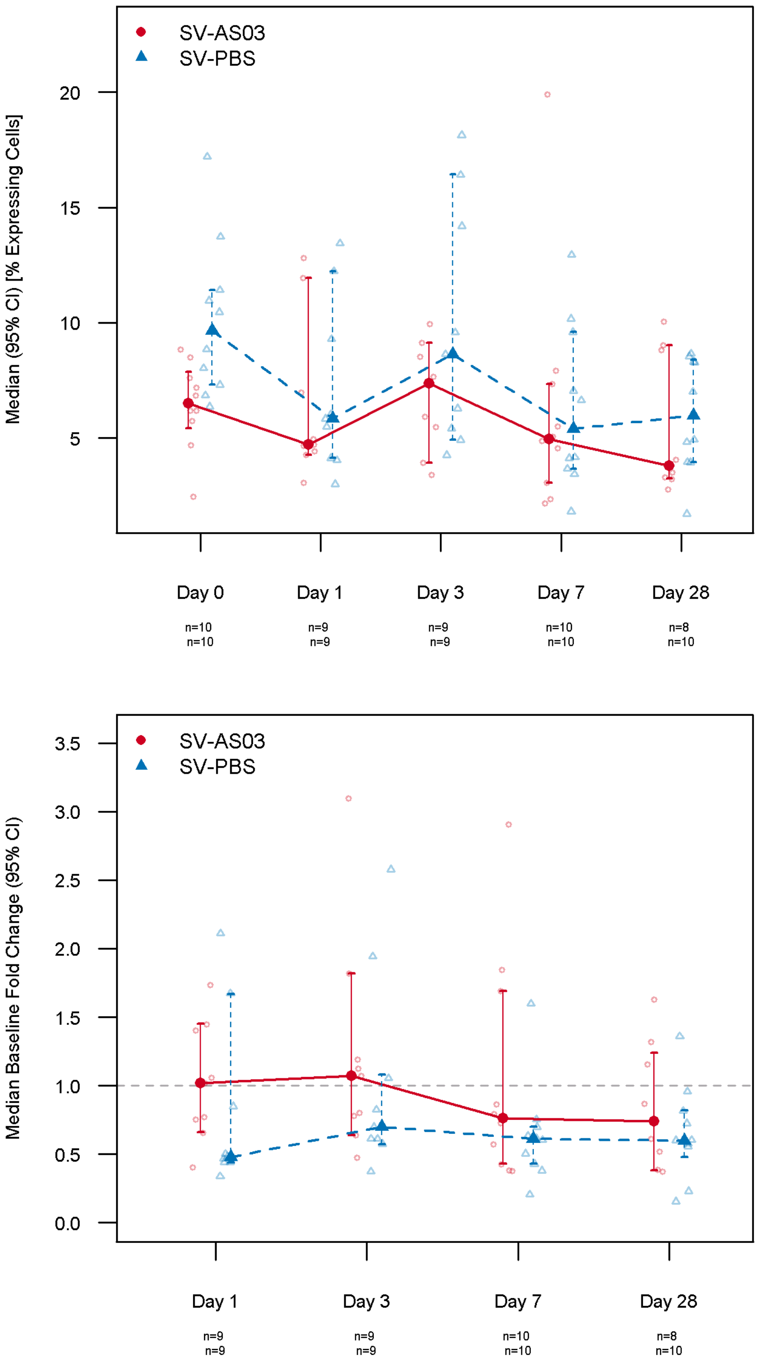

Figure A201: Median normalized cell activation plots for CD86 (FACS, Monocytes)

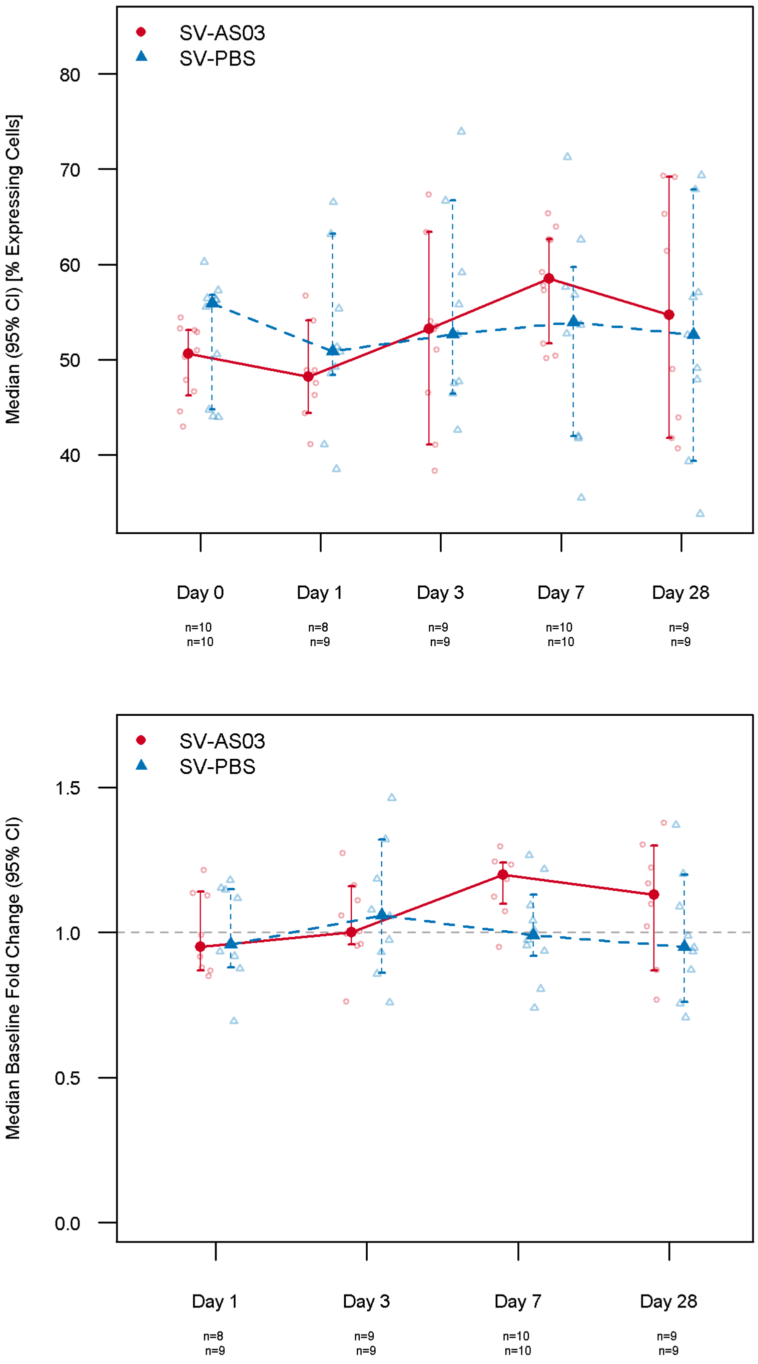

Figure A202: Median normalized cell activation plots for CD134 (FACS, Monocytes)

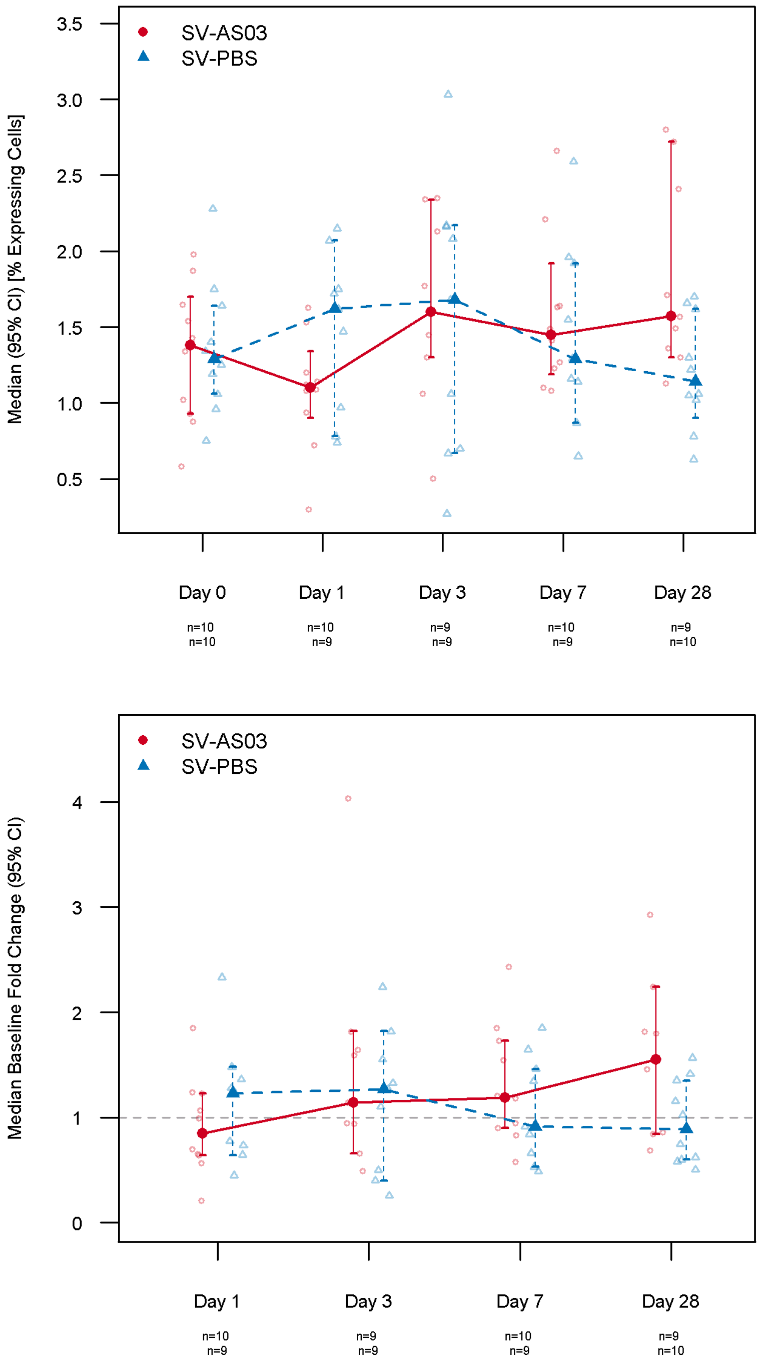

Figure A203: Median normalized cell activation plots for CD69 (FACS, Neutrophils)

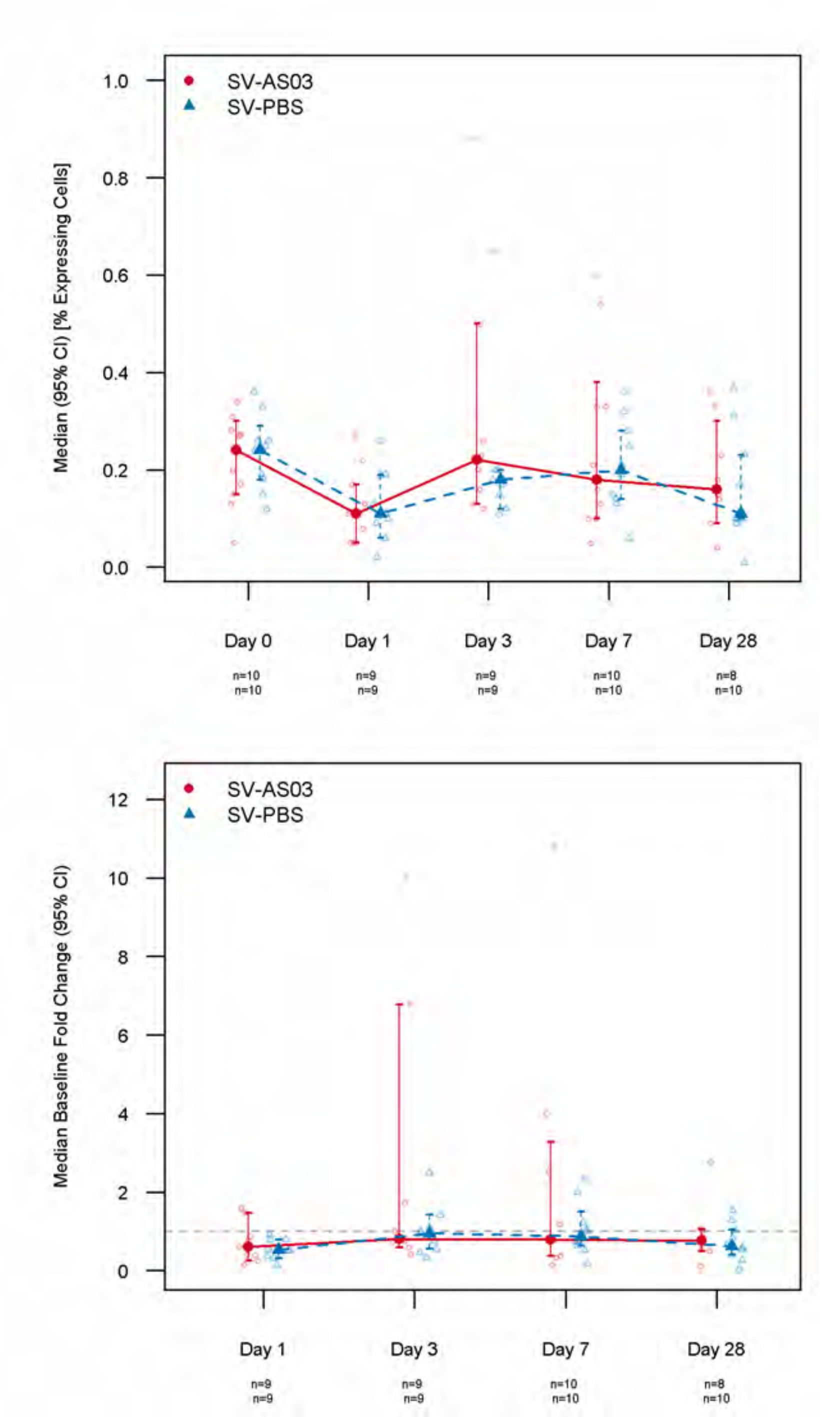

Figure A204: Median normalized cell activation plots for CD86 (FACS, Neutrophils)

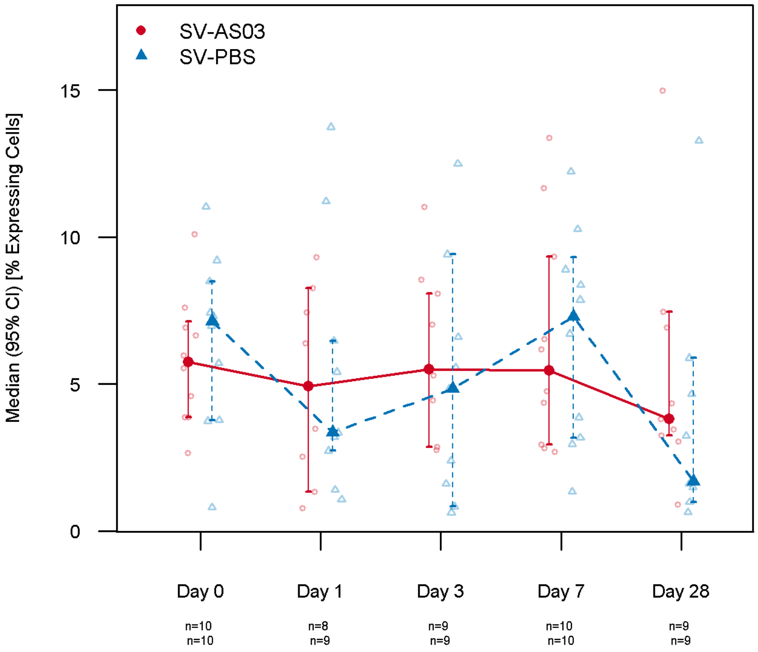

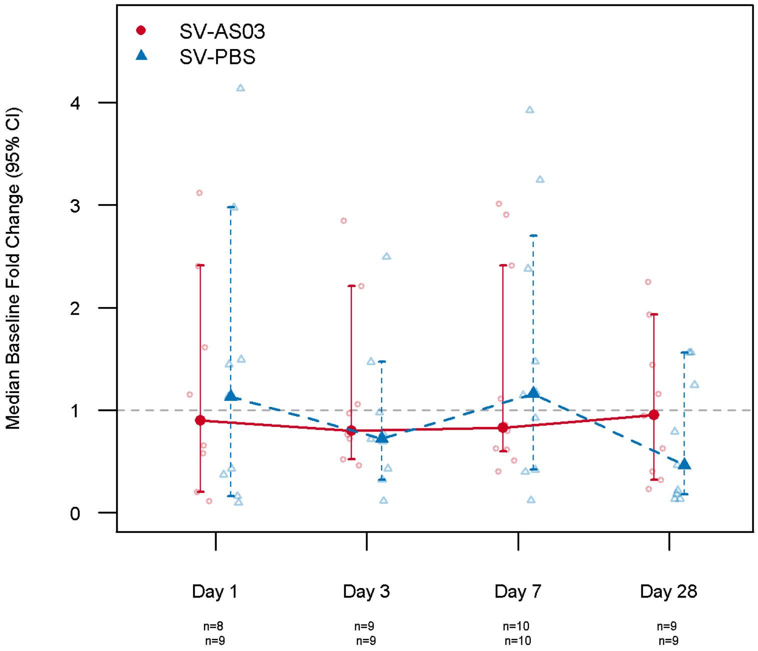

Figure A205: Median normalized cell activation plots for CD134 (FACS, Neutrophils)

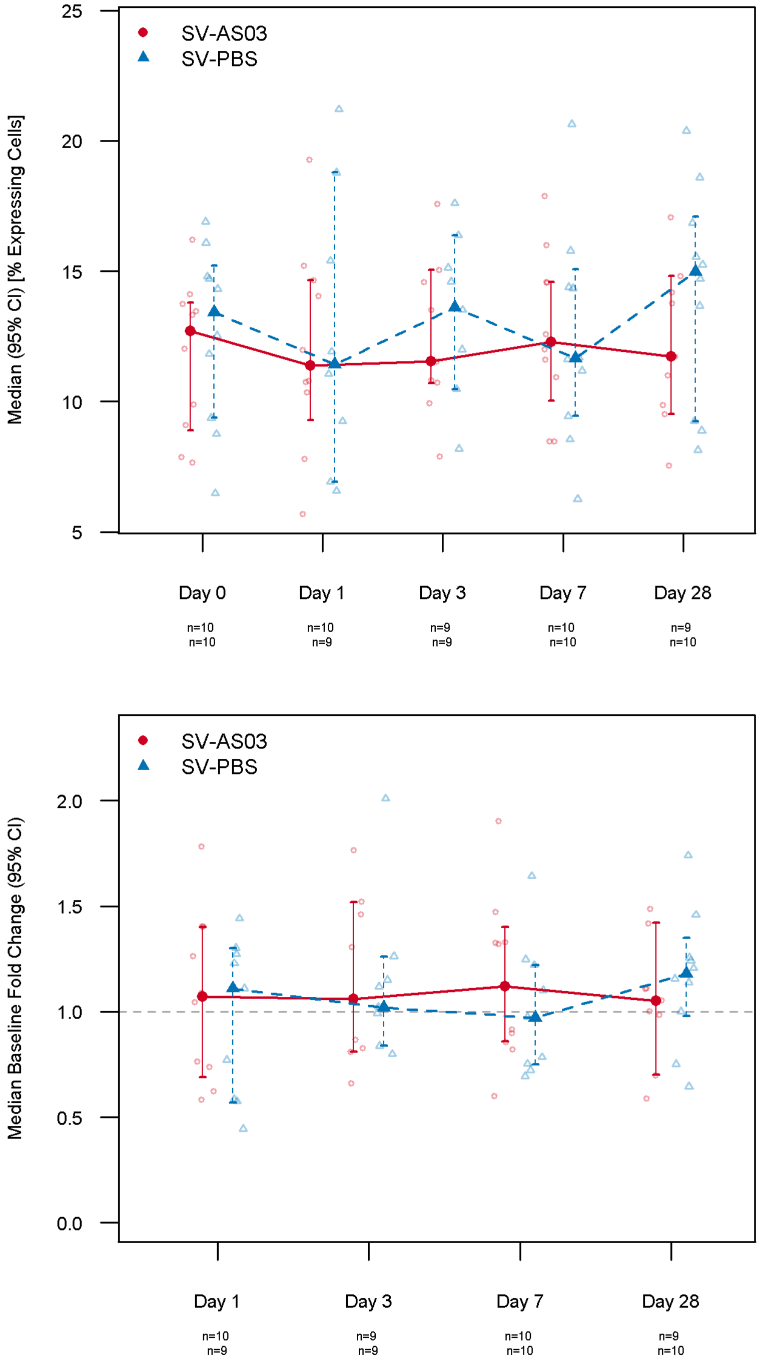

Figure A206: Median normalized cell activation plots for CD69 (FACS, NK-Cells)

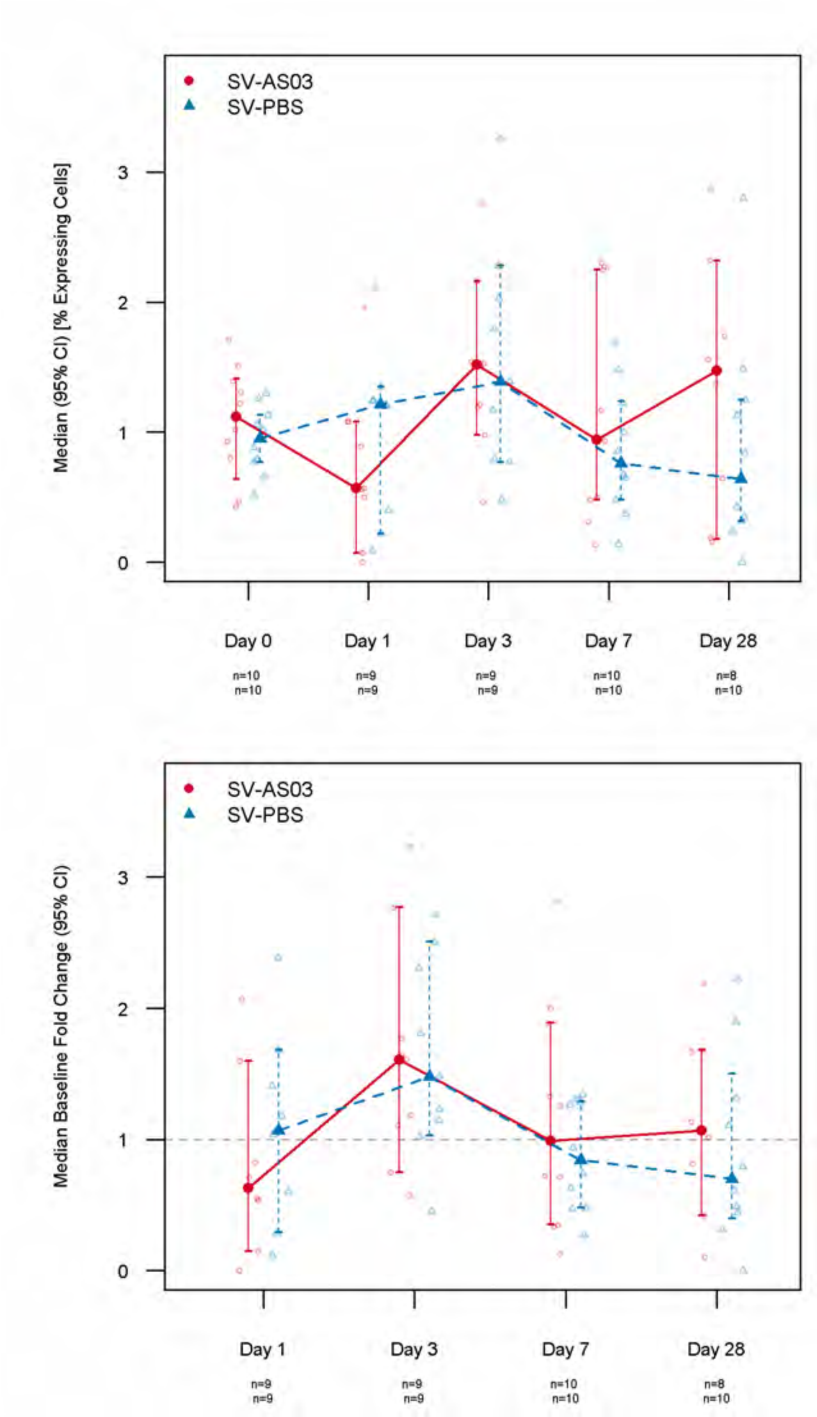

Figure A207: Median normalized cell activation plots for CD86 (FACS, NK-Cells)

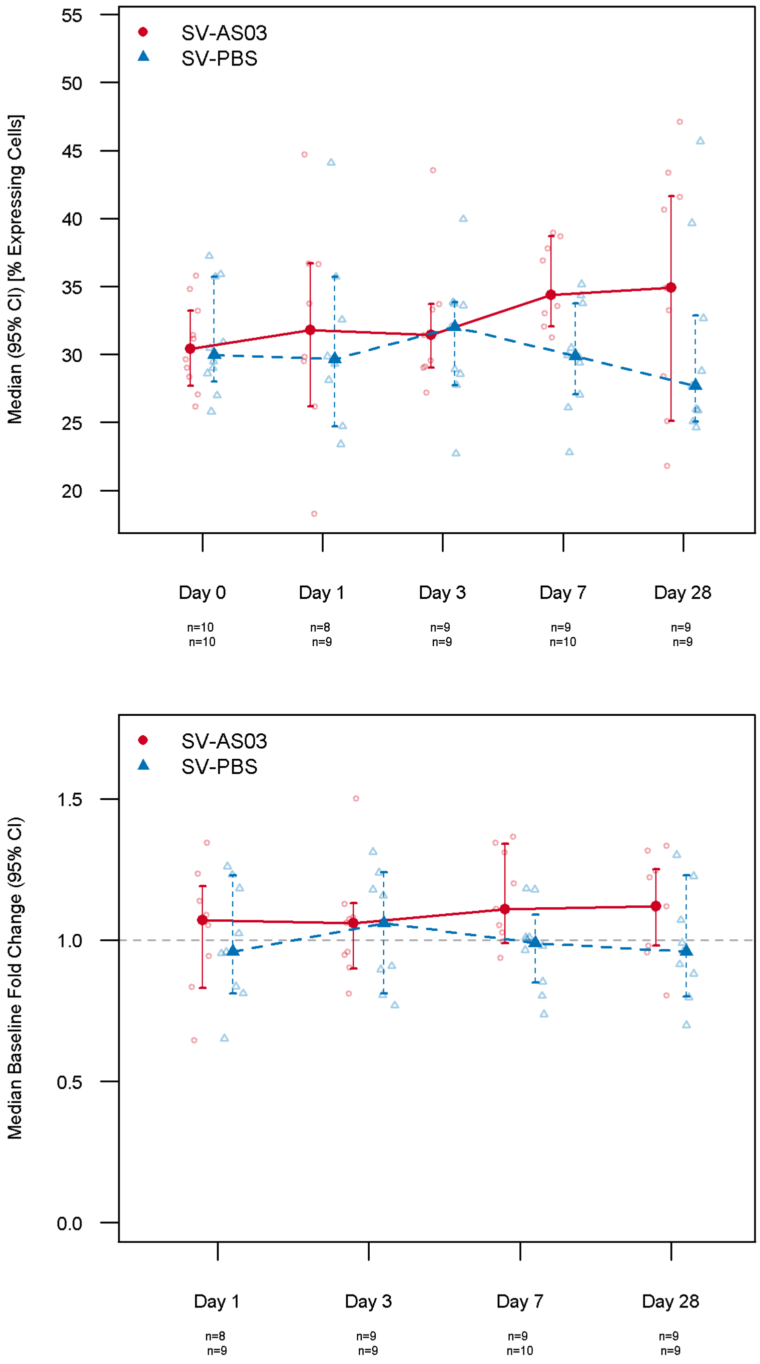

Figure A208: Median normalized cell activation plots for CD134 (FACS, NK-Cells)

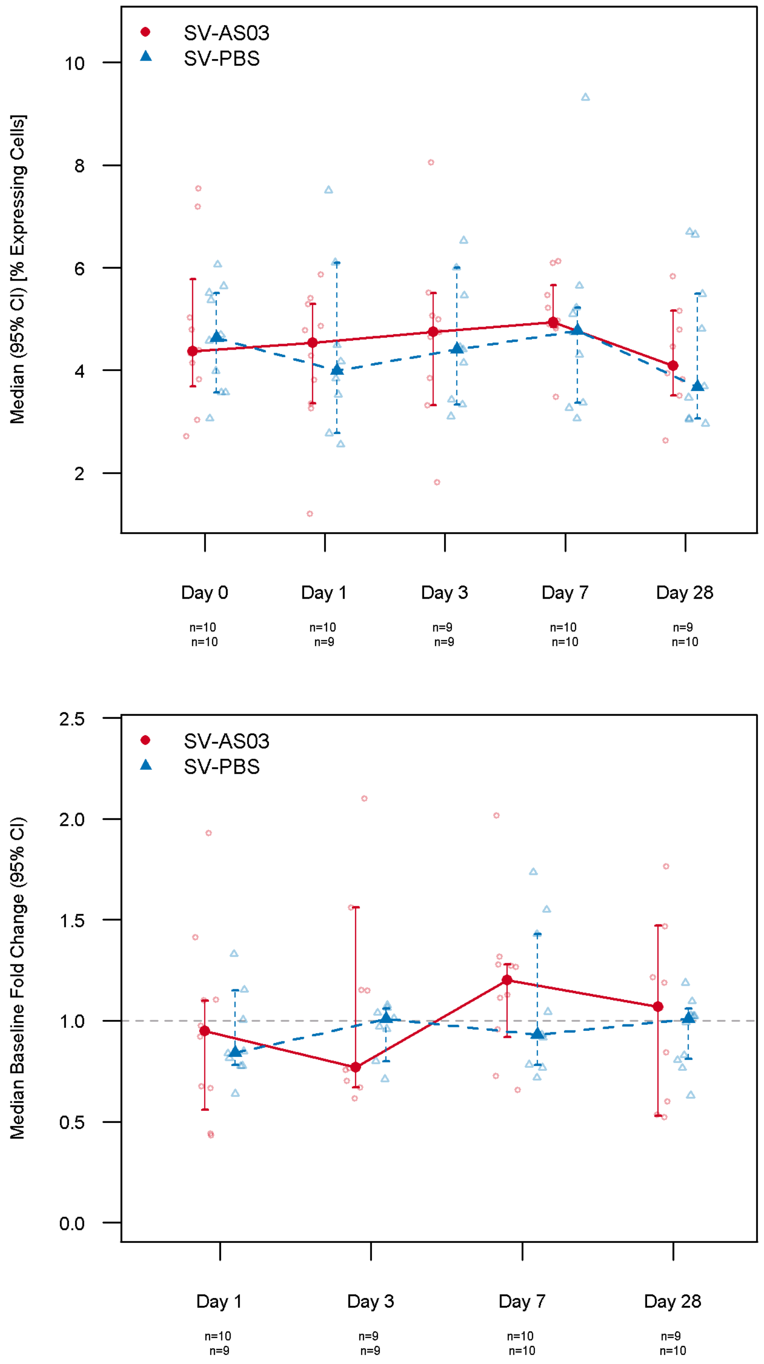

Figure A209: Median normalized cell activation plots for CD69 (FACS, T-Cells)

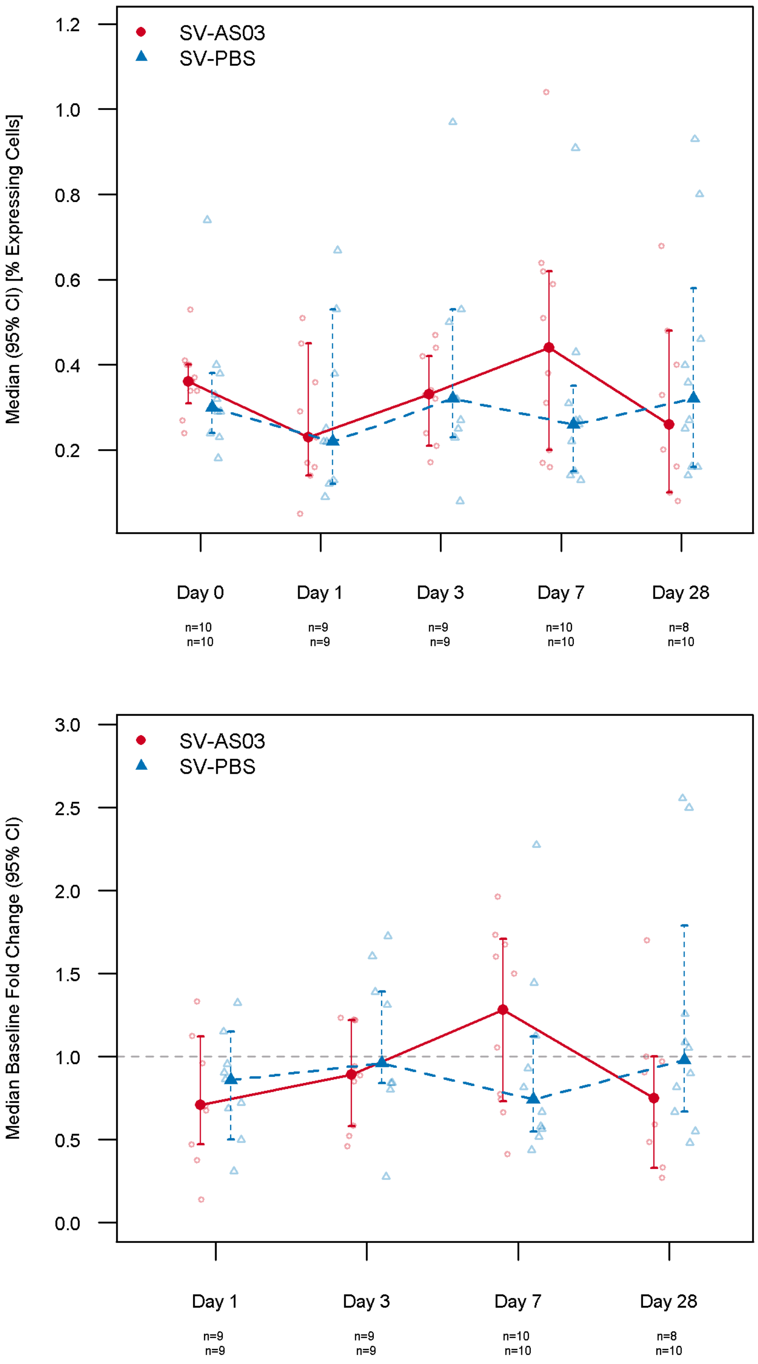

Figure A210: Median normalized cell activation plots for CD86 (FACS, T-Cells)

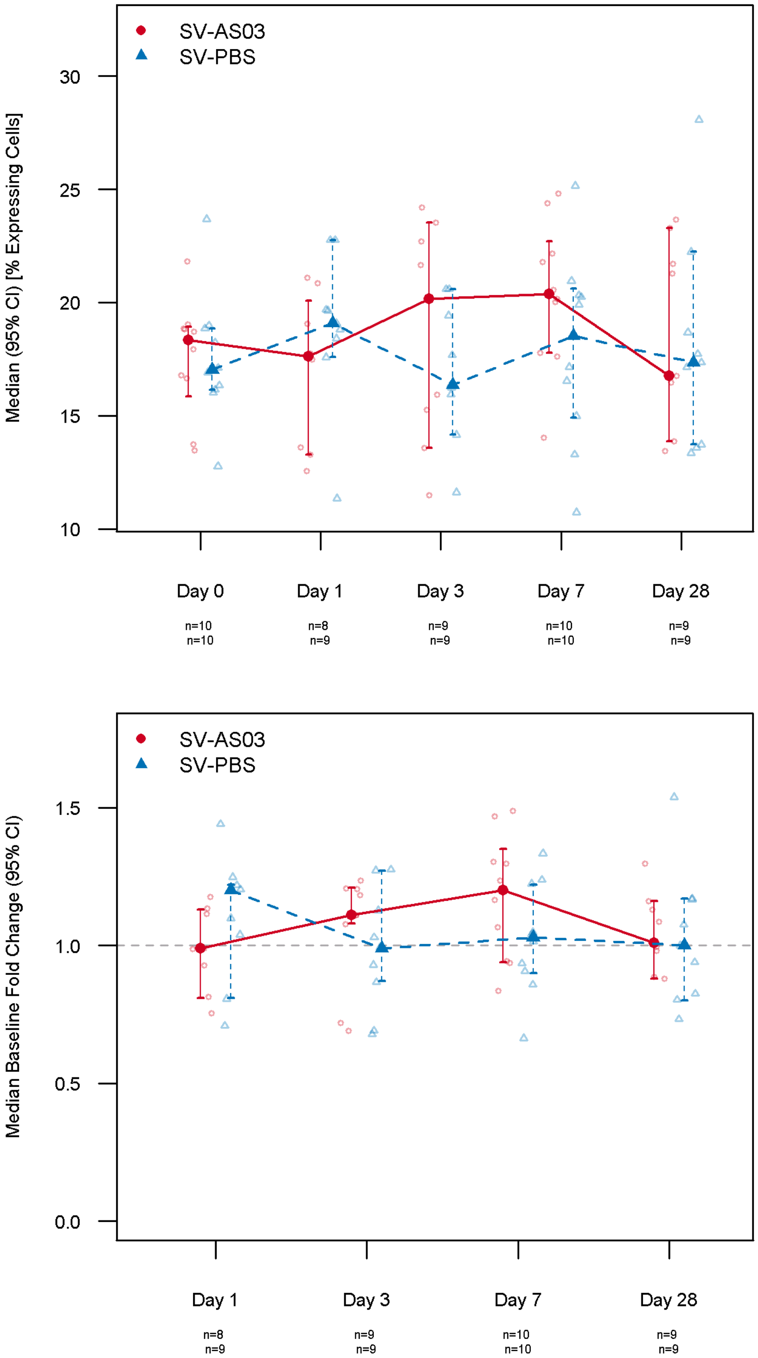

Figure A211: Median normalized cell activation plots for CD134 (FACS, T-Cells)

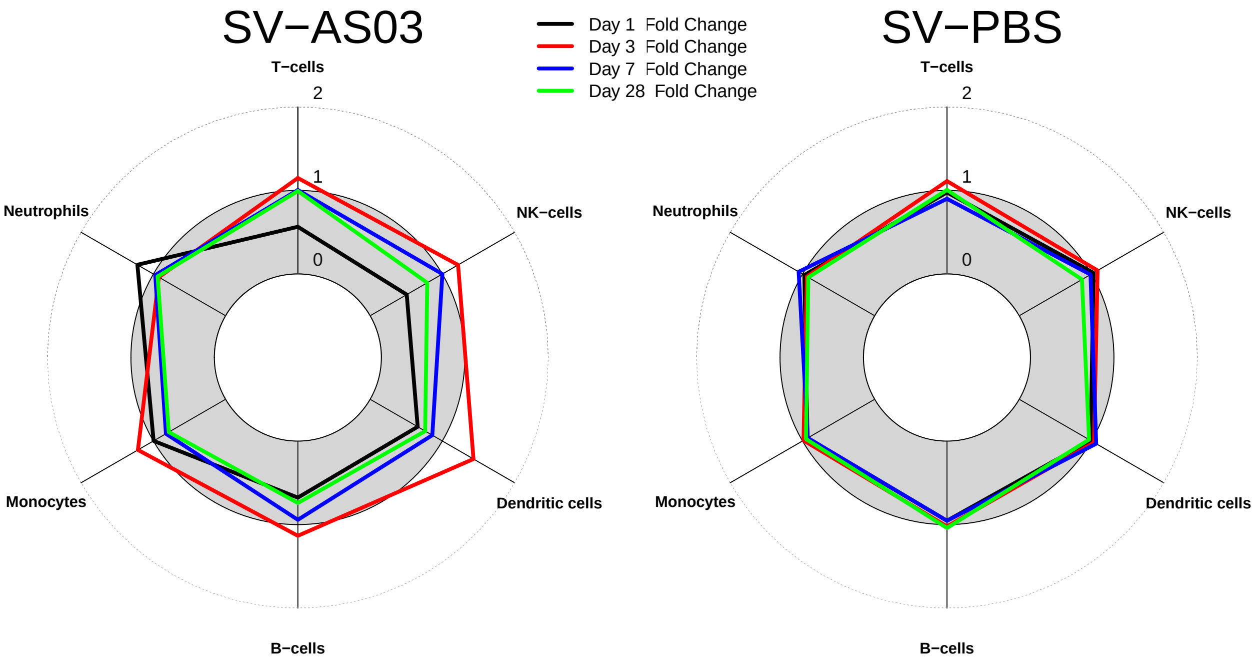

Figure A212: Spider plots of median fold change in the percentage of live cells for each immune cell type.

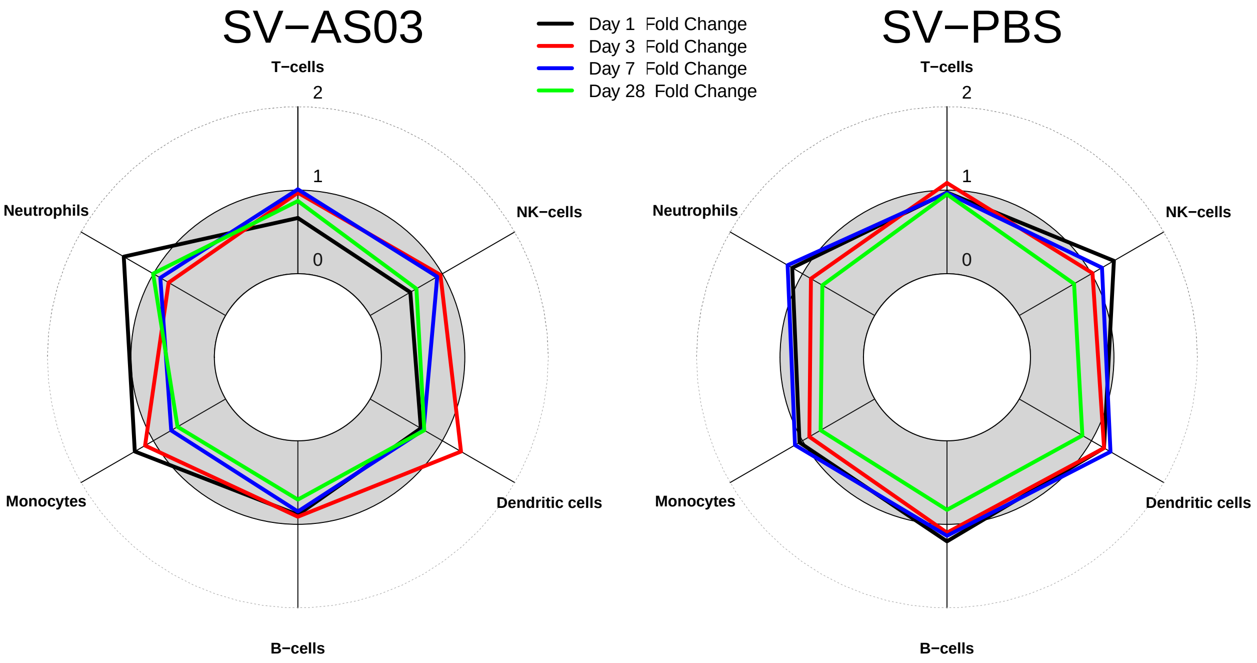

Figure A213: Spider plots of median fold change in the number of live cells per mL for each immune cell type.

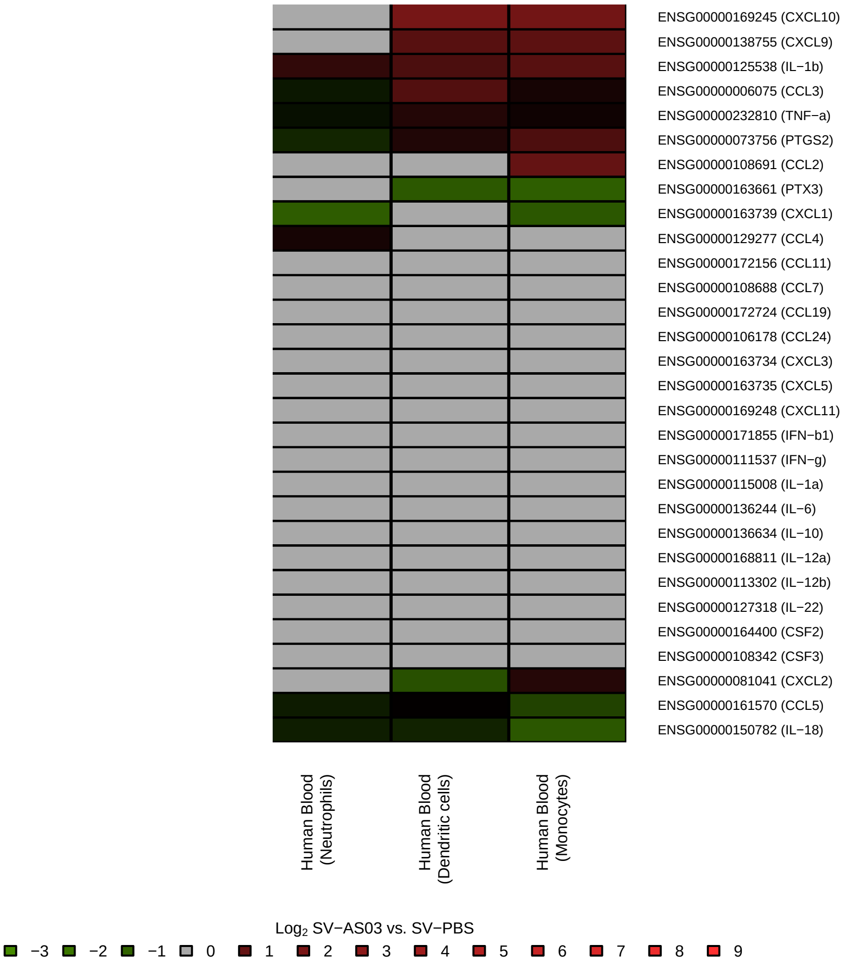

Figure A214: Morel et al. (2011) SV-AS03-responsive genes in mouse (RNA-Seq). Genes were mapped from mouse to human using MGI annotations (accessed November 2015). Cells are color-coded by fold change difference (LFCD) between SV-AS03 and SV-PBS vaccine groups. Cells highlighted in grey represent genes that did not meet the minimum expression level cut off.

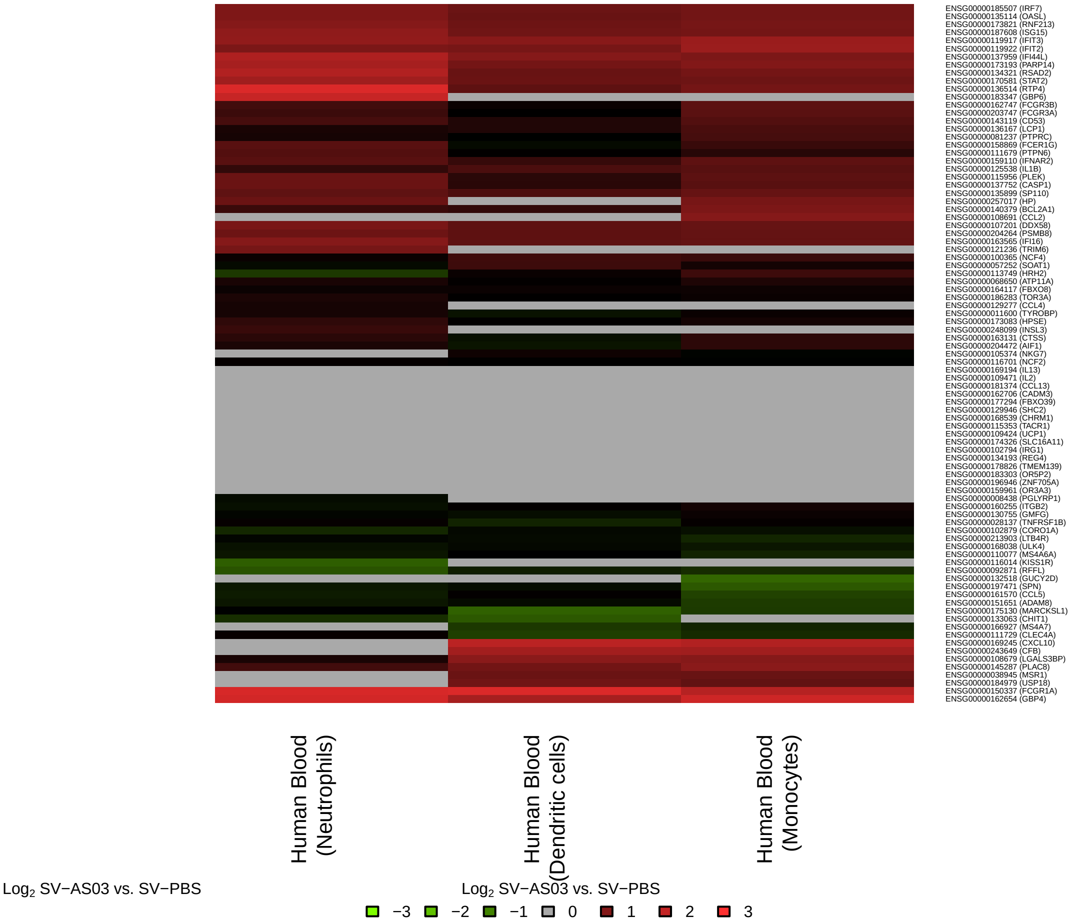

Figure A215: Mosca et al. (2008) MF59, CpG, MF59+CpG, and Alum-responsive genes in mouse (RNA-Seq). Adjuvants tested include MF59, CpG, MF59+CpG, and Alum. Genes were mapped from mouse to human using MGI annotations (accessed November 2015). Cells are color-coded by fold change difference (LFCD) between SV-AS03 and SV-PBS vaccine groups. Cells highlighted in grey represent genes that did not meet the minimum expression level cut off.

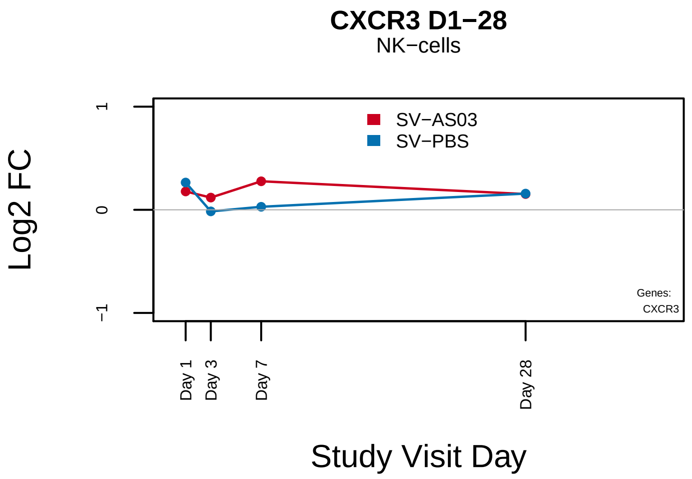

Figure A216: Time trends of baseline log fold change for CXCR3 (RNA-Seq, NK-Cells).

Tables

		All Subjects	SV-AS03	SV-PBS
		(N=20)	(N=10)	(N=10)
Gender – N(%)				
	Male	9 (45)	6 (60)	3 (30)
	Female	11 (55)	4 (40)	7 (70)
Ethnicity – N(%)				
	Non-Hispanic	20 (100)	10 (100)	10 (100)
	Hispanic	0	0	0
Race – N(%)				
	American Indian/Alaskan Native	0	0	0
	Asian	0	0	0
	Hawaiian/Pacific Islander	0	0	0
	Black/African American	0	0	0
	White	20 (100)	10 (100)	10 (100)
	Multi-Racial	0	0	0
	Other/Unknown	0	0	0
Age (years)				
	Mean (Standard Deviation)	28.3 (5.5)	27.4 (5.8)	29.3 (5.4)
	Median	27.6	26.1	29.1
	Minimum,Maximum	(19.3, 39.3)	(20.6, 37.8)	(19.3, 39.3)

Table A1: Demographic data by study group

SV-AS03

Reactogenicity		Vaccination 1				Vaccination 2			
		None N (%)	Mild N (%)	Moderate N (%)	Severe N (%)	None N (%)	Mild N (%)	Moderate N (%)	Severe N (%)
Systemic	Elevated Oral Temperature	10 (100)				10 (100)			
	Feverishness	9 (90)	1 (10)			10 (100)			
	Malaise	7 (70)	2 (20)	1 (10)		7 (70)	3 (30)		
	Myalgia	7 (70)	2 (20)	1 (10)		9 (90)	1 (10)		
	Headache	5 (50)	5 (50)			7 (70)	2 (20)	1 (10)	
	Nausea	10 (100)				10 (100)			
	Chills	10 (100)				10 (100)			
	Arthralgia	10 (100)				10 (100)			
	Shivering	10 (100)				10 (100)			
	Asthenia	8 (80)	1 (10)	1 (10)		8 (80)	2 (20)		
	Any Systemic Symptoms (Total)	4 (40)	5 (50)	1 (10)		5 (50)	4 (40)	1 (10)	
Local	Pain	1 (10)	7 (70)	2 (20)		2 (20)	8 (80)		
	Tenderness	2 (20)	7 (70)	1 (10)		4 (40)	6 (60)		
	Redness	10 (100)				9 (90)	1 (10)		
	Redness (Measurement grade)	10 (100)				9 (90)	1 (10)		
	Swelling	9 (90)		1 (10)		9 (90)	1 (10)		
	Swelling (Measurement grade)	10 (100)				10 (100)			
	Any Local Symptoms (Total)	1 (10)	7 (70)	2 (20)		2 (20)	8 (80)		
Any	Any Symptoms	1 (10)	7 (70)	2 (20)		2 (20)	7 (70)	1 (10)	

Table A2: Maximum severity of solicited adverse events by vaccination (SV-AS03)

SV-PBS

Reactogenicity		Vaccination 1				Vaccination 2			
		None N (%)	Mild N (%)	Moderate N (%)	Severe N (%)	None N (%)	Mild N (%)	Moderate N (%)	Severe N (%)
Systemic	Elevated Oral Temperature	10 (100)				10 (100)			
	Feverishness	9 (90)	1 (10)			9 (90)	1 (10)		
	Malaise	7 (70)	2 (20)	1 (10)		9 (90)	1 (10)		
	Myalgia	9 (90)		1 (10)		9 (90)	1 (10)		
	Headache	9 (90)	1 (10)			9 (90)	1 (10)		
	Nausea	9 (90)	1 (10)			10 (100)			
	Chills	10 (100)				10 (100)			
	Arthralgia	9 (90)	1 (10)			9 (90)	1 (10)		
	Shivering	10 (100)				10 (100)			
	Asthenia	9 (90)	1 (10)			10 (100)			
	Any Systemic Symptoms (Total)	6 (60)	2 (20)	2 (20)		7 (70)	3 (30)		
Local	Pain	8 (80)	2 (20)			9 (90)	1 (10)		
	Tenderness	7 (70)	3 (30)			10 (100)			
	Redness	10 (100)				10 (100)			
	Redness (Measurement grade)	10 (100)				10 (100)			
	Swelling	10 (100)				10 (100)			
	Swelling (Measurement grade)	10 (100)				10 (100)			
	Any Local Symptoms (Total)	6 (60)	4 (40)			9 (90)	1 (10)		
Any	Any Symptoms	5 (50)	3 (30)	2 (20)		7 (70)	3 (30)		

Table A3: Maximum severity of solicited adverse events by vaccination (SV-PBS)

Study Arm	Study Visit	N	GMT (95% CI)	SD	Number of Responders (Titer≥1:40)	Proportion of Responder (95% CI)] (titer≥1:40)	Number of Responders (Titer≥1:40 and 4-fold change from baseline)	Proportion of Responders (95% CI) (Titer≥1:40 and 4 fold change from baseline)
All Subjects	Pre-Vaccination	20	5.0 (-)	-	0	0.00 (0.00, 0.17)	-	-
All Subjects	Day 1 Post Vac 1	20	5.0 (-)	-	0	0.00 (0.00, 0.17)	0	0.00 (0.00, 0.17)
All Subjects	Day 3 Post Vac 1	20	5.0 (-)	-	0	0.00 (0.00, 0.17)	0	0.00 (0.00, 0.17)
All Subjects	Day 7 Post Vac 1	20	5.0 (-)	-	0	0.00 (0.00, 0.17)	0	0.00 (0.00, 0.17)
All Subjects	Day 28 Post Vac 1	20	6.8 (5.0, 9.3)	0.66	1	0.05 (0.00, 0.25)	1	0.05 (0.00, 0.25)
All Subjects	Day 28 Post Vac 2	20	31.4 (14.4, 68.4)	1.66	9	0.45 (0.23, 0.68)	9	0.45 (0.23, 0.68)
SV-AS03	Pre-Vaccination	10	5.0 (-)	-	0	0.00 (0.00, 0.31)	-	-
SV-AS03	Day 1 Post Vac 1	10	5.0 (-)	-	0	0.00 (0.00, 0.31)	0	0.00 (0.00, 0.31)
SV-AS03	Day 3 Post Vac 1	10	5.0 (-)	-	0	0.00 (0.00, 0.31)	0	0.00 (0.00, 0.31)
SV-AS03	Day 7 Post Vac 1	10	5.0 (-)	-	0	0.00 (0.00, 0.31)	0	0.00 (0.00, 0.31)
SV-AS03	Day 28 Post Vac 1	10	9.0 (4.9, 16.7)	0.86	1	0.10 (0.00, 0.45)	1	0.10 (0.00, 0.45)
SV-AS03	Day 28 Post Vac 2	10	130.0 (63.1, 267.6)	1.01	9	0.90 (0.55, 1.00)	9	0.90 (0.55, 1.00)
SV-PBS	Pre-Vaccination	10	5.0 (-)	-	0	0.00 (0.00, 0.31)	-	-
SV-PBS	Day 1 Post Vac 1	10	5.0 (-)	-	0	0.00 (0.00, 0.31)	0	0.00 (0.00, 0.31)
SV-PBS	Day 3 Post Vac 1	10	5.0 (-)	-	0	0.00 (0.00, 0.31)	0	0.00 (0.00, 0.31)
SV-PBS	Day 7 Post Vac 1	10	5.0 (-)	-	0	0.00 (0.00, 0.31)	0	0.00 (0.00, 0.31)
SV-PBS	Day 28 Post Vac 1	10	5.2 (4.8, 5.6)	0.11	0	0.00 (0.00, 0.31)	0	0.00 (0.00, 0.31)
SV-PBS	Day 28 Post Vac 2	10	7.6 (5.0, 11.5)	0.58	0	0.00 (0.00, 0.31)	0	0.00 (0.00, 0.31)

Table A4: Intend-to-treat population. Summary of hemagglutination inhibition titer/responses against A/Indonesia/05/2005 by study arm and visit (Immunogenicity). SD: standard deviation on the log scale.

Study Arm	Study Visit	N	GMT (95% CI)	SD	Number of Responders (Titer≥1:40)	Proportion of Responder (95% CI)] (titer≥1:40)	Number of Responders (Titer≥1:40 and 4-fold change from baseline)	Proportion of Responders (95% CI) (Titer≥1:40 and 4 fold change from baseline)
All Subjects	Pre-Vaccination	20	5.0 (-)	-	0	0.00 (0.00, 0.17)	-	-
All Subjects	Day 1 Post Vac 1	20	5.0 (-)	-	0	0.00 (0.00, 0.17)	0	0.00 (0.00, 0.17)
All Subjects	Day 3 Post Vac 1	20	5.0 (-)	-	0	0.00 (0.00, 0.17)	0	0.00 (0.00, 0.17)
All Subjects	Day 7 Post Vac 1	20	5.0 (-)	-	0	0.00 (0.00, 0.17)	0	0.00 (0.00, 0.17)
All Subjects	Day 28 Post Vac 1	19	6.8 (4.9, 9.5)	0.68	1	0.05 (0.00, 0.26)	1	0.05 (0.00, 0.26)
All Subjects	Day 28 Post Vac 2	19	29.3 (13.0, 66.0)	1.68	8	0.42 (0.20, 0.67)	8	0.42 (0.20, 0.67)
SV-AS03	Pre-Vaccination	10	5.0 (-)	-	0	0.00 (0.00, 0.31)	-	-
SV-AS03	Day 1 Post Vac 1	10	5.0 (-)	-	0	0.00 (0.00, 0.31)	0	0.00 (0.00, 0.31)
SV-AS03	Day 3 Post Vac 1	10	5.0 (-)	-	0	0.00 (0.00, 0.31)	0	0.00 (0.00, 0.31)
SV-AS03	Day 7 Post Vac 1	10	5.0 (-)	-	0	0.00 (0.00, 0.31)	0	0.00 (0.00, 0.31)
SV-AS03	Day 28 Post Vac 1	9	9.3 (4.6, 18.7)	0.91	1	0.11 (0.00, 0.48)	1	0.11 (0.00, 0.48)
SV-AS03	Day 28 Post Vac 2	9	132.0 (58.0, 300.3)	1.07	8	0.89 (0.52, 1.00)	8	0.89 (0.52, 1.00)
SV-PBS	Pre-Vaccination	10	5.0 (-)	-	0	0.00 (0.00, 0.31)	-	-
SV-PBS	Day 1 Post Vac 1	10	5.0 (-)	-	0	0.00 (0.00, 0.31)	0	0.00 (0.00, 0.31)
SV-PBS	Day 3 Post Vac 1	10	5.0 (-)	-	0	0.00 (0.00, 0.31)	0	0.00 (0.00, 0.31)
SV-PBS	Day 7 Post Vac 1	10	5.0 (-)	-	0	0.00 (0.00, 0.31)	0	0.00 (0.00, 0.31)
SV-PBS	Day 28 Post Vac 1	10	5.2 (4.8, 5.6)	0.11	0	0.00 (0.00, 0.31)	0	0.00 (0.00, 0.31)
SV-PBS	Day 28 Post Vac 2	10	7.6 (5.0, 11.5)	0.58	0	0.00 (0.00, 0.31)	0	0.00 (0.00, 0.31)

Table A5: Per-protocol population. Summary of hemagglutination inhibition titer/responses against A/Indonesia/05/2005 by study arm and visit (Immunogenicity). SD: standard deviation on the log scale.

Study Arm	Study Visit	N	GMT (95% CI)	SD	Number of Responders (Titer≥1:40)	Proportion of Responder (95% CI)] (titer≥1:40)	Number of Responders (Titer≥1:40 and 4-fold change from baseline)	Proportion of Responders (95% CI) (Titer≥1:40 and 4 fold change from baseline)
All Subjects	Pre-Vaccination	20	6.5 (5.3, 8.0)	0.45	0	0.00 (0.00, 0.17)	-	-
All Subjects	Day 1 Post Vac 1	20	6.3 (5.0, 7.8)	0.47	0	0.00 (0.00, 0.17)	0	0.00 (0.00, 0.17)
All Subjects	Day 3 Post Vac 1	20	5.7 (5.0, 6.6)	0.28	0	0.00 (0.00, 0.17)	0	0.00 (0.00, 0.17)
All Subjects	Day 7 Post Vac 1	20	8.1 (6.3, 10.5)	0.54	1	0.05 (0.00, 0.25)	1	0.05 (0.00, 0.25)
All Subjects	Day 28 Post Vac 1	19	16.7 (11.3, 24.6)	0.80	4	0.21 (0.06, 0.46)	4	0.21 (0.06, 0.46)
All Subjects	Day 28 Post Vac 2	19	89.3 (37.1, 214.9)	1.81	12	0.63 (0.38, 0.84)	12	0.63 (0.38, 0.84)
SV-AS03	Pre-Vaccination	10	5.5 (4.7, 6.6)	0.23	0	0.00 (0.00, 0.31)	-	-
SV-AS03	Day 1 Post Vac 1	10	5.7 (4.5, 7.3)	0.34	0	0.00 (0.00, 0.31)	0	0.00 (0.00, 0.31)
SV-AS03	Day 3 Post Vac 1	10	5.4 (4.8, 5.9)	0.15	0	0.00 (0.00, 0.31)	0	0.00 (0.00, 0.31)
SV-AS03	Day 7 Post Vac 1	10	8.4 (6.4, 11.0)	0.37	0	0.00 (0.00, 0.31)	0	0.00 (0.00, 0.31)
SV-AS03	Day 28 Post Vac 1	9	27.2 (15.6, 47.5)	0.75	3	0.33 (0.07, 0.70)	3	0.33 (0.07, 0.70)
SV-AS03	Day 28 Post Vac 2	9	452.5 (197.0, 1039.7)	1.02	9	1.00 (0.66, 1.00)	9	1.00 (0.66, 1.00)
SV-PBS	Pre-Vaccination	10	7.6 (5.1, 11.3)	0.56	0	0.00 (0.00, 0.31)	-	-
SV-PBS	Day 1 Post Vac 1	10	6.8 (4.5, 10.3)	0.58	0	0.00 (0.00, 0.31)	0	0.00 (0.00, 0.31)
SV-PBS	Day 3 Post Vac 1	10	6.2 (4.7, 8.0)	0.37	0	0.00 (0.00, 0.31)	0	0.00 (0.00, 0.31)
SV-PBS	Day 7 Post Vac 1	10	7.8 (4.8, 12.9)	0.69	1	0.10 (0.00, 0.45)	1	0.10 (0.00, 0.45)
SV-PBS	Day 28 Post Vac 1	10	10.7 (6.8, 16.8)	0.63	1	0.10 (0.00, 0.45)	1	0.10 (0.00, 0.45)
SV-PBS	Day 28 Post Vac 2	10	20.7 (11.9, 36.0)	0.77	3	0.30 (0.07, 0.65)	3	0.30 (0.07, 0.65)

Table A6: Intend-to-treat population. Summary of neutralizing antibody titer/response against A/Indonesia/05/2005 by study arm and visit (Immunogenicity). SD: standard deviation on the log scale.

Study Arm	Study Visit	N	GMT (95% CI)	SD	Number of Responders (Titer≥1:40)	Proportion of Responder (95% CI)] (titer≥1:40)	Number of Responders (Titer≥1:40 and 4-fold change from baseline)	Proportion of Responders (95% CI) (Titer≥1:40 and 4 fold change from baseline)
All Subjects	Pre-Vaccination	20	6.5 (5.3, 8.0)	0.45	0	0.00 (0.00, 0.17)	-	-
All Subjects	Day 1 Post Vac 1	20	6.3 (5.0, 7.8)	0.47	0	0.00 (0.00, 0.17)	0	0.00 (0.00, 0.17)
All Subjects	Day 3 Post Vac 1	20	5.7 (5.0, 6.6)	0.28	0	0.00 (0.00, 0.17)	0	0.00 (0.00, 0.17)
All Subjects	Day 7 Post Vac 1	20	8.1 (6.3, 10.5)	0.54	1	0.05 (0.00, 0.25)	1	0.05 (0.00, 0.25)
All Subjects	Day 28 Post Vac 1	19	16.7 (11.3, 24.6)	0.81	4	0.21 (0.06, 0.46)	4	0.21 (0.06, 0.46)
All Subjects	Day 28 Post Vac 2	19	89.3 (37.1, 214.9)	1.82	12	0.63 (0.38, 0.84)	12	0.63 (0.38, 0.84)
SV-AS03	Pre-Vaccination	10	5.5 (4.7, 6.6)	0.23	0	0.00 (0.00, 0.31)	-	-
SV-AS03	Day 1 Post Vac 1	10	5.7 (4.5, 7.3)	0.34	0	0.00 (0.00, 0.31)	0	0.00 (0.00, 0.31)
SV-AS03	Day 3 Post Vac 1	10	5.4 (4.8, 5.9)	0.15	0	0.00 (0.00, 0.31)	0	0.00 (0.00, 0.31)
SV-AS03	Day 7 Post Vac 1	10	8.4 (6.4, 11.0)	0.37	0	0.00 (0.00, 0.31)	0	0.00 (0.00, 0.31)
SV-AS03	Day 28 Post Vac 1	9	27.2 (15.6, 47.5)	0.72	3	0.33 (0.07, 0.70)	3	0.33 (0.07, 0.70)
SV-AS03	Day 28 Post Vac 2	9	452.5 (197.0, 1039.7)	1.08	9	1.00 (0.66, 1.00)	9	1.00 (0.66, 1.00)
SV-PBS	Pre-Vaccination	10	7.6 (5.1, 11.3)	0.56	0	0.00 (0.00, 0.31)	-	-
SV-PBS	Day 1 Post Vac 1	10	6.8 (4.5, 10.3)	0.58	0	0.00 (0.00, 0.31)	0	0.00 (0.00, 0.31)
SV-PBS	Day 3 Post Vac 1	10	6.2 (4.7, 8.0)	0.37	0	0.00 (0.00, 0.31)	0	0.00 (0.00, 0.31)
SV-PBS	Day 7 Post Vac 1	10	7.8 (4.8, 12.9)	0.69	1	0.10 (0.00, 0.45)	1	0.10 (0.00, 0.45)
SV-PBS	Day 28 Post Vac 1	10	10.7 (6.8, 16.8)	0.63	1	0.10 (0.00, 0.45)	1	0.10 (0.00, 0.45)
SV-PBS	Day 28 Post Vac 2	10	20.7 (11.9, 36.0)	0.77	3	0.30 (0.07, 0.65)	3	0.30 (0.07, 0.65)

Table A7: Per-protocol population. Summary of neutralizing antibody titer/response against A/Indonesia/05/2005 by study arm and visit (Immunogenicity). SD: standard deviation on the log scale.

Cytokine/Chemokine	LOD
Chemokine (C-C motif) ligand 5 (RANTES)	0.002 pg/mL
Monocyte chemoattractant protein-1 (MCP-1)	1.3 pg/mL
Interferon gamma-induced protein 10 (IP-10)	0.5 pg/mL
Interleukin 8 (IL-8)	69.9 fg/mL
Eotaxin-1	0.8 pg/mL
Interleukin-1 alpha (IL-1α)	1.0 pg/mL
Interleukin 12 p70 (IL-12p70)	12.6 fg/mL
Interleukin-10 (IL-10)	13.7 fg/mL
Tumor necrosis factors (TNF)	67.3 fg/mL
Lymphotoxin-alpha (LT-α)	0.2 pg/mL
Macrophage Inflammatory Protein (MIP-1α)	0.2 pg/mL
Interleukin 6 (IL-6)	68.4 fg/mL
Interleukin-1 beta (IL-1β)	48.4 fg/mL
Interferon gamma (IFNγ)	14.84 fg/mL

Table A8: Cytokine and chemokine limits of detection.

Category/Chromosome	#Genes
Mt_rRNA	2
Mt_tRNA	22
Mt_tRNA_pseudogene	580
rRNA	530
rRNA_pseudogene	179
tRNA_pseudogene	128
X	2326
Y	509
Total	4185

Table A9: Number of excluded genes by category/chromosome (RNA-Seq).

	Day 1	Day 3	Day 7	Day 28	Day 1-28
All Cells	13425	13406	13427	13437	13589
B-cells	14111	14145	14149	14081	14427
Dendritic cells	13057	13060	13054	13016	13279
Monocytes	12863	12878	12858	12889	13101
Neutrophils	10745	10765	10780	10655	11014
NK-cells	14048	14042	14040	14148	14349
T-cells	14236	14223	14283	14295	14511

Table A10: Number of genes that passed the low expression cut off (RNA-Seq).

	Min	Q1	Median	Mean	Q3	Max	SD	MAD
Total Mapped Reads [10^6]	11.11	49.61	53.71	56.20	59.91	199.50	12.83	7.41
Uniquely Mapped Reads [10^6]	9.43	41.08	44.96	46.89	50.03	162.81	10.77	6.42
Uniquely Mapped Reads [%]	72.70	81.70	84.00	83.41	85.00	87.90	2.29	2.67
Paired Reads (Mapped to Different Chromosomes)	0.00	0.00	0.00	0.00	0.00	4.00	0.14	0.00
Counted Fragments [10^6]	0.01	13.82	16.18	16.65	18.62	59.93	4.37	3.56
Median GC [%]	48.00	50.00	50.00	50.74	52.00	54.00	1.15	0.00
Mean GC [%]	47.26	49.70	50.36	50.32	50.92	53.23	0.83	0.90
FACS Cells [10^6]	0.04	1.50	2.51	2.22	3.00	4.50	1.16	1.50
Illumina Flow Cells	1.00	2.00	3.00	2.80	4.00	7.00	1.16	1.48
Illumina Flow Lanes	1.00	10.00	11.00	10.48	12.00	15.00	2.65	1.48

Table A11: Summary statistics (RNA-Seq, technical variables)

	Min	Q1	Median	Mean	Q3	Max	SD	MAD
Exon Tags [%]	67.83	79.42	81.64	81.58	83.72	88.57	3.17	3.17
Intron Tags [%]	10.03	14.55	16.46	16.59	18.48	29.75	2.91	2.94
Intergenic Tags [%]	1.15	1.51	1.87	1.83	2.08	2.95	0.34	0.39
CDS Exons Tags Per Kb	86.93	422.41	467.15	485.07	526.06	1721.93	115.41	78.25
Intron Tags Per Kb	1.55	5.48	6.40	6.63	7.50	23.68	1.91	1.50
3' UTR Tags Per Kb	60.90	269.29	301.80	317.47	343.84	1006.24	78.36	53.95
5' UTR Tags Per Kb	19.68	135.64	156.71	160.88	178.56	506.64	39.89	31.59
TSS upstream 10Kb Tags Per Kb	0.38	0.84	1.03	1.06	1.22	3.84	0.32	0.28
TES downstream 10Kb Tags Per Kb	0.73	2.70	3.23	3.34	3.80	13.60	0.99	0.82
Splicing Events [10^5]	8.93	52.42	58.58	61.09	67.35	215.54	15.24	10.87
Known Splicing Events [%]	98.14	98.51	98.89	98.82	99.08	99.96	0.31	0.38
Partially Known Splicing Events [%]	0.00	0.62	0.73	0.74	0.81	1.27	0.16	0.14
Novel Splicing Events [%]	0.00	0.27	0.33	0.41	0.52	1.21	0.18	0.12
Splicing Junctions [10^5]	0.58	1.48	1.55	1.51	1.61	2.11	0.16	0.09
Known Splicing Junctions [%]	83.62	90.17	91.04	90.90	91.89	100.00	1.65	1.28
Partially Known Splicing Junctions [%]	0.00	5.92	6.51	6.59	7.15	12.05	1.19	0.91
Novel Splicing Junctions [%]	0.00	2.14	2.47	2.51	2.80	4.33	0.52	0.49

Table A12: Summary statistics (RNA-Seq, gene model variables)

	1st PC (56.8%) Before TMM Normalization.	2nd PC (19.7%) Before TMM Normalization.	1st PC (52.2%) After TMM Normalization.	2nd PC (21.6%) After TMM Normalization.
Total Mapped Reads $[10^6]$	-0.09	-0.06	-0.11	-0.02
Uniquely Mapped Reads $[10^6]$	0.04	-0.04	0.02	-0.00
Uniquely Mapped Reads [%]	0.73	0.02	0.74	-0.02
Median GC [%]	0.42	0.04	0.38	0.06
Mean GC [%]	0.39	0.07	0.32	0.10
FACS Cells $[10^6]$	0.29	-0.39	0.29	-0.42
Illumina Flow Cells	-0.07	0.08	-0.01	0.03
Illumina Flow Lanes	-0.02	0.07	0.01	0.05

Table A13: Spearman correlation with technical variables (RNA-Seq, original variables).

	1st PC (50.9%) Before TMM Normalization.	2nd PC (15.7%) Before TMM Normalization.	1st PC (45.1%) After TMM Normalization.	2nd PC (17.2%) After TMM Normalization.
Total Mapped Reads $[10^6]$	-0.08	-0.08	-0.10	-0.01
Uniquely Mapped Reads $[10^6]$	0.05	-0.01	0.03	0.08
Uniquely Mapped Reads [%]	0.73	0.31	0.76	0.47
Median GC [%]	0.45	0.27	0.38	0.34
Mean GC [%]	0.44	0.30	0.33	0.36
FACS Cells $[10^6]$	0.28	-0.30	0.29	-0.18
Illumina Flow Cells	-0.11	0.00	-0.03	-0.03
Illumina Flow Lanes	-0.03	0.05	0.00	0.03

Table A14: Spearman correlation with technical variables (RNA-Seq, standardized variables).

	1st PC (56.8%) Before TMM Normalization.	2nd PC (19.7%) Before TMM Normalization.	1st PC (52.2%) After TMM Normalization.	2nd PC (21.6%) After TMM Normalization.
Exon Tags [%]	0.14	0.57	0.07	0.63
Intron Tags [%]	-0.09	-0.55	-0.02	-0.61
Intergenic Tags [%]	-0.57	-0.58	-0.53	-0.60
CDS Exons Tags Per Kb	-0.07	0.18	-0.11	0.24
Intron Tags Per Kb	-0.09	-0.42	-0.06	-0.44
3' UTR Tags Per Kb	0.40	-0.10	0.39	-0.10
5' UTR Tags Per Kb	0.13	0.06	0.09	0.11
TSS upstream 10Kb Tags Per Kb	-0.35	-0.59	-0.35	-0.58
TES downstream 10Kb Tags Per Kb	-0.49	-0.47	-0.47	-0.46
Splicing Events $[10^5]$	-0.01	0.23	-0.05	0.29
Known Splicing Events [%]	0.08	0.73	0.07	0.75
Partially Known Splicing Events [%]	0.03	-0.77	0.03	-0.79
Novel Splicing Events [%]	-0.19	-0.66	-0.19	-0.67
Splicing Junctions $[10^5]$	-0.71	0.08	-0.72	0.14
Known Splicing Junctions [%]	-0.14	0.60	-0.11	0.59

	1st PC (56.8%) Before TMM Normalization.	2nd PC (19.7%) Before TMM Normalization.	1st PC (52.2%) After TMM Normalization.	2nd PC (21.6%) After TMM Normalization.
Partially Known Splicing Junctions [%]	0.22	-0.48	0.19	-0.46
Novel Splicing Junctions [%]	-0.04	-0.78	-0.05	-0.78

Table A15: Spearman correlation with gene model variables (RNA-Seq, original variables).

	1st PC (50.9%) Before TMM Normalization.	2nd PC (15.7%) Before TMM Normalization.	1st PC (45.1%) After TMM Normalization.	2nd PC (17.2%) After TMM Normalization.
Exon Tags [%]	0.18	0.66	0.06	0.64
Intron Tags [%]	-0.14	-0.63	-0.01	-0.59
Intergenic Tags [%]	-0.60	-0.80	-0.53	-0.87
CDS Exons Tags Per Kb	-0.04	0.17	-0.11	0.21
Intron Tags Per Kb	-0.11	-0.47	-0.04	-0.39
3’ UTR Tags Per Kb	0.41	0.08	0.40	0.24
5’ UTR Tags Per Kb	0.15	0.15	0.10	0.25
TSS upstream 10Kb Tags Per Kb	-0.36	-0.70	-0.35	-0.67
TES downstream 10Kb Tags Per Kb	-0.50	-0.62	-0.47	-0.61
Splicing Events $[10^5]$	0.02	0.25	-0.05	0.31
Known Splicing Events [%]	0.08	0.69	0.07	0.66
Partially Known Splicing Events [%]	0.03	-0.67	0.04	-0.57
Novel Splicing Events [%]	-0.19	-0.67	-0.20	-0.68
Splicing Junctions $[10^5]$	-0.71	-0.13	-0.72	-0.23
Known Splicing Junctions [%]	-0.15	0.44	-0.11	0.29
Partially Known Splicing Junctions [%]	0.23	-0.30	0.19	-0.14
Novel Splicing Junctions [%]	-0.02	-0.67	-0.05	-0.56

Table A16: Spearman correlation with gene model variables (RNA-Seq, standardized variables).

	Df	Sum Sq	Mean Sq	F value	Pr(>F)
Celltype	5	5041589.01	1008317.80	8652.07	<0.0001
Subject	19	4140.38	217.91	1.87	0.0135
Day	6	1187.07	197.84	1.7	0.1186
Residuals	808	94164.89	116.54		

Table A17: Sources of variation (RNA-Seq, n=839). ANOVA results for PC1 (total explained variance=45.1%).

	Df	Sum Sq	Mean Sq	F value	Pr(>F)
Celltype	5	1816118.96	363223.79	2358.42	<0.0001
Subject	19	15062.05	792.74	5.15	<0.0001
Day	6	748.45	124.74	0.81	0.5623
Residuals	808	124441.53	154.01		

Table A18: Sources of variation (RNA-Seq, n=839). ANOVA results for PC2 (total explained variance=17.2%).

Subject ID	Celltype	Study Visit Day	Experimental Comment
P	B-cells	Day 1	
H	B-cells	Day 3	
B	B-cells	Day 28	Unscheduled visit, no FACS time available for activation stain. Performed phenotypic analysis at time of cell sort.
D	B-cells	Day 28	
H	Dendritic cells	Day -28	very low PBMC count after initial fractionation of the whole blood
J	Dendritic cells	Day 28	problem w/ FACS machine on DC Sort, so low recovery
H	Monocytes	Day -28	very low PBMC count after initial fractionation of the whole blood
H	Neutrophils	Day -28	very low PBMC count after initial fractionation of the whole blood
D	Neutrophils	Day 28	
H	NK-cells	Day -28	very low PBMC count after initial fractionation of the whole blood
P	NK-cells	Day 0	
J	T-cells	Day 0	
C	T-cells	Day 0	
P	T-cells	Day 1	

Table A19: Outlying observations (RNA-Seq).

Celltype	Pearson Correlation
B-cells	0.932
Dendritic cells	0.968
Monocytes	0.966
Neutrophils	0.882
NK-cells	0.911
T-cells	0.874

Table A20: Correlation between TMM normalization factors (RNA-Seq, all versus per-cell).

	1st PC (18.2%) Before TMM Normalization.	2nd PC (8.7%) Before TMM Normalization.	1st PC (17.2%) After TMM Normalization.	2nd PC (8.9%) After TMM Normalization.
Total Mapped Reads [10^6]	-0.16	0.30	-0.14	-0.29
Uniquely Mapped Reads [10^6]	-0.17	0.33	-0.15	-0.31
Uniquely Mapped Reads [%]	-0.12	0.38	-0.11	-0.39
Median GC [%]	-0.65	0.25	-0.65	-0.25
Mean GC [%]	-0.93	0.10	-0.93	-0.10
FACS Cells [10^6]	0.18	0.05	0.17	-0.05
Illumina Flow Cells	0.55	-0.09	0.52	0.09
Illumina Flow Lanes	0.25	0.08	0.24	-0.08

Table A21: Spearman correlation with technical variables (RNA-Seq, B-cells, standardized variables, outliers excluded).

	1st PC (16.8%) Before TMM Normalization.	2nd PC (12.7%) Before TMM Normalization.	1st PC (15.3%) After TMM Normalization.	2nd PC (12.8%) After TMM Normalization.
Total Mapped Reads [10^6]	-0.13	-0.12	-0.11	0.14
Uniquely Mapped Reads [10^6]	-0.15	-0.11	-0.13	0.13
Uniquely Mapped Reads [%]	-0.46	0.14	-0.48	-0.09
Median GC [%]	-0.75	-0.39	-0.71	0.47
Mean GC [%]	-0.81	-0.41	-0.77	0.50
FACS Cells [10^6]	-0.21	0.07	-0.22	-0.06
Illumina Flow Cells	0.39	0.37	0.34	-0.41
Illumina Flow Lanes	0.19	0.14	0.18	-0.17

Table A22: Spearman correlation with technical variables (RNA-Seq, Dendritic cells, standardized variables, outliers excluded).

	1st PC (16%) Before TMM Normalization.	2nd PC (10.2%) Before TMM Normalization.	1st PC (14.7%) After TMM Normalization.	2nd PC (10.7%) After TMM Normalization.
Total Mapped Reads [10^6]	-0.12	0.28	-0.08	-0.26
Uniquely Mapped Reads [10^6]	-0.13	0.29	-0.09	-0.27
Uniquely Mapped Reads [%]	-0.36	0.30	-0.27	-0.35
Median GC [%]	-0.74	0.25	-0.72	-0.39
Mean GC [%]	-0.84	0.34	-0.82	-0.50
FACS Cells [10^6]	-0.13	0.04	-0.10	-0.08
Illumina Flow Cells	0.53	-0.27	0.45	0.36
Illumina Flow Lanes	0.17	0.01	0.15	0.04

Table A23: Spearman correlation with technical variables (RNA-Seq, Monocytes, standardized variables, outliers excluded).

	1st PC (18.6%) Before TMM Normalization.	2nd PC (8.8%) Before TMM Normalization.	1st PC (11.3%) After TMM Normalization.	2nd PC (9.5%) After TMM Normalization.
Total Mapped Reads [10^6]	-0.24	0.02	0.26	-0.01
Uniquely Mapped Reads [10^6]	-0.24	0.00	0.26	0.01
Uniquely Mapped Reads [%]	-0.12	-0.37	0.14	0.35
Median GC [%]	-0.36	-0.67	0.33	0.68
Mean GC [%]	-0.40	-0.80	0.35	0.78
FACS Cells [10^6]	0.00	-0.02	0.01	0.03
Illumina Flow Cells	0.45	0.33	-0.39	-0.25
Illumina Flow Lanes	0.02	0.21	0.07	-0.13

Table A24: Spearman correlation with technical variables (RNA-Seq, Neutrophils, standardized variables, outliers excluded).

	1st PC (17.3%) Before TMM Normalization.	2nd PC (9.9%) Before TMM Normalization.	1st PC (16.1%) After TMM Normalization.	2nd PC (10.8%) After TMM Normalization.
Total Mapped Reads [10^6]	0.14	-0.02	0.15	-0.07
Uniquely Mapped Reads [10^6]	0.16	-0.01	0.16	-0.05
Uniquely Mapped Reads [%]	0.31	0.39	0.28	0.40
Median GC [%]	0.85	-0.09	0.84	0.02
Mean GC [%]	0.96	-0.06	0.95	0.05
FACS Cells [10^6]	0.14	-0.14	0.16	-0.16
Illumina Flow Cells	-0.44	-0.19	-0.41	-0.20
Illumina Flow Lanes	-0.13	-0.27	-0.11	-0.27

Table A25: Spearman correlation with technical variables (RNA-Seq, NK-cells, standardized variables, outliers excluded).

	1st PC (17.1%) Before TMM Normalization.	2nd PC (11.9%) Before TMM Normalization.	1st PC (16.4%) After TMM Normalization.	2nd PC (12.1%) After TMM Normalization.
Total Mapped Reads [10^6]	-0.01	-0.25	-0.02	-0.24
Uniquely Mapped Reads [10^6]	0.03	-0.25	0.01	-0.25
Uniquely Mapped Reads [%]	0.22	-0.32	0.19	-0.33
Median GC [%]	0.69	-0.20	0.68	-0.24
Mean GC [%]	0.95	-0.00	0.94	-0.06
FACS Cells [10^6]	-0.25	-0.23	-0.25	-0.22
Illumina Flow Cells	-0.44	0.28	-0.41	0.30
Illumina Flow Lanes	-0.29	0.07	-0.28	0.07

Table A26: Spearman correlation with technical variables (RNA-Seq, T-cells, standardized variables, outliers excluded).

	1st PC (18.2%) Before TMM Normalization.	2nd PC (8.7%) Before TMM Normalization.	1st PC (17.2%) After TMM Normalization.	2nd PC (8.9%) After TMM Normalization.
Exon Tags [%]	-0.69	-0.47	-0.69	0.48
Intron Tags [%]	0.68	0.48	0.68	-0.49
Intergenic Tags [%]	0.76	0.33	0.75	-0.34
CDS Exons Tags Per Kb	-0.34	0.23	-0.32	-0.22
Intron Tags Per Kb	0.24	0.44	0.24	-0.43
3' UTR Tags Per Kb	-0.04	0.27	-0.02	-0.25
5' UTR Tags Per Kb	-0.37	0.35	-0.35	-0.34
TSS upstream 10Kb Tags Per Kb	0.08	0.39	0.08	-0.38
TES downstream 10Kb Tags Per Kb	0.28	0.34	0.28	-0.34
Splicing Events [10^5]	-0.37	0.21	-0.35	-0.20
Known Splicing Events [%]	0.09	-0.67	0.07	0.68
Partially Known Splicing Events [%]	0.05	0.72	0.07	-0.72
Novel Splicing Events [%]	-0.17	0.41	-0.16	-0.41
Splicing Junctions [10^5]	-0.17	0.18	-0.16	-0.16
Known Splicing Junctions [%]	0.27	-0.29	0.25	0.28
Partially Known Splicing Junctions [%]	-0.31	0.31	-0.29	-0.29
Novel Splicing Junctions [%]	-0.14	0.25	-0.14	-0.23

Table A27: Spearman correlation with gene model variables (RNA-Seq, B-cells, standardized variables, outliers excluded).

	1st PC (16.8%) Before TMM Normalization.	2nd PC (12.7%) Before TMM Normalization.	1st PC (15.3%) After TMM Normalization.	2nd PC (12.8%) After TMM Normalization.
Exon Tags [%]	-0.36	-0.48	-0.30	0.50
Intron Tags [%]	0.35	0.48	0.29	-0.50
Intergenic Tags [%]	0.50	0.43	0.45	-0.47
CDS Exons Tags Per Kb	-0.22	-0.21	-0.19	0.24
Intron Tags Per Kb	0.12	0.22	0.09	-0.22
3' UTR Tags Per Kb	-0.02	-0.04	-0.01	0.04
5' UTR Tags Per Kb	-0.37	-0.17	-0.35	0.21
TSS upstream 10Kb Tags Per Kb	-0.01	0.03	-0.00	-0.02
TES downstream 10Kb Tags Per Kb	0.17	0.13	0.17	-0.14
Splicing Events [10^5]	-0.22	-0.21	-0.19	0.23
Known Splicing Events [%]	0.47	-0.28	0.51	0.22
Partially Known Splicing Events [%]	-0.53	0.33	-0.58	-0.26
Novel Splicing Events [%]	-0.13	0.02	-0.12	-0.01
Splicing Junctions [10^5]	-0.11	-0.24	-0.09	0.26
Known Splicing Junctions [%]	0.41	0.02	0.42	-0.07
Partially Known Splicing Junctions [%]	-0.44	0.01	-0.45	0.05
Novel Splicing Junctions [%]	-0.30	-0.00	-0.28	0.04

Table A28: Spearman correlation with gene model variables (RNA-Seq, Dendritic cells, standardized variables, outliers excluded).

	1st PC (16%) Before TMM Normalization.	2nd PC (10.2%) Before TMM Normalization.	1st PC (14.7%) After TMM Normalization.	2nd PC (10.7%) After TMM Normalization.
Exon Tags [%]	-0.72	-0.49	-0.82	0.34
Intron Tags [%]	0.72	0.50	0.82	-0.34
Intergenic Tags [%]	0.75	0.35	0.82	-0.20
CDS Exons Tags Per Kb	-0.32	0.18	-0.30	-0.19
Intron Tags Per Kb	0.34	0.50	0.44	-0.39
3' UTR Tags Per Kb	-0.06	0.13	-0.03	-0.09
5' UTR Tags Per Kb	-0.35	0.38	-0.31	-0.41
TSS upstream 10Kb Tags Per Kb	0.15	0.52	0.23	-0.44
TES downstream 10Kb Tags Per Kb	0.30	0.37	0.36	-0.27
Splicing Events [10^5]	-0.31	0.12	-0.31	-0.14
Known Splicing Events [%]	-0.21	-0.80	-0.32	0.71
Partially Known Splicing Events [%]	0.10	0.81	0.21	-0.74
Novel Splicing Events [%]	0.18	0.53	0.24	-0.46
Splicing Junctions [10^5]	-0.09	0.27	-0.08	-0.24
Known Splicing Junctions [%]	0.15	-0.40	0.11	0.39
Partially Known Splicing Junctions [%]	-0.17	0.47	-0.11	-0.47
Novel Splicing Junctions [%]	-0.07	0.28	-0.04	-0.25

Table A29: Spearman correlation with gene model variables (RNA-Seq, Monocytes, standardized variables, outliers excluded).

	1st PC (18.6%) Before TMM Normalization.	2nd PC (8.8%) Before TMM Normalization.	1st PC (11.3%) After TMM Normalization.	2nd PC (9.5%) After TMM Normalization.
Exon Tags [%]	-0.55	-0.37	0.66	0.50
Intron Tags [%]	0.55	0.36	-0.67	-0.50
Intergenic Tags [%]	0.45	0.44	-0.55	-0.57
CDS Exons Tags Per Kb	-0.34	-0.10	0.38	0.14
Intron Tags Per Kb	0.14	0.25	-0.20	-0.32
3' UTR Tags Per Kb	-0.25	0.11	0.29	-0.07
5' UTR Tags Per Kb	-0.35	-0.21	0.35	0.23
TSS upstream 10Kb Tags Per Kb	-0.02	0.19	-0.03	-0.24
TES downstream 10Kb Tags Per Kb	-0.02	0.26	0.01	-0.29
Splicing Events [10^5]	-0.34	-0.10	0.40	0.16
Known Splicing Events [%]	-0.40	0.02	0.52	0.09
Partially Known Splicing Events [%]	0.35	-0.02	-0.48	-0.11
Novel Splicing Events [%]	0.27	-0.03	-0.34	-0.02
Splicing Junctions [10^5]	0.03	-0.10	0.01	0.13
Known Splicing Junctions [%]	0.23	0.15	-0.21	-0.11
Partially Known Splicing Junctions [%]	-0.15	-0.20	0.13	0.17
Novel Splicing Junctions [%]	-0.31	-0.01	0.30	-0.01

Table A30: Spearman correlation with gene model variables (RNA-Seq, Neutrophils, standardized variables, outliers excluded).

	1st PC (17.3%) Before TMM Normalization.	2nd PC (9.9%) Before TMM Normalization.	1st PC (16.1%) After TMM Normalization.	2nd PC (10.8%) After TMM Normalization.
Exon Tags [%]	0.55	-0.82	0.61	-0.74
Intron Tags [%]	-0.54	0.83	-0.60	0.75
Intergenic Tags [%]	-0.74	0.43	-0.76	0.34
CDS Exons Tags Per Kb	0.29	-0.20	0.31	-0.22
Intron Tags Per Kb	-0.19	0.43	-0.22	0.34
3' UTR Tags Per Kb	0.01	-0.16	0.04	-0.21
5' UTR Tags Per Kb	0.46	-0.04	0.46	-0.05
TSS upstream 10Kb Tags Per Kb	-0.04	0.15	-0.05	0.08
TES downstream 10Kb Tags Per Kb	-0.26	0.12	-0.25	0.02
Splicing Events [10^5]	0.30	-0.26	0.33	-0.28
Known Splicing Events [%]	-0.31	-0.60	-0.27	-0.59
Partially Known Splicing Events [%]	0.40	0.63	0.35	0.64
Novel Splicing Events [%]	0.11	0.18	0.11	0.17
Splicing Junctions [10^5]	0.20	-0.06	0.22	-0.08
Known Splicing Junctions [%]	-0.39	-0.06	-0.40	-0.05
Partially Known Splicing Junctions [%]	0.42	0.13	0.42	0.12
Novel Splicing Junctions [%]	0.24	-0.03	0.25	-0.07

Table A31: Spearman correlation with gene model variables (RNA-Seq, NK-cells, standardized variables, outliers excluded).

	1st PC (17.1%) Before TMM Normalization.	2nd PC (11.9%) Before TMM Normalization.	1st PC (16.4%) After TMM Normalization.	2nd PC (12.1%) After TMM Normalization.
Exon Tags [%]	0.71	0.56	0.74	0.53
Intron Tags [%]	-0.70	-0.56	-0.73	-0.52
Intergenic Tags [%]	-0.78	-0.55	-0.81	-0.51
CDS Exons Tags Per Kb	0.22	-0.13	0.22	-0.14
Intron Tags Per Kb	-0.33	-0.43	-0.35	-0.41
3' UTR Tags Per Kb	-0.21	-0.14	-0.21	-0.13
5' UTR Tags Per Kb	0.37	-0.18	0.36	-0.19
TSS upstream 10Kb Tags Per Kb	-0.22	-0.44	-0.24	-0.42
TES downstream 10Kb Tags Per Kb	-0.38	-0.37	-0.39	-0.35
Splicing Events [10^5]	0.28	-0.11	0.28	-0.12
Known Splicing Events [%]	0.08	0.68	0.12	0.67
Partially Known Splicing Events [%]	0.09	-0.63	0.04	-0.64
Novel Splicing Events [%]	-0.19	-0.51	-0.21	-0.50
Splicing Junctions [10^5]	0.23	-0.12	0.22	-0.13
Known Splicing Junctions [%]	-0.29	0.20	-0.27	0.21
Partially Known Splicing Junctions [%]	0.32	-0.20	0.30	-0.21
Novel Splicing Junctions [%]	0.16	-0.30	0.14	-0.31

Table A32: Spearman correlation with gene model variables (RNA-Seq, T-cells, standardized variables, outliers excluded).

	Df	Sum Sq	Mean Sq	F value	Pr(>F)
Celltype	5	2405316.38	481063.28	30536.79	<0.0001
Subject	19	970.13	51.06	3.24	<0.0001
Day	2	54.78	27.39	1.74	0.1773
Residuals	333	5245.94	15.75		

Table A33: Baseline sources of variation (RNA-Seq, All Cells). ANOVA results for PC1 (total explained variance=49.4%)

	Df	Sum Sq	Mean Sq	F value	Pr(>F)
Celltype	5	929050.76	185810.15	11276.63	<0.0001
Subject	19	3398.57	178.87	10.86	<0.0001
Day	2	9.31	4.66	0.28	0.754
Residuals	333	5486.99	16.48		

Table A34: Baseline sources of variation (RNA-Seq, All Cells). ANOVA results for PC2 (total explained variance=19.2%)

	Df	Sum Sq	Mean Sq	F value	Pr(>F)
Subject	19	133717.41	7037.76	18.26	<0.0001
Residuals	40	15420.40	385.51		

Table A35: Baseline sources of variation (RNA-Seq, B-cells). ANOVA results for PC1 (total explained variance=17.5%)

	Df	Sum Sq	Mean Sq	F value	Pr(>F)
Subject	19	75762.72	3987.51	19.87	<0.0001
Residuals	40	8026.66	200.67		

Table A36: Baseline sources of variation (RNA-Seq, B-cells). ANOVA results for PC2 (total explained variance=9.8%)

	Df	Sum Sq	Mean Sq	F value	Pr(>F)
Subject	19	85692.32	4510.12	3.63	0.0003
Residuals	39	48464.24	1242.67		

Table A37: Baseline sources of variation (RNA-Seq, Dendritic cells). ANOVA results for PC1 (total explained variance=17.4%)

	Df	Sum Sq	Mean Sq	F value	Pr(>F)
Subject	19	88998.21	4684.12	7.48	<0.0001
Residuals	39	24426.66	626.32		

Table A38: Baseline sources of variation (RNA-Seq, Dendritic cells). ANOVA results for PC2 (total explained variance=14.7%)

	Df	Sum Sq	Mean Sq	F value	Pr(>F)
Subject	19	102597.23	5399.85	14.32	<0.0001
Residuals	39	14708.41	377.14		

Table A39: Baseline sources of variation (RNA-Seq, Monocytes). ANOVA results for PC1 (total explained variance=15.4%)

	Df	Sum Sq	Mean Sq	F value	Pr(>F)
Subject	19	87583.85	4609.68	22.65	<0.0001
Residuals	39	7938.62	203.55		

Table A40: Baseline sources of variation (RNA-Seq, Monocytes). ANOVA results for PC2 (total explained variance=12.6%)

	Df	Sum Sq	Mean Sq	F value	Pr(>F)
Subject	19	72085.04	3793.95	20.12	<0.0001
Residuals	39	7354.10	188.57		

Table A41: Baseline sources of variation (RNA-Seq, Neutrophils). ANOVA results for PC1 (total explained variance=12.4%)

	Df	Sum Sq	Mean Sq	F value	Pr(>F)
Subject	19	51558.22	2713.59	12.88	<0.0001
Residuals	39	8214.03	210.62		

Table A42: Baseline sources of variation (RNA-Seq, Neutrophils). ANOVA results for PC2 (total explained variance=9.4%)

	Df	Sum Sq	Mean Sq	F value	Pr(>F)
Subject	19	105016.39	5527.18	13.96	<0.0001
Residuals	38	15050.03	396.05		

Table A43: Baseline sources of variation (RNA-Seq, NK-cells). ANOVA results for PC1 (total explained variance=14.7%)

	Df	Sum Sq	Mean Sq	F value	Pr(>F)
Subject	19	92908.47	4889.92	15.95	<0.0001
Residuals	38	11649.44	306.56		

Table A44: Baseline sources of variation (RNA-Seq, NK-cells). ANOVA results for PC2 (total explained variance=12.8%)

	Df	Sum Sq	Mean Sq	F value	Pr(>F)
Subject	19	100623.44	5295.97	7.22	<0.0001
Residuals	38	27858.49	733.12		

Table A45: Baseline sources of variation (RNA-Seq, T-cells). ANOVA results for PC1 (total explained variance=15.5%)

	Df	Sum Sq	Mean Sq	F value	Pr(>F)
Subject	19	85656.24	4508.22	21.21	<0.0001
Residuals	38	8076.98	212.55		

Table A46: Baseline sources of variation (RNA-Seq, T-cells). ANOVA results for PC2 (total explained variance=11.3%)

Gene ID	Gene Name	Gene Description	Coefficient of Determination
ENSG00000141522	ARHGDIA	Rho GDP dissociation inhibitor (GDI) alpha [Source:HGNC Symbol;Acc:678]	0.88
ENSG00000102879	CORO1A	coronin, actin binding protein, 1A [Source:HGNC Symbol;Acc:2252]	0.88
ENSG00000196961	AP2A1	adaptor-related protein complex 2, alpha 1 subunit [Source:HGNC Symbol;Acc:561]	0.86
ENSG00000188994	ZNF292	zinc finger protein 292 [Source:HGNC Symbol;Acc:18410]	0.86
ENSG00000163714	U2SURP	U2 snRNP-associated SURP domain containing [Source:HGNC Symbol;Acc:30855]	0.85
ENSG00000137770	CTDSPL2	CTD (carboxy-terminal domain, RNA polymerase II, polypeptide A) small phosphatase like 2 [Source:HGNC Symbol;Acc:26936]	0.85
ENSG00000113441	LNPEP	leucyl/cystinyl aminopeptidase [Source:HGNC Symbol;Acc:6656]	0.84
ENSG00000167671	UBXN6	UBX domain protein 6 [Source:HGNC Symbol;Acc:14928]	0.84
ENSG00000105701	FKBP8	FK506 binding protein 8, 38kDa [Source:HGNC Symbol;Acc:3724]	0.84
ENSG00000112200	ZNF451	zinc finger protein 451 [Source:HGNC Symbol;Acc:21091]	0.84
ENSG00000142186	SCYL1	SCY1-like 1 (S. cerevisiae) [Source:HGNC Symbol;Acc:14372]	0.84
ENSG00000144747	TMF1	TATA element modulatory factor 1 [Source:HGNC Symbol;Acc:11870]	0.84
ENSG00000075292	ZNF638	zinc finger protein 638 [Source:HGNC Symbol;Acc:17894]	0.83
ENSG00000159069	FBXW5	F-box and WD repeat domain containing 5 [Source:HGNC Symbol;Acc:13613]	0.83
ENSG00000083312	TNPO1	transportin 1 [Source:HGNC Symbol;Acc:6401]	0.82
ENSG00000103653	CSK	c-src tyrosine kinase [Source:HGNC Symbol;Acc:2444]	0.82
ENSG00000089693	MLF2	myeloid leukemia factor 2 [Source:HGNC Symbol;Acc:7126]	0.82
ENSG00000138592	USP8	ubiquitin specific peptidase 8 [Source:HGNC Symbol;Acc:12631]	0.82
ENSG00000188986	COBRA1	cofactor of BRCA1 [Source:HGNC Symbol;Acc:24324]	0.82
ENSG00000110711	AIP	aryl hydrocarbon receptor interacting protein [Source:HGNC Symbol;Acc:358]	0.82
ENSG00000160410	SHKBP1	SH3KBP1 binding protein 1 [Source:HGNC Symbol;Acc:19214]	0.82
ENSG00000033867	SLC4A7	solute carrier family 4, sodium bicarbonate cotransporter, member 7 [Source:HGNC Symbol;Acc:11033]	0.81
ENSG00000128585	MKLN1	muskelin 1, intracellular mediator containing kelch motifs [Source:HGNC Symbol;Acc:7109]	0.81
ENSG00000120802	TMPO	thymopoietin [Source:HGNC Symbol;Acc:11875]	0.81
ENSG00000087088	BAX	BCL2-associated X protein [Source:HGNC Symbol;Acc:959]	0.81
ENSG00000198887	SMC5	structural maintenance of chromosomes 5 [Source:HGNC Symbol;Acc:20465]	0.81
ENSG00000065243	PKN2	protein kinase N2 [Source:HGNC Symbol;Acc:9406]	0.81
ENSG00000092439	TRPM7	transient receptor potential cation channel, subfamily M, member 7 [Source:HGNC Symbol;Acc:17994]	0.81
ENSG00000130429	ARPC1B	actin related protein 2/3 complex, subunit 1B, 41kDa [Source:HGNC Symbol;Acc:704]	0.81

Table A47: Genes with high baseline variability (RNA-Seq, B-cells, PC1 (17.5%)). Coefficient of determination > 0.80 (>80% explained variance.)

Gene ID	Gene Name	Gene Description	Coefficient of Determination
ENSG00000136286	MYO1G	myosin IG [Source:HGNC Symbol;Acc:13880]	0.95
ENSG00000196961	AP2A1	adaptor-related protein complex 2, alpha 1 subunit [Source:HGNC Symbol;Acc:561]	0.94
ENSG00000173542	MOBKL1A	MOB1, Mps One Binder kinase activator-like 1A (yeast) [Source:HGNC Symbol;Acc:29801]	0.90
ENSG00000090554	FLT3LG	fms-related tyrosine kinase 3 ligand [Source:HGNC Symbol;Acc:3766]	0.89

Gene ID	Gene Name	Gene Description	Coefficient of Determination
ENSG00000160883	HK3	hexokinase 3 (white cell) [Source:HGNC Symbol;Acc:4925]	0.88
ENSG00000110697	PITPNM1	phosphatidylinositol transfer protein, membrane-associated 1 [Source:HGNC Symbol;Acc:9003]	0.88
ENSG00000146094	DOK3	docking protein 3 [Source:HGNC Symbol;Acc:24583]	0.87
ENSG00000171777	RASGRP4	RAS guanyl releasing protein 4 [Source:HGNC Symbol;Acc:18958]	0.87
ENSG00000076944	STXBP2	syntaxin binding protein 2 [Source:HGNC Symbol;Acc:11445]	0.87
ENSG00000186635	ARAP1	ArfGAP with RhoGAP domain, ankyrin repeat and PH domain 1 [Source:HGNC Symbol;Acc:16925]	0.87
ENSG00000090674	MCOLN1	mucolipin 1 [Source:HGNC Symbol;Acc:13356]	0.87
ENSG00000107954	NEURL	neuralized homolog (Drosophila) [Source:HGNC Symbol;Acc:7761]	0.87
ENSG00000099992	TBC1D10A	TBC1 domain family, member 10A [Source:HGNC Symbol;Acc:23609]	0.87
ENSG00000100852	ARHGAP5	Rho GTPase activating protein 5 [Source:HGNC Symbol;Acc:675]	0.87
ENSG00000095370	SH2D3C	SH2 domain containing 3C [Source:HGNC Symbol;Acc:16884]	0.86
ENSG00000144579	CTDSP1	CTD (carboxy-terminal domain, RNA polymerase II, polypeptide A) small phosphatase 1 [Source:HGNC Symbol;Acc:21614]	0.86
ENSG00000006459	JHDM1D	jumonji C domain containing histone demethylase 1 homolog D (S. cerevisiae) [Source:HGNC Symbol;Acc:22224]	0.86
ENSG00000028137	TNFRSF1B	tumor necrosis factor receptor superfamily, member 1B [Source:HGNC Symbol;Acc:11917]	0.86
ENSG00000111371	SLC38A1	solute carrier family 38, member 1 [Source:HGNC Symbol;Acc:13447]	0.85
ENSG00000104164	PLDN	pallidin homolog (mouse) [Source:HGNC Symbol;Acc:8549]	0.85
ENSG00000099308	MAST3	microtubule associated serine/threonine kinase 3 [Source:HGNC Symbol;Acc:19036]	0.85
ENSG00000204592	HLA-E	major histocompatibility complex, class I, E [Source:HGNC Symbol;Acc:4962]	0.85
ENSG00000123143	PKN1	protein kinase N1 [Source:HGNC Symbol;Acc:9405]	0.85
ENSG00000160570	DEDD2	death effector domain containing 2 [Source:HGNC Symbol;Acc:24450]	0.85
ENSG00000119403	PHF19	PHD finger protein 19 [Source:HGNC Symbol;Acc:24566]	0.84
ENSG00000105287	PRKD2	protein kinase D2 [Source:HGNC Symbol;Acc:17293]	0.84
ENSG00000151746	BICD1	bicaudal D homolog 1 (Drosophila) [Source:HGNC Symbol;Acc:1049]	0.83
ENSG00000170017	ALCAM	activated leukocyte cell adhesion molecule [Source:HGNC Symbol;Acc:400]	0.83
ENSG00000160999	SH2B2	SH2B adaptor protein 2 [Source:HGNC Symbol;Acc:17381]	0.83
ENSG00000008869	HEATR5B	HEAT repeat containing 5B [Source:HGNC Symbol;Acc:29273]	0.82
ENSG00000173020	ADRBK1	adrenergic, beta, receptor kinase 1 [Source:HGNC Symbol;Acc:289]	0.82
ENSG00000168067	MAP4K2	mitogen-activated protein kinase kinase kinase kinase 2 [Source:HGNC Symbol;Acc:6864]	0.82
ENSG00000134294	SLC38A2	solute carrier family 38, member 2 [Source:HGNC Symbol;Acc:13448]	0.82
ENSG00000105122	RASAL3	RAS protein activator like 3 [Source:HGNC Symbol;Acc:26129]	0.82
ENSG00000175489	LRRC25	leucine rich repeat containing 25 [Source:HGNC Symbol;Acc:29806]	0.82
ENSG00000007312	CD79B	CD79b molecule, immunoglobulin-associated beta [Source:HGNC Symbol;Acc:1699]	0.82
ENSG00000198925	ATG9A	ATG9 autophagy related 9 homolog A (S. cerevisiae) [Source:HGNC Symbol;Acc:22408]	0.82
ENSG00000125898	FAM110A	family with sequence similarity 110, member A [Source:HGNC Symbol;Acc:16188]	0.82
ENSG00000104205	SGK3	serum/glucocorticoid regulated kinase family, member 3 [Source:HGNC Symbol;Acc:10812]	0.82
ENSG00000178950	GAK	cyclin G associated kinase [Source:HGNC Symbol;Acc:4113]	0.82
ENSG00000175550	DRAP1	DR1-associated protein 1 (negative cofactor 2 alpha) [Source:HGNC Symbol;Acc:3019]	0.82
ENSG00000186350	RXRA	retinoid X receptor, alpha [Source:HGNC Symbol;Acc:10477]	0.82
ENSG00000196839	ADA	adenosine deaminase [Source:HGNC Symbol;Acc:186]	0.81

Gene ID	Gene Name	Gene Description	Coefficient of Determination
ENSG00000101412	E2F1	E2F transcription factor 1 [Source:HGNC Symbol;Acc:3113]	0.81
ENSG00000136636	KCTD3	potassium channel tetramerisation domain containing 3 [Source:HGNC Symbol;Acc:21305]	0.81
ENSG00000178719	GRINA	glutamate receptor, ionotropic, N-methyl D-aspartate-associated protein 1 (glutamate binding) [Source:HGNC Symbol;Acc:4589]	0.81
ENSG00000198055	GRK6	G protein-coupled receptor kinase 6 [Source:HGNC Symbol;Acc:4545]	0.81
ENSG00000187531	SIRT7	sirtuin 7 [Source:HGNC Symbol;Acc:14935]	0.81
ENSG00000063854	HAGH	hydroxyacylglutathione hydrolase [Source:HGNC Symbol;Acc:4805]	0.81
ENSG00000205268	PDE7A	phosphodiesterase 7A [Source:HGNC Symbol;Acc:8791]	0.81
ENSG00000130511	SSBP4	single stranded DNA binding protein 4 [Source:HGNC Symbol;Acc:15676]	0.81
ENSG00000152133	CCDC75	coiled-coil domain containing 75 [Source:HGNC Symbol;Acc:26768]	0.81
ENSG00000130382	MLLT1	myeloid/lymphoid or mixed-lineage leukemia (trithorax homolog, Drosophila); translocated to, 1 [Source:HGNC Symbol;Acc:7134]	0.81
ENSG00000111679	PTPN6	protein tyrosine phosphatase, non-receptor type 6 [Source:HGNC Symbol;Acc:9658]	0.81

Table A48: Genes with high baseline variability (RNA-Seq, Dendritic cells, PC1 (17.4%)). Coefficient of determination > 0.80 (>80% explained variance.)

Gene ID	Gene Name	Gene Description	Coefficient of Determination
ENSG00000172531	PPP1CA	protein phosphatase 1, catalytic subunit, alpha isozyme [Source:HGNC Symbol;Acc:9281]	0.91
ENSG00000105723	GSK3A	glycogen synthase kinase 3 alpha [Source:HGNC Symbol;Acc:4616]	0.87
ENSG00000072958	AP1M1	adaptor-related protein complex 1, mu 1 subunit [Source:HGNC Symbol;Acc:13667]	0.87
ENSG00000010256	UQCRC1	ubiquinol-cytochrome c reductase core protein I [Source:HGNC Symbol;Acc:12585]	0.87
ENSG00000149925	ALDOA	aldolase A, fructose-bisphosphate [Source:HGNC Symbol;Acc:414]	0.87
ENSG00000125970	RALY	RNA binding protein, autoantigenic (hnRNP-associated with lethal yellow homolog (mouse)) [Source:HGNC Symbol;Acc:15921]	0.87
ENSG00000124155	PIGT	phosphatidylinositol glycan anchor biosynthesis, class T [Source:HGNC Symbol;Acc:14938]	0.86
ENSG00000114353	GNAI2	guanine nucleotide binding protein (G protein), alpha inhibiting activity polypeptide 2 [Source:HGNC Symbol;Acc:4385]	0.86
ENSG00000116685	KIAA2013	KIAA2013 [Source:HGNC Symbol;Acc:28513]	0.85
ENSG00000089327	FXYD5	FXYD domain containing ion transport regulator 5 [Source:HGNC Symbol;Acc:4029]	0.85
ENSG00000101745	ANKRD12	ankyrin repeat domain 12 [Source:HGNC Symbol;Acc:29135]	0.84
ENSG00000105568	PPP2R1A	protein phosphatase 2, regulatory subunit A, alpha [Source:HGNC Symbol;Acc:9302]	0.84
ENSG00000103502	CDIPT	CDP-diacylglycerol–inositol 3-phosphatidyltransferase [Source:HGNC Symbol;Acc:1769]	0.84
ENSG00000110446	SLC15A3	solute carrier family 15, member 3 [Source:HGNC Symbol;Acc:18068]	0.84
ENSG00000074800	ENO1	enolase 1, (alpha) [Source:HGNC Symbol;Acc:3350]	0.84
ENSG00000105669	COPE	coatomer protein complex, subunit epsilon [Source:HGNC Symbol;Acc:2234]	0.84
ENSG00000127914	AKAP9	A kinase (PRKA) anchor protein (yotiao) 9 [Source:HGNC Symbol;Acc:379]	0.83
ENSG00000100354	TNRC6B	trinucleotide repeat containing 6B [Source:HGNC Symbol;Acc:29190]	0.83
ENSG00000179950	PUF60	poly-U binding splicing factor 60KDa [Source:HGNC Symbol;Acc:17042]	0.83
ENSG00000181817	LSM10	LSM10, U7 small nuclear RNA associated [Source:HGNC Symbol;Acc:17562]	0.83

Gene ID	Gene Name	Gene Description	Coefficient of Determination
ENSG00000177105	RHOG	ras homolog gene family, member G (rho G) [Source:HGNC Symbol;Acc:672]	0.83
ENSG00000066322	ELOVL1	elongation of very long chain fatty acids (FEN1/Elo2, SUR4/Elo3, yeast)-like 1 [Source:HGNC Symbol;Acc:14418]	0.83
ENSG00000172757	CFL1	cofilin 1 (non-muscle) [Source:HGNC Symbol;Acc:1874]	0.82
ENSG00000108528	SLC25A11	solute carrier family 25 (mitochondrial carrier; oxoglutarate carrier), member 11 [Source:HGNC Symbol;Acc:10981]	0.82
ENSG00000099341	PSMD8	proteasome (prosome, macropain) 26S subunit, non-ATPase, 8 [Source:HGNC Symbol;Acc:9566]	0.82
ENSG00000126934	MAP2K2	mitogen-activated protein kinase kinase 2 [Source:HGNC Symbol;Acc:6842]	0.81
ENSG00000105701	FKBP8	FK506 binding protein 8, 38kDa [Source:HGNC Symbol;Acc:3724]	0.81
ENSG00000249348	RP11-814P23.1		0.81
ENSG00000188994	ZNF292	zinc finger protein 292 [Source:HGNC Symbol;Acc:18410]	0.81
ENSG00000130429	ARPC1B	actin related protein 2/3 complex, subunit 1B, 41kDa [Source:HGNC Symbol;Acc:704]	0.81
ENSG00000108774	RAB5C	RAB5C, member RAS oncogene family [Source:HGNC Symbol;Acc:9785]	0.81
ENSG00000189180	ZNF33A	zinc finger protein 33A [Source:HGNC Symbol;Acc:13096]	0.81
ENSG00000161203	AP2M1	adaptor-related protein complex 2, mu 1 subunit [Source:HGNC Symbol;Acc:564]	0.81

Table A49: Genes with high baseline variability (RNA-Seq, Monocytes, PC1 (15.4%)). Coefficient of determination > 0.80 (>80% explained variance.)

Gene ID	Gene Name	Gene Description	Coefficient of Determination
ENSG00000144118	RALB	v-ral simian leukemia viral oncogene homolog B (ras related; GTP binding protein) [Source:HGNC Symbol;Acc:9840]	0.85
ENSG00000103168	TAF1C	TATA box binding protein (TBP)-associated factor, RNA polymerase I, C, 110kDa [Source:HGNC Symbol;Acc:11534]	0.83
ENSG00000176953	NFATC2IP	nuclear factor of activated T-cells, cytoplasmic, calcineurin-dependent 2 interacting protein [Source:HGNC Symbol;Acc:25906]	0.82
ENSG00000109332	UBE2D3	ubiquitin-conjugating enzyme E2D 3 (UBC4/5 homolog, yeast) [Source:HGNC Symbol;Acc:12476]	0.82
ENSG00000165006	UBAP1	ubiquitin associated protein 1 [Source:HGNC Symbol;Acc:12461]	0.81

Table A50: Genes with high baseline variability (RNA-Seq, Neutrophils, PC1 (12.4%)). Coefficient of determination > 0.80 (>80% explained variance.)

Gene ID	Gene Name	Gene Description	Coefficient of Determination
ENSG00000182087	C19orf6	chromosome 19 open reading frame 6 [Source:HGNC Symbol;Acc:17039]	0.87
ENSG00000127419	TMEM175	transmembrane protein 175 [Source:HGNC Symbol;Acc:28709]	0.86
ENSG00000141258	SGSM2	small G protein signaling modulator 2 [Source:HGNC Symbol;Acc:29026]	0.86
ENSG00000140983	RHOT2	ras homolog gene family, member T2 [Source:HGNC Symbol;Acc:21169]	0.86
ENSG00000131584	ACAP3	ArfGAP with coiled-coil, ankyrin repeat and PH domains 3 [Source:HGNC Symbol;Acc:16754]	0.85
ENSG00000176978	DPP7	dipeptidyl-peptidase 7 [Source:HGNC Symbol;Acc:14892]	0.85
ENSG00000167302	C17orf56	chromosome 17 open reading frame 56 [Source:HGNC Symbol;Acc:26458]	0.85
ENSG00000103249	CLCN7	chloride channel 7 [Source:HGNC Symbol;Acc:2025]	0.84
ENSG00000125503	PPP1R12C	protein phosphatase 1, regulatory (inhibitor) subunit 12C [Source:HGNC Symbol;Acc:14947]	0.84

Gene ID	Gene Name	Gene Description	Coefficient of Determination
ENSG00000242802	KIAA0415	KIAA0415 [Source:HGNC Symbol;Acc:22197]	0.84
ENSG00000101199	ARFGAP1	ADP-ribosylation factor GTPase activating protein 1 [Source:HGNC Symbol;Acc:15852]	0.84
ENSG00000197070	ARRDC1	arrestin domain containing 1 [Source:HGNC Symbol;Acc:28633]	0.83
ENSG00000138834	MAPK8IP3	mitogen-activated protein kinase 8 interacting protein 3 [Source:HGNC Symbol;Acc:6884]	0.83
ENSG00000149541	B3GAT3	beta-1,3-glucuronyltransferase 3 (glucuronosyltransferase I) [Source:HGNC Symbol;Acc:923]	0.83
ENSG00000162032	SPSB3	splA/ryanodine receptor domain and SOCS box containing 3 [Source:HGNC Symbol;Acc:30629]	0.83
ENSG00000073169	RP3-402G11.5	Selenoprotein O [Source:UniProtKB/Swiss-Prot;Acc:Q9BVL4]	0.82
ENSG00000149499	EML3	echinoderm microtubule associated protein like 3 [Source:HGNC Symbol;Acc:26666]	0.82
ENSG00000091039	OSBPL8	oxysterol binding protein-like 8 [Source:HGNC Symbol;Acc:16396]	0.82
ENSG00000076928	ARHGEF1	Rho guanine nucleotide exchange factor (GEF) 1 [Source:HGNC Symbol;Acc:681]	0.82
ENSG00000142330	CAPN10	calpain 10 [Source:HGNC Symbol;Acc:1477]	0.81
ENSG00000176248	ANAPC2	anaphase promoting complex subunit 2 [Source:HGNC Symbol;Acc:19989]	0.81
ENSG00000104960	PTOV1	prostate tumor overexpressed 1 [Source:HGNC Symbol;Acc:9632]	0.81

Table A51: Genes with high baseline variability (RNA-Seq, NK-cells, PC1 (14.7%)). Coefficient of determination > 0.80 (>80% explained variance.)

Gene ID	Gene Name	Gene Description	Coefficient of Determination
ENSG00000102879	CORO1A	coronin, actin binding protein, 1A [Source:HGNC Symbol;Acc:2252]	0.90
ENSG00000011009	LYPLA2	lysophospholipase II [Source:HGNC Symbol;Acc:6738]	0.87
ENSG00000169727	GPS1	G protein pathway suppressor 1 [Source:HGNC Symbol;Acc:4549]	0.86
ENSG00000185236	RAB11B	RAB11B, member RAS oncogene family [Source:HGNC Symbol;Acc:9761]	0.85
ENSG00000145907	G3BP1	GTPase activating protein (SH3 domain) binding protein 1 [Source:HGNC Symbol;Acc:30292]	0.84
ENSG00000176978	DPP7	dipeptidyl-peptidase 7 [Source:HGNC Symbol;Acc:14892]	0.83
ENSG00000112200	ZNF451	zinc finger protein 451 [Source:HGNC Symbol;Acc:21091]	0.83
ENSG00000099817	POLR2E	polymerase (RNA) II (DNA directed) polypeptide E, 25kDa [Source:HGNC Symbol;Acc:9192]	0.82
ENSG00000176101	SSNA1	Sjogren syndrome nuclear autoantigen 1 [Source:HGNC Symbol;Acc:11321]	0.82
ENSG00000105701	FKBP8	FK506 binding protein 8, 38kDa [Source:HGNC Symbol;Acc:3724]	0.82
ENSG00000186010	NDUFA13	NADH dehydrogenase (ubiquinone) 1 alpha subcomplex, 13 [Source:HGNC Symbol;Acc:17194]	0.82
ENSG00000141985	SH3GL1	SH3-domain GRB2-like 1 [Source:HGNC Symbol;Acc:10830]	0.82
ENSG00000092439	TRPM7	transient receptor potential cation channel, subfamily M, member 7 [Source:HGNC Symbol;Acc:17994]	0.82
ENSG00000162032	SPSB3	splA/ryanodine receptor domain and SOCS box containing 3 [Source:HGNC Symbol;Acc:30629]	0.82
ENSG00000164897	TMUB1	transmembrane and ubiquitin-like domain containing 1 [Source:HGNC Symbol;Acc:21709]	0.82
ENSG00000153922	CHD1	chromodomain helicase DNA binding protein 1 [Source:HGNC Symbol;Acc:1915]	0.81
ENSG00000197114	ZGPAT	zinc finger, CCCH-type with G patch domain [Source:HGNC Symbol;Acc:15948]	0.81

Gene ID	Gene Name	Gene Description	Coefficient of Determination
ENSG00000196504	PRPF40A	PRP40 pre-mRNA processing factor 40 homolog A (S. cerevisiae) [Source:HGNC Symbol;Acc:16463]	0.81
ENSG00000063244	U2AF2	U2 small nuclear RNA auxiliary factor 2 [Source:HGNC Symbol;Acc:23156]	0.81
ENSG00000142186	SCYL1	SCY1-like 1 (S. cerevisiae) [Source:HGNC Symbol;Acc:14372]	0.81

Table A52: Genes with high baseline variability (RNA-Seq, T-cells, PC1 (15.5%)). Coefficient of determination > 0.80 (>80% explained variance.)

Cell Type	Day	Genes (3 days)	Genes (2 days)	Genes (1 day)	Sensitivity (2 days)	Sensitivity (1 day)	FDR (2 days)	FDR (1 day)
B-cells	1	30	35	76	0.87	0.90	0.26	0.64
B-cells	3	58	65	85	0.95	0.91	0.15	0.38
Dendritic cells	1	230	221	256	0.88	0.90	0.08	0.19
Dendritic cells	3	33	34	58	0.76	0.73	0.26	0.59
Monocytes	1	421	445	473	0.94	0.93	0.11	0.17
Monocytes	3	37	34	53	0.78	0.78	0.15	0.45
Neutrophils	1	742	811	853	0.97	0.95	0.11	0.17
Neutrophils	3	143	144	179	0.94	0.93	0.07	0.26
NK-cells	1	106	73	99	0.60	0.61	0.12	0.34
NK-cells	3	94	99	132	0.94	0.99	0.11	0.30
T-cells	1	57	57	93	0.89	0.93	0.11	0.43
T-cells	3	5	10	30	0.80	0.60	0.60	0.90

Table A53: Impact of using multiple baseline measures on gene response (RNA-Seq). Genes were selected based on a mean fold change $\geq$ 1.5 (up or down) in the SV-AS03 group. Sensitivity and FDR were calculated by comparing gene lists obtained when using the mean of one (day 0) or two baseline days (day 0, -14) to the list obtained when using the mean of three baseline days (day 0, -14, -28).

Gene Type	B-cells Day 1	Dendritic cells Day 1	Dendritic cells Day 3	Monocytes Day 1	Neutrophils Day 1	Neutrophils Day 3	Neutrophils Day 28	NK-cells Day 1	NK-cells Day 3	NK-cells Day 7	NK-cells Day 28	T-cells Day 1
protein coding	14	189	11	333	499	124	1	21	68	1	61	44
processed transcript	0	16	1	33	30	11	0	0	1	0	1	1
pseudogene	0	10	1	23	22	3	0	0	1	0	0	1
lincRNA	0	8	0	7	17	2	0	1	0	0	1	2
snoRNA	0	1	0	3	0	0	0	1	0	0	0	0
misc RNA	0	0	0	1	2	0	0	0	0	0	0	1
scRNA pseudogene	0	0	0	2	1	0	0	0	0	0	0	0
IG C pseudogene	0	1	0	1	0	0	0	0	0	0	0	0
snRNA	0	0	0	1	1	0	0	0	0	0	0	0
miRNA	0	0	0	1	0	0	0	1	0	0	0	0
polymorphic pseudogene	0	0	0	1	0	0	0	0	0	0	0	0

Table A54: Statistically significant genes by gene type, cell type, and day (RNA-Seq).

Gene Type	B-cells	Dendritic cells	Monocytes	Neutrophils	NK-cells	T-cells
protein coding	14	192	333	528	150	44
processed transcript	0	17	33	36	2	1
pseudogene	0	10	23	24	1	1
lincRNA	0	8	7	18	2	2
snoRNA	0	1	3	0	1	0
misc RNA	0	0	1	2	0	1
scRNA pseudogene	0	0	2	1	0	0
IG C pseudogene	0	1	1	0	0	0
snRNA	0	0	1	1	0	0
miRNA	0	0	1	0	1	0
polymorphic pseudogene	0	0	1	0	0	0

Table A55: Statistically significant genes by gene type and cell type (RNA-Seq).

	B-cells	Dendritic cells	Monocytes	Neutrophils	NK-cells	T-cells
B-cells	-	12	12	12	6	11
Dendritic cells	12	-	130	90	14	29
Monocytes	12	130	-	138	15	32
Neutrophils	12	90	138	-	14	35
NK-cells	6	14	15	14	-	15
T-cells	11	29	32	35	15	-

Table A56: Overlap of significantly differentially expressed genes between cell types (RNA-Seq, Day 1)

Gene ID	Gene Name	Gene Description	B-cells Log_2 Fold Change	Dendritic cells Log_2 Fold Change	Monocytes Log_2 Fold Change	Neutrophils Log_2 Fold Change	NK-cells Log_2 Fold Change	T-cells Log_2 Fold Change
ENSG00000133106	EPSTI1	epithelial stromal interaction 1 (breast) [Source:HGNC Symbol;Acc:16465]	0.59	1.06	1.14	1.08	0.76	1.12
ENSG00000117228	GBP1	guanylate binding protein 1, interferon-inducible [Source:HGNC Symbol;Acc:4182]	1.33	1.86	2.67	2.17	1.21	1.24
ENSG00000125347	IRF1	interferon regulatory factor 1 [Source:HGNC Symbol;Acc:6116]	1.38	1.45	1.46	1.32	1.23	1.43
ENSG00000138496	PARP9	poly (ADP-ribose) polymerase family, member 9 [Source:HGNC Symbol;Acc:24118]	0.59	1.11	1.31	2.42	0.67	0.99
ENSG00000115415	STAT1	signal transducer and activator of transcription 1, 91kDa [Source:HGNC Symbol;Acc:11362]	1.15	1.05	1.04	0.94	0.66	1.00

Table A57: Genes significantly differentially expressed in all six cell types (RNA-Seq, Day 1)

Cluster ID	Cluster Size	Gene ID	Gene Name	Gene Description	Log FC
CBCLD1-001	4	ENSG00000117228	GBP1	guanylate binding protein 1, interferon-inducible [Source:HGNC Symbol;Acc:4182]	1.33
CBCLD1-001	4	ENSG00000162645	GBP2	guanylate binding protein 2, interferon-inducible [Source:HGNC Symbol;Acc:4183]	1.20
CBCLD1-001	4	ENSG00000162654	GBP4	guanylate binding protein 4 [Source:HGNC Symbol;Acc:20480]	1.05
CBCLD1-001	4	ENSG00000115415	STAT1	signal transducer and activator of transcription 1, 91kDa [Source:HGNC Symbol;Acc:11362]	1.38

Table A58: Significant gene clusters (RNA-Seq, B-cells, Day 1)

Cluster ID	Cluster Size	Gene ID	Gene Name	Gene Description
CBCLD1D28-001	5	ENSG00000117228	GBP1	guanylate binding protein 1, interferon-inducible [Source:HGNC Symbol;Acc:4182]

Cluster ID	Cluster Size	Gene ID	Gene Name	Gene Description
CBCLD1D28-001	5	ENSG00000162645	GBP2	guanylate binding protein 2, interferon-inducible [Source:HGNC Symbol;Acc:4183]
CBCLD1D28-001	5	ENSG00000162654	GBP4	guanylate binding protein 4 [Source:HGNC Symbol;Acc:20480]
CBCLD1D28-001	5	ENSG00000154451	GBP5	guanylate binding protein 5 [Source:HGNC Symbol;Acc:19895]
CBCLD1D28-001	5	ENSG00000115415	STAT1	signal transducer and activator of transcription 1, 91kDa [Source:HGNC Symbol;Acc:11362]

Table A59: Significant gene clusters (RNA-Seq, B-cells, Day 1-28)

Cluster ID	Cluster Size	Gene ID	Gene Name	Gene Description	Log FC
CDNCD1-001	2	ENSG00000117228	GBP1	guanylate binding protein 1, interferon-inducible [Source:HGNC Symbol;Acc:4182]	1.86
CDNCD1-001	2	ENSG00000138496	PARP9	poly (ADP-ribose) polymerase family, member 9 [Source:HGNC Symbol;Acc:24118]	1.06
CDNCD1-002	2	ENSG00000158517	NCF1	neutrophil cytosolic factor 1 [Source:HGNC Symbol;Acc:7660]	0.96
CDNCD1-002	2	ENSG00000165178	NCF1C	neutrophil cytosolic factor 1C pseudogene [Source:HGNC Symbol;Acc:32523]	0.94
CDNCD1-003	7	ENSG00000133106	EPSTI1	epithelial stromal interaction 1 (breast) [Source:HGNC Symbol;Acc:16465]	1.11
CDNCD1-003	7	ENSG00000108679	LGALS3BP	lectin, galactoside-binding, soluble, 3 binding protein [Source:HGNC Symbol;Acc:6564]	1.12
CDNCD1-003	7	ENSG00000100911	PSME2	proteasome (prosome, macropain) activator subunit 2 (PA28 beta) [Source:HGNC Symbol;Acc:9569]	0.87
CDNCD1-003	7	ENSG00000177409	SAMD9L	sterile alpha motif domain containing 9-like [Source:HGNC Symbol;Acc:1349]	0.97
CDNCD1-003	7	ENSG00000149131	SERPING1	serpin peptidase inhibitor, clade G (C1 inhibitor), member 1 [Source:HGNC Symbol;Acc:1228]	2.16
CDNCD1-003	7	ENSG00000115415	STAT1	signal transducer and activator of transcription 1, 91kDa [Source:HGNC Symbol;Acc:11362]	1.45
CDNCD1-003	7	ENSG00000132530	XAF1	XIAP associated factor 1 [Source:HGNC Symbol;Acc:30932]	0.66
CDNCD1-004	2	ENSG00000166278	C2	complement component 2 [Source:HGNC Symbol;Acc:1248]	0.91
CDNCD1-004	2	ENSG00000026751	SLAMF7	SLAM family member 7 [Source:HGNC Symbol;Acc:21394]	0.85
CDNCD1-005	2	ENSG00000163823	CCR1	chemokine (C-C motif) receptor 1 [Source:HGNC Symbol;Acc:1602]	0.61
CDNCD1-005	2	ENSG00000198959	TGM2	transglutaminase 2 (C polypeptide, protein-glutamine-gamma-glutamyltransferase) [Source:HGNC Symbol;Acc:11778]	1.40
CDNCD1-006	2	ENSG00000115594	IL1R1	interleukin 1 receptor, type I [Source:HGNC Symbol;Acc:5993]	-0.63
CDNCD1-006	2	ENSG00000115590	IL1R2	interleukin 1 receptor, type II [Source:HGNC Symbol;Acc:5994]	-0.71
CDNCD1-007	3	ENSG00000173369	C1QB	complement component 1, q subcomponent, B chain [Source:HGNC Symbol;Acc:1242]	1.16
CDNCD1-007	3	ENSG00000229754	CXCR2P1	chemokine (C-X-C motif) receptor 2 pseudogene 1 [Source:HGNC Symbol;Acc:6028]	0.82

Cluster ID	Cluster Size	Gene ID	Gene Name	Gene Description	Log FC
CDNCD1-007	3	ENSG00000111181	SLC6A12	solute carrier family 6 (neurotransmitter transporter, betaine/GABA), member 12 [Source:HGNC Symbol;Acc:11045]	0.62

Table A60: Significant gene clusters (RNA-Seq, Dendritic cells, Day 1)

Cluster ID	Cluster Size	Gene ID	Gene Name	Gene Description
CDNCD1D28-001	2	ENSG00000162645	GBP2	guanylate binding protein 2, interferon-inducible [Source:HGNC Symbol;Acc:4183]
CDNCD1D28-001	2	ENSG00000140105	WARS	tryptophanyl-tRNA synthetase [Source:HGNC Symbol;Acc:12729]
CDNCD1D28-002	2	ENSG00000162654	GBP4	guanylate binding protein 4 [Source:HGNC Symbol;Acc:20480]
CDNCD1D28-002	2	ENSG00000154451	GBP5	guanylate binding protein 5 [Source:HGNC Symbol;Acc:19895]
CDNCD1D28-003	2	ENSG00000158517	NCF1	neutrophil cytosolic factor 1 [Source:HGNC Symbol;Acc:7660]
CDNCD1D28-003	2	ENSG00000165178	NCF1C	neutrophil cytosolic factor 1C pseudogene [Source:HGNC Symbol;Acc:32523]
CDNCD1D28-004	2	ENSG00000115594	IL1R1	interleukin 1 receptor, type I [Source:HGNC Symbol;Acc:5993]
CDNCD1D28-004	2	ENSG00000115590	IL1R2	interleukin 1 receptor, type II [Source:HGNC Symbol;Acc:5994]
CDNCD1D28-005	2	ENSG00000059804	SLC2A3	solute carrier family 2 (facilitated glucose transporter), member 3 [Source:HGNC Symbol;Acc:11007]
CDNCD1D28-005	2	ENSG00000184557	SOCS3	suppressor of cytokine signaling 3 [Source:HGNC Symbol;Acc:19391]
CDNCD1D28-006	2	ENSG00000171695	C20orf201	chromosome 20 open reading frame 201 [Source:HGNC Symbol;Acc:33718]
CDNCD1D28-006	2	ENSG00000245848	CEBPA	CCAAT/enhancer binding protein (C/EBP), alpha [Source:HGNC Symbol;Acc:1833]

Table A61: Significant gene clusters (RNA-Seq, Dendritic cells, Day 1-28)

Cluster ID	Cluster Size	Gene ID	Gene Name	Gene Description	Log FC
CMNCD1-001	6	ENSG00000168062	BATF2	basic leucine zipper transcription factor, ATF-like 2 [Source:HGNC Symbol;Acc:25163]	2.15
CMNCD1-001	6	ENSG00000055130	CUL1	cullin 1 [Source:HGNC Symbol;Acc:2551]	0.96
CMNCD1-001	6	ENSG00000133106	EPSTI1	epithelial stromal interaction 1 (breast) [Source:HGNC Symbol;Acc:16465]	1.31
CMNCD1-001	6	ENSG00000240065	PSMB9	proteasome (prosome, macropain) subunit, beta type, 9 (large multifunctional peptidase 2) [Source:HGNC Symbol;Acc:9546]	0.84
CMNCD1-001	6	ENSG00000149131	SERPING1	serpin peptidase inhibitor, clade G (C1 inhibitor), member 1 [Source:HGNC Symbol;Acc:1228]	2.32
CMNCD1-001	6	ENSG00000156587	UBE2L6	ubiquitin-conjugating enzyme E2L 6 [Source:HGNC Symbol;Acc:12490]	1.02
CMNCD1-002	2	ENSG00000244682	FCGR2C	Fc fragment of IgG, low affinity IIc, receptor for (CD32) (gene/pseudogene) [Source:HGNC Symbol;Acc:15626]	0.60

Cluster ID	Cluster Size	Gene ID	Gene Name	Gene Description	Log FC
CMNCD1-002	2	ENSG00000225217	HSPA7	heat shock 70kDa protein 7 (HSP70B) [Source:HGNC Symbol;Acc:5240]	0.62
CMNCD1-003	5	ENSG00000137965	IFI44	interferon-induced protein 44 [Source:HGNC Symbol;Acc:16938]	0.76
CMNCD1-003	5	ENSG00000137959	IFI44L	interferon-induced protein 44-like [Source:HGNC Symbol;Acc:17817]	0.90
CMNCD1-003	5	ENSG00000157601	MX1	myxovirus (influenza virus) resistance 1, interferon-inducible protein p78 (mouse) [Source:HGNC Symbol;Acc:7532]	0.64
CMNCD1-003	5	ENSG00000111335	OAS2	2'-5'-oligoadenylate synthetase 2, 69/71kDa [Source:HGNC Symbol;Acc:8087]	0.71
CMNCD1-003	5	ENSG00000111331	OAS3	2'-5'-oligoadenylate synthetase 3, 100kDa [Source:HGNC Symbol;Acc:8088]	0.74
CMNCD1-004	2	ENSG00000101017	CD40	CD40 molecule, TNF receptor superfamily member 5 [Source:HGNC Symbol;Acc:11919]	1.05
CMNCD1-004	2	ENSG00000135114	OASL	2'-5'-oligoadenylate synthetase-like [Source:HGNC Symbol;Acc:8090]	0.72
CMNCD1-005	10	ENSG00000117228	GBP1	guanylate binding protein 1, interferon-inducible [Source:HGNC Symbol;Acc:4182]	2.67
CMNCD1-005	10	ENSG00000225492	GBP1P1	guanylate binding protein 1, interferon-inducible pseudogene 1 [Source:HGNC Symbol;Acc:39561]	3.52
CMNCD1-005	10	ENSG00000162645	GBP2	guanylate binding protein 2, interferon-inducible [Source:HGNC Symbol;Acc:4183]	1.65
CMNCD1-005	10	ENSG00000117226	GBP3	guanylate binding protein 3 [Source:HGNC Symbol;Acc:4184]	1.17
CMNCD1-005	10	ENSG00000162654	GBP4	guanylate binding protein 4 [Source:HGNC Symbol;Acc:20480]	2.19
CMNCD1-005	10	ENSG00000154451	GBP5	guanylate binding protein 5 [Source:HGNC Symbol;Acc:19895]	3.00
CMNCD1-005	10	ENSG00000183762	KREMEN1	kringle containing transmembrane protein 1 [Source:HGNC Symbol;Acc:17550]	1.95
CMNCD1-005	10	ENSG00000002549	LAP3	leucine aminopeptidase 3 [Source:HGNC Symbol;Acc:18449]	1.44
CMNCD1-005	10	ENSG00000138496	PARP9	poly (ADP-ribose) polymerase family, member 9 [Source:HGNC Symbol;Acc:24118]	1.14
CMNCD1-005	10	ENSG00000115415	STAT1	signal transducer and activator of transcription 1, 91kDa [Source:HGNC Symbol;Acc:11362]	1.46
CMNCD1-006	3	ENSG00000125347	IRF1	interferon regulatory factor 1 [Source:HGNC Symbol;Acc:6116]	1.04
CMNCD1-006	3	ENSG00000140853	NLRC5	NLR family, CARD domain containing 5 [Source:HGNC Symbol;Acc:29933]	0.77
CMNCD1-006	3	ENSG00000233029	RP11-439A17.9		2.03
CMNCD1-007	3	ENSG00000164308	ERAP2	endoplasmic reticulum aminopeptidase 2 [Source:HGNC Symbol;Acc:29499]	0.62
CMNCD1-007	3	ENSG00000115392	FANCL	Fanconi anemia, complementation group L [Source:HGNC Symbol;Acc:20748]	0.65
CMNCD1-007	3	ENSG00000136231	IGF2BP3	insulin-like growth factor 2 mRNA binding protein 3 [Source:HGNC Symbol;Acc:28868]	1.35
CMNCD1-008	5	ENSG00000169136	ATF5	activating transcription factor 5 [Source:HGNC Symbol;Acc:790]	0.59
CMNCD1-008	5	ENSG00000138119	MYOF	myoferlin [Source:HGNC Symbol;Acc:3656]	0.97

Cluster ID	Cluster Size	Gene ID	Gene Name	Gene Description	Log FC
CMNCD1-008	5	ENSG00000145287	PLAC8	placenta-specific 8 [Source:HGNC Symbol;Acc:19254]	1.11
CMNCD1-008	5	ENSG00000100911	PSME2	proteasome (prosome, macropain) activator subunit 2 (PA28 beta) [Source:HGNC Symbol;Acc:9569]	0.97
CMNCD1-008	5	ENSG00000026751	SLAMF7	SLAM family member 7 [Source:HGNC Symbol;Acc:21394]	2.01
CMNCD1-009	4	ENSG00000198087	CD2AP	CD2-associated protein [Source:HGNC Symbol;Acc:14258]	0.59
CMNCD1-009	4	ENSG00000134326	CMPK2	cytidine monophosphate (UMP-CMP) kinase 2, mitochondrial [Source:HGNC Symbol;Acc:27015]	0.74
CMNCD1-009	4	ENSG00000137628	DDX60	DEAD (Asp-Glu-Ala-Asp) box polypeptide 60 [Source:HGNC Symbol;Acc:25942]	0.91
CMNCD1-009	4	ENSG00000111181	SLC6A12	solute carrier family 6 (neurotransmitter transporter, betaine/GABA), member 12 [Source:HGNC Symbol;Acc:11045]	0.88
CMNCD1-010	6	ENSG00000145349	CAMK2D	calcium/calmodulin-dependent protein kinase II delta [Source:HGNC Symbol;Acc:1462]	0.69
CMNCD1-010	6	ENSG00000172159	FRMD3	FERM domain containing 3 [Source:HGNC Symbol;Acc:24125]	0.85
CMNCD1-010	6	ENSG00000138646	HERC5	hect domain and RLD 5 [Source:HGNC Symbol;Acc:24368]	0.94
CMNCD1-010	6	ENSG00000158517	NCF1	neutrophil cytosolic factor 1 [Source:HGNC Symbol;Acc:7660]	0.62
CMNCD1-010	6	ENSG00000165178	NCF1C	neutrophil cytosolic factor 1C pseudogene [Source:HGNC Symbol;Acc:32523]	0.64
CMNCD1-010	6	ENSG00000124256	ZBP1	Z-DNA binding protein 1 [Source:HGNC Symbol;Acc:16176]	0.81
CMNCD1-011	2	ENSG00000108691	CCL2	chemokine (C-C motif) ligand 2 [Source:HGNC Symbol;Acc:10618]	1.03
CMNCD1-011	2	ENSG00000171621	SPSB1	splA/ryanodine receptor domain and SOCS box containing 1 [Source:HGNC Symbol;Acc:30628]	0.62
CMNCD1-012	2	ENSG00000226321	AC104809.3		-0.93
CMNCD1-012	2	ENSG00000233392	AC104809.4		-0.95
CMNCD1-013	6	ENSG00000197381	ADARB1	adenosine deaminase, RNA-specific, B1 [Source:HGNC Symbol;Acc:226]	0.87
CMNCD1-013	6	ENSG00000103569	AQP9	aquaporin 9 [Source:HGNC Symbol;Acc:643]	0.82
CMNCD1-013	6	ENSG00000150347	ARID5B	AT rich interactive domain 5B (MRF1-like) [Source:HGNC Symbol;Acc:17362]	1.28
CMNCD1-013	6	ENSG00000257017	HP	haptoglobin [Source:HGNC Symbol;Acc:5141]	0.81
CMNCD1-013	6	ENSG00000105639	JAK3	Janus kinase 3 [Source:HGNC Symbol;Acc:6193]	0.72
CMNCD1-013	6	ENSG00000184557	SOCS3	suppressor of cytokine signaling 3 [Source:HGNC Symbol;Acc:19391]	1.31
CMNCD1-014	2	ENSG00000239899	AC011994.1		1.21
CMNCD1-014	2	ENSG00000244642	AP005717.1		1.11
CMNCD1-015	2	ENSG00000256579	AC135776.2		0.69
CMNCD1-015	2	ENSG00000122420	PTGFR	prostaglandin F receptor (FP) [Source:HGNC Symbol;Acc:9600]	0.61
CMNCD1-016	2	ENSG00000148926	ADM	adrenomedullin [Source:HGNC Symbol;Acc:259]	0.84
CMNCD1-016	2	ENSG00000167680	SEMA6B	sema domain, transmembrane domain (TM), and cytoplasmic domain, (semaphorin) 6B [Source:HGNC Symbol;Acc:10739]	0.81

Table A62: Significant gene clusters (RNA-Seq, Monocytes, Day 1)

Cluster ID	Cluster Size	Gene ID	Gene Name	Gene Description
CMNCD1D28-001	2	ENSG00000119922	IFIT2	interferon-induced protein with tetratricopeptide repeats 2 [Source:HGNC Symbol;Acc:5409]
CMNCD1D28-001	2	ENSG00000119917	IFIT3	interferon-induced protein with tetratricopeptide repeats 3 [Source:HGNC Symbol;Acc:5411]
CMNCD1D28-002	2	ENSG00000137965	IFI44	interferon-induced protein 44 [Source:HGNC Symbol;Acc:16938]
CMNCD1D28-002	2	ENSG00000137959	IFI44L	interferon-induced protein 44-like [Source:HGNC Symbol;Acc:17817]
CMNCD1D28-003	2	ENSG00000158517	NCF1	neutrophil cytosolic factor 1 [Source:HGNC Symbol;Acc:7660]
CMNCD1D28-003	2	ENSG00000165178	NCF1C	neutrophil cytosolic factor 1C pseudogene [Source:HGNC Symbol;Acc:32523]
CMNCD1D28-004	2	ENSG00000150337	FCGR1A	Fc fragment of IgG, high affinity Ia, receptor (CD64) [Source:HGNC Symbol;Acc:3613]
CMNCD1D28-004	2	ENSG00000198019	FCGR1B	Fc fragment of IgG, high affinity Ib, receptor (CD64) [Source:HGNC Symbol;Acc:3614]
CMNCD1D28-005	3	ENSG00000177989	ODF3B	outer dense fiber of sperm tails 3B [Source:HGNC Symbol;Acc:34388]
CMNCD1D28-005	3	ENSG00000130489	SCO2	SCO cytochrome oxidase deficient homolog 2 (yeast) [Source:HGNC Symbol;Acc:10604]
CMNCD1D28-005	3	ENSG00000025708	TYMP	thymidine phosphorylase [Source:HGNC Symbol;Acc:3148]
CMNCD1D28-006	2	ENSG00000221963	APOL6	apolipoprotein L, 6 [Source:HGNC Symbol;Acc:14870]
CMNCD1D28-006	2	ENSG00000173821	RNF213	ring finger protein 213 [Source:HGNC Symbol;Acc:14539]
CMNCD1D28-007	2	ENSG00000226321	AC104809.3	
CMNCD1D28-007	2	ENSG00000233392	AC104809.4	
CMNCD1D28-008	2	ENSG00000164308	ERAP2	endoplasmic reticulum aminopeptidase 2 [Source:HGNC Symbol;Acc:29499]
CMNCD1D28-008	2	ENSG00000115419	GLS	glutaminase [Source:HGNC Symbol;Acc:4331]
CMNCD1D28-009	2	ENSG00000239899	AC011994.1	
CMNCD1D28-009	2	ENSG00000244642	AP005717.1	
CMNCD1D28-010	11	ENSG00000144218	AFF3	AF4/FMR2 family, member 3 [Source:HGNC Symbol;Acc:6473]
CMNCD1D28-010	11	ENSG00000172232	AZU1	azurocidin 1 [Source:HGNC Symbol;Acc:913]
CMNCD1D28-010	11	ENSG00000116824	CD2	CD2 molecule [Source:HGNC Symbol;Acc:1639]
CMNCD1D28-010	11	ENSG00000049089	COL9A2	collagen, type IX, alpha 2 [Source:HGNC Symbol;Acc:2218]
CMNCD1D28-010	11	ENSG00000179639	FCER1A	Fc fragment of IgE, high affinity I, receptor for; alpha polypeptide [Source:HGNC Symbol;Acc:3609]
CMNCD1D28-010	11	ENSG00000213366	GSTM2	glutathione S-transferase mu 2 (muscle) [Source:HGNC Symbol;Acc:4634]
CMNCD1D28-010	11	ENSG00000104814	MAP4K1	mitogen-activated protein kinase kinase kinase kinase 1 [Source:HGNC Symbol;Acc:6863]
CMNCD1D28-010	11	ENSG00000165795	NDRG2	NDRG family member 2 [Source:HGNC Symbol;Acc:14460]
CMNCD1D28-010	11	ENSG00000166428	PLD4	phospholipase D family, member 4 [Source:HGNC Symbol;Acc:23792]
CMNCD1D28-010	11	ENSG00000103485	QPRT	quinolinate phosphoribosyltransferase [Source:HGNC Symbol;Acc:9755]
CMNCD1D28-010	11	ENSG00000182557	SPNS3	spinster homolog 3 (Drosophila) [Source:HGNC Symbol;Acc:28433]
CMNCD1D28-011	9	ENSG00000138031	ADCY3	adenylate cyclase 3 [Source:HGNC Symbol;Acc:234]
CMNCD1D28-011	9	ENSG00000137757	CASP5	caspase 5, apoptosis-related cysteine peptidase [Source:HGNC Symbol;Acc:1506]
CMNCD1D28-011	9	ENSG00000243649	CFB	complement factor B [Source:HGNC Symbol;Acc:1037]

Cluster ID	Cluster Size	Gene ID	Gene Name	Gene Description
CMNCD1D28-011	9	ENSG00000120885	CLU	clusterin [Source:HGNC Symbol;Acc:2095]
CMNCD1D28-011	9	ENSG00000108950	FAM20A	family with sequence similarity 20, member A [Source:HGNC Symbol;Acc:23015]
CMNCD1D28-011	9	ENSG00000183087	GAS6	growth arrest-specific 6 [Source:HGNC Symbol;Acc:4168]
CMNCD1D28-011	9	ENSG00000173110	HSPA6	heat shock 70kDa protein 6 (HSP70B') [Source:HGNC Symbol;Acc:5239]
CMNCD1D28-011	9	ENSG00000105639	JAK3	Janus kinase 3 [Source:HGNC Symbol;Acc:6193]
CMNCD1D28-011	9	ENSG00000104972	LILRB1	leukocyte immunoglobulin-like receptor, subfamily B (with TM and ITIM domains), member 1 [Source:HGNC Symbol;Acc:6605]
CMNCD1D28-012	7	ENSG00000151726	ACSL1	acyl-CoA synthetase long-chain family member 1 [Source:HGNC Symbol;Acc:3569]
CMNCD1D28-012	7	ENSG00000103569	AQP9	aquaporin 9 [Source:HGNC Symbol;Acc:643]
CMNCD1D28-012	7	ENSG00000150347	ARID5B	AT rich interactive domain 5B (MRF1-like) [Source:HGNC Symbol;Acc:17362]
CMNCD1D28-012	7	ENSG00000140379	BCL2A1	BCL2-related protein A1 [Source:HGNC Symbol;Acc:991]
CMNCD1D28-012	7	ENSG00000242616	GNG10	guanine nucleotide binding protein (G protein), gamma 10 [Source:HGNC Symbol;Acc:4402]
CMNCD1D28-012	7	ENSG00000257017	HP	haptoglobin [Source:HGNC Symbol;Acc:5141]
CMNCD1D28-012	7	ENSG00000184557	SOCS3	suppressor of cytokine signaling 3 [Source:HGNC Symbol;Acc:19391]

Table A63: Significant gene clusters (RNA-Seq, Monocytes, Day 1-28)

Cluster ID	Cluster Size	Gene ID	Gene Name	Gene Description	Log FC
CNEUD1-001	2	ENSG00000070501	POLB	polymerase (DNA directed), beta [Source:HGNC Symbol;Acc:9174]	0.65
CNEUD1-001	2	ENSG00000204267	TAP2	transporter 2, ATP-binding cassette, sub-family B (MDR/-TAP) [Source:HGNC Symbol;Acc:44]	0.91
CNEUD1-002	2	ENSG00000134326	CMPK2	cytidine monophosphate (UMP-CMP) kinase 2, mitochondrial [Source:HGNC Symbol;Acc:27015]	1.79
CNEUD1-002	2	ENSG00000119917	IFIT3	interferon-induced protein with tetratricopeptide repeats 3 [Source:HGNC Symbol;Acc:5411]	1.22
CNEUD1-003	2	ENSG00000092010	PSME1	proteasome (prosome, macropain) activator subunit 1 (PA28 alpha) [Source:HGNC Symbol;Acc:9568]	0.70
CNEUD1-003	2	ENSG00000034510	TMSB10	thymosin beta 10 [Source:HGNC Symbol;Acc:11879]	1.08
CNEUD1-004	2	ENSG00000137200	FTSJD2	FtsJ methyltransferase domain containing 2 [Source:HGNC Symbol;Acc:21077]	1.09
CNEUD1-004	2	ENSG00000059378	PARP12	poly (ADP-ribose) polymerase family, member 12 [Source:HGNC Symbol;Acc:21919]	0.87
CNEUD1-005	2	ENSG00000099860	GADD45B	growth arrest and DNA-damage-inducible, beta [Source:HGNC Symbol;Acc:4096]	0.94
CNEUD1-005	2	ENSG00000125347	IRF1	interferon regulatory factor 1 [Source:HGNC Symbol;Acc:6116]	0.94
CNEUD1-006	2	ENSG00000173239	LIPM	lipase, family member M [Source:HGNC Symbol;Acc:23455]	3.53
CNEUD1-006	2	ENSG00000135093	USP30	ubiquitin specific peptidase 30 [Source:HGNC Symbol;Acc:20065]	0.79
CNEUD1-007	8	ENSG00000138646	HERC5	hect domain and RLD 5 [Source:HGNC Symbol;Acc:24368]	1.00

Cluster ID	Cluster Size	Gene ID	Gene Name	Gene Description	Log FC
CNEUD1-007	8	ENSG00000137965	IFI44	interferon-induced protein 44 [Source:HGNC Symbol;Acc:16938]	1.58
CNEUD1-007	8	ENSG00000137959	IFI44L	interferon-induced protein 44-like [Source:HGNC Symbol;Acc:17817]	1.69
CNEUD1-007	8	ENSG00000089127	OAS1	2‘,5‘-oligoadenylate synthetase 1, 40/46kDa [Source:HGNC Symbol;Acc:8086]	2.02
CNEUD1-007	8	ENSG00000111335	OAS2	2‘-5‘-oligoadenylate synthetase 2, 69/71kDa [Source:HGNC Symbol;Acc:8087]	1.97
CNEUD1-007	8	ENSG00000111331	OAS3	2‘-5‘-oligoadenylate synthetase 3, 100kDa [Source:HGNC Symbol;Acc:8088]	1.65
CNEUD1-007	8	ENSG00000134321	RSAD2	radical S-adenosyl methionine domain containing 2 [Source:HGNC Symbol;Acc:30908]	1.75
CNEUD1-007	8	ENSG00000141664	ZCCHC2	zinc finger, CCHC domain containing 2 [Source:HGNC Symbol;Acc:22916]	0.63
CNEUD1-008	4	ENSG00000130303	BST2	bone marrow stromal cell antigen 2 [Source:HGNC Symbol;Acc:1119]	1.35
CNEUD1-008	4	ENSG00000104518	GSDMD	gasdermin D [Source:HGNC Symbol;Acc:25697]	0.63
CNEUD1-008	4	ENSG00000185507	IRF7	interferon regulatory factor 7 [Source:HGNC Symbol;Acc:6122]	0.91
CNEUD1-008	4	ENSG00000240065	PSMB9	proteasome (prosome, macropain) subunit, beta type, 9 (large multifunctional peptidase 2) [Source:HGNC Symbol;Acc:9546]	0.79
CNEUD1-009	2	ENSG00000138772	ANXA3	annexin A3 [Source:HGNC Symbol;Acc:541]	0.69
CNEUD1-009	2	ENSG00000187554	TLR5	toll-like receptor 5 [Source:HGNC Symbol;Acc:11851]	0.65
CNEUD1-010	3	ENSG00000137757	CASP5	caspase 5, apoptosis-related cysteine peptidase [Source:HGNC Symbol;Acc:1506]	1.08
CNEUD1-010	3	ENSG00000163565	IFI16	interferon, gamma-inducible protein 16 [Source:HGNC Symbol;Acc:5395]	1.01
CNEUD1-010	3	ENSG00000157551	KCNJ15	potassium inwardly-rectifying channel, subfamily J, member 15 [Source:HGNC Symbol;Acc:6261]	0.70
CNEUD1-011	5	ENSG00000075399	C16orf7	chromosome 16 open reading frame 7 [Source:HGNC Symbol;Acc:13526]	1.27
CNEUD1-011	5	ENSG00000197448	GSTK1	glutathione S-transferase kappa 1 [Source:HGNC Symbol;Acc:16906]	1.00
CNEUD1-011	5	ENSG00000158411	MITD1	MIT, microtubule interacting and transport, domain containing 1 [Source:HGNC Symbol;Acc:25207]	0.67
CNEUD1-011	5	ENSG00000141574	SECTM1	secreted and transmembrane 1 [Source:HGNC Symbol;Acc:10707]	0.99
CNEUD1-011	5	ENSG00000145365	TIFA	TRAF-interacting protein with forkhead-associated domain [Source:HGNC Symbol;Acc:19075]	1.05
CNEUD1-012	2	ENSG00000119950	MXI1	MAX interactor 1 [Source:HGNC Symbol;Acc:7534]	-0.66
CNEUD1-012	2	ENSG00000185989	RASA3	RAS p21 protein activator 3 [Source:HGNC Symbol;Acc:20331]	-0.71
CNEUD1-013	2	ENSG00000205730	ITPRIPL2	inositol 1,4,5-trisphosphate receptor interacting protein-like 2 [Source:HGNC Symbol;Acc:27257]	2.30
CNEUD1-013	2	ENSG00000067221	STOML1	stomatin (EPB72)-like 1 [Source:HGNC Symbol;Acc:14560]	1.62
CNEUD1-014	4	ENSG00000166002	C11orf75	chromosome 11 open reading frame 75 [Source:HGNC Symbol;Acc:24810]	1.11
CNEUD1-014	4	ENSG00000085063	CD59	CD59 molecule, complement regulatory protein [Source:HGNC Symbol;Acc:1689]	0.78

Cluster ID	Cluster Size	Gene ID	Gene Name	Gene Description	Log FC
CNEUD1-014	4	ENSG00000203666	EFCAB2	EF-hand calcium binding domain 2 [Source:HGNC Symbol;Acc:28166]	1.17
CNEUD1-014	4	ENSG00000170542	SERPINB9	serpin peptidase inhibitor, clade B (ovalbumin), member 9 [Source:HGNC Symbol;Acc:8955]	1.22
CNEUD1-015	3	ENSG00000114770	ABCC5	ATP-binding cassette, sub-family C (CFTR/MRP), member 5 [Source:HGNC Symbol;Acc:56]	-0.69
CNEUD1-015	3	ENSG00000058866	DGKG	diacylglycerol kinase, gamma 90kDa [Source:HGNC Symbol;Acc:2853]	-0.72
CNEUD1-015	3	ENSG00000236345	RP11-59D5__B.2		-0.61
CNEUD1-016	2	ENSG00000154188	ANGPT1	angiopoietin 1 [Source:HGNC Symbol;Acc:484]	-0.77
CNEUD1-016	2	ENSG00000151136	BTBD11	BTB (POZ) domain containing 11 [Source:HGNC Symbol;Acc:23844]	-0.62
CNEUD1-017	3	ENSG00000225131	PSME2P2	proteasome activator subunit 2 pseudogene 2 [Source:HGNC Symbol;Acc:30160]	1.41
CNEUD1-017	3	ENSG00000138760	SCARB2	scavenger receptor class B, member 2 [Source:HGNC Symbol;Acc:1665]	
CNEUD1-017	3	ENSG00000106785	TRIM14	tripartite motif containing 14 [Source:HGNC Symbol;Acc:16283]	
CNEUD1-018	6	ENSG00000133313	CNDP2	CNDP dipeptidase 2 (metallopeptidase M20 family) [Source:HGNC Symbol;Acc:24437]	1.09
CNEUD1-018	6	ENSG00000002549	LAP3	leucine aminopeptidase 3 [Source:HGNC Symbol;Acc:18449]	1.76
CNEUD1-018	6	ENSG00000135362	PRR5L	proline rich 5 like [Source:HGNC Symbol;Acc:25878]	0.98
CNEUD1-018	6	ENSG00000100401	RANGAP1	Ran GTPase activating protein 1 [Source:HGNC Symbol;Acc:9854]	1.09
CNEUD1-018	6	ENSG00000104312	RIPK2	receptor-interacting serine-threonine kinase 2 [Source:HGNC Symbol;Acc:10020]	0.91
CNEUD1-018	6	ENSG00000185338	SOCS1	suppressor of cytokine signaling 1 [Source:HGNC Symbol;Acc:19383]	2.20
CNEUD1-019	2	ENSG00000038210	PI4K2B	phosphatidylinositol 4-kinase type 2 beta [Source:HGNC Symbol;Acc:18215]	0.74
CNEUD1-019	2	ENSG00000155366	RHOC	ras homolog gene family, member C [Source:HGNC Symbol;Acc:669]	1.12
CNEUD1-020	2	ENSG00000226012	AP001434.2		0.64
CNEUD1-020	2	ENSG00000114541	FRMD4B	FERM domain containing 4B [Source:HGNC Symbol;Acc:24886]	0.82
CNEUD1-021	4	ENSG00000215068	AC025171.1		0.81
CNEUD1-021	4	ENSG00000064601	CTSA	cathepsin A [Source:HGNC Symbol;Acc:9251]	0.67
CNEUD1-021	4	ENSG00000100364	KIAA0930	KIAA0930 [Source:HGNC Symbol;Acc:1314]	0.62
CNEUD1-021	4	ENSG00000240342	RPS2P5	ribosomal protein S2 pseudogene 5 [Source:HGNC Symbol;Acc:31386]	0.63
CNEUD1-022	4	ENSG00000030110	BAK1	BCL2-antagonist/killer 1 [Source:HGNC Symbol;Acc:949]	0.59
CNEUD1-022	4	ENSG00000179044	EXOC3L1	exocyst complex component 3-like 1 [Source:HGNC Symbol;Acc:27540]	1.27
CNEUD1-022	4	ENSG00000131203	IDO1	indoleamine 2,3-dioxygenase 1 [Source:HGNC Symbol;Acc:6059]	3.70
CNEUD1-022	4	ENSG00000148450	MSRB2	methionine sulfoxide reductase B2 [Source:HGNC Symbol;Acc:17061]	0.63
CNEUD1-023	3	ENSG00000119686	AC007182.1	Feline leukemia virus subgroup C receptor-related protein 2 [Source:UniProtKB/Swiss-Prot;Acc:Q9UPI3]	2.37
CNEUD1-023	3	ENSG00000104626	ERI1	exoribonuclease 1 [Source:HGNC Symbol;Acc:23994]	0.68

Cluster ID	Cluster Size	Gene ID	Gene Name	Gene Description	Log FC
CNEUD1-023	3	ENSG00000234571	RP5-998N21.4		3.20
CNEUD1-024	6	ENSG00000234290	AC116366.6		0.68
CNEUD1-024	6	ENSG00000175643	C16orf75	chromosome 16 open reading frame 75 [Source:HGNC Symbol;Acc:28349]	1.33
CNEUD1-024	6	ENSG00000115392	FANCL	Fanconi anemia, complementation group L [Source:HGNC Symbol;Acc:20748]	1.13
CNEUD1-024	6	ENSG00000108375	RNF43	ring finger protein 43 [Source:HGNC Symbol;Acc:18505]	0.73
CNEUD1-024	6	ENSG00000251136	RP11-37B2.1		1.32
CNEUD1-024	6	ENSG00000202533	Y_RNA_202533	Y RNA [Source:RFAM;Acc:RF00019]	0.89
CNEUD1-025	5	ENSG00000216588	IGSF23	immunoglobulin superfamily, member 23 [Source:HGNC Symbol;Acc:40040]	-0.75
CNEUD1-025	5	ENSG00000174004	LRRC33	leucine rich repeat containing 33 [Source:HGNC Symbol;Acc:24613]	-0.73
CNEUD1-025	5	ENSG00000021762	OSBPL5	oxysterol binding protein-like 5 [Source:HGNC Symbol;Acc:16392]	-0.62
CNEUD1-025	5	ENSG00000149782	PLCB3	phospholipase C, beta 3 (phosphatidylinositol-specific) [Source:HGNC Symbol;Acc:9056]	-0.61
CNEUD1-025	5	ENSG00000161980	POLR3K	polymerase (RNA) III (DNA directed) polypeptide K, 12.3 kDa [Source:HGNC Symbol;Acc:14121]	-0.80
CNEUD1-026	6	ENSG00000143162	CREG1	cellular repressor of E1A-stimulated genes 1 [Source:HGNC Symbol;Acc:2351]	0.91
CNEUD1-026	6	ENSG00000107566	ERLIN1	ER lipid raft associated 1 [Source:HGNC Symbol;Acc:16947]	1.22
CNEUD1-026	6	ENSG00000164715	LMTK2	lemur tyrosine kinase 2 [Source:HGNC Symbol;Acc:17880]	0.73
CNEUD1-026	6	ENSG00000068971	PPP2R5B	protein phosphatase 2, regulatory subunit B‘, beta [Source:HGNC Symbol;Acc:9310]	0.89
CNEUD1-026	6	ENSG00000139832	RAB20	RAB20, member RAS oncogene family [Source:HGNC Symbol;Acc:18260]	0.93
CNEUD1-026	6	ENSG00000250608	RP11-933H2.4		0.89

Table A64: Significant gene clusters (RNA-Seq, Neutrophils, Day 1)

Cluster ID	Cluster Size	Gene ID	Gene Name	Gene Description	Log FC
CNEUD3-001	2	ENSG00000137628	DDX60	DEAD (Asp-Glu-Ala-Asp) box polypeptide 60 [Source:HGNC Symbol;Acc:25942]	1.58
CNEUD3-001	2	ENSG00000152778	IFIT5	interferon-induced protein with tetratricopeptide repeats 5 [Source:HGNC Symbol;Acc:13328]	1.10
CNEUD3-002	2	ENSG00000178685	PARP10	poly (ADP-ribose) polymerase family, member 10 [Source:HGNC Symbol;Acc:25895]	0.59
CNEUD3-002	2	ENSG00000124256	ZBP1	Z-DNA binding protein 1 [Source:HGNC Symbol;Acc:16176]	0.66
CNEUD3-003	6	ENSG00000138642	HERC6	hect domain and RLD 6 [Source:HGNC Symbol;Acc:26072]	1.08
CNEUD3-003	6	ENSG00000137965	IFI44	interferon-induced protein 44 [Source:HGNC Symbol;Acc:16938]	2.14
CNEUD3-003	6	ENSG00000137959	IFI44L	interferon-induced protein 44-like [Source:HGNC Symbol;Acc:17817]	2.37

Cluster ID	Cluster Size	Gene ID	Gene Name	Gene Description	Log FC
CNEUD3-003	6	ENSG00000111335	OAS2	2‘-5‘-oligoadenylate synthetase 2, 69/71kDa [Source:HGNC Symbol;Acc:8087]	2.86
CNEUD3-003	6	ENSG00000111331	OAS3	2‘-5‘-oligoadenylate synthetase 3, 100kDa [Source:HGNC Symbol;Acc:8088]	2.15
CNEUD3-003	6	ENSG00000141664	ZCCHC2	zinc finger, CCHC domain containing 2 [Source:HGNC Symbol;Acc:22916]	0.79
CNEUD3-004	5	ENSG00000126709	IFI6	interferon, alpha-inducible protein 6 [Source:HGNC Symbol;Acc:4054]	1.49
CNEUD3-004	5	ENSG00000187608	ISG15	ISG15 ubiquitin-like modifier [Source:HGNC Symbol;Acc:4053]	1.54
CNEUD3-004	5	ENSG00000160932	LY6E	lymphocyte antigen 6 complex, locus E [Source:HGNC Symbol;Acc:6727]	3.41
CNEUD3-004	5	ENSG00000125148	MT2A	metallothionein 2A [Source:HGNC Symbol;Acc:7406]	1.72
CNEUD3-004	5	ENSG00000089127	OAS1	2‘,5‘-oligoadenylate synthetase 1, 40/46kDa [Source:HGNC Symbol;Acc:8086]	2.69
CNEUD3-005	3	ENSG00000228318	AP001610.5		1.39
CNEUD3-005	3	ENSG00000181381	DDX60L	DEAD (Asp-Glu-Ala-Asp) box polypeptide 60-like [Source:HGNC Symbol;Acc:26429]	
CNEUD3-005	3	ENSG00000112343	TRIM38	tripartite motif containing 38 [Source:HGNC Symbol;Acc:10059]	
CNEUD3-006	3	ENSG00000130303	BST2	bone marrow stromal cell antigen 2 [Source:HGNC Symbol;Acc:1119]	0.99
CNEUD3-006	3	ENSG00000185885	IFITM1	interferon induced transmembrane protein 1 (9-27) [Source:HGNC Symbol;Acc:5412]	
CNEUD3-006	3	ENSG00000156587	UBE2L6	ubiquitin-conjugating enzyme E2L 6 [Source:HGNC Symbol;Acc:12490]	
CNEUD3-007	3	ENSG00000225964	AC017076.5		2.06
CNEUD3-007	3	ENSG00000106100	NOD1	nucleotide-binding oligomerization domain containing 1 [Source:HGNC Symbol;Acc:16390]	
CNEUD3-007	3	ENSG00000138760	SCARB2	scavenger receptor class B, member 2 [Source:HGNC Symbol;Acc:1665]	0.62
CNEUD3-008	3	ENSG00000078081	LAMP3	lysosomal-associated membrane protein 3 [Source:HGNC Symbol;Acc:14582]	0.97
CNEUD3-008	3	ENSG00000002549	LAP3	leucine aminopeptidase 3 [Source:HGNC Symbol;Acc:18449]	0.83
CNEUD3-008	3	ENSG00000013374	NUB1	negative regulator of ubiquitin-like proteins 1 [Source:HGNC Symbol;Acc:17623]	
CNEUD3-009	3	ENSG00000172159	FRMD3	FERM domain containing 3 [Source:HGNC Symbol;Acc:24125]	0.98
CNEUD3-009	3	ENSG00000184898	RBM43	RNA binding motif protein 43 [Source:HGNC Symbol;Acc:24790]	
CNEUD3-009	3	ENSG00000085449	WDFY1	WD repeat and FYVE domain containing 1 [Source:HGNC Symbol;Acc:20451]	
CNEUD3-010	4	ENSG00000168405	CMAHP	cytidine monophospho-N-acetylneuraminic acid hydroxylase, pseudogene [Source:HGNC Symbol;Acc:2098]	0.61
CNEUD3-010	4	ENSG00000023171	GRAMD1B	GRAM domain containing 1B [Source:HGNC Symbol;Acc:29214]	0.66
CNEUD3-010	4	ENSG00000140853	NLRC5	NLR family, CARD domain containing 5 [Source:HGNC Symbol;Acc:29933]	
CNEUD3-010	4	ENSG00000071575	TRIB2	tribbles homolog 2 (Drosophila) [Source:HGNC Symbol;Acc:30809]	1.68
CNEUD3-011	9	ENSG00000163568	AIM2	absent in melanoma 2 [Source:HGNC Symbol;Acc:357]	0.67

Cluster ID	Cluster Size	Gene ID	Gene Name	Gene Description	Log FC
CNEUD3-011	9	ENSG00000100342	APOL1	apolipoprotein L, 1 [Source:HGNC Symbol;Acc:618]	
CNEUD3-011	9	ENSG00000128335	APOL2	apolipoprotein L, 2 [Source:HGNC Symbol;Acc:619]	
CNEUD3-011	9	ENSG00000111801	BTN3A3	butyrophilin, subfamily 3, member A3 [Source:HGNC Symbol;Acc:1140]	0.68
CNEUD3-011	9	ENSG00000166801	FAM111A	family with sequence similarity 111, member A [Source:HGNC Symbol;Acc:24725]	
CNEUD3-011	9	ENSG00000106560	GIMAP2	GTPase, IMAP family member 2 [Source:HGNC Symbol;Acc:21789]	0.63
CNEUD3-011	9	ENSG00000168394	TAP1	transporter 1, ATP-binding cassette, sub-family B (MDR/-TAP) [Source:HGNC Symbol;Acc:43]	
CNEUD3-011	9	ENSG00000204267	TAP2	transporter 2, ATP-binding cassette, sub-family B (MDR/-TAP) [Source:HGNC Symbol;Acc:44]	
CNEUD3-011	9	ENSG00000132109	TRIM21	tripartite motif containing 21 [Source:HGNC Symbol;Acc:11312]	
CNEUD3-012	3	ENSG00000165732	DDX21	DEAD (Asp-Glu-Ala-Asp) box polypeptide 21 [Source:HGNC Symbol;Acc:2744]	-0.60
CNEUD3-012	3	ENSG00000090339	ICAM1	intercellular adhesion molecule 1 [Source:HGNC Symbol;Acc:5344]	
CNEUD3-012	3	ENSG00000185022	MAFF	v-maf musculoaponeurotic fibrosarcoma oncogene homolog F (avian) [Source:HGNC Symbol;Acc:6780]	
CNEUD3-013	4	ENSG00000251002	AE000661.37		0.72
CNEUD3-013	4	ENSG00000110848	CD69	CD69 molecule [Source:HGNC Symbol;Acc:1694]	
CNEUD3-013	4	ENSG00000150337	FCGR1A	Fc fragment of IgG, high affinity Ia, receptor (CD64) [Source:HGNC Symbol;Acc:3613]	0.61
CNEUD3-013	4	ENSG00000198019	FCGR1B	Fc fragment of IgG, high affinity Ib, receptor (CD64) [Source:HGNC Symbol;Acc:3614]	
CNEUD3-014	3	ENSG00000183347	GBP6	guanylate binding protein family, member 6 [Source:HGNC Symbol;Acc:25395]	0.89
CNEUD3-014	3	ENSG00000130487	KLHDC7B	kelch domain containing 7B [Source:HGNC Symbol;Acc:25145]	0.63
CNEUD3-014	3	ENSG00000070501	POLB	polymerase (DNA directed), beta [Source:HGNC Symbol;Acc:9174]	
CNEUD3-015	3	ENSG00000139679	LPAR6	lysophosphatidic acid receptor 6 [Source:HGNC Symbol;Acc:15520]	0.66
CNEUD3-015	3	ENSG00000100889	PCK2	phosphoenolpyruvate carboxykinase 2 (mitochondrial) [Source:HGNC Symbol;Acc:8725]	0.86
CNEUD3-015	3	ENSG00000138385	SSB	Sjogren syndrome antigen B (autoantigen La) [Source:HGNC Symbol;Acc:11316]	

Table A65: Significant gene clusters (RNA-Seq, Neutrophils, Day 3)

Cluster ID	Cluster Size	Gene ID	Gene Name	Gene Description
CNEUD1D28-001	2	ENSG00000117228	GBP1	guanylate binding protein 1, interferon-inducible [Source:HGNC Symbol;Acc:4182]
CNEUD1D28-001	2	ENSG00000154451	GBP5	guanylate binding protein 5 [Source:HGNC Symbol;Acc:19895]
CNEUD1D28-002	2	ENSG00000100342	APOL1	apolipoprotein L, 1 [Source:HGNC Symbol;Acc:618]
CNEUD1D28-002	2	ENSG00000128335	APOL2	apolipoprotein L, 2 [Source:HGNC Symbol;Acc:619]
CNEUD1D28-003	2	ENSG00000119950	MXI1	MAX interactor 1 [Source:HGNC Symbol;Acc:7534]
CNEUD1D28-003	2	ENSG00000185989	RASA3	RAS p21 protein activator 3 [Source:HGNC Symbol;Acc:20331]

Cluster ID	Cluster Size	Gene ID	Gene Name	Gene Description
CNEUD1D28-004	2	ENSG00000236525	AC007278.2	
CNEUD1D28-004	2	ENSG00000234389	AC007278.3	
CNEUD1D28-005	7	ENSG00000197142	ACSL5	acyl-CoA synthetase long-chain family member 5 [Source:HGNC Symbol;Acc:16526]
CNEUD1D28-005	7	ENSG00000157693	C9orf91	chromosome 9 open reading frame 91 [Source:HGNC Symbol;Acc:24513]
CNEUD1D28-005	7	ENSG00000079385	CEACAM1	carcinoembryonic antigen-related cell adhesion molecule 1 (biliary glycoprotein) [Source:HGNC Symbol;Acc:1814]
CNEUD1D28-005	7	ENSG00000164308	ERAP2	endoplasmic reticulum aminopeptidase 2 [Source:HGNC Symbol;Acc:29499]
CNEUD1D28-005	7	ENSG00000166801	FAM111A	family with sequence similarity 111, member A [Source:HGNC Symbol;Acc:24725]
CNEUD1D28-005	7	ENSG00000070501	POLB	polymerase (DNA directed), beta [Source:HGNC Symbol;Acc:9174]
CNEUD1D28-005	7	ENSG00000085449	WDFY1	WD repeat and FYVE domain containing 1 [Source:HGNC Symbol;Acc:20451]
CNEUD1D28-006	2	ENSG00000090339	ICAM1	intercellular adhesion molecule 1 [Source:HGNC Symbol;Acc:5344]
CNEUD1D28-006	2	ENSG00000185022	MAFF	v-maf musculoaponeurotic fibrosarcoma oncogene homolog F (avian) [Source:HGNC Symbol;Acc:6780]
CNEUD1D28-007	2	ENSG00000226012	AP001434.2	
CNEUD1D28-007	2	ENSG00000237135	RP11-330O11.2	

Table A66: Significant gene clusters (RNA-Seq, Neutrophils, Day 1-28)

Cluster ID	Cluster Size	Gene ID	Gene Name	Gene Description	Log FC
CNKCD1-001	2	ENSG00000116016	EPAS1	endothelial PAS domain protein 1 [Source:HGNC Symbol;Acc:3374]	0.95
CNKCD1-001	2	ENSG00000121621	KIF18A	kinesin family member 18A [Source:HGNC Symbol;Acc:29441]	
CNKCD1-002	19	ENSG00000247275	AL160008.1		0.76
CNKCD1-002	19	ENSG00000125462	C1orf61	chromosome 1 open reading frame 61 [Source:HGNC Symbol;Acc:30780]	0.69
CNKCD1-002	19	ENSG00000087237	CETP	cholesteryl ester transfer protein, plasma [Source:HGNC Symbol;Acc:1869]	0.99
CNKCD1-002	19	ENSG00000134326	CMPK2	cytidine monophosphate (UMP-CMP) kinase 2, mitochondrial [Source:HGNC Symbol;Acc:27015]	0.60
CNKCD1-002	19	ENSG00000184371	CSF1	colony stimulating factor 1 (macrophage) [Source:HGNC Symbol;Acc:2432]	1.36
CNKCD1-002	19	ENSG00000133106	EPSTI1	epithelial stromal interaction 1 (breast) [Source:HGNC Symbol;Acc:16465]	0.67
CNKCD1-002	19	ENSG00000010030	ETV7	ets variant 7 [Source:HGNC Symbol;Acc:18160]	1.62
CNKCD1-002	19	ENSG00000117228	GBP1	guanylate binding protein 1, interferon-inducible [Source:HGNC Symbol;Acc:4182]	1.21
CNKCD1-002	19	ENSG00000140511	HAPLN3	hyaluronan and proteoglycan link protein 3 [Source:HGNC Symbol;Acc:21446]	1.02
CNKCD1-002	19	ENSG00000137959	IFI44L	interferon-induced protein 44-like [Source:HGNC Symbol;Acc:17817]	1.09
CNKCD1-002	19	ENSG00000119917	IFIT3	interferon-induced protein with tetratricopeptide repeats 3 [Source:HGNC Symbol;Acc:5411]	0.61

Cluster ID	Cluster Size	Gene ID	Gene Name	Gene Description	Log FC
CNKCD1-002	19	ENSG00000125347	IRF1	interferon regulatory factor 1 [Source:HGNC Symbol;Acc:6116]	0.66
CNKCD1-002	19	ENSG00000120254	MTHFD1L	methylenetetrahydrofolate dehydrogenase (NADP+ dependent) 1-like [Source:HGNC Symbol;Acc:21055]	0.65
CNKCD1-002	19	ENSG00000111331	OAS3	2‘-5‘-oligoadenylate synthetase 3, 100kDa [Source:HGNC Symbol;Acc:8088]	0.61
CNKCD1-002	19	ENSG00000177989	ODF3B	outer dense fiber of sperm tails 3B [Source:HGNC Symbol;Acc:34388]	0.72
CNKCD1-002	19	ENSG00000138496	PARP9	poly (ADP-ribose) polymerase family, member 9 [Source:HGNC Symbol;Acc:24118]	0.76
CNKCD1-002	19	ENSG00000170476	RP11-1280I22.1	Plasma cell-induced resident endoplasmic reticulum protein [Source:UniProtKB/Swiss-Prot;Acc:Q8WU39]	0.64
CNKCD1-002	19	ENSG00000115415	STAT1	signal transducer and activator of transcription 1, 91kDa [Source:HGNC Symbol;Acc:11362]	1.23
CNKCD1-002	19	ENSG00000025708	TYMP	thymidine phosphorylase [Source:HGNC Symbol;Acc:3148]	0.73

Table A67: Significant gene clusters (RNA-Seq, NK-cells, Day 1)

Cluster ID	Cluster Size	Gene ID	Gene Name	Gene Description	Log FC
CNKCD3-001	3	ENSG00000101412	E2F1	E2F transcription factor 1 [Source:HGNC Symbol;Acc:3113]	0.86
CNKCD3-001	3	ENSG00000104738	MCM4	minichromosome maintenance complex component 4 [Source:HGNC Symbol;Acc:6947]	0.60
CNKCD3-001	3	ENSG00000051341	POLQ	polymerase (DNA directed), theta [Source:HGNC Symbol;Acc:9186]	0.78
CNKCD3-002	5	ENSG00000237649	KIFC1	kinesin family member C1 [Source:HGNC Symbol;Acc:6389]	1.14
CNKCD3-002	5	ENSG00000121152	NCAPH	non-SMC condensin I complex, subunit H [Source:HGNC Symbol;Acc:1112]	0.77
CNKCD3-002	5	ENSG00000137804	NUSAP1	nucleolar and spindle associated protein 1 [Source:HGNC Symbol;Acc:18538]	0.97
CNKCD3-002	5	ENSG00000117632	STMN1	stathmin 1 [Source:HGNC Symbol;Acc:6510]	0.64
CNKCD3-002	5	ENSG00000176890	TYMS	thymidylate synthetase [Source:HGNC Symbol;Acc:12441]	0.89
CNKCD3-003	10	ENSG00000066279	ASPM	asp (abnormal spindle) homolog, microcephaly associated (Drosophila) [Source:HGNC Symbol;Acc:19048]	1.18
CNKCD3-003	10	ENSG00000089685	BIRC5	baculoviral IAP repeat containing 5 [Source:HGNC Symbol;Acc:593]	1.02
CNKCD3-003	10	ENSG00000169679	BUB1	budding uninhibited by benzimidazoles 1 homolog (yeast) [Source:HGNC Symbol;Acc:1148]	0.85
CNKCD3-003	10	ENSG00000117399	CDC20	cell division cycle 20 homolog (S. cerevisiae) [Source:HGNC Symbol;Acc:1723]	1.10
CNKCD3-003	10	ENSG00000117724	CENPF	centromere protein F, 350/400kDa (mitosin) [Source:HGNC Symbol;Acc:1857]	0.89
CNKCD3-003	10	ENSG00000075218	GTSE1	G-2 and S-phase expressed 1 [Source:HGNC Symbol;Acc:13698]	0.70
CNKCD3-003	10	ENSG00000148773	MKI67	antigen identified by monoclonal antibody Ki-67 [Source:HGNC Symbol;Acc:7107]	1.01
CNKCD3-003	10	ENSG00000165480	SKA3	spindle and kinetochore associated complex subunit 3 [Source:HGNC Symbol;Acc:20262]	1.06

Cluster ID	Cluster Size	Gene ID	Gene Name	Gene Description	Log FC
CNKCD3-003	10	ENSG00000131747	TOP2A	topoisomerase (DNA) II alpha 170kDa [Source:HGNC Symbol;Acc:11989]	1.17
CNKCD3-003	10	ENSG00000088325	TPX2	TPX2, microtubule-associated, homolog (Xenopus laevis) [Source:HGNC Symbol;Acc:1249]	0.96

Table A68: Significant gene clusters (RNA-Seq, NK-cells, Day 3)

Cluster ID	Cluster Size	Gene ID	Gene Name	Gene Description	Log FC
CNKCD28-001	2	ENSG00000196189	SEMA4A	sema domain, immunoglobulin domain (Ig), transmembrane domain (TM) and short cytoplasmic domain, (semaphorin) 4A [Source:HGNC Symbol;Acc:10729]	-1.10
CNKCD28-001	2	ENSG00000196562	SULF2	sulfatase 2 [Source:HGNC Symbol;Acc:20392]	-0.97
CNKCD28-002	3	ENSG00000214212	C19orf38	chromosome 19 open reading frame 38 [Source:HGNC Symbol;Acc:34073]	-1.04
CNKCD28-002	3	ENSG00000216490	IFI30	interferon, gamma-inducible protein 30 [Source:HGNC Symbol;Acc:5398]	-1.09
CNKCD28-002	3	ENSG00000185215	TNFAIP2	tumor necrosis factor, alpha-induced protein 2 [Source:HGNC Symbol;Acc:11895]	-1.46
CNKCD28-003	4	ENSG00000105383	CD33	CD33 molecule [Source:HGNC Symbol;Acc:1659]	-1.16
CNKCD28-003	4	ENSG00000239998	LILRA2	leukocyte immunoglobulin-like receptor, subfamily A (with TM domain), member 2 [Source:HGNC Symbol;Acc:6603]	-1.41
CNKCD28-003	4	ENSG00000112799	LY86	lymphocyte antigen 86 [Source:HGNC Symbol;Acc:16837]	-1.28
CNKCD28-003	4	ENSG00000035862	TIMP2	TIMP metallopeptidase inhibitor 2 [Source:HGNC Symbol;Acc:11821]	-0.94
CNKCD28-004	2	ENSG00000187796	CARD9	caspase recruitment domain family, member 9 [Source:HGNC Symbol;Acc:16391]	-0.80
CNKCD28-004	2	ENSG00000109113	RAB34	RAB34, member RAS oncogene family [Source:HGNC Symbol;Acc:16519]	-1.08
CNKCD28-005	3	ENSG00000101439	CST3	cystatin C [Source:HGNC Symbol;Acc:2475]	-0.89
CNKCD28-005	3	ENSG00000103811	CTSH	cathepsin H [Source:HGNC Symbol;Acc:2535]	-0.94
CNKCD28-005	3	ENSG00000025708	TYMP	thymidine phosphorylase [Source:HGNC Symbol;Acc:3148]	
CNKCD28-006	8	ENSG00000146192	FGD2	FYVE, RhoGEF and PH domain containing 2 [Source:HGNC Symbol;Acc:3664]	-0.88
CNKCD28-006	8	ENSG00000204681	GABBR1	gamma-aminobutyric acid (GABA) B receptor, 1 [Source:HGNC Symbol;Acc:4070]	-0.67
CNKCD28-006	8	ENSG00000175857	GAPT	GRB2-binding adaptor protein, transmembrane [Source:HGNC Symbol;Acc:26588]	-1.11
CNKCD28-006	8	ENSG00000242574	HLA-DMB	major histocompatibility complex, class II, DM beta [Source:HGNC Symbol;Acc:4935]	-0.69
CNKCD28-006	8	ENSG00000231389	HLA-DPA1	major histocompatibility complex, class II, DP alpha 1 [Source:HGNC Symbol;Acc:4938]	-0.61
CNKCD28-006	8	ENSG00000204287	HLA-DRA	major histocompatibility complex, class II, DR alpha [Source:HGNC Symbol;Acc:4947]	-1.13
CNKCD28-006	8	ENSG00000196126	HLA-DRB1	major histocompatibility complex, class II, DR beta 1 [Source:HGNC Symbol;Acc:4948]	-0.84

Cluster ID	Cluster Size	Gene ID	Gene Name	Gene Description	Log FC
CNKCD28-006	8	ENSG00000167207	NOD2	nucleotide-binding oligomerization domain containing 2 [Source:HGNC Symbol;Acc:5331]	-0.72
CNKCD28-007	6	ENSG00000177706	FAM20C	family with sequence similarity 20, member C [Source:HGNC Symbol;Acc:22140]	-1.08
CNKCD28-007	6	ENSG00000179344	HLA-DQB1	major histocompatibility complex, class II, DQ beta 1 [Source:HGNC Symbol;Acc:4944]	-0.94
CNKCD28-007	6	ENSG00000173801	JUP	junction plakoglobin [Source:HGNC Symbol;Acc:6207]	-1.14
CNKCD28-007	6	ENSG00000154237	LRRK1	leucine-rich repeat kinase 1 [Source:HGNC Symbol;Acc:18608]	-0.96
CNKCD28-007	6	ENSG00000138119	MYOF	myoferlin [Source:HGNC Symbol;Acc:3656]	-1.30
CNKCD28-007	6	ENSG00000183023	SLC8A1	solute carrier family 8 (sodium/calcium exchanger), member 1 [Source:HGNC Symbol;Acc:11068]	-1.44
CNKCD28-008	3	ENSG00000173083	HPSE	heparanase [Source:HGNC Symbol;Acc:5164]	-0.62
CNKCD28-008	3	ENSG00000238113	RP11-262H14.1		-0.74
CNKCD28-008	3	ENSG00000075223	SEMA3C	sema domain, immunoglobulin domain (Ig), short basic domain, secreted, (semaphorin) 3C [Source:HGNC Symbol;Acc:10725]	-0.88

Table A69: Significant gene clusters (RNA-Seq, NK-cells, Day 28)

Cluster ID	Cluster Size	Gene ID	Gene Name	Gene Description
CNKCD1D28-001	2	ENSG00000138496	PARP9	poly (ADP-ribose) polymerase family, member 9 [Source:HGNC Symbol;Acc:24118]
CNKCD1D28-001	2	ENSG00000115415	STAT1	signal transducer and activator of transcription 1, 91kDa [Source:HGNC Symbol;Acc:11362]
CNKCD1D28-002	2	ENSG00000177989	ODF3B	outer dense fiber of sperm tails 3B [Source:HGNC Symbol;Acc:34388]
CNKCD1D28-002	2	ENSG00000025708	TYMP	thymidine phosphorylase [Source:HGNC Symbol;Acc:3148]
CNKCD1D28-003	3	ENSG00000105383	CD33	CD33 molecule [Source:HGNC Symbol;Acc:1659]
CNKCD1D28-003	3	ENSG00000239998	LILRA2	leukocyte immunoglobulin-like receptor, subfamily A (with TM domain), member 2 [Source:HGNC Symbol;Acc:6603]
CNKCD1D28-003	3	ENSG00000035862	TIMP2	TIMP metallopeptidase inhibitor 2 [Source:HGNC Symbol;Acc:11821]

Table A70: Significant gene clusters (RNA-Seq, NK-cells, Day 1-28)

Cluster ID	Cluster Size	Gene ID	Gene Name	Gene Description	Log FC
CTCLD1-001	2	ENSG00000135148	TRAFD1	TRAF-type zinc finger domain containing 1 [Source:HGNC Symbol;Acc:24808]	0.60
CTCLD1-001	2	ENSG00000025708	TYMP	thymidine phosphorylase [Source:HGNC Symbol;Acc:3148]	0.94
CTCLD1-002	7	ENSG00000133106	EPSTI1	epithelial stromal interaction 1 (breast) [Source:HGNC Symbol;Acc:16465]	0.99
CTCLD1-002	7	ENSG00000117228	GBP1	guanylate binding protein 1, interferon-inducible [Source:HGNC Symbol;Acc:4182]	1.24

Cluster ID	Cluster Size	Gene ID	Gene Name	Gene Description	Log FC
CTCLD1-002	7	ENSG00000162645	GBP2	guanylate binding protein 2, interferon-inducible [Source:HGNC Symbol;Acc:4183]	0.62
CTCLD1-002	7	ENSG00000162654	GBP4	guanylate binding protein 4 [Source:HGNC Symbol;Acc:20480]	0.87
CTCLD1-002	7	ENSG00000138496	PARP9	poly (ADP-ribose) polymerase family, member 9 [Source:HGNC Symbol;Acc:24118]	1.12
CTCLD1-002	7	ENSG00000115415	STAT1	signal transducer and activator of transcription 1, 91kDa [Source:HGNC Symbol;Acc:11362]	1.43
CTCLD1-002	7	ENSG00000156587	UBE2L6	ubiquitin-conjugating enzyme E2L 6 [Source:HGNC Symbol;Acc:12490]	0.66
CTCLD1-003	3	ENSG00000134326	CMPK2	cytidine monophosphate (UMP-CMP) kinase 2, mitochondrial [Source:HGNC Symbol;Acc:27015]	0.78
CTCLD1-003	3	ENSG00000119917	IFIT3	interferon-induced protein with tetratricopeptide repeats 3 [Source:HGNC Symbol;Acc:5411]	1.34
CTCLD1-003	3	ENSG00000134321	RSAD2	radical S-adenosyl methionine domain containing 2 [Source:HGNC Symbol;Acc:30908]	0.77
CTCLD1-004	3	ENSG00000225492	GBP1P1	guanylate binding protein 1, interferon-inducible pseudogene 1 [Source:HGNC Symbol;Acc:39561]	2.04
CTCLD1-004	3	ENSG00000090339	ICAM1	intercellular adhesion molecule 1 [Source:HGNC Symbol;Acc:5344]	0.73
CTCLD1-004	3	ENSG00000125347	IRF1	interferon regulatory factor 1 [Source:HGNC Symbol;Acc:6116]	1.00

Table A71: Significant gene clusters (RNA-Seq, T-cells, Day 1)

Cluster ID	Cluster Size	Gene ID	Gene Name	Gene Description
CTCLD1D28-001	3	ENSG00000068079	IFI35	interferon-induced protein 35 [Source:HGNC Symbol;Acc:5399]
CTCLD1D28-001	3	ENSG00000135148	TRAFD1	TRAF-type zinc finger domain containing 1 [Source:HGNC Symbol;Acc:24808]
CTCLD1D28-001	3	ENSG00000156587	UBE2L6	ubiquitin-conjugating enzyme E2L 6 [Source:HGNC Symbol;Acc:12490]
CTCLD1D28-002	17	ENSG00000134326	CMPK2	cytidine monophosphate (UMP-CMP) kinase 2, mitochondrial [Source:HGNC Symbol;Acc:27015]
CTCLD1D28-002	17	ENSG00000163840	DTX3L	deltex 3-like (Drosophila) [Source:HGNC Symbol;Acc:30323]
CTCLD1D28-002	17	ENSG00000133106	EPSTI1	epithelial stromal interaction 1 (breast) [Source:HGNC Symbol;Acc:16465]
CTCLD1D28-002	17	ENSG00000010030	ETV7	ets variant 7 [Source:HGNC Symbol;Acc:18160]
CTCLD1D28-002	17	ENSG00000117228	GBP1	guanylate binding protein 1, interferon-inducible [Source:HGNC Symbol;Acc:4182]
CTCLD1D28-002	17	ENSG00000162645	GBP2	guanylate binding protein 2, interferon-inducible [Source:HGNC Symbol;Acc:4183]
CTCLD1D28-002	17	ENSG00000162654	GBP4	guanylate binding protein 4 [Source:HGNC Symbol;Acc:20480]
CTCLD1D28-002	17	ENSG00000154451	GBP5	guanylate binding protein 5 [Source:HGNC Symbol;Acc:19895]
CTCLD1D28-002	17	ENSG00000137965	IFI44	interferon-induced protein 44 [Source:HGNC Symbol;Acc:16938]
CTCLD1D28-002	17	ENSG00000137959	IFI44L	interferon-induced protein 44-like [Source:HGNC Symbol;Acc:17817]

Cluster ID	Cluster Size	Gene ID	Gene Name	Gene Description
CTCLD1D28-002	17	ENSG00000119917	IFIT3	interferon-induced protein with tetratricopeptide repeats 3 [Source:HGNC Symbol;Acc:5411]
CTCLD1D28-002	17	ENSG00000089127	OAS1	2‘,5‘-oligoadenylate synthetase 1, 40/46kDa [Source:HGNC Symbol;Acc:8086]
CTCLD1D28-002	17	ENSG00000138496	PARP9	poly (ADP-ribose) polymerase family, member 9 [Source:HGNC Symbol;Acc:24118]
CTCLD1D28-002	17	ENSG00000134321	RSAD2	radical S-adenosyl methionine domain containing 2 [Source:HGNC Symbol;Acc:30908]
CTCLD1D28-002	17	ENSG00000177409	SAMD9L	sterile alpha motif domain containing 9-like [Source:HGNC Symbol;Acc:1349]
CTCLD1D28-002	17	ENSG00000115415	STAT1	signal transducer and activator of transcription 1, 91kDa [Source:HGNC Symbol;Acc:11362]
CTCLD1D28-002	17	ENSG00000132530	XAF1	XIAP associated factor 1 [Source:HGNC Symbol;Acc:30932]

Table A72: Significant gene clusters (RNA-Seq, T-cells, Day 1-28)

Category Type	Categories	Distinct #Genes In Sets	Median #Genes Per Set
MSigDB BioCarta Pathways	217	1228	18
MSigDB Chemical/Genetic Perturbations	3398	18288	44
MSigDB GO Biological Processes	825	5914	26
MSigDB GO Cellular Components	233	5065	27
MSigDB GO Molecular Functions	396	5108	26
MSigDB Immunologic Signatures	1910	17782	193
KEGG Modules	162	1075	6
KEGG Pathways	286	6107	58
MSigDB Reactome Pathways	674	5595	26

Table A73: Overview of gene sets used for the enrichment analysis (RNA-Seq).

Cell Type (Study Visit Day)	KEGGP n/N(%)	KEGGM n/N(%)	GOBP n/N(%)	GOCC n/N(%)	GOMF n/N(%)	REACP n/N(%)	BIOCP n/N(%)	CHEMG n/N(%)	IMMO n/N(%)
B-cells (Day 1)	5/14(36)	2/14(14)	6/14(43)	2/14(14)	5/14(36)	8/14(57)	2/14(14)	14/14(100)	14/14(100)
Dendritic cells (Day 1)	68/225(30)	11/225(5)	83/225(37)	54/225(24)	63/225(28)	80/225(36)	29/225(13)	190/225(84)	187/225(83)
Dendritic cells (Day 3)	8/13(62)	0/13(0)	7/13(54)	6/13(46)	5/13(38)	5/13(38)	3/13(23)	13/13(100)	13/13(100)
Monocytes (Day 1)	131/406(32)	16/406(4)	155/406(38)	113/406(28)	123/406(30)	137/406(34)	40/406(10)	336/406(83)	334/406(82)
Neutrophils (Day 1)	196/572(34)	40/572(7)	186/572(33)	169/572(30)	162/572(28)	182/572(32)	45/572(8)	499/572(87)	497/572(87)
Neutrophils (Day 3)	37/140(26)	5/140(4)	39/140(28)	40/140(29)	25/140(18)	41/140(29)	4/140(3)	124/140(89)	123/140(88)
NK-cells (Day 1)	11/24(46)	3/24(12)	11/24(46)	8/24(33)	8/24(33)	9/24(38)	3/24(12)	21/24(88)	21/24(88)
NK-cells (Day 3)	25/70(36)	2/70(3)	33/70(47)	27/70(39)	23/70(33)	33/70(47)	10/70(14)	68/70(97)	68/70(97)
NK-cells (Day 28)	29/63(46)	2/63(3)	25/63(40)	26/63(41)	26/63(41)	26/63(41)	6/63(10)	61/63(97)	60/63(95)
T-cells (Day 1)	18/49(37)	3/49(6)	21/49(43)	16/49(33)	17/49(35)	19/49(39)	6/49(12)	44/49(90)	45/49(92)

Table A74: Overview of differential genes that mapped to any gene set by category (RNA-Seq). KEGGP: KEGG Pathways, KEGGM: KEGG Modules, GOBP: GO Biological Processes, GOCC: GO Cellular Components, GOMF: GO Molecular Functions, REACP: Reactome Pathways, BIOCP: BioCarta Pathways, CHEMG: Chemical/Genetic Perturbations, IMMO: Immunologic Signatures. n=#mapped SDEG, N=#SDEG.

Category Name	Category Genes #	Any-reg. SDEG #(%)	Up-reg. SDEG #(%)	Down-reg. SDEG #(%)	P	FDR
NUYTTEN EZH2 TARGETS UP	1002	9 (0.9)	9 (0.9)	0 (0)	<0.0001	0.0012
ZWANG TRANSIENTLY UP BY 1ST EGF PULSE ONLY	1723	6 (0.3)	6 (0.3)	0 (0)	<0.0001	0.0012
ICHIBA GRAFT VERSUS HOST DISEASE D7 UP	105	5 (4.8)	5 (4.8)	0 (0)	<0.0001	0.0012
MARKEY RB1 ACUTE LOF UP	214	5 (2.3)	5 (2.3)	0 (0)	<0.0001	0.0012
WALLACE PROSTATE CANCER RACE UP	275	5 (1.8)	5 (1.8)	0 (0)	<0.0001	0.0012
BERTUCCI MEDULLARY VS DUCTAL BREAST CANCER UP	200	4 (2)	4 (2)	0 (0)	<0.0001	0.0012
BROWNE INTERFERON RESPONSIVE GENES	68	4 (5.9)	4 (5.9)	0 (0)	<0.0001	0.0012
HECKER IFNB1 TARGETS	92	4 (4.3)	4 (4.3)	0 (0)	<0.0001	0.0012
JISON SICKLE CELL DISEASE UP	174	4 (2.3)	4 (2.3)	0 (0)	<0.0001	0.0012
MONNIER POSTRADIATION TUMOR ESCAPE DN	357	4 (1.1)	4 (1.1)	0 (0)	<0.0001	0.0012
SANA RESPONSE TO IFNG UP	74	4 (5.4)	4 (5.4)	0 (0)	<0.0001	0.0012
TAKEDA TARGETS OF NUP98 HOXA9 FUSION 10D UP	186	4 (2.2)	4 (2.2)	0 (0)	<0.0001	0.0012
TAKEDA TARGETS OF NUP98 HOXA9 FUSION 3D UP	178	4 (2.2)	4 (2.2)	0 (0)	<0.0001	0.0012
WIELAND UP BY HBV INFECTION	100	4 (4)	4 (4)	0 (0)	<0.0001	0.0012
ALTEMEIER RESPONSE TO LPS WITH MECHANICAL VENTILATION	126	3 (2.4)	3 (2.4)	0 (0)	<0.0001	0.0012
BOSCO INTERFERON INDUCED ANTIVIRAL MODULE	76	3 (3.9)	3 (3.9)	0 (0)	<0.0001	0.0012
DER IFN ALPHA RESPONSE UP	74	3 (4.1)	3 (4.1)	0 (0)	<0.0001	0.0012
DER IFN BETA RESPONSE UP	101	3 (3)	3 (3)	0 (0)	<0.0001	0.0012
DER IFN GAMMA RESPONSE UP	72	3 (4.2)	3 (4.2)	0 (0)	<0.0001	0.0012
FLECHNER BIOPSY KIDNEY TRANSPLANT REJECTED VS OK UP	85	3 (3.5)	3 (3.5)	0 (0)	<0.0001	0.0012
GRANDVAUX IFN RESPONSE NOT VIA IRF3	14	3 (21.4)	3 (21.4)	0 (0)	<0.0001	0.0012
ICHIBA GRAFT VERSUS HOST DISEASE 35D UP	126	3 (2.4)	3 (2.4)	0 (0)	<0.0001	0.0012
LIU VAV3 PROSTATE CARCINOGENESIS UP	85	3 (3.5)	3 (3.5)	0 (0)	<0.0001	0.0012
MOSERLE IFNA RESPONSE	31	3 (9.7)	3 (9.7)	0 (0)	<0.0001	0.0012
RADAEVA RESPONSE TO IFNA1 UP	52	3 (5.8)	3 (5.8)	0 (0)	<0.0001	0.0012
SEKI INFLAMMATORY RESPONSE LPS UP	77	3 (3.9)	3 (3.9)	0 (0)	<0.0001	0.0012
WATANABE ULCERATIVE COLITIS WITH CANCER DN	13	3 (23.1)	3 (23.1)	0 (0)	<0.0001	0.0012
IIZUKA LIVER CANCER PROGRESSION G1 G2 UP	12	2 (16.7)	2 (16.7)	0 (0)	<0.0001	0.0012
NUYTTEN NIPP1 TARGETS UP	748	5 (0.7)	5 (0.7)	0 (0)	<0.0001	0.002
FULCHER INFLAMMATORY RESPONSE LECTIN VS LPS DN	462	4 (0.9)	4 (0.9)	0 (0)	<0.0001	0.002
GRUETZMANN PANCREATIC CANCER UP	351	4 (1.1)	4 (1.1)	0 (0)	<0.0001	0.002
TAKEDA TARGETS OF NUP98 HOXA9 FUSION 8D UP	155	3 (1.9)	3 (1.9)	0 (0)	<0.0001	0.002
BOWIE RESPONSE TO EXTRACELLULAR MATRIX	17	2 (11.8)	2 (11.8)	0 (0)	<0.0001	0.002
YAN ESCAPE FROM ANOIKIS	24	2 (8.3)	2 (8.3)	0 (0)	<0.0001	0.002
COLINA TARGETS OF 4EBP1 AND 4EBP2	339	4 (1.2)	4 (1.2)	0 (0)	<0.0001	0.0028
RUAN RESPONSE TO TNF UP	12	2 (16.7)	2 (16.7)	0 (0)	<0.0001	0.0028
CHICAS RB1 TARGETS CONFLUENT	550	4 (0.7)	4 (0.7)	0 (0)	<0.0001	0.0033
KRIEG KDM3A TARGETS NOT HYPOXIA	189	3 (1.6)	3 (1.6)	0 (0)	<0.0001	0.0033

Category Name	Category Genes #	Any-reg. SDEG #(%)	Up-reg. SDEG #(%)	Down-reg. SDEG #(%)	P	FDR
BOWIE RESPONSE TO TAMOXIFEN	18	2 (11.1)	2 (11.1)	0 (0)	<0.0001	0.0033
EINAV INTERFERON SIGNATURE IN CANCER	27	2 (7.4)	2 (7.4)	0 (0)	<0.0001	0.0033
ZHANG INTERFERON RESPONSE	23	2 (8.7)	2 (8.7)	0 (0)	<0.0001	0.0033
HAN JNK SINGALING UP	34	2 (5.9)	2 (5.9)	0 (0)	<0.0001	0.004
RUAN RESPONSE TO TNF TROGLITAZONE UP	17	2 (11.8)	2 (11.8)	0 (0)	<0.0001	0.004
WAKABAYASHI ADIPOGENESIS PPARG BOUND 8D	658	4 (0.6)	4 (0.6)	0 (0)	<0.0001	0.0045
GEISS RESPONSE TO DSRNA UP	35	2 (5.7)	2 (5.7)	0 (0)	<0.0001	0.0045
PLASARI TGFB1 TARGETS 10HR DN	233	3 (1.3)	3 (1.3)	0 (0)	<0.0001	0.005
RIGGINS TAMOXIFEN RESISTANCE DN	215	3 (1.4)	3 (1.4)	0 (0)	<0.0001	0.005
ZHAN MULTIPLE MYELOMA LB DN	39	2 (5.1)	2 (5.1)	0 (0)	<0.0001	0.005
GRAESSMANN RESPONSE TO MC AND SERUM DEPRIVATION UP	203	3 (1.5)	3 (1.5)	0 (0)	<0.0001	0.0055
DEBIASI APOPTOSIS BY REOVIRUS INFECTION UP	317	3 (0.9)	3 (0.9)	0 (0)	0.0001	0.0091
DURAND STROMA MAX UP	270	3 (1.1)	3 (1.1)	0 (0)	0.0001	0.0091
MIYAGAWA TARGETS OF EWSR1 ETS FUSIONS UP	247	3 (1.2)	3 (1.2)	0 (0)	0.0001	0.0091

Table A75: Significantly enriched MSigDB Chemical/Genetic Perturbation Sets (RNA-Seq, B-cells, Day 1). Results sorted by FDR and #SDEG genes.

Category Name	Category Genes #	Any-reg. SDEG #(%)	Up-reg. SDEG #(%)	Down-reg. SDEG #(%)	P	FDR
Pertussis	70	6 (8.6)	6 (8.6)	0 (0)	<0.0001	0.0029
Staphylococcus aureus infection	47	5 (10.6)	5 (10.6)	0 (0)	<0.0001	0.0029
Complement and coagulation cascades	61	5 (8.2)	5 (8.2)	0 (0)	<0.0001	0.0029
Herpes simplex infection	154	7 (4.5)	7 (4.5)	0 (0)	<0.0001	0.0057
Transcriptional misregulation in cancer	163	7 (4.3)	5 (3.1)	2 (1.2)	0.0002	0.0082
Hepatitis C	117	6 (5.1)	6 (5.1)	0 (0)	0.0001	0.0082
Systemic lupus erythematosus	106	5 (4.7)	5 (4.7)	0 (0)	0.0002	0.0082

Table A76: Significantly enriched KEGG Pathways (RNA-Seq, Dendritic cells, Day 1). Results sorted by FDR and #SDEG genes.

Category Name	Category Genes #	Any-reg. SDEG #(%)	Up-reg. SDEG #(%)	Down-reg. SDEG #(%)	P	FDR
IMMUNE RESPONSE	231	11 (4.8)	9 (3.9)	2 (0.9)	<0.0001	0.0082

Table A77: Significantly enriched MSigDB GO Biological Processes (RNA-Seq, Dendritic cells, Day 1). Results sorted by FDR and #SDEG genes.

Category Name	Category Genes #	Any-reg. SDEG #(%)	Up-reg. SDEG #(%)	Down-reg. SDEG #(%)	P	FDR
MEMBRANE	1902	31 (1.6)	25 (1.3)	6 (0.3)	<0.0001	0.0012
PLASMA MEMBRANE	1358	25 (1.8)	22 (1.6)	3 (0.2)	<0.0001	0.0012
MEMBRANE PART	1597	25 (1.6)	20 (1.3)	5 (0.3)	<0.0001	0.0078

Table A78: Significantly enriched MSigDB GO Cellular Components (RNA-Seq, Dendritic cells, Day 1). Results sorted by FDR and #SDEG genes.

Category Name	Category Genes #	Any-reg. SDEG #(%)	Up-reg. SDEG #(%)	Down-reg. SDEG #(%)	P	FDR
BIOCARTA COMP PATHWAY	19	4 (21.1)	4 (21.1)	0 (0)	<0.0001	0.0022

Table A79: Significantly enriched MSigDB BioCarta Pathways (RNA-Seq, Dendritic cells, Day 1). Results sorted by FDR and #SDEG genes.

Category Name	Category Genes #	Any-reg. SDEG #(%)	Up-reg. SDEG #(%)	Down-reg. SDEG #(%)	P	FDR
NUYTTEN EZH2 TARGETS UP	1002	48 (4.8)	46 (4.6)	2 (0.2)	<0.0001	0.0003
FULCHER INFLAMMATORY RESPONSE LECTIN VS LPS DN	462	39 (8.4)	39 (8.4)	0 (0)	<0.0001	0.0003
MARKEY RB1 ACUTE LOF UP	214	34 (15.9)	34 (15.9)	0 (0)	<0.0001	0.0003
TAKEDA TARGETS OF NUP98 HOXA9 FUSION 3D UP	178	33 (18.5)	32 (18)	1 (0.6)	<0.0001	0.0003
NUYTTEN NIPP1 TARGETS UP	748	32 (4.3)	31 (4.1)	1 (0.1)	<0.0001	0.0003
BLALOCK ALZHEIMERS DISEASE UP	1628	31 (1.9)	24 (1.5)	7 (0.4)	<0.0001	0.0003
HECKER IFNB1 TARGETS	92	31 (33.7)	31 (33.7)	0 (0)	<0.0001	0.0003
WALLACE PROSTATE CANCER RACE UP	275	30 (10.9)	28 (10.2)	2 (0.7)	<0.0001	0.0003
SANA RESPONSE TO IFNG UP	74	29 (39.2)	29 (39.2)	0 (0)	<0.0001	0.0003
CHEN METABOLIC SYNDROM NETWORK	1172	28 (2.4)	27 (2.3)	1 (0.1)	<0.0001	0.0003
GRAESSMANN APOPTOSIS BY SERUM DEPRIVATION UP	541	28 (5.2)	25 (4.6)	3 (0.6)	<0.0001	0.0003
JISON SICKLE CELL DISEASE UP	174	28 (16.1)	27 (15.5)	1 (0.6)	<0.0001	0.0003
TAKEDA TARGETS OF NUP98 HOXA9 FUSION 10D UP	186	28 (15.1)	27 (14.5)	1 (0.5)	<0.0001	0.0003
DODD NASOPHARYNGEAL CARCINOMA DN	1330	27 (2)	25 (1.9)	2 (0.2)	<0.0001	0.0003
GRAESSMANN APOPTOSIS BY DOXORUBICIN UP	1132	25 (2.2)	23 (2)	2 (0.2)	<0.0001	0.0003
ONKEN UVEAL MELANOMA UP	763	25 (3.3)	22 (2.9)	3 (0.4)	<0.0001	0.0003
GOZGIT ESR1 TARGETS DN	741	24 (3.2)	23 (3.1)	1 (0.1)	<0.0001	0.0003
RODWELL AGING KIDNEY UP	465	24 (5.2)	24 (5.2)	0 (0)	<0.0001	0.0003
TAKEDA TARGETS OF NUP98 HOXA9 FUSION 8D UP	155	24 (15.5)	23 (14.8)	1 (0.6)	<0.0001	0.0003
BROWNE INTERFERON RESPONSIVE GENES	68	23 (33.8)	23 (33.8)	0 (0)	<0.0001	0.0003
FEVR CTNNB1 TARGETS UP	672	23 (3.4)	21 (3.1)	2 (0.3)	<0.0001	0.0003
YOSHIMURA MAPK8 TARGETS UP	1281	23 (1.8)	19 (1.5)	4 (0.3)	<0.0001	0.0003
BOSCO INTERFERON INDUCED ANTIVIRAL MODULE	76	22 (28.9)	22 (28.9)	0 (0)	<0.0001	0.0003

Category Name	Category Genes #	Any-reg. SDEG #(%)	Up-reg. SDEG #(%)	Down-reg. SDEG #(%)	P	FDR
DEBIASI APOPTOSIS BY REOVIRUS INFECTION UP	317	22 (6.9)	22 (6.9)	0 (0)	<0.0001	0.0003
GRAESSMANN RESPONSE TO MC AND SERUM DEPRIVATION UP	203	22 (10.8)	20 (9.9)	2 (1)	<0.0001	0.0003
HELLER SILENCED BY METHYLATION UP	273	22 (8.1)	21 (7.7)	1 (0.4)	<0.0001	0.0003
ICHIBA GRAFT VERSUS HOST DISEASE D7 UP	105	22 (21)	22 (21)	0 (0)	<0.0001	0.0003
ALTEMEIER RESPONSE TO LPS WITH MECHANICAL VENTILATION	126	21 (16.7)	20 (15.9)	1 (0.8)	<0.0001	0.0003
CHICAS RB1 TARGETS SENESCENT	552	21 (3.8)	21 (3.8)	0 (0)	<0.0001	0.0003
TAKEDA TARGETS OF NUP98 HOXA9 FUSION 16D UP	169	21 (12.4)	21 (12.4)	0 (0)	<0.0001	0.0003
BERTUCCI MEDULLARY VS DUCTAL BREAST CANCER UP	200	20 (10)	20 (10)	0 (0)	<0.0001	0.0003
CHICAS RB1 TARGETS CONFLUENT	550	20 (3.6)	16 (2.9)	4 (0.7)	<0.0001	0.0003
MARTENS TRETINOIN RESPONSE UP	816	18 (2.2)	15 (1.8)	3 (0.4)	<0.0001	0.0003
SMID BREAST CANCER BASAL UP	607	18 (3)	14 (2.3)	4 (0.7)	<0.0001	0.0003
SMID BREAST CANCER LUMINAL B DN	513	18 (3.5)	15 (2.9)	3 (0.6)	<0.0001	0.0003
YANG BCL3 TARGETS UP	351	18 (5.1)	17 (4.8)	1 (0.3)	<0.0001	0.0003
FOSTER TOLERANT MACROPHAGE DN	405	17 (4.2)	15 (3.7)	2 (0.5)	<0.0001	0.0003
MOSERLE IFNA RESPONSE	31	17 (54.8)	17 (54.8)	0 (0)	<0.0001	0.0003
RADAEVA RESPONSE TO IFNA1 UP	52	17 (32.7)	17 (32.7)	0 (0)	<0.0001	0.0003
RUTELLA RESPONSE TO HGF UP	398	17 (4.3)	15 (3.8)	2 (0.5)	<0.0001	0.0003
RUTELLA RESPONSE TO HGF VS CSF2RB AND IL4 UP	391	17 (4.3)	17 (4.3)	0 (0)	<0.0001	0.0003
SPIELMAN LYMPHOBLAST EUROPEAN VS ASIAN DN	570	17 (3)	17 (3)	0 (0)	<0.0001	0.0003
WIELAND UP BY HBV INFECTION	100	17 (17)	17 (17)	0 (0)	<0.0001	0.0003
ZHOU INFLAMMATORY RESPONSE LPS UP	401	17 (4.2)	15 (3.7)	2 (0.5)	<0.0001	0.0003
COLINA TARGETS OF 4EBP1 AND 4EBP2	339	16 (4.7)	16 (4.7)	0 (0)	<0.0001	0.0003
DELYS THYROID CANCER UP	435	16 (3.7)	13 (3)	3 (0.7)	<0.0001	0.0003
ICHIBA GRAFT VERSUS HOST DISEASE 35D UP	126	16 (12.7)	16 (12.7)	0 (0)	<0.0001	0.0003
HORIUCHI WTAP TARGETS UP	295	15 (5.1)	14 (4.7)	1 (0.3)	<0.0001	0.0003
SCHUETZ BREAST CANCER DUCTAL INVASIVE UP	338	15 (4.4)	14 (4.1)	1 (0.3)	<0.0001	0.0003
ZHANG RESPONSE TO IKK INHIBITOR AND TNF UP	221	15 (6.8)	14 (6.3)	1 (0.5)	<0.0001	0.0003
BENNETT SYSTEMIC LUPUS ERYTHEMATOSUS	32	14 (43.8)	14 (43.8)	0 (0)	<0.0001	0.0003
BOSCO TH1 CYTOTOXIC MODULE	114	14 (12.3)	14 (12.3)	0 (0)	<0.0001	0.0003
CHARAFE BREAST CANCER LUMINAL VS BASAL DN	440	14 (3.2)	12 (2.7)	2 (0.5)	<0.0001	0.0003
DAUER STAT3 TARGETS DN	51	14 (27.5)	14 (27.5)	0 (0)	<0.0001	0.0003
DER IFN BETA RESPONSE UP	101	14 (13.9)	14 (13.9)	0 (0)	<0.0001	0.0003
MCLACHLAN DENTAL CARIES UP	227	14 (6.2)	14 (6.2)	0 (0)	<0.0001	0.0003
RICKMAN METASTASIS DN	258	14 (5.4)	13 (5)	1 (0.4)	<0.0001	0.0003
RUTELLA RESPONSE TO CSF2RB AND IL4 UP	319	14 (4.4)	12 (3.8)	2 (0.6)	<0.0001	0.0003
SEITZ NEOPLASTIC TRANSFORMATION BY 8P DELETION UP	69	14 (20.3)	13 (18.8)	1 (1.4)	<0.0001	0.0003
SMID BREAST CANCER NORMAL LIKE UP	443	14 (3.2)	11 (2.5)	3 (0.7)	<0.0001	0.0003
SWEET LUNG CANCER KRAS DN	425	14 (3.3)	9 (2.1)	5 (1.2)	<0.0001	0.0003

Category Name	Category Genes #	Any-reg. SDEG #(%)	Up-reg. SDEG #(%)	Down-reg. SDEG #(%)	P	FDR
WANG SMARCE1 TARGETS UP	265	14 (5.3)	11 (4.2)	3 (1.1)	<0.0001	0.0003
DER IFN ALPHA RESPONSE UP	74	13 (17.6)	13 (17.6)	0 (0)	<0.0001	0.0003
HIRSCH CELLULAR TRANSFORMATION SIGNATURE UP	239	13 (5.4)	12 (5)	1 (0.4)	<0.0001	0.0003
MCLACHLAN DENTAL CARIES DN	219	13 (5.9)	13 (5.9)	0 (0)	<0.0001	0.0003
MIKKELSEN MCV6 LCP WITH H3K4ME3	153	13 (8.5)	12 (7.8)	1 (0.7)	<0.0001	0.0003
QI PLASMACYTOMA UP	251	13 (5.2)	11 (4.4)	2 (0.8)	<0.0001	0.0003
VERHAAK AML WITH NPM1 MUTATED DN	238	13 (5.5)	10 (4.2)	3 (1.3)	<0.0001	0.0003
BROWN MYELOID CELL DEVELOPMENT UP	159	12 (7.5)	10 (6.3)	2 (1.3)	<0.0001	0.0003
DURAND STROMA MAX UP	270	12 (4.4)	9 (3.3)	3 (1.1)	<0.0001	0.0003
PICCALUGA ANGIOIMMUNOBLASTIC LYMPHOMA UP	205	12 (5.9)	11 (5.4)	1 (0.5)	<0.0001	0.0003
POOLA INVASIVE BREAST CANCER UP	272	12 (4.4)	12 (4.4)	0 (0)	<0.0001	0.0003
RIGGINS TAMOXIFEN RESISTANCE DN	215	12 (5.6)	12 (5.6)	0 (0)	<0.0001	0.0003
ROZANOV MMP14 TARGETS UP	270	12 (4.4)	10 (3.7)	2 (0.7)	<0.0001	0.0003
SANA TNF SIGNALING UP	81	12 (14.8)	12 (14.8)	0 (0)	<0.0001	0.0003
TORCHIA TARGETS OF EWSR1 FLI1 FUSION DN	306	12 (3.9)	9 (2.9)	3 (1)	<0.0001	0.0003
VANTVEER BREAST CANCER ESR1 DN	232	12 (5.2)	10 (4.3)	2 (0.9)	<0.0001	0.0003
VERHAAK AML WITH NPM1 MUTATED UP	190	12 (6.3)	12 (6.3)	0 (0)	<0.0001	0.0003
WANG MLL TARGETS	280	12 (4.3)	10 (3.6)	2 (0.7)	<0.0001	0.0003
ZHU CMV ALL UP	121	12 (9.9)	12 (9.9)	0 (0)	<0.0001	0.0003
BOWIE RESPONSE TO EXTRACELLULAR MATRIX	17	11 (64.7)	11 (64.7)	0 (0)	<0.0001	0.0003
BROCKE APOPTOSIS REVERSED BY IL6	144	11 (7.6)	11 (7.6)	0 (0)	<0.0001	0.0003
DER IFN GAMMA RESPONSE UP	72	11 (15.3)	11 (15.3)	0 (0)	<0.0001	0.0003
FLECHNER BIOPSY KIDNEY TRANSPLANT REJECTED VS OK UP	85	11 (12.9)	11 (12.9)	0 (0)	<0.0001	0.0003
KRASNOSELSKAYA ILF3 TARGETS UP	37	11 (29.7)	11 (29.7)	0 (0)	<0.0001	0.0003
STAMBOLSKY TARGETS OF MUTATED TP53 DN	51	11 (21.6)	11 (21.6)	0 (0)	<0.0001	0.0003
BOWIE RESPONSE TO TAMOXIFEN	18	10 (55.6)	10 (55.6)	0 (0)	<0.0001	0.0003
FARMER BREAST CANCER CLUSTER 1	39	10 (25.6)	10 (25.6)	0 (0)	<0.0001	0.0003
FOSTER TOLERANT MACROPHAGE UP	155	10 (6.5)	9 (5.8)	1 (0.6)	<0.0001	0.0003
LIANG SILENCED BY METHYLATION 2	55	10 (18.2)	10 (18.2)	0 (0)	<0.0001	0.0003
TONKS TARGETS OF RUNX1 RUNX1T1 FUSION ERYTHROCYTE UP	151	10 (6.6)	9 (6)	1 (0.7)	<0.0001	0.0003
TONKS TARGETS OF RUNX1 RUNX1T1 FUSION MONOCYTE UP	201	10 (5)	10 (5)	0 (0)	<0.0001	0.0003
CHIARADONNA NEOPLASTIC TRANSFORMATION KRAS DN	143	9 (6.3)	8 (5.6)	1 (0.7)	<0.0001	0.0003
EINAV INTERFERON SIGNATURE IN CANCER	27	9 (33.3)	9 (33.3)	0 (0)	<0.0001	0.0003
JOHNSTONE PARVB TARGETS 2 UP	136	9 (6.6)	8 (5.9)	1 (0.7)	<0.0001	0.0003
KATSANOU ELAVL1 TARGETS UP	163	9 (5.5)	8 (4.9)	1 (0.6)	<0.0001	0.0003
KRIEG KDM3A TARGETS NOT HYPOXIA	189	9 (4.8)	9 (4.8)	0 (0)	<0.0001	0.0003
MIKKELSEN IPS LCP WITH H3K4ME3	170	9 (5.3)	9 (5.3)	0 (0)	<0.0001	0.0003
UROSEVIC RESPONSE TO IMIQUIMOD	23	9 (39.1)	9 (39.1)	0 (0)	<0.0001	0.0003

Category Name	Category Genes #	Any-reg. SDEG #(%)	Up-reg. SDEG #(%)	Down-reg. SDEG #(%)	P	FDR
ZHU CMV 24 HR UP	93	9 (9.7)	9 (9.7)	0 (0)	<0.0001	0.0003
CHANG IMMORTALIZED BY HPV31 DN	63	8 (12.7)	7 (11.1)	1 (1.6)	<0.0001	0.0003
ELVIDGE HYPOXIA BY DMOG UP	126	8 (6.3)	7 (5.6)	1 (0.8)	<0.0001	0.0003
GRANDVAUX IFN RESPONSE NOT VIA IRF3	14	8 (57.1)	8 (57.1)	0 (0)	<0.0001	0.0003
LENAOUR DENDRITIC CELL MATURATION UP	112	8 (7.1)	7 (6.2)	1 (0.9)	<0.0001	0.0003
LIU VAV3 PROSTATE CARCINOGENESIS UP	85	8 (9.4)	8 (9.4)	0 (0)	<0.0001	0.0003
MISSIAGLIA REGULATED BY METHYLATION UP	120	8 (6.7)	8 (6.7)	0 (0)	<0.0001	0.0003
SEKI INFLAMMATORY RESPONSE LPS UP	77	8 (10.4)	8 (10.4)	0 (0)	<0.0001	0.0003
WUNDER INFLAMMATORY RESPONSE AND CHOLESTEROL UP	61	8 (13.1)	8 (13.1)	0 (0)	<0.0001	0.0003
XU AKT1 TARGETS 6HR	27	8 (29.6)	8 (29.6)	0 (0)	<0.0001	0.0003
ZHANG INTERFERON RESPONSE	23	8 (34.8)	8 (34.8)	0 (0)	<0.0001	0.0003
ZHU CMV 8 HR UP	48	8 (16.7)	8 (16.7)	0 (0)	<0.0001	0.0003
CASTELLANO NRAS TARGETS UP	68	7 (10.3)	7 (10.3)	0 (0)	<0.0001	0.0003
HAN JNK SINGALING UP	34	7 (20.6)	7 (20.6)	0 (0)	<0.0001	0.0003
JACKSON DNMT1 TARGETS UP	76	7 (9.2)	6 (7.9)	1 (1.3)	<0.0001	0.0003
JECHLINGER EPITHELIAL TO MESENCHYMAL TRANSITION UP	71	7 (9.9)	7 (9.9)	0 (0)	<0.0001	0.0003
KIM RESPONSE TO TSA AND DECITABINE UP	120	7 (5.8)	6 (5)	1 (0.8)	<0.0001	0.0003
LEE AGING CEREBELLUM UP	81	7 (8.6)	7 (8.6)	0 (0)	<0.0001	0.0003
LINDSTEDT DENDRITIC CELL MATURATION A	67	7 (10.4)	7 (10.4)	0 (0)	<0.0001	0.0003
NAKAYAMA SOFT TISSUE TUMORS PCA1 UP	69	7 (10.1)	7 (10.1)	0 (0)	<0.0001	0.0003
TSAI RESPONSE TO RADIATION THERAPY	32	7 (21.9)	7 (21.9)	0 (0)	<0.0001	0.0003
ZHAN MULTIPLE MYELOMA LB DN	39	7 (17.9)	7 (17.9)	0 (0)	<0.0001	0.0003
BECKER TAMOXIFEN RESISTANCE UP	49	6 (12.2)	6 (12.2)	0 (0)	<0.0001	0.0003
BROWNE HCMV INFECTION 4HR UP	53	6 (11.3)	5 (9.4)	1 (1.9)	<0.0001	0.0003
GEISS RESPONSE TO DSRNA UP	35	6 (17.1)	5 (14.3)	1 (2.9)	<0.0001	0.0003
GRANDVAUX IRF3 TARGETS UP	15	6 (40)	6 (40)	0 (0)	<0.0001	0.0003
ROETH TERT TARGETS UP	14	6 (42.9)	6 (42.9)	0 (0)	<0.0001	0.0003
TSAI DNAJB4 TARGETS UP	13	6 (46.2)	6 (46.2)	0 (0)	<0.0001	0.0003
XU HGF TARGETS INDUCED BY AKT1 6HR	18	6 (33.3)	6 (33.3)	0 (0)	<0.0001	0.0003
ZHANG ANTIVIRAL RESPONSE TO RIBAVIRIN UP	29	6 (20.7)	6 (20.7)	0 (0)	<0.0001	0.0003
KAAB FAILED HEART VENTRICLE DN	41	5 (12.2)	5 (12.2)	0 (0)	<0.0001	0.0003
KIM LRRC3B TARGETS	30	5 (16.7)	5 (16.7)	0 (0)	<0.0001	0.0003
PARK TRETINOIN RESPONSE AND PML RARA FUSION	29	5 (17.2)	3 (10.3)	2 (6.9)	<0.0001	0.0003
YAN ESCAPE FROM ANOIKIS	24	5 (20.8)	5 (20.8)	0 (0)	<0.0001	0.0003
ZHENG IL22 SIGNALING UP	56	5 (8.9)	5 (8.9)	0 (0)	<0.0001	0.0003
WATANABE ULCERATIVE COLITIS WITH CANCER DN	13	4 (30.8)	4 (30.8)	0 (0)	<0.0001	0.0003
BAELDE DIABETIC NEPHROPATHY DN	425	13 (3.1)	13 (3.1)	0 (0)	<0.0001	0.0005
KUMAR TARGETS OF MLL AF9 FUSION	389	13 (3.3)	12 (3.1)	1 (0.3)	<0.0001	0.0005

Category Name	Category Genes #	Any-reg. SDEG #(%)	Up-reg. SDEG #(%)	Down-reg. SDEG #(%)	P	FDR
SENGUPTA NASOPHARYNGEAL CARCINOMA WITH LMP1 UP	387	13 (3.4)	13 (3.4)	0 (0)	<0.0001	0.0005
THUM SYSTOLIC HEART FAILURE UP	404	13 (3.2)	12 (3)	1 (0.2)	<0.0001	0.0005
BOQUEST STEM CELL CULTURED VS FRESH UP	409	12 (2.9)	11 (2.7)	1 (0.2)	<0.0001	0.0005
DANG REGULATED BY MYC DN	247	10 (4)	9 (3.6)	1 (0.4)	<0.0001	0.0005
TAKEDA TARGETS OF NUP98 HOXA9 FUSION 10D DN	135	8 (5.9)	5 (3.7)	3 (2.2)	<0.0001	0.0005
LENAOUR DENDRITIC CELL MATURATION DN	139	7 (5)	6 (4.3)	1 (0.7)	<0.0001	0.0005
FUJII YBX1 TARGETS UP	40	5 (12.5)	5 (12.5)	0 (0)	<0.0001	0.0005
LEE EARLY T LYMPHOCYTE DN	55	5 (9.1)	5 (9.1)	0 (0)	<0.0001	0.0005
STEGER ADIPOGENESIS UP	21	4 (19)	3 (14.3)	1 (4.8)	<0.0001	0.0005
LEE BMP2 TARGETS UP	723	18 (2.5)	16 (2.2)	2 (0.3)	<0.0001	0.0007
KRIEG HYPOXIA NOT VIA KDM3A	718	17 (2.4)	14 (1.9)	3 (0.4)	<0.0001	0.0007
WIERENGA STAT5A TARGETS DN	219	9 (4.1)	7 (3.2)	2 (0.9)	<0.0001	0.0007
SERVITJA ISLET HNF1A TARGETS UP	160	8 (5)	8 (5)	0 (0)	<0.0001	0.0007
WANG ESOPHAGUS CANCER VS NORMAL UP	118	7 (5.9)	6 (5.1)	1 (0.8)	<0.0001	0.0007
GARGALOVIC RESPONSE TO OXIDIZED PHOSPHOLIPIDS GREY DN	73	6 (8.2)	6 (8.2)	0 (0)	<0.0001	0.0007
DAUER STAT3 TARGETS UP	50	5 (10)	5 (10)	0 (0)	<0.0001	0.0007
LIAN LIPA TARGETS 3M	56	5 (8.9)	4 (7.1)	1 (1.8)	<0.0001	0.0007
NOJIMA SFRP2 TARGETS DN	25	4 (16)	4 (16)	0 (0)	<0.0001	0.0007
RUTELLA RESPONSE TO CSF2RB AND IL4 DN	303	11 (3.6)	11 (3.6)	0 (0)	<0.0001	0.0008
LU EZH2 TARGETS UP	289	9 (3.1)	9 (3.1)	0 (0)	<0.0001	0.0008
MIYAGAWA TARGETS OF EWSR1 ETS FUSIONS DN	219	9 (4.1)	5 (2.3)	4 (1.8)	<0.0001	0.0008
VERHAAK GLIOBLASTOMA NEURAL	205	9 (4.4)	6 (2.9)	3 (1.5)	<0.0001	0.0008
WIERENGA STAT5A TARGETS UP	210	9 (4.3)	8 (3.8)	1 (0.5)	<0.0001	0.0008
SMIRNOV RESPONSE TO IR 6HR DN	113	7 (6.2)	6 (5.3)	1 (0.9)	<0.0001	0.0008
HESS TARGETS OF HOXA9 AND MEIS1 DN	76	6 (7.9)	5 (6.6)	1 (1.3)	<0.0001	0.0008
LEE LIVER CANCER MYC TGFA UP	62	5 (8.1)	4 (6.5)	1 (1.6)	<0.0001	0.0008
BAUS TFF2 TARGETS UP	32	4 (12.5)	4 (12.5)	0 (0)	<0.0001	0.0008
HASEGAWA TUMORIGENESIS BY RET C634R	11	3 (27.3)	3 (27.3)	0 (0)	<0.0001	0.0008
LEI MYB TARGETS	307	10 (3.3)	10 (3.3)	0 (0)	<0.0001	0.001
SENGUPTA NASOPHARYNGEAL CARCINOMA UP	287	10 (3.5)	10 (3.5)	0 (0)	<0.0001	0.001
ALCALAY AML BY NPM1 LOCALIZATION DN	178	8 (4.5)	5 (2.8)	3 (1.7)	<0.0001	0.001
TAKEDA TARGETS OF NUP98 HOXA9 FUSION 8D DN	193	8 (4.1)	5 (2.6)	3 (1.6)	<0.0001	0.001
HUANG DASATINIB RESISTANCE UP	79	6 (7.6)	4 (5.1)	2 (2.5)	<0.0001	0.001
LEE TARGETS OF PTCH1 AND SUFU UP	55	5 (9.1)	3 (5.5)	2 (3.6)	<0.0001	0.001
WONG ADULT TISSUE STEM MODULE	697	16 (2.3)	12 (1.7)	4 (0.6)	<0.0001	0.0012
RODWELL AGING KIDNEY NO BLOOD UP	213	9 (4.2)	8 (3.8)	1 (0.5)	<0.0001	0.0012
MIKKELSEN MEF LCP WITH H3K4ME3	121	7 (5.8)	7 (5.8)	0 (0)	<0.0001	0.0012
BOYLAN MULTIPLE MYELOMA PCA1 UP	96	6 (6.2)	6 (6.2)	0 (0)	<0.0001	0.0012

Category Name	Category Genes #	Any-reg. SDEG #(%)	Up-reg. SDEG #(%)	Down-reg. SDEG #(%)	P	FDR
NEMETH INFLAMMATORY RESPONSE LPS UP	85	6 (7.1)	5 (5.9)	1 (1.2)	<0.0001	0.0012
DEMAGALHAES AGING UP	56	5 (8.9)	5 (8.9)	0 (0)	<0.0001	0.0012
ONDER CDH1 TARGETS 2 DN	454	12 (2.6)	10 (2.2)	2 (0.4)	<0.0001	0.0013
HELLER HDAC TARGETS DN	281	10 (3.6)	9 (3.2)	1 (0.4)	<0.0001	0.0013
ELVIDGE HYPOXIA UP	166	8 (4.8)	7 (4.2)	1 (0.6)	<0.0001	0.0013
GRAHAM NORMAL QUIESCENT VS NORMAL DIVIDING UP	66	5 (7.6)	5 (7.6)	0 (0)	<0.0001	0.0013
KANG GIST WITH PDGFRA UP	50	5 (10)	5 (10)	0 (0)	<0.0001	0.0013
LEE LIVER CANCER E2F1 UP	62	5 (8.1)	5 (8.1)	0 (0)	<0.0001	0.0013
PEREZ TP53 TARGETS	1124	21 (1.9)	13 (1.2)	8 (0.7)	<0.0001	0.0014
HAN SATB1 TARGETS DN	421	12 (2.9)	12 (2.9)	0 (0)	<0.0001	0.0014
ODONNELL TFRC TARGETS UP	409	12 (2.9)	12 (2.9)	0 (0)	<0.0001	0.0014
CHANG CORE SERUM RESPONSE DN	205	8 (3.9)	7 (3.4)	1 (0.5)	<0.0001	0.0014
SARRIO EPITHELIAL MESENCHYMAL TRANSITION DN	149	7 (4.7)	6 (4)	1 (0.7)	<0.0001	0.0014
KRIGE RESPONSE TO TOSEDOSTAT 6HR UP	915	18 (2)	18 (2)	0 (0)	<0.0001	0.0018
BEIER GLIOMA STEM CELL DN	63	5 (7.9)	4 (6.3)	1 (1.6)	<0.0001	0.0018
FURUKAWA DUSP6 TARGETS PCI35 UP	73	5 (6.8)	5 (6.8)	0 (0)	<0.0001	0.0018
LIAN LIPA TARGETS 6M	70	5 (7.1)	4 (5.7)	1 (1.4)	<0.0001	0.0018
RUAN RESPONSE TO TNF UP	12	3 (25)	3 (25)	0 (0)	<0.0001	0.0018
DIAZ CHRONIC MEYLOGENOUS LEUKEMIA DN	112	6 (5.4)	5 (4.5)	1 (0.9)	0.0001	0.0019
LABBE TARGETS OF TGFB1 AND WNT3A DN	106	6 (5.7)	6 (5.7)	0 (0)	0.0001	0.0019
GRUETZMANN PANCREATIC CANCER UP	351	10 (2.8)	10 (2.8)	0 (0)	0.0001	0.0021
FRIDMAN SENESCENCE UP	78	5 (6.4)	4 (5.1)	1 (1.3)	0.0001	0.0021
WANG SMARCE1 TARGETS DN	340	10 (2.9)	10 (2.9)	0 (0)	0.0001	0.0024
WORSCHECH TUMOR EVASION AND TOLEROGENICITY DN	15	3 (20)	2 (13.3)	1 (6.7)	0.0001	0.0024
SHEDDEN LUNG CANCER GOOD SURVIVAL A12	280	9 (3.2)	8 (2.9)	1 (0.4)	0.0001	0.0025
BROWNE HCMV INFECTION 12HR UP	105	6 (5.7)	6 (5.7)	0 (0)	0.0001	0.0025
CHEBOTAEV GR TARGETS DN	114	6 (5.3)	5 (4.4)	1 (0.9)	0.0001	0.0025
GENTILE UV LOW DOSE DN	64	5 (7.8)	2 (3.1)	3 (4.7)	0.0001	0.0025
ZWANG TRANSIENTLY UP BY 1ST EGF PULSE ONLY	1723	26 (1.5)	20 (1.2)	6 (0.3)	0.0002	0.0026
FOSTER KDM1A TARGETS UP	250	8 (3.2)	8 (3.2)	0 (0)	0.0002	0.0026
RUIZ TNC TARGETS UP	149	7 (4.7)	5 (3.4)	2 (1.3)	0.0002	0.0026
AMIT EGF RESPONSE 40 HELA	42	4 (9.5)	4 (9.5)	0 (0)	0.0002	0.0026
SATO SILENCED BY DEACETYLATION IN PANCREATIC CANCER	43	4 (9.3)	2 (4.7)	2 (4.7)	0.0002	0.0026
MONNIER POSTRADIATION TUMOR ESCAPE DN	357	10 (2.8)	10 (2.8)	0 (0)	0.0002	0.0027
JI RESPONSE TO FSH UP	85	5 (5.9)	5 (5.9)	0 (0)	0.0002	0.0027
XU RESPONSE TO TRETINOIN AND NSC682994 UP	16	3 (18.8)	3 (18.8)	0 (0)	0.0002	0.0027
RASHI RESPONSE TO IONIZING RADIATION 6	79	5 (6.3)	4 (5.1)	1 (1.3)	0.0002	0.0029
SANSOM APC TARGETS DN	359	10 (2.8)	9 (2.5)	1 (0.3)	0.0002	0.0031

Category Name	Category Genes #	Any-reg. SDEG #(%)	Up-reg. SDEG #(%)	Down-reg. SDEG #(%)	P	FDR
LI INDUCED T TO NATURAL KILLER UP	302	9 (3)	9 (3)	0 (0)	0.0002	0.0031
GAL LEUKEMIC STEM CELL DN	244	8 (3.3)	8 (3.3)	0 (0)	0.0002	0.0031
KHETCHOUMIAN TRIM24 TARGETS UP	45	4 (8.9)	4 (8.9)	0 (0)	0.0002	0.0031
MAHADEVAN IMATINIB RESISTANCE UP	17	3 (17.6)	1 (5.9)	2 (11.8)	0.0002	0.0031
MEISSNER BRAIN HCP WITH H3K4ME3 AND H3K27ME3	1060	19 (1.8)	15 (1.4)	4 (0.4)	0.0002	0.0033
RUTELLA RESPONSE TO HGF DN	224	8 (3.6)	8 (3.6)	0 (0)	0.0002	0.0033
LEE RECENT THYMIC EMIGRANT	217	8 (3.7)	8 (3.7)	0 (0)	0.0002	0.0034
SASAKI ADULT T CELL LEUKEMIA	186	7 (3.8)	6 (3.2)	1 (0.5)	0.0002	0.0034
BASSO HAIRY CELL LEUKEMIA DN	79	5 (6.3)	2 (2.5)	3 (3.8)	0.0002	0.0034
OSWALD HEMATOPOIETIC STEM CELL IN COLLAGEN GEL UP	227	8 (3.5)	6 (2.6)	2 (0.9)	0.0002	0.0035
LEONARD HYPOXIA	46	4 (8.7)	4 (8.7)	0 (0)	0.0002	0.0035
ZHAN MULTIPLE MYELOMA SPIKED	22	3 (13.6)	3 (13.6)	0 (0)	0.0002	0.0035
KIM GLIS2 TARGETS UP	84	5 (6)	5 (6)	0 (0)	0.0002	0.0036
MASSARWEH TAMOXIFEN RESISTANCE UP	561	13 (2.3)	9 (1.6)	4 (0.7)	0.0002	0.0037
BOYLAN MULTIPLE MYELOMA C D DN	244	8 (3.3)	8 (3.3)	0 (0)	0.0002	0.0037
RUTELLA RESPONSE TO HGF VS CSF2RB AND IL4 DN	231	8 (3.5)	6 (2.6)	2 (0.9)	0.0002	0.0037
MAHADEVAN RESPONSE TO MP470 UP	19	3 (15.8)	3 (15.8)	0 (0)	0.0002	0.0037
RUAN RESPONSE TO TNF TROGLITAZONE UP	17	3 (17.6)	3 (17.6)	0 (0)	0.0003	0.0038
PHONG TNF RESPONSE NOT VIA P38	333	10 (3)	9 (2.7)	1 (0.3)	0.0003	0.004
JIANG HYPOXIA NORMAL	300	9 (3)	7 (2.3)	2 (0.7)	0.0003	0.0041
NIELSEN SYNOVIAL SARCOMA DN	19	3 (15.8)	3 (15.8)	0 (0)	0.0003	0.0041
AMIT EGF RESPONSE 60 HELA	45	4 (8.9)	4 (8.9)	0 (0)	0.0003	0.0043
OSADA ASCL1 TARGETS UP	45	4 (8.9)	4 (8.9)	0 (0)	0.0003	0.0043
JOHNSTONE PARVB TARGETS 3 UP	425	11 (2.6)	11 (2.6)	0 (0)	0.0003	0.0044
HINATA NFKB TARGETS KERATINOCYTE UP	91	5 (5.5)	4 (4.4)	1 (1.1)	0.0003	0.0044
YAMASHITA METHYLATED IN PROSTATE CANCER	53	4 (7.5)	4 (7.5)	0 (0)	0.0003	0.0044
CROONQUIST IL6 DEPRIVATION UP	20	3 (15)	3 (15)	0 (0)	0.0003	0.0045
SARTIPY BLUNTED BY INSULIN RESISTANCE UP	19	3 (15.8)	3 (15.8)	0 (0)	0.0003	0.0047
PLASARI TGFB1 TARGETS 10HR DN	233	8 (3.4)	8 (3.4)	0 (0)	0.0003	0.0049
KRIGE RESPONSE TO TOSEDOSTAT 24HR UP	740	15 (2)	15 (2)	0 (0)	0.0004	0.005
PROVENZANI METASTASIS DN	133	6 (4.5)	5 (3.8)	1 (0.8)	0.0004	0.0051
PELLICCIOTTA HDAC IN ANTIGEN PRESENTATION DN	51	4 (7.8)	4 (7.8)	0 (0)	0.0004	0.0051
ZHENG GLIOBLASTOMA PLASTICITY UP	252	8 (3.2)	6 (2.4)	2 (0.8)	0.0004	0.0054
CHIANG LIVER CANCER SUBCLASS INTERFERON UP	24	3 (12.5)	3 (12.5)	0 (0)	0.0004	0.0056
WIERENGA STAT5A TARGETS GROUP1	130	6 (4.6)	5 (3.8)	1 (0.8)	0.0004	0.0058
KOYAMA SEMA3B TARGETS DN	394	10 (2.5)	7 (1.8)	3 (0.8)	0.0004	0.0059
BROWNE HCMV INFECTION 48HR DN	477	11 (2.3)	8 (1.7)	3 (0.6)	0.0004	0.006
HUANG GATA2 TARGETS UP	146	6 (4.1)	5 (3.4)	1 (0.7)	0.0005	0.0062

Category Name	Category Genes #	Any-reg. SDEG #(%)	Up-reg. SDEG #(%)	Down-reg. SDEG #(%)	P	FDR
ZHANG TARGETS OF EWSR1 FLI1 FUSION	84	5 (6)	4 (4.8)	1 (1.2)	0.0005	0.0062
BROWNE HCMV INFECTION 30MIN UP	51	4 (7.8)	4 (7.8)	0 (0)	0.0005	0.0066
MCBRYAN PUBERTAL BREAST 4 5WK UP	264	8 (3)	6 (2.3)	2 (0.8)	0.0005	0.0067
VART KSHV INFECTION ANGIOGENIC MARKERS DN	137	6 (4.4)	3 (2.2)	3 (2.2)	0.0005	0.0067
TSAI RESPONSE TO IONIZING RADIATION	146	6 (4.1)	6 (4.1)	0 (0)	0.0005	0.0069
JAATINEN HEMATOPOIETIC STEM CELL DN	221	7 (3.2)	5 (2.3)	2 (0.9)	0.0005	0.007
FULCHER INFLAMMATORY RESPONSE LECTIN VS LPS UP	564	12 (2.1)	9 (1.6)	3 (0.5)	0.0005	0.0071
KEEN RESPONSE TO ROSIGLITAZONE DN	106	5 (4.7)	4 (3.8)	1 (0.9)	0.0005	0.0072
MURATA VIRULENCE OF H PILORI	23	3 (13)	3 (13)	0 (0)	0.0005	0.0072
GYORFFY DOXORUBICIN RESISTANCE	53	4 (7.5)	3 (5.7)	1 (1.9)	0.0006	0.0073
LEE LIVER CANCER MYC E2F1 UP	56	4 (7.1)	4 (7.1)	0 (0)	0.0006	0.0075
ZWANG TRANSIENTLY UP BY 2ND EGF PULSE ONLY	1568	22 (1.4)	17 (1.1)	5 (0.3)	0.0006	0.0076
BASSO CD40 SIGNALING UP	99	5 (5.1)	5 (5.1)	0 (0)	0.0006	0.0076
ACEVEDO FGFR1 TARGETS IN PROSTATE CANCER MODEL UP	278	8 (2.9)	8 (2.9)	0 (0)	0.0006	0.0077
DACOSTA UV RESPONSE VIA ERCC3 UP	301	8 (2.7)	5 (1.7)	3 (1)	0.0006	0.008
DIRMEIER LMP1 RESPONSE LATE DN	30	3 (10)	3 (10)	0 (0)	0.0006	0.0083
GAURNIER PSMD4 TARGETS	68	4 (5.9)	3 (4.4)	1 (1.5)	0.0007	0.0083
WEST ADRENOCORTICAL CARCINOMA VS ADENOMA DN	24	3 (12.5)	3 (12.5)	0 (0)	0.0007	0.0083
BOYAULT LIVER CANCER SUBCLASS G5 DN	27	3 (11.1)	3 (11.1)	0 (0)	0.0007	0.0084
LIM MAMMARY STEM CELL DN	422	10 (2.4)	9 (2.1)	1 (0.2)	0.0007	0.0087
GAVIN FOXP3 TARGETS CLUSTER P4	99	5 (5.1)	2 (2)	3 (3)	0.0007	0.0091
SHAFFER IRF4 TARGETS IN MYELOMA VS MATURE B LYMPHOCYTE	97	5 (5.2)	5 (5.2)	0 (0)	0.0007	0.0091
MARTINEZ RB1 AND TP53 TARGETS UP	582	12 (2.1)	10 (1.7)	2 (0.3)	0.0007	0.0093
OLSSON E2F3 TARGETS UP	28	3 (10.7)	3 (10.7)	0 (0)	0.0008	0.0095
YAMASHITA SILENCED BY METHYLATION	7	2 (28.6)	2 (28.6)	0 (0)	0.0008	0.0095
SHARMA PILOCYTIC ASTROCYTOMA LOCATION UP	25	3 (12)	3 (12)	0 (0)	0.0008	0.0099

Table A80: Significantly enriched MSigDB Chemical/Genetic Perturbation Sets (RNA-Seq, Dendritic cells, Day 1). Results sorted by FDR and #SDEG genes.

Category Name	Category Genes #	Any-reg. SDEG #(%)	Up-reg. SDEG #(%)	Down-reg. SDEG #(%)	P	FDR
FULCHER INFLAMMATORY RESPONSE LECTIN VS LPS DN	462	7 (1.5)	7 (1.5)	0 (0)	<0.0001	0.0068
MARKEY RB1 ACUTE LOF UP	214	4 (1.9)	4 (1.9)	0 (0)	<0.0001	0.0068
VERHAAK AML WITH NPM1 MUTATED UP	190	4 (2.1)	4 (2.1)	0 (0)	<0.0001	0.0068
IWANAGA CARCINOGENESIS BY KRAS PTEN UP	167	3 (1.8)	3 (1.8)	0 (0)	<0.0001	0.0068
KATSANOU ELAVL1 TARGETS UP	163	3 (1.8)	3 (1.8)	0 (0)	<0.0001	0.0068

Table A81: Significantly enriched MSigDB Chemical/Genetic Perturbation Sets (RNA-Seq, Dendritic cells, Day 3). Results sorted by FDR and #SDEG genes.

Category Name	Category Genes #	Any-reg. SDEG #(%)	Up-reg. SDEG #(%)	Down-reg. SDEG #(%)	P	FDR
Influenza A	149	14 (9.4)	14 (9.4)	0 (0)	<0.0001	0.001
Herpes simplex infection	154	12 (7.8)	12 (7.8)	0 (0)	<0.0001	0.001
Measles	115	10 (8.7)	10 (8.7)	0 (0)	<0.0001	0.001
Staphylococcus aureus infection	47	6 (12.8)	6 (12.8)	0 (0)	<0.0001	0.0021
Hepatitis C	117	8 (6.8)	8 (6.8)	0 (0)	<0.0001	0.0048
Antigen processing and presentation	64	6 (9.4)	6 (9.4)	0 (0)	<0.0001	0.0048
Osteoclast differentiation	113	8 (7.1)	7 (6.2)	1 (0.9)	0.0001	0.005
Phagosome	131	8 (6.1)	7 (5.3)	1 (0.8)	0.0001	0.005
Hematopoietic cell lineage	74	6 (8.1)	2 (2.7)	4 (5.4)	0.0002	0.006

Table A82: Significantly enriched KEGG Pathways (RNA-Seq, Monocytes, Day 1). Results sorted by FDR and #SDEG genes.

Category Name	Category Genes #	Any-reg. SDEG #(%)	Up-reg. SDEG #(%)	Down-reg. SDEG #(%)	P	FDR
SIGNAL TRANSDUCTION	1580	44 (2.8)	32 (2)	12 (0.8)	<0.0001	0.0007
SYSTEM PROCESS	527	23 (4.4)	12 (2.3)	11 (2.1)	<0.0001	0.0007
IMMUNE SYSTEM PROCESS	324	22 (6.8)	18 (5.6)	4 (1.2)	<0.0001	0.0007
CELL PROLIFERATION GO 0008283	493	21 (4.3)	15 (3)	6 (1.2)	<0.0001	0.0007
DEFENSE RESPONSE	262	18 (6.9)	15 (5.7)	3 (1.1)	<0.0001	0.0007
REGULATION OF DEVELOPMENTAL PROCESS	434	18 (4.1)	12 (2.8)	6 (1.4)	<0.0001	0.0007
IMMUNE RESPONSE	231	17 (7.4)	16 (6.9)	1 (0.4)	<0.0001	0.0007
MULTI ORGANISM PROCESS	157	11 (7)	10 (6.4)	1 (0.6)	<0.0001	0.0007
MEMBRANE ORGANIZATION AND BIOGENESIS	135	10 (7.4)	5 (3.7)	5 (3.7)	<0.0001	0.0007
RESPONSE TO BIOTIC STIMULUS	117	9 (7.7)	8 (6.8)	1 (0.9)	<0.0001	0.0007
RESPONSE TO OTHER ORGANISM	79	8 (10.1)	7 (8.9)	1 (1.3)	<0.0001	0.0007
RESPONSE TO VIRUS	47	7 (14.9)	7 (14.9)	0 (0)	<0.0001	0.0007
JAK STAT CASCADE	31	6 (19.4)	6 (19.4)	0 (0)	<0.0001	0.0013
REGULATION OF PROGRAMMED CELL DEATH	339	14 (4.1)	11 (3.2)	3 (0.9)	<0.0001	0.0024
PHAGOCYTOSIS	18	4 (22.2)	2 (11.1)	2 (11.1)	<0.0001	0.0033
RESPONSE TO WOUNDING	185	10 (5.4)	9 (4.9)	1 (0.5)	<0.0001	0.0034
INFLAMMATORY RESPONSE	129	8 (6.2)	7 (5.4)	1 (0.8)	<0.0001	0.0034
POSITIVE REGULATION OF BIOLOGICAL PROCESS	690	22 (3.2)	16 (2.3)	6 (0.9)	<0.0001	0.0046
POSITIVE REGULATION OF CELLULAR PROCESS	652	21 (3.2)	15 (2.3)	6 (0.9)	0.0001	0.0052
PROTEIN METABOLIC PROCESS	1185	31 (2.6)	22 (1.9)	9 (0.8)	0.0001	0.0054
PROGRAMMED CELL DEATH	429	15 (3.5)	12 (2.8)	3 (0.7)	0.0002	0.0071
CELL DEVELOPMENT	571	18 (3.2)	13 (2.3)	5 (0.9)	0.0002	0.0079
REGULATION OF APOPTOSIS	338	13 (3.8)	10 (3)	3 (0.9)	0.0002	0.0079
DEFENSE RESPONSE TO VIRUS	10	3 (30)	3 (30)	0 (0)	0.0002	0.0079

Category Name	Category Genes #	Any-reg. SDEG #(%)	Up-reg. SDEG #(%)	Down-reg. SDEG #(%)	P	FDR

Table A83: Significantly enriched MSigDB GO Biological Processes (RNA-Seq, Monocytes, Day 1). Results sorted by FDR and #SDEG genes.

Category Name	Category Genes #	Any-reg. SDEG #(%)	Up-reg. SDEG #(%)	Down-reg. SDEG #(%)	P	FDR
MEMBRANE	1902	49 (2.6)	32 (1.7)	17 (0.9)	<0.0001	0.0023

Table A84: Significantly enriched MSigDB GO Cellular Components (RNA-Seq, Monocytes, Day 1). Results sorted by FDR and #SDEG genes.

Category Name	Category Genes #	Any-reg. SDEG #(%)	Up-reg. SDEG #(%)	Down-reg. SDEG #(%)	P	FDR
RECEPTOR ACTIVITY	554	20 (3.6)	11 (2)	9 (1.6)	<0.0001	0.0053
TRANSMEMBRANE RECEPTOR ACTIVITY	398	16 (4)	9 (2.3)	7 (1.8)	<0.0001	0.0053
HEMATOPOIETIN INTERFERON CLASSD200 DOMAIN CYTOKINE RECEPTOR ACTIVITY	29	5 (17.2)	3 (10.3)	2 (6.9)	<0.0001	0.0053

Table A85: Significantly enriched MSigDB GO Molecular Functions (RNA-Seq, Monocytes, Day 1). Results sorted by FDR and #SDEG genes.

Category Name	Category Genes #	Any-reg. SDEG #(%)	Up-reg. SDEG #(%)	Down-reg. SDEG #(%)	P	FDR
BIOCARTA IL22BP PATHWAY	16	4 (25)	4 (25)	0 (0)	<0.0001	0.0087

Table A86: Significantly enriched MSigDB BioCarta Pathways (RNA-Seq, Monocytes, Day 1). Results sorted by FDR and #SDEG genes.

Category Name	Category Genes #	Any-reg. SDEG #(%)	Up-reg. SDEG #(%)	Down-reg. SDEG #(%)	P	FDR
NUYTTEN EZH2 TARGETS UP	1002	70 (7)	66 (6.6)	4 (0.4)	<0.0001	0.0002
FULCHER INFLAMMATORY RESPONSE LECTIN VS LPS DN	462	64 (13.9)	63 (13.6)	1 (0.2)	<0.0001	0.0002
NUYTTEN NIPP1 TARGETS UP	748	49 (6.6)	46 (6.1)	3 (0.4)	<0.0001	0.0002
BLALOCK ALZHEIMERS DISEASE UP	1628	47 (2.9)	39 (2.4)	8 (0.5)	<0.0001	0.0002
GRAESSMANN APOPTOSIS BY DOXORUBICIN UP	1132	43 (3.8)	38 (3.4)	5 (0.4)	<0.0001	0.0002
HECKER IFNB1 TARGETS	92	43 (46.7)	42 (45.7)	1 (1.1)	<0.0001	0.0002
CHEN METABOLIC SYNDROM NETWORK	1172	41 (3.5)	28 (2.4)	13 (1.1)	<0.0001	0.0002
TAKEDA TARGETS OF NUP98 HOXA9 FUSION 3D UP	178	41 (23)	39 (21.9)	2 (1.1)	<0.0001	0.0002
GRAESSMANN APOPTOSIS BY SERUM DEPRIVATION UP	541	40 (7.4)	33 (6.1)	7 (1.3)	<0.0001	0.0002
GOZGIT ESR1 TARGETS DN	741	39 (5.3)	30 (4)	9 (1.2)	<0.0001	0.0002
WALLACE PROSTATE CANCER RACE UP	275	39 (14.2)	34 (12.4)	5 (1.8)	<0.0001	0.0002

Category Name	Category Genes #	Any-reg. SDEG #(%)	Up-reg. SDEG #(%)	Down-reg. SDEG #(%)	P	FDR
CHICAS RB1 TARGETS CONFLUENT	550	38 (6.9)	27 (4.9)	11 (2)	<0.0001	0.0002
TAKEDA TARGETS OF NUP98 HOXA9 FUSION 10D UP	186	38 (20.4)	32 (17.2)	6 (3.2)	<0.0001	0.0002
PEREZ TP53 TARGETS	1124	37 (3.3)	24 (2.1)	13 (1.2)	<0.0001	0.0002
CHICAS RB1 TARGETS SENESCENT	552	36 (6.5)	31 (5.6)	5 (0.9)	<0.0001	0.0002
BOSCO INTERFERON INDUCED ANTIVIRAL MODULE	76	34 (44.7)	34 (44.7)	0 (0)	<0.0001	0.0002
BROWNE INTERFERON RESPONSIVE GENES	68	34 (50)	34 (50)	0 (0)	<0.0001	0.0002
FEVR CTNNB1 TARGETS UP	672	34 (5.1)	29 (4.3)	5 (0.7)	<0.0001	0.0002
MARKEY RB1 ACUTE LOF UP	214	34 (15.9)	32 (15)	2 (0.9)	<0.0001	0.0002
FOSTER TOLERANT MACROPHAGE DN	405	33 (8.1)	29 (7.2)	4 (1)	<0.0001	0.0002
ZHOU INFLAMMATORY RESPONSE LPS UP	401	33 (8.2)	27 (6.7)	6 (1.5)	<0.0001	0.0002
JISON SICKLE CELL DISEASE UP	174	32 (18.4)	30 (17.2)	2 (1.1)	<0.0001	0.0002
LEE BMP2 TARGETS UP	723	32 (4.4)	21 (2.9)	11 (1.5)	<0.0001	0.0002
SANA RESPONSE TO IFNG UP	74	32 (43.2)	32 (43.2)	0 (0)	<0.0001	0.0002
ONKEN UVEAL MELANOMA UP	763	31 (4.1)	26 (3.4)	5 (0.7)	<0.0001	0.0002
YANG BCL3 TARGETS UP	351	31 (8.8)	26 (7.4)	5 (1.4)	<0.0001	0.0002
GRAESSMANN RESPONSE TO MC AND SERUM DEPRIVATION UP	203	30 (14.8)	27 (13.3)	3 (1.5)	<0.0001	0.0002
HELLER SILENCED BY METHYLATION UP	273	30 (11)	27 (9.9)	3 (1.1)	<0.0001	0.0002
TAKEDA TARGETS OF NUP98 HOXA9 FUSION 16D UP	169	30 (17.8)	25 (14.8)	5 (3)	<0.0001	0.0002
RODWELL AGING KIDNEY UP	465	29 (6.2)	27 (5.8)	2 (0.4)	<0.0001	0.0002
SMID BREAST CANCER LUMINAL B DN	513	29 (5.7)	22 (4.3)	7 (1.4)	<0.0001	0.0002
SMID BREAST CANCER NORMAL LIKE UP	443	29 (6.5)	20 (4.5)	9 (2)	<0.0001	0.0002
TAKEDA TARGETS OF NUP98 HOXA9 FUSION 8D UP	155	29 (18.7)	26 (16.8)	3 (1.9)	<0.0001	0.0002
BERTUCCI MEDULLARY VS DUCTAL BREAST CANCER UP	200	28 (14)	27 (13.5)	1 (0.5)	<0.0001	0.0002
BOQUEST STEM CELL CULTURED VS FRESH UP	409	28 (6.8)	20 (4.9)	8 (2)	<0.0001	0.0002
DEBIASI APOPTOSIS BY REOVIRUS INFECTION UP	317	28 (8.8)	27 (8.5)	1 (0.3)	<0.0001	0.0002
JOHNSTONE PARVB TARGETS 3 UP	425	28 (6.6)	22 (5.2)	6 (1.4)	<0.0001	0.0002
RUTELLA RESPONSE TO HGF UP	398	28 (7)	22 (5.5)	6 (1.5)	<0.0001	0.0002
SMID BREAST CANCER BASAL UP	607	28 (4.6)	24 (4)	4 (0.7)	<0.0001	0.0002
WONG ADULT TISSUE STEM MODULE	697	28 (4)	16 (2.3)	12 (1.7)	<0.0001	0.0002
SPIELMAN LYMPHOBLAST EUROPEAN VS ASIAN DN	570	26 (4.6)	23 (4)	3 (0.5)	<0.0001	0.0002
ALTEMEIER RESPONSE TO LPS WITH MECHANICAL VENTILATION	126	25 (19.8)	25 (19.8)	0 (0)	<0.0001	0.0002
DELYS THYROID CANCER UP	435	25 (5.7)	16 (3.7)	9 (2.1)	<0.0001	0.0002
VERHAAK AML WITH NPM1 MUTATED UP	190	25 (13.2)	22 (11.6)	3 (1.6)	<0.0001	0.0002
HIRSCH CELLULAR TRANSFORMATION SIGNATURE UP	239	23 (9.6)	20 (8.4)	3 (1.3)	<0.0001	0.0002
MOSERLE IFNA RESPONSE	31	23 (74.2)	23 (74.2)	0 (0)	<0.0001	0.0002
RUTELLA RESPONSE TO HGF VS CSF2RB AND IL4 UP	391	23 (5.9)	22 (5.6)	1 (0.3)	<0.0001	0.0002
BAELDE DIABETIC NEPHROPATHY DN	425	22 (5.2)	19 (4.5)	3 (0.7)	<0.0001	0.0002
ENK UV RESPONSE EPIDERMIS DN	496	22 (4.4)	13 (2.6)	9 (1.8)	<0.0001	0.0002

Category Name	Category Genes #	Any-reg. SDEG #(%)	Up-reg. SDEG #(%)	Down-reg. SDEG #(%)	P	FDR
HORIUCHI WTAP TARGETS UP	295	22 (7.5)	20 (6.8)	2 (0.7)	<0.0001	0.0002
MASSARWEH TAMOXIFEN RESISTANCE UP	561	22 (3.9)	14 (2.5)	8 (1.4)	<0.0001	0.0002
ZHANG RESPONSE TO IKK INHIBITOR AND TNF UP	221	22 (10)	21 (9.5)	1 (0.5)	<0.0001	0.0002
DUTERTRE ESTRADIOL RESPONSE 24HR DN	502	21 (4.2)	15 (3)	6 (1.2)	<0.0001	0.0002
ICHIBA GRAFT VERSUS HOST DISEASE D7 UP	105	21 (20)	20 (19)	1 (1)	<0.0001	0.0002
RUTELLA RESPONSE TO CSF2RB AND IL4 UP	319	21 (6.6)	13 (4.1)	8 (2.5)	<0.0001	0.0002
DAUER STAT3 TARGETS DN	51	20 (39.2)	20 (39.2)	0 (0)	<0.0001	0.0002
KUMAR TARGETS OF MLL AF9 FUSION	389	20 (5.1)	15 (3.9)	5 (1.3)	<0.0001	0.0002
LI INDUCED T TO NATURAL KILLER UP	302	20 (6.6)	16 (5.3)	4 (1.3)	<0.0001	0.0002
LIU PROSTATE CANCER DN	455	20 (4.4)	10 (2.2)	10 (2.2)	<0.0001	0.0002
MITSIADES RESPONSE TO APLIDIN UP	450	20 (4.4)	15 (3.3)	5 (1.1)	<0.0001	0.0002
PHONG TNF RESPONSE NOT VIA P38	333	20 (6)	20 (6)	0 (0)	<0.0001	0.0002
POOLA INVASIVE BREAST CANCER UP	272	20 (7.4)	17 (6.2)	3 (1.1)	<0.0001	0.0002
RICKMAN METASTASIS DN	258	20 (7.8)	17 (6.6)	3 (1.2)	<0.0001	0.0002
VERHAAK AML WITH NPM1 MUTATED DN	238	20 (8.4)	12 (5)	8 (3.4)	<0.0001	0.0002
BOSCO TH1 CYTOTOXIC MODULE	114	19 (16.7)	18 (15.8)	1 (0.9)	<0.0001	0.0002
LINDGREN BLADDER CANCER CLUSTER 2B	376	19 (5.1)	16 (4.3)	3 (0.8)	<0.0001	0.0002
RADAEVA RESPONSE TO IFNA1 UP	52	19 (36.5)	19 (36.5)	0 (0)	<0.0001	0.0002
RUTELLA RESPONSE TO CSF2RB AND IL4 DN	303	19 (6.3)	17 (5.6)	2 (0.7)	<0.0001	0.0002
SEITZ NEOPLASTIC TRANSFORMATION BY 8P DELETION UP	69	19 (27.5)	18 (26.1)	1 (1.4)	<0.0001	0.0002
WIELAND UP BY HBV INFECTION	100	19 (19)	19 (19)	0 (0)	<0.0001	0.0002
BENNETT SYSTEMIC LUPUS ERYTHEMATOSUS	32	18 (56.2)	18 (56.2)	0 (0)	<0.0001	0.0002
CHANG CORE SERUM RESPONSE DN	205	18 (8.8)	12 (5.9)	6 (2.9)	<0.0001	0.0002
COLINA TARGETS OF 4EBP1 AND 4EBP2	339	18 (5.3)	17 (5)	1 (0.3)	<0.0001	0.0002
DER IFN BETA RESPONSE UP	101	18 (17.8)	18 (17.8)	0 (0)	<0.0001	0.0002
ODONNELL TFRC TARGETS UP	409	18 (4.4)	15 (3.7)	3 (0.7)	<0.0001	0.0002
RIGGINS TAMOXIFEN RESISTANCE DN	215	18 (8.4)	17 (7.9)	1 (0.5)	<0.0001	0.0002
SATO SILENCED BY METHYLATION IN PANCREATIC CANCER 1	412	18 (4.4)	13 (3.2)	5 (1.2)	<0.0001	0.0002
VANTVEER BREAST CANCER ESR1 DN	232	18 (7.8)	18 (7.8)	0 (0)	<0.0001	0.0002
DER IFN ALPHA RESPONSE UP	74	17 (23)	17 (23)	0 (0)	<0.0001	0.0002
DURAND STROMA MAX UP	270	17 (6.3)	12 (4.4)	5 (1.9)	<0.0001	0.0002
LEI MYB TARGETS	307	17 (5.5)	14 (4.6)	3 (1)	<0.0001	0.0002
MCLACHLAN DENTAL CARIES UP	227	17 (7.5)	17 (7.5)	0 (0)	<0.0001	0.0002
PICCALUGA ANGIOIMMUNOBLASTIC LYMPHOMA UP	205	17 (8.3)	15 (7.3)	2 (1)	<0.0001	0.0002
ROZANOV MMP14 TARGETS UP	270	17 (6.3)	12 (4.4)	5 (1.9)	<0.0001	0.0002
RUTELLA RESPONSE TO HGF VS CSF2RB AND IL4 DN	231	17 (7.4)	8 (3.5)	9 (3.9)	<0.0001	0.0002
SCHUETZ BREAST CANCER DUCTAL INVASIVE UP	338	17 (5)	11 (3.3)	6 (1.8)	<0.0001	0.0002
ZHU CMV ALL UP	121	17 (14)	17 (14)	0 (0)	<0.0001	0.0002

Category Name	Category Genes #	Any-reg. SDEG #(%)	Up-reg. SDEG #(%)	Down-reg. SDEG #(%)	P	FDR
BROCKE APOPTOSIS REVERSED BY IL6	144	16 (11.1)	15 (10.4)	1 (0.7)	<0.0001	0.0002
DANG REGULATED BY MYC DN	247	16 (6.5)	14 (5.7)	2 (0.8)	<0.0001	0.0002
LU EZH2 TARGETS UP	289	16 (5.5)	12 (4.2)	4 (1.4)	<0.0001	0.0002
SANA TNF SIGNALING UP	81	16 (19.8)	16 (19.8)	0 (0)	<0.0001	0.0002
TONKS TARGETS OF RUNX1 RUNX1T1 FUSION ERYTHROCYTE UP	151	16 (10.6)	14 (9.3)	2 (1.3)	<0.0001	0.0002
ACEVEDO FGFR1 TARGETS IN PROSTATE CANCER MODEL UP	278	15 (5.4)	13 (4.7)	2 (0.7)	<0.0001	0.0002
HOLLMANN APOPTOSIS VIA CD40 DN	259	15 (5.8)	9 (3.5)	6 (2.3)	<0.0001	0.0002
ICHIBA GRAFT VERSUS HOST DISEASE 35D UP	126	15 (11.9)	13 (10.3)	2 (1.6)	<0.0001	0.0002
MCLACHLAN DENTAL CARIES DN	219	15 (6.8)	15 (6.8)	0 (0)	<0.0001	0.0002
PLASARI TGFB1 TARGETS 10HR DN	233	15 (6.4)	11 (4.7)	4 (1.7)	<0.0001	0.0002
SENGUPTA NASOPHARYNGEAL CARCINOMA UP	287	15 (5.2)	14 (4.9)	1 (0.3)	<0.0001	0.0002
VERHAAK GLIOBLASTOMA NEURAL	205	15 (7.3)	10 (4.9)	5 (2.4)	<0.0001	0.0002
WANG MLL TARGETS	280	15 (5.4)	12 (4.3)	3 (1.1)	<0.0001	0.0002
DER IFN GAMMA RESPONSE UP	72	14 (19.4)	14 (19.4)	0 (0)	<0.0001	0.0002
FARMER BREAST CANCER CLUSTER 1	39	14 (35.9)	13 (33.3)	1 (2.6)	<0.0001	0.0002
HUTTMANN B CLL POOR SURVIVAL UP	258	14 (5.4)	8 (3.1)	6 (2.3)	<0.0001	0.0002
MISSIAGLIA REGULATED BY METHYLATION UP	120	14 (11.7)	11 (9.2)	3 (2.5)	<0.0001	0.0002
QI PLASMACYTOMA UP	251	14 (5.6)	13 (5.2)	1 (0.4)	<0.0001	0.0002
TONKS TARGETS OF RUNX1 RUNX1T1 FUSION MONOCYTE UP	201	14 (7)	11 (5.5)	3 (1.5)	<0.0001	0.0002
ZHU CMV 8 HR UP	48	14 (29.2)	14 (29.2)	0 (0)	<0.0001	0.0002
ALCALAY AML BY NPM1 LOCALIZATION DN	178	13 (7.3)	8 (4.5)	5 (2.8)	<0.0001	0.0002
LEE RECENT THYMIC EMIGRANT	217	13 (6)	11 (5.1)	2 (0.9)	<0.0001	0.0002
MIKKELSEN MCV6 LCP WITH H3K4ME3	153	13 (8.5)	12 (7.8)	1 (0.7)	<0.0001	0.0002
RIGGI EWING SARCOMA PROGENITOR DN	185	13 (7)	8 (4.3)	5 (2.7)	<0.0001	0.0002
STAMBOLSKY TARGETS OF MUTATED TP53 DN	51	13 (25.5)	12 (23.5)	1 (2)	<0.0001	0.0002
UROSEVIC RESPONSE TO IMIQUIMOD	23	13 (56.5)	13 (56.5)	0 (0)	<0.0001	0.0002
WIERENGA STAT5A TARGETS UP	210	13 (6.2)	12 (5.7)	1 (0.5)	<0.0001	0.0002
ZHANG INTERFERON RESPONSE	23	13 (56.5)	13 (56.5)	0 (0)	<0.0001	0.0002
KRASNOSELSKAYA ILF3 TARGETS UP	37	12 (32.4)	12 (32.4)	0 (0)	<0.0001	0.0002
LIANG SILENCED BY METHYLATION 2	55	12 (21.8)	12 (21.8)	0 (0)	<0.0001	0.0002
NAKAYAMA SOFT TISSUE TUMORS PCA1 UP	69	12 (17.4)	12 (17.4)	0 (0)	<0.0001	0.0002
SEKI INFLAMMATORY RESPONSE LPS UP	77	12 (15.6)	12 (15.6)	0 (0)	<0.0001	0.0002
TAKEDA TARGETS OF NUP98 HOXA9 FUSION 8D DN	193	12 (6.2)	4 (2.1)	8 (4.1)	<0.0001	0.0002
BOWIE RESPONSE TO EXTRACELLULAR MATRIX	17	11 (64.7)	11 (64.7)	0 (0)	<0.0001	0.0002
BOWIE RESPONSE TO TAMOXIFEN	18	11 (61.1)	11 (61.1)	0 (0)	<0.0001	0.0002
EINAV INTERFERON SIGNATURE IN CANCER	27	11 (40.7)	11 (40.7)	0 (0)	<0.0001	0.0002
JECHLINGER EPITHELIAL TO MESENCHYMAL TRANSITION UP	71	11 (15.5)	8 (11.3)	3 (4.2)	<0.0001	0.0002
KATSANOU ELAVL1 TARGETS UP	163	11 (6.7)	8 (4.9)	3 (1.8)	<0.0001	0.0002

Category Name	Category Genes #	Any-reg. SDEG #(%)	Up-reg. SDEG #(%)	Down-reg. SDEG #(%)	P	FDR
SARRIO EPITHELIAL MESENCHYMAL TRANSITION DN	149	11 (7.4)	9 (6)	2 (1.3)	<0.0001	0.0002
SMIRNOV CIRCULATING ENDOTHELIOCYTES IN CANCER UP	152	11 (7.2)	9 (5.9)	2 (1.3)	<0.0001	0.0002
CHANG IMMORTALIZED BY HPV31 DN	63	10 (15.9)	10 (15.9)	0 (0)	<0.0001	0.0002
FLECHNER BIOPSY KIDNEY TRANSPLANT REJECTED VS OK UP	85	10 (11.8)	9 (10.6)	1 (1.2)	<0.0001	0.0002
GERY CEBP TARGETS	137	10 (7.3)	6 (4.4)	4 (2.9)	<0.0001	0.0002
HINATA NFKB TARGETS KERATINOCYTE UP	91	10 (11)	8 (8.8)	2 (2.2)	<0.0001	0.0002
ZHU CMV 24 HR UP	93	10 (10.8)	10 (10.8)	0 (0)	<0.0001	0.0002
BROWNE HCMV INFECTION 12HR UP	105	9 (8.6)	9 (8.6)	0 (0)	<0.0001	0.0002
DAUER STAT3 TARGETS UP	50	9 (18)	9 (18)	0 (0)	<0.0001	0.0002
GHANDHI BYSTANDER IRRADIATION UP	78	9 (11.5)	9 (11.5)	0 (0)	<0.0001	0.0002
JACKSON DNMT1 TARGETS UP	76	9 (11.8)	8 (10.5)	1 (1.3)	<0.0001	0.0002
LENAOUR DENDRITIC CELL MATURATION UP	112	9 (8)	7 (6.2)	2 (1.8)	<0.0001	0.0002
LINDSTEDT DENDRITIC CELL MATURATION A	67	9 (13.4)	9 (13.4)	0 (0)	<0.0001	0.0002
LIU VAV3 PROSTATE CARCINOGENESIS UP	85	9 (10.6)	9 (10.6)	0 (0)	<0.0001	0.0002
ROSS AML WITH MLL FUSIONS	76	9 (11.8)	7 (9.2)	2 (2.6)	<0.0001	0.0002
SHAFFER IRF4 TARGETS IN ACTIVATED DENDRITIC CELL	61	9 (14.8)	7 (11.5)	2 (3.3)	<0.0001	0.0002
TAVOR CEBPA TARGETS UP	47	9 (19.1)	7 (14.9)	2 (4.3)	<0.0001	0.0002
WUNDER INFLAMMATORY RESPONSE AND CHOLESTEROL UP	61	9 (14.8)	8 (13.1)	1 (1.6)	<0.0001	0.0002
ZHAN MULTIPLE MYELOMA LB DN	39	9 (23.1)	9 (23.1)	0 (0)	<0.0001	0.0002
BRUECKNER TARGETS OF MIRLET7A3 DN	74	8 (10.8)	6 (8.1)	2 (2.7)	<0.0001	0.0002
GRANDVAUX IFN RESPONSE NOT VIA IRF3	14	8 (57.1)	8 (57.1)	0 (0)	<0.0001	0.0002
GRANDVAUX IRF3 TARGETS UP	15	8 (53.3)	8 (53.3)	0 (0)	<0.0001	0.0002
ROETH TERT TARGETS UP	14	8 (57.1)	8 (57.1)	0 (0)	<0.0001	0.0002
SHAFFER IRF4 TARGETS IN PLASMA CELL VS MATURE B LYMPHOCYTE	64	8 (12.5)	6 (9.4)	2 (3.1)	<0.0001	0.0002
XU AKT1 TARGETS 6HR	27	8 (29.6)	8 (29.6)	0 (0)	<0.0001	0.0002
ZHENG IL22 SIGNALING UP	56	8 (14.3)	6 (10.7)	2 (3.6)	<0.0001	0.0002
CASTELLANO NRAS TARGETS UP	68	7 (10.3)	6 (8.8)	1 (1.5)	<0.0001	0.0002
FUJII YBX1 TARGETS UP	40	7 (17.5)	7 (17.5)	0 (0)	<0.0001	0.0002
GEISS RESPONSE TO DSRNA UP	35	7 (20)	7 (20)	0 (0)	<0.0001	0.0002
TSAI RESPONSE TO RADIATION THERAPY	32	7 (21.9)	7 (21.9)	0 (0)	<0.0001	0.0002
YAN ESCAPE FROM ANOIKIS	24	7 (29.2)	7 (29.2)	0 (0)	<0.0001	0.0002
ZHANG ANTIVIRAL RESPONSE TO RIBAVIRIN UP	29	7 (24.1)	7 (24.1)	0 (0)	<0.0001	0.0002
BURTON ADIPOGENESIS 7	45	6 (13.3)	5 (11.1)	1 (2.2)	<0.0001	0.0002
CHIBA RESPONSE TO TSA	48	6 (12.5)	5 (10.4)	1 (2.1)	<0.0001	0.0002
HAN JNK SINGALING UP	34	6 (17.6)	6 (17.6)	0 (0)	<0.0001	0.0002
KIM LRRC3B TARGETS	30	6 (20)	6 (20)	0 (0)	<0.0001	0.0002
MAHADEVAN RESPONSE TO MP470 UP	19	6 (31.6)	6 (31.6)	0 (0)	<0.0001	0.0002
TSAI DNAJB4 TARGETS UP	13	6 (46.2)	6 (46.2)	0 (0)	<0.0001	0.0002

Category Name	Category Genes #	Any-reg. SDEG #(%)	Up-reg. SDEG #(%)	Down-reg. SDEG #(%)	P	FDR
XU HGF TARGETS INDUCED BY AKT1 6HR	18	6 (33.3)	6 (33.3)	0 (0)	<0.0001	0.0002
RUAN RESPONSE TO TNF TROGLITAZONE UP	17	5 (29.4)	5 (29.4)	0 (0)	<0.0001	0.0002
RUAN RESPONSE TO TNF UP	12	5 (41.7)	5 (41.7)	0 (0)	<0.0001	0.0002
STEGER ADIPOGENESIS UP	21	5 (23.8)	5 (23.8)	0 (0)	<0.0001	0.0002
WATANABE ULCERATIVE COLITIS WITH CANCER DN	13	5 (38.5)	5 (38.5)	0 (0)	<0.0001	0.0002
FORTSCHEGGER PHF8 TARGETS DN	749	26 (3.5)	20 (2.7)	6 (0.8)	<0.0001	0.0004
KRIGE RESPONSE TO TOSEDOSTAT 24HR UP	740	25 (3.4)	23 (3.1)	2 (0.3)	<0.0001	0.0004
KRIEG HYPOXIA NOT VIA KDM3A	718	24 (3.3)	18 (2.5)	6 (0.8)	<0.0001	0.0004
CHARAFE BREAST CANCER LUMINAL VS BASAL DN	440	21 (4.8)	20 (4.5)	1 (0.2)	<0.0001	0.0004
ACEVEDO LIVER CANCER DN	533	20 (3.8)	12 (2.3)	8 (1.5)	<0.0001	0.0004
ZHOU INFLAMMATORY RESPONSE LIVE UP	448	19 (4.2)	15 (3.3)	4 (0.9)	<0.0001	0.0004
SWEET LUNG CANCER KRAS DN	425	17 (4)	10 (2.4)	7 (1.6)	<0.0001	0.0004
WANG SMARCE1 TARGETS DN	340	17 (5)	14 (4.1)	3 (0.9)	<0.0001	0.0004
WANG SMARCE1 TARGETS UP	265	15 (5.7)	11 (4.2)	4 (1.5)	<0.0001	0.0004
GAUSSMANN MLL AF4 FUSION TARGETS F UP	182	12 (6.6)	8 (4.4)	4 (2.2)	<0.0001	0.0004
FOSTER TOLERANT MACROPHAGE UP	155	11 (7.1)	11 (7.1)	0 (0)	<0.0001	0.0004
KRIEG KDM3A TARGETS NOT HYPOXIA	189	11 (5.8)	11 (5.8)	0 (0)	<0.0001	0.0004
JOHNSTONE PARVB TARGETS 2 UP	136	9 (6.6)	9 (6.6)	0 (0)	<0.0001	0.0004
MAHAJAN RESPONSE TO IL1A UP	79	9 (11.4)	7 (8.9)	2 (2.5)	<0.0001	0.0004
TAKEDA TARGETS OF NUP98 HOXA9 FUSION 10D DN	135	9 (6.7)	3 (2.2)	6 (4.4)	<0.0001	0.0004
WANG ESOPHAGUS CANCER VS NORMAL UP	118	9 (7.6)	7 (5.9)	2 (1.7)	<0.0001	0.0004
ZHONG SECRETOME OF LUNG CANCER AND FIBROBLAST	129	9 (7)	6 (4.7)	3 (2.3)	<0.0001	0.0004
BROWNE HCMV INFECTION 4HR UP	53	7 (13.2)	7 (13.2)	0 (0)	<0.0001	0.0004
LEE LIVER CANCER E2F1 UP	62	7 (11.3)	5 (8.1)	2 (3.2)	<0.0001	0.0004
LIEN BREAST CARCINOMA METAPLASTIC VS DUCTAL UP	78	7 (9)	6 (7.7)	1 (1.3)	<0.0001	0.0004
BECKER TAMOXIFEN RESISTANCE UP	49	6 (12.2)	6 (12.2)	0 (0)	<0.0001	0.0004
MUELLER COMMON TARGETS OF AML FUSIONS DN	42	6 (14.3)	5 (11.9)	1 (2.4)	<0.0001	0.0004
MURATA VIRULENCE OF H PILORI	23	5 (21.7)	5 (21.7)	0 (0)	<0.0001	0.0004
SHARMA PILOCYTIC ASTROCYTOMA LOCATION UP	25	5 (20)	3 (12)	2 (8)	<0.0001	0.0004
ZWANG CLASS 1 TRANSIENTLY INDUCED BY EGF	505	19 (3.8)	13 (2.6)	6 (1.2)	<0.0001	0.0005
BASAKI YBX1 TARGETS DN	361	16 (4.4)	14 (3.9)	2 (0.6)	<0.0001	0.0005
TARTE PLASMA CELL VS PLASMABLAST UP	388	15 (3.9)	14 (3.6)	1 (0.3)	<0.0001	0.0005
ZWANG CLASS 3 TRANSIENTLY INDUCED BY EGF	220	13 (5.9)	12 (5.5)	1 (0.5)	<0.0001	0.0005
GAL LEUKEMIC STEM CELL DN	244	12 (4.9)	10 (4.1)	2 (0.8)	<0.0001	0.0005
LEE LIVER CANCER SURVIVAL UP	167	11 (6.6)	7 (4.2)	4 (2.4)	<0.0001	0.0005
HESS TARGETS OF HOXA9 AND MEIS1 DN	76	8 (10.5)	7 (9.2)	1 (1.3)	<0.0001	0.0005
GUTIERREZ CHRONIC LYMPHOCYTIC LEUKEMIA DN	56	7 (12.5)	4 (7.1)	3 (5.4)	<0.0001	0.0005
DASU IL6 SIGNALING UP	59	6 (10.2)	5 (8.5)	1 (1.7)	<0.0001	0.0005

Category Name	Category Genes #	Any-reg. SDEG #(%)	Up-reg. SDEG #(%)	Down-reg. SDEG #(%)	P	FDR
ONDER CDH1 TARGETS 2 UP	235	13 (5.5)	8 (3.4)	5 (2.1)	<0.0001	0.0007
MIKKELSEN MEF LCP WITH H3K4ME3	121	9 (7.4)	7 (5.8)	2 (1.7)	<0.0001	0.0007
NEMETH INFLAMMATORY RESPONSE LPS UP	85	8 (9.4)	8 (9.4)	0 (0)	<0.0001	0.0007
GARGALOVIC RESPONSE TO OXIDIZED PHOSPHOLIPIDS GREY DN	73	7 (9.6)	7 (9.6)	0 (0)	<0.0001	0.0007
LINDSTEDT DENDRITIC CELL MATURATION B	51	6 (11.8)	6 (11.8)	0 (0)	<0.0001	0.0007
XU RESPONSE TO TRETINOIN AND NSC682994 UP	16	4 (25)	4 (25)	0 (0)	<0.0001	0.0007
KOYAMA SEMA3B TARGETS DN	394	16 (4.1)	14 (3.6)	2 (0.5)	<0.0001	0.0008
THUM SYSTOLIC HEART FAILURE UP	404	16 (4)	15 (3.7)	1 (0.2)	<0.0001	0.0008
SANSOM APC TARGETS DN	359	15 (4.2)	12 (3.3)	3 (0.8)	<0.0001	0.0008
RUTELLA RESPONSE TO HGF DN	224	12 (5.4)	8 (3.6)	4 (1.8)	<0.0001	0.0008
CAIRO HEPATOBLASTOMA CLASSES DN	204	11 (5.4)	6 (2.9)	5 (2.5)	<0.0001	0.0008
BROWNE HCMV INFECTION 8HR UP	102	8 (7.8)	7 (6.9)	1 (1)	<0.0001	0.0008
SHAFFER IRF4 TARGETS IN MYELOMA VS MATURE B LYMPHOCYTE	97	8 (8.2)	7 (7.2)	1 (1)	<0.0001	0.0008
FURUKAWA DUSP6 TARGETS PCI35 UP	73	7 (9.6)	7 (9.6)	0 (0)	<0.0001	0.0008
YAO HOXA10 TARGETS VIA PROGESTERONE UP	79	7 (8.9)	4 (5.1)	3 (3.8)	<0.0001	0.0008
ZWANG TRANSIENTLY UP BY 2ND EGF PULSE ONLY	1568	37 (2.4)	20 (1.3)	17 (1.1)	<0.0001	0.0009
SENGUPTA NASOPHARYNGEAL CARCINOMA WITH LMP1 UP	387	17 (4.4)	14 (3.6)	3 (0.8)	<0.0001	0.0009
SERVITJA ISLET HNF1A TARGETS UP	160	10 (6.2)	8 (5)	2 (1.2)	<0.0001	0.0009
DIAZ CHRONIC MEYLOGENOUS LEUKEMIA DN	112	8 (7.1)	7 (6.2)	1 (0.9)	<0.0001	0.0009
KIM GLIS2 TARGETS UP	84	7 (8.3)	7 (8.3)	0 (0)	<0.0001	0.0009
DIRMEIER LMP1 RESPONSE EARLY	67	6 (9)	5 (7.5)	1 (1.5)	<0.0001	0.0009
KRIGE RESPONSE TO TOSEDOSTAT 6HR UP	915	27 (3)	24 (2.6)	3 (0.3)	<0.0001	0.0011
WIERENGA STAT5A TARGETS GROUP1	130	9 (6.9)	8 (6.2)	1 (0.8)	<0.0001	0.0011
YIH RESPONSE TO ARSENITE C5	9	3 (33.3)	3 (33.3)	0 (0)	<0.0001	0.0011
DODD NASOPHARYNGEAL CARCINOMA DN	1330	35 (2.6)	30 (2.3)	5 (0.4)	<0.0001	0.0012
ALFANO MYC TARGETS	234	12 (5.1)	9 (3.8)	3 (1.3)	<0.0001	0.0012
KIM WT1 TARGETS 12HR DN	206	11 (5.3)	9 (4.4)	2 (1)	<0.0001	0.0012
CHIARADONNA NEOPLASTIC TRANSFORMATION KRAS DN	143	9 (6.3)	6 (4.2)	3 (2.1)	<0.0001	0.0012
CREIGHTON ENDOCRINE THERAPY RESISTANCE 3	696	22 (3.2)	16 (2.3)	6 (0.9)	<0.0001	0.0013
FLECHNER BIOPSY KIDNEY TRANSPLANT OK VS DONOR UP	529	19 (3.6)	18 (3.4)	1 (0.2)	<0.0001	0.0013
CHICAS RB1 TARGETS GROWING	233	12 (5.2)	8 (3.4)	4 (1.7)	<0.0001	0.0013
GAVIN FOXP3 TARGETS CLUSTER P2	77	7 (9.1)	6 (7.8)	1 (1.3)	<0.0001	0.0013
KANG GIST WITH PDGFRA UP	50	6 (12)	5 (10)	1 (2)	<0.0001	0.0013
NIELSEN SYNOVIAL SARCOMA DN	19	4 (21.1)	4 (21.1)	0 (0)	<0.0001	0.0013
SARTIPY BLUNTED BY INSULIN RESISTANCE UP	19	4 (21.1)	4 (21.1)	0 (0)	<0.0001	0.0014
ONDER CDH1 TARGETS 2 DN	454	17 (3.7)	13 (2.9)	4 (0.9)	0.0001	0.0016
RODRIGUES THYROID CARCINOMA DN	75	7 (9.3)	6 (8)	1 (1.3)	0.0001	0.0016
GUO HEX TARGETS DN	67	6 (9)	4 (6)	2 (3)	0.0001	0.0016

Category Name	Category Genes #	Any-reg. SDEG #(%)	Up-reg. SDEG #(%)	Down-reg. SDEG #(%)	P	FDR
MCBRYAN PUBERTAL TGFB1 TARGETS DN	65	6 (9.2)	4 (6.2)	2 (3.1)	0.0001	0.0016
MONNIER POSTRADIATION TUMOR ESCAPE DN	357	14 (3.9)	12 (3.4)	2 (0.6)	0.0001	0.0018
OSWALD HEMATOPOIETIC STEM CELL IN COLLAGEN GEL UP	227	11 (4.8)	9 (4)	2 (0.9)	0.0001	0.0018
BROWN MYELOID CELL DEVELOPMENT UP	159	9 (5.7)	9 (5.7)	0 (0)	0.0001	0.0018
VALK AML WITH FLT3 ITD	38	5 (13.2)	3 (7.9)	2 (5.3)	0.0001	0.0018
ZWANG TRANSIENTLY UP BY 1ST EGF PULSE ONLY	1723	39 (2.3)	32 (1.9)	7 (0.4)	0.0001	0.0019
BRUINS UVC RESPONSE LATE	1111	29 (2.6)	22 (2)	7 (0.6)	0.0001	0.0019
IVANOVA HEMATOPOIESIS STEM CELL AND PROGENITOR	654	21 (3.2)	11 (1.7)	10 (1.5)	0.0001	0.0019
MEISSNER BRAIN HCP WITH H3K4ME3 AND H3K27ME3	1060	29 (2.7)	17 (1.6)	12 (1.1)	0.0001	0.002
RODRIGUES THYROID CARCINOMA ANAPLASTIC UP	707	22 (3.1)	19 (2.7)	3 (0.4)	0.0001	0.002
MEISSNER NPC HCP WITH H3K4ME2	477	17 (3.6)	10 (2.1)	7 (1.5)	0.0001	0.002
YAMAZAKI TCEB3 TARGETS UP	172	10 (5.8)	7 (4.1)	3 (1.7)	0.0001	0.002
SCHAEFFER PROSTATE DEVELOPMENT 48HR DN	411	16 (3.9)	6 (1.5)	10 (2.4)	0.0002	0.0021
VECCHI GASTRIC CANCER EARLY UP	413	15 (3.6)	7 (1.7)	8 (1.9)	0.0002	0.0021
DIRMEIER LMP1 RESPONSE LATE DN	30	4 (13.3)	3 (10)	1 (3.3)	0.0002	0.0021
HAN SATB1 TARGETS DN	421	16 (3.8)	15 (3.6)	1 (0.2)	0.0002	0.0025
PURBEY TARGETS OF CTBP1 NOT SATB1 DN	412	15 (3.6)	9 (2.2)	6 (1.5)	0.0002	0.0025
ZHONG SECRETOME OF LUNG CANCER AND MACROPHAGE	75	6 (8)	6 (8)	0 (0)	0.0002	0.0025
HWANG PROSTATE CANCER MARKERS	26	4 (15.4)	4 (15.4)	0 (0)	0.0002	0.0026
SHETH LIVER CANCER VS TXNIP LOSS PAM1	217	10 (4.6)	6 (2.8)	4 (1.8)	0.0002	0.0028
PEDRIOLI MIR31 TARGETS DN	398	15 (3.8)	13 (3.3)	2 (0.5)	0.0002	0.0029
PEREZ TP63 TARGETS	343	14 (4.1)	10 (2.9)	4 (1.2)	0.0002	0.003
FOSTER KDM1A TARGETS UP	250	11 (4.4)	10 (4)	1 (0.4)	0.0002	0.003
MULLIGHAN NPM1 SIGNATURE 3 DN	155	9 (5.8)	6 (3.9)	3 (1.9)	0.0002	0.003
SASAKI ADULT T CELL LEUKEMIA	186	9 (4.8)	8 (4.3)	1 (0.5)	0.0002	0.003
HAN SATB1 TARGETS UP	380	14 (3.7)	9 (2.4)	5 (1.3)	0.0002	0.0031
BAUS TFF2 TARGETS UP	32	4 (12.5)	4 (12.5)	0 (0)	0.0002	0.0031
HELLER HDAC TARGETS SILENCED BY METHYLATION UP	439	15 (3.4)	11 (2.5)	4 (0.9)	0.0002	0.0031
DOANE RESPONSE TO ANDROGEN DN	232	11 (4.7)	9 (3.9)	2 (0.9)	0.0002	0.0031
ELVIDGE HYPOXIA UP	166	9 (5.4)	7 (4.2)	2 (1.2)	0.0002	0.0031
VANHARANTA UTERINE FIBROID DN	66	6 (9.1)	4 (6.1)	2 (3)	0.0002	0.0031
ELVIDGE HYPOXIA BY DMOG UP	126	8 (6.3)	6 (4.8)	2 (1.6)	0.0003	0.0032
GHANDHI DIRECT IRRADIATION UP	106	7 (6.6)	7 (6.6)	0 (0)	0.0003	0.0032
PELLICCIOTTA HDAC IN ANTIGEN PRESENTATION DN	51	5 (9.8)	5 (9.8)	0 (0)	0.0003	0.0032
NOJIMA SFRP2 TARGETS DN	25	4 (16)	4 (16)	0 (0)	0.0003	0.0032
HOSHIDA LIVER CANCER SUBCLASS S3	259	11 (4.2)	10 (3.9)	1 (0.4)	0.0003	0.0033
ZHONG SECRETOME OF LUNG CANCER AND ENDOTHELIUM	66	6 (9.1)	5 (7.6)	1 (1.5)	0.0003	0.0034
ZHONG RESPONSE TO AZACITIDINE AND TSA UP	180	9 (5)	5 (2.8)	4 (2.2)	0.0003	0.0035

Category Name	Category Genes #	Any-reg. SDEG #(%)	Up-reg. SDEG #(%)	Down-reg. SDEG #(%)	P	FDR
JI RESPONSE TO FSH UP	85	6 (7.1)	6 (7.1)	0 (0)	0.0003	0.0035
LABBE TARGETS OF TGFB1 AND WNT3A DN	106	7 (6.6)	7 (6.6)	0 (0)	0.0003	0.0036
NATSUME RESPONSE TO INTERFERON BETA UP	71	6 (8.5)	5 (7)	1 (1.4)	0.0003	0.0036
SERVITJA LIVER HNF1A TARGETS UP	135	8 (5.9)	3 (2.2)	5 (3.7)	0.0003	0.0037
KIM RESPONSE TO TSA AND DECITABINE UP	120	7 (5.8)	6 (5)	1 (0.8)	0.0003	0.0038
GILDEA METASTASIS	30	4 (13.3)	2 (6.7)	2 (6.7)	0.0003	0.0038
LU TUMOR VASCULATURE UP	27	4 (14.8)	4 (14.8)	0 (0)	0.0003	0.0038
YAGI AML WITH 11Q23 REARRANGED	332	13 (3.9)	11 (3.3)	2 (0.6)	0.0003	0.0039
ZHAN MULTIPLE MYELOMA HP UP	50	5 (10)	4 (8)	1 (2)	0.0003	0.0039
LEE AGING CEREBELLUM UP	81	6 (7.4)	6 (7.4)	0 (0)	0.0003	0.004
BOYLAN MULTIPLE MYELOMA PCA3 UP	76	6 (7.9)	4 (5.3)	2 (2.6)	0.0003	0.0041
WHITESIDE CISPLATIN RESISTANCE UP	11	3 (27.3)	3 (27.3)	0 (0)	0.0003	0.0041
HILLION HMGA1B TARGETS	90	6 (6.7)	4 (4.4)	2 (2.2)	0.0004	0.0042
MARTORIATI MDM4 TARGETS FETAL LIVER UP	224	10 (4.5)	6 (2.7)	4 (1.8)	0.0004	0.0043
VILIMAS NOTCH1 TARGETS UP	51	5 (9.8)	4 (7.8)	1 (2)	0.0004	0.0043
CROONQUIST STROMAL STIMULATION DN	12	3 (25)	2 (16.7)	1 (8.3)	0.0004	0.0043
CAIRO HEPATOBLASTOMA DN	259	11 (4.2)	8 (3.1)	3 (1.2)	0.0004	0.0044
GRUETZMANN PANCREATIC CANCER UP	351	13 (3.7)	11 (3.1)	2 (0.6)	0.0004	0.0045
IIZUKA LIVER CANCER PROGRESSION G1 G2 UP	12	3 (25)	3 (25)	0 (0)	0.0004	0.0045
CASORELLI ACUTE PROMYELOCYTIC LEUKEMIA DN	624	19 (3)	14 (2.2)	5 (0.8)	0.0004	0.0045
TORCHIA TARGETS OF EWSR1 FLI1 FUSION DN	306	12 (3.9)	11 (3.6)	1 (0.3)	0.0004	0.0045
DEMAGALHAES AGING UP	56	5 (8.9)	5 (8.9)	0 (0)	0.0004	0.0045
GRAHAM CML QUIESCENT VS NORMAL DIVIDING UP	55	5 (9.1)	3 (5.5)	2 (3.6)	0.0004	0.0046
HINATA NFKB TARGETS FIBROBLAST UP	83	6 (7.2)	4 (4.8)	2 (2.4)	0.0004	0.0049
KAN RESPONSE TO ARSENIC TRIOXIDE	114	7 (6.1)	6 (5.3)	1 (0.9)	0.0005	0.0054
PEDERSEN METASTASIS BY ERBB2 ISOFORM 7	401	14 (3.5)	11 (2.7)	3 (0.7)	0.0005	0.0055
LEE LIVER CANCER MYC E2F1 UP	56	5 (8.9)	4 (7.1)	1 (1.8)	0.0005	0.0055
PATTERSON DOCETAXEL RESISTANCE	28	4 (14.3)	4 (14.3)	0 (0)	0.0005	0.0056
BRUINS UVC RESPONSE VIA TP53 GROUP A	872	23 (2.6)	14 (1.6)	9 (1)	0.0005	0.0059
NOJIMA SFRP2 TARGETS UP	31	4 (12.9)	4 (12.9)	0 (0)	0.0005	0.0059
HSIAO LIVER SPECIFIC GENES	234	10 (4.3)	8 (3.4)	2 (0.9)	0.0005	0.006
LI WILMS TUMOR VS FETAL KIDNEY 1 UP	176	9 (5.1)	7 (4)	2 (1.1)	0.0005	0.006
LEE LIVER CANCER ACOX1 UP	63	5 (7.9)	4 (6.3)	1 (1.6)	0.0006	0.0061
LEE EARLY T LYMPHOCYTE DN	55	5 (9.1)	4 (7.3)	1 (1.8)	0.0006	0.0066
PETROVA ENDOTHELIUM LYMPHATIC VS BLOOD DN	157	8 (5.1)	8 (5.1)	0 (0)	0.0006	0.0067
GROSS ELK3 TARGETS DN	32	4 (12.5)	4 (12.5)	0 (0)	0.0006	0.0067
XU RESPONSE TO TRETINOIN UP	16	3 (18.8)	2 (12.5)	1 (6.2)	0.0006	0.007
MARKEY RB1 CHRONIC LOF UP	112	7 (6.2)	3 (2.7)	4 (3.6)	0.0007	0.0071

Category Name	Category Genes #	Any-reg. SDEG #(%)	Up-reg. SDEG #(%)	Down-reg. SDEG #(%)	P	FDR
VECCHI GASTRIC CANCER ADVANCED VS EARLY UP	168	9 (5.4)	7 (4.2)	2 (1.2)	0.0007	0.0074
DUTTA APOPTOSIS VIA NFKB	32	4 (12.5)	4 (12.5)	0 (0)	0.0007	0.0075
MARTENS BOUND BY PML RARA FUSION	446	15 (3.4)	9 (2)	6 (1.3)	0.0007	0.0077
MULLIGHAN MLL SIGNATURE 1 DN	231	10 (4.3)	5 (2.2)	5 (2.2)	0.0007	0.0077
CADWELL ATG16L1 TARGETS UP	94	6 (6.4)	5 (5.3)	1 (1.1)	0.0007	0.0079
CONCANNON APOPTOSIS BY EPOXOMICIN DN	172	8 (4.7)	4 (2.3)	4 (2.3)	0.0007	0.0079
YOSHIMURA MAPK8 TARGETS UP	1281	29 (2.3)	21 (1.6)	8 (0.6)	0.0008	0.008
SHEDDEN LUNG CANCER GOOD SURVIVAL A12	280	11 (3.9)	10 (3.6)	1 (0.4)	0.0008	0.008
LEE SP4 THYMOCYTE	14	3 (21.4)	3 (21.4)	0 (0)	0.0008	0.008
NAKAJIMA MAST CELL	43	4 (9.3)	3 (7)	1 (2.3)	0.0008	0.0086
GOBERT OLIGODENDROCYTE DIFFERENTIATION DN	1051	27 (2.6)	16 (1.5)	11 (1)	0.0008	0.0087
VALK AML CLUSTER 9	34	4 (11.8)	1 (2.9)	3 (8.8)	0.0008	0.0087
IZADPANAH STEM CELL ADIPOSE VS BONE UP	125	7 (5.6)	6 (4.8)	1 (0.8)	0.0008	0.0088
BOYLAN MULTIPLE MYELOMA PCA1 UP	96	6 (6.2)	5 (5.2)	1 (1)	0.0008	0.0088
DOUGLAS BMI1 TARGETS DN	308	12 (3.9)	12 (3.9)	0 (0)	0.0009	0.0089
BOYLAN MULTIPLE MYELOMA C D DN	244	10 (4.1)	8 (3.3)	2 (0.8)	0.0009	0.0089
PLASARI TGFB1 SIGNALING VIA NFIC 10HR UP	54	5 (9.3)	5 (9.3)	0 (0)	0.0009	0.0089
KOINUMA COLON CANCER MSI DN	16	3 (18.8)	2 (12.5)	1 (6.2)	0.0009	0.0089
CHARAFE BREAST CANCER LUMINAL VS MESENCHYMAL DN	447	15 (3.4)	10 (2.2)	5 (1.1)	0.0009	0.009
RUIZ TNC TARGETS UP	149	8 (5.4)	7 (4.7)	1 (0.7)	0.0009	0.009
ZHOU INFLAMMATORY RESPONSE FIMA UP	504	16 (3.2)	11 (2.2)	5 (1)	0.0009	0.0092
SENESE HDAC3 TARGETS UP	477	16 (3.4)	14 (2.9)	2 (0.4)	0.0009	0.0093
MARTINEZ RB1 AND TP53 TARGETS UP	582	17 (2.9)	12 (2.1)	5 (0.9)	0.0009	0.0094
BROWNE HCMV INFECTION 48HR DN	477	15 (3.1)	9 (1.9)	6 (1.3)	0.001	0.0097
MIKKELSEN IPS LCP WITH H3K4ME3	170	8 (4.7)	7 (4.1)	1 (0.6)	0.001	0.0098
PROVENZANI METASTASIS DN	133	7 (5.3)	6 (4.5)	1 (0.8)	0.001	0.0098
GAJATE RESPONSE TO TRABECTEDIN UP	68	5 (7.4)	5 (7.4)	0 (0)	0.001	0.0098
KAAB FAILED HEART VENTRICLE DN	41	4 (9.8)	4 (9.8)	0 (0)	0.001	0.0098

Table A87: Significantly enriched MSigDB Chemical/Genetic Perturbation Sets (RNA-Seq, Monocytes, Day 1). Results sorted by FDR and #SDEG genes.

Category Name	Category Genes #	Any-reg. SDEG #(%)	Up-reg. SDEG #(%)	Down-reg. SDEG #(%)	P	FDR
Influenza A	149	23 (15.4)	22 (14.8)	1 (0.7)	<0.0001	0.0001
Herpes simplex infection	154	23 (14.9)	22 (14.3)	1 (0.6)	<0.0001	0.0001
Tuberculosis	159	16 (10.1)	16 (10.1)	0 (0)	<0.0001	0.0001
Antigen processing and presentation	64	15 (23.4)	15 (23.4)	0 (0)	<0.0001	0.0001
Systemic lupus erythematosus	106	13 (12.3)	12 (11.3)	1 (0.9)	<0.0001	0.0001

Category Name	Category Genes #	Any-reg. SDEG #(%)	Up-reg. SDEG #(%)	Down-reg. SDEG #(%)	P	FDR
Rheumatoid arthritis	77	13 (16.9)	9 (11.7)	4 (5.2)	<0.0001	0.0001
Measles	115	12 (10.4)	11 (9.6)	1 (0.9)	<0.0001	0.0001
Inflammatory bowel disease (IBD)	53	12 (22.6)	12 (22.6)	0 (0)	<0.0001	0.0001
Toxoplasmosis	101	11 (10.9)	10 (9.9)	1 (1)	<0.0001	0.0001
Staphylococcus aureus infection	47	11 (23.4)	11 (23.4)	0 (0)	<0.0001	0.0001
Hepatitis C	117	11 (9.4)	10 (8.5)	1 (0.9)	<0.0001	0.0001
Hematopoietic cell lineage	74	10 (13.5)	8 (10.8)	2 (2.7)	<0.0001	0.0001
Leishmaniasis	60	10 (16.7)	9 (15)	1 (1.7)	<0.0001	0.0001
Intestinal immune network for IgA production	37	9 (24.3)	9 (24.3)	0 (0)	<0.0001	0.0001
Viral myocarditis	51	9 (17.6)	9 (17.6)	0 (0)	<0.0001	0.0001
Type I diabetes mellitus	37	7 (18.9)	7 (18.9)	0 (0)	<0.0001	0.0001
Asthma	23	7 (30.4)	7 (30.4)	0 (0)	<0.0001	0.0001
Autoimmune thyroid disease	45	7 (15.6)	7 (15.6)	0 (0)	<0.0001	0.0001
Allograft rejection	31	7 (22.6)	7 (22.6)	0 (0)	<0.0001	0.0001
Graft-versus-host disease	33	7 (21.2)	7 (21.2)	0 (0)	<0.0001	0.0001
HTLV-I infection	229	15 (6.6)	13 (5.7)	2 (0.9)	<0.0001	0.0004
Phagosome	131	11 (8.4)	11 (8.4)	0 (0)	<0.0001	0.0004
RIG-I-like receptor signaling pathway	62	7 (11.3)	5 (8.1)	2 (3.2)	<0.0001	0.0005
Cell adhesion molecules (CAMs)	123	11 (8.9)	11 (8.9)	0 (0)	<0.0001	0.0006
Pertussis	70	8 (11.4)	6 (8.6)	2 (2.9)	<0.0001	0.0006
NOD-like receptor signaling pathway	52	6 (11.5)	4 (7.7)	2 (3.8)	0.0003	0.0032
Proteasome	41	5 (12.2)	5 (12.2)	0 (0)	0.0003	0.0037

Table A88: Significantly enriched KEGG Pathways (RNA-Seq, Neutrophils, Day 1). Results sorted by FDR and #SDEG genes.

Category Name	Category Genes #	Any-reg. SDEG #(%)	Up-reg. SDEG #(%)	Down-reg. SDEG #(%)	P	FDR
Immunoproteasome	16	5 (31.2)	5 (31.2)	0 (0)	<0.0001	0.0016

Table A89: Significantly enriched KEGG Modules (RNA-Seq, Neutrophils, Day 1). Results sorted by FDR and #SDEG genes.

Category Name	Category Genes #	Any-reg. SDEG #(%)	Up-reg. SDEG #(%)	Down-reg. SDEG #(%)	P	FDR
SIGNAL TRANSDUCTION	1580	62 (3.9)	50 (3.2)	12 (0.8)	<0.0001	0.0009
INTRACELLULAR SIGNALING CASCADE	647	33 (5.1)	28 (4.3)	5 (0.8)	<0.0001	0.0009
IMMUNE SYSTEM PROCESS	324	23 (7.1)	22 (6.8)	1 (0.3)	<0.0001	0.0009
IMMUNE RESPONSE	231	20 (8.7)	20 (8.7)	0 (0)	<0.0001	0.0009

Category Name	Category Genes #	Any-reg. SDEG #(%)	Up-reg. SDEG #(%)	Down-reg. SDEG #(%)	P	FDR
PROTEIN KINASE CASCADE	285	20 (7)	17 (6)	3 (1.1)	<0.0001	0.0009
DEFENSE RESPONSE	262	18 (6.9)	14 (5.3)	4 (1.5)	<0.0001	0.0009
RESPONSE TO WOUNDING	185	13 (7)	9 (4.9)	4 (2.2)	<0.0001	0.0009
RESPONSE TO OTHER ORGANISM	79	9 (11.4)	9 (11.4)	0 (0)	<0.0001	0.0009
RESPONSE TO VIRUS	47	7 (14.9)	7 (14.9)	0 (0)	<0.0001	0.0009
PROTEIN METABOLIC PROCESS	1185	43 (3.6)	34 (2.9)	9 (0.8)	<0.0001	0.0012
CELL PROLIFERATION GO 0008283	493	24 (4.9)	20 (4.1)	4 (0.8)	<0.0001	0.0012
I KAPPAB KINASE NF KAPPAB CASCADE	113	12 (10.6)	11 (9.7)	1 (0.9)	<0.0001	0.0012
MULTI ORGANISM PROCESS	157	12 (7.6)	11 (7)	1 (0.6)	<0.0001	0.0012
REGULATION OF I KAPPAB KINASE NF KAPPAB CASCADE	95	10 (10.5)	9 (9.5)	1 (1.1)	<0.0001	0.0012
POSITIVE REGULATION OF I KAPPAB KINASE NF KAPPAB CASCADE	89	9 (10.1)	8 (9)	1 (1.1)	<0.0001	0.0027
NUCLEOBASENUCLEOSIDENUCLEOTIDE AND NUCLEIC ACID METABOLIC PROCESS	1196	41 (3.4)	37 (3.1)	4 (0.3)	0.0002	0.0082
RESPONSE TO BIOTIC STIMULUS	117	9 (7.7)	9 (7.7)	0 (0)	0.0002	0.0087
POSITIVE REGULATION OF SIGNAL TRANSDUCTION	127	10 (7.9)	8 (6.3)	2 (1.6)	0.0002	0.0092
POSITIVE REGULATION OF BIOLOGICAL PROCESS	690	27 (3.9)	20 (2.9)	7 (1)	0.0002	0.0093
POSITIVE REGULATION OF CELLULAR PROCESS	652	26 (4)	20 (3.1)	6 (0.9)	0.0003	0.0093
RESPONSE TO EXTERNAL STIMULUS	304	15 (4.9)	11 (3.6)	4 (1.3)	0.0002	0.0093
MEMBRANE ORGANIZATION AND BIOGENESIS	135	10 (7.4)	8 (5.9)	2 (1.5)	0.0002	0.0093
INFLAMMATORY RESPONSE	129	9 (7)	7 (5.4)	2 (1.6)	0.0003	0.0093

Table A90: Significantly enriched MSigDB GO Biological Processes (RNA-Seq, Neutrophils, Day 1). Results sorted by FDR and #SDEG genes.

Category Name	Category Genes #	Any-reg. SDEG #(%)	Up-reg. SDEG #(%)	Down-reg. SDEG #(%)	P	FDR
CYTOPLASM	2054	86 (4.2)	75 (3.7)	11 (0.5)	<0.0001	0.0012
CYTOPLASMIC PART	1335	51 (3.8)	44 (3.3)	7 (0.5)	<0.0001	0.0012
LYSOSOME	57	7 (12.3)	6 (10.5)	1 (1.8)	<0.0001	0.0017
LYTIC VACUOLE	57	7 (12.3)	6 (10.5)	1 (1.8)	<0.0001	0.0017
VACUOLE	65	7 (10.8)	6 (9.2)	1 (1.5)	<0.0001	0.0047
CELL FRACTION	480	22 (4.6)	16 (3.3)	6 (1.2)	0.0001	0.005

Table A91: Significantly enriched MSigDB GO Cellular Components (RNA-Seq, Neutrophils, Day 1). Results sorted by FDR and #SDEG genes.

Category Name	Category Genes #	Any-reg. SDEG #(%)	Up-reg. SDEG #(%)	Down-reg. SDEG #(%)	P	FDR
ENZYME REGULATOR ACTIVITY	310	20 (6.5)	17 (5.5)	3 (1)	<0.0001	0.004

Table A92: Significantly enriched MSigDB GO Molecular Functions (RNA-Seq, Neutrophils, Day 1). Results sorted by FDR and #SDEG genes.

Category Name	Category Genes #	Any-reg. SDEG #(%)	Up-reg. SDEG #(%)	Down-reg. SDEG #(%)	P	FDR
NUYTTEN EZH2 TARGETS UP	1002	104 (10.4)	93 (9.3)	11 (1.1)	<0.0001	0.0002
NUYTTEN NIPP1 TARGETS UP	748	74 (9.9)	69 (9.2)	5 (0.7)	<0.0001	0.0002
CHEN METABOLIC SYNDROM NETWORK	1172	71 (6.1)	60 (5.1)	11 (0.9)	<0.0001	0.0002
GRAESSMANN APOPTOSIS BY DOXORUBICIN UP	1132	70 (6.2)	56 (4.9)	14 (1.2)	<0.0001	0.0002
FULCHER INFLAMMATORY RESPONSE LECTIN VS LPS DN	462	67 (14.5)	63 (13.6)	4 (0.9)	<0.0001	0.0002
BLALOCK ALZHEIMERS DISEASE UP	1628	66 (4.1)	54 (3.3)	12 (0.7)	<0.0001	0.0002
DODD NASOPHARYNGEAL CARCINOMA DN	1330	61 (4.6)	53 (4)	8 (0.6)	<0.0001	0.0002
ZWANG TRANSIENTLY UP BY 1ST EGF PULSE ONLY	1723	60 (3.5)	51 (3)	9 (0.5)	<0.0001	0.0002
TAKEDA TARGETS OF NUP98 HOXA9 FUSION 3D UP	178	51 (28.7)	50 (28.1)	1 (0.6)	<0.0001	0.0002
PUJANA ATM PCC NETWORK	1390	50 (3.6)	42 (3)	8 (0.6)	<0.0001	0.0002
GRAESSMANN APOPTOSIS BY SERUM DEPRIVATION UP	541	49 (9.1)	46 (8.5)	3 (0.6)	<0.0001	0.0002
WALLACE PROSTATE CANCER RACE UP	275	49 (17.8)	46 (16.7)	3 (1.1)	<0.0001	0.0002
KRIGE RESPONSE TO TOSEDOSTAT 6HR UP	915	47 (5.1)	40 (4.4)	7 (0.8)	<0.0001	0.0002
ONKEN UVEAL MELANOMA UP	763	47 (6.2)	39 (5.1)	8 (1)	<0.0001	0.0002
FEVR CTNNB1 TARGETS UP	672	46 (6.8)	42 (6.2)	4 (0.6)	<0.0001	0.0002
FOSTER TOLERANT MACROPHAGE DN	405	46 (11.4)	42 (10.4)	4 (1)	<0.0001	0.0002
BOSCO INTERFERON INDUCED ANTIVIRAL MODULE	76	45 (59.2)	45 (59.2)	0 (0)	<0.0001	0.0002
PEREZ TP53 TARGETS	1124	45 (4)	34 (3)	11 (1)	<0.0001	0.0002
RODWELL AGING KIDNEY UP	465	45 (9.7)	38 (8.2)	7 (1.5)	<0.0001	0.0002
HECKER IFNB1 TARGETS	92	44 (47.8)	43 (46.7)	1 (1.1)	<0.0001	0.0002
GOZGIT ESR1 TARGETS DN	741	43 (5.8)	38 (5.1)	5 (0.7)	<0.0001	0.0002
MEISSNER BRAIN HCP WITH H3K4ME3 AND H3K27ME3	1060	43 (4.1)	31 (2.9)	12 (1.1)	<0.0001	0.0002
ACEVEDO LIVER CANCER UP	947	42 (4.4)	33 (3.5)	9 (1)	<0.0001	0.0002
JISON SICKLE CELL DISEASE UP	174	42 (24.1)	40 (23)	2 (1.1)	<0.0001	0.0002
SANA RESPONSE TO IFNG UP	74	41 (55.4)	41 (55.4)	0 (0)	<0.0001	0.0002
BROWNE INTERFERON RESPONSIVE GENES	68	40 (58.8)	40 (58.8)	0 (0)	<0.0001	0.0002
KRIGE RESPONSE TO TOSEDOSTAT 24HR DN	993	40 (4)	28 (2.8)	12 (1.2)	<0.0001	0.0002
CHICAS RB1 TARGETS SENESCENT	552	39 (7.1)	32 (5.8)	7 (1.3)	<0.0001	0.0002
KRIGE RESPONSE TO TOSEDOSTAT 24HR UP	740	39 (5.3)	33 (4.5)	6 (0.8)	<0.0001	0.0002
TAKEDA TARGETS OF NUP98 HOXA9 FUSION 10D UP	186	39 (21)	37 (19.9)	2 (1.1)	<0.0001	0.0002
LOPEZ MBD TARGETS	925	38 (4.1)	30 (3.2)	8 (0.9)	<0.0001	0.0002
DEBIASI APOPTOSIS BY REOVIRUS INFECTION UP	317	37 (11.7)	36 (11.4)	1 (0.3)	<0.0001	0.0002
FORTSCHEGGER PHF8 TARGETS DN	749	36 (4.8)	33 (4.4)	3 (0.4)	<0.0001	0.0002
BERTUCCI MEDULLARY VS DUCTAL BREAST CANCER UP	200	35 (17.5)	34 (17)	1 (0.5)	<0.0001	0.0002
GRAESSMANN RESPONSE TO MC AND SERUM DEPRIVATION UP	203	35 (17.2)	34 (16.7)	1 (0.5)	<0.0001	0.0002
HELLER SILENCED BY METHYLATION UP	273	35 (12.8)	33 (12.1)	2 (0.7)	<0.0001	0.0002
LEE BMP2 TARGETS UP	723	35 (4.8)	26 (3.6)	9 (1.2)	<0.0001	0.0002
ZHOU INFLAMMATORY RESPONSE LPS UP	401	35 (8.7)	32 (8)	3 (0.7)	<0.0001	0.0002

Category Name	Category Genes #	Any-reg. SDEG #(%)	Up-reg. SDEG #(%)	Down-reg. SDEG #(%)	P	FDR
JOHNSTONE PARVB TARGETS 3 UP	425	33 (7.8)	29 (6.8)	4 (0.9)	<0.0001	0.0002
KRIEG HYPOXIA NOT VIA KDM3A	718	33 (4.6)	21 (2.9)	12 (1.7)	<0.0001	0.0002
MARKEY RB1 ACUTE LOF UP	214	33 (15.4)	31 (14.5)	2 (0.9)	<0.0001	0.0002
RUTELLA RESPONSE TO HGF VS CSF2RB AND IL4 UP	391	33 (8.4)	26 (6.6)	7 (1.8)	<0.0001	0.0002
SMID BREAST CANCER NORMAL LIKE UP	443	33 (7.4)	25 (5.6)	8 (1.8)	<0.0001	0.0002
YANG BCL3 TARGETS UP	351	33 (9.4)	29 (8.3)	4 (1.1)	<0.0001	0.0002
ZHANG RESPONSE TO IKK INHIBITOR AND TNF UP	221	33 (14.9)	30 (13.6)	3 (1.4)	<0.0001	0.0002
BUYTAERT PHOTODYNAMIC THERAPY STRESS DN	618	32 (5.2)	23 (3.7)	9 (1.5)	<0.0001	0.0002
DER IFN BETA RESPONSE UP	101	32 (31.7)	32 (31.7)	0 (0)	<0.0001	0.0002
ONDER CDH1 TARGETS 2 DN	454	32 (7)	22 (4.8)	10 (2.2)	<0.0001	0.0002
RUTELLA RESPONSE TO HGF UP	398	32 (8)	28 (7)	4 (1)	<0.0001	0.0002
BAELDE DIABETIC NEPHROPATHY DN	425	31 (7.3)	28 (6.6)	3 (0.7)	<0.0001	0.0002
COLINA TARGETS OF 4EBP1 AND 4EBP2	339	31 (9.1)	30 (8.8)	1 (0.3)	<0.0001	0.0002
TAKEDA TARGETS OF NUP98 HOXA9 FUSION 8D UP	155	31 (20)	30 (19.4)	1 (0.6)	<0.0001	0.0002
CHICAS RB1 TARGETS CONFLUENT	550	30 (5.5)	24 (4.4)	6 (1.1)	<0.0001	0.0002
FULCHER INFLAMMATORY RESPONSE LECTIN VS LPS UP	564	30 (5.3)	18 (3.2)	12 (2.1)	<0.0001	0.0002
ICHIBA GRAFT VERSUS HOST DISEASE D7 UP	105	30 (28.6)	30 (28.6)	0 (0)	<0.0001	0.0002
ZHENG BOUND BY FOXP3	483	30 (6.2)	21 (4.3)	9 (1.9)	<0.0001	0.0002
DER IFN ALPHA RESPONSE UP	74	29 (39.2)	29 (39.2)	0 (0)	<0.0001	0.0002
HAN SATB1 TARGETS DN	421	29 (6.9)	24 (5.7)	5 (1.2)	<0.0001	0.0002
TAKEDA TARGETS OF NUP98 HOXA9 FUSION 16D UP	169	29 (17.2)	26 (15.4)	3 (1.8)	<0.0001	0.0002
BOQUEST STEM CELL CULTURED VS FRESH UP	409	28 (6.8)	23 (5.6)	5 (1.2)	<0.0001	0.0002
LI INDUCED T TO NATURAL KILLER UP	302	28 (9.3)	22 (7.3)	6 (2)	<0.0001	0.0002
MITSIADES RESPONSE TO APLIDIN UP	450	28 (6.2)	26 (5.8)	2 (0.4)	<0.0001	0.0002
SWEET LUNG CANCER KRAS UP	482	28 (5.8)	23 (4.8)	5 (1)	<0.0001	0.0002
WANG RESPONSE TO GSK3 INHIBITOR SB216763 UP	378	28 (7.4)	26 (6.9)	2 (0.5)	<0.0001	0.0002
DAUER STAT3 TARGETS DN	51	27 (52.9)	26 (51)	1 (2)	<0.0001	0.0002
DOUGLAS BMI1 TARGETS DN	308	27 (8.8)	26 (8.4)	1 (0.3)	<0.0001	0.0002
HOLLMANN APOPTOSIS VIA CD40 DN	259	27 (10.4)	23 (8.9)	4 (1.5)	<0.0001	0.0002
HORIUCHI WTAP TARGETS UP	295	27 (9.2)	23 (7.8)	4 (1.4)	<0.0001	0.0002
IVANOVA HEMATOPOIESIS LATE PROGENITOR	534	27 (5.1)	23 (4.3)	4 (0.7)	<0.0001	0.0002
MOSERLE IFNA RESPONSE	31	27 (87.1)	27 (87.1)	0 (0)	<0.0001	0.0002
SATO SILENCED BY METHYLATION IN PANCREATIC CANCER 1	412	27 (6.6)	24 (5.8)	3 (0.7)	<0.0001	0.0002
SCHUETZ BREAST CANCER DUCTAL INVASIVE UP	338	27 (8)	24 (7.1)	3 (0.9)	<0.0001	0.0002
WIELAND UP BY HBV INFECTION	100	27 (27)	27 (27)	0 (0)	<0.0001	0.0002
BOQUEST STEM CELL DN	213	26 (12.2)	19 (8.9)	7 (3.3)	<0.0001	0.0002
CHARAFE BREAST CANCER LUMINAL VS BASAL DN	440	26 (5.9)	24 (5.5)	2 (0.5)	<0.0001	0.0002
DER IFN GAMMA RESPONSE UP	72	26 (36.1)	26 (36.1)	0 (0)	<0.0001	0.0002

Category Name	Category Genes #	Any-reg. SDEG #(%)	Up-reg. SDEG #(%)	Down-reg. SDEG #(%)	P	FDR
GARY CD5 TARGETS UP	458	26 (5.7)	22 (4.8)	4 (0.9)	<0.0001	0.0002
LINDGREN BLADDER CANCER CLUSTER 2B	376	26 (6.9)	25 (6.6)	1 (0.3)	<0.0001	0.0002
SMID BREAST CANCER LUMINAL B DN	513	26 (5.1)	22 (4.3)	4 (0.8)	<0.0001	0.0002
ALTEMEIER RESPONSE TO LPS WITH MECHANICAL VENTILATION	126	25 (19.8)	24 (19)	1 (0.8)	<0.0001	0.0002
GRUETZMANN PANCREATIC CANCER UP	351	25 (7.1)	23 (6.6)	2 (0.6)	<0.0001	0.0002
KUMAR TARGETS OF MLL AF9 FUSION	389	25 (6.4)	20 (5.1)	5 (1.3)	<0.0001	0.0002
ODONNELL TFRC TARGETS UP	409	25 (6.1)	17 (4.2)	8 (2)	<0.0001	0.0002
DELYS THYROID CANCER UP	435	24 (5.5)	16 (3.7)	8 (1.8)	<0.0001	0.0002
LIM MAMMARY STEM CELL DN	422	24 (5.7)	19 (4.5)	5 (1.2)	<0.0001	0.0002
PEDRIOLI MIR31 TARGETS DN	398	24 (6)	18 (4.5)	6 (1.5)	<0.0001	0.0002
RIGGI EWING SARCOMA PROGENITOR UP	429	24 (5.6)	17 (4)	7 (1.6)	<0.0001	0.0002
RUTELLA RESPONSE TO CSF2RB AND IL4 DN	303	24 (7.9)	19 (6.3)	5 (1.7)	<0.0001	0.0002
HIRSCH CELLULAR TRANSFORMATION SIGNATURE UP	239	23 (9.6)	21 (8.8)	2 (0.8)	<0.0001	0.0002
SENGUPTA NASOPHARYNGEAL CARCINOMA WITH LMP1 UP	387	23 (5.9)	20 (5.2)	3 (0.8)	<0.0001	0.0002
WANG SMARCE1 TARGETS DN	340	23 (6.8)	17 (5)	6 (1.8)	<0.0001	0.0002
DACOSTA UV RESPONSE VIA ERCC3 UP	301	22 (7.3)	17 (5.6)	5 (1.7)	<0.0001	0.0002
GOLDRATH ANTIGEN RESPONSE	333	22 (6.6)	19 (5.7)	3 (0.9)	<0.0001	0.0002
RADAEVA RESPONSE TO IFNA1 UP	52	22 (42.3)	22 (42.3)	0 (0)	<0.0001	0.0002
RIGGINS TAMOXIFEN RESISTANCE DN	215	22 (10.2)	19 (8.8)	3 (1.4)	<0.0001	0.0002
SANSOM APC TARGETS DN	359	22 (6.1)	21 (5.8)	1 (0.3)	<0.0001	0.0002
ACEVEDO FGFR1 TARGETS IN PROSTATE CANCER MODEL UP	278	21 (7.6)	14 (5)	7 (2.5)	<0.0001	0.0002
BOYLAN MULTIPLE MYELOMA C D DN	244	21 (8.6)	17 (7)	4 (1.6)	<0.0001	0.0002
QI PLASMACYTOMA UP	251	21 (8.4)	20 (8)	1 (0.4)	<0.0001	0.0002
SANA TNF SIGNALING UP	81	21 (25.9)	19 (23.5)	2 (2.5)	<0.0001	0.0002
SEITZ NEOPLASTIC TRANSFORMATION BY 8P DELETION UP	69	21 (30.4)	21 (30.4)	0 (0)	<0.0001	0.0002
VERHAAK AML WITH NPM1 MUTATED DN	238	21 (8.8)	15 (6.3)	6 (2.5)	<0.0001	0.0002
RICKMAN METASTASIS DN	258	20 (7.8)	18 (7)	2 (0.8)	<0.0001	0.0002
SENGUPTA NASOPHARYNGEAL CARCINOMA UP	287	20 (7)	19 (6.6)	1 (0.3)	<0.0001	0.0002
STAMBOLSKY TARGETS OF MUTATED TP53 DN	51	20 (39.2)	19 (37.3)	1 (2)	<0.0001	0.0002
WIERENGA STAT5A TARGETS UP	210	20 (9.5)	13 (6.2)	7 (3.3)	<0.0001	0.0002
ZHU CMV ALL UP	121	20 (16.5)	20 (16.5)	0 (0)	<0.0001	0.0002
EINAV INTERFERON SIGNATURE IN CANCER	27	19 (70.4)	19 (70.4)	0 (0)	<0.0001	0.0002
MCLACHLAN DENTAL CARIES UP	227	19 (8.4)	17 (7.5)	2 (0.9)	<0.0001	0.0002
ROZANOV MMP14 TARGETS UP	270	19 (7)	15 (5.6)	4 (1.5)	<0.0001	0.0002
BENNETT SYSTEMIC LUPUS ERYTHEMATOSUS	32	18 (56.2)	18 (56.2)	0 (0)	<0.0001	0.0002
BOSCO ALLERGEN INDUCED TH2 ASSOCIATED MODULE	160	18 (11.2)	14 (8.8)	4 (2.5)	<0.0001	0.0002
HOSHIDA LIVER CANCER SUBCLASS S1	225	18 (8)	16 (7.1)	2 (0.9)	<0.0001	0.0002
ICHIBA GRAFT VERSUS HOST DISEASE 35D UP	126	18 (14.3)	18 (14.3)	0 (0)	<0.0001	0.0002

Category Name	Category Genes #	Any-reg. SDEG #(%)	Up-reg. SDEG #(%)	Down-reg. SDEG #(%)	P	FDR
LEI MYB TARGETS	307	18 (5.9)	14 (4.6)	4 (1.3)	<0.0001	0.0002
LU EZH2 TARGETS UP	289	18 (6.2)	16 (5.5)	2 (0.7)	<0.0001	0.0002
MCLACHLAN DENTAL CARIES DN	219	18 (8.2)	16 (7.3)	2 (0.9)	<0.0001	0.0002
TONKS TARGETS OF RUNX1 RUNX1T1 FUSION MONOCYTE UP	201	18 (9)	14 (7)	4 (2)	<0.0001	0.0002
UROSEVIC RESPONSE TO IMIQUIMOD	23	18 (78.3)	18 (78.3)	0 (0)	<0.0001	0.0002
DOANE RESPONSE TO ANDROGEN DN	232	17 (7.3)	11 (4.7)	6 (2.6)	<0.0001	0.0002
WUNDER INFLAMMATORY RESPONSE AND CHOLESTEROL UP	61	17 (27.9)	16 (26.2)	1 (1.6)	<0.0001	0.0002
ZHANG INTERFERON RESPONSE	23	17 (73.9)	17 (73.9)	0 (0)	<0.0001	0.0002
ALCALAY AML BY NPM1 LOCALIZATION DN	178	16 (9)	14 (7.9)	2 (1.1)	<0.0001	0.0002
BOWIE RESPONSE TO EXTRACELLULAR MATRIX	17	16 (94.1)	16 (94.1)	0 (0)	<0.0001	0.0002
FOSTER KDM1A TARGETS UP	250	16 (6.4)	14 (5.6)	2 (0.8)	<0.0001	0.0002
FOSTER TOLERANT MACROPHAGE UP	155	16 (10.3)	15 (9.7)	1 (0.6)	<0.0001	0.0002
JOHNSTONE PARVB TARGETS 2 UP	136	16 (11.8)	16 (11.8)	0 (0)	<0.0001	0.0002
KRIEG KDM3A TARGETS NOT HYPOXIA	189	16 (8.5)	15 (7.9)	1 (0.5)	<0.0001	0.0002
MISSIAGLIA REGULATED BY METHYLATION UP	120	16 (13.3)	14 (11.7)	2 (1.7)	<0.0001	0.0002
TONKS TARGETS OF RUNX1 RUNX1T1 FUSION HSC UP	180	16 (8.9)	12 (6.7)	4 (2.2)	<0.0001	0.0002
BOSCO TH1 CYTOTOXIC MODULE	114	15 (13.2)	15 (13.2)	0 (0)	<0.0001	0.0002
BOWIE RESPONSE TO TAMOXIFEN	18	15 (83.3)	15 (83.3)	0 (0)	<0.0001	0.0002
BROCKE APOPTOSIS REVERSED BY IL6	144	15 (10.4)	13 (9)	2 (1.4)	<0.0001	0.0002
FARMER BREAST CANCER CLUSTER 1	39	15 (38.5)	15 (38.5)	0 (0)	<0.0001	0.0002
FLECHNER BIOPSY KIDNEY TRANSPLANT REJECTED VS OK UP	85	15 (17.6)	13 (15.3)	2 (2.4)	<0.0001	0.0002
KRASNOSELSKAYA ILF3 TARGETS UP	37	15 (40.5)	15 (40.5)	0 (0)	<0.0001	0.0002
LIANG SILENCED BY METHYLATION 2	55	15 (27.3)	13 (23.6)	2 (3.6)	<0.0001	0.0002
SEKI INFLAMMATORY RESPONSE LPS UP	77	15 (19.5)	14 (18.2)	1 (1.3)	<0.0001	0.0002
ZHANG TLX TARGETS 36HR UP	219	15 (6.8)	11 (5)	4 (1.8)	<0.0001	0.0002
ZHU CMV 8 HR UP	48	15 (31.2)	15 (31.2)	0 (0)	<0.0001	0.0002
VERHAAK AML WITH NPM1 MUTATED UP	190	14 (7.4)	11 (5.8)	3 (1.6)	<0.0001	0.0002
ZHU CMV 24 HR UP	93	14 (15.1)	14 (15.1)	0 (0)	<0.0001	0.0002
BASSO CD40 SIGNALING UP	99	13 (13.1)	11 (11.1)	2 (2)	<0.0001	0.0002
FURUKAWA DUSP6 TARGETS PCI35 UP	73	13 (17.8)	12 (16.4)	1 (1.4)	<0.0001	0.0002
KIM GLIS2 TARGETS UP	84	13 (15.5)	13 (15.5)	0 (0)	<0.0001	0.0002
LENAOUR DENDRITIC CELL MATURATION UP	112	12 (10.7)	10 (8.9)	2 (1.8)	<0.0001	0.0002
LIU VAV3 PROSTATE CARCINOGENESIS UP	85	12 (14.1)	10 (11.8)	2 (2.4)	<0.0001	0.0002
MORI MATURE B LYMPHOCYTE UP	89	12 (13.5)	9 (10.1)	3 (3.4)	<0.0001	0.0002
ZHAN MULTIPLE MYELOMA LB DN	39	12 (30.8)	12 (30.8)	0 (0)	<0.0001	0.0002
BROWNE HCMV INFECTION 6HR UP	69	11 (15.9)	10 (14.5)	1 (1.4)	<0.0001	0.0002
BROWNE HCMV INFECTION 8HR UP	102	11 (10.8)	9 (8.8)	2 (2)	<0.0001	0.0002
GERY CEBP TARGETS	137	11 (8)	9 (6.6)	2 (1.5)	<0.0001	0.0002

Category Name	Category Genes #	Any-reg. SDEG #(%)	Up-reg. SDEG #(%)	Down-reg. SDEG #(%)	P	FDR
GRANDVAUX IFN RESPONSE NOT VIA IRF3	14	11 (78.6)	11 (78.6)	0 (0)	<0.0001	0.0002
KIM LRRC3B TARGETS	30	11 (36.7)	11 (36.7)	0 (0)	<0.0001	0.0002
NEMETH INFLAMMATORY RESPONSE LPS UP	85	11 (12.9)	11 (12.9)	0 (0)	<0.0001	0.0002
ODONNELL TARGETS OF MYC AND TFRC UP	78	11 (14.1)	8 (10.3)	3 (3.8)	<0.0001	0.0002
SAGIV CD24 TARGETS DN	46	11 (23.9)	7 (15.2)	4 (8.7)	<0.0001	0.0002
BECKER TAMOXIFEN RESISTANCE UP	49	10 (20.4)	10 (20.4)	0 (0)	<0.0001	0.0002
BEIER GLIOMA STEM CELL DN	63	10 (15.9)	10 (15.9)	0 (0)	<0.0001	0.0002
CHANG IMMORTALIZED BY HPV31 DN	63	10 (15.9)	10 (15.9)	0 (0)	<0.0001	0.0002
GAURNIER PSMD4 TARGETS	68	10 (14.7)	9 (13.2)	1 (1.5)	<0.0001	0.0002
XU AKT1 TARGETS 6HR	27	10 (37)	10 (37)	0 (0)	<0.0001	0.0002
CASTELLANO NRAS TARGETS UP	68	9 (13.2)	9 (13.2)	0 (0)	<0.0001	0.0002
DEMAGALHAES AGING UP	56	9 (16.1)	6 (10.7)	3 (5.4)	<0.0001	0.0002
GEISS RESPONSE TO DSRNA UP	35	9 (25.7)	9 (25.7)	0 (0)	<0.0001	0.0002
HOFFMANN SMALL PRE BII TO IMMATURE B LYMPHOCYTE UP	70	9 (12.9)	8 (11.4)	1 (1.4)	<0.0001	0.0002
JECHLINGER EPITHELIAL TO MESENCHYMAL TRANSITION UP	71	9 (12.7)	8 (11.3)	1 (1.4)	<0.0001	0.0002
ROETH TERT TARGETS UP	14	9 (64.3)	9 (64.3)	0 (0)	<0.0001	0.0002
TAVOR CEBPA TARGETS UP	47	9 (19.1)	6 (12.8)	3 (6.4)	<0.0001	0.0002
ZHANG ANTIVIRAL RESPONSE TO RIBAVIRIN UP	29	9 (31)	9 (31)	0 (0)	<0.0001	0.0002
DACOSTA UV RESPONSE VIA ERCC3 TTD UP	60	8 (13.3)	7 (11.7)	1 (1.7)	<0.0001	0.0002
FAELT B CLL WITH VH REARRANGEMENTS UP	46	8 (17.4)	6 (13)	2 (4.3)	<0.0001	0.0002
GRANDVAUX IRF3 TARGETS UP	15	8 (53.3)	8 (53.3)	0 (0)	<0.0001	0.0002
LEE LIVER CANCER MYC E2F1 UP	56	8 (14.3)	7 (12.5)	1 (1.8)	<0.0001	0.0002
MUELLER COMMON TARGETS OF AML FUSIONS DN	42	8 (19)	7 (16.7)	1 (2.4)	<0.0001	0.0002
PELLICCIOTTA HDAC IN ANTIGEN PRESENTATION DN	51	8 (15.7)	8 (15.7)	0 (0)	<0.0001	0.0002
XU HGF TARGETS INDUCED BY AKT1 6HR	18	8 (44.4)	8 (44.4)	0 (0)	<0.0001	0.0002
YU MYC TARGETS DN	51	8 (15.7)	8 (15.7)	0 (0)	<0.0001	0.0002
FUJII YBX1 TARGETS UP	40	7 (17.5)	6 (15)	1 (2.5)	<0.0001	0.0002
HAN JNK SINGALING UP	34	7 (20.6)	6 (17.6)	1 (2.9)	<0.0001	0.0002
KAAB FAILED HEART VENTRICLE DN	41	7 (17.1)	6 (14.6)	1 (2.4)	<0.0001	0.0002
MAHADEVAN RESPONSE TO MP470 UP	19	7 (36.8)	5 (26.3)	2 (10.5)	<0.0001	0.0002
TSAI DNAJB4 TARGETS UP	13	7 (53.8)	7 (53.8)	0 (0)	<0.0001	0.0002
TSAI RESPONSE TO RADIATION THERAPY	32	7 (21.9)	7 (21.9)	0 (0)	<0.0001	0.0002
YAN ESCAPE FROM ANOIKIS	24	7 (29.2)	7 (29.2)	0 (0)	<0.0001	0.0002
HU GENOTOXIC DAMAGE 24HR	35	6 (17.1)	4 (11.4)	2 (5.7)	<0.0001	0.0002
IIZUKA LIVER CANCER PROGRESSION G1 G2 UP	12	5 (41.7)	5 (41.7)	0 (0)	<0.0001	0.0002
WATANABE ULCERATIVE COLITIS WITH CANCER DN	13	5 (38.5)	5 (38.5)	0 (0)	<0.0001	0.0002
MULLIGHAN MLL SIGNATURE 1 UP	375	20 (5.3)	14 (3.7)	6 (1.6)	<0.0001	0.0003
MIKKELSEN MCV6 LCP WITH H3K4ME3	153	13 (8.5)	12 (7.8)	1 (0.7)	<0.0001	0.0003

Category Name	Category Genes #	Any-reg. SDEG #(%)	Up-reg. SDEG #(%)	Down-reg. SDEG #(%)	P	FDR
AMIT SERUM RESPONSE 120 MCF10A	64	9 (14.1)	9 (14.1)	0 (0)	<0.0001	0.0003
MAHAJAN RESPONSE TO IL1A UP	79	9 (11.4)	8 (10.1)	1 (1.3)	<0.0001	0.0003
BROWNE HCMV INFECTION 4HR UP	53	8 (15.1)	8 (15.1)	0 (0)	<0.0001	0.0003
HOFFMANN IMMATURE TO MATURE B LYMPHOCYTE UP	44	8 (18.2)	7 (15.9)	1 (2.3)	<0.0001	0.0003
LINDSTEDT DENDRITIC CELL MATURATION B	51	8 (15.7)	8 (15.7)	0 (0)	<0.0001	0.0003
BAUS TFF2 TARGETS UP	32	6 (18.8)	6 (18.8)	0 (0)	<0.0001	0.0003
KRIGE RESPONSE TO TOSEDOSTAT 6HR DN	886	35 (4)	24 (2.7)	11 (1.2)	<0.0001	0.0005
SMID BREAST CANCER BASAL UP	607	28 (4.6)	23 (3.8)	5 (0.8)	<0.0001	0.0005
POOLA INVASIVE BREAST CANCER UP	272	18 (6.6)	17 (6.2)	1 (0.4)	<0.0001	0.0005
WANG ESOPHAGUS CANCER VS NORMAL UP	118	12 (10.2)	12 (10.2)	0 (0)	<0.0001	0.0005
HINATA NFKB TARGETS KERATINOCYTE UP	91	9 (9.9)	6 (6.6)	3 (3.3)	<0.0001	0.0005
GUTIERREZ CHRONIC LYMPHOCYTIC LEUKEMIA DN	56	8 (14.3)	6 (10.7)	2 (3.6)	<0.0001	0.0005
MORI IMMATURE B LYMPHOCYTE UP	52	7 (13.5)	7 (13.5)	0 (0)	<0.0001	0.0005
DIRMEIER LMP1 RESPONSE LATE DN	30	5 (16.7)	5 (16.7)	0 (0)	<0.0001	0.0005
NIELSEN SYNOVIAL SARCOMA DN	19	5 (26.3)	5 (26.3)	0 (0)	<0.0001	0.0005
PILON KLF1 TARGETS DN	1922	63 (3.3)	51 (2.7)	12 (0.6)	<0.0001	0.0006
PATIL LIVER CANCER	729	31 (4.3)	22 (3)	9 (1.2)	<0.0001	0.0006
DUTERTRE ESTRADIOL RESPONSE 24HR DN	502	25 (5)	24 (4.8)	1 (0.2)	<0.0001	0.0006
BLUM RESPONSE TO SALIRASIB UP	240	15 (6.2)	14 (5.8)	1 (0.4)	<0.0001	0.0006
WANG CISPLATIN RESPONSE AND XPC UP	189	13 (6.9)	9 (4.8)	4 (2.1)	<0.0001	0.0006
BROWNE HCMV INFECTION 12HR UP	105	10 (9.5)	9 (8.6)	1 (1)	<0.0001	0.0006
OUELLET OVARIAN CANCER INVASIVE VS LMP UP	115	10 (8.7)	8 (7)	2 (1.7)	<0.0001	0.0006
BOYLAN MULTIPLE MYELOMA PCA1 UP	96	9 (9.4)	8 (8.3)	1 (1)	<0.0001	0.0006
GAVIN FOXP3 TARGETS CLUSTER T4	93	9 (9.7)	9 (9.7)	0 (0)	<0.0001	0.0006
LINDSTEDT DENDRITIC CELL MATURATION A	67	8 (11.9)	7 (10.4)	1 (1.5)	<0.0001	0.0006
CROONQUIST IL6 DEPRIVATION UP	20	5 (25)	4 (20)	1 (5)	<0.0001	0.0006
BOCHKIS FOXA2 TARGETS	425	20 (4.7)	16 (3.8)	4 (0.9)	<0.0001	0.0008
PLASARI TGFB1 TARGETS 10HR DN	233	16 (6.9)	14 (6)	2 (0.9)	<0.0001	0.0008
TAKEDA TARGETS OF NUP98 HOXA9 FUSION 10D DN	135	11 (8.1)	7 (5.2)	4 (3)	<0.0001	0.0008
LUI THYROID CANCER CLUSTER 4	13	4 (30.8)	4 (30.8)	0 (0)	<0.0001	0.0008
BASAKI YBX1 TARGETS DN	361	20 (5.5)	13 (3.6)	7 (1.9)	<0.0001	0.0009
TORCHIA TARGETS OF EWSR1 FLI1 FUSION DN	306	18 (5.9)	16 (5.2)	2 (0.7)	<0.0001	0.0009
CHANG CORE SERUM RESPONSE DN	205	14 (6.8)	10 (4.9)	4 (2)	<0.0001	0.0009
PETROVA ENDOTHELIUM LYMPHATIC VS BLOOD DN	157	12 (7.6)	9 (5.7)	3 (1.9)	<0.0001	0.0009
TONKS TARGETS OF RUNX1 RUNX1T1 FUSION ERYTHROCYTE UP	151	12 (7.9)	10 (6.6)	2 (1.3)	<0.0001	0.0009
DARWICHE SKIN TUMOR PROMOTER UP	139	10 (7.2)	8 (5.8)	2 (1.4)	<0.0001	0.0009
BROWNE HCMV INFECTION 2HR UP	38	6 (15.8)	5 (13.2)	1 (2.6)	<0.0001	0.0009
SATO SILENCED BY DEACETYLATION IN PANCREATIC CANCER	43	6 (14)	2 (4.7)	4 (9.3)	<0.0001	0.0009

Category Name	Category Genes #	Any-reg. SDEG #(%)	Up-reg. SDEG #(%)	Down-reg. SDEG #(%)	P	FDR
EHLERS ANEUPLOIDY DN	12	4 (33.3)	4 (33.3)	0 (0)	<0.0001	0.0009
BRUINS UVC RESPONSE LATE	1111	39 (3.5)	26 (2.3)	13 (1.2)	<0.0001	0.001
WAKABAYASHI ADIPOGENESIS PPARG RXRA BOUND 8D	899	34 (3.8)	20 (2.2)	14 (1.6)	<0.0001	0.001
MULLIGHAN MLL SIGNATURE 2 UP	413	20 (4.8)	16 (3.9)	4 (1)	<0.0001	0.001
ENK UV RESPONSE EPIDERMIS UP	298	16 (5.4)	12 (4)	4 (1.3)	<0.0001	0.001
SASAKI ADULT T CELL LEUKEMIA	186	12 (6.5)	9 (4.8)	3 (1.6)	<0.0001	0.001
SARRIO EPITHELIAL MESENCHYMAL TRANSITION DN	149	11 (7.4)	9 (6)	2 (1.3)	<0.0001	0.001
SCHAEFFER PROSTATE DEVELOPMENT 48HR UP	465	22 (4.7)	15 (3.2)	7 (1.5)	<0.0001	0.0011
ENK UV RESPONSE KERATINOCYTE UP	529	21 (4)	19 (3.6)	2 (0.4)	<0.0001	0.0011
HSIAO HOUSEKEEPING GENES	374	17 (4.5)	16 (4.3)	1 (0.3)	<0.0001	0.0011
SMIRNOV RESPONSE TO IR 6HR UP	162	12 (7.4)	11 (6.8)	1 (0.6)	<0.0001	0.0011
AZARE NEOPLASTIC TRANSFORMATION BY STAT3 DN	115	10 (8.7)	8 (7)	2 (1.7)	<0.0001	0.0011
KOBAYASHI EGFR SIGNALING 24HR UP	110	9 (8.2)	7 (6.4)	2 (1.8)	<0.0001	0.0011
JOHANSSON GLIOMAGENESIS BY PDGFB UP	57	7 (12.3)	2 (3.5)	5 (8.8)	<0.0001	0.0011
MORI LARGE PRE BII LYMPHOCYTE DN	56	7 (12.5)	6 (10.7)	1 (1.8)	<0.0001	0.0011
KUWANO RNA STABILIZED BY NO	7	3 (42.9)	2 (28.6)	1 (14.3)	<0.0001	0.0011
GRAESSMANN RESPONSE TO MC AND DOXORUBICIN UP	615	26 (4.2)	17 (2.8)	9 (1.5)	<0.0001	0.0012
RUTELLA RESPONSE TO CSF2RB AND IL4 UP	319	18 (5.6)	16 (5)	2 (0.6)	<0.0001	0.0012
KATSANOU ELAVL1 TARGETS UP	163	11 (6.7)	10 (6.1)	1 (0.6)	<0.0001	0.0012
HESS TARGETS OF HOXA9 AND MEIS1 DN	76	8 (10.5)	8 (10.5)	0 (0)	<0.0001	0.0012
YAO TEMPORAL RESPONSE TO PROGESTERONE CLUSTER 9	72	7 (9.7)	7 (9.7)	0 (0)	<0.0001	0.0012
JAATINEN HEMATOPOIETIC STEM CELL UP	294	17 (5.8)	9 (3.1)	8 (2.7)	<0.0001	0.0013
DARWICHE SQUAMOUS CELL CARCINOMA UP	143	10 (7)	9 (6.3)	1 (0.7)	<0.0001	0.0013
MARTINEZ RESPONSE TO TRABECTEDIN UP	66	7 (10.6)	7 (10.6)	0 (0)	<0.0001	0.0013
NEWMAN ERCC6 TARGETS UP	26	5 (19.2)	3 (11.5)	2 (7.7)	<0.0001	0.0013
NOJIMA SFRP2 TARGETS DN	25	5 (20)	5 (20)	0 (0)	<0.0001	0.0013
CUI TCF21 TARGETS 2 DN	813	34 (4.2)	26 (3.2)	8 (1)	0.0001	0.0014
DEURIG T CELL PROLYMPHOCYTIC LEUKEMIA DN	302	17 (5.6)	16 (5.3)	1 (0.3)	0.0001	0.0014
CHYLA CBFA2T3 TARGETS DN	233	14 (6)	12 (5.2)	2 (0.9)	0.0001	0.0014
DAZARD RESPONSE TO UV NHEK UP	243	13 (5.3)	10 (4.1)	3 (1.2)	0.0001	0.0014
WIERENGA STAT5A TARGETS GROUP2	59	7 (11.9)	5 (8.5)	2 (3.4)	0.0001	0.0014
ZHAN MULTIPLE MYELOMA MF UP	44	6 (13.6)	3 (6.8)	3 (6.8)	0.0001	0.0014
WORSCHECH TUMOR EVASION AND TOLEROGENICITY DN	15	4 (26.7)	4 (26.7)	0 (0)	0.0001	0.0014
VANTVEER BREAST CANCER ESR1 DN	232	14 (6)	14 (6)	0 (0)	0.0001	0.0016
SWEET LUNG CANCER KRAS DN	425	20 (4.7)	13 (3.1)	7 (1.6)	0.0001	0.0017
LEE AGING NEOCORTEX UP	87	8 (9.2)	5 (5.7)	3 (3.4)	0.0001	0.0017
RODRIGUES THYROID CARCINOMA ANAPLASTIC UP	707	30 (4.2)	27 (3.8)	3 (0.4)	0.0001	0.0018
CHUNG BLISTER CYTOTOXICITY UP	130	10 (7.7)	8 (6.2)	2 (1.5)	0.0001	0.0018

Category Name	Category Genes #	Any-reg. SDEG #(%)	Up-reg. SDEG #(%)	Down-reg. SDEG #(%)	P	FDR
IZADPANAH STEM CELL ADIPOSE VS BONE UP	125	10 (8)	8 (6.4)	2 (1.6)	0.0001	0.0018
CREIGHTON ENDOCRINE THERAPY RESISTANCE 3	696	29 (4.2)	26 (3.7)	3 (0.4)	0.0001	0.0019
HUANG GATA2 TARGETS UP	146	10 (6.8)	8 (5.5)	2 (1.4)	0.0001	0.0019
HUANG DASATINIB RESISTANCE UP	79	8 (10.1)	8 (10.1)	0 (0)	0.0001	0.0019
LU TUMOR ANGIOGENESIS UP	25	5 (20)	3 (12)	2 (8)	0.0001	0.0019
ACEVEDO LIVER TUMOR VS NORMAL ADJACENT TISSUE UP	850	30 (3.5)	22 (2.6)	8 (0.9)	0.0002	0.002
CREIGHTON ENDOCRINE THERAPY RESISTANCE 2	453	21 (4.6)	15 (3.3)	6 (1.3)	0.0002	0.002
GRUETZMANN PANCREATIC CANCER DN	190	12 (6.3)	9 (4.7)	3 (1.6)	0.0002	0.002
NAKAYAMA SOFT TISSUE TUMORS PCA1 UP	69	7 (10.1)	7 (10.1)	0 (0)	0.0002	0.002
GOBERT OLIGODENDROCYTE DIFFERENTIATION DN	1051	39 (3.7)	29 (2.8)	10 (1)	0.0002	0.0021
CONCANNON APOPTOSIS BY EPOXOMICIN UP	227	14 (6.2)	12 (5.3)	2 (0.9)	0.0002	0.0021
BOYLAN MULTIPLE MYELOMA PCA3 UP	76	8 (10.5)	6 (7.9)	2 (2.6)	0.0002	0.0021
HINATA NFKB TARGETS FIBROBLAST UP	83	8 (9.6)	5 (6)	3 (3.6)	0.0002	0.0021
ZHAN MULTIPLE MYELOMA CD1 VS CD2 UP	64	7 (10.9)	6 (9.4)	1 (1.6)	0.0002	0.0021
YAO TEMPORAL RESPONSE TO PROGESTERONE CLUSTER 5	29	5 (17.2)	5 (17.2)	0 (0)	0.0002	0.0021
DIAZ CHRONIC MEYLOGENOUS LEUKEMIA DN	112	9 (8)	9 (8)	0 (0)	0.0002	0.0022
WONG ADULT TISSUE STEM MODULE	697	28 (4)	21 (3)	7 (1)	0.0002	0.0023
VERHAAK GLIOBLASTOMA NEURAL	205	13 (6.3)	13 (6.3)	0 (0)	0.0002	0.0023
MARTENS BOUND BY PML RARA FUSION	446	21 (4.7)	14 (3.1)	7 (1.6)	0.0002	0.0024
POTTI ETOPOSIDE SENSITIVITY	43	6 (14)	3 (7)	3 (7)	0.0002	0.0024
MACLACHLAN BRCA1 TARGETS DN	18	4 (22.2)	3 (16.7)	1 (5.6)	0.0002	0.0024
SPIELMAN LYMPHOBLAST EUROPEAN VS ASIAN UP	464	20 (4.3)	14 (3)	6 (1.3)	0.0002	0.0025
MAHADEVAN RESPONSE TO MP470 DN	20	4 (20)	4 (20)	0 (0)	0.0002	0.0026
GREGORY SYNTHETIC LETHAL WITH IMATINIB	144	10 (6.9)	9 (6.2)	1 (0.7)	0.0002	0.0027
WIERENGA STAT5A TARGETS GROUP1	130	10 (7.7)	6 (4.6)	4 (3.1)	0.0002	0.0027
LEE CALORIE RESTRICTION NEOCORTEX DN	87	8 (9.2)	5 (5.7)	3 (3.4)	0.0002	0.0027
IIZUKA LIVER CANCER PROGRESSION L0 L1 DN	20	4 (20)	4 (20)	0 (0)	0.0002	0.0027
GRAHAM CML DIVIDING VS NORMAL QUIESCENT DN	88	8 (9.1)	6 (6.8)	2 (2.3)	0.0002	0.0028
BOYLAN MULTIPLE MYELOMA PCA3 DN	65	7 (10.8)	6 (9.2)	1 (1.5)	0.0002	0.0028
SHIN B CELL LYMPHOMA CLUSTER 2	30	5 (16.7)	3 (10)	2 (6.7)	0.0002	0.0028
HELLER HDAC TARGETS DN	281	15 (5.3)	11 (3.9)	4 (1.4)	0.0002	0.0028
WONG MITOCHONDRIA GENE MODULE	209	11 (5.3)	10 (4.8)	1 (0.5)	0.0002	0.0028
FAELT B CLL WITH VH3 21 DN	47	6 (12.8)	5 (10.6)	1 (2.1)	0.0002	0.0028
MCBRYAN PUBERTAL BREAST 4 5WK UP	264	14 (5.3)	6 (2.3)	8 (3)	0.0003	0.0029
TARTE PLASMA CELL VS PLASMABLAST DN	297	15 (5.1)	14 (4.7)	1 (0.3)	0.0003	0.003
KOBAYASHI EGFR SIGNALING 24HR DN	260	14 (5.4)	11 (4.2)	3 (1.2)	0.0003	0.0032
MONNIER POSTRADIATION TUMOR ESCAPE DN	357	17 (4.8)	17 (4.8)	0 (0)	0.0003	0.0032
CHIARADONNA NEOPLASTIC TRANSFORMATION KRAS DN	143	10 (7)	7 (4.9)	3 (2.1)	0.0003	0.0032

Category Name	Category Genes #	Any-reg. SDEG #(%)	Up-reg. SDEG #(%)	Down-reg. SDEG #(%)	P	FDR
PENG LEUCINE DEPRIVATION UP	150	10 (6.7)	9 (6)	1 (0.7)	0.0003	0.0032
ZHONG SECRETOME OF LUNG CANCER AND FIBROBLAST	129	9 (7)	7 (5.4)	2 (1.6)	0.0003	0.0032
ISHIKAWA STING SIGNALING	8	3 (37.5)	3 (37.5)	0 (0)	0.0003	0.0032
MIKKELSEN ES ICP WITH H3K4ME3	678	26 (3.8)	21 (3.1)	5 (0.7)	0.0003	0.0033
YAGI AML WITH T 8 21 TRANSLOCATION	349	17 (4.9)	15 (4.3)	2 (0.6)	0.0003	0.0033
DANG REGULATED BY MYC DN	247	14 (5.7)	12 (4.9)	2 (0.8)	0.0003	0.0034
LIAO METASTASIS	505	22 (4.4)	17 (3.4)	5 (1)	0.0003	0.0035
TOOKER GEMCITABINE RESISTANCE UP	77	7 (9.1)	7 (9.1)	0 (0)	0.0003	0.0036
MULLIGHAN MLL SIGNATURE 2 DN	271	15 (5.5)	13 (4.8)	2 (0.7)	0.0003	0.0037
MIYAGAWA TARGETS OF EWSR1 ETS FUSIONS UP	247	14 (5.7)	10 (4)	4 (1.6)	0.0003	0.0037
VANHARANTA UTERINE FIBROID DN	66	7 (10.6)	7 (10.6)	0 (0)	0.0003	0.0037
LEE RECENT THYMIC EMIGRANT	217	13 (6)	12 (5.5)	1 (0.5)	0.0003	0.0038
THUM SYSTOLIC HEART FAILURE UP	404	19 (4.7)	16 (4)	3 (0.7)	0.0004	0.0038
KLEIN PRIMARY EFFUSION LYMPHOMA DN	54	6 (11.1)	6 (11.1)	0 (0)	0.0004	0.0038
CROONQUIST NRAS SIGNALING UP	35	5 (14.3)	3 (8.6)	2 (5.7)	0.0004	0.0038
KEEN RESPONSE TO ROSIGLITAZONE DN	106	8 (7.5)	6 (5.7)	2 (1.9)	0.0004	0.0039
GHANDHI BYSTANDER IRRADIATION UP	78	7 (9)	5 (6.4)	2 (2.6)	0.0004	0.0039
CHIANG LIVER CANCER SUBCLASS INTERFERON UP	24	4 (16.7)	4 (16.7)	0 (0)	0.0004	0.0039
SCHLOSSER SERUM RESPONSE DN	680	27 (4)	23 (3.4)	4 (0.6)	0.0004	0.004
JI RESPONSE TO FSH UP	85	7 (8.2)	6 (7.1)	1 (1.2)	0.0004	0.004
GESERICK TERT TARGETS DN	21	4 (19)	4 (19)	0 (0)	0.0004	0.004
MIKKELSEN MEF LCP WITH H3K4ME3	121	9 (7.4)	8 (6.6)	1 (0.8)	0.0004	0.0041
GAUSSMANN MLL AF4 FUSION TARGETS A DN	90	8 (8.9)	6 (6.7)	2 (2.2)	0.0004	0.0041
BOYLAN MULTIPLE MYELOMA C CLUSTER DN	41	5 (12.2)	2 (4.9)	3 (7.3)	0.0004	0.0041
GARGALOVIC RESPONSE TO OXIDIZED PHOSPHOLIPIDS GREY DN	73	7 (9.6)	6 (8.2)	1 (1.4)	0.0004	0.0042
ONO AML1 TARGETS UP	23	4 (17.4)	4 (17.4)	0 (0)	0.0004	0.0044
POTTI PACLITAXEL SENSITIVITY	36	5 (13.9)	4 (11.1)	1 (2.8)	0.0004	0.0045
ENK UV RESPONSE KERATINOCYTE DN	481	21 (4.4)	17 (3.5)	4 (0.8)	0.0005	0.0047
HELLER HDAC TARGETS UP	318	15 (4.7)	9 (2.8)	6 (1.9)	0.0005	0.0048
WIERENGA STAT5A TARGETS DN	219	12 (5.5)	8 (3.7)	4 (1.8)	0.0005	0.0048
ELVIDGE HYPOXIA UP	166	11 (6.6)	8 (4.8)	3 (1.8)	0.0005	0.005
BOYLAN MULTIPLE MYELOMA D DN	71	7 (9.9)	5 (7)	2 (2.8)	0.0005	0.005
HOFMANN CELL LYMPHOMA DN	37	5 (13.5)	5 (13.5)	0 (0)	0.0005	0.005
YAGI AML WITH T 9 11 TRANSLOCATION	138	9 (6.5)	7 (5.1)	2 (1.4)	0.0005	0.0052
LU IL4 SIGNALING	94	8 (8.5)	8 (8.5)	0 (0)	0.0005	0.0052
JACKSON DNMT1 TARGETS UP	76	7 (9.2)	7 (9.2)	0 (0)	0.0005	0.0052
UZONYI RESPONSE TO LEUKOTRIENE AND THROMBIN	37	5 (13.5)	2 (5.4)	3 (8.1)	0.0005	0.0052
TERAMOTO OPN TARGETS CLUSTER 7	19	4 (21.1)	3 (15.8)	1 (5.3)	0.0005	0.0052

Category Name	Category Genes #	Any-reg. SDEG #(%)	Up-reg. SDEG #(%)	Down-reg. SDEG #(%)	P	FDR
SENESE HDAC1 AND HDAC2 TARGETS UP	231	13 (5.6)	9 (3.9)	4 (1.7)	0.0005	0.0052
MATTIOLI MULTIPLE MYELOMA WITH 14Q32 TRANSLOCATIONS	33	5 (15.2)	3 (9.1)	2 (6.1)	0.0005	0.0052
MASSARWEH TAMOXIFEN RESISTANCE UP	561	24 (4.3)	21 (3.7)	3 (0.5)	0.0005	0.0054
GREENBAUM E2A TARGETS DN	21	4 (19)	4 (19)	0 (0)	0.0005	0.0054
BIDUS METASTASIS DN	153	10 (6.5)	8 (5.2)	2 (1.3)	0.0006	0.0055
TARTE PLASMA CELL VS B LYMPHOCYTE DN	36	5 (13.9)	5 (13.9)	0 (0)	0.0006	0.0056
BROWN MYELOID CELL DEVELOPMENT UP	159	10 (6.3)	9 (5.7)	1 (0.6)	0.0006	0.0056
RASHI RESPONSE TO IONIZING RADIATION 2	126	9 (7.1)	8 (6.3)	1 (0.8)	0.0006	0.0056
DASU IL6 SIGNALING UP	59	6 (10.2)	5 (8.5)	1 (1.7)	0.0006	0.0056
HELLER HDAC TARGETS SILENCED BY METHYLATION DN	269	14 (5.2)	11 (4.1)	3 (1.1)	0.0006	0.0057
MARTORIATI MDM4 TARGETS FETAL LIVER UP	224	12 (5.4)	9 (4)	3 (1.3)	0.0006	0.0059
FRASOR RESPONSE TO ESTRADIOL DN	79	7 (8.9)	5 (6.3)	2 (2.5)	0.0006	0.006
SMIRNOV CIRCULATING ENDOTHELIOCYTES IN CANCER UP	152	10 (6.6)	5 (3.3)	5 (3.3)	0.0006	0.006
FLECHNER BIOPSY KIDNEY TRANSPLANT OK VS DONOR UP	529	22 (4.2)	20 (3.8)	2 (0.4)	0.0006	0.0061
DARWICHE PAPILLOMA RISK HIGH UP	145	9 (6.2)	8 (5.5)	1 (0.7)	0.0007	0.0064
ALCALA APOPTOSIS	84	7 (8.3)	6 (7.1)	1 (1.2)	0.0007	0.0064
BURTON ADIPOGENESIS PEAK AT 0HR	61	6 (9.8)	5 (8.2)	1 (1.6)	0.0007	0.0064
PLASARI TGFB1 SIGNALING VIA NFIC 10HR UP	54	6 (11.1)	5 (9.3)	1 (1.9)	0.0007	0.0064
ZHAN V2 LATE DIFFERENTIATION GENES	45	5 (11.1)	3 (6.7)	2 (4.4)	0.0007	0.0068
MOOTHA MITOCHONDRIA	424	17 (4)	11 (2.6)	6 (1.4)	0.0007	0.0068
HAMAI APOPTOSIS VIA TRAIL DN	172	10 (5.8)	9 (5.2)	1 (0.6)	0.0007	0.0068
HUMMERICH SKIN CANCER PROGRESSION UP	87	7 (8)	6 (6.9)	1 (1.1)	0.0007	0.0068
SPIELMAN LYMPHOBLAST EUROPEAN VS ASIAN DN	570	23 (4)	22 (3.9)	1 (0.2)	0.0007	0.0069
GAVIN FOXP3 TARGETS CLUSTER P6	85	7 (8.2)	7 (8.2)	0 (0)	0.0007	0.0069
VALK AML CLUSTER 11	36	5 (13.9)	5 (13.9)	0 (0)	0.0007	0.0069
WINNEPENNINCKX MELANOMA METASTASIS DN	43	5 (11.6)	3 (7)	2 (4.7)	0.0007	0.0069
DAZARD UV RESPONSE CLUSTER G5	16	3 (18.8)	2 (12.5)	1 (6.2)	0.0008	0.0071
MCBRYAN PUBERTAL TGFB1 TARGETS DN	65	6 (9.2)	3 (4.6)	3 (4.6)	0.0008	0.0072
PASQUALUCCI LYMPHOMA BY GC STAGE DN	157	10 (6.4)	10 (6.4)	0 (0)	0.0008	0.0072
MCBRYAN PUBERTAL BREAST 3 4WK UP	212	12 (5.7)	5 (2.4)	7 (3.3)	0.0008	0.0073
HAHTOLA MYCOSIS FUNGOIDES CD4 UP	61	6 (9.8)	5 (8.2)	1 (1.6)	0.0008	0.0074
PURBEY TARGETS OF CTBP1 NOT SATB1 DN	412	18 (4.4)	15 (3.6)	3 (0.7)	0.0008	0.0075
WEST ADRENOCORTICAL CARCINOMA VS ADENOMA DN	24	4 (16.7)	4 (16.7)	0 (0)	0.0008	0.0075
RHODES UNDIFFERENTIATED CANCER	68	6 (8.8)	5 (7.4)	1 (1.5)	0.0008	0.0076
MATHEW FANCONI ANEMIA GENES	10	3 (30)	3 (30)	0 (0)	0.0008	0.0076
ELVIDGE HYPOXIA BY DMOG UP	126	9 (7.1)	6 (4.8)	3 (2.4)	0.0008	0.0076
HASEGAWA TUMORIGENESIS BY RET C634R	11	3 (27.3)	3 (27.3)	0 (0)	0.0008	0.0076
ROSS ACUTE MYELOID LEUKEMIA CBF	81	7 (8.6)	7 (8.6)	0 (0)	0.0009	0.0077

Category Name	Category Genes #	Any-reg. SDEG #(%)	Up-reg. SDEG #(%)	Down-reg. SDEG #(%)	P	FDR
SEMBA FHIT TARGETS UP	11	3 (27.3)	3 (27.3)	0 (0)	0.0009	0.0079
NAKAMURA ADIPOGENESIS LATE UP	103	8 (7.8)	6 (5.8)	2 (1.9)	0.0009	0.0081
GAJATE RESPONSE TO TRABECTEDIN UP	68	6 (8.8)	6 (8.8)	0 (0)	0.0009	0.0083
ZHONG RESPONSE TO AZACITIDINE AND TSA UP	180	10 (5.6)	7 (3.9)	3 (1.7)	0.0009	0.0084
TENEDINI MEGAKARYOCYTE MARKERS	64	6 (9.4)	3 (4.7)	3 (4.7)	0.0009	0.0084
GENTILE UV LOW DOSE UP	27	4 (14.8)	3 (11.1)	1 (3.7)	0.0009	0.0084
BHAT ESR1 TARGETS VIA AKT1 UP	283	14 (4.9)	8 (2.8)	6 (2.1)	0.001	0.0084
GAL LEUKEMIC STEM CELL DN	244	12 (4.9)	10 (4.1)	2 (0.8)	0.001	0.0087
MARSON BOUND BY FOXP3 UNSTIMULATED	1207	38 (3.1)	30 (2.5)	8 (0.7)	0.001	0.0088
LU AGING BRAIN UP	249	13 (5.2)	12 (4.8)	1 (0.4)	0.001	0.0088
DURAND STROMA MAX UP	270	14 (5.2)	10 (3.7)	4 (1.5)	0.001	0.009
MARZEC IL2 SIGNALING UP	111	8 (7.2)	4 (3.6)	4 (3.6)	0.001	0.009
WINTER HYPOXIA METAGENE	226	12 (5.3)	5 (2.2)	7 (3.1)	0.0011	0.0092
BRUINS UVC RESPONSE EARLY LATE	313	15 (4.8)	12 (3.8)	3 (1)	0.0011	0.0092
HUTTMANN B CLL POOR SURVIVAL UP	258	13 (5)	10 (3.9)	3 (1.2)	0.0011	0.0092
DIRMEIER LMP1 RESPONSE EARLY	67	6 (9)	6 (9)	0 (0)	0.0011	0.0092
HOLLEMAN PREDNISOLONE RESISTANCE B ALL DN	12	3 (25)	3 (25)	0 (0)	0.0011	0.0092
GAVIN FOXP3 TARGETS CLUSTER P3	155	10 (6.5)	6 (3.9)	4 (2.6)	0.0011	0.0093
SMIRNOV RESPONSE TO IR 6HR DN	113	8 (7.1)	7 (6.2)	1 (0.9)	0.0011	0.0095
WANG HCP PROSTATE CANCER	112	8 (7.1)	8 (7.1)	0 (0)	0.0011	0.0096
BOYAULT LIVER CANCER SUBCLASS G5 DN	27	4 (14.8)	4 (14.8)	0 (0)	0.0012	0.0098
WANG SMARCE1 TARGETS UP	265	14 (5.3)	11 (4.2)	3 (1.1)	0.0012	0.0099
IIZUKA LIVER CANCER EARLY RECURRENCE	11	3 (27.3)	2 (18.2)	1 (9.1)	0.0012	0.0099

Table A93: Significantly enriched MSigDB Chemical/Genetic Perturbation Sets (RNA-Seq, Neutrophils, Day 1). Results sorted by FDR and #SDEG genes.

Category Name	Category Genes #	Any-reg. SDEG #(%)	Up-reg. SDEG #(%)	Down-reg. SDEG #(%)	P	FDR
Influenza A	149	8 (5.4)	8 (5.4)	0 (0)	<0.0001	0.0029
Measles	115	6 (5.2)	6 (5.2)	0 (0)	<0.0001	0.0048
Herpes simplex infection	154	6 (3.9)	6 (3.9)	0 (0)	<0.0001	0.0048

Table A94: Significantly enriched KEGG Pathways (RNA-Seq, Neutrophils, Day 3). Results sorted by FDR and #SDEG genes.

Category Name	Category Genes #	Any-reg. SDEG #(%)	Up-reg. SDEG #(%)	Down-reg. SDEG #(%)	P	FDR
IMMUNE RESPONSE	231	8 (3.5)	8 (3.5)	0 (0)	<0.0001	0.0082

Category Name	Category Genes #	Any-reg. SDEG #(%)	Up-reg. SDEG #(%)	Down-reg. SDEG #(%)	P	FDR

Table A95: Significantly enriched MSigDB GO Biological Processes (RNA-Seq, Neutrophils, Day 3). Results sorted by FDR and #SDEG genes.

Category Name	Category Genes #	Any-reg. SDEG #(%)	Up-reg. SDEG #(%)	Down-reg. SDEG #(%)	P	FDR
NUYTTEN EZH2 TARGETS UP	1002	48 (4.8)	47 (4.7)	1 (0.1)	<0.0001	0.0004
FULCHER INFLAMMATORY RESPONSE LECTIN VS LPS DN	462	38 (8.2)	38 (8.2)	0 (0)	<0.0001	0.0004
NUYTTEN NIPP1 TARGETS UP	748	36 (4.8)	36 (4.8)	0 (0)	<0.0001	0.0004
HECKER IFNB1 TARGETS	92	35 (38)	35 (38)	0 (0)	<0.0001	0.0004
TAKEDA TARGETS OF NUP98 HOXA9 FUSION 3D UP	178	34 (19.1)	34 (19.1)	0 (0)	<0.0001	0.0004
BOSCO INTERFERON INDUCED ANTIVIRAL MODULE	76	33 (43.4)	32 (42.1)	1 (1.3)	<0.0001	0.0004
GRAESSMANN APOPTOSIS BY SERUM DEPRIVATION UP	541	28 (5.2)	28 (5.2)	0 (0)	<0.0001	0.0004
GRAESSMANN APOPTOSIS BY DOXORUBICIN UP	1132	26 (2.3)	25 (2.2)	1 (0.1)	<0.0001	0.0004
TAKEDA TARGETS OF NUP98 HOXA9 FUSION 10D UP	186	25 (13.4)	25 (13.4)	0 (0)	<0.0001	0.0004
TAKEDA TARGETS OF NUP98 HOXA9 FUSION 8D UP	155	24 (15.5)	24 (15.5)	0 (0)	<0.0001	0.0004
BROWNE INTERFERON RESPONSIVE GENES	68	23 (33.8)	23 (33.8)	0 (0)	<0.0001	0.0004
DAUER STAT3 TARGETS DN	51	23 (45.1)	23 (45.1)	0 (0)	<0.0001	0.0004
DODD NASOPHARYNGEAL CARCINOMA DN	1330	22 (1.7)	21 (1.6)	1 (0.1)	<0.0001	0.0004
GRAESSMANN RESPONSE TO MC AND SERUM DEPRIVATION UP	203	22 (10.8)	22 (10.8)	0 (0)	<0.0001	0.0004
MOSERLE IFNA RESPONSE	31	22 (71)	22 (71)	0 (0)	<0.0001	0.0004
SANA RESPONSE TO IFNG UP	74	22 (29.7)	22 (29.7)	0 (0)	<0.0001	0.0004
DEBIASI APOPTOSIS BY REOVIRUS INFECTION UP	317	21 (6.6)	21 (6.6)	0 (0)	<0.0001	0.0004
DER IFN BETA RESPONSE UP	101	21 (20.8)	20 (19.8)	1 (1)	<0.0001	0.0004
GOZGIT ESR1 TARGETS DN	741	21 (2.8)	20 (2.7)	1 (0.1)	<0.0001	0.0004
WALLACE PROSTATE CANCER RACE UP	275	21 (7.6)	21 (7.6)	0 (0)	<0.0001	0.0004
YANG BCL3 TARGETS UP	351	21 (6)	21 (6)	0 (0)	<0.0001	0.0004
CHEN METABOLIC SYNDROM NETWORK	1172	20 (1.7)	20 (1.7)	0 (0)	<0.0001	0.0004
COLINA TARGETS OF 4EBP1 AND 4EBP2	339	20 (5.9)	20 (5.9)	0 (0)	<0.0001	0.0004
TAKEDA TARGETS OF NUP98 HOXA9 FUSION 16D UP	169	19 (11.2)	19 (11.2)	0 (0)	<0.0001	0.0004
JISON SICKLE CELL DISEASE UP	174	18 (10.3)	18 (10.3)	0 (0)	<0.0001	0.0004
DER IFN ALPHA RESPONSE UP	74	17 (23)	17 (23)	0 (0)	<0.0001	0.0004
FEVR CTNNB1 TARGETS UP	672	16 (2.4)	16 (2.4)	0 (0)	<0.0001	0.0004
HELLER SILENCED BY METHYLATION UP	273	16 (5.9)	16 (5.9)	0 (0)	<0.0001	0.0004
ALTEMEIER RESPONSE TO LPS WITH MECHANICAL VENTILATION	126	15 (11.9)	15 (11.9)	0 (0)	<0.0001	0.0004
BAELDE DIABETIC NEPHROPATHY DN	425	15 (3.5)	14 (3.3)	1 (0.2)	<0.0001	0.0004
BENNETT SYSTEMIC LUPUS ERYTHEMATOSUS	32	15 (46.9)	15 (46.9)	0 (0)	<0.0001	0.0004
FARMER BREAST CANCER CLUSTER 1	39	15 (38.5)	15 (38.5)	0 (0)	<0.0001	0.0004
MARKEY RB1 ACUTE LOF UP	214	15 (7)	15 (7)	0 (0)	<0.0001	0.0004

Category Name	Category Genes #	Any-reg. SDEG #(%)	Up-reg. SDEG #(%)	Down-reg. SDEG #(%)	P	FDR
SANA TNF SIGNALING UP	81	15 (18.5)	15 (18.5)	0 (0)	<0.0001	0.0004
SEITZ NEOPLASTIC TRANSFORMATION BY 8P DELETION UP	69	15 (21.7)	15 (21.7)	0 (0)	<0.0001	0.0004
DOUGLAS BMI1 TARGETS DN	308	14 (4.5)	14 (4.5)	0 (0)	<0.0001	0.0004
EINAV INTERFERON SIGNATURE IN CANCER	27	14 (51.9)	14 (51.9)	0 (0)	<0.0001	0.0004
FOSTER TOLERANT MACROPHAGE DN	405	14 (3.5)	14 (3.5)	0 (0)	<0.0001	0.0004
RADAEVA RESPONSE TO IFNA1 UP	52	14 (26.9)	14 (26.9)	0 (0)	<0.0001	0.0004
SENGUPTA NASOPHARYNGEAL CARCINOMA WITH LMP1 UP	387	14 (3.6)	14 (3.6)	0 (0)	<0.0001	0.0004
ZHOU INFLAMMATORY RESPONSE LPS UP	401	14 (3.5)	14 (3.5)	0 (0)	<0.0001	0.0004
CHICAS RB1 TARGETS CONFLUENT	550	13 (2.4)	13 (2.4)	0 (0)	<0.0001	0.0004
HORIUCHI WTAP TARGETS UP	295	12 (4.1)	12 (4.1)	0 (0)	<0.0001	0.0004
KUMAR TARGETS OF MLL AF9 FUSION	389	12 (3.1)	11 (2.8)	1 (0.3)	<0.0001	0.0004
RODWELL AGING KIDNEY UP	465	12 (2.6)	12 (2.6)	0 (0)	<0.0001	0.0004
STAMBOLSKY TARGETS OF MUTATED TP53 DN	51	12 (23.5)	12 (23.5)	0 (0)	<0.0001	0.0004
TONKS TARGETS OF RUNX1 RUNX1T1 FUSION MONOCYTE UP	201	12 (6)	12 (6)	0 (0)	<0.0001	0.0004
ZHU CMV ALL UP	121	11 (9.1)	11 (9.1)	0 (0)	<0.0001	0.0004
BERTUCCI MEDULLARY VS DUCTAL BREAST CANCER UP	200	10 (5)	9 (4.5)	1 (0.5)	<0.0001	0.0004
KRASNOSELSKAYA ILF3 TARGETS UP	37	10 (27)	10 (27)	0 (0)	<0.0001	0.0004
LIANG SILENCED BY METHYLATION 2	55	10 (18.2)	10 (18.2)	0 (0)	<0.0001	0.0004
RIGGINS TAMOXIFEN RESISTANCE DN	215	10 (4.7)	10 (4.7)	0 (0)	<0.0001	0.0004
RUTELLA RESPONSE TO HGF VS CSF2RB AND IL4 UP	391	10 (2.6)	10 (2.6)	0 (0)	<0.0001	0.0004
ZHANG INTERFERON RESPONSE	23	10 (43.5)	10 (43.5)	0 (0)	<0.0001	0.0004
ZHANG RESPONSE TO IKK INHIBITOR AND TNF UP	221	10 (4.5)	10 (4.5)	0 (0)	<0.0001	0.0004
ZHU CMV 24 HR UP	93	10 (10.8)	10 (10.8)	0 (0)	<0.0001	0.0004
BOWIE RESPONSE TO TAMOXIFEN	18	9 (50)	9 (50)	0 (0)	<0.0001	0.0004
ICHIBA GRAFT VERSUS HOST DISEASE D7 UP	105	9 (8.6)	9 (8.6)	0 (0)	<0.0001	0.0004
JOHNSTONE PARVB TARGETS 2 UP	136	9 (6.6)	9 (6.6)	0 (0)	<0.0001	0.0004
KRIEG KDM3A TARGETS NOT HYPOXIA	189	9 (4.8)	8 (4.2)	1 (0.5)	<0.0001	0.0004
ROETH TERT TARGETS UP	14	9 (64.3)	9 (64.3)	0 (0)	<0.0001	0.0004
VERHAAK AML WITH NPM1 MUTATED DN	238	9 (3.8)	8 (3.4)	1 (0.4)	<0.0001	0.0004
XU AKT1 TARGETS 6HR	27	9 (33.3)	9 (33.3)	0 (0)	<0.0001	0.0004
ZHAN MULTIPLE MYELOMA LB DN	39	9 (23.1)	9 (23.1)	0 (0)	<0.0001	0.0004
BOSCO TH1 CYTOTOXIC MODULE	114	8 (7)	8 (7)	0 (0)	<0.0001	0.0004
BOWIE RESPONSE TO EXTRACELLULAR MATRIX	17	8 (47.1)	8 (47.1)	0 (0)	<0.0001	0.0004
CHANG IMMORTALIZED BY HPV31 DN	63	8 (12.7)	8 (12.7)	0 (0)	<0.0001	0.0004
DER IFN GAMMA RESPONSE UP	72	8 (11.1)	8 (11.1)	0 (0)	<0.0001	0.0004
GRANDVAUX IRF3 TARGETS UP	15	8 (53.3)	8 (53.3)	0 (0)	<0.0001	0.0004
PLASARI TGFB1 TARGETS 10HR DN	233	8 (3.4)	8 (3.4)	0 (0)	<0.0001	0.0004
UROSEVIC RESPONSE TO IMIQUIMOD	23	8 (34.8)	8 (34.8)	0 (0)	<0.0001	0.0004

Category Name	Category Genes #	Any-reg. SDEG #(%)	Up-reg. SDEG #(%)	Down-reg. SDEG #(%)	P	FDR
ZHU CMV 8 HR UP	48	8 (16.7)	8 (16.7)	0 (0)	<0.0001	0.0004
BECKER TAMOXIFEN RESISTANCE UP	49	7 (14.3)	7 (14.3)	0 (0)	<0.0001	0.0004
BROWNE HCMV INFECTION 4HR UP	53	7 (13.2)	7 (13.2)	0 (0)	<0.0001	0.0004
WIELAND UP BY HBV INFECTION	100	7 (7)	7 (7)	0 (0)	<0.0001	0.0004
BROWNE HCMV INFECTION 6HR UP	69	6 (8.7)	6 (8.7)	0 (0)	<0.0001	0.0004
CASTELLANO NRAS TARGETS UP	68	6 (8.8)	6 (8.8)	0 (0)	<0.0001	0.0004
XU HGF TARGETS INDUCED BY AKT1 6HR	18	6 (33.3)	6 (33.3)	0 (0)	<0.0001	0.0004
HAN JNK SINGALING UP	34	5 (14.7)	5 (14.7)	0 (0)	<0.0001	0.0004
JECHLINGER EPITHELIAL TO MESENCHYMAL TRANSITION UP	71	5 (7)	5 (7)	0 (0)	<0.0001	0.0004
LEE LIVER CANCER E2F1 UP	62	5 (8.1)	5 (8.1)	0 (0)	<0.0001	0.0004
MAHADEVAN RESPONSE TO MP470 UP	19	5 (26.3)	5 (26.3)	0 (0)	<0.0001	0.0004
TSAI RESPONSE TO RADIATION THERAPY	32	5 (15.6)	5 (15.6)	0 (0)	<0.0001	0.0004
WUNDER INFLAMMATORY RESPONSE AND CHOLESTEROL UP	61	5 (8.2)	5 (8.2)	0 (0)	<0.0001	0.0004
GRANDVAUX IFN RESPONSE NOT VIA IRF3	14	4 (28.6)	4 (28.6)	0 (0)	<0.0001	0.0004
TSAI DNAJB4 TARGETS UP	13	4 (30.8)	4 (30.8)	0 (0)	<0.0001	0.0004
ONKEN UVEAL MELANOMA UP	763	14 (1.8)	14 (1.8)	0 (0)	<0.0001	0.0007
JOHNSTONE PARVB TARGETS 3 UP	425	10 (2.4)	10 (2.4)	0 (0)	<0.0001	0.0007
BOQUEST STEM CELL DN	213	8 (3.8)	7 (3.3)	1 (0.5)	<0.0001	0.0007
MIKKELSEN MCV6 LCP WITH H3K4ME3	153	7 (4.6)	7 (4.6)	0 (0)	<0.0001	0.0007
MISSIAGLIA REGULATED BY METHYLATION UP	120	7 (5.8)	7 (5.8)	0 (0)	<0.0001	0.0007
WANG ESOPHAGUS CANCER VS NORMAL UP	118	6 (5.1)	6 (5.1)	0 (0)	<0.0001	0.0007
GARGALOVIC RESPONSE TO OXIDIZED PHOSPHOLIPIDS GREY DN	73	5 (6.8)	5 (6.8)	0 (0)	<0.0001	0.0007
LEE LIVER CANCER MYC E2F1 UP	56	5 (8.9)	5 (8.9)	0 (0)	<0.0001	0.0007
KIM LRRC3B TARGETS	30	4 (13.3)	4 (13.3)	0 (0)	<0.0001	0.0007
NOJIMA SFRP2 TARGETS DN	25	4 (16)	4 (16)	0 (0)	<0.0001	0.0007
ZHANG ANTIVIRAL RESPONSE TO RIBAVIRIN UP	29	4 (13.8)	4 (13.8)	0 (0)	<0.0001	0.0007
ICHIBA GRAFT VERSUS HOST DISEASE 35D UP	126	6 (4.8)	6 (4.8)	0 (0)	<0.0001	0.001
GEISS RESPONSE TO DSRNA UP	35	4 (11.4)	4 (11.4)	0 (0)	<0.0001	0.001
PEDRIOLI MIR31 TARGETS DN	398	10 (2.5)	10 (2.5)	0 (0)	<0.0001	0.0013
HIRSCH CELLULAR TRANSFORMATION SIGNATURE UP	239	8 (3.3)	7 (2.9)	1 (0.4)	<0.0001	0.0013
JACKSON DNMT1 TARGETS UP	76	5 (6.6)	5 (6.6)	0 (0)	<0.0001	0.0013
HASEGAWA TUMORIGENESIS BY RET C634R	11	3 (27.3)	3 (27.3)	0 (0)	<0.0001	0.0013
LEE RECENT THYMIC EMIGRANT	217	7 (3.2)	4 (1.8)	3 (1.4)	<0.0001	0.0016
CHEBOTAEV GR TARGETS UP	77	5 (6.5)	4 (5.2)	1 (1.3)	<0.0001	0.0016
HESS TARGETS OF HOXA9 AND MEIS1 DN	76	5 (6.6)	5 (6.6)	0 (0)	<0.0001	0.0016
WATANABE ULCERATIVE COLITIS WITH CANCER DN	13	3 (23.1)	3 (23.1)	0 (0)	<0.0001	0.0016
CHICAS RB1 TARGETS SENESCENT	552	11 (2)	11 (2)	0 (0)	<0.0001	0.0019
ACEVEDO FGFR1 TARGETS IN PROSTATE CANCER MODEL UP	278	8 (2.9)	8 (2.9)	0 (0)	<0.0001	0.0019

Category Name	Category Genes #	Any-reg. SDEG #(%)	Up-reg. SDEG #(%)	Down-reg. SDEG #(%)	P	FDR
LEE BMP2 TARGETS UP	723	12 (1.7)	12 (1.7)	0 (0)	<0.0001	0.0028
CHARAFE BREAST CANCER LUMINAL VS BASAL DN	440	10 (2.3)	9 (2)	1 (0.2)	<0.0001	0.0028
RUTELLA RESPONSE TO CSF2RB AND IL4 DN	303	8 (2.6)	8 (2.6)	0 (0)	0.0001	0.0033
FOSTER KDM1A TARGETS UP	250	7 (2.8)	7 (2.8)	0 (0)	0.0001	0.0033
LU EZH2 TARGETS UP	289	7 (2.4)	7 (2.4)	0 (0)	0.0001	0.0033
SEKI INFLAMMATORY RESPONSE LPS UP	77	5 (6.5)	5 (6.5)	0 (0)	0.0001	0.0033
LEE LIVER CANCER MYC TGFA UP	62	4 (6.5)	4 (6.5)	0 (0)	0.0001	0.0041
BOYLAN MULTIPLE MYELOMA C D DN	244	7 (2.9)	7 (2.9)	0 (0)	0.0001	0.0044
HOSHIDA LIVER CANCER SUBCLASS S3	259	7 (2.7)	7 (2.7)	0 (0)	0.0002	0.0049
ALCALAY AML BY NPM1 LOCALIZATION DN	178	6 (3.4)	5 (2.8)	1 (0.6)	0.0002	0.0049
SENGUPTA NASOPHARYNGEAL CARCINOMA UP	287	8 (2.8)	8 (2.8)	0 (0)	0.0002	0.0051
BROWNE HCMV INFECTION 12HR UP	105	5 (4.8)	5 (4.8)	0 (0)	0.0002	0.0052
MIKKELSEN MEF LCP WITH H3K4ME3	121	5 (4.1)	5 (4.1)	0 (0)	0.0002	0.0052
KANG GIST WITH PDGFRA UP	50	4 (8)	4 (8)	0 (0)	0.0002	0.0052
POOLA INVASIVE BREAST CANCER UP	272	7 (2.6)	7 (2.6)	0 (0)	0.0002	0.0055
PEREZ TP53 TARGETS	1124	16 (1.4)	15 (1.3)	1 (0.1)	0.0002	0.0057
AMIT EGF RESPONSE 480 HELA	164	6 (3.7)	6 (3.7)	0 (0)	0.0002	0.0057
LINDSTEDT DENDRITIC CELL MATURATION A	67	4 (6)	4 (6)	0 (0)	0.0002	0.0059
SMID BREAST CANCER NORMAL LIKE UP	443	9 (2)	9 (2)	0 (0)	0.0002	0.0063
WANG SMARCE1 TARGETS DN	340	8 (2.4)	7 (2.1)	1 (0.3)	0.0002	0.0063
TAVOR CEBPA TARGETS DN	30	3 (10)	3 (10)	0 (0)	0.0002	0.0063
HOLLMANN APOPTOSIS VIA CD40 DN	259	7 (2.7)	6 (2.3)	1 (0.4)	0.0002	0.0064
GILDEA METASTASIS	30	3 (10)	3 (10)	0 (0)	0.0002	0.0064
HAN SATB1 TARGETS DN	421	9 (2.1)	9 (2.1)	0 (0)	0.0003	0.0074
ONDER CDH1 TARGETS 2 DN	454	9 (2)	8 (1.8)	1 (0.2)	0.0003	0.0081
ROZANOV MMP14 TARGETS UP	270	7 (2.6)	6 (2.2)	1 (0.4)	0.0003	0.0086
GROSS ELK3 TARGETS DN	32	3 (9.4)	3 (9.4)	0 (0)	0.0004	0.009
GAUSSMANN MLL AF4 FUSION TARGETS G DN	30	3 (10)	3 (10)	0 (0)	0.0004	0.0092

Table A96: Significantly enriched MSigDB Chemical/Genetic Perturbation Sets (RNA-Seq, Neutrophils, Day 3). Results sorted by FDR and #SDEG genes.

Category Name	Category Genes #	Any-reg. SDEG #(%)	Up-reg. SDEG #(%)	Down-reg. SDEG #(%)	P	FDR
HECKER IFNB1 TARGETS	92	9 (9.8)	9 (9.8)	0 (0)	<0.0001	0.001
TAKEDA TARGETS OF NUP98 HOXA9 FUSION 3D UP	178	8 (4.5)	8 (4.5)	0 (0)	<0.0001	0.001
GRAESSMANN APOPTOSIS BY SERUM DEPRIVATION UP	541	7 (1.3)	7 (1.3)	0 (0)	<0.0001	0.001
NUYTTEN EZH2 TARGETS UP	1002	7 (0.7)	7 (0.7)	0 (0)	<0.0001	0.001
TAKEDA TARGETS OF NUP98 HOXA9 FUSION 10D UP	186	7 (3.8)	7 (3.8)	0 (0)	<0.0001	0.001

Category Name	Category Genes #	Any-reg. SDEG #(%)	Up-reg. SDEG #(%)	Down-reg. SDEG #(%)	P	FDR
FULCHER INFLAMMATORY RESPONSE LECTIN VS LPS DN	462	6 (1.3)	6 (1.3)	0 (0)	<0.0001	0.001
GRAESSMANN RESPONSE TO MC AND SERUM DEPRIVATION UP	203	6 (3)	6 (3)	0 (0)	<0.0001	0.001
MOSERLE IFNA RESPONSE	31	6 (19.4)	6 (19.4)	0 (0)	<0.0001	0.001
TAKEDA TARGETS OF NUP98 HOXA9 FUSION 16D UP	169	6 (3.6)	6 (3.6)	0 (0)	<0.0001	0.001
TAKEDA TARGETS OF NUP98 HOXA9 FUSION 8D UP	155	6 (3.9)	6 (3.9)	0 (0)	<0.0001	0.001
WALLACE PROSTATE CANCER RACE UP	275	6 (2.2)	6 (2.2)	0 (0)	<0.0001	0.001
BROWNE INTERFERON RESPONSIVE GENES	68	5 (7.4)	5 (7.4)	0 (0)	<0.0001	0.001
SANA TNF SIGNALING UP	81	5 (6.2)	5 (6.2)	0 (0)	<0.0001	0.001
ALTEMEIER RESPONSE TO LPS WITH MECHANICAL VENTILATION	126	4 (3.2)	4 (3.2)	0 (0)	<0.0001	0.001
BOSCO INTERFERON INDUCED ANTIVIRAL MODULE	76	4 (5.3)	4 (5.3)	0 (0)	<0.0001	0.001
DER IFN ALPHA RESPONSE UP	74	4 (5.4)	4 (5.4)	0 (0)	<0.0001	0.001
DER IFN BETA RESPONSE UP	101	4 (4)	4 (4)	0 (0)	<0.0001	0.001
DER IFN GAMMA RESPONSE UP	72	4 (5.6)	4 (5.6)	0 (0)	<0.0001	0.001
FARMER BREAST CANCER CLUSTER 1	39	4 (10.3)	4 (10.3)	0 (0)	<0.0001	0.001
ICHIBA GRAFT VERSUS HOST DISEASE D7 UP	105	4 (3.8)	4 (3.8)	0 (0)	<0.0001	0.001
JISON SICKLE CELL DISEASE UP	174	4 (2.3)	4 (2.3)	0 (0)	<0.0001	0.001
KRIEG KDM3A TARGETS NOT HYPOXIA	189	4 (2.1)	4 (2.1)	0 (0)	<0.0001	0.001
PLASARI TGFB1 TARGETS 10HR DN	233	4 (1.7)	4 (1.7)	0 (0)	<0.0001	0.001
SANA RESPONSE TO IFNG UP	74	4 (5.4)	4 (5.4)	0 (0)	<0.0001	0.001
BENNETT SYSTEMIC LUPUS ERYTHEMATOSUS	32	3 (9.4)	3 (9.4)	0 (0)	<0.0001	0.001
BOWIE RESPONSE TO EXTRACELLULAR MATRIX	17	3 (17.6)	3 (17.6)	0 (0)	<0.0001	0.001
BOWIE RESPONSE TO TAMOXIFEN	18	3 (16.7)	3 (16.7)	0 (0)	<0.0001	0.001
DAUER STAT3 TARGETS DN	51	3 (5.9)	3 (5.9)	0 (0)	<0.0001	0.001
EINAV INTERFERON SIGNATURE IN CANCER	27	3 (11.1)	3 (11.1)	0 (0)	<0.0001	0.001
LIANG SILENCED BY METHYLATION 2	55	3 (5.5)	3 (5.5)	0 (0)	<0.0001	0.001
RADAEVA RESPONSE TO IFNA1 UP	52	3 (5.8)	3 (5.8)	0 (0)	<0.0001	0.001
TSAI DNAJB4 TARGETS UP	13	3 (23.1)	3 (23.1)	0 (0)	<0.0001	0.001
ZHANG INTERFERON RESPONSE	23	3 (13)	3 (13)	0 (0)	<0.0001	0.001
BERTUCCI MEDULLARY VS DUCTAL BREAST CANCER UP	200	4 (2)	4 (2)	0 (0)	<0.0001	0.0017
BENPORATH ES CORE NINE CORRELATED	95	3 (3.2)	3 (3.2)	0 (0)	<0.0001	0.0017
LIU VAV3 PROSTATE CARCINOGENESIS UP	85	3 (3.5)	3 (3.5)	0 (0)	<0.0001	0.0017
SEITZ NEOPLASTIC TRANSFORMATION BY 8P DELETION UP	69	3 (4.3)	3 (4.3)	0 (0)	<0.0001	0.0017
WUNDER INFLAMMATORY RESPONSE AND CHOLESTEROL UP	61	3 (4.9)	3 (4.9)	0 (0)	<0.0001	0.0017
GRANDVAUX IFN RESPONSE NOT VIA IRF3	14	2 (14.3)	2 (14.3)	0 (0)	<0.0001	0.0017
HELLER SILENCED BY METHYLATION UP	273	4 (1.5)	4 (1.5)	0 (0)	<0.0001	0.0024
GARGALOVIC RESPONSE TO OXIDIZED PHOSPHOLIPIDS GREY DN	73	3 (4.1)	3 (4.1)	0 (0)	<0.0001	0.0024
RASHI NFKB1 TARGETS	19	2 (10.5)	2 (10.5)	0 (0)	<0.0001	0.0024
DEBIASI APOPTOSIS BY REOVIRUS INFECTION UP	317	4 (1.3)	4 (1.3)	0 (0)	<0.0001	0.0031

Category Name	Category Genes #	Any-reg. SDEG #(%)	Up-reg. SDEG #(%)	Down-reg. SDEG #(%)	P	FDR
RUAN RESPONSE TO TNF TROGLITAZONE UP	17	2 (11.8)	2 (11.8)	0 (0)	<0.0001	0.0031
MARKEY RB1 ACUTE LOF UP	214	4 (1.9)	4 (1.9)	0 (0)	<0.0001	0.0036
ROETH TERT TARGETS UP	14	2 (14.3)	2 (14.3)	0 (0)	<0.0001	0.0036
RUAN RESPONSE TO TNF UP	12	2 (16.7)	2 (16.7)	0 (0)	<0.0001	0.0036
FEVR CTNNB1 TARGETS UP	672	5 (0.7)	5 (0.7)	0 (0)	<0.0001	0.0062
GRANDVAUX IRF3 TARGETS UP	15	2 (13.3)	2 (13.3)	0 (0)	<0.0001	0.0062
YAN ESCAPE FROM ANOIKIS	24	2 (8.3)	2 (8.3)	0 (0)	0.0001	0.0088

Table A97: Significantly enriched MSigDB Chemical/Genetic Perturbation Sets (RNA-Seq, NK-cells, Day 1). Results sorted by FDR and #SDEG genes.

Category Name	Category Genes #	Any-reg. SDEG #(%)	Up-reg. SDEG #(%)	Down-reg. SDEG #(%)	P	FDR
Cell cycle	113	13 (11.5)	12 (10.6)	1 (0.9)	<0.0001	0.0006
MicroRNAs in cancer	228	7 (3.1)	7 (3.1)	0 (0)	<0.0001	0.0006
Oocyte meiosis	97	6 (6.2)	6 (6.2)	0 (0)	<0.0001	0.0006
Progesterone-mediated oocyte maturation	74	5 (6.8)	5 (6.8)	0 (0)	<0.0001	0.0006
Hepatitis B	131	5 (3.8)	5 (3.8)	0 (0)	<0.0001	0.0006
p53 signaling pathway	62	4 (6.5)	4 (6.5)	0 (0)	<0.0001	0.001

Table A98: Significantly enriched KEGG Pathways (RNA-Seq, NK-cells, Day 3). Results sorted by FDR and #SDEG genes.

Category Name	Category Genes #	Any-reg. SDEG #(%)	Up-reg. SDEG #(%)	Down-reg. SDEG #(%)	P	FDR
CELL CYCLE GO 0007049	308	25 (8.1)	24 (7.8)	1 (0.3)	<0.0001	0.0003
CELL CYCLE PROCESS	188	23 (12.2)	22 (11.7)	1 (0.5)	<0.0001	0.0003
MITOTIC CELL CYCLE	149	22 (14.8)	21 (14.1)	1 (0.7)	<0.0001	0.0003
CELL CYCLE PHASE	165	20 (12.1)	19 (11.5)	1 (0.6)	<0.0001	0.0003
M PHASE	112	17 (15.2)	17 (15.2)	0 (0)	<0.0001	0.0003
M PHASE OF MITOTIC CELL CYCLE	84	17 (20.2)	17 (20.2)	0 (0)	<0.0001	0.0003
MITOSIS	81	16 (19.8)	16 (19.8)	0 (0)	<0.0001	0.0003
ORGANELLE ORGANIZATION AND BIOGENESIS	449	12 (2.7)	12 (2.7)	0 (0)	<0.0001	0.0003
REGULATION OF CELL CYCLE	179	11 (6.1)	10 (5.6)	1 (0.6)	<0.0001	0.0003
CELL PROLIFERATION GO 0008283	493	10 (2)	9 (1.8)	1 (0.2)	<0.0001	0.0003
REGULATION OF MITOSIS	41	9 (22)	9 (22)	0 (0)	<0.0001	0.0003
CHROMOSOME SEGREGATION	31	8 (25.8)	8 (25.8)	0 (0)	<0.0001	0.0003
CHROMOSOME ORGANIZATION AND BIOGENESIS	116	7 (6)	7 (6)	0 (0)	<0.0001	0.0003
MITOTIC SISTER CHROMATID SEGREGATION	15	6 (40)	6 (40)	0 (0)	<0.0001	0.0003

Category Name	Category Genes #	Any-reg. SDEG #(%)	Up-reg. SDEG #(%)	Down-reg. SDEG #(%)	P	FDR
SISTER CHROMATID SEGREGATION	16	6 (37.5)	6 (37.5)	0 (0)	<0.0001	0.0003
CELL CYCLE CHECKPOINT GO 0000075	48	5 (10.4)	5 (10.4)	0 (0)	<0.0001	0.0003
ESTABLISHMENT OF ORGANELLE LOCALIZATION	17	5 (29.4)	5 (29.4)	0 (0)	<0.0001	0.0003
INTERPHASE	65	5 (7.7)	4 (6.2)	1 (1.5)	<0.0001	0.0003
INTERPHASE OF MITOTIC CELL CYCLE	59	5 (8.5)	4 (6.8)	1 (1.7)	<0.0001	0.0003
ORGANELLE LOCALIZATION	24	5 (20.8)	5 (20.8)	0 (0)	<0.0001	0.0003
CELL DIVISION	19	4 (21.1)	4 (21.1)	0 (0)	<0.0001	0.0003
CHROMOSOME CONDENSATION	10	4 (40)	4 (40)	0 (0)	<0.0001	0.0003
CYTOKINESIS	17	4 (23.5)	4 (23.5)	0 (0)	<0.0001	0.0003
DNA PACKAGING	32	4 (12.5)	4 (12.5)	0 (0)	<0.0001	0.0003
MICROTUBULE CYTOSKELETON ORGANIZATION AND BIOGENESIS	31	4 (12.9)	4 (12.9)	0 (0)	<0.0001	0.0003
MITOTIC CELL CYCLE CHECKPOINT	21	4 (19)	4 (19)	0 (0)	<0.0001	0.0003
MICROTUBULE BASED PROCESS	75	4 (5.3)	4 (5.3)	0 (0)	<0.0001	0.0015
CYTOSKELETON ORGANIZATION AND BIOGENESIS	196	5 (2.6)	5 (2.6)	0 (0)	<0.0001	0.0021
REGULATION OF CYCLIN DEPENDENT PROTEIN KINASE ACTIVITY	43	3 (7)	2 (4.7)	1 (2.3)	0.0001	0.0031
ESTABLISHMENT OF CELLULAR LOCALIZATION	341	6 (1.8)	6 (1.8)	0 (0)	0.0001	0.0036
CELLULAR LOCALIZATION	359	6 (1.7)	6 (1.7)	0 (0)	0.0002	0.0043
MITOTIC SPINDLE ORGANIZATION AND BIOGENESIS	9	2 (22.2)	2 (22.2)	0 (0)	0.0002	0.0045
SPINDLE ORGANIZATION AND BIOGENESIS	10	2 (20)	2 (20)	0 (0)	0.0002	0.0045
G1 PHASE OF MITOTIC CELL CYCLE	11	2 (18.2)	1 (9.1)	1 (9.1)	0.0003	0.007

Table A99: Significantly enriched MSigDB GO Biological Processes (RNA-Seq, NK-cells, Day 3). Results sorted by FDR and #SDEG genes.

Category Name	Category Genes #	Any-reg. SDEG #(%)	Up-reg. SDEG #(%)	Down-reg. SDEG #(%)	P	FDR
INTRACELLULAR ORGANELLE PART	1155	19 (1.6)	19 (1.6)	0 (0)	<0.0001	0.0001
ORGANELLE PART	1160	19 (1.6)	19 (1.6)	0 (0)	<0.0001	0.0001
INTRACELLULAR NON MEMBRANE BOUND ORGANELLE	607	18 (3)	18 (3)	0 (0)	<0.0001	0.0001
NON MEMBRANE BOUND ORGANELLE	607	18 (3)	18 (3)	0 (0)	<0.0001	0.0001
CYTOSKELETAL PART	225	14 (6.2)	14 (6.2)	0 (0)	<0.0001	0.0001
CYTOSKELETON	347	14 (4)	14 (4)	0 (0)	<0.0001	0.0001
MACROMOLECULAR COMPLEX	925	13 (1.4)	13 (1.4)	0 (0)	<0.0001	0.0001
MICROTUBULE CYTOSKELETON	146	13 (8.9)	13 (8.9)	0 (0)	<0.0001	0.0001
NUCLEUS	1353	13 (1)	13 (1)	0 (0)	<0.0001	0.0001
PROTEIN COMPLEX	801	13 (1.6)	13 (1.6)	0 (0)	<0.0001	0.0001
CHROMOSOME	130	8 (6.2)	8 (6.2)	0 (0)	<0.0001	0.0001
SPINDLE	38	8 (21.1)	8 (21.1)	0 (0)	<0.0001	0.0001
CHROMOSOMAL PART	106	7 (6.6)	7 (6.6)	0 (0)	<0.0001	0.0001

Category Name	Category Genes #	Any-reg. SDEG #(%)	Up-reg. SDEG #(%)	Down-reg. SDEG #(%)	P	FDR
MICROTUBULE ORGANIZING CENTER	65	7 (10.8)	7 (10.8)	0 (0)	<0.0001	0.0001
CENTROSOME	56	6 (10.7)	6 (10.7)	0 (0)	<0.0001	0.0001
CHROMOSOMEPERICENTRIC REGION	31	6 (19.4)	6 (19.4)	0 (0)	<0.0001	0.0001
KINETOCHORE	25	5 (20)	5 (20)	0 (0)	<0.0001	0.0001
SPINDLE POLE	18	4 (22.2)	4 (22.2)	0 (0)	<0.0001	0.0001
KINESIN COMPLEX	15	3 (20)	3 (20)	0 (0)	<0.0001	0.0002
CYTOPLASM	2054	15 (0.7)	14 (0.7)	1 (0)	<0.0001	0.0003
CYTOPLASMIC PART	1335	11 (0.8)	11 (0.8)	0 (0)	0.0002	0.0019
MICROTUBULE ASSOCIATED COMPLEX	45	3 (6.7)	3 (6.7)	0 (0)	0.0002	0.002
SPINDLE MICROTUBULE	15	2 (13.3)	2 (13.3)	0 (0)	0.0007	0.0068
MICROTUBULE ORGANIZING CENTER PART	18	2 (11.1)	2 (11.1)	0 (0)	0.0009	0.0084

Table A100: Significantly enriched MSigDB GO Cellular Components (RNA-Seq, NK-cells, Day 3). Results sorted by FDR and #SDEG genes.

Category Name	Category Genes #	Any-reg. SDEG #(%)	Up-reg. SDEG #(%)	Down-reg. SDEG #(%)	P	FDR
MICROTUBULE MOTOR ACTIVITY	15	3 (20)	3 (20)	0 (0)	<0.0001	0.0079

Table A101: Significantly enriched MSigDB GO Molecular Functions (RNA-Seq, NK-cells, Day 3). Results sorted by FDR and #SDEG genes.

Category Name	Category Genes #	Any-reg. SDEG #(%)	Up-reg. SDEG #(%)	Down-reg. SDEG #(%)	P	FDR
BIOCARTA CELLCYCLE PATHWAY	23	4 (17.4)	4 (17.4)	0 (0)	<0.0001	0.0011
BIOCARTA G1 PATHWAY	28	4 (14.3)	4 (14.3)	0 (0)	<0.0001	0.0011

Table A102: Significantly enriched MSigDB BioCarta Pathways (RNA-Seq, NK-cells, Day 3). Results sorted by FDR and #SDEG genes.

Category Name	Category Genes #	Any-reg. SDEG #(%)	Up-reg. SDEG #(%)	Down-reg. SDEG #(%)	P	FDR
KINSEY TARGETS OF EWSR1 FLII FUSION UP	1244	55 (4.4)	55 (4.4)	0 (0)	<0.0001	0.0002
GOBERT OLIGODENDROCYTE DIFFERENTIATION UP	554	54 (9.7)	54 (9.7)	0 (0)	<0.0001	0.0002
DUTERTRE ESTRADIOL RESPONSE 24HR UP	313	51 (16.3)	51 (16.3)	0 (0)	<0.0001	0.0002
DODD NASOPHARYNGEAL CARCINOMA DN	1330	47 (3.5)	47 (3.5)	0 (0)	<0.0001	0.0002
NUYTTEN EZH2 TARGETS DN	985	47 (4.8)	47 (4.8)	0 (0)	<0.0001	0.0002
SHEDDEN LUNG CANCER POOR SURVIVAL A6	453	46 (10.2)	46 (10.2)	0 (0)	<0.0001	0.0002
MARSON BOUND BY E2F4 UNSTIMULATED	722	45 (6.2)	45 (6.2)	0 (0)	<0.0001	0.0002

Category Name	Category Genes #	Any-reg. SDEG #(%)	Up-reg. SDEG #(%)	Down-reg. SDEG #(%)	P	FDR
ROSTY CERVICAL CANCER PROLIFERATION CLUSTER	137	45 (32.8)	45 (32.8)	0 (0)	<0.0001	0.0002
SOTIRIOU BREAST CANCER GRADE 1 VS 3 UP	157	43 (27.4)	43 (27.4)	0 (0)	<0.0001	0.0002
BENPORATH CYCLING GENES	653	42 (6.4)	42 (6.4)	0 (0)	<0.0001	0.0002
KOBAYASHI EGFR SIGNALING 24HR DN	260	41 (15.8)	41 (15.8)	0 (0)	<0.0001	0.0002
VECCHI GASTRIC CANCER EARLY UP	413	40 (9.7)	40 (9.7)	0 (0)	<0.0001	0.0002
HORIUCHI WTAP TARGETS DN	308	39 (12.7)	39 (12.7)	0 (0)	<0.0001	0.0002
PATIL LIVER CANCER	729	34 (4.7)	34 (4.7)	0 (0)	<0.0001	0.0002
CAIRO HEPATOBLASTOMA CLASSES UP	585	33 (5.6)	33 (5.6)	0 (0)	<0.0001	0.0002
CHANG CYCLING GENES	141	33 (23.4)	33 (23.4)	0 (0)	<0.0001	0.0002
PUJANA BRCA1 PCC NETWORK	1608	33 (2.1)	33 (2.1)	0 (0)	<0.0001	0.0002
GRAHAM CML DIVIDING VS NORMAL QUIESCENT UP	175	32 (18.3)	32 (18.3)	0 (0)	<0.0001	0.0002
BERENJENO TRANSFORMED BY RHOA UP	520	30 (5.8)	30 (5.8)	0 (0)	<0.0001	0.0002
CASORELLI ACUTE PROMYELOCYTIC LEUKEMIA DN	624	30 (4.8)	30 (4.8)	0 (0)	<0.0001	0.0002
CHIANG LIVER CANCER SUBCLASS PROLIFERATION UP	183	30 (16.4)	30 (16.4)	0 (0)	<0.0001	0.0002
LEE EARLY T LYMPHOCYTE UP	101	30 (29.7)	30 (29.7)	0 (0)	<0.0001	0.0002
WHITEFORD PEDIATRIC CANCER MARKERS	127	30 (23.6)	30 (23.6)	0 (0)	<0.0001	0.0002
BASAKI YBX1 TARGETS UP	285	29 (10.2)	29 (10.2)	0 (0)	<0.0001	0.0002
GEORGES TARGETS OF MIR192 AND MIR215	858	29 (3.4)	29 (3.4)	0 (0)	<0.0001	0.0002
JOHNSTONE PARVB TARGETS 3 DN	885	29 (3.3)	29 (3.3)	0 (0)	<0.0001	0.0002
PUJANA BRCA2 PCC NETWORK	408	29 (7.1)	29 (7.1)	0 (0)	<0.0001	0.0002
BLUM RESPONSE TO SALIRASIB DN	327	27 (8.3)	27 (8.3)	0 (0)	<0.0001	0.0002
GOLDRATH ANTIGEN RESPONSE	333	27 (8.1)	27 (8.1)	0 (0)	<0.0001	0.0002
WANG RESPONSE TO GSK3 INHIBITOR SB216763 DN	379	27 (7.1)	27 (7.1)	0 (0)	<0.0001	0.0002
CHEMNITZ RESPONSE TO PROSTAGLANDIN E2 UP	140	26 (18.6)	26 (18.6)	0 (0)	<0.0001	0.0002
LINDGREN BLADDER CANCER CLUSTER 3 UP	324	26 (8)	26 (8)	0 (0)	<0.0001	0.0002
PUJANA CHEK2 PCC NETWORK	779	26 (3.3)	26 (3.3)	0 (0)	<0.0001	0.0002
ZHANG TLX TARGETS 60HR DN	269	26 (9.7)	26 (9.7)	0 (0)	<0.0001	0.0002
GRAHAM NORMAL QUIESCENT VS NORMAL DIVIDING DN	85	25 (29.4)	25 (29.4)	0 (0)	<0.0001	0.0002
HOFFMANN LARGE TO SMALL PRE BII LYMPHOCYTE UP	164	25 (15.2)	25 (15.2)	0 (0)	<0.0001	0.0002
LINDGREN BLADDER CANCER CLUSTER 1 DN	370	25 (6.8)	25 (6.8)	0 (0)	<0.0001	0.0002
MITSIADES RESPONSE TO APLIDIN DN	246	24 (9.8)	24 (9.8)	0 (0)	<0.0001	0.0002
SARRIO EPITHELIAL MESENCHYMAL TRANSITION UP	189	24 (12.7)	24 (12.7)	0 (0)	<0.0001	0.0002
SMID BREAST CANCER BASAL UP	607	24 (4)	24 (4)	0 (0)	<0.0001	0.0002
CROONQUIST IL6 DEPRIVATION DN	97	23 (23.7)	23 (23.7)	0 (0)	<0.0001	0.0002
GAL LEUKEMIC STEM CELL DN	244	23 (9.4)	23 (9.4)	0 (0)	<0.0001	0.0002
KIM WT1 TARGETS DN	448	23 (5.1)	23 (5.1)	0 (0)	<0.0001	0.0002
MARKEY RB1 ACUTE LOF DN	218	23 (10.6)	23 (10.6)	0 (0)	<0.0001	0.0002
NAKAYAMA SOFT TISSUE TUMORS PCA2 UP	82	23 (28)	23 (28)	0 (0)	<0.0001	0.0002

Category Name	Category Genes #	Any-reg. SDEG #(%)	Up-reg. SDEG #(%)	Down-reg. SDEG #(%)	P	FDR
POOLA INVASIVE BREAST CANCER UP	272	23 (8.5)	23 (8.5)	0 (0)	<0.0001	0.0002
RODRIGUES THYROID CARCINOMA POORLY DIFFERENTIATED UP	628	23 (3.7)	23 (3.7)	0 (0)	<0.0001	0.0002
ODONNELL TFRC TARGETS DN	138	22 (15.9)	22 (15.9)	0 (0)	<0.0001	0.0002
SENGUPTA NASOPHARYNGEAL CARCINOMA UP	287	22 (7.7)	22 (7.7)	0 (0)	<0.0001	0.0002
FERREIRA EWINGS SARCOMA UNSTABLE VS STABLE UP	165	21 (12.7)	21 (12.7)	0 (0)	<0.0001	0.0002
FEVR CTNNB1 TARGETS DN	531	21 (4)	21 (4)	0 (0)	<0.0001	0.0002
GAVIN FOXP3 TARGETS CLUSTER P6	85	21 (24.7)	21 (24.7)	0 (0)	<0.0001	0.0002
KANG DOXORUBICIN RESISTANCE UP	56	21 (37.5)	21 (37.5)	0 (0)	<0.0001	0.0002
KONG E2F3 TARGETS	107	21 (19.6)	21 (19.6)	0 (0)	<0.0001	0.0002
RODRIGUES THYROID CARCINOMA ANAPLASTIC UP	707	21 (3)	21 (3)	0 (0)	<0.0001	0.0002
TOYOTA TARGETS OF MIR34B AND MIR34C	463	21 (4.5)	21 (4.5)	0 (0)	<0.0001	0.0002
WONG EMBRYONIC STEM CELL CORE	324	21 (6.5)	20 (6.2)	1 (0.3)	<0.0001	0.0002
ZHOU CELL CYCLE GENES IN IR RESPONSE 24HR	126	21 (16.7)	21 (16.7)	0 (0)	<0.0001	0.0002
BENPORATH PROLIFERATION	147	20 (13.6)	20 (13.6)	0 (0)	<0.0001	0.0002
MEISSNER BRAIN HCP WITH H3K4ME3 AND H3K27ME3	1060	20 (1.9)	19 (1.8)	1 (0.1)	<0.0001	0.0002
TIEN INTESTINE PROBIOTICS 24HR UP	538	20 (3.7)	20 (3.7)	0 (0)	<0.0001	0.0002
WINNEPENNINCKX MELANOMA METASTASIS UP	159	20 (12.6)	20 (12.6)	0 (0)	<0.0001	0.0002
BURTON ADIPOGENESIS 3	97	19 (19.6)	19 (19.6)	0 (0)	<0.0001	0.0002
MISSIAGLIA REGULATED BY METHYLATION DN	114	19 (16.7)	19 (16.7)	0 (0)	<0.0001	0.0002
RUIZ TNC TARGETS DN	140	19 (13.6)	19 (13.6)	0 (0)	<0.0001	0.0002
YANG BCL3 TARGETS UP	351	19 (5.4)	19 (5.4)	0 (0)	<0.0001	0.0002
BUYTAERT PHOTODYNAMIC THERAPY STRESS DN	618	18 (2.9)	18 (2.9)	0 (0)	<0.0001	0.0002
ISHIDA E2F TARGETS	52	18 (34.6)	18 (34.6)	0 (0)	<0.0001	0.0002
KRIEG HYPOXIA NOT VIA KDM3A	718	18 (2.5)	18 (2.5)	0 (0)	<0.0001	0.0002
SHEPARD CRUSH AND BURN MUTANT DN	186	18 (9.7)	18 (9.7)	0 (0)	<0.0001	0.0002
WEST ADRENOCORTICAL TUMOR UP	291	18 (6.2)	18 (6.2)	0 (0)	<0.0001	0.0002
WHITFIELD CELL CYCLE G2 M	212	18 (8.5)	18 (8.5)	0 (0)	<0.0001	0.0002
ALCALAY AML BY NPM1 LOCALIZATION DN	178	17 (9.6)	17 (9.6)	0 (0)	<0.0001	0.0002
FUJII YBX1 TARGETS DN	193	17 (8.8)	17 (8.8)	0 (0)	<0.0001	0.0002
LE EGR2 TARGETS UP	106	17 (16)	16 (15.1)	1 (0.9)	<0.0001	0.0002
SHEPARD BMYB MORPHOLINO DN	193	17 (8.8)	17 (8.8)	0 (0)	<0.0001	0.0002
TARTE PLASMA CELL VS PLASMABLAST DN	297	17 (5.7)	17 (5.7)	0 (0)	<0.0001	0.0002
WHITFIELD CELL CYCLE G2	178	17 (9.6)	17 (9.6)	0 (0)	<0.0001	0.0002
ZHENG GLIOBLASTOMA PLASTICITY UP	252	17 (6.7)	17 (6.7)	0 (0)	<0.0001	0.0002
ZWANG TRANSIENTLY UP BY 1ST EGF PULSE ONLY	1723	17 (1)	17 (1)	0 (0)	<0.0001	0.0002
AFFAR YY1 TARGETS DN	229	16 (7)	16 (7)	0 (0)	<0.0001	0.0002
BENPORATH ES 1	361	16 (4.4)	16 (4.4)	0 (0)	<0.0001	0.0002
CHICAS RB1 TARGETS GROWING	233	16 (6.9)	16 (6.9)	0 (0)	<0.0001	0.0002

Category Name	Category Genes #	Any-reg. SDEG #(%)	Up-reg. SDEG #(%)	Down-reg. SDEG #(%)	P	FDR
CROONQUIST NRAS SIGNALING DN	72	16 (22.2)	16 (22.2)	0 (0)	<0.0001	0.0002
CUI TCF21 TARGETS 2 UP	416	16 (3.8)	16 (3.8)	0 (0)	<0.0001	0.0002
LI WILMS TUMOR VS FETAL KIDNEY 1 DN	160	16 (10)	16 (10)	0 (0)	<0.0001	0.0002
RHEIN ALL GLUCOCORTICOID THERAPY DN	352	16 (4.5)	16 (4.5)	0 (0)	<0.0001	0.0002
TANG SENESCENCE TP53 TARGETS DN	59	16 (27.1)	16 (27.1)	0 (0)	<0.0001	0.0002
ZHANG TLX TARGETS 36HR DN	177	16 (9)	16 (9)	0 (0)	<0.0001	0.0002
ZHAN MULTIPLE MYELOMA PR UP	42	16 (38.1)	16 (38.1)	0 (0)	<0.0001	0.0002
BIDUS METASTASIS UP	199	15 (7.5)	15 (7.5)	0 (0)	<0.0001	0.0002
HAMAI APOPTOSIS VIA TRAIL UP	556	15 (2.7)	15 (2.7)	0 (0)	<0.0001	0.0002
MORI IMMATURE B LYMPHOCYTE DN	90	15 (16.7)	15 (16.7)	0 (0)	<0.0001	0.0002
MORI LARGE PRE BII LYMPHOCYTE UP	86	15 (17.4)	15 (17.4)	0 (0)	<0.0001	0.0002
PUJANA ATM PCC NETWORK	1390	15 (1.1)	15 (1.1)	0 (0)	<0.0001	0.0002
PUJANA XPRSS INT NETWORK	163	15 (9.2)	15 (9.2)	0 (0)	<0.0001	0.0002
SHEPARD BMYB TARGETS	74	15 (20.3)	15 (20.3)	0 (0)	<0.0001	0.0002
WHITFIELD CELL CYCLE LITERATURE	44	15 (34.1)	15 (34.1)	0 (0)	<0.0001	0.0002
COLINA TARGETS OF 4EBP1 AND 4EBP2	339	14 (4.1)	14 (4.1)	0 (0)	<0.0001	0.0002
CONCANNON APOPTOSIS BY EPOXOMICIN DN	172	14 (8.1)	14 (8.1)	0 (0)	<0.0001	0.0002
OXFORD RALA OR RALB TARGETS UP	45	14 (31.1)	14 (31.1)	0 (0)	<0.0001	0.0002
WEI MYCN TARGETS WITH E BOX	766	14 (1.8)	14 (1.8)	0 (0)	<0.0001	0.0002
WILCOX PRESPONSE TO ROGESTERONE UP	149	14 (9.4)	14 (9.4)	0 (0)	<0.0001	0.0002
WU APOPTOSIS BY CDKN1A VIA TP53	55	14 (25.5)	14 (25.5)	0 (0)	<0.0001	0.0002
ZHANG BREAST CANCER PROGENITORS UP	410	14 (3.4)	14 (3.4)	0 (0)	<0.0001	0.0002
CROONQUIST NRAS VS STROMAL STIMULATION DN	109	13 (11.9)	13 (11.9)	0 (0)	<0.0001	0.0002
MANALO HYPOXIA DN	280	13 (4.6)	13 (4.6)	0 (0)	<0.0001	0.0002
MUELLER PLURINET	302	13 (4.3)	13 (4.3)	0 (0)	<0.0001	0.0002
SASAKI ADULT T CELL LEUKEMIA	186	13 (7)	13 (7)	0 (0)	<0.0001	0.0002
SERVITJA LIVER HNF1A TARGETS UP	135	13 (9.6)	12 (8.9)	1 (0.7)	<0.0001	0.0002
VANTVEER BREAST CANCER METASTASIS DN	117	13 (11.1)	13 (11.1)	0 (0)	<0.0001	0.0002
ZHOU CELL CYCLE GENES IN IR RESPONSE 6HR	84	13 (15.5)	13 (15.5)	0 (0)	<0.0001	0.0002
AMUNDSON GAMMA RADIATION RESPONSE	39	12 (30.8)	12 (30.8)	0 (0)	<0.0001	0.0002
BURTON ADIPOGENESIS PEAK AT 24HR	43	12 (27.9)	12 (27.9)	0 (0)	<0.0001	0.0002
FOURNIER ACINAR DEVELOPMENT LATE 2	269	12 (4.5)	12 (4.5)	0 (0)	<0.0001	0.0002
FURUKAWA DUSP6 TARGETS PCI35 DN	72	12 (16.7)	12 (16.7)	0 (0)	<0.0001	0.0002
GARCIA TARGETS OF FLI1 AND DAX1 DN	179	12 (6.7)	12 (6.7)	0 (0)	<0.0001	0.0002
GRAHAM CML QUIESCENT VS NORMAL QUIESCENT UP	84	12 (14.3)	12 (14.3)	0 (0)	<0.0001	0.0002
LEE BMP2 TARGETS DN	856	12 (1.4)	12 (1.4)	0 (0)	<0.0001	0.0002
MARTENS TRETINOIN RESPONSE DN	823	12 (1.5)	12 (1.5)	0 (0)	<0.0001	0.0002
PEDERSEN METASTASIS BY ERBB2 ISOFORM 7	401	12 (3)	12 (3)	0 (0)	<0.0001	0.0002

Category Name	Category Genes #	Any-reg. SDEG #(%)	Up-reg. SDEG #(%)	Down-reg. SDEG #(%)	P	FDR
PETROVA ENDOTHELIUM LYMPHATIC VS BLOOD UP	143	12 (8.4)	11 (7.7)	1 (0.7)	<0.0001	0.0002
RHODES UNDIFFERENTIATED CANCER	68	12 (17.6)	12 (17.6)	0 (0)	<0.0001	0.0002
WHITFIELD CELL CYCLE S	180	12 (6.7)	12 (6.7)	0 (0)	<0.0001	0.0002
FARMER BREAST CANCER CLUSTER 2	32	11 (34.4)	11 (34.4)	0 (0)	<0.0001	0.0002
LIAO METASTASIS	505	11 (2.2)	10 (2)	1 (0.2)	<0.0001	0.0002
MOLENAAR TARGETS OF CCND1 AND CDK4 DN	54	11 (20.4)	11 (20.4)	0 (0)	<0.0001	0.0002
MORI PRE BI LYMPHOCYTE UP	79	11 (13.9)	11 (13.9)	0 (0)	<0.0001	0.0002
REICHERT MITOSIS LIN9 TARGETS	29	11 (37.9)	11 (37.9)	0 (0)	<0.0001	0.0002
SMIRNOV RESPONSE TO IR 6HR DN	113	11 (9.7)	11 (9.7)	0 (0)	<0.0001	0.0002
YU MYC TARGETS UP	42	11 (26.2)	11 (26.2)	0 (0)	<0.0001	0.0002
ZHANG TLX TARGETS UP	85	11 (12.9)	11 (12.9)	0 (0)	<0.0001	0.0002
CHICAS RB1 TARGETS SENESCENT	552	10 (1.8)	10 (1.8)	0 (0)	<0.0001	0.0002
CREIGHTON ENDOCRINE THERAPY RESISTANCE 1	500	10 (2)	10 (2)	0 (0)	<0.0001	0.0002
LY AGING OLD DN	56	10 (17.9)	10 (17.9)	0 (0)	<0.0001	0.0002
ODONNELL TARGETS OF MYC AND TFRC DN	44	10 (22.7)	10 (22.7)	0 (0)	<0.0001	0.0002
PUJANA BRCA CENTERED NETWORK	113	10 (8.8)	10 (8.8)	0 (0)	<0.0001	0.0002
VANTVEER BREAST CANCER ESR1 DN	232	10 (4.3)	10 (4.3)	0 (0)	<0.0001	0.0002
CHARAFE BREAST CANCER LUMINAL VS MESENCHYMAL DN	447	9 (2)	9 (2)	0 (0)	<0.0001	0.0002
DACOSTA UV RESPONSE VIA ERCC3 UP	301	9 (3)	8 (2.7)	1 (0.3)	<0.0001	0.0002
FARMER BREAST CANCER APOCRINE VS BASAL	314	9 (2.9)	9 (2.9)	0 (0)	<0.0001	0.0002
FRASOR RESPONSE TO SERM OR FULVESTRANT DN	50	9 (18)	9 (18)	0 (0)	<0.0001	0.0002
GARGALOVIC RESPONSE TO OXIDIZED PHOSPHOLIPIDS TURQUOISE DN	50	9 (18)	9 (18)	0 (0)	<0.0001	0.0002
NAKAMURA TUMOR ZONE PERIPHERAL VS CENTRAL UP	277	9 (3.2)	9 (3.2)	0 (0)	<0.0001	0.0002
YAMAZAKI TCEB3 TARGETS DN	214	9 (4.2)	8 (3.7)	1 (0.5)	<0.0001	0.0002
EGUCHI CELL CYCLE RB1 TARGETS	23	8 (34.8)	8 (34.8)	0 (0)	<0.0001	0.0002
GARY CD5 TARGETS DN	416	8 (1.9)	8 (1.9)	0 (0)	<0.0001	0.0002
GRADE COLON AND RECTAL CANCER UP	279	8 (2.9)	8 (2.9)	0 (0)	<0.0001	0.0002
GREENBAUM E2A TARGETS UP	30	8 (26.7)	8 (26.7)	0 (0)	<0.0001	0.0002
GROSS HYPOXIA VIA ELK3 UP	206	8 (3.9)	8 (3.9)	0 (0)	<0.0001	0.0002
HELLER HDAC TARGETS UP	318	8 (2.5)	7 (2.2)	1 (0.3)	<0.0001	0.0002
MOHANKUMAR TLX1 TARGETS UP	418	8 (1.9)	8 (1.9)	0 (0)	<0.0001	0.0002
PYEON CANCER HEAD AND NECK VS CERVICAL UP	165	8 (4.8)	8 (4.8)	0 (0)	<0.0001	0.0002
RIGGI EWING SARCOMA PROGENITOR DN	185	8 (4.3)	8 (4.3)	0 (0)	<0.0001	0.0002
SONG TARGETS OF IE86 CMV PROTEIN	60	8 (13.3)	8 (13.3)	0 (0)	<0.0001	0.0002
VERNELL RETINOBLASTOMA PATHWAY UP	69	8 (11.6)	8 (11.6)	0 (0)	<0.0001	0.0002
BOHN PRIMARY IMMUNODEFICIENCY SYNDROM UP	47	7 (14.9)	7 (14.9)	0 (0)	<0.0001	0.0002
BOYAULT LIVER CANCER SUBCLASS G3 UP	185	7 (3.8)	7 (3.8)	0 (0)	<0.0001	0.0002
DOANE RESPONSE TO ANDROGEN DN	232	7 (3)	7 (3)	0 (0)	<0.0001	0.0002

Category Name	Category Genes #	Any-reg. SDEG #(%)	Up-reg. SDEG #(%)	Down-reg. SDEG #(%)	P	FDR
GEORGES CELL CYCLE MIR192 TARGETS	61	7 (11.5)	7 (11.5)	0 (0)	<0.0001	0.0002
LY AGING MIDDLE DN	16	7 (43.8)	7 (43.8)	0 (0)	<0.0001	0.0002
PAL PRMT5 TARGETS UP	194	7 (3.6)	7 (3.6)	0 (0)	<0.0001	0.0002
PETROVA PROX1 TARGETS UP	28	7 (25)	6 (21.4)	1 (3.6)	<0.0001	0.0002
PUJANA BREAST CANCER LIT INT NETWORK	97	7 (7.2)	7 (7.2)	0 (0)	<0.0001	0.0002
REN BOUND BY E2F	69	7 (10.1)	7 (10.1)	0 (0)	<0.0001	0.0002
SMID BREAST CANCER LUMINAL A DN	16	7 (43.8)	7 (43.8)	0 (0)	<0.0001	0.0002
WHITFIELD CELL CYCLE G1 S	134	7 (5.2)	7 (5.2)	0 (0)	<0.0001	0.0002
AMUNDSON GENOTOXIC SIGNATURE	101	6 (5.9)	6 (5.9)	0 (0)	<0.0001	0.0002
BROWNE HCMV INFECTION 2HR DN	50	6 (12)	6 (12)	0 (0)	<0.0001	0.0002
FERRANDO HOX11 NEIGHBORS	22	6 (27.3)	6 (27.3)	0 (0)	<0.0001	0.0002
KAMMINGA EZH2 TARGETS	41	6 (14.6)	6 (14.6)	0 (0)	<0.0001	0.0002
KOKKINAKIS METHIONINE DEPRIVATION 96HR DN	73	6 (8.2)	6 (8.2)	0 (0)	<0.0001	0.0002
KUMAMOTO RESPONSE TO NUTLIN 3A DN	10	6 (60)	6 (60)	0 (0)	<0.0001	0.0002
LE NEURONAL DIFFERENTIATION DN	18	6 (33.3)	6 (33.3)	0 (0)	<0.0001	0.0002
LI WILMS TUMOR ANAPLASTIC UP	19	6 (31.6)	6 (31.6)	0 (0)	<0.0001	0.0002
MARKEY RB1 CHRONIC LOF UP	112	6 (5.4)	6 (5.4)	0 (0)	<0.0001	0.0002
MONTERO THYROID CANCER POOR SURVIVAL UP	12	6 (50)	6 (50)	0 (0)	<0.0001	0.0002
NADERI BREAST CANCER PROGNOSIS UP	47	6 (12.8)	6 (12.8)	0 (0)	<0.0001	0.0002
OLSSON E2F3 TARGETS DN	68	6 (8.8)	6 (8.8)	0 (0)	<0.0001	0.0002
PUJANA BREAST CANCER WITH BRCA1 MUTATED UP	55	6 (10.9)	6 (10.9)	0 (0)	<0.0001	0.0002
SCIAN CELL CYCLE TARGETS OF TP53 AND TP73 DN	22	6 (27.3)	6 (27.3)	0 (0)	<0.0001	0.0002
SCIBETTA KDM5B TARGETS DN	75	6 (8)	6 (8)	0 (0)	<0.0001	0.0002
TURASHVILI BREAST DUCTAL CARCINOMA VS LOBULAR NORMAL UP	73	6 (8.2)	6 (8.2)	0 (0)	<0.0001	0.0002
WEST ADRENOCORTICAL TUMOR MARKERS UP	21	6 (28.6)	6 (28.6)	0 (0)	<0.0001	0.0002
BHATI G2M ARREST BY 2METHOXYESTRADIOL UP	128	5 (3.9)	5 (3.9)	0 (0)	<0.0001	0.0002
BHATTACHARYA EMBRYONIC STEM CELL	92	5 (5.4)	5 (5.4)	0 (0)	<0.0001	0.0002
BOYAULT LIVER CANCER SUBCLASS G23 UP	49	5 (10.2)	5 (10.2)	0 (0)	<0.0001	0.0002
CHIARADONNA NEOPLASTIC TRANSFORMATION KRAS UP	117	5 (4.3)	5 (4.3)	0 (0)	<0.0001	0.0002
DANG MYC TARGETS UP	139	5 (3.6)	5 (3.6)	0 (0)	<0.0001	0.0002
DELPUECH FOXO3 TARGETS DN	42	5 (11.9)	5 (11.9)	0 (0)	<0.0001	0.0002
HADDAD T LYMPHOCYTE AND NK PROGENITOR DN	62	5 (8.1)	5 (8.1)	0 (0)	<0.0001	0.0002
HONRADO BREAST CANCER BRCA1 VS BRCA2	19	5 (26.3)	5 (26.3)	0 (0)	<0.0001	0.0002
JAEGER METASTASIS UP	41	5 (12.2)	5 (12.2)	0 (0)	<0.0001	0.0002
JOHANSSON GLIOMAGENESIS BY PDGFB UP	57	5 (8.8)	5 (8.8)	0 (0)	<0.0001	0.0002
KAN RESPONSE TO ARSENIC TRIOXIDE	114	5 (4.4)	5 (4.4)	0 (0)	<0.0001	0.0002
KAUFFMANN MELANOMA RELAPSE UP	61	5 (8.2)	5 (8.2)	0 (0)	<0.0001	0.0002
PUIFFE INVASION INHIBITED BY ASCITES UP	80	5 (6.2)	5 (6.2)	0 (0)	<0.0001	0.0002

Category Name	Category Genes #	Any-reg. SDEG #(%)	Up-reg. SDEG #(%)	Down-reg. SDEG #(%)	P	FDR
RIZ ERYTHROID DIFFERENTIATION	72	5 (6.9)	5 (6.9)	0 (0)	<0.0001	0.0002
STEIN ESR1 TARGETS	80	5 (6.2)	5 (6.2)	0 (0)	<0.0001	0.0002
SU TESTIS	72	5 (6.9)	5 (6.9)	0 (0)	<0.0001	0.0002
YAO TEMPORAL RESPONSE TO PROGESTERONE CLUSTER 15	32	5 (15.6)	5 (15.6)	0 (0)	<0.0001	0.0002
BENPORATH ES 2	37	4 (10.8)	4 (10.8)	0 (0)	<0.0001	0.0002
CAFFAREL RESPONSE TO THC 24HR 5 DN	74	4 (5.4)	4 (5.4)	0 (0)	<0.0001	0.0002
CAFFAREL RESPONSE TO THC DN	32	4 (12.5)	4 (12.5)	0 (0)	<0.0001	0.0002
FINETTI BREAST CANCER KINOME RED	16	4 (25)	4 (25)	0 (0)	<0.0001	0.0002
GENTLES LEUKEMIC STEM CELL DN	18	4 (22.2)	4 (22.2)	0 (0)	<0.0001	0.0002
LY AGING PREMATURE DN	30	4 (13.3)	4 (13.3)	0 (0)	<0.0001	0.0002
NAKAMURA CANCER MICROENVIRONMENT DN	46	4 (8.7)	4 (8.7)	0 (0)	<0.0001	0.0002
STEIN ESRRA TARGETS RESPONSIVE TO ESTROGEN DN	40	4 (10)	4 (10)	0 (0)	<0.0001	0.0002
THILLAINADESAN ZNF217 TARGETS UP	46	4 (8.7)	4 (8.7)	0 (0)	<0.0001	0.0002
XU HGF SIGNALING NOT VIA AKT1 48HR DN	20	4 (20)	4 (20)	0 (0)	<0.0001	0.0002
XU HGF TARGETS INDUCED BY AKT1 48HR DN	30	4 (13.3)	4 (13.3)	0 (0)	<0.0001	0.0002
ZAMORA NOS2 TARGETS UP	66	4 (6.1)	4 (6.1)	0 (0)	<0.0001	0.0002
INAMURA LUNG CANCER SCC UP	14	3 (21.4)	3 (21.4)	0 (0)	<0.0001	0.0002
OHASHI AURKB TARGETS	11	3 (27.3)	3 (27.3)	0 (0)	<0.0001	0.0002
SIMBULAN PARP1 TARGETS DN	17	3 (17.6)	3 (17.6)	0 (0)	<0.0001	0.0002
PILON KLF1 TARGETS DN	1922	16 (0.8)	16 (0.8)	0 (0)	<0.0001	0.0003
DANG BOUND BY MYC	1086	11 (1)	11 (1)	0 (0)	<0.0001	0.0003
NUYTTEN NIPP1 TARGETS DN	813	10 (1.2)	9 (1.1)	1 (0.1)	<0.0001	0.0003
SENESE HDAC3 TARGETS DN	510	9 (1.8)	9 (1.8)	0 (0)	<0.0001	0.0003
IVANOVA HEMATOPOIESIS LATE PROGENITOR	534	8 (1.5)	8 (1.5)	0 (0)	<0.0001	0.0003
MARTINEZ RESPONSE TO TRABECTEDIN DN	276	8 (2.9)	8 (2.9)	0 (0)	<0.0001	0.0003
ZWANG DOWN BY 2ND EGF PULSE	287	7 (2.4)	7 (2.4)	0 (0)	<0.0001	0.0003
DUTERTRE ESTRADIOL RESPONSE 6HR UP	220	6 (2.7)	6 (2.7)	0 (0)	<0.0001	0.0003
LABBE WNT3A TARGETS UP	107	6 (5.6)	6 (5.6)	0 (0)	<0.0001	0.0003
LEE LIVER CANCER SURVIVAL DN	186	6 (3.2)	6 (3.2)	0 (0)	<0.0001	0.0003
LINSLEY MIR16 TARGETS	206	6 (2.9)	6 (2.9)	0 (0)	<0.0001	0.0003
KAUFFMANN DNA REPLICATION GENES	139	5 (3.6)	5 (3.6)	0 (0)	<0.0001	0.0003
MORI MATURE B LYMPHOCYTE DN	72	4 (5.6)	4 (5.6)	0 (0)	<0.0001	0.0003
SESTO RESPONSE TO UV C7	67	4 (6)	4 (6)	0 (0)	<0.0001	0.0003
SUNG METASTASIS STROMA DN	53	4 (7.5)	4 (7.5)	0 (0)	<0.0001	0.0003
MARKS HDAC TARGETS DN	14	3 (21.4)	3 (21.4)	0 (0)	<0.0001	0.0003
SEMBA FHIT TARGETS DN	10	3 (30)	3 (30)	0 (0)	<0.0001	0.0003
ACEVEDO FGFR1 TARGETS IN PROSTATE CANCER MODEL UP	278	6 (2.2)	6 (2.2)	0 (0)	<0.0001	0.0004
BORCZUK MALIGNANT MESOTHELIOMA UP	282	6 (2.1)	6 (2.1)	0 (0)	<0.0001	0.0004

Category Name	Category Genes #	Any-reg. SDEG #(%)	Up-reg. SDEG #(%)	Down-reg. SDEG #(%)	P	FDR
KATSANOU ELAVL1 TARGETS DN	144	5 (3.5)	5 (3.5)	0 (0)	<0.0001	0.0004
PYEON HPV POSITIVE TUMORS UP	91	5 (5.5)	5 (5.5)	0 (0)	<0.0001	0.0004
MCBRYAN PUBERTAL BREAST 6 7WK DN	77	4 (5.2)	4 (5.2)	0 (0)	<0.0001	0.0004
WEIGEL OXIDATIVE STRESS BY HNE AND H2O2	36	3 (8.3)	3 (8.3)	0 (0)	<0.0001	0.0004
DAZARD RESPONSE TO UV NHEK DN	313	7 (2.2)	7 (2.2)	0 (0)	<0.0001	0.0006
SPIELMAN LYMPHOBLAST EUROPEAN VS ASIAN UP	464	7 (1.5)	7 (1.5)	0 (0)	<0.0001	0.0006
PLASARI TGFB1 TARGETS 10HR DN	233	6 (2.6)	6 (2.6)	0 (0)	<0.0001	0.0006
FOURNIER ACINAR DEVELOPMENT LATE DN	22	3 (13.6)	3 (13.6)	0 (0)	<0.0001	0.0006
BROWNE HCMV INFECTION 30MIN DN	144	5 (3.5)	5 (3.5)	0 (0)	<0.0001	0.0007
DAZARD UV RESPONSE CLUSTER G6	149	5 (3.4)	5 (3.4)	0 (0)	<0.0001	0.0007
FERRANDO T ALL WITH MLL ENL FUSION DN	97	4 (4.1)	4 (4.1)	0 (0)	<0.0001	0.0007
KOKKINAKIS METHIONINE DEPRIVATION 48HR DN	62	4 (6.5)	4 (6.5)	0 (0)	<0.0001	0.0007
HU GENOTOXIC DAMAGE 4HR	36	3 (8.3)	3 (8.3)	0 (0)	<0.0001	0.0007
KORKOLA TERATOMA	40	3 (7.5)	3 (7.5)	0 (0)	<0.0001	0.0007
NUNODA RESPONSE TO DASATINIB IMATINIB UP	29	3 (10.3)	3 (10.3)	0 (0)	<0.0001	0.0007
WANG METASTASIS OF BREAST CANCER ESR1 UP	35	3 (8.6)	3 (8.6)	0 (0)	<0.0001	0.0007
WILLIAMS ESR1 TARGETS UP	26	3 (11.5)	3 (11.5)	0 (0)	<0.0001	0.0007
LOPEZ MBD TARGETS	925	10 (1.1)	9 (1)	1 (0.1)	<0.0001	0.0008
ACEVEDO LIVER TUMOR VS NORMAL ADJACENT TISSUE UP	850	9 (1.1)	9 (1.1)	0 (0)	<0.0001	0.0008
BENPORATH SOX2 TARGETS	735	9 (1.2)	9 (1.2)	0 (0)	<0.0001	0.0008
BOQUEST STEM CELL CULTURED VS FRESH UP	409	7 (1.7)	6 (1.5)	1 (0.2)	<0.0001	0.0008
LASTOWSKA NEUROBLASTOMA COPY NUMBER UP	176	5 (2.8)	5 (2.8)	0 (0)	<0.0001	0.0008
LI WILMS TUMOR VS FETAL KIDNEY 2 UP	30	3 (10)	3 (10)	0 (0)	<0.0001	0.0008
MATSUDA NATURAL KILLER DIFFERENTIATION	463	7 (1.5)	7 (1.5)	0 (0)	<0.0001	0.0009
FARMER BREAST CANCER BASAL VS LULMINAL	316	6 (1.9)	6 (1.9)	0 (0)	<0.0001	0.0009
SMID BREAST CANCER RELAPSE IN BRAIN UP	35	3 (8.6)	3 (8.6)	0 (0)	<0.0001	0.0009
YU BAP1 TARGETS	29	3 (10.3)	3 (10.3)	0 (0)	<0.0001	0.0009
SANSOM APC TARGETS REQUIRE MYC	207	5 (2.4)	5 (2.4)	0 (0)	<0.0001	0.001
SHETH LIVER CANCER VS TXNIP LOSS PAM1	217	5 (2.3)	5 (2.3)	0 (0)	<0.0001	0.001
BOSCO TH1 CYTOTOXIC MODULE	114	4 (3.5)	4 (3.5)	0 (0)	<0.0001	0.0012
BENPORATH NANOG TARGETS	974	10 (1)	10 (1)	0 (0)	<0.0001	0.0013
HERNANDEZ MITOTIC ARREST BY DOCETAXEL 1 DN	51	3 (5.9)	3 (5.9)	0 (0)	<0.0001	0.0013
CROSBY E2F4 TARGETS	6	2 (33.3)	2 (33.3)	0 (0)	<0.0001	0.0013
MORI EMU MYC LYMPHOMA BY ONSET TIME UP	107	4 (3.7)	4 (3.7)	0 (0)	0.0001	0.0014
FAELT B CLL WITH VH3 21 UP	41	3 (7.3)	3 (7.3)	0 (0)	0.0001	0.0014
KUNINGER IGF1 VS PDGFB TARGETS DN	43	3 (7)	3 (7)	0 (0)	0.0001	0.0014
GLINSKY CANCER DEATH UP	6	2 (33.3)	2 (33.3)	0 (0)	0.0001	0.0014
BONCI TARGETS OF MIR15A AND MIR16 1	93	4 (4.3)	4 (4.3)	0 (0)	0.0001	0.0015

Category Name	Category Genes #	Any-reg. SDEG #(%)	Up-reg. SDEG #(%)	Down-reg. SDEG #(%)	P	FDR
KRASNOSELSKAYA ILF3 TARGETS DN	43	3 (7)	3 (7)	0 (0)	0.0001	0.0015
SMITH LIVER CANCER	43	3 (7)	3 (7)	0 (0)	0.0001	0.0016
GRAHAM CML QUIESCENT VS CML DIVIDING DN	11	2 (18.2)	2 (18.2)	0 (0)	0.0001	0.0016
LIANG SILENCED BY METHYLATION DN	10	2 (20)	2 (20)	0 (0)	0.0001	0.0016
REICHERT G1S REGULATORS AS PI3K TARGETS	8	2 (25)	2 (25)	0 (0)	0.0001	0.0016
BURTON ADIPOGENESIS PEAK AT 16HR	38	3 (7.9)	3 (7.9)	0 (0)	0.0001	0.0017
WONG PROTEASOME GENE MODULE	48	3 (6.2)	3 (6.2)	0 (0)	0.0001	0.0017
KIM WT1 TARGETS 8HR DN	123	4 (3.3)	3 (2.4)	1 (0.8)	0.0001	0.0018
LEE LIVER CANCER MYC UP	53	3 (5.7)	3 (5.7)	0 (0)	0.0001	0.0018
JIANG VHL TARGETS	135	4 (3)	4 (3)	0 (0)	0.0002	0.0023
KRIEG HYPOXIA VIA KDM3A	51	3 (5.9)	3 (5.9)	0 (0)	0.0002	0.0023
TURASHVILI BREAST DUCTAL CARCINOMA VS DUCTAL NORMAL UP	41	3 (7.3)	3 (7.3)	0 (0)	0.0002	0.0023
ACEVEDO LIVER CANCER UP	947	9 (1)	9 (1)	0 (0)	0.0002	0.0024
GRUETZMANN PANCREATIC CANCER UP	351	6 (1.7)	6 (1.7)	0 (0)	0.0002	0.0024
PRAMOONJAGO SOX4 TARGETS DN	51	3 (5.9)	3 (5.9)	0 (0)	0.0002	0.0024
KONG E2F1 TARGETS	10	2 (20)	2 (20)	0 (0)	0.0002	0.0025
KALMA E2F1 TARGETS	11	2 (18.2)	2 (18.2)	0 (0)	0.0002	0.0026
ZHU SKIL TARGETS DN	9	2 (22.2)	2 (22.2)	0 (0)	0.0002	0.0026
GRAESSMANN APOPTOSIS BY DOXORUBICIN DN	1714	13 (0.8)	13 (0.8)	0 (0)	0.0002	0.0027
LEE LIVER CANCER MYC E2F1 UP	56	3 (5.4)	3 (5.4)	0 (0)	0.0002	0.0027
KOKKINAKIS METHIONINE DEPRIVATION 48HR UP	126	4 (3.2)	4 (3.2)	0 (0)	0.0002	0.0028
LOPEZ MESOTELIOMA SURVIVAL TIME UP	12	2 (16.7)	2 (16.7)	0 (0)	0.0002	0.0028
RHODES CANCER META SIGNATURE	61	3 (4.9)	3 (4.9)	0 (0)	0.0003	0.0031
BILD E2F3 ONCOGENIC SIGNATURE	236	5 (2.1)	4 (1.7)	1 (0.4)	0.0003	0.0033
CHIANG LIVER CANCER SUBCLASS UNANNOTATED DN	177	4 (2.3)	4 (2.3)	0 (0)	0.0003	0.0034
BORLAK LIVER CANCER EGF UP	55	3 (5.5)	3 (5.5)	0 (0)	0.0003	0.0034
CUI GLUCOSE DEPRIVATION	70	3 (4.3)	3 (4.3)	0 (0)	0.0003	0.0034
NEBEN AML WITH FLT3 OR NRAS DN	12	2 (16.7)	2 (16.7)	0 (0)	0.0003	0.0038
PEART HDAC PROLIFERATION CLUSTER UP	56	3 (5.4)	3 (5.4)	0 (0)	0.0004	0.0042
WU APOPTOSIS BY CDKN1A NOT VIA TP53	12	2 (16.7)	2 (16.7)	0 (0)	0.0004	0.0045
ONKEN UVEAL MELANOMA UP	763	8 (1)	8 (1)	0 (0)	0.0005	0.0051
PENG GLUCOSE DEPRIVATION DN	162	4 (2.5)	4 (2.5)	0 (0)	0.0005	0.0053
CAIRO HEPATOBLASTOMA POOR SURVIVAL	14	2 (14.3)	2 (14.3)	0 (0)	0.0005	0.0057
DANG REGULATED BY MYC UP	72	3 (4.2)	3 (4.2)	0 (0)	0.0005	0.006
SCHWAB TARGETS OF BMYB POLYMORPHIC VARIANTS UP	12	2 (16.7)	2 (16.7)	0 (0)	0.0005	0.0061
FERNANDEZ BOUND BY MYC	174	4 (2.3)	4 (2.3)	0 (0)	0.0006	0.0061
KOINUMA COLON CANCER MSI DN	16	2 (12.5)	2 (12.5)	0 (0)	0.0006	0.0061
BAKER HEMATOPOIESIS STAT3 TARGETS	15	2 (13.3)	2 (13.3)	0 (0)	0.0006	0.0067

Category Name	Category Genes #	Any-reg. SDEG #(%)	Up-reg. SDEG #(%)	Down-reg. SDEG #(%)	P	FDR
CHUANG OXIDATIVE STRESS RESPONSE DN	24	2 (8.3)	2 (8.3)	0 (0)	0.0006	0.0068
KARAKAS TGFB1 SIGNALING	18	2 (11.1)	2 (11.1)	0 (0)	0.0007	0.0073
SHEN SMARCA2 TARGETS DN	329	5 (1.5)	5 (1.5)	0 (0)	0.0007	0.0074
SLEBOS HEAD AND NECK CANCER WITH HPV UP	79	3 (3.8)	3 (3.8)	0 (0)	0.0007	0.0077
CHICAS RB1 TARGETS LOW SERUM	106	3 (2.8)	3 (2.8)	0 (0)	0.0007	0.0078
CAFFAREL RESPONSE TO THC 24HR 3 DN	26	2 (7.7)	2 (7.7)	0 (0)	0.0008	0.0084
WELCSH BRCA1 TARGETS UP	193	4 (2.1)	4 (2.1)	0 (0)	0.0008	0.0084
WANG CISPLATIN RESPONSE AND XPC UP	189	4 (2.1)	4 (2.1)	0 (0)	0.0008	0.0086
MEINHOLD OVARIAN CANCER LOW GRADE DN	20	2 (10)	2 (10)	0 (0)	0.0008	0.0086
CHEOK RESPONSE TO MERCAPTOPURINE DN	20	2 (10)	2 (10)	0 (0)	0.0008	0.0089
WIELAND UP BY HBV INFECTION	100	3 (3)	3 (3)	0 (0)	0.0009	0.0091
JEON SMAD6 TARGETS DN	19	2 (10.5)	2 (10.5)	0 (0)	0.0009	0.0092
BENPORATH NOS TARGETS	178	4 (2.2)	4 (2.2)	0 (0)	0.0009	0.0092
WANG LSD1 TARGETS UP	23	2 (8.7)	1 (4.3)	1 (4.3)	0.0009	0.0097

Table A103: Significantly enriched MSigDB Chemical/Genetic Perturbation Sets (RNA-Seq, NK-cells, Day 3). Results sorted by FDR and #SDEG genes.

Category Name	Category Genes #	Any-reg. SDEG #(%)	Up-reg. SDEG #(%)	Down-reg. SDEG #(%)	P	FDR
Tuberculosis	159	10 (6.3)	0 (0)	10 (6.3)	<0.0001	0.0002
Antigen processing and presentation	64	7 (10.9)	0 (0)	7 (10.9)	<0.0001	0.0002
Toxoplasmosis	101	7 (6.9)	0 (0)	7 (6.9)	<0.0001	0.0002
Influenza A	149	7 (4.7)	0 (0)	7 (4.7)	<0.0001	0.0002
Inflammatory bowel disease (IBD)	53	7 (13.2)	0 (0)	7 (13.2)	<0.0001	0.0002
Phagosome	131	6 (4.6)	0 (0)	6 (4.6)	<0.0001	0.0002
Cell adhesion molecules (CAMs)	123	6 (4.9)	0 (0)	6 (4.9)	<0.0001	0.0002
Leishmaniasis	60	6 (10)	0 (0)	6 (10)	<0.0001	0.0002
Herpes simplex infection	154	6 (3.9)	0 (0)	6 (3.9)	<0.0001	0.0002
Asthma	23	6 (26.1)	0 (0)	6 (26.1)	<0.0001	0.0002
Rheumatoid arthritis	77	6 (7.8)	0 (0)	6 (7.8)	<0.0001	0.0002
Intestinal immune network for IgA production	37	5 (13.5)	0 (0)	5 (13.5)	<0.0001	0.0002
Type I diabetes mellitus	37	5 (13.5)	0 (0)	5 (13.5)	<0.0001	0.0002
Staphylococcus aureus infection	47	5 (10.6)	0 (0)	5 (10.6)	<0.0001	0.0002
Autoimmune thyroid disease	45	5 (11.1)	0 (0)	5 (11.1)	<0.0001	0.0002
Allograft rejection	31	5 (16.1)	0 (0)	5 (16.1)	<0.0001	0.0002
Graft-versus-host disease	33	5 (15.2)	0 (0)	5 (15.2)	<0.0001	0.0002
Viral myocarditis	51	5 (9.8)	0 (0)	5 (9.8)	<0.0001	0.0002
Systemic lupus erythematosus	106	5 (4.7)	0 (0)	5 (4.7)	<0.0001	0.0003

Category Name	Category Genes #	Any-reg. SDEG #(%)	Up-reg. SDEG #(%)	Down-reg. SDEG #(%)	P	FDR
Hematopoietic cell lineage	74	4 (5.4)	0 (0)	4 (5.4)	<0.0001	0.0003
HTLV-I infection	229	5 (2.2)	0 (0)	5 (2.2)	0.0001	0.0016
Epstein-Barr virus infection	176	4 (2.3)	0 (0)	4 (2.3)	0.0004	0.0051
Arrhythmogenic right ventricular cardiomyopathy (ARVC)	64	3 (4.7)	0 (0)	3 (4.7)	0.0004	0.0051

Table A104: Significantly enriched KEGG Pathways (RNA-Seq, NK-cells, Day 28). Results sorted by FDR and #SDEG genes.

Category Name	Category Genes #	Any-reg. SDEG #(%)	Up-reg. SDEG #(%)	Down-reg. SDEG #(%)	P	FDR
SIGNAL TRANSDUCTION	1580	12 (0.8)	0 (0)	12 (0.8)	<0.0001	0.0082

Table A105: Significantly enriched MSigDB GO Biological Processes (RNA-Seq, NK-cells, Day 28). Results sorted by FDR and #SDEG genes.

Category Name	Category Genes #	Any-reg. SDEG #(%)	Up-reg. SDEG #(%)	Down-reg. SDEG #(%)	P	FDR
MEMBRANE	1902	16 (0.8)	0 (0)	16 (0.8)	<0.0001	0.0012
PLASMA MEMBRANE	1358	14 (1)	0 (0)	14 (1)	<0.0001	0.0012
INTEGRAL TO MEMBRANE	1272	11 (0.9)	0 (0)	11 (0.9)	<0.0001	0.0023
INTRINSIC TO MEMBRANE	1289	11 (0.9)	0 (0)	11 (0.9)	<0.0001	0.0023
INTEGRAL TO PLASMA MEMBRANE	930	9 (1)	0 (0)	9 (1)	0.0001	0.0062
INTRINSIC TO PLASMA MEMBRANE	943	9 (1)	0 (0)	9 (1)	0.0002	0.0062
MEMBRANE PART	1597	11 (0.7)	0 (0)	11 (0.7)	0.0002	0.0073

Table A106: Significantly enriched MSigDB GO Cellular Components (RNA-Seq, NK-cells, Day 28). Results sorted by FDR and #SDEG genes.

Category Name	Category Genes #	Any-reg. SDEG #(%)	Up-reg. SDEG #(%)	Down-reg. SDEG #(%)	P	FDR
RECEPTOR ACTIVITY	554	8 (1.4)	0 (0)	8 (1.4)	<0.0001	0.004

Table A107: Significantly enriched MSigDB GO Molecular Functions (RNA-Seq, NK-cells, Day 28). Results sorted by FDR and #SDEG genes.

Category Name	Category Genes #	Any-reg. SDEG #(%)	Up-reg. SDEG #(%)	Down-reg. SDEG #(%)	P	FDR
BIOCARTA TH1TH2 PATHWAY	18	3 (16.7)	0 (0)	3 (16.7)	<0.0001	0.0022
BIOCARTA IL5 PATHWAY	10	2 (20)	0 (0)	2 (20)	<0.0001	0.0081

Category Name	Category Genes #	Any-reg. SDEG #(%)	Up-reg. SDEG #(%)	Down-reg. SDEG #(%)	P	FDR
BIOCARTA ASBCELL PATHWAY	11	2 (18.2)	0 (0)	2 (18.2)	0.0001	0.0081
BIOCARTA TCRA PATHWAY	11	2 (18.2)	0 (0)	2 (18.2)	0.0001	0.0081

Table A108: Significantly enriched MSigDB BioCarta Pathways (RNA-Seq, NK-cells, Day 28). Results sorted by FDR and #SDEG genes.

Category Name	Category Genes #	Any-reg. SDEG #(%)	Up-reg. SDEG #(%)	Down-reg. SDEG #(%)	P	FDR
CHEN METABOLIC SYNDROM NETWORK	1172	17 (1.5)	0 (0)	17 (1.5)	<0.0001	0.001
RODWELL AGING KIDNEY UP	465	14 (3)	0 (0)	14 (3)	<0.0001	0.001
HELLER SILENCED BY METHYLATION UP	273	10 (3.7)	0 (0)	10 (3.7)	<0.0001	0.001
RUTELLA RESPONSE TO CSF2RB AND IL4 DN	303	10 (3.3)	0 (0)	10 (3.3)	<0.0001	0.001
WANG RESPONSE TO GSK3 INHIBITOR SB216763 UP	378	10 (2.6)	0 (0)	10 (2.6)	<0.0001	0.001
RUTELLA RESPONSE TO HGF VS CSF2RB AND IL4 UP	391	9 (2.3)	0 (0)	9 (2.3)	<0.0001	0.001
SCHUETZ BREAST CANCER DUCTAL INVASIVE UP	338	9 (2.7)	0 (0)	9 (2.7)	<0.0001	0.001
CHARAFE BREAST CANCER LUMINAL VS MESENCHYMAL DN	447	8 (1.8)	0 (0)	8 (1.8)	<0.0001	0.001
HELLER HDAC TARGETS SILENCED BY METHYLATION UP	439	8 (1.8)	0 (0)	8 (1.8)	<0.0001	0.001
JOHNSTONE PARVB TARGETS 3 UP	425	8 (1.9)	0 (0)	8 (1.9)	<0.0001	0.001
SMID BREAST CANCER NORMAL LIKE UP	443	8 (1.8)	0 (0)	8 (1.8)	<0.0001	0.001
LI INDUCED T TO NATURAL KILLER UP	302	7 (2.3)	0 (0)	7 (2.3)	<0.0001	0.001
MCLACHLAN DENTAL CARIES DN	219	7 (3.2)	0 (0)	7 (3.2)	<0.0001	0.001
MCLACHLAN DENTAL CARIES UP	227	7 (3.1)	0 (0)	7 (3.1)	<0.0001	0.001
RUTELLA RESPONSE TO HGF DN	224	7 (3.1)	0 (0)	7 (3.1)	<0.0001	0.001
SWEET LUNG CANCER KRAS UP	482	7 (1.5)	0 (0)	7 (1.5)	<0.0001	0.001
YAGI AML WITH T 8 21 TRANSLOCATION	349	7 (2)	0 (0)	7 (2)	<0.0001	0.001
BEIER GLIOMA STEM CELL DN	63	6 (9.5)	0 (0)	6 (9.5)	<0.0001	0.001
BROWN MYELOID CELL DEVELOPMENT UP	159	6 (3.8)	0 (0)	6 (3.8)	<0.0001	0.001
DIAZ CHRONIC MEYLOGENOUS LEUKEMIA DN	112	6 (5.4)	0 (0)	6 (5.4)	<0.0001	0.001
GAL LEUKEMIC STEM CELL DN	244	6 (2.5)	0 (0)	6 (2.5)	<0.0001	0.001
WIELAND UP BY HBV INFECTION	100	6 (6)	0 (0)	6 (6)	<0.0001	0.001
GAURNIER PSMD4 TARGETS	68	5 (7.4)	0 (0)	5 (7.4)	<0.0001	0.001
HOFFMANN SMALL PRE BII TO IMMATURE B LYMPHOCYTE UP	70	5 (7.1)	0 (0)	5 (7.1)	<0.0001	0.001
ICHIBA GRAFT VERSUS HOST DISEASE D7 UP	105	5 (4.8)	0 (0)	5 (4.8)	<0.0001	0.001
NAKAYAMA SOFT TISSUE TUMORS PCA1 UP	69	5 (7.2)	0 (0)	5 (7.2)	<0.0001	0.001
GRAHAM CML QUIESCENT VS NORMAL QUIESCENT DN	45	4 (8.9)	0 (0)	4 (8.9)	<0.0001	0.001
KIM GLIS2 TARGETS UP	84	4 (4.8)	0 (0)	4 (4.8)	<0.0001	0.001
LIAN LIPA TARGETS 6M	70	4 (5.7)	0 (0)	4 (5.7)	<0.0001	0.001
MORI IMMATURE B LYMPHOCYTE UP	52	4 (7.7)	0 (0)	4 (7.7)	<0.0001	0.001
SANA RESPONSE TO IFNG UP	74	4 (5.4)	0 (0)	4 (5.4)	<0.0001	0.001

Category Name	Category Genes #	Any-reg. SDEG #(%)	Up-reg. SDEG #(%)	Down-reg. SDEG #(%)	P	FDR
YU MYC TARGETS DN	51	4 (7.8)	0 (0)	4 (7.8)	<0.0001	0.001
LUI THYROID CANCER CLUSTER 4	13	3 (23.1)	0 (0)	3 (23.1)	<0.0001	0.001
VERHAAK AML WITH NPM1 MUTATED DN	238	6 (2.5)	0 (0)	6 (2.5)	<0.0001	0.0017
ZHANG TLX TARGETS 60HR UP	289	6 (2.1)	0 (0)	6 (2.1)	<0.0001	0.0017
ALCALAY AML BY NPM1 LOCALIZATION DN	178	5 (2.8)	0 (0)	5 (2.8)	<0.0001	0.0017
YAGI AML FAB MARKERS	178	5 (2.8)	0 (0)	5 (2.8)	<0.0001	0.0017
BURTON ADIPOGENESIS 9	88	4 (4.5)	0 (0)	4 (4.5)	<0.0001	0.0017
ICHIBA GRAFT VERSUS HOST DISEASE 35D UP	126	4 (3.2)	0 (0)	4 (3.2)	<0.0001	0.0017
MORI MATURE B LYMPHOCYTE UP	89	4 (4.5)	0 (0)	4 (4.5)	<0.0001	0.0017
FULCHER INFLAMMATORY RESPONSE LECTIN VS LPS DN	462	7 (1.5)	0 (0)	7 (1.5)	<0.0001	0.0032
VALK AML CLUSTER 5	30	3 (10)	0 (0)	3 (10)	<0.0001	0.0032
KOINUMA TARGETS OF SMAD2 OR SMAD3	831	9 (1.1)	0 (0)	9 (1.1)	<0.0001	0.0035
GOLDRATH ANTIGEN RESPONSE	333	6 (1.8)	0 (0)	6 (1.8)	<0.0001	0.0035
HELLER HDAC TARGETS UP	318	6 (1.9)	0 (0)	6 (1.9)	<0.0001	0.0035
HOSHIDA LIVER CANCER SUBCLASS S1	225	5 (2.2)	0 (0)	5 (2.2)	<0.0001	0.0035
LENAOUR DENDRITIC CELL MATURATION UP	112	4 (3.6)	0 (0)	4 (3.6)	<0.0001	0.0035
TARTE PLASMA CELL VS B LYMPHOCYTE DN	36	3 (8.3)	0 (0)	3 (8.3)	<0.0001	0.0035
WINNEPENNINCKX MELANOMA METASTASIS DN	43	3 (7)	0 (0)	3 (7)	<0.0001	0.0035
LEE BMP2 TARGETS UP	723	8 (1.1)	0 (0)	8 (1.1)	<0.0001	0.0037
CHYLA CBFA2T3 TARGETS UP	372	6 (1.6)	0 (0)	6 (1.6)	<0.0001	0.0037
GRAHAM CML DIVIDING VS NORMAL QUIESCENT DN	88	4 (4.5)	0 (0)	4 (4.5)	<0.0001	0.0037
CASORELLI APL SECONDARY VS DE NOVO UP	39	3 (7.7)	0 (0)	3 (7.7)	<0.0001	0.0037
ROSS AML WITH CBFB MYH11 FUSION	48	3 (6.2)	0 (0)	3 (6.2)	<0.0001	0.0037
ZHAN V1 LATE DIFFERENTIATION GENES UP	32	3 (9.4)	0 (0)	3 (9.4)	<0.0001	0.0037
REN ALVEOLAR RHABDOMYOSARCOMA DN	378	6 (1.6)	0 (0)	6 (1.6)	<0.0001	0.0042
JAATINEN HEMATOPOIETIC STEM CELL DN	221	5 (2.3)	0 (0)	5 (2.3)	<0.0001	0.0042
IVANOVA HEMATOPOIESIS LATE PROGENITOR	534	7 (1.3)	0 (0)	7 (1.3)	<0.0001	0.0046
GAZDA DIAMOND BLACKFAN ANEMIA PROGENITOR UP	40	3 (7.5)	0 (0)	3 (7.5)	<0.0001	0.0046
BOQUEST STEM CELL DN	213	5 (2.3)	0 (0)	5 (2.3)	<0.0001	0.0049
BOYLAN MULTIPLE MYELOMA C D DN	244	5 (2)	0 (0)	5 (2)	<0.0001	0.0049
CHANG CORE SERUM RESPONSE DN	205	5 (2.4)	0 (0)	5 (2.4)	<0.0001	0.0049
BROWN MYELOID CELL DEVELOPMENT DN	126	4 (3.2)	0 (0)	4 (3.2)	<0.0001	0.0049
SMIRNOV CIRCULATING ENDOTHELIOCYTES IN CANCER UP	152	4 (2.6)	0 (0)	4 (2.6)	0.0001	0.0058
ONKEN UVEAL MELANOMA UP	763	8 (1)	0 (0)	8 (1)	0.0001	0.0062
FULCHER INFLAMMATORY RESPONSE LECTIN VS LPS UP	564	7 (1.2)	0 (0)	7 (1.2)	0.0001	0.0062
KLEIN PRIMARY EFFUSION LYMPHOMA DN	54	3 (5.6)	0 (0)	3 (5.6)	0.0001	0.0064
LIAN LIPA TARGETS 3M	56	3 (5.4)	0 (0)	3 (5.4)	0.0001	0.0064
BUDHU LIVER CANCER METASTASIS UP	10	2 (20)	0 (0)	2 (20)	0.0001	0.0064

Category Name	Category Genes #	Any-reg. SDEG #(%)	Up-reg. SDEG #(%)	Down-reg. SDEG #(%)	P	FDR
MORI LARGE PRE BII LYMPHOCYTE DN	56	3 (5.4)	0 (0)	3 (5.4)	0.0001	0.0067
WUNDER INFLAMMATORY RESPONSE AND CHOLESTEROL UP	61	3 (4.9)	0 (0)	3 (4.9)	0.0001	0.0067
TONKS TARGETS OF RUNX1 RUNX1T1 FUSION ERYTHROCYTE UP	151	4 (2.6)	0 (0)	4 (2.6)	0.0001	0.0071
JISON SICKLE CELL DISEASE UP	174	4 (2.3)	0 (0)	4 (2.3)	0.0002	0.0092
ZHONG SECRETOME OF LUNG CANCER AND MACROPHAGE	75	3 (4)	0 (0)	3 (4)	0.0002	0.0092
DURAND STROMA MAX UP	270	5 (1.9)	0 (0)	5 (1.9)	0.0002	0.0095
VERHAAK AML WITH NPM1 MUTATED UP	190	4 (2.1)	0 (0)	4 (2.1)	0.0002	0.0095
FURUKAWA DUSP6 TARGETS PCI35 UP	73	3 (4.1)	0 (0)	3 (4.1)	0.0002	0.0095
LINDSTEDT DENDRITIC CELL MATURATION D	65	3 (4.6)	0 (0)	3 (4.6)	0.0002	0.0095
GUTIERREZ WALDENSTROEMS MACROGLOBULINEMIA 2	13	2 (15.4)	0 (0)	2 (15.4)	0.0002	0.0095
SERVITJA ISLET HNF1A TARGETS UP	160	4 (2.5)	0 (0)	4 (2.5)	0.0002	0.0098

Table A109: Significantly enriched MSigDB Chemical/Genetic Perturbation Sets (RNA-Seq, NK-cells, Day 28). Results sorted by FDR and #SDEG genes.

Category Name	Category Genes #	Any-reg. SDEG #(%)	Up-reg. SDEG #(%)	Down-reg. SDEG #(%)	P	FDR
Influenza A	149	5 (3.4)	5 (3.4)	0 (0)	<0.0001	0.0019
TNF signaling pathway	102	4 (3.9)	4 (3.9)	0 (0)	<0.0001	0.0019
Hepatitis C	117	4 (3.4)	4 (3.4)	0 (0)	<0.0001	0.0019
Prolactin signaling pathway	67	3 (4.5)	3 (4.5)	0 (0)	<0.0001	0.0064
Rheumatoid arthritis	77	3 (3.9)	3 (3.9)	0 (0)	0.0001	0.0074

Table A110: Significantly enriched KEGG Pathways (RNA-Seq, T-cells, Day 1). Results sorted by FDR and #SDEG genes.

Category Name	Category Genes #	Any-reg. SDEG #(%)	Up-reg. SDEG #(%)	Down-reg. SDEG #(%)	P	FDR
NUYTTEN EZH2 TARGETS UP	1002	18 (1.8)	18 (1.8)	0 (0)	<0.0001	0.0005
FULCHER INFLAMMATORY RESPONSE LECTIN VS LPS DN	462	15 (3.2)	15 (3.2)	0 (0)	<0.0001	0.0005
HECKER IFNB1 TARGETS	92	15 (16.3)	15 (16.3)	0 (0)	<0.0001	0.0005
TAKEDA TARGETS OF NUP98 HOXA9 FUSION 3D UP	178	14 (7.9)	14 (7.9)	0 (0)	<0.0001	0.0005
BERTUCCI MEDULLARY VS DUCTAL BREAST CANCER UP	200	13 (6.5)	13 (6.5)	0 (0)	<0.0001	0.0005
BROWNE INTERFERON RESPONSIVE GENES	68	13 (19.1)	13 (19.1)	0 (0)	<0.0001	0.0005
TAKEDA TARGETS OF NUP98 HOXA9 FUSION 10D UP	186	13 (7)	13 (7)	0 (0)	<0.0001	0.0005
WALLACE PROSTATE CANCER RACE UP	275	13 (4.7)	13 (4.7)	0 (0)	<0.0001	0.0005
NUYTTEN NIPP1 TARGETS UP	748	12 (1.6)	12 (1.6)	0 (0)	<0.0001	0.0005
SANA RESPONSE TO IFNG UP	74	12 (16.2)	12 (16.2)	0 (0)	<0.0001	0.0005
BOSCO INTERFERON INDUCED ANTIVIRAL MODULE	76	11 (14.5)	11 (14.5)	0 (0)	<0.0001	0.0005

Category Name	Category Genes #	Any-reg. SDEG #(%)	Up-reg. SDEG #(%)	Down-reg. SDEG #(%)	P	FDR
RODWELL AGING KIDNEY UP	465	11 (2.4)	11 (2.4)	0 (0)	<0.0001	0.0005
GRAESSMANN APOPTOSIS BY SERUM DEPRIVATION UP	541	10 (1.8)	10 (1.8)	0 (0)	<0.0001	0.0005
GRAESSMANN RESPONSE TO MC AND SERUM DEPRIVATION UP	203	10 (4.9)	10 (4.9)	0 (0)	<0.0001	0.0005
JISON SICKLE CELL DISEASE UP	174	10 (5.7)	10 (5.7)	0 (0)	<0.0001	0.0005
MOSERLE IFNA RESPONSE	31	10 (32.3)	10 (32.3)	0 (0)	<0.0001	0.0005
YANG BCL3 TARGETS UP	351	10 (2.8)	10 (2.8)	0 (0)	<0.0001	0.0005
CHICAS RB1 TARGETS CONFLUENT	550	9 (1.6)	9 (1.6)	0 (0)	<0.0001	0.0005
DEBIASI APOPTOSIS BY REOVIRUS INFECTION UP	317	9 (2.8)	9 (2.8)	0 (0)	<0.0001	0.0005
FEVR CTNNB1 TARGETS UP	672	9 (1.3)	9 (1.3)	0 (0)	<0.0001	0.0005
HELLER SILENCED BY METHYLATION UP	273	9 (3.3)	9 (3.3)	0 (0)	<0.0001	0.0005
ICHIBA GRAFT VERSUS HOST DISEASE D7 UP	105	9 (8.6)	9 (8.6)	0 (0)	<0.0001	0.0005
MARKEY RB1 ACUTE LOF UP	214	9 (4.2)	9 (4.2)	0 (0)	<0.0001	0.0005
TAKEDA TARGETS OF NUP98 HOXA9 FUSION 16D UP	169	9 (5.3)	9 (5.3)	0 (0)	<0.0001	0.0005
TAKEDA TARGETS OF NUP98 HOXA9 FUSION 8D UP	155	9 (5.8)	9 (5.8)	0 (0)	<0.0001	0.0005
DER IFN ALPHA RESPONSE UP	74	8 (10.8)	8 (10.8)	0 (0)	<0.0001	0.0005
DER IFN BETA RESPONSE UP	101	8 (7.9)	8 (7.9)	0 (0)	<0.0001	0.0005
EINAV INTERFERON SIGNATURE IN CANCER	27	8 (29.6)	8 (29.6)	0 (0)	<0.0001	0.0005
FARMER BREAST CANCER CLUSTER 1	39	8 (20.5)	8 (20.5)	0 (0)	<0.0001	0.0005
KRIEG KDM3A TARGETS NOT HYPOXIA	189	8 (4.2)	8 (4.2)	0 (0)	<0.0001	0.0005
RADAEVA RESPONSE TO IFNA1 UP	52	8 (15.4)	8 (15.4)	0 (0)	<0.0001	0.0005
SANA TNF SIGNALING UP	81	8 (9.9)	8 (9.9)	0 (0)	<0.0001	0.0005
BROCKE APOPTOSIS REVERSED BY IL6	144	7 (4.9)	7 (4.9)	0 (0)	<0.0001	0.0005
DELYS THYROID CANCER UP	435	7 (1.6)	7 (1.6)	0 (0)	<0.0001	0.0005
DER IFN GAMMA RESPONSE UP	72	7 (9.7)	7 (9.7)	0 (0)	<0.0001	0.0005
GRUETZMANN PANCREATIC CANCER UP	351	7 (2)	7 (2)	0 (0)	<0.0001	0.0005
HORIUCHI WTAP TARGETS UP	295	7 (2.4)	7 (2.4)	0 (0)	<0.0001	0.0005
LIANG SILENCED BY METHYLATION 2	55	7 (12.7)	7 (12.7)	0 (0)	<0.0001	0.0005
MCLACHLAN DENTAL CARIES UP	227	7 (3.1)	7 (3.1)	0 (0)	<0.0001	0.0005
PHONG TNF RESPONSE NOT VIA P38	333	7 (2.1)	7 (2.1)	0 (0)	<0.0001	0.0005
SENGUPTA NASOPHARYNGEAL CARCINOMA WITH LMP1 UP	387	7 (1.8)	7 (1.8)	0 (0)	<0.0001	0.0005
ZHANG RESPONSE TO IKK INHIBITOR AND TNF UP	221	7 (3.2)	7 (3.2)	0 (0)	<0.0001	0.0005
ALTEMEIER RESPONSE TO LPS WITH MECHANICAL VENTILATION	126	6 (4.8)	6 (4.8)	0 (0)	<0.0001	0.0005
BENNETT SYSTEMIC LUPUS ERYTHEMATOSUS	32	6 (18.8)	6 (18.8)	0 (0)	<0.0001	0.0005
MCLACHLAN DENTAL CARIES DN	219	6 (2.7)	6 (2.7)	0 (0)	<0.0001	0.0005
QI PLASMACYTOMA UP	251	6 (2.4)	6 (2.4)	0 (0)	<0.0001	0.0005
SEKI INFLAMMATORY RESPONSE LPS UP	77	6 (7.8)	6 (7.8)	0 (0)	<0.0001	0.0005
UROSEVIC RESPONSE TO IMIQUIMOD	23	6 (26.1)	6 (26.1)	0 (0)	<0.0001	0.0005
WIELAND UP BY HBV INFECTION	100	6 (6)	6 (6)	0 (0)	<0.0001	0.0005

Category Name	Category Genes #	Any-reg. SDEG #(%)	Up-reg. SDEG #(%)	Down-reg. SDEG #(%)	P	FDR
BOWIE RESPONSE TO EXTRACELLULAR MATRIX	17	5 (29.4)	5 (29.4)	0 (0)	<0.0001	0.0005
BOWIE RESPONSE TO TAMOXIFEN	18	5 (27.8)	5 (27.8)	0 (0)	<0.0001	0.0005
DAUER STAT3 TARGETS DN	51	5 (9.8)	5 (9.8)	0 (0)	<0.0001	0.0005
FLECHNER BIOPSY KIDNEY TRANSPLANT REJECTED VS OK UP	85	5 (5.9)	5 (5.9)	0 (0)	<0.0001	0.0005
GAVIN FOXP3 TARGETS CLUSTER P6	85	5 (5.9)	5 (5.9)	0 (0)	<0.0001	0.0005
KRASNOSELSKAYA ILF3 TARGETS UP	37	5 (13.5)	5 (13.5)	0 (0)	<0.0001	0.0005
LIU VAV3 PROSTATE CARCINOGENESIS UP	85	5 (5.9)	5 (5.9)	0 (0)	<0.0001	0.0005
SEITZ NEOPLASTIC TRANSFORMATION BY 8P DELETION UP	69	5 (7.2)	5 (7.2)	0 (0)	<0.0001	0.0005
STAMBOLSKY TARGETS OF MUTATED TP53 DN	51	5 (9.8)	5 (9.8)	0 (0)	<0.0001	0.0005
ZHANG INTERFERON RESPONSE	23	5 (21.7)	5 (21.7)	0 (0)	<0.0001	0.0005
ZHAN MULTIPLE MYELOMA LB DN	39	5 (12.8)	5 (12.8)	0 (0)	<0.0001	0.0005
BOYAULT LIVER CANCER SUBCLASS G5 DN	27	4 (14.8)	4 (14.8)	0 (0)	<0.0001	0.0005
CHANG IMMORTALIZED BY HPV31 DN	63	4 (6.3)	4 (6.3)	0 (0)	<0.0001	0.0005
GRANDVAUX IFN RESPONSE NOT VIA IRF3	14	4 (28.6)	4 (28.6)	0 (0)	<0.0001	0.0005
GRANDVAUX IRF3 TARGETS UP	15	4 (26.7)	4 (26.7)	0 (0)	<0.0001	0.0005
JECHLINGER EPITHELIAL TO MESENCHYMAL TRANSITION UP	71	4 (5.6)	4 (5.6)	0 (0)	<0.0001	0.0005
KHETCHOUMIAN TRIM24 TARGETS UP	45	4 (8.9)	4 (8.9)	0 (0)	<0.0001	0.0005
MAHADEVAN RESPONSE TO MP470 UP	19	4 (21.1)	4 (21.1)	0 (0)	<0.0001	0.0005
ROETH TERT TARGETS UP	14	4 (28.6)	4 (28.6)	0 (0)	<0.0001	0.0005
WUNDER INFLAMMATORY RESPONSE AND CHOLESTEROL UP	61	4 (6.6)	4 (6.6)	0 (0)	<0.0001	0.0005
IIZUKA LIVER CANCER PROGRESSION G1 G2 UP	12	3 (25)	3 (25)	0 (0)	<0.0001	0.0005
WATANABE ULCERATIVE COLITIS WITH CANCER DN	13	3 (23.1)	3 (23.1)	0 (0)	<0.0001	0.0005
DODD NASOPHARYNGEAL CARCINOMA DN	1330	10 (0.8)	10 (0.8)	0 (0)	<0.0001	0.0008
COLINA TARGETS OF 4EBP1 AND 4EBP2	339	6 (1.8)	6 (1.8)	0 (0)	<0.0001	0.0008
FOSTER TOLERANT MACROPHAGE UP	155	5 (3.2)	5 (3.2)	0 (0)	<0.0001	0.0008
MIKKELSEN MCV6 LCP WITH H3K4ME3	153	5 (3.3)	5 (3.3)	0 (0)	<0.0001	0.0008
PLASARI TGFB1 TARGETS 10HR DN	233	5 (2.1)	5 (2.1)	0 (0)	<0.0001	0.0008
RIGGINS TAMOXIFEN RESISTANCE DN	215	5 (2.3)	5 (2.3)	0 (0)	<0.0001	0.0008
TONKS TARGETS OF RUNX1 RUNX1T1 FUSION MONOCYTE UP	201	5 (2.5)	5 (2.5)	0 (0)	<0.0001	0.0008
BASSO CD40 SIGNALING UP	99	4 (4)	4 (4)	0 (0)	<0.0001	0.0008
DAUER STAT3 TARGETS UP	50	4 (8)	4 (8)	0 (0)	<0.0001	0.0008
KIM GLIS2 TARGETS UP	84	4 (4.8)	4 (4.8)	0 (0)	<0.0001	0.0008
MISSIAGLIA REGULATED BY METHYLATION UP	120	4 (3.3)	4 (3.3)	0 (0)	<0.0001	0.0008
GROSS ELK3 TARGETS DN	32	3 (9.4)	3 (9.4)	0 (0)	<0.0001	0.0008
NIKOLSKY OVERCONNECTED IN BREAST CANCER	21	3 (14.3)	3 (14.3)	0 (0)	<0.0001	0.0008
SHIN B CELL LYMPHOMA CLUSTER 8	36	3 (8.3)	3 (8.3)	0 (0)	<0.0001	0.0008
YAN ESCAPE FROM ANOIKIS	24	3 (12.5)	3 (12.5)	0 (0)	<0.0001	0.0008
GRAESSMANN APOPTOSIS BY DOXORUBICIN UP	1132	9 (0.8)	9 (0.8)	0 (0)	<0.0001	0.0011

Category Name	Category Genes #	Any-reg. SDEG #(%)	Up-reg. SDEG #(%)	Down-reg. SDEG #(%)	P	FDR
LIM MAMMARY STEM CELL DN	422	6 (1.4)	6 (1.4)	0 (0)	<0.0001	0.0011
ACEVEDO FGFR1 TARGETS IN PROSTATE CANCER MODEL UP	278	5 (1.8)	5 (1.8)	0 (0)	<0.0001	0.0011
MIYAGAWA TARGETS OF EWSR1 ETS FUSIONS UP	247	5 (2)	5 (2)	0 (0)	<0.0001	0.0011
TONKS TARGETS OF RUNX1 RUNX1T1 FUSION ERYTHROCYTE UP	151	4 (2.6)	4 (2.6)	0 (0)	<0.0001	0.0011
ABBUD LIF SIGNALING 1 UP	46	3 (6.5)	3 (6.5)	0 (0)	<0.0001	0.0011
FOSTER TOLERANT MACROPHAGE DN	405	6 (1.5)	6 (1.5)	0 (0)	<0.0001	0.0015
RICKMAN METASTASIS DN	258	5 (1.9)	5 (1.9)	0 (0)	<0.0001	0.0018
HAN JNK SINGALING UP	34	3 (8.8)	3 (8.8)	0 (0)	<0.0001	0.0018
GOZGIT ESR1 TARGETS DN	741	7 (0.9)	7 (0.9)	0 (0)	<0.0001	0.0021
DOUGLAS BMI1 TARGETS DN	308	5 (1.6)	5 (1.6)	0 (0)	<0.0001	0.0021
POOLA INVASIVE BREAST CANCER UP	272	5 (1.8)	5 (1.8)	0 (0)	<0.0001	0.0021
SCHUETZ BREAST CANCER DUCTAL INVASIVE UP	338	5 (1.5)	5 (1.5)	0 (0)	<0.0001	0.0021
SEMBA FHIT TARGETS UP	11	2 (18.2)	2 (18.2)	0 (0)	<0.0001	0.0024
CASTELLANO NRAS TARGETS UP	68	3 (4.4)	3 (4.4)	0 (0)	<0.0001	0.0026
GRAHAM CML QUIESCENT VS NORMAL DIVIDING UP	55	3 (5.5)	3 (5.5)	0 (0)	<0.0001	0.0026
PLASARI TGFB1 SIGNALING VIA NFIC 10HR UP	54	3 (5.6)	3 (5.6)	0 (0)	<0.0001	0.0026
MONNIER POSTRADIATION TUMOR ESCAPE DN	357	5 (1.4)	5 (1.4)	0 (0)	0.0001	0.0036
FURUKAWA DUSP6 TARGETS PCI35 UP	73	3 (4.1)	3 (4.1)	0 (0)	0.0001	0.0038
GARGALOVIC RESPONSE TO OXIDIZED PHOSPHOLIPIDS GREY DN	73	3 (4.1)	3 (4.1)	0 (0)	0.0001	0.0038
SHIN B CELL LYMPHOMA CLUSTER 5	17	2 (11.8)	2 (11.8)	0 (0)	0.0001	0.0038
SMID BREAST CANCER BASAL UP	607	6 (1)	6 (1)	0 (0)	0.0001	0.004
PAPASPYRIDONOS UNSTABLE ATEROSCLEROTIC PLAQUE UP	51	3 (5.9)	3 (5.9)	0 (0)	0.0001	0.004
RUAN RESPONSE TO TNF UP	12	2 (16.7)	2 (16.7)	0 (0)	0.0001	0.004
WORSCHECH TUMOR EVASION AND TOLEROGENICITY DN	15	2 (13.3)	2 (13.3)	0 (0)	0.0001	0.004
LINDSTEDT DENDRITIC CELL MATURATION C	69	3 (4.3)	3 (4.3)	0 (0)	0.0001	0.0041
NATSUME RESPONSE TO INTERFERON BETA UP	71	3 (4.2)	3 (4.2)	0 (0)	0.0001	0.0041
LEE SP4 THYMOCYTE	14	2 (14.3)	2 (14.3)	0 (0)	0.0001	0.0041
TSAI DNAJB4 TARGETS UP	13	2 (15.4)	2 (15.4)	0 (0)	0.0001	0.0041
CHEN METABOLIC SYNDROM NETWORK	1172	8 (0.7)	8 (0.7)	0 (0)	0.0002	0.0046
LINDSTEDT DENDRITIC CELL MATURATION A	67	3 (4.5)	3 (4.5)	0 (0)	0.0002	0.0046
ZHOU INFLAMMATORY RESPONSE LPS UP	401	5 (1.2)	5 (1.2)	0 (0)	0.0002	0.0048
LEE RECENT THYMIC EMIGRANT	217	4 (1.8)	4 (1.8)	0 (0)	0.0002	0.0048
WIERENGA STAT5A TARGETS UP	210	4 (1.9)	4 (1.9)	0 (0)	0.0002	0.0048
JACKSON DNMT1 TARGETS UP	76	3 (3.9)	3 (3.9)	0 (0)	0.0002	0.0048
WANG RESPONSE TO GSK3 INHIBITOR SB216763 UP	378	5 (1.3)	5 (1.3)	0 (0)	0.0002	0.0052
GALINDO IMMUNE RESPONSE TO ENTEROTOXIN	85	3 (3.5)	3 (3.5)	0 (0)	0.0002	0.0052
HINATA NFKB TARGETS KERATINOCYTE UP	91	3 (3.3)	3 (3.3)	0 (0)	0.0002	0.0062
YORDY RECIPROCAL REGULATION BY ETS1 AND SP100 DN	81	3 (3.7)	3 (3.7)	0 (0)	0.0002	0.0062

Category Name	Category Genes #	Any-reg. SDEG #(%)	Up-reg. SDEG #(%)	Down-reg. SDEG #(%)	P	FDR
ZHU CMV 24 HR UP	93	3 (3.2)	3 (3.2)	0 (0)	0.0002	0.0062
SARTIPY BLUNTED BY INSULIN RESISTANCE UP	19	2 (10.5)	2 (10.5)	0 (0)	0.0002	0.0067
DANG REGULATED BY MYC DN	247	4 (1.6)	4 (1.6)	0 (0)	0.0003	0.0068
BENPORATH ES CORE NINE CORRELATED	95	3 (3.2)	3 (3.2)	0 (0)	0.0003	0.0068
JOHNSTONE PARVB TARGETS 3 UP	425	5 (1.2)	5 (1.2)	0 (0)	0.0003	0.007
RASHI NFKB1 TARGETS	19	2 (10.5)	2 (10.5)	0 (0)	0.0003	0.007
BAELDE DIABETIC NEPHROPATHY DN	425	5 (1.2)	5 (1.2)	0 (0)	0.0003	0.0072
RUAN RESPONSE TO TNF TROGLITAZONE UP	17	2 (11.8)	2 (11.8)	0 (0)	0.0003	0.0072
ZWANG TRANSIENTLY UP BY 1ST EGF PULSE ONLY	1723	9 (0.5)	9 (0.5)	0 (0)	0.0003	0.0081
MARTENS BOUND BY PML RARA FUSION	446	5 (1.1)	5 (1.1)	0 (0)	0.0003	0.0081
LEE BMP2 TARGETS UP	723	6 (0.8)	6 (0.8)	0 (0)	0.0003	0.0084
VERHAAK AML WITH NPM1 MUTATED DN	238	4 (1.7)	4 (1.7)	0 (0)	0.0003	0.0084
HELLER HDAC TARGETS SILENCED BY METHYLATION DN	269	4 (1.5)	4 (1.5)	0 (0)	0.0003	0.0086
CHARAFE BREAST CANCER LUMINAL VS BASAL DN	440	5 (1.1)	5 (1.1)	0 (0)	0.0004	0.009
GARY CD5 TARGETS UP	458	5 (1.1)	5 (1.1)	0 (0)	0.0004	0.01

Table A111: Significantly enriched MSigDB Chemical/Genetic Perturbation Sets (RNA-Seq, T-cells, Day 1). Results sorted by FDR and #SDEG genes.

Study Visit	Treatment Group	N	Gene Variables	IMO Variables	CV	Canonical Correlation (C1)	Canonical Correlation (C2)	Explained Variance (Gene Variables, C1+C2)	Explained Variance (IMO Variables, C1+C2)
Day 1	SV-AS03	10	40	13	0.96	0.96	0.94	0.38	0.40
Day 1	SV-PBS	9	40	13	0.88	0.99	0.98	0.56	0.32
Day 3	SV-AS03	10	70	13	0.91	0.99	0.93	0.14	0.52
Day 3	SV-PBS	9	70	13	0.73	0.94	0.93	0.33	0.64
Day 7	SV-AS03	10	23	13	0.76	0.97	0.86	0.15	0.54
Day 7	SV-PBS	10	23	13	0.65	0.98	0.95	0.14	0.51
Day 28	SV-AS03	8	33	14	0.72	0.93	0.88	0.34	0.57
Day 28	SV-PBS	10	33	14	0.57	0.94	0.94	0.32	0.39

Table A112: Canonical correlation analysis summary statistics (RNA-Seq, B-cells)

Study Visit	Treatment Group	N	Gene Variables	IMO Variables	CV	Canonical Correlation (C1)	Canonical Correlation (C2)	Explained Variance (Gene Variables, C1+C2)	Explained Variance (IMO Variables, C1+C2)
Day 1	SV-AS03	10	246	13	0.86	0.94	0.94	0.41	0.51
Day 1	SV-PBS	10	246	13	0.65	0.96	0.96	0.23	0.34
Day 3	SV-AS03	10	54	13	0.83	0.96	0.93	0.30	0.28
Day 3	SV-PBS	10	54	13	0.71	0.96	0.95	0.23	0.58
Day 7	SV-AS03	10	24	13	0.54	0.98	0.97	0.26	0.34
Day 7	SV-PBS	10	24	13	0.78	0.97	0.95	0.39	0.34
Day 28	SV-AS03	10	36	14	0.38	0.97	0.94	0.19	0.57
Day 28	SV-PBS	9	36	14	0.57	0.96	0.95	0.52	0.24

Table A113: Canonical correlation analysis summary statistics (RNA-Seq, Dendritic cells)

Study Visit	Treatment Group	N	Gene Variables	IMO Variables	CV	Canonical Correlation (C1)	Canonical Correlation (C2)	Explained Variance (Gene Variables, C1+C2)	Explained Variance (IMO Variables, C1+C2)
Day 1	SV-AS03	10	425	13	0.86	0.99	0.97	0.44	0.33
Day 1	SV-PBS	10	425	13	0.56	0.96	0.94	0.24	0.36
Day 3	SV-AS03	10	40	13	0.92	0.98	0.96	0.49	0.35
Day 3	SV-PBS	10	40	13	0.80	0.99	0.98	0.21	0.65
Day 7	SV-AS03	10	16	13	0.20	0.98	0.93	0.51	0.23
Day 7	SV-PBS	10	16	13	0.65	0.99	0.98	0.55	0.34
Day 28	SV-AS03	10	20	14	0.88	0.98	0.96	0.23	0.54
Day 28	SV-PBS	10	20	14	0.83	0.97	0.95	0.44	0.16

Table A114: Canonical correlation analysis summary statistics (RNA-Seq, Monocytes)

Study Visit	Treatment Group	N	Gene Variables	IMO Variables	CV	Canonical Correlation (C1)	Canonical Correlation (C2)	Explained Variance (Gene Variables, C1+C2)	Explained Variance (IMO Variables, C1+C2)
Day 1	SV-AS03	10	756	13	0.81	1.00	0.99	0.66	0.23
Day 1	SV-PBS	10	756	13	0.90	0.96	0.93	0.23	0.40
Day 3	SV-AS03	10	153	13	0.45	0.98	0.91	0.17	0.52

Study Visit	Treatment Group	N	Gene Variables	IMO Variables	CV	Canonical Correlation (C1)	Canonical Correlation (C2)	Explained Variance (Gene Variables, C1+C2)	Explained Variance (IMO Variables, C1+C2)
Day 3	SV-PBS	10	153	13	0.32	1.00	1.00	0.45	0.15
Day 7	SV-AS03	10	39	13	0.68	0.95	0.94	0.35	0.49
Day 7	SV-PBS	10	39	13	0.81	0.97	0.95	0.26	0.45
Day 28	SV-AS03	9	73	14	0.93	0.98	0.96	0.30	0.57
Day 28	SV-PBS	10	73	14	0.78	0.99	0.98	0.32	0.29

Table A115: Canonical correlation analysis summary statistics (RNA-Seq, Neutrophils)

Study Visit	Treatment Group	N	Gene Variables	IMO Variables	CV	Canonical Correlation (C1)	Canonical Correlation (C2)	Explained Variance (Gene Variables, C1+C2)	Explained Variance (IMO Variables, C1+C2)
Day 1	SV-AS03	10	113	13	0.88	0.99	0.98	0.54	0.35
Day 1	SV-PBS	10	113	13	0.63	0.98	0.97	0.17	0.38
Day 3	SV-AS03	10	110	13	0.78	0.96	0.94	0.60	0.45
Day 3	SV-PBS	10	110	13	0.81	0.93	0.90	0.33	0.57
Day 7	SV-AS03	10	33	13	0.60	0.93	0.92	0.31	0.41
Day 7	SV-PBS	10	33	13	0.89	0.97	0.94	0.40	0.53
Day 28	SV-AS03	10	294	14	0.74	1.00	1.00	0.42	0.57
Day 28	SV-PBS	10	294	14	0.64	0.98	0.97	0.19	0.45

Table A116: Canonical correlation analysis summary statistics (RNA-Seq, NK-cells)

Study Visit	Treatment Group	N	Gene Variables	IMO Variables	CV	Canonical Correlation (C1)	Canonical Correlation (C2)	Explained Variance (Gene Variables, C1+C2)	Explained Variance (IMO Variables, C1+C2)
Day 1	SV-AS03	10	60	13	0.95	0.95	0.90	0.72	0.32
Day 1	SV-PBS	9	60	13	0.47	0.94	0.94	0.55	0.39
Day 3	SV-AS03	9	17	13	0.82	0.98	0.94	0.13	0.56
Day 3	SV-PBS	10	17	13	0.72	0.96	0.94	0.22	0.61
Day 7	SV-AS03	10	15	13	0.35	0.97	0.97	0.37	0.37
Day 7	SV-PBS	10	15	13	0.76	0.96	0.93	0.29	0.44
Day 28	SV-AS03	10	15	14	0.54	0.94	0.94	0.49	0.21
Day 28	SV-PBS	10	15	14	0.90	0.96	0.94	0.23	0.34

Table A117: Canonical correlation analysis summary statistics (RNA-Seq, T-cells)

Gene ID	Gene Name	G1 (SV-AS03)	G1 (SV-PBS)	G2 (SV-AS03)	G2 (SV-PBS)	V1 (SV-AS03)	V1 (SV-PBS)	V2 (SV-AS03)	V2 (SV-PBS)	V1+V2 (SV-AS03)	V1+V2 (SV-PBS)
ENSG00000116663	FBXO6	0.54	-0.81	0.31	-0.56	28.7	65.1	9.5	31.6	38.2	96.7
ENSG00000137959	IFI44L	0.63	-0.86	0.57	-0.45	39.6	73.8	33.0	19.9	72.5	93.7
ENSG00000163993	S100P	-0.46	0.80	0.30	-0.54	20.7	64.2	9.0	29.5	29.7	93.7
ENSG00000117228	GBP1	0.42	-0.90	0.62	-0.34	17.7	81.6	39.1	11.3	56.8	92.9
ENSG00000115415	STAT1	0.30	-0.89	0.71	-0.34	9.3	79.0	50.0	11.7	59.3	90.7
ENSG00000162645	GBP2	0.44	-0.91	0.60	-0.26	19.4	82.1	35.7	6.6	55.1	88.7
ENSG00000162654	GBP4	0.54	-0.89	0.63	-0.29	28.9	79.6	40.2	8.4	69.2	88.0
ENSG00000087237	CETP	0.26	-0.80	0.30	-0.44	6.9	64.2	9.2	19.3	16.1	83.5
ENSG00000125347	IRF1	0.55	-0.90	0.34	0.10	30.1	81.9	11.9	1.0	42.0	82.9
ENSG00000140511	HAPLN3	0.54	-0.84	0.42	-0.34	28.7	70.6	17.5	11.5	46.2	82.1
ENSG00000180287	PLD5	0.24	-0.90	0.04	0.00	5.9	81.0	0.1	0.0	6.0	81.0
ENSG00000119917	IFIT3	0.63	-0.76	0.50	-0.47	39.3	58.2	24.6	21.7	63.9	79.9
ENSG00000134326	CMPK2	0.28	-0.88	0.48	-0.06	7.7	78.1	22.8	0.4	30.5	78.5
ENSG00000154451	GBP5	0.53	-0.80	0.64	-0.34	28.2	63.5	40.8	11.5	68.9	75.0
ENSG00000151689	INPP1	0.48	-0.86	0.61	0.02	22.7	74.5	37.5	0.0	60.2	74.5
ENSG00000184779	RPS17	0.08	-0.79	-0.50	-0.32	0.6	62.4	24.8	10.4	25.4	72.8
ENSG00000125810	CD93	-0.73	0.44	0.41	0.62	52.8	19.3	17.0	39.0	69.7	58.3
ENSG00000185745	IFIT1	0.56	-0.54	0.32	-0.64	30.8	29.1	10.4	40.3	41.2	69.4
ENSG00000221963	APOL6	0.27	-0.75	0.52	-0.34	7.2	56.6	26.6	11.9	33.8	68.5
ENSG00000123689	G0S2	-0.35	0.72	-0.34	0.34	12.2	52.2	11.4	11.4	23.6	63.6
ENSG00000163464	CXCR1	-0.62	0.73	-0.18	-0.08	38.9	53.9	3.2	0.6	42.1	54.4
ENSG00000171051	FPR1	-0.71	0.50	0.19	0.52	50.0	25.1	3.8	27.2	53.8	52.3
ENSG00000172159	FRMD3	0.34	-0.65	0.64	-0.21	11.5	41.7	41.4	4.6	53.0	46.3
ENSG00000085265	FCN1	-0.72	-0.18	-0.07	0.59	51.7	3.2	0.5	34.6	52.2	37.9
ENSG00000225972	RP5-857K21.9	-0.08	-0.63	-0.49	-0.30	0.7	39.3	23.5	8.7	24.2	48.0
ENSG00000203747	FCGR3A	-0.29	-0.35	0.26	0.59	8.6	12.4	6.5	34.8	15.1	47.2
ENSG00000155974	GRIP1	0.43	-0.65	-0.33	-0.20	18.6	42.7	10.6	4.1	29.2	46.8
ENSG00000248121	AC005562.2	-0.22	0.13	0.63	-0.12	4.9	1.6	39.6	1.4	44.5	3.0
ENSG00000257924	RP11-493L12.5	0.31	-0.07	0.58	0.27	9.6	0.4	33.2	7.6	42.8	8.0
ENSG00000214872	SMTNL1	0.42	-0.62	0.27	-0.16	17.8	38.9	7.3	2.7	25.1	41.5
ENSG00000126822	PLEKHG3	0.48	-0.13	-0.43	0.47	22.9	1.7	18.2	21.7	41.0	23.4
ENSG00000010030	ETV7	0.29	-0.60	-0.25	0.19	8.3	36.2	6.2	3.8	14.5	40.0
ENSG00000073756	PTGS2	0.17	0.63	0.26	-0.09	2.8	39.1	6.9	0.9	9.7	40.0
ENSG00000114315	HES1	0.37	0.06	-0.37	0.57	13.8	0.4	13.5	32.4	27.3	32.8
ENSG00000225131	PSME2P2	0.51	-0.21	0.25	0.24	26.0	4.5	6.1	5.8	32.1	10.4
ENSG00000128849	CGNL1	-0.54	0.39	0.03	0.11	29.2	15.3	0.1	1.1	29.3	16.4
ENSG00000178997	EXD1	-0.41	-0.16	-0.07	0.48	16.8	2.6	0.5	23.3	17.3	25.9
ENSG00000090382	LYZ	-0.34	0.18	0.36	0.12	11.8	3.1	13.1	1.5	24.8	4.6
ENSG00000212743	AL137145.1	0.19	0.34	-0.20	0.19	3.8	11.4	3.8	3.5	7.6	15.0
ENSG00000011600	TYROBP	-0.35	0.19	-0.12	0.25	12.6	3.4	1.6	6.3	14.1	9.8

Table A118: Canonical loadings and explained variance (RNA-Seq, B-cells, Day 1, Gene variables). Gene variables with at least 25% of explained variance for the first two canonical variates are listed. GX: canonical loadings for canonical variate X, VX: % explained variance for canonical variate X, V1+V2: total % explained variance for both canonical variates. Values for CCA results with a poor cross validation score (< 0.70) are left blank. Sorted by maximum explained variance across treatments (V1+V2).

IMO Variable	IMO Variable Name	I1 (SV-AS03)	I1 (SV-PBS)	I2 (SV-AS03)	I2 (SV-PBS)	V1 (SV-AS03)	V1 (SV-PBS)	V2 (SV-AS03)	V2 (SV-PBS)	V1+V2 (SV-AS03)	V1+V2 (SV-PBS)
IP-10	Interferon gamma-induced protein 10	0.63	-0.78	0.37	-0.43	39.3	60.8	13.7	18.2	53.1	79.0

IMO Variable	IMO Variable Name	I1 (SV-AS03)	I1 (SV-PBS)	I2 (SV-AS03)	I2 (SV-PBS)	V1 (SV-AS03)	V1 (SV-PBS)	V2 (SV-AS03)	V2 (SV-PBS)	V1+V2 (SV-AS03)	V1+V2 (SV-PBS)
IL-1β	Interleukin-1 beta	0.85	-0.07	-0.11	-0.27	71.9	0.4	1.2	7.5	73.1	7.9
IL-8	Interleukin-8	0.33	-0.62	-0.41	0.57	10.6	38.3	17.0	33.0	27.6	71.3
MIP-1α	Macrophage inflammatory protein	0.57	-0.54	0.61	-0.26	32.5	29.5	36.9	6.5	69.4	36.0
IL-6	Interleukin-6	0.78	-0.08	0.00	0.21	61.4	0.6	0.0	4.4	61.4	5.0
IL-10	Interleukin-10	0.60	-0.69	-0.03	0.36	35.7	47.4	0.1	12.9	35.8	60.3
MCP-1	Monocyte chemoattractant protein-1	0.51	-0.74	-0.46	-0.12	25.6	54.7	20.9	1.6	46.5	56.2
Eotaxin-1	Eotaxin-1	-0.36	0.27	-0.55	0.11	12.9	7.3	30.1	1.2	43.0	8.5
Nt	Microneutralization inhibition	0.01	-0.47	-0.60	-0.35	0.0	22.1	35.5	12.1	35.6	34.3
IL-12p70	Interleukin-12 p70	0.14	0.01	0.50	0.55	2.0	0.0	25.1	30.0	27.1	30.0
TNF	Tumor necrosis factors	0.05	-0.14	-0.53	-0.40	0.2	2.0	28.4	16.1	28.7	18.1
RANTES	Chemokine (C-C motif) ligand 5	0.23	-0.08	0.29	0.22	5.2	0.6	8.3	4.8	13.5	5.4
IFN-γ	Interferon gamma	-0.28	-0.06	0.01	0.19	8.0	0.4	0.0	3.7	8.0	4.1

Table A119: Canonical loadings and explained variance (RNA-Seq, B-cells, Day 1, IMO variables). IMO variables with at least 25% of explained variance for the first two canonical variates are listed. GX: canonical loadings for canonical variate X, VX: % explained variance for canonical variate X, V1+V2: total % explained variance for both canonical variates. Values for CCA results with a poor cross validation score (< 0.70) are left blank. Sorted by maximum explained variance across treatments (V1+V2).

Gene ID	Gene Name	G1 (SV-AS03)	G1 (SV-PBS)	G2 (SV-AS03)	G2 (SV-PBS)	V1 (SV-AS03)	V1 (SV-PBS)	V2 (SV-AS03)	V2 (SV-PBS)	V1+V2 (SV-AS03)	V1+V2 (SV-PBS)
ENSG00000232553	CLK2P	0.84	-0.77	0.28	0.06	70.2	58.7	7.7	0.3	77.9	59.1
ENSG00000221869	CEBPD	-0.36	-0.83	0.10	0.15	12.8	68.4	1.1	2.1	13.9	70.5
ENSG00000163464	CXCR1	0.02	-0.65	0.21	0.45	0.0	41.8	4.3	20.3	4.4	62.0
ENSG00000147459	DOCK5	-0.24	-0.56	0.05	0.51	5.7	31.6	0.2	26.4	5.9	58.0
ENSG00000082074	FYB	-0.35	-0.63	-0.02	0.43	12.3	39.3	0.0	18.2	12.3	57.6
ENSG00000228960	OR2A9P	0.37	0.74	0.16	-0.15	13.8	55.0	2.6	2.1	16.3	57.1
ENSG00000011600	TYROBP	-0.34	-0.72	0.19	0.20	11.4	51.2	3.4	3.9	14.9	55.1
ENSG00000120318	ARAP3	-0.05	-0.71	-0.33	0.20	0.2	50.9	11.1	4.0	11.3	54.9
ENSG00000119917	IFIT3	-0.64	-0.24	-0.34	0.29	41.1	5.6	11.7	8.5	52.8	14.1
ENSG00000225603	RP11-325P15.1	0.53	0.72	-0.12	0.05	28.2	52.5	1.4	0.3	29.6	52.7
ENSG00000123689	G0S2	-0.08	-0.66	0.19	0.31	0.6	43.1	3.5	9.4	4.1	52.5
ENSG00000232838	AL050321.1	-0.40	-0.68	0.21	-0.25	16.0	45.7	4.4	6.2	20.4	51.9
ENSG00000166825	ANPEP	-0.17	-0.56	0.15	0.45	2.8	31.2	2.2	20.4	5.0	51.6
ENSG00000203747	FCGR3A	-0.44	-0.66	0.40	0.28	19.8	43.0	16.3	8.1	36.1	51.1
ENSG00000172243	CLEC7A	-0.36	-0.50	0.12	0.50	12.9	24.9	1.5	25.4	14.4	50.3
ENSG00000067182	TNFRSF1A	-0.13	-0.42	0.27	0.57	1.7	17.7	7.3	32.2	9.1	49.9
ENSG00000140749	IGSF6	-0.44	-0.69	-0.12	0.11	19.2	48.3	1.5	1.2	20.7	49.5
ENSG00000151948	GLT1D1	-0.26	-0.46	0.25	0.50	6.8	21.3	6.2	24.8	12.9	46.1
ENSG00000112742	TTK	-0.27	-0.41	0.24	0.54	7.4	16.7	6.0	29.0	13.3	45.8
ENSG00000143546	S100A8	-0.28	-0.58	0.04	0.32	7.7	34.0	0.2	10.1	7.9	44.1
ENSG00000072110	ACTN1	-0.40	-0.59	-0.04	0.29	16.0	35.0	0.1	8.7	16.2	43.7
ENSG00000197249	SERPINA1	-0.31	-0.65	0.02	0.10	9.8	42.3	0.0	1.0	9.8	43.3
ENSG00000133048	CHI3L1	0.16	-0.63	0.18	0.16	2.4	40.2	3.3	2.6	5.7	42.8
ENSG00000252331	AP002812.1	0.22	-0.62	0.04	0.17	4.7	38.1	0.1	2.9	4.9	41.0
ENSG00000146592	CREB5	-0.42	-0.30	0.04	0.56	18.0	9.1	0.1	31.8	18.1	40.9
ENSG00000162747	FCGR3B	0.02	-0.61	0.25	0.18	0.0	36.9	6.2	3.2	6.3	40.1
ENSG00000185215	TNFAIP2	-0.46	-0.50	0.06	0.38	21.1	25.4	0.4	14.2	21.5	39.6
ENSG00000038427	VCAN	-0.57	0.62	0.07	0.12	32.0	37.9	0.6	1.4	32.6	39.3

Gene ID	Gene Name	G1 (SV-AS03)	G1 (SV-PBS)	G2 (SV-AS03)	G2 (SV-PBS)	V1 (SV-AS03)	V1 (SV-PBS)	V2 (SV-AS03)	V2 (SV-PBS)	V1+V2 (SV-AS03)	V1+V2 (SV-PBS)
ENSG00000186529	CYP4F3	-0.22	-0.53	0.27	0.33	4.8	28.0	7.3	11.1	12.1	39.1
ENSG00000126822	PLEKHG3	-0.03	-0.22	0.34	0.57	0.1	5.0	11.2	32.4	11.3	37.4
ENSG00000160593	AMICA1	-0.45	-0.57	0.19	0.22	20.3	32.7	3.4	4.7	23.7	37.4
ENSG00000131669	NINJ1	-0.13	-0.59	0.22	0.15	1.7	34.8	4.6	2.3	6.4	37.1
ENSG00000075426	FOSL2	-0.19	-0.57	0.25	0.19	3.6	32.9	6.3	3.7	9.9	36.6
ENSG00000135636	DYSF	-0.29	-0.48	0.13	0.37	8.6	22.8	1.6	13.5	10.3	36.3
ENSG00000185745	IFIT1	-0.45	-0.22	-0.40	0.36	20.1	4.8	15.8	12.9	35.9	17.7
ENSG00000169429	IL8	-0.18	-0.49	0.01	0.33	3.4	24.3	0.0	11.1	3.4	35.4
ENSG00000188056	TREML4	0.33	0.24	0.49	0.17	10.9	5.8	24.1	2.9	35.0	8.7
ENSG00000124731	TREM1	-0.22	-0.49	0.14	0.33	4.9	23.8	1.9	10.8	6.8	34.5
ENSG00000252318	AC097532.1	0.52	-0.25	-0.26	-0.10	27.0	6.1	6.7	0.9	33.7	7.0
ENSG00000162551	ALPL	0.05	-0.46	0.29	0.35	0.3	21.1	8.4	12.5	8.6	33.7
ENSG00000197405	C5AR1	-0.47	-0.51	0.01	0.27	21.6	25.9	0.0	7.2	21.6	33.1
ENSG00000180871	CXCR2	-0.04	-0.50	0.23	0.28	0.1	24.6	5.4	7.8	5.5	32.4
ENSG00000165801	ARHGEF40	-0.42	-0.05	0.38	0.29	17.8	0.2	14.2	8.6	32.0	8.8
ENSG00000134830	GPR77	0.40	-0.08	-0.26	0.56	16.2	0.6	6.8	30.9	23.0	31.5
ENSG00000119535	CSF3R	-0.18	-0.51	0.14	0.20	3.3	26.1	1.9	4.0	5.3	30.1
ENSG00000163220	S100A9	-0.29	-0.52	0.13	0.17	8.3	27.0	1.6	2.9	9.9	29.9
ENSG00000171051	FPR1	-0.33	-0.52	0.05	0.15	11.0	27.4	0.3	2.3	11.3	29.8
ENSG00000236496	RP11-255J3.2	-0.43	0.09	-0.33	-0.38	18.4	0.9	11.2	14.7	29.6	15.5
ENSG00000128383	APOBEC3A	-0.28	-0.35	0.00	0.41	8.0	11.9	0.0	16.8	8.0	28.7
ENSG00000100504	PYGL	-0.23	-0.29	0.07	0.44	5.1	8.2	0.5	19.3	5.6	27.5
ENSG00000126262	FFAR2	0.01	-0.48	0.08	0.21	0.0	23.0	0.7	4.5	0.7	27.4
ENSG00000125810	CD93	-0.50	-0.48	0.06	0.07	25.0	22.8	0.4	0.5	25.4	23.3
ENSG00000173868	PHOSPHO1	-0.18	-0.26	0.15	0.41	3.1	6.7	2.1	16.5	5.3	23.2
ENSG00000252197	AC091047.1	0.21	-0.48	0.10	0.01	4.5	23.2	1.0	0.0	5.5	23.2
ENSG00000179087	MGAM	-0.05	-0.37	0.02	0.31	0.3	13.5	0.1	9.4	0.3	22.8
ENSG00000252229	AC098691.1	0.27	-0.48	0.12	-0.03	7.5	22.6	1.5	0.1	9.0	22.7
ENSG00000073756	PTGS2	-0.07	-0.43	0.45	0.16	0.5	18.7	20.4	2.7	20.9	21.4
ENSG00000173535	TNFRSF10C	-0.10	-0.34	0.11	0.30	1.0	11.8	1.2	8.8	2.1	20.7
ENSG00000228696	ARL17B	-0.03	-0.42	-0.05	-0.17	0.1	17.3	0.2	2.9	0.3	20.3
ENSG00000252666	AD000090.1	0.45	-0.26	-0.01	0.06	19.9	6.7	0.0	0.3	20.0	7.0
ENSG00000244734	HBB	-0.13	-0.44	0.25	-0.03	1.6	19.1	6.5	0.1	8.1	19.2
ENSG00000008516	MMP25	-0.11	-0.40	-0.07	0.16	1.1	16.0	0.5	2.7	1.6	18.7
ENSG00000163993	S100P	-0.29	-0.39	0.25	0.15	8.3	15.2	6.3	2.4	14.6	17.6
ENSG00000184524	CEND1	0.39	-0.23	0.14	0.34	15.2	5.2	1.9	11.4	17.1	16.6
ENSG00000103569	AQP9	-0.12	-0.40	0.04	-0.03	1.5	15.9	0.2	0.1	1.7	16.1
ENSG00000117115	PADI2	0.00	-0.26	0.05	0.27	0.0	6.7	0.3	7.3	0.3	13.9
ENSG00000163823	CCR1	-0.23	-0.13	-0.28	0.28	5.5	1.7	7.9	7.7	13.4	9.4
ENSG00000198754	OXCT2	-0.11	-0.06	0.24	-0.23	1.2	0.4	5.7	5.5	6.9	5.9
ENSG00000127954	STEAP4	-0.18	-0.20	0.08	0.17	3.2	4.0	0.6	2.8	3.8	6.7
ENSG00000182885	GPR97	-0.09	-0.14	0.19	0.14	0.7	2.1	3.4	1.9	4.2	4.0

Table A120: Canonical loadings and explained variance (RNA-Seq, B-cells, Day 3, Gene variables). Gene variables with at least 25% of explained variance for the first two canonical variates are listed. GX: canonical loadings for canonical variate X, VX: % explained variance for canonical variate X, V1+V2: total % explained variance for both canonical variates. Values for CCA results with a poor cross validation score (< 0.70) are left blank. Sorted by maximum explained variance across treatments (V1+V2).

IMO Variable	IMO Variable Name	I1 (SV-AS03)	I1 (SV-PBS)	I2 (SV-AS03)	I2 (SV-PBS)	V1 (SV-AS03)	V1 (SV-PBS)	V2 (SV-AS03)	V2 (SV-PBS)	V1+V2 (SV-AS03)	V1+V2 (SV-PBS)
IL-12p70	Interleukin-12 p70	-0.98	0.95	-0.21	0.17	95.5	90.7	4.2	2.9	99.7	93.6
IFN-γ	Interferon gamma	-0.34	0.07	0.22	0.97	11.5	0.6	4.9	93.4	16.3	94.0
IL-10	Interleukin-10	-0.74	0.41	0.40	0.86	54.2	17.1	16.0	73.7	70.1	90.9
TNF	Tumor necrosis factors	-0.70	0.16	0.06	0.92	49.2	2.6	0.4	85.4	49.6	88.0
IL-1β	Interleukin-1 beta	-0.69	0.09	0.62	0.93	48.1	0.8	38.3	85.9	86.4	86.7
IL-8	Interleukin-8	-0.74	-0.02	0.55	0.87	54.4	0.1	29.8	75.1	84.2	75.1
IL-6	Interleukin-6	-0.62	-0.12	0.13	0.88	39.0	1.4	1.7	77.2	40.8	78.7
MCP-1	Monocyte chemoattractant protein-1	0.52	-0.18	0.69	-0.11	26.5	3.1	47.1	1.1	73.7	4.3
MIP-1α	Macrophage inflammatory protein	-0.46	-0.53	0.56	0.63	21.1	27.7	31.6	39.9	52.7	67.6
Nt	Microneutralization inhibition	0.41	-0.01	0.43	0.77	16.6	0.0	18.2	58.8	34.8	58.8
IP-10	Interferon gamma-induced protein 10	-0.41	-0.39	-0.25	0.66	16.8	14.9	6.3	43.7	23.1	58.7
RANTES	Chemokine (C-C motif) ligand 5	0.01	-0.40	0.50	-0.25	0.0	16.2	25.3	6.3	25.4	22.4
Eotaxin-1	Eotaxin-1	-0.03	-0.26	0.45	0.04	0.1	6.9	20.7	0.2	20.8	7.0

Table A121: Canonical loadings and explained variance (RNA-Seq, B-cells, Day 3, IMO variables). IMO variables with at least 25% of explained variance for the first two canonical variates are listed. GX: canonical loadings for canonical variate X, VX: % explained variance for canonical variate X, V1+V2: total % explained variance for both canonical variates. Values for CCA results with a poor cross validation score (< 0.70) are left blank. Sorted by maximum explained variance across treatments (V1+V2).

Gene ID	Gene Name	G1 (SV-AS03)	G1 (SV-PBS)	G2 (SV-AS03)	G2 (SV-PBS)	V1 (SV-AS03)	V1 (SV-PBS)	V2 (SV-AS03)	V2 (SV-PBS)	V1+V2 (SV-AS03)	V1+V2 (SV-PBS)
ENSG00000234925	RP11-254B13.3	0.72		-0.20		51.4		4.0		55.4	
ENSG00000206885	SNORA75_206885	0.66		-0.26		43.0		6.5		49.6	
ENSG00000182985	CADM1	0.06		-0.56		0.4		31.3		31.7	
ENSG00000105974	CAV1	0.50		0.22		25.3		5.0		30.3	
ENSG00000126262	FFAR2	-0.48		-0.23		23.1		5.1		28.3	
ENSG00000169679	BUB1	0.41		0.11		16.6		1.2		17.8	
ENSG00000089685	BIRC5	0.26		-0.32		7.0		10.6		17.5	
ENSG00000211643	IGLV5-52	0.37		-0.19		13.5		3.7		17.2	
ENSG00000148773	MKI67	0.23		-0.29		5.4		8.2		13.6	
ENSG00000153162	BMP6	0.32		-0.14		10.3		1.9		12.2	
ENSG00000253755	IGHGP	-0.19		-0.29		3.6		8.2		11.8	
ENSG00000112378	PERP	0.24		-0.20		5.8		4.2		9.9	
ENSG00000174946	GPR171	-0.30		0.06		9.1		0.4		9.5	
ENSG00000121807	CCR2	0.28		0.09		7.6		0.9		8.4	
ENSG00000214544	GTF2IRD2P1	0.01		-0.25		0.0		6.3		6.3	
ENSG00000171848	RRM2	0.14		-0.12		1.9		1.5		3.4	
ENSG00000117399	CDC20	0.17		-0.07		2.9		0.5		3.4	
ENSG00000169429	IL8	-0.17		0.00		2.8		0.0		2.8	
ENSG00000244734	HBB	0.16		0.01		2.6		0.0		2.6	
ENSG00000111424	VDR	0.13		-0.05		1.7		0.2		1.9	
ENSG00000128311	TST	0.08		0.10		0.6		1.0		1.6	
ENSG00000180535	BHLHA15	0.12		-0.02		1.5		0.0		1.5	
ENSG00000066279	ASPM	-0.01		-0.04		0.0		0.2		0.2	

Gene ID	Gene Name	G1 (SV-AS03)	G1 (SV-PBS)	G2 (SV-AS03)	G2 (SV-PBS)	V1 (SV-AS03)	V1 (SV-PBS)	V2 (SV-AS03)	V2 (SV-PBS)	V1+V2 (SV-AS03)	V1+V2 (SV-PBS)

Table A122: Canonical loadings and explained variance (RNA-Seq, B-cells, Day 7, Gene variables). Gene variables with at least 25% of explained variance for the first two canonical variates are listed. GX: canonical loadings for canonical variate X, VX: % explained variance for canonical variate X, V1+V2: total % explained variance for both canonical variates. Values for CCA results with a poor cross validation score (< 0.70) are left blank. Sorted by maximum explained variance across treatments (V1+V2).

IMO Variable	IMO Variable Name	I1 (SV-AS03)	I1 (SV-PBS)	I2 (SV-AS03)	I2 (SV-PBS)	V1 (SV-AS03)	V1 (SV-PBS)	V2 (SV-AS03)	V2 (SV-PBS)	V1+V2 (SV-AS03)	V1+V2 (SV-PBS)
IL-12p70	Interleukin-12 p70	0.96		0.07		93.0		0.5		93.6	
IL-1β	Interleukin-1 beta	0.69		-0.67		47.4		44.3		91.7	
IL-6	Interleukin-6	0.63		0.69		39.7		47.3		87.0	
TNF	Tumor necrosis factors	0.87		-0.12		76.0		1.4		77.4	
Nt	Microneutralization inhibition	-0.14		-0.85		1.9		72.2		74.1	
IL-10	Interleukin-10	0.82		-0.13		66.6		1.7		68.3	
IL-8	Interleukin-8	0.72		-0.05		52.2		0.3		52.4	
RANTES	Chemokine (C-C motif) ligand 5	0.60		-0.38		35.5		14.1		49.6	
Eotaxin-1	Eotaxin-1	0.53		0.30		27.7		8.8		36.5	
IP-10	Interferon gamma-induced protein 10	-0.06		-0.55		0.4		30.1		30.5	
MCP-1	Monocyte chemoattractant protein-1	0.38		0.28		14.7		8.1		22.8	
IFN-γ	Interferon gamma	0.29		0.11		8.4		1.3		9.7	
MIP-1α	Macrophage inflammatory protein	0.21		0.14		4.5		2.0		6.4	

Table A123: Canonical loadings and explained variance (RNA-Seq, B-cells, Day 7, IMO variables). IMO variables with at least 25% of explained variance for the first two canonical variates are listed. GX: canonical loadings for canonical variate X, VX: % explained variance for canonical variate X, V1+V2: total % explained variance for both canonical variates. Values for CCA results with a poor cross validation score (< 0.70) are left blank. Sorted by maximum explained variance across treatments (V1+V2).

Gene ID	Gene Name	G1 (SV-AS03)	G1 (SV-PBS)	G2 (SV-AS03)	G2 (SV-PBS)	V1 (SV-AS03)	V1 (SV-PBS)	V2 (SV-AS03)	V2 (SV-PBS)	V1+V2 (SV-AS03)	V1+V2 (SV-PBS)
ENSG00000253712	RP11-697M17.2	0.89		-0.12		78.9		1.3		80.3	
ENSG00000256875	RP11-503G7.2	0.77		-0.39		59.9		15.5		75.3	
ENSG00000221535	AC027319.1	0.67		0.44		44.5		19.0		63.5	
ENSG00000038427	VCAN	-0.13		-0.77		1.8		59.7		61.5	
ENSG00000243469	AC018755.10	-0.19		0.74		3.6		54.2		57.8	
ENSG00000180644	PRF1	-0.23		0.72		5.5		51.4		56.9	
ENSG00000241431	RP11-17A4.1	-0.71		-0.14		50.0		1.9		51.8	
ENSG00000123689	G0S2	-0.69		0.06		48.0		0.4		48.4	
ENSG00000253326	RP11-261C10.6	0.55		-0.39		30.6		15.3		45.9	
ENSG00000233280	AC110491.1	-0.40		0.54		15.6		29.5		45.2	
ENSG00000162571	TTLL10	0.52		-0.38		27.1		14.2		41.4	
ENSG00000112742	TTK	-0.49		0.41		23.8		17.2		41.0	
ENSG00000237588	RP11-66D17.3	0.51		0.37		26.2		13.6		39.8	
ENSG00000239559	RPL37P2	-0.63		-0.04		39.3		0.2		39.5	
ENSG00000223551	TMSL4	-0.60		-0.13		36.1		1.6		37.6	
ENSG00000228655	AC096558.1	-0.58		0.17		34.0		2.9		36.9	
ENSG00000213590	RP11-229P13.2	0.51		0.26		26.2		6.9		33.1	

Gene ID	Gene Name	G1 (SV-AS03)	G1 (SV-PBS)	G2 (SV-AS03)	G2 (SV-PBS)	V1 (SV-AS03)	V1 (SV-PBS)	V2 (SV-AS03)	V2 (SV-PBS)	V1+V2 (SV-AS03)	V1+V2 (SV-PBS)
ENSG00000152939	MARVELD2	-0.05		0.57		0.3		32.8		33.1	
ENSG00000244734	HBB	-0.43		0.34		18.7		11.8		30.5	
ENSG00000182885	GPR97	0.28		0.46		7.9		21.2		29.1	
ENSG00000235613	CCDC55P1	0.38		0.32		14.5		10.1		24.6	
ENSG00000187951	ARHGAP11B	-0.40		0.27		16.1		7.2		23.4	
ENSG00000230850	BX571672.1	-0.38		0.19		14.1		3.7		17.8	
ENSG00000197249	SERPINA1	-0.25		-0.32		6.3		10.1		16.5	
ENSG00000230280	RP11-38O14.1	-0.39		0.07		15.1		0.5		15.6	
ENSG00000170345	FOS	-0.18		-0.32		3.2		10.4		13.6	
ENSG00000215086	RP11-522H2.3	-0.34		0.09		11.4		0.8		12.2	
ENSG00000011600	TYROBP	-0.26		-0.22		6.7		5.0		11.6	
ENSG00000242766	IGKV1D-17	-0.01		-0.28		0.0		7.9		7.9	
ENSG00000251209	AC024651.1	-0.14		0.22		2.0		5.0		7.0	
ENSG00000155428	TRIM74	0.10		-0.22		1.0		5.0		6.0	
ENSG00000127954	STEAP4	0.16		-0.11		2.7		1.1		3.8	
ENSG00000171051	FPR1	-0.12		-0.04		1.5		0.2		1.7	

Table A124: Canonical loadings and explained variance (RNA-Seq, B-cells, Day 28, Gene variables). Gene variables with at least 25% of explained variance for the first two canonical variates are listed. GX: canonical loadings for canonical variate X, VX: % explained variance for canonical variate X, V1+V2: total % explained variance for both canonical variates. Values for CCA results with a poor cross validation score ($<$ 0.70) are left blank. Sorted by maximum explained variance across treatments (V1+V2).

IMO Variable	IMO Variable Name	I1 (SV-AS03)	I1 (SV-PBS)	I2 (SV-AS03)	I2 (SV-PBS)	V1 (SV-AS03)	V1 (SV-PBS)	V2 (SV-AS03)	V2 (SV-PBS)	V1+V2 (SV-AS03)	V1+V2 (SV-PBS)
IL-12p70	Interleukin-12 p70	-0.95		0.25		90.2		6.4		96.6	
IL-10	Interleukin-10	-0.33		0.91		10.9		83.0		93.9	
HAI	Hemagglutination inhibition	0.71		0.59		50.8		35.0		85.8	
Nt	Microneutralization inhibition	0.73		0.55		53.9		30.3		84.2	
Eotaxin-1	Eotaxin-1	-0.32		0.79		10.4		62.5		72.9	
IL-6	Interleukin-6	-0.75		-0.23		56.8		5.4		62.2	
IL-8	Interleukin-8	-0.46		0.63		20.8		40.3		61.1	
IP-10	Interferon gamma-induced protein 10	0.41		0.61		16.9		36.9		53.8	
IL-1β	Interleukin-1 beta	-0.40		0.52		16.4		27.4		43.8	
TNF	Tumor necrosis factors	-0.30		0.58		8.7		33.3		42.1	
RANTES	Chemokine (C-C motif) ligand 5	-0.26		0.50		6.9		24.7		31.6	
IFN-γ	Interferon gamma	0.37		0.41		13.7		17.0		30.7	
MIP-1α	Macrophage inflammatory protein	-0.31		-0.40		9.8		16.0		25.8	
MCP-1	Monocyte chemoattractant protein-1	-0.39		0.21		15.3		4.4		19.7	

Table A125: Canonical loadings and explained variance (RNA-Seq, B-cells, Day 28, IMO variables). IMO variables with at least 25% of explained variance for the first two canonical variates are listed. GX: canonical loadings for canonical variate X, VX: % explained variance for canonical variate X, V1+V2: total % explained variance for both canonical variates. Values for CCA results with a poor cross validation score ($<$ 0.70) are left blank. Sorted by maximum explained variance across treatments (V1+V2).

Gene ID	Gene Name	G1 (SV-AS03)	G1 (SV-PBS)	G2 (SV-AS03)	G2 (SV-PBS)	V1 (SV-AS03)	V1 (SV-PBS)	V2 (SV-AS03)	V2 (SV-PBS)	V1+V2 (SV-AS03)	V1+V2 (SV-PBS)
ENSG00000156113	KCNMA1	0.97		0.05		94.3		0.3		94.5	
ENSG00000050628	PTGER3	-0.92		0.24		84.4		5.9		90.2	
ENSG00000115594	IL1R1	-0.93		0.16		87.3		2.6		89.9	
ENSG00000229754	CXCR2P1	0.93		-0.16		85.7		2.5		88.2	
ENSG00000173369	C1QB	0.93		0.13		86.3		1.8		88.1	
ENSG00000138646	HERC5	0.93		-0.11		85.6		1.2		86.8	
ENSG00000169994	MYO7B	0.89		-0.15		80.0		2.2		82.3	
ENSG00000026751	SLAMF7	0.90		-0.08		80.9		0.7		81.5	
ENSG00000187608	ISG15	0.90		0.07		80.4		0.5		81.0	
ENSG00000185745	IFIT1	0.89		-0.09		79.9		0.8		80.7	
ENSG00000166278	C2	0.86		-0.23		74.5		5.3		79.8	
ENSG00000126262	FFAR2	0.87		0.14		75.9		1.9		77.8	
ENSG00000135114	OASL	0.88		-0.02		76.9		0.1		77.0	
ENSG00000225886	RP11-288L9.4	0.80		0.32		64.6		10.3		74.9	
ENSG00000133321	RARRES3	0.86		-0.03		74.6		0.1		74.7	
ENSG00000168899	VAMP5	0.85		-0.13		72.9		1.7		74.6	
ENSG00000126709	IFI6	0.85		-0.16		72.1		2.4		74.5	
ENSG00000115590	IL1R2	-0.83		0.25		68.4		6.1		74.5	
ENSG00000064300	NGFR	0.85		-0.09		73.0		0.8		73.9	
ENSG00000165806	CASP7	0.85		-0.12		72.4		1.4		73.8	
ENSG00000166444	ST5	-0.85		0.08		72.9		0.6		73.4	
ENSG00000111335	OAS2	0.86		-0.04		73.2		0.2		73.4	
ENSG00000185885	IFITM1	0.85		0.06		72.8		0.3		73.2	
ENSG00000135636	DYSF	0.82		-0.22		67.7		4.7		72.4	
ENSG00000188282	RUFY4	0.85		0.04		72.0		0.1		72.1	
ENSG00000125148	MT2A	0.84		0.09		69.9		0.8		70.7	
ENSG00000025708	TYMP	0.83		-0.08		69.7		0.7		70.4	
ENSG00000218537	AP000350.4	-0.60		0.59		35.5		34.5		70.0	
ENSG00000007129	CEACAM21	0.80		-0.23		64.4		5.3		69.7	
ENSG00000100911	PSME2	0.80		-0.21		64.2		4.6		68.8	
ENSG00000111181	SLC6A12	0.82		0.10		67.5		1.0		68.5	
ENSG00000163568	AIM2	0.78		-0.28		60.3		8.1		68.4	
ENSG00000147408	CSGALNACT1	-0.69		-0.45		47.4		20.1		67.5	
ENSG00000233355	RP11-343J24.1	-0.81		-0.15		64.9		2.2		67.2	
ENSG00000131203	IDO1	0.78		0.22		61.2		4.9		66.2	
ENSG00000119917	IFIT3	0.80		-0.06		64.3		0.4		64.7	
ENSG00000172183	ISG20	0.80		0.04		64.5		0.1		64.6	
ENSG00000149131	SERPING1	0.79		-0.16		62.0		2.5		64.6	
ENSG00000137628	DDX60	0.77		-0.23		59.2		5.4		64.5	
ENSG00000121858	TNFSF10	0.79		-0.12		62.7		1.4		64.1	
ENSG00000137965	IFI44	0.78		-0.19		60.1		3.6		63.7	
ENSG00000137959	IFI44L	0.78		-0.17		60.6		3.0		63.6	
ENSG00000119922	IFIT2	0.78		-0.17		60.6		2.9		63.5	
ENSG00000253882	RP11-61L23.2	0.79		-0.01		63.1		0.0		63.1	
ENSG00000130489	SCO2	0.79		-0.06		62.7		0.4		63.0	
ENSG00000198829	SUCNR1	0.77		-0.21		58.6		4.4		63.0	
ENSG00000137193	PIM1	0.78		0.12		61.6		1.5		63.0	
ENSG00000020577	SAMD4A	0.79		-0.04		62.8		0.2		63.0	
ENSG00000100342	APOL1	0.79		-0.09		62.1		0.8		62.9	
ENSG00000159189	C1QC	0.78		-0.12		60.7		1.4		62.1	
ENSG00000238000	RP11-274E7.2	0.65		-0.45		41.6		20.3		62.0	

Gene ID	Gene Name	G1 (SV-AS03)	G1 (SV-PBS)	G2 (SV-AS03)	G2 (SV-PBS)	V1 (SV-AS03)	V1 (SV-PBS)	V2 (SV-AS03)	V2 (SV-PBS)	V1+V2 (SV-AS03)	V1+V2 (SV-PBS)
ENSG00000111331	OAS3	0.78		-0.10		60.8		1.1		61.9	
ENSG00000172159	FRMD3	0.78		-0.10		60.6		1.0		61.6	
ENSG00000173821	RNF213	0.76		-0.18		58.2		3.2		61.4	
ENSG00000145287	PLAC8	0.75		-0.23		56.0		5.4		61.4	
ENSG00000240065	PSMB9	0.77		-0.07		60.0		0.5		60.5	
ENSG00000165178	NCF1C	0.78		-0.04		60.3		0.2		60.5	
ENSG00000068079	IFI35	0.78		0.03		60.3		0.1		60.4	
ENSG00000101017	CD40	0.77		-0.08		59.7		0.6		60.3	
ENSG00000177409	SAMD9L	0.74		-0.24		54.3		5.8		60.1	
ENSG00000165092	ALDH1A1	0.71		0.32		49.8		10.1		59.9	
ENSG00000204267	TAP2	0.75		-0.18		56.4		3.1		59.5	
ENSG00000163736	PPBP	-0.08		-0.77		0.7		58.8		59.5	
ENSG00000204060	FOXO6	-0.53		0.56		28.2		31.3		59.4	
ENSG00000152766	ANKRD22	0.76		-0.11		57.9		1.3		59.2	
ENSG00000169245	CXCL10	0.77		0.04		58.9		0.1		59.1	
ENSG00000158517	NCF1	0.76		-0.09		58.0		0.9		58.9	
ENSG00000183734	ASCL2	0.76		-0.09		57.6		0.8		58.3	
ENSG00000157601	MX1	0.76		-0.06		57.7		0.4		58.1	
ENSG00000198959	TGM2	0.72		-0.25		51.4		6.4		57.9	
ENSG00000142089	IFITM3	0.76		-0.07		57.1		0.5		57.7	
ENSG00000162654	GBP4	0.74		-0.15		54.4		2.3		56.8	
ENSG00000177989	ODF3B	0.74		-0.13		54.1		1.8		55.9	
ENSG00000255760	RP11-428G5.5	0.64		-0.39		40.4		15.1		55.5	
ENSG00000124256	ZBP1	0.74		0.00		55.1		0.0		55.1	
ENSG00000197506	SLC28A3	-0.73		-0.12		53.6		1.4		55.0	
ENSG00000128284	APOL3	0.71		-0.21		50.4		4.6		55.0	
ENSG00000182487	NCF1B	0.73		-0.11		53.4		1.2		54.6	
ENSG00000136514	RTP4	0.72		0.13		52.4		1.8		54.2	
ENSG00000156587	UBE2L6	0.72		-0.13		52.5		1.6		54.1	
ENSG00000100276	RASL10A	-0.64		-0.35		41.5		12.1		53.6	
ENSG00000132530	XAF1	0.72		-0.12		52.1		1.5		53.6	
ENSG00000117226	GBP3	0.68		-0.26		46.4		6.9		53.3	
ENSG00000162645	GBP2	0.72		-0.12		51.8		1.4		53.1	
ENSG00000128335	APOL2	0.70		-0.20		49.1		4.1		53.1	
ENSG00000140105	WARS	0.72		-0.11		51.9		1.2		53.1	
ENSG00000162512	SDC3	0.71		-0.18		49.8		3.2		53.0	
ENSG00000198019	FCGR1B	0.73		0.02		52.7		0.0		52.8	
ENSG00000108679	LGALS3BP	0.71		-0.15		50.4		2.2		52.6	
ENSG00000148175	STOM	0.70		-0.16		49.6		2.6		52.3	
ENSG00000154188	ANGPT1	-0.52		-0.50		26.8		25.4		52.2	
ENSG00000163412	EIF4E3	0.68		-0.25		46.1		6.1		52.2	
ENSG00000133106	EPSTI1	0.69		-0.22		47.1		4.9		52.0	
ENSG00000118557	PMFBP1	-0.26		0.67		7.0		44.8		51.8	
ENSG00000256948	RP11-598F7.3	0.47		-0.54		22.0		29.7		51.7	
ENSG00000170581	STAT2	0.71		-0.11		50.0		1.3		51.3	
ENSG00000179044	EXOC3L1	0.71		-0.00		50.8		0.0		50.8	
ENSG00000168062	BATF2	0.71		-0.06		50.4		0.3		50.7	
ENSG00000143801	PSEN2	0.70		-0.11		49.6		1.1		50.7	
ENSG00000221955	SLC12A8	0.71		0.03		50.4		0.1		50.5	
ENSG00000253981	RP11-556O5.4	-0.66		-0.26		43.5		6.7		50.2	
ENSG00000154451	GBP5	0.70		-0.10		48.9		1.0		49.9	

Gene ID	Gene Name	G1 (SV-AS03)	G1 (SV-PBS)	G2 (SV-AS03)	G2 (SV-PBS)	V1 (SV-AS03)	V1 (SV-PBS)	V2 (SV-AS03)	V2 (SV-PBS)	V1+V2 (SV-AS03)	V1+V2 (SV-PBS)
ENSG00000176358	TAC4	0.60		-0.37		35.8		13.5		49.2	
ENSG00000138496	PARP9	0.68		-0.15		46.6		2.4		49.0	
ENSG00000100453	GZMB	-0.44		0.54		19.5		29.4		48.9	
ENSG00000173193	PARP14	0.66		-0.24		43.1		5.8		48.9	
ENSG00000185507	IRF7	0.70		0.04		48.6		0.1		48.8	
ENSG00000256262	RP11-117B7.1	0.64		-0.27		41.3		7.2		48.4	
ENSG00000167371	PRRT2	0.69		-0.09		47.6		0.7		48.3	
ENSG00000104970	KIR3DX1	0.67		0.19		44.2		3.4		47.7	
ENSG00000120217	CD274	0.64		-0.25		41.4		6.3		47.7	
ENSG00000130487	KLHDC7B	0.68		-0.04		46.6		0.2		46.7	
ENSG00000221963	APOL6	0.67		-0.15		44.4		2.3		46.7	
ENSG00000117228	GBP1	0.67		-0.12		44.6		1.4		46.0	
ENSG00000115415	STAT1	0.63		-0.26		39.1		6.8		45.9	
ENSG00000163840	DTX3L	0.64		-0.19		41.3		3.5		44.8	
ENSG00000203734	ECT2L	-0.05		-0.67		0.2		44.4		44.6	
ENSG00000225492	GBP1P1	0.65		-0.15		42.3		2.1		44.5	
ENSG00000203814	HIST2H2BF	0.64		-0.17		41.2		3.0		44.2	
ENSG00000225131	PSME2P2	0.63		-0.20		39.4		4.1		43.5	
ENSG00000129673	AANAT	0.64		-0.17		40.5		2.9		43.4	
ENSG00000134321	RSAD2	0.64		-0.15		40.5		2.4		42.9	
ENSG00000136960	ENPP2	0.64		0.12		41.4		1.4		42.8	
ENSG00000089169	RPH3A	-0.61		0.24		36.8		5.7		42.5	
ENSG00000002549	LAP3	0.65		-0.04		42.1		0.2		42.3	
ENSG00000136867	SLC31A2	0.64		-0.09		41.1		0.8		41.9	
ENSG00000180739	S1PR5	-0.21		0.61		4.6		37.2		41.8	
ENSG00000186187	ZNRF1	-0.62		0.18		38.2		3.3		41.5	
ENSG00000247275	AL160008.1	0.64		0.06		40.8		0.4		41.1	
ENSG00000125347	IRF1	0.63		-0.12		39.5		1.5		41.0	
ENSG00000197121	PGAP1	0.63		-0.10		39.8		1.1		40.8	
ENSG00000198719	DLL1	0.63		0.05		40.1		0.3		40.3	
ENSG00000055130	CUL1	0.63		0.03		40.2		0.1		40.2	
ENSG00000150337	FCGR1A	0.63		-0.04		39.9		0.2		40.1	
ENSG00000168394	TAP1	0.62		-0.10		39.0		1.0		40.0	
ENSG00000010030	ETV7	0.60		-0.21		35.5		4.5		40.0	
ENSG00000214872	SMTNL1	0.63		-0.01		39.9		0.0		39.9	
ENSG00000159674	SPON2	-0.13		0.61		1.8		36.7		38.5	
ENSG00000116663	FBXO6	0.62		0.00		38.2		0.0		38.2	
ENSG00000234518	RP5-837D10.2	0.62		-0.01		38.1		0.0		38.1	
ENSG00000164684	ZNF704	-0.59		0.16		35.0		2.7		37.7	
ENSG00000134326	CMPK2	0.61		-0.04		37.5		0.2		37.6	
ENSG00000243649	CFB	0.61		0.03		37.0		0.1		37.1	
ENSG00000224083	RP11-39K24.4	0.21		-0.56		4.6		31.3		35.9	
ENSG00000236254	RP11-39K24.8	0.27		-0.53		7.1		27.6		34.7	
ENSG00000111186	WNT5B	-0.58		-0.09		33.9		0.8		34.7	
ENSG00000168497	SDPR	-0.13		-0.57		1.7		32.8		34.5	
ENSG00000121966	CXCR4	-0.53		0.24		28.3		5.8		34.1	
ENSG00000140511	HAPLN3	0.58		-0.02		33.8		0.0		33.9	
ENSG00000169255	B3GALNT1	-0.11		0.57		1.1		32.5		33.7	
ENSG00000183762	KREMEN1	0.57		0.11		32.4		1.2		33.6	
ENSG00000237989	AP001046.5	0.58		0.05		33.1		0.3		33.3	
ENSG00000140853	NLRC5	0.56		-0.15		31.0		2.3		33.3	

Gene ID	Gene Name	G1 (SV-AS03)	G1 (SV-PBS)	G2 (SV-AS03)	G2 (SV-PBS)	V1 (SV-AS03)	V1 (SV-PBS)	V2 (SV-AS03)	V2 (SV-PBS)	V1+V2 (SV-AS03)	V1+V2 (SV-PBS)
ENSG00000240710	RP11-430C7.4	0.54		-0.20		29.4		3.8		33.3	
ENSG00000145555	MYO10	-0.57		0.07		32.1		0.4		32.6	
ENSG00000175084	DES	0.53		-0.20		28.3		4.2		32.4	
ENSG00000162614	NEXN	0.42		-0.37		17.9		13.6		31.5	
ENSG00000181036	FCRL6	0.39		0.38		15.1		14.4		29.6	
ENSG00000100336	APOL4	0.54		-0.07		28.6		0.5		29.1	
ENSG00000158156	XKR8	0.53		0.12		27.7		1.3		29.1	
ENSG00000004468	CD38	0.54		0.04		28.8		0.1		28.9	
ENSG00000179388	EGR3	0.27		0.45		7.5		20.1		27.6	
ENSG00000206942	SNORA9_206942	-0.52		-0.02		27.5		0.0		27.5	
ENSG00000180644	PRF1	-0.15		0.50		2.1		24.8		27.0	
ENSG00000214076	CPSF1P1	-0.16		0.49		2.6		24.2		26.8	
ENSG00000167779	IGFBP6	-0.47		0.22		22.0		4.7		26.7	
ENSG00000120586	MRC1	-0.07		0.51		0.5		25.7		26.2	
ENSG00000141854	AC022098.1	-0.26		0.44		6.6		19.3		25.9	
ENSG00000169184	MN1	-0.50		-0.02		25.1		0.0		25.2	
ENSG00000233029	RP11-439A17.9	0.47		-0.17		22.1		2.8		24.9	
ENSG00000196436	NPIPL2	-0.18		0.46		3.3		21.5		24.8	
ENSG00000100385	IL2RB	-0.40		0.29		16.2		8.6		24.8	
ENSG00000130720	FIBCD1	-0.27		0.41		7.6		16.9		24.5	
ENSG00000145685	LHFPL2	0.42		-0.26		17.5		6.9		24.5	
ENSG00000138378	STAT4	-0.08		0.49		0.6		23.5		24.1	
ENSG00000219607	PPP1R3G	-0.45		-0.20		19.9		4.1		24.0	
ENSG00000162433	AK4	0.42		0.25		17.9		6.0		23.9	
ENSG00000228060	RP11-15J6.1	-0.10		-0.47		0.9		22.5		23.4	
ENSG00000099250	NRP1	-0.36		-0.32		12.7		10.5		23.2	
ENSG00000205084	TMEM231	-0.47		0.00		22.4		0.0		22.4	
ENSG00000162772	ATF3	0.42		-0.22		17.6		4.7		22.3	
ENSG00000130300	PLVAP	-0.46		-0.09		21.5		0.7		22.2	
ENSG00000167550	RHEBL1	0.34		-0.32		11.7		10.2		21.8	
ENSG00000073861	TBX21	-0.45		0.13		20.0		1.7		21.7	
ENSG00000158714	SLAMF8	0.45		0.11		20.3		1.2		21.4	
ENSG00000203818	HIST2H3PS2	0.19		0.42		3.7		17.6		21.3	
ENSG00000074181	NOTCH3	-0.41		0.20		16.6		4.0		20.5	
ENSG00000235750	KIAA0040	0.45		-0.02		20.3		0.0		20.3	
ENSG00000120738	EGR1	-0.44		0.10		19.2		0.9		20.1	
ENSG00000067057	PFKP	0.44		-0.05		19.5		0.3		19.7	
ENSG00000252331	AP002812.1	0.14		-0.42		2.0		17.4		19.4	
ENSG00000231528	NCRNA00256A	0.41		-0.16		16.7		2.6		19.3	
ENSG00000230225	RP11-39K24.15	-0.17		-0.40		2.8		16.3		19.1	
ENSG00000249835	CTC-348L14.1	-0.44		-0.03		19.0		0.1		19.1	
ENSG00000248201	RP4-671G15.3	-0.40		-0.16		16.4		2.7		19.0	
ENSG00000139211	AMIGO2	0.34		-0.27		11.5		7.3		18.8	
ENSG00000131355	EMR3	-0.43		-0.07		18.1		0.4		18.5	
ENSG00000227028	AC007254.3	0.37		-0.20		13.9		3.9		17.8	
ENSG00000160183	TMPRSS3	0.11		0.39		1.3		15.0		16.4	
ENSG00000173762	CD7	0.20		0.35		4.0		12.4		16.3	
ENSG00000224875	AC083949.1	0.31		-0.26		9.5		6.7		16.2	
ENSG00000113368	LMNB1	0.38		0.08		14.5		0.7		15.2	
ENSG00000235865	C9orf31	-0.36		-0.15		12.9		2.3		15.2	
ENSG00000131187	F12	-0.37		-0.10		13.7		0.9		14.6	

Gene ID	Gene Name	G1 (SV-AS03)	G1 (SV-PBS)	G2 (SV-AS03)	G2 (SV-PBS)	V1 (SV-AS03)	V1 (SV-PBS)	V2 (SV-AS03)	V2 (SV-PBS)	V1+V2 (SV-AS03)	V1+V2 (SV-PBS)
ENSG00000123689	G0S2	-0.32		-0.19		10.5		3.7		14.2	
ENSG00000090104	RGS1	-0.32		-0.18		10.0		3.4		13.3	
ENSG00000139174	PRICKLE1	0.36		-0.01		13.2		0.0		13.2	
ENSG00000236567	RP11-39K24.7	0.36		-0.05		12.8		0.3		13.1	
ENSG00000069399	BCL3	0.32		0.16		10.5		2.5		13.1	
ENSG00000185499	MUC1	0.32		-0.16		10.5		2.5		13.0	
ENSG00000184557	SOCS3	0.33		-0.12		11.0		1.5		12.5	
ENSG00000054690	PLEKHH1	0.32		-0.13		10.2		1.8		11.9	
ENSG00000254017	IGHEP2	0.32		-0.14		10.0		1.8		11.8	
ENSG00000165702	GFI1B	0.01		-0.34		0.0		11.7		11.7	
ENSG00000205978	NYNRIN	-0.22		0.26		4.7		6.6		11.2	
ENSG00000164292	RHOBTB3	0.33		0.06		10.7		0.4		11.2	
ENSG00000198520	C1orf228	-0.28		0.15		8.1		2.2		10.3	
ENSG00000183019	C19orf59	-0.29		-0.13		8.4		1.8		10.2	
ENSG00000138134	STAMBPL1	0.32		0.00		10.0		0.0		10.0	
ENSG00000110092	CCND1	0.28		-0.15		7.6		2.2		9.8	
ENSG00000231865	AP000936.3	0.23		-0.19		5.4		3.5		8.9	
ENSG00000185950	IRS2	-0.28		0.11		7.6		1.3		8.8	
ENSG00000236790	NCRNA00299	-0.24		0.17		5.8		3.0		8.8	
ENSG00000112299	VNN1	-0.16		0.25		2.4		6.2		8.6	
ENSG00000135074	ADAM19	0.24		0.15		6.0		2.2		8.2	
ENSG00000145365	TIFA	0.26		-0.09		6.9		0.8		7.8	
ENSG00000183087	GAS6	0.28		0.02		7.7		0.0		7.8	
ENSG00000156127	BATF	0.24		-0.15		5.6		2.2		7.7	
ENSG00000229436	AC073850.6	-0.13		0.22		1.7		5.0		6.7	
ENSG00000110076	NRXN2	0.00		-0.24		0.0		5.9		5.9	
ENSG00000110944	IL23A	0.17		0.16		2.9		2.4		5.4	
ENSG00000125869	C20orf103	-0.20		0.11		3.9		1.3		5.2	
ENSG00000166220	C10orf27	0.16		-0.13		2.7		1.6		4.3	
ENSG00000163814	CDCP1	0.20		0.01		4.1		0.0		4.1	
ENSG00000256049	PADI6	-0.10		0.11		1.1		1.2		2.2	
ENSG00000136630	HLX	-0.01		0.15		0.0		2.1		2.1	
ENSG00000139626	ITGB7	-0.02		0.14		0.1		1.9		2.0	
ENSG00000161647	MPP3	-0.12		-0.06		1.4		0.3		1.7	
ENSG00000175505	CLCF1	-0.03		-0.12		0.1		1.4		1.5	
ENSG00000143546	S100A8	-0.03		-0.11		0.1		1.3		1.3	
ENSG00000169385	RNASE2	-0.09		-0.00		0.9		0.0		0.9	
ENSG00000101187	SLCO4A1	-0.07		0.02		0.5		0.0		0.5	
ENSG00000059804	SLC2A3	-0.05		0.05		0.2		0.2		0.5	
ENSG00000154025	SLC5A10	0.03		0.04		0.1		0.1		0.2	
ENSG00000226067	RP11-403I13.7	0.01		-0.04		0.0		0.1		0.1	
ENSG00000151693	ASAP2	0.03		0.00		0.1		0.0		0.1	

Table A126: Canonical loadings and explained variance (RNA-Seq, Dendritic cells, Day 1, Gene variables). Gene variables with at least 25% of explained variance for the first two canonical variates are listed. GX: canonical loadings for canonical variate X, VX: % explained variance for canonical variate X, V1+V2: total % explained variance for both canonical variates. Values for CCA results with a poor cross validation score (< 0.70) are left blank. Sorted by maximum explained variance across treatments (V1+V2).

IMO Variable	IMO Variable Name	I1 (SV-AS03)	I1 (SV-PBS)	I2 (SV-AS03)	I2 (SV-PBS)	V1 (SV-AS03)	V1 (SV-PBS)	V2 (SV-AS03)	V2 (SV-PBS)	V1+V2 (SV-AS03)	V1+V2 (SV-PBS)
MIP-1α	Macrophage inflammatory protein	0.91		-0.27		82.8		7.5		90.3	
TNF	Tumor necrosis factors	-0.46		0.74		21.5		55.2		76.7	
IL-12p70	Interleukin-12 p70	0.08		0.86		0.6		74.8		75.4	
IL-10	Interleukin-10	0.31		0.80		9.9		64.2		74.1	
IL-1β	Interleukin-1 beta	0.54		0.66		29.1		43.2		72.3	
IL-6	Interleukin-6	0.48		0.68		22.8		46.3		69.1	
IP-10	Interferon gamma-induced protein 10	0.82		-0.09		67.5		0.9		68.4	
Eotaxin-1	Eotaxin-1	-0.66		0.13		43.3		1.8		45.1	
IL-8	Interleukin-8	-0.08		0.63		0.6		39.6		40.2	
MCP-1	Monocyte chemoattractant protein-1	0.08		0.37		0.7		13.5		14.2	
RANTES	Chemokine (C-C motif) ligand 5	0.30		0.22		9.2		4.7		13.9	
IFN-γ	Interferon gamma	-0.32		0.17		10.3		2.8		13.0	
Nt	Microneutralization inhibition	-0.31		0.07		9.9		0.5		10.4	

Table A127: Canonical loadings and explained variance (RNA-Seq, Dendritic cells, Day 1, IMO variables). IMO variables with at least 25% of explained variance for the first two canonical variates are listed. GX: canonical loadings for canonical variate X, VX: % explained variance for canonical variate X, V1+V2: total % explained variance for both canonical variates. Values for CCA results with a poor cross validation score (< 0.70) are left blank. Sorted by maximum explained variance across treatments (V1+V2).

Gene ID	Gene Name	G1 (SV-AS03)	G1 (SV-PBS)	G2 (SV-AS03)	G2 (SV-PBS)	V1 (SV-AS03)	V1 (SV-PBS)	V2 (SV-AS03)	V2 (SV-PBS)	V1+V2 (SV-AS03)	V1+V2 (SV-PBS)
ENSG00000188536	HBA2	-1.00	-0.14	-0.06	-0.51	99.2	2.0	0.3	26.2	99.5	28.2
ENSG00000244734	HBB	-0.99	-0.43	0.11	-0.34	98.1	18.2	1.1	11.2	99.2	29.4
ENSG00000206172	HBA1	-0.99	-0.52	0.05	-0.36	97.5	26.7	0.2	13.2	97.7	39.9
ENSG00000158856	EPB49	-0.24	0.40	0.82	-0.15	5.6	16.4	68.0	2.1	73.6	18.5
ENSG00000123689	G0S2	-0.04	-0.44	0.83	0.13	0.2	19.2	69.6	1.6	69.8	20.7
ENSG00000165092	ALDH1A1	-0.57	-0.55	0.58	0.33	32.7	29.8	34.1	11.1	66.8	40.9
ENSG00000088827	SIGLEC1	-0.11	-0.09	0.80	0.01	1.2	0.9	64.5	0.0	65.7	0.9
ENSG00000160179	ABCG1	0.31	-0.79	0.26	0.16	9.6	62.9	6.7	2.7	16.3	65.6
ENSG00000165029	ABCA1	0.14	-0.67	-0.05	0.45	2.1	45.0	0.3	20.0	2.4	65.0
ENSG00000163568	AIM2	-0.32	-0.49	0.71	0.52	10.2	23.9	50.9	26.9	61.1	50.8
ENSG00000225886	RP11-288L9.4	0.14	-0.75	0.62	0.16	2.0	56.1	38.4	2.5	40.4	58.6
ENSG00000125740	FOSB	0.15	0.30	0.74	-0.12	2.4	8.9	54.9	1.4	57.3	10.3
ENSG00000213993	AC092067.1	-0.73	-0.17	0.01	-0.25	53.3	2.8	0.0	6.2	53.3	9.0
ENSG00000137959	IFI44L	-0.07	-0.27	0.72	0.30	0.5	7.2	51.5	8.9	52.0	16.1
ENSG00000235847	LDHAP7	-0.67	-0.33	0.26	-0.35	44.4	11.0	6.5	12.3	50.9	23.3
ENSG00000211899	IGHM	0.16	-0.62	0.56	-0.35	2.6	38.6	31.4	12.1	33.9	50.7
ENSG00000177807	KCNJ10	0.13	-0.56	-0.27	-0.43	1.6	31.1	7.1	18.6	8.8	49.7
ENSG00000108679	LGALS3BP	-0.09	-0.05	0.70	0.03	0.8	0.2	48.7	0.1	49.5	0.3
ENSG00000090104	RGS1	0.03	0.29	0.70	-0.07	0.1	8.2	49.0	0.4	49.1	8.7
ENSG00000252331	AP002812.1	-0.16	-0.35	-0.42	-0.59	2.6	12.5	17.8	34.7	20.4	47.2
ENSG00000162512	SDC3	-0.05	-0.64	0.34	0.16	0.3	40.9	11.8	2.5	12.1	43.4
ENSG00000006075	CCL3	0.49	0.20	0.43	-0.43	23.7	4.2	18.2	18.6	41.9	22.8
ENSG00000152766	ANKRD22	0.07	-0.52	0.42	0.38	0.5	27.0	17.4	14.5	18.0	41.6
ENSG00000179750	APOBEC3B	-0.21	-0.62	0.60	0.13	4.5	38.3	36.0	1.6	40.5	39.9
ENSG00000159189	C1QC	-0.47	-0.39	0.39	0.50	22.1	15.5	15.1	24.8	37.2	40.3
ENSG00000149131	SERPING1	-0.28	-0.54	0.56	0.14	7.8	29.2	31.4	2.0	39.2	31.2
ENSG00000252197	AC091047.1	-0.16	-0.34	-0.10	-0.52	2.5	11.6	0.9	27.3	3.4	38.9

Gene ID	Gene Name	G1 (SV-AS03)	G1 (SV-PBS)	G2 (SV-AS03)	G2 (SV-PBS)	V1 (SV-AS03)	V1 (SV-PBS)	V2 (SV-AS03)	V2 (SV-PBS)	V1+V2 (SV-AS03)	V1+V2 (SV-PBS)
ENSG00000187492	CDHR4	0.58	-0.01	-0.22	-0.06	33.2	0.0	4.7	0.4	37.9	0.4
ENSG00000251826	AL365332.1	-0.27	-0.37	-0.42	-0.48	7.1	13.9	17.6	23.4	24.7	37.3
ENSG00000255478	RP11-867O8.5	-0.29	-0.19	-0.47	-0.35	8.2	3.5	22.0	12.0	30.2	15.4
ENSG00000120738	EGR1	0.01	-0.26	0.54	-0.22	0.0	6.5	29.0	4.7	29.0	11.2
ENSG00000173369	C1QB	-0.51	-0.47	-0.13	0.23	25.7	22.4	1.6	5.5	27.4	27.8
ENSG00000252318	AC097532.1	-0.29	-0.21	-0.16	-0.48	8.5	4.2	2.7	23.5	11.2	27.7
ENSG00000185745	IFIT1	-0.04	-0.35	0.52	0.21	0.2	12.2	27.3	4.4	27.5	16.5
ENSG00000214548	MEG3	0.44	0.18	-0.24	0.29	19.2	3.3	5.6	8.4	24.7	11.7
ENSG00000198435	NRARP	0.19	-0.11	0.34	-0.47	3.5	1.2	11.5	22.4	15.0	23.6
ENSG00000137441	FGFBP2	0.33	-0.29	0.31	0.15	11.2	8.7	9.8	2.3	21.0	11.0
ENSG00000235297	RP11-504H5.1	0.04	-0.29	-0.08	-0.35	0.1	8.2	0.6	12.3	0.8	20.4
ENSG00000237039	AC018738.2	-0.11	-0.23	0.13	-0.38	1.3	5.3	1.8	14.2	3.0	19.6
ENSG00000223825	AC079354.4	0.15	-0.20	0.23	-0.37	2.3	4.2	5.5	13.7	7.8	17.9
ENSG00000253984	CTD-3056O22.1	0.17	-0.38	0.04	-0.17	2.8	14.4	0.2	3.0	3.0	17.4
ENSG00000168685	IL7R	-0.01	0.41	0.23	-0.01	0.0	16.5	5.5	0.0	5.5	16.5
ENSG00000019169	MARCO	-0.11	-0.33	0.39	-0.00	1.1	11.0	14.9	0.0	16.0	11.0
ENSG00000257531	RP3-405J10.2	-0.24	0.08	0.30	-0.35	5.7	0.6	8.8	12.0	14.5	12.6
ENSG00000196656	AC004057.1	0.09	0.17	-0.35	-0.20	0.8	3.0	12.5	4.0	13.3	7.0
ENSG00000103196	CRISPLD2	0.27	0.16	0.22	0.18	7.2	2.7	5.0	3.1	12.2	5.8
ENSG00000244479	RP4-798C17.6	-0.06	-0.04	-0.19	-0.33	0.4	0.2	3.6	11.0	4.0	11.2
ENSG00000143416	SELENBP1	-0.07	0.16	0.32	0.20	0.5	2.4	10.2	3.8	10.8	6.2
ENSG00000140545	MFGE8	0.18	-0.04	0.25	-0.11	3.1	0.2	6.4	1.1	9.5	1.3
ENSG00000230666	CEACAM22P	0.22	-0.15	-0.03	-0.25	4.7	2.2	0.1	6.1	4.8	8.3
ENSG00000248791	CTD-2165H16.3	0.06	0.01	-0.21	-0.08	0.4	0.0	4.5	0.7	4.8	0.7
ENSG00000122877	EGR2	-0.15	0.16	0.04	0.08	2.3	2.5	0.2	0.6	2.4	3.2
ENSG00000203818	HIST2H3PS2	-0.05	-0.17	0.16	-0.05	0.2	2.8	2.5	0.2	2.7	3.0
ENSG00000160183	TMPRSS3	0.06	-0.04	0.07	0.15	0.3	0.2	0.5	2.1	0.9	2.3

Table A128: Canonical loadings and explained variance (RNA-Seq, Dendritic cells, Day 3, Gene variables). Gene variables with at least 25% of explained variance for the first two canonical variates are listed. GX: canonical loadings for canonical variate X, VX: % explained variance for canonical variate X, V1+V2: total % explained variance for both canonical variates. Values for CCA results with a poor cross validation score ($<$ 0.70) are left blank. Sorted by maximum explained variance across treatments (V1+V2).

IMO Variable	IMO Variable Name	I1 (SV-AS03)	I1 (SV-PBS)	I2 (SV-AS03)	I2 (SV-PBS)	V1 (SV-AS03)	V1 (SV-PBS)	V2 (SV-AS03)	V2 (SV-PBS)	V1+V2 (SV-AS03)	V1+V2 (SV-PBS)
IL-12p70	Interleukin-12 p70	0.62	0.59	0.30	0.75	38.8	34.6	9.2	57.0	48.0	91.6
IL-10	Interleukin-10	0.07	-0.30	0.09	0.89	0.6	9.0	0.8	78.5	1.4	87.5
MIP-1α	Macrophage inflammatory protein	0.11	-0.88	-0.18	0.04	1.1	77.6	3.2	0.1	4.4	77.7
IFN-γ	Interferon gamma	0.43	-0.40	-0.35	0.77	18.2	16.0	12.5	59.0	30.8	75.1
TNF	Tumor necrosis factors	0.17	-0.39	0.42	0.77	2.8	15.5	17.9	59.3	20.7	74.8
IP-10	Interferon gamma-induced protein 10	-0.19	-0.71	0.78	0.04	3.5	50.5	61.2	0.2	64.7	50.7
IL-1β	Interleukin-1 beta	0.09	-0.30	-0.32	0.74	0.8	9.1	9.9	55.0	10.7	64.1
MCP-1	Monocyte chemoattractant protein-1	-0.62	-0.44	-0.48	-0.15	38.5	19.6	22.9	2.3	61.3	21.8
IL-8	Interleukin-8	0.02	-0.43	-0.25	0.65	0.0	18.4	6.3	42.3	6.3	60.7
Nt	Microneutralization inhibition	-0.37	-0.45	-0.35	0.60	13.9	20.6	12.5	36.4	26.4	57.0
IL-6	Interleukin-6	0.37	-0.50	-0.37	0.57	13.9	24.5	13.4	32.3	27.3	56.8
Eotaxin-1	Eotaxin-1	-0.52	-0.23	0.26	-0.13	27.3	5.5	6.7	1.7	34.1	7.2
RANTES	Chemokine (C-C motif) ligand 5	-0.52	-0.23	-0.03	-0.44	27.2	5.1	0.1	19.1	27.3	24.2

IMO Variable	IMO Variable Name	I1 (SV-AS03)	I1 (SV-PBS)	I2 (SV-AS03)	I2 (SV-PBS)	V1 (SV-AS03)	V1 (SV-PBS)	V2 (SV-AS03)	V2 (SV-PBS)	V1+V2 (SV-AS03)	V1+V2 (SV-PBS)

Table A129: Canonical loadings and explained variance (RNA-Seq, Dendritic cells, Day 3, IMO variables). IMO variables with at least 25% of explained variance for the first two canonical variates are listed. GX: canonical loadings for canonical variate X, VX: % explained variance for canonical variate X, V1+V2: total % explained variance for both canonical variates. Values for CCA results with a poor cross validation score (< 0.70) are left blank. Sorted by maximum explained variance across treatments (V1+V2).

Gene ID	Gene Name	G1 (SV-AS03)	G1 (SV-PBS)	G2 (SV-AS03)	G2 (SV-PBS)	V1 (SV-AS03)	V1 (SV-PBS)	V2 (SV-AS03)	V2 (SV-PBS)	V1+V2 (SV-AS03)	V1+V2 (SV-PBS)
ENSG00000163736	PPBP		-0.65		0.63		42.0		39.7		81.7
ENSG00000188536	HBA2		-0.87		-0.23		75.8		5.2		80.9
ENSG00000245205	RP11-841C19.6		-0.67		-0.56		45.4		31.3		76.7
ENSG00000158856	EPB49		-0.57		0.65		32.7		42.4		75.2
ENSG00000244734	HBB		-0.80		-0.23		63.8		5.2		69.0
ENSG00000120885	CLU		-0.41		0.68		17.1		46.3		63.4
ENSG00000125968	ID1		-0.74		-0.25		54.8		6.3		61.1
ENSG00000163737	PF4		-0.57		0.53		32.7		27.6		60.3
ENSG00000088053	GP6		0.08		0.73		0.6		53.3		54.0
ENSG00000113140	SPARC		-0.44		0.58		19.2		33.3		52.5
ENSG00000206172	HBA1		-0.53		-0.45		27.6		20.0		47.6
ENSG00000225492	GBP1P1		-0.59		0.33		34.6		11.1		45.8
ENSG00000005961	ITGA2B		-0.34		0.57		11.8		32.9		44.7
ENSG00000228323	AC008753.6		-0.31		0.50		9.5		25.0		34.5
ENSG00000006075	CCL3		-0.31		0.32		9.4		10.5		20.0
ENSG00000099864	PALM		-0.27		0.26		7.2		6.8		14.1
ENSG00000159189	C1QC		0.36		0.10		12.8		0.9		13.8
ENSG00000137267	TUBB2A		-0.21		-0.26		4.5		6.5		11.0
ENSG00000125740	FOSB		-0.08		0.32		0.6		10.0		10.7
ENSG00000164930	FZD6		0.17		-0.27		3.0		7.1		10.0
ENSG00000090104	RGS1		-0.21		0.15		4.5		2.2		6.7
ENSG00000069696	DRD4		-0.15		-0.17		2.1		2.8		5.0
ENSG00000123689	G0S2		-0.16		-0.16		2.5		2.5		4.9
ENSG00000256049	PADI6		0.14		-0.05		2.0		0.2		2.2

Table A130: Canonical loadings and explained variance (RNA-Seq, Dendritic cells, Day 7, Gene variables). Gene variables with at least 25% of explained variance for the first two canonical variates are listed. GX: canonical loadings for canonical variate X, VX: % explained variance for canonical variate X, V1+V2: total % explained variance for both canonical variates. Values for CCA results with a poor cross validation score (< 0.70) are left blank. Sorted by maximum explained variance across treatments (V1+V2).

IMO Variable	IMO Variable Name	I1 (SV-AS03)	I1 (SV-PBS)	I2 (SV-AS03)	I2 (SV-PBS)	V1 (SV-AS03)	V1 (SV-PBS)	V2 (SV-AS03)	V2 (SV-PBS)	V1+V2 (SV-AS03)	V1+V2 (SV-PBS)
Nt	Microneutralization inhibition		0.18		-0.80		3.3		63.4		66.7
RANTES	Chemokine (C-C motif) ligand 5		-0.20		0.79		4.0		62.5		66.5
IL-12p70	Interleukin-12 p70		-0.81		0.01		66.4		0.0		66.4
IFN-γ	Interferon gamma		0.44		-0.53		19.7		28.5		48.2
IL-6	Interleukin-6		0.40		-0.55		15.9		30.3		46.1
TNF	Tumor necrosis factors		0.53		0.33		28.6		11.0		39.6

IMO Variable	IMO Variable Name	I1 (SV-AS03)	I1 (SV-PBS)	I2 (SV-AS03)	I2 (SV-PBS)	V1 (SV-AS03)	V1 (SV-PBS)	V2 (SV-AS03)	V2 (SV-PBS)	V1+V2 (SV-AS03)	V1+V2 (SV-PBS)
Eotaxin-1	Eotaxin-1		-0.54		0.11		29.1		1.2		30.4
IL-8	Interleukin-8		0.31		-0.40		9.7		16.2		25.8
MIP-1α	Macrophage inflammatory protein		0.42		-0.23		17.7		5.5		23.2
IP-10	Interferon gamma-induced protein 10		0.32		0.09		10.2		0.8		11.0
IL-1β	Interleukin-1 beta		-0.14		-0.25		1.8		6.5		8.3
IL-10	Interleukin-10		0.10		0.19		1.0		3.4		4.4
MCP-1	Monocyte chemoattractant protein-1		0.18		0.10		3.2		1.0		4.2

Table A131: Canonical loadings and explained variance (RNA-Seq, Dendritic cells, Day 7, IMO variables). IMO variables with at least 25% of explained variance for the first two canonical variates are listed. GX: canonical loadings for canonical variate X, VX: % explained variance for canonical variate X, V1+V2: total % explained variance for both canonical variates. Values for CCA results with a poor cross validation score (< 0.70) are left blank. Sorted by maximum explained variance across treatments (V1+V2).

Gene ID	Gene Name	G1 (SV-AS03)	G1 (SV-PBS)	G2 (SV-AS03)	G2 (SV-PBS)	V1 (SV-AS03)	V1 (SV-PBS)	V2 (SV-AS03)	V2 (SV-PBS)	V1+V2 (SV-AS03)	V1+V2 (SV-PBS)
ENSG00000126368	NR1D1	0.88		-0.29		77.6		8.4		86.0	
ENSG00000144218	AFF3	0.90		-0.22		80.3		4.8		85.1	
ENSG00000225217	HSPA7	-0.91		-0.17		81.9		2.9		84.8	
ENSG00000232931	AC008268.3	0.85		0.33		72.9		10.6		83.5	
ENSG00000104972	LILRB1	-0.91		0.07		82.7		0.5		83.2	
ENSG00000173110	HSPA6	-0.89		-0.13		79.2		1.7		80.9	
ENSG00000235081	AC010492.2	-0.26		-0.86		6.7		74.0		80.7	
ENSG00000139899	CBLN3	-0.88		-0.14		77.7		2.1		79.8	
ENSG00000128383	APOBEC3A	-0.89		-0.00		79.3		0.0		79.3	
ENSG00000120885	CLU	-0.84		-0.28		71.3		7.9		79.2	
ENSG00000187608	ISG15	-0.88		0.09		77.6		0.8		78.4	
ENSG00000149294	NCAM1	0.88		0.03		78.3		0.1		78.4	
ENSG00000160789	LMNA	0.87		-0.13		76.2		1.6		77.8	
ENSG00000168546	GFRA2	0.79		-0.39		62.6		15.1		77.7	
ENSG00000100100	PIK3IP1	0.82		0.31		67.4		9.7		77.1	
ENSG00000213420	GPC2	0.22		0.85		4.9		71.5		76.4	
ENSG00000183087	GAS6	-0.85		-0.21		71.4		4.6		76.0	
ENSG00000244682	FCGR2C	-0.85		-0.19		72.6		3.4		76.0	
ENSG00000203416	FAM32B	-0.84		0.20		71.2		3.8		75.0	
ENSG00000090920	FCGBP	0.86		0.04		74.3		0.2		74.5	
ENSG00000185885	IFITM1	-0.86		-0.09		73.3		0.9		74.2	
ENSG00000243811	APOBEC3D	-0.85		-0.12		72.6		1.5		74.1	
ENSG00000108950	FAM20A	-0.75		-0.41		56.8		17.1		73.9	
ENSG00000179044	EXOC3L1	-0.86		0.07		73.2		0.5		73.8	
ENSG00000142089	IFITM3	-0.83		-0.21		69.4		4.3		73.7	
ENSG00000019102	VSIG2	0.83		0.22		68.2		5.0		73.2	
ENSG00000165178	NCF1C	-0.85		0.01		73.0		0.0		73.1	
ENSG00000155761	SPAG17	0.85		0.03		72.9		0.1		73.0	
ENSG00000175262	C1orf127	0.84		-0.12		70.9		1.5		72.4	
ENSG00000229754	CXCR2P1	-0.84		0.11		71.2		1.1		72.3	
ENSG00000203747	FCGR3A	-0.59		-0.61		35.0		37.0		72.1	
ENSG00000096996	IL12RB1	-0.85		0.00		71.8		0.0		71.8	
ENSG00000158270	COLEC12	0.83		0.14		69.1		2.1		71.2	

Gene ID	Gene Name	G1 (SV-AS03)	G1 (SV-PBS)	G2 (SV-AS03)	G2 (SV-PBS)	V1 (SV-AS03)	V1 (SV-PBS)	V2 (SV-AS03)	V2 (SV-PBS)	V1+V2 (SV-AS03)	V1+V2 (SV-PBS)
ENSG00000168899	VAMP5	-0.84		0.05		70.4		0.3		70.7	
ENSG00000107201	DDX58	-0.84		-0.04		70.1		0.1		70.2	
ENSG00000157601	MX1	-0.83		0.10		69.0		1.0		70.0	
ENSG00000225131	PSME2P2	-0.82		0.14		67.9		2.1		69.9	
ENSG00000149564	ESAM	0.73		0.40		54.0		15.7		69.7	
ENSG00000116824	CD2	0.82		0.13		67.9		1.7		69.6	
ENSG00000160932	LY6E	-0.83		0.08		68.8		0.7		69.6	
ENSG00000172878	METAP1D	0.83		0.03		69.4		0.1		69.5	
ENSG00000172183	ISG20	-0.83		0.08		68.8		0.6		69.4	
ENSG00000158517	NCF1	-0.82		0.12		67.7		1.5		69.2	
ENSG00000125148	MT2A	-0.83		-0.01		69.2		0.0		69.2	
ENSG00000182389	CACNB4	0.83		0.07		68.8		0.4		69.2	
ENSG00000145287	PLAC8	-0.83		0.07		68.5		0.5		69.0	
ENSG00000004468	CD38	-0.83		0.01		68.9		0.0		68.9	
ENSG00000163568	AIM2	-0.82		0.12		67.2		1.5		68.7	
ENSG00000115155	OTOF	-0.69		0.46		47.3		21.2		68.5	
ENSG00000059588	TARBP1	0.81		0.15		66.1		2.1		68.2	
ENSG00000167371	PRRT2	-0.70		-0.44		48.7		19.1		67.8	
ENSG00000108679	LGALS3BP	-0.82		0.04		67.5		0.1		67.6	
ENSG00000025708	TYMP	-0.82		-0.06		67.1		0.4		67.5	
ENSG00000130489	SCO2	-0.82		-0.04		67.1		0.2		67.3	
ENSG00000138646	HERC5	-0.82		-0.02		67.0		0.1		67.1	
ENSG00000198959	TGM2	-0.82		0.06		66.5		0.3		66.8	
ENSG00000185745	IFIT1	-0.81		0.06		66.0		0.3		66.3	
ENSG00000137757	CASP5	-0.66		-0.47		43.8		22.5		66.3	
ENSG00000174721	FGFBP3	0.78		-0.23		60.9		5.2		66.1	
ENSG00000134321	RSAD2	-0.81		-0.04		66.0		0.1		66.1	
ENSG00000241106	HLA-DOB	-0.80		0.17		63.3		2.8		66.1	
ENSG00000013364	MVP	-0.80		-0.12		64.4		1.4		65.7	
ENSG00000010278	CD9	0.77		-0.26		58.9		6.7		65.6	
ENSG00000100911	PSME2	-0.80		0.14		63.5		2.0		65.5	
ENSG00000136235	GPNMB	0.64		-0.50		40.4		25.1		65.5	
ENSG00000152672	CLEC4F	0.69		-0.42		47.8		17.5		65.3	
ENSG00000213366	GSTM2	0.81		-0.03		65.2		0.1		65.3	
ENSG00000139572	GPR84	-0.81		-0.02		65.3		0.0		65.3	
ENSG00000124256	ZBP1	-0.80		0.08		64.5		0.6		65.1	
ENSG00000101017	CD40	-0.81		0.02		64.9		0.1		65.0	
ENSG00000138755	CXCL9	-0.75		-0.28		57.0		8.0		65.0	
ENSG00000133321	RARRES3	-0.80		0.06		64.4		0.4		64.8	
ENSG00000204653	ASPDH	0.80		0.03		64.6		0.1		64.7	
ENSG00000026751	SLAMF7	-0.80		0.02		64.5		0.0		64.6	
ENSG00000115392	FANCL	-0.80		-0.05		64.3		0.2		64.5	
ENSG00000171049	FPR2	-0.77		-0.22		59.5		4.9		64.4	
ENSG00000182557	SPNS3	0.80		0.09		63.2		0.8		64.1	
ENSG00000185339	TCN2	-0.80		0.08		63.3		0.7		64.0	
ENSG00000239899	AC011994.1	-0.66		0.45		43.6		20.4		64.0	
ENSG00000166278	C2	-0.80		0.04		63.5		0.1		63.6	
ENSG00000154258	ABCA9	0.73		-0.31		53.7		9.7		63.3	
ENSG00000135114	OASL	-0.79		-0.06		62.8		0.3		63.2	
ENSG00000224940	PRRT4	0.79		-0.02		63.0		0.1		63.1	
ENSG00000163006	CCDC138	0.41		-0.68		16.5		46.5		62.9	

Gene ID	Gene Name	G1 (SV-AS03)	G1 (SV-PBS)	G2 (SV-AS03)	G2 (SV-PBS)	V1 (SV-AS03)	V1 (SV-PBS)	V2 (SV-AS03)	V2 (SV-PBS)	V1+V2 (SV-AS03)	V1+V2 (SV-PBS)
ENSG00000198133	TMEM229B	-0.79		0.09		62.0		0.8		62.8	
ENSG00000173369	C1QB	-0.56		-0.56		31.3		31.5		62.8	
ENSG00000135047	CTSL1	-0.79		-0.05		62.3		0.3		62.6	
ENSG00000177989	ODF3B	-0.79		0.04		62.0		0.2		62.2	
ENSG00000213557	RP11-240E2.2	0.79		-0.00		62.1		0.0		62.1	
ENSG00000137959	IFI44L	-0.78		0.08		61.3		0.7		62.1	
ENSG00000133121	STARD13	0.79		0.05		61.7		0.3		61.9	
ENSG00000204930	C9orf128	0.61		-0.50		36.6		25.2		61.8	
ENSG00000136514	RTP4	-0.78		-0.09		60.9		0.8		61.7	
ENSG00000132530	XAF1	-0.78		-0.08		61.0		0.7		61.7	
ENSG00000141096	DPEP3	0.76		0.20		57.6		4.0		61.6	
ENSG00000205413	SAMD9	-0.78		0.01		61.5		0.0		61.5	
ENSG00000087589	CASS4	0.78		-0.04		61.3		0.1		61.4	
ENSG00000137965	IFI44	-0.78		0.06		61.1		0.3		61.4	
ENSG00000115267	IFIH1	-0.78		0.04		61.1		0.1		61.2	
ENSG00000172159	FRMD3	-0.78		-0.02		61.2		0.1		61.2	
ENSG00000111335	OAS2	-0.78		0.03		60.7		0.1		60.8	
ENSG00000155363	MOV10	-0.78		-0.02		60.6		0.1		60.6	
ENSG00000166689	PLEKHA7	0.71		-0.31		50.8		9.7		60.5	
ENSG00000126262	FFAR2	-0.77		-0.13		58.8		1.6		60.4	
ENSG00000160111	CPAMD8	0.77		-0.06		60.0		0.4		60.4	
ENSG00000164867	NOS3	0.69		-0.37		47.0		13.4		60.3	
ENSG00000240065	PSMB9	-0.77		0.01		59.9		0.0		59.9	
ENSG00000049089	COL9A2	0.77		0.10		58.7		1.1		59.8	
ENSG00000162694	EXTL2	0.57		-0.51		32.9		26.4		59.3	
ENSG00000236783	RP11-272G11.1	0.75		-0.17		56.3		2.9		59.3	
ENSG00000121858	TNFSF10	-0.77		-0.03		59.2		0.1		59.2	
ENSG00000213172	RP1-228H13.2	-0.54		-0.55		28.7		30.5		59.2	
ENSG00000111331	OAS3	-0.77		0.06		58.8		0.4		59.1	
ENSG00000168062	BATF2	-0.77		-0.04		58.9		0.1		59.1	
ENSG00000072694	FCGR2B	-0.72		-0.27		51.2		7.3		58.6	
ENSG00000111110	PPM1H	0.76		-0.03		58.2		0.1		58.3	
ENSG00000197381	ADARB1	-0.72		-0.25		51.8		6.5		58.2	
ENSG00000068079	IFI35	-0.76		-0.03		58.1		0.1		58.2	
ENSG00000256262	RP11-117B7.1	-0.58		-0.49		34.2		24.0		58.1	
ENSG00000123609	NMI	-0.76		-0.02		57.8		0.0		57.9	
ENSG00000156265	C21orf7	-0.76		0.06		57.3		0.4		57.7	
ENSG00000173706	HEG1	-0.70		-0.29		49.1		8.5		57.7	
ENSG00000115594	IL1R1	0.68		-0.33		46.5		11.2		57.7	
ENSG00000130589	RP4-697K14.7	-0.75		-0.13		55.8		1.8		57.6	
ENSG00000166428	PLD4	0.73		-0.20		53.6		3.8		57.4	
ENSG00000019169	MARCO	-0.75		0.13		55.6		1.7		57.4	
ENSG00000145349	CAMK2D	-0.74		0.17		54.5		2.8		57.2	
ENSG00000204267	TAP2	-0.76		-0.00		57.2		0.0		57.2	
ENSG00000197536	C5orf56	-0.76		0.00		57.0		0.0		57.0	
ENSG00000108691	CCL2	-0.75		-0.05		56.6		0.2		56.8	
ENSG00000152766	ANKRD22	-0.75		-0.06		56.2		0.3		56.5	
ENSG00000138031	ADCY3	-0.68		-0.33		45.7		10.7		56.4	
ENSG00000132518	GUCY2D	0.73		0.18		53.0		3.3		56.3	
ENSG00000100342	APOL1	-0.75		0.00		56.3		0.0		56.3	
ENSG00000189007	ADAT2	0.75		-0.06		55.7		0.4		56.0	

Gene ID	Gene Name	G1 (SV-AS03)	G1 (SV-PBS)	G2 (SV-AS03)	G2 (SV-PBS)	V1 (SV-AS03)	V1 (SV-PBS)	V2 (SV-AS03)	V2 (SV-PBS)	V1+V2 (SV-AS03)	V1+V2 (SV-PBS)
ENSG00000137628	DDX60	-0.75		-0.03		55.8		0.1		56.0	
ENSG00000134326	CMPK2	-0.75		0.03		55.6		0.1		55.6	
ENSG00000183496	MEX3B	0.74		-0.04		55.5		0.2		55.6	
ENSG00000123610	TNFAIP6	-0.70		0.25		49.2		6.4		55.6	
ENSG00000177409	SAMD9L	-0.74		-0.06		55.1		0.4		55.5	
ENSG00000128394	APOBEC3F	-0.72		-0.21		51.2		4.2		55.5	
ENSG00000141574	SECTM1	-0.74		-0.12		54.1		1.4		55.4	
ENSG00000225886	RP11-288L9.4	-0.74		-0.01		55.4		0.0		55.4	
ENSG00000157551	KCNJ15	-0.50		-0.55		25.2		30.1		55.2	
ENSG00000162512	SDC3	-0.71		-0.22		50.2		4.8		55.0	
ENSG00000149131	SERPING1	-0.74		-0.02		54.7		0.0		54.7	
ENSG00000165806	CASP7	-0.73		-0.12		53.2		1.5		54.6	
ENSG00000169136	ATF5	-0.74		-0.03		54.4		0.1		54.5	
ENSG00000137801	THBS1	0.73		-0.06		53.6		0.4		53.9	
ENSG00000185567	AHNAK2	0.71		0.17		50.9		3.0		53.9	
ENSG00000144354	CDCA7	0.70		0.21		49.3		4.5		53.8	
ENSG00000133313	CNDP2	-0.72		-0.15		51.3		2.3		53.6	
ENSG00000131203	IDO1	-0.73		-0.02		53.5		0.1		53.6	
ENSG00000132386	SERPINF1	0.73		-0.06		53.3		0.3		53.6	
ENSG00000119917	IFIT3	-0.73		-0.06		52.9		0.4		53.2	
ENSG00000205220	PSMB10	-0.72		0.08		52.4		0.6		53.1	
ENSG00000173372	C1QA	-0.61		-0.39		37.5		15.5		52.9	
ENSG00000156587	UBE2L6	-0.73		0.04		52.7		0.1		52.9	
ENSG00000185507	IRF7	-0.72		-0.10		51.5		1.0		52.5	
ENSG00000243649	CFB	-0.70		0.15		49.1		2.4		51.5	
ENSG00000135636	DYSF	-0.71		-0.11		50.4		1.2		51.5	
ENSG00000106976	DNM1	0.67		-0.26		44.5		7.0		51.5	
ENSG00000103569	AQP9	-0.57		-0.44		32.5		19.0		51.4	
ENSG00000196081	ZNF724P	0.53		-0.48		27.9		23.4		51.2	
ENSG00000139832	RAB20	-0.70		-0.16		48.5		2.7		51.2	
ENSG00000129673	AANAT	-0.71		-0.06		50.8		0.4		51.1	
ENSG00000253193	FCGR1C	-0.65		-0.29		42.5		8.7		51.1	
ENSG00000079263	SP140	-0.71		0.05		50.3		0.2		50.5	
ENSG00000152778	IFIT5	-0.65		-0.29		41.8		8.6		50.3	
ENSG00000184500	PROS1	0.22		-0.67		4.9		45.4		50.2	
ENSG00000135362	PRR5L	-0.65		-0.28		42.3		7.8		50.0	
ENSG00000196220	SRGAP3	0.71		0.04		49.8		0.2		50.0	
ENSG00000188820	FAM26F	-0.70		0.06		49.6		0.4		50.0	
ENSG00000171621	SPSB1	-0.71		0.01		50.0		0.0		50.0	
ENSG00000133106	EPSTI1	-0.70		-0.07		49.4		0.5		50.0	
ENSG00000071242	RPS6KA2	0.69		0.15		47.4		2.3		49.7	
ENSG00000121966	CXCR4	0.68		-0.20		45.8		3.9		49.7	
ENSG00000169245	CXCL10	-0.70		-0.07		48.8		0.5		49.3	
ENSG00000226321	AC104809.3	0.49		-0.50		24.2		25.0		49.2	
ENSG00000111181	SLC6A12	-0.70		-0.02		49.2		0.0		49.2	
ENSG00000136960	ENPP2	-0.66		-0.24		43.2		5.9		49.1	
ENSG00000184313	HEATR8	0.70		-0.05		48.7		0.3		49.0	
ENSG00000233392	AC104809.4	0.23		-0.66		5.5		43.0		48.5	
ENSG00000116663	FBXO6	-0.69		-0.06		48.1		0.4		48.5	
ENSG00000234290	AC116366.6	-0.67		-0.20		44.5		3.9		48.4	
ENSG00000164308	ERAP2	-0.68		0.14		46.3		2.0		48.3	

Gene ID	Gene Name	G1 (SV-AS03)	G1 (SV-PBS)	G2 (SV-AS03)	G2 (SV-PBS)	V1 (SV-AS03)	V1 (SV-PBS)	V2 (SV-AS03)	V2 (SV-PBS)	V1+V2 (SV-AS03)	V1+V2 (SV-PBS)
ENSG00000170581	STAT2	-0.68		-0.14		46.3		2.0		48.3	
ENSG00000162654	GBP4	-0.69		0.01		48.2		0.0		48.2	
ENSG00000138821	SLC39A8	-0.67		-0.19		44.5		3.5		48.1	
ENSG00000131979	GCH1	-0.69		-0.09		47.1		0.9		48.0	
ENSG00000163840	DTX3L	-0.67		-0.16		45.4		2.5		48.0	
ENSG00000138496	PARP9	-0.68		-0.14		45.9		2.0		47.9	
ENSG00000226004	RP11-10J5.1	-0.69		-0.01		47.8		0.0		47.8	
ENSG00000138119	MYOF	-0.69		0.00		47.7		0.0		47.7	
ENSG00000113368	LMNB1	-0.68		-0.09		46.8		0.8		47.7	
ENSG00000140511	HAPLN3	-0.69		-0.00		47.5		0.0		47.5	
ENSG00000179639	FCER1A	0.67		-0.15		45.1		2.4		47.5	
ENSG00000072858	SIDT1	0.69		0.01		47.4		0.0		47.4	
ENSG00000136231	IGF2BP3	-0.68		0.07		46.7		0.5		47.3	
ENSG00000105639	JAK3	-0.62		-0.29		38.4		8.6		47.0	
ENSG00000150337	FCGR1A	-0.64		-0.25		40.8		6.2		46.9	
ENSG00000002549	LAP3	-0.68		-0.07		46.4		0.4		46.9	
ENSG00000158714	SLAMF8	-0.65		-0.21		42.2		4.3		46.5	
ENSG00000133048	CHI3L1	-0.32		-0.60		10.1		36.4		46.5	
ENSG00000059378	PARP12	-0.68		0.00		46.5		0.0		46.5	
ENSG00000132274	TRIM22	-0.68		-0.06		46.1		0.3		46.5	
ENSG00000162337	LRP5	0.68		0.03		46.3		0.1		46.4	
ENSG00000028116	VRK2	-0.66		0.17		43.6		2.8		46.4	
ENSG00000162614	NEXN	-0.67		0.09		45.5		0.8		46.4	
ENSG00000198019	FCGR1B	-0.63		-0.26		39.3		7.0		46.3	
ENSG00000163814	CDCP1	-0.67		-0.10		45.3		1.1		46.3	
ENSG00000171766	GATM	0.55		0.40		30.5		15.7		46.2	
ENSG00000099377	HSD3B7	-0.57		-0.36		32.8		13.3		46.1	
ENSG00000128335	APOL2	-0.67		-0.08		45.3		0.7		46.1	
ENSG00000254859	RP11-661A12.5	-0.68		-0.01		46.0		0.0		46.0	
ENSG00000140464	PML	-0.68		-0.03		45.9		0.1		45.9	
ENSG00000169313	P2RY12	-0.66		0.13		44.2		1.7		45.9	
ENSG00000173193	PARP14	-0.67		-0.10		44.7		1.0		45.7	
ENSG00000020577	SAMD4A	-0.68		0.03		45.6		0.1		45.7	
ENSG00000152580	IGSF10	0.56		-0.37		31.6		13.6		45.2	
ENSG00000171954	CYP4F22	-0.44		-0.50		19.7		25.1		44.8	
ENSG00000108387	SEPT4	-0.67		-0.06		44.5		0.3		44.8	
ENSG00000123240	OPTN	-0.65		0.17		41.8		2.9		44.7	
ENSG00000010030	ETV7	-0.67		-0.04		44.5		0.1		44.7	
ENSG00000130487	KLHDC7B	-0.67		0.01		44.4		0.0		44.4	
ENSG00000117228	GBP1	-0.66		-0.07		43.9		0.5		44.4	
ENSG00000238000	RP11-274E7.2	-0.66		0.09		43.4		0.8		44.2	
ENSG00000244642	AP005717.1	-0.62		0.24		38.4		5.8		44.2	
ENSG00000150347	ARID5B	-0.62		-0.23		38.7		5.2		43.9	
ENSG00000170955	PRKCDBP	0.60		-0.28		35.7		8.0		43.7	
ENSG00000119922	IFIT2	-0.66		-0.06		43.0		0.3		43.3	
ENSG00000146072	TNFRSF21	0.66		-0.03		43.0		0.1		43.1	
ENSG00000148175	STOM	-0.64		-0.14		40.9		2.0		42.9	
ENSG00000134470	IL15RA	-0.65		-0.04		42.4		0.2		42.6	
ENSG00000128203	ASPHD2	-0.64		0.14		40.5		2.0		42.5	
ENSG00000055130	CUL1	-0.65		0.02		42.4		0.0		42.5	
ENSG00000128284	APOL3	-0.65		0.03		42.2		0.1		42.3	

Gene ID	Gene Name	G1 (SV-AS03)	G1 (SV-PBS)	G2 (SV-AS03)	G2 (SV-PBS)	V1 (SV-AS03)	V1 (SV-PBS)	V2 (SV-AS03)	V2 (SV-PBS)	V1+V2 (SV-AS03)	V1+V2 (SV-PBS)
ENSG00000168685	IL7R	0.52		0.38		27.4		14.6		42.0	
ENSG00000154451	GBP5	-0.65		-0.02		41.9		0.0		42.0	
ENSG00000137193	PIM1	-0.65		0.02		41.9		0.1		41.9	
ENSG00000242616	GNG10	-0.53		-0.37		28.4		13.3		41.7	
ENSG00000164715	LMTK2	-0.63		-0.16		39.3		2.4		41.7	
ENSG00000177732	SOX12	0.60		0.25		35.5		6.0		41.6	
ENSG00000107282	APBA1	0.60		-0.22		36.6		5.0		41.5	
ENSG00000138166	DUSP5	-0.63		-0.12		39.8		1.4		41.3	
ENSG00000173821	RNF213	-0.64		-0.00		41.2		0.0		41.2	
ENSG00000142798	HSPG2	0.50		-0.40		24.9		16.3		41.2	
ENSG00000231528	NCRNA00256A	-0.63		-0.11		39.9		1.2		41.1	
ENSG00000168016	TRANK1	-0.63		-0.07		40.2		0.4		40.6	
ENSG00000182580	EPHB3	0.63		-0.12		39.1		1.5		40.6	
ENSG00000174749	C4orf32	-0.59		-0.25		34.5		6.0		40.5	
ENSG00000129244	ATP1B2	-0.45		-0.45		19.9		20.5		40.5	
ENSG00000203814	HIST2H2BF	-0.57		-0.29		32.2		8.3		40.5	
ENSG00000144668	ITGA9	0.63		-0.05		40.0		0.2		40.2	
ENSG00000163606	CD200R1	0.53		0.35		27.8		12.3		40.1	
ENSG00000117226	GBP3	-0.63		-0.07		39.6		0.5		40.1	
ENSG00000175322	ZNF519	0.59		-0.23		34.7		5.4		40.0	
ENSG00000234571	RP5-998N21.4	-0.51		-0.38		25.9		14.2		40.0	
ENSG00000225492	GBP1P1	-0.63		-0.07		39.4		0.5		40.0	
ENSG00000175445	LPL	0.61		-0.14		37.6		2.0		39.6	
ENSG00000138134	STAMBPL1	-0.63		0.01		39.6		0.0		39.6	
ENSG00000168394	TAP1	-0.63		-0.04		39.1		0.2		39.3	
ENSG00000122420	PTGFR	-0.23		-0.58		5.3		33.9		39.3	
ENSG00000162645	GBP2	-0.63		-0.01		39.1		0.0		39.1	
ENSG00000075399	C16orf7	-0.60		-0.17		36.1		2.9		38.9	
ENSG00000109819	PPARGC1A	0.62		-0.06		38.5		0.4		38.9	
ENSG00000112290	WASF1	-0.38		-0.50		14.1		24.5		38.6	
ENSG00000156535	CD109	0.60		0.15		36.1		2.2		38.3	
ENSG00000234518	RP5-837D10.2	-0.62		0.05		38.0		0.2		38.2	
ENSG00000185499	MUC1	-0.62		-0.06		37.8		0.3		38.1	
ENSG00000115415	STAT1	-0.61		-0.08		37.4		0.6		38.0	
ENSG00000255045	RP11-677M14.2-001	0.21		0.58		4.5		33.5		37.9	
ENSG00000127585	FBXL16	0.60		0.14		35.5		1.9		37.3	
ENSG00000120217	CD274	-0.59		-0.12		35.4		1.5		36.8	
ENSG00000140105	WARS	-0.60		-0.11		35.5		1.3		36.8	
ENSG00000140859	KIFC3	-0.15		-0.59		2.1		34.6		36.7	
ENSG00000118513	MYB	0.42		0.43		17.9		18.7		36.5	
ENSG00000211899	IGHM	-0.60		0.03		36.3		0.1		36.4	
ENSG00000182118	FAM89A	-0.60		-0.09		35.4		0.8		36.2	
ENSG00000179750	APOBEC3B	-0.52		0.30		27.1		9.0		36.1	
ENSG00000197121	PGAP1	-0.59		0.11		34.9		1.2		36.1	
ENSG00000229644	NAMPTL	-0.50		-0.33		24.8		11.0		35.9	
ENSG00000164509	IL31RA	-0.59		-0.11		34.2		1.1		35.4	
ENSG00000142303	ADAMTS10	0.59		0.10		34.3		1.0		35.3	
ENSG00000166532	RIMKLB	-0.59		-0.04		35.2		0.2		35.3	
ENSG00000247275	AL160008.1	-0.59		0.08		34.5		0.7		35.3	
ENSG00000170525	PFKFB3	-0.59		-0.01		35.2		0.0		35.2	
ENSG00000139211	AMIGO2	-0.51		0.31		25.6		9.6		35.2	

Gene ID	Gene Name	G1 (SV-AS03)	G1 (SV-PBS)	G2 (SV-AS03)	G2 (SV-PBS)	V1 (SV-AS03)	V1 (SV-PBS)	V2 (SV-AS03)	V2 (SV-PBS)	V1+V2 (SV-AS03)	V1+V2 (SV-PBS)
ENSG00000145365	TIFA	-0.58		-0.09		34.1		0.9		35.0	
ENSG00000124216	SNAI1	0.49		0.33		23.9		11.0		35.0	
ENSG00000103528	SYT17	0.56		-0.20		31.0		3.8		34.9	
ENSG00000163485	ADORA1	0.54		-0.23		29.3		5.3		34.7	
ENSG00000131355	EMR3	0.58		-0.08		34.0		0.7		34.6	
ENSG00000257017	HP	-0.53		-0.26		28.0		6.5		34.5	
ENSG00000243181	RP11-734J24.1	0.40		0.43		15.6		18.7		34.4	
ENSG00000143847	PPFIA4	0.57		0.11		33.0		1.3		34.3	
ENSG00000029153	ARNTL2	-0.58		0.05		33.8		0.3		34.1	
ENSG00000187474	FPR3	0.56		-0.17		30.9		3.0		33.9	
ENSG00000198569	SLC34A3	0.25		-0.52		6.5		27.3		33.7	
ENSG00000140379	BCL2A1	-0.58		-0.06		33.3		0.4		33.7	
ENSG00000134755	DSC2	-0.41		-0.40		17.1		16.0		33.1	
ENSG00000073111	MCM2	0.32		-0.48		10.1		22.7		32.8	
ENSG00000169750	RAC3	0.08		0.56		0.6		31.8		32.4	
ENSG00000116717	GADD45A	0.55		0.16		29.7		2.7		32.4	
ENSG00000090975	PITPNM2	0.49		-0.30		23.6		8.7		32.4	
ENSG00000111371	SLC38A1	0.51		0.24		26.5		5.8		32.3	
ENSG00000182541	LIMK2	-0.55		-0.15		30.0		2.2		32.1	
ENSG00000233029	RP11-439A17.9	-0.53		-0.19		28.2		3.7		31.8	
ENSG00000115419	GLS	-0.56		-0.10		30.9		0.9		31.8	
ENSG00000167767	KRT80	0.47		-0.30		22.4		9.2		31.6	
ENSG00000013374	NUB1	-0.56		-0.07		31.0		0.5		31.5	
ENSG00000183762	KREMEN1	-0.54		-0.16		28.8		2.6		31.4	
ENSG00000174080	CTSF	0.34		0.45		11.4		19.9		31.2	
ENSG00000100336	APOL4	-0.56		0.02		31.0		0.0		31.1	
ENSG00000172232	AZU1	0.55		0.07		30.5		0.5		31.0	
ENSG00000184221	OLIG1	0.54		0.14		28.9		1.9		30.8	
ENSG00000179715	FAM113B	-0.53		0.15		28.5		2.1		30.6	
ENSG00000151623	NR3C2	0.51		0.21		26.2		4.4		30.6	
ENSG00000252206	RNU7-40P	-0.38		-0.40		14.1		16.4		30.4	
ENSG00000214872	SMTNL1	-0.55		-0.04		30.3		0.1		30.4	
ENSG00000174370	C11orf45	0.55		-0.07		29.8		0.5		30.3	
ENSG00000221963	APOL6	-0.54		-0.10		29.3		0.9		30.3	
ENSG00000111696	NT5DC3	0.54		-0.11		29.0		1.2		30.2	
ENSG00000246203	RP11-29H23.5	-0.46		0.28		21.3		8.0		29.3	
ENSG00000109099	PMP22	0.53		0.06		28.6		0.4		29.0	
ENSG00000151693	ASAP2	-0.53		-0.10		27.7		1.1		28.8	
ENSG00000179593	ALOX15B	0.50		-0.19		24.9		3.7		28.6	
ENSG00000105516	DBP	0.52		0.13		27.0		1.6		28.6	
ENSG00000162894	FAIM3	-0.04		0.53		0.2		27.8		28.0	
ENSG00000149927	DOC2A	0.52		0.11		26.7		1.2		27.9	
ENSG00000188549	C15orf52	0.47		0.23		22.5		5.2		27.8	
ENSG00000242797	RP11-168J18.4	-0.52		0.07		26.8		0.5		27.3	
ENSG00000108244	KRT23	0.30		0.43		9.0		18.2		27.2	
ENSG00000205846	CLEC6A	-0.51		-0.09		26.3		0.9		27.1	
ENSG00000135604	STX11	-0.52		-0.02		26.9		0.0		26.9	
ENSG00000119986	AVPI1	0.48		-0.20		22.6		3.9		26.5	
ENSG00000254838	GVINP1	-0.51		0.02		26.4		0.0		26.5	
ENSG00000197272	IL27	-0.43		-0.28		18.6		7.8		26.5	
ENSG00000183780	SLC35F3	0.50		-0.09		25.3		0.9		26.2	

Gene ID	Gene Name	G1 (SV-AS03)	G1 (SV-PBS)	G2 (SV-AS03)	G2 (SV-PBS)	V1 (SV-AS03)	V1 (SV-PBS)	V2 (SV-AS03)	V2 (SV-PBS)	V1+V2 (SV-AS03)	V1+V2 (SV-PBS)
ENSG00000188385	JAKMIP3	0.25		0.44		6.2		19.6		25.7	
ENSG00000096968	JAK2	-0.51		0.00		25.7		0.0		25.7	
ENSG00000167680	SEMA6B	-0.19		-0.47		3.6		22.0		25.5	
ENSG00000114127	XRN1	-0.49		-0.13		23.8		1.7		25.5	
ENSG00000168209	DDIT4	0.25		0.44		6.3		19.1		25.3	
ENSG00000008441	NFIX	-0.50		0.02		25.2		0.1		25.2	
ENSG00000111186	WNT5B	0.44		0.23		19.7		5.3		24.9	
ENSG00000151726	ACSL1	-0.42		-0.26		17.6		7.0		24.5	
ENSG00000087237	CETP	-0.47		-0.16		21.9		2.6		24.5	
ENSG00000166405	RIC3	-0.21		-0.44		4.5		19.7		24.3	
ENSG00000229759	MRPS18AP1	0.48		-0.11		23.0		1.3		24.3	
ENSG00000099834	CDHR5	-0.48		-0.11		22.9		1.1		24.1	
ENSG00000248487	ABHD14A	0.28		0.40		7.7		15.9		23.7	
ENSG00000187912	CLEC17A	0.39		0.28		15.4		7.8		23.2	
ENSG00000158156	XKR8	-0.42		-0.18		17.9		3.1		21.0	
ENSG00000163251	FZD5	-0.41		-0.20		16.8		3.8		20.6	
ENSG00000165795	NDRG2	0.40		-0.21		16.2		4.4		20.6	
ENSG00000112096	SOD2	-0.39		-0.22		15.5		4.7		20.2	
ENSG00000130595	TNNT3	0.44		0.09		19.2		0.7		19.9	
ENSG00000213949	ITGA1	-0.43		-0.13		18.1		1.7		19.8	
ENSG00000184557	SOCS3	-0.38		0.23		14.5		5.2		19.7	
ENSG00000181036	FCRL6	-0.42		-0.13		17.9		1.7		19.6	
ENSG00000253368	TRNP1	0.41		-0.15		16.6		2.2		18.9	
ENSG00000148926	ADM	-0.36		-0.23		13.2		5.2		18.4	
ENSG00000179388	EGR3	-0.28		-0.32		7.8		10.3		18.1	
ENSG00000125347	IRF1	-0.42		0.01		17.9		0.0		17.9	
ENSG00000253882	RP11-61L23.2	-0.38		0.18		14.5		3.4		17.9	
ENSG00000154102	C16orf74	0.42		-0.02		17.8		0.0		17.9	
ENSG00000152229	PSTPIP2	-0.37		-0.18		14.0		3.4		17.4	
ENSG00000222179	7SK_222179	-0.24		-0.33		5.7		10.8		16.5	
ENSG00000140853	NLRC5	-0.37		-0.14		14.0		2.1		16.0	
ENSG00000180644	PRF1	0.39		-0.03		15.6		0.1		15.7	
ENSG00000196549	MME	0.37		-0.14		13.7		1.9		15.6	
ENSG00000254017	IGHEP2	-0.32		0.22		10.1		4.7		14.8	
ENSG00000083454	P2RX5	-0.36		0.13		12.8		1.8		14.7	
ENSG00000254521	SIGLEC12	-0.33		-0.16		11.0		2.6		13.6	
ENSG00000057294	PKP2	0.23		-0.28		5.2		7.9		13.2	
ENSG00000125538	IL1B	0.26		-0.25		6.6		6.2		12.8	
ENSG00000103485	QPRT	0.29		0.20		8.4		4.0		12.4	
ENSG00000182048	TRPC2	-0.10		-0.34		0.9		11.4		12.3	
ENSG00000158715	SLC45A3	0.29		-0.19		8.4		3.7		12.1	
ENSG00000256579	AC135776.2	-0.24		-0.25		5.5		6.5		12.0	
ENSG00000134769	DTNA	0.33		0.05		10.9		0.2		11.2	
ENSG00000164292	RHOBTB3	-0.30		-0.15		9.0		2.1		11.1	
ENSG00000116544	DLGAP3	0.29		0.16		8.4		2.5		10.9	
ENSG00000154997	SEPT14	0.19		-0.27		3.5		7.4		10.8	
ENSG00000176749	CDK5R1	0.31		-0.10		9.7		1.1		10.8	
ENSG00000241360	PDXP	0.06		-0.32		0.4		10.2		10.6	
ENSG00000248936	RP11-36B15.1	0.24		-0.22		5.8		4.8		10.6	
ENSG00000135476	ESPL1	-0.25		0.21		6.2		4.3		10.5	
ENSG00000105835	NAMPT	-0.27		-0.16		7.1		2.7		9.7	

Gene ID	Gene Name	G1 (SV-AS03)	G1 (SV-PBS)	G2 (SV-AS03)	G2 (SV-PBS)	V1 (SV-AS03)	V1 (SV-PBS)	V2 (SV-AS03)	V2 (SV-PBS)	V1+V2 (SV-AS03)	V1+V2 (SV-PBS)
ENSG00000228166	RP11-39K24.11	0.26		0.16		6.9		2.5		9.4	
ENSG00000110848	CD69	-0.14		0.26		2.1		7.0		9.0	
ENSG00000122877	EGR2	-0.23		-0.19		5.1		3.5		8.7	
ENSG00000236254	RP11-39K24.8	-0.21		-0.20		4.5		3.9		8.4	
ENSG00000255221	CARD17	-0.26		0.12		6.7		1.6		8.3	
ENSG00000255398	GPR109B	0.24		-0.15		5.9		2.3		8.2	
ENSG00000136848	DAB2IP	-0.28		0.03		7.9		0.1		8.0	
ENSG00000227730	MTND6P5	-0.07		0.27		0.4		7.5		8.0	
ENSG00000182782	GPR109A	0.19		-0.20		3.8		3.9		7.7	
ENSG00000235917	RP11-39K24.14	-0.27		0.01		7.6		0.0		7.6	
ENSG00000254528	RP11-728F11.4	0.07		-0.25		0.6		6.4		6.9	
ENSG00000173762	CD7	-0.18		0.20		3.1		3.8		6.9	
ENSG00000236567	RP11-39K24.7	-0.18		0.18		3.1		3.3		6.4	
ENSG00000204228	HSD17B8	0.21		-0.14		4.5		1.9		6.4	
ENSG00000203804	C1orf138	-0.13		0.20		1.6		3.8		5.4	
ENSG00000251390	RP11-276A18.2	0.23		-0.02		5.1		0.1		5.2	
ENSG00000101280	ANGPT4	0.19		0.10		3.8		1.1		4.9	
ENSG00000056661	PCGF2	0.20		0.09		4.0		0.7		4.7	
ENSG00000237711	RP11-39K24.13	-0.20		0.08		4.0		0.6		4.7	
ENSG00000232229	RP11-248C1.2	0.08		0.20		0.6		4.1		4.6	
ENSG00000224745	RP11-380G5.2	0.21		-0.06		4.2		0.3		4.6	
ENSG00000228097	RP11-39K24.12	-0.09		0.19		0.8		3.5		4.3	
ENSG00000177586	RP11-113C12.5	-0.16		0.12		2.7		1.5		4.2	
ENSG00000124225	PMEPA1	-0.18		0.09		3.2		0.9		4.1	
ENSG00000230138	RP11-117D22.2	0.08		-0.18		0.7		3.2		3.8	
ENSG00000185022	MAFF	0.00		-0.19		0.0		3.5		3.5	
ENSG00000163629	PTPN13	-0.13		0.12		1.6		1.4		3.1	
ENSG00000224083	RP11-39K24.4	-0.17		0.04		2.8		0.2		3.0	
ENSG00000139174	PRICKLE1	0.04		0.16		0.2		2.5		2.6	
ENSG00000160298	C21orf58	0.09		0.11		0.9		1.1		2.0	
ENSG00000162772	ATF3	0.13		0.06		1.6		0.3		2.0	
ENSG00000238042	RP11-815M8.1	-0.08		-0.06		0.6		0.4		1.0	
ENSG00000223534	HLA-DQB1-AS1	-0.03		-0.05		0.1		0.2		0.3	
ENSG00000202364	SNORD3A	-0.04		-0.02		0.2		0.0		0.2	
ENSG00000230225	RP11-39K24.15	-0.05		0.00		0.2		0.0		0.2	

Table A132: Canonical loadings and explained variance (RNA-Seq, Monocytes, Day 1, Gene variables). Gene variables with at least 25% of explained variance for the first two canonical variates are listed. GX: canonical loadings for canonical variate X, VX: % explained variance for canonical variate X, V1+V2: total % explained variance for both canonical variates. Values for CCA results with a poor cross validation score (< 0.70) are left blank. Sorted by maximum explained variance across treatments (V1+V2).

IMO Variable	IMO Variable Name	I1 (SV-AS03)	I1 (SV-PBS)	I2 (SV-AS03)	I2 (SV-PBS)	V1 (SV-AS03)	V1 (SV-PBS)	V2 (SV-AS03)	V2 (SV-PBS)	V1+V2 (SV-AS03)	V1+V2 (SV-PBS)
MIP-1α	Macrophage inflammatory protein	-0.96		-0.13		91.3		1.6		92.9	
IP-10	Interferon gamma-induced protein 10	-0.86		0.21		74.3		4.3		78.6	
Eotaxin-1	Eotaxin-1	0.63		0.15		40.2		2.3		42.6	
IL-12p70	Interleukin-12 p70	-0.01		-0.61		0.0		37.5		37.5	
TNF	Tumor necrosis factors	0.56		0.25		31.3		6.1		37.4	

IMO Variable	IMO Variable Name	I1 (SV-AS03)	I1 (SV-PBS)	I2 (SV-AS03)	I2 (SV-PBS)	V1 (SV-AS03)	V1 (SV-PBS)	V2 (SV-AS03)	V2 (SV-PBS)	V1+V2 (SV-AS03)	V1+V2 (SV-PBS)
Nt	Microneutralization inhibition	0.26		0.53		7.0		28.5		35.5	
IL-1β	Interleukin-1 beta	-0.39		0.41		15.5		16.7		32.2	
IL-8	Interleukin-8	0.18		0.45		3.1		20.3		23.4	
IL-6	Interleukin-6	-0.37		-0.04		14.0		0.2		14.2	
IFN-γ	Interferon gamma	0.35		-0.07		12.0		0.5		12.6	
MCP-1	Monocyte chemoattractant protein-1	-0.07		0.34		0.5		11.7		12.2	
RANTES	Chemokine (C-C motif) ligand 5	-0.30		-0.02		9.1		0.0		9.2	
IL-10	Interleukin-10	-0.17		0.14		2.8		2.1		4.9	

Table A133: Canonical loadings and explained variance (RNA-Seq, Monocytes, Day 1, IMO variables). IMO variables with at least 25% of explained variance for the first two canonical variates are listed. GX: canonical loadings for canonical variate X, VX: % explained variance for canonical variate X, V1+V2: total % explained variance for both canonical variates. Values for CCA results with a poor cross validation score (< 0.70) are left blank. Sorted by maximum explained variance across treatments (V1+V2).

Gene ID	Gene Name	G1 (SV-AS03)	G1 (SV-PBS)	G2 (SV-AS03)	G2 (SV-PBS)	V1 (SV-AS03)	V1 (SV-PBS)	V2 (SV-AS03)	V2 (SV-PBS)	V1+V2 (SV-AS03)	V1+V2 (SV-PBS)
ENSG00000006075	CCL3	-0.89	0.14	0.33	0.02	78.5	2.0	10.7	0.0	89.2	2.0
ENSG00000102760	C13orf15	-0.83	0.08	0.44	-0.02	68.8	0.7	19.7	0.0	88.5	0.7
ENSG00000120738	EGR1	-0.91	-0.14	0.22	0.02	82.8	2.0	4.7	0.0	87.5	2.1
ENSG00000125740	FOSB	-0.87	0.18	0.21	-0.22	75.9	3.1	4.5	4.9	80.4	8.0
ENSG00000255398	GPR109B	-0.82	0.05	0.33	0.20	67.9	0.2	10.8	3.9	78.7	4.2
ENSG00000151704	KCNJ1	-0.85	-0.07	0.23	0.21	72.6	0.5	5.3	4.6	78.0	5.1
ENSG00000182782	GPR109A	-0.80	0.19	0.37	0.19	64.1	3.8	13.8	3.7	77.9	7.5
ENSG00000169429	IL8	-0.84	0.30	0.24	0.06	71.1	8.8	5.9	0.4	77.0	9.2
ENSG00000090104	RGS1	-0.80	0.65	-0.35	0.02	63.7	42.8	12.5	0.1	76.2	42.9
ENSG00000100385	IL2RB	0.76	-0.33	0.38	0.07	57.7	10.9	14.7	0.5	72.4	11.5
ENSG00000115085	ZAP70	0.74	-0.37	0.41	0.29	54.9	13.5	16.5	8.5	71.5	22.0
ENSG00000175592	FOSL1	-0.76	0.11	0.35	-0.29	57.6	1.1	12.3	8.7	69.9	9.8
ENSG00000234961	RP11-124N14.3	-0.78	0.29	-0.24	-0.32	61.3	8.2	5.6	10.0	66.9	18.3
ENSG00000179388	EGR3	-0.75	-0.09	0.29	-0.03	55.6	0.8	8.6	0.1	64.2	0.9
ENSG00000177606	JUN	-0.79	0.26	-0.08	-0.21	61.7	6.9	0.7	4.3	62.4	11.2
ENSG00000108679	LGALS3BP	0.01	0.57	-0.79	-0.13	0.0	32.7	62.1	1.7	62.1	34.4
ENSG00000180644	PRF1	0.60	-0.44	0.48	-0.06	36.4	19.1	23.1	0.4	59.5	19.5
ENSG00000207166	SNORA68_207166	-0.00	0.46	0.03	0.60	0.0	21.0	0.1	36.5	0.1	57.5
ENSG00000168685	IL7R	0.35	-0.60	0.67	0.16	11.9	35.7	45.5	2.6	57.5	38.3
ENSG00000125968	ID1	-0.73	0.48	-0.17	-0.18	52.7	23.0	2.9	3.3	55.5	26.3
ENSG00000081059	TCF7	0.43	-0.60	0.59	-0.11	18.4	35.7	34.6	1.1	53.0	36.8
ENSG00000107742	SPOCK2	0.70	-0.55	-0.08	-0.20	49.0	30.5	0.6	4.2	49.6	34.7
ENSG00000129538	RNASE1	0.22	0.66	0.00	-0.21	5.1	43.9	0.0	4.4	5.1	48.3
ENSG00000252678	RPPH1	0.03	0.40	-0.69	0.03	0.1	16.1	47.7	0.1	47.8	16.2
ENSG00000184979	USP18	-0.25	0.51	-0.65	-0.06	6.0	26.4	41.7	0.3	47.7	26.7
ENSG00000169902	TPST1	0.22	0.00	-0.64	-0.12	4.9	0.0	41.2	1.4	46.2	1.4
ENSG00000137959	IFI44L	-0.15	0.15	-0.66	-0.26	2.3	2.3	43.9	6.8	46.1	9.2
ENSG00000088827	SIGLEC1	-0.05	0.30	-0.67	-0.37	0.3	8.8	45.6	13.8	45.8	22.6
ENSG00000019485	PRDM11	0.52	0.58	0.08	0.34	26.5	33.5	0.6	11.5	27.1	45.1
ENSG00000129757	CDKN1C	-0.35	0.64	-0.28	-0.15	12.1	40.5	7.7	2.2	19.8	42.7
ENSG00000115155	OTOF	-0.11	0.16	-0.61	-0.10	1.1	2.4	37.6	1.0	38.7	3.4
ENSG00000173369	C1QB	-0.31	0.48	-0.50	-0.05	9.6	22.9	25.3	0.3	34.8	23.2

Gene ID	Gene Name	G1 (SV-AS03)	G1 (SV-PBS)	G2 (SV-AS03)	G2 (SV-PBS)	V1 (SV-AS03)	V1 (SV-PBS)	V2 (SV-AS03)	V2 (SV-PBS)	V1+V2 (SV-AS03)	V1+V2 (SV-PBS)
ENSG00000164082	GRM2	-0.57	0.44	-0.04	0.29	32.0	19.5	0.2	8.3	32.1	27.8
ENSG00000173372	C1QA	-0.12	0.56	-0.44	-0.08	1.5	31.5	19.0	0.6	20.5	32.1
ENSG00000188290	HES4	-0.40	0.49	-0.40	0.03	16.3	24.0	15.7	0.1	32.0	24.1
ENSG00000120885	CLU	0.11	-0.48	-0.49	-0.28	1.2	23.0	23.8	7.7	25.0	30.7
ENSG00000211899	IGHM	-0.10	-0.53	-0.10	0.12	0.9	28.4	0.9	1.6	1.9	30.0
ENSG00000111371	SLC38A1	-0.13	-0.48	0.19	-0.23	1.6	22.9	3.8	5.5	5.4	28.4
ENSG00000235917	RP11-39K24.14	-0.20	-0.05	0.34	-0.41	4.0	0.2	11.3	17.2	15.3	17.4
ENSG00000238035	AC138035.1	0.21	0.16	-0.17	-0.31	4.4	2.5	3.0	9.8	7.3	12.2

Table A134: Canonical loadings and explained variance (RNA-Seq, Monocytes, Day 3, Gene variables). Gene variables with at least 25% of explained variance for the first two canonical variates are listed. GX: canonical loadings for canonical variate X, VX: % explained variance for canonical variate X, V1+V2: total % explained variance for both canonical variates. Values for CCA results with a poor cross validation score (< 0.70) are left blank. Sorted by maximum explained variance across treatments (V1+V2).

IMO Variable	IMO Variable Name	I1 (SV-AS03)	I1 (SV-PBS)	I2 (SV-AS03)	I2 (SV-PBS)	V1 (SV-AS03)	V1 (SV-PBS)	V2 (SV-AS03)	V2 (SV-PBS)	V1+V2 (SV-AS03)	V1+V2 (SV-PBS)
IFN-γ	Interferon gamma	-0.42	0.09	0.68	-0.98	17.4	0.8	46.4	95.4	63.7	96.2
IL-1β	Interleukin-1 beta	-0.07	0.01	0.43	-0.93	0.5	0.0	18.9	87.2	19.3	87.2
TNF	Tumor necrosis factors	-0.85	0.11	0.07	-0.92	72.6	1.3	0.5	85.2	73.2	86.4
MIP-1α	Macrophage inflammatory protein	-0.43	0.89	0.52	-0.13	18.5	79.5	27.3	1.7	45.8	81.1
IL-10	Interleukin-10	-0.53	0.02	0.13	-0.88	27.8	0.0	1.8	78.0	29.6	78.0
IL-6	Interleukin-6	0.03	0.24	0.42	-0.84	0.1	5.7	17.8	70.4	17.9	76.1
IL-8	Interleukin-8	-0.26	0.18	0.50	-0.82	6.6	3.3	25.2	67.6	31.8	70.9
IL-12p70	Interleukin-12 p70	-0.44	-0.74	-0.14	-0.37	19.3	54.6	2.1	13.6	21.4	68.3
IP-10	Interferon gamma-induced protein 10	-0.31	0.76	-0.70	0.04	9.4	58.5	49.5	0.2	58.9	58.6
Nt	Microneutralization inhibition	-0.03	0.24	0.49	-0.71	0.1	5.7	23.8	50.1	23.8	55.9
MCP-1	Monocyte chemoattractant protein-1	0.10	0.58	0.67	0.31	1.0	34.2	45.4	9.5	46.4	43.7
RANTES	Chemokine (C-C motif) ligand 5	-0.14	0.40	0.26	0.42	2.0	16.2	6.8	17.7	8.8	33.9
Eotaxin-1	Eotaxin-1	-0.43	0.32	0.16	0.09	18.6	9.9	2.5	0.8	21.0	10.8

Table A135: Canonical loadings and explained variance (RNA-Seq, Monocytes, Day 3, IMO variables). IMO variables with at least 25% of explained variance for the first two canonical variates are listed. GX: canonical loadings for canonical variate X, VX: % explained variance for canonical variate X, V1+V2: total % explained variance for both canonical variates. Values for CCA results with a poor cross validation score (< 0.70) are left blank. Sorted by maximum explained variance across treatments (V1+V2).

Gene ID	Gene Name	G1 (SV-AS03)	G1 (SV-PBS)	G2 (SV-AS03)	G2 (SV-PBS)	V1 (SV-AS03)	V1 (SV-PBS)	V2 (SV-AS03)	V2 (SV-PBS)	V1+V2 (SV-AS03)	V1+V2 (SV-PBS)
ENSG00000115085	ZAP70	-0.27	0.88	-0.17	0.38	7.3	77.0	2.9	14.4	10.2	91.3
ENSG00000168685	IL7R	0.23	0.91	0.02	-0.29	5.1	82.5	0.0	8.5	5.1	91.0
ENSG00000081059	TCF7	0.12	0.95	0.01	-0.04	1.4	90.5	0.0	0.2	1.4	90.7
ENSG00000120738	EGR1	0.83	-0.02	-0.46	-0.93	68.9	0.0	21.3	85.6	90.2	85.7
ENSG00000180644	PRF1	0.53	0.50	0.48	0.71	27.9	25.2	23.4	50.3	51.2	75.4
ENSG00000107742	SPOCK2	0.12	0.84	-0.18	-0.09	1.5	71.3	3.1	0.9	4.5	72.1
ENSG00000100385	IL2RB	0.16	0.50	-0.09	0.61	2.4	25.2	0.8	37.1	3.2	62.4
ENSG00000252678	RPPH1	-0.28	0.17	0.70	0.08	7.6	2.9	48.9	0.6	56.6	3.5
ENSG00000213085	CCDC19	0.00	-0.52	-0.03	0.50	0.0	27.3	0.1	24.9	0.1	52.1

Gene ID	Gene Name	G1 (SV-AS03)	G1 (SV-PBS)	G2 (SV-AS03)	G2 (SV-PBS)	V1 (SV-AS03)	V1 (SV-PBS)	V2 (SV-AS03)	V2 (SV-PBS)	V1+V2 (SV-AS03)	V1+V2 (SV-PBS)
ENSG00000090920	FCGBP	-0.45	0.16	-0.38	-0.67	20.5	2.5	14.7	44.8	35.2	47.2
ENSG00000244642	AP005717.1	-0.20	0.26	-0.62	0.56	3.9	7.0	38.5	30.9	42.4	37.9
ENSG00000110848	CD69	0.15	0.58	0.41	-0.30	2.3	33.5	17.2	8.8	19.5	42.3
ENSG00000125968	ID1	-0.16	0.13	0.52	-0.58	2.6	1.7	26.6	33.1	29.1	34.9
ENSG00000211899	IGHM	0.21	-0.00	0.51	0.38	4.4	0.0	25.9	14.4	30.2	14.4
ENSG00000225774	SIRPAP1	-0.02	0.49	-0.16	-0.20	0.0	24.4	2.6	4.1	2.6	28.6
ENSG00000177606	JUN	0.46	0.28	0.20	0.04	20.8	7.6	4.2	0.2	25.0	7.8
ENSG00000134028	ADAMDEC1	-0.18	-0.11	0.44	-0.03	3.1	1.2	19.4	0.1	22.5	1.4
ENSG00000255221	CARD17	-0.13	-0.07	-0.27	0.44	1.8	0.4	7.3	19.8	9.1	20.2
ENSG00000135476	ESPL1	-0.44	-0.03	-0.00	-0.02	19.1	0.1	0.0	0.1	19.1	0.1
ENSG00000254521	SIGLEC12	-0.27	-0.33	-0.16	-0.10	7.5	11.0	2.7	1.0	10.2	11.9

Table A136: Canonical loadings and explained variance (RNA-Seq, Monocytes, Day 28, Gene variables). Gene variables with at least 25% of explained variance for the first two canonical variates are listed. GX: canonical loadings for canonical variate X, VX: % explained variance for canonical variate X, V1+V2: total % explained variance for both canonical variates. Values for CCA results with a poor cross validation score (< 0.70) are left blank. Sorted by maximum explained variance across treatments (V1+V2).

IMO Variable	IMO Variable Name	I1 (SV-AS03)	I1 (SV-PBS)	I2 (SV-AS03)	I2 (SV-PBS)	V1 (SV-AS03)	V1 (SV-PBS)	V2 (SV-AS03)	V2 (SV-PBS)	V1+V2 (SV-AS03)	V1+V2 (SV-PBS)
IL-12p70	Interleukin-12 p70	0.97	-0.06	0.14	-0.28	93.8	0.3	2.0	7.9	95.8	8.2
IL-6	Interleukin-6	0.81	0.50	-0.48	-0.11	65.4	25.4	23.0	1.2	88.4	26.6
IP-10	Interferon gamma-induced protein 10	-0.50	0.28	0.77	-0.22	24.6	7.6	59.0	5.0	83.6	12.5
IL-10	Interleukin-10	0.40	0.65	0.80	0.24	16.0	42.1	63.7	5.6	79.8	47.7
Nt	Microneutralization inhibition	-0.56	-0.38	0.58	0.08	31.0	14.4	33.9	0.6	64.9	15.1
HAI	Hemagglutination inhibition	-0.48	-0.17	0.59	0.13	22.7	2.9	34.2	1.7	57.0	4.6
Eotaxin-1	Eotaxin-1	0.40	-0.07	0.62	0.55	16.1	0.4	38.3	29.8	54.4	30.3
IL-8	Interleukin-8	0.54	0.14	0.48	0.25	29.3	2.0	23.1	6.4	52.5	8.4
IL-1β	Interleukin-1 beta	0.35	0.14	0.60	-0.32	12.2	1.8	35.5	10.4	47.8	12.2
RANTES	Chemokine (C-C motif) ligand 5	0.40	-0.07	0.53	-0.52	16.2	0.5	28.0	27.4	44.1	27.9
TNF	Tumor necrosis factors	0.53	-0.43	0.39	0.16	28.5	18.4	15.2	2.6	43.7	21.0
MCP-1	Monocyte chemoattractant protein-1	0.60	0.27	0.02	0.14	36.6	7.3	0.0	2.0	36.6	9.3
IFN-γ	Interferon gamma	-0.19	0.04	0.27	-0.08	3.5	0.1	7.6	0.7	11.1	0.9
MIP-1α	Macrophage inflammatory protein	-0.06	0.16	-0.10	0.01	0.3	2.4	1.1	0.0	1.4	2.4

Table A137: Canonical loadings and explained variance (RNA-Seq, Monocytes, Day 28, IMO variables). IMO variables with at least 25% of explained variance for the first two canonical variates are listed. GX: canonical loadings for canonical variate X, VX: % explained variance for canonical variate X, V1+V2: total % explained variance for both canonical variates. Values for CCA results with a poor cross validation score (< 0.70) are left blank. Sorted by maximum explained variance across treatments (V1+V2).

Gene ID	Gene Name	G1 (SV-AS03)	G1 (SV-PBS)	G2 (SV-AS03)	G2 (SV-PBS)	V1 (SV-AS03)	V1 (SV-PBS)	V2 (SV-AS03)	V2 (SV-PBS)	V1+V2 (SV-AS03)	V1+V2 (SV-PBS)
ENSG00000101347	SAMHD1	-0.99	-0.38	0.10	0.06	98.5	14.8	1.0	0.3	99.5	15.1
ENSG00000119917	IFIT3	-0.98	-0.40	0.20	0.12	95.3	16.0	4.0	1.4	99.3	17.4
ENSG00000149131	SERPING1	-0.99	0.15	-0.10	-0.03	97.8	2.2	1.1	0.1	98.9	2.3
ENSG00000214872	SMTNL1	-0.99	-0.39	-0.02	0.14	98.8	15.0	0.1	2.0	98.8	16.9
ENSG00000107201	DDX58	-0.99	-0.24	0.02	0.05	98.7	6.0	0.1	0.3	98.8	6.3

Gene ID	Gene Name	G1 (SV-AS03)	G1 (SV-PBS)	G2 (SV-AS03)	G2 (SV-PBS)	V1 (SV-AS03)	V1 (SV-PBS)	V2 (SV-AS03)	V2 (SV-PBS)	V1+V2 (SV-AS03)	V1+V2 (SV-PBS)
ENSG00000183347	GBP6	-0.99	0.32	0.09	0.16	97.8	10.2	0.8	2.6	98.6	12.9
ENSG00000068079	IFI35	-0.97	-0.13	0.18	-0.00	94.9	1.6	3.4	0.0	98.3	1.6
ENSG00000144848	ATG3	-0.91	-0.05	0.40	0.44	82.4	0.2	15.9	19.1	98.3	19.4
ENSG00000115267	IFIH1	-0.99	-0.31	0.10	0.20	97.2	9.4	1.1	4.1	98.2	13.5
ENSG00000132530	XAF1	-0.99	-0.34	0.08	0.16	97.4	11.9	0.7	2.6	98.1	14.5
ENSG00000025708	TYMP	-0.99	0.34	0.04	0.09	98.0	11.7	0.1	0.8	98.1	12.5
ENSG00000152766	ANKRD22	-0.96	0.21	0.22	0.17	92.8	4.5	4.8	3.0	97.6	7.5
ENSG00000225492	GBP1P1	-0.98	0.23	0.15	0.16	95.4	5.3	2.2	2.4	97.5	7.8
ENSG00000154451	GBP5	-0.98	-0.39	0.10	0.14	96.5	15.2	1.0	2.0	97.5	17.2
ENSG00000136147	PHF11	-0.96	-0.34	0.23	0.37	91.9	11.4	5.5	13.8	97.4	25.2
ENSG00000156587	UBE2L6	-0.99	-0.31	-0.01	-0.07	97.3	9.4	0.0	0.5	97.3	9.9
ENSG00000138646	HERC5	-0.98	-0.42	0.14	0.07	95.4	17.7	1.9	0.5	97.3	18.2
ENSG00000157601	MX1	-0.98	-0.34	0.12	0.10	95.5	11.3	1.5	1.0	97.0	12.3
ENSG00000225886	RP11-288L9.4	-0.95	-0.31	0.26	-0.37	90.4	9.4	6.6	14.1	97.0	23.5
ENSG00000112343	TRIM38	-0.98	0.16	0.06	0.09	96.6	2.6	0.3	0.8	97.0	3.5
ENSG00000119922	IFIT2	-0.95	-0.55	0.26	0.11	90.3	30.4	6.6	1.2	97.0	31.6
ENSG00000101342	C20orf118	-0.97	0.10	0.14	-0.03	94.9	1.0	2.1	0.1	96.9	1.1
ENSG00000145246	ATP10D	-0.98	-0.31	-0.04	0.20	96.7	9.7	0.2	3.9	96.9	13.6
ENSG00000134326	CMPK2	-0.96	-0.43	0.21	0.18	92.3	18.9	4.4	3.1	96.8	22.0
ENSG00000254838	GVINP1	-0.98	-0.57	-0.01	-0.01	96.7	32.8	0.0	0.0	96.8	32.8
ENSG00000164308	ERAP2	-0.96	-0.54	-0.23	-0.08	91.5	29.7	5.2	0.7	96.7	30.4
ENSG00000128284	APOL3	-0.98	-0.68	0.02	-0.35	96.7	46.9	0.0	12.5	96.7	59.4
ENSG00000124256	ZBP1	-0.98	-0.14	-0.04	-0.23	96.5	2.1	0.1	5.4	96.7	7.5
ENSG00000173193	PARP14	-0.98	-0.38	0.05	0.06	96.5	14.7	0.2	0.3	96.7	15.1
ENSG00000137965	IFI44	-0.98	-0.37	0.12	0.21	95.2	14.0	1.5	4.5	96.7	18.5
ENSG00000185745	IFIT1	-0.98	-0.48	0.01	0.10	96.4	23.5	0.0	1.0	96.4	24.6
ENSG00000205413	SAMD9	-0.98	-0.36	0.05	0.28	96.1	13.1	0.2	7.8	96.3	20.9
ENSG00000079385	CEACAM1	-0.98	-0.40	0.04	0.13	96.1	16.1	0.2	1.6	96.3	17.7
ENSG00000105967	TFEC	-0.97	-0.20	-0.14	-0.07	94.2	4.2	2.0	0.6	96.2	4.7
ENSG00000100342	APOL1	-0.97	-0.35	0.13	-0.22	94.5	12.0	1.6	4.7	96.2	16.7
ENSG00000137959	IFI44L	-0.98	-0.36	0.06	0.28	95.7	13.3	0.3	8.0	96.1	21.3
ENSG00000141664	ZCCHC2	-0.96	-0.49	0.20	0.19	91.9	24.2	4.1	3.7	96.1	28.0
ENSG00000162654	GBP4	-0.98	-0.60	-0.04	-0.04	95.7	35.9	0.2	0.2	95.9	36.0
ENSG00000178685	PARP10	-0.97	-0.32	0.10	-0.21	95.0	10.2	1.0	4.5	95.9	14.7
ENSG00000185507	IRF7	-0.92	0.03	0.33	0.20	85.3	0.1	10.6	3.9	95.9	4.0
ENSG00000134809	TIMM10	-0.98	-0.20	-0.02	0.41	95.7	4.0	0.0	17.0	95.7	20.9
ENSG00000114127	XRN1	-0.96	-0.31	0.19	-0.08	92.0	9.6	3.7	0.7	95.7	10.3
ENSG00000155629	PIK3AP1	-0.98	-0.27	-0.03	0.07	95.5	7.5	0.1	0.5	95.6	8.0
ENSG00000162772	ATF3	-0.96	0.24	0.17	-0.15	92.7	5.8	2.9	2.2	95.6	8.0
ENSG00000117226	GBP3	-0.98	-0.46	-0.04	0.32	95.1	20.9	0.2	10.1	95.3	30.9
ENSG00000075151	EIF4G3	-0.97	-0.52	0.07	0.25	94.7	26.7	0.5	6.2	95.2	32.9
ENSG00000132256	TRIM5	-0.94	-0.57	0.27	0.38	88.1	32.0	7.0	14.6	95.2	46.5
ENSG00000112137	PHACTR1	0.97	-0.21	-0.07	0.31	94.7	4.6	0.5	9.9	95.1	14.5
ENSG00000133321	RARRES3	-0.97	-0.58	-0.07	-0.13	94.6	33.9	0.6	1.6	95.1	35.5
ENSG00000196116	TDRD7	-0.97	-0.01	0.01	0.16	95.0	0.0	0.0	2.5	95.0	2.5
ENSG00000148834	GSTO1	-0.97	0.20	0.02	-0.29	94.9	4.1	0.0	8.3	95.0	12.4
ENSG00000134321	RSAD2	-0.96	-0.51	0.15	0.01	92.6	25.6	2.3	0.0	95.0	25.7
ENSG00000075399	C16orf7	-0.94	0.56	0.26	0.36	88.2	31.3	6.7	13.0	94.9	44.3
ENSG00000168062	BATF2	-0.96	-0.60	0.14	0.03	92.9	36.3	1.9	0.1	94.9	36.4
ENSG00000010030	ETV7	-0.97	-0.39	0.11	0.17	93.6	15.1	1.3	2.8	94.9	17.9
ENSG00000140464	PML	-0.93	-0.28	0.27	-0.16	87.4	8.1	7.4	2.7	94.8	10.7

Gene ID	Gene Name	G1 (SV-AS03)	G1 (SV-PBS)	G2 (SV-AS03)	G2 (SV-PBS)	V1 (SV-AS03)	V1 (SV-PBS)	V2 (SV-AS03)	V2 (SV-PBS)	V1+V2 (SV-AS03)	V1+V2 (SV-PBS)
ENSG00000137628	DDX60	-0.97	-0.39	0.02	0.06	94.7	15.2	0.0	0.4	94.7	15.6
ENSG00000104805	NUCB1	-0.96	0.09	0.18	-0.24	91.4	0.8	3.3	5.6	94.7	6.4
ENSG00000204264	PSMB8	-0.91	-0.37	0.34	0.12	82.7	14.0	11.9	1.4	94.6	15.3
ENSG00000197536	C5orf56	-0.97	-0.20	-0.06	0.58	94.2	3.9	0.4	33.7	94.6	37.6
ENSG00000130940	CASZ1	-0.97	-0.09	-0.10	-0.08	93.6	0.8	0.9	0.7	94.6	1.5
ENSG00000111331	OAS3	-0.97	-0.49	0.07	0.01	94.1	23.8	0.4	0.0	94.5	23.8
ENSG00000177409	SAMD9L	-0.97	-0.40	0.06	0.06	94.0	16.0	0.4	0.4	94.4	16.4
ENSG00000136874	STX17	-0.96	-0.33	0.12	0.03	92.9	11.0	1.5	0.1	94.3	11.1
ENSG00000123610	TNFAIP6	-0.97	-0.05	0.10	-0.12	93.3	0.2	1.0	1.5	94.3	1.7
ENSG00000185885	IFITM1	-0.97	0.44	0.04	0.03	94.1	19.0	0.1	0.1	94.3	19.1
ENSG00000108771	DHX58	-0.96	0.01	0.16	-0.10	91.7	0.0	2.5	1.0	94.3	1.0
ENSG00000111912	NCOA7	-0.93	-0.45	0.28	-0.53	86.1	20.0	8.0	28.1	94.2	48.1
ENSG00000198133	TMEM229B	-0.97	-0.27	-0.04	0.12	93.9	7.4	0.1	1.4	94.0	8.9
ENSG00000079263	SP140	-0.96	-0.59	-0.10	-0.06	93.1	34.5	0.9	0.3	94.0	34.9
ENSG00000145685	LHFPL2	-0.95	-0.53	0.17	0.32	90.9	27.9	3.0	10.1	93.9	38.0
ENSG00000168394	TAP1	-0.93	-0.34	0.28	0.06	86.3	11.8	7.6	0.4	93.9	12.2
ENSG00000129667	RHBDF2	-0.95	-0.22	0.19	-0.34	90.2	4.8	3.5	11.5	93.8	16.3
ENSG00000174944	P2RY14	-0.97	-0.36	0.03	-0.07	93.5	12.9	0.1	0.5	93.6	13.5
ENSG00000136770	DNAJC1	-0.97	-0.49	0.02	0.24	93.5	23.9	0.0	6.0	93.6	29.9
ENSG00000132274	TRIM22	-0.95	-0.09	0.17	0.20	90.7	0.7	2.9	3.9	93.6	4.7
ENSG00000177989	ODF3B	-0.97	0.51	0.02	-0.03	93.4	25.5	0.0	0.1	93.5	25.6
ENSG00000074660	SCARF1	-0.94	-0.06	0.21	-0.03	89.1	0.3	4.3	0.1	93.4	0.4
ENSG00000126709	IFI6	-0.96	-0.22	0.07	-0.03	92.8	5.1	0.5	0.1	93.3	5.2
ENSG00000129673	AANAT	-0.96	0.45	0.14	0.56	91.3	20.0	2.0	31.1	93.3	51.1
ENSG00000176454	LPCAT4	0.96	0.23	0.12	-0.26	91.9	5.1	1.4	6.8	93.3	11.9
ENSG00000131979	GCH1	-0.97	-0.11	-0.04	0.04	93.1	1.3	0.1	0.2	93.3	1.5
ENSG00000133106	EPSTI1	-0.97	-0.58	-0.02	0.00	93.2	33.8	0.1	0.0	93.2	33.8
ENSG00000155363	MOV10	-0.97	-0.30	0.01	0.00	93.2	9.1	0.0	0.0	93.2	9.1
ENSG00000138134	STAMBPL1	-0.74	-0.33	0.63	-0.23	54.0	11.0	39.1	5.2	93.2	16.2
ENSG00000059378	PARP12	-0.95	-0.40	0.17	0.06	90.0	16.0	2.7	0.3	92.8	16.3
ENSG00000117228	GBP1	-0.96	-0.48	0.09	0.23	91.9	23.3	0.9	5.3	92.8	28.6
ENSG00000168016	TRANK1	-0.96	-0.62	0.03	-0.43	92.6	38.2	0.1	18.4	92.7	56.5
ENSG00000070501	POLB	-0.96	-0.47	0.09	0.22	91.9	22.3	0.7	4.7	92.7	27.0
ENSG00000134243	SORT1	-0.83	-0.37	0.49	-0.08	68.5	13.9	24.1	0.6	92.7	14.5
ENSG00000087253	LPCAT2	-0.95	-0.22	0.14	0.12	90.7	5.0	1.9	1.4	92.7	6.3
ENSG00000105402	NAPA	-0.90	-0.39	0.34	-0.10	81.1	14.9	11.5	0.9	92.6	15.8
ENSG00000130589	RP4-697K14.7	-0.92	-0.03	0.28	-0.07	84.7	0.1	7.8	0.4	92.5	0.6
ENSG00000013374	NUB1	-0.96	-0.60	0.07	-0.05	91.8	36.3	0.6	0.2	92.4	36.6
ENSG00000111335	OAS2	-0.95	-0.26	0.12	0.37	90.8	7.0	1.6	13.5	92.3	20.4
ENSG00000108292	MLLT6	-0.91	0.04	0.32	-0.40	82.0	0.2	10.3	16.0	92.3	16.2
ENSG00000239713	APOBEC3G	-0.96	-0.51	0.06	0.04	91.9	25.9	0.4	0.1	92.3	26.1
ENSG00000078081	LAMP3	-0.96	-0.03	0.04	0.03	92.0	0.1	0.2	0.1	92.2	0.1
ENSG00000128335	APOL2	-0.95	-0.30	0.10	0.05	91.2	9.3	0.9	0.3	92.1	9.6
ENSG00000068784	SRBD1	-0.94	-0.64	-0.21	-0.21	87.5	40.7	4.6	4.5	92.1	45.2
ENSG00000186470	BTN3A2	-0.96	-0.61	-0.03	0.00	91.9	37.8	0.1	0.0	92.0	37.8
ENSG00000240065	PSMB9	-0.95	-0.15	0.16	-0.10	89.4	2.2	2.6	1.1	92.0	3.3
ENSG00000107957	SH3PXD2A	-0.96	-0.58	0.06	-0.12	91.6	33.6	0.4	1.5	92.0	35.1
ENSG00000089127	OAS1	-0.96	-0.48	0.05	0.23	91.7	22.6	0.2	5.4	92.0	28.0
ENSG00000106683	LIMK1	-0.95	-0.59	0.12	0.09	90.5	34.9	1.4	0.8	91.9	35.7
ENSG00000225131	PSME2P2	-0.95	-0.31	-0.09	0.21	90.9	9.5	0.9	4.2	91.8	13.8
ENSG00000132109	TRIM21	-0.95	-0.14	0.12	0.27	90.2	1.9	1.3	7.1	91.5	9.0

Gene ID	Gene Name	G1 (SV-AS03)	G1 (SV-PBS)	G2 (SV-AS03)	G2 (SV-PBS)	V1 (SV-AS03)	V1 (SV-PBS)	V2 (SV-AS03)	V2 (SV-PBS)	V1+V2 (SV-AS03)	V1+V2 (SV-PBS)
ENSG00000145365	TIFA	-0.90	-0.22	0.32	0.03	81.1	4.9	10.3	0.1	91.4	5.0
ENSG00000137200	FTSJD2	-0.93	-0.39	0.20	-0.20	87.3	14.8	4.1	3.9	91.4	18.7
ENSG00000184898	RBM43	-0.92	-0.54	0.25	0.21	84.9	29.4	6.5	4.5	91.4	33.9
ENSG00000183421	RIPK4	0.91	0.53	-0.30	0.10	82.3	28.0	9.0	1.1	91.3	29.1
ENSG00000170581	STAT2	-0.94	-0.32	0.14	0.20	89.1	10.4	1.9	4.0	91.0	14.4
ENSG00000119397	CEP110	-0.95	-0.35	-0.05	0.14	90.7	11.9	0.3	1.9	90.9	13.8
ENSG00000008283	CYB561	-0.95	-0.44	0.11	-0.11	89.7	19.2	1.1	1.3	90.8	20.4
ENSG00000203666	EFCAB2	-0.95	0.74	0.05	0.25	90.5	55.0	0.2	6.1	90.8	61.1
ENSG00000144893	MED12L	-0.90	-0.06	0.31	-0.31	80.8	0.4	9.8	9.7	90.6	10.1
ENSG00000225342	AC079630.4	-0.95	-0.68	0.02	0.27	90.6	45.6	0.0	7.3	90.6	52.9
ENSG00000130303	BST2	-0.94	-0.05	0.12	0.14	89.1	0.2	1.5	1.9	90.6	2.1
ENSG00000038274	MAT2B	-0.94	-0.51	0.16	0.06	88.2	25.7	2.4	0.4	90.6	26.1
ENSG00000174749	C4orf32	-0.88	-0.33	0.35	-0.00	78.1	11.0	12.5	0.0	90.6	11.0
ENSG00000075303	SLC25A40	0.91	-0.65	0.28	0.12	82.4	42.0	8.1	1.4	90.5	43.3
ENSG00000023171	GRAMD1B	-0.95	-0.59	-0.00	-0.30	90.5	34.4	0.0	9.0	90.5	43.4
ENSG00000158411	MITD1	-0.91	0.07	0.28	0.35	82.5	0.6	8.0	12.5	90.5	13.1
ENSG00000122729	ACO1	-0.95	-0.03	-0.01	0.15	90.3	0.1	0.0	2.3	90.4	2.4
ENSG00000139597	N4BP2L1	-0.94	-0.38	0.16	-0.00	87.7	14.6	2.7	0.0	90.3	14.6
ENSG00000204267	TAP2	-0.95	-0.54	0.05	0.11	90.0	29.2	0.3	1.3	90.2	30.5
ENSG00000111860	C6orf204	0.94	-0.53	0.13	-0.33	88.6	27.7	1.6	11.0	90.2	38.7
ENSG00000152778	IFIT5	-0.94	-0.41	0.14	0.28	88.2	17.0	2.0	8.0	90.2	25.0
ENSG00000173821	RNF213	-0.95	-0.26	-0.02	0.12	90.1	6.5	0.0	1.5	90.2	8.0
ENSG00000135900	MRPL44	-0.94	-0.46	0.09	0.13	89.1	21.2	0.8	1.6	89.9	22.7
ENSG00000216490	IFI30	-0.94	0.05	0.14	-0.16	87.7	0.3	2.1	2.5	89.8	2.8
ENSG00000164715	LMTK2	-0.88	-0.23	0.36	-0.18	76.8	5.2	13.0	3.1	89.8	8.2
ENSG00000118162	KPTN	-0.90	0.12	0.31	0.02	80.1	1.4	9.6	0.0	89.7	1.4
ENSG00000157693	C9orf91	-0.95	-0.53	0.00	-0.15	89.6	27.7	0.0	2.3	89.6	30.0
ENSG00000228318	AP001610.5	-0.94	0.30	0.10	-0.07	88.6	9.2	0.9	0.6	89.5	9.8
ENSG00000168899	VAMP5	-0.95	-0.22	-0.01	-0.18	89.5	5.0	0.0	3.4	89.5	8.4
ENSG00000204516	MICB	-0.94	-0.55	0.06	0.08	89.0	29.8	0.4	0.6	89.4	30.4
ENSG00000092010	PSME1	-0.94	-0.56	-0.10	-0.06	88.3	31.5	1.1	0.4	89.3	31.9
ENSG00000135114	OASL	-0.93	-0.40	0.15	0.09	87.0	15.6	2.3	0.9	89.3	16.5
ENSG00000110492	MDK	-0.89	-0.41	-0.32	0.48	79.3	17.1	9.9	22.9	89.3	40.0
ENSG00000211899	IGHM	-0.57	0.01	-0.75	-0.30	33.0	0.0	56.3	9.2	89.3	9.2
ENSG00000125826	RBCK1	-0.94	-0.04	0.11	-0.12	88.0	0.2	1.2	1.3	89.2	1.5
ENSG00000130489	SCO2	-0.94	0.01	-0.07	0.32	88.7	0.0	0.5	10.3	89.2	10.3
ENSG00000085449	WDFY1	-0.94	-0.57	-0.04	-0.28	89.0	32.8	0.2	8.0	89.1	40.9
ENSG00000255848	AC007326.1	-0.94	0.00	0.02	0.19	89.1	0.0	0.0	3.6	89.1	3.6
ENSG00000155506	LARP1	-0.90	-0.67	0.29	-0.43	80.7	45.4	8.3	18.4	89.0	63.8
ENSG00000187608	ISG15	-0.94	-0.20	0.11	-0.08	87.7	4.2	1.3	0.6	89.0	4.8
ENSG00000126351	THRA	0.94	-0.27	-0.11	-0.31	87.8	7.2	1.2	9.5	89.0	16.7
ENSG00000185404	SP140L	-0.94	-0.51	0.06	0.09	88.6	26.0	0.3	0.8	88.9	26.8
ENSG00000102445	C13orf18	0.93	-0.49	-0.12	0.37	87.2	24.0	1.5	14.0	88.7	38.0
ENSG00000182108	DEXI	-0.94	-0.36	0.10	-0.64	87.6	13.3	1.0	41.2	88.6	54.4
ENSG00000026103	FAS	-0.90	-0.27	0.26	0.09	81.7	7.1	6.9	0.9	88.6	8.0
ENSG00000136732	GYPC	-0.92	0.07	0.20	-0.07	84.5	0.4	4.1	0.4	88.6	0.9
ENSG00000128394	APOBEC3F	-0.93	-0.44	-0.11	0.25	87.4	19.3	1.2	6.2	88.6	25.5
ENSG00000221963	APOL6	-0.94	-0.33	0.03	0.23	88.4	10.8	0.1	5.1	88.5	15.9
ENSG00000169871	TRIM56	-0.91	0.30	0.23	-0.62	83.3	8.8	5.2	39.0	88.4	47.8
ENSG00000012124	CD22	-0.73	0.13	-0.59	-0.61	53.2	1.8	35.2	37.2	88.4	39.0
ENSG00000163251	FZD5	-0.86	-0.23	0.38	0.08	73.7	5.2	14.7	0.7	88.3	5.9

Gene ID	Gene Name	G1 (SV-AS03)	G1 (SV-PBS)	G2 (SV-AS03)	G2 (SV-PBS)	V1 (SV-AS03)	V1 (SV-PBS)	V2 (SV-AS03)	V2 (SV-PBS)	V1+V2 (SV-AS03)	V1+V2 (SV-PBS)
ENSG00000116663	FBXO6	-0.91	0.14	0.22	0.04	83.3	2.0	5.0	0.2	88.2	2.2
ENSG00000170542	SERPINB9	-0.94	0.72	0.04	-0.01	88.0	52.0	0.2	0.0	88.2	52.0
ENSG00000198948	MFAP3L	0.83	-0.61	-0.43	0.00	69.7	36.8	18.5	0.0	88.2	36.8
ENSG00000107798	LIPA	-0.92	-0.57	0.19	0.34	84.5	32.6	3.6	11.3	88.1	43.9
ENSG00000247275	AL160008.1	-0.94	-0.47	0.04	0.06	87.8	21.7	0.2	0.4	88.0	22.1
ENSG00000140105	WARS	-0.93	-0.69	0.09	-0.21	87.1	47.5	0.8	4.6	87.9	52.1
ENSG00000125967	NECAB3	0.86	-0.35	0.38	0.19	73.1	12.5	14.7	3.7	87.8	16.2
ENSG00000223865	HLA-DPB1	-0.51	-0.10	-0.79	0.05	25.5	1.0	62.3	0.2	87.8	1.2
ENSG00000100911	PSME2	-0.93	-0.65	-0.06	0.02	87.4	41.9	0.4	0.0	87.8	41.9
ENSG00000129003	VPS13C	-0.91	-0.55	-0.20	-0.11	83.7	30.6	4.0	1.1	87.7	31.7
ENSG00000104312	RIPK2	-0.88	-0.37	0.32	-0.08	77.4	13.6	10.3	0.6	87.7	14.3
ENSG00000122643	NT5C3	-0.93	-0.39	-0.14	0.17	85.6	15.2	2.1	2.8	87.7	17.9
ENSG00000135148	TRAFD1	-0.85	-0.63	0.38	0.12	72.9	39.4	14.8	1.3	87.7	40.7
ENSG00000135604	STX11	-0.89	0.02	0.28	0.24	79.7	0.0	7.9	6.0	87.6	6.0
ENSG00000102554	KLF5	-0.75	-0.05	0.56	0.01	56.1	0.3	31.5	0.0	87.6	0.3
ENSG00000162645	GBP2	-0.92	-0.30	0.19	0.33	83.8	8.9	3.7	10.6	87.6	19.6
ENSG00000141574	SECTM1	-0.89	0.24	0.28	0.03	79.9	6.0	7.7	0.1	87.6	6.1
ENSG00000100401	RANGAP1	-0.88	-0.13	0.32	-0.01	77.4	1.7	10.1	0.0	87.5	1.7
ENSG00000048140	TSPAN17	-0.85	-0.14	0.40	0.08	71.5	2.1	16.0	0.7	87.5	2.8
ENSG00000111801	BTN3A3	-0.93	-0.71	-0.04	-0.19	87.3	49.8	0.2	3.8	87.5	53.6
ENSG00000065427	KARS	-0.91	-0.33	0.23	-0.19	82.0	10.9	5.5	3.6	87.4	14.6
ENSG00000108387	SEPT4	-0.93	-0.12	-0.03	0.15	87.4	1.6	0.1	2.4	87.4	3.9
ENSG00000204287	HLA-DRA	-0.91	-0.25	-0.22	-0.23	82.5	6.0	4.9	5.2	87.4	11.2
ENSG00000115109	EPB41L5	-0.87	-0.37	0.34	-0.09	75.8	13.5	11.5	0.9	87.3	14.4
ENSG00000179344	HLA-DQB1	-0.93	-0.26	0.03	-0.18	87.1	6.9	0.1	3.3	87.2	10.1
ENSG00000166002	C11orf75	-0.91	0.48	0.20	0.14	83.1	23.2	4.0	2.0	87.1	25.2
ENSG00000166801	FAM111A	-0.92	-0.70	-0.14	0.08	85.0	48.5	2.1	0.7	87.1	49.2
ENSG00000183688	FAM101B	0.89	-0.61	-0.28	-0.29	79.3	37.3	7.7	8.4	87.1	45.7
ENSG00000119950	MXI1	0.91	-0.15	0.21	-0.71	82.5	2.3	4.3	51.0	86.9	53.3
ENSG00000153029	MR1	-0.92	-0.15	0.11	0.17	85.5	2.4	1.2	2.8	86.7	5.2
ENSG00000136514	RTP4	-0.92	-0.41	-0.15	-0.41	84.2	16.7	2.4	16.9	86.6	33.6
ENSG00000115956	PLEK	-0.88	0.10	0.30	0.11	77.3	1.1	9.3	1.1	86.6	2.2
ENSG00000140030	GPR65	-0.93	-0.14	0.08	0.02	85.9	2.1	0.7	0.1	86.6	2.1
ENSG00000187741	FANCA	-0.89	0.01	0.26	-0.42	79.7	0.0	6.9	17.5	86.6	17.5
ENSG00000136573	BLK	-0.43	-0.08	-0.82	0.08	18.7	0.7	67.7	0.6	86.3	1.3
ENSG00000179583	CIITA	-0.92	-0.30	0.14	-0.15	84.3	8.7	2.0	2.2	86.3	10.9
ENSG00000168404	MLKL	-0.73	-0.18	0.58	0.22	53.2	3.2	33.1	4.9	86.3	8.1
ENSG00000104518	GSDMD	-0.90	0.03	0.21	-0.12	81.8	0.1	4.4	1.5	86.2	1.6
ENSG00000160216	AGPAT3	-0.92	-0.56	-0.11	0.01	84.9	31.1	1.3	0.0	86.2	31.1
ENSG00000176438	C14orf49	0.89	0.58	-0.25	-0.25	79.8	33.4	6.4	6.5	86.2	39.8
ENSG00000155287	SLC25A28	-0.92	-0.22	0.12	0.10	84.6	4.8	1.6	1.0	86.1	5.8
ENSG00000233552	AL139035.1	-0.92	-0.39	0.13	0.11	84.5	15.0	1.6	1.1	86.1	16.1
ENSG00000164691	TAGAP	-0.77	-0.30	0.52	0.39	58.8	8.9	27.2	15.0	86.0	23.9
ENSG00000197142	ACSL5	-0.93	-0.19	0.01	-0.40	86.0	3.6	0.0	15.6	86.0	19.3
ENSG00000185989	RASA3	0.89	-0.32	0.25	-0.64	79.5	10.0	6.4	40.6	86.0	50.6
ENSG00000100292	HMOX1	0.92	-0.08	0.06	-0.12	85.5	0.6	0.4	1.5	85.9	2.1
ENSG00000067221	STOML1	-0.87	-0.02	0.32	-0.09	75.6	0.0	10.2	0.9	85.8	0.9
ENSG00000099860	GADD45B	-0.86	0.06	0.34	0.06	74.5	0.3	11.3	0.4	85.8	0.7
ENSG00000197555	SIPA1L1	-0.93	-0.28	0.05	-0.41	85.6	7.9	0.2	16.9	85.8	24.7
ENSG00000242574	HLA-DMB	-0.92	-0.34	-0.05	0.23	85.3	11.6	0.3	5.4	85.6	17.1
ENSG00000234518	RP5-837D10.2	-0.89	-0.28	0.25	0.48	79.3	7.7	6.2	23.5	85.5	31.2

Gene ID	Gene Name	G1 (SV-AS03)	G1 (SV-PBS)	G2 (SV-AS03)	G2 (SV-PBS)	V1 (SV-AS03)	V1 (SV-PBS)	V2 (SV-AS03)	V2 (SV-PBS)	V1+V2 (SV-AS03)	V1+V2 (SV-PBS)
ENSG00000203814	HIST2H2BF	-0.80	0.04	0.45	0.30	64.8	0.2	20.7	8.7	85.4	8.9
ENSG00000034510	TMSB10	-0.91	-0.46	-0.14	-0.14	83.5	21.0	1.9	2.1	85.4	23.1
ENSG00000106560	GIMAP2	-0.92	-0.05	0.09	-0.11	84.6	0.3	0.8	1.1	85.3	1.4
ENSG00000106049	HIBADH	0.38	-0.91	0.25	-0.16	14.4	82.8	6.5	2.4	20.9	85.3
ENSG00000229677	RP11-383F6.1	-0.74	0.52	0.55	0.43	55.2	27.3	30.0	18.7	85.2	46.0
ENSG00000185022	MAFF	-0.67	0.05	0.64	-0.07	44.5	0.3	40.6	0.5	85.1	0.8
ENSG00000065911	MTHFD2	-0.90	-0.33	0.19	0.29	81.4	11.0	3.6	8.7	85.1	19.6
ENSG00000163875	MEAF6	0.89	-0.33	-0.26	-0.31	78.4	10.6	6.6	9.4	85.1	20.0
ENSG00000250233	AC015802.1	-0.92	-0.03	0.10	-0.08	84.1	0.1	0.9	0.6	85.0	0.7
ENSG00000124795	DEK	-0.87	-0.11	0.31	0.36	75.2	1.1	9.8	12.9	85.0	14.0
ENSG00000148110	HIATL1	-0.92	0.00	0.01	-0.08	84.9	0.0	0.0	0.7	84.9	0.7
ENSG00000247982	AC016525.1	-0.56	0.38	-0.73	-0.07	31.8	14.3	53.1	0.5	84.9	14.8
ENSG00000135378	PRRG4	-0.92	-0.50	0.04	0.54	84.6	24.9	0.2	29.5	84.8	54.3
ENSG00000176871	WSB2	-0.92	-0.25	0.09	-0.18	84.1	6.0	0.7	3.3	84.8	9.3
ENSG00000198087	CD2AP	-0.92	-0.68	-0.02	0.18	84.7	46.8	0.0	3.1	84.7	49.8
ENSG00000110848	CD69	-0.84	-0.55	0.38	0.12	70.3	30.5	14.4	1.4	84.6	31.9
ENSG00000128203	ASPHD2	-0.90	-0.45	-0.21	-0.17	80.3	20.2	4.4	2.8	84.6	22.9
ENSG00000177738	CTD-2201E18.3	-0.79	0.28	0.48	0.22	61.8	7.6	22.8	4.6	84.6	12.3
ENSG00000168906	MAT2A	0.91	-0.28	0.15	-0.59	82.3	7.7	2.2	35.4	84.5	43.1
ENSG00000155561	NUP205	-0.92	-0.55	0.06	0.13	84.0	29.9	0.4	1.6	84.4	31.5
ENSG00000035664	DAPK2	0.90	-0.62	-0.18	-0.19	81.0	38.5	3.3	3.8	84.3	42.3
ENSG00000168264	IRF2BP2	0.88	-0.13	-0.26	-0.42	77.8	1.8	6.5	17.7	84.3	19.5
ENSG00000123609	NMI	-0.90	-0.23	0.19	0.26	80.6	5.2	3.7	6.8	84.3	12.0
ENSG00000172159	FRMD3	-0.87	-0.90	-0.10	-0.20	76.4	80.1	1.0	4.1	77.3	84.2
ENSG00000152689	RASGRP3	-0.80	0.15	-0.45	0.51	64.3	2.2	19.8	25.8	84.2	28.1
ENSG00000148175	STOM	-0.91	-0.02	0.15	0.07	81.9	0.1	2.1	0.5	84.0	0.6
ENSG00000254503	CTD-2521M24.4	-0.91	-0.21	0.08	0.13	83.2	4.6	0.6	1.6	83.9	6.2
ENSG00000180628	PCGF5	-0.91	-0.57	-0.11	0.06	82.7	32.4	1.1	0.4	83.8	32.8
ENSG00000124875	CXCL6	0.91	0.04	-0.14	0.36	81.9	0.2	1.9	12.7	83.8	12.9
ENSG00000198216	CACNA1E	-0.91	0.48	-0.12	-0.14	82.2	22.9	1.5	2.0	83.8	24.8
ENSG00000107960	OBFC1	-0.85	-0.09	0.33	0.25	72.7	0.9	11.0	6.1	83.7	6.9
ENSG00000185338	SOCS1	-0.83	-0.74	0.39	0.19	68.5	54.3	15.1	3.8	83.7	58.0
ENSG00000163840	DTX3L	-0.89	-0.35	0.21	0.33	79.2	12.2	4.2	10.6	83.4	22.7
ENSG00000233030	RP11-196G18.3	-0.91	0.09	0.04	0.24	82.9	0.9	0.2	5.9	83.1	6.8
ENSG00000179833	SERTAD2	0.91	-0.51	-0.05	0.45	82.8	26.2	0.2	20.7	83.0	46.9
ENSG00000152229	PSTPIP2	-0.90	-0.33	0.13	0.43	81.2	10.7	1.8	18.2	83.0	28.9
ENSG00000211898	IGHD	-0.45	0.18	-0.79	0.30	20.2	3.3	62.8	8.9	82.9	12.1
ENSG00000055332	EIF2AK2	-0.90	-0.11	0.16	0.41	80.4	1.3	2.5	16.6	82.9	17.9
ENSG00000168421	RHOH	-0.77	-0.81	0.31	-0.41	59.5	66.3	9.6	16.6	69.2	82.9
ENSG00000106100	NOD1	-0.91	-0.82	0.05	-0.25	82.4	67.8	0.3	6.4	82.7	74.2
ENSG00000196126	HLA-DRB1	-0.80	-0.15	-0.43	0.42	64.0	2.4	18.5	17.3	82.5	19.7
ENSG00000253193	FCGR1C	-0.88	0.15	0.21	0.16	78.1	2.1	4.3	2.6	82.4	4.8
ENSG00000143390	RFX5	-0.90	-0.16	-0.12	-0.32	81.0	2.5	1.4	10.5	82.4	13.0
ENSG00000160932	LY6E	-0.89	-0.19	0.18	0.22	78.9	3.4	3.4	4.8	82.3	8.3
ENSG00000183784	C9orf66	-0.86	-0.08	0.28	0.24	74.3	0.7	7.9	5.6	82.2	6.3
ENSG00000090339	ICAM1	-0.84	0.45	0.33	-0.21	71.2	20.5	10.9	4.5	82.1	25.1
ENSG00000132470	ITGB4	0.88	0.20	0.20	-0.14	78.0	3.9	4.1	2.0	82.1	5.9
ENSG00000128346	C22orf23	-0.87	-0.36	0.24	-0.41	76.1	12.7	6.0	16.6	82.1	29.4
ENSG00000028116	VRK2	-0.90	-0.29	0.07	-0.69	81.6	8.6	0.5	47.2	82.1	55.7
ENSG00000228203	AC017076.4	-0.90	-0.19	0.06	-0.37	81.7	3.8	0.4	13.6	82.1	17.4
ENSG00000138660	AP1AR	-0.80	-0.34	0.42	0.05	64.3	11.7	17.7	0.3	82.0	12.0

Gene ID	Gene Name	G1 (SV-AS03)	G1 (SV-PBS)	G2 (SV-AS03)	G2 (SV-PBS)	V1 (SV-AS03)	V1 (SV-PBS)	V2 (SV-AS03)	V2 (SV-PBS)	V1+V2 (SV-AS03)	V1+V2 (SV-PBS)
ENSG00000197448	GSTK1	-0.86	0.58	0.28	-0.01	74.2	34.1	7.8	0.0	82.0	34.1
ENSG00000172493	AFF1	-0.87	-0.05	0.23	0.33	76.4	0.2	5.5	10.6	82.0	10.8
ENSG00000135362	PRR5L	-0.83	-0.25	0.35	0.15	69.5	6.3	12.5	2.4	82.0	8.7
ENSG00000163644	PPM1K	-0.84	-0.67	-0.33	-0.42	71.0	44.6	10.9	18.0	81.9	62.6
ENSG00000254470	RP11-770G2.3	-0.86	0.36	0.30	0.39	73.1	12.9	8.7	15.4	81.8	28.3
ENSG00000181381	DDX60L	-0.90	0.07	0.03	0.32	81.7	0.5	0.1	10.1	81.8	10.6
ENSG00000167208	SNX20	-0.88	-0.24	0.21	0.25	77.2	6.0	4.6	6.2	81.8	12.2
ENSG00000140968	IRF8	-0.68	0.06	-0.59	-0.35	46.6	0.3	35.1	12.2	81.6	12.5
ENSG00000124785	NRN1	-0.85	0.15	0.30	0.30	72.8	2.3	8.7	9.3	81.6	11.6
ENSG00000047621	C12orf4	-0.75	-0.25	0.51	0.06	56.0	6.3	25.6	0.3	81.5	6.6
ENSG00000234571	RP5-998N21.4	-0.90	0.20	-0.07	0.47	80.9	4.2	0.5	22.0	81.4	26.2
ENSG00000075240	GRAMD4	0.89	-0.74	0.17	-0.25	78.5	55.4	2.9	6.2	81.4	61.6
ENSG00000172183	ISG20	-0.90	-0.28	0.08	-0.16	80.7	7.7	0.7	2.7	81.3	10.4
ENSG00000126246	IGFLR1	-0.79	0.34	0.43	-0.15	62.9	11.8	18.4	2.3	81.3	14.1
ENSG00000136104	RNASEH2B	-0.90	-0.25	0.08	-0.15	80.7	6.3	0.6	2.3	81.3	8.6
ENSG00000100908	FAM158A	-0.90	-0.32	0.00	0.27	81.3	10.3	0.0	7.5	81.3	17.8
ENSG00000159459	UBR1	-0.86	-0.07	0.28	0.25	73.4	0.5	7.8	6.4	81.2	6.9
ENSG00000204345	CD300LD	-0.71	-0.36	0.56	-0.68	50.1	13.3	31.0	46.8	81.1	60.1
ENSG00000236943	AL592284.1_236943	-0.84	-0.50	0.34	0.57	69.8	24.8	11.3	32.1	81.1	56.9
ENSG00000205730	ITPRIPL2	-0.86	0.31	0.28	-0.17	73.3	9.6	7.8	2.8	81.1	12.4
ENSG00000121858	TNFSF10	-0.85	-0.24	0.29	0.23	72.7	5.8	8.4	5.4	81.0	11.3
ENSG00000254667	AP000783.1	-0.90	0.05	0.08	-0.03	80.2	0.2	0.7	0.1	80.9	0.3
ENSG00000120217	CD274	-0.90	0.02	-0.05	0.36	80.5	0.0	0.3	12.8	80.8	12.8
ENSG00000235999	RP11-403I13.8	-0.90	-0.17	0.05	0.26	80.5	2.8	0.2	6.7	80.7	9.5
ENSG00000177119	ANO6	-0.88	-0.06	-0.16	0.11	78.0	0.3	2.7	1.2	80.6	1.5
ENSG00000115392	FANCL	-0.87	0.02	0.22	0.13	75.6	0.0	4.8	1.7	80.4	1.7
ENSG00000133574	GIMAP4	-0.89	-0.54	0.11	-0.02	79.1	29.2	1.3	0.0	80.4	29.3
ENSG00000237927	RP3-393E18.2	-0.90	-0.14	0.04	0.12	80.2	1.9	0.2	1.4	80.4	3.2
ENSG00000211895	IGHA1	-0.59	0.34	-0.67	-0.09	35.0	11.2	45.3	0.7	80.3	12.0
ENSG00000196141	SPATS2L	-0.88	-0.11	0.16	-0.30	77.8	1.3	2.5	8.8	80.3	10.1
ENSG00000156738	MS4A1	-0.59	0.47	-0.68	0.06	34.3	22.3	45.9	0.3	80.2	22.6
ENSG00000166750	SLFN5	-0.89	-0.30	-0.11	-0.24	78.8	8.7	1.3	5.5	80.1	14.3
ENSG00000104921	FCER2	-0.49	0.23	-0.75	-0.21	23.9	5.1	56.1	4.5	80.0	9.6
ENSG00000138496	PARP9	-0.89	-0.07	0.02	0.41	79.9	0.4	0.0	16.4	80.0	16.9
ENSG00000134070	IRAK2	-0.88	0.15	0.15	-0.10	77.7	2.2	2.2	1.0	79.9	3.2
ENSG00000162714	ZNF496	-0.88	0.39	0.13	-0.03	78.2	14.9	1.7	0.1	79.9	15.0
ENSG00000249173	RP11-701P16.4	-0.35	0.76	0.28	0.46	12.4	58.4	7.6	21.4	19.9	79.8
ENSG00000135473	PAN2	-0.89	-0.31	-0.11	-0.01	78.6	9.6	1.3	0.0	79.8	9.6
ENSG00000152223	EPG5	-0.78	-0.46	0.43	0.22	61.3	20.7	18.5	4.7	79.8	25.5
ENSG00000152818	UTRN	-0.86	-0.43	-0.26	-0.04	73.1	18.4	6.6	0.2	79.8	18.5
ENSG00000235953	AL353805.1	-0.89	-0.01	0.11	0.26	78.5	0.0	1.2	6.6	79.8	6.6
ENSG00000129038	LOXL1	-0.67	0.57	0.59	0.09	45.1	32.6	34.6	0.8	79.7	33.4
ENSG00000125148	MT2A	-0.89	-0.28	-0.09	-0.02	78.7	7.7	0.8	0.0	79.5	7.8
ENSG00000100918	REC8	-0.88	0.15	0.16	0.38	76.9	2.2	2.6	14.1	79.5	16.3
ENSG00000184221	OLIG1	0.88	-0.48	-0.13	-0.16	77.9	23.0	1.6	2.4	79.4	25.5
ENSG00000128512	DOCK4	-0.89	-0.01	-0.01	-0.31	79.4	0.0	0.0	9.8	79.4	9.8
ENSG00000234515	PPP1R2P1	-0.89	-0.68	-0.06	0.21	79.0	46.0	0.4	4.3	79.4	50.3
ENSG00000167207	NOD2	-0.87	-0.21	0.20	0.37	75.6	4.2	3.8	13.9	79.4	18.1
ENSG00000254859	RP11-661A12.5	-0.89	0.32	0.04	-0.31	79.1	10.0	0.2	9.6	79.3	19.6
ENSG00000187091	PLCD1	0.89	0.01	0.06	-0.34	78.7	0.0	0.3	11.8	79.0	11.8
ENSG00000119537	KDSR	-0.75	0.20	0.48	-0.27	56.3	4.2	22.7	7.4	79.0	11.6

Gene ID	Gene Name	G1 (SV-AS03)	G1 (SV-PBS)	G2 (SV-AS03)	G2 (SV-PBS)	V1 (SV-AS03)	V1 (SV-PBS)	V2 (SV-AS03)	V2 (SV-PBS)	V1+V2 (SV-AS03)	V1+V2 (SV-PBS)
ENSG00000058866	DGKG	0.88	-0.26	0.08	0.27	78.3	6.8	0.6	7.0	78.9	13.9
ENSG00000069424	KCNAB2	0.86	-0.39	0.21	-0.49	74.5	14.9	4.4	24.0	78.9	38.9
ENSG00000179044	EXOC3L1	-0.88	-0.10	-0.09	0.48	78.1	1.0	0.7	23.2	78.8	24.2
ENSG00000064601	CTSA	-0.79	-0.23	0.41	-0.22	62.1	5.4	16.7	4.7	78.8	10.2
ENSG00000104164	PLDN	-0.86	-0.23	0.21	0.23	74.2	5.2	4.5	5.4	78.7	10.7
ENSG00000103642	LACTB	-0.74	-0.02	0.49	0.19	55.0	0.1	23.6	3.5	78.6	3.6
ENSG00000213839	RP11-3J10.4	0.82	0.08	-0.33	-0.17	67.9	0.6	10.8	3.0	78.6	3.7
ENSG00000138642	HERC6	-0.89	-0.07	0.00	-0.12	78.6	0.5	0.0	1.3	78.6	1.8
ENSG00000065613	SLK	-0.76	-0.11	0.45	0.21	58.2	1.2	20.4	4.2	78.6	5.4
ENSG00000107566	ERLIN1	-0.44	0.18	0.77	-0.08	19.2	3.1	59.3	0.7	78.5	3.9
ENSG00000249244	RP11-548H18.2	-0.88	-0.20	0.10	0.40	77.6	4.2	0.9	15.6	78.5	19.8
ENSG00000205220	PSMB10	-0.88	-0.23	0.06	-0.24	78.1	5.4	0.4	5.8	78.5	11.1
ENSG00000196092	PAX5	-0.51	-0.03	-0.72	0.26	26.4	0.1	52.1	6.7	78.4	6.8
ENSG00000136193	SCRN1	-0.63	-0.39	-0.62	-0.45	40.0	14.9	38.3	20.2	78.4	35.1
ENSG00000232680	AC002511.3	-0.89	0.65	-0.01	0.05	78.3	41.9	0.0	0.3	78.3	42.2
ENSG00000090554	FLT3LG	-0.88	-0.44	0.08	0.05	77.6	19.2	0.7	0.2	78.3	19.4
ENSG00000177721	C5orf39	-0.85	-0.65	0.25	-0.17	71.8	42.0	6.5	2.9	78.2	45.0
ENSG00000196123	KIAA0895L	-0.88	0.23	-0.10	0.02	77.1	5.4	1.1	0.0	78.2	5.4
ENSG00000119655	NPC2	-0.88	-0.60	-0.07	-0.02	77.7	36.1	0.5	0.0	78.2	36.2
ENSG00000132780	NASP	-0.86	-0.02	0.18	0.27	74.8	0.0	3.3	7.2	78.1	7.2
ENSG00000175643	C16orf75	-0.84	0.25	0.27	0.48	70.6	6.1	7.4	23.3	78.0	29.4
ENSG00000245602	AC073548.1	-0.81	-0.13	0.34	0.19	66.3	1.6	11.7	3.7	78.0	5.3
ENSG00000143891	GALM	-0.88	-0.63	-0.00	0.19	77.9	39.1	0.0	3.7	77.9	42.8
ENSG00000125347	IRF1	-0.77	-0.05	0.43	0.37	59.5	0.3	18.3	14.1	77.8	14.3
ENSG00000150337	FCGR1A	-0.87	0.30	0.16	0.43	75.3	9.3	2.5	18.7	77.8	27.9
ENSG00000162976	PQLC3	0.85	-0.10	-0.23	-0.38	72.3	1.0	5.5	14.5	77.8	15.5
ENSG00000187231	SESTD1	-0.88	-0.12	-0.11	0.28	76.6	1.5	1.1	7.9	77.8	9.4
ENSG00000164134	NAA15	-0.81	0.02	0.34	0.26	66.1	0.0	11.5	6.7	77.6	6.8
ENSG00000151692	RNF144A	-0.87	-0.60	0.12	0.27	76.2	36.0	1.4	7.3	77.6	43.3
ENSG00000183018	SPNS2	0.87	0.02	-0.11	0.02	76.4	0.0	1.2	0.1	77.6	0.1
ENSG00000103489	XYLT1	0.88	-0.46	-0.10	-0.41	76.6	21.2	0.9	16.6	77.5	37.8
ENSG00000141971	FAM125A	-0.83	-0.45	0.28	0.40	69.5	20.6	8.0	16.3	77.5	37.0
ENSG00000139832	RAB20	-0.74	-0.07	0.47	-0.05	55.3	0.4	22.0	0.3	77.3	0.7
ENSG00000106804	C5	-0.88	-0.24	0.03	-0.10	77.2	5.6	0.1	0.9	77.3	6.5
ENSG00000019582	CD74	-0.87	-0.25	-0.10	-0.39	76.1	6.5	1.1	14.8	77.2	21.3
ENSG00000167705	RILP	-0.84	-0.46	0.25	0.08	70.9	21.3	6.2	0.6	77.1	21.9
ENSG00000110060	PUS3	-0.80	-0.48	0.36	0.67	64.2	22.6	12.9	45.2	77.1	67.8
ENSG00000204396	C6orf27	0.77	0.30	0.43	-0.64	58.9	8.8	18.2	41.1	77.1	49.9
ENSG00000198520	C1orf228	-0.87	-0.79	0.08	-0.33	76.2	63.0	0.6	11.2	76.7	74.2
ENSG00000002549	LAP3	-0.75	-0.50	0.45	-0.03	56.7	25.3	20.0	0.1	76.7	25.3
ENSG00000153064	BANK1	-0.62	-0.29	-0.62	0.11	38.0	8.2	38.6	1.2	76.6	9.3
ENSG00000114737	CISH	-0.65	-0.09	0.59	0.21	42.1	0.9	34.5	4.4	76.6	5.3
ENSG00000168405	CMAHP	-0.87	-0.32	0.11	-0.34	75.4	10.3	1.2	11.6	76.6	21.8
ENSG00000119522	DENND1A	-0.87	0.22	-0.02	-0.12	76.5	4.7	0.0	1.5	76.6	6.2
ENSG00000038210	PI4K2B	-0.78	-0.13	-0.39	0.14	61.6	1.7	15.0	1.9	76.5	3.6
ENSG00000111110	PPM1H	0.82	-0.64	0.30	-0.24	67.3	41.1	9.0	5.7	76.4	46.9
ENSG00000243811	APOBEC3D	-0.87	-0.30	-0.09	0.03	75.5	8.7	0.8	0.1	76.3	8.8
ENSG00000107796	ACTA2	-0.82	0.48	0.30	0.55	67.0	23.3	9.2	30.5	76.2	53.9
ENSG00000143110	C1orf162	0.85	-0.04	-0.17	-0.54	73.0	0.2	3.0	29.6	76.0	29.8
ENSG00000111328	CDK2AP1	0.79	0.44	0.37	-0.20	62.3	19.1	13.7	4.1	76.0	23.2
ENSG00000067066	SP100	-0.86	-0.08	0.15	0.40	73.6	0.7	2.3	15.7	76.0	16.4

Gene ID	Gene Name	G1 (SV-AS03)	G1 (SV-PBS)	G2 (SV-AS03)	G2 (SV-PBS)	V1 (SV-AS03)	V1 (SV-PBS)	V2 (SV-AS03)	V2 (SV-PBS)	V1+V2 (SV-AS03)	V1+V2 (SV-PBS)
ENSG00000106462	EZH2	-0.80	0.59	0.35	-0.03	63.4	34.7	12.4	0.1	75.8	34.9
ENSG00000152558	TMEM123	-0.86	-0.32	-0.15	0.03	73.4	10.5	2.4	0.1	75.8	10.6
ENSG00000108828	VAT1	-0.79	0.44	0.37	-0.09	62.3	19.7	13.4	0.7	75.8	20.4
ENSG00000244509	APOBEC3C	-0.87	-0.75	-0.06	-0.09	75.3	56.2	0.4	0.8	75.7	57.0
ENSG00000143067	ZNF697	0.84	0.04	-0.24	0.55	70.1	0.1	5.6	30.3	75.7	30.4
ENSG00000159176	CSRP1	0.73	-0.39	-0.48	-0.56	52.8	15.5	22.8	31.6	75.6	47.1
ENSG00000122547	EEPD1	0.84	-0.62	-0.23	-0.52	70.1	38.7	5.5	26.7	75.6	65.4
ENSG00000184224	C11orf72	-0.87	0.02	-0.06	0.23	75.1	0.0	0.4	5.3	75.5	5.3
ENSG00000113273	ARSB	-0.85	-0.29	-0.17	-0.41	72.4	8.1	3.0	16.7	75.4	24.9
ENSG00000139180	NDUFA9	-0.86	0.30	0.12	0.32	73.9	9.0	1.5	10.3	75.4	19.4
ENSG00000175073	VCPIP1	-0.78	-0.28	0.37	0.11	61.5	7.9	13.8	1.2	75.4	9.1
ENSG00000183621	ZNF438	-0.77	0.78	0.03	0.38	59.5	60.7	0.1	14.6	59.6	75.2
ENSG00000030110	BAK1	-0.87	-0.46	0.03	0.07	75.1	21.4	0.1	0.5	75.2	21.9
ENSG00000131943	C19orf12	-0.83	-0.39	0.25	0.08	68.8	15.4	6.3	0.7	75.1	16.1
ENSG00000087237	CETP	-0.83	-0.12	0.26	0.51	68.3	1.4	6.7	25.7	75.0	27.1
ENSG00000138385	SSB	-0.84	-0.38	0.22	-0.07	70.2	14.1	4.9	0.5	75.0	14.6
ENSG00000187260	WDR86	-0.87	0.28	-0.02	0.06	74.9	8.1	0.0	0.3	74.9	8.4
ENSG00000211896	IGHG1	-0.57	-0.16	-0.65	-0.29	32.8	2.7	42.0	8.6	74.9	11.3
ENSG00000155158	TTC39B	-0.85	-0.48	0.17	-0.34	71.6	23.0	3.1	11.4	74.7	34.4
ENSG00000255221	CARD17	-0.84	-0.14	-0.22	-0.02	69.9	1.9	4.7	0.0	74.6	1.9
ENSG00000115415	STAT1	-0.85	-0.40	0.13	0.29	72.8	16.0	1.7	8.2	74.5	24.2
ENSG00000241106	HLA-DOB	-0.86	-0.00	-0.02	0.15	74.5	0.0	0.0	2.3	74.5	2.3
ENSG00000051128	HOMER3	0.86	-0.30	0.07	0.02	73.9	9.1	0.5	0.1	74.4	9.2
ENSG00000181788	SIAH2	-0.78	-0.39	0.36	0.41	61.3	14.9	13.0	16.5	74.3	31.4
ENSG00000186265	BTLA	-0.67	-0.26	-0.55	0.40	44.4	6.9	29.8	15.8	74.2	22.7
ENSG00000047579	DTNBP1	-0.59	0.45	0.63	0.17	34.4	20.5	39.8	2.8	74.1	23.3
ENSG00000069020	MAST4	0.54	0.86	0.17	-0.06	29.5	73.7	3.0	0.4	32.5	74.1
ENSG00000105939	ZC3HAV1	-0.71	0.24	0.48	0.36	50.9	5.5	23.2	13.2	74.1	18.8
ENSG00000176170	SPHK1	-0.78	0.60	0.37	0.09	60.4	36.2	13.7	0.9	74.1	37.1
ENSG00000100889	PCK2	-0.86	-0.34	-0.04	-0.45	73.8	11.8	0.2	20.2	73.9	32.0
ENSG00000143162	CREG1	-0.67	-0.58	0.53	0.23	45.4	34.0	28.5	5.2	73.9	39.1
ENSG00000225964	AC017076.5	-0.86	-0.29	0.07	0.29	73.4	8.5	0.4	8.7	73.8	17.2
ENSG00000013583	HEBP1	-0.83	-0.59	-0.23	-0.46	68.3	35.0	5.4	21.6	73.8	56.6
ENSG00000251136	RP11-37B2.1	-0.84	0.31	0.18	0.25	70.3	9.4	3.3	6.2	73.7	15.6
ENSG00000140853	NLRC5	-0.84	-0.47	0.19	0.11	69.9	22.5	3.7	1.3	73.6	23.8
ENSG00000141086	CTRL	-0.84	-0.11	0.17	-0.09	70.6	1.2	2.8	0.9	73.5	2.1
ENSG00000178026	C22orf36	0.58	-0.76	0.13	-0.40	33.2	57.5	1.6	15.9	34.8	73.4
ENSG00000131203	IDO1	-0.84	-0.31	-0.17	0.25	70.4	9.6	2.7	6.4	73.1	15.9
ENSG00000057657	PRDM1	-0.37	-0.64	0.77	0.09	14.0	41.2	59.1	0.9	73.0	42.1
ENSG00000233029	RP11-439A17.9	-0.81	0.11	0.28	0.61	65.3	1.2	7.7	37.4	73.0	38.6
ENSG00000127359	KIAA1147	0.20	-0.13	-0.83	-0.21	3.9	1.6	69.0	4.3	72.8	6.0
ENSG00000139318	DUSP6	-0.84	-0.02	0.12	0.31	71.3	0.0	1.4	9.6	72.8	9.7
ENSG00000114770	ABCC5	0.83	-0.26	0.21	0.16	68.4	7.0	4.3	2.7	72.6	9.7
ENSG00000196154	S100A4	0.84	-0.30	0.13	0.21	70.8	8.8	1.8	4.6	72.6	13.4
ENSG00000224940	PRRT4	0.72	-0.25	0.46	0.02	51.4	6.4	21.1	0.0	72.5	6.4
ENSG00000102796	DHRS12	-0.80	0.77	0.29	0.22	64.0	59.8	8.4	4.9	72.4	64.7
ENSG00000251442	RP11-792D21.2	-0.51	0.84	-0.25	0.11	26.3	71.1	6.2	1.3	32.5	72.3
ENSG00000111300	NAA25	-0.85	-0.34	0.07	0.35	71.7	11.6	0.5	12.3	72.2	23.9
ENSG00000072134	EPN2	0.84	-0.64	-0.09	0.18	71.2	40.3	0.9	3.4	72.1	43.7
ENSG00000085063	CD59	-0.84	0.57	0.08	0.04	71.4	32.1	0.6	0.1	72.0	32.3
ENSG00000176595	KBTBD11	0.85	0.19	-0.01	0.18	71.6	3.5	0.0	3.1	71.6	6.6

Gene ID	Gene Name	G1 (SV-AS03)	G1 (SV-PBS)	G2 (SV-AS03)	G2 (SV-PBS)	V1 (SV-AS03)	V1 (SV-PBS)	V2 (SV-AS03)	V2 (SV-PBS)	V1+V2 (SV-AS03)	V1+V2 (SV-PBS)
ENSG00000185950	IRS2	0.83	0.03	0.16	-0.36	69.1	0.1	2.5	13.0	71.6	13.1
ENSG00000213373	AC100793.2	0.84	-0.12	-0.05	0.17	71.3	1.5	0.3	2.9	71.6	4.4
ENSG00000173239	LIPM	-0.84	-0.27	0.07	0.07	71.0	7.3	0.4	0.5	71.4	7.8
ENSG00000241666	RP3-455J7.4	-0.22	-0.84	-0.29	0.11	4.9	70.2	8.1	1.2	13.0	71.4
ENSG00000118785	SPP1	0.79	0.37	0.29	0.30	62.6	13.3	8.7	8.9	71.2	22.2
ENSG00000070190	DAPP1	-0.84	-0.24	0.09	-0.03	70.5	5.9	0.7	0.1	71.2	6.0
ENSG00000184887	BTBD6	0.84	-0.69	0.06	0.43	70.7	47.1	0.4	18.6	71.1	65.7
ENSG00000230438	RP11-420G6.4	-0.84	0.58	-0.03	0.15	70.9	34.0	0.1	2.2	71.0	36.2
ENSG00000174004	LRRC33	0.78	-0.43	-0.33	-0.13	60.2	18.9	10.8	1.7	71.0	20.6
ENSG00000204634	TBC1D8	-0.40	0.84	0.19	0.05	15.9	70.7	3.7	0.2	19.6	71.0
ENSG00000109107	ALDOC	0.81	-0.18	0.22	-0.46	66.1	3.1	4.8	21.5	70.9	24.6
ENSG00000135093	USP30	-0.84	-0.12	0.07	-0.09	70.3	1.5	0.5	0.8	70.7	2.2
ENSG00000105369	CD79A	-0.61	-0.08	-0.58	-0.31	36.9	0.7	33.8	9.8	70.6	10.5
ENSG00000100324	TAB1	0.48	-0.65	-0.00	-0.53	22.8	42.2	0.0	28.3	22.8	70.5
ENSG00000139572	GPR84	-0.54	-0.52	-0.00	-0.66	29.3	27.1	0.0	43.4	29.3	70.5
ENSG00000146859	TMEM140	-0.78	-0.28	0.30	0.27	61.4	7.7	9.1	7.3	70.5	15.0
ENSG00000119686	AC007182.1	-0.84	0.42	0.06	0.50	70.0	17.5	0.4	24.5	70.4	42.0
ENSG00000088827	SIGLEC1	-0.82	-0.10	0.19	0.07	66.6	1.0	3.7	0.5	70.2	1.4
ENSG00000130881	LRP3	0.79	-0.11	0.28	0.12	62.1	1.2	8.1	1.5	70.2	2.6
ENSG00000015532	XYLT2	0.76	-0.29	0.35	-0.39	57.9	8.3	12.2	15.3	70.1	23.6
ENSG00000251002	AE000661.37	-0.83	-0.58	0.10	0.50	69.1	33.4	0.9	25.0	70.0	58.3
ENSG00000068971	PPP2R5B	-0.72	-0.70	0.42	0.30	52.1	48.6	17.9	9.1	70.0	57.8
ENSG00000073737	DHRS9	-0.84	-0.42	0.03	0.28	69.9	17.7	0.1	7.9	70.0	25.6
ENSG00000215068	AC025171.1	-0.80	0.23	0.26	-0.18	63.4	5.5	6.5	3.2	70.0	8.7
ENSG00000088992	TESC	0.83	0.32	0.11	0.12	68.8	10.5	1.1	1.4	69.9	11.9
ENSG00000091127	PUS7	0.56	-0.07	-0.62	-0.80	31.1	0.5	38.8	63.3	69.9	63.7
ENSG00000236345	RP11-59D5__B.2	0.81	-0.29	0.19	0.27	66.1	8.3	3.5	7.2	69.6	15.5
ENSG00000133624	ZNF767	0.79	-0.02	-0.26	-0.05	62.8	0.0	6.8	0.2	69.6	0.2
ENSG00000108375	RNF43	-0.82	0.01	0.17	0.17	66.7	0.0	2.9	2.9	69.6	2.9
ENSG00000134470	IL15RA	-0.82	0.15	0.14	-0.24	67.5	2.3	1.9	5.7	69.4	8.0
ENSG00000102755	FLT1	0.83	-0.12	-0.01	0.22	69.4	1.3	0.0	4.8	69.4	6.2
ENSG00000169429	IL8	0.83	-0.49	0.05	0.35	69.1	23.9	0.3	11.9	69.4	35.9
ENSG00000182810	DDX28	0.76	-0.29	-0.34	0.15	57.8	8.6	11.4	2.2	69.2	10.8
ENSG00000164674	SYTL3	0.81	0.08	0.19	-0.18	65.4	0.6	3.6	3.1	69.0	3.7
ENSG00000100403	ZC3H7B	-0.82	-0.16	0.12	-0.49	67.4	2.5	1.6	23.5	68.9	26.1
ENSG00000069849	ATP1B3	-0.82	0.14	0.11	-0.19	67.5	2.1	1.3	3.5	68.8	5.6
ENSG00000198019	FCGR1B	-0.80	0.42	0.22	0.35	63.8	17.5	4.9	11.9	68.7	29.4
ENSG00000121933	ADORA3	0.81	-0.07	0.17	-0.48	65.9	0.4	2.8	23.0	68.6	23.4
ENSG00000096996	IL12RB1	-0.83	-0.69	-0.01	-0.10	68.6	48.2	0.0	1.0	68.6	49.2
ENSG00000183134	GPR44	0.00	-0.77	-0.09	-0.30	0.0	59.2	0.9	9.2	0.9	68.4
ENSG00000175730	BAK1P1	-0.69	-0.10	0.45	0.34	47.8	1.0	20.6	11.8	68.4	12.8
ENSG00000256812	CAPNS2	-0.80	-0.04	0.19	-0.06	64.7	0.1	3.7	0.3	68.4	0.5
ENSG00000156127	BATF	-0.45	0.18	0.70	0.07	20.0	3.3	48.4	0.5	68.3	3.8
ENSG00000021762	OSBPL5	0.82	-0.54	0.05	-0.06	68.0	29.6	0.3	0.3	68.3	29.9
ENSG00000135315	KIAA1009	-0.81	-0.27	0.14	0.22	66.1	7.1	1.9	4.7	68.1	11.8
ENSG00000162946	DISC1	-0.71	0.82	0.04	0.09	50.7	67.0	0.1	0.9	50.8	67.9
ENSG00000134256	CD101	0.80	-0.68	-0.18	-0.12	64.6	45.9	3.3	1.5	67.9	47.5
ENSG00000213465	ARL2	-0.66	0.38	0.49	0.10	43.9	14.2	23.8	1.1	67.7	15.3
ENSG00000102524	TNFSF13B	-0.81	0.27	0.15	0.03	65.3	7.2	2.2	0.1	67.5	7.4
ENSG00000149782	PLCB3	0.70	-0.04	-0.43	-0.01	49.1	0.2	18.2	0.0	67.4	0.2
ENSG00000115419	GLS	-0.78	-0.34	0.23	0.31	61.4	11.3	5.4	9.4	66.7	20.6

Gene ID	Gene Name	G1 (SV-AS03)	G1 (SV-PBS)	G2 (SV-AS03)	G2 (SV-PBS)	V1 (SV-AS03)	V1 (SV-PBS)	V2 (SV-AS03)	V2 (SV-PBS)	V1+V2 (SV-AS03)	V1+V2 (SV-PBS)
ENSG00000136826	KLF4	-0.50	0.17	0.64	0.32	25.2	2.9	41.4	10.1	66.6	13.0
ENSG00000142089	IFITM3	-0.81	0.42	-0.02	-0.22	66.4	17.8	0.1	4.7	66.5	22.5
ENSG00000165806	CASP7	-0.81	-0.47	0.05	-0.01	66.0	22.0	0.3	0.0	66.3	22.1
ENSG00000226491	AC098824.5	0.37	0.75	-0.32	0.33	13.7	55.5	10.2	10.8	23.9	66.3
ENSG00000117984	CTSD	0.81	-0.20	0.11	-0.14	64.9	3.9	1.2	1.9	66.1	5.8
ENSG00000026751	SLAMF7	-0.81	-0.05	0.07	-0.15	65.7	0.2	0.4	2.3	66.1	2.5
ENSG00000178585	CTNNBIP1	0.80	-0.54	0.12	0.13	64.6	29.4	1.5	1.7	66.1	31.1
ENSG00000188042	ARL4C	-0.64	-0.79	-0.43	0.18	40.8	62.9	18.1	3.1	58.9	66.0
ENSG00000202533	Y_RNA_202533	-0.80	0.04	-0.12	0.73	64.6	0.2	1.4	53.2	65.9	53.4
ENSG00000122783	C7orf49	-0.79	-0.22	0.19	0.26	62.1	5.0	3.8	6.9	65.9	11.9
ENSG00000255150	EID3	0.37	-0.49	-0.36	0.65	13.4	24.1	13.1	41.6	26.4	65.8
ENSG00000170558	CDH2	0.74	0.27	0.34	-0.11	54.1	7.5	11.3	1.2	65.4	8.8
ENSG00000174697	LEP	0.53	0.42	0.61	0.21	28.4	17.6	37.0	4.5	65.3	22.0
ENSG00000234290	AC116366.6	-0.80	0.16	0.08	0.32	64.6	2.6	0.6	10.0	65.2	12.6
ENSG00000178719	GRINA	-0.70	0.57	0.41	0.13	48.3	32.4	16.8	1.7	65.1	34.1
ENSG00000133313	CNDP2	-0.75	-0.51	0.28	-0.32	56.9	26.1	7.9	10.1	64.9	36.2
ENSG00000112053	SLC26A8	-0.49	0.79	-0.05	0.16	24.2	62.3	0.3	2.5	24.5	64.8
ENSG00000189068	VSTM1	0.69	-0.75	-0.42	-0.13	47.4	56.2	17.3	1.7	64.8	57.9
ENSG00000166145	SPINT1	0.76	-0.43	-0.28	-0.24	57.1	18.5	7.6	5.8	64.7	24.3
ENSG00000103154	NECAB2	0.80	0.02	0.05	-0.02	64.4	0.0	0.2	0.0	64.7	0.1
ENSG00000188820	FAM26F	-0.80	-0.74	-0.01	-0.00	64.6	54.0	0.0	0.0	64.7	54.0
ENSG00000107020	C9orf46	-0.78	0.22	0.18	0.42	61.3	4.7	3.2	17.9	64.5	22.6
ENSG00000213366	GSTM2	0.04	-0.78	-0.06	-0.20	0.2	60.6	0.3	3.8	0.5	64.4
ENSG00000197646	PDCD1LG2	-0.70	-0.30	-0.38	0.10	49.6	9.1	14.6	1.0	64.2	10.1
ENSG00000178607	ERN1	0.79	0.57	0.14	-0.02	62.2	32.0	2.1	0.0	64.2	32.1
ENSG00000124215	CDH26	0.80	0.65	-0.06	-0.21	63.8	41.6	0.3	4.4	64.1	46.0
ENSG00000113407	TARS	-0.74	0.21	0.30	0.26	55.2	4.4	8.9	6.7	64.1	11.1
ENSG00000123838	C4BPA	-0.80	0.29	0.09	0.12	63.3	8.7	0.8	1.4	64.0	10.1
ENSG00000143434	SEMA6C	0.79	-0.33	0.10	0.39	63.0	10.6	1.0	15.1	64.0	25.7
ENSG00000103056	SMPD3	0.13	-0.80	-0.64	-0.01	1.8	63.7	40.8	0.0	42.6	63.7
ENSG00000113552	GNPDA1	0.79	-0.49	-0.06	-0.29	63.2	23.9	0.4	8.4	63.5	32.2
ENSG00000123992	DNPEP	-0.79	0.21	-0.06	-0.45	63.1	4.6	0.4	20.1	63.5	24.7
ENSG00000141858	SAMD1	0.71	-0.71	-0.37	-0.22	49.9	50.8	13.4	5.1	63.3	55.8
ENSG00000181826	RELL1	0.75	0.25	0.27	0.17	56.0	6.4	7.3	2.7	63.2	9.2
ENSG00000163568	AIM2	-0.79	0.26	-0.09	0.34	62.3	7.0	0.9	11.8	63.1	18.8
ENSG00000100336	APOL4	-0.79	-0.42	0.05	-0.18	62.9	18.0	0.2	3.3	63.1	21.3
ENSG00000163565	IFI16	-0.71	0.15	0.34	0.31	51.0	2.4	11.9	9.9	62.9	12.3
ENSG00000143537	ADAM15	0.78	-0.08	0.13	-0.10	61.2	0.7	1.6	1.0	62.8	1.7
ENSG00000145391	SETD7	-0.52	0.13	-0.46	0.78	26.9	1.6	20.8	60.9	47.6	62.5
ENSG00000124380	SNRNP27	-0.57	-0.09	0.55	0.03	32.0	0.8	30.4	0.1	62.3	0.9
ENSG00000121236	TRIM6	-0.78	0.16	0.11	-0.05	60.9	2.7	1.2	0.3	62.1	3.0
ENSG00000130202	PVRL2	-0.64	0.23	0.46	0.21	40.7	5.4	21.4	4.3	62.1	9.7
ENSG00000141013	GAS8	-0.73	-0.20	0.29	-0.11	53.7	4.1	8.4	1.2	62.1	5.3
ENSG00000059588	TARBP1	-0.64	-0.16	-0.45	0.45	41.4	2.7	20.7	20.4	62.1	23.1
ENSG00000115761	NOL10	-0.54	-0.14	0.57	0.62	29.0	1.9	33.0	37.9	62.0	39.8
ENSG00000172985	SH3RF3	0.67	-0.51	0.41	-0.21	45.1	25.9	16.7	4.2	61.8	30.1
ENSG00000155366	RHOC	-0.77	0.26	-0.14	0.41	59.8	6.9	1.9	17.1	61.7	24.0
ENSG00000181908	AP003774.4	-0.77	-0.28	-0.16	0.34	58.8	7.8	2.6	11.5	61.4	19.3
ENSG00000139618	BRCA2	-0.75	-0.46	0.24	0.11	55.6	20.9	5.6	1.3	61.2	22.1
ENSG00000143847	PPFIA4	0.39	-0.32	-0.68	0.09	15.2	10.5	46.0	0.8	61.2	11.2
ENSG00000034239	EFCAB1	0.71	-0.16	-0.32	-0.21	50.8	2.5	10.0	4.2	60.8	6.7

Gene ID	Gene Name	G1 (SV-AS03)	G1 (SV-PBS)	G2 (SV-AS03)	G2 (SV-PBS)	V1 (SV-AS03)	V1 (SV-PBS)	V2 (SV-AS03)	V2 (SV-PBS)	V1+V2 (SV-AS03)	V1+V2 (SV-PBS)
ENSG00000177542	SLC25A22	-0.78	-0.09	-0.03	0.37	60.7	0.8	0.1	13.4	60.8	14.2
ENSG00000243650	AC010149.1	-0.78	0.51	-0.04	0.34	60.6	26.2	0.2	11.4	60.8	37.6
ENSG00000162066	AMDHD2	0.78	0.00	0.03	-0.23	60.7	0.0	0.1	5.3	60.8	5.3
ENSG00000155393	HEATR3	0.67	-0.20	-0.40	0.15	45.0	3.9	15.8	2.3	60.8	6.1
ENSG00000213928	IRF9	-0.76	0.07	0.17	0.23	57.9	0.5	2.8	5.4	60.7	5.9
ENSG00000130487	KLHDC7B	-0.58	0.72	0.31	0.29	33.5	51.8	9.4	8.6	42.9	60.4
ENSG00000089486	C16orf5	0.77	-0.11	-0.09	-0.48	59.5	1.2	0.8	22.8	60.3	24.0
ENSG00000161277	THAP8	0.76	-0.33	-0.14	0.09	58.4	10.8	1.9	0.8	60.3	11.6
ENSG00000162813	BPNT1	-0.72	-0.20	0.28	-0.13	52.5	4.0	7.7	1.6	60.2	5.6
ENSG00000179832	HEATR7A	0.69	-0.26	0.36	0.04	47.1	6.8	13.0	0.2	60.0	6.9
ENSG00000161981	SNRNP25	0.76	-0.63	-0.12	0.08	58.4	39.8	1.6	0.7	60.0	40.5
ENSG00000138772	ANXA3	-0.18	0.74	0.10	0.21	3.3	55.0	1.1	4.6	4.4	59.6
ENSG00000162073	PAQR4	0.77	-0.28	-0.00	-0.20	59.5	8.0	0.0	4.1	59.5	12.1
ENSG00000141837	CACNA1A	-0.60	-0.20	0.23	0.74	36.3	3.9	5.4	55.5	41.7	59.4
ENSG00000140577	CRTC3	0.77	0.20	0.08	-0.24	58.7	4.1	0.7	5.8	59.3	9.9
ENSG00000102981	PARD6A	0.77	-0.35	0.08	0.02	58.7	12.2	0.6	0.0	59.3	12.3
ENSG00000205927	OLIG2	0.74	-0.27	0.22	-0.01	54.6	7.4	4.7	0.0	59.3	7.4
ENSG00000105366	SIGLEC8	-0.17	-0.63	-0.34	-0.44	2.9	40.2	11.3	19.0	14.2	59.2
ENSG00000143771	CNIH4	-0.40	0.73	0.42	0.25	16.3	52.8	17.9	6.3	34.2	59.1
ENSG00000188483	IER5L	0.77	0.57	0.03	-0.10	59.0	32.6	0.1	0.9	59.0	33.6
ENSG00000119403	PHF19	0.73	0.13	-0.24	-0.52	53.1	1.8	5.8	27.0	58.9	28.7
ENSG00000163517	HDAC11	0.76	0.26	0.05	-0.25	58.4	6.5	0.3	6.5	58.6	13.0
ENSG00000250608	RP11-933H2.4	-0.65	0.35	0.41	0.03	41.8	12.1	16.6	0.1	58.4	12.2
ENSG00000176834	VSIG10	-0.76	-0.10	0.04	-0.12	58.1	1.0	0.1	1.5	58.2	2.6
ENSG00000237059	AC083863.7	-0.76	0.31	0.04	-0.27	58.0	9.4	0.1	7.5	58.1	16.9
ENSG00000226067	RP11-403I13.7	-0.58	-0.66	-0.38	0.39	34.2	43.2	14.2	14.9	48.3	58.1
ENSG00000165140	FBP1	0.59	-0.75	0.02	-0.10	34.9	56.8	0.0	1.1	35.0	57.9
ENSG00000127838	PNKD	0.73	-0.32	0.21	0.35	53.5	10.5	4.4	12.3	57.9	22.9
ENSG00000231711	RP11-398F12.1	0.76	0.57	-0.07	0.46	57.4	32.9	0.5	21.6	57.9	54.5
ENSG00000065308	TRAM2	0.69	0.20	-0.33	-0.31	47.2	4.1	10.6	9.4	57.8	13.5
ENSG00000222179	7SK_222179	-0.72	-0.56	0.26	-0.21	51.2	31.3	6.5	4.4	57.7	35.7
ENSG00000110852	CLEC2B	-0.74	-0.28	0.16	0.41	55.2	8.1	2.5	17.2	57.7	25.3
ENSG00000167264	DUS2L	0.71	-0.60	-0.27	-0.22	50.5	36.4	7.2	4.7	57.7	41.1
ENSG00000130222	GADD45G	-0.64	0.41	0.41	0.04	40.7	17.2	16.8	0.2	57.5	17.4
ENSG00000160716	CHRNB2	-0.75	-0.03	0.08	-0.37	56.6	0.1	0.7	13.4	57.3	13.5
ENSG00000070729	CNGB1	-0.53	0.43	-0.17	-0.62	27.6	18.4	3.0	38.9	30.6	57.2
ENSG00000171045	TSNARE1	-0.20	-0.75	-0.15	-0.07	3.9	56.6	2.2	0.5	6.1	57.1
ENSG00000137752	CASP1	-0.73	0.41	0.18	0.63	53.6	16.4	3.3	40.2	56.9	56.6
ENSG00000059122	FLYWCH1	0.25	-0.59	-0.36	-0.47	6.0	35.1	13.0	21.8	19.0	56.9
ENSG00000136999	NOV	0.74	-0.72	0.02	-0.21	54.7	52.4	0.0	4.5	54.8	56.9
ENSG00000179918	SEPHS2	-0.49	-0.53	0.57	-0.05	24.0	28.4	32.6	0.3	56.6	28.6
ENSG00000185730	ZNF696	0.03	0.08	-0.75	-0.15	0.1	0.6	56.4	2.2	56.5	2.9
ENSG00000164105	SAP30	0.61	-0.20	0.44	-0.24	37.5	3.9	18.9	5.8	56.4	9.8
ENSG00000184470	TXNRD2	0.73	-0.36	-0.19	-0.19	52.7	12.9	3.7	3.6	56.4	16.5
ENSG00000157551	KCNJ15	-0.71	0.45	0.24	0.56	50.8	20.4	5.5	31.7	56.3	52.1
ENSG00000104626	ERI1	-0.69	0.46	0.30	0.56	47.1	21.4	9.2	31.6	56.3	53.0
ENSG00000183696	UPP1	-0.31	0.72	0.27	0.20	9.3	52.1	7.5	4.0	16.9	56.1
ENSG00000007516	BAIAP3	0.68	0.03	0.32	-0.09	46.0	0.1	10.0	0.7	56.0	0.9
ENSG00000233214	AC002511.2	-0.73	0.27	-0.14	0.31	53.8	7.3	2.0	9.5	55.9	16.8
ENSG00000106392	C1GALT1	-0.75	-0.33	-0.03	0.27	55.7	11.1	0.1	7.2	55.8	18.3
ENSG00000167515	TRAPPC2L	0.63	-0.12	0.40	-0.21	39.8	1.4	16.0	4.6	55.8	6.0

Gene ID	Gene Name	G1 (SV-AS03)	G1 (SV-PBS)	G2 (SV-AS03)	G2 (SV-PBS)	V1 (SV-AS03)	V1 (SV-PBS)	V2 (SV-AS03)	V2 (SV-PBS)	V1+V2 (SV-AS03)	V1+V2 (SV-PBS)
ENSG00000167074	TEF	0.66	-0.74	-0.27	-0.09	43.8	54.8	7.5	0.8	51.2	55.7
ENSG00000151136	BTBD11	0.59	-0.72	0.08	-0.20	34.5	51.5	0.6	4.1	35.1	55.6
ENSG00000256579	AC135776.2	-0.74	0.02	0.04	0.10	55.3	0.0	0.2	1.0	55.4	1.1
ENSG00000213492	AC092661.2	-0.74	-0.31	0.05	0.43	55.1	9.9	0.3	18.6	55.4	28.5
ENSG00000153406	NMRAL1	0.67	-0.38	0.32	-0.56	45.2	14.6	10.1	31.5	55.3	46.0
ENSG00000138166	DUSP5	-0.65	0.24	0.37	0.03	41.7	5.7	13.6	0.1	55.2	5.7
ENSG00000204397	CARD16	-0.71	0.50	-0.22	0.29	50.2	25.3	4.9	8.5	55.1	33.8
ENSG00000227032	RP11-34E5.4	-0.74	-0.30	0.05	-0.15	54.8	9.0	0.2	2.1	55.0	11.1
ENSG00000136231	IGF2BP3	-0.66	-0.63	0.33	-0.06	43.9	39.5	10.6	0.4	54.6	39.9
ENSG00000103355	PRSS33	0.01	-0.69	-0.49	-0.27	0.0	47.2	23.5	7.3	23.5	54.5
ENSG00000187840	EIF4EBP1	0.73	-0.43	-0.10	-0.55	53.4	18.4	1.1	30.0	54.5	48.4
ENSG00000105516	DBP	0.38	-0.23	-0.63	-0.37	14.7	5.3	39.5	13.9	54.2	19.2
ENSG00000205786	AC002511.1	-0.50	0.61	-0.03	0.42	24.6	36.8	0.1	17.3	24.7	54.1
ENSG00000134242	PTPN22	0.68	-0.64	-0.27	0.00	46.6	40.9	7.5	0.0	54.1	40.9
ENSG00000095397	DFNB31	-0.63	0.03	-0.38	0.43	39.2	0.1	14.8	18.9	54.0	19.0
ENSG00000188486	H2AFX	0.72	-0.06	-0.16	0.06	51.5	0.4	2.4	0.4	54.0	0.8
ENSG00000111696	NT5DC3	0.73	-0.00	-0.05	-0.12	52.7	0.0	0.3	1.5	52.9	1.5
ENSG00000186918	ZNF395	0.53	-0.47	-0.50	-0.41	28.0	22.0	24.9	16.6	52.9	38.6
ENSG00000082269	FAM135A	-0.70	0.02	0.18	0.11	49.3	0.0	3.4	1.3	52.7	1.3
ENSG00000184702	SEPT5	0.72	-0.26	0.09	-0.28	51.3	6.6	0.8	7.7	52.1	14.3
ENSG00000076555	ACACB	0.58	-0.57	-0.06	-0.44	33.5	32.4	0.4	19.7	33.9	52.1
ENSG00000103876	FAH	0.50	-0.67	0.45	-0.26	25.1	45.4	20.1	6.7	45.1	52.1
ENSG00000127311	HELB	-0.58	0.15	0.43	0.36	33.7	2.2	18.2	13.1	51.9	15.3
ENSG00000154188	ANGPT1	0.63	-0.70	0.14	-0.18	39.2	48.5	1.9	3.4	41.1	51.9
ENSG00000137757	CASP5	-0.57	0.69	0.22	0.20	32.5	47.9	5.0	4.0	37.4	51.9
ENSG00000134072	CAMK1	0.59	-0.37	-0.42	-0.06	34.4	13.9	17.4	0.4	51.7	14.2
ENSG00000168874	ATOH8	0.52	0.24	-0.02	0.68	27.2	5.7	0.0	45.9	27.2	51.6
ENSG00000205583	STAG3L1	0.66	-0.07	-0.29	-0.34	43.0	0.5	8.5	11.6	51.5	12.2
ENSG00000229894	GK3P	-0.58	-0.14	0.43	0.28	33.3	2.0	18.1	8.0	51.4	9.9
ENSG00000110799	VWF	0.71	-0.16	0.11	-0.50	50.2	2.5	1.1	25.4	51.3	27.9
ENSG00000054179	ENTPD2	0.71	-0.02	0.08	0.00	50.8	0.0	0.6	0.0	51.3	0.0
ENSG00000165389	C14orf147	-0.70	-0.23	0.15	-0.08	48.7	5.5	2.4	0.6	51.1	6.1
ENSG00000134802	SLC43A3	-0.65	-0.23	0.28	-0.15	42.8	5.1	8.0	2.4	50.8	7.5
ENSG00000111731	KIAA0528	0.44	-0.70	-0.06	0.07	19.7	49.7	0.3	0.5	20.1	50.1
ENSG00000132182	NUP210	-0.20	-0.50	-0.68	-0.45	4.0	24.5	46.0	19.9	50.0	44.4
ENSG00000188282	RUFY4	-0.61	0.66	-0.04	0.24	36.9	43.9	0.1	5.7	37.1	49.6
ENSG00000105205	CLC	0.22	-0.70	-0.27	-0.09	4.8	48.6	7.1	0.8	12.0	49.4
ENSG00000159618	GPR114	-0.30	-0.42	-0.64	-0.08	8.7	18.0	40.7	0.6	49.4	18.6
ENSG00000182541	LIMK2	-0.56	0.46	0.42	0.24	31.4	20.9	17.8	5.9	49.3	26.8
ENSG00000006015	C19orf60	0.67	-0.24	0.20	0.03	45.2	5.8	3.9	0.1	49.1	5.9
ENSG00000230795	HLA-K	-0.45	-0.07	-0.54	0.44	20.1	0.5	28.8	19.7	48.9	20.2
ENSG00000188313	PLSCR1	-0.70	0.60	0.03	0.32	48.8	35.9	0.1	10.1	48.9	46.0
ENSG00000143842	SOX13	0.27	-0.28	0.64	-0.03	7.2	7.6	41.3	0.1	48.5	7.7
ENSG00000106537	TSPAN13	0.45	-0.69	-0.46	0.03	20.5	48.2	20.9	0.1	41.4	48.2
ENSG00000136002	ARHGEF4	0.64	-0.16	0.27	0.22	40.4	2.6	7.2	4.7	47.6	7.3
ENSG00000257017	HP	0.43	0.68	-0.21	0.09	18.7	46.5	4.3	0.8	22.9	47.3
ENSG00000134900	TPP2	0.68	-0.49	0.13	-0.13	45.6	23.5	1.7	1.6	47.3	25.1
ENSG00000164096	C4orf3	-0.65	0.18	0.22	0.14	42.5	3.1	4.7	1.8	47.2	5.0
ENSG00000105948	TTC26	-0.49	0.55	0.48	0.14	24.3	29.8	22.9	2.0	47.2	31.9
ENSG00000166881	TMEM194A	0.53	-0.40	0.43	-0.07	28.4	15.9	18.8	0.5	47.1	16.4
ENSG00000147443	DOK2	0.19	-0.65	-0.45	-0.21	3.6	42.3	20.1	4.5	23.7	46.7

Gene ID	Gene Name	G1 (SV-AS03)	G1 (SV-PBS)	G2 (SV-AS03)	G2 (SV-PBS)	V1 (SV-AS03)	V1 (SV-PBS)	V2 (SV-AS03)	V2 (SV-PBS)	V1+V2 (SV-AS03)	V1+V2 (SV-PBS)
ENSG00000130695	CEP85	-0.16	-0.33	-0.66	-0.25	2.5	10.8	44.2	6.2	46.7	17.0
ENSG00000246013	NCRNA00282	-0.65	0.51	0.20	0.30	42.8	26.4	3.8	8.8	46.6	35.3
ENSG00000154274	C4orf19	0.67	0.21	0.15	0.31	44.2	4.6	2.3	9.4	46.6	14.0
ENSG00000185880	TRIM69	-0.65	0.41	0.20	0.20	42.5	17.0	4.0	4.0	46.4	21.0
ENSG00000188549	C15orf52	0.59	-0.67	0.26	-0.10	35.0	44.9	6.7	1.0	41.7	45.9
ENSG00000184979	USP18	-0.68	-0.14	0.04	-0.43	45.7	2.1	0.2	18.4	45.8	20.5
ENSG00000177606	JUN	0.67	-0.60	-0.02	-0.15	45.3	36.4	0.0	2.4	45.3	38.8
ENSG00000110042	DTX4	0.49	-0.59	-0.45	-0.32	24.5	34.8	20.5	10.5	45.0	45.3
ENSG00000226942	IL9RP3	0.66	-0.30	0.14	-0.24	43.1	9.1	2.1	5.6	45.2	14.7
ENSG00000231826	AC016735.2	0.58	-0.36	0.33	0.07	34.0	12.6	11.0	0.5	45.0	13.1
ENSG00000161980	POLR3K	0.67	-0.20	-0.03	0.08	44.9	4.0	0.1	0.6	45.0	4.6
ENSG00000229515	RP11-793E15.1	-0.24	0.43	0.23	0.52	5.8	18.2	5.2	26.8	11.0	45.0
ENSG00000233461	RP11-295G20.2	-0.66	0.31	0.14	0.50	43.0	9.4	1.9	25.4	44.9	34.8
ENSG00000197586	ENTPD6	-0.66	-0.63	0.08	0.22	43.8	39.9	0.6	4.9	44.3	44.8
ENSG00000004799	PDK4	0.50	-0.45	0.18	0.50	24.9	19.9	3.1	24.9	28.0	44.8
ENSG00000223759	RP11-353N4.4	-0.64	0.21	0.18	0.47	41.4	4.3	3.2	22.2	44.6	26.5
ENSG00000169683	LRRC45	0.66	-0.48	-0.07	0.03	44.1	23.5	0.4	0.1	44.5	23.6
ENSG00000114631	PODXL2	0.60	0.43	-0.29	-0.09	35.7	18.4	8.3	0.8	44.0	19.3
ENSG00000124772	CPNE5	-0.28	0.58	-0.07	-0.32	7.8	34.0	0.5	9.9	8.3	43.9
ENSG00000247645	AC034110.2	0.64	-0.31	-0.17	0.10	40.7	9.7	2.9	1.0	43.7	10.6
ENSG00000167106	FAM102A	-0.03	-0.57	-0.62	-0.33	0.1	32.2	37.9	11.1	37.9	43.3
ENSG00000173762	CD7	-0.53	-0.05	0.39	0.01	28.4	0.2	14.9	0.0	43.3	0.2
ENSG00000164430	MB21D1	-0.44	-0.31	0.49	-0.47	19.1	9.9	24.2	22.3	43.3	32.1
ENSG00000158445	KCNB1	0.65	0.36	-0.04	-0.34	42.7	13.2	0.2	11.6	42.9	24.8
ENSG00000117318	ID3	-0.60	0.16	0.26	-0.07	35.7	2.5	7.0	0.5	42.6	3.1
ENSG00000216588	IGSF23	0.64	-0.25	-0.12	-0.00	40.8	6.5	1.4	0.0	42.2	6.5
ENSG00000112759	SLC29A1	0.47	-0.56	-0.45	0.03	22.0	31.0	20.1	0.1	42.1	31.1
ENSG00000181350	C17orf76	0.61	-0.18	-0.20	-0.44	37.4	3.4	4.2	19.0	41.6	22.4
ENSG00000185420	SMYD3	0.51	-0.49	-0.12	-0.41	26.2	24.5	1.4	17.1	27.6	41.6
ENSG00000198825	INPP5F	0.14	0.42	-0.24	0.49	2.1	17.6	6.0	23.8	8.1	41.4
ENSG00000187554	TLR5	-0.22	0.62	0.01	0.16	4.8	38.5	0.0	2.7	4.8	41.1
ENSG00000068489	PRR11	-0.56	-0.52	-0.30	-0.25	31.9	27.3	9.2	6.2	41.1	33.5
ENSG00000161905	ALOX15	0.30	-0.59	-0.38	-0.25	9.3	34.7	14.7	6.1	24.0	40.9
ENSG00000106004	HOXA5	0.64	-0.24	0.01	0.04	40.5	5.8	0.0	0.2	40.5	6.0
ENSG00000183762	KREMEN1	-0.62	0.49	0.12	0.11	38.8	23.5	1.5	1.2	40.4	24.7
ENSG00000103202	NME4	0.57	0.38	0.28	-0.28	32.4	14.6	7.9	7.8	40.3	22.4
ENSG00000162636	FAM102B	0.58	-0.35	-0.24	0.07	34.1	12.1	5.9	0.4	40.1	12.5
ENSG00000133317	LGALS12	0.42	-0.55	-0.10	0.31	18.0	30.5	1.0	9.4	19.0	39.9
ENSG00000137842	TMEM62	-0.63	-0.17	0.07	-0.44	39.5	3.0	0.4	19.5	39.9	22.6
ENSG00000204371	EHMT2	0.63	-0.09	0.03	-0.30	39.6	0.8	0.1	8.9	39.7	9.7
ENSG00000104375	STK3	-0.55	0.61	0.23	0.11	30.7	37.2	5.3	1.2	35.9	38.4
ENSG00000225572	AC003077.1	-0.61	0.26	0.07	-0.09	37.8	6.7	0.5	0.8	38.3	7.5
ENSG00000179242	CDH4	0.47	-0.06	0.39	-0.40	22.1	0.4	15.1	16.1	37.2	16.5
ENSG00000010278	CD9	0.61	-0.49	-0.01	-0.25	37.0	24.3	0.0	6.2	37.0	30.5
ENSG00000214425	AC091132.2	0.32	-0.36	-0.05	-0.49	10.2	12.7	0.3	24.2	10.4	36.9
ENSG00000144647	C3orf39	0.60	-0.29	-0.05	-0.21	36.4	8.6	0.3	4.4	36.6	13.0
ENSG00000204936	CD177	0.16	0.51	-0.16	0.33	2.5	25.7	2.5	11.0	5.0	36.6
ENSG00000163958	ZDHHC19	-0.47	0.45	0.38	0.32	22.4	20.4	14.1	9.9	36.5	30.3
ENSG00000237135	RP11-330O11.2	-0.42	0.29	-0.23	0.53	17.4	8.6	5.3	27.7	22.7	36.3
ENSG00000162894	FAIM3	0.26	-0.60	-0.29	0.01	6.7	36.2	8.4	0.0	15.1	36.2
ENSG00000223779	RP11-403I13.4	-0.58	-0.07	-0.16	0.25	33.4	0.5	2.4	6.1	35.9	6.6

Gene ID	Gene Name	G1 (SV-AS03)	G1 (SV-PBS)	G2 (SV-AS03)	G2 (SV-PBS)	V1 (SV-AS03)	V1 (SV-PBS)	V2 (SV-AS03)	V2 (SV-PBS)	V1+V2 (SV-AS03)	V1+V2 (SV-PBS)
ENSG00000226012	AP001434.2	-0.37	0.18	-0.10	0.57	14.0	3.1	1.0	32.8	15.0	35.9
ENSG00000134352	IL6ST	-0.51	0.47	0.31	-0.01	26.1	22.4	9.7	0.0	35.8	22.5
ENSG00000165644	COMTD1	0.16	-0.18	-0.07	-0.57	2.5	3.1	0.5	32.6	3.0	35.7
ENSG00000196954	CASP4	-0.58	0.38	0.16	0.32	33.2	14.5	2.4	10.2	35.6	24.7
ENSG00000108861	DUSP3	-0.58	0.36	0.15	0.38	33.2	13.1	2.2	14.6	35.5	27.7
ENSG00000023909	GCLM	-0.17	-0.33	0.57	0.27	2.8	10.8	32.5	7.1	35.4	17.9
ENSG00000105270	CLIP3	0.57	-0.41	0.15	-0.06	32.9	17.1	2.3	0.4	35.2	17.5
ENSG00000248936	RP11-36B15.1	0.47	0.31	0.02	0.48	22.3	9.7	0.1	23.4	22.4	33.1
ENSG00000141577	AZI1	0.30	0.14	0.48	-0.38	8.9	2.0	23.2	14.6	32.1	16.5
ENSG00000242313	AC002073.1	0.40	0.02	-0.40	-0.19	16.2	0.0	15.8	3.7	32.0	3.7
ENSG00000169413	RNASE6	0.32	-0.56	0.34	-0.01	9.9	31.1	11.8	0.0	21.7	31.1
ENSG00000229023	AC067945.3	-0.04	0.03	0.14	0.55	0.1	0.1	2.0	30.6	2.2	30.6
ENSG00000244490	RWDD4P1	0.35	-0.42	-0.02	0.36	12.0	17.7	0.0	12.8	12.0	30.5
ENSG00000146215	CRIP3	0.10	0.52	-0.47	-0.18	0.9	27.1	22.3	3.2	23.3	30.3
ENSG00000008710	PKD1	-0.10	0.03	-0.50	-0.55	1.0	0.1	25.0	30.1	26.0	30.2
ENSG00000230124	RP5-1180C10.2	0.53	0.18	0.11	0.41	28.3	3.3	1.2	16.8	29.4	20.1
ENSG00000214106	AC093726.6	-0.49	0.31	-0.22	-0.22	24.4	9.3	4.9	4.8	29.3	14.1
ENSG00000109685	WHSC1	0.38	-0.32	-0.26	-0.44	14.6	10.0	7.0	19.0	21.6	29.0
ENSG00000224073	RP1-56L9.7	-0.49	0.31	-0.03	0.44	24.2	9.4	0.1	19.6	24.3	29.0
ENSG00000245549	AL137798.2	-0.07	-0.07	-0.13	0.53	0.5	0.5	1.7	28.3	2.2	28.8
ENSG00000119457	SLC46A2	0.43	0.22	-0.32	0.19	18.3	4.7	10.2	3.8	28.4	8.4
ENSG00000164023	SGMS2	0.38	0.53	-0.24	-0.05	14.7	27.7	5.7	0.3	20.4	27.9
ENSG00000185087	FAM169B	-0.34	-0.11	0.40	0.09	11.7	1.2	15.9	0.9	27.7	2.1
ENSG00000226742	HSBP1L1	-0.52	0.28	0.09	-0.36	26.9	8.1	0.7	12.7	27.7	20.8
ENSG00000141854	AC022098.1	0.25	0.32	-0.32	0.42	6.5	10.3	10.5	17.3	17.0	27.6
ENSG00000116990	MYCL1	0.42	0.29	-0.32	-0.21	17.3	8.3	10.2	4.6	27.4	12.9
ENSG00000165490	C11orf82	-0.43	0.08	-0.29	0.50	18.7	0.7	8.5	25.4	27.2	26.1
ENSG00000237520	RP11-443B7.2	0.08	0.42	-0.04	0.31	0.7	17.5	0.2	9.6	0.8	27.2
ENSG00000182621	PLCB1	0.52	-0.19	-0.05	-0.03	26.7	3.8	0.2	0.1	26.9	3.9
ENSG00000185897	FFAR3	-0.42	0.47	0.25	-0.21	17.6	22.4	6.2	4.3	23.8	26.8
ENSG00000253214	RP11-1149M10.2	-0.45	0.51	0.21	-0.06	20.3	26.2	4.3	0.4	24.6	26.6
ENSG00000175040	CHST2	-0.10	0.06	-0.16	0.51	0.9	0.3	2.6	26.3	3.5	26.6
ENSG00000243477	NAT6	0.36	0.32	-0.36	0.17	12.9	10.5	13.3	2.8	26.2	13.3
ENSG00000117983	MUC5B	0.35	0.24	-0.37	0.38	12.1	5.5	13.8	14.3	25.9	19.9
ENSG00000233038	AC011899.9	0.47	-0.31	-0.19	-0.38	22.1	9.6	3.7	14.1	25.8	23.7
ENSG00000179715	FAM113B	-0.48	-0.16	0.16	-0.30	23.2	2.4	2.4	8.9	25.7	11.3
ENSG00000235944	ZNF815	0.50	-0.29	0.09	-0.37	24.9	8.2	0.8	13.8	25.7	22.0
ENSG00000223692	AP000337.3	0.39	0.04	-0.32	-0.44	15.2	0.2	10.2	19.2	25.4	19.4
ENSG00000141569	TRIM65	-0.09	0.25	-0.48	-0.43	0.8	6.1	23.1	18.9	23.9	25.0
ENSG00000198892	SHISA4	0.50	0.14	0.03	-0.11	24.8	2.1	0.1	1.2	24.9	3.3
ENSG00000124713	GNMT	0.33	-0.09	0.37	-0.17	11.0	0.9	13.8	3.0	24.8	3.9
ENSG00000125457	MIF4GD	0.33	-0.15	0.37	-0.31	11.1	2.3	13.6	9.4	24.7	11.7
ENSG00000146373	RNF217	-0.27	0.08	0.41	0.40	7.5	0.6	17.0	16.0	24.5	16.6
ENSG00000204257	HLA-DMA	-0.47	-0.28	0.12	0.06	22.5	7.7	1.5	0.3	24.0	8.0
ENSG00000255139	AP000442.1	-0.23	0.01	0.43	-0.35	5.1	0.0	18.9	12.4	24.0	12.4
ENSG00000148334	PTGES2	0.41	-0.39	-0.26	0.10	16.8	15.3	6.9	1.1	23.7	16.4
ENSG00000238249	HMGN2P17	0.30	-0.04	-0.05	0.48	8.9	0.2	0.2	23.4	9.2	23.6
ENSG00000181610	MRPS23	0.23	-0.08	-0.43	-0.04	5.4	0.6	18.1	0.2	23.5	0.8
ENSG00000179094	PER1	0.41	0.09	0.21	-0.47	17.0	0.8	4.3	21.9	21.3	22.7
ENSG00000154102	C16orf74	0.24	0.31	-0.41	-0.08	5.9	9.6	16.8	0.7	22.6	10.3
ENSG00000163357	DCST1	0.21	0.04	0.42	-0.07	4.2	0.2	18.1	0.5	22.3	0.7

Gene ID	Gene Name	G1 (SV-AS03)	G1 (SV-PBS)	G2 (SV-AS03)	G2 (SV-PBS)	V1 (SV-AS03)	V1 (SV-PBS)	V2 (SV-AS03)	V2 (SV-PBS)	V1+V2 (SV-AS03)	V1+V2 (SV-PBS)
ENSG00000163746	PLSCR2	-0.46	-0.19	0.10	0.07	20.9	3.6	0.9	0.5	21.8	4.2
ENSG00000134824	FADS2	0.46	0.16	0.05	-0.30	21.5	2.7	0.3	8.9	21.8	11.6
ENSG00000225792	AC004540.4	-0.41	-0.06	0.22	0.41	16.8	0.3	5.0	17.1	21.7	17.5
ENSG00000157404	KIT	0.35	-0.46	0.24	-0.04	11.9	21.3	5.5	0.2	17.5	21.5
ENSG00000244242	IFITM10	0.46	-0.32	0.08	0.29	20.8	10.6	0.7	8.2	21.5	18.7
ENSG00000090269	CLEC5A	-0.22	-0.11	-0.40	0.01	5.0	1.1	15.7	0.0	20.7	1.1
ENSG00000135847	ACBD6	0.24	-0.38	-0.38	0.24	5.9	14.5	14.3	5.6	20.2	20.1
ENSG00000137094	DNAJB5	0.41	0.25	-0.07	0.37	16.4	6.1	0.5	13.9	16.9	19.9
ENSG00000104804	TULP2	0.10	0.23	0.43	0.27	0.9	5.5	18.8	7.4	19.8	12.8
ENSG00000224804	AC004878.2	-0.18	-0.30	-0.31	-0.31	3.2	9.1	9.8	9.7	13.0	18.8
ENSG00000242498	C15orf38	-0.25	0.16	0.11	-0.40	6.1	2.6	1.3	15.9	7.3	18.5
ENSG00000073060	SCARB1	0.38	-0.17	0.19	0.02	14.5	3.0	3.7	0.0	18.2	3.0
ENSG00000100997	ABHD12	0.22	-0.20	0.37	-0.20	4.7	3.9	13.4	4.0	18.1	7.9
ENSG00000159713	TPPP3	0.26	-0.01	-0.15	0.42	6.8	0.0	2.3	17.7	9.0	17.7
ENSG00000168685	IL7R	-0.17	-0.42	0.21	-0.06	3.0	17.3	4.4	0.3	7.4	17.6
ENSG00000114541	FRMD4B	-0.41	-0.17	0.08	0.38	16.6	3.0	0.6	14.5	17.2	17.5
ENSG00000053371	AKR7A2	0.23	0.22	-0.34	0.29	5.3	4.9	11.4	8.3	16.7	13.2
ENSG00000116237	ICMT	0.32	-0.17	-0.02	0.37	10.2	3.0	0.0	13.5	10.2	16.5
ENSG00000116455	WDR77	-0.17	-0.35	0.25	-0.17	3.0	12.5	6.3	3.1	9.3	15.6
ENSG00000073350	LLGL2	0.01	0.16	-0.39	-0.22	0.0	2.5	15.3	5.0	15.3	7.5
ENSG00000174837	EMR1	-0.01	-0.38	0.02	0.03	0.0	14.6	0.0	0.1	0.1	14.7
ENSG00000167969	ECI1	0.30	0.24	0.03	-0.29	8.7	5.6	0.1	8.7	8.8	14.2
ENSG00000196814	FAM125B	0.35	-0.33	-0.14	0.01	12.1	10.8	1.9	0.0	14.0	10.8
ENSG00000224460	RP11-439L18.2	-0.18	0.18	-0.31	0.06	3.3	3.3	9.7	0.3	13.0	3.6
ENSG00000146540	C7orf50	0.26	-0.18	-0.25	0.04	6.7	3.1	6.1	0.2	12.8	3.2
ENSG00000125538	IL1B	-0.29	-0.13	0.20	0.13	8.2	1.8	4.1	1.8	12.3	3.6
ENSG00000121454	LHX4	0.05	-0.22	-0.15	0.27	0.3	4.8	2.1	7.4	2.4	12.3
ENSG00000171962	LRRC48	0.03	-0.04	-0.33	-0.34	0.1	0.2	11.1	11.7	11.2	11.9
ENSG00000199695	U6_199695	-0.21	0.18	0.05	-0.27	4.6	3.4	0.3	7.5	4.8	10.9
ENSG00000103254	FAM173A	-0.17	0.24	-0.26	0.03	3.0	5.7	6.8	0.1	9.7	5.8
ENSG00000179915	NRXN1	0.14	-0.10	-0.26	0.26	2.0	1.0	6.5	6.9	8.6	7.9
ENSG00000196636	ACN9	0.03	0.22	0.16	0.14	0.1	4.7	2.5	2.1	2.6	6.8
ENSG00000172728	FUT10	-0.05	0.13	0.25	-0.20	0.3	1.7	6.3	4.2	6.6	5.9
ENSG00000236829	Z97634.5	0.05	0.03	0.21	-0.04	0.2	0.1	4.2	0.2	4.5	0.3
ENSG00000038427	VCAN	-0.19	-0.03	0.05	-0.17	3.7	0.1	0.3	3.0	4.0	3.1
ENSG00000127564	PKMYT1	0.06	0.07	-0.04	-0.14	0.4	0.5	0.1	1.9	0.6	2.4
ENSG00000090924	PLEKHG2	-0.04	-0.01	0.11	0.09	0.1	0.0	1.2	0.8	1.3	0.8

Table A138: Canonical loadings and explained variance (RNA-Seq, Neutrophils, Day 1, Gene variables). Gene variables with at least 25% of explained variance for the first two canonical variates are listed. GX: canonical loadings for canonical variate X, VX: % explained variance for canonical variate X, V1+V2: total % explained variance for both canonical variates. Values for CCA results with a poor cross validation score (< 0.70) are left blank. Sorted by maximum explained variance across treatments (V1+V2).

IMO Variable	IMO Variable Name	I1 (SV-AS03)	I1 (SV-PBS)	I2 (SV-AS03)	I2 (SV-PBS)	V1 (SV-AS03)	V1 (SV-PBS)	V2 (SV-AS03)	V2 (SV-PBS)	V1+V2 (SV-AS03)	V1+V2 (SV-PBS)
IL-12p70	Interleukin-12 p70	0.01	-0.93	0.22	-0.29	0.0	86.3	4.9	8.6	5.0	95.0
IP-10	Interferon gamma-induced protein 10	-0.88	0.62	-0.21	-0.48	77.1	38.9	4.5	23.1	81.7	62.0
MIP-1α	Macrophage inflammatory protein	-0.75	0.54	-0.39	-0.65	56.5	29.2	14.9	42.4	71.5	71.6

IMO Variable	IMO Variable Name	I1 (SV-AS03)	I1 (SV-PBS)	I2 (SV-AS03)	I2 (SV-PBS)	V1 (SV-AS03)	V1 (SV-PBS)	V2 (SV-AS03)	V2 (SV-PBS)	V1+V2 (SV-AS03)	V1+V2 (SV-PBS)
IFN-γ	Interferon gamma	0.41	0.40	-0.17	-0.70	16.9	15.8	2.8	49.2	19.7	65.0
Eotaxin-1	Eotaxin-1	0.38	-0.73	0.53	-0.31	14.3	53.2	28.5	9.5	42.8	62.6
RANTES	Chemokine (C-C motif) ligand 5	-0.31	0.10	0.02	-0.74	9.3	1.0	0.1	54.8	9.4	55.8
Nt	Microneutralization inhibition	-0.03	0.49	0.14	0.44	0.1	23.6	2.0	19.8	2.1	43.4
TNF	Tumor necrosis factors	0.41	-0.09	0.33	-0.12	16.5	0.8	11.1	1.4	27.7	2.2
IL-10	Interleukin-10	-0.19	0.00	0.24	-0.41	3.8	0.0	5.7	16.6	9.5	16.6
IL-8	Interleukin-8	0.16	-0.07	0.17	-0.34	2.6	0.4	2.9	11.6	5.5	12.0
IL-1β	Interleukin-1 beta	-0.21	0.29	-0.27	-0.17	4.6	8.4	7.3	3.0	11.9	11.4
MCP-1	Monocyte chemoattractant protein-1	0.01	0.10	0.21	-0.30	0.0	1.0	4.2	9.2	4.2	10.1
IL-6	Interleukin-6	-0.19	0.19	0.21	-0.24	3.4	3.7	4.4	5.7	7.8	9.4

Table A139: Canonical loadings and explained variance (RNA-Seq, Neutrophils, Day 1, IMO variables). IMO variables with at least 25% of explained variance for the first two canonical variates are listed. GX: canonical loadings for canonical variate X, VX: % explained variance for canonical variate X, V1+V2: total % explained variance for both canonical variates. Values for CCA results with a poor cross validation score ($<$ 0.70) are left blank. Sorted by maximum explained variance across treatments (V1+V2).

Gene ID	Gene Name	G1 (SV-AS03)	G1 (SV-PBS)	G2 (SV-AS03)	G2 (SV-PBS)	V1 (SV-AS03)	V1 (SV-PBS)	V2 (SV-AS03)	V2 (SV-PBS)	V1+V2 (SV-AS03)	V1+V2 (SV-PBS)
ENSG00000177575	CD163		-0.57		0.63		32.3		39.4		71.7
ENSG00000105366	SIGLEC8		-0.84		-0.01		71.3		0.0		71.3
ENSG00000188536	HBA2		-0.37		0.68		13.9		46.6		60.5
ENSG00000112759	SLC29A1		-0.76		-0.06		57.3		0.3		57.6
ENSG00000206172	HBA1		-0.11		0.74		1.3		54.3		55.5
ENSG00000244734	HBB		-0.17		0.68		3.0		46.8		49.8
ENSG00000183134	GPR44		-0.68		0.07		46.0		0.5		46.5
ENSG00000110651	CD81		-0.57		0.21		33.0		4.5		37.5
ENSG00000211895	IGHA1		-0.36		0.44		12.7		19.7		32.4
ENSG00000103355	PRSS33		-0.57		-0.04		32.0		0.1		32.1
ENSG00000105369	CD79A		-0.28		0.48		8.0		22.6		30.6
ENSG00000038427	VCAN		-0.52		0.10		27.4		1.0		28.4
ENSG00000163534	FCRL1		-0.20		0.49		3.9		24.3		28.3
ENSG00000089169	RPH3A		-0.35		0.39		12.1		15.0		27.1
ENSG00000196576	PLXNB2		-0.41		0.30		16.5		9.1		25.6
ENSG00000107551	RASSF4		-0.36		0.35		13.2		12.4		25.6
ENSG00000136573	BLK		-0.38		0.31		14.7		9.4		24.1
ENSG00000123384	LRP1		-0.47		0.12		22.0		1.5		23.6
ENSG00000089127	OAS1		0.38		-0.26		14.6		6.5		21.1
ENSG00000172967	XKR3		0.08		0.45		0.6		20.2		20.8
ENSG00000140968	IRF8		-0.35		0.28		12.4		8.0		20.4
ENSG00000012124	CD22		-0.34		0.29		11.5		8.4		19.9
ENSG00000153064	BANK1		-0.35		0.26		12.0		6.8		18.8
ENSG00000135218	CD36		-0.41		0.13		16.5		1.6		18.1
ENSG00000223946	RP11-533O20.2		0.32		0.26		10.1		6.9		17.1
ENSG00000124942	AHNAK		-0.40		0.08		15.7		0.6		16.2
ENSG00000211898	IGHD		-0.29		0.26		8.2		6.9		15.1
ENSG00000196092	PAX5		-0.38		-0.04		14.3		0.2		14.5
ENSG00000010327	STAB1		-0.33		0.17		10.9		2.8		13.7
ENSG00000234571	RP5-998N21.4		-0.30		0.19		9.1		3.7		12.8

Gene ID	Gene Name	G1 (SV-AS03)	G1 (SV-PBS)	G2 (SV-AS03)	G2 (SV-PBS)	V1 (SV-AS03)	V1 (SV-PBS)	V2 (SV-AS03)	V2 (SV-PBS)	V1+V2 (SV-AS03)	V1+V2 (SV-PBS)
ENSG00000247982	AC016525.1		-0.33		0.13		11.0		1.7		12.7
ENSG00000204345	CD300LD		-0.11		0.31		1.1		9.8		10.9
ENSG00000186407	CD300E		-0.22		0.24		4.9		5.9		10.8
ENSG00000004799	PDK4		0.32		0.06		10.4		0.4		10.7
ENSG00000156738	MS4A1		-0.27		0.17		7.5		2.7		10.2
ENSG00000231389	HLA-DPA1		-0.26		0.05		6.9		0.3		7.2
ENSG00000211899	IGHM		-0.25		0.06		6.1		0.3		6.4
ENSG00000211896	IGHG1		0.12		0.10		1.5		1.0		2.5
ENSG00000124772	CPNE5		0.05		0.04		0.2		0.1		0.4

Table A140: Canonical loadings and explained variance (RNA-Seq, Neutrophils, Day 7, Gene variables). Gene variables with at least 25% of explained variance for the first two canonical variates are listed. GX: canonical loadings for canonical variate X, VX: % explained variance for canonical variate X, V1+V2: total % explained variance for both canonical variates. Values for CCA results with a poor cross validation score ($<$ 0.70) are left blank. Sorted by maximum explained variance across treatments (V1+V2).

IMO Variable	IMO Variable Name	I1 (SV-AS03)	I1 (SV-PBS)	I2 (SV-AS03)	I2 (SV-PBS)	V1 (SV-AS03)	V1 (SV-PBS)	V2 (SV-AS03)	V2 (SV-PBS)	V1+V2 (SV-AS03)	V1+V2 (SV-PBS)
MIP-1α	Macrophage inflammatory protein		0.91		0.24		81.9		5.9		87.8
IP-10	Interferon gamma-induced protein 10		0.92		0.19		83.8		3.7		87.5
MCP-1	Monocyte chemoattractant protein-1		0.74		0.52		55.2		26.7		81.8
TNF	Tumor necrosis factors		-0.36		0.78		12.8		61.1		73.9
IL-1β	Interleukin-1 beta		-0.73		-0.36		52.6		12.7		65.4
Eotaxin-1	Eotaxin-1		-0.73		0.21		52.6		4.4		57.0
IL-12p70	Interleukin-12 p70		-0.60		-0.09		35.5		0.9		36.4
Nt	Microneutralization inhibition		0.54		-0.08		29.6		0.6		30.2
IL-6	Interleukin-6		-0.28		-0.46		7.9		21.0		29.0
IFN-γ	Interferon gamma		-0.24		-0.48		5.8		22.8		28.6
IL-10	Interleukin-10		-0.16		0.11		2.7		1.3		3.9
RANTES	Chemokine (C-C motif) ligand 5		0.02		0.13		0.0		1.7		1.8
IL-8	Interleukin-8		0.08		0.03		0.7		0.1		0.8

Table A141: Canonical loadings and explained variance (RNA-Seq, Neutrophils, Day 7, IMO variables). IMO variables with at least 25% of explained variance for the first two canonical variates are listed. GX: canonical loadings for canonical variate X, VX: % explained variance for canonical variate X, V1+V2: total % explained variance for both canonical variates. Values for CCA results with a poor cross validation score ($<$ 0.70) are left blank. Sorted by maximum explained variance across treatments (V1+V2).

Gene ID	Gene Name	G1 (SV-AS03)	G1 (SV-PBS)	G2 (SV-AS03)	G2 (SV-PBS)	V1 (SV-AS03)	V1 (SV-PBS)	V2 (SV-AS03)	V2 (SV-PBS)	V1+V2 (SV-AS03)	V1+V2 (SV-PBS)
ENSG00000104738	MCM4	0.83	-0.27	0.42	0.17	69.3	7.3	17.9	2.9	87.2	10.3
ENSG00000244694	C6orf138	0.86	0.10	-0.28	-0.62	73.4	1.0	7.6	38.6	81.0	39.6
ENSG00000204705	UBTFL5	0.68	0.14	0.55	-0.44	45.7	2.0	30.3	19.5	76.0	21.5
ENSG00000012124	CD22	0.46	0.74	0.17	-0.41	21.0	55.2	2.8	16.5	23.8	71.7
ENSG00000004799	PDK4	0.71	0.07	-0.03	0.83	51.0	0.4	0.1	69.2	51.1	69.7
ENSG00000204655	MOG	0.47	0.07	0.68	-0.64	22.4	0.4	46.0	40.9	68.4	41.4
ENSG00000175592	FOSL1	0.77	-0.39	0.21	0.31	59.8	15.2	4.5	9.4	64.3	24.6
ENSG00000110651	CD81	0.39	0.37	-0.68	0.38	15.5	13.3	46.3	14.5	61.9	27.9

Gene ID	Gene Name	G1 (SV-AS03)	G1 (SV-PBS)	G2 (SV-AS03)	G2 (SV-PBS)	V1 (SV-AS03)	V1 (SV-PBS)	V2 (SV-AS03)	V2 (SV-PBS)	V1+V2 (SV-AS03)	V1+V2 (SV-PBS)
ENSG00000252197	AC091047.1	-0.77	0.39	0.07	-0.11	59.6	15.0	0.5	1.2	60.0	16.2
ENSG00000252331	AP002812.1	-0.76	0.35	0.12	0.07	58.3	11.9	1.4	0.5	59.7	12.4
ENSG00000211899	IGHM	0.36	0.76	-0.00	0.02	12.9	57.2	0.0	0.0	12.9	57.2
ENSG00000119686	AC007182.1	-0.51	-0.29	-0.55	-0.13	26.3	8.6	30.7	1.7	57.0	10.3
ENSG00000123384	LRP1	-0.02	0.04	0.75	-0.64	0.0	0.1	56.4	40.8	56.5	40.9
ENSG00000160932	LY6E	0.00	0.59	0.21	0.46	0.0	34.5	4.2	21.2	4.2	55.7
ENSG00000231389	HLA-DPA1	0.46	0.35	-0.05	0.66	21.1	12.0	0.3	42.9	21.4	55.0
ENSG00000247982	AC016525.1	0.55	0.55	0.09	-0.49	29.8	30.3	0.8	23.7	30.6	54.0
ENSG00000222852	Y_RNA_222852	-0.19	0.70	-0.23	0.20	3.6	49.1	5.2	3.9	8.9	53.1
ENSG00000164403	SHROOM1	-0.57	0.23	-0.44	-0.59	32.6	5.3	19.7	34.9	52.4	40.2
ENSG00000141750	STAC2	-0.19	0.23	0.50	-0.68	3.7	5.4	25.4	46.5	29.1	51.9
ENSG00000117632	STMN1	0.71	0.04	0.11	-0.46	50.3	0.2	1.3	21.2	51.6	21.4
ENSG00000146192	FGD2	0.71	0.23	-0.11	0.00	50.2	5.5	1.3	0.0	51.5	5.5
ENSG00000108576	SLC6A4	0.70	0.19	0.11	-0.57	49.0	3.7	1.3	32.4	50.2	36.2
ENSG00000251826	AL365332.1	-0.70	0.41	-0.10	0.00	48.8	16.9	1.0	0.0	49.8	16.9
ENSG00000182310	NCRNA00085	0.46	-0.56	-0.53	-0.11	20.7	31.8	28.6	1.1	49.3	32.9
ENSG00000252229	AC098691.1	-0.69	0.33	0.13	0.50	47.2	10.9	1.7	24.6	48.8	35.5
ENSG00000211898	IGHD	0.47	0.66	0.07	0.20	22.2	43.8	0.5	3.9	22.8	47.7
ENSG00000252318	AC097532.1	-0.67	0.46	-0.15	-0.18	45.3	21.6	2.2	3.2	47.5	24.8
ENSG00000188536	HBA2	0.31	0.60	-0.45	-0.34	9.9	36.0	20.3	11.3	30.2	47.3
ENSG00000257475	RP11-983P16.2	0.50	0.20	0.44	-0.65	24.8	4.0	19.8	42.8	44.6	46.8
ENSG00000109919	MTCH2	0.54	-0.52	-0.17	0.43	28.8	27.3	2.9	18.5	31.7	45.8
ENSG00000244734	HBB	0.26	0.45	-0.26	-0.49	6.7	20.7	6.7	23.7	13.4	44.4
ENSG00000168685	IL7R	0.64	-0.33	-0.18	-0.10	41.1	10.6	3.2	1.0	44.4	11.6
ENSG00000211895	IGHA1	0.49	0.65	-0.06	-0.09	24.2	42.9	0.4	0.7	24.5	43.6
ENSG00000197816	C9orf174	0.48	0.18	0.16	-0.62	23.2	3.2	2.4	38.8	25.6	42.0
ENSG00000197928	ZNF677	0.44	0.22	0.28	-0.61	19.5	4.7	7.9	36.9	27.4	41.5
ENSG00000167037	SGSM1	0.34	0.13	0.05	-0.63	11.5	1.8	0.2	39.7	11.7	41.5
ENSG00000078114	NEBL	0.61	-0.13	0.19	-0.47	37.6	1.7	3.6	22.1	41.3	23.7
ENSG00000206172	HBA1	0.23	0.52	-0.43	-0.38	5.2	26.6	18.6	14.6	23.8	41.2
ENSG00000188290	HES4	0.01	-0.01	-0.38	0.64	0.0	0.0	14.3	40.9	14.3	41.0
ENSG00000205929	C21orf62	0.41	0.13	-0.00	-0.63	16.5	1.7	0.0	39.2	16.5	40.9
ENSG00000158286	RNF207	-0.25	0.26	0.39	-0.58	6.3	6.7	15.5	33.2	21.8	39.9
ENSG00000223865	HLA-DPB1	0.62	0.25	-0.10	0.08	38.7	6.4	1.1	0.7	39.8	7.1
ENSG00000153064	BANK1	0.58	0.02	0.23	0.55	34.1	0.0	5.2	29.8	39.3	29.9
ENSG00000211896	IGHG1	0.59	0.49	0.19	-0.29	34.4	23.7	3.6	8.5	38.0	32.2
ENSG00000171320	ESCO2	0.02	0.16	-0.14	-0.59	0.0	2.5	2.0	35.2	2.1	37.7
ENSG00000111335	OAS2	0.25	0.49	0.49	0.36	6.1	24.3	24.1	13.0	30.2	37.3
ENSG00000172000	ZNF556	0.17	0.26	0.07	-0.55	2.8	6.9	0.5	29.9	3.3	36.9
ENSG00000196208	GREB1	0.48	0.30	-0.24	-0.52	23.4	9.2	5.9	27.5	29.3	36.7
ENSG00000038427	VCAN	-0.08	-0.09	0.44	-0.59	0.6	0.7	19.3	34.4	19.9	35.1
ENSG00000235297	RP11-504H5.1	-0.02	0.16	0.59	-0.47	0.1	2.6	34.3	22.2	34.4	24.8
ENSG00000255478	RP11-867O8.5	0.19	0.09	0.50	-0.58	3.7	0.8	24.8	33.5	28.4	34.3
ENSG00000232406	RP11-234K24.3	-0.41	0.19	0.23	-0.54	17.2	3.7	5.1	29.0	22.3	32.7
ENSG00000135702	CHST5	-0.01	0.17	0.46	-0.54	0.0	2.8	21.2	28.9	21.2	31.7
ENSG00000134762	DSC3	0.20	0.24	0.28	-0.51	4.0	5.8	7.8	25.7	11.7	31.5
ENSG00000109684	CLNK	-0.00	0.16	0.41	-0.53	0.0	2.7	16.8	28.5	16.8	31.2
ENSG00000134321	RSAD2	-0.14	0.45	0.20	0.31	1.9	20.5	3.8	9.5	5.7	30.0
ENSG00000187492	CDHR4	0.01	0.14	0.47	-0.52	0.0	2.0	21.7	27.0	21.7	29.0
ENSG00000111331	OAS3	-0.48	0.44	0.18	0.31	23.3	19.2	3.2	9.5	26.5	28.7
ENSG00000249383	RP11-1018N14.1	0.19	-0.01	-0.22	-0.53	3.7	0.0	4.8	28.0	8.5	28.0

Gene ID	Gene Name	G1 (SV-AS03)	G1 (SV-PBS)	G2 (SV-AS03)	G2 (SV-PBS)	V1 (SV-AS03)	V1 (SV-PBS)	V2 (SV-AS03)	V2 (SV-PBS)	V1+V2 (SV-AS03)	V1+V2 (SV-PBS)
ENSG00000238272	RP3-436N22.3	-0.14	0.34	-0.34	-0.39	2.0	11.4	11.4	15.1	13.5	26.5
ENSG00000009765	IYD	0.26	0.12	-0.16	-0.48	6.7	1.4	2.4	23.5	9.1	24.9
ENSG00000132915	PDE6A	-0.00	0.19	0.25	-0.45	0.0	3.5	6.0	20.6	6.0	24.1
ENSG00000176406	RIMS2	-0.24	0.10	-0.30	-0.48	5.9	1.0	9.0	23.1	14.8	24.1
ENSG00000123243	ITIH5	-0.00	0.18	-0.14	-0.45	0.0	3.3	2.0	19.9	2.0	23.2
ENSG00000184979	USP18	0.12	0.32	-0.18	0.35	1.5	10.5	3.1	11.9	4.6	22.5
ENSG00000140968	IRF8	0.31	0.33	0.34	0.15	9.4	10.8	11.3	2.2	20.7	13.0
ENSG00000156738	MS4A1	0.44	0.44	0.05	-0.04	19.1	19.1	0.3	0.2	19.4	19.3
ENSG00000256745	RP11-680H20.1	-0.19	-0.05	0.05	-0.43	3.6	0.2	0.3	18.4	3.9	18.6
ENSG00000078081	LAMP3	0.05	0.28	-0.40	0.31	0.2	8.0	15.9	9.7	16.2	17.6
ENSG00000118432	CNR1	0.39	-0.23	-0.12	-0.21	15.0	5.3	1.4	4.3	16.4	9.5
ENSG00000136573	BLK	0.37	0.17	0.03	0.07	13.9	2.9	0.1	0.4	14.0	3.3
ENSG00000196092	PAX5	0.27	0.15	-0.24	0.02	7.5	2.2	6.0	0.0	13.5	2.3
ENSG00000090104	RGS1	0.34	0.13	-0.07	0.24	11.9	1.8	0.5	5.6	12.3	7.4

Table A142: Canonical loadings and explained variance (RNA-Seq, Neutrophils, Day 28, Gene variables). Gene variables with at least 25% of explained variance for the first two canonical variates are listed. GX: canonical loadings for canonical variate X, VX: % explained variance for canonical variate X, V1+V2: total % explained variance for both canonical variates. Values for CCA results with a poor cross validation score ($<$ 0.70) are left blank. Sorted by maximum explained variance across treatments (V1+V2).

IMO Variable	IMO Variable Name	I1 (SV-AS03)	I1 (SV-PBS)	I2 (SV-AS03)	I2 (SV-PBS)	V1 (SV-AS03)	V1 (SV-PBS)	V2 (SV-AS03)	V2 (SV-PBS)	V1+V2 (SV-AS03)	V1+V2 (SV-PBS)
IL-12p70	Interleukin-12 p70	0.95	0.52	0.24	0.56	90.8	27.4	5.9	31.7	96.7	59.2
IL-10	Interleukin-10	0.27	0.10	0.91	-0.32	7.4	0.9	83.6	10.1	91.0	11.0
Nt	Microneutralization inhibition	-0.78	0.20	0.50	0.34	60.3	4.1	25.3	11.5	85.6	15.6
HAI	Hemagglutination inhibition	-0.68	-0.17	0.58	0.60	46.8	3.1	33.6	35.5	80.4	38.6
IL-8	Interleukin-8	0.40	0.89	0.64	-0.02	15.9	78.8	41.3	0.1	57.2	78.8
IL-6	Interleukin-6	0.83	-0.07	-0.18	-0.04	68.4	0.6	3.3	0.2	71.7	0.7
MIP-1α	Macrophage inflammatory protein	0.13	0.71	-0.38	-0.41	1.7	49.7	14.8	16.8	16.4	66.5
Eotaxin-1	Eotaxin-1	0.20	0.22	0.78	-0.28	4.0	4.8	61.0	8.1	65.0	12.8
IP-10	Interferon gamma-induced protein 10	-0.57	0.40	0.52	-0.57	32.7	16.1	26.8	32.6	59.5	48.7
TNF	Tumor necrosis factors	0.44	0.10	0.52	-0.42	19.0	1.1	27.3	17.5	46.3	18.5
IL-1β	Interleukin-1 beta	0.37	0.19	0.52	-0.34	13.9	3.7	27.2	11.5	41.1	15.2
RANTES	Chemokine (C-C motif) ligand 5	0.35	0.09	0.47	-0.28	12.5	0.7	21.8	8.0	34.3	8.8
MCP-1	Monocyte chemoattractant protein-1	0.51	0.01	0.20	-0.51	25.8	0.0	4.1	25.8	29.9	25.8
IFN-γ	Interferon gamma	-0.26	0.15	0.40	-0.02	6.9	2.2	16.2	0.0	23.2	2.3

Table A143: Canonical loadings and explained variance (RNA-Seq, Neutrophils, Day 28, IMO variables). IMO variables with at least 25% of explained variance for the first two canonical variates are listed. GX: canonical loadings for canonical variate X, VX: % explained variance for canonical variate X, V1+V2: total % explained variance for both canonical variates. Values for CCA results with a poor cross validation score ($<$ 0.70) are left blank. Sorted by maximum explained variance across treatments (V1+V2).

Gene ID	Gene Name	G1 (SV-AS03)	G1 (SV-PBS)	G2 (SV-AS03)	G2 (SV-PBS)	V1 (SV-AS03)	V1 (SV-PBS)	V2 (SV-AS03)	V2 (SV-PBS)	V1+V2 (SV-AS03)	V1+V2 (SV-PBS)
ENSG00000106565	TMEM176B	0.97		0.03		94.8		0.1		94.9	
ENSG00000204103	MAFB	0.96		-0.15		91.4		2.2		93.5	

Gene ID	Gene Name	G1 (SV-AS03)	G1 (SV-PBS)	G2 (SV-AS03)	G2 (SV-PBS)	V1 (SV-AS03)	V1 (SV-PBS)	V2 (SV-AS03)	V2 (SV-PBS)	V1+V2 (SV-AS03)	V1+V2 (SV-PBS)
ENSG00000170458	CD14	0.96		-0.08		92.3		0.6		92.9	
ENSG00000182022	CHST15	0.95		-0.17		89.9		2.9		92.7	
ENSG00000163221	S100A12	0.87		-0.40		75.3		15.8		91.1	
ENSG00000104783	KCNN4	0.88		0.33		78.2		11.1		89.3	
ENSG00000172243	CLEC7A	0.94		-0.03		88.7		0.1		88.8	
ENSG00000105967	TFEC	0.93		0.03		87.2		0.1		87.3	
ENSG00000155465	SLC7A7	0.91		-0.20		83.1		4.0		87.1	
ENSG00000116701	NCF2	0.91		0.08		82.7		0.6		83.3	
ENSG00000074964	ARHGEF10L	0.91		0.00		83.0		0.0		83.0	
ENSG00000181631	P2RY13	0.77		-0.49		58.6		24.3		82.9	
ENSG00000163563	MNDA	0.91		0.05		82.7		0.2		82.9	
ENSG00000121316	PLBD1	0.90		-0.04		81.3		0.1		81.4	
ENSG00000135218	CD36	0.88		0.20		76.6		4.0		80.5	
ENSG00000038427	VCAN	0.86		0.27		73.2		7.1		80.3	
ENSG00000178965	C1orf173	-0.74		-0.49		55.4		24.0		79.3	
ENSG00000186407	CD300E	0.85		0.26		72.3		6.8		79.2	
ENSG00000140511	HAPLN3	-0.55		-0.70		30.0		48.5		78.5	
ENSG00000153823	PID1	0.80		0.37		64.4		14.0		78.4	
ENSG00000198053	SIRPA	0.88		0.12		76.8		1.3		78.2	
ENSG00000085265	FCN1	0.80		0.38		63.4		14.4		77.8	
ENSG00000235568	NFAM1	0.88		0.03		77.6		0.1		77.8	
ENSG00000187554	TLR5	0.78		-0.41		60.2		17.0		77.2	
ENSG00000204472	AIF1	0.87		0.13		75.3		1.8		77.1	
ENSG00000090382	LYZ	0.86		0.17		74.3		2.8		77.1	
ENSG00000119535	CSF3R	0.86		0.16		74.1		2.6		76.7	
ENSG00000161944	ASGR2	0.65		0.58		42.2		34.1		76.3	
ENSG00000156587	UBE2L6	-0.65		-0.58		42.8		33.2		76.0	
ENSG00000106066	CPVL	0.56		0.66		31.7		44.0		75.7	
ENSG00000224397	RP11-290F20.3	0.75		-0.44		56.1		19.7		75.7	
ENSG00000123384	LRP1	0.87		0.03		75.4		0.1		75.5	
ENSG00000186074	CD300LF	0.86		0.05		73.9		0.2		74.1	
ENSG00000182578	CSF1R	0.84		0.16		71.0		2.6		73.6	
ENSG00000119917	IFIT3	-0.67		-0.53		45.3		27.8		73.1	
ENSG00000010327	STAB1	0.85		0.06		72.5		0.4		73.0	
ENSG00000169385	RNASE2	0.70		0.49		48.6		24.2		72.8	
ENSG00000188906	LRRK2	0.81		0.22		66.4		4.8		71.2	
ENSG00000110077	MS4A6A	0.76		0.36		58.3		12.7		71.1	
ENSG00000187116	LILRA5	0.82		-0.18		66.7		3.4		70.0	
ENSG00000053918	KCNQ1	0.81		0.18		66.3		3.1		69.4	
ENSG00000139970	RTN1	0.76		0.32		57.8		10.1		67.8	
ENSG00000137959	IFI44L	-0.61		-0.54		37.7		29.1		66.9	
ENSG00000117228	GBP1	-0.64		-0.51		40.7		26.0		66.7	
ENSG00000138496	PARP9	-0.64		-0.51		40.7		25.8		66.5	
ENSG00000177575	CD163	0.79		0.18		63.1		3.3		66.4	
ENSG00000185215	TNFAIP2	0.80		0.16		63.9		2.4		66.3	
ENSG00000116962	NID1	0.81		0.05		66.0		0.2		66.2	
ENSG00000171051	FPR1	0.70		0.40		49.4		15.7		65.1	
ENSG00000158481	CD1C	0.78		0.19		61.4		3.7		65.0	
ENSG00000183023	SLC8A1	0.80		0.06		64.4		0.3		64.7	
ENSG00000146592	CREB5	0.78		0.20		60.3		3.9		64.2	
ENSG00000117115	PADI2	0.78		-0.13		61.3		1.7		63.0	

Gene ID	Gene Name	G1 (SV-AS03)	G1 (SV-PBS)	G2 (SV-AS03)	G2 (SV-PBS)	V1 (SV-AS03)	V1 (SV-PBS)	V2 (SV-AS03)	V2 (SV-PBS)	V1+V2 (SV-AS03)	V1+V2 (SV-PBS)
ENSG00000100079	LGALS2	0.70		0.38		48.6		14.1		62.8	
ENSG00000196209	SIRPB2	0.75		0.26		55.6		7.0		62.7	
ENSG00000111331	OAS3	-0.50		-0.61		25.2		36.8		61.9	
ENSG00000255197	RP11-750H9.5	0.75		0.17		56.9		2.8		59.6	
ENSG00000167600	CYP2S1	0.67		0.37		44.8		13.9		58.7	
ENSG00000214872	SMTNL1	-0.55		-0.52		30.2		26.7		56.9	
ENSG00000243836	RP4-555L14.5	0.06		0.75		0.3		56.3		56.7	
ENSG00000132694	ARHGEF11	0.75		0.05		56.4		0.2		56.6	
ENSG00000010030	ETV7	-0.63		-0.40		40.1		15.7		55.8	
ENSG00000123358	NR4A1	0.74		-0.09		54.7		0.9		55.6	
ENSG00000115415	STAT1	-0.53		-0.52		28.5		27.0		55.5	
ENSG00000164684	ZNF704	0.67		0.31		44.6		9.4		54.0	
ENSG00000163625	WDFY3	0.67		0.29		45.5		8.2		53.7	
ENSG00000114013	CD86	0.60		0.42		35.7		17.8		53.6	
ENSG00000004799	PDK4	0.42		-0.59		17.8		35.2		53.0	
ENSG00000125347	IRF1	-0.58		-0.40		34.2		16.3		50.5	
ENSG00000109743	BST1	0.60		0.38		35.8		14.4		50.2	
ENSG00000025708	TYMP	-0.16		-0.69		2.6		47.2		49.8	
ENSG00000196497	IPO4	-0.55		-0.44		30.2		19.6		49.8	
ENSG00000164125	FAM198B	0.69		0.14		47.6		1.8		49.5	
ENSG00000121552	CSTA	0.52		0.46		26.6		21.4		48.0	
ENSG00000133106	EPSTI1	-0.58		-0.34		33.1		11.7		44.8	
ENSG00000158473	CD1D	0.57		0.36		32.2		12.6		44.8	
ENSG00000213928	IRF9	-0.58		-0.33		33.4		10.8		44.2	
ENSG00000087237	CETP	-0.45		-0.47		20.6		22.3		42.9	
ENSG00000105501	SIGLEC5	0.49		0.42		24.0		17.4		41.5	
ENSG00000184371	CSF1	-0.58		-0.14		33.2		1.9		35.1	
ENSG00000140044	JDP2	0.49		0.31		24.2		9.9		34.0	
ENSG00000105205	CLC	0.35		0.45		12.2		20.6		32.7	
ENSG00000168913	ENHO	0.38		0.43		14.1		18.2		32.3	
ENSG00000082438	COBLL1	-0.14		0.55		1.8		29.9		31.7	
ENSG00000184811	TUSC5	-0.55		-0.12		30.2		1.5		31.7	
ENSG00000164023	SGMS2	0.38		0.42		14.1		17.5		31.6	
ENSG00000140287	HDC	0.13		0.54		1.6		29.6		31.2	
ENSG00000157240	FZD1	0.34		0.42		11.8		17.9		29.7	
ENSG00000163534	FCRL1	-0.51		-0.12		26.4		1.5		27.9	
ENSG00000177989	ODF3B	-0.13		-0.51		1.6		26.0		27.6	
ENSG00000114737	CISH	-0.19		-0.47		3.4		21.9		25.3	
ENSG00000125462	C1orf61	-0.45		-0.21		20.1		4.6		24.7	
ENSG00000151650	VENTX	0.44		0.19		19.8		3.6		23.4	
ENSG00000104044	OCA2	0.03		0.48		0.1		23.3		23.4	
ENSG00000139132	FGD4	0.46		0.13		21.0		1.6		22.6	
ENSG00000123094	RASSF8	-0.34		0.30		11.4		9.2		20.6	
ENSG00000166927	MS4A7	0.42		-0.13		17.9		1.7		19.5	
ENSG00000211899	IGHM	0.13		0.42		1.6		17.7		19.3	
ENSG00000170476	RP11-1280I22.1	-0.39		-0.11		15.4		1.3		16.7	
ENSG00000183625	CCR3	0.32		-0.22		10.0		4.7		14.7	
ENSG00000141096	DPEP3	0.12		0.35		1.3		12.3		13.7	
ENSG00000172037	LAMB2	0.19		0.31		3.5		9.8		13.4	
ENSG00000144369	FAM171B	0.05		-0.36		0.3		12.9		13.2	
ENSG00000179348	GATA2	0.08		0.34		0.7		11.6		12.2	

Gene ID	Gene Name	G1 (SV-AS03)	G1 (SV-PBS)	G2 (SV-AS03)	G2 (SV-PBS)	V1 (SV-AS03)	V1 (SV-PBS)	V2 (SV-AS03)	V2 (SV-PBS)	V1+V2 (SV-AS03)	V1+V2 (SV-PBS)
ENSG00000183779	ZNF703	0.32		-0.14		10.3		1.9		12.2	
ENSG00000137968	SLC44A5	-0.30		-0.12		9.2		1.4		10.6	
ENSG00000185758	CLDN24	0.09		-0.25		0.7		6.5		7.2	
ENSG00000230084	RP4-613B23.1	-0.24		0.08		5.7		0.6		6.3	
ENSG00000188886	ASTL	0.15		0.15		2.3		2.2		4.6	
ENSG00000231865	AP000936.3	-0.14		-0.06		2.0		0.4		2.3	
ENSG00000109321	AREG	-0.04		-0.13		0.1		1.6		1.8	
ENSG00000197594	ENPP1	0.07		0.08		0.5		0.6		1.1	
ENSG00000211895	IGHA1	-0.01		0.01		0.0		0.0		0.0	

Table A144: Canonical loadings and explained variance (RNA-Seq, NK-cells, Day 1, Gene variables). Gene variables with at least 25% of explained variance for the first two canonical variates are listed. GX: canonical loadings for canonical variate X, VX: % explained variance for canonical variate X, V1+V2: total % explained variance for both canonical variates. Values for CCA results with a poor cross validation score ($<$ 0.70) are left blank. Sorted by maximum explained variance across treatments (V1+V2).

IMO Variable	IMO Variable Name	I1 (SV-AS03)	I1 (SV-PBS)	I2 (SV-AS03)	I2 (SV-PBS)	V1 (SV-AS03)	V1 (SV-PBS)	V2 (SV-AS03)	V2 (SV-PBS)	V1+V2 (SV-AS03)	V1+V2 (SV-PBS)
MIP-1α	Macrophage inflammatory protein	-0.76		-0.54		58.0		29.0		87.0	
IL-12p70	Interleukin-12 p70	-0.53		0.75		27.6		56.3		83.9	
IP-10	Interferon gamma-induced protein 10	-0.53		-0.61		28.4		37.0		65.4	
IL-6	Interleukin-6	-0.61		0.32		37.2		10.2		47.4	
TNF	Tumor necrosis factors	0.36		0.56		12.9		31.8		44.7	
Nt	Microneutralization inhibition	0.48		-0.23		23.1		5.1		28.2	
Eotaxin-1	Eotaxin-1	0.50		0.19		24.6		3.6		28.2	
IL-10	Interleukin-10	-0.28		0.43		7.8		18.6		26.4	
IL-1β	Interleukin-1 beta	-0.33		0.18		10.6		3.3		13.9	
RANTES	Chemokine (C-C motif) ligand 5	-0.36		-0.09		12.9		0.8		13.8	
IFN-γ	Interferon gamma	0.16		0.23		2.6		5.2		7.8	
IL-8	Interleukin-8	0.13		0.15		1.7		2.1		3.8	
MCP-1	Monocyte chemoattractant protein-1	-0.13		-0.10		1.8		0.9		2.7	

Table A145: Canonical loadings and explained variance (RNA-Seq, NK-cells, Day 1, IMO variables). IMO variables with at least 25% of explained variance for the first two canonical variates are listed. GX: canonical loadings for canonical variate X, VX: % explained variance for canonical variate X, V1+V2: total % explained variance for both canonical variates. Values for CCA results with a poor cross validation score ($<$ 0.70) are left blank. Sorted by maximum explained variance across treatments (V1+V2).

Gene ID	Gene Name	G1 (SV-AS03)	G1 (SV-PBS)	G2 (SV-AS03)	G2 (SV-PBS)	V1 (SV-AS03)	V1 (SV-PBS)	V2 (SV-AS03)	V2 (SV-PBS)	V1+V2 (SV-AS03)	V1+V2 (SV-PBS)
ENSG00000094804	CDC6	-0.74	0.35	-0.64	0.51	54.1	12.1	41.2	26.5	95.4	38.7
ENSG00000167900	TK1	-0.85	0.51	-0.48	0.49	71.7	26.4	23.2	23.8	94.9	50.2
ENSG00000093009	CDC45	-0.73	0.48	-0.64	0.71	53.2	22.8	41.5	50.5	94.7	73.3
ENSG00000178999	AURKB	-0.80	0.21	-0.55	-0.39	64.0	4.4	30.3	15.4	94.3	19.8
ENSG00000176890	TYMS	-0.78	0.39	-0.57	0.53	60.7	15.1	32.1	27.7	92.8	42.9
ENSG00000121152	NCAPH	-0.74	0.49	-0.59	0.52	55.0	23.7	34.3	27.4	89.3	51.1
ENSG00000165891	E2F7	-0.64	0.47	-0.69	0.58	40.6	22.4	48.1	33.9	88.7	56.3
ENSG00000135451	TROAP	-0.79	0.59	-0.51	0.12	62.6	34.4	26.0	1.6	88.6	35.9

Gene ID	Gene Name	G1 (SV-AS03)	G1 (SV-PBS)	G2 (SV-AS03)	G2 (SV-PBS)	V1 (SV-AS03)	V1 (SV-PBS)	V2 (SV-AS03)	V2 (SV-PBS)	V1+V2 (SV-AS03)	V1+V2 (SV-PBS)
ENSG00000101412	E2F1	-0.72	0.67	-0.60	0.18	52.4	44.8	36.2	3.2	88.6	48.0
ENSG00000051341	POLQ	-0.67	0.76	-0.66	0.24	45.2	57.7	43.3	5.9	88.6	63.6
ENSG00000007968	E2F2	-0.73	0.51	-0.59	-0.12	53.0	25.5	35.2	1.5	88.2	27.0
ENSG00000089685	BIRC5	-0.61	0.20	-0.71	0.72	37.7	4.0	49.7	52.1	87.4	56.0
ENSG00000100162	CENPM	-0.88	-0.57	-0.29	0.07	78.1	32.2	8.6	0.5	86.7	32.7
ENSG00000127564	PKMYT1	-0.71	0.51	-0.59	0.71	50.4	26.4	35.1	50.7	85.5	77.1
ENSG00000244734	HBB	0.44	-0.43	-0.81	0.01	19.7	18.8	65.5	0.0	85.3	18.8
ENSG00000129173	E2F8	-0.68	0.59	-0.62	-0.02	45.9	34.9	38.7	0.1	84.5	35.0
ENSG00000066279	ASPM	-0.60	0.64	-0.70	0.51	35.9	41.4	48.4	25.9	84.4	67.3
ENSG00000122966	CIT	-0.64	0.31	-0.65	-0.28	41.4	9.6	41.9	7.9	83.4	17.5
ENSG00000101057	MYBL2	-0.71	0.63	-0.57	0.51	50.5	39.6	32.2	26.2	82.7	65.7
ENSG00000171848	RRM2	-0.60	0.63	-0.57	0.65	35.9	39.6	32.5	42.8	68.4	82.4
ENSG00000140534	C15orf42	-0.79	0.32	-0.43	0.51	62.4	10.4	18.8	26.2	81.2	36.7
ENSG00000189057	FAM111B	-0.56	0.64	-0.70	-0.14	31.9	41.1	48.8	2.0	80.7	43.1
ENSG00000105173	CCNE1	-0.80	0.66	-0.40	0.28	64.2	43.0	16.4	7.7	80.6	50.7
ENSG00000011426	ANLN	-0.82	0.53	-0.36	0.07	67.1	28.0	13.3	0.5	80.4	28.5
ENSG00000131153	GINS2	-0.87	0.47	-0.19	0.27	76.3	21.9	3.7	7.2	80.0	29.1
ENSG00000137812	CASC5	-0.67	0.00	-0.60	0.18	44.3	0.0	35.6	3.3	80.0	3.3
ENSG00000188536	HBA2	0.34	-0.46	-0.83	0.06	11.4	21.3	68.6	0.3	79.9	21.6
ENSG00000148773	MKI67	-0.56	0.53	-0.70	0.60	31.3	27.6	48.6	36.1	79.9	63.7
ENSG00000164045	CDC25A	-0.46	0.49	-0.76	0.09	21.6	23.9	57.8	0.8	79.4	24.7
ENSG00000131747	TOP2A	-0.70	0.33	-0.55	0.64	48.5	10.7	30.7	40.5	79.3	51.2
ENSG00000105011	ASF1B	-0.81	0.49	-0.38	-0.15	64.9	23.9	14.3	2.4	79.1	26.2
ENSG00000065328	MCM10	-0.79	0.51	-0.40	0.50	62.5	25.6	16.3	25.4	78.9	51.0
ENSG00000170312	CDK1	-0.74	0.05	-0.49	0.18	54.2	0.2	23.9	3.2	78.1	3.4
ENSG00000167513	CDT1	-0.64	0.32	-0.61	0.56	41.3	10.3	36.8	31.6	78.0	41.9
ENSG00000122952	ZWINT	-0.80	0.73	-0.38	-0.36	63.6	53.9	14.3	13.0	78.0	66.9
ENSG00000072571	HMMR	-0.74	0.30	-0.47	-0.07	55.2	9.1	21.9	0.5	77.0	9.6
ENSG00000135476	ESPL1	-0.51	0.53	-0.71	-0.17	25.7	28.0	51.0	2.8	76.8	30.8
ENSG00000118193	KIF14	-0.44	0.01	-0.75	0.01	19.6	0.0	57.0	0.0	76.6	0.0
ENSG00000112984	KIF20A	-0.74	0.16	-0.46	0.47	54.9	2.7	21.6	22.2	76.4	24.9
ENSG00000123485	HJURP	-0.68	0.81	-0.55	-0.06	46.1	66.1	30.0	0.3	76.1	66.4
ENSG00000137807	KIF23	-0.48	0.26	-0.73	0.32	23.4	6.6	52.6	10.1	76.0	16.8
ENSG00000109805	NCAPG	-0.63	0.13	-0.61	0.64	39.2	1.6	36.8	40.6	76.0	42.2
ENSG00000075218	GTSE1	-0.47	0.28	-0.73	0.40	22.4	7.6	53.5	15.6	75.9	23.2
ENSG00000137804	NUSAP1	-0.77	0.54	-0.41	0.48	59.1	29.7	16.6	22.7	75.7	52.4
ENSG00000151725	MLF1IP	-0.66	0.56	-0.56	0.20	44.1	31.3	31.6	4.1	75.7	35.4
ENSG00000143476	DTL	-0.75	0.38	-0.44	0.49	55.7	14.4	19.6	24.0	75.3	38.4
ENSG00000146670	CDCA5	-0.62	0.59	-0.60	0.57	38.3	35.2	36.6	32.2	74.9	67.5
ENSG00000156970	BUB1B	-0.59	0.05	-0.63	-0.45	35.0	0.3	39.5	20.1	74.5	20.4
ENSG00000171241	SHCBP1	-0.46	0.65	-0.72	-0.08	20.8	42.4	52.2	0.7	73.1	43.1
ENSG00000237649	KIFC1	-0.64	0.59	-0.57	0.40	40.8	35.0	32.3	16.0	73.0	51.0
ENSG00000169607	CKAP2L	-0.68	0.10	-0.51	0.15	46.2	0.9	26.2	2.4	72.3	3.3
ENSG00000088325	TPX2	-0.55	-0.14	-0.64	0.78	30.2	2.0	40.9	60.3	71.2	62.3
ENSG00000180875	GREM2	0.02	-0.35	-0.84	-0.19	0.1	12.4	71.1	3.8	71.1	16.2
ENSG00000138180	CEP55	-0.48	0.83	-0.37	0.17	23.2	68.2	14.0	2.9	37.2	71.1
ENSG00000085999	RAD54L	-0.74	0.72	-0.40	0.14	55.2	51.4	15.7	1.9	70.9	53.3
ENSG00000163808	KIF15	-0.66	0.46	-0.52	0.22	43.5	21.2	27.2	4.8	70.7	26.0
ENSG00000243838	PSMC1P7	-0.15	0.18	-0.82	0.46	2.2	3.1	67.6	21.3	69.8	24.4
ENSG00000206172	HBA1	0.34	-0.36	-0.76	0.13	11.3	13.0	58.2	1.6	69.5	14.6
ENSG00000139618	BRCA2	-0.55	0.66	-0.63	0.33	29.9	43.5	39.6	10.8	69.5	54.3

Gene ID	Gene Name	G1 (SV-AS03)	G1 (SV-PBS)	G2 (SV-AS03)	G2 (SV-PBS)	V1 (SV-AS03)	V1 (SV-PBS)	V2 (SV-AS03)	V2 (SV-PBS)	V1+V2 (SV-AS03)	V1+V2 (SV-PBS)
ENSG00000175643	C16orf75	-0.70	-0.08	-0.44	-0.37	49.3	0.6	19.6	13.5	69.0	14.1
ENSG00000165304	MELK	-0.59	0.58	-0.57	0.49	34.8	34.0	32.7	24.5	67.5	58.5
ENSG00000186185	KIF18B	-0.61	0.54	-0.55	-0.35	37.4	29.1	29.7	12.1	67.1	41.2
ENSG00000186638	KIF24	-0.62	0.60	-0.53	-0.42	38.2	36.4	28.2	17.7	66.4	54.1
ENSG00000179750	APOBEC3B	-0.47	0.15	-0.66	-0.20	22.2	2.1	43.5	3.9	65.7	6.0
ENSG00000090104	RGS1	0.17	-0.30	0.09	0.75	2.9	8.9	0.7	56.3	3.7	65.1
ENSG00000169679	BUB1	-0.32	0.18	-0.73	0.17	10.4	3.3	52.9	2.8	63.2	6.1
ENSG00000129195	FAM64A	-0.74	0.41	-0.29	-0.53	54.1	16.8	8.7	27.7	62.8	44.6
ENSG00000174371	EXO1	-0.64	0.54	-0.46	0.11	40.6	29.5	21.2	1.2	61.8	30.7
ENSG00000117724	CENPF	-0.47	0.51	-0.63	0.12	22.0	26.3	39.3	1.5	61.3	27.8
ENSG00000165480	SKA3	-0.58	-0.28	-0.52	0.65	33.7	7.8	26.8	42.3	60.5	50.1
ENSG00000175063	UBE2C	-0.41	0.35	-0.66	-0.42	16.8	12.3	43.3	18.0	60.1	30.3
ENSG00000186193	C9orf140	-0.56	-0.51	-0.53	0.32	31.5	25.7	28.4	10.5	59.9	36.1
ENSG00000256663	RP11-424C20.2	-0.64	0.40	-0.42	-0.05	40.6	16.3	17.6	0.3	58.2	16.6
ENSG00000182022	CHST15	0.55	-0.43	-0.52	0.03	30.5	18.8	26.6	0.1	57.0	18.9
ENSG00000136689	IL1RN	0.56	-0.48	-0.51	0.19	31.4	22.6	25.6	3.6	57.0	26.2
ENSG00000134531	EMP1	0.60	-0.55	-0.44	-0.43	36.4	30.6	19.4	18.4	55.8	49.0
ENSG00000169385	RNASE2	0.35	-0.65	-0.66	-0.17	12.3	42.8	43.2	2.7	55.4	45.6
ENSG00000211895	IGHA1	0.44	-0.70	-0.54	0.24	19.7	49.6	29.2	5.8	48.9	55.3
ENSG00000121621	KIF18A	-0.69	0.45	-0.12	0.12	48.2	20.2	1.3	1.4	49.6	21.7
ENSG00000172037	LAMB2	0.18	-0.15	-0.66	0.61	3.2	2.2	44.0	37.2	47.2	39.4
ENSG00000170345	FOS	0.23	-0.47	-0.10	0.48	5.1	22.3	1.0	23.3	6.1	45.5
ENSG00000126787	DLGAP5	-0.67	-0.40	-0.09	0.20	44.4	16.2	0.8	4.2	45.2	20.4
ENSG00000182240	BACE2	0.12	-0.64	-0.08	-0.20	1.4	40.9	0.6	4.0	2.0	44.9
ENSG00000137959	IFI44L	-0.49	0.46	-0.44	-0.09	23.8	21.3	19.5	0.7	43.3	22.0
ENSG00000112742	TTK	-0.58	0.41	-0.28	-0.27	33.4	16.5	7.7	7.3	41.0	23.8
ENSG00000224397	RP11-290F20.3	0.21	-0.21	-0.60	0.25	4.3	4.3	36.0	6.2	40.4	10.5
ENSG00000138778	CENPE	-0.33	0.47	-0.53	-0.11	11.2	22.6	27.8	1.2	39.0	23.7
ENSG00000115828	QPCT	0.10	-0.59	-0.44	-0.19	1.1	34.6	19.3	3.6	20.4	38.3
ENSG00000142945	KIF2C	-0.45	0.61	-0.41	-0.04	20.5	36.8	16.9	0.1	37.4	36.9
ENSG00000197249	SERPINA1	0.23	-0.38	-0.57	-0.12	5.2	14.3	32.0	1.4	37.2	15.7
ENSG00000184661	CDCA2	-0.33	0.48	-0.51	0.15	11.1	23.2	26.1	2.2	37.2	25.4
ENSG00000103832	RP5-1086D14.1	-0.23	0.02	-0.54	0.34	5.4	0.1	29.5	11.8	34.9	11.8
ENSG00000204103	MAFB	0.45	-0.22	-0.37	0.01	20.7	4.7	13.9	0.0	34.5	4.7
ENSG00000153234	NR4A2	-0.50	-0.42	-0.23	0.41	24.7	17.3	5.2	17.2	29.9	34.5
ENSG00000125740	FOSB	0.53	-0.31	0.25	0.38	27.8	9.4	6.0	14.2	33.8	23.5
ENSG00000166927	MS4A7	0.03	-0.38	-0.55	-0.19	0.1	14.3	30.7	3.7	30.8	18.0
ENSG00000117399	CDC20	-0.39	0.04	-0.37	-0.12	15.5	0.2	14.0	1.4	29.5	1.6
ENSG00000232487	RASA3-IT1	-0.02	0.01	-0.49	0.51	0.0	0.0	24.3	25.6	24.3	25.6
ENSG00000197405	C5AR1	0.24	-0.09	-0.44	0.06	5.8	0.8	19.7	0.4	25.5	1.2
ENSG00000174837	EMR1	-0.34	-0.40	-0.18	0.28	11.3	16.1	3.2	7.9	14.6	23.9
ENSG00000254873	RP11-770J1.5	0.29	0.29	-0.38	0.23	8.4	8.5	14.7	5.3	23.1	13.8
ENSG00000109321	AREG	-0.11	-0.02	0.05	0.46	1.2	0.0	0.3	20.8	1.4	20.8
ENSG00000167641	PPP1R14A	-0.42	-0.42	0.09	0.18	17.9	17.7	0.8	3.1	18.7	20.8
ENSG00000035499	DEPDC1B	-0.38	0.21	-0.15	-0.10	14.7	4.3	2.2	1.0	16.9	5.3
ENSG00000004799	PDK4	0.41	-0.05	0.02	-0.17	16.4	0.3	0.0	2.9	16.5	3.2
ENSG00000233392	AC104809.4	0.11	-0.05	0.23	0.36	1.2	0.2	5.1	13.2	6.3	13.4
ENSG00000162367	TAL1	0.26	-0.21	0.23	-0.30	6.8	4.2	5.3	9.2	12.0	13.4
ENSG00000183779	ZNF703	-0.22	0.11	-0.26	0.02	4.8	1.3	6.9	0.0	11.7	1.3
ENSG00000107954	NEURL	0.01	-0.13	-0.28	0.12	0.0	1.6	7.8	1.3	7.8	2.9

Gene ID	Gene Name	G1 (SV-AS03)	G1 (SV-PBS)	G2 (SV-AS03)	G2 (SV-PBS)	V1 (SV-AS03)	V1 (SV-PBS)	V2 (SV-AS03)	V2 (SV-PBS)	V1+V2 (SV-AS03)	V1+V2 (SV-PBS)
ENSG00000129757	CDKN1C	-0.08	-0.01	0.02	-0.22	0.6	0.0	0.0	4.8	0.6	4.8

Table A146: Canonical loadings and explained variance (RNA-Seq, NK-cells, Day 3, Gene variables). Gene variables with at least 25% of explained variance for the first two canonical variates are listed. GX: canonical loadings for canonical variate X, VX: % explained variance for canonical variate X, V1+V2: total % explained variance for both canonical variates. Values for CCA results with a poor cross validation score ($<$ 0.70) are left blank. Sorted by maximum explained variance across treatments (V1+V2).

IMO Variable	IMO Variable Name	I1 (SV-AS03)	I1 (SV-PBS)	I2 (SV-AS03)	I2 (SV-PBS)	V1 (SV-AS03)	V1 (SV-PBS)	V2 (SV-AS03)	V2 (SV-PBS)	V1+V2 (SV-AS03)	V1+V2 (SV-PBS)
IP-10	Interferon gamma-induced protein 10	-0.69	0.12	-0.62	0.88	47.3	1.6	38.3	76.9	85.7	78.4
TNF	Tumor necrosis factors	-0.52	0.91	0.20	-0.10	27.0	82.8	3.9	1.0	30.9	83.8
MIP-1α	Macrophage inflammatory protein	-0.38	0.29	0.42	0.85	14.3	8.1	17.7	73.0	32.0	81.1
MCP-1	Monocyte chemoattractant protein-1	0.55	-0.06	-0.03	0.89	29.7	0.3	0.1	79.4	29.8	79.7
IL-12p70	Interleukin-12 p70	-0.78	0.60	0.41	-0.33	61.1	35.8	17.1	11.1	78.2	46.8
IL-10	Interleukin-10	-0.73	0.86	0.17	-0.15	53.2	74.4	2.8	2.2	56.0	76.6
IFN-γ	Interferon gamma	0.04	0.71	0.81	-0.27	0.2	50.0	65.5	7.3	65.6	57.3
IL-1β	Interleukin-1 beta	-0.71	0.65	0.37	-0.32	50.7	42.4	13.6	10.3	64.3	52.7
IL-8	Interleukin-8	-0.56	0.72	0.41	0.08	31.1	51.5	16.6	0.6	47.7	52.1
RANTES	Chemokine (C-C motif) ligand 5	0.07	-0.24	-0.24	0.66	0.5	5.7	5.6	44.1	6.0	49.8
IL-6	Interleukin-6	-0.20	0.55	0.65	-0.18	4.0	30.6	42.6	3.1	46.6	33.7
Nt	Microneutralization inhibition	0.56	0.41	-0.00	0.06	31.6	17.0	0.0	0.4	31.6	17.4
Eotaxin-1	Eotaxin-1	0.04	0.10	-0.30	0.54	0.1	1.0	8.8	29.2	8.9	30.1

Table A147: Canonical loadings and explained variance (RNA-Seq, NK-cells, Day 3, IMO variables). IMO variables with at least 25% of explained variance for the first two canonical variates are listed. GX: canonical loadings for canonical variate X, VX: % explained variance for canonical variate X, V1+V2: total % explained variance for both canonical variates. Values for CCA results with a poor cross validation score ($<$ 0.70) are left blank. Sorted by maximum explained variance across treatments (V1+V2).

Gene ID	Gene Name	G1 (SV-AS03)	G1 (SV-PBS)	G2 (SV-AS03)	G2 (SV-PBS)	V1 (SV-AS03)	V1 (SV-PBS)	V2 (SV-AS03)	V2 (SV-PBS)	V1+V2 (SV-AS03)	V1+V2 (SV-PBS)
ENSG00000105967	TFEC		0.86		-0.46		74.7		20.9		95.6
ENSG00000121552	CSTA		0.86		-0.34		73.4		11.3		84.7
ENSG00000198053	SIRPA		0.84		-0.25		70.9		6.2		77.2
ENSG00000177575	CD163		0.81		-0.31		65.9		9.5		75.4
ENSG00000136826	KLF4		0.78		-0.36		61.1		13.3		74.4
ENSG00000198478	SH3BGRL2		-0.29		-0.80		8.5		63.7		72.2
ENSG00000136689	IL1RN		0.72		-0.42		51.6		17.8		69.3
ENSG00000074964	ARHGEF10L		0.81		-0.17		65.6		2.8		68.4
ENSG00000049323	LTBP1		0.75		-0.31		56.1		9.7		65.8
ENSG00000029534	ANK1		0.75		0.13		56.6		1.6		58.3
ENSG00000103832	RP5-1086D14.1		0.74		0.04		54.8		0.2		55.0
ENSG00000180071	ANKRD18A		-0.08		0.72		0.6		52.4		53.1
ENSG00000169385	RNASE2		0.68		-0.23		46.0		5.3		51.3
ENSG00000224397	RP11-290F20.3		0.07		-0.64		0.5		40.3		40.8
ENSG00000184060	ADAP2		0.54		-0.17		28.8		2.8		31.6
ENSG00000206172	HBA1		0.26		-0.49		6.8		23.9		30.7

Gene ID	Gene Name	G1 (SV-AS03)	G1 (SV-PBS)	G2 (SV-AS03)	G2 (SV-PBS)	V1 (SV-AS03)	V1 (SV-PBS)	V2 (SV-AS03)	V2 (SV-PBS)	V1+V2 (SV-AS03)	V1+V2 (SV-PBS)
ENSG00000134531	EMP1		0.36		-0.41		12.8		17.1		29.9
ENSG00000117399	CDC20		-0.51		-0.19		25.8		3.7		29.6
ENSG00000211895	IGHA1		-0.38		-0.38		14.8		14.4		29.2
ENSG00000244734	HBB		-0.07		-0.53		0.5		28.5		29.0
ENSG00000188536	HBA2		-0.03		-0.50		0.1		25.5		25.5
ENSG00000236304	AP001189.4		-0.05		-0.50		0.2		25.1		25.3
ENSG00000211899	IGHM		0.05		-0.50		0.3		24.8		25.1
ENSG00000126787	DLGAP5		-0.08		-0.49		0.6		24.4		25.1
ENSG00000157456	CCNB2		-0.32		-0.31		10.0		9.5		19.5
ENSG00000138180	CEP55		0.39		-0.06		14.9		0.4		15.2
ENSG00000165480	SKA3		0.37		-0.04		13.5		0.2		13.7
ENSG00000169679	BUB1		-0.01		0.34		0.0		11.8		11.8
ENSG00000185736	ADARB2		-0.20		0.28		3.9		7.7		11.7
ENSG00000233392	AC104809.4		0.22		-0.19		5.0		3.6		8.6
ENSG00000168913	ENHO		-0.27		0.11		7.3		1.2		8.5
ENSG00000125740	FOSB		-0.02		-0.19		0.0		3.7		3.7
ENSG00000225556	C2CD4D		0.12		0.05		1.4		0.3		1.7

Table A148: Canonical loadings and explained variance (RNA-Seq, NK-cells, Day 7, Gene variables). Gene variables with at least 25% of explained variance for the first two canonical variates are listed. GX: canonical loadings for canonical variate X, VX: % explained variance for canonical variate X, V1+V2: total % explained variance for both canonical variates. Values for CCA results with a poor cross validation score (< 0.70) are left blank. Sorted by maximum explained variance across treatments (V1+V2).

IMO Variable	IMO Variable Name	I1 (SV-AS03)	I1 (SV-PBS)	I2 (SV-AS03)	I2 (SV-PBS)	V1 (SV-AS03)	V1 (SV-PBS)	V2 (SV-AS03)	V2 (SV-PBS)	V1+V2 (SV-AS03)	V1+V2 (SV-PBS)
MIP-1α	Macrophage inflammatory protein		0.87		0.47		75.2		22.3		97.6
IP-10	Interferon gamma-induced protein 10		0.95		0.03		90.4		0.1		90.4
IL-1β	Interleukin-1 beta		-0.80		-0.04		64.1		0.2		64.3
MCP-1	Monocyte chemoattractant protein-1		0.75		0.19		55.6		3.5		59.1
TNF	Tumor necrosis factors		-0.38		0.62		14.3		38.0		52.3
RANTES	Chemokine (C-C motif) ligand 5		0.24		-0.67		5.7		44.8		50.6
Eotaxin-1	Eotaxin-1		-0.68		-0.16		45.9		2.6		48.5
Nt	Microneutralization inhibition		0.36		0.59		13.1		34.5		47.6
IL-8	Interleukin-8		0.00		0.68		0.0		46.3		46.3
IL-6	Interleukin-6		-0.42		0.54		17.3		28.9		46.2
IFN-γ	Interferon gamma		-0.38		0.55		14.7		30.6		45.2
IL-12p70	Interleukin-12 p70		-0.48		-0.33		23.0		10.9		33.8
IL-10	Interleukin-10		-0.04		-0.00		0.1		0.0		0.1

Table A149: Canonical loadings and explained variance (RNA-Seq, NK-cells, Day 7, IMO variables). IMO variables with at least 25% of explained variance for the first two canonical variates are listed. GX: canonical loadings for canonical variate X, VX: % explained variance for canonical variate X, V1+V2: total % explained variance for both canonical variates. Values for CCA results with a poor cross validation score (< 0.70) are left blank. Sorted by maximum explained variance across treatments (V1+V2).

Gene ID	Gene Name	G1 (SV-AS03)	G1 (SV-PBS)	G2 (SV-AS03)	G2 (SV-PBS)	V1 (SV-AS03)	V1 (SV-PBS)	V2 (SV-AS03)	V2 (SV-PBS)	V1+V2 (SV-AS03)	V1+V2 (SV-PBS)
ENSG00000110446	SLC15A3	0.90		0.02		81.5		0.0		81.6	
ENSG00000179467	AL109806.1	0.51		-0.74		26.1		54.6		80.7	
ENSG00000087253	LPCAT2	0.88		-0.05		77.7		0.3		78.0	
ENSG00000242498	C15orf38	0.88		-0.05		77.7		0.3		78.0	
ENSG00000162882	HAAO	0.76		-0.43		57.1		18.5		75.6	
ENSG00000153162	BMP6	-0.86		-0.05		74.6		0.3		74.8	
ENSG00000169403	PTAFR	0.78		0.36		61.5		13.3		74.8	
ENSG00000175538	KCNE3	0.81		0.31		65.1		9.6		74.6	
ENSG00000136205	TNS3	0.75		0.42		56.7		17.6		74.3	
ENSG00000002933	TMEM176A	0.68		0.53		45.8		28.0		73.9	
ENSG00000171777	RASGRP4	0.86		0.02		73.4		0.0		73.4	
ENSG00000184113	CLDN5	-0.82		-0.23		67.6		5.4		73.0	
ENSG00000135218	CD36	0.84		-0.11		71.2		1.3		72.4	
ENSG00000106565	TMEM176B	0.81		0.23		66.0		5.5		71.4	
ENSG00000131042	LILRB2	0.79		0.31		61.9		9.4		71.3	
ENSG00000100095	SEZ6L	0.83		0.03		69.2		0.1		69.3	
ENSG00000188906	LRRK2	0.82		-0.13		67.4		1.8		69.2	
ENSG00000161642	ZNF385A	0.83		-0.05		68.2		0.3		68.4	
ENSG00000111452	GPR133	0.61		0.56		36.6		31.7		68.3	
ENSG00000161944	ASGR2	0.82		0.04		68.0		0.1		68.2	
ENSG00000074964	ARHGEF10L	0.82		-0.03		67.5		0.1		67.6	
ENSG00000127954	STEAP4	0.68		-0.45		46.6		20.6		67.2	
ENSG00000163694	RBM47	0.80		0.17		64.3		2.9		67.1	
ENSG00000060982	BCAT1	0.81		-0.07		66.1		0.4		66.5	
ENSG00000103196	CRISPLD2	0.67		0.46		44.9		21.6		66.5	
ENSG00000173334	TRIB1	0.75		0.31		56.7		9.6		66.3	
ENSG00000103313	MEFV	0.81		0.06		65.6		0.3		65.9	
ENSG00000123384	LRP1	0.80		0.12		64.4		1.4		65.8	
ENSG00000198053	SIRPA	0.81		0.05		65.4		0.2		65.7	
ENSG00000033627	ATP6V0A1	0.80		0.10		64.3		1.1		65.4	
ENSG00000132694	ARHGEF11	0.80		-0.07		63.9		0.5		64.5	
ENSG00000038427	VCAN	0.80		-0.03		63.9		0.1		64.0	
ENSG00000127920	GNG11	-0.77		-0.21		59.4		4.4		63.8	
ENSG00000129226	CD68	0.78		0.18		60.4		3.2		63.6	
ENSG00000215458	AP001053.11	0.79		0.13		61.8		1.7		63.5	
ENSG00000119535	CSF3R	0.79		0.07		62.6		0.5		63.1	
ENSG00000177706	FAM20C	0.77		0.19		59.5		3.6		63.1	
ENSG00000186407	CD300E	0.79		-0.08		62.1		0.7		62.8	
ENSG00000116962	NID1	0.77		-0.18		59.6		3.2		62.7	
ENSG00000187796	CARD9	0.79		0.01		61.9		0.0		61.9	
ENSG00000198502	HLA-DRB5	0.78		0.08		61.2		0.7		61.8	
ENSG00000151650	VENTX	0.73		-0.28		53.7		7.8		61.6	
ENSG00000250510	GPR162	0.74		-0.27		54.3		7.0		61.4	
ENSG00000178695	KCTD12	0.78		0.04		61.2		0.2		61.3	
ENSG00000110077	MS4A6A	0.78		-0.10		60.1		0.9		61.1	
ENSG00000153823	PID1	0.74		-0.24		54.7		6.0		60.7	
ENSG00000198598	MMP17	0.75		0.19		57.0		3.6		60.6	
ENSG00000105352	CEACAM4	0.67		-0.39		44.8		15.4		60.2	
ENSG00000182578	CSF1R	0.77		-0.05		59.8		0.3		60.1	
ENSG00000196189	SEMA4A	0.77		-0.03		60.0		0.1		60.0	
ENSG00000153885	KCTD15	0.56		-0.54		31.1		28.7		59.8	

Gene ID	Gene Name	G1 (SV-AS03)	G1 (SV-PBS)	G2 (SV-AS03)	G2 (SV-PBS)	V1 (SV-AS03)	V1 (SV-PBS)	V2 (SV-AS03)	V2 (SV-PBS)	V1+V2 (SV-AS03)	V1+V2 (SV-PBS)
ENSG00000100079	LGALS2	0.76		-0.14		57.9		1.9		59.8	
ENSG00000085265	FCN1	0.77		0.03		59.6		0.1		59.7	
ENSG00000159399	HK2	0.75		0.16		56.9		2.7		59.6	
ENSG00000116701	NCF2	0.77		-0.03		59.3		0.1		59.4	
ENSG00000196562	SULF2	0.77		0.01		59.2		0.0		59.3	
ENSG00000216490	IFI30	0.77		0.03		59.1		0.1		59.3	
ENSG00000166145	SPINT1	0.76		0.14		57.2		2.0		59.2	
ENSG00000235568	NFAM1	0.76		0.09		57.9		0.8		58.6	
ENSG00000131398	KCNC3	0.75		-0.13		56.9		1.7		58.6	
ENSG00000139132	FGD4	0.70		-0.31		48.4		9.7		58.2	
ENSG00000167600	CYP2S1	0.76		-0.02		57.9		0.0		57.9	
ENSG00000106066	CPVL	0.75		-0.14		55.5		2.1		57.6	
ENSG00000079308	TNS1	-0.74		-0.17		54.4		3.0		57.5	
ENSG00000172243	CLEC7A	0.76		-0.01		57.3		0.0		57.3	
ENSG00000197249	SERPINA1	0.75		0.05		56.9		0.3		57.2	
ENSG00000170909	OSCAR	0.73		0.18		53.8		3.1		56.9	
ENSG00000244482	LILRA6	0.75		0.06		56.4		0.3		56.7	
ENSG00000214212	C19orf38	0.75		-0.09		55.7		0.8		56.4	
ENSG00000181631	P2RY13	0.75		-0.06		55.9		0.4		56.3	
ENSG00000109113	RAB34	0.75		-0.05		55.5		0.2		55.7	
ENSG00000185215	TNFAIP2	0.73		-0.15		53.3		2.3		55.6	
ENSG00000010327	STAB1	0.72		0.19		52.0		3.4		55.5	
ENSG00000166927	MS4A7	0.73		-0.12		53.6		1.6		55.2	
ENSG00000169385	RNASE2	0.73		0.12		53.4		1.4		54.8	
ENSG00000177575	CD163	0.74		-0.06		54.4		0.3		54.7	
ENSG00000163563	MNDA	0.73		0.12		53.3		1.5		54.7	
ENSG00000164125	FAM198B	0.74		0.01		54.6		0.0		54.6	
ENSG00000120708	TGFBI	0.73		0.09		53.6		0.9		54.4	
ENSG00000010610	CD4	0.73		0.12		52.9		1.4		54.3	
ENSG00000165801	ARHGEF40	0.71		-0.21		49.9		4.3		54.2	
ENSG00000101336	HCK	0.70		-0.22		49.1		4.9		54.0	
ENSG00000088827	SIGLEC1	0.73		-0.06		53.6		0.4		53.9	
ENSG00000066336	SPI1	0.73		0.02		53.7		0.0		53.8	
ENSG00000146592	CREB5	0.71		-0.17		50.8		2.7		53.6	
ENSG00000137462	TLR2	0.73		-0.02		53.4		0.0		53.4	
ENSG00000122025	FLT3	0.64		-0.35		40.9		12.4		53.2	
ENSG00000196576	PLXNB2	0.72		0.10		52.1		0.9		53.1	
ENSG00000204472	AIF1	0.73		0.04		52.9		0.1		53.0	
ENSG00000160712	IL6R	0.72		-0.09		52.1		0.7		52.9	
ENSG00000158517	NCF1	0.49		0.54		24.0		28.8		52.8	
ENSG00000064225	ST3GAL6	0.45		-0.57		20.6		32.2		52.8	
ENSG00000008394	MGST1	0.70		-0.17		49.6		3.0		52.6	
ENSG00000187800	PEAR1	-0.67		-0.26		45.3		6.9		52.2	
ENSG00000196126	HLA-DRB1	0.72		-0.07		51.7		0.5		52.2	
ENSG00000121316	PLBD1	0.72		-0.06		51.6		0.4		52.0	
ENSG00000139970	RTN1	0.72		-0.05		51.2		0.2		51.4	
ENSG00000116990	MYCL1	0.69		0.19		47.7		3.6		51.3	
ENSG00000111729	CLEC4A	0.70		-0.13		49.3		1.8		51.1	
ENSG00000163625	WDFY3	0.71		0.10		49.9		1.0		50.9	
ENSG00000198734	F5	0.71		0.09		50.1		0.8		50.9	
ENSG00000205730	ITPRIPL2	0.70		0.13		49.1		1.7		50.8	

Gene ID	Gene Name	G1 (SV-AS03)	G1 (SV-PBS)	G2 (SV-AS03)	G2 (SV-PBS)	V1 (SV-AS03)	V1 (SV-PBS)	V2 (SV-AS03)	V2 (SV-PBS)	V1+V2 (SV-AS03)	V1+V2 (SV-PBS)
ENSG00000106034	C7orf58	0.71		-0.04		50.5		0.2		50.7	
ENSG00000101439	CST3	0.71		-0.02		50.6		0.0		50.7	
ENSG00000119862	AC008074.1	-0.69		-0.17		47.8		2.8		50.6	
ENSG00000170458	CD14	0.70		0.10		49.6		0.9		50.5	
ENSG00000171105	INSR	0.65		-0.28		42.4		8.1		50.5	
ENSG00000140044	JDP2	0.64		0.31		40.6		9.8		50.4	
ENSG00000135363	LMO2	0.70		-0.10		49.3		1.1		50.4	
ENSG00000105967	TFEC	0.71		-0.02		50.3		0.0		50.4	
ENSG00000179104	TMTC2	0.61		0.36		37.1		13.0		50.1	
ENSG00000134243	SORT1	0.70		-0.10		48.5		1.0		49.5	
ENSG00000155465	SLC7A7	0.69		0.11		48.1		1.3		49.5	
ENSG00000088726	TMEM40	-0.70		-0.09		48.6		0.9		49.4	
ENSG00000158481	CD1C	0.54		-0.45		29.1		20.1		49.2	
ENSG00000114013	CD86	0.70		0.05		48.9		0.2		49.1	
ENSG00000196209	SIRPB2	0.67		-0.22		44.4		4.7		49.1	
ENSG00000136830	FAM129B	0.69		0.09		48.2		0.8		49.0	
ENSG00000143546	S100A8	0.69		-0.07		48.1		0.5		48.6	
ENSG00000115828	QPCT	0.59		0.37		34.3		14.0		48.4	
ENSG00000104974	LILRA1	0.69		-0.04		48.2		0.1		48.3	
ENSG00000146192	FGD2	0.67		-0.15		45.0		2.2		47.1	
ENSG00000163737	PF4	-0.64		-0.24		41.6		5.5		47.1	
ENSG00000163220	S100A9	0.68		0.03		46.7		0.1		46.9	
ENSG00000085733	CTTN	-0.66		-0.18		43.5		3.3		46.8	
ENSG00000109743	BST1	0.66		0.17		44.0		2.7		46.7	
ENSG00000103811	CTSH	0.67		0.12		45.2		1.5		46.7	
ENSG00000090382	LYZ	0.68		-0.03		46.5		0.1		46.6	
ENSG00000012779	ALOX5	0.68		-0.03		46.4		0.1		46.5	
ENSG00000111275	ALDH2	0.68		0.08		45.7		0.7		46.4	
ENSG00000175489	LRRC25	0.68		0.06		45.8		0.3		46.1	
ENSG00000184702	SEPT5	-0.67		-0.11		44.8		1.3		46.1	
ENSG00000138119	MYOF	0.66		0.14		44.1		2.0		46.1	
ENSG00000100365	NCF4	0.56		0.38		31.3		14.7		46.0	
ENSG00000158473	CD1D	0.67		-0.06		45.5		0.4		45.9	
ENSG00000160593	AMICA1	0.62		-0.24		38.9		6.0		44.9	
ENSG00000168497	SDPR	-0.65		-0.17		42.1		2.8		44.9	
ENSG00000006534	ALDH3B1	0.67		0.05		44.4		0.3		44.7	
ENSG00000184060	ADAP2	0.51		0.43		25.9		18.7		44.6	
ENSG00000104870	FCGRT	0.67		0.04		44.2		0.1		44.4	
ENSG00000124762	CDKN1A	-0.66		-0.08		43.7		0.6		44.3	
ENSG00000132205	EMILIN2	0.65		0.13		42.3		1.7		44.0	
ENSG00000154611	PSMA8	-0.48		-0.46		22.8		21.1		43.8	
ENSG00000140749	IGSF6	0.64		0.17		40.8		2.8		43.6	
ENSG00000121552	CSTA	0.64		-0.16		41.0		2.5		43.5	
ENSG00000136869	TLR4	0.66		-0.06		43.0		0.3		43.4	
ENSG00000101162	TUBB1	-0.65		-0.11		42.0		1.3		43.3	
ENSG00000159128	IFNGR2	0.64		-0.15		41.1		2.2		43.3	
ENSG00000170017	ALCAM	0.65		-0.06		42.5		0.4		42.9	
ENSG00000035862	TIMP2	0.65		-0.08		42.3		0.6		42.9	
ENSG00000134955	SLC37A2	0.64		-0.13		41.1		1.7		42.8	
ENSG00000109436	TBC1D9	0.65		0.08		42.0		0.6		42.6	
ENSG00000140090	SLC24A4	0.54		-0.37		28.8		13.7		42.5	

Gene ID	Gene Name	G1 (SV-AS03)	G1 (SV-PBS)	G2 (SV-AS03)	G2 (SV-PBS)	V1 (SV-AS03)	V1 (SV-PBS)	V2 (SV-AS03)	V2 (SV-PBS)	V1+V2 (SV-AS03)	V1+V2 (SV-PBS)
ENSG00000149564	ESAM	-0.64		-0.12		41.0		1.5		42.5	
ENSG00000197629	MPEG1	0.65		0.09		41.6		0.9		42.5	
ENSG00000049323	LTBP1	-0.64		-0.12		40.9		1.4		42.3	
ENSG00000100077	ADRBK2	0.63		-0.16		39.7		2.5		42.2	
ENSG00000187164	KIAA1598	0.65		-0.03		42.0		0.1		42.1	
ENSG00000186074	CD300LF	0.64		0.13		40.5		1.6		42.1	
ENSG00000160883	HK3	0.64		0.09		41.3		0.7		42.0	
ENSG00000198478	SH3BGRL2	-0.64		-0.12		40.5		1.4		41.9	
ENSG00000168389	MFSD2A	0.64		-0.12		40.4		1.5		41.9	
ENSG00000167207	NOD2	0.63		0.12		40.2		1.3		41.5	
ENSG00000239998	LILRA2	0.59		-0.24		35.3		5.8		41.1	
ENSG00000018280	SLC11A1	0.63		0.12		39.6		1.4		41.1	
ENSG00000171611	PTCRA	-0.61		-0.20		37.0		4.0		41.0	
ENSG00000029534	ANK1	-0.61		-0.20		36.8		4.2		41.0	
ENSG00000163736	PPBP	-0.62		-0.17		38.1		2.8		40.9	
ENSG00000014914	MTMR11	0.64		0.04		40.6		0.1		40.8	
ENSG00000105383	CD33	0.63		-0.09		39.7		0.8		40.5	
ENSG00000155254	MARVELD1	0.64		0.01		40.3		0.0		40.3	
ENSG00000169704	GP9	-0.62		-0.12		38.8		1.5		40.3	
ENSG00000114812	VIPR1	0.62		-0.13		38.7		1.6		40.3	
ENSG00000187116	LILRA5	0.63		-0.04		39.1		0.1		39.2	
ENSG00000163803	PLB1	0.62		0.09		38.3		0.9		39.1	
ENSG00000163221	S100A12	0.62		0.06		38.7		0.4		39.1	
ENSG00000053918	KCNQ1	0.62		0.09		38.0		0.8		38.8	
ENSG00000117115	PADI2	0.62		0.03		38.5		0.1		38.5	
ENSG00000133246	PRAM1	0.57		-0.24		32.8		5.6		38.4	
ENSG00000204103	MAFB	0.49		0.38		23.6		14.8		38.4	
ENSG00000171051	FPR1	0.50		0.36		25.4		12.9		38.3	
ENSG00000145936	KCNMB1	0.02		0.62		0.0		38.3		38.3	
ENSG00000186818	LILRB4	0.62		-0.02		37.9		0.0		38.0	
ENSG00000132514	CLEC10A	0.61		-0.07		37.4		0.5		37.9	
ENSG00000183023	SLC8A1	0.50		0.36		25.0		12.8		37.9	
ENSG00000157240	FZD1	0.61		-0.00		37.6		0.0		37.6	
ENSG00000157445	CACNA2D3	0.52		-0.32		26.8		10.3		37.1	
ENSG00000180340	FZD2	0.61		0.04		36.8		0.1		36.9	
ENSG00000113140	SPARC	-0.60		-0.12		35.5		1.4		36.9	
ENSG00000168913	ENHO	0.53		0.30		28.0		8.8		36.8	
ENSG00000188536	HBA2	-0.51		-0.32		26.5		10.0		36.5	
ENSG00000229754	CXCR2P1	-0.57		-0.19		32.5		3.7		36.2	
ENSG00000198848	CES1	0.60		-0.04		35.9		0.2		36.0	
ENSG00000120885	CLU	-0.58		-0.13		34.2		1.7		35.9	
ENSG00000075223	SEMA3C	0.56		-0.22		30.9		4.7		35.6	
ENSG00000172322	CLEC12A	0.59		-0.01		35.1		0.0		35.2	
ENSG00000154237	LRRK1	0.56		0.20		31.2		3.9		35.1	
ENSG00000113749	HRH2	0.59		0.06		34.6		0.3		34.9	
ENSG00000206172	HBA1	-0.44		-0.39		19.7		15.0		34.7	
ENSG00000164181	ELOVL7	-0.58		0.04		34.2		0.2		34.4	
ENSG00000244734	HBB	-0.55		-0.21		30.0		4.3		34.4	
ENSG00000138678	AGPAT9	0.53		-0.25		27.7		6.4		34.2	
ENSG00000187554	TLR5	0.57		0.13		32.3		1.8		34.1	
ENSG00000112414	GPR126	0.52		-0.26		27.0		6.5		33.5	

Gene ID	Gene Name	G1 (SV-AS03)	G1 (SV-PBS)	G2 (SV-AS03)	G2 (SV-PBS)	V1 (SV-AS03)	V1 (SV-PBS)	V2 (SV-AS03)	V2 (SV-PBS)	V1+V2 (SV-AS03)	V1+V2 (SV-PBS)
ENSG00000165178	NCF1C	0.40		0.41		16.3		17.2		33.5	
ENSG00000204287	HLA-DRA	0.55		-0.18		30.2		3.2		33.4	
ENSG00000136630	HLX	0.17		-0.55		3.0		30.1		33.1	
ENSG00000101307	SIRPB1	0.57		0.03		32.9		0.1		33.0	
ENSG00000254415	SIGLEC14	0.40		-0.40		15.7		16.2		31.8	
ENSG00000166825	ANPEP	0.56		-0.03		31.7		0.1		31.8	
ENSG00000108846	ABCC3	-0.55		-0.11		30.5		1.2		31.7	
ENSG00000115919	KYNU	0.53		-0.16		28.4		2.5		31.0	
ENSG00000154146	NRGN	-0.52		-0.18		27.1		3.4		30.4	
ENSG00000182022	CHST15	0.51		0.21		25.9		4.4		30.3	
ENSG00000164023	SGMS2	0.49		-0.25		23.8		6.3		30.1	
ENSG00000173530	TNFRSF10D	0.54		-0.09		29.2		0.8		30.0	
ENSG00000011105	TSPAN9	-0.52		-0.17		27.1		2.9		30.0	
ENSG00000014257	ACPP	0.51		-0.20		25.7		3.8		29.5	
ENSG00000101335	MYL9	-0.53		-0.10		28.2		1.1		29.3	
ENSG00000142552	RCN3	0.54		0.03		28.9		0.1		29.0	
ENSG00000161911	TREML1	-0.44		-0.31		18.9		9.8		28.8	
ENSG00000104267	CA2	-0.48		-0.22		23.4		5.0		28.4	
ENSG00000174837	EMR1	0.23		0.47		5.4		22.5		27.9	
ENSG00000185340	GAS2L1	-0.45		-0.28		19.9		7.9		27.8	
ENSG00000005961	ITGA2B	-0.52		-0.04		27.5		0.2		27.7	
ENSG00000160013	PTGIR	-0.37		-0.37		13.5		13.8		27.3	
ENSG00000173210	ABLIM3	-0.39		-0.35		15.2		12.1		27.2	
ENSG00000112799	LY86	0.51		-0.12		25.5		1.4		26.9	
ENSG00000134256	CD101	0.48		0.19		23.2		3.8		26.9	
ENSG00000075618	FSCN1	0.51		-0.11		25.7		1.2		26.8	
ENSG00000082781	ITGB5	-0.51		-0.07		26.2		0.5		26.7	
ENSG00000251060	U66061.31	-0.52		-0.02		26.6		0.1		26.7	
ENSG00000136689	IL1RN	0.51		-0.03		26.3		0.1		26.4	
ENSG00000020577	SAMD4A	0.27		0.44		7.1		19.1		26.2	
ENSG00000154928	EPHB1	-0.09		0.50		0.7		25.3		26.1	
ENSG00000136826	KLF4	0.39		0.33		15.0		11.1		26.1	
ENSG00000118515	SGK1	0.34		0.38		11.4		14.6		25.9	
ENSG00000114923	SLC4A3	0.37		-0.35		13.4		12.5		25.9	
ENSG00000182487	NCF1B	0.07		0.50		0.5		25.3		25.8	
ENSG00000105501	SIGLEC5	0.47		0.20		21.7		4.1		25.7	
ENSG00000185758	CLDN24	0.31		-0.39		9.9		15.2		25.1	
ENSG00000144677	CTDSPL	-0.48		-0.12		23.1		1.4		24.5	
ENSG00000120738	EGR1	-0.41		0.27		17.1		7.2		24.3	
ENSG00000172037	LAMB2	0.44		-0.21		19.7		4.4		24.1	
ENSG00000100504	PYGL	0.48		-0.09		23.0		0.8		23.8	
ENSG00000159339	PADI4	0.47		0.11		22.4		1.2		23.6	
ENSG00000136929	HEMGN	-0.41		-0.26		16.6		6.5		23.1	
ENSG00000166428	PLD4	0.47		0.04		22.0		0.1		22.2	
ENSG00000121807	CCR2	0.47		0.05		21.7		0.3		22.0	
ENSG00000224888	RP5-1142A6.2	0.35		-0.31		12.5		9.3		21.8	
ENSG00000134531	EMP1	0.39		-0.24		15.5		5.9		21.5	
ENSG00000255197	RP11-750H9.5	0.46		-0.02		21.3		0.0		21.3	
ENSG00000146094	DOK3	0.45		-0.06		19.8		0.3		20.2	
ENSG00000134061	CD180	0.45		-0.03		19.9		0.1		20.0	
ENSG00000103832	RP5-1086D14.1	-0.32		-0.31		10.1		9.6		19.7	

Gene ID	Gene Name	G1 (SV-AS03)	G1 (SV-PBS)	G2 (SV-AS03)	G2 (SV-PBS)	V1 (SV-AS03)	V1 (SV-PBS)	V2 (SV-AS03)	V2 (SV-PBS)	V1+V2 (SV-AS03)	V1+V2 (SV-PBS)
ENSG00000203710	CR1	0.23		0.37		5.3		13.4		18.8	
ENSG00000115271	GCA	0.43		-0.01		18.3		0.0		18.3	
ENSG00000196735	HLA-DQA1	0.42		0.07		17.5		0.4		17.9	
ENSG00000167642	SPINT2	0.33		-0.27		10.7		7.2		17.9	
ENSG00000173262	SLC2A14	-0.25		0.33		6.3		10.7		17.0	
ENSG00000131401	NAPSB	0.39		-0.11		15.1		1.2		16.3	
ENSG00000179344	HLA-DQB1	0.33		0.23		10.7		5.3		15.9	
ENSG00000142627	EPHA2	-0.34		0.19		11.4		3.7		15.1	
ENSG00000171812	COL8A2	0.38		-0.05		14.7		0.3		15.0	
ENSG00000104783	KCNN4	0.04		0.38		0.1		14.4		14.5	
ENSG00000111424	VDR	0.31		0.20		9.9		4.0		13.9	
ENSG00000179639	FCER1A	0.30		-0.22		8.7		4.9		13.6	
ENSG00000082397	EPB41L3	0.32		-0.16		10.5		2.7		13.2	
ENSG00000155367	PPM1J	-0.36		-0.03		13.1		0.1		13.2	
ENSG00000184602	SNN	-0.35		-0.09		12.1		0.9		13.0	
ENSG00000138449	SLC40A1	-0.32		-0.12		10.1		1.5		11.7	
ENSG00000165959	CLMN	0.24		-0.24		5.9		5.8		11.7	
ENSG00000149418	ST14	0.34		-0.04		11.4		0.1		11.5	
ENSG00000173801	JUP	0.31		0.10		9.6		0.9		10.6	
ENSG00000120875	DUSP4	0.23		-0.23		5.1		5.3		10.4	
ENSG00000168461	RAB31	0.29		-0.10		8.6		1.0		9.6	
ENSG00000127831	VIL1	-0.30		-0.05		9.3		0.2		9.5	
ENSG00000119866	BCL11A	0.29		0.08		8.5		0.7		9.2	
ENSG00000178038	ALS2CL	0.21		0.20		4.2		4.0		8.2	
ENSG00000129993	CBFA2T3	0.27		-0.08		7.5		0.7		8.1	
ENSG00000197766	CFD	-0.14		0.24		1.9		5.6		7.5	
ENSG00000123405	NFE2	-0.25		-0.06		6.4		0.3		6.7	
ENSG00000175857	GAPT	0.24		0.05		5.8		0.2		6.0	
ENSG00000141447	OSBPL1A	0.10		0.21		1.0		4.2		5.2	
ENSG00000124491	F13A1	-0.22		-0.02		4.8		0.0		4.8	
ENSG00000072422	RHOBTB1	-0.21		-0.04		4.5		0.2		4.7	
ENSG00000205639	MFSD2B	-0.09		0.19		0.8		3.7		4.5	
ENSG00000118508	RAB32	0.15		-0.15		2.2		2.2		4.4	
ENSG00000255833	TIFAB	-0.17		-0.11		3.0		1.2		4.2	
ENSG00000108405	P2RX1	-0.17		0.08		2.9		0.7		3.7	
ENSG00000140287	HDC	-0.17		-0.07		2.9		0.4		3.3	
ENSG00000060138	CSDA	0.14		-0.11		1.9		1.1		3.0	
ENSG00000237541	HLA-DQA2	0.15		0.08		2.2		0.7		2.9	
ENSG00000004799	PDK4	-0.04		0.07		0.2		0.5		0.6	

Table A150: Canonical loadings and explained variance (RNA-Seq, NK-cells, Day 28, Gene variables). Gene variables with at least 25% of explained variance for the first two canonical variates are listed. GX: canonical loadings for canonical variate X, VX: % explained variance for canonical variate X, V1+V2: total % explained variance for both canonical variates. Values for CCA results with a poor cross validation score ($<$ 0.70) are left blank. Sorted by maximum explained variance across treatments (V1+V2).

IMO Variable	IMO Variable Name	I1 (SV-AS03)	I1 (SV-PBS)	I2 (SV-AS03)	I2 (SV-PBS)	V1 (SV-AS03)	V1 (SV-PBS)	V2 (SV-AS03)	V2 (SV-PBS)	V1+V2 (SV-AS03)	V1+V2 (SV-PBS)
IL-12p70	Interleukin-12 p70	-0.93		0.30		86.7		9.1		95.7	

IMO Variable	IMO Variable Name	I1 (SV-AS03)	I1 (SV-PBS)	I2 (SV-AS03)	I2 (SV-PBS)	V1 (SV-AS03)	V1 (SV-PBS)	V2 (SV-AS03)	V2 (SV-PBS)	V1+V2 (SV-AS03)	V1+V2 (SV-PBS)
Nt	Microneutralization inhibition	0.70		0.61		49.5		37.4		86.8	
HAI	Hemagglutination inhibition	0.64		0.66		40.7		44.0		84.7	
Eotaxin-1	Eotaxin-1	-0.22		0.85		4.8		71.5		76.3	
IL-6	Interleukin-6	-0.82		-0.18		66.9		3.4		70.3	
IL-10	Interleukin-10	-0.28		0.79		8.0		61.9		70.0	
IL-8	Interleukin-8	-0.38		0.74		14.4		54.3		68.6	
IP-10	Interferon gamma-induced protein 10	0.55		0.50		30.1		24.5		54.6	
TNF	Tumor necrosis factors	-0.45		0.53		19.9		27.7		47.6	
MCP-1	Monocyte chemoattractant protein-1	-0.48		0.40		23.4		15.8		39.2	
RANTES	Chemokine (C-C motif) ligand 5	-0.34		0.52		11.3		27.4		38.7	
IL-1β	Interleukin-1 beta	-0.37		0.33		13.6		11.2		24.8	
MIP-1α	Macrophage inflammatory protein	-0.11		-0.48		1.2		23.0		24.2	
IFN-γ	Interferon gamma	0.26		0.32		6.9		10.4		17.3	

Table A151: Canonical loadings and explained variance (RNA-Seq, NK-cells, Day 28, IMO variables). IMO variables with at least 25% of explained variance for the first two canonical variates are listed. GX: canonical loadings for canonical variate X, VX: % explained variance for canonical variate X, V1+V2: total % explained variance for both canonical variates. Values for CCA results with a poor cross validation score (< 0.70) are left blank. Sorted by maximum explained variance across treatments (V1+V2).

Gene ID	Gene Name	G1 (SV-AS03)	G1 (SV-PBS)	G2 (SV-AS03)	G2 (SV-PBS)	V1 (SV-AS03)	V1 (SV-PBS)	V2 (SV-AS03)	V2 (SV-PBS)	V1+V2 (SV-AS03)	V1+V2 (SV-PBS)
ENSG00000163563	MNDA	0.62		0.77		38.2		59.4		97.5	
ENSG00000117228	GBP1	-0.85		0.49		72.6		23.8		96.3	
ENSG00000156587	UBE2L6	-0.78		0.60		60.1		35.9		96.1	
ENSG00000025708	TYMP	-0.78		0.58		61.3		33.5		94.8	
ENSG00000133106	EPSTI1	-0.81		0.54		65.1		29.6		94.7	
ENSG00000138496	PARP9	-0.84		0.48		71.1		23.3		94.4	
ENSG00000102524	TNFSF13B	-0.52		0.82		27.1		66.6		93.7	
ENSG00000162654	GBP4	-0.81		0.53		65.3		28.2		93.5	
ENSG00000163220	S100A9	0.62		0.74		38.2		55.1		93.3	
ENSG00000177409	SAMD9L	-0.86		0.45		73.1		20.1		93.2	
ENSG00000162645	GBP2	-0.83		0.49		69.4		23.7		93.1	
ENSG00000244734	HBB	0.96		0.04		91.8		0.1		92.0	
ENSG00000010030	ETV7	-0.90		0.33		81.2		10.7		91.8	
ENSG00000115415	STAT1	-0.73		0.61		54.0		37.7		91.7	
ENSG00000132530	XAF1	-0.82		0.48		68.0		22.8		90.9	
ENSG00000225492	GBP1P1	-0.83		0.47		68.1		22.5		90.6	
ENSG00000089127	OAS1	-0.84		0.44		70.8		19.5		90.3	
ENSG00000184371	CSF1	-0.84		0.44		71.0		19.2		90.2	
ENSG00000134326	CMPK2	-0.91		0.27		82.3		7.5		89.8	
ENSG00000171051	FPR1	0.46		0.82		20.9		67.9		88.8	
ENSG00000154451	GBP5	-0.82		0.46		67.5		21.1		88.5	
ENSG00000185499	MUC1	-0.60		0.70		36.4		49.4		85.9	
ENSG00000163840	DTX3L	-0.84		0.39		70.1		15.3		85.4	
ENSG00000143546	S100A8	0.62		0.69		38.1		47.2		85.3	
ENSG00000116663	FBXO6	-0.76		0.51		58.0		25.9		84.0	
ENSG00000125347	IRF1	-0.69		0.59		48.3		34.9		83.2	
ENSG00000221963	APOL6	-0.80		0.42		64.5		17.7		82.2	

Gene ID	Gene Name	G1 (SV-AS03)	G1 (SV-PBS)	G2 (SV-AS03)	G2 (SV-PBS)	V1 (SV-AS03)	V1 (SV-PBS)	V2 (SV-AS03)	V2 (SV-PBS)	V1+V2 (SV-AS03)	V1+V2 (SV-PBS)
ENSG00000069399	BCL3	-0.86		0.25		74.8		6.4		81.2	
ENSG00000121380	BCL2L14	-0.89		0.11		78.8		1.1		80.0	
ENSG00000137959	IFI44L	-0.73		0.51		53.5		25.6		79.1	
ENSG00000247275	AL160008.1	-0.85		0.24		72.9		5.7		78.6	
ENSG00000087237	CETP	-0.71		0.52		51.0		26.9		77.9	
ENSG00000119917	IFIT3	-0.75		0.47		55.5		22.1		77.7	
ENSG00000020577	SAMD4A	-0.82		0.33		66.7		10.7		77.4	
ENSG00000090339	ICAM1	-0.68		0.55		45.9		30.7		76.6	
ENSG00000176788	BASP1	0.72		0.49		51.8		24.1		75.9	
ENSG00000134321	RSAD2	-0.70		0.52		48.8		26.6		75.4	
ENSG00000101336	HCK	0.53		0.68		28.5		46.7		75.2	
ENSG00000166825	ANPEP	0.54		0.68		28.8		46.1		74.9	
ENSG00000078081	LAMP3	-0.69		0.52		47.0		27.4		74.5	
ENSG00000162747	FCGR3B	0.52		0.67		27.2		45.1		72.3	
ENSG00000173535	TNFRSF10C	0.58		0.61		33.1		37.6		70.7	
ENSG00000214872	SMTNL1	-0.78		0.25		61.3		6.4		67.8	
ENSG00000103569	AQP9	0.54		0.61		29.7		36.7		66.3	
ENSG00000125462	C1orf61	-0.67		0.46		45.1		20.9		66.0	
ENSG00000216490	IFI30	-0.70		0.34		48.3		11.7		60.0	
ENSG00000140968	IRF8	-0.65		0.41		42.5		17.0		59.5	
ENSG00000097021	ACOT7	-0.49		0.56		24.0		30.8		54.8	
ENSG00000143226	FCGR2A	0.53		0.49		27.8		23.9		51.7	
ENSG00000184557	SOCS3	-0.69		0.19		47.7		3.5		51.2	
ENSG00000163464	CXCR1	0.38		0.57		14.6		32.6		47.3	
ENSG00000161055	SCGB3A1	0.50		0.44		24.6		19.5		44.1	
ENSG00000237627	THA1P	-0.63		0.12		39.8		1.4		41.1	
ENSG00000225131	PSME2P2	-0.50		0.15		24.8		2.2		27.1	
ENSG00000162614	NEXN	-0.30		0.36		9.0		13.1		22.1	
ENSG00000189152	AC007952.1_189152	0.31		-0.35		9.5		11.9		21.4	
ENSG00000250081	CTD-2116N20.1	0.36		-0.26		13.3		6.6		19.9	
ENSG00000145975	C6orf146	-0.18		-0.26		3.4		6.6		10.0	
ENSG00000213058	RP4-765C7.2	-0.03		-0.26		0.1		6.7		6.8	
ENSG00000253590	IGLV3-13	0.05		0.16		0.2		2.6		2.8	

Table A152: Canonical loadings and explained variance (RNA-Seq, T-cells, Day 1, Gene variables). Gene variables with at least 25% of explained variance for the first two canonical variates are listed. GX: canonical loadings for canonical variate X, VX: % explained variance for canonical variate X, V1+V2: total % explained variance for both canonical variates. Values for CCA results with a poor cross validation score (< 0.70) are left blank. Sorted by maximum explained variance across treatments (V1+V2).

IMO Variable	IMO Variable Name	I1 (SV-AS03)	I1 (SV-PBS)	I2 (SV-AS03)	I2 (SV-PBS)	V1 (SV-AS03)	V1 (SV-PBS)	V2 (SV-AS03)	V2 (SV-PBS)	V1+V2 (SV-AS03)	V1+V2 (SV-PBS)
IP-10	Interferon gamma-induced protein 10	-0.85		0.44		72.5		19.3		91.9	
MIP-1α	Macrophage inflammatory protein	-0.69		0.43		47.8		18.7		66.6	
MCP-1	Monocyte chemoattractant protein-1	-0.34		-0.66		11.6		44.1		55.7	
IL-6	Interleukin-6	-0.47		-0.46		22.5		21.3		43.8	
RANTES	Chemokine (C-C motif) ligand 5	-0.57		-0.02		33.0		0.0		33.0	
Eotaxin-1	Eotaxin-1	0.16		-0.55		2.5		29.8		32.2	
TNF	Tumor necrosis factors	0.35		-0.44		12.5		19.1		31.7	

IMO Variable	IMO Variable Name	I1 (SV-AS03)	I1 (SV-PBS)	I2 (SV-AS03)	I2 (SV-PBS)	V1 (SV-AS03)	V1 (SV-PBS)	V2 (SV-AS03)	V2 (SV-PBS)	V1+V2 (SV-AS03)	V1+V2 (SV-PBS)
IFN-γ	Interferon gamma	0.46		-0.05		21.2		0.3		21.4	
IL-8	Interleukin-8	-0.08		-0.39		0.6		15.4		16.1	
IL-10	Interleukin-10	-0.30		-0.11		9.3		1.3		10.6	
IL-1β	Interleukin-1 beta	-0.30		-0.00		8.7		0.0		8.7	
IL-12p70	Interleukin-12 p70	-0.16		-0.22		2.6		4.8		7.4	
Nt	Microneutralization inhibition	0.11		-0.09		1.2		0.9		2.1	

Table A153: Canonical loadings and explained variance (RNA-Seq, T-cells, Day 1, IMO variables). IMO variables with at least 25% of explained variance for the first two canonical variates are listed. GX: canonical loadings for canonical variate X, VX: % explained variance for canonical variate X, V1+V2: total % explained variance for both canonical variates. Values for CCA results with a poor cross validation score ($<$ 0.70) are left blank. Sorted by maximum explained variance across treatments (V1+V2).

Gene ID	Gene Name	G1 (SV-AS03)	G1 (SV-PBS)	G2 (SV-AS03)	G2 (SV-PBS)	V1 (SV-AS03)	V1 (SV-PBS)	V2 (SV-AS03)	V2 (SV-PBS)	V1+V2 (SV-AS03)	V1+V2 (SV-PBS)
ENSG00000171051	FPR1	-0.13	-0.72	-0.44	-0.11	1.7	51.9	19.3	1.2	21.0	53.1
ENSG00000185792	NLRP9	-0.26	0.11	0.60	-0.43	6.8	1.1	36.3	18.8	43.1	19.9
ENSG00000173535	TNFRSF10C	0.31	-0.42	-0.10	-0.42	9.4	17.7	1.0	18.0	10.4	35.7
ENSG00000197249	SERPINA1	-0.51	-0.49	0.23	-0.09	26.0	24.3	5.4	0.9	31.3	25.1
ENSG00000252666	AD000090.1	0.02	0.50	0.18	0.21	0.1	25.3	3.4	4.4	3.4	29.7
ENSG00000143546	S100A8	-0.27	-0.39	0.07	0.38	7.4	15.2	0.4	14.4	7.9	29.6
ENSG00000188906	LRRK2	0.09	-0.52	-0.02	0.16	0.7	26.9	0.1	2.5	0.8	29.4
ENSG00000177606	JUN	0.32	0.33	0.43	-0.24	10.1	11.2	18.6	5.7	28.8	16.9
ENSG00000170954	ZNF415	0.28	0.12	-0.45	0.29	7.9	1.5	20.0	8.5	27.8	10.0
ENSG00000162747	FCGR3B	-0.25	-0.45	-0.25	0.24	6.3	20.0	6.2	5.9	12.5	25.9
ENSG00000119535	CSF3R	-0.22	-0.46	0.03	0.11	5.0	21.0	0.1	1.2	5.1	22.1
ENSG00000103569	AQP9	0.17	-0.46	-0.23	0.08	3.0	20.8	5.1	0.6	8.1	21.4
ENSG00000251826	AL365332.1	-0.12	0.34	0.01	0.26	1.5	11.8	0.0	6.5	1.5	18.3
ENSG00000244734	HBB	0.24	0.40	0.03	0.10	5.6	16.0	0.1	1.1	5.7	17.1
ENSG00000169429	IL8	-0.31	-0.29	0.26	0.16	9.8	8.3	6.7	2.5	16.5	10.8
ENSG00000240371	RP11-624G17.1	0.01	0.25	-0.01	-0.10	0.0	6.3	0.0	1.1	0.0	7.4
ENSG00000176788	BASP1	-0.03	0.18	0.07	-0.04	0.1	3.4	0.5	0.1	0.6	3.5

Table A154: Canonical loadings and explained variance (RNA-Seq, T-cells, Day 3, Gene variables). Gene variables with at least 25% of explained variance for the first two canonical variates are listed. GX: canonical loadings for canonical variate X, VX: % explained variance for canonical variate X, V1+V2: total % explained variance for both canonical variates. Values for CCA results with a poor cross validation score ($<$ 0.70) are left blank. Sorted by maximum explained variance across treatments (V1+V2).

IMO Variable	IMO Variable Name	I1 (SV-AS03)	I1 (SV-PBS)	I2 (SV-AS03)	I2 (SV-PBS)	V1 (SV-AS03)	V1 (SV-PBS)	V2 (SV-AS03)	V2 (SV-PBS)	V1+V2 (SV-AS03)	V1+V2 (SV-PBS)
IL-12p70	Interleukin-12 p70	0.98	-0.91	-0.17	-0.21	96.7	83.7	2.8	4.4	99.6	88.1
IFN-γ	Interferon gamma	0.31	-0.54	0.20	0.79	9.8	29.4	4.2	62.8	14.0	92.1
TNF	Tumor necrosis factors	0.91	-0.54	0.06	0.76	82.9	28.9	0.3	58.1	83.2	87.1
IL-8	Interleukin-8	0.65	-0.42	0.65	0.76	41.8	17.3	42.5	57.4	84.3	74.7
IL-1β	Interleukin-1 beta	0.59	-0.55	0.70	0.70	35.0	29.9	49.1	49.1	84.1	79.0
IL-10	Interleukin-10	0.69	-0.63	0.49	0.66	47.9	40.0	23.6	43.6	71.5	83.6
IL-6	Interleukin-6	0.46	-0.30	0.29	0.79	20.8	9.3	8.3	61.8	29.1	71.1

IMO Variable	IMO Variable Name	I1 (SV-AS03)	I1 (SV-PBS)	I2 (SV-AS03)	I2 (SV-PBS)	V1 (SV-AS03)	V1 (SV-PBS)	V2 (SV-AS03)	V2 (SV-PBS)	V1+V2 (SV-AS03)	V1+V2 (SV-PBS)
MCP-1	Monocyte chemoattractant protein-1	-0.43	0.47	0.70	0.16	18.4	22.1	48.9	2.5	67.4	24.6
MIP-1α	Macrophage inflammatory protein	0.53	0.44	0.52	0.68	28.3	19.0	27.4	46.5	55.7	65.5
Nt	Microneutralization inhibition	-0.41	-0.29	0.40	0.70	17.1	8.5	15.8	48.4	32.9	56.9
IP-10	Interferon gamma-induced protein 10	0.60	0.41	-0.24	0.47	36.4	16.5	5.7	22.0	42.1	38.5
Eotaxin-1	Eotaxin-1	0.35	0.27	0.51	0.14	12.5	7.4	25.6	2.1	38.1	9.4
RANTES	Chemokine (C-C motif) ligand 5	0.11	0.54	0.49	-0.03	1.3	28.7	24.5	0.1	25.7	28.8

Table A155: Canonical loadings and explained variance (RNA-Seq, T-cells, Day 3, IMO variables). IMO variables with at least 25% of explained variance for the first two canonical variates are listed. GX: canonical loadings for canonical variate X, VX: % explained variance for canonical variate X, V1+V2: total % explained variance for both canonical variates. Values for CCA results with a poor cross validation score ($<$ 0.70) are left blank. Sorted by maximum explained variance across treatments (V1+V2).

Gene ID	Gene Name	G1 (SV-AS03)	G1 (SV-PBS)	G2 (SV-AS03)	G2 (SV-PBS)	V1 (SV-AS03)	V1 (SV-PBS)	V2 (SV-AS03)	V2 (SV-PBS)	V1+V2 (SV-AS03)	V1+V2 (SV-PBS)
ENSG00000101057	MYBL2		0.29		0.86		8.6		74.4		82.9
ENSG00000208317	SNORD78_208317		-0.12		0.68		1.4		46.1		47.5
ENSG00000168546	GFRA2		-0.63		0.24		39.8		5.7		45.5
ENSG00000228696	ARL17B		-0.15		0.66		2.4		43.0		45.4
ENSG00000197465	GYPE		0.61		-0.21		37.3		4.6		41.9
ENSG00000244734	HBB		-0.40		-0.45		16.1		19.8		35.9
ENSG00000171051	FPR1		-0.44		0.35		19.7		12.2		31.9
ENSG00000169429	IL8		0.16		-0.50		2.4		24.9		27.3
ENSG00000243746	EEF1A1P10		-0.15		-0.47		2.2		22.5		24.8
ENSG00000038427	VCAN		-0.20		-0.39		4.2		15.5		19.7
ENSG00000188820	FAM26F		0.31		-0.14		9.7		1.9		11.5
ENSG00000225217	HSPA7		-0.19		0.25		3.6		6.3		9.9
ENSG00000221957	KIR2DS4		-0.16		0.13		2.7		1.6		4.3
ENSG00000156453	PCDH1		0.00		0.08		0.0		0.6		0.6
ENSG00000171848	RRM2		0.02		-0.04		0.1		0.1		0.2

Table A156: Canonical loadings and explained variance (RNA-Seq, T-cells, Day 7, Gene variables). Gene variables with at least 25% of explained variance for the first two canonical variates are listed. GX: canonical loadings for canonical variate X, VX: % explained variance for canonical variate X, V1+V2: total % explained variance for both canonical variates. Values for CCA results with a poor cross validation score ($<$ 0.70) are left blank. Sorted by maximum explained variance across treatments (V1+V2).

IMO Variable	IMO Variable Name	I1 (SV-AS03)	I1 (SV-PBS)	I2 (SV-AS03)	I2 (SV-PBS)	V1 (SV-AS03)	V1 (SV-PBS)	V2 (SV-AS03)	V2 (SV-PBS)	V1+V2 (SV-AS03)	V1+V2 (SV-PBS)
MIP-1α	Macrophage inflammatory protein		0.97		-0.03		94.8		0.1		94.9
IL-12p70	Interleukin-12 p70		-0.57		-0.75		32.7		55.6		88.3
IP-10	Interferon gamma-induced protein 10		0.89		0.01		79.9		0.0		80.0
MCP-1	Monocyte chemoattractant protein-1		0.81		-0.12		65.6		1.5		67.2
IL-1β	Interleukin-1 beta		-0.76		0.12		57.5		1.5		59.0
Eotaxin-1	Eotaxin-1		-0.63		-0.35		39.6		12.0		51.6
Nt	Microneutralization inhibition		0.61		-0.23		37.5		5.3		42.8
TNF	Tumor necrosis factors		-0.14		0.57		2.1		32.1		34.2
IFN-γ	Interferon gamma		-0.25		0.42		6.2		18.1		24.2

IMO Variable	IMO Variable Name	I1 (SV-AS03)	I1 (SV-PBS)	I2 (SV-AS03)	I2 (SV-PBS)	V1 (SV-AS03)	V1 (SV-PBS)	V2 (SV-AS03)	V2 (SV-PBS)	V1+V2 (SV-AS03)	V1+V2 (SV-PBS)
IL-6	Interleukin-6		-0.28		0.38		7.6		14.3		21.9
IL-8	Interleukin-8		0.21		0.06		4.3		0.4		4.7
IL-10	Interleukin-10		-0.08		0.15		0.7		2.2		2.9
RANTES	Chemokine (C-C motif) ligand 5		-0.08		-0.02		0.6		0.0		0.7

Table A157: Canonical loadings and explained variance (RNA-Seq, T-cells, Day 7, IMO variables). IMO variables with at least 25% of explained variance for the first two canonical variates are listed. GX: canonical loadings for canonical variate X, VX: % explained variance for canonical variate X, V1+V2: total % explained variance for both canonical variates. Values for CCA results with a poor cross validation score (< 0.70) are left blank. Sorted by maximum explained variance across treatments (V1+V2).

Gene ID	Gene Name	G1 (SV-AS03)	G1 (SV-PBS)	G2 (SV-AS03)	G2 (SV-PBS)	V1 (SV-AS03)	V1 (SV-PBS)	V2 (SV-AS03)	V2 (SV-PBS)	V1+V2 (SV-AS03)	V1+V2 (SV-PBS)
ENSG00000113721	PDGFRB		0.83		0.09		69.7		0.8		70.5
ENSG00000169429	IL8		-0.21		-0.77		4.2		59.2		63.4
ENSG00000112183	RBM24		0.57		-0.29		32.0		8.3		40.3
ENSG00000252666	AD000090.1		0.62		-0.13		38.7		1.6		40.3
ENSG00000173535	TNFRSF10C		-0.16		-0.47		2.7		22.4		25.1
ENSG00000232618	RP11-439L18.1		-0.10		-0.44		0.9		19.5		20.5
ENSG00000066336	SPI1		-0.13		-0.42		1.7		18.0		19.7
ENSG00000143776	CDC42BPA		0.25		0.32		6.3		10.4		16.8
ENSG00000162747	FCGR3B		0.01		0.34		0.0		11.3		11.3
ENSG00000182578	CSF1R		-0.19		-0.25		3.6		6.3		9.9
ENSG00000131042	LILRB2		0.10		-0.28		0.9		8.0		8.9
ENSG00000129194	SOX15		0.01		-0.26		0.0		7.0		7.0
ENSG00000197249	SERPINA1		0.04		-0.21		0.2		4.4		4.6
ENSG00000244734	HBB		0.12		-0.18		1.4		3.1		4.5
ENSG00000103569	AQP9		0.20		-0.01		3.9		0.0		3.9

Table A158: Canonical loadings and explained variance (RNA-Seq, T-cells, Day 28, Gene variables). Gene variables with at least 25% of explained variance for the first two canonical variates are listed. GX: canonical loadings for canonical variate X, VX: % explained variance for canonical variate X, V1+V2: total % explained variance for both canonical variates. Values for CCA results with a poor cross validation score (< 0.70) are left blank. Sorted by maximum explained variance across treatments (V1+V2).

IMO Variable	IMO Variable Name	I1 (SV-AS03)	I1 (SV-PBS)	I2 (SV-AS03)	I2 (SV-PBS)	V1 (SV-AS03)	V1 (SV-PBS)	V2 (SV-AS03)	V2 (SV-PBS)	V1+V2 (SV-AS03)	V1+V2 (SV-PBS)
IL-12p70	Interleukin-12 p70		0.93		-0.01		86.4		0.0		86.4
MIP-1α	Macrophage inflammatory protein		-0.28		-0.76		8.1		57.1		65.2
IFN-γ	Interferon gamma		-0.34		0.69		11.7		47.3		59.0
TNF	Tumor necrosis factors		-0.64		0.32		41.4		10.2		51.6
IL-6	Interleukin-6		-0.10		0.68		1.0		46.5		47.5
IL-1β	Interleukin-1 beta		-0.20		0.54		4.0		29.6		33.5
MCP-1	Monocyte chemoattractant protein-1		-0.52		0.25		26.6		6.1		32.7
IL-10	Interleukin-10		-0.39		0.32		15.5		10.2		25.7
IP-10	Interferon gamma-induced protein 10		-0.43		-0.20		18.7		3.9		22.6
RANTES	Chemokine (C-C motif) ligand 5		-0.11		0.46		1.3		20.9		22.2
Nt	Microneutralization inhibition		-0.36		-0.03		12.8		0.1		12.9

IMO Variable	IMO Variable Name	I1 (SV-AS03)	I1 (SV-PBS)	I2 (SV-AS03)	I2 (SV-PBS)	V1 (SV-AS03)	V1 (SV-PBS)	V2 (SV-AS03)	V2 (SV-PBS)	V1+V2 (SV-AS03)	V1+V2 (SV-PBS)
Eotaxin-1	Eotaxin-1		-0.09		0.35		0.8		11.9		12.8
IL-8	Interleukin-8		0.12		-0.24		1.5		5.7		7.2
HAI	Hemagglutination inhibition		-0.02		0.13		0.1		1.7		1.7

Table A159: Canonical loadings and explained variance (RNA-Seq, T-cells, Day 28, IMO variables). IMO variables with at least 25% of explained variance for the first two canonical variates are listed. GX: canonical loadings for canonical variate X, VX: % explained variance for canonical variate X, V1+V2: total % explained variance for both canonical variates. Values for CCA results with a poor cross validation score (< 0.70) are left blank. Sorted by maximum explained variance across treatments (V1+V2).

Cell Type	N [Pos, Neg]	Input Gene Variables	Selected Gene Variables	Mean Misclassification Error	SDEG [%]	Opt. λ	Intercept
B-cells	9, 10	44	24	0.05	33	0.0478	-2.2502
Dendritic cells	9, 11	252	16	0.10	94	0.6123	-0.6088
Monocytes	9, 11	494	32	0.15	88	0.4517	-0.9111
Neutrophils	9, 11	869	26	0.05	85	0.5335	-0.9094
NK-cells	9, 11	122	9	0.10	67	0.5097	-0.7060
T-cells	9, 10	68	14	0.05	86	0.4449	-0.7319

Table A160: Regularized seroprotection logistic regression model summary statistics (RNA-Seq, Day 1). N Pos.: subjects with HAI $\geq$ 1:40, N Neg.: subjects with HAI $<$ 1:40, SDEG [%]: percent overlap between selected genes (HAI pos. vs. neg.) and differentially expressed genes (SV-AS03 vs. SV-PBS).

Cell Type	Day	Median Fold Change (SV-AS03)	Median Fold Change (SV-PBS)	Median Fold Change (SV-AS03/SV-PBS)
B-cells	1	0.68	0.95	0.71
B-cells	3	1.13	1.02	1.11
B-cells	7	0.94	0.95	0.99
B-cells	28	0.74	1.04	0.71
Dendritic cells	1	0.66	0.99	0.67
Dendritic cells	3	1.43	1.03	1.38
Dendritic cells	7	0.86	1.06	0.81
Dendritic cells	28	0.76	0.97	0.79
Monocytes	1	1	0.93	1.07
Monocytes	3	1.21	0.98	1.23
Monocytes	7	0.82	0.93	0.89
Monocytes	28	0.79	0.95	0.83
Neutrophils	1	1.22	0.97	1.25
Neutrophils	3	0.92	0.94	0.98
Neutrophils	7	0.97	1.05	0.92
Neutrophils	28	0.94	0.92	1.02
NK-cells	1	0.51	1.03	0.49
NK-cells	3	1.22	1.08	1.12
NK-cells	7	1	0.99	1.01
NK-cells	28	0.79	0.87	0.91
T-cells	1	0.57	0.98	0.58
T-cells	3	1.15	1.11	1.03
T-cells	7	1	0.9	1.12
T-cells	28	0.99	1.01	0.99

Table A161: Median fold change in the percentage of live cells by immune cell type and treatment group.

Cell Type	Day	Median Fold Change (SV-AS03)	Median Fold Change (SV-PBS)	Median Fold Change (SV-AS03/SV-PBS)
B-cells	1	0.89	1.2	0.74
B-cells	3	0.91	1.1	0.83
B-cells	7	0.85	1.14	0.74
B-cells	28	0.7	0.83	0.85
Dendritic cells	1	0.7	1.17	0.6
Dendritic cells	3	1.26	1.17	1.07
Dendritic cells	7	0.74	1.26	0.59
Dendritic cells	28	0.75	0.87	0.86
Monocytes	1	1.26	1.04	1.21
Monocytes	3	1.11	0.9	1.23
Monocytes	7	0.75	1.1	0.68
Monocytes	28	0.66	0.75	0.89
Neutrophils	1	1.41	1.14	1.23
Neutrophils	3	0.79	0.88	0.89
Neutrophils	7	0.9	1.2	0.75
Neutrophils	28	1	0.72	1.39
NK-cells	1	0.56	1.31	0.42
NK-cells	3	0.97	1.01	0.96
NK-cells	7	0.93	1.14	0.81
NK-cells	28	0.64	0.76	0.84
T-cells	1	0.67	0.97	0.69
T-cells	3	0.97	1.09	0.89
T-cells	7	1.01	0.97	1.04
T-cells	28	0.87	0.95	0.92

Table A162: Median fold change in the number of live cells per mL by immune cell type and treatment group.

Subject ID	Study Visit Day	Missing Values	Experimental Comment
83FVD001	Day -14	Missing values for all celltypes and markers	FACS machine down; no activation samples collected
83FVD011	Day 0	Missing values for NK-cells and dendritic cells	Missed CD11c & CD56 Abs in FMO stains (no FMO data for NK/mDC)
83FVD015	Day 0	Missing values for T-cells	Missing CD3 in APC-cy7 FMO stain
83FVD015	Day 28	Missing values for all celltypes and markers	Out of window visit. No activation staining
83FVD016	Day 0	Missing values for T-cells	Missing CD3 in APC-cy7 FMO stain
83FVD018	Day 3	Missing values for all celltypes and markers	FACS machine down. No end of day activation staining
83FVD019	Day -14	Missing values for all celltypes and markers	FACS machine down. No activation staining (phen from sort stain)
83FVD019	Day 3	Missing values for all celltypes and markers	PBMC EXPOSED TO BLEACH (WB and PMN ok). No FMO staining
83FVD020	Day -14	Missing values for all celltypes and markers	FACS machine down. No activation staining (phen from sort stain)
83FVD022	Day 28	Missing CD86 values for all 6 cell types	PerCP detector up; missing PerCP FMO
83FVD023	Day 1	Missing values for all celltypes and markers	Lost samples. No stains.

Table A163: Samples with incomplete cell activation measures (FACS).

Subject ID	Study Visit Day	Obser-vations	Markers	Cell Type	Experimental Comment
83FVD002	Day 1	12	CD134, CD86	All	No difference in expression of activation markers between all FMO samples and the "cocktail" sample. High background in FMO samples suggests that all FMO samples may have actually gotten antibody.
83FVD003	Day 1	6	CD134	All	No difference in expression of activation markers between all FMO samples and the "cocktail" sample. High background in FMO samples suggests that all FMO samples may have actually gotten antibody.
83FVD011	Day 28	6	CD134	All	No difference in expression of CD134 between FMO and "cocktail" sample. High background in CD134 FMO suggests that this FMO may have actually gotten antibody.
83FVD015	Day -28	1	CD69	Dendritic cells	Very few CD11c+ (mDC) at any time point w/this subject
83FVD015	Day -14	1	CD69	Dendritic cells	Very few CD11c+ (mDC) at any time point w/this subject
83FVD015	Day 0	1	CD69	Dendritic cells	Very few CD11c+ (mDC) at any time point w/this subject
83FVD015	Day 1	1	CD69	Dendritic cells	Very few CD11c+ (mDC) at any time point w/this subject
83FVD015	Day 3	1	CD69	Dendritic cells	Very few CD11c+ (mDC) at any time point w/this subject
83FVD015	Day 7	1	CD69	Dendritic cells	Very few CD11c+ (mDC) at any time point w/this subject
83FVD015	Day 7	1	CD134	NK-cells	
83FVD017	Day -14	1	CD86	Neutrophils	The position of the CD15 population looked off, suggesting that machine compensation on that day was not optimal for the CD15 marker.
83FVD017	Day 7	1	CD69	Neutrophils	
83FVD017	Day 0	1	CD86	B-cells	
83FVD012	Day -28	1	CD86	B-cells	

Table A164: Outlying cell activation samples (FACS).

Package Name	Version	Package Name	Version	Package Name	Version
bitops	1.0-6	RGCCA	2.0	foreach	1.4.1
caTools	1.16	rgl	0.93.996	Matrix	1.1-2
chron	2.3-45	R.methodsS3	1.6.1	plyr	1.8.1
codetools	0.2-8	R.oo	1.18.0	mixOmics	5.0-3
digest	0.6.4	R.utils	1.29.8	gtools	3.3.0
evaluate	0.5.1	stats4	3.0.1	vegan	2.0-10
formatR	0.10	stringr	0.6.2	lattice	0.20-27
gdata	2.13.2	tools	3.0.1	permute	0.8-3
grid	3.0.1	coin	1.0-23	gplots	2.12.1
igraph	0.7.1	survival	2.37-7	MASS	7.3-29
iterators	1.0.6	impute	1.34.0	car	2.0-19
KernSmooth	2.23-10	sqldf	0.4-7	png	0.1-7
modeltools	0.2-21	RSQLite.extfuns	0.0.1	edgeR	3.2.4
mvtnorm	0.9-9997	RSQLite	0.11.4	limma	3.16.8
nnet	7.3-7	DBI	0.2-7	xtable	1.7-1
pheatmap	0.7.7	gsubfn	0.6-5	knitr	1.5
RColorBrewer	1.0-5	proto	0.3-10		
Rcpp	0.11.0	glmnet	2.0-2		

Table A165: List of R packages and versions used for the analyses presented in this report. R version 3.0.1 (2013-05-16) 'Good Sport' run on Ubuntu (release 13.04, x86-64-pc-linux-gnu (64-bit) platform).

References

Robinson, Mark D., and Alicia Oshlack. "A scaling normalization method for differential expression analysis of RNA-seq data." Genome Biol 11.3 (2010): R25.

McCarthy, Davis J., Yunshun Chen, and Gordon K. Smyth. "Differential expression analysis of multifactor RNA-Seq experiments with respect to biological variation." Nucleic acids research (2012): gks042.

Young, Matthew D., et al. "Method Gene ontology analysis for RNA-seq: accounting for selection bias." Genome Biol 11 (2010): R14
